# Molecular Weevil Identification Project: A thoroughly curated barcode release of 1300 Western Palearctic weevil species (Coleoptera, Curculionoidea)

**DOI:** 10.3897/BDJ.11.e96438

**Published:** 2023-01-24

**Authors:** André Schütte, Peter E. Stüben, Jonas J. Astrin

**Affiliations:** 1 Leibniz Institute for the Analysis of Biodiversity Change, Museum Koenig, Bonn, Germany Leibniz Institute for the Analysis of Biodiversity Change, Museum Koenig Bonn Germany; 2 Curculio Institute, Mönchengladbach, Germany Curculio Institute Mönchengladbach Germany

**Keywords:** DNA barcoding, integrative taxonomy, thresholds, Cryptorhynchinae, Curculionidae, Apionidae, Western Palearctic, Europe, Canary Islands

## Abstract

The Molecular Weevil Identification project (MWI) studies the systematics of Western Palearctic weevils (superfamily Curculionoidea) in an integrative taxonomic approach of DNA barcoding, morphology and ecology. This barcode release provides almost 3600 curated CO1 sequences linked to morphological vouchers in about 1300 weevil species. The dataset is presented in statistical distance tables and as a Neighbour-Joining tree. Bayesian Inference trees are computed for the subfamilies Cryptorhynchinae, Apioninae and Ceutorhynchinae. Altogether, 18 unresolved taxonomic issues are discussed. A new barcode primer set is presented. Finally, we establish group-specific genetic distances for many weevil genera to serve as a tool in species delineation. These values are statistically based on distances between "good species" and their congeners. With this morphologically calibrated approach, we could resolve most alpha-taxonomic questions within the MWI project.

## Introduction

With 400,000 described species, beetles (Coleoptera) constitute the most diverse animal order (Slipinski et al. 2011, Bouchard et al. 2017). Amongst them, weevils (superfamily Curculionoidea) form one of the most species-rich taxa, with 51,000 known species worldwide (Oberprieler et al. 2007). Exactly 15,407 weevil species are listed in the most recent catalogue covering the entire Palearctic realm (Alonso-Zarazaga et al. 2017) and about 3,500 species in the Western Palearctic (Löbl and Smetana 2011, Löbl and Smetana 2013). Weevils have a global distribution. Their larvae predominantly develop inside various plant parts, while adults mostly feed on leaves or roots. Many species are highly specialised; others feed on a wide range of plants (Zwölfer and Herbst 1988, Oberprieler et al. 2007, Letsch et al. 2018). Weevils play an important ecological role. Some species are pests in agriculture or forestry, for example, the large pine weevil *Hylobiusabietis* (Leather et al. 1999), the rice weevil *Sitophilusoryzae* or the maize weevil *Sitophiluszeamais* (Wu and Yan 2018). Many *Otiorhynchus* species are greenhouse or horticultural pests (Thiem 1932, Sprick 2009). Taxonomic identification is easy for some common weevil species, but for many others, it is challenging and requires genital preparation and considerable taxonomic expertise.

The **taxonomic impediment** (Godfray 2002, Godfray and Knapp 2004, Wheeler et al. 2004) implies that the number of experts able to identify organisms to species level is constantly decreasing (Irfanullah 2006), resulting - amongst other drawbacks - in inaccurate biodiversity assessments (Giangrande 2003). To compensate for this deficiency in times of the biodiversity crisis, two DNA-based approaches were simultaneously proposed (reviewed in Meier et al. (2006), Hansen et al. (2007), Teletchea (2010)): The **DNA taxonomy** concept by Tautz (Tautz et al. 2002, Tautz et al. 2003) proposed to utilise DNA sequences of several predefined standard genes as the scaffold for taxonomy, but not necessarily linked to the Linnaean binominal system. Hebert (Hebert et al. 2003a, Hebert et al. 2003b) envisioned relatively short DNA barcodes from a single gene as a universal system for re-identification purposes, ideally linked to current Linnaean names. Since then, **DNA barcoding** (sequencing the 5'-half of the COI gene, Folmer et al. 1994, for animals) has been widely adopted by the scientific community and is the most commonly-used molecular marker in animals (Waugh 2007, Blaxter 2016), also demonstrated by over 11,000 barcoding-related publications by November 2022 (WOS, Web of Science search for the term "DNA barcod*" in title/abstract/keywords). The Barcode of Life Database (BOLD, Ratnasingham and Hebert 2007), currently contains barcodes from almost 10 million specimens.

Genetic distances can be measured as a proportion of different nucleotide positions in percent. An important prerequisite for DNA barcoding is that interspecific genetic distances vary significantly for at least a large majority of cases from intraspecific genetic distances. This concept is often referred to as the **barcoding gap** (Meyer and Paulay 2005). Its existence was first stated for birds and various arthropod taxa (Hebert et al. 2003a, Hebert et al. 2003b, Hebert et al. 2004a, Hebert et al. 2004b, Barrett and Hebert 2005, Hajibabaei et al. 2006a, Hajibabaei et al. 2006b). The existence of the barcoding gap was thought to be an artefact of insufficient sampling across taxa (Meyer and Paulay 2005, Wiemers and Fiedler 2007, Bergsten et al. 2012). Over the years, it became apparent that some datasets showed more or less pronounced barcoding gaps, while others did not (depending on sampling, geographic region, taxon biology, degree of morphological crypsis, state of taxonomic revision of the group under study etc.).

Underlying morphological misidentifications pose a major problem to DNA barcoding datasets and reference collections. Unfortunately, specimen misidentification is common in literature, in collections and particularly widespread in public sequence databases (Pentinsaari et al. 2020) and might reach up to 56% for taxa difficult to identify (Shea et al. 2011). The use of obsolete taxonomic names can lead to similar problematic effects (Mulcahy et al. 2022), especially in the absence of material vouchers (Pleijel et al. 2008, Astrin et al. 2013).

The **Molecular Weevil Identification project** (MWI) presented here strives to avoid pitfalls that arise in DNA barcoding studies, when not backed up by an extensive voucher collection. MWI created a reference database of high-quality DNA barcodes from scratch. Almost 1300 Western Palearctic weevil species have been barcoded, based on rigorous vouchering routines and project criteria: DNA was extracted non-invasively from specimens, then mounted as morphological vouchers for the dry collection, accessible at a public natural history collection (Leibniz Institute for the Analysis of Biodiversity Change, Museum Koenig, Bonn, Germany). These morphological vouchers are accompanied by stored DNA extracts and tissue samples in a dedicated biobank at the same institute. The laboratory infrastructure used in MWI was that of the German Barcode of Life (GBOL) project (Geiger et al. 2016). In several taxa, type localities were revisited to sample for the MWI project. Only experienced researchers from the European coleopterists' association Curculio Institute collected and identified the specimens morphologically (see specimen data table in Suppl. material 1). In this barcode release, we did not add any publicly available sequences from GenBank, BOLD or other third parties to the dataset for quality control reasons. For new species described during MWI, the DNA barcodes were mostly generated from paratypes (collected at the same location as the holotype). If no paratypes were available, the holotype was used for barcode generation. Specimens were recovered during the lysis step to allow future validation of the initial identification. Embedding the DNA barcoding method within an **integrative taxonomic approach** (Will et al. 2005, Padial and De La Riva 2010) often helps to reveal cryptic diversity or synonyms (Hebert et al. 2004b, Pons et al. 2006, Kerr et al. 2007). Over several years of preparation for the present barcode release, dozens of alpha-taxonomic changes have been carried out, most of which began as conspicuous molecular findings and were then corroborated morphologically (often including the study of type material) and ecologically in a taxonomic feedback loop (Page et al. 2005). Until now, 157 taxonomic changes, including 80 new species descriptions, were based on MWI sequences. Most taxonomic changes refer to the subfamilies Cryptorhynchinae, Apioninae and Ceutorhynchinae, for example, Schütte et al. (2013), Stüben et al. (2013a), Stüben and Schütte (2013), Stüben (2014b), Stüben et al. (2015), Stüben and Bayer (2015), Stüben et al. (2016a), Stüben (2017b), Stüben (2018b), Stüben (2018c), Stüben (2018a), Stüben and Schütte (2018).

In practice, most of these initial conspicuous molecular findings consisted of simple genetic distances that were considerably higher (in relation to other intraspecific comparisons) or lower (in relation to other interspecific comparisons) than expected. Previous research shows that genetic distance values very often coincide with species limits, but vary widely by taxon and geographic setting, for example, 2.7% for a species delineation threshold for North American birds (Hebert et al. 2004a), 4% for North American spiders (Barrett and Hebert 2005) or 2% to 14% for Madagascan water beetles (Monaghan et al. 2005). The relevant question in this context is: **How does one know which values to expect**?

Considerable discussion has gone into the topic of genetic thresholds as criteria for species delimitation (10x rule in Hebert et al. (2004a), Monaghan et al. (2005)). We agree that such threshold values cannot be used to delineate species (Wiemers and Fiedler 2007, Hubert et al. 2010) as a subset under the argument that DNA barcodes alone are generally a poor criterion to describe species (Ahrens et al. 2021, Zamani et al. 2022). However, based on our experience, genetic thresholds can be profitably used as a heuristic criterion to highlight cryptic or problematic taxa in the vast majority of cases (new species, synonyms, species complexes), to be confirmed or refuted by morphological analysis.

This study presents a validated, taxonomically thoroughly curated barcode release with almost 3600 sequences, the most extensive Western Palearctic weevil barcode dataset until now, covering ca. 1300 weevil species. Based on this data and considering different distribution patterns, we also give group-specific, statistically derived heuristic hints regarding which genetic distance values fall within the typical interspecific range. Cryptorhynchinae, Ceutorhynchinae and Apioninae are represented in our dataset with a high species coverage for the sampled region. Therefore, we specify such values only for genera of those three subfamilies, providing minimum and average p‑distance values. By sharing these data, we hope to accelerate specimen re-identification within the discussed taxa and aid in prefiltering future cases for thorough integrative alpha-taxonomic investigation.

## Material and Methods

We analysed 3573 mitochondrial CO1 sequences of the DNA barcoding region (Hebert et al. 2003a, based on Folmer et al. (1994)), representing 1296 valid species (1391 taxa if infraspecific epithets and taxon qualifiers such as "cf." and "sp." are also counted). The dataset contains 2017 sequences newly released in this study, see Suppl. material 1 (GenBank accession numbers followed by a "new" tag). See Suppl. material 2 for the entire dataset in FASTA format. Both supplements can also be downloaded externally (DOI: 10.5281/zenodo.7430106).

### Sampling: collecting locations

**Figure 1.**
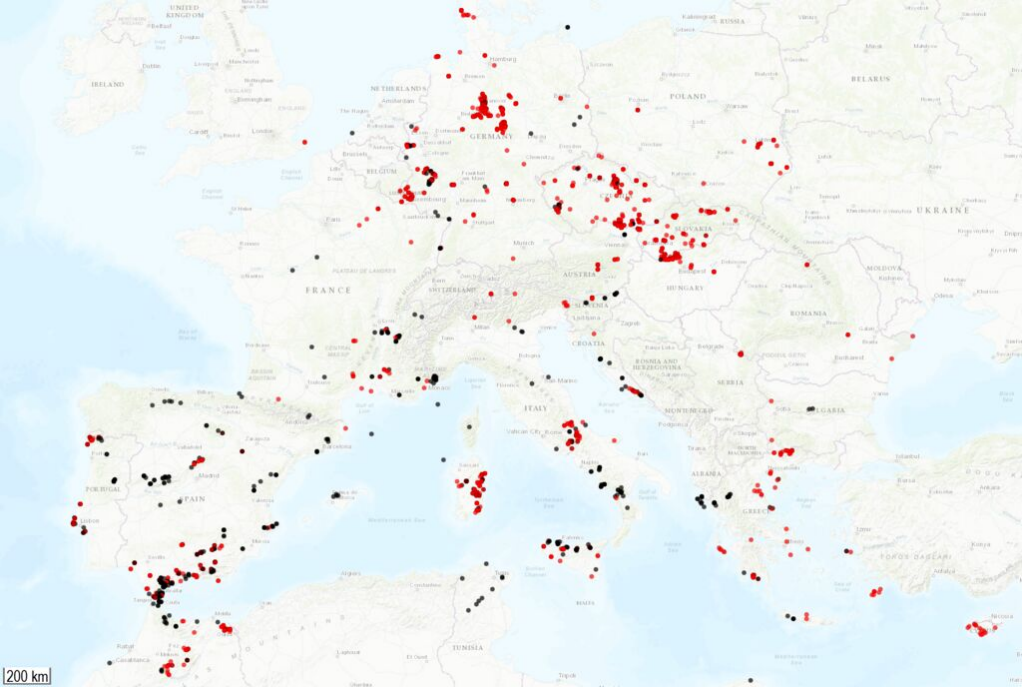
Continental collecting locations. Black dots = previously-released MWI sequences, red dots = newly published with this study.

**Figure 2.**
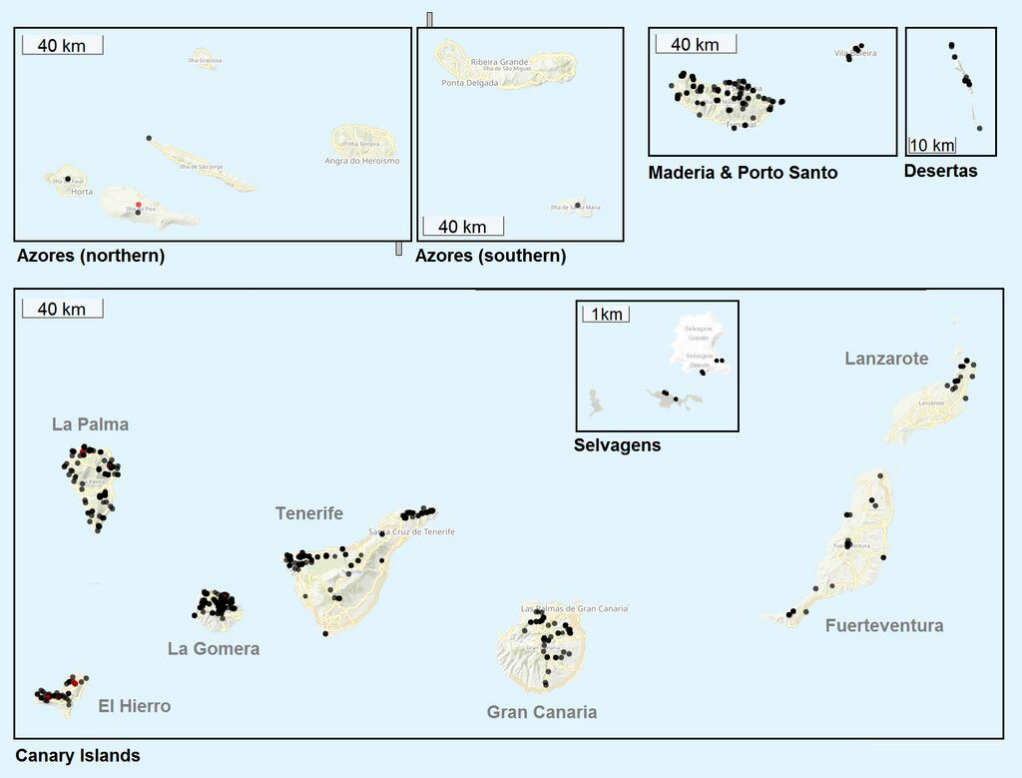
Collecting locations of Atlantic islands. Black dots = previously-released MWI sequences, red dots = newly published with this study.

The geographic origin of the collected weevils is as follows: 2510 specimens (70% of the dataset) were collected throughout continental Europe including the Mediterranean islands; 889 specimens (25% of the dataset) were collected on the Macaronesian islands including the Canaries, Azores, Madeira Archipelago with Desertas Islands and Savage Islands (Ilhas Selvagens); 164 specimens (5% of the dataset) were collected in continental North Africa, mostly Morocco and Tunisia. Collecting locations of the sampled specimens are plotted on two ArcGIS map baselayers with GPS Visualizer, see Fig. 1 (continental) and Fig. 2 (Atlantic islands).

**Figure 3.**
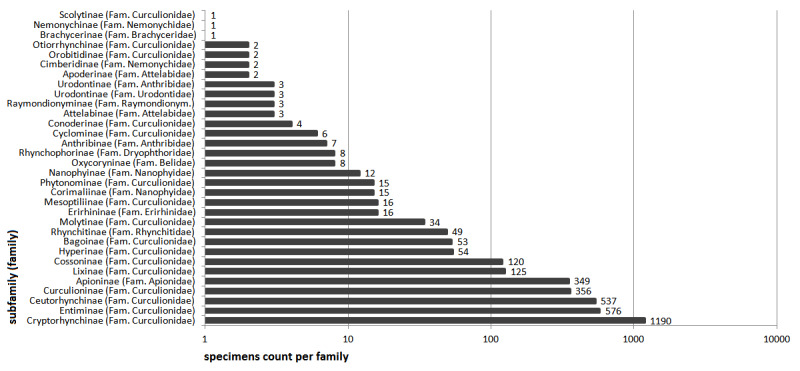
Number of specimens per subfamily.

**Figure 4.**
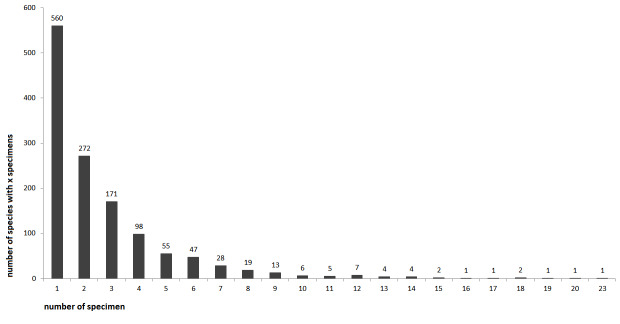
Number of specimens sampled per species (x-axis). Number above the bars show the specimen count (y-axis). Subspecies level not considered, unclear identifications are not counted in this figure (58x cf., 18x sp.). For 560 species, only one specimen was sampled.

The most frequently collected Curculionidae subfamilies were: Cryptorhynchinae (1190 sequences, 278 species, subspecies not differentiated in the species count), Entiminae (576 sequences, 269 species), Ceutorhynchinae (537 sequences, 203 species), Curculioninae (356 sequences, 168 species) and Apioninae (349 sequences, 115 species). A complete overview of sequences per subfamily is shown in Fig. 3. The number of specimens collected per species is illustrated in Fig. 4: 16% of sequences are singletons, the median is six specimens per species.

### Sampling: methodology

Most specimens were collected directly into 96% non-denatured ethanol without killing agents. In some cases, we sequenced previously-collected dried specimens, usually not more than five years old. Field data, voucher numbers and GenBank accession numbers for all specimens are provided in Suppl. material 1. The pinned specimen vouchers (dry), DNA vouchers and, where available, tissue vouchers (frozen, same population as the sequenced individual) are deposited at the Coleoptera collection respectively at the Biobank of the Leibniz Institute for the Analysis of Biodiversity Change, Museum Koenig, Bonn, Germany (ZFMK). For most specimens, the pinned specimen voucher was the source of the non-destructively isolated DNA. In previous extractions, the DNA derived from the frozen tissue samples with equal emphasis on morphological integrity (MWI samples with specimen ID lower than 1823-PST; see Suppl. material 1 for naming scheme).

### Laboratory processing

The laboratory routine for Cryptorhynchinae is described in Astrin et al. (2012). The laboratory routine for 91 sequences (specimen id 2906-PST to 3023-PST) is described in Stüben and Kramp (2019). The laboratory routine for all other samples is as follows: Genomic DNA was extracted from different parts of the beetle or non-destructively (for sample ID 1823-PST and higher) from whole specimens. Tissue lysis was performed at 56° Celsius overnight. For DNA extraction, a BioSprint 96 magnetic bead extractor was used with the corresponding kits, following the manufacturer's protocol for 200 µl elution volume (Qiagen: Hilden, Germany). We amplified the 5'-end of the CO1 (Cytochrome c oxidase subunit 1) gene with degenerate primers (Table 1): reaction volume of 20 μl; 2.5 μl of undiluted DNA template; Multiplex PCR Master Mix (Qiagen) with 16 pmol primer concentration for each primer (1.6 µl of 10 pmol/µl primer). The standard PCR product retrieved for weevils is 658 nucleotides (nt/bp) in length.

Thermal cycling was performed on GeneAmp PCR System 2700 instruments (Life Technologies, Carlsbad, USA) as follows: hot start Taq activation: 15 min at 95°C; first cycle set ("touch down" with 15 repeats): 35 s denaturation at 94°C, 90 s annealing at 55°C (−1°C/cycle) and 90 s extension at 72°C. Second cycle set (25 repeats): 35 s denaturation at 94°C, 90 s annealing at 40°C and 90 s extension at 72°C; final elongation: 10 min at 72°C. Amplicons were purified with the ExoSAP-IT kit (USB Corporation, Cleveland, Ohio) and sequenced bidirectionnally using the PCR primers (Table 1) at BGI Genomics (Shenzhen, China) or Macrogen (Amsterdam, The Netherlands) facility.

### Data analyses

Contig assembly and trimming of primer regions were performed in Geneious Pro 6.1.8 (Kearse et al. 2012). The sequences were screened for: 1) pseudogenes (NUMTs, Song et al. (2008)) by inspection of the reading frame for stop-codons and 2) endosymbionts by visual inspection of each taxon position in the NJ tree and NCBI BLAST (Madden 2002). In case of inconsistency, the sequence was excluded, followed by re-amplification or re-extraction and re-amplification.

The dataset contains **3573** weevil barcodes, of which **3302** sequences cover the full barcode length (658 bp), 272 sequences are shorter. Two sequences (GU987885, MG229813) barely failed to reach the 500 bp minimum required by BOLD (Milton et al. 2013) and were kept in the dataset.

**Alignment.** DNA sequences were aligned with the Muscle (Edgar 2004) plug-in in Geneious using default parameters (Drummond et al. 2012) and visually inspected for misaligned ends. The alignment is provided in Suppl. material 2.

**Neighbour-Joining tree.** The Neighbour-Joining (NJ, Saitou and Nei (1987)) tree is based on the nucleotide sequence alignment of the entire dataset of **3573** weevil sequences plus one outgroup species (Chrysomelidae, GenBank FJ867810). The NJ tree was created in Geneious Pro 6.1.8 (Kearse et al. 2012) with JC69 nucleotide substitution model (Jukes and Cantor 1969). The tree is provided in Suppl. material 3.

**Bayesian Inference.** Phylogenetic trees, based on Bayesian Inference, were reconstructed for three sub-datasets:

1) MrBayes sub-dataset for **Cryptorhynchinae + Cossoninae**: 1311 sequences in total, 1190 sequences from Cryptorhynchinae, 120 additional sequences from Cossoninae plus one outgroup species (Anthribidae, GenBank FJ867818).

2) MrBayes sub-dataset for **Apioninae + Nanophyinae** + **Attelabidae**: 367 sequences in total, 349 sequences from Apioninae, 5 additional from Attelabidae, 12 additional from Nanophyinae plus one outgroup species (*Cryptorhynchuslapathi*, D-0354-lap, GenBank EU286523).

3) MrBayes sub-dataset for **Ceutorhynchinae**: 537 Ceutorhynchinae sequences plus one outgroup (*Cryptorhynchuslapathi*, D-0354-lap, GenBank EU286523).

Based on the Bayesian information criterion value (BIC, Schwarz (1978)), calculated with jModelTest 0.1.1 (Posada 2008), we applied the GTR+I+G substitution model (Lanave et al. 1984) for all Bayesian analyses. We ran MrBayes (Ronquist and Huelsenbeck 2003) MPI version 3.2.7 multiprocessor version with eight cores in two independent replicates, each with one cold chain and three chains of different temperatures (standard setting). The genetic code for metazoan mitochondrial DNA (metmt) was defined. The third codon position of the GO1 gene was unlinked in shape, revmat, statefreq and pinvar. The analyses ran for 20 million generations, sampling 20,000 trees. Negative log-likelihood score stabilisation was checked in a separate visualisation in Microsoft Excel 2013. Accordingly, we retained 19,900 trees after discarding the burn-in data, of which a 50%-majority rule consensus tree with posterior probabilities was built. Geneious was used for the graphical display of the tree. The trees are provided in Suppl. material 5.

### DiStats statistics (p-distance calculation)

The Perl script DiStats (Astrin et al. 2016) simplifies the processing and statistical inspection of DNA barcode datasets. Amongst other functions, it calculates intraspecific and interspecific genetic distances for a given nucleotide alignment. The interspecific p-distance values per genus and distribution (island, continental) are provided for the genera of the three weevil subfamilies in the focus of this study. See Suppl. material 6 for an in-depth description of input data selection, raw output files, DiStats results and data compilation. The following references are used exclusively in the suppl. material: Stüben and Behne (2010), Morris (2011), Kratky (2015), Morris and Barclay (2015), Russell and Velazquez de Castro (2015), Stüben (2017a) and Sprick (2019).

The description below is the shortened version.

**Confidence groups.** For DiStats analysis, only Cryptorhynchinae, Ceutorhynchinae and Apioninae species are taken into account (datasets of the best-sampled subfamilies). The sequences of each species are assigned to one of three confidence groups:

- Confidence group **1** (reference species / "good species"): Contains taxa, which were morphologically clear in the past; one synonym allowed for Cryptorhynchinae, three synonyms allowed for Ceutorhynchinae and Apioninae, otherwise moved to confidence group 2;
- confidence group **2** (congener dataset): Contains valid taxa which created some or many synonyms or subspecies, not evaluated as reference species (not a "good species"), but available as congeners in the dataset; taxa, which were morphologically difficult or ambiguous to identify;
- confidence group **3**: (omitted species or specimens): Contains problematic taxa like species complexes or potentially new species, those were excluded from DiStats analyses.

**Only sequences / specimens from confidence groups 1 and 2 were used in DiStats statistics.**

**Distribution groups.** The reference species ("good species)" were also assigned to one out of four geographical distribution groups (Table 2) to assess the effect of different geographical distribution sizes with respect to interspecific genetic distances. The external supplement (DOI: 10.5281/zenodo.7430565) contains the geographical distribution maps used for the estimation of the maximum distribution range of each reference species.

**Interspecific distances per genus and distribution.** We examined the p-distances from the reference species to its closest congeners. Only for the reference species, the distance values to each closest congener were used to create genus lists with minimum and average interspecific distance values per geographic distribution group. The closest congener can be another reference species or a taxon assigned to confidence group 2 (congener dataset). We never used the p-distances from taxa of confidence group 2; those were kept only to increase the amount of congeners in the DiStats dataset.

### ASAP analysis

The programme 'Assemble Species by Automatic Partitioning' (ASAP, Puillandre et al. (2021)) estimates the species amount in a barcode dataset and suggests 10 p-distance threshold values for species delineation. ASAP is the successor of the programme 'Automated Barcode Gap Discovery' (ABGD, Puillandre et al. (2012)). We used the three sub-datasets created for DiStats with ASAP (Cryptorhynchinae, Apioninae, Ceutorhynchinae). Those sub-datasets only contain taxa from confidence group 1 ("good species") and confidence group 2 ("congener dataset"), see DiStats above. We compare the ASAP-calculated genospecies (MOTUs, Floyd et al. (2002), Blaxter (2004)) with the morphological identification and counted wrongly assigned species for each dataset. We have two questions: 1) how reliable is a species delineation, based on a single threshold per dataset and 2) is the best fitting threshold suggested by the programme the best possible one to match the morphological species identifications? See Suppl. material 7 for further details about the data assembly.

## Results

There remain 18 apparent contradictions between morphological identification and molecular results, see NJ tree in Suppl. material 3. These have been deliberately included in the dataset and are discussed in the **results of taxonomy** chapter in Suppl. material 4. Most of these cases await synonymisation or constitute very young species or species complexes challenging to disentangle with the CO1 gene. The following references are used exclusively in the suppl. material 4: Dieckmann (1979), Freude et al. (1981), Freude et al. (1983), Stüben (1994), Funk and Omland (2003), Bahr et al. (2008), Skuhrovec (2009), Stüben and Astrin (2010), Stüben et al. (2012), Stüben et al. (2013b), Stüben (2014a), Schütte and Stüben (2015), Stüben et al. (2016b).

### Neighbour-Joining tree

The NJ tree with the complete MWI dataset is shown in Suppl. material 3. At genus level, the NJ tree shows a very high congruence with the initial morphological identifications. Even higher taxa are mostly recovered as monophyletic and often cluster in a very similar way as reconstructed in Bayesian analysis, although NJ is neither a phylogenetic method nor is the mitochondrial CO1 gene alone considered suitable to resolve the relationships of higher taxa due to genetic saturation (Arbogast et al. 2002, Hebert and Gregory 2005). Additionally, at the species level, the neighbour-joining clustering algorithm delivers results that are consistently concordant with the Bayesian Inference.

Misidentified specimens are easy to spot in trees when embedded into a matrix of congeneric sequences – misidentified singletons are much more difficult to detect. Beyond misidentified specimens, conflicts can be caused by cryptic species or unresolved synonyms. Several of such inconsistencies have been clarified by taxonomists of the Curculio Institute over the last years, especially in Cryptorhynchinae, Ceutorhynchinae and Apioninae (see Introduction), thus delivering a cleaner picture for this barcode release.

### Bayesian trees

The Bayesian consensus trees focusing on three groups within the dataset are provided in Suppl. material 5: Cryptorhynchinae and Cossoninae with 1311 sequences; Apioninae, Nanophyinae and Attelabidae with 367 sequences; Ceutorhynchinae with 538 sequences.

The Bayesian posterior probabilities mostly show full or at least high (> 90) support in between species. The phylogenetic trees show substantially more polytomies than the phenetic NJ tree. Nevertheless, taxon placements with regard to the closest related species in the dataset mostly coincide between both methods or have marginal deviations. Thus, the Bayesian tree overall confirms the morphological species identifications and also the naming contradictions, based on unresolved taxonomic issues in the same way as the NJ tree.

### DiStats analysis (p-distance values)

The DiStats statistics are presented in Table 3 (Cryptorhynchinae), Table 4 (Apioninae) and Table 5 (Ceutorhynchinae). For each genus the minimum and the average value of the smallest available distance value to the closest congener is provided. The results per genus are separated into the four distribution groups defined in Table 2, referring to the size of the distribution area: island distribution (ISL), continental endemic (C1), medium distribution (C2) and large distribution (C3).

For genera of the subfamily Cryptorhynchinae, the **average** distance value between the closest available congener (often sister species) ranges from 3.8% (*Silvacalles*) to 19.9% (*Torneuma*). For genera of Apioninae, the average distance between the closest related congener ranges from 1.7% (*Taeniapion*) to 18.2% (*Pseudoperapion*). For genera of Ceutorhynchinae, the average distance between the closest related congener ranges from 6.4% (*Hesperorrhynchus*) to 17.8% (*Scleropterus*).

For some genera, the **smallest** p-distance value is significantly lower than the average one. This is often caused by just a single specimen within the genus. For example, in *Exapion*, the average distance between species is 7.0%, while the lowest value between two species is 2.3%. The closest conge Accept ner pair in this case is *Exapioncompactum* vs. *Exapionuliciperda*. All taxa and their closest congeners are listed in the spreadsheets in tab "DiStats_results" (Suppl. material 6) .

### ASAP analysis

See Table 6 for summarised results of the ASAP calculation and subsequent evaluation of concordance between calculated MOTUs and morphospecies; see Suppl. material 7 for data assembly and full length results. Based on the programme's calculation, the ASAP-score is not a reliable identifier for the confidence level of the suggested threshold (ASAP-score: "the lower, the better"), at least not for the three sub-datasets: **Cryptorhynchinae** partition 1 with the lowest ASAP-score of "**8.5**" shows 19% wrongly assigned taxa, while partition 6 with an ASAP-score of "17" shows 16% wrongly assigned taxa (3% less). The ASAP-score does not deliver helpful information here. **Apioninae** partition 1 with the lowest ASAP-score of "**3**" shows 14% of wrongly assigned taxa, while partition 6 with an ASAP-score of "9.5" shows just 7% of wrongly assigned taxa. Thus, the partition/threshold with the higher ASAP-score is providing a much better threshold for that subfamily. **Ceutorhynchinae** partition 1, with the lowest ASAP-score of "**1**", shows 6% wrongly assigned taxa, while Partition 10, with an ASAP-score of "18.5", also shows 6% wrongly assigned taxa. The suggested p-distance threshold value for partition 1 is 5.1%, while it is 5.5% for partition 10. The threshold values only show minor differences and a similar amount of wrongly assigned taxa are to be expected. Still, one time, the ASAP-score is the lowest (highest confidence) and the other time, the highest (least confidence). On the contrary, partition 3, with a relatively high ASAP-score of "11" and a threshold value of 5.0% (nearly the same as partition 1), shows the lowest percentage of wrongly assigned taxa (5% error rate). Thus, the partition/threshold with the higher ASAP-score can provide a better one.

## Discussion

The present DNA barcode release provides results for almost 1300 Western Palearctic weevil taxa. This dataset's strength lies in its thorough validation of specimens, including the actual nomenclatorial resolution of many cases of previous taxonomic conflicts (in preceding publications within the MWI project). The correct identifications are mirrored in a high consistency between **morphological identifications** and **molecular results**. The ambiguous cases where molecular and morphological evidence could not be reconciled are discussed (Suppl. material 4). These conflicts mainly have their basis in pending synonymisations or are caused by species complexes that cannot be resolved via DNA barcoding. In most cases, initial discrepancies between morphological identification and molecular results could be resolved by confirming or falsifying initial identification. In the latter case, additional resampling was needed and, in some situations, holotype comparisons, partly leading to taxonomic changes.

**DiStats statistics**. The most densely sampled subfamilies in the dataset often show genus-specific distances between species; see Suppl. material 6. Within each subfamily in focus, there are genera with small mean interspecific distances and others with high mean interspecific distances. For example, the Cryptorhynchinae dataset shows an average distance of 3.8% in the genus *Silvacalles*, while in the genus *Torneuma*, the average lies at 10.2% (both with island distribution). In Apioninae, the interspecific average ranges from 1.7% (*Taeniapion*) to 18.2% (*Pseudoperapion*; both taxa have a large distribution). In Ceutorhynchinae, it ranges from 6.4% (*Hesperorrhynchus*, island distribution) to 17.8% (*Scleropterus*, medium distribution, continental). Contrary to our initial expectations, it is clear that there does not exist a single threshold per subfamily that would characterise usual species limits. We also expected the species' distribution scales to be correlated in some form with the average genetic distances between species, but this is not the case either. For example, in the genus *Echinodera*, the average distances for the mainland distribution groups are 11.6% (small, endemic), 11.4% (medium), 10.8% (large), showing a slightly decreasing tendency from small to large geographical distribution. For the genus *Kyklioacalles*, this tendency is reverted: 7.8% (small, endemic), 9% (medium), 10.2% (large).

By summarising the statistical findings, it can be concluded that applying a single general genetic threshold for species delineation leads to mismatches between morphospecies and MOTUs, either false positives (oversplits) or false negatives (lumps). These mismatches are also clearly demonstrated in the ASAP results (see Table 6 and Suppl. material 7), with 10 widely-varying thresholds. No matter whether an increased or decreased threshold is applied, there remain significant deviations between morphospecies and calculated MOTUs, although no questionable taxa are included in the ASAP sub-datasets.

Targeting alpha-taxonomic questions with a single threshold approach likely leads to unsatisfactory error rates between 5% and 43% (see ASAP results in Table 6, right column).

ASAP or other single-threshold approaches are a convenient option to estimate species richness in widely-unknown biota or when there is no option to resort to using morphological information. Additionally, within a rough biodiversity assessment (e.g. metabarcoding), a small taxonomic error rate might not distort the final result. However, incorrect identifications can subsequently be incorporated into further studies. In the worst case, long-term environmental programmes could generate error cascades which can have a negative impact on environmental management and conservation (Bortolus 2008, Isaac et al. 2004).

It is known that undersampling leads to artificially increased interspecific genetic distances, creating deeper splits in trees and wider barcoding gaps (Moritz and Cicero 2004, Morando et al. 2003). Undersampling leads likewise to higher interspecific genetic distances in the DiStats statistics (Tables 3, 4, 5). This study's **species coverage** of the subfamilies **Apioninae** and **Ceutorhynchinae** is far from complete. The dataset contains 115 (29%) of roughly 400 binomial Apioninae taxa and 204 (51%) of roughly 400 binomial Ceutorhynchinae taxa of the Western Palearctic (Löbl and Smetana 2011, Löbl and Smetana 2013). By adding further sequences to the dataset, we assume the minimum average p-distance values will decrease significantly for some genera. Unlike in Apioninae and Ceutorhynchinae, the genera-specific distances of the **Cryptorhynchinae** dataset are not strongly affected by undersampling. We have covered 278 of 384 (72%) currently known binomial Cryptorhynchinae taxa (Stüben 2018b). From our observation, if the sister species is missing in the dataset, the remaining congeners are marginally more distant (higher p-distance values). For example, the adelphotaxon to *Acallesgranulimaculosus* is *Acallespilula*, both taxa are included in the dataset and they show a genetic distance of 11.3%. By removing *Acallespilula* from the dataset, the closest congener is *Acallesglobulipennis*, with a genetic distance of 12.3%. Thus, the minimum interspecific genetic distance for this taxon would increase by 1% in the DiStats output data table and the calculated average minimum distance for the genus *Acalles* from 9% to 9.2% in the island species compilation (Table 3).

Besides missing several species, sampling usually could not cover the entire **geographic distribution** of most continental taxa in our dataset. Complete sampling within each species' full geographic distribution range would likely reveal higher intraspecific distances than we can observe in the trees. Thus, we have not focused on intraspecific variation for the time being. Bergsten et al. (2012) found the intraspecific variation in Agabini diving beetles to be significantly correlated with the geographic scale of sampling: up to 70 individuals were required to sample 95% of the intraspecific variation.

Future collecting of weevils on the Western Palearctic mainland should bear this in mind and should strive to fill the mentioned gaps. The Canary Islands were extensively sampled. The dataset usually contains at least one specimen per taxon from each island (for multi-island distributions) or several collecting spots per island (for endemic/single island distributions). Most species occurring in the Canaries do not occur on the mainland. Many mainland species, however, especially in Apioninae and Ceutorhynchinae, occur far beyond the Western Palearctic. Species distribution maps for the three subfamilies in focus are provided as external supplement under DOI 10.5281/zenodo.7430565.

The many different examples from the literature (Coyne and Orr 1997, Sasa et al. 1998, Presgraves 2002, Mendelson 2003, Hebert et al. 2003a, Hebert et al. 2003b, Hebert et al. 2004a, Hebert et al. 2004b, Barrett and Hebert 2005, Monaghan et al. 2005, Vences et al. 2005, Zigler et al. 2005, Hickerson et al. 2006, Lefebure et al. 2006, Mikkelsen et al. 2007, Wiemers and Fiedler 2007, Hubert et al. 2008, Hundsdoerfer et al. 2009, Robinson et al. 2009, Acs et al. 2010, Hubert et al. 2010, Candek and Kuntner 2015, Spasojevic et al. 2016) clearly demonstrate that different groups have different genetic variabilities (see Suppl. material 8 for a summary of the previously mentioned references). Useful thresholds to delineate animal species boundaries using CO1 barcodes are often found between 4% and 15% genetic distance. However, almost every reference has **deviant taxa** with larger or smaller values. Besides subsampling, a taxon's age, modes of evolution and reproduction and its current and historical distribution (range size, climate, isolation barriers) can shape such distances. No single threshold would hold for all taxa, but knowing the average and minimum genetic distances between species within a specific (taxonomically limited group of interest) can be a vital supplementary tool in resolving taxonomic issues. For the weevil subfamilies we studied, we found that the taxonomic level for defining a common barcode threshold cannot meaningfully be established above the genus. Applying a threshold on genus level usually allows comparisons of species sharing a similar distribution pattern, evolutionary age and/or occupying similar ecological niches.

The thresholds we propose to use as heuristic support tools for future research in these groups are well-calibrated morphologically and based on "good species". These values can provide a reference for future alpha-taxonomic weevil research **consistent with the definitions or understanding of existing species**. Genetic distances are easy to measure, but prompt the question of how much distance is needed to delineate species. On the morphological side, each weevil group has its own set of particular characteristics used for morphological identification, which specialists have agreed upon over time, often somewhat subjectively. Morphological variations (intraspecific and interspecific) have been the basis of discussions in taxonomy ever since and are crucial to study prior to a taxonomic change. Those **morphological characters** used for identification and delineation are mostly based on the **consensus principle** of the scientific community. Essential characters in one group, bristle length, for example, might not play any role in another group, where perhaps the colouration pattern on the elytra or the protrusion of the eyes may constitute the central diagnostic characters. Based on many years of experience, a specialist will know about those morphological characters in his/her studied group. Known morphological variability within species is well factored in when examining differential characters for a new species description. Comparable situations arise when NJ trees or their underlying genetic distances are discussed (molecular intraspecific and interspecific variation), for example, when a single species appears in two neighbouring clusters. Based on a solely molecular point of view, those clusters might be separated by sufficient distance to infer the existence of a new species. Still, the morphologist may know from experience that those two clusters belong to a geographic variation.

A - mostly historical - quantitative approach for species delineation was morphological phenetics or "numerical taxonomy" (Sneath and Sokal 1973). In weevils, for example, comparative results were published to delineate *Ischnopterapionmodestum* and *Ischnopterapionplumbeomicans* with dozens of minimum, maximum and average length measurements from each body part of the two weevil species (Ehret 1991). However, phenetics is unable to recover evolutionary relationships as it does not differentiate between homology and homoplasy. Thus, it has been substituted with computational methods which can deliver an approximation of phylogeny (Wägele 2005).

New species descriptions, based solely on DNA barcoding, have been carried out or at least suggested for **cryptic species** or **species complexes** soon after DNA barcoding was established (Brower 2010, Cook et al. 2010, Jörger and Schrödl 2013, Doerder 2018). This strategy has recently been used for **hyperdiverse taxa** from the tropics as well (Butcher et al. 2012, Riedel et al. 2013a, Riedel et al. 2013b, Meierotto et al. 2019, Sharkey et al. 2021). Providing only a DNA barcode (Doerder 2018) or a consensus sequence (Sharkey et al. 2021) without thoroughly investigating the (group-specific) interspecific distances means postponing the (molecular) differential diagnosis. Discriminatory positions as differential diagnosis (proposed by Cook et al. (2010), applied by Jörger and Schrödl (2013) and Meierotto et al. (2019)) involve some uncertainty. They might become invalid as more specimens are collected, covering additional intraspecific variation from the same species or from closely-related species. Nevertheless, these approaches may constitute a way to capture the biodiversity of underdescribed taxa quickly. Riedel et al. (2013b) state: "A combination of digital imaging and molecular techniques allows the reduction of formal species descriptions to brief but highly accurate diagnoses. Although none of these tools is novel in itself, the progressive element is their combination and streamlining to produce a large number of usable species descriptions," provided the identifier has access to a sequencing facility and sufficient morphological knowledge to seek and find differential characters on the pictures provided.

**Yet**, **excluding morphology is not commonly accepted in the scientific community** (Pante et al. 2015, Ahrens et al. 2021, Zamani et al. 2022), even not for protists (Warren et al. 2017). Using a universal genetic distance threshold to compellingly delineate species would decouple taxonomy from the previously-established systematics. It would lead into the direction of a "parallel taxonomy", which has been rejected 20 years ago (see Sperling (2003) on Tautz' DNA taxonomy concept in Tautz et al. (2002) and Tautz et al. (2003)).

Relying solely or predominantly on DNA barcodes for species descriptions promises a **turbo taxonomy** (Butcher et al. 2012) or **fast-track taxonomy** (Riedel et al. 2013a). It seems appealing when morphology reaches its limits or performance increase is in focus (Fernandez-Triana 2022). In the long run, a trade-off between molecular quick-wins and morphological expertise may occur, for example, how to examine type specimens. Waiving morphological diagnoses in taxonomically challenging cases will most likely supersede conventional species descriptions soon if precautions are not being taken.

The **taxonomic inflation** issue was addressed before DNA barcoding was introduced. Concerns were based on the practice of raising taxa from subspecies to species level, thus resulting in a change of the species concept rather than new species discoveries (Isaac et al. 2004, Rylands and Mittermeier 2014). This issue gains special relevance in light of DNA-based approaches. Even moderate differences in genetic distances between clusters of individuals can reach high statistical significance (Galtier 2019, Vences 2020), further prompting taxonomists to rank such clusters as subspecies or species (Hey 2009). However, we propose **subspecies** should not be seen as anything other than **heuristic guides**. They should prompt the community to consult an integrative array of methods, such as morphological, molecular, biogeographic, ecological or ethological, prior to elevating subspecies to species status. This would make decisions on species status much more sustainable.

Adding **nuclear markers** in combination with phylogenetic models like multi-species coalescent model improve the accuracy of species delineation drastically (Eberle et al. 2020, Miralles et al. 2020, Dietz et al. 2021). They can easily uncover mitochondrial introgression, a possible downside of extrachromosal inheritance of the CO1 barcoding gene. Within the dataset of this study, we encountered two conspicuous cases of introgression: *Hesperorrhynchuslinaeotesselatus* (Stüben and Kratky 2016) and *Cionusgriseus* (Stüben and Behne 2015, Stüben et al. 2021, see further information in Suppl. material 4). Still, simply adding genes cannot assist in developing a **molecular species concept** (Galtier 2019) and does not answer questions regarding genetic thresholds in species delineation, since speciation circumstances (e.g. divergence times, population size) and sampling depth affect the dataset in the same way. Barcoding the **holotype** (as non-destructively as possible) is the **gold standard** in molecular taxonomy. This has been done for some MWI holotypes, for example, *Madeiracallesbeelzebubi* Stüben & Kratky, 2018 or *Torneumaalexi* Stüben, 2018 (both described in Stüben (2018b)). Only a holotype provides an objective link to its Linnean binomen. Type material is often difficult to access. Most type-holding institutions still offer on-site inspections, which requires travelling. Often they only refer to photos of the specimens available on their web page. Loaning is still offered in some cases, but comes with waiting periods (sometimes even several years, based on the second author´s experience). To send out type specimens is time-consuming and exposes them to the risk of getting lost. If barcodes of most holotypes were openly available, requesting shipping of type material could be omitted in many cases. Using a **paratype** specimen to retrieve the DNA barcode is the second best choice if the collecting location matches the holotype's and sympatric occurrence is unlikely, for example, *Torneumakorwitzi* (Stüben and Schütte 2015). Surprisingly, the latter is rarely discussed in literature. Most insects were described before the advent of molecular tools. Barcoding of **historical type material (hDNA)** is not a new idea, but still controversial due to often invasive processing of the most valuable collection specimens (Townson et al. 1999, Mayer et al. 2021, Raxworthy and Smith 2021). However, we should be aware that retroactive barcoding of type material facilitates **robust and sustainable knowledge gain** in taxonomy to solve existing and future research questions (Strutzenberger et al. 2012, Prosser et al. 2015, Speidel et al. 2015, Hausmann et al. 2016, Scherz et al. 2020, Raxworthy and Smith 2021, Roycroft et al. 2022, Mulcahy et al. 2022). A final option to gain reliable DNA barcodes after the new species has been described, is recollecting specimens from the **type locality** (Jörger and Schrödl 2013, Bell et al. 2020), which was also one focus of the MWI project.

During the past 250 years, almost every taxonomic change was based on morphological characters, continuously re-evaluating the underlying morphological characters. Hence for weevils, we can assume this "cleanup process" has built a strong foundation of valid morphological characters in most cases. We suggest preserving the already established and globally-accepted **Linnean understanding of species as taxonomic backbone**. This will ease the progression from a morphology-based past into a strong molecular-based future taxonomy, which will be compatible with the past. The risk of a disjunct parallel taxonomy would be decreased and the potential taxonomic inflation restrained to a minimum. The morphologically calibrated genus-specific distance values, based on "good species" (Tables 3, 4, 5), constitute a somewhat reliable direction for species of the molecularly well-sampled subfamily Cryptorhynchinae and an initial approximation for Apioninae and Ceutorhynchinae. In general, the results of the molecular dataset (CO1) should be utilised in an integrative taxonomy approach, i.e. discussed with morphological and ecological aspects, geographical distribution and evolutionary age of the taxon in focus.

**Here, we should address some pitfalls to prevent future inflationary species descriptions**:

**1. Ignoring the minimum interspecific distances of the sister species.** The interspecific genetic distances for weevils are mostly group-specific. A group can be a genus (e.g. *Torneuma* or *Silvacalles*), but it can also be a subgenus (e.g. subgenus Euphorbioacalles of the genus *Dendroacalles*) or even a species complex (e.g. *Acallesmaraoensis* complex). The interspecific distances of the sister species should be considered. If no sister species pair is available in the dataset, the closest congeners can be taken for an approximation. Otherwise, newly-collected specimens originating from a different population might be potentially classified as new species. Even small distances can create a split in a tree and might justify a new species description at first glance. If the interspecific distance of the potential new species falls below the previously known minimal one, the researcher should be **cautious not to describe a synonym**. A description can still be carried out if strong reasons justify the new species (Stüben and Schütte 2015, Garcia et al. 2019), for example, the young age of the new species or clear differences in morphologic characters under strong selective pressure, for example, in the aedeagi.

**2. Single sequences per taxon or population.** Using a single sequence per taxon drastically increases the risk of wrong conclusions when applied to alpha-taxonomic questions because intraspecific variation is not shown, but can be high for some taxa. In addition to the increased risk of misidentifications in singletons, not including intermediate specimens (of the same species) can create an artificial split in a tree which could be misinterpreted as a newly-discovered species, especially if the analysed individual was collected far from the previously-known sequence. If a species has a disjunct distribution, providing just one sequence from each population increases the likelihood of producing a synonym. This risk especially applies to islands. Artificial deep splits can be produced if the intraspecific distances within a population coincide with or even exceed the interspecific distances. On dataset compilation, the full **sampling depth should be used**. Using a single sequence from each population instead of all available sequences means leaving out all intermediate specimens belonging to the same species. The intraspecific distances then present themselves as an artificial deep split. The latter might be the case for some *Laparocerus* taxa described recently (Faria et al. 2016, Machado et al. 2017). For a more detailes explanation see Stüben (2022).

**3. Gaps in existing sequence databases.** A large genetic distance to the closest congener in a sequence database is not proof of having discovered a new species. Often, no reference sequences of the sequenced species have been previously deposited. Subsequently, a misinterpretation of the interspecific genetic distance to the closest database match, for example, 15% to the closest deposited one, can lead to describing a synonym, particularly if the sister species' type material is not consulted. Although potentially new species can be discovered very quickly with DNA barcoding (Riedel et al. 2013a, Meierotto et al. 2019, Sharkey et al. 2021), they must be taxonomically secured in the same way as traditionally done, at least for well-revised taxa: either by comparing all closely-related species morphologically to the potential new species and/or (especially where the former option is not an option) or by consulting barcodes from all closely-related species. Unfortunately, a described species lacking this validation can be laborious to refute. Most of the work (type comparison, sequencing) has to be carried out by a third party if the original author failed to do so. Unfortunately, it can be assumed that there will be a relatively large number of quickly described species in the future, only based on barcodes and not backed up by holotype comparisons. This situation can arise in **island biota**, for which one quickly tends to assume endemism: a candidate species is discovered by molecular means and described without **holotype comparison** to the continental fauna (e.g. in Garcia et al. (2022)). In this context, the candidate species carries the risk of being a synonym, because the species might have been described already from the mainland. The wrong conclusion "it must be a new species" is quickly made if no public sequence is available. Vice versa, new descriptions should always be supported by molecular data to prevent describing a synonym – also by other researchers at a later point. Sometimes, formerly established unique morphological traits used for species description can turn out to be misleading diagnostic characters after molecular data become available. The cryptorhynchine species *Calacallesagana* Stüben, 2010 (Stüben 2010) can serve as an example: the author described the species without molecular support and synonymised it several years later (Stüben 2015). Another case with conflicting results between morphology and DNA barcoding is *Aeoniacallesaeoniibodegensis* (see Suppl. material 9, Stüben (2005), Stüben and Germann (2005), Stüben and Astrin (2011)).

Following the biological species concept (Mayr 1942), one could consider conducting **cross-breeding experiments** (Stüben 2005) for some generations prior to a new species description.

## External supplementary material

**Data Type**: geographical distribution maps. **Brief description**: the ZIP file contains 613 distribution maps from Western Palearctic weevil taxa. The distribution maps showing Europe originate from the Curculio Institute's website (www.curci.de). Additional information on distribution range and known synonyms were based on the information from the Löbl catalogues (Löbl and Smetana 2011, Löbl and Smetana 2013). The maximum distribution range of each species was measured in km with Google Earth's ruler function. **Download via Zenodo DOI: 10.5281/zenodo.7430565 (368.1 MB).**

**Data Type**: Material Table and CO1 sequences. **Brief description**: alternative download source for the material table and the CO1 sequences used in this study. **Download via Zenodo DOI: 10.5281/zenodo.7430106 (3.9 MB).**

## Supplementary Material

6DE1213B-C98E-53B7-A107-58DCE3CDF7B1 10.3897/BDJ.10.e96438.suppl1 Supplementary material 1 Material Table
Data type spreadsheet with collecting data, voucher numbers and GenBank acc. numbers Brief description The material table (248 pages in PDF format) contains information about each specimen's collecting spot, GPS position, collector, identifier, voucher numbers (DNA and tissue) and GenBank accession numbers. File: oo_781319.pdf https://binary.pensoft.net/file/781319 Schütte A, Stüben PE, Astrin JJ

E92C9B49-80EB-5B9B-A6B4-DD5A3D8EC84C 10.3897/BDJ.10.e96438.suppl2 Supplementary material 2 CO1 Sequences Data type CO1 sequences, DNA barcodes, nucleotide alignment, list with GenBank accession numbers Brief description This study's complete DNA barcode dataset is provided as nucleotide alignment and unaligned sequences in *.fasta file format. The *.fasta files can be opened with any text editor. A read_me.txt file is included explaining the naming scheme. A list of GenBank accession numbers only is included. File: oo_781371.zip https://binary.pensoft.net/file/781371 Schütte A, Astrin JJ

FA94D83A-61FC-5B01-AADE-D0F0F32CD52B 10.3897/BDJ.10.e96438.suppl3 Supplementary material 3 NJ Tree Data type MWI neighbour-joining tree Brief description The ZIP file contains the neighbour-joining tree in four different file formats (newick, nex, png, svg). The tree is based on the complete DNA barcode dataset published in this study. File: oo_735069.zip https://binary.pensoft.net/file/735069 Schütte A, Astrin JJ

1AADD759-868C-50EF-A7CF-C44A98CCA2DB 10.3897/BDJ.10.e96438.suppl4 Supplementary material 4 Results of Taxonomy Data type morphological identification versus molecular results Brief description Contradictions between morphological identifications and molecular results are discussed (PDF file). File: oo_784627.pdf https://binary.pensoft.net/file/784627 Schütte A, Stüben PE

DACE88C1-1EB9-5247-8773-73518738B022 10.3897/BDJ.10.e96438.suppl5 Supplementary material 5 Bayesian Trees Data type Bayesian Inference calculation, 50% majority rule consensus trees, Cryptorhynchinae, Apioninae, Ceutorhynchinae Brief description The ZIP file contains the 50% majority rule consensus trees for three sub-datasets of the study, calculated with MrBayes: 1) Cryptorhynchinae + Cossoninae; 2) Apioninae + Nanophyinae + Attelabidae; 3) Ceutorhynchinae. Each tree is provided in three different file formats (NEX, PNG, SVG). The searchable SVG file can be opened with any internet browser. File: oo_735074.zip https://binary.pensoft.net/file/735074 Schütte A

C9AFA73C-5320-5793-BD06-74769EF803D9 10.3897/BDJ.10.e96438.suppl6 Supplementary material 6 DiStats statistics Data type DiStats method description in detail (PDF), DiStats input data (nucleotide alignments) and output data (TXT), spreadsheets (XLSX) with data compilation Brief description The supplement contains an in-depth description of the used method (which taxon was used as a reference taxon to create the morphologically calibrated p-distance statistics for Cryptorhynchinae, Apioninae and Ceutorhynchinae). Besides the input data to the DiStats scripts and unformatted output data, the data compilation leading to the output tables is described and available in three Excel spreadsheets. File: oo_754323.zip https://binary.pensoft.net/file/754323 Schütte A

3CB553D3-FF57-5835-8BC1-4E781E780B06 10.3897/BDJ.10.e96438.suppl7 Supplementary material 7 ASAP analyses Data type nucleotide alignments (FASTA), raw data output (HTML, TXT), Excel spreadsheets (XLSX) Brief description Detailed description of the ASAP program and data compilation leading to the final summarised results table. File: oo_754318.zip https://binary.pensoft.net/file/754318 Schütte A

B046AABA-93DC-5A30-95CD-5705A733F531 10.3897/BDJ.10.e96438.suppl8 Supplementary material 8 Thresholds in various taxa Data type text Brief description Excursus about various taxa and their thresholds for species delineation. File: oo_754321.pdf https://binary.pensoft.net/file/754321 Schütte A

A63B0E35-6BE0-5D78-9360-09AB4686B0A3 10.3897/BDJ.10.e96438.suppl9 Supplementary material 9 Aeoniacallesaeoniibodegensis
Data type text Brief description Additional information to the unsolved taxonomic status of *Aeoniacallesaeoniibodegensis* (Stüben, 2000). File: oo_776983.pdf https://binary.pensoft.net/file/776983 Schütte A, Stüben PE, Astrin, JJ

## Figures and Tables

**Table 1.** PCR primer sets. LCO1490-JJ & HCO2198-JJ (Astrin and Stüben 2008) were used for all samples first, success rate was about 95% in both directions. If PCR or sequencing failed, reactions were repeated with a different primer set, either LCO1490-JJ2 & HCO2198-JJ2 (higher degeneracy, Astrin et al. (2016)) or repeated with the newly-developed primer set LCO1490-MWI & HCO2198-MWI.

Primer Name	5'-3' Read Direction	Reference	Optimised for
LCO1490-MWI	ACWAAYCATAARRAYATYGG	this study (new)	Apioninae & Ceutorhynchinae
HCO2198-MWI	TADACTTCDGGRTGDCCRAARAATCA	this study (new)	
LCO1490-JJ	CHACWAAYCATAAAGATATYGG	Astrin and Stüben (2008)	Cryptorhynchinae
HCO2198-JJ	AWACTTCVGGRTGVCCAAARAATCA	Astrin and Stüben (2008)	
LCO1490-JJ2	CHACWAAYCAYAARGAYATYGG	Astrin et al. (2016)	universal (arthropods)
HCO2198-JJ2	ANACTTCNGGRTGNCCAAARAATCA	Astrin et al. (2016)	

**Table 2.** Geographical distribution groups defined for the genera lists.

Distribution group	ISL (island)	C1 (endemic)	C2 (medium)	C3 (large)
Cryptorhynchinae	island(s)	up to 50 km	50 to 500 km	500 km and above
Apioninae	island(s)	up to 50 km	50 to 2,000 km	2,000 km and above
Ceutorhynchinae	island(s)	up to 50 km	50 to 2,000 km	2,000 km and above

**Table 3.** Summarised DiStats results for genera of Cryptorhynchinae. Numbers indicate uncorrected p-distance values (genetic distances) expressed in percent. Two values are given per genus and distribution range: 1. minimum distance to the closest congener within all species in the dataset and 2. average distance to the closest congener within all species in the dataset. Abbreviations: min. = minimum, dist. = distance.

**Cryptorhynchinae**	island(s)/ archipel	island(s)/ archipel	endemic (50 km)	endemic (50 km)	medium (50-500 km)	medium (50-500 km)	large (> 500 km)	large (> 500 km)
Distribution group	ISL	ISL	C1	C1	C2	C2	C3	C3
**Genus**	min. dist. to closest congener	min. dist. to closest congener	min. dist. to closest congener	min. dist. to closest congener	min. dist. to closest congener	min. dist. to closest congener	min. dist. to closest congener	min. dist. to closest congener
*Acalles*	8.4	9.0	3.2	7.0	6.2	10.6	7.1	11.7
*Acallocrates*							13.0	13.3
*Acallorneuma*	5.8	7.3			3.0	6.8	8.8	8.8
*Aeoniacalles*	8.8	9.1						
*Calacalles*	3.2	6.0					14.3	14.3
*Canariacalles*	6.4	6.4						
*Caucasusacalles*							15.3	15.3
*Coloracalles*							12.2	12.2
*Dendroacalles*	3.7	7.3						
*Dichromacalles*	7.3	7.3			12.3	13.4	12.9	13.7
*Echinodera*	3.3	9.6	5.8	11.6	5.8	11.4	6.1	10.8
*Echiumacalles*	6.8	6.8						
*Elliptacalles*							7.1	7.1
*Ficusacalles*	6.5	6.5						
*Kyklioacalles*	8.8	8.8	4.3	7.8	4.9	9.0	7.1	10.2
*Lauriacalles*	10.2	10.2						
*Madeiracalles*	1.8	8.5						
*Montanacalles*			13.7	13.7				
*Onyxacalles*	7.6	8.8			4.0	7.1	4.0	7.1
*Pseudodichromacalles*	6.4	7.7						
*Silvacalles*	0.9	3.8						
*Sonchiacalles*	8.3	8.8						
*Torneuma*	5.8	10.2	16.4	16.9				

**Table 4.** Summarised DiStats results for genera of **Apioninae**. Numbers indicate uncorrected p-distance values (genetic distances) expressed in percent. Two values are given per genus and distribution range: 1. minimum distance to the closest congener within all species in the dataset and 2. average distance to the closest congener within all species in the dataset. Abbreviations: min. = minimum, dist. = distance.

**Apioninae**	island(s)/ archipel	island(s)/ archipel	endemic (50 km)	endemic (50 km)	medium (50-2000 km)	medium (50-2000 km)	large (> 2000 km)	large (> 2000 km)
Distribution group	ISL	ISL	C1	C1	C2	C2	C3	C3
**Genus**	min. dist. to closest congener	min. dist. to closest congener	min. dist. to closest congener	min. dist. to closest congener	min. dist. to closest congener	min. dist. to closest congener	min. dist. to closest congener	min. dist. to closest congener
*Aizobius*							10.8	10.8
*Alocentron*							10.5	10.5
*Apion*							6.3	8.6
*Aspidapion*	4.4	4.4					4.9	4.9
*Catapion*							8.5	11.2
*Ceratapion*	10.8	10.8					4.4	8.6
*Cistapion*							15.8	15.8
*Cyanapion*							10.9	11.8
*Diplapion*	8.1	8.1					3.0	3.0
*Eutrichapion*							11.0	11.0
*Exapion*					10.5	10.5	2.3	7.0
*Hemitrichapion*							12.6	13.5
*Holotrichapion*	5.0	8.7					7.5	10.3
*Ischnopterapion*							13.3	13.3
*Ixapion*							12.6	12.6
*Kalcapion*	4.3	4.7					4.1	4.1
*Lepidapion*	2.7	2.7						
*Loborhynchapion*							13.2	13.2
*Malvapion*							11.7	11.7
*Omphalapion*					13.2	13.2	13.2	13.2
*Onychapion*							13.2	13.2
*Oryxolaemus*							8.5	8.5
*Oxystoma*							11.1	11.5
*Perapion*					2.1	2.1		
*Phrissotrichum*	8.2	8.2					8.2	8.2
*Protapion*							4.4	7.2
*Protopirapion*							14.3	14.3
*Pseudapion*					7.8	7.8	7.8	10.5
*Pseudaplemonus*							15.5	15.5
*Pseudoperapion*							18.2	18.2
*Pseudoprotapion*							14.6	14.6
*Pseudostenapion*							17.6	17.6
*Rhopalapion*							11.6	11.6
*Stenopterapion*					12.6	12.6	12.6	13.2
*Synapion*							13.2	13.2
*Taeniapion*	4.2	7.0					1.7	1.7
*Taphrotopium*							13.4	13.4
*Trichopterapion*							16.4	16.4

**Table 5.** Summarised DiStats results for genera of Ceutorhynchinae. Numbers indicate uncorrected p-distance values (genetic distances) expressed in percent. Two values are given per genus and distribution range: 1. minimum distance to the closest congener within all species in the dataset and 2. average distance to the closest congener within all species in the dataset. Abbreviations: min. = minimum, dist. = distance

**Ceutorhynchinae**	island(s)/ archipel	island(s)/ archipel	endemic (50 km)	endemic (50 km)	medium (50-2000 km)	medium (50-2000 km)	large (> 2000 km)	large (> 2000 km)
Distribution group	ISL	ISL	C1	C1	C2	C2	C3	C3
**Genus**	min. dist. to closest congener	min. dist. to closest congener	min. dist. to closest congener	min. dist. to closest congener	min. dist. to closest congener	min. dist. to closest congener	min. dist. to closest congener	min. dist. to closest congener
*Aphytobius*					7.6	7.6	7.6	7.6
*Auleutes*							15.7	15.7
*Barioxyonyx*					14.1	14.9		
*Brachiodontus*					15.7	15.8		
*Ceutorhynchus*	5.5	9.8			6.8	9.8	4.1	9.1
*Coeliodinus*							13.7	13.7
*Datonychidius*					15.4	15.4		
*Drupenatus*							13.5	13.5
*Eucoeliodes*					14.6	14.6		
*Eubrychius*							12.1	12.1
*Glocianus*							13.2	13.3
*Hadroplontus*							10.9	10.9
*Hesperorrhynchus*	5.0	6.4						
*Homorosoma*							14.3	14.3
*Marmaropus*							15.9	15.9
*Mesoxyonyx*					14.6	14.6		
*Micrelus*							11.9	11.9
*Microplontus*							12.3	14.0
*Mogulones*/ *Datonychus*	7.8	8.7			11.6	13.1	6.1	11.1
*Mogulonoides*							13.4	13.4
*Neoglocianus*							10.7	10.7
*Neophytobius*							14.9	14.9
*Oprohinus*							15.1	15.1
*Oreorrhynchaeus*			12.5	12.5				
*Parethelcus*	8.6	8.6						
*Paroxyonyx*					9.7	11.3	9.7	10.9
*Pelenomus*							11.5	13.4
*Perioxyonyx*					16.0	16.0		
*Phrydiuchus*					12.2	12.2	10.5	11.5
*Poophagus*							14.4	14.4
*Prisistus*					15.7	15.7	12.9	12.9
*Ranunculiphilus*							12.8	12.8
*Rhinoncus*					8.4	8.4	7.0	10.1
*Scleropterus*					17.8	17.8		
*Scleropteridius*							15.9	15.9
*Sirocalodes*	10.6	10.6					10.6	10.6
*Thamiocolus*	13.1	13.2					12.8	13.8
*Trichosirocalus*					12.0	12.0	12.6	13.6
*Zacladus*							12.2	12.2

**Table 6.** Left side of table: summarised ASAP results, right side of table: evaluation of concordance between MOTUs and morphospecies. For each subfamily dataset, 10 different thresholds ("ASAP partitions") and derived MOTUs are calculated by ASAP. The evaluation of concordance provides the deviations between MOTUs and morphospecies for each given threshold; wrongly assigned MOTUs are given in absolute numbers and in percent. Marked tables point to the threshold which fits best to each subfamily dataset (lowest number of deviation between MOTUs and morphospecies).

ASAP results	ASAP results	ASAP results	ASAP results	evaluation of concordance	evaluation of concordance	evaluation of concordance
**ASAP Partition**	**MOTUs**	**Threshold** [%]	**ASAP-score**	**no of wrongly assigned taxa**	**no of wrongly assigned seqs.**	% **of wrongly assigned seqs.**
Cryptorhynchinae sub-dataset (contains 265 morphospecies, 1106 sequences)						
1	236	7.3	8.5	74	214	19%
2	241	7.2	9.0	73	214	19%
3	315	3.8	9.5	84	190	17%
4	348	2.4	15.0	104	206	19%
5	639	0.4	16.5	373	480	43%
6	251	6.8	17.0	68	181	16%
7	325	3.4	24.5	86	193	17%
8	316	3.7	25.5	83	186	17%
9	302	4.3	29.0	84	205	19%
10	329	3.1	29.5	89	189	17%
Apioninae sub-dataset (contains 114 morphospecies, 342 sequences)						
1	95	6.0	3.0	19	47	14%
2	93	7.3	4.5	21	52	15%
3	92	7.5	5.0	22	52	15%
4	87	8.2	5.5	28	61	18%
5	94	6.8	7.5	17	41	12%
6	111	3.8	9.5	13	25	7%
7	88	8.1	11.0	24	57	17%
8	129	1.9	14.5	24	29	8%
9	116	3.0	14.5	16	28	8%
10	112	3.3	15.0	13	27	8%
Ceutorhynchinae sub-dataset (contains 199 morphospecies, 491 sequences)						
1	204	5.1	1.0	17	28	6%
2	191	6.9	7.0	19	38	8%
3	206	5.0	11.0	15	24	5%
4	186	7.7	11.5	19	40	8%
5	235	2.2	13.0	37	59	12%
6	190	7.1	13.5	18	37	8%
7	178	8.5	14.0	23	64	13%
8	183	7.8	16.0	20	42	9%
9	178	8.6	16.5	23	46	9%
10	203	5.5	18.5	18	31	6%
