# Supplementary material for: Molecular Weevil Identification Project: A thoroughly curated barcode release of 1300 Western Palearctic weevil species (Coleoptera, Curculionoidea)
Source: Biodivers Data J. 2023 Jan 24;11:e96438. doi: 10.3897/BDJ.11.e96438 (PMC10865102; doi:10.3897/BDJ.11.e96438)
Supplement: Supplementary material 1 — Material Table [file bdj-11-e96438-s001.pdf]

The table of materials, starting on page 5, provides collecting data and voucher numbers for all specimens analyzed in this study. The entries are sorted alphabetically by organism name. Each specimen received a unique sample ID (MWI internal term is "Collector's No.").

Abbreviations

ZFMK = Leibniz Institute for the Analysis of Biodiversity Change, Museum Koenig, Bonn, Germany
SDEI = Senckenberg Senckenberg German Entomological Institute, Müncheberg, Germany
NMB = Naturhistorisches Museum Basel, Basel, Switzerland
Curci = Curculio Institute, Mönchengladbach, Germany

Origin of CO1 sequences (publications)

The GenBank accession number is followed by the term "new" in brackets for new sequences published with this study. For CO1 sequences previously published within MWI / ZFMK /SDEI derived sequences, the GenBank accession number is followed by a reference number in brackets. The corresponding publications are listed on pages 3 and 4. Only MWI project-related sequences were used in this study, except one Chrysomelidae sequence for outgroup purposes.

Future updates of GenBank records

The Curculio Institute is in charge of future taxonomic updates of the GenBank records. If the provided taxonomic information in this document or species names contradicts newly added sequences, please refer to the corresponding GenBank record first to ensure the taxon name on your side is still up-to-date.

Vouchers: pinned reference vouchers, freezed tissue in EtOH, freezed DNA

- MWI pinned voucher reference collection: "MWI Collection" within Coleoptera section of the ZFMK, available on request via sample ID. ZFMK tissue number (ZFMK-TIS-nnnn) and DNA extract number (ZFMK-DNA-nnnn) from the ZFMK Biobank are listed, if available. Especially for collector Peter Sprick (PSP in sample ID) additional untouched EtOH specimens are available at ZFMK tissue bank from the same collection spot. Availability can be requested via ZFMK via sample id.

- SDEI pinned voucher reference collection: "MWI Collection" within Coleoptera section at of the NMB. DNA extracts are stored at SDEI and available via sample ID. 91 sequences have been provided from SDEI and incorporated to this study.

Sample ID naming scheme for specimens

1) ZFMK Cryptorhynchinae projects (2008-2011)

The specimen IDs mainly include a single or triple character for country, island, or region followed by a consecutive number and the first three characters of the *initial* species determination as a suffix, for example, I-0129-rol (*Dichromacalles rolletii* from Italy, 129th sample). The last sample of the batch is GR-953-bor (*Dichromacalles boroveci* from Greece, 953rd sample).

Additional 138 specimens from ZFMK Biobank were processed to enhance the MWI dataset. Two different naming schemes were applied:
Country prefix and continued number from the last ZFMK Cryptorhynchinae publication; the numbers range is between ES1001 and IT1098c.
Country prefix plus hyphen plus consecutive number, all samples from Italy, numbers range is between IT-0001w and IT-0050w.

2) ZFMK MWI (2011-2016)

The specimen IDs are built with a consecutive number followed by a three-letter suffix; the three-letter suffix is the abbreviated collector´s name; MWI sample IDs range from 1-PST up to 2903-PST. In previous MWI-related publications, the sample IDs were often followed by a suffix, for example, 2756-PST_26853. The suffix was mainly the ZFMK tissue ID number, intended for the collectors to recognize their specimens in the published trees. We have kept the suffix in this material table for DNAtype samples only. Abbreviation of the most active collectors: PST=Peter Stüben (DE), JKR/KRA=Jiri Krátky (CZ), PSP=Peter Sprick (DE), CBR=Carlo Braunert (LUX), RGO=Rafal Gosik (PL), HAR=Julien Haran (FR).

3) SDEI MWI follow up project (2017- ongoing)

The MWI naming scheme continued. The dataset of this barcode release contains 91 sequences provided by SDEI.
The SDEI sample IDs are 2906-PST and above.

References for previously published GenBank records

The GenBank acc. numbers in the table of materials, starting at page 5, are followed by a reference number in square bracktes. This reference number corresponds to a publication listed below [Ref.].

**Ref.**	**Publication**
[01]	Astrin, J.J. & Stüben, P. (2008): Phylogeny in cryptic weevils: molecules, morphology and new genera of western Palaearctic Cryptorhynchinae (Coleoptera: Curculionidae) - *Invertebrate Systematics* 22: 503-522. doi: 10.1071/IS07057
[02]	Stüben, P.E. & Astrin, J.J. (2011): *Aeoniacalles tabladoensis* sp. n. and *Aeoniacalles aeonii* bodegensis (Stüben, 2000) resyn. - Canary Islands (Coleoptera: Curculionidae: Cryptorhynchinae) - *Weevil News* 68: 1-5.
[03]	Stüben, P.E., Torres, J.L., Astrin, J.J. (2011): *Kyklioacalles alcornocalensis* sp.n. from Spain (Cádiz) (Coleoptera: Curculionidae: Cryptorhynchinae) - *Weevil News* 65: 1-5.
[04]	Astrin, J.J., Stüben, P.E., Misof, B., Wägele, J.W., Gimnich, F., Raupach, M.J., Ahrens, D. (2012): Exploring diversity in cryptorhynchine weevils (Coleoptera) using distance-, character- and tree-based species delineation - *Molecular phylogenetics and evolution* 63(1): 1-14. doi: 10.1016/j.ympev.2011.11.018
[05]	Stüben, P.E. & Astrin, J.J. (2012): Integrative Taxonomy, Phylogeny, and New Species of the Weevil Genus *Onyxacalles* Stüben (Coleoptera: Curculionidae: Cryptorhynchinae) - *Psyche* 2012: 1-22. doi: 10.1155/2012/654948
[06]	Stüben, P.E. (2012): *Kyklioacalles flavomaculatus* sp.n. from Morocco (Coleoptera: Curculionidae: Cryptorhynchinae) - *Weevil News* 73: 1-4.
[07]	Stüben, P.E. (2012): *Calacalles bandamaensis* sp.n. von Gran Canaria (Kanarische Inseln) (Coleoptera: Curculionidae: Cryptorhynchinae) - *Weevil News* 83: 1-8.
[08]	Borovec, R. & Bahr, F. (2012): *Omias winkelmanni* sp. n. aus Griechenland (Coleoptera: Curculionidae: Entiminae) - *Weevil News* 78: 1-3.
[09]	Stüben, P.E., Schütte, A., Astrin, J.J. (2013): Molecular phylogeny of the weevil genus *Dichromacalles* Stüben (Curculionidae: Cryptorhynchinae) and description of a new species - *Zootaxa* 3718(2): 101-127. doi: 10.11646/zootaxa.3718.2.1
[10]	Stüben, P.E. (2013): *Calacalles* (Nanoacalles) *lepensis* sp.n. von La Gomera - *Snudebiller* 14(216): 1-8.
[11]	Stüben, P.E. & Behne, L. (2013): *Lixus* (Compsolixus) *erysimi* sp.n. von Gran Canaria (Kanarische Inseln) (Coleoptera: Curculionidae: Lixinae) - *Snudebiller* 14(233): 1-5.
[12]	Stüben, P.E. & Schütte, A. (2013): Die Wiederentdeckung von *Acalles xerampelinus* Wollaston, 1864, und die Stellung im phylogenetischen System der kanarischen Cryptorhynchinae (Curculionidae) - *Snudebiller* 14(218): 1-14.
[13]	Stüben, P.E. & Schütte, A. (2014): Zwei neue Arten aus dem *Thamiocolus wollastoni* – Komplex von den Kanarischen Inseln (Coleoptera: Curculionidae: Ceutorhynchinae) - *Snudebiller* 15(230): 1-9.
[14]	Stüben, P.E. (2014): *Dichromacalles* (s.str.) *andalusiensis* sp.n. aus Spanien (Coleoptera: Curculionidae: Cryptorhynchinae) - *Snudebiller* 15(223): 1-5.
[15]	Stüben, P.E. (2014): *Echinodera* (Ruteria) *lusitanica* sp.n. aus Portugal (Coleoptera: Curculionidae: Cryptorhynchinae) - *Snudebiller* 15(228): 1-5.
[16]	Stüben, P.E. & Schütte, A. (2014): *Silvacalles* (s. str.) *carlinavorus* sp.n. from La Gomera (Canary Island) (Coleoptera: Curculionidae: Cryptorhynchinae) - *Snudebiller* 15(219): 1-8.
[17]	Schütte, A. & Stüben, P.E. (2015): Molecular systematics and morphological identification of the cryptic species of the genus *Acalles* Schoenherr, 1825, with descriptions of new species (Coleoptera: Curculionidae: Cryptorhynchinae) - *Zootaxa* 3915(1): 1-51. doi: 10.11646/zootaxa.3915.1.1
[18]	Bahr, F. & Winkelmann, H. (2015): *Psallidium* (Axyraeus) *talparum* sp. n. aus Griechenland 7. Beitrag zur Kenntnis der griechischen Rüsselkäfer (Coleoptera: Curculionidae: Entiminae) - *Snudebiller* 16(235): 1-5.
[19]	Germann, C., Wolf, I., Schütte, A. (2015): *Echinodera* (Ruteria) *soumasi* sp. n. from Greece (Coleoptera, Curculionidae) - *Mitteilungen der Schweizerischen Entomologischen Gesellschaft* 88: 285-293. doi: 10.5281/zenodo.33992
[20]	Stüben, P.E. (2015): *Auletobius* (Canarauletes) *garajonay* sp.n. from La Gomera (Canary Islands) - *Snudebiller* 16(244): 1-9.
[21]	Stüben, P.E. & Schütte, A. (2015): *Torneuma korwitzi* sp.n. from Madeira (Coleoptera: Curculionidae: Cryptorhynchinae) - *Snudebiller* 16(236): 1-8.

[22] Stüben, P.E. (2015): Zwei neue *Calacalles* (Nanoacalles)-Arten von La Palma (Kanarische Inseln) (Coleoptera: Curculionidae: Cryptorhynchinae) - *Snudebiller* 16(243): 1-10.

[23] Stüben, P.E. (2015): *Acalles abstersus* Bohemann, 1837 resyn. – die dritte *Kyklioacalles*-Art des Subgenus Palaeoacalles Stüben, 2003 für Deutschland (Coleoptera: Curculionidae: Cryptorhynchinae) - *Snudebiller* 16 (232): 1-18.

[24] Stüben, P. & Bayer, C. (2015): New nomenclatural and taxonomic acts, and Comments - *Snudebiller* 16(246): 1-8.

[25] Kratky, J. (2016): *Ceutorhynchus paroliniae* sp.n. from Gran Canaria (Coleoptera, Curculionidae, Ceutorhynchinae) - *Snudebiller* 17(250): 1-6.

[26] Stüben, P.E. & Kratky, J. (2016): *Hesperorrhynchus glutinosus* sp.n. and *H. palmensis* sp.n. with a review of all *Hesperorrhynchus* species from the Macaronesian Islands - *Snudebiller* 17(247): 1-12.

[27] Stüben, P.E. (2016): *Ceutorhynchus descurainiae* sp.n. von La Gomera und Tenerife (Kanarische Inseln) (Coleoptera: Curculionidae: Ceutorhynchinae) - *Snudebiller* 17(252): 1-9.

[28] Stüben, P.E., Schütte, A., López, H., Astrin, J.J. (2016): Molecular and morphological systematics of soil-inhabiting Cryptorhynchinae of the genus *Acallorneuma* and the tribe Torneumatini (Coleoptera: Curculionidae), with description of two new species - *Contributions to Entomology* 66(2): 169 – 199.

[29] Caldara, R., O'Brien, C., Meregalli, M. (2017): A phylogenetic analysis of the aquatic weevil tribe Bagoini (Coleoptera: Curculionidae) based on morphological characters of adults - *Zootaxa* 4287(1): 1-63. doi: 10.11646/zootaxa.4287.1.1

[30] Haran, J., Schütte, A., Friedman, A.L. (2017): A review of *Smicronyx* Schoenherr (Coleoptera, Curculionidae) of Israel, with description of two new species - *Zootaxa 4237*(1): 17-40. doi: 10.11646/zootaxa.4237.1.2

[31] Stüben, P.E. (2018): Beschreibung neuer Arten aus der Gattung *Caulotrupis* Wollaston, 1854 (Coleoptera: Curculionidae: Cossoninae) von den Makaronesischen Inseln - *Contributions to Entomology* 68(1): 083 – 096. doi: 10.21248/contrib.entomol.68.1.083-096

[32] Stüben, P.E. (2018): *Pseudocaulotrupis* gen. nov. and two new species in the Azores (Coleoptera: Curculionidae: Cossoninae) - *Snudebiller* 19(274): 1-8.

[33] Stüben, P.E. & Schütte, A. (2018): Revision of the genus *Caulotrupis* from Macaronesian Islands (Coleoptera: Curculionidae: Cossoninae) - *Snudebiller* 19(272): 1-38.

[34] Stüben, P.E. (2018): The Cryptorhynchinae of the Western Palearctic / Die Cryptorhynchinae der Westpaläarktis (Coleoptera: Curculionidae). 1st edition, Curculio Institute, Mönchengladbach: 518pp. ISBN: 9783000580383, URL: https://www.curci.de/?beitrag=281

[35] Verdugo, A., Stüben, P.E., Torres, J.L., Coello, P. (2020): The relationship among the *Auletobius* species of the subgenus Canarauletes and the description of a new species (Attelabidae: Rhynchitinae) - *Contibutions to Entomology* 70(1): 189-196. doi: 10.21248/contrib.entomol.70.1.189-196

[36] Stüben, P.E. & Kramp, K. (2019): Neue *Echinodera* aus Griechenland – Beitrag zur integrativen Taxonomie (Coleoptera: Curculionidae: Cryptorhynchinae) - *Contibutions to Entomology* 69(2): 1-12. doi: 10.21248/contrib.entomol.69.2.319-330

[37] Stüben, P.E., Schütte, A., Andrade, M. (2020): Two new species of *Cyphocleonus* from Macaronesian Islands (Coleoptera: Curculionidae: Lixinae) - *Bocagiana* 2020(248): 1-16.

[38] Machado, A. & Suarez, D. (2020): The Genus *Herpisticus* Germar, 1823 from the Canary Islands (Coleoptera: Curculionidae: Entiminae: Tanymecini). *Graellsia* 76(1): e104. doi: 10.3989/graellsia.2020.v76.250

[39] Germann, C. & Schütte, A. (2021): Description of a new species of the genus *Echinodera* Wollaston, 1863 (Coleoptera: Curculionidae) and supplement to the weevil fauna of Kefalonia Island (Greece) - *Contributions to Entomology* 72(2): 301-320. doi: 10.21248/contrib.entomol.71.2.301-320

[40] Stüben, P.E., Schütte, A., Astrin, J.J. (2021): Barcoding and interspecific relationships of Macaronesian Weevils (Coleoptera: Curculionoidea) - *Contributions to Entomology* 71(1): 127-135. doi: 10.21248/contrib.entomol.71.1.127-135

[new] this barcode release

**Name**<br>**Authority**<br>**Additional Information**	**GenBank Acc No (Ref.)**<br>**Specimen ID**<br>**Sequence Length**	**ZFMK Tissue ID**<br>**ZFMK DNA Sample ID**<br>**(SDEI DNA Sample ID)**	**Locality, GPS, Collection Date, Plant, Collector, Identifier**
Acalles alcarazensis<br>Stüben, 2009	GU987921 [01]<br>E-457-alc<br>615nt	ZFMK-TIS-cE457<br>ZFMK-DNA-0100400282	Spain, Castilla-La Mancha, Sierra de Alcaraz, 12 km SE of Alcaraz near Mt. Almenara, N38°34'17" W02°25'53", 1650m, 22-Apr-2003, *Amelanchier ovalis, Acer*, leg. Stüben,P., det. Stüben,P.
Acalles alcarazensis<br>Stüben, 2009	GU987941 [01]<br>E-579-alc<br>655nt	ZFMK-TIS-cE579<br>ZFMK-DNA-0100404964	Spain, Castilla-La Mancha, Sierra de Alcaraz, 12 km SE of Alcaraz near Mt. Almenara, N38°34'17" W02°25'53", 1650m, 22-Apr-2003, *Amelanchier ovalis, Acer*, leg. Stüben,P., det. Stüben,P.
Acalles alcarazensis<br>Stüben, 2009	GU987942 [01]<br>E-580-alc<br>658nt	ZFMK-TIS-cE580<br>ZFMK-DNA-0100404265	Spain, Castilla-La Mancha, Sierra de Alcaraz, 12 km SE of Alcaraz near Mt. Almenara, N38°34'17" W02°25'53", 1650m, 22-Apr-2003, *Amelanchier ovalis, Acer*, leg. Stüben,P., det. Stüben,P.
Acalles almeriaensis<br>Stüben, 2001	KF680246 [17]<br>1280-PST<br>658nt	ZFMK-TIS-4096<br>ZFMK-DNA-0100426197	Spain, Malaga, S of Fornes, Sierra de Almijara (N. P.), N36°53'21" W03°51'14", 1002m, 08-Mai-2013, *Quercus, Viburnum*, similar to buckthorn, sieving, leg. Stüben,P. & Schütte,A., det. Stüben,P.
Acalles almeriaensis<br>Stüben, 2001	EU286497 [01]<br>E-0186-alm<br>658nt	ZFMK-TIS-cE0186<br>ZFMK-DNA-0100400123	Spain, Almeria, 11 km NW of Laujar de Andarax, Sierra Nevada, Bayarcal, N37°02'27" W03°00'12", 1291m, 07-Apr-2007, *Quercus ilex,* broom, *Euphorbia*, leg. Astrin,J. & Stüben,P., det. Stüben,P.
Acalles almeriaensis<br>Stüben, 2001	GU987825 [01]<br>E-0192-alme<br>658nt	ZFMK-TIS-cE0192<br>ZFMK-DNA-0100400322	Spain, Granada, 5 km N of Laujar de Andarax, Sierra Nevada, N37°02'16" W02°54'51", 1597m, 07-Apr-2007, *Erinacea anthyllis*, leg. Astrin,J. & Stüben,P., det. Stüben,P.
Acalles almeriaensis<br>Stüben, 2001	GU987949 [01]<br>E-601-alm<br>658nt	ZFMK-TIS-cE601<br>ZFMK-DNA-0100404318	Spain, Teruel, S. Javalambre, Fuente la Risca near Arcos de las Salinas, N39°59'56" W01°01'21", 1121m, 17-Jun-2008, *Amelanchier ovalis, Acer monspessulanum, Erinacea anthyllis, Ulex*, leg. Astrin,J., det. Stüben,P.
Acalles almeriaensis<br>Stüben, 2001	GU987951 [01]<br>E-603-alm<br>658nt	ZFMK-TIS-cE603<br>ZFMK-DNA-0100404940	Spain, Teruel, near Mora de Rubielos, N40°19'08" W00°43'19", 1369m, 18-Jun-2008, *Erinacea anthyllis*, leg. Astrin,J., det. Stüben,P.
Acalles almeriaensis<br>Stüben, 2001	GU987954 [01]<br>E-608-alm<br>658nt	ZFMK-TIS-cE608<br>ZFMK-DNA-0100404930	Spain, Castellon, Morella, Barranco de la Bota, N40°33'12" W00°00'27", 814m, 21-Jun-2008, *Quercus ilex, Hedera helix*, leg. Astrin,J., det. Stüben,P.
Acalles almeriaensis<br>Stüben, 2001	GU988058 [01]<br>E-759-alm<br>658nt	ZFMK-TIS-cE759<br>ZFMK-DNA-0100405035	Spain, Malaga, NE of Malaga, Sierra de Tejeda, Alcaucin, N36°54'54" W04°05'27", 751m, 07-Jan-2009, *Viburnum tinus*, stream valley, leg. Stüben,P., det. Stüben,P.
Acalles almeriaensis<br>Stüben, 2001	GU988054 [01]<br>E-760-alm<br>658nt	ZFMK-TIS-cE760<br>ZFMK-DNA-0100405041	Spain, Malaga, NE of Malaga, Sierra de Tejeda, Alcaucin, N36°56'13" W04°04'59", 1020m, 07-Jan-2009, *Quercus ilex*, leg. Stüben,P., det. Stüben,P.
Acalles asniensis<br>Stüben, 2003	MG229716 [new]<br>1586-JKR<br>658nt	ZFMK-TIS-3634<br>ZFMK-DNA-0155635632	Morocco, Haut Atlas, Oukaimeden, N31°12'10" W07°52'13", 2691m, 20-Apr-2013, *Alyssum spinosum*, sieving, leg. Krátky,J., det. Stüben,P.
Acalles asniensis<br>Stüben, 2003	GU988129 [01]<br>E-882-asn<br>658nt	ZFMK-TIS-cE882<br>ZFMK-DNA-0100405508	Morocco, High Atlas, E of Asni, Oukaimeden, N31°12'14" W07°52'21", 2646m, 01-Mai-2009, *Erinacea* , under stones, leg. Stüben,P., det. Stüben,P.
Acalles asniensis<br>Stüben, 2003	GU988130 [01]<br>E-884-asn<br>658nt	ZFMK-TIS-cE884<br>ZFMK-DNA-0100405510	Morocco, High Atlas, SE of Asni, Tacheddirt (near Imlil), N31°09'07" W07°50'18", 2420m, 02-Mai-2009, broom*, Daphne*, leg. Stüben,P., det. Stüben,P.
Acalles biokovoensis<br>Stüben, 2008	GU987819 [01]<br>HR-0330-bio<br>658nt	ZFMK-TIS-cHR0330<br>ZFMK-DNA-0100400206	Croatia, Dalmatia, 3 km NE of Makarska, Biokovo Mts., "Vosac", N43°18'53" E17°03'09", 1367m, 11-Jul-2007, *Fagus*, limestone, leg. Stüben,P., det. Stüben,P.

**Name**<br>**Authority**<br>**Additional Information**	**GenBank Acc No (Ref.)**<br>**Specimen ID**<br>**Sequence Length**	**ZFMK Tissue ID**<br>**ZFMK DNA Sample ID**<br>**(SDEI DNA Sample ID)**	**Locality, GPS, Collection Date, Plant, Collector, Identifier**
Acalles biokovoensis<br>Stüben, 2008	GU987820 [01]<br>HR-0341-bio<br>658nt	ZFMK-TIS-cHR0341<br>ZFMK-DNA-0100400355	Croatia, Dalmatia, 4 km E of Makarska, Biokovo Mts., "Mt. Sinjal", N43°18'39" E17°03'49", 1320m, 16-Jul-2007, *Fagus*, limestone, leg. Stüben,P., det. Stüben,P.
Acalles breiti<br>A. & F. Solari, 1909	GU987911 [01]<br>E-542-bre<br>658nt	ZFMK-TIS-cE542<br>ZFMK-DNA-0100400624	Spain, Mallorca, Serra de Tramuntana, 2.5 km NO Puig Major, N39°48'59" E02°49'26", 29-Dez-2007, *Quercus ilex*, leg. Astrin,J., det. Astrin,J.
Acalles breiti<br>A. & F. Solari, 1909	GU987912 [01]<br>E-543-bre<br>658nt	ZFMK-TIS-cE543<br>ZFMK-DNA-0100400623	Spain, Mallorca, Serra de Tramuntana, Sa Calobra, N39°51'04" E02°48'23", 27-Dez-2007, *Quercus ilex*, leg. Astrin,J., det. Astrin,J.
Acalles camelus<br>(Fabricius, 1792)	MG229698 [new]<br>1392-PST<br>658nt	ZFMK-TIS-4688<br>ZFMK-DNA-0155628541	Italy, Abruzzo, P.N. Majella near Gamberale, Sant'António, N41°53'53" E14°10'44", 1510m, 24-Aug-2013, *Fagus*, sieving, leg. Stüben,P., det. Stüben,P.
Acalles camelus<br>(Fabricius, 1792)	MG229785 [new]<br>2417-JKR<br>658nt	ZFMK-TIS-25994<br>ZFMK-DNA-0171600639	Czech Republic, Bohemia, Horni Morava, N50°10'15" E16°49'22", 780m, 25-Aug-2014, *Fagus sylvatica*, collecting by hand, leg. Krátky,J., det. Krátky,J.
Acalles camelus<br>(Fabricius, 1792)	MG229790 [new]<br>2446-JKR<br>658nt	ZFMK-TIS-26023<br>ZFMK-DNA-0171600668	Slovakia, Bardejov, Gerlachov, N49°18'55" E21°05'57", 420m, 21-Sep-2014, *Fagetum*, sieving, leg. Pavel,F., det. Krátky,J.
Acalles camelus<br>(Fabricius, 1792)	MG229826 [new]<br>2897-PST<br>658nt	ZFMK-TIS-25903<br>ZFMK-DNA-0169166897	Slovenia, NE of Kobarid, above Drezniske Ravne, Planina Zaplec, N46°16'59" E13°37'49", 1186m, 29-Jun-2016, Fagus forest, sifting, leg. Stüben,P, det. Stüben,P.
Acalles camelus<br>(Fabricius, 1792)	MG229829 [new]<br>2902-PST<br>658nt	ZFMK-TIS-25909<br>ZFMK-DNA-0169166892	Germany, Baden-Wuerttemberg (BW), Kaiserstuhl, W of Bahlingen, Silberbrunnen, N48°06'58" E07°42'40", 299m, 15-Jul-2016, *Fagus, Fraxinus*, sifting, leg. Stüben,P, det. Stüben,P.
Acalles camelus<br>(Fabricius, 1792)	GU987814 [01]<br>A-0228-cam<br>658nt	ZFMK-TIS-cA0228<br>ZFMK-DNA-0100400104	Austria, Lower Austria, Merkersdorf, National Park Thayatal; Carpinetum, N48°32' E16°17', 18-Apr-2006, *Carpinetum*, leg. Stejskal,R., det. Stejskal,R.
Acalles camelus<br>(Fabricius, 1792)	KF680229 [17]<br>CZ1054<br>658nt	9637<br>ZFMK-TIS-cCZ1054<br>ZFMK-DNA-0112704615	Czech Republic, SE Moravia (UH), Lopenik, Bile Karpaty Mts., 48°56'00"N 17°46'00"E, 727m, 15-Mai-2009, *Fagus*, leg. Kresl,P., det. Kresl,P.
Acalles camelus<br>(Fabricius, 1792)	GU988032 [01]<br>D-666-cam<br>658nt	ZFMK-TIS-cD666	Germany, Ruegen Isl. (MV), Sassnitz near Rusewase, N54°32'29" E13°37'46", 144m, 15-Aug-2008, *Fagus*, leg. Astrin,J., det. Stüben,P.
Acalles camelus<br>(Fabricius, 1792)	EU286511 [01]<br>HR-0336-cam<br>658nt	ZFMK-TIS-cHR0336<br>ZFMK-DNA-0100400114	Croatia, Dalmatia, 8 km E of Karlobag, Velebit Mts., Stupacinovo, N44°32'41" E15°09'58", 1049m, 14-Jul-2007, *Fagus*, limestone, leg. Stüben,P., det. Stüben,P.
Acalles camelus<br>(Fabricius, 1792)	GU987808 [01]<br>I-0016-cam<br>658nt	ZFMK-TIS-cI0016<br>ZFMK-DNA-0100400023	France, Isere, 9 km N of Grenoble, Mont St. Martin, N45°16'15" E05°40'44", 813m, 12-Aug-2005, *Fagus, Fraxinus, Quercus*, limestone, leg. Stüben,P., det. Stüben,P.
Acalles camelus<br>(Fabricius, 1792)	EU286447 [01]<br>I-0026-cam<br>658nt	ZFMK-TIS-cI0026<br>ZFMK-DNA-0100400755	France, Isere, 2 km SE of Lans en Vercors, Montagne de Lans, N45°06'45" E05°36'21", 1352m, 12-Aug-2005, *Abies, Fagus, Fraxinus*, limestone: mixed forest, leg. Stüben,P., det. Stüben,P.
Acalles camelus<br>(Fabricius, 1792)	GU987776 [01]<br>I-0034-cam<br>658nt	ZFMK-TIS-cI0034<br>ZFMK-DNA-0100400882	France, Isere, 14 km N of Grenoble, Massif de la Chartreus, NW of Col de Porte, N45°18'40" E05°45'17", 1649m, 13-Aug-2005, *Abies, Fagus, Fraxinus*, limestone (tree line), leg. Stüben,P., det. Stüben,P.

**Name**<br>**Authority**<br>**Additional Information**	**GenBank Acc No (Ref.)**<br>**Specimen ID**<br>**Sequence Length**	**ZFMK Tissue ID**<br>**ZFMK DNA Sample ID**<br>**(SDEI DNA Sample ID)**	**Locality, GPS, Collection Date, Plant, Collector, Identifier**
Acalles camelus<br>(Fabricius, 1792)	GU987971 [01]<br>I-628-cam<br>658nt	ZFMK-TIS-cI628<br>ZFMK-DNA-0100404915	Italy, Campania, Cilento, 6 km SE of Vallo d. Lucania, M. Sacro o Gelbison, N40°12'41" E15°19'42", 1544m, 30-Jun-2008, *Fagus*, leg. Stüben,P., det. Stüben,P.
Acalles camelus<br>(Fabricius, 1792)	GU987978 [01]<br>I-637-cam<br>658nt	ZFMK-TIS-cI637<br>ZFMK-DNA-0100404911	Italy, Basilicata, Monte Pollino, 9 km SE of Rotonda, Rif. de Gasperi, N39°54'37" E16°07'15", 1486m, 05-Jul-2008, *Fagus*, leg. Stüben,P., det. Stüben,P.
Acalles camelus<br>(Fabricius, 1792)	GU987989 [01]<br>I-656-cam<br>658nt	ZFMK-TIS-cI656<br>ZFMK-DNA-0100404882	Italy, Abruzzo, P.N. Majella, 11 km N of Roccaraso, Bosco di S. Antonio, N41°56'27" E14°01'41", 1321m, 14-Jul-2008, *Pyrus/Acer*, old forest, leg. Stüben,P., det. Stüben,P.
Acalles camelus<br>(Fabricius, 1792)	GU988028 [01]<br>S-662-cam<br>658nt	ZFMK-TIS-cS662<br>ZFMK-DNA-0100404814	Sweden, Simrishamn, Stenshuvud N.P., N55°39'15" E14°15'50", 80m, 12-Aug-2008, leg. Astrin,J., det. Stüben,P.
Acalles camelus<br>(Fabricius, 1792)	GU987813 [01]<br>SLO-0227-cam<br>658nt	ZFMK-TIS-cSLO0227<br>ZFMK-DNA-0100400103	Slovenia, Apasko Polje, Podgorje, Podgorje env., 30 km NE of Maribor; Quercus, Carpinus, N46°42' E15°55', 05-Aug-2006, *Quercus, Carpinus*, leg. Stejskal,R., det. Stejskal,R.
Acalles camelus<br>(Fabricius, 1792)	GU987842 [01]<br>SLO-0229-cam<br>615nt	ZFMK-TIS-cSLO0229<br>ZFMK-DNA-0100400299	Slovenia, Drava Valley, Cresnjevec, Cresnjevec env., 22 km W of Maribo, N46°32' E15°22', 04-Aug-2006, *Fagus, Acer, Abies*, ravine forest, leg. Stejskal,R., det. Stejskal,R.
Acalles cazorlaensis<br>Stüben, 2004	KF680250 [17]<br>1312-PST<br>658nt	ZFMK-TIS-4128<br>ZFMK-DNA-0100426164	Spain, Jaen, Sierra de Cazorla, S of Cazorla near El Chorro, N37°50'20" W02°59'26", 1581m, 16-Mai-2013, *Erinacea anthyllis, (Euphorbia nicaensis)*, sieving, leg. Stüben,P. & Schütte,A., det. Stüben,P.
Acalles cazorlaensis<br>Stüben, 2004	GU987884 [01]<br>E-464-caz<br>658nt	ZFMK-TIS-cE464<br>ZFMK-DNA-0100400998	Spain, Andalucia, Sierra de Segura (Sierra de Pozo), 12 km SE of Cazorla, N37°52'53" W02°52'39", 1300m, 17-Apr-2003, *Quercus ilex*, leg. Stüben,P., det. Stüben,P.
Acalles cazorlaensis<br>Stüben, 2004	GU987946 [01]<br>E-596-caz<br>658nt	ZFMK-TIS-cE596<br>ZFMK-DNA-0100404947	Spain, Jaen, Cazorla, El Chorro, N37°50'25" W02°59'24", 1559m, 12-Jun-2008, *Erinacea anthyllis*, leg. Astrin,J., det. Stüben,P.
Acalles cazorlaensis<br>Stüben, 2004	GU987948 [01]<br>E-599-caz<br>658nt	ZFMK-TIS-cE599<br>ZFMK-DNA-0100404944	Spain, Jaen, Cazorla near El Chorro, N37°50'52" W02°57'34", 1610m, 12-Jun-2008, *Erinacea anthyllis*, leg. Astrin,J., det. Stüben,P.
Acalles cytisi<br>Stüben, 2004	GU987883 [01]<br>E-462-cyt<br>658nt	ZFMK-TIS-cE462<br>ZFMK-DNA-0100400996	Spain, Caceres, 28 km N of Plasencia, Montes de Tras la Sierra, Puerto de Honduras, N40°13'58" W05°53'02", 1273m, 09-Apr-2004, *Cytisus*, leg. Stüben,P., det. Stüben,P.
Acalles cytisi<br>Stüben, 2004	GU988104 [01]<br>E-845-cyt<br>658nt	ZFMK-TIS-cE845<br>ZFMK-DNA-0100404524	Spain, Avila, NW of Arenas de San Pedro, Gredos NP, "la plataforma", N40°16'06" W05°14'22", 1880m, 24-Apr-2009, *Cytisus*, leg. Astrin,J., det. Stüben,P.
Acalles cytisi<br>Stüben, 2004	GU988105 [01]<br>E-847-cyt<br>627nt	ZFMK-TIS-cE847<br>ZFMK-DNA-0100405543	Spain, Avila, E of Bejar, S of La Hoya, La Covatilla, N40°21'26" W05°41'06", 1950m, 24-Apr-2009, *Cytisus*, leg. Astrin,J., det. Stüben,P.
Acalles dieckmanni<br>Péricart, 1989	KF680237 [17]<br>816-PST<br>658nt	ZFMK-TIS-3729<br>ZFMK-DNA-0100414275	France, Hte Corse, Vallee Restonica, N42°14'10" E09°02'10", 1324m, 25-Mai-2011, leg. Lempereur,J.-M., det. Stüben,P.
Acalles dubius<br>A. & F. Solari, 1907	KF680236 [17]<br>794-PST<br>658nt	ZFMK-TIS-3707<br>ZFMK-DNA-0100414288	Germany, Rhineland-Palatinate (RLP), E of Treis-Karden, Pommern, Pommernbachtal (river valley), N50°10'52" E07°16'42", 116m, 09-Jun-2012, *Fagus*, forest path, beating, leg. Stüben,P., det. Stüben,P.

**Name**<br>**Authority**<br>**Additional Information**	**GenBank Acc No (Ref.)**<br>**Specimen ID**<br>**Sequence Length**	**ZFMK Tissue ID**<br>**ZFMK DNA Sample ID**<br>**(SDEI DNA Sample ID)**	**Locality, GPS, Collection Date, Plant, Collector, Identifier**
Acalles dubius<br>A. & F. Solari, 1907	GU987806 [01]<br>D-0094-dub<br>658nt	ZFMK-TIS-cD0094<br>ZFMK-DNA-0100400060	Germany, Baden-Wuerttemberg (BW), Kaiserstuhl, "Auf dem Eck", N48°06'32" E07°40'04", 432m, 27-Mai-2006, *Fagus, Carpinus, Quercus*, leg. Stüben,P., det. Stüben,P.
Acalles dubius<br>A. & F. Solari, 1907	GU987881 [01]<br>D-0423-dub<br>658nt	ZFMK-TIS-cD0423<br>ZFMK-DNA-0100401007	Germany, Rhineland-Palatinate (RLP), Kaltenborn, Hohe Acht, N50°23'08" E07°01'25", 605m, 03-Nov-2007, *Quercus, Fagus*, leg. Stüben,P., det. Stüben,P.
Acalles dubius<br>A. & F. Solari, 1907	GU987767 [01]<br>M-0066-dub<br>658nt	ZFMK-TIS-cM0066<br>ZFMK-DNA-0100400929	Germany, Rhineland-Palatinate (RLP), Bausenberg, Niederzissen, N50°27'52" E07°13'29", 291m, 17-Sep-2005, *Quercus, Carpinus, Fagus*, leg. Stüben,P., det. Stüben,P.
Acalles echinatus<br>(Germar, 1824)	KF680257 [17]<br>461-PST<br>658nt	ZFMK-TIS-20247<br>ZFMK-DNA-0100417598	Italy, Vicenza, Monti Berici, dintorni Villabalzana, N45°26' E11°31', 385m, 26-Feb-2011, leg. Diotti,L., det. Stüben,P.
Acalles echinatus<br>(Germar, 1824)	KF680241 [17]<br>1221-JKR<br>658nt	ZFMK-TIS-3557<br>ZFMK-DNA-0100426063	Slovakia, Driencansky kras, Slizke env., N48°29'52" E20°05'28", 284m, 29-Sep-2012, *Quercetum*, sieving, leg. Krátky,J., det. Krátky,J.
Acalles echinatus<br>(Germar, 1824)	GU987786 [01]<br>A-0093-ech<br>609nt	ZFMK-TIS-cA0093<br>ZFMK-DNA-0100400702	Austria, Lower Austria, Merkersdorf, National Park Thayatal, N48°32' E16°17', 18-Apr-2006, *Carpinetum*, leg. Stejskal,R., det. Stejskal,R.
Acalles echinatus<br>(Germar, 1824)	GU987871 [01]<br>BUL-0344-ech<br>618nt	ZFMK-TIS-cBUL0344<br>ZFMK-DNA-0100400442	Bulgaria, Gabrovo West, Aprilci, N42°50'26" E24°55'10", 515m, 08-Jun-2007, leg. Bialooki, det. Bialooki
Acalles echinatus<br>(Germar, 1824)	GU987872 [01]<br>BUL-0345-ech<br>612nt	ZFMK-TIS-cBUL0345<br>ZFMK-DNA-0100401019	Bulgaria, Gabrovo West, Stokite, N42°51'47" E25°04'19", 372m, 11-Jun-2007, leg. Bialooki, det. Bialooki
Acalles echinatus<br>(Germar, 1824)	GU987843 [01]<br>CZ-0231-ech<br>658nt	ZFMK-TIS-cCZ0231<br>ZFMK-DNA-0100400301	Czech Republic, Moravia mer., Pavlov env, Palava Protected Landscape Area, N48°52' E16°40', 28-Mai-2006, *Tilia, Carpinus, Quercus*, leg. Stejskal,R., det. Stejskal,R.
Acalles echinatus<br>(Germar, 1824)	GU987816 [01]<br>D-0234-ech<br>658nt	ZFMK-TIS-cD0234<br>ZFMK-DNA-0100400383	Germany, Bavaria (BY), N of Karlstadt, Gambach, NSG Grainberg-Kalbenstein, N49°59'28" E09°45'45", 260m, 19-Mai-2006, *Fagus*, arid area, leg. Stüben,P., det. Stüben,P.
Acalles echinatus<br>(Germar, 1824)	EU286510 [01]<br>HR-0334-ech<br>658nt	ZFMK-TIS-cHR0334<br>ZFMK-DNA-0100400113	Croatia, Dalmatia, 6 km E of Karlobag, Velebit Mts., Ostarijska vrata, N44°31'45" E15°08'34", 927m, 14-Jul-2007, *Fagus*, limestone, leg. Stüben,P., det. Stüben,P.
Acalles echinatus<br>(Germar, 1824)	GU987870 [01]<br>HR-0338-ech<br>658nt	ZFMK-TIS-cHR0338<br>ZFMK-DNA-0100400416	Croatia, Dalmatia, 8 km E of Karlobag, Velebit Mts., Stupacinovo, N44°32'41" E15°09'58", 1049m, 14-Jul-2007, *Fagus*, limestone, leg. Stüben,P., det. Stüben,P.
Acalles echinatus<br>(Germar, 1824)	KF680227 [17]<br>IT1049<br>658nt	ZFMK-TIS-cIT1049<br>ZFMK-DNA-0112704620	Italy, Monti Berici (VI), dint. San Gottardo, 45°27'00"N 11°28'00"E, 25-Feb-2009, leg. Diotti,L., det. Diotti,L.
Acalles echinatus<br>(Germar, 1824)	GU988033 [01]<br>R-668-ech<br>658nt	ZFMK-TIS-24651<br>ZFMK-DNA-0100404732	Russia, Moskow, near Ivanteevka, N55°59'18" E37°59'00", 170m, 01-Sep-2008, leg. Astrin,J., det. Stüben,P.
Acalles echinatus<br>(Germar, 1824)	GU988034 [01]<br>R-669-ech<br>658nt	ZFMK-TIS-24651<br>ZFMK-DNA-0100404720	Russia, Moskow, Fryazino, Grebnevo, N55°57'01" E38°05'05", 160m, 02-Sep-2008, *Betula, Tilia*, leg. Astrin,J., det. Stüben,P.

**Name**<br>**Authority**<br>**Additional Information**	**GenBank Acc No (Ref.)**<br>**Specimen ID**<br>**Sequence Length**	**ZFMK Tissue ID**<br>**ZFMK DNA Sample ID**<br>**(SDEI DNA Sample ID)**	**Locality, GPS, Collection Date, Plant, Collector, Identifier**
Acalles echinatus<br>(Germar, 1824)	GU987879 [01]<br>RU-0410-ech<br>658nt	ZFMK-TIS-cRU0410<br>ZFMK-DNA-0100401009	Russia, Ivanteevka, near Moscow, N55°59'14" E37°58'37", 38m, 18-Aug-2007, *Quercus, Fagus, Corylus*, leg. Astrin,J., det. Astrin,J.
Acalles fallax<br>Boheman, 1844	KJ867591 [new]<br>1212-JKR<br>658nt	ZFMK-TIS-3548<br>ZFMK-DNA-0100426081	Czech Republic, Bohemia, sumava, Hojsova Straz, PR Lakmal, N49°13'22" E13°11'02", 712m, 26-Aug-2012, *Fagus sylvatica*, beating, leg. Krátky,J., det. Krátky,J.
Acalles fallax<br>Boheman, 1844	MG229688 [new]<br>1214-JKR<br>658nt	ZFMK-TIS-3550<br>ZFMK-DNA-0155630461	Czech Republic, Bohemia, sumava, Hojsova Straz, PR Lakmal, N49°13'22" E13°11'02", 712m, 26-Aug-2012, *Picea excelsa*, beating, leg. Krátky,J., det. Krátky,J.
Acalles fallax<br>Boheman, 1844	MG229704 [new]<br>1446-JKR<br>658nt	ZFMK-TIS-4215<br>ZFMK-DNA-0155628484	Czech Republic, Bohemia, Slatina, Belysov Mt., N49°26'56" E13°12'09", 600m, 31-Aug-2013, *quercetum*, sieving, leg. Krátky,J., det. Krátky,J.
Acalles fallax<br>Boheman, 1844	MG229779 [new]<br>2084-JKR<br>658nt	ZFMK-TIS-23724<br>ZFMK-DNA-0169169642	Czech Republic, Bohemia, Predhradi, N49°49'56" E16°02'33", 427m, 08-Mai-2014, sieving, leg. Krátky,J., det. Krátky,J.
Acalles fallax<br>Boheman, 1844	MG229784 [new]<br>2416-JKR<br>658nt	ZFMK-TIS-25993<br>ZFMK-DNA-0171600638	Czech Republic, Bohemia, Horni Morava, N50°10'15" E16°49'22", 780m, 25-Aug-2014, *Picea excelsa*, collecting by hand, leg. Krátky,J., det. Krátky,J.
Acalles fallax<br>Boheman, 1844	MG229786 [new]<br>2419-JKR<br>658nt	ZFMK-TIS-25996<br>ZFMK-DNA-0171600641	Czech Republic, Bohemia, Horni Morava, N50°10'15" E16°49'22", 780m, 25-Aug-2014, *Fagus sylvatica*, collecting by hand, leg. Krátky,J., det. Krátky,J.
Acalles fallax<br>Boheman, 1844	MG229791 [new]<br>2448-JKR<br>658nt	ZFMK-TIS-26025<br>ZFMK-DNA-0171600670	Slovakia, Bardejov, Gerlachov, N49°18'55" E21°05'57", 420m, 21-Sep-2014, *Fagetum*, sieving, leg. Pavel,F., det. Krátky,J.
Acalles fallax<br>Boheman, 1844	MG229827 [new]<br>2898-PST<br>658nt	ZFMK-TIS-25904<br>ZFMK-DNA-0169166896	Slovenia, N of Magozd to Trnova near Soca river, N46°16'54" E13°33'43", 347m, 04-Jul-2016, sifting, leg. Stüben,P, det. Stüben,P.
Acalles fallax<br>Boheman, 1844	MG229828 [new]<br>2899-PST<br>658nt	ZFMK-TIS-25905<br>ZFMK-DNA-0169166895	Croatia, E of Delnice near Dedin, N45°22'20" E14°51'24", 853m, 06-Jul-2016, *Fagus*, sifting, leg. Stüben,P, det. Stüben,P.
Acalles fallax<br>Boheman, 1844	KF680228 [17]<br>CZ1052<br>658nt	ZFMK-TIS-cCZ1052<br>ZFMK-DNA-0112704617	Czech Republic, SE Moravia (UH), Lopenik, Bile Karpaty Mts., 48°56'00"N 17°46'00"E, 727m, 15-Mai-2009, *Fagus*, leg. Kresl,P., det. Kresl,P.
Acalles fallax<br>Boheman, 1844	GU988078 [01]<br>Cz-797-fal<br>658nt	ZFMK-TIS-cCz797<br>ZFMK-DNA-0100405000	Czech Republic, Bohemia W (KT), Klenova (6645), N49°20' E13°13', 24-Aug-2008, leg. Kresl,P., det. Kresl,P.
Acalles fallax<br>Boheman, 1844	GU988093 [01]<br>D-831-fal<br>658nt	ZFMK-TIS-cD831<br>ZFMK-DNA-0100405554	Germany, North Rhine-Westphalia (NRW), N of Aachen, S. Herzogenrath, N50°51'25" E06°05'44", 262m, 28-Mrz-2009, leg. Stüben,P., det. Stüben,P.
Acalles fallax<br>Boheman, 1844	EU286508 [01]<br>HR-0332-fal<br>658nt	ZFMK-TIS-cHR0332<br>ZFMK-DNA-0100400111	Croatia, Dalmatia, 22 km SE of Gospic, Velebit Mts., Medak, N44°24'32" E15°34'13", 617m, 13-Jul-2007, *Quercus*, limestone, leg. Stüben,P., det. Stüben,P.
Acalles fallax<br>Boheman, 1844	GU987844 [01]<br>SLO-0235-fal<br>639nt	ZFMK-TIS-cSLO0235<br>ZFMK-DNA-0100400303	Slovenia, Drava Valley, Cresnjevec env., 22 km W of Maribor, N46°32' E15°22', 04-Aug-2006, *Fagus, Acer, Abies*, leg. Stejskal,R., det. Stejskal,R.

**Name**<br>**Authority**<br>**Additional Information**	**GenBank Acc No (Ref.)**<br>**Specimen ID**<br>**Sequence Length**	**ZFMK Tissue ID**<br>**ZFMK DNA Sample ID**<br>**(SDEI DNA Sample ID)**	**Locality, GPS, Collection Date, Plant, Collector, Identifier**
Acalles gadorensis<br>Stüben, 2001	EU286459 [01]<br>E-0097-gad<br>658nt	ZFMK-TIS-cE0097<br>ZFMK-DNA-0100400699	Spain, Murcia, 13 km N of Totana, Sierra de Espuna, N37°52'07" W01°33'46", 1361m, 29-Dez-2005, *Erinacea anthyllis, Quercus ilex under Pinus*, limestone, leg. Stüben,P., det. Stüben,P.
Acalles globulipennis<br>Wollaston, 1854	JN701882 [17]<br>14-PST<br>658nt	ZFMK-TIS-2D100447024<br>ZFMK-DNA-0100438096	Spain, Canary Islands, Gran Canaria, Moya, Barranco de los Tilos, N28°05'18" W15°35'36", 446m, 07-Dez-2010, *Laurisilva*, beating, leg. Stüben,P., det. Stüben,P.
Acalles globulipennis<br>Wollaston, 1854	KF680240 [17]<br>843-PST<br>658nt	ZFMK-TIS-3756<br>ZFMK-DNA-0100413782	Portugal, Estremadura, NW of Sintra, Serra de Sintra, Colares, Pe da Serra, N38°47'13" W09°27'46", 323m, 16-Mai-2012, *Quercus*, beating, leg. Stüben,P., det. Stüben,P.
Acalles globulipennis<br>Wollaston, 1854	MG229750 [40]<br>1845-PST<br>658nt	ZFMK-TIS-26048<br>ZFMK-DNA-0171606130	Portugal, Madeira, SE of Ribeira da Janela, Fanal, N32°48'48" W17°08'57", 1084m, 29-Jun-2014, *Ocotea foetens*, beating, leg. Stüben,P., det. Stüben,P.
Acalles globulipennis<br>Wollaston, 1854	FJ716562 [01]<br>E-676-glo<br>658nt	ZFMK-TIS-cE676<br>ZFMK-DNA-0100404279	Spain, Canary Islands, Tenerife, NE of La Laguna, Anaga Mts. near Moquinal, N28°31'55" W16°17'24", 840m, 29-Sep-2008, *Laurisilva*, leg. Astrin,J. & Stüben,P., det. Stüben,P.
Acalles globulipennis<br>Wollaston, 1854	GU987997 [01]<br>E-679-glo<br>658nt	ZFMK-TIS-cE679<br>ZFMK-DNA-0100404876	Spain, Canary Islands, Tenerife, NE of La Laguna, Anaga Mts. near Chinobre, N28°33'21" W16°10'46", 808m, 30-Sep-2008, *Laurus, Ixanthus viscosus*, leg. Astrin,J. & Stüben,P., det. Stüben,P.
Acalles globulipennis<br>Wollaston, 1854	GU988117 [01]<br>E-866-glo<br>658nt	ZFMK-TIS-cE866<br>ZFMK-DNA-0100405525	Portugal, N Caldas da Rainha., SW of Nazare near São Martinho do Porto, N39°30'23" W09°09'16", 53m, 28-Apr-2009, *Pistacia lentiscus*, leg. Astrin,J., det. Stüben,P.
Acalles globulipennis<br>Wollaston, 1854	GU987919 [01]<br>P-521-glo<br>627nt	ZFMK-TIS-cP521<br>ZFMK-DNA-0100400290	Portugal, Madeira, 3 km W of Porto Moniz, Santa Madalena, N32°51'31" W17°12'11", 391m, 19-Mrz-2008, *Ficus carica*, leg. Astrin,J. & Stüben,P., det. Astrin,J. and Stüben,P.
Acalles globulipennis<br>Wollaston, 1854	FJ716546 [01]<br>P-528-glo<br>658nt	ZFMK-TIS-cP528<br>ZFMK-DNA-0100400637	Portugal, Madeira, 4.5 km S of São Vicent Boca da Encumeada, "Folhadal", N32°45'08" W17°01'40", 1004m, 22-Mrz-2008, *Laurisilva, Euphorbia mellifera*, leg. Astrin,J. & Stüben,P., det. Astrin,J. and Stüben,P.
Acalles granulimaculosus<br>Stüben, 2014<br>**Paratype (DNAtype)**	GU988017 [01]<br>E-717-pil<br>658nt	ZFMK-TIS-cE717<br>ZFMK-DNA-0100404835	Spain, Canary Islands, La Gomera, S of Hermigua, El Cedro, Las Mimbreras, N28°07'27" W17°13'26", 901m, 06-Okt-2008, *Laurisilva*, leg. Astrin,J. & Stüben,P., det. Stüben,P.
Acalles granulimaculosus<br>Stüben, 2014<br>**Paratype (DNAtype)**	GU988021 [01]<br>E-724-pil<br>658nt	ZFMK-TIS-cE724<br>ZFMK-DNA-0100404247	Spain, Canary Islands, La Gomera, S of Vallehermoso, La Meseta, "La Piedra Encantada", N28°09'15" W17°17'36", 819m, 07-Okt-2008, *Ocotea foetens*, leg. Astrin,J. & Stüben,P., det. Stüben,P.
Acalles guadarramaensis<br>Stüben, 2004	KF680244 [17]<br>1268-PST<br>658nt	ZFMK-TIS-4084<br>ZFMK-DNA-0100426462	Spain, Community of Madrid, NE of Montejo de la Sierra, NE of Hiruela, Hayedo de Montejo, N41°05'23" W03°25'41", 1183m, 02-Mai-2013, *Quercus*, sieving, leg. Stüben,P. & Schütte,A., det. Stüben,P.
Acalles guadarramaensis<br>Stüben, 2004	KF680245 [17]<br>1270-PST<br>658nt	ZFMK-TIS-4086<br>ZFMK-DNA-0100426486	Spain, Community of Madrid, N of Lozoya, S of Puerto de Lozoya o de Navafria, N40°58'08" W03°48'00", 1294m, 03-Mai-2013, *Cytisus*, sieving, leg. Stüben,P. & Schütte,A., det. Stüben,P.
Acalles guadarramaensis<br>Stüben, 2004	GU987963 [01]<br>AND-617-gua<br>615nt	ZFMK-TIS-cAND617<br>ZFMK-DNA-0100404926	Andorra, near El Serrat, N42°37'38" E01°33'07", 1800m, 24-Jun-2008, *Corylus avellana, Betula*, leg. Astrin,J., det. Stüben,P.
Acalles guadarramaensis<br>Stüben, 2004	GU988096 [01]<br>E-836-gua<br>658nt	ZFMK-TIS-cE836<br>ZFMK-DNA-0100405559	Spain, Madrid, N of Somosierra, Pto. de Somosierra, N41°08'39" W03°34'41", 1380m, 22-Apr-2009, *Cytisus, Ilex, Salix*, leg. Astrin,J., det. Stüben,P.

**Name**<br>**Authority**<br>**Additional Information**	**GenBank Acc No (Ref.)**<br>**Specimen ID**<br>**Sequence Length**	**ZFMK Tissue ID**<br>**ZFMK DNA Sample ID**<br>**(SDEI DNA Sample ID)**	**Locality, GPS, Collection Date, Plant, Collector, Identifier**
Acalles iblanensis<br>Stüben, 2014<br>**Paratype (DNAtype)**	KC783816 [17]<br>186-PST_44MO2011<br>658nt	ZFMK-TIS-2D100440232<br>ZFMK-DNA-0100438120	Morocco, Atlas Mts., N of Imouzzer-des-Marmoucha, Jbel Bou Iblane, Tizi-Bou-Zabel, N33°38'44" W04°09'18", 2275m, 21-Mai-2011, broom, *Bupleurum, Astragalus*, sieving, leg. Stüben,P., det. Stüben,P.
Acalles kippenbergi<br>Dieckmann, 1982	KJ867571 [17]<br>460-PST<br>658nt	ZFMK-TIS-20246<br>ZFMK-DNA-0100449382	Italy, Verona, Badia Calavena, frazione RAUSI, N45°34' E11°09', 500m, 27-Feb-2007, leg. Diotti,L., det. Stüben,P.
Acalles lemur cisalpinus<br>Stüben, 2003	MG229689 [new]<br>1362-PST<br>658nt	ZFMK-TIS-4658<br>ZFMK-DNA-0155628571	Italy, Abruzzo, Gran Sasso, Vado di Sole, N42°23'46" E13°47'17", 1636m, 14-Aug-2013, *Fagus*, sieving, leg. Stüben,P., det. Stüben,P.
Acalles lemur cisalpinus<br>Stüben, 2003	MG229691 [new]<br>1369-PST<br>658nt	ZFMK-TIS-4665<br>ZFMK-DNA-0155628564	Italy, Abruzzo, P.N. Majella, E of Sulmona near Pacentro, N42°03'13" E14°01'49", 983m, 16-Aug-2013, *Acer*, sieving, leg. Stüben,P., det. Stüben,P.
Acalles lemur cisalpinus<br>Stüben, 2003	MG229699 [new]<br>1393-PST<br>658nt	ZFMK-TIS-4689<br>ZFMK-DNA-0155628540	Italy, Abruzzo, P.N. Majella near Gamberale, Sant'António, N41°53'53" E14°10'44", 1510m, 24-Aug-2013, *Fagus*, sieving, leg. Stüben,P., det. Stüben,P.
Acalles lemur cisalpinus<br>Stüben, 2003	GU987970 [01]<br>I-626-cis<br>592nt	ZFMK-TIS-cI626<br>ZFMK-DNA-0100404288	Italy, Campania, Cilento, 6 km SE of Vallo d. Lucania, M. Sacro o Gelbison, N40°12'41" E15°19'42", 1544m, 30-Jun-2008, *Fagus*, leg. Stüben,P., det. Stüben,P.
Acalles lemur cisalpinus<br>Stüben, 2003	GU987976 [01]<br>I-634-cis<br>658nt	ZFMK-TIS-cI634<br>ZFMK-DNA-0100404908	Italy, Campania, Monti Picentini, 9 km N of Acerno, Piano Laceno, N40°48'58" E15°07'35", 1210m, 03-Jul-2008, *Fagus*, leg. Stüben,P., det. Stüben,P.
Acalles lemur cisalpinus<br>Stüben, 2003	GU987988 [01]<br>I-655-cis<br>649nt	ZFMK-TIS-cI655<br>ZFMK-DNA-0100404881	Italy, Abruzzo, P.N. Majella, 11 km N of Roccaraso, Bosco di S. Antonio, N41°56'27" E14°01'41", 1321m, 14-Jul-2008, *Pyrus/Acer*, old forest, leg. Stüben,P., det. Stüben,P.
Acalles lemur cisalpinus<br>Stüben, 2003	GU988090 [01]<br>I-824-cis<br>658nt	ZFMK-TIS-cI824<br>ZFMK-DNA-0100405566	Italy, Lazio, Monti Lepini, Prati, N41°28' E13°06', 31-Mrz-2007, leg. Brandstetter, det. Brandstetter
Acalles lemur lemur<br>(Germar, 1824)	GU987880 [01]<br>D-0422-lem<br>658nt	ZFMK-TIS-cD0422<br>ZFMK-DNA-0100401008	Germany, Rhineland-Palatinate (RLP), Altenahr,Teufelsloch, N50°30'36" E06°59'24", 150m, 02-Nov-2007, *Quercus, Fagus*, leg. Stüben,P., det. Stüben,P.
Acalles lemur lemur<br>(Germar, 1824)	GU988042 [04]<br>D-673-lem<br>658nt	ZFMK-TIS-cD673<br>ZFMK-DNA-0100405070	Germany, Rhineland-Palatinate (RLP), Eppenbrunn, „Lourdesgrotte", N49°06'58" E07°33'20", 277m, 09-Mai-2008, *Fagus*, leg. Stüben,P., det. Stüben,P.
Acalles lemur lemur<br>(Germar, 1824)	GU987896 [01]<br>F-438-lem<br>658nt	ZFMK-TIS-cF438<br>ZFMK-DNA-0100400664	France, Alpes-Maritimes, 9 km N of Sospel, N of Moulinet, N43°57'58" E07°24'55", 1111m, 24-Dez-2007, *Castanea sativa*, leg. Stüben,P., det. Stüben,P.
Acalles lemur lemur<br>(Germar, 1824)	GU987755 [01]<br>I-0005-leL<br>658nt	ZFMK-TIS-cI0005<br>ZFMK-DNA-0100400751	France, Isere, 22 km S of Bourgoin, La Cote St. Andre, N45°24'04" E05°14'04", 469m, 08-Aug-2005, *Castanea*, leg. Stüben,P., det. Stüben,P.
Acalles lemur lemur<br>(Germar, 1824)	EU286446 [01]<br>I-0010-leL<br>658nt	ZFMK-TIS-cI0010<br>ZFMK-DNA-0100400752	France, Isere, 19 km S of Bourgoin, N of Semons, N45°26'01" E05°12'06", 448m, 08-Aug-2005, *Quercus, Fagus*, leg. Stüben,P., det. Stüben,P.
Acalles lemur lemur<br>(Germar, 1824)	GU987809 [01]<br>I-0015-leL<br>658nt	ZFMK-TIS-cI0015<br>ZFMK-DNA-0100400131	France, Isere, 12 km SW of Bourgoin, NE of St. Jean de Bournay, N45°31'02" E05°10'23", 395m, 10-Aug-2005, *Quercus, Castanea*, leg. Stüben,P., det. Stüben,P.

**Name**<br>**Authority**<br>**Additional Information**	**GenBank Acc No (Ref.)**<br>**Specimen ID**<br>**Sequence Length**	**ZFMK Tissue ID**<br>**ZFMK DNA Sample ID**<br>**(SDEI DNA Sample ID)**	**Locality, GPS, Collection Date, Plant, Collector, Identifier**
Acalles lemur lemur<br>(Germar, 1824)	GU987772 [01]<br>M-0059-leL<br>658nt	ZFMK-TIS-cM0059<br>ZFMK-DNA-0100400912	Germany, Rhineland-Palatinate (RLP), Moselle valley, 10 km S of Cochem, Bullay, N50°03'27" E07°08'51", 222m, 16-Sep-2005, *Quercus, Carpinus*, leg. Stüben,P., det. Stüben,P.
Acalles lemur lemur<br>(Germar, 1824)	GU987771 [01]<br>M-0061-leL<br>658nt	ZFMK-TIS-cM0061<br>ZFMK-DNA-0100400879	Germany, Rhineland-Palatinate (RLP), Moselle valley, 1 km N of Treis-Karden, N50°11'08" E07°18'20", 250m, 17-Sep-2005, *Quercus, Carpinus*, leg. Stüben,P., det. Stüben,P.
Acalles maraoensis<br>Stüben, 2001	GU987943 [01]<br>E-581-mar<br>626nt	ZFMK-TIS-cE581<br>ZFMK-DNA-0100404951	Spain, Caceres, 8 km W of Guadalupe, Sierra de Guadalupe, Las Villueras Mt., N39°28'00" W05°24'22", 1475m, 11-Apr-2004, *Quercus ilex, Quercus pubescens*, leg. Stüben,P., det. Stüben,P.
Acalles maraoensis<br>Stüben, 2001	GU988109 [01]<br>E-852-mar<br>658nt	ZFMK-TIS-cE852<br>ZFMK-DNA-0100405527	Portugal, Sa. Estrela, S of Manteigas, N40°19'26" W07°34'20", 1532m, 25-Apr-2009, *Betula, Cytisus, Erica*, leg. Astrin,J., det. Stüben,P.
Acalles maraoensis<br>Stüben, 2001	GU988112 [01]<br>E-857-mar<br>658nt	ZFMK-TIS-cE857<br>ZFMK-DNA-0100405532	Portugal, Sa. Estrela, SW of Manteigas, N40°18'45" W07°35'03", 1558m, 25-Apr-2009, *Cytisus*, leg. Astrin,J., det. Stüben,P.
Acalles maraoensis<br>Stüben, 2001	GU988118 [01]<br>E-867-mar<br>658nt	ZFMK-TIS-cE867<br>ZFMK-DNA-0100405524	Portugal, Sa. Marao, W of Vila Real, Alto do Espinho, N41°16'15" W07°54'17", 1145m, 30-Apr-2009, *Amelanchier, Quercus pyrenaica, Cytisus* , leg. Astrin,J., det. Stüben,P.
Acalles maraoensis<br>Stüben, 2001	GU988120 [01]<br>E-869-mar<br>658nt	ZFMK-TIS-cE869<br>ZFMK-DNA-0100405522	Portugal, Sa. Geres, NE of Braga, S of Lindoso, N41°49'48" W08°12'15", 974m, 01-Mai-2009, *Amelanchier, Erica, Castanea*, leg. Astrin,J., det. Stüben,P.
Acalles maraoensis<br>Stüben, 2001	GU987909 [01]<br>P-540-mar<br>658nt	ZFMK-TIS-cP540<br>ZFMK-DNA-0100400626	Portugal, Manteigas, N40°19'26" W07°34'20", 1531m, 26-Jan-2008, *Betula*, leg. Astrin,J., det. Astrin,J.
Acalles maraoensis<br>Stüben, 2001	GU988095 [01]<br>E-835-mar<br>658nt	ZFMK-TIS-cE835<br>ZFMK-DNA-0100405558	Spain, Toledo, SW of Navahermosa near Hontanar, N39°35'46" W04°30'11", 1100m, 21-Apr-2009, *Quercus ilex*, leg. Astrin,J., det. Stüben,P.
Acalles maraoensis<br>Stüben, 2001	GU988126 [01]<br>E-875-mar<br>658nt	ZFMK-TIS-cE875<br>ZFMK-DNA-0100405516	Spain, Burgos, SE of Burgos, W of slope of Trigaza Mt., N42°16'36" W03°15'32", 1496m, 05-Mai-2009, *Cytisus, Fagus*, leg. Astrin,J., det. Stüben,P.
Acalles micros<br>Dieckmann, 1982	EU286493 [01]<br>I-0030-mic<br>658nt	ZFMK-TIS-cI0030<br>ZFMK-DNA-0100400014	France, Isere, 2 km SE of Lans en Vercors, Montagne de Lans, N45°06'45" E05°36'21", 1352m, 12-Aug-2005, *Abies, Fagus, Fraxinus*, limestone: mixed forest, leg. Stüben,P., det. Stüben,P.
Acalles micros<br>Dieckmann, 1982	GU987753 [01]<br>I-0017-mic<br>658nt	ZFMK-TIS-cI0017<br>ZFMK-DNA-0100400768	France, Isere, 9 km N of Grenoble, Mont St. Martin, N45°16'15" E05°40'44", 813m, 12-Aug-2005, *Fagus, Fraxinus, Quercus*, leg. Stüben,P., det. Stüben,P.
Acalles micros<br>Dieckmann, 1982	GU987756 [01]<br>I-0003-mic<br>658nt	ZFMK-TIS-cI0003<br>ZFMK-DNA-0100400958	France, Isere, 14 km SW of Bourgoin, St. Jean de Bournay, N45°30'28" E05°09'09", 406m, 07-Aug-2005, *Castanea, Ilex, Quercus*, leg. Stüben,P., det. Stüben,P.
Acalles micros<br>Dieckmann, 1982	GU987845 [01]<br>F-0242-mic<br>658nt	ZFMK-TIS-cF0242<br>ZFMK-DNA-0100400305	France, Isere, 4 km E of La Chapelle-de-Surieu, N45°24'18" E04°56'26", 325m, 09-Apr-2006, *Fagus, Alnus*, leg. Stüben,P., det. Stüben,P.
Acalles micros<br>Dieckmann, 1982	GU988157 [01]<br>F-929-mic<br>658nt	ZFMK-TIS-cF929<br>ZFMK-DNA-0100405254	France, Dep. Loire, SW of Pelussin, Mont Pilat near Crêt de l'Oeillon, N45°24'24" E04°37'06", 1122m, 15-Jul-2009, *Fagus*, leg. Stüben,P., det. Stüben,P.

**Name**<br>**Authority**<br>**Additional Information**	**GenBank Acc No (Ref.)**<br>**Specimen ID**<br>**Sequence Length**	**ZFMK Tissue ID**<br>**ZFMK DNA Sample ID**<br>**(SDEI DNA Sample ID)**	**Locality, GPS, Collection Date, Plant, Collector, Identifier**
Acalles micros<br>Dieckmann, 1982	GU987846 [01]<br>D-0243-mic<br>658nt	ZFMK-TIS-cD0243<br>ZFMK-TIS-cD0243	Germany, Baden-Wuerttemberg (BW), Kaiserstuhl, "Auf dem Eck", N48°06'32" E07°40'04", 432m, 27-Mai-2006, *Fagus, Carpinus, Quercus*, leg. Stüben,P., det. Stüben,P.
Acalles micros<br>Dieckmann, 1982	GU988041 [01]<br>D-672-mic<br>658nt	ZFMK-TIS-cD672<br>ZFMK-DNA-0100405082	Germany, Rhineland-Palatinate (RLP), 12 km W of Pirmasens, Walshausen, N49°13'15" E07°26'43", 347m, 10-Mai-2008, *Fagus*, leg. Stüben,P., det. Stüben,P.
Acalles minutissimus<br>(LeConte, 1876)	KJ767020 [17]<br>1763-BAN<br>658nt	ZFMK-TIS-24999<br>ZFMK-DNA-0155668049	USA, Florida, Putnam County, 5 km E of Melrose, N29°42' W81°59', 27-Feb-1998, leg. Howden,H.& 38;A., det. Anderson,R.
Acalles misellus<br>Boheman, 1844	GU988159 [01]<br>F-937-mis<br>658nt	ZFMK-TIS-cF937<br>ZFMK-DNA-0100405241	France, Dep. Pyrenees-Atlantique, S of Sarnear Col de Lizarrieta, N43°15'46" W01°36'27", 382m, 24-Jul-2009, *Quercus*, leg. Stüben,P., det. Stüben,P.
Acalles misellus<br>Boheman, 1844	GU988160 [01]<br>F-938-mis<br>651nt	ZFMK-TIS-cF938<br>ZFMK-DNA-0100405242	France, Dep. Pyrenees-Atlantique, W of Ciboure near coast, N43°22'49" W01°42'59", 41m, 26-Jul-2009, *Quercus*, leg. Stüben,P., det. Stüben,P.
Acalles misellus<br>Boheman, 1844	KC783822 [new]<br>684-PST<br>658nt	ZFMK-TIS-20462<br>ZFMK-DNA-0100448261	France, Hautes-Pyrenees, Barbazan Debat, Bois de Rebisclou, N43°11' E00°07', 370m, 03-Jul-2009, leg. Brustel, det. Stüben,P.
Acalles misellus<br>Boheman, 1844	GU988094 [01]<br>NL-832-mis<br>658nt	ZFMK-TIS-cNL832<br>ZFMK-DNA-0100405555	Netherlands, Zeeland, Walcheren, NE of Oostkapelle, De Manteling (Oranjezon), N51°34'49" E03°33'45", 15-Apr-2009, *Quercus*, leg. Stüben,P., det. Stüben,P.
Acalles misellus<br>Boheman, 1844	GU987956 [01]<br>E-610-mis<br>627nt	ZFMK-TIS-cE610<br>ZFMK-DNA-0100404313	Spain, Barcelona, near Vallirana, N41°22'04" E01°55'05", 22-Jun-2008, *Silax aspera, Hedera helix, Laurus*, basin within quarry, leg. Astrin,J., det. Stüben,P.
Acalles misellus<br>Boheman, 1844	GU987959 [01]<br>E-613-mis<br>658nt	ZFMK-TIS-cE613<br>ZFMK-DNA-0100404935	Spain, Barcelona, S. Montseny, Collformic, Font St. Jordi, N41°48'12" E02°20'48", 1132m, 23-Jun-2008, *Rubus, Populus*, leg. Astrin,J., det. Stüben,P.
Acalles misellus<br>Boheman, 1844	GU987961 [01]<br>E-615-mis<br>658nt	ZFMK-TIS-cE615<br>ZFMK-DNA-0100404937	Spain, Barcelona, S. Montseny, Tordera valley near St. Marçal, N41°48'01" E02°25'15", 1060m, 23-Jun-2008, very diverse mixed forest, leg. Astrin,J., det. Stüben,P.
Acalles misellus<br>Boheman, 1844	GU987962 [01]<br>E-616-mis<br>658nt	ZFMK-TIS-cE616<br>ZFMK-DNA-0100404938	Spain, Barcelona, St. Esteve de Palautordera, Sta. Margarida, N41°43'07" E02°25'45", 254m, 24-Jun-2008, *Ruscus, Hedera, Platanus*, leg. Astrin,J., det. Stüben,P.
Acalles misellus<br>Boheman, 1844	GU988123 [01]<br>E-872-mis<br>658nt	ZFMK-TIS-cE872<br>ZFMK-DNA-0100405519	Spain, Leon, NE of León, N of Riano, SW of Posada de Valdeón, N43°06'54" W04°59'33", 1431m, 04-Mai-2009, *Fagus*, leg. Astrin,J., det. Stüben,P.
Acalles misellus<br>Boheman, 1844	GU988125 [01]<br>E-874-mis<br>658nt	ZFMK-TIS-cE874<br>ZFMK-DNA-0100405517	Spain, Palencia, N of Cervera de Pisuerga, Pto. de Piedrasluengas, N43°02'57" W04°27'19", 1314m, 04-Mai-2009, *Fagus, Ilex*, leg. Astrin,J., det. Stüben,P.
Acalles misellus<br>Boheman, 1844	KF680242 [17]<br>1262-PST<br>658nt	ZFMK-TIS-4078<br>ZFMK-DNA-0100426409	Spain, La Rioja, E of Montenegro de Cameros, N42°06'04" W02°42'38", 1109m, 01-Mai-2013, *Fagus*, Fagus trees near roadside, sieving, leg. Stüben,P. & Schütte,A., det. Stüben,P.
Acalles misellus<br>Boheman, 1844	KF680243 [17]<br>1266-PST<br>658nt	ZFMK-TIS-4082<br>ZFMK-DNA-0100426210	Spain, Castilla y Leon, N of Almarza, N42°01'24" W02°30'46", 1366m, 01-Mai-2013, *Ilex, Quercus*, sieving, leg. Stüben,P. & Schütte,A., det. Stüben,P.

**Name**<br>**Authority**<br>**Additional Information**	**GenBank Acc No (Ref.)**<br>**Specimen ID**<br>**Sequence Length**	**ZFMK Tissue ID**<br>**ZFMK DNA Sample ID**<br>**(SDEI DNA Sample ID)**	**Locality, GPS, Collection Date, Plant, Collector, Identifier**
Acalles monasterialis<br>Stüben, 2004	GU987891 [01]<br>E-505-mon<br>658nt	ZFMK-TIS-cE505<br>ZFMK-DNA-0100400655	Spain, Salamanca, 38 km NW of Bejar, Sierra de la Pena de Francia, Monasterio, N40°31'01" W06°10'15", 1636m, 10-Apr-2004, *Cytisus* sp., moist hillside, leg. Stüben,P., det. Stüben,P.
Acalles monasterialis<br>Stüben, 2004	GU988106 [01]<br>E-848-mon<br>658nt	ZFMK-TIS-cE848<br>ZFMK-DNA-0100405542	Spain, Salamanca, NW of Bejar, NW of Monforte de la Sierra, Pena de Francia near cloister, N40°31'01" W06°10'15", 1636m, 24-Apr-2009, *Cytisus*, leg. Astrin,J., det. Stüben,P.
Acalles ossetiensis<br>Stüben, 2018<br>**Paratype (DNAtype)**	MF426965 [34]<br>2756-PST_26853<br>658nt	ZFMK-TIS-26853<br>ZFMK-DNA-0171661899	Russia, Krasnodar, Chvizhepse vill. env. , N43°38'32" E40°04'45", 300m, 11-Jul-2014, leg. Kovalev,A.V., det. Stüben,P.
Acalles ossetiensis<br>Stüben, 2018<br>**Paratype (DNAtype)**	MF426966 [34]<br>2759-PST_26856<br>658nt	ZFMK-TIS-26856<br>ZFMK-DNA-0171661902	Russia, Krasnodar, Krasnaya Polyana env., N43°41'17" E40°12'20", 720m, 22-Jul-2014, leg. Kovalev,A.V., det. Stüben,P.
Acalles papei<br>A. & F. Solari, 1905	EU286507 [01]<br>HR-0327-pap<br>658nt	ZFMK-TIS-cHR0327<br>ZFMK-DNA-0100400110	Croatia, Dalmatia, 4.5 km N of Drnis, Promina Mts., N43°54'20" E16°10'01", 738m, 09-Jul-2007, *Macchia, Quercus, Carpinus, Corylus*, limestone, leg. Stüben,P., det. Stüben,P.
Acalles papei<br>A. & F. Solari, 1905	GU987873 [01]<br>KO-0369-pap<br>658nt	ZFMK-TIS-cKO0369<br>ZFMK-DNA-0100401021	Greece, Korfu Isl., 18 km N of Kerkyra, Loutses, Megali Grava, N39°46'38" E19°53'21", 479m, 23-Sep-2007, *Quercus pubescens, Laurus nobilis, Asphodelus*, cave floor, under stones, leg. Stüben,P., det. Stüben,P.
Acalles papei<br>A. & F. Solari, 1905	GU988044 [01]<br>KO-0378-pap<br>658nt	ZFMK-TIS-cKO0378<br>ZFMK-DNA-0100404712	Greece, Korfu Isl., 10 km SW of Kerkyra, Kato Garouna, N39°31'14" E19°51'29", 357m, 26-Sep-2007, *Quercus ilex*, leg. Stüben,P., det. Stüben,P.
Acalles papei<br>A. & F. Solari, 1905	GU987878 [01]<br>EP-0403-pap<br>658nt	ZFMK-TIS-cEP0403<br>ZFMK-DNA-0100400413	Greece, Epirus, 25 km SE of Igoumenitsa, Chrisavgi, N39°26'43" E20°32'04", 187m, 02-Okt-2007, *Quercus ilex, Platanus*, stream, leg. Stüben,P., det. Stüben,P.
Acalles papei<br>A. & F. Solari, 1905	GU988046 [01]<br>EP-0399-pap<br>640nt	ZFMK-TIS-cEP0399<br>ZFMK-DNA-0100405064	Greece, Epirus, 19 km E of Igoumenitsa, Petrovitsa, N39°33'30" E20°28'12", 355m, 01-Okt-2007, *Arbutus/Platanus*, stream, leg. Stüben,P., det. Stüben,P.
Acalles parasierrae<br>Stüben, 2002	GU988146 [01]<br>E-910-prs<br>658nt	ZFMK-TIS-cE910<br>ZFMK-DNA-0100405488	Morocco, Rif Mts., 10 km W of Ketama, N34°57'40" W04°40'51", 1600m, 10-Mai-2009, *Cedrus, Prunus*, leg. Stüben,P., det. Stüben,P.
Acalles parvulus<br>Boheman, 1837	MG229695 [new]<br>1378-PST<br>658nt	ZFMK-TIS-4674<br>ZFMK-DNA-0155628560	Italy, Abruzzo, SE of L'Aquila near Rocca di Cambio Parco Sirente-Yelino, N42°16'31" E13°27'47", 1012m, 18-Aug-2013, *hazelnut*, mountain mixed forest, sieving, leg. Stüben,P., det. Stüben,P.
Acalles parvulus parvulus<br>Boheman, 1837	EU286448 [01]<br>I-0008-par<br>658nt	ZFMK-TIS-cI0008<br>ZFMK-DNA-0100400954	France, Isere, 19 km S of Bourgoin, N of Semons, N45°26'01" E05°12'06", 448m, 08-Aug-2005, *Quercus, Fagus*, leg. Stüben,P., det. Stüben,P.
Acalles parvulus parvulus<br>Boheman, 1837	GU987764 [01]<br>D-0069-par<br>658nt	ZFMK-TIS-cD0069<br>ZFMK-DNA-0100400966	France, Isere, 10 km SE of Vienne, N45°28'00" E04°56'00", 23-Jul-2001, leg. , det. Stüben,P.
Acalles parvulus parvulus<br>Boheman, 1837	GU987777 [01]<br>I-0002-par<br>658nt	ZFMK-TIS-cI0002<br>ZFMK-DNA-0100400734	France, Isere, 14 km SW of Bourgoin, St. Jean de Bournay, N45°30'28" E05°09'09", 406m, 07-Aug-2005, *Castanea, Ilex, Quercus*, leg. Stüben,P., det. Stüben,P.
Acalles parvulus parvulus<br>Boheman, 1837	GU987849 [01]<br>F-0248-par<br>658nt	ZFMK-TIS-cF0248<br>ZFMK-DNA-0100401060	France, Isere, 4 km E of La Chapelle-de-Surieu, N45°24'18" E04°56'26", 325m, 09-Apr-2006, *Fagus, Alnus*, leg. Stüben,P., det. Stüben,P.

**Name**<br>**Authority**<br>**Additional Information**	**GenBank Acc No (Ref.)**<br>**Specimen ID**<br>**Sequence Length**	**ZFMK Tissue ID**<br>**ZFMK DNA Sample ID**<br>**(SDEI DNA Sample ID)**	**Locality, GPS, Collection Date, Plant, Collector, Identifier**
Acalles parvulus parvulus<br>Boheman, 1837	GU987850 [01]<br>F-0249-par<br>658nt	ZFMK-TIS-cF0249<br>ZFMK-DNA-0100401059	France, Var, 27 km W of Brignoles, Massif de la Sainte Baume, N43°19'42" E05°45'17", 703m, 18-Apr-2006, *Fagus*, leg. Stüben,P., det. Stüben,P.
Acalles parvulus parvulus<br>Boheman, 1837	GU987892 [01]<br>F-432-par<br>658nt	ZFMK-TIS-cF432<br>ZFMK-DNA-0100400658	France, Alpes-Maritimes, 6 km NE of Sospel, Col de Brouis, N43°55'28" E07°28'37", 882m, 23-Dez-2007, broom, *Helleborus*, leg. Stüben,P., det. Stüben,P.
Acalles parvulus parvulus<br>Boheman, 1837	GU987770 [01]<br>M-0060-par<br>658nt	ZFMK-TIS-cM0060<br>ZFMK-DNA-0100400739	Germany, Rhineland-Palatinate (RLP), Moselle valley, 1 km N of Treis-Karden, N50°11'08" E07°18'20", 250m, 17-Sep-2005, *Quercus, Carpinus*, leg. Stüben,P., det. Stüben,P.
Acalles parvulus parvulus<br>Boheman, 1837	GU987851 [01]<br>D-0250-par<br>613nt	ZFMK-TIS-cD0250<br>ZFMK-TIS-cD0250	Germany, Rhineland-Palatinate (RLP), Moselle valley, 5 km E of Cochem, "Apolloweg", N50°08'46" E07°12'51", 237m, 04-Jul-2006, *Quercus*, leg. Astrin,J. & Stüben,P., det. Stüben,P.
Acalles parvulus parvulus<br>Boheman, 1837	GU987966 [01]<br>I-621-par<br>658nt	ZFMK-TIS-cI621<br>ZFMK-DNA-0100404294	Italy, Campania, Cilento, 6 km SE of Vallo d. Lucania, M. Sacro o Gelbison, N40°12'41" E15°19'42", 1544m, 30-Jun-2008, *Fagus*, leg. Stüben,P., det. Stüben,P.
Acalles parvulus parvulus<br>Boheman, 1837	GU988151 [04]<br>I-654-par<br>618nt	ZFMK-TIS-cI654<br>ZFMK-DNA-0100404783	Italy, Abruzzo, P.N. Majella, 11 km N of Roccaraso, Bosco di S. Antonio, N41°56'27" E14°01'41", 1321m, 14-Jul-2008, *Pyrus/Acer*, leg. Stüben,P., det. Stüben,P.
Acalles parvulus temperei<br>Péricart, 1987	EU286449 [01]<br>I-0023-tem<br>658nt	ZFMK-TIS-cI0023<br>ZFMK-DNA-0100400757	France, Isere, 9 km N of Grenoble, Mont St. Martin, N45°16'15" E05°40'13", 719m, 12-Aug-2005, *Quercus*, limestone: wood pile, leg. Stüben,P., det. Stüben,P.
Acalles parvulus temperei<br>Péricart, 1987	GU987758 [01]<br>I-0029-tem<br>658nt	ZFMK-TIS-cI0029<br>ZFMK-DNA-0100400758	France, Isere, 2 km SE of Lans en Vercors, Montagne de Lans, N45°06'45" E05°36'21", 1352m, 12-Aug-2005, *Abies, Fagus, Fraxinus*, limestone: mixed forest, leg. Stüben,P., det. Stüben,P.
Acalles parvulus temperei<br>Péricart, 1987	GU987810 [01]<br>I-0042-tem<br>658nt	ZFMK-TIS-cI0042<br>ZFMK-DNA-0100400133	France, Isere, 7 km N of Grenoble, Massif de la Chartreus, Umg. Quaix en Ch., N45°14'53" E05°44'31", 613m, 13-Aug-2005, *Fagus, Ilex*, limestone (hillside forest), leg. Stüben,P., det. Stüben,P.
Acalles parvulus temperei<br>Péricart, 1987	GU988080 [01]<br>F-804-tem<br>658nt	ZFMK-TIS-cF804<br>ZFMK-DNA-0100405006	France, Les Houches, Carlaveyron, N45°55'40" E06°48'17", 2119m, 24-Sep-2007, leg. Brustel, det. Brustel
Acalles pilula<br>Wollaston, 1864	EU286461 [01]<br>C-0099-pil<br>585nt	ZFMK-TIS-cC0099<br>ZFMK-DNA-0100400919	Spain, Canary Islands, El Hierro, 7 km W of La Frontera, Pista Derrabado, N27°44'29" W18°03'24", 895m, 30-Dez-2006, *Laurus azorica*, leg. Stüben,P., det. Stüben,P.
Acalles pilula<br>Wollaston, 1864	GU987852 [01]<br>E-0251-pil<br>621nt	ZFMK-TIS-cE0251<br>ZFMK-DNA-0100401057	Spain, Canary Islands, El Hierro, 9.5 km W of La Frontera, Pista de Mencafite, N27°44'06" W18°05'08", 929m, 22-Dez-2006, *Chamaecytisus, Pericallis, Ficus*, leg. Stüben,P., det. Stüben,P.
Acalles pilula<br>Wollaston, 1864	GU987854 [01]<br>E-0253-pil<br>617nt	ZFMK-TIS-cE0253<br>ZFMK-DNA-0100401055	Spain, Canary Islands, El Hierro, 3 km N of San Andres, Montana de la Fara, N27°47'39" W17°56'55", 936m, 25-Dez-2006, *Sonchus hierrensis*, leg. Stüben,P., det. Stüben,P.
Acalles pilula<br>Wollaston, 1864	GU987920 [01]<br>E-0254-pil<br>658nt	ZFMK-TIS-cE0254<br>ZFMK-DNA-0100400601	Spain, Canary Islands, El Hierro, 4 km SW of La Frontera, El Parque, N27°44'20" W18°01'30", 739m, 24-Dez-2006, Detritus: foliage, leg. Stüben,P., det. Stüben,P.
Acalles pilula<br>Wollaston, 1864	GU987853 [01]<br>E-0252-pil<br>612nt	ZFMK-TIS-cE0252<br>ZFMK-DNA-0100401056	Spain, Canary Islands, La Palma, 2.5 km W of La Galga, above Cubo de la Galga, N28°45'18" W17°46'37", 857m, 03-Jul-2006, *Laurisilva, Juglans*, leg. Stüben,P., det. Stüben,P.

**Name**<br>**Authority**<br>**Additional Information**	**GenBank Acc No (Ref.)**<br>**Specimen ID**<br>**Sequence Length**	**ZFMK Tissue ID**<br>**ZFMK DNA Sample ID**<br>**(SDEI DNA Sample ID)**	**Locality, GPS, Collection Date, Plant, Collector, Identifier**
Acalles pilula<br>Wollaston, 1864	GU987856 [01]<br>E-0255-pil<br>658nt	ZFMK-TIS-cE0255<br>ZFMK-DNA-0100400876	Spain, Canary Islands, La Palma, 5 km SE of El Paso, Cumbre Nueva, N28°37'51" W17°49'36", 1415m, 28-Jun-2006, *Laurisilva*, leg. Stüben,P., det. Stüben,P.
Acalles pilula<br>Wollaston, 1864	GU988007 [01]<br>E-698-pil<br>658nt	ZFMK-TIS-cE698<br>ZFMK-DNA-0100404864	Spain, Canary Islands, Tenerife, E of Los Silos, Teno Mts., El Tanque Bajo, N28°21'39" W16°46'19", 534m, 03-Okt-2008, compost, leg. Astrin,J. & Stüben,P., det. Stüben,P.
Acalles ptinoides<br>(Marsham, 1802)	EU286522 [01]<br>D-0256-pti<br>658nt	ZFMK-TIS-cD0256<br>ZFMK-DNA-0100400033	Germany, North Rhine-Westphalia (NRW), lower Rhine, W of Mönchengladbach, 3 km E of Niederkrüchten, N51°11'56" E06°15'38", 07-Jun-2007, *Sphagnum*, leg. Stüben,P., det. Stüben,P.
Acalles ptinoides<br>(Marsham, 1802)	GU988036 [01]<br>D-671-pti<br>658nt	ZFMK-TIS-cD671<br>ZFMK-DNA-0100405091	Germany, North Rhine-Westphalia (NRW), N of Mönchengladbach, Helenabrunn, N51°13'34" E06°24'00", 60m, 15-Nov-2007, *Fagus*, leg. Stüben,P., det. Stüben,P.
Acalles ptinoides<br>(Marsham, 1802)	GU988155 [01]<br>D-927-pti<br>658nt	ZFMK-TIS-cD927<br>ZFMK-DNA-0100405256	Germany, Rhineland-Palatinate (RLP), W of Karlsruhe, Bienwald, N49°00'49" E08°05'18", 130m, 12-Jun-2009, *Quercus*, leg. Stüben,P., det. Stüben,P.
Acalles ptinoides<br>(Marsham, 1802)	MG229757 [new]<br>1893-PST<br>658nt	ZFMK-TIS-23998<br>ZFMK-DNA-0169168520	Germany, North Rhine-Westphalia (NRW), Elmpt, NSG "Elmpter Schwalmbruch", Venekotensee, N51°13'43" E06°06'59", 35m, 27-Aug-2014, *Quercus robur*, beating, leg. Stüben,P., det. Stüben,P.
Acalles ptinoides<br>(Marsham, 1802)	MG229758 [new]<br>1904-PST<br>658nt	ZFMK-TIS-24009<br>ZFMK-DNA-0169170408	Germany, North Rhine-Westphalia (NRW), lower Rhine, 2 km S of Brüggen, Elmpter creek, N51°13'24" E06°11'31", 57m, 03-Sep-2014, *Calluna culgaris*, beating, leg. Stüben,P., det. Stüben,P.
Acalles ptinoides<br>(Marsham, 1802)	GU988121 [01]<br>E-870-pti<br>658nt	ZFMK-TIS-cE870<br>ZFMK-DNA-0100405521	Spain, Asturias, S of Oviedo, SW of Pto. de Pajares, Valgrande ski station, N42°58'25" W05°46'30", 1649m, 03-Mai-2009, *Betula, Erica, Ilex, Cytisus*, leg. Astrin,J., det. Stüben,P.
Acalles ptinoides<br>(Marsham, 1802)	GU988122 [01]<br>E-871-pti<br>658nt	ZFMK-TIS-cE871<br>ZFMK-DNA-0100405520	Spain, Asturias, SE of Oviedo near Tarna, N43°06'16" W05°13'03", 1322m, 04-Mai-2009, *Fagus, Cytisus*, leg. Astrin,J., det. Stüben,P.
Acalles ptinoides<br>(Marsham, 1802)	GU988127 [01]<br>E-876-pti<br>658nt	ZFMK-TIS-cE876<br>ZFMK-DNA-0100404583	Spain, Burgos, SE of Burgos, W of slope of Trigaza Mt., N42°16'36" W03°15'32", 1496m, 05-Mai-2009, *Cytisus, Fagus*, leg. Astrin,J., det. Stüben,P.
Acalles ptinoides<br>(Marsham, 1802)	KJ867597 [new]<br>1264-PST<br>658nt	ZFMK-TIS-4080<br>ZFMK-DNA-0100426438	Spain, Castilla y Leon, N of Almarza, N42°01'24" W02°30'46", 1366m, 01-Mai-2013, *Ilex, Quercus*, sieving, leg. Stüben,P. & Schütte,A., det. Stüben,P.
Acalles reitteri<br>Meyer, 1896	MK347581 [40]<br>3018-KRA<br>658nt	(SDEI-DNA-3018-KRA)	Armenia, Syunik prov., Davit Bek, 2,5 km NW, N39°19'52" E46°28'8", 1312m, 07-Jun-2017, leg. Krátky,J., det. Stüben,P.
Acalles sablensis<br>Blatchley, 1920	KP065589 [17]<br>1764-BAN<br>658nt	ZFMK-TIS-25001<br>ZFMK-DNA-0155668048	USA, Florida, Monroe County, Long Key State Park, Recreation Area, N24°48' W80°48', 22-Mai-1990, *Borrichia* spp., near mangrove forest border, sweeping, leg. Anderson,R., det. Anderson,R.
Acalles sardiniaensis<br>Stüben, 2001	GU987882 [01]<br>I-460-sar<br>658nt	ZFMK-TIS-cI460<br>ZFMK-DNA-0100400994	Italy, Sardinia Isl., 21 km SO Aritzo, Barbagia Seulo, Monte Arqueri, N39°49'02" E09°21'59", 950m, 22-Okt-2004, *Quercus ilex* , limestone/cliff, leg. Stüben,P., det. Stüben,P.
Acalles sardiniaensis<br>Stüben, 2001	KF680230 [17]<br>IT1072c<br>649nt	ZFMK-TIS-cIT1072c<br>ZFMK-DNA-0112704597	Italy, Sardinia Isl. East, NE of Seui, M. Perda Liana, N39°55'13" E09°24'20", 973m, 28-Sep-2010, *Quercus ilex, Ilex*, leg. Stüben,P., det. Stüben,P.

**Name**<br>**Authority**<br>**Additional Information**	**GenBank Acc No (Ref.)**<br>**Specimen ID**<br>**Sequence Length**	**ZFMK Tissue ID**<br>**ZFMK DNA Sample ID**<br>**(SDEI DNA Sample ID)**	**Locality, GPS, Collection Date, Plant, Collector, Identifier**
Acalles sardiniaensis<br>Stüben, 2001	KF680231 [17]<br>IT1074c<br>658nt	ZFMK-TIS-cIT1074c<br>ZFMK-DNA-0112704595	Italy, Sardinia Isl. East, NE of Seui, M. Tonneri, N39°53'59" E9°23'24", 926m, 28-Sep-2010, *Quercus ilex*, cliff, leg. Stüben,P., det. Stüben,P.
Acalles sardiniaensis<br>Stüben, 2001	KF680232 [17]<br>IT1078c<br>658nt	ZFMK-TIS-cIT1078c<br>ZFMK-DNA-0112704591	Italy, Sardinia Isl. East, E of Seui, M. Arqueri, N39°49'04" E09°22'02", 925m, 29-Sep-2010, *Quercus ilex*, cliff, leg. Stüben,P., det. Stüben,P.
Acalles sardiniaensis<br>Stüben, 2001	KF680233 [17]<br>IT1080c<br>658nt	ZFMK-TIS-cIT1080c<br>ZFMK-DNA-0112704589	Italy, Sardinia Isl. East, E of Seui, Ulassai, N39°48'35" E09°29'48", 745m, 29-Sep-2010, *Quercus ilex*, cliff, leg. Stüben,P., det. Stüben,P.
Acalles sardiniaensis<br>Stüben, 2001	KF680234 [17]<br>IT1081c<br>658nt	ZFMK-TIS-cIT1081c<br>ZFMK-DNA-0112702465	Italy, Sardinia Isl., NE of Aritzo, Gennargentu, M. di Iscudu, N40°01'10" E09°16'39", 1511m, 30-Sep-2010, *Alnus, moos*, creek, leg. Stüben,P., det. Stüben,P.
Acalles sardiniaensis<br>Stüben, 2001	KF680235 [17]<br>IT1087c<br>658nt	ZFMK-TIS-cIT1087c<br>ZFMK-DNA-0112704581	Italy, Sardinia Isl. South, E of Cagliari, Nationalpark M. d. Sette Fratelli, M. Funtana-Ona, N39°17'30" E09°23'17", 596m, 03-Okt-2010, *Quercus, Arbutus, Pistacia*, leg. Stüben,P., det. Stüben,P.
Acalles sardiniaensis<br>Stüben, 2001	MK891638 [new]<br>1787-PST<br>658nt	ZFMK-TIS-24111<br>ZFMK-DNA-0171624078	Italy, Sardinia Isl., N of Gairo, N39°52'06" E09°30'13", 906m, 11-Mai-2014, *Quercus*, sieving, leg. Stüben,P. & Schütte,A., det. Stüben,P.
Acalles sarothamni<br>Stüben, 2003	GU987885 [01]<br>E-466-sar<br>461nt	ZFMK-TIS-cE466<br>ZFMK-DNA-0100401000	Spain, Castilla y Leon, Sierra de Gredos, 14 km NE of Arenas de S. Pedro, Pto. del Pico, N40°19'20" W05°00'50", 1396m, 23-Apr-2003, *Sarothamnus* sp. , leg. Stüben,P., det. Stüben,P.
Acalles sarothamni<br>Stüben, 2003	GU987886 [01]<br>E-467-sar<br>658nt	ZFMK-TIS-cE467<br>ZFMK-DNA-0100400946	Spain, Avila, 46 km SW of Ávila, Sierra de Villafranca, Navacepedilla, N40°28'59" W05°11'08", 1266m, 06-Apr-2004, *Cytisus*, leg. Stüben,P., det. Stüben,P.
Acalles sierrae<br>H. Brisout de Barneville, 1865	EU286492 [01]<br>E-0100-sie<br>658nt	ZFMK-TIS-cE0100<br>ZFMK-DNA-0100400149	Spain, Andalucia, 12 km N of Laujar, Sierra Nevada **(type locality)**, N37°05'45" W02°57'37", 2285m, 15-Okt-2005, *Erinacea anthyllis*, leg. Stüben,P., det. Stüben,P.
Acalles sierrae<br>H. Brisout de Barneville, 1865	GU987859 [01]<br>E-0261-sie<br>658nt	ZFMK-TIS-cE0261<br>ZFMK-DNA-0100400437	Spain, Andalucia, 17 km NW of Laujar, Sierra Nevada, S of Puerto de la Ragua, N37°06'11" W03°01'40", 2070m, 12-Okt-2005, *Bupleurum,* broom, stream valley, leg. Stüben,P., det. Stüben,P.
Acalles sierrae<br>H. Brisout de Barneville, 1865	KF680247 [17]<br>1292-PST<br>658nt	ZFMK-TIS-4108<br>ZFMK-DNA-0100426184	Spain, Granada, NE of Granada, Sierra de La Yedra, N37°15'50" W03°32'13", 1414m, 13-Mai-2013, *Quercus ilex*, sieving, leg. Stüben,P. & Schütte,A., det. Stüben,P.
Acalles sierrae<br>H. Brisout de Barneville, 1865	KF680248 [17]<br>1304-PST<br>658nt	ZFMK-TIS-4120<br>ZFMK-DNA-0100426173	Spain, Jaen, SE of Mancha Real, Sierra Almaden (El Almaden, **type locality**), N37°44'09" W03°31'26", 1963m, 15-Mai-2013, *Euphorbia nicaeensis, Bupleurum spinosum*, sieving, leg. Stüben,P. & Schütte,A., det. Stüben,P.
Acalles sierrae<br>H. Brisout de Barneville, 1865	KF680249 [17]<br>1310-PST<br>658nt	ZFMK-TIS-4126<br>ZFMK-DNA-0100426166	Spain, Jaen, SE of Mancha Real, Sierra Almaden (El Almaden), N37°44'23" W03°32'07", 1662m, 15-Mai-2013, *Erinacea anthyllis*, sieving, leg. Stüben,P. & Schütte,A., det. Stüben,P.
Acalles sierrae<br>H. Brisout de Barneville, 1865<br>formerly: *A. bazaensis* - Syn!	KF680251 [17]<br>1316-PST<br>658nt	ZFMK-TIS-4132<br>ZFMK-DNA-0100426160	Spain, Granada, E of Baul, Sierra de Baza, Santa Barbara, N37°22'25" W02°51'03", 2023m, 17-Mai-2013, *Bupleurum, (Astragalus* sp.*)*, sieving, leg. Stüben,P. & Schütte,A., det. Stüben,P.
Acalles sierrae<br>H. Brisout de Barneville, 1865<br>formerly: *A. bazaensis* - Syn!	KF680252 [17]<br>1318-PST<br>658nt	ZFMK-TIS-4134<br>ZFMK-DNA-0100426158	Spain, Granada, E of Baul, Sierra de Baza, Santa Barbara, N37°22'50" W02°51'06", 1990m, 17-Mai-2013, *Bupleurum, (Astragalus* sp.*)*, sieving, leg. Stüben,P. & Schütte,A., det. Stüben,P.

**Name**<br>**Authority**<br>**Additional Information**	**GenBank Acc No (Ref.)**<br>**Specimen ID**<br>**Sequence Length**	**ZFMK Tissue ID**<br>**ZFMK DNA Sample ID**<br>**(SDEI DNA Sample ID)**	**Locality, GPS, Collection Date, Plant, Collector, Identifier**
Acalles sierrae<br>H. Brisout de Barneville, 1865	KF680253 [17]<br>1330-PST<br>658nt	ZFMK-TIS-4146<br>ZFMK-DNA-0100426151	Spain, Jaen, Sierra Magina, SE of Torres, N37°44'46" W03°29'53", 1527m, 19-Mai-2013, *Erinacea anthyllis, Quercus ilex, Astragalus* sp., *Bupleurum*, sieving, leg. Stüben,P. & Schütte,A., det. Stüben,P.
Acalles sierrae<br>H. Brisout de Barneville, 1865	KF680254 [17]<br>1332-PST<br>658nt	ZFMK-TIS-4148<br>ZFMK-DNA-0100426153	Spain, Jaen, Sierra Magina, SE of Torres, N37°44'06" W03°30'13", 1663m, 19-Mai-2013, *Erinacea anthyllis, Euphorbia nicaeensis*, sieving, leg. Stüben,P. & Schütte,A., det. Stüben,P.
Acalles sierrae<br>H. Brisout de Barneville, 1865	KF680255 [17]<br>1344-PST<br>658nt	ZFMK-TIS-4160<br>ZFMK-DNA-0100426265	Spain, Jaen, S of La Guardia, Sierra de la Pandera, N37°40'20" W03°41'48", 1223m, 19-Mai-2013, *Erinacea anthyllis, Cytisus* sp., sieving, leg. Stüben,P. & Schütte,A., det. Stüben,P.
Acalles sintraniensis<br>Stüben, 1999	GU988110 [01]<br>E-853-sin<br>658nt	ZFMK-TIS-cE853<br>ZFMK-DNA-0100405528	Portugal, Sa. Estrela, S of Manteigas, N40°19'26" W07°34'20", 1532m, 25-Apr-2009, *Betula, Cytisus, Erica*, leg. Astrin,J., det. Stüben,P.
Acalles sintraniensis<br>Stüben, 1999	GU988111 [01]<br>E-856-sin<br>658nt	ZFMK-TIS-cE856<br>ZFMK-DNA-0100405531	Portugal, Sa. Estrela, SW of Manteigas, N40°18'45" W07°35'03", 1558m, 25-Apr-2009, *Cytisus*, leg. Astrin,J., det. Stüben,P.
Acalles sintraniensis<br>Stüben, 1999	KF680238 [17]<br>827-PST<br>658nt	ZFMK-TIS-3740<br>ZFMK-DNA-0100414273	Portugal, Minho, NW of Ponte de Lima, Vila Nova de Cerveira, Rio Coura, N41°52'27" W08°41'57", 123m, 07-Mai-2012, sieving, leg. Stüben,P., det. Stüben,P.
Acalles sintraniensis<br>Stüben, 1999	KF680239 [17]<br>833-PST<br>658nt	ZFMK-TIS-3746<br>ZFMK-DNA-0100414258	Portugal, Minho, PN of Peneda-Geres near Soajo, N41°52'14" W08°16'30", 200m, 09-Mai-2012, *Sarothamnus*, sieving, leg. Stüben,P., det. Stüben,P.
Acalles sp.	MG229842 [new]<br>X-0426-AcS<br>658nt	ZFMK-TIS-24477<br>ZFMK-DNA-0155630413	Mexico, Chiapas, Reserva Huitepec, , 2600m, 11-Jul-2007, leg. Longino,J. & Anderson,R., det. Longino,J. with Anderson,R.
Acalles spec.nov.4	KJ767022 [17]<br>1767-BAN<br>658nt	ZFMK-TIS-25751<br>ZFMK-DNA-0155668045	USA, Florida, Dade County, Chekika State Rec. Area, N25°36' W80°29', 12-Okt-1990, hardwood hammock, beating, leg. Anderson,R., det. Anderson,R.
Acalles spec.nov.5	KP065590 [17]<br>1768-BAN<br>658nt	ZFMK-TIS-25791<br>ZFMK-DNA-0155668044	USA, Florida, Dade County, Miami, Charles Deering Estate Park, N25°36' W80°18', 07-Okt-1990, leg. Anderson,R., det. Anderson,R.
Acalles sylvosus<br>Blatchley, 1916	KJ767021 [17]<br>1765-BAN<br>658nt	ZFMK-TIS-25739<br>ZFMK-DNA-0155668047	USA, Florida, Dade County, Miami, Camp Mahachee, N25°40' W80°16', 17-Okt-1990, hardwood hammock, beating, leg. Anderson,R., det. Anderson,R.
Acalles testensis<br>Stüben, 2003	GU988128 [01]<br>E-880-tes<br>658nt	ZFMK-TIS-cE880<br>ZFMK-DNA-0100405506	Morocco, High Atlas, N of Tizi-n-Test, Tachguette, N30°52'25" W08°21'25", 2047m, 30-Apr-2009, *Euphorbia nicaeensis, Quercus ilex,* broom, leg. Stüben,P., det. Stüben,P.
Acalles tibialis<br>(Weise, 1891)	GU987895 [01]<br>F-435-tib<br>658nt	ZFMK-TIS-cF435<br>ZFMK-DNA-0100400661	France, Alpes-Maritimes, 8 km NW of Sospel, 2 km S of Peira Cava, Baisse de la Cabanette, N43°54'53" E07°21'11", 1371m, 24-Dez-2007, *Fagus* , leg. Stüben,P., det. Stüben,P.
Acalles tibialis<br>(Weise, 1891)	GU987898 [01]<br>F-442-tib<br>658nt	ZFMK-TIS-cF442<br>ZFMK-DNA-0100400653	France, Alpes-Maritimes, 3 km W of Sospel, Col de Braus, N43°52'34" E07°24'17", 1051m, 26-Dez-2007, *Quercus pubescens, Ostrya carpinifolia,* broom, leg. Stüben,P., det. Stüben,P.
Acalles vorsti<br>Stüben, 2014<br>**Paratype (DNAtype)**	KF680256 [17]<br>1358-PST_22183<br>658nt	ZFMK-TIS-22183<br>ZFMK-DNA-0100417602	Spain, Mallorca, Soller, Son Coll, N39°46'06" E02°40'12", 287m, 04-Apr-2002, *Vorst, Quercus forest*, sieving, leg. Vorst,O., det. Stüben,P.

**Name**<br>**Authority**<br>**Additional Information**	**GenBank Acc No (Ref.)**<br>**Specimen ID**<br>**Sequence Length**	**ZFMK Tissue ID**<br>**ZFMK DNA Sample ID**<br>**(SDEI DNA Sample ID)**	**Locality, GPS, Collection Date, Plant, Collector, Identifier**
Acallocrates colonnellii<br>Bahr, 2003	EU286509 [01]<br>HR-0333-col<br>658nt	ZFMK-TIS-cHR0333<br>ZFMK-DNA-0100400074	Croatia, Dalmatia, 6 km E of Karlobag, Velebit Mts., Ostarijska vrata, N44°31'45" E15°08'34", 927m, 14-Jul-2007, *Fagus*, limestone, leg. Stüben,P., det. Stüben,P.
Acallocrates colonnellii<br>Bahr, 2003	MG322652 [new]<br>CZ1055<br>658nt	ZFMK-TIS-cCZ1055<br>ZFMK-DNA-0112704614	Czech Republic, SE Moravia (UH), Lopenik, Bile Karpaty Mts., 48°56'00"N 17°46'00"E, 727m, 15-Mai-2009, *Fagus*, leg. Kresl,P., det. Kresl,P.
Acallocrates colonnellii<br>Bahr, 2003	MK347566 [new]<br>2990-PST<br>658nt	(SDEI-DNA-2990-PST)	Greece, Macedonia, N Neo Petritsi (near Serres), N41°19'29" E23°16'06", 861m, 15-Jul-2017, *Fagus*, leg. Stüben,P., det. Stüben,P.
Acallocrates colonnellii<br>Bahr, 2003	KJ867592 [new]<br>1222-JKR<br>658nt	ZFMK-TIS-3558<br>ZFMK-DNA-0100426258	Slovakia, Driencansky kras, Slizke env., N48°29'52" E20°05'28", 284m, 29-Sep-2012, *Quercetum*, sieving, leg. Krátky,J., det. Krátky,J.
Acallocrates colonnellii<br>Bahr, 2003	EU286462 [01]<br>SLO-0119-col<br>658nt	ZFMK-TIS-cSLO0119<br>ZFMK-DNA-0100400896	Slovenia, Sv. Ana, env., 20 km NE of Maribor, N46°39' E15°49', 05-Aug-2006, *Fagus, Quercus*, leg. Stejskal,R., det. Stejskal,R.
Acallocrates denticollis<br>(Germar, 1824)	GU987815 [01]<br>HR-0320-den<br>658nt	ZFMK-TIS-cHR0320<br>ZFMK-DNA-0100400109	Croatia, Dalmatia, 7 km N of Makarska, Biokovo Mts., Bast, N43°21'26" E16°59'18", 415m, 05-Jul-2007, *Quercus*, limestone, leg. Stüben,P., det. Stüben,P.
Acallocrates denticollis<br>(Germar, 1824)	GU987868 [01]<br>HR-0325-den<br>658nt	ZFMK-TIS-cHR0325<br>ZFMK-DNA-0100400888	Croatia, Dalmatia, 4.5 km N of Drnis, Promina Mts., N43°54'20" E16°10'01", 738m, 09-Jul-2007, *Quercus, Carpinus, Corylus*, limestone: Maccia, leg. Stüben,P., det. Stüben,P.
Acallocrates denticollis<br>(Germar, 1824)	GU987794 [01]<br>GR-0118-den<br>658nt	ZFMK-TIS-cGR0118<br>ZFMK-DNA-0100400688	Greece, Crete Isl. West, Levka Ori, Imbros, above the Imbros Gorge, N35°14'51" E24°10'30", 900m, 02-Okt-2006, *Quercus*, leg. Bahr,F. & Bayer,B., det. Bahr,F. & Bayer,B.
Acallocrates denticollis<br>(Germar, 1824)	GU987875 [01]<br>KO-0382-den<br>658nt	ZFMK-TIS-cKO0382<br>ZFMK-DNA-0100401023	Greece, Korfu Isl., 24 km S of Kerkyra, S of Perivoli, N39°24'08" E20°00'19, 93m, 27-Sep-2007, *Laurus, Quercus, Arbutus, Smilax*, leg. Stüben,P., det. Stüben,P.
Acallocrates denticollis<br>(Germar, 1824)	KJ867589 [new]<br>863-PST<br>658nt	ZFMK-TIS-3776<br>ZFMK-DNA-0100414228	Greece, Zakynthos Isl., near Alikanas, Hotel Zante Village, N37°49'54" E20°47'10", 5m, 15-Okt-2012, *hotel wall*, beating, leg. Stüben,P., det. Stüben,P.
Acallocrates denticollis<br>(Germar, 1824)	GU987876 [01]<br>EP-0387-den<br>658nt	ZFMK-TIS-cEP0387<br>ZFMK-DNA-0100401014	Greece, Epirus, N of Metsovo, Milia [Milea], N39°51'15" E21°13'32", 1146m, 29-Sep-2007, *Fagus*, leg. Stüben,P., det. Stüben,P.
Acallocrates denticollis<br>(Germar, 1824)	GU988045 [01]<br>EP-0389-den<br>658nt	ZFMK-TIS-cEP0389<br>ZFMK-DNA-0100405065	Greece, Epirus, 9 km SW of Metsovo, Mikro Peristeri, N39°45'03" E21°05'09", 693m, 30-Sep-2007, *Quercus ilex*, leg. Stüben,P., det. Stüben,P.
Acallocrates denticollis<br>(Germar, 1824)	MK347558 [new]<br>2975-PST<br>658nt	(SDEI-DNA-2975-PST)	Greece, Pieria, 2 km E Kryovrysi, N39°58'47" E22°021'7", 1139m, 30-Jun-2017, *Quercus ilex*, leg. Stüben,P., det. Stüben,P.
Acallocrates minutesquamosus<br>(Reiche, 1860)	EU286504 [01]<br>F-0262-min<br>658nt	ZFMK-TIS-cF0262<br>ZFMK-DNA-0100400105	France, Var, 27 km W of Brignoles, Massif de la Sainte Baume, N43°19'42" E05°45'17", 703m, 18-Apr-2006, *Fagus*, leg. Stüben,P., det. Stüben,P.
Acallocrates minutesquamosus<br>(Reiche, 1860)	GU987972 [01]<br>I-630-min<br>614nt	ZFMK-TIS-cI630<br>ZFMK-DNA-0100404904	Italy, Campania, Cilento, 14 km SW of Vallo d. Lucania, Marina di Ascea, N40°07'42" E15°10'53", 13m, 30-Jun-2008, *Quercus ilex*, leg. Stüben,P., det. Stüben,P.

**Name**<br>**Authority**<br>**Additional Information**	**GenBank Acc No (Ref.)**<br>**Specimen ID**<br>**Sequence Length**	**ZFMK Tissue ID**<br>**ZFMK DNA Sample ID**<br>**(SDEI DNA Sample ID)**	**Locality, GPS, Collection Date, Plant, Collector, Identifier**
Acallocrates minutesquamosus<br>(Reiche, 1860)	GU987983 [01]<br>I-643-min<br>620nt	ZFMK-TIS-cI643<br>ZFMK-DNA-0100404900	Italy, Basilicata, Monte Pollino, 4 km SE of Rotonda, Zarafa, N39°56'33" E16°04'28", 677m, 07-Jul-2008, *Quercus ilex*, leg. Stüben,P., det. Stüben,P.
Acallocrates minutesquamosus<br>(Reiche, 1860)	MG229696 [new]<br>1384-PST<br>658nt	ZFMK-TIS-4680<br>ZFMK-DNA-0155628549	Italy, Lazio, forest above San Donato Val di Camino, N41°42'34" E13°48'49", 679m, 21-Aug-2013, *Fraxinus, Carpinus, Quercus*, sieving, leg. Stüben,P., det. Stüben,P.
Acallorneuma doderoi<br>A. & F. Solari, 1909	EU286457 [01]<br>S-0082-dod<br>658nt	ZFMK-TIS-cS0082<br>ZFMK-DNA-0100400941	Italy, Sicilia Isl. (PA), 6 km SW of Godrano, Bosco Ficuzza, Mte. Rocca Busambra, N37°51'38" E13°23'24", 1200m, 16-Okt-2002, *Quercus, Fraxinus*, leg. Stüben,P., det. Stüben,P.
Acallorneuma doderoi<br>A. & F. Solari, 1909	GU987795 [01]<br>I-0120-dod<br>572nt	ZFMK-TIS-cI0120<br>ZFMK-DNA-0100400689	Italy, Sicilia Isl. (PA), 5 km E of Partinico, Santuario del Romitello, N38°02'37" E13°09'51", 720m, 10-Okt-2006, *Quercus ilex, Fraxinus*, leg. Stüben,P., det. Stüben,P.
Acallorneuma ibericum<br>Stüben, 2005	KJ867608 [28]<br>1296-PST<br>658nt	ZFMK-TIS-4112<br>ZFMK-DNA-0100426180	Spain, Jaen, Sierra de Cazorla, N of Arroyo Frio, N38°00'13" W02°53'10", 762m, 14-Mai-2013, *Quercus ilex, Smilax*, sieving, leg. Stüben,P. & Schütte,A., det. Stüben,P.
Acallorneuma ibericum<br>Stüben, 2005	KJ867616 [28]<br>1324-PST<br>658nt	ZFMK-TIS-4140<br>ZFMK-DNA-0100426145	Spain, Jaen, Sierra Magina, SE of Torres, N37°44'46" W03°29'53", 1527m, 19-Mai-2013, *Erinacea anthyllis, Quercus ilex, Astragalus* sp., *Bupleurum*, sieving, leg. Stüben,P. & Schütte,A., det. Stüben,P.
Acallorneuma ibericum<br>Stüben, 2005	KJ867619 [28]<br>1336-PST<br>658nt	ZFMK-TIS-4152<br>ZFMK-DNA-0100426140	Spain, Jaen, S of La Guardia, Sierra de la Pandera, N37°40'20" W03°41'48", 1223m, 19-Mai-2013, *Erinacea anthyllis, Cytisus* sp., sieving, leg. Stüben,P. & Schütte,A., det. Stüben,P.
Acallorneuma ingoi<br>G. Osella & Zuppa, 2002	KP776636 [28]<br>1774-PST<br>658nt	ZFMK-TIS-24098<br>ZFMK-DNA-0171624090	Italy, Lazio, above S. Donato Val Di Comino, La grotta dei tedeschi , N41°43'67" E13°48'45", 995m, 06-Mai-2014, *Carpinus*, sieving, leg. Stüben,P. & Schütte,A., det. Stüben,P.
Acallorneuma ingoi<br>G. Osella & Zuppa, 2002	KP776637 [new]<br>1778-PST<br>658nt	ZFMK-TIS-24102<br>ZFMK-DNA-0171624086	Italy, Molise, Montenero Val Cocchiara, N41°43'22" E14°04'54", 915m, 06-Mai-2014, *Quercus, Rubus, Fraxinus*, sieving, leg. Stüben,P. & Schütte,A., det. Stüben,P.
Acallorneuma ingoi<br>G. Osella & Zuppa, 2002	KP776642 [28]<br>1771-PST<br>658nt	ZFMK-TIS-24095<br>ZFMK-DNA-0171624093	Italy, Lazio, Alvito, S. Onófria, Val de Rio, N41°44'19" E13°43'41", 840m, 05-Mai-2014, *Carpinus*, sieving, leg. Stüben,P. & Schütte,A., det. Stüben,P.
Acallorneuma ingoi<br>G. Osella & Zuppa, 2002	KP776643 [new]<br>1776-PST<br>658nt	ZFMK-TIS-24100<br>ZFMK-DNA-0171624088	Italy, Lazio, Cassino, Terme Varroniane, N41°28'59" E13°49'52", 47m, 06-Mai-2014, Laurus forest, sieving, leg. Stüben,P. & Schütte,A., det. Stüben,P.
Acallorneuma mainardii<br>A. & F. Solari, 1909	KP776638 [28]<br>1785-PST<br>658nt	ZFMK-TIS-24109<br>ZFMK-DNA-0171624076	Italy, Sardinia Isl., NW of Ussassai, above Cant. Arqueri, N39°48'60" E09°21'59", 976m, 11-Mai-2014, *Quercus*, sieving, leg. Stüben,P. & Schütte,A., det. Stüben,P.
Acallorneuma montisalbi<br>G. Osella & Zuppa, 2002	KP776633 [28]<br>IT1098c<br>658nt	ZFMK-TIS-cIT1098c	Italy, Sardinia Isl. East, SW of Siniscola, Monte Albo, N40°32'07" E09°36'13", 748m, 26-Sep-2010, *Quercus ilex*, limestone, leg. Stüben,P., det. Stüben,P.
Acallorneuma montisalbi<br>G. Osella & Zuppa, 2002	KP776635 [28]<br>IT1066c<br>658nt	ZFMK-TIS-cIT1066c<br>ZFMK-DNA-0112704608	Italy, Sardinia Isl. East, W of Siniscola, Monte Albo, N40°33'37" E09°38'01", 778m, 26-Sep-2010, *Quercus ilex*, cliff, limestone, leg. Stüben,P., det. Stüben,P.

**Name**<br>**Authority**<br>**Additional Information**	**GenBank Acc No (Ref.)**<br>**Specimen ID**<br>**Sequence Length**	**ZFMK Tissue ID**<br>**ZFMK DNA Sample ID**<br>**(SDEI DNA Sample ID)**	**Locality, GPS, Collection Date, Plant, Collector, Identifier**
Acallorneuma montisalbi<br>G. Osella & Zuppa, 2002	MG229745 [new]<br>1813-PST<br>658nt	ZFMK-TIS-24118<br>ZFMK-DNA-0171624056	Italy, Sardinia Isl., near Lula, below Mt. Turuddó, N40°28'29" E09°31'31", 583m, 18-Mai-2014, *Quercus ilex*, sieving, leg. Stüben,P. & Schütte,A., det. Stüben,P.
Acallorneuma reitteri<br>Mainardi, 1906	KP776639 [28]<br>1780-PST<br>658nt	ZFMK-TIS-24104<br>ZFMK-DNA-0171624084	Italy, Abruzzo, Castel di Ieri, Forca Caruso, Cantoniera, N42°04'16" E13°42'17", 1092m, 07-Mai-2014, *Corylus, Acer*, sieving, leg. Stüben,P. & Schütte,A., det. Stüben,P.
Acallorneuma reitteri<br>Mainardi, 1906	KP776640 [28]<br>1782-PST<br>658nt	ZFMK-TIS-24106<br>ZFMK-DNA-0171624073	Italy, Abruzzo, Castel di Ieri, Forca Caruso, Cantoniera, N42°04'16" E13°42'17", 1092m, 07-Mai-2014, *Corylus, Acer*, sieving, leg. Stüben,P. & Schütte,A., det. Stüben,P.
Acallorneuma reitteri<br>Mainardi, 1906	KP776641 [28]<br>1777-PST<br>658nt	ZFMK-TIS-24101<br>ZFMK-DNA-0171624087	Italy, Molise, Montenero Val Cocchiara, N41°43'22" E14°04'54", 915m, 06-Mai-2014, *Quercus, Rubus, Fraxinus*, sieving, leg. Stüben,P. & Schütte,A., det. Stüben,P.
Acallorneuma sardiniense<br>G. Osella & Zuppa, 2002	JX181781 [09]<br>IT1096c<br>658nt	ZFMK-TIS-cIT1096c	Italy, Sardinia Isl. East, N of Dorgali, M. Tuttavista (Gipfel), N40°22'46" E09°38'22", 801m, 06-Okt-2010, *Quercus ilex*, leg. Stüben,P., det. Stüben,P.
Acallorneuma sardiniense<br>G. Osella & Zuppa, 2002	KU170191 [28]<br>1783-PST<br>658nt	ZFMK-TIS-24107<br>ZFMK-DNA-0171624074	Italy, Sardinia Isl., N of Tortoli, P. Pedra Longa, N40°01'16" E09°41'17", 343m, 10-Mai-2014, *Pistacia*, sieving, leg. Stüben,P. & Schütte,A., det. Stüben,P.
Acalyptus carpini<br>(Fabricius, 1792)	MK891076 [new]<br>979-PSP<br>658nt	ZFMK-TIS-3124<br>ZFMK-DNA-0100426221	Germany, Lower Saxony (NI), near Hannover, 2 km W of Kananohe, N52°28'26" E09°38'39", 49m, 05-Apr-2012, *Salix cinerea*, Salix shrubs, beating, leg. Sprick,P., det. Sprick,P.
Adexius scrobipennis<br>Gyllenhal, 1834	EU286454 [01]<br>I-0020-AdS<br>658nt	ZFMK-TIS-cI0020	France, Isere, 9 km N of Grenoble, Mont St. Martin, N45°16'15" E05°40'44", 813m, 12-Aug-2005, *Fagus, Fraxinus, Quercus*, limestone, leg. Stüben,P., det. Stüben,P.
Adosomus roridus<br>(Pallas, 1781)	MK891935 [new]<br>2203-JKR<br>658nt	ZFMK-TIS-23941<br>ZFMK-DNA-0169170579	Slovakia, Pezinok, Pezinok, Grinava, N48°15'59" E17°14'02", 151m, 15-Mai-2014, *Artemisia vulgaris*, collecting by hand, leg. F. Trnka, det. Trnka,F.
Aeoniacalles aeonii aeonii<br>(Wollaston, 1864)	FJ716567 [01]<br>E-689-aeo<br>658nt	ZFMK-TIS-cE689<br>ZFMK-DNA-0100404855	Spain, Canary Islands, Tenerife, NE of La Laguna, Anaga Mts., Afur, N28°33'04" W16°15'12", 831m, 01-Okt-2008, *Aeonium cuneatum*, leg. Astrin,J. & Stüben,P., det. Stüben,P.
Aeoniacalles aeonii aeonii<br>(Wollaston, 1864)	GU987800 [01]<br>C-0101-aeo<br>658nt	ZFMK-TIS-cC0101<br>ZFMK-DNA-0100400148	Spain, Canary Islands, Tenerife, Anaga Mts., 10 km NE of La Laguna, Afur, N28°33'10" W16°14'59", 300m, 29-Dez-2003, *Aeonium cuneatum*, leg. Stüben,P., det. Stüben,P.
Aeoniacalles aeonii aeonii<br>(Wollaston, 1864)	GU988009 [01]<br>E-704-aeo<br>658nt	ZFMK-TIS-cE704<br>ZFMK-DNA-0100404851	Spain, Canary Islands, Tenerife, SW of Los Silos, Teno Mts. near Masca, Los Carizales, N28°19'11" W16°52'08", 434m, 03-Okt-2008, *Aeonium tabulaeforme*, leg. Astrin,J. & Stüben,P., det. Stüben,P.
Aeoniacalles aeonii aeonii<br>(Wollaston, 1864)	KC783832 [40]<br>747-PST<br>658nt	ZFMK-TIS-3084<br>ZFMK-DNA-0100448140	Spain, Canary Islands, Tenerife, Anaga Mts. near Batan de Arriba, N28°32'27" W16°17'57", 746m, 06-Feb-2012, *Aeonium urbicum*, beating, leg. Stüben,P. & Schütte,A., det. Stüben,P.
Aeoniacalles aeonii bodegensis<br>(Stüben, 2000)	GU987927 [01]<br>E-547-bod<br>656nt	ZFMK-TIS-cE547<br>ZFMK-DNA-0100404996	Spain, Canary Islands, Tenerife, East Anaga Mts., 19 km NE of La Laguna, Lomo de las Bodegas, N28°33'43" W16°09'25", 500m, 22-Dez-2003, *Aeonium holochrysum*, leg. Stüben,P., det. Stüben,P.

**Name**<br>**Authority**<br>**Additional Information**	**GenBank Acc No (Ref.)**<br>**Specimen ID**<br>**Sequence Length**	**ZFMK Tissue ID**<br>**ZFMK DNA Sample ID**<br>**(SDEI DNA Sample ID)**	**Locality, GPS, Collection Date, Plant, Collector, Identifier**
Aeoniacalles aeonisimilis<br>(Stüben, 2000)	FJ716577 [01]<br>E-725-aes<br>658nt	ZFMK-TIS-cE725<br>ZFMK-DNA-0100404830	Spain, Canary Islands, La Gomera, SE of Hermigua near Casas del Palmar, N28°09'29" W17°09'37", 627m, 08-Okt-2008, *Aeonium*, thermophilic brushwood, leg. Astrin,J. & Stüben,P., det. Stüben,P.
Aeoniacalles aeonisimilis<br>(Stüben, 2000)	JN627854 [02]<br>ES1007<br>658nt	ZFMK-TIS-cES1007<br>ZFMK-DNA-0112704662	Spain, Canary Islands, La Gomera, El Cedro, 28°8'19.44"N 17°12'51.53"W, 773m, 06-Dez-2009, *Persea indica*, leg. Stüben,P., det. Stüben,P.
Aeoniacalles aeonisimilis<br>(Stüben, 2000)	JN627855 [02]<br>ES1011<br>658nt	ZFMK-TIS-cES1011<br>ZFMK-DNA-0112704649	Spain, Canary Islands, La Gomera, El Cedro, Mirador de El Bailadero, 28°7'27"N 17°12'29"W, 1015m, 09-Dez-2009, *Aeonium subplanum*, leg. Stüben,P., det. Stüben,P.
Aeoniacalles aeonisimilis<br>(Stüben, 2000)	JN627858 [02]<br>ES1038<br>658nt	ZFMK-TIS-cES1038<br>ZFMK-DNA-0112704628	Spain, Canary Islands, La Gomera, near Tamargada **(type locality)**, 28°11'32"N 17°13'54"W, 609m, 26-Feb-2010, *Aeonium subplanum*, leg. Stüben,P., det. Stüben,P.
Aeoniacalles aeonisimilis<br>(Stüben, 2000)	MG229794 [40]<br>2714-PST<br>658nt	ZFMK-TIS-23864<br>ZFMK-DNA-0171661285	Spain, Canary Islands, La Gomera, Mirador de Bailadero, Cumbre de Juan Tome, N28°07'24" W17°12'32", 1018m, 27-Dez-2014, *Aeonium subplanum*, beating, leg. Stüben,P., det. Stüben,P.
Aeoniacalles argillosus<br>(Boheman, 1837)	GU987802 [01]<br>C-0105-arg<br>658nt	ZFMK-TIS-cC0105<br>ZFMK-DNA-0100400223	Spain, Canary Islands, El Hierro, 2 km E of Sabinosa, La Tabla, N27°44'54" W18°04'53", 182m, 31-Dez-2006, *Kleinia neriifolia*, leg. Stüben,P., det. Stüben,P.
Aeoniacalles argillosus<br>(Boheman, 1837)	GU988026 [01]<br>E-736-arg<br>658nt	ZFMK-TIS-cE736<br>ZFMK-DNA-0100404819	Spain, Canary Islands, La Gomera, Hermigua, Ermita de San Juan, N28°09'39" W17°12'19", 513m, 09-Okt-2008, *Kleinia*, leg. Astrin,J. & Stüben,P., det. Stüben,P.
Aeoniacalles argillosus<br>(Boheman, 1837)	JN627856 [02]<br>ES1031<br>658nt	ZFMK-TIS-cES1031<br>ZFMK-DNA-0112704638	Spain, Canary Islands, La Gomera, E of Hermigua, El Palmar, 28°9'34"N 17°10'01"W, 411m, 27-Jan-2010, *Kleinia*, thermophilic brushwood, leg. Stüben,P., det. Stüben,P.
Aeoniacalles argillosus<br>(Boheman, 1837)	FJ716539 [01]<br>C-0103-arg<br>658nt	ZFMK-TIS-cC0103<br>ZFMK-DNA-0100400222	Spain, Canary Islands, La Palma, 4.5 km NE of Garafia, 1 km N of El Mudo, N28°50'36" W17°54'07", 254m, 09-Jul-2006, *Kleinia neriifolia*, leg. Stüben,P., det. Stüben,P.
Aeoniacalles argillosus<br>(Boheman, 1837)	GU987839 [01]<br>E-0221-arg<br>658nt	ZFMK-TIS-cE0221<br>ZFMK-DNA-0100400296	Spain, Canary Islands, La Palma, 9 km NW of Las Caletas, El Remo, N28°33'07" W17°53'09", 63m, 29-Jun-2006, *Kleinia neriifolia*, leg. Stüben,P., det. Stüben,P.
Aeoniacalles argillosus<br>(Boheman, 1837)	GU987840 [01]<br>E-0222-arg<br>658nt	ZFMK-TIS-cE0222<br>ZFMK-DNA-0100400297	Spain, Canary Islands, La Palma, 4 km NW of Tijarafe, N28°43'19" W17°58'47", 35m, 06-Jul-2006, *Kleinia neriifolia*, leg. Stüben,P., det. Stüben,P.
Aeoniacalles argillosus<br>(Boheman, 1837)	GU987841 [01]<br>E-0223-arg<br>658nt	ZFMK-TIS-cE0223<br>ZFMK-DNA-0100400298	Spain, Canary Islands, La Palma, 2.5 km SW of Puntagorda, Llanos de Fagundo, N28°45'22" W18°00'12", 172m, 15-Jul-2006, *Kleinia neriifolia*, leg. Stüben,P., det. Stüben,P.
Aeoniacalles argillosus<br>(Boheman, 1837)	MG229709 [40]<br>1561-JKR<br>658nt	ZFMK-TIS-3609<br>ZFMK-DNA-0155635668	Spain, Canary Islands, La Palma, Juan Adalid, N28°50'38" W17°54'09", 247m, 29-Jan-2013, *Kleinia neirofolia*, beating, leg. Krátky,J., det. Krátky,J.
Aeoniacalles argillosus<br>(Boheman, 1837)	GU987928 [01]<br>E-550-arg<br>658nt	ZFMK-TIS-cE550<br>ZFMK-DNA-0100404993	Spain, Canary Islands, Tenerife, East Anaga Mts., 19 km NE of La Laguna, Lomo de las Bodegas, N28°33'43" W16°09'25", 500m, 22-Dez-2003, *Kleinia*, leg. Stüben,P., det. Stüben,P.
Aeoniacalles cf. *aeonisimilis*	JN627857 [02]<br>ES1035<br>658nt	ZFMK-TIS-cES1035<br>ZFMK-DNA-0112704625	Spain, Canary Islands, La Gomera, NE of Taguluche, Lomo del Carretón, 28°9'19"N 17°18'40"W, 551m, 22-Feb-2010, *Aeonium holochrysum*, leg. Stüben,P., det. Stüben,P.

**Name**<br>**Authority**<br>**Additional Information**	**GenBank Acc No (Ref.)**<br>**Specimen ID**<br>**Sequence Length**	**ZFMK Tissue ID**<br>**ZFMK DNA Sample ID**<br>**(SDEI DNA Sample ID)**	**Locality, GPS, Collection Date, Plant, Collector, Identifier**
Aeoniacalles grancanariensis<br>(Stüben, 2000)	GU988039 [01]<br>E-752-gra<br>636nt	ZFMK-TIS-cE752<br>ZFMK-DNA-0100405072	Spain, Canary Islands, Gran Canaria, Las Lagunetas, Bco. La Mina; 1300m, N27°59'17" W15°35'10" , 1300m, 01-Jul-1999, leg. Stüben,P., det. Stüben,P.
Aeoniacalles grancanariensis<br>(Stüben, 2000)	JN555592 [02]<br>6-PST<br>648nt	ZFMK-TIS-2D100447041<br>ZFMK-DNA-0100437972	Spain, Canary Islands, Gran Canaria, Marzagan, Caldera de Bandama, N28°02'07" W15°27'36", 401m, 23-Dez-2010, *Aeonium manriqueorum*, beating, leg. Stüben,P., det. Stüben,P.
Aeoniacalles grancanariensis<br>(Stüben, 2000)	JN555593 [02]<br>24-PST<br>658nt	ZFMK-TIS-2D100446911<br>ZFMK-DNA-0100438106	Spain, Canary Islands, Gran Canaria, Las Lagunetas, Barranco de la Mina, N27°59'56" W15°35'13", 1252m, 10-Dez-2010, *Aeonium undulatum*, beating, leg. Stüben,P., det. Stüben,P.
Aeoniacalles neptunus<br>(Wollaston, 1854)	MG229763 [40]<br>1954-PST<br>658nt	ZFMK-TIS-24033<br>ZFMK-DNA-0169170384	Portugal, Madeira, Ilhas Selvagens, Selvagem Pequena, around Pico do Veado, N30°02'09" W16°01'39", 15m, 07-Okt-2014, *Cistanche phelypaea*, sieving, leg. Stüben,P., det. Stüben,P.
Aeoniacalles neptunus<br>(Wollaston, 1854)	MG229768 [40]<br>1966-PST<br>658nt	ZFMK-TIS-24045<br>ZFMK-DNA-0169170383	Portugal, Madeira, Ilhas Selvagens, Selvagem Pequena, W of Pico do Veado, N30°02'10" W16°01'40", 30m, 20-Dez-2014, *Cistanche phelypaea*, sieving, leg. Stüben,P., det. Stüben,P.
Aeoniacalles tabladoensis<br>Stüben & Astrin, 2011	GU987801 [01]<br>C-0102-aes<br>658nt	ZFMK-TIS-cC0102<br>ZFMK-DNA-0100400146	Spain, Canary Islands, La Palma, 6.5 km E of Garafia, El Tablado, El Portal, N28°50'04" W17°52'36", 300m, 12-Jul-2003, *Aeonium palmense*, leg. Stüben,P., det. Stüben,P.
Aglycyderes setifer<br>Westwood, 1864	MK347555 [40]<br>2969-PST<br>658nt	(SDEI-DNA-2969-PST)	Spain, Canary Islands, El Hierro, S El Pinar, N27°42'03" W17°58'59", 804m, 17-Apr-2016, *Kleinia*, leg. Stüben & Schütte, det. Stüben,P.
Aglycyderes setifer<br>Westwood, 1864	MK892395 [40]<br>2872-PST<br>658nt	ZFMK-TIS-25877<br>ZFMK-DNA-0169166920	Spain, Canary Islands, El Hierro, W of Sabinosa near Montana Escoba, N27°44'54" W18°08'32", 303m, 11-Apr-2016, *Kleinia*, beating, leg. Stüben,P. & Schütte,A., det. Stüben,P.
Aglycyderes setifer<br>Westwood, 1864	KC783756 [40]<br>45-PST<br>658nt	ZFMK-TIS-2D100447007<br>ZFMK-DNA-0100438079	Spain, Canary Islands, Gran Canaria, N of Maspalomas near San Bartolome, N27°48'02" W15°34'57", 320m, 09-Jan-2011, *Kleinia*, beating, leg. Stüben,P., det. Stüben,P.
Aglycyderes setifer<br>Westwood, 1864	KC784297 [40]<br>726-PST<br>658nt	ZFMK-TIS-3063<br>ZFMK-DNA-0100448152	Spain, Canary Islands, Tenerife, El Tanque nach Ruigomez near El Lomo Morin, N28°11'01" W16°47'24", 745m, 24-Jan-2012, *Sonchus congestus*, beating, leg. Stüben,P., det. Stüben,P.
Aglycyderes tavakiliani<br>Menier, 1974	KC783798 [40]<br>91-PST<br>638nt	ZFMK-TIS-2D100446893<br>ZFMK-DNA-0100438015	Spain, Canary Islands, La Gomera, La Caleta – Punta San Lorenzo, N28°10'15" W17°9'38", 41m, 25-Feb-2011, *Kleinia neriifolia*, beating, leg. Stüben,P., det. Stüben,P.
Aglycyderes tavakiliani<br>Menier, 1974	MK891617 [40]<br>1724-PST<br>658nt	ZFMK-TIS-24442<br>ZFMK-DNA-0155622457	Spain, Canary Islands, La Palma, SW of Garafia near El Castillo (Barranco), N28°48'15" W17°57'55", 475m, 29-Dez-2013, *Ficus*, beating, leg. Stüben,P., det. Stüben,P.
Aglycyderes tavakiliani<br>Menier, 1974	MK891747 [40]<br>2000-JKR<br>658nt	ZFMK-TIS-23640<br>ZFMK-DNA-0169170501	Spain, Canary Islands, La Palma, El Remo, Paisaje protegio de Tamanca, N28°33'08" W17°53'07", 50m, 12-Feb-2014, *Kleinia, Euphorbia, Lavandula*, beating, leg. Krátky,J., det. Krátky,J.
Aglycyderes tavakiliani<br>Menier, 1974	KC784296 [40]<br>725-PST<br>658nt	ZFMK-TIS-3062<br>ZFMK-DNA-0100446734	Spain, Canary Islands, Tenerife, Reserva Natural Especial Mailpais de la Rasco, N28°00'57" W16°41'19", 56m, 23-Jan-2012, beating, leg. Stüben,P., det. Stüben,P.
Aizobius robustirostris<br>(Desbrochers des Loges, 1870)	MK890950 [new]<br>825-PST<br>658nt	ZFMK-TIS-3738<br>ZFMK-DNA-0100414271	Portugal, Minho, NW of Ponte de Lima, Vila Nova de Cerveira, Rio Coura, N41°52'27" W08°41'57", 123m, 07-Mai-2012, *Umbilicus rupestris*, beating, leg. Stüben,P., det. Stüben,P.

**Name**<br>**Authority**<br>**Additional Information**	**GenBank Acc No (Ref.)**<br>**Specimen ID**<br>**Sequence Length**	**ZFMK Tissue ID**<br>**ZFMK DNA Sample ID**<br>**(SDEI DNA Sample ID)**	**Locality, GPS, Collection Date, Plant, Collector, Identifier**
Aizobius sedi<br>(Germar, 1818)	MK891965 [new]<br>2235-JKR<br>658nt	ZFMK-TIS-23973<br>ZFMK-DNA-0169170563	Slovakia, Topolcany, Solcany, PR Solcansky haj, N48°31'06" E18°13'37", 350m, 10-Okt-2014, *Sedum*, sieving, leg. Krátky,J., det. Krátky,J.
Allomalia quadrivirgata<br>(A. Costa, 1863)	MK891479 [new]<br>1549-PSP<br>658nt	ZFMK-TIS-3309<br>ZFMK-DNA-0155630480	Cyprus, Lemessos, Agios Tychon, Prov. Lemessos, N34°42'46" E33°08'15", 15m, 15-Apr-2013, *Tamarix* sp., Zucht aus Frucht, leg. Sprick,P., det. Sprick,P.
Allomalia quadrivirgata<br>(A. Costa, 1863)	KC784277 [new]<br>IT-0019w<br>619nt	ZFMK-TIS-2D100446558	Italy, Sardinia Isl. South, SE of Ballao, F. Flumendosa, N39°31'51" E09°23'27", 75m, 01-Okt-2010, *Tamarix*, leg. Stüben,P., det. Stüben,P.
Allomalia quadrivirgata<br>(A. Costa, 1863)	MK890800 [new]<br>IT-0037w<br>658nt	ZFMK-DNA-0100437834	Italy, Sardinia Isl. East, E of Siniscola, La Caletta, coast, N40°35'30" E09°45'25", 3m, 09-Okt-2010, *Tamarix*, leg. Stüben,P., det. Stüben,P.
Alocentron curvirostre<br>(Gyllenhal, 1833)	MK891926 [new]<br>2194-JKR<br>658nt	ZFMK-TIS-23932<br>ZFMK-DNA-0169170593	Czech Republic, Moravia, Znojmo, Konice, N48°49'55" E16°01'10", 320m, 27-Apr-2014, *Alcea* sp., collecting by hand, leg. Stejskal,R., det. Stejskal,R.
Amalus scortillum<br>(Herbst, 1795)	KC783927 [new]<br>348-JKR<br>658nt	ZFMK-TIS-20134<br>ZFMK-DNA-0100438735	Czech Republic, Bohemia or., Hradec Kralove, Placky, N50°13'48.497" E15°49'25.886", 230m, 29-Mai-2011, sweeping, leg. Krátky,J., det. Krátky,J.
Amalus scortillum<br>(Herbst, 1795)	KC784073 [new]<br>550-RST<br>658nt	ZFMK-TIS-20335<br>ZFMK-DNA-0100448429	Czech Republic, Moravia mer., Podyji NP, 0.9 km N of Hnanice - Stare vinice hill, N48°48'18.81" E15°59'26.12", 300m, 26-Sep-2011, dry heathland, sweeping, leg. Stejskal,R., det. Stejskal,R.
Amalus scortillum<br>(Herbst, 1795)	MK891696 [new]<br>1914-FBA<br>658nt	ZFMK-TIS-23305<br>ZFMK-DNA-0169170362	Greece, Central Macedonia, Serres, Achladohori, N41°19'00" E23°34'20", 670m, 12-Aug-2014, leg. Bayer & Brunner, det. Bayer,F.
Amalus scortillum<br>(Herbst, 1795)	MK891243 [new]<br>1163-JKR<br>658nt	ZFMK-TIS-3499<br>ZFMK-DNA-0100449552	Hungary, Jasz-Nagykun-Szolnok megye, Tiszaroff, N47°21'55" E20°27'32", 87m, 25-Mai-2012, *Polygonum aviculare*, sweeping, leg. Krátky,J., det. Krátky,J.
Amaurorhinus clermonti<br>Desbrochers des Loges, 1908<br>formerly: *Amaurorhinus clermonti clermonti*	MH051952 [33]<br>2736-PST<br>658nt	ZFMK-TIS-23886<br>ZFMK-DNA-0171661263	Spain, Canary Islands, Fuerteventura, La Pared (Jandia), N28°13'01" W14°13'10", 11m, 09-Jan-2015, *Chenopodiaceae*, sieving, leg. Stüben,P., det. Stüben,P.
Amaurorhinus clermonti<br>Desbrochers des Loges, 1908<br>formerly: *Amaurorhinus clermonti clermonti*	MK347528 [40]<br>2923-PST<br>658nt	(SDEI-DNA-2923-PST)	Spain, Canary Islands, Lanzarote, Órzola, N29°13'29" W13°27'31", 4m, 03-Jan-2017, *Suedae vera*, sifting, leg. Stüben,P., det. Stüben,P.
Amaurorhinus clermonti<br>Desbrochers des Loges, 1908<br>formerly: *Amaurorhinus clermonti salvagis* (Syn!)	MK891723 [40]<br>1955-PST<br>658nt	ZFMK-TIS-24034<br>ZFMK-DNA-0169170385	Portugal, Madeira, Ilhas Selvagens, Selvagem Pequena, around Pico do Veado, N30°02'09" W16°01'39", 15m, 07-Okt-2014, *Patellifolia patellaris*, sieving, leg. Stüben,P., det. Stüben,P.
Amaurorhinus clermonti<br>Desbrochers des Loges, 1908<br>formerly: *Amaurorhinus clermonti salvagis* (Syn!)	MK891728 [40]<br>1963-PST<br>658nt	ZFMK-TIS-24042<br>ZFMK-DNA-0169170393	Portugal, Madeira, Ilhas Selvagens, Selvagem Grande, slope S of (near) Cave Kidd, N30°8'30" W15°51'30", 51m, 20-Okt-2014, *Lotus glaucus*, sieving, leg. Stüben,P., det. Stüben,P.

**Name**<br>**Authority**<br>**Additional Information**	**GenBank Acc No (Ref.)**<br>**Specimen ID**<br>**Sequence Length**	**ZFMK Tissue ID**<br>**ZFMK DNA Sample ID**<br>**(SDEI DNA Sample ID)**	**Locality, GPS, Collection Date, Plant, Collector, Identifier**
Amaurorhinus clermonti<br>Desbrochers des Loges, 1908<br>formerly: *Amaurorhinus clermonti salvagis* (Syn!)	MK891729 [40]<br>1968-PST<br>658nt	ZFMK-TIS-24047<br>ZFMK-DNA-0169170381	Portugal, Madeira, Ilhas Selvagens, Selvagem Pequena, W of Pico do Veado, N30°02'10" W16°01'40", 30m, 20-Dez-2014, *Suaeda vera*, sieving, leg. Stüben,P., det. Stüben,P.
Amaurorhinus monizianus<br>(Wollaston, 1860)	MK892349 [40]<br>2732-PST<br>658nt	ZFMK-TIS-23882<br>ZFMK-DNA-0171661267	Spain, Canary Islands, Fuerteventura, Barranco de los Molinos, N28°32'34" W14°03'41", 9m, 06-Jan-2015, *Chenopodiaceae*, sieving, leg. Stüben,P., det. Stüben,P.
Amaurorhinus monizianus<br>(Wollaston, 1860)	MH051957 [33]<br>2786-PST<br>658nt	ZFMK-TIS-26140<br>ZFMK-DNA-0169166959	Portugal, Madeira, Ilhas Desertas, Deserta Grande, Doca, N32°30'51" W16°30'37", 5m, 02-Apr-2015, *Crithmum maritimum (ger. "Meeresfenchel")*, collecting by hand, leg. Stüben,P., det. Stüben,P.
Amaurorhinus monizianus<br>(Wollaston, 1860)	MK891726 [40]<br>1960-PST<br>658nt	ZFMK-TIS-24039<br>ZFMK-DNA-0169170390	Portugal, Madeira, Ilhas Selvagens, Selvagem Grande, Cave Inferno, N30°08'18" W15°51'51", 18m, 09-Okt-2014, leg. Stüben,P., det. Stüben,P.
Amaurorhinus monizianus<br>(Wollaston, 1860)	MK891727 [40]<br>1962-PST<br>658nt	ZFMK-TIS-24041<br>ZFMK-DNA-0169170392	Portugal, Madeira, Ilhas Selvagens, Selvagem Grande, Cave Inferno, N30°08'18" W15°51'51", 18m, 09-Okt-2014, leg. Stüben,P., det. Stüben,P.
Amaurorhinus monizianus<br>(Wollaston, 1860)<br>formerly: *A. folwacznyi, A. punctipennis* - Syn!	KC783789 [40]<br>80-PST<br>641nt	ZFMK-TIS-2D100446968<br>ZFMK-DNA-0100438020	Spain, Canary Islands, La Gomera, Valle Gran Rey, N28°5'55" W17°20'53", 18m, 15-Feb-2011, *under beach vegetation*, sieving, leg. Stüben,P., det. Stüben,P.
Amaurorhinus monizianus<br>(Wollaston, 1860)<br>formerly: *A. folwacznyi, A. punctipennis* - Syn!	MK891591 [40]<br>1664-PST<br>658nt	ZFMK-TIS-24382<br>ZFMK-DNA-0155622526	Spain, Canary Islands, La Gomera, Valle Gran Rey, Playa del Ingles, N28°05'56" W17°20'53", 10m, 03-Dez-2013, *Chenopodiacea, Astydamia latifolia*, sieving, leg. Stüben,P., det. Stüben,P.
Amaurorhinus monizianus<br>(Wollaston, 1860)<br>formerly: *A. folwacznyi, A. punctipennis* - Syn!	MK891593 [40]<br>1668-PST<br>658nt	ZFMK-TIS-24386<br>ZFMK-DNA-0155622522	Spain, Canary Islands, La Gomera, Tamargada, Playa de la Sepultura, N28°12'01" W17°14'40", 28m, 04-Dez-2013, *Euphorbia aphylla, Limonium, Astydamia latifolia,*, sieving, leg. Stüben,P., det. Stüben,P.
Amaurorhinus monizianus<br>(Wollaston, 1860)<br>formerly: *A. folwacznyi, A. punctipennis* - Syn!	MN614444 [40]<br>ES1028<br>658nt	ZFMK-TIS-cES1028<br>ZFMK-DNA-0112704641	Spain, Canary Islands, La Gomera, below Agulo/Lepe **(type locality)**, 28°11'17"N 17°11'12"W, 19m, 15-Jan-2010, gardens: trash pile near the beach, leg. Stüben,P., det. Stüben,P.
Amaurorhinus monizianus<br>(Wollaston, 1860)	MK347534 [40]<br>2939-PST<br>658nt	(SDEI-DNA-2939-PST)	Spain, Canary Islands, Lanzarote, SE of Guatiza near Urb. los Cocoteros (Salinas), N29°03'24" W13°27'57", 4m, 15-Jan-2017, *Zygophyllum fontanesli*, sifting, leg. Stüben,P., det. Stüben,P.
Amaurorhinus monizianus<br>(Wollaston, 1860)	MK891631 [40]<br>1756-PST<br>658nt	ZFMK-TIS-24474<br>ZFMK-DNA-0155622441	Spain, Canary Islands, La Palma, N of Puntallana, Playa de Nogales, N28°45'27" W17°44'08", 10m, 22-Jan-2014, *Crithmum maritimum (ger: "Meeresfenchel")*, beating, leg. Stüben,P., det. Stüben,P.
Amaurorhinus monizianus<br>(Wollaston, 1860)<br>formerly: *A. folwacznyi, A. punctipennis* - Syn!	KC784312 [40]<br>757-PST<br>658nt	ZFMK-TIS-3094<br>ZFMK-DNA-0100448135	Spain, Canary Islands, Tenerife, near San Juan de Rambla, Playa del Socorro, coast, N28°23'38" W16°36'11", 5m, 20-Feb-2012, *Crithmum maritimum (german: "Meeresfenchel")*, beating/sifting, leg. Stüben,P., det. Stüben,P.
Andrion regensteinense<br>(Herbst, 1797)	KC784088 [new]<br>537-RST<br>658nt	ZFMK-TIS-20322<br>ZFMK-DNA-0100448447	Czech Republic, Bohemia occ., Belysov Nature Reserve, 2.5 km SE of Chudenice, N49°26'49.54" E13°11'53.68", 540m, 28-Aug-2011, *Cytisus scoparius*, beating, leg. Stejskal,R., det. Stejskal,R.

**Name**<br>**Authority**<br>**Additional Information**	**GenBank Acc No (Ref.)**<br>**Specimen ID**<br>**Sequence Length**	**ZFMK Tissue ID**<br>**ZFMK DNA Sample ID**<br>**(SDEI DNA Sample ID)**	**Locality, GPS, Collection Date, Plant, Collector, Identifier**
Andrion regensteinense<br>(Herbst, 1797)	KC784022 [new]<br>214-PSP<br>658nt	ZFMK-TIS-2D100439156<br>ZFMK-DNA-0100439828	Germany, Lower Saxony (NI), Brelingen, Hannover region, N52°33'35" E09°41'04", 70m, 16-Apr-2011, *Cytisus scoparius*, sand pit, beating, leg. Sprick,P., det. Sprick,P.
Andrion regensteinense<br>(Herbst, 1797)	MK891008 [new]<br>902-CBR<br>658nt	ZFMK-TIS-3815<br>ZFMK-DNA-0100449709	Luxembourg, 10 km NE of Mersch, 2 km NE of Nommern, N49°48'15" E06°11'16", 340m, 01-Mai-2011, *Cytisus scoparius*, beating, leg. Braunert,C., det. Braunert,C.
Anoplus roboris<br>Suffrian, 1840	MK892176 [new]<br>2468-PSP<br>658nt	ZFMK-TIS-23440<br>ZFMK-DNA-0171661806	Germany, Lower Saxony (NI), Harz National Park, Torfhaus, Marienbruch, N51°49'54" E10°31'46", 625m, 30-Mai-2013, *Alnus glutinosa*, beating, leg. Sprick,P., det. Sprick,P.
Anthonomus conspersus<br>Desbrochers des Loges, 1868<br>Synonym of A. pedicularius?	MK892183 [new]<br>2477-PSP<br>658nt	ZFMK-TIS-23449<br>ZFMK-DNA-0171661796	Germany, Lower Saxony (NI), Harz National Park, Torfhaus, Marienbruch, N51°49'54" E10°31'46", 625m, 18-Jul-2013, *Sorbus aucuparia*, beating, leg. Sprick,P., det. Sprick,P.
Anthonomus conspersus<br>Desbrochers des Loges, 1868<br>Synonym of *A. pedicularius*?	MK891022 [new]<br>919-CBR<br>658nt	ZFMK-TIS-3832<br>ZFMK-DNA-0100449693	Luxembourg, 15 km SW of Wiltz, 2 km SSW of Boulaide, N49°52'16" E05°48'26", 400m, 01-Jul-2011, *Sorbus aucuparia*, beating, leg. Braunert,C., det. Braunert,C.
Anthonomus pedicularius<br>(Linnaeus, 1758)	MK891938 [new]<br>2206-JKR<br>658nt	ZFMK-TIS-23944<br>ZFMK-DNA-0169170582	Czech Republic, Moravia, Vevcice, N48°58'8.5" E16°01'31", 325m, 04-Apr-2014, *Crataegus* sp., beating, leg. Stejskal,R., det. Stejskal,R.
Anthonomus pedicularius<br>(Linnaeus, 1758)	KC784001 [new]<br>251-PSP<br>658nt	ZFMK-TIS-2D100438542<br>ZFMK-DNA-0100439792	Germany, Lower Saxony (NI), Hemmingen, Hannover region, N52°19'07" E09°44'02", 54m, 08-Mai-2011, *Crataegus monogyna*, fallow, beating, leg. Sprick,P., det. Sprick,P.
Anthonomus pedicularius<br>(Linnaeus, 1758)	MK891768 [new]<br>2025-JKR<br>658nt	ZFMK-TIS-23665<br>ZFMK-DNA-0169170476	Italy, Sicilia Isl. (PA), Palermo, Bosco di Ficuzza, N37°51'50" E13°24'56", 995m, 22-Apr-2014, *Crataegus*, beating, leg. Krátky,J., det. Krátky,J.
Anthonomus pedicularius<br>(Linnaeus, 1758)	KC783878 [new]<br>126-PST<br>609nt	ZFMK-TIS-2D100440301<br>ZFMK-DNA-0100438189	Morocco, S of Berkane, Beni Snassen Mts., Vallee Zegzel, N34°48'56" W02°24'01", 678m, 12-Mai-2011, *Crataegus*, beating, leg. Stüben,P., det. Stüben,P./vid. Behne,L.
Anthonomus phyllocola<br>(Herbst, 1795)	MK891927 [new]<br>2195-JKR<br>658nt	ZFMK-TIS-23933<br>ZFMK-DNA-0169170592	Czech Republic, Moravia, Vevcice, N48°58'08" E16°01'31", 325m, 04-Apr-2014, *Pinus sylvestris*, beating, leg. Stejskal,R., det. Stejskal,R.
Anthonomus phyllocola<br>(Herbst, 1795)	KC784028 [new]<br>220-PSP<br>658nt	ZFMK-TIS-2D100438541<br>ZFMK-DNA-0100439834	Germany, Lower Saxony (NI), Berkhof, Hannover region, N52°36'54" E09°43'49", 36m, 17-Apr-2011, *Pinus sylvestris*, former sand pit, beating, leg. Sprick,P., det. Sprick,P.
Anthonomus phyllocola<br>(Herbst, 1795)	MK892306 [new]<br>2607-PSP<br>658nt	ZFMK-TIS-23581<br>ZFMK-DNA-0171661947	Germany, Bavaria (BY), Unterfranken, Ebern, N50°05'17" E10°45'52", 345m, 14-Jun-2014, *Pinus sylvestris*, leg. Sprick,P., det. Sprick,P.
Anthonomus phyllocola<br>(Herbst, 1795)	MK891752 [new]<br>2006-JKR<br>658nt	ZFMK-TIS-23646<br>ZFMK-DNA-0169170495	Slovakia, Zvolen, Michalkova, N48°32'05" E19°07'53", 503m, 11-Okt-2014, sieving, leg. Krátky,J., det. Krátky,J.
Anthonomus pomorum<br>(Linnaeus, 1758)	KC784166 [new]<br>594-PSP<br>658nt	ZFMK-TIS-20378<br>ZFMK-DNA-0100448673	Germany, Lower Saxony (NI), Bad Zwischenahn, Aschhausen, N53°12'00" E08°04'00", 11m, 10-Jun-2011, *Malus* sp., beating, leg. Sprick,P., det. Sprick,P.
Anthonomus pomorum<br>(Linnaeus, 1758)	MK891643 [new]<br>1796-PST<br>658nt	ZFMK-TIS-24130<br>ZFMK-DNA-0171624068	Germany, Rhineland-Palatinate (RLP), Ahr valley, Dernau, below Krausberg near Steinbergsmühle, N50°31'40" E07°02'43", 204m, 29-Mai-2014, beating, leg. Stüben,P., det. Stüben,P.

**Name**<br>**Authority**<br>**Additional Information**	**GenBank Acc No (Ref.)**<br>**Specimen ID**<br>**Sequence Length**	**ZFMK Tissue ID**<br>**ZFMK DNA Sample ID**<br>**(SDEI DNA Sample ID)**	**Locality, GPS, Collection Date, Plant, Collector, Identifier**
Anthonomus rectirostris<br>(Linnaeus, 1758)	KC784014 [new]<br>228-PSP<br>657nt	ZFMK-TIS-2D100439143<br>ZFMK-DNA-0100439815	Germany, Lower Saxony (NI), Lemmie, Hannover region, Gehrdener Berg, N52°17'44" E09°35'23", 100m, 22-Apr-2011, *Prunus padus*, field edge, beating, leg. Sprick,P., det. Sprick,P.
Anthonomus rectirostris<br>(Linnaeus, 1758)	MK890864 [new]<br>432-RGO<br>658nt	ZFMK-TIS-20218<br>ZFMK-DNA-0100449402	Poland, Lublin, N51°13'23.7" E22°31'16.6", 192m, 03-Aug-2011, leg. Gosik,R., det. Gosik,R.
Anthonomus rubi<br>(Herbst, 1795)	MK892003 [new]<br>2275-JKR<br>658nt	ZFMK-TIS-24206<br>ZFMK-DNA-0169170989	Austria, Styria, Hochschwab, Spitzkögel, N47°35'27" E15°01'26", 1659m, 27-Jun-2014, alpine grassland, sieving, leg. Krátky,J., det. Krátky,J.
Anthonomus rubi<br>(Herbst, 1795)	MK892027 [new]<br>2301-JKR<br>658nt	ZFMK-TIS-24232<br>ZFMK-DNA-0169170967	Czech Republic, Bohemia, Krinec, PP Chotuc, N50°15'59" E15°06'59", 236m, 02-Jul-2014, *Agrimonia eupatoria*, sweeping, leg. Krátky,J., det. Krátky,J.
Anthonomus rubi<br>(Herbst, 1795)	KC784174 [new]<br>485-PST<br>658nt	ZFMK-TIS-20270<br>ZFMK-DNA-0100449067	France, Isere, St.-Jeam-de-Bournay, N45°30'28" E05°09'09", 403m, 22-Jul-2011, beating, leg. Stüben,P., det. Stüben,P.
Anthonomus rubi<br>(Herbst, 1795)	MK890931 [new]<br>800-PST<br>658nt	ZFMK-TIS-3713<br>ZFMK-DNA-0100413758	Germany, Rhineland-Palatinate (RLP), E of Treis-Karden, Pommern, Pommerner Goldberg, high road (vineyard), N50°10'14" E07°16'29", 95m, 10-Jun-2012, beating, leg. Stüben,P., det. Stüben,P.
Anthonomus rubi<br>(Herbst, 1795)	MK891082 [new]<br>985-PSP<br>658nt	ZFMK-TIS-3130<br>ZFMK-DNA-0100426863	Germany, Lower Saxony (NI), Braunschweig, Riddagshausen, N52°16'37" E10°33'49", 82m, 27-Apr-2012, *Rubus idaeus*, beating, leg. Sprick,P., det. Sprick,P.
Anthonomus rubi<br>(Herbst, 1795)	MK892308 [new]<br>2609-PSP<br>658nt	ZFMK-TIS-23583<br>ZFMK-DNA-0171661949	Germany, Bavaria (BY), Unterfranken, Ebern, N50°05'00" E10°45'40", 320m, 14-Jun-2014, *Rubus fruticosus agg.*, leg. Sprick,P., det. Sprick,P.
Anthonomus rubi<br>(Herbst, 1795)	MK890991 [new]<br>883-CBR<br>658nt	ZFMK-TIS-3796<br>ZFMK-DNA-0100449728	Luxembourg, 10 km E of Luxembourg, 2 km S of Mensdorf, N49°38'09" E06°17'48", 330m, 11-Apr-2011, *Rubus*, beating, leg. Braunert,C., det. Braunert,C.
Anthonomus rubi<br>(Herbst, 1795)	MK891226 [new]<br>1146-JKR<br>658nt	ZFMK-TIS-3482<br>ZFMK-DNA-0100449562	Slovakia, Nove Zamky, Kamenica nad Hronom, N47°49'52" E18°44'20", 181m, 12-Mai-2012, *Fragaria*, sweeping, leg. Krátky,J., det. Krátky,J.
Anthonomus rubi<br>(Herbst, 1795)	MK891985 [new]<br>2256-JKR<br>658nt	ZFMK-TIS-23994<br>ZFMK-DNA-0169170536	Slovakia, Zvolen, Hronska Dubrava, NPR Boky, N48°33'58" E19°01'10.5", 472m, 12-Okt-2014, sieving, leg. Krátky,J., det. Krátky,J.
Anthonomus rubi<br>(Herbst, 1795)	MK892056 [new]<br>2332-JKR<br>658nt	ZFMK-TIS-24263<br>ZFMK-DNA-0169170933	Slovakia, Povazska Bystrica, Bodina, N49°06'46" E18°33'25", 450m, 13-Jul-2014, *Agrimonia eupatoria*, sweeping, leg. Krátky,J., det. Krátky,J.
Anthonomus rubripes<br>Gyllenhal, 1836	KC784103 [new]<br>510-RST<br>658nt	ZFMK-TIS-20295<br>ZFMK-DNA-0100448468	Czech Republic, Moravia mer., Naceraticky kopec hill, 4.5 km SE of Znojmo, N48°50'7.28" E16°5'46.34", 350m, 11-Jun-2011, *Potentilla argentata*, sweeping, leg. Stejskal,R., det. Stejskal,R.
Anthonomus sorbi<br>Germar, 1821	KC784146 [new]<br>632-PSP<br>658nt	ZFMK-TIS-20415<br>ZFMK-DNA-0100448638	Germany, Schleswig-Holstein (SH), Neukirchen, Lkr. Nordfriesland, N54°51'53" E08°44'51", -1m, 28-Aug-2011, *Crataegus monogyna*, wayside shrub, beating, leg. Sprick,P., det. Sprick,P.
Anthonomus ulmi<br>(De Geer, 1775)	MK891540 [new]<br>1629-JKR<br>658nt	ZFMK-TIS-3677<br>ZFMK-DNA-0155635600	Slovakia, Nove Zamky, Cenkov, Dunaj riv., N47°47'50" E18°31'48", 108m, 20-Mai-2013, *Ulmus campestris*, beating, leg. Krátky,J., det. Krátky,J.

**Name**<br>**Authority**<br>**Additional Information**	**GenBank Acc No (Ref.)**<br>**Specimen ID**<br>**Sequence Length**	**ZFMK Tissue ID**<br>**ZFMK DNA Sample ID**<br>**(SDEI DNA Sample ID)**	**Locality, GPS, Collection Date, Plant, Collector, Identifier**
Anthribus nebulosus<br>Forster, 1771	MK892006 [new]<br>2278-JKR<br>658nt	ZFMK-TIS-24209<br>ZFMK-DNA-0169170992	Austria, Styria, Hochschwab, Stallmauer, N47°35'29" E15°02'20", 1260m, 27-Jun-2014, mountain forest, sweeping, leg. Krátky,J., det. Krátky,J.
Anthribus nebulosus<br>Forster, 1771	MK890828 [new]<br>265-PSP<br>658nt	ZFMK-TIS-2D100438536<br>ZFMK-DNA-0100439783	Germany, Lower Saxony (NI), Helstorfer Moor, Resse, Hannover region, N52°31'50" E09°36'35", 43m, 14-Mai-2011, *Corylus avellana, Salix aurita*, edge of broad-leaved forest (east side), beating, leg. Sprick,P., det. Sprick,P.
Anthribus nebulosus<br>Forster, 1771	MK892177 [new]<br>2469-PSP<br>658nt	ZFMK-TIS-23441<br>ZFMK-DNA-0171661807	Germany, Lower Saxony (NI), Aerzen-Königsförde, Lkr. Hameln-Pyrmont, N52°03'58" E09°16'17", 92m, 02-Jun-2013, *Picea abies*, garden hedge, beating, leg. Sprick,P., det. Sprick,P.
Anthribus nebulosus<br>Forster, 1771	MK892198 [new]<br>2493-PSP<br>658nt	ZFMK-TIS-23465<br>ZFMK-DNA-0171661783	Germany, Saxony-Anhalt (ST), Harz National Park, Drei Annen Hohne, Hohneklippen, Hohnekopf, N51°46'37" E10°41'55", 840m, 24-Apr-2014, *Picea abies*, beating, leg. Sprick,P., det. Sprick,P.
Anthribus nebulosus<br>Forster, 1771	MK892213 [new]<br>2509-PSP<br>658nt	ZFMK-TIS-23481<br>ZFMK-DNA-0171661764	Germany, Lower Saxony (NI), Hannover region, Brelingen, N52°33'52" E09°41'09", 75m, 08-Mai-2014, *Quercus robur*, beating, leg. Sprick,P., det. Sprick,P.
Aparopion chevrolati<br>(Jaquelin Duval, 1855)	MK891356 [new]<br>1406-JKR<br>658nt	ZFMK-TIS-4175<br>ZFMK-DNA-0155628527	France, Cevennes Mts., 1 km N of Pommiers, N43°57'43" E03°36'44", 456m, 13-Jun-2013, *Castanea sativa*, sieving, leg. Krátky,J., det. Krátky,J.
Aparopion chevrolati<br>(Jaquelin Duval, 1855)	MK891336 [new]<br>1364-PST<br>658nt	ZFMK-TIS-4660<br>ZFMK-DNA-0155628569	Italy, Abruzzo, Gran Sasso, Vado di Sole, N42°23'46" E13°47'17", 1636m, 14-Aug-2013, *Fagus*, sieving, leg. Stüben,P., det. Stüben,P.
Aparopion suturidens<br>Reitter, 1891	MK891765 [new]<br>2021-JKR<br>658nt	ZFMK-TIS-23661<br>ZFMK-DNA-0169170491	Italy, Sicilia Isl., Messina, Nebrodi NP, Monte Soro, N37°55'28" E14°39'57", 1563m, 20-Apr-2014, sieving, leg. Krátky,J., det. Krátky,J.
Aphytobius sphaerion<br>(Boheman, 1845)	MK891189 [new]<br>1106-JKR<br>658nt	ZFMK-TIS-3442<br>ZFMK-DNA-0100449591	Czech Republic, Moravia, Dukovany, PR Dukovansky mlyn, N49°05'45" E16°11'53", 304m, 01-Mai-2012, *Silene vulgaris*, sieving, leg. Krátky,J., det. Krátky,J.
Aphytobius veronicae<br>(Frivaldszky, 1884)<br>*A. sphaerion* - resyn in 2015	MK891230 [new]<br>1150-JKR<br>658nt	ZFMK-TIS-3486<br>ZFMK-DNA-0100449558	Hungary, Pest megye, Gödöllö, Mariabesnyo, N47°34'48" E19°24'11", 190m, 13-Mai-2012, *Silene latofolia* ssp. *Alba*, beating, leg. Krátky,J., det. Krátky,J.
Apion cruentatum<br>Walton, 1844	KC784057 [new]<br>588-PSP<br>632nt	ZFMK-TIS-20372<br>ZFMK-DNA-0100448402	Germany, Lower Saxony (NI), Braunschweig, Hafen, N52°18'53" E10°29'36", 66m, 07-Jun-2011, *Rumex thyrsiflorus*, nutrient-poor grassland, dipnet, leg. Sprick,P., det. Sprick,P.
Apion frumentarium<br>(Linnaeus, 1758)	MK891448 [new]<br>1513-PSP<br>658nt	ZFMK-TIS-3273<br>ZFMK-DNA-0155633316	Denmark, Syddanmark, Emmerlev Klev (Højer), N54°59'53" E08°39'08", 3m, 30-Aug-2012, *Rumex crispus*, dipnet, leg. Sprick,P., det. Sprick,P.
Apion frumentarium<br>(Linnaeus, 1758)	KC784136 [new]<br>642-PSP<br>658nt	ZFMK-TIS-20425<br>ZFMK-DNA-0100448625	Germany, Saxony-Anhalt (ST), Drömling, Mannhausen, island, N52°25'38" E11°12'07", 55m, 08-Sep-2011, *Rumex obtusifolius*, beating, leg. Sprick,P., det. Sprick,P.
Apion frumentarium<br>(Linnaeus, 1758)	KC784186 [new]<br>476-PST<br>658nt	ZFMK-TIS-20261<br>ZFMK-DNA-0100449363	Germany, Rhineland-Palatinate (RLP), Mosellus area, Kobern-Gondorf, "Sürzer Höfe", ruderal area, N50°20'20" E07°25'25", 330m, 30-Sep-2011, *Rumex*, hand-collecting, leg. Stüben,P., det. Stüben,P.
Apion frumentarium<br>(Linnaeus, 1758)	MK892336 [new]<br>2657-PSP<br>658nt	ZFMK-TIS-23807<br>ZFMK-DNA-0171661342	Germany, Lower Saxony (NI), Hannover, Herrenhausen, N52°23'22.5" E09°40'36", 47m, 02-Aug-2014, *Rumex maritimus*, dipnet, leg. Sprick,P., det. Sprick,P.

**Name**<br>**Authority**<br>**Additional Information**	**GenBank Acc No (Ref.)**<br>**Specimen ID**<br>**Sequence Length**	**ZFMK Tissue ID**<br>**ZFMK DNA Sample ID**<br>**(SDEI DNA Sample ID)**	**Locality, GPS, Collection Date, Plant, Collector, Identifier**
Apion frumentarium<br>(Linnaeus, 1758)	MK891054 [new]<br>954-CBR<br>658nt	ZFMK-TIS-3867<br>ZFMK-DNA-0100449657	Luxembourg, 10 km E of Luxembourg, 1 km SW of Mensdorf, N49°39'00" E06°17'40", 240m, 21-Jun-2012, *Rumex acetosa*, beating, leg. Braunert,C., det. Braunert,C.
Apion frumentarium<br>(Linnaeus, 1758)	KC784193 [new]<br>455-RGO<br>658nt	ZFMK-TIS-20241<br>ZFMK-DNA-0100449377	Poland, Rudnik, N51°14'31.02" E22°32'22.92", 191m, 03-Okt-2011, leg. Gosik,R., det. Gosik,R.
Apion haematodes haematodes<br>Kirby, 1808	KC784124 [new]<br>650-PSP<br>658nt	ZFMK-TIS-20433<br>ZFMK-DNA-0100448608	Germany, Lower Saxony (NI), Hannover, Vahrenheide, Kugelfangtrift, N52°25'22" E09°45'25'', 51m, 02-Okt-2011, *Rumex acetosella*, nutrient-poor sand grassland, dipnet, leg. Sprick,P., det. Behne,L.
Apion haematodes haematodes<br>Kirby, 1808	MK891669 [40]<br>1871-PST<br>658nt	ZFMK-TIS-26074<br>ZFMK-DNA-0171606095	Portugal, Madeira, Pico do Areeiro – Paso de Poiso, N32°42'54" W16°54'05", 1489m, 07-Jul-2014, *Rumex acetosella*, beating, leg. Stüben,P., det. Stüben,P.
Apion haematodes haematodes<br>Kirby, 1808	KC783840 [new]<br>190-PST<br>658nt	ZFMK-TIS-2D100438690<br>ZFMK-DNA-0100438124	Morocco, Atlas Mts., S of Ifrane, Michliffen, N33°24'20" W05°06'11", 2052m, 22-Mai-2011, beating, leg. Stüben,P., det. Behne,L.
Apion haematodes haematodes<br>Kirby, 1808	MK890957 [new]<br>837-PST<br>658nt	ZFMK-TIS-3750<br>ZFMK-DNA-0100414254	Portugal, Minho, S of Viana, Castelo do Neiva, N41°37'07" W08°48'35", 8m, 10-Mai-2012, *Rumex*, beating, leg. Stüben,P., det. Stüben,P.
Apion rubiginosum<br>Grill, 1893	MK891736 [new]<br>1978-JKR<br>655nt	ZFMK-TIS-23618<br>ZFMK-DNA-0169170523	Czech Republic, Bohemia, Zastava, N50°06'42" E15°50'10", 234m, 11-Mrz-2014, sieving, leg. Krátky,J., det. Krátky,J.
Apion rubiginosum<br>Grill, 1893	KC784123 [new]<br>649-PSP<br>655nt	ZFMK-TIS-20432<br>ZFMK-DNA-0100448607	Germany, Lower Saxony (NI), Hannover, Vahrenheide, Kugelfangtrift, N52°25'22" E09°45'25'', 51m, 02-Okt-2011, *Rumex acetosella*, nutrient-poor sand grassland, dipnet, leg. Sprick,P., det. Sprick,P.
Apion rubiginosum<br>Grill, 1893	MK891690 [new]<br>1907-PST<br>655nt	ZFMK-TIS-24012<br>ZFMK-DNA-0169170411	Germany, North Rhine-Westphalia (NRW), lower Rhine, 2 km S of Brüggen, Elmpter creek, N51°13'24" E06°11'31", 57m, 03-Sep-2014, beating, leg. Stüben,P., det. Stüben,P.
Apoderus coryli<br>(Linnaeus, 1758)	MK890843 [new]<br>317-JKR<br>658nt	ZFMK-TIS-20103<br>ZFMK-DNA-0100438766	Czech Republic, Bohemia or., Dvakacovice, N49°58'35.447" E15°54'2.243", 245m, 14-Mai-2011, *Corylus avellana*, beating, leg. Krátky,J., det. Krátky,J.
Apoderus coryli<br>(Linnaeus, 1758)	MK892304 [new]<br>2605-PSP<br>658nt	ZFMK-TIS-23579<br>ZFMK-DNA-0171661945	Germany, Bavaria (BY), Unterfranken, Ebern, N50°05'01" E10°45'26", 334m, 14-Jun-2014, *Carpinus betulus*, leg. Sprick,P., det. Sprick,P.
Archarius crux<br>(Fabricius, 1777)	MK890896 [new]<br>602-PSP<br>643nt	ZFMK-TIS-20385<br>ZFMK-DNA-0100448656	Germany, Lower Saxony (NI), Koldingen, Hannover region, Leine river, N52°16'30'' E09°48'31'', 57m, 12-Jun-2011, *Salix viminalis*, beating, leg. Sprick,P., det. Sprick,P.
Archarius crux<br>(Fabricius, 1777)	MK891471 [new]<br>1538-PSP<br>658nt	ZFMK-TIS-3298<br>ZFMK-DNA-0155633280	Germany, Lower Saxony (NI), Koldingen, Hannover region, "Leine" river bank, N52°16'27" E09°48'29", 58m, 13-Jun-2013, *Salix fragilis*, beating, leg. Sprick,P., det. Sprick,P.
Archarius crux<br>(Fabricius, 1777)	MK891566 [new]<br>892-CBR<br>599nt	ZFMK-TIS-3805<br>ZFMK-DNA-0155630483	Luxembourg, 10 km E of Luxembourg, 0.5 km W of Uebersyren, N49°38'19" E06°16'31", 240m, 19-Apr-2011, *Salix (fragilis)*, beating, leg. Braunert,C., det. Braunert,C.
Archarius pyrrhoceras<br>(Marsham, 1802)	MK891575 [new]<br>229-PSP<br>658nt	ZFMK-TIS-2D100439142<br>ZFMK-DNA-0155630454	Germany, Lower Saxony (NI), Lemmie, Hannover region, Gehrdener Berg, N52°17'44" E09°35'23", 100m, 22-Apr-2011, *Quercus robur*, field edge, beating, leg. Sprick,P., det. Sprick,P.

**Name**<br>**Authority**<br>**Additional Information**	**GenBank Acc No (Ref.)**<br>**Specimen ID**<br>**Sequence Length**	**ZFMK Tissue ID**<br>**ZFMK DNA Sample ID**<br>**(SDEI DNA Sample ID)**	**Locality, GPS, Collection Date, Plant, Collector, Identifier**
Archarius pyrrhoceras<br>(Marsham, 1802)	MK892190 [new]<br>2485-PSP<br>658nt	ZFMK-TIS-23457<br>ZFMK-DNA-0171661788	Germany, Lower Saxony (NI), Hannover region, Brelingen, N52°33'52" E09°41'09", 75m, 18-Apr-2014, *Quercus robur*, beating, leg. Sprick,P., det. Sprick,P.
Archarius pyrrhoceras<br>(Marsham, 1802)	MK890995 [new]<br>887-CBR<br>658nt	ZFMK-TIS-3800<br>ZFMK-DNA-0100449724	Luxembourg, 10 km E of Luxembourg, 2 km S of Mensdorf, N49°38'09" E06°17'48", 330m, 11-Apr-2011, *Quercus*, beating, leg. Braunert,C., det. Braunert,C.
Archarius salicivorus<br>(Paykull, 1792)	MK891578 [new]<br>285-PSP<br>658nt	ZFMK-TIS-2D100439083<br>ZFMK-DNA-0155630457	Germany, Lower Saxony (NI), Berkhof, Hannover region, N52°36'44" E09°43'57", 36m, 21-Mai-2011, *Salix aurita*, former gravel pit, beating, leg. Sprick,P., det. Sprick,P.
Archarius salicivorus<br>(Paykull, 1792)	MK890988 [new]<br>880-CBR<br>658nt	ZFMK-TIS-3793<br>ZFMK-DNA-0100449731	Luxembourg, 10 km E of Luxembourg, 2 km SE of Mensdorf, N49°38'18" E06°19'04", 300m, 09-Apr-2011, *Salix (caprea)*, beating, leg. Braunert,C., det. Braunert,C.
Archarius salicivorus<br>(Paykull, 1792)	MK892036 [new]<br>2312-JKR<br>658nt	ZFMK-TIS-24243<br>ZFMK-DNA-0169170953	Slovakia, Tatry Mts., Dolina Siedmich prame?ov, N49°12'57" E20°17'46", 981m, 05-Jul-2014, *Salix*, sweeping, leg. Krátky,J., det. Krátky,J.
Archeophloeus inermis<br>(Boheman, 1842)	MK891504 [new]<br>1590-JKR<br>658nt	ZFMK-TIS-3638<br>ZFMK-DNA-0155635636	Czech Republic, Moravia, Cejc - Spidlaky, N48°55'47" E16°58'03", 184m, 01-Mai-2013, sieving, leg. Krátky,J., det. Krátky,J.
Aspidapion acerifoliae<br>Suppantschitsch, 1996	KC783775 [40]<br>66-PST<br>658nt	ZFMK-TIS-2D100446979<br>ZFMK-DNA-0100437950	Spain, Canary Islands, La Gomera, Agulo – Las Rosas, N28°11'26" W17°12'6", 388m, 16-Dez-2010, *Lavatera acerifolia*, beating, leg. Stüben,P., det. Stüben,P.
Aspidapion acerifoliae<br>Suppantschitsch, 1996	KC784293 [40]<br>719-PST<br>658nt	ZFMK-TIS-3056<br>ZFMK-DNA-0100448168	Spain, Canary Islands, Tenerife, 4 km W of Buenavista del Norte **(type locality)**, N28°21'55" W16°52'59", 155m, 15-Jan-2012, *Lavatera acerifolia*, beating, leg. Stüben,P., det. Stüben,P.
Aspidapion aeneum<br>(Fabricius, 1775)	KC783990 [new]<br>270-PSP<br>658nt	ZFMK-TIS-2D100438510<br>ZFMK-DNA-0100439773	Germany, Hesse (HE), Wiesbaden-Erbenheim, N50°03'02" E08°18'12", 274m, 06-Sep-2011, *Malva sylvestris*, dipnet, leg. Sprick,P., det. Sprick,P.
Aspidapion aeneum<br>(Fabricius, 1775)	MK890933 [new]<br>802-PST<br>658nt	ZFMK-TIS-3715<br>ZFMK-DNA-0100414296	Germany, Rhineland-Palatinate (RLP), E of Treis-Karden, Pommern, Pommerner Goldberg, high road (vineyard), N50°10'14" E07°16'29", 95m, 10-Jun-2012, *Malva*, beating, leg. Stüben,P., det. Stüben,P.
Aspidapion aeneum<br>(Fabricius, 1775)	MK891458 [new]<br>1523-PSP<br>658nt	ZFMK-TIS-3283<br>ZFMK-DNA-0155633313	Germany, Lower Saxony (NI), Mittelrode, Hannover region, N52°12'20" E09°39'20", 97m, 09-Sep-2012, *Alcea rosea*, house garden, beating, leg. Sprick,P., det. Sprick,P.
Aspidapion aeneum<br>(Fabricius, 1775)	MK347568 [new]<br>2992-PST<br>658nt	(SDEI-DNA-2992-PST)	Greece, Macedonia, Thessaloniki, Hortiatis Mt., 1 km S Hortiatis, N40°35'58" E23°06'13", 822m, 19-Jul-2017, *Malva*, leg. Stüben,P., det. Stüben,P.
Aspidapion aeneum<br>(Fabricius, 1775)	MK891337 [new]<br>1367-PST<br>658nt	ZFMK-TIS-4663<br>ZFMK-DNA-0155628566	Italy, Abruzzo, Galascio nach Ofena, „Oasi Colonia Frasca", N42°18'32" E13°43'59", 619m, 15-Aug-2013, *Fraxinus, Quercus*, beating, leg. Stüben,P., det. Stüben,P.
Aspidapion aeneum<br>(Fabricius, 1775)	KC783880 [new]<br>116-PST<br>658nt	ZFMK-TIS-2D100440306<br>ZFMK-DNA-0100438194	Morocco, S of Berkane, Beni Snassen Mts., Vallee Zegzel, N34°49'33" W02°22'37", 590m, 11-Mai-2011, *Malvaceae*, beating, leg. Stüben,P., det. Stüben,P./vid. Behne,L.
Aspidapion aeneum<br>(Fabricius, 1775)	MK891307 [new]<br>1293-PST<br>658nt	ZFMK-TIS-4109<br>ZFMK-DNA-0100426183	Spain, Community of Madrid, SW of Lozoya, Pinilla del Valle, N40°55'44" W03°49'29", 1108m, 03-Mai-2013, *Malveae*, beating, leg. Stüben,P. & Schütte,A., det. Stüben,P.

**Name**<br>**Authority**<br>**Additional Information**	**GenBank Acc No (Ref.)**<br>**Specimen ID**<br>**Sequence Length**	**ZFMK Tissue ID**<br>**ZFMK DNA Sample ID**<br>**(SDEI DNA Sample ID)**	**Locality, GPS, Collection Date, Plant, Collector, Identifier**
Aspidapion radiolus<br>(Marsham, 1802)	KC783989 [new]<br>271-PSP<br>658nt	ZFMK-TIS-2D100439100<br>ZFMK-DNA-0100439772	Germany, Lower Saxony (NI), Nordstemmen, Lkr. Hildesheim, N52°09'00" E09°47'00", 72m, 17-Mai-2011, *Malva moschata*, garden, beating, leg. Sprick,P., det. Sprick,P.
Aspidapion radiolus<br>(Marsham, 1802)<br>formerly: *Aspidapion radiolus chalybeipenne*	KC783769 [40]<br>59-PST<br>658nt	ZFMK-TIS-2D100446989<br>ZFMK-DNA-0100438061	Spain, Canary Islands, La Gomera, Hermigua, Las Nuevitas, Barranquillos los Alamos, N28°09'31" W17°11'09", 251m, 13-Dez-2010, *Malvae*, beating, leg. Stüben,P., det. Stüben,P.
Aspidapion radiolus<br>(Marsham, 1802)<br>formerly: *Aspidapion radiolus chalybeipenne*	KC783743 [40]<br>22-PST<br>631nt	ZFMK-TIS-2D100447032<br>ZFMK-DNA-0100438104	Spain, Canary Islands, Gran Canaria, Moya, Barranco de los Tilos (upper section), N28°04'15" W15°36'27", 798m, 07-Dez-2010, *Malvae*, beating, leg. Stüben,P., det. Stüben,P.
Aspidapion radiolus<br>(Marsham, 1802)<br>formerly: *Aspidapion radiolus chalybeipenne*	MK891602 [40]<br>1684-PST<br>658nt	ZFMK-TIS-24402<br>ZFMK-DNA-0155622513	Spain, Canary Islands, La Palma, near San Isidro, N28°38'48" W17°47'51", 526m, 18-Dez-2013, *Malvaceae*, beating, leg. Stüben,P., det. Stüben,P.
Aspidapion radiolus<br>(Marsham, 1802)	MK891047 [new]<br>946-CBR<br>658nt	ZFMK-TIS-3859<br>ZFMK-DNA-0100449672	Luxembourg, 10 km E of Luxembourg, Mensdorf, N49°39'33" E06°18'13", 240m, 10-Jun-2012, *Alcea rosea*, pooter, leg. Braunert,C., det. Braunert,C.
Aspidapion radiolus<br>(Marsham, 1802)<br>formerly: *Aspidapion radiolus chalybeipenne*	MK891663 [40]<br>1858-PST<br>658nt	ZFMK-TIS-26061<br>ZFMK-DNA-0171606108	Portugal, Madeira, Faja da Ovelha, N32°46'24" W17°13'48", 550m, 02-Jul-2014, *Malva*, beating, leg. Stüben,P., det. Stüben,P.
Aspidapion radiolus<br>(Marsham, 1802)<br>formerly: *Aspidapion radiolus chalybeipenne*	MK892366 [40]<br>2787-PST<br>658nt	ZFMK-TIS-26141<br>ZFMK-DNA-0169166960	Portugal, Madeira, E of Funchal, W of Garajau, Rua do Lazareto, N32°38'49" W16°53'17", 37m, 03-Apr-2015, *Malva*, collecting by hand, leg. Stüben,P., det. Stüben,P.
Aspidapion radiolus<br>(Marsham, 1802)<br>formerly: *Aspidapion radiolus chalybeipenne*	KC784286 [40]<br>701-PST<br>658nt	ZFMK-TIS-3038<br>ZFMK-DNA-0100448175	Spain, Canary Islands, Tenerife, Teno Mts. near Erjos, N28°19'31" W16°48'32", 1017m, 04-Jan-2012, *Malvae*, beating, leg. Stüben,P., det. Stüben,P.
Aspidapion validum<br>(Germar, 1817)	MK892163 [new]<br>2454-PSP<br>658nt	ZFMK-TIS-23426<br>ZFMK-DNA-0171661819	Germany, Berlin (BE), Berlin-Tegel, N52°34'16" E13°16'58", 35m, 20-Okt-2013, *Alcea rosea*, collecting by hand, leg. Sprick,P., det. Sprick,P.
Aspidapion validum<br>(Germar, 1817)	MK891046 [new]<br>945-CBR<br>658nt	ZFMK-TIS-3858<br>ZFMK-DNA-0100449671	Luxembourg, 10 km E of Luxembourg, Mensdorf, N49°39'33" E06°18'13", 240m, 10-Jun-2012, *Alcea rosea*, pooter, leg. Braunert,C., det. Braunert,C.
Aspidiotes cottyi<br>(Lucas, 1858)	MK891492 [new]<br>1575-JKR<br>658nt	ZFMK-TIS-3623<br>ZFMK-DNA-0155635654	Morocco, Marrakech-Tensift-El Haouz, Tamanar, 3 km SW, N31°29'35" W09°43'00", 88m, 13-Apr-2013, *grassy steppe*, sweeping, leg. Krátky,J., det. Krátky,J.
Asproparthenis punctiventris<br>(Germar, 1824)	MK891901 [new]<br>2168-JKR<br>658nt	ZFMK-TIS-23904<br>ZFMK-DNA-0169170619	Czech Republic, Moravia, Bozice, N48°49'21" E16°15'55", 220m, 02-Mai-2014, collecting by hand, leg. Stejskal,R., det. Stejskal,R.
Attactagenus dispar<br>(Graells, 1858)	MK890948 [new]<br>823-PST<br>658nt	ZFMK-TIS-3736<br>ZFMK-DNA-0100414269	Portugal, Minho, Ponte de Lima, Rio Lima, N41°45'48" W08°35'40", 13m, 06-Mai-2012, beating, leg. Stüben,P., det. Stüben,P.

**Name**<br>**Authority**<br>**Additional Information**	**GenBank Acc No (Ref.)**<br>**Specimen ID**<br>**Sequence Length**	**ZFMK Tissue ID**<br>**ZFMK DNA Sample ID**<br>**(SDEI DNA Sample ID)**	**Locality, GPS, Collection Date, Plant, Collector, Identifier**
Attelabus nitens<br>(Scopoli, 1763)	MK891396 [new]<br>1456-PSP<br>658nt	ZFMK-TIS-3216<br>ZFMK-DNA-0155633373	Germany, Lower Saxony (NI), Berkhof, Hannover region, N52°36'38" E09°43'50", 38m, 30-Jun-2012, *Quercus robur*, edge of a forest with Oaks, beating, leg. Sprick,P., det. Sprick,P.
Attelabus nitens<br>(Scopoli, 1763)	KC784119 [new]<br>666-FBA<br>658nt	ZFMK-TIS-20447<br>ZFMK-DNA-0100448601	Greece, Peloponnese, Messinia, W of Mt. Taygetos, E of Saidona, N36°53'01'' E22°17'07'', 680m, 23-Mai-2011, leg. Bahr,F., det. Bahr,F.
Attelabus variolosus<br>Fabricius, 1801	MK890812 [new]<br>156-PST<br>658nt	ZFMK-TIS-2D100440271<br>ZFMK-DNA-0100438159	Morocco, SW of Berkane near Taforalt, Beni Snassen Mts., N34°47'52" W02°25'31" , 839m, 15-Mai-2011, *Quercus ilex*, beating, leg. Stüben,P., det. Behne,L.
Aulacobaris angusta<br>(Brullé, 1832)	MK891568 [new]<br>1129-JKR<br>658nt	ZFMK-TIS-3465<br>ZFMK-DNA-0155630468	Slovakia, Komarno, Imel, N47°54'34" E18°09'16", 112m, 11-Mai-2012, *Sisymbrium ?orientale*, beating, leg. Krátky,J., det. Krátky,J.
Aulacobaris cf. *cuprirostris*	KC783846 [new]<br>178-PST<br>658nt	ZFMK-TIS-2D100440249<br>ZFMK-DNA-0100438137	Morocco, Atlas Mts., SE of Ifrane, Tizi-n-Tretten, N33°27'25" W05°02'16", 1936m, 19-Mai-2011, *Sinapis*, beating, leg. Stüben,P., det. Stüben,P./vid. Behne,L.
Aulacobaris cuprirostris<br>(Fabricius, 1787)	MK891294 [new]<br>1263-PST<br>658nt	ZFMK-TIS-4079<br>ZFMK-DNA-0100426436	Spain, Zaragoza, Aragon, NO of Calatayud, N41°22'20" W01°35'01", 703m, 30-Apr-2013, *Brassicaceae*, beating, leg. Stüben,P. & Schütte,A., det. Stüben,P.
Aulacobaris fallax<br>(H. Brisout de Barneville, 1870)	MK891763 [new]<br>2019-JKR<br>658nt	ZFMK-TIS-23659<br>ZFMK-DNA-0169170489	Italy, Sicilia Isl., Catania, Nicolosi, Monti Monpilieri, N37°36'20" E15°01'19", 670m, 20-Apr-2014, *Isatis tinctoria*, collecting by hand, leg. Krátky,J., det. Krátky,J.
Aulacobaris gudenusi<br>(Schultze, 1901)	KC784032 [new]<br>504-RST<br>658nt	ZFMK-TIS-20289<br>ZFMK-DNA-0100446702	Czech Republic, Moravia mer., 11 km SE of Znojmo, Jecmenist? near Dyjakovicky, N48°45'10.63" E16°8'11.16", 280m, 27-Mai-2011, *Rapistrum perenne*, beating, leg. Stejskal,R., det. Stejskal,R.
Aulacobaris gudenusi<br>(Schultze, 1901)	MK891570 [new]<br>1166-JKR<br>658nt	ZFMK-TIS-3502<br>ZFMK-DNA-0155630466	Hungary, Pest megye, Pecel, N47°29'50" E19°22'45", 192m, 26-Mai-2012, *Rapistrum perenne*, beating, leg. Krátky,J., det. Krátky,J.
Aulacobaris lepidii<br>(Germar, 1824)	MK891913 [new]<br>2181-JKR<br>658nt	ZFMK-TIS-23919<br>ZFMK-DNA-0169170605	Czech Republic, Moravia, Znojmo, Popice, N48°49'26" E16°01'14", 280m, 03-Apr-2014, *Berteroa incana*, collecting by hand, leg. Stejskal,R., det. Stejskal,R.
Aulacobaris lepidii<br>(Germar, 1824)	MK891106 [new]<br>1011-PSP<br>658nt	ZFMK-TIS-3156<br>ZFMK-DNA-0100426841	Germany, Lower Saxony (NI), Hannover, Stöcken, N52°24'23" E09°39'04.5", 43m, 07-Mai-2012, *Rorippa sylvestris*, shore of Leine river, soil-search, leg. Sprick,P., det. Sprick,P.
Aulacobaris lepidii<br>(Germar, 1824)	MK891905 [new]<br>2172-JKR<br>658nt	ZFMK-TIS-23910<br>ZFMK-DNA-0169170615	Slovakia, Nove Zamky, Kamenin, PP Cistiny, N47°51'55" E18°38'05", 111m, 22-Mai-2014, sweeping, leg. Krátky,J., det. Krátky,J.
Aulacobaris picicornis<br>(Marsham, 1802)	MK891774 [new]<br>2032-JKR<br>658nt	ZFMK-TIS-23672<br>ZFMK-DNA-0169170469	Czech Republic, Bohemia, Kosatky, N50°19'23" E14°39'58", 218m, 02-Mai-2014, *Reseda lutea*, collecting by hand, leg. Krátky,J., det. Stüben,P.
Aulacobaris picicornis<br>(Marsham, 1802)	MK892328 [new]<br>2630-PSP<br>658nt	ZFMK-TIS-23604<br>ZFMK-DNA-0171661922	Germany, Lower Saxony (NI), Hannover, Leinhausen, N52°23'44" E09°42'53", 53m, 22-Jun-2014, *Reseda lutea*, collecting by hand, leg. Sprick,P., det. Sprick,P.
Aulacobaris picicornis<br>(Marsham, 1802)	MK892144 [new]<br>2431-JKR<br>658nt	ZFMK-TIS-26008<br>ZFMK-DNA-0171600654	Slovakia, Nitra, Koli?any, Koli?ansky vrch, N48°20'15.5" E18°10'47", 261m, 21-Sep-2014, *Reseda lutea*, sweeping, leg. Krátky,J., det. Stüben,P.

**Name**<br>**Authority**<br>**Additional Information**	**GenBank Acc No (Ref.)**<br>**Specimen ID**<br>**Sequence Length**	**ZFMK Tissue ID**<br>**ZFMK DNA Sample ID**<br>**(SDEI DNA Sample ID)**	**Locality, GPS, Collection Date, Plant, Collector, Identifier**
Aulacobaris picicornis<br>(Marsham, 1802)	MK891182 [new]<br>1099-JKR<br>658nt	ZFMK-TIS-3435<br>ZFMK-DNA-0100449609	Spain, Andalucia, 3 km SW of Monda, N36°36'37" W04°51'04", 460m, 22-Apr-2012, *Reseda* sp., sweeping, leg. Krátky,J., det. Krátky,J.
Auletobius anceps<br>(Wollaston, 1864)	MK347576 [35]<br>3009-KRA<br>658nt	(SDEI-DNA-3009-KRA)	Spain, Canary Islands, El Hierro, SW of Frontera, Pista del Derrabado, N27°44'27" W18°03'52", 850m, 28-Jan-2017, *Laurus*, leg. Krátky,J., det. Krátky
Auletobius anceps<br>(Wollaston, 1864)	MK347577 [35]<br>3010-KRA<br>658nt	(SDEI-DNA-3010-KRA)	Spain, Canary Islands, El Hierro, N of El Pinar, Montana del Gajo, N27°43'44" W17°59'30", 1213m, 30-Jan-2017, *Laurus*, leg. Krátky,J., det. Krátky
Auletobius anceps<br>(Wollaston, 1864)	MK347584 [35]<br>3023-PST<br>658nt	(SDEI-DNA-3023-PST)	Spain, Canary Islands, El Hierro, SE Sabinosa, near El Gretime, N27°44'24" W18°04'47", 651m, 11-Apr-2016, leg. Stüben & Schütte, det. Stüben,P.
Auletobius anceps<br>(Wollaston, 1864)	KC783744 [20]<br>23-PST<br>617nt	ZFMK-TIS-2D100447033<br>ZFMK-DNA-0100438105	Spain, Canary Islands, Gran Canaria, San Mateo, La Lechuza, N28°00'12" W15°33'12", 1039m, 10-Dez-2010, *Chamaecytiscus proliferus*, beating, leg. Stüben,P., det. Stüben,P.
Auletobius anceps<br>(Wollaston, 1864)	KC783786 [20]<br>77-PST<br>631nt	ZFMK-TIS-2D100446971<br>ZFMK-DNA-0100438043	Spain, Canary Islands, La Gomera, Arure, N28°08'23" W17°18'29", 801m, 13-Feb-2011, *Cistus monspeliensis*, creek, beating, leg. Stüben,P., det. Stüben,P.
Auletobius anceps<br>(Wollaston, 1864)	KR817571 [20]<br>2717-PST<br>658nt	ZFMK-TIS-23867<br>ZFMK-DNA-0171661273	Spain, Canary Islands, La Gomera, Las Rosas, N28°09'59" W17°14'53", 614m, 29-Dez-2014, *Cistus*, beating, leg. Stüben,P., det. Stüben,P.
Auletobius anceps<br>(Wollaston, 1864)	KC784284 [20]<br>699-PST<br>658nt	ZFMK-TIS-3036<br>ZFMK-DNA-0100448188	Spain, Canary Islands, Tenerife, Teno Mts., Teno Alto, N28°20'32" W16°51'50", 830m, 03-Jan-2012, beating, leg. Stüben,P., det. Stüben,P.
Auletobius anceps<br>(Wollaston, 1864)	KC784298 [20]<br>727-PST<br>658nt	ZFMK-TIS-3064<br>ZFMK-DNA-0100448153	Spain, Canary Islands, Tenerife, La Tierra del Trigo, N28°21'37" W16°48'07", 372m, 24-Jan-2012, *Marcetella moquiniana*, beating, leg. Stüben,P., det. Stüben,P.
Auletobius convexifrons<br>(Wollaston, 1864)	KC783736 [20]<br>12-PST<br>629nt	ZFMK-TIS-2D100447035<br>ZFMK-DNA-0100437977	Spain, Canary Islands, Gran Canaria, Telde, El Palmital, N28°01'10" W15°27'17", 476m, 05-Dez-2010, *Tamarix canariensis*, beating, leg. Stüben,P., det. Stüben,P.
Auletobius cylindricollis<br>(Wollaston, 1864)	KR817563 [20]<br>1706-PST<br>658nt	ZFMK-TIS-24424<br>ZFMK-DNA-0155622487	Spain, Canary Islands, La Palma, NW of Puntallana, Cubo de la Galga, N28°45'38" W17°46'42", 537m, 27-Dez-2013, *Rubus*, beating, leg. Stüben,P., det. Stüben,P.
Auletobius cylindricollis<br>(Wollaston, 1864)	KR817566 [20]<br>1990-JKR<br>658nt	ZFMK-TIS-23630<br>ZFMK-DNA-0169170508	Spain, Canary Islands, La Palma, Cubo de Galga, N28°45'42" W17°46'34", 467m, 07-Feb-2014, *Rubus*, beating, leg. Krátky,J., det. Krátky,J.
Auletobius garajonay<br>Stüben, 2015	MK347575 [35]<br>3008-KRA<br>658nt	(SDEI-DNA-3008-KRA)	Spain, Canary Islands, El Hierro, Villa de Valverde, Ajare, N27°48'08" W17°55'10", 710m, 30-Jan-2017, *Cistus*, leg. Krátky,J., det. Krátky
Auletobius garajonay<br>Stüben, 2015	MK892475 [35]<br>2871-PST<br>658nt	ZFMK-TIS-4261<br>ZFMK-DNA-0169166921	Spain, Canary Islands, El Hierro, SE of Sabinosa near Las Tabladas, N27°44'24" W18°05'06", 716m, 11-Apr-2016, *Rubus*, beating, leg. Stüben,P. & Schütte,A., det. Stüben,P.
Auletobius garajonay<br>Stüben, 2015<br>**Paratype (DNAtype)**	KC783803 [20]<br>96-PST_3G2011<br>627nt	ZFMK-TIS-2D100446888<br>ZFMK-DNA-0100438007	Spain, Canary Islands, La Gomera, S of Hermigua, Meriga, N28°09'15" W17°14'10", 824m, 07-Feb-2011, *Rubus*, beating, leg. Stüben,P., det. Stüben,P.

**Name**<br>**Authority**<br>**Additional Information**	**GenBank Acc No (Ref.)**<br>**Specimen ID**<br>**Sequence Length**	**ZFMK Tissue ID**<br>**ZFMK DNA Sample ID**<br>**(SDEI DNA Sample ID)**	**Locality, GPS, Collection Date, Plant, Collector, Identifier**
Auletobius garajonay<br>Stüben, 2015<br>**Paratype (DNAtype)**	KR817568 [20]<br>2709-PST_23859<br>658nt	ZFMK-TIS-23859<br>ZFMK-DNA-0171661290	Spain, Canary Islands, La Gomera, E of Hermigua, Parque Natural de Majona near Casas del Palmar, N28°09'33" W17°09'36", 554m, 22-Dez-2014, *Rubus*, beating, leg. Stüben,P., det. Stüben,P.
Auletobius garajonay<br>Stüben, 2015<br>**Paratype (DNAtype)**	KR817569 [20]<br>2712-PST_23862<br>658nt	ZFMK-TIS-23862<br>ZFMK-DNA-0171661287	Spain, Canary Islands, La Gomera, N of Igualero, N28°06'29" W17°15'23", 1295m, 24-Dez-2014, *Cistus*, beating, leg. Stüben,P., det. Stüben,P.
Auletobius garajonay<br>Stüben, 2015<br>**Paratype (DNAtype)**	KR817570 [20]<br>2716-PST_23866<br>658nt	ZFMK-TIS-23866<br>ZFMK-DNA-0171661272	Spain, Canary Islands, La Gomera, Las Rosas, N28°09'59" W17°14'53", 614m, 29-Dez-2014, *Rubus*, beating, leg. Stüben,P., det. Stüben,P.
Auletobius maderensis<br>(Wollaston, 1854)	KR817564 [20]<br>1836-PST<br>658nt	ZFMK-TIS-26039<br>ZFMK-DNA-0171606121	Portugal, Madeira, S of Sao Jorge, Ilha, Rib. de S. Jorge, N32°48'37" W16°55'27", 235m, 28-Jun-2014, *Rubus*, beating, leg. Stüben,P., det. Stüben,P.
Auletobius maderensis<br>(Wollaston, 1854)	KR817565 [20]<br>1868-PST<br>658nt	ZFMK-TIS-26071<br>ZFMK-DNA-0171606105	Portugal, Madeira, E of Arco de S. Jorge, Caminho da Cancelinha, N32°49'22" W16°56'27", 469m, 05-Jul-2014, *Rubus*, beating, leg. Stüben,P., det. Stüben,P.
Auletobius maderensis<br>(Wollaston, 1854)	MK347579 [35]<br>3016-KRA<br>658nt	(SDEI-DNA-3016-KRA)	Portugal, Madeira, Caniçal, ca 1km NW, (Córrego Curralinho), N32°44'49.6" W16°45'00.48", 224m, 28-Mrz-2017, leg. Pelikan, det. Stüben,P.
Auletobius pubescens<br>(Kiesenwetter, 1851)	KR817560 [20]<br>1085-JKR<br>658nt	ZFMK-TIS-3421<br>ZFMK-DNA-0100449618	Spain, Andalucia, Rociana del Condado, N37°21'01" W06°36'55", 150m, 18-Apr-2012, *Cistus*, sweeping, leg. Krátky,J., det. Krátky,J.
Auletobius sp.	KR817562 [20]<br>1699-PST<br>658nt	ZFMK-TIS-24417<br>ZFMK-DNA-0155622480	Spain, Canary Islands, La Palma, N of Santa Cruz, M. de Tagoja, N28°43'18" W17°47'07", 1047m, 21-Dez-2013, *Cistus*, beating, leg. Stüben,P., det. Stüben,P.
Auleutes epilobii<br>(Paykull, 1800)	MK892043 [new]<br>2319-JKR<br>658nt	ZFMK-TIS-24250<br>ZFMK-DNA-0169170937	Slovakia, Tatry Mts., Dolina Siedmich prame?ov, N49°13'30" E20°16'24", 1404m, 05-Jul-2014, *Epilobium*, sweeping, leg. Krátky,J., det. Krátky,J.
Bagous bagdatensis<br>Pic, 1904	MK891265 [new]<br>1203-JKR<br>658nt	ZFMK-TIS-3539<br>ZFMK-DNA-0100426072	Czech Republic, Moravia, Breclav, Pohansko, N48°43'30" E16°53'44", 154m, 05-Aug-2012, individual collecting by hand, leg. Krátky,J., det. Krátky,J.
Bagous binodulus<br>(Herbst, 1795)	KC784230 [29]<br>398-RGO<br>615nt	ZFMK-TIS-20184<br>ZFMK-DNA-0100449441	Poland, ad. Urszulin , N51°22'16" E23°15'04" , 172m, 06-Jun-2011, leg. Gosik,R., det. Gosik,R.
Bagous elegans<br>(Fabricius, 1801)	KY849854 [29]<br>1441-JKR<br>658nt	ZFMK-TIS-4210<br>ZFMK-DNA-0155628492	Germany, Brandenburg (BB), Jessern, Schwielochsee, N52°01'59" E14°11'26", 44m, 05-Aug-2013, *Fragmites australis*, individual collecting by hand, leg. Krátky,J., det. Krátky,J.
Bagous exilis<br>Jacquelin du Val, 1855	KC784310 [29]<br>755-PST<br>658nt	ZFMK-TIS-3092<br>ZFMK-DNA-0100448133	Spain, Canary Islands, Tenerife, near San Juan de Rambla, Playa del Socorro, coast, N28°23'38" W16°36'11", 5m, 20-Feb-2012, *Limonium*, beating/sifting, leg. Stüben,P., det. Stüben,P.
Bagous longitarsis<br>C. G. Thomson, 1868	KC783917 [new]<br>370-JKR<br>632nt	ZFMK-TIS-20156<br>ZFMK-DNA-0100438713	Czech Republic, Bohemia or., Steblova, Oplatil pond, N50°6'15.584" E15°44'42.978", 230m, 29-Jun-2011, *Myriophyllum spicatum*, hand-collecting, leg. Krátky,J., det. Krátky,J.
Bagous lutulentus<br>(Gyllenhal, 1813)	KY849851 [29]<br>246-PSP<br>658nt	ZFMK-TIS-2D100439125<br>ZFMK-DNA-0100438530	Germany, Lower Saxony (NI), Essel, Lkr. Soltau-Fallingbostel, N52°41'57" E09°38'36", 25m, 10-Mai-2011, *Equisetum fluviatile*, sand field, kick sampling in mud, leg. Sprick,P., det. Sprick,P.

**Name**<br>**Authority**<br>**Additional Information**	**GenBank Acc No (Ref.)**<br>**Specimen ID**<br>**Sequence Length**	**ZFMK Tissue ID**<br>**ZFMK DNA Sample ID**<br>**(SDEI DNA Sample ID)**	**Locality, GPS, Collection Date, Plant, Collector, Identifier**
Bagous monanthiphagus<br>Stüben, 2010	KY849855 [29]<br>1666-PST<br>658nt	ZFMK-TIS-24384<br>ZFMK-DNA-0155622524	Spain, Canary Islands, La Gomera, Parque Natural de Majona, La Guerode – Enchereda, N28°07'52" W17°09'38", 689m, 05-Dez-2013, *Monantes*, beating, leg. Stüben,P., det. Stüben,P.
Bagous monanthiphagus<br>Stüben, 2010<br>**Paratype (DNAtype)**	ON421854 [new]<br>ES1046<br>658nt	ZFMK-TIS-cES1046<br>ZFMK-DNA-0112704623	Spain, Canary Islands, La Gomera, NW of San Sebastian, La Gerode, behind Casas de Cuevas Blancas ("Stangenpfad"), 28°07'46"N 17°08'48"W, 621m, 22-Jan-2010, *Monanthes laxiflora*, leg. Stüben,P., det. Stüben,P.
Bagous petro<br>(Herbst, 1795)	KY849856 [29]<br>2234-JKR<br>658nt	ZFMK-TIS-23972<br>ZFMK-DNA-0169170562	Czech Republic, Bohemia, Horni redice, Mordyr pond, N50°05'30" E15°57'03.5", 247m, 05-Sep-2014, sieving, leg. J. Pelikán, det. Pelikan,J.
Bagous robustus<br>H. Brisout de Barneville, 1863	KY849853 [29]<br>1253-FBA<br>639nt	ZFMK-TIS-3589<br>ZFMK-DNA-0100426026	Greece, Peloponnese, Lakonia, W of Gythio, Sminos river, N36°44'57" E22°31'01", 16m, 25-Mai-2011, leg. Bahr,F., det. Bayer,B.
Bagous subcarinatus<br>Gyllenhal, 1836	KY849852 [29]<br>1204-JKR<br>658nt	ZFMK-TIS-3540<br>ZFMK-DNA-0100426073	Czech Republic, Moravia, Breclav, Pohansko, N48°43'30" E16°53'44", 154m, 05-Aug-2012, *Cerophyllum demersum*, individual collecting by hand, leg. Krátky,J., det. Krátky,J.
Bagous subcarinatus<br>Gyllenhal, 1836	KC784000 [new]<br>252-PSP<br>658nt	ZFMK-TIS-2D100439119<br>ZFMK-DNA-0100439791	Germany, Lower Saxony (NI), Averhoy, Hannover region, N52°34'12" E09°32'30", 30m, 08-Mai-2011, *Ceratophyllum demersum*, standing water body, waterdipnet, leg. Sprick,P., det. Sprick,P.
Bagous subcarinatus<br>Gyllenhal, 1836	MK891390 [new]<br>1449-JKR<br>658nt	ZFMK-TIS-4218<br>ZFMK-DNA-0155628487	Slovakia, Nove Zamky, Male Kosihy, N47°55'58" E18°45'14", 108m, 28-Sep-2013, sieving, leg. Krátky,J., det. Krátky,J.
Bagous subcarinatus<br>Gyllenhal, 1836	MK891533 [new]<br>1621-JKR<br>658nt	ZFMK-TIS-3669<br>ZFMK-DNA-0155635619	Slovakia, Nove Zamky, Chlaba env., N47°49'28" E18°50'34", 108m, 18-Mai-2013, sweeping, leg. Krátky,J., det. Krátky,J.
Barioxyonyx retectus<br>(Peyerimhoff, 1925)	MK891183 [new]<br>1100-JKR<br>658nt	ZFMK-TIS-3436<br>ZFMK-DNA-0100449608	Spain, Andalucia, 3 km SW of Monda, N36°36'37" W04°51'04", 460m, 22-Apr-2012, *Ephedra alata*, beating, leg. Krátky,J., det. Krátky,J.
Barioxyonyx tournieri<br>(Fourcroi, 1891)	MK891493 [new]<br>1576-JKR<br>658nt	ZFMK-TIS-3624<br>ZFMK-DNA-0155635653	Morocco, Taroudant, N of Aoulouz, N30°41'47" W08°09'10", 708m, 16-Apr-2013, *Ephedra*, beating, leg. Krátky,J., det. Krátky,J.
Baris analis<br>(Olivier, 1791)	MK890972 [new]<br>861-PST<br>658nt	ZFMK-TIS-3774<br>ZFMK-DNA-0100414230	Greece, Zakynthos Isl., Skopos Mt. near Panagoula (W of Argasi) near abbey, N37°45'53" E20°54'44", 19m, 17-Okt-2012, wetlands, beating, leg. Stüben,P., det. Stüben,P.
Baris artemisiae<br>(Herbst, 1794)	MK891782 [new]<br>2041-JKR<br>658nt	ZFMK-TIS-23681<br>ZFMK-DNA-0169170463	Czech Republic, Bohemia, Kosatky, N50°19'23" E14°39'58", 218m, 02-Mai-2014, *Artemisia vulgaris*, sweeping, leg. Krátky,J., det. Krátky,J.
Baris artemisiae<br>(Herbst, 1794)	MK891112 [new]<br>1017-PSP<br>658nt	ZFMK-TIS-3162<br>ZFMK-DNA-0100426847	Germany, Lower Saxony (NI), Hannover, Nordhafen, N52°25'24.6" E09°38'53", 53m, 10-Mai-2012, *Artemisia vulgaris*, south exposed slope with annual ruderal area, beating, leg. Sprick,P., det. Sprick,P.
Baris artemisiae<br>(Herbst, 1794)	MK891033 [new]<br>930-CBR<br>658nt	ZFMK-TIS-3843<br>ZFMK-DNA-0100449681	Luxembourg, 12 km NE of Luxembourg, 1 km NW of Wecker, N49°41'53" E06°22'53", 215m, 07-Jun-2012, *Artemisia vulgaris*, beating, leg. Braunert,C., det. Braunert,C.
Baris artemisiae<br>(Herbst, 1794)	KC784177 [new]<br>402-RGO<br>658nt	ZFMK-TIS-20188<br>ZFMK-DNA-0100449101	Poland, ad. Urszulin , N51°22'16" E23°15'04" , 172m, 06-Jun-2011, leg. Gosik,R., det. Gosik,R.

**Name**<br>**Authority**<br>**Additional Information**	**GenBank Acc No (Ref.)**<br>**Specimen ID**<br>**Sequence Length**	**ZFMK Tissue ID**<br>**ZFMK DNA Sample ID**<br>**(SDEI DNA Sample ID)**	**Locality, GPS, Collection Date, Plant, Collector, Identifier**
Baris artemisiae<br>(Herbst, 1794)	KC784220 [new]<br>418-RGO<br>658nt	ZFMK-TIS-20204<br>ZFMK-DNA-0100449421	Poland, Tarnogóra, N50°41'11.6" E23°07'13.4", 249m, 11-Jun-2011, leg. Gosik,R., det. Gosik,R.
Baris artemisiae<br>(Herbst, 1794)	MK892260 [new]<br>2560-PSP<br>658nt	ZFMK-TIS-23534<br>ZFMK-DNA-0171661996	Slovakia, Nitra, Muzla, N47°48'00" E18°31'55", 114m, 19-Mai-2014, *Artemisia vulgaris*, leg. Sprick,P., det. Sprick,P.
Baris carbonaria<br>Boheman, 1836	MK891942 [new]<br>2210-JKR<br>658nt	ZFMK-TIS-23948<br>ZFMK-DNA-0169170586	Slovakia, Nove Zamky, Sturovo, Hegyfarok, N47°49'06" E18°38'38", 205m, 18-Mai-2014, *Crambe tataria*, collecting by hand, leg. Stejskal,R., det. Stejskal,R.
Baris memnonia<br>(Boheman, 1836)	MK892341 [new]<br>2680-PSP<br>658nt	ZFMK-TIS-23830<br>ZFMK-DNA-0171661319	Cyprus, Larnaca district, Larnaca, NW, N34°54'13" E33°36'00", 0m, 11-Apr-2013, *Suaeda maritima*, collecting by hand, leg. Sprick,P., det. Sprick,P.
Baromiamima concinna<br>(Boheman, 1834)	MK891357 [new]<br>1408-JKR<br>658nt	ZFMK-TIS-4177<br>ZFMK-DNA-0155628525	France, Cevennes Mts., 1 km N of Pommiers, N43°57'43" E03°36'44", 456m, 13-Jun-2013, *Castanea sativa*, sieving, leg. Krátky,J., det. Krátky,J.
Baromiamima concinna<br>(Boheman, 1834)	MK891552 [new]<br>1642-JKR<br>658nt	ZFMK-TIS-3690<br>ZFMK-DNA-0155635592	France, Alpes-Marritimes, Mandelieu la Napoule, N43°33'28" E06°54'54", 317m, 10-Jun-2013, *Pistacia*, sieving, leg. Krátky,J., det. Krátky,J.
Barretonus hinterseheri<br>Folwaczny, 1975	MH915694 [new]<br>2956-PST<br>658nt	(SDEI-DNA-2956-PST)	Portugal, Madeira, Machico, Ladeira env., , 18-Nov-2016, leg. Krasensky, det. Stüben,P.
Barretonus minor<br>Folwaczny, 1972	FJ716582 [01]<br>P-536-BAR<br>618nt	ZFMK-TIS-cP536<br>ZFMK-DNA-0100400628	Portugal, Madeira, Porto Santo Isl., 2.5 km N of Vila Baleira, Pico Castelo - Pico do Facho, N33°04'27" W16°19'25", 146m, 26-Mrz-2008, *Cynara cardunculus*, willow, leg. Astrin,J. & Stüben,P., det. Astrin,J. and Stüben,P.
Barretonus sp.	MH915693 [40]<br>2955-PST<br>658nt	(SDEI-DNA-2995-PST)	Portugal, Madeira, Santa Maria Madalena, Pombais, N32°51'32" W17°12'10", 400m, 20-Nov-2016, leg. Krasensky, det. Stüben,P.
Barretonus sp.	MH915695 [40]<br>2957-PST<br>658nt	(SDEI-DNA-2957-PST)	Portugal, Madeira, Ribeira da Janela, coast, , 16-Nov-2016, leg. Krasensky, det. Stüben,P.
Barynotus moerens<br>(Fabricius, 1792)	MK891524 [new]<br>1611-JKR<br>658nt	ZFMK-TIS-3659<br>ZFMK-DNA-0155633775	Czech Republic, Bohemia, Frydlant, PP Krizovy vrch, N50°54'48" E15°04'50", 322m, 08-Mai-2013, sieving, leg. Krátky,J., det. Krátky,J.
Barynotus moerens<br>(Fabricius, 1792)	MK890819 [new]<br>205-PSP<br>658nt	ZFMK-TIS-2D100438552<br>ZFMK-DNA-0100438507	Germany, Lower Saxony (NI), Königsförde, Lkr. Hameln-Pyrmont, N52°03'58" E09°16'17", 90m, 09-Apr-2011, *Primula* sp., house garden, collecting by hand, leg. Sprick,P., det. Sprick,P.
Barynotus moerens<br>(Fabricius, 1792)	MK892187 [new]<br>2482-PSP<br>658nt	ZFMK-TIS-23454<br>ZFMK-DNA-0171661791	Germany, Lower Saxony (NI), Hannover, Herrenhausen, Berggarten area, N52°23'40" E09°41'52.5", 54m, 09-Jan-2014, *Hosta lancifolia*, soil excavation, leg. Sprick,P., det. Sprick,P.
Barynotus obscurus<br>(Fabricius, 1775)	MK891976 [new]<br>2247-JKR<br>658nt	ZFMK-TIS-23985<br>ZFMK-DNA-0169170540	Czech Republic, Bohemia, Chudenin, N49°17'54" E13°06'00", 475m, 27-Sep-2014, leg. Benedikt,S., det. Benedikt,S.
Barynotus obscurus<br>(Fabricius, 1775)	MK890891 [new]<br>568-PSP<br>658nt	ZFMK-TIS-20353<br>ZFMK-DNA-0100448411	Germany, Lower Saxony (NI), Hannover, Herrenhausen, 3 km SW of "Berggarten" area, N52°23'41" E09°41'54", 54m, 21-Jun-2011, *Astilbe arendsii, Geranium ptilostemon*, next to river, dipnet at night, leg. Sprick,P., det. Sprick,P.

**Name**<br>**Authority**<br>**Additional Information**	**GenBank Acc No (Ref.)**<br>**Specimen ID**<br>**Sequence Length**	**ZFMK Tissue ID**<br>**ZFMK DNA Sample ID**<br>**(SDEI DNA Sample ID)**	**Locality, GPS, Collection Date, Plant, Collector, Identifier**
Barynotus obscurus<br>(Fabricius, 1775)	MK892290 [new]<br>2591-PSP<br>658nt	ZFMK-TIS-23565<br>ZFMK-DNA-0171661979	Germany, Bavaria (BY), Unterfranken, Ebern, N50°05'17" E10°45'53", 343m, 13-Jun-2014, *Primula veris*, leg. Sprick,P., det. Sprick,P.
Betulapion simile<br>(Kirby, 1811)	KC784145 [new]<br>631-PSP<br>658nt	ZFMK-TIS-20414<br>ZFMK-DNA-0100448637	Germany, Schleswig-Holstein (SH), Jardelund, Lkr. Nordfriesland, N54°49'07" E09°13'34", 29m, 26-Aug-2011, *Betula pendula*, bog edge shrub, beating, leg. Sprick,P., det. Sprick,P.
Betulapion simile<br>(Kirby, 1811)	MK891429 [new]<br>1492-PSP<br>658nt	ZFMK-TIS-3252<br>ZFMK-DNA-0155633330	Germany, Lower Saxony (NI), Harz National Park, Eckertal valley, Carici remotae-Fraxinetum, N51°53'13" E10°37'33", 380m, 01-Aug-2012, *Betula pendula*, beating, leg. Sprick,P., det. Sprick,P.
Betulapion simile<br>(Kirby, 1811)	MK891679 [new]<br>1895-PST<br>658nt	ZFMK-TIS-24000<br>ZFMK-DNA-0169168518	Germany, North Rhine-Westphalia (NRW), Elmpt, NSG "Elmpter Schwalmbruch", Venekotensee, N51°13'43" E06°06'59", 35m, 27-Aug-2014, *Betula*, beating, leg. Stüben,P., det. Stüben,P.
Betulapion simile<br>(Kirby, 1811)	MK891068 [new]<br>969-CBR<br>658nt	ZFMK-TIS-3882<br>ZFMK-DNA-0100449094	Luxembourg, 10 km NW of Esch/Alzette, 1 km NW of Niedercorn, N49°32'31" E05°52'39", 370m, 28-Jun-2012, *Betula pendula*, sweeping, leg. Braunert,C., det. Braunert,C.
Bothynoderes affinis<br>(Schrank, 1781)	MK891924 [new]<br>2192-JKR<br>658nt	ZFMK-TIS-23930<br>ZFMK-DNA-0169170595	Czech Republic, Moravia, Bozice, N48°49'21" E16°15'55", 220m, 02-Mai-2014, collecting by hand, leg. Stejskal,R., det. Stejskal,R.
Bothynoderes declivis<br>(Olivier, 1807)	MK892148 [new]<br>2435-JKR<br>658nt	ZFMK-TIS-26012<br>ZFMK-DNA-0171600650	Slovakia, Nove Zamky, Imel, PR Liscie diery, N47°55'17" E18°09'37", 114m, 20-Sep-2014, *Kochia laniflora*, collecting by hand, leg. Krátky,J., det. Krátky,J.
Brachiodontus alpinus<br>(Hampe, 1867)	MK891992 [new]<br>2263-JKR<br>658nt	ZFMK-TIS-24193<br>ZFMK-DNA-0169171003	Austria, Styria, Hochschwab, Spitzkögel, N47°35'27" E15°01'26", 1659m, 27-Jun-2014, *Primula clusiana*, collecting by hand, leg. Krátky,J., det. Krátky,J.
Brachiodontus reitteri<br>(Weise, 1878)	MK891273 [new]<br>1217-JKR<br>658nt	ZFMK-TIS-3553<br>ZFMK-DNA-0100426067	Romania, Maramures, Rodna Mts., Gargalau Mt., N47°35'15" E24°48'27", 1549m, 07-Sep-2012, *Soldanella carpatica*, sieving, leg. Krátky,J., det. Krátky,J.
Brachonyx pineti<br>(Paykull, 1792)	MK890837 [new]<br>296-JKR<br>658nt	ZFMK-TIS-20082<br>ZFMK-DNA-0100438331	Czech Republic, Bohemia or., Hradec Kralove, Plachta, N50°11'12.892" E15°51'37.148", 230m, 19-Apr-2011, *Pinus silvestris*, beating, leg. Krátky,J., det. Krátky,J.
Brachonyx pineti<br>(Paykull, 1792)	KC784027 [new]<br>219-PSP<br>658nt	ZFMK-TIS-2D100438540<br>ZFMK-DNA-0100439833	Germany, Lower Saxony (NI), Berkhof, Hannover region, N52°36'54" E09°43'49", 36m, 17-Apr-2011, *Pinus sylvestris*, former sand pit, beating, leg. Sprick,P., det. Sprick,P.
Brachycerus plicatus<br>Gyllenhal, 1833	MK892358 [40]<br>2748-PST<br>658nt	ZFMK-TIS-23898<br>ZFMK-DNA-0171661256	Spain, Canary Islands, Fuerteventura, 1 km SW of La Oliva, N28°36'32" W13°56'22", 213m, 19-Jan-2015, leg. Stüben,P., det. Stüben,P.
Brachyderes albicans<br>Desbrochers des Loges, 1896	KC783841 [new]<br>196-PST<br>611nt	ZFMK-TIS-2D100440961<br>ZFMK-DNA-0100438130	Morocco, S of Al-Hoceima, Rif Mts., S of Kassita, Tizi-Ouzli, N34°43'43" W03°49'33", 1169m, 27-Mai-2011, *Pinus*, beating, leg. Stüben,P., det. Behne,L.
Brachyderes illaesus<br>Boheman, 1833	KC784265 [new]<br>IT-0035w<br>635nt	ZFMK-TIS-2D100446520<br>ZFMK-DNA-0100437832	Italy, Sardinia Isl. East, E of Siniscola, La Caletta, coast, N40°35'30" E09°45'25", 3m, 09-Okt-2010, *Halimione (Chenopodiaceae)*, leg. Stüben,P., det. Stüben,P.
Brachyderes incanus<br>(Linnaeus, 1758)	KC784064 [new]<br>556-RST<br>658nt	ZFMK-TIS-20341<br>ZFMK-DNA-0100448418	Czech Republic, Moravia mer., Podyji NP, Kravi hora hill, 1 km SW of Znojmo, N48°50'56.89" E16°2'26.53", 320m, 10-Sep-2011, *Quercus*, beating, leg. Stejskal,R., det. Stejskal,R.

**Name**<br>**Authority**<br>**Additional Information**	**GenBank Acc No (Ref.)**<br>**Specimen ID**<br>**Sequence Length**	**ZFMK Tissue ID**<br>**ZFMK DNA Sample ID**<br>**(SDEI DNA Sample ID)**	**Locality, GPS, Collection Date, Plant, Collector, Identifier**
Brachyderes incanus<br>(Linnaeus, 1758)	KC784026 [new]<br>218-PSP<br>645nt	ZFMK-TIS-2D100438549<br>ZFMK-DNA-0100439832	Germany, Lower Saxony (NI), Berkhof, Hannover region, N52°36'54" E09°43'49", 36m, 17-Apr-2011, *Pinus sylvestris*, former sand pit, beating, leg. Sprick,P., det. Sprick,P.
Brachyderes incanus<br>(Linnaeus, 1758)	MK891298 [new]<br>1273-PST<br>658nt	ZFMK-TIS-4089<br>ZFMK-DNA-0100425382	Spain, Community of Madrid, NE of Montejo de la Sierra, Hayedo de Montejo, Puerto de El Cardoso, N41°05'14" W03°29'21", 1386m, 02-Mai-2013, *Quercus*, sieving, leg. Stüben,P. & Schütte,A., det. Sprick,P.
Brachyderes lusitanicus<br>(Fabricius, 1781)	MK890944 [new]<br>819-PST<br>658nt	ZFMK-TIS-3732<br>ZFMK-DNA-0100414265	Portugal, Minho, W of Ponte de Lima, Moreira do Lima, N41°47'05" W08°37'36", 41m, 06-Mai-2012, *Pinus*, beating, leg. Stüben,P., det. Stüben,P.
Brachyderes pubescens<br>(Boheman, 1833)	MK891584 [new]<br>488-PST<br>658nt	ZFMK-TIS-20273<br>ZFMK-DNA-0155630439	France, Vaucluse, Malaucene, Suzette, Dentelles de Montmirail, N44°10'47" E05°03'16", 542m, 02-Aug-2011, *Pinus*, beating, leg. Stüben,P., det. Stüben,P.
Brachyderes pubescens<br>(Boheman, 1833)	KC784274 [new]<br>IT-0048w<br>626nt	ZFMK-DNA-0100417641	Italy, Sardinia Isl. East, E of Seui, Ulassai, N39°48'35" E09°29'48", 745m, 29-Sep-2010, *Quercus ilex*, cliff, leg. Stüben,P., det. Stüben,P.
Brachyderes pubescens<br>(Boheman, 1833)	KC783859 [new]<br>157-PST<br>658nt	ZFMK-TIS-2D100438676<br>ZFMK-DNA-0100438158	Morocco, SW of Berkane near Taforalt, Beni Snassen Mts., N34°47'52" W02°25'31" , 839m, 15-Mai-2011, beating, leg. Stüben,P., det. Stüben,P./vid. Behne,L.
Brachyderes rugatus<br>Wollaston, 1864<br>formerly: *B. rugatus hierroensis*	MK347554 [40]<br>2968-PST<br>658nt	(SDEI-DNA-2968-PST)	Spain, Canary Islands, El Hierro, N El Pinar, N27°43'03" W17°58'55", 974m, 17-Apr-2016, *Pinus canariensis*, leg. Stüben & Schütte, det. Stüben,P.
Brachyderes rugatus<br>Wollaston, 1864<br>formerly: *B. rugatus hierroensis*	MK892402 [40]<br>2880-PST<br>658nt	ZFMK-TIS-25885<br>ZFMK-DNA-0169166907	Spain, Canary Islands, El Hierro, El Tomillar, N27°43'08" W18°05'32", 940m, 14-Apr-2016, *Pinus canariensis*, beating, leg. Stüben,P. & Schütte,A., det. Stüben,P.
Brachyderes rugatus<br>Wollaston, 1864<br>formerly: *B. rugatus calvus*	KC783758 [40]<br>47-PST<br>658nt	ZFMK-TIS-2D100447009<br>ZFMK-DNA-0100438081	Spain, Canary Islands, Gran Canaria, San Bartolome, N27°54'51" W15°34'26", 946m, 09-Jan-2011, *Pinus*, beating, leg. Stüben,P., det. Stüben,P.
Brachyderes rugatus<br>Wollaston, 1864<br>formerly: *B. rugatus rugatus*	MK891632 [40]<br>1757-PST<br>658nt	ZFMK-TIS-24475<br>ZFMK-DNA-0155622442	Spain, Canary Islands, La Palma, W of Tinizana, N28°44'31" W17°58'20", 793m, 24-Jan-2014, *Pinus canariensis*, beating, leg. Stüben,P., det. Stüben,P.
Brachyderes rugatus<br>Wollaston, 1864<br>formerly: *B. rugatus sculpturatus*	KC784313 [40]<br>759-PST<br>658nt	ZFMK-TIS-3096<br>ZFMK-DNA-0100448137	Spain, Canary Islands, Tenerife, N of Vilaflor, Lomo de Topo Negro, Casa Calinda, N28°10'22" W16°37'31", 1701m, 23-Feb-2012, *Pinus canariensis*, beating, leg. Stüben,P., det. Stüben,P.
Brachypera brevirostris<br>(Capiomont, 1868)	MK891314 [new]<br>1307-PST<br>658nt	ZFMK-TIS-4123<br>ZFMK-DNA-0100426176	Spain, Cordoba, W of Montoro, N38°00'21" W04°24'56", 170m, 04-Mai-2013, *roadside vegetation*, beating, leg. Stüben,P. & Schütte,A., det. Winkelmann,H.
Brachypera crinita<br>(Boheman, 1834)<br>formerly: *Donus crinitus*	MK891754 [new]<br>2009-JKR<br>658nt	ZFMK-TIS-23649<br>ZFMK-DNA-0169170492	Italy, Sicilia Isl. (TP), Trapani, Salinagrande, Isolotto, N37°57'14" E12°29'45", 3m, 17-Apr-2014, sweeping, leg. Krátky,J., det. Krátky,J.
Brachypera dauci<br>(Olivier, 1807)	MK890877 [40]<br>496-RST<br>658nt	ZFMK-TIS-20281<br>ZFMK-DNA-0100448483	Romania, Caras-Severin, Sfanta Elena env., N44°40'42.32" E21°43'13.01", 350m, 29-Apr-2011, *Erodium cicutarium*, hand-collecting, leg. Stejskal,R., det. Stejskal,R.

**Name**<br>**Authority**<br>**Additional Information**	**GenBank Acc No (Ref.)**<br>**Specimen ID**<br>**Sequence Length**	**ZFMK Tissue ID**<br>**ZFMK DNA Sample ID**<br>**(SDEI DNA Sample ID)**	**Locality, GPS, Collection Date, Plant, Collector, Identifier**
Brachypera dauci<br>(Olivier, 1807)	MK891947 [new]<br>2215-JKR<br>658nt	ZFMK-TIS-23953<br>ZFMK-DNA-0169170572	Slovakia, Komarno, Virt, N47°45'39" E18°20'13.5", 123m, 20-Sep-2014, *Erodium cicutarium*, leg. Benedikt,S., det. Benedikt,S.
Brachypera grandini<br>(Capiomont, 1868)	KC783847 [new]<br>176-PST<br>658nt	ZFMK-TIS-2D100440251<br>ZFMK-DNA-0100438139	Morocco, Atlas Mts., E of Ifrane, N33°27'26" W04°51'24", 1566m, 18-Mai-2011, beating, leg. Stüben,P., det. Winkelmann,H.
Brachypera lunata<br>(Wollaston, 1854)	MK890803 [40]<br>28-PST<br>658nt	ZFMK-TIS-2D100447019<br>ZFMK-DNA-0100437943	Spain, Canary Islands, Gran Canaria, S of Guia, Barranco del Pinar, field edge, N28°04'21" W15°37'38", 969m, 21-Dez-2010, beating, leg. Stüben,P., det. Stüben,P.
Brachypera lunata<br>(Wollaston, 1854)	MK892384 [40]<br>2844-PST<br>658nt	ZFMK-TIS-4239<br>ZFMK-DNA-FC17941518	Portugal, Madeira, Porto Santo Isl., Pico do Branco (Urze), N33°05'28" W16°18'17", 288m, 28-Nov-2015, *Cynara cardunculus*, sieving, leg. Stüben,P. & Schütte,A., det. Stüben,P.
Brachypera lunata<br>(Wollaston, 1854)	MK891312 [new]<br>1303-PST<br>658nt	ZFMK-TIS-4119<br>ZFMK-DNA-0100426172	Spain, Cordoba, Montoro, N38°01'49" W04°22'46", 161m, 04-Mai-2013, *roadside vegetation*, beating, leg. Stüben,P. & Schütte,A., det. Stüben,P.
Brachypera zoilus<br>(Scopoli, 1763)	MK890977 [new]<br>868-PST<br>658nt	ZFMK-TIS-3781<br>ZFMK-DNA-0100414218	Germany, Rhineland-Palatinate (RLP), Adenau, N50°24'43" E06°55'48", 259m, 24-Jul-2012, beating, leg. Stüben,P., det. Stüben,P.
Brachysomus echinatus<br>(Bonsdorff, 1785)	MK891785 [new]<br>2044-JKR<br>658nt	ZFMK-TIS-23684<br>ZFMK-DNA-0169170466	Czech Republic, Bohemia, Kosatky, N50°19'23" E14°39'58", 218m, 02-Mai-2014, sieving, leg. Krátky,J., det. Krátky,J.
Brachysomus echinatus<br>(Bonsdorff, 1785)	MK891101 [new]<br>1005-PSP<br>658nt	ZFMK-TIS-3150<br>ZFMK-DNA-0100426854	Germany, Lower Saxony (NI), near Hannover, Bredenbeck-Steinkrug, Deister, N52°14'01" E09°37'41", 182m, 05-Mai-2012, *Convallaria majalis*, broad-leaved forest, dipnet, leg. Sprick,P., det. Sprick,P.
Brachysomus echinatus<br>(Bonsdorff, 1785)	MK892173 [new]<br>2465-PSP<br>658nt	ZFMK-TIS-23437<br>ZFMK-DNA-0171661803	Germany, Saxony-Anhalt (ST), Harz National Park, Ilsenburg, Wienberg, N51°52'16" E10°39'57", 293m, 29-Mai-2013, *Fagus sylvatica*, beating, leg. Sprick,P., det. Sprick,P.
Brachysomus echinatus<br>(Bonsdorff, 1785)	MK891365 [new]<br>1416-JKR<br>658nt	ZFMK-TIS-4185<br>ZFMK-DNA-0155628517	Slovakia, Vysoke Tatry Mts., Skalne vrata, N49°13'39" E20°16'42", 1512m, 30-Jun-2013, sweeping, leg. Krátky,J., det. Krátky,J.
Brachysomus hirtus<br>(Boheman, 1845)	MK891636 [new]<br>1779-PST<br>658nt	ZFMK-TIS-24103<br>ZFMK-DNA-0171624085	Italy, Lazio, near Arpino, N41°38'53" E13°38'15", 732m, 08-Mai-2014, sieving, leg. Stüben,P. & Schütte,A., det. Stüben,P.
Brachysomus hirtus<br>(Boheman, 1845)	MK891980 [new]<br>2251-JKR<br>658nt	ZFMK-TIS-23989<br>ZFMK-DNA-0169170531	Slovakia, Zvolen, Hronska Dubrava, NPR Boky, N48°33'58" E19°01'10.5", 472m, 12-Okt-2014, sieving, leg. Krátky,J., det. Krátky,J.
Brachysomus setiger<br>(Gyllenhal, 1840)	MK891866 [new]<br>2130-JKR<br>658nt	ZFMK-TIS-23770<br>ZFMK-DNA-0169169592	Slovakia, Nove Zamky, Sturovo, Hegyfarok, N47°49'06" E18°38'38", 205m, 17-Mai-2014, *Crambe tataria*, sweeping, leg. Krátky,J., det. Krátky,J.
Brachysomus villosulus<br>(Germar, 1824)	KC783960 [new]<br>346-JKR<br>658nt	ZFMK-TIS-20132<br>ZFMK-DNA-0100438922	Czech Republic, Bohemia or., Uhretice env., N49°58'39.655" E15°51'28.603", 240m, 28-Mai-2011, sweeping, leg. Krátky,J., det. Krátky,J.
Brachysomus villosulus<br>(Germar, 1824)	MK891541 [new]<br>1630-JKR<br>658nt	ZFMK-TIS-3678<br>ZFMK-DNA-0155635599	Slovakia, Komarno, Palarikovo, Neded, Jahodnianske jazierko NR, N48°01'33" E17°59'49", 110m, 20-Mai-2013, sweeping, leg. Krátky,J., det. Krátky,J.

**Name**<br>**Authority**<br>**Additional Information**	**GenBank Acc No (Ref.)**<br>**Specimen ID**<br>**Sequence Length**	**ZFMK Tissue ID**<br>**ZFMK DNA Sample ID**<br>**(SDEI DNA Sample ID)**	**Locality, GPS, Collection Date, Plant, Collector, Identifier**
Bradybatus creutzeri<br>Germar, 1824	KC783964 [new]<br>316-JKR<br>658nt	ZFMK-TIS-20102<br>ZFMK-DNA-0100438927	Czech Republic, Bohemia or., Dvakacovice, N49°58'35.447" E15°54'2.243", 245m, 14-Mai-2011, *Acer*, beating, leg. Krátky,J., det. Krátky,J.
Bradybatus creutzeri<br>Germar, 1824	MK891634 [new]<br>1772-PST<br>658nt	ZFMK-TIS-24096<br>ZFMK-DNA-0171624092	Italy, Lazio, Alvito, S. Onófria, Val de Rio, N41°44'19" E13°43'41", 840m, 05-Mai-2014, *Carpinus*, sieving, leg. Stüben,P. & Schütte,A., det. Stüben,P.
Bradybatus elongatulus<br>(Boheman, 1843)	MK891349 [new]<br>1388-PST<br>658nt	ZFMK-TIS-4684<br>ZFMK-DNA-0155628545	Italy, Abruzzo, P.N. Majella, Roccaraso near Valico d. Forchetta, Mad. dell Altare, N41°56'19" E14°07'09", 1240m, 22-Aug-2013, *wet mixed forest*, sieving, leg. Stüben,P., det. Stüben,P.
Bradybatus elongatulus<br>(Boheman, 1843)	MK891637 [new]<br>1781-PST<br>658nt	ZFMK-TIS-24105<br>ZFMK-DNA-0171624072	Italy, Lazio, near Arpino, N41°38'53" E13°38'15", 732m, 08-Mai-2014, beating, leg. Stüben,P. & Schütte,A., det. Stüben,P.
Bradybatus kellneri<br>Bach, 1854	MK891823 [new]<br>2083-JKR<br>658nt	ZFMK-TIS-23723<br>ZFMK-DNA-0169169641	Czech Republic, Bohemia, Predhradi, N49°49'56" E16°02'33", 427m, 08-Mai-2014, sieving, leg. Krátky,J., det. Krátky,J.
Bradybatus kellneri<br>Bach, 1854	KC784005 [new]<br>235-PSP<br>658nt	ZFMK-TIS-2D100439129<br>ZFMK-DNA-0100439801	Germany, Hesse (HE), Wiesbaden-Erbenheim, N50°02'58" E08°18'08", 274m, 28-Apr-2011, *Acer platanoides*, waysidege shrub, beating, leg. Sprick,P., det. Sprick,P.
Bradybatus kellneri<br>Bach, 1854	MK892164 [new]<br>2455-PSP<br>658nt	ZFMK-TIS-23427<br>ZFMK-DNA-0171661818	Germany, Lower Saxony (NI), Hameln-Rohrsen, Dütberg, N52°06'28" E09°25'12", 98m, 10-Mai-2013, *Acer platanoides*, beating, leg. Sprick,P., det. Sprick,P.
Bruchela rufipes<br>(Olivier, 1790)	MK892264 [new]<br>2564-PSP<br>658nt	ZFMK-TIS-23538<br>ZFMK-DNA-0171662000	Slovakia, Nitra, Muzla, N47°48'00" E18°31'55", 114m, 19-Mai-2014, *Reseda lutea*, leg. Sprick,P., det. Sprick,P.
Bruchela schusteri<br>(Schilsky, 1912)	MK891376 [new]<br>1428-JKR<br>658nt	ZFMK-TIS-4197<br>ZFMK-DNA-0155628514	Czech Republic, Moravia, Pavlov, D?vin Mt., N48°52'28" E16°39'37", 401m, 06-Jul-2013, *Sysimbrium strictissimum*, beating, leg. Stanek,T., det. Krátky,J.
Bruchela schusteri<br>(Schilsky, 1912)	MK891875 [new]<br>2139-JKR<br>658nt	ZFMK-TIS-23779<br>ZFMK-DNA-0169169601	Slovakia, Komarno, Buc, N47°46'55.5" E18°25'10", 107m, 18-Mai-2014, *Erysimum* sp., sweeping, leg. Krátky,J., det. Krátky,J.
Bruchela schusteri<br>Schilsky, 1912	MK892233 [new]<br>2531-PSP<br>658nt	ZFMK-TIS-23503<br>ZFMK-DNA-0171661742	Slovakia, Nitra, SW of Buc, N47°46'56" E18°25'10", 110m, 18-Mai-2014, *Erysimum canum*, leg. Sprick,P., det. Sprick,P.
Bruchela suturalis<br>Schoenherr, 1826	MK892262 [new]<br>2562-PSP<br>658nt	ZFMK-TIS-23536<br>ZFMK-DNA-0171661998	Slovakia, Nitra, Muzla, N47°48'00" E18°31'55", 114m, 19-Mai-2014, *Reseda lutea*, leg. Sprick,P., det. Sprick,P.
Bruchela suturalis suturalis<br>(Fabricius, 1892)	MK891777 [new]<br>2036-JKR<br>658nt	ZFMK-TIS-23676<br>ZFMK-DNA-0169170458	Czech Republic, Bohemia, Kosatky, N50°19'23" E14°39'58", 218m, 02-Mai-2014, *Reseda lutea*, collecting by hand, leg. Krátky,J., det. Krátky,J.
Bryodaemon kocsirenae<br>Podlussány, 1998	MK891950 [new]<br>2218-JKR<br>658nt	ZFMK-TIS-23956<br>ZFMK-DNA-0169170569	Slovakia, Vranov nad Toplou, Slanske vrchy Mts., Petrovce, Oblik hill, N48°58'13" E21°28'15", 792m, 18-Jun-2015, leg. Benedikt,S., det. Benedikt,S.
Caenopsis fissirostris<br>(Walton, 1847)	MK891687 [40]<br>1903-PST<br>658nt	ZFMK-TIS-24008<br>ZFMK-DNA-0169170420	Germany, North Rhine-Westphalia (NRW), lower Rhine, Schwalmtal near Lüttelforst, N51°12'00" E06°15'37", 82m, 03-Sep-2014, mixed forest on sandy soil, sieving, leg. Stüben,P., det. Stüben,P.

**Name**<br>**Authority**<br>**Additional Information**	**GenBank Acc No (Ref.)**<br>**Specimen ID**<br>**Sequence Length**	**ZFMK Tissue ID**<br>**ZFMK DNA Sample ID**<br>**(SDEI DNA Sample ID)**	**Locality, GPS, Collection Date, Plant, Collector, Identifier**
Caenopsis fissirostris<br>(Walton, 1847)	MK890949 [new]<br>824-PST<br>658nt	ZFMK-TIS-3737<br>ZFMK-DNA-0100414270	Portugal, Minho, NW of Ponte de Lima, Vila Nova de Cerveira, Rio Coura, N41°52'27" W08°41'57", 123m, 07-Mai-2012, sieving, leg. Stüben,P., det. Stüben,P.
Caenopsis peyerimhoffi<br>Hustache, 1935	KC783849 [new]<br>174-PST<br>653nt	ZFMK-TIS-2D100440253<br>ZFMK-DNA-0100438141	Morocco, Atlas Mts., SW of Taza, Jbel Tazzeka near Bat-Bou-Idir, N34°03'43" W04°08'29", 1533m, 18-Mai-2011, *Quercus*, sieving, leg. Stüben,P., det. Behne,L.
Caenopsis waltoni<br>(Boheman, 1842)	MK890945 [40]<br>820-PST<br>658nt	ZFMK-TIS-3733<br>ZFMK-DNA-0100414266	Portugal, Minho, W of Ponte de Lima, Moreira do Lima, N41°47'05" W08°37'36", 41m, 06-Mai-2012, sieving, leg. Stüben,P., det. Stüben,P.
Calacalles affinis<br>Bahr, 2000	MG229813 [40]<br>2815-PST<br>498nt	ZFMK-TIS-25861<br>ZFMK-DNA-FC17941468	Spain, Canary Islands, Tenerife, Monte Anaga, 8 km east El Bailadero , N28°33'46" W16°08'55", 800m, 15-Jul-2010, collecting by hand, leg. Paladini,A., det. Stüben,P.
Calacalles affinis<br>Bahr, 2000	MG229814 [40]<br>2816-PST<br>658nt	ZFMK-TIS-25862<br>ZFMK-DNA-FC17941476	Spain, Canary Islands, Tenerife, Monte Anaga, 8 km east El Bailadero , N28°33'46" W16°08'55", 800m, 15-Jul-2010, collecting by hand, leg. Paladini,A., det. Stüben,P.
Calacalles atomarius<br>Bahr, 2000	FJ716561 [01]<br>E-576-ato<br>658nt	ZFMK-TIS-cE576<br>ZFMK-DNA-0100404967	Spain, Canary Islands, Tenerife, East Anaga Mts., E of La Laguna, E of Lomo de las Bodegas, N28°33'38" W16°09'20", 500m, 26-Dez-2003, *Sonchus spp., A. cuneatum*, cliff, leg. Stüben,P., det. Stüben,P.
Calacalles bandamaensis<br>Stüben, 2012	JN701880 [07]<br>7-PST<br>651nt	ZFMK-TIS-2D100446901<br>ZFMK-DNA-0100438112	Spain, Canary Islands, Gran Canaria, Marzagan, Caldera de Bandama, N28°02'07" W15°27'36", 401m, 23-Dez-2010, *Ficus carica*, beating, leg. Stüben,P., det. Stüben,P.
Calacalles bandamaensis<br>Stüben, 2013	JX306090 [07]<br>107-PST<br>607nt	ZFMK-TIS-2D100438641<br>ZFMK-DNA-0100438373	Spain, Canary Islands, Gran Canaria, Marzagan near Bandama, Lomo Mocanal, N28°02'06" W15°28'06", 537m, 28-Dez-2010, *Citrus*, beating, leg. Stüben,P., det. Stüben,P.
Calacalles bandamaensis<br>Stüben, 2013	JX306091 [07]<br>110-PST<br>607nt	ZFMK-TIS-2D100440317<br>ZFMK-DNA-0100438388	Spain, Canary Islands, Gran Canaria, NE of Santa Brigida, Angostura valley, N28°03'14" W15°28'49", 485m, 31-Dez-2010, *Rubus*, beating, leg. Stüben,P., det. Stüben,P.
Calacalles **cf**. *bandamaensis*	JN701884 [07]<br>20-PST<br>656nt	ZFMK-TIS-2D100447030<br>ZFMK-DNA-0100438102	Spain, Canary Islands, Gran Canaria, Moya, Barranco de los Tilos, N28°04'33" W15°36'05", 643m, 07-Dez-2010, *Castanea sativa*, beating, leg. Stüben,P., det. Stüben,P.
Calacalles **cf**. *bandamaensis*	GQ332342 [01]<br>E-821-set<br>658nt	ZFMK-TIS-cE821<br>ZFMK-DNA-0100405046	Spain, Canary Islands, Tenerife, Teno Mts., Erjos, W of Casa Forestal, N28°19'30" W16°48'32", 1030m, 05-Apr-2005, leg. Schoenfeld, det. Schoenfeld
Calacalles citvorator<br>Stüben, 2015<br>**Paratype (DNAtype)**	KT289382 [22]<br>1703-PST_24421<br>658nt	ZFMK-TIS-24421<br>ZFMK-DNA-0155622484	Spain, Canary Islands, La Palma, NW of Puntallana, El Corcho, N28°45'22" W17°45'34", 405m, 23-Dez-2013, *Citrus*, garden, beating, leg. Stüben,P., det. Stüben,P.
Calacalles citvorator<br>Stüben, 2015<br>**Paratype (DNAtype)**	KT289392 [22]<br>E-0266-set<br>658nt	ZFMK-TIS-cE0266<br>ZFMK-DNA-0100401043	Spain, Canary Islands, La Palma, 2.5 km W of La Galga, above Cubo de la Galga, N28°45'18" W17°46'37", 857m, 03-Jul-2006, *Laurisilva, Juglans*, leg. Stüben,P., det. Stüben,P.
Calacalles droueti azoricus<br>Stüben 2015<br>formerly: *C. azoricus*	EU286468 [01]<br>P-0125-azo<br>658nt	ZFMK-TIS-cP0125<br>ZFMK-DNA-0100400195	Portugal, Azores, Faial, "Res. Natural da Caldeira do Faial", N38°35'10" W28°42'04", 886m, 24-Aug-2003, *Tolpis azorica*, leg. Stüben,P., det. Stüben,P.
Calacalles droueti droueti<br>(Crotch, 1867)<br>formerly: *C. droueti*	GU987940 [01]<br>E-578-dro<br>627nt	ZFMK-TIS-cE578<br>ZFMK-DNA-0100404314	Portugal, Azores, Faial, Caldeira,"Res. Natural da Caldeira do Faial", N38°35'10" W28°42'04", 886m, 24-Aug-2003, *Euphorbia stygiana*, leg. Stüben,P., det. Stüben,P.

Suppl. material 1: Material Table
Schütte A, Stüben PE, Astrin JJ (2022): Molecular Weevil Identification Project: A Thoroughly Curated Barcode Release of 1300 Western Palearctic Weevil Species (Coleoptera: Curculionoidea) - *Biodiversity Data Journal* **10**

**Name**<br>**Authority**<br>**Additional Information**	**GenBank Acc No (Ref.)**<br>**Specimen ID**<br>**Sequence Length**	**ZFMK Tissue ID**<br>**ZFMK DNA Sample ID**<br>**(SDEI DNA Sample ID)**	**Locality, GPS, Collection Date, Plant, Collector, Identifier**
Calacalles droueti droueti<br>(Crotch, 1867)<br>formerly: *C. droueti*	EU286467 [01]<br>P-0123-dro<br>658nt	ZFMK-TIS-cP0123<br>ZFMK-DNA-0100400197	Portugal, Azores, Pico, Pico da Urze, N38°27'21" W28°21'04", 811m, 19-Aug-2003, *Euphorbia stygiana*, leg. Stüben,P., det. Stüben,P.
Calacalles droueti droueti<br>(Crotch, 1867)<br>formerly: *C. droueti*	MG229841 [new]<br>P-0264-dro<br>658nt	ZFMK-TIS-24624<br>ZFMK-DNA-0155629975	Portugal, Azores, Pico, near main road "EN3", N38°29'15" W28°20'47", 841m, 18-Aug-2003, *Euphorbia stygiana*, leg. Stüben,P., det. Stüben,P.
Calacalles exiguus<br>Bahr, 2000	GQ332337 [01]<br>E-817-exi<br>658nt	ZFMK-TIS-cE817<br>ZFMK-DNA-0100405052	Spain, Canary Islands, Tenerife, NE of La Laguna, Anaga Mts., forest Las Mercedes, N28°31'49" W16°17'12", 905m, 14-Okt-2008, *Laurus novocanariensis*, leg. Floren & Machado, det. Floren and Machado
Calacalles exiguus<br>Bahr, 2000	GU988051 [01]<br>E-818-exi<br>658nt	ZFMK-TIS-cE818<br>ZFMK-DNA-0100405056	Spain, Canary Islands, Tenerife, NE of La Laguna, Anaga Mts., forest Las Mercedes, N28°31'49" W16°17'12", 905m, 14-Okt-2008, *Ilex platyfolia*, leg. Floren & Machado, det. Floren and Machado
Calacalles exiguus<br>Bahr, 2000	GU988052 [01]<br>E-820-exi<br>658nt	ZFMK-TIS-cE820<br>ZFMK-DNA-0100404719	Spain, Canary Islands, Tenerife, NE of La Laguna, Anaga Mts., forest Las Mercedes, N28°31'49" W16°17'16", 889m, 16-Okt-2008, *Ilex platyfolia*, leg. Floren & Machado, det. Floren and Machado
Calacalles fuerteventurensis<br>Bahr, 2000	KT289390 [22]<br>2739-PST<br>658nt	ZFMK-TIS-23889<br>ZFMK-DNA-0171661260	Spain, Canary Islands, Fuerteventura, Cumbre de Jandia, Pico de Zarza , N28°06'06" W14°21'19", 802m, 12-Jan-2015, *Nauplius sericeus*, beating, leg. Stüben,P., det. Stüben,P.
Calacalles fuerteventurensis<br>Bahr, 2000	KT289391 [22]<br>2742-PST<br>654nt	ZFMK-TIS-23892<br>ZFMK-DNA-0171661250	Spain, Canary Islands, Fuerteventura, Cumbre de Jandia, Morro del Jorao (via Barranco de los Canarios), N28°07'03" W14°19'59", 625m, 17-Jan-2015, *Nauplius sericeus*, beating, leg. Stüben,P., det. Stüben,P.
Calacalles hermigua<br>Stüben & Astrin, 2009<br>**Paratype (DNAtype)**	GQ332331 [01]<br>E-742-set<br>658nt	ZFMK-TIS-cE742<br>ZFMK-DNA-0100405090	Spain, Canary Islands, La Gomera, SE of Hermigua near Casas del Palmar, N28°09'29" W17°09'37", 627m, 08-Okt-2008, thermophilous shrub forest, leg. Astrin,J. & Stüben,P., det. Stüben,P.
Calacalles hermigua<br>Stüben & Astrin, 2009<br>**Paratype (DNAtype)**	GQ332334 [01]<br>E-747-set<br>658nt	ZFMK-TIS-cE747<br>ZFMK-DNA-0100405086	Spain, Canary Islands, La Gomera, S of Hermigua, El Cedro, N28°08'18" W17°12'52", 791m, 09-Okt-2008, *Sonchus*, leg. Astrin,J., det. Stüben,P.
Calacalles hermigua<br>Stüben & Astrin, 2009	GU988038 [01]<br>E-746-set<br>658nt	ZFMK-TIS-cE746<br>ZFMK-DNA-0100405087	Spain, Canary Islands, La Gomera, S of Hermigua, El Cedro, Mirador El Bailadero, N28°07'22" W17°12'29", 994m, 09-Okt-2008, *Sonchus gomerensis, Sonchus radicata, Aeonium*, leg. Astrin,J. & Stüben,P., det. Stüben,P.
Calacalles hermigua<br>Stüben & Astrin, 2009	KT289394 [22]<br>ES1006<br>658nt	ZFMK-TIS-cES1006<br>ZFMK-DNA-0112704663	Spain, Canary Islands, La Gomera, El Cedro, Mirador de El Bailadero, N28°07'22" W17°12'29", 1045m, 05-Dez-2009, *Sonchus gomerensis*, leg. Stüben,P., det. Stüben,P.
Calacalles kabylianus<br>(Desbrochers des Loges, 1897)	GU988154 [01]<br>L-948-kab<br>658nt	ZFMK-TIS-cL948<br>ZFMK-DNA-0100405257	Libya, Cyrenaique, Ras el Hilal (battage), N32°52'00" E22°10'00", 29-Mrz-2009, leg. Pelletier,J., det. Pelletier,J.
Calacalles lepensis<br>Stüben, 2013	KF498965 [10]<br>1345-PST<br>658nt	ZFMK-TIS-4161<br>ZFMK-DNA-0100426275	Spain, Canary Islands, La Gomera, above Agulo, barrier lake, N28°11'04" W17°11'58", 496m, 15-Dez-2012, *Carlina salicifolia*, beating, leg. Stüben,P., det. Stüben,P.
Calacalles lepensis<br>Stüben, 2013	KF498966 [10]<br>ES1025<br>658nt	ZFMK-TIS-cES1025<br>ZFMK-DNA-0112704644	Spain, Canary Islands, La Gomera, Agulo, 28°11'23"N 17°11'56"W, 353m, 11-Jan-2010, *Carlina salicifolia*, steep face, leg. Stüben,P., det. Stüben,P.
Calacalles lepensis<br>Stüben, 2013	KJ867596 [22]<br>1258-PST<br>658nt	ZFMK-TIS-3594<br>ZFMK-DNA-0100426031	Spain, Canary Islands, La Gomera, above Agulo, barrier lake, N28°11'04" W17°11'58", 496m, 15-Dez-2012, *Aeonium spathulatum*, beating, leg. Stüben,P., det. Stüben,P.

**Name**<br>**Authority**<br>**Additional Information**	**GenBank Acc No (Ref.)**<br>**Specimen ID**<br>**Sequence Length**	**ZFMK Tissue ID**<br>**ZFMK DNA Sample ID**<br>**(SDEI DNA Sample ID)**	**Locality, GPS, Collection Date, Plant, Collector, Identifier**
Calacalles manriquei<br>Stüben, 2018	MF946563 [34]<br>2933-PST<br>658nt	(SDEI-DNA-2933-PST)	Spain, Canary Islands, Lanzarote, above Haria, Valle de Malpaso, N29°07'57" W13°31'03", 453m, 04-Jan-2017, *Carlina salicifolia*, beating, leg. Stüben,P., det. Stüben,P.
Calacalles minutus<br>Bahr, 2000	GU988050 [01]<br>E-822-min<br>658nt	ZFMK-TIS-cE822<br>ZFMK-DNA-0100405050	Spain, Canary Islands, Tenerife, Anaga Mts., 2 km E of La Cumbre, N28°32'22" W16°13'21", 750m, 21-Mrz-2005, leg. Schoenfeld, det. Schönfeld
Calacalles montelunatus<br>Stüben, 2015<br>**Paratype (DNAtype)**	KT289383 [22]<br>1716-PST_24434<br>658nt	ZFMK-TIS-24434<br>ZFMK-DNA-0155622474	Spain, Canary Islands, La Palma, Montes de Luna, N28°31'57" W17°48'37", 583m, 28-Dez-2013, *Bituminaria*, beating, leg. Stüben,P., det. Stüben,P.
Calacalles montelunatus<br>Stüben, 2015<br>**Paratype (DNAtype)**	KT289384 [22]<br>1720-PST_24438<br>658nt	ZFMK-TIS-24438<br>ZFMK-DNA-0155622470	Spain, Canary Islands, La Palma, Montes de Luna, N28°31'57" W17°48'37", 583m, 15-Jan-2014, *Ficus carica*, beating, leg. Stüben,P., det. Stüben,P.
Calacalles montelunatus<br>Stüben, 2015<br>**Paratype (DNAtype)**	KT289385 [22]<br>1721-PST_24439<br>658nt	ZFMK-TIS-24439<br>ZFMK-DNA-0155622469	Spain, Canary Islands, La Palma, Las Caletas, N28°29'36" W17°49'59", 539m, 28-Dez-2013, *Retama raetam*, beating, leg. Stüben,P., det. Stüben,P.
Calacalles mulagua<br>Stüben, 2010	KT289389 [22]<br>2719-PST<br>658nt	ZFMK-TIS-23869<br>ZFMK-DNA-0171661275	Spain, Canary Islands, La Gomera, Las Rosas, N28°09'59" W17°14'53", 613m, 31-Dez-2014, *Castanea sativa*, beating, leg. Stüben,P., det. Stüben,P.
Calacalles mulagua<br>Stüben, 2010<br>**Paratype (DNAtype)**	KT289393 [22]<br>ES1002<br>658nt	ZFMK-TIS-cES1002<br>ZFMK-DNA-0112704667	Spain, Canary Islands, La Gomera, E of Hermigua, El Palmar, 28°09'26"N 17°09'47"W, 627m, 03-Dez-2009, *Descuraina millefolia*, thermophilic brushwood, leg. Stüben,P., det. Stüben,P.
Calacalles mulagua<br>Stüben, 2010	KT289395 [22]<br>ES1026<br>658nt	ZFMK-TIS-cES1026<br>ZFMK-DNA-0112704643	Spain, Canary Islands, La Gomera, La Palmita, 28°10'58"N 17°12'05"W, 591m, 13-Jan-2010, *Castanea sativa*, leg. Stüben,P., det. Stüben,P.
Calacalles mulagua<br>Stüben, 2010<br>**Paratype (DNAtype)**	KT289397 [22]<br>ES1030<br>658nt	ZFMK-TIS-cES1030<br>ZFMK-DNA-0112704639	Spain, Canary Islands, La Gomera, Las Rosas, El Tion, 28°9'59"N 17°14'47"W, 637m, 21-Jan-2010, *Castanea sativa*, near creek, sieving, leg. Stüben,P., det. Stüben,P.
Calacalles mulagua<br>Stüben, 2010<br>**Paratype (DNAtype)**	KT289398 [22]<br>ES1034<br>658nt	ZFMK-TIS-cES1034<br>ZFMK-DNA-0112704624	Spain, Canary Islands, La Gomera, S of Vallehermoso, Los Loros, 28°8'57"N 17°16'36"W, 609m, 11-Feb-2010, *Castanea sativa*, gardens at river, connected to Laurisilva, leg. Stüben,P., det. Stüben,P.
Calacalles mulagua<br>Stüben, 2010<br>**Paratype (DNAtype)**	KT289399 [22]<br>ES1036<br>658nt	ZFMK-TIS-cES1036<br>ZFMK-DNA-0112704626	Spain, Canary Islands, La Gomera, La Palmita, 28°10'45"N 17°12'39"W, 736m, 25-Feb-2010, *Ficus carica*, leg. Stüben,P., det. Stüben,P.
Calacalles mulagua<br>Stüben, 2010<br>**Paratype (DNAtype)**	KT289400 [22]<br>ES1037<br>658nt	ZFMK-TIS-cES1037<br>ZFMK-DNA-0112704627	Spain, Canary Islands, La Gomera, La Palmita, 28°10'18"N 17°12'53"W, 672m, 25-Feb-2010, *Castanea sativa*, leg. Stüben,P., det. Stüben,P.
Calacalles mulagua<br>Stüben, 2010<br>**Paratype (DNAtype)**	KT289401 [22]<br>ES1039<br>658nt	ZFMK-TIS-cES1039<br>ZFMK-DNA-0112704629	Spain, Canary Islands, La Gomera, S of Vallehermoso (behind El Tion), 28°10'17"N 17°15'19"W, 632m, 26-Feb-2010, *Ficus carica, herbaceous vegetation*, leg. Stüben,P., det. Stüben,P.
Calacalles nataliae<br>Astrin & Stüben, 2009<br>**Paratype (DNAtype)**	GQ332332 [01]<br>E-743-nat<br>658nt	ZFMK-TIS-cE743	Spain, Canary Islands, La Gomera, SE of Hermigua near Casas del Palmar, N28°09'29" W17°09'37", 627m, 08-Okt-2008, thermophilous shrub forest, leg. Astrin,J. & Stüben,P., det. Stüben,P.
Calacalles nataliae<br>Astrin & Stüben, 2009<br>**Paratype (DNAtype)**	GQ332333 [01]<br>E-745-nat<br>658nt	ZFMK-TIS-cE745<br>ZFMK-DNA-0100405088	Spain, Canary Islands, La Gomera, SE of Hermigua near Casas del Palmar, N28°09'29" W17°09'37", 627m, 08-Okt-2008, thermophilous shrub forest, leg. Astrin,J. & Stüben,P., det. Stüben,P.

**Name**<br>**Authority**<br>**Additional Information**	**GenBank Acc No (Ref.)**<br>**Specimen ID**<br>**Sequence Length**	**ZFMK Tissue ID**<br>**ZFMK DNA Sample ID**<br>**(SDEI DNA Sample ID)**	**Locality, GPS, Collection Date, Plant, Collector, Identifier**
Calacalles nataliae<br>Astrin & Stüben, 2009	GU988037 [01]<br>E-744-nat<br>658nt	ZFMK-TIS-cE744<br>ZFMK-DNA-0100405089	Spain, Canary Islands, La Gomera, SE of Hermigua near Casas del Palmar, N28°09'29" W17°09'37", 627m, 08-Okt-2008, thermophilous shrub forest, leg. Astrin,J. & Stüben,P., det. Stüben,P.
Calacalles nataliae<br>Astrin & Stüben, 2009	KF498967 [10]<br>1259-PST<br>658nt	ZFMK-TIS-3595<br>ZFMK-DNA-0100426242	Spain, Canary Islands, La Gomera, NW of San Sebastian, La Gerode to Casas de Cuevas Blancas ("Stangenpfad"), N28°07'44" W17°8'54", 626m, 24-Dez-2012, *Aeonium spathulatum*, beating, leg. Stüben,P., det. Stüben,P.
Calacalles nataliae<br>Astrin & Stüben, 2009	KF498968 [10]<br>ES1012<br>658nt	ZFMK-TIS-cES1012<br>ZFMK-DNA-0112704650	Spain, Canary Islands, La Gomera, N of San Sebastian, Encherada, N28°07'44" W17°09'18", 608m, 10-Dez-2009, *Descuraina millefolia*, leg. Stüben,P., det. Stüben,P.
Calacalles palmensis<br>(Roudier, 1954)	EU286466 [01]<br>C-0121-set<br>658nt	ZFMK-TIS-cC0121<br>ZFMK-DNA-0100400690	Spain, Canary Islands, La Palma, 11 km NE of El Paso, Mt. Tagoja, N28°43'18" W17°47'07", 1047m, 01-Jul-2006, *Laurisilva, Chamaecytisus proliferus*, leg. Stüben,P., det. Stüben,P.
Calacalles palmensis<br>(Roudier, 1954)	KT289381 [22]<br>1700-PST<br>658nt	ZFMK-TIS-24418<br>ZFMK-DNA-0155622481	Spain, Canary Islands, La Palma, N of Santa Cruz, M. de Tagoja, N28°43'18" W17°47'07", 1047m, 21-Dez-2013, *Laurisilva*, beating, leg. Stüben,P., det. Stüben,P.
Calacalles palmensis<br>(Roudier, 1954)	KT289386 [22]<br>1739-PST<br>658nt	ZFMK-TIS-24457<br>ZFMK-DNA-0155622451	Spain, Canary Islands, La Palma, SE of Garafia, La Zarza, N28°48'30" W17°54'26", 961m, 08-Jan-2014, *Castanea sativa*, beating, leg. Stüben,P., det. Stüben,P.
Calacalles pumilio<br>Bahr, 2000	GQ332336 [01]<br>E-811-pum<br>658nt	ZFMK-TIS-cE811<br>ZFMK-DNA-0100405051	Spain, Canary Islands, La Gomera, S of Hermigua, El Cedro, Las Mimbreras, N28°07'27" W17°13'26", 901m, 05-Okt-2008, *Myrica faya*, leg. Astrin,J.,Stüben,P.,Behne,L.,Floren,L., det. Stüben,P.
Calacalles pumilio<br>Bahr, 2000	KT289388 [22]<br>2713-PST<br>658nt	ZFMK-TIS-23863<br>ZFMK-DNA-0171661286	Spain, Canary Islands, La Gomera, El Cedro, N28°07'59" W17°13'03", 841m, 24-Dez-2014, *Laurisilva*, beating, leg. Stüben,P., det. Stüben,P.
Calacalles pumilio<br>Bahr, 2000<br>formerly: *C. agana* - Syn!	KT289396 [22]<br>ES1029<br>658nt	ZFMK-TIS-cES1029<br>ZFMK-DNA-0112704640	Spain, Canary Islands, La Gomera, Las Rosas, El Tion, 28°9'59"N 17°14'47"W, 637m, 21-Jan-2010, *Castanea sativa*, near creek, sieving, leg. Stüben,P., det. Stüben,P.
Calacalles pumilio<br>Bahr, 2000	GQ332339 [01]<br>E-812-pum<br>658nt	ZFMK-TIS-cE812<br>ZFMK-DNA-0100405054	Spain, Canary Islands, Tenerife, SW of Los Silos, Teno Mts., Monte del Agua, Chupadero, N28°19'23" W16°49'15", 942m, 02-Okt-2008, *Picconia excelsa, Laurus novocanariensis*, leg. Astrin,J.,Stüben,P.,Behne,L.,Floren,L., det. Stüben,P.
Calacalles pusillus<br>Bahr, 2000	FJ716570 [01]<br>E-702-pus<br>658nt	ZFMK-TIS-cE702<br>ZFMK-DNA-0100404853	Spain, Canary Islands, Tenerife, E of Los Silos, Teno Mts., El Tanque Bajo, N28°21'39" W16°46'19", 534m, 03-Okt-2008, compost, leg. Astrin,J. & Stüben,P., det. Stüben,P.
Calacalles pusillus<br>Bahr, 2000	KC783827 [22]<br>709-PST<br>658nt	ZFMK-TIS-3046<br>ZFMK-DNA-0100448183	Spain, Canary Islands, Tenerife, Teno Mts., Erjos, N28°19'45" W16°48'21", 1003m, 07-Jan-2012, *Ficus carica*, beating, leg. Stüben,P., det. Stüben,P.
Calacalles pusillus<br>Bahr, 2000	KC783829 [22]<br>722-PST<br>658nt	ZFMK-TIS-3059<br>ZFMK-DNA-0100448165	Spain, Canary Islands, Tenerife, Anaga Mts., Las Carboneras, N28°33'20" W16°16'43", 628m, 19-Jan-2012, *Foeniculum vulgare*, beating, leg. Stüben,P., det. Stüben,P.
Calacalles pusillus<br>Bahr, 2000	KC783831 [22]<br>738-PST<br>658nt	ZFMK-TIS-3075<br>ZFMK-DNA-0100448149	Spain, Canary Islands, Tenerife, Teno Mts., Erjos – El Palmar, N28°19'43" W16°49'19", 903m, 02-Feb-2012, *Ficus carica*, beating, leg. Stüben,P. & Schütte,A., det. Stüben,P.
Calacalles seticollis<br>(Wollaston, 1864)	GQ332328 [01]<br>C-0122-set<br>658nt	ZFMK-TIS-cC0122<br>ZFMK-DNA-0100400198	Spain, Canary Islands, El Hierro, 4 km SW of La Frontera, El Parque, N27°44'20" W18°01'30", 739m, 24-Dez-2006, *Sonchus hierrensis*, leg. Stüben,P., det. Stüben,P.

**Name**<br>**Authority**<br>**Additional Information**	**GenBank Acc No (Ref.)**<br>**Specimen ID**<br>**Sequence Length**	**ZFMK Tissue ID**<br>**ZFMK DNA Sample ID**<br>**(SDEI DNA Sample ID)**	**Locality, GPS, Collection Date, Plant, Collector, Identifier**
Calacalles seticollis<br>(Wollaston, 1864)	GU987860 [01]<br>E-0265-set<br>658nt	ZFMK-TIS-cE0265<br>ZFMK-DNA-0100401042	Spain, Canary Islands, El Hierro, 1 km SW of Guarazoca, Camino de la Pena, N27°48'06" W17°58'47", 746m, 02-Jan-2007, *Carlina salicifolia*, leg. Stüben,P., det. Stüben,P.
Calacalles theryi<br>(Peyerimhoff, 1925)	GQ332341 [01]<br>E-914-the<br>658nt	ZFMK-TIS-cE914<br>ZFMK-DNA-0100405057	Morocco, 2 km S Essaouira, S of Diabat, N31°28'08" W09°45'27", 22m, 12-Mai-2009, *Senecio anteuphorbium*, leg. Stüben,P., det. Stüben,P.
Calacalles theryi<br>(Peyerimhoff, 1925)	GQ332338 [01]<br>E-865-the<br>658nt	ZFMK-TIS-cE865<br>ZFMK-DNA-0100405053	Portugal, N Caldas da Rainha, SW of Nazare, São Martinho do Porto, N39°30'24" W09°08'48", 3m, 28-Apr-2009, *Crithmum maritimum (ger. "Meeresfenchel")*, leg. Astrin,J., det. Stüben,P.
Calacalles theryi<br>(Peyerimhoff, 1925)	KJ867594 [22]<br>1248-PST<br>658nt	ZFMK-TIS-3584<br>ZFMK-DNA-0100426036	Spain, Cadiz, La Linea, Sierra Carbonera, Zona Cortijo 4 vientos , N36°10'54" W05°21'23", 41m, 00-Jan-1900, *Calicotome villosa*, beating, leg. Torres,J. L., det. Stüben,P.
Calacalles wollastoni<br>(Chevrolat, 1852)	FJ716557 [01]<br>P-520-wol<br>658nt	ZFMK-TIS-cP520<br>ZFMK-DNA-0100400614	Portugal, Madeira, 3 km W of Porto Moniz, Santa Madalena, N32°51'31" W17°12'11", 391m, 19-Mrz-2008, *Ficus carica*, leg. Astrin,J. & Stüben,P., det. Astrin,J. and Stüben,P.
Calacalles wollastoni<br>(Chevrolat, 1852)	GQ332329 [01]<br>P-507-wol<br>658nt	ZFMK-TIS-cP507<br>ZFMK-DNA-0100400622	Portugal, Madeira, Santana, N32°48'12" W16°52'53", 457m, 15-Mrz-2008, *Castanea sativa*, leg. Stüben,P., det. Stüben,P.
Calacalles wollastoni<br>(Chevrolat, 1852)	KT289387 [22]<br>1841-PST<br>658nt	ZFMK-TIS-26044<br>ZFMK-DNA-0171606126	Portugal, Madeira, W of Porto Moniz, Santa Madalena, Rib. do Tristao, N32°51'20" W17°12'21", 154m, 29-Jun-2014, *Ficus*, beating, leg. Stüben,P., det. Stüben,P.
Calacalles wollastoni<br>(Chevrolat, 1852)	MG229816 [40]<br>2832-PST<br>658nt	ZFMK-TIS-4742<br>ZFMK-DNA-FC17941517	Portugal, Madeira, N of Santana, Quebrada, N32°48'49" W16°52'27", 124m, 25-Nov-2015, beating, leg. Stüben,P. & Schütte,A., det. Stüben,P.
Calacalles wollastoni<br>(Chevrolat, 1852)	MG229817 [40]<br>2845-PST<br>658nt	ZFMK-TIS-4240<br>ZFMK-DNA-FC17941526	Portugal, Madeira, Porto Santo Isl., Pico do Branco (Urze), N33°05'28" W16°18'17", 288m, 28-Nov-2015, *Ficus carica*, sieving, leg. Stüben,P. & Schütte,A., det. Stüben,P.
Calosirus cf. *terminatus*	MK891766 [new]<br>2023-JKR<br>658nt	ZFMK-TIS-23663<br>ZFMK-DNA-0169170478	Italy, Sicilia Isl. (PA), Palermo, Bosco di Ficuzza, N37°51'50" E13°24'56", 995m, 22-Apr-2014, *Ferula*, sweeping, leg. Krátky,J., det. Krátky,J.
Calosirus terminatus<br>(Herbst, 1795)	MK892107 [new]<br>2384-JKR<br>658nt	ZFMK-TIS-25961<br>ZFMK-DNA-0171600605	Czech Republic, Moravia, Hnanice, N48°47'59" E15°58'15", 310m, 10-Sep-2014, sweeping, leg. R. Stejskal, det. Stejskal,R.
Canariacalles alluaudi<br>(Uyttenboogaart, 1940)	JN701887 [12]<br>31-PST<br>658nt	ZFMK-TIS-2D100447016<br>ZFMK-DNA-0100438088	Spain, Canary Islands, Gran Canaria, Las Lagunetas, La Siberia, N27°59'56" W15°35'13", 1252m, 26-Dez-2010, *Ferula linkii*, beating, leg. Stüben,P., det. Stüben,P.
Canariacalles alluaudi<br>(Uyttenboogaart, 1940)	MG229683 [40]<br>9-PST<br>658nt	ZFMK-TIS-2D100447038<br>ZFMK-DNA-0155630448	Spain, Canary Islands, Gran Canaria, Santa Lucia, below Cruz las Vueltas, Mirador de las Tederas, N27°54'47" W15°31'48", 818m, 03-Dez-2010, *Ferula linkii*, collecting by hand, leg. Stüben,P., det. Stüben,P.
Canariacalles lanzarotensis<br>(Stüben, 2000)	MG229800 [40]<br>2762-PST<br>658nt	ZFMK-TIS-26211<br>ZFMK-DNA-0171661905	Spain, Canary Islands, Lanzarote, Jameos del Aqua, N29°9'16.5" W13°25'49", 4m, 07-Mai-2014, leg. Brustel, det. Stüben,P.
Canariacalles lanzarotensis<br>(Stüben, 2000)	MG229885 [40]<br>2932-PST<br>658nt	(SDEI-DNA-2932-PST)	Spain, Canary Islands, Lanzarote, above Haria, Valle de Malpaso, N29°07'57" W13°31'03", 453m, 04-Jan-2017, *Ferula lancerottensis*, beating, leg. Stüben,P., det. Stüben,P.

**Name**<br>**Authority**<br>**Additional Information**	**GenBank Acc No (Ref.)**<br>**Specimen ID**<br>**Sequence Length**	**ZFMK Tissue ID**<br>**ZFMK DNA Sample ID**<br>**(SDEI DNA Sample ID)**	**Locality, GPS, Collection Date, Plant, Collector, Identifier**
Canariacalles sp.	FJ716572 [01]<br>E-709-all<br>658nt	ZFMK-TIS-cE709<br>ZFMK-DNA-0100404846	Spain, Canary Islands, Tenerife, W of Los Silos, Teno Mts., Casa Blanca near Buenavista del Norte, Levada, N28°21'35" W16°52'05", 230m, 03-Okt-2008, *Foeniculum vulgare*, leg. Astrin,J. & Stüben,P., det. Stüben,P.
Canariacalles sp.	GQ332330 [01]<br>E-695-all<br>658nt	ZFMK-TIS-cE695<br>ZFMK-DNA-0100404861	Spain, Canary Islands, Tenerife, W of Los Silos, Teno Mts., Casa Blanca near Buenavista del Norte, N28°21'36" W16°52'10", 231m, 02-Okt-2008, *Ceropegia dichotoma*, leg. Astrin,J. & Stüben,P., det. Stüben,P.
Canariacalles sp.	GU987838 [01]<br>E-0220-all<br>658nt	ZFMK-TIS-cE0220<br>ZFMK-DNA-0100400295	Spain, Canary Islands, Tenerife, 6 km W of Los Silos, Casa Blanca, N28°21'40" W16°52'15", 120m, 21-Dez-2003, *Ceropegia dichotoma*, succulent bush, leg. Stüben,P., det. Stüben,P.
Canariacalles sp.	GU988008 [01]<br>E-700-all<br>658nt	ZFMK-TIS-cE700<br>ZFMK-DNA-0100404324	Spain, Canary Islands, Tenerife, E of Los Silos, Teno Mts., El Tanque Bajo, N28°21'39" W16°46'19", 534m, 03-Okt-2008, compost, leg. Astrin,J. & Stüben,P., det. Stüben,P.
Catapion meieri<br>(Desbrochers des Loges, 1901)<br>Synonym of *C. seniculus*?	KC784041 [new]<br>617-PSP<br>658nt	ZFMK-TIS-20400<br>ZFMK-DNA-0100448248	Germany, Lower Saxony (NI), Braunschweig, Rautheim, Wabeniederung, N52°15'22" E10°35'17", 74m, 20-Jul-2011, *Trifolium hybridum*, beating, leg. Sprick,P., det. Sprick,P.
Catapion meieri<br>(Desbrochers des Loges, 1901)<br>Synonym of *C. seniculus*?	MK892295 [new]<br>2596-PSP<br>658nt	ZFMK-TIS-23570<br>ZFMK-DNA-0171661963	Germany, Bavaria (BY), Unterfranken, Ebern, N50°05'18.5" E10°46'02", 328m, 13-Jun-2014, *Trifolium hybridum*, leg. Sprick,P., det. Sprick,P.
Catapion pubescens<br>(Kirby, 1811)	MK892087 [40]<br>2363-JKR<br>658nt	ZFMK-TIS-25923<br>ZFMK-DNA-0171600578	Czech Republic, Moravia, NP Podyji, Cizov, Siroke pole, N48°51'36" E15°51'02", 300m, 17-Aug-2014, sweeping, leg. R. Stejskal, det. Stejskal,R.
Catapion seniculus<br>(Kirby, 1808)	MK891443 [new]<br>1506-PSP<br>658nt	ZFMK-TIS-3266<br>ZFMK-DNA-0155633323	Denmark, Syddanmark, Emmerlev Klev (Højer), N54°59'16" E08°39'34", 3m, 30-Aug-2012, *Trifolium pratense*, meadow near the coast, dipnet, leg. Sprick,P., det. Sprick,P.
Catapion seriatosetosulum<br>(Wencker, 1864)	KC783890 [new]<br>124-PST<br>658nt	ZFMK-TIS-2D100440314<br>ZFMK-DNA-0100438353	Morocco, S of Berkane, Beni Snassen Mts., Vallee Zegzel, N34°49'26" W02°22'59", 634m, 12-Mai-2011, beating, leg. Stüben,P., det. Behne,L.
Cathormiocerus aristatus<br>(Gyllenhal, 1827)	MK891502 [new]<br>1588-JKR<br>658nt	ZFMK-TIS-3636<br>ZFMK-DNA-0155635634	Czech Republic, Moravia, Cejc - Spidlaky, N48°55'47" E16°58'03", 184m, 01-Mai-2013, sieving, leg. Krátky,J., det. Krátky,J.
Cathormiocerus aristatus<br>(Gyllenhal, 1827)	MK891787 [new]<br>2046-JKR<br>658nt	ZFMK-TIS-23686<br>ZFMK-DNA-0169170455	Czech Republic, Bohemia, Kosatky, N50°19'23" E14°39'58", 218m, 02-Mai-2014, sieving, leg. Krátky,J., det. Krátky,J.
Cathormiocerus aristatus<br>(Gyllenhal, 1827)	KC784045 [new]<br>624-PSP<br>658nt	ZFMK-TIS-20407<br>ZFMK-DNA-0100448281	Germany, Lower Saxony (NI), Königsförde, Lkr.Hameln-Pyrmont, N52°03'58" E09°16'17", 92m, 07-Aug-2011, *Achillea millefolium u.a.*, house garden, gras sifting, leg. Sprick,P., det. Sprick,P.
Cathormiocerus atlanticus<br>Escalera, 1918	MK891501 [new]<br>1587-JKR<br>658nt	ZFMK-TIS-3635<br>ZFMK-DNA-0155635633	Morocco, Haut Atlas, Oukaimeden, N31°12'10" W07°52'13", 2691m, 20-Apr-2013, *Alyssum spinosum*, sieving, leg. Krátky,J., det. Borovec,R.
Cathormiocerus curvipes<br>(Wollaston, 1854)	MK891713 [40]<br>1939-PST<br>658nt	ZFMK-TIS-24018<br>ZFMK-DNA-0169170417	Portugal, Madeira, Faial, coast, N32°47'37" W16°50'57", 26m, 04-Okt-2014, sieving, leg. Stüben,P., det. Stüben,P.

**Name**<br>**Authority**<br>**Additional Information**	**GenBank Acc No (Ref.)**<br>**Specimen ID**<br>**Sequence Length**	**ZFMK Tissue ID**<br>**ZFMK DNA Sample ID**<br>**(SDEI DNA Sample ID)**	**Locality, GPS, Collection Date, Plant, Collector, Identifier**
Cathormiocerus curvipes<br>(Wollaston, 1854)	MK892371 [40]<br>2820-PST<br>658nt	ZFMK-TIS-25866<br>ZFMK-DNA-FC17941508	Portugal, Madeira, below Pico Ruivo, N32°45'31" W16°56'34", 1702m, 22-Nov-2015, *Ulex europaeus, Cytisus*, sieving, leg. Stüben,P. & Schütte,A., det. Stüben,P.
Cathormiocerus discors<br>Desbrochers des Loges, 1875	MK892377 [40]<br>2829-PST<br>658nt	ZFMK-TIS-4739<br>ZFMK-DNA-FC17941493	Portugal, Madeira, N of Santana, Quebrada, N32°48'49" W16°52'27", 124m, 25-Nov-2015, sieving, leg. Stüben,P. & Schütte,A., det. Stüben,P.
Cathormiocerus hircus<br>Desbrochers des Loges, 1896	MK891297 [new]<br>1271-PST<br>658nt	ZFMK-TIS-4087<br>ZFMK-DNA-0100426205	Spain, Community of Madrid, NE of Montejo de la Sierra, Hayedo de Montejo, Puerto de El Cardoso, N41°05'14" W03°29'21", 1386m, 02-Mai-2013, *Quercus, Fabaceae*, sieving, leg. Stüben,P. & Schütte,A., det. Borovec,R.
Cathormiocerus horrens<br>(Gyllenhal, 1834)	MK890946 [new]<br>821-PST<br>658nt	ZFMK-TIS-3734<br>ZFMK-DNA-0100413826	Portugal, Minho, Ponte de Lima, Rio Lima, N41°45'48" W08°35'40", 13m, 06-Mai-2012, beating, leg. Stüben,P., det. Borovec,R.
Cathormiocerus myrmecophilus<br>(Seidlitz, 1868)	MK891719 [40]<br>1947-PST<br>658nt	ZFMK-TIS-24026<br>ZFMK-DNA-0169170402	Portugal, Madeira, SW of Santana, Achada do Teixeira, N32°45'51" W16°55'12", 1589m, 04-Okt-2014, *Ulex europaeus, Cytisus scoparius,*, sieving, leg. Stüben,P., det. Stüben,P.
Cathormiocerus myrmecophilus<br>(Seidlitz, 1868)	MK891720 [40]<br>1949-PST<br>658nt	ZFMK-TIS-24028<br>ZFMK-DNA-0169170400	Portugal, Madeira, N of Canhas, Loiral, N32°44'22" W17°06'04", 1338m, 05-Okt-2014, *Ulex europaeus, Cytisus scoparius*, sieving, leg. Stüben,P., det. Stüben,P.
Cathormiocerus peteri<br>Borovec & Bahr, 2008	MK890959 [new]<br>839-PST<br>658nt	ZFMK-TIS-3752<br>ZFMK-DNA-0100414252	Portugal, Estremadura, Sao Martinho do Porto, N39°30'06" W09°08'30", 11m, 15-Mai-2012, sieving, leg. Stüben,P., det. Stüben,P. & Borovec,R.
Cathormiocerus porculus<br>Uhagón, 1885	MK891303 [new]<br>1285-PST<br>658nt	ZFMK-TIS-4101<br>ZFMK-DNA-0100425407	Spain, Community of Madrid, NE of Montejo de la Sierra, Hayedo de Montejo, NE of Hiruela, Puerto de la Hiruela, N41°04'02" W03°28'28", 1478m, 02-Mai-2013, hand collecting, leg. Stüben,P. & Schütte,A., det. Borovec,R.
Cathormiocerus sinuatiscapus<br>Pic, 1915	MK891255 [new]<br>1189-JKR<br>658nt	ZFMK-TIS-3525<br>ZFMK-DNA-0100426106	Spain, Andalucia, Sierra da Baza, Santa Barbara, N37°21'53" W02°51'00", 2019m, 11-Jun-2012, sieving, leg. Krátky,J., det. Krátky,J.
Cathormiocerus spinosus<br>(Goeze, 1777)	MK891514 [new]<br>1601-JKR<br>658nt	ZFMK-TIS-3649<br>ZFMK-DNA-0155635628	Czech Republic, Moravia, Cejc - Spidlaky, N48°55'47" E16°58'03", 184m, 01-Mai-2013, sieving, leg. Krátky,J., det. Krátky,J.
Cathormiocerus spinosus<br>(Goeze, 1777)	MK891786 [new]<br>2045-JKR<br>658nt	ZFMK-TIS-23685<br>ZFMK-DNA-0169170467	Czech Republic, Bohemia, Kosatky, N50°19'23" E14°39'58", 218m, 02-Mai-2014, sieving, leg. Krátky,J., det. Krátky,J.
Caucasusacalles lederi<br>Stüben, 2018<br>**Paratype (DNAtype)**<br>formerly: *Acalles lederi*	MF426961 [34]<br>2754-PST_26851<br>658nt	ZFMK-TIS-26851<br>ZFMK-DNA-0171661897	Russia, Krasnodar, Chvizhepse vill. env. , N43°38'32" E40°04'45", 300m, 10-Jul-2014, leg. Kovalev,A.V., det. Stüben,P.
Caulophilus oryzae<br>(Gyllenhal, 1838)	KC783784 [40]<br>75-PST<br>640nt	ZFMK-TIS-2D100446973<br>ZFMK-DNA-0100437960	Spain, Canary Islands, La Gomera, Agulo – Las Rosas, N28°11'13" W17°12'30", 549m, 12-Feb-2011, *Avocado*, beating, leg. Stüben,P., det. Stüben,P.
Caulophilus sculpturatus<br>Wollaston, 1854<br>formerly: *C. oryzae* - Resyn!	MK347526 [40]<br>2916-PST<br>658nt	(SDEI-DNA-2916-PST)	Portugal, Madeira, E of Porto da Cruz, Larano (Cova das Pedras), N32°45'32" W16°47'42", 490m, 06-Dez-2016, beating, leg. Stüben,P., det. Stüben,P.

**Name**<br>**Authority**<br>**Additional Information**	**GenBank Acc No (Ref.)**<br>**Specimen ID**<br>**Sequence Length**	**ZFMK Tissue ID**<br>**ZFMK DNA Sample ID**<br>**(SDEI DNA Sample ID)**	**Locality, GPS, Collection Date, Plant, Collector, Identifier**
Caulostrophus subsulcatus<br>(Boheman, 1833)	KC784270 [new]<br>IT-0044w<br>651nt	ZFMK-DNA-0100437841	Italy, Sardinia Isl. East, NE of Seui, Mt. Perda Liana, N39°55'13" E09°24'20", 973m, 28-Sep-2010, *Quercus ilex, Ilex*, leg. Stüben,P., det. Stüben,P.
Caulotrupis cf. *chevrolatii*	MH051974 [33]<br>2947-PST<br>658nt	(SDEI-DNA-2947-PST)	Portugal, Madeira, 1 km S of Ribeira da Janela, N32°50'22" W17°08'59", 530m, 16-Nov-2016, *Rubus*, beating, leg. Krátky,J., det. Stüben,P.
Caulotrupis cf. *conicollis*	MH051966 [33]<br>2830-PST<br>658nt	ZFMK-TIS-4740<br>ZFMK-DNA-FC17941501	Portugal, Madeira, N of Santana, Quebrada, N32°48'49" W16°52'27", 124m, 25-Nov-2015, sieving, leg. Stüben,P. & Schütte,A., det. Stüben,P.
Caulotrupis chevrolatii<br>Wollaston, 1854	MH051940 [33]<br>1837-PST<br>658nt	ZFMK-TIS-26040<br>ZFMK-DNA-0171606122	Portugal, Madeira, S of Sao Vicente, Lameiros, N32°47'32" W17°01'29", 288m, 28-Jun-2014, *Rubus*, beating, leg. Stüben,P., det. Stüben,P.
Caulotrupis conicollis<br>Wollaston, 1854	MH051941 [33]<br>1839-PST<br>658nt	ZFMK-TIS-26042<br>ZFMK-DNA-0171606124	Portugal, Madeira, W of Porto Moniz, Santa Madalena, Rib. do Tristao, N32°51'20" W17°12'21", 154m, 29-Jun-2014, *Moraceae*, sieving, leg. Stüben,P., det. Stüben,P.
Caulotrupis conicollis<br>Wollaston, 1854	MH051977 [33]<br>3002-PST<br>658nt	(SDEI-DNA-3002-PST)	Portugal, Madeira, Arco de Sao Jorge to Boaventura, N32°49'34" W16°57'54", 209m, 04-Dez-2016, leg. Stüben,P., det. Stüben,P.
Caulotrupis ficvorator ficvorator<br>Stüben, 2018<br>**Paratype (DNAtype)**	MH013275 [31]<br>1872-PST_26075<br>658nt	ZFMK-TIS-26075<br>ZFMK-DNA-0171606094	Portugal, Madeira, Santa Madalena, Salao, N32°51'59" W17°11'55", 320m, 08-Jul-2014, *Ficus*, step coast, beating, leg. Stüben,P., det. Stüben,P.
Caulotrupis ficvorator ficvorator<br>Stüben, 2018<br>**Paratype (DNAtype)**	MH013277 [31]<br>1889-PST_26092<br>658nt	ZFMK-TIS-26092<br>ZFMK-DNA-0171606078	Portugal, Madeira, near Boaventura, Sao Cristovao, N32°49'35" W16°58'16", 32m, 03-Jul-2014, *Ficus*, beating, leg. Stüben,P., det. Stüben,P.
Caulotrupis ficvorator ficvorator<br>Stüben, 2018<br>**Paratype (DNAtype)**	MH013279 [31]<br>2800-PST_26154<br>658nt	ZFMK-TIS-26154<br>ZFMK-DNA-0169166942	Portugal, Madeira, Faial, N32°47'37" W16°50'57", 26m, 20-Mrz-2015, under boards, collecting by hand, leg. Stüben,P., det. Stüben,P.
Caulotrupis ficvorator ficvorator<br>Stüben, 2018	MH013287 [31]<br>2917-PST<br>658nt	(SDEI-DNA-2917-PST)	Portugal, Madeira, E of Porto da Cruz, Larano (Cova das Pedras), N32°45'32" W16°47'42", 490m, 06-Dez-2016, sifting, leg. Stüben,P., det. Stüben,P.
Caulotrupis ficvorator ficvorator<br>Stüben, 2018	MH013288 [31]<br>2918-PST<br>658nt	(SDEI-DNA-2918-PST)	Portugal, Madeira, N of Machico, Boca do Risco, N32°45'19" W16°46'19", 348m, 07-Dez-2016, sifting, leg. Stüben,P., det. Stüben,P.
Caulotrupis ficvorator ficvorator<br>Stüben, 2018	MH051979 [33]<br>3014-KRA<br>658nt	(SDEI-DNA-3014-KRA)	Portugal, Madeira, Santa M. Madalena, Pombais, costal slopes, N32°51'32" W17°12'10", 400m, 29-Mrz-2017, *Hedera maderensis*, leg. Pelikan, det. Stüben,P.
Caulotrupis ficvorator isambertoi<br>Stüben, 2018<br>**Paratype (DNAtype)**	MH013280 [31]<br>2803-PST_26157<br>658nt	ZFMK-TIS-26157<br>ZFMK-DNA-0169166928	Portugal, Madeira, Ilhas Desertas, Deserta Grande (south), above Focinha, N32°30'18" W16°29'59", 215m, 24-Mrz-2015, collecting by hand, leg. Stüben,P., det. Stüben,P.

**Name**<br>**Authority**<br>**Additional Information**	**GenBank Acc No (Ref.)**<br>**Specimen ID**<br>**Sequence Length**	**ZFMK Tissue ID**<br>**ZFMK DNA Sample ID**<br>**(SDEI DNA Sample ID)**	**Locality, GPS, Collection Date, Plant, Collector, Identifier**
Caulotrupis impius<br>Wollaston, 1854	MH051960 [33]<br>2802-PST<br>658nt	ZFMK-TIS-26156<br>ZFMK-DNA-0169166940	Portugal, Madeira, Ilhas Desertas, Deserta Grande (south), above Furna, N32°30'39" W16°30'09", 247m, 24-Mrz-2015, *Galactites tomentosa*, collecting by hand, leg. Stüben,P., det. Stüben,P.
Caulotrupis impius<br>Wollaston, 1854	MH051961 [33]<br>2804-PST<br>658nt	ZFMK-TIS-26158<br>ZFMK-DNA-0169166929	Portugal, Madeira, Ilhas Desertas, Deserta Grande (south), above Focinha, N32°30'18" W16°29'59", 215m, 24-Mrz-2015, collecting by hand, leg. Stüben,P., det. Stüben,P.
Caulotrupis impius<br>Wollaston, 1854	MH051962 [33]<br>2806-PST<br>658nt	ZFMK-TIS-26160<br>ZFMK-DNA-0169166931	Portugal, Madeira, Ilhas Desertas, Ilheu Chao, above Furna, N32°35'17" W16°32'40", 38m, 26-Mrz-2015, *Sonchus ustulatus*, sieving, leg. Stüben,P., det. Stüben,P.
Caulotrupis impius<br>Wollaston, 1854	MH051949 [33]<br>1888-PST<br>658nt	ZFMK-TIS-26091<br>ZFMK-DNA-0171606077	Portugal, Madeira, near Machico, Queimado, N32°42'45" W16°45'51", 162m, 16-Jul-2014, *Euphorbia piscatoria*, beating, leg. Stüben,P., det. Stüben,P.
Caulotrupis impius<br>Wollaston, 1854	MH051965 [33]<br>2823-PST<br>658nt	ZFMK-TIS-25869<br>ZFMK-DNA-FC17941532	Portugal, Madeira, Ponta de Sao Lourenco, Pedras Brancas, N32°44'49" W16°42'12", 100m, 23-Nov-2015, *Cynara cardunculus*, sieving, leg. Stüben,P. & Schütte,A., det. Stüben,P.
Caulotrupis impius<br>Wollaston, 1854	MH051969 [33]<br>2915-PST<br>658nt	(SDEI-DNA-2915-PST)	Portugal, Madeira, Achada da Cruz, Quebrada Nova, N32°51'21" W17°12'50", 21m, 05-Dez-2016, sifting, leg. Stüben,P., det. Stüben,P.
Caulotrupis impius<br>Wollaston, 1854	MH051970 [33]<br>2919-PST<br>658nt	(SDEI-DNA-2919-PST)	Portugal, Madeira, Porto da Cruz, N32°46'05" W16°49'21", 11m, 10-Dez-2016, *Asteraceae*, beating, leg. Stüben,P., det. Stüben,P.
Caulotrupis impius<br>Wollaston, 1854<br>formerly: *C. terebrans* - Syn!	MH051968 [33]<br>2836-PST<br>658nt	ZFMK-TIS-4746<br>ZFMK-DNA-FC17941549	Portugal, Madeira, Porto Santo Isl., Pico d Ana Ferreira, N33°02'43" W16°22'13", 206m, 26-Nov-2015, *Cynara cardunculus*, sieving, leg. Stüben,P. & Schütte,A., det. Stüben,P.
Caulotrupis lacertosus<br>Wollaston, 1854	MH051938 [33]<br>1827-PST<br>658nt	ZFMK-TIS-26030<br>ZFMK-DNA-0171606139	Portugal, Madeira, S of Seixal, S of Chao da Ribeira, N32°48'41" W17°06'38", 411m, 27-Jun-2014, *Euphorbia mellifera*, beating, leg. Stüben,P., det. Stüben,P.
Caulotrupis lacertosus<br>Wollaston, 1854<br>formerly: *C. subnitidus* - Syn!	MH051943 [33]<br>1848-PST<br>658nt	ZFMK-TIS-26051<br>ZFMK-DNA-0171606118	Portugal, Madeira, Ribeira da Janela near coast, N32°51'15" W17°09'14", 27m, 29-Jun-2014, *Euphorbia piscatoria*, beating, leg. Stüben,P., det. Stüben,P.
Caulotrupis lacertosus<br>Wollaston, 1854	MH051947 [33]<br>1866-PST<br>658nt	ZFMK-TIS-26069<br>ZFMK-DNA-0171606103	Portugal, Madeira, S of Boaventura, Achada da Madeira, N32°47'03" W16°58'41", 488m, 03-Jul-2014, *Euphorbia mellifera*, beating, leg. Stüben,P., det. Stüben,P.
Caulotrupis lacertosus<br>Wollaston, 1854<br>formerly: *C. subnitidus* - Syn!	MH051948 [33]<br>1886-PST<br>658nt	ZFMK-TIS-26089<br>ZFMK-DNA-0171606075	Portugal, Madeira, near Machico, Queimado, N32°42'45" W16°45'51", 162m, 16-Jul-2014, *Euphorbia piscatoria*, beating, leg. Stüben,P., det. Stüben,P.
Caulotrupis lacertosus<br>Wollaston, 1854	MH051958 [33]<br>2799-PST<br>658nt	ZFMK-TIS-26153<br>ZFMK-DNA-0169166943	Portugal, Madeira, Faial, N32°47'37" W16°50'57", 26m, 20-Mrz-2015, under boards, collecting by hand, leg. Stüben,P., det. Stüben,P.
Caulotrupis lacertosus<br>Wollaston, 1854	MH051964 [33]<br>2810-PST<br>658nt	ZFMK-TIS-26164<br>ZFMK-DNA-0169166935	Portugal, Madeira, Paul do Mar, N32°45'34" W17°13'43", 62m, 04-Apr-2015, *Ficus carica, Euphorbia piscatoria*, banana terraces, sieving, leg. Stüben,P., det. Stüben,P.
Caulotrupis lacertosus<br>Wollaston, 1854	MH051978 [33]<br>3012-KRA<br>658nt	(SDEI-DNA-3012-KRA)	Portugal, Madeira, Salao, Santa M. Madalena, N32°51'56" W17°11'58", 322m, 26-Mrz-2017, *Ficus, Hedera*, leg. Pelikan, det. Stüben,P.

**Name**<br>**Authority**<br>**Additional Information**	**GenBank Acc No (Ref.)**<br>**Specimen ID**<br>**Sequence Length**	**ZFMK Tissue ID**<br>**ZFMK DNA Sample ID**<br>**(SDEI DNA Sample ID)**	**Locality, GPS, Collection Date, Plant, Collector, Identifier**
Caulotrupis lourencoensis<br>Stüben, 2018	MH013283 [31]<br>2824a-PST<br>658nt	ZFMK-TIS-25870<br>ZFMK-DNA-FC17941540	Portugal, Madeira, Ponta de Sao Lourenco, Pedras Brancas, N32°44'49" W16°42'12", 100m, 23-Nov-2015, *Cynara cardunculus*, sieving, leg. Stüben,P. & Schütte,A., det. Stüben,P.
Caulotrupis lourencoensis<br>Stüben, 2018	MH013289 [31]<br>2920-PST<br>656nt	(SDEI-DNA-2920-PST)	Portugal, Madeira, Ponta des Sao Lourenco, N32°44'51" W16°42'13", 115m, 12-Dez-2016, *deadwood*, beating, leg. Stüben,P., det. Stüben,P.
Caulotrupis lucifugus faialensis<br>Stüben, 2018<br>**Paratype (DNAtype)**	MH013276 [31]<br>1882-PST_26085<br>658nt	ZFMK-TIS-26085<br>ZFMK-DNA-0171606084	Portugal, Madeira, W of Sao Jorge, Achada da Cruz, N32°49'37" W16°53'37", 205m, 14-Jul-2014, *Ficus*, beating, leg. Stüben,P., det. Stüben,P.
Caulotrupis lucifugus faialensis<br>Stüben, 2018<br>**Paratype (DNAtype)**	MH013278 [31]<br>1940-PST_24019<br>658nt	ZFMK-TIS-24019<br>ZFMK-DNA-0169170418	Portugal, Madeira, Faial, coast, N32°47'37" W16°50'57", 26m, 04-Okt-2014, sieving, leg. Stüben,P., det. Stüben,P.
Caulotrupis lucifugus faialensis<br>Stüben, 2018	MH013285 [31]<br>2906-PST<br>658nt	(SDEI-DNA-2906-PST)	Portugal, Madeira, Ponta Delgada, N32°49'39" W16°59'12", 46m, 29-Nov-2016, *Ficus*, sifting, leg. Stüben,P., det. Stüben,P.
Caulotrupis lucifugus faialensis<br>Stüben, 2018	MH013286 [31]<br>2907-PST<br>658nt	(SDEI-DNA-2907-PST)	Portugal, Madeira, Santa Madalena, N32°51'54" W17°12'03", 290m, 30-Nov-2016, *Hedera*, sifting, leg. Stüben,P., det. Stüben,P.
Caulotrupis lucifugus faialensis<br>Stüben, 2018	MH051976 [33]<br>3001-PST<br>658nt	(SDEI-DNA-3001-PST)	Portugal, Madeira, Boaventura, N32°49'33" W16°58'10", 90m, 03-Dez-2016, *Ficus*, leg. Stüben,P., det. Stüben,P.
Caulotrupis lucifugus lucifugus<br>Wollaston, 1854	MH051959 [33]<br>2801-PST<br>658nt	ZFMK-TIS-26155<br>ZFMK-DNA-0169166941	Portugal, Madeira, Ilhas Desertas, Deserta Grande, Doca, N32°30'49" W16°30'33", 23m, 22-Mrz-2015, under boards, collecting by hand, leg. Stüben,P., det. Stüben,P.
Caulotrupis lucifugus lucifugus<br>Wollaston, 1854	MH051945 [33]<br>1861-PST<br>658nt	ZFMK-TIS-26064<br>ZFMK-DNA-0171606098	Portugal, Madeira, Paul do Mar, N32°45'34" W17°13'43", 62m, 02-Jul-2014, *Ficus*, sieving, leg. Stüben,P., det. Stüben,P.
Caulotrupis lucifugus lucifugus<br>Wollaston, 1854	MH051963 [33]<br>2809-PST<br>658nt	ZFMK-TIS-26163<br>ZFMK-DNA-0169166934	Portugal, Madeira, E of Funchal, W of Garajau, Rua do Lazareto, N32°38'49" W16°53'16", 41m, 03-Apr-2015, *Ficus*, sieving, leg. Stüben,P., det. Stüben,P.
Caulotrupis lucifugus lucifugus<br>Wollaston, 1854	MH051975 [33]<br>3000-PST<br>658nt	(SDEI-DNA-3000-PST)	Portugal, Madeira, Madalena do Mar, N32°41'26" W17°07'11", 49m, 02-Dez-2016, *Musca*, leg. Stüben,P., det. Stüben,P.
Caulotrupis mauli<br>(Folwaczny, 1972)<br>formerly: *Salvopselactus*	MH051950 [33]<br>1965-PST<br>658nt	ZFMK-TIS-24044<br>ZFMK-DNA-0169170395	Portugal, Madeira, Ilhas Selvagens, Selvagem Grande, barranco above Cave Kidd, N30°08'30" W15°51'36", 85m, 20-Okt-2014, *Suaeda vera*, sieving, leg. Stüben,P., det. Stüben,P.
Caulotrupis mauli<br>(Folwaczny, 1972)<br>formerly: *Salvopselactus*	MK891730 [40]<br>1970-PST<br>658nt	ZFMK-TIS-24049<br>ZFMK-DNA-0169170379	Portugal, Madeira, Ilhas Selvagens, Selvagem Pequena, N of Pico do Veado, N30°02'09" W16°01'39", 40m, 20-Dez-2014, *Patellifolia patellaris var. campunulata*, sieving, leg. Stüben,P., det. Stüben,P.
Caulotrupis opacus<br>Wollaston, 1854	MH051944 [33]<br>1852-PST<br>658nt	ZFMK-TIS-26055<br>ZFMK-DNA-0171606114	Portugal, Madeira, near Boca da Encumeada, Folhadal, N32°45'10" W17°01'59", 1103m, 01-Jul-2014, *Euphorbia mellifera*, beating, leg. Stüben,P., det. Stüben,P.

**Name**<br>**Authority**<br>**Additional Information**	**GenBank Acc No (Ref.)**<br>**Specimen ID**<br>**Sequence Length**	**ZFMK Tissue ID**<br>**ZFMK DNA Sample ID**<br>**(SDEI DNA Sample ID)**	**Locality, GPS, Collection Date, Plant, Collector, Identifier**
Caulotrupis xerophilus desertagrandensis<br>Stüben, 2018<br>**Paratype (DNAtype)**	MH013282 [31]<br>2807-PST_26161<br>658nt	ZFMK-TIS-26161<br>ZFMK-DNA-0169166932	Portugal, Madeira, Ilhas Desertas, Deserta Grande (north), above Quebrada, N32°33'47" W16°32'07", 204m, 30-Mrz-2015, *Calendula maderensis, Helminthotheca echioides*, sieving, leg. Stüben,P., det. Stüben,P.
Caulotrupis xerophilus xerophilus<br>Stüben, 2018<br>**Paratype (DNAtype)**	MH013281 [31]<br>2805-PST_26159<br>658nt	ZFMK-TIS-26159<br>ZFMK-DNA-0169166930	Portugal, Madeira, Ilhas Desertas, Ilheu Chao, center, N32°35'07" W16°32'40", 61m, 26-Mrz-2015, *Crepis divaricata, Sonchus ustulatus*, sieving, leg. Stüben,P., det. Stüben,P.
Caulotrupis xerophilus xerophilus<br>Stüben, 2018<br>**Paratype (DNAtype)**	MH013284 [31]<br>2841-PST_4237<br>658nt	ZFMK-TIS-4237<br>ZFMK-DNA-FC17941502	Portugal, Madeira, Porto Santo Isl., Pico do Castelo, N33°04'44" W16°19'57", 273m, 27-Nov-2015, *Quercus ilex, Cynara cardunculus*, sieving, leg. Stüben,P., det. Stüben,P.
Centricnemus leucogrammus<br>(Germar, 1824)	KC784114 [new]<br>494-RST<br>658nt	ZFMK-TIS-20279<br>ZFMK-DNA-0100448485	Czech Republic, Moravia mer., 1 km NW of Derflice, N48°49'8.80" E16°8'13.24", 300m, 16-Apr-2011, sweeping, leg. Stejskal,R., det. Stejskal,R.
Centricnemus leucogrammus<br>(Germar, 1824)	MK891503 [new]<br>1589-JKR<br>658nt	ZFMK-TIS-3637<br>ZFMK-DNA-0155635635	Czech Republic, Moravia, Cejc - Spidlaky, N48°55'47" E16°58'03", 184m, 01-Mai-2013, sieving, leg. Krátky,J., det. Krátky,J.
Centricnemus leucogrammus<br>(Germar, 1824)	MK891953 [new]<br>2221-JKR<br>658nt	ZFMK-TIS-23959<br>ZFMK-DNA-0169170566	Slovakia, Komarno, Iza, N47°44'54" E18°15'27", 107m, 15-Jun-2015, leg. Benedikt,S., det. Benedikt,S.
Centricnemus leucogrammus<br>(Germar, 1824)	MK892222 [new]<br>2519-PSP<br>658nt	ZFMK-TIS-23491<br>ZFMK-DNA-0171661761	Slovakia, Nitra, Imel, N47°55'10" E18°09'37", 113m, 18-Mai-2014, *herb layer*, leg. Sprick,P., det. Sprick,P.
Centricnemus leucogrammus<br>(Germar, 1824)	MK892249 [new]<br>2548-PSP<br>658nt	ZFMK-TIS-23522<br>ZFMK-DNA-0171662011	Slovakia, Nitra, Cenkov, N47°46'07" E18°31'11", 109m, 19-Mai-2014, *Alyssum montanum gmelini*, leg. Sprick,P., det. Sprick,P.
Ceratapion armatum<br>(Gerstaecker, 1854)	MK892274 [new]<br>2575-PSP<br>658nt	ZFMK-TIS-23549<br>ZFMK-DNA-0171661984	Germany, Bavaria (BY), Unterfranken, Ebern, N50°05'18" E10°45'58", 334m, 13-Jun-2014, *Centaurea jacea*, leg. Sprick,P., det. Sprick,P.
Ceratapion austriacum<br>(Wagner, 1904)	KC784087 [new]<br>536-RST<br>658nt	ZFMK-TIS-20321<br>ZFMK-DNA-0100448446	Czech Republic, Bohemia occ., 2.1 km SE of Horazdovice env., N49°18'49.17" E13°43'53.59", 440m, 28-Aug-2011, *Centaurea scabiosa*, beating, leg. Stejskal,R., det. Stejskal,R.
Ceratapion austriacum<br>(Wagner, 1904)	MK892109 [new]<br>2386-JKR<br>658nt	ZFMK-TIS-25963<br>ZFMK-DNA-0171600603	Czech Republic, Moravia, Dyjakovicky, Jecmenist?, N48°44'48" E16°08'47", 250m, 11-Sep-2014, *Centaurea scabiosa*, beating, leg. R. Stejskal, det. Stejskal,R.
Ceratapion calcaratum<br>(Wollaston, 1864)	MK347546 [40]<br>2959a-PST<br>658nt	(SDEI-DNA-2959a-PST)	Spain, Canary Islands, El Hierro, W Frontera, Pista al Derrabado, N27°44'23" W18°02'58", 929m, 11-Apr-2016, leg. Stüben & Schütte, det. Stüben,P.
Ceratapion calcaratum<br>(Wollaston, 1864)	MK891572 [40]<br>25-PST<br>658nt	ZFMK-TIS-2D100446877<br>ZFMK-DNA-0155630449	Spain, Canary Islands, Gran Canaria, Las Lagunetas, Barranco de la Mina, N27°59'56" W15°35'13", 1252m, 10-Dez-2010, *Carduus clavulatus*, beating, leg. Stüben,P., det. Stüben,P.
Ceratapion calcaratum<br>(Wollaston, 1864)	KC783787 [40]<br>78-PST<br>658nt	ZFMK-TIS-2D100446970<br>ZFMK-DNA-0100438042	Spain, Canary Islands, La Gomera, E of Chipude, Fayal-Brezal, N28°6'26" W17°15'33", 1247m, 14-Feb-2011, *Carduus clavulatus*, beating, leg. Stüben,P., det. Stüben,P.

**Name**<br>**Authority**<br>**Additional Information**	**GenBank Acc No (Ref.)**<br>**Specimen ID**<br>**Sequence Length**	**ZFMK Tissue ID**<br>**ZFMK DNA Sample ID**<br>**(SDEI DNA Sample ID)**	**Locality, GPS, Collection Date, Plant, Collector, Identifier**
Ceratapion calcaratum<br>(Wollaston, 1864)	MK891612 [40]<br>1708-PST<br>658nt	ZFMK-TIS-24426<br>ZFMK-DNA-0155622489	Spain, Canary Islands, La Palma, NW of Puntallana, Cubo de la Galga, N28°45'38" W17°46'42", 537m, 27-Dez-2013, *Carduus clavulatus*, beating, leg. Stüben,P., det. Stüben,P.
Ceratapion calcaratum<br>(Wollaston, 1864)	KC784288 [40]<br>707-PST<br>658nt	ZFMK-TIS-3044<br>ZFMK-DNA-0100448181	Spain, Canary Islands, Tenerife, Icod, La Vega, N28°21'25" W16°43'44", 647m, 04-Jan-2012, *Carduus clavulatus*, beating, leg. Stüben,P., det. Stüben,P.
Ceratapion cylindricolle<br>(Gyllenhal, 1839)	KC784095 [new]<br>522-RST<br>658nt	ZFMK-TIS-20307<br>ZFMK-DNA-0100448457	Romania, Caras-Severin, 3.3 km NW of Coronini, N44°42'20.88" E21°39'56.65", 250m, 26-Jul-2011, *pastures*, along Danube (ger. Donau) river, sweeping, leg. Stejskal,R., det. Stejskal,R.
Ceratapion cylindricolle<br>(Gyllenhal, 1839)	MK892156 [new]<br>2444-JKR<br>658nt	ZFMK-TIS-26021<br>ZFMK-DNA-0171600666	Slovakia, Nove Zamky, Imel, PR Liscie diery, N47°55'17" E18°09'37", 114m, 20-Sep-2014, *Xeranthemum annuum*, sweeping, leg. Krátky,J., det. Krátky,J.
Ceratapion damryi<br>(Desbrochers des Loges, 1894)	MK890810 [new]<br>144-PST<br>658nt	ZFMK-TIS-2D100440286<br>ZFMK-DNA-0100438174	Morocco, S of Berkane, N of Aïn-es-Sfa, Beni Snassen Mts., N34°51'30" W02°08'41", 840m, 13-Mai-2011, beating, leg. Stüben,P., det. Behne,L.
Ceratapion gibbirostre<br>(Gyllenhal, 1813)	KC784060 [new]<br>573-PSP<br>658nt	ZFMK-TIS-20358<br>ZFMK-DNA-0100448406	Germany, Lower Saxony (NI), Neindorf, Lkr. Wolfenbüttel, Öselberg, N52°07'40" E10°35'32", 132m, 24-Jun-2011, *Carduus acanthoides*, beating, leg. Sprick,P., det. Sprick,P.
Ceratapion gibbirostre<br>(Gyllenhal, 1813)	KC784184 [new]<br>481-PST<br>658nt	ZFMK-TIS-20266<br>ZFMK-DNA-0100449355	Germany, Rhineland-Palatinate (RLP), Mosellus area, Kobern-Gondorf, "Sürzer Höfe", ruderal area, N50°20'20" E07°25'25", 330m, 30-Sep-2011, hand-collecting, leg. Stüben,P., det. Stüben,P.
Ceratapion onopordi onopordi<br>(Kirby, 1808)	MK891810 [new]<br>2070-JKR<br>658nt	ZFMK-TIS-23710<br>ZFMK-DNA-0169168581	Czech Republic, Bohemia, Ujezd u Sezemic, N50°07'7.5" E15°51'37", 234m, 08-Mai-2014, *Cirsium* sp., sweeping, leg. Krátky,J., det. Krátky,J.
Ceratapion onopordi onopordi<br>(Kirby, 1808)	MK892097 [new]<br>2374-JKR<br>658nt	ZFMK-TIS-25951<br>ZFMK-DNA-0171600596	Czech Republic, Moravia, Znojmo, N48°51'05" E16°05'59", 250m, 23-Aug-2014, sweeping, leg. R. Stejskal, det. Stejskal,R.
Ceratapion onopordi onopordi<br>(Kirby, 1808)	KC783981 [new]<br>244-PSP<br>658nt	ZFMK-TIS-2D100439138<br>ZFMK-DNA-0100439558	Germany, Lower Saxony (NI), Koldingen, Hannover region, N52°16'29" E09°49'08", 59m, 10-Sep-2011, *Cirsium arvense, C. vulgare*, Leine river wetland, wayside/fallow, beating, leg. Sprick,P., det. Sprick,P.
Ceratapion onopordi onopordi<br>(Kirby, 1808)	KC784188 [40]<br>474-PST<br>658nt	ZFMK-TIS-20259<br>ZFMK-DNA-0100449365	Germany, Rhineland-Palatinate (RLP), "Korretsberg" near Kruft, Eifel, N50°22'49" E07°21'11", 238m, 30-Sep-2011, volcanic cone, hand-collecting, leg. Stüben,P., det. Stüben,P.
Ceratapion onopordi onopordi<br>(Kirby, 1808)	MK892312 [new]<br>2613-PSP<br>658nt	ZFMK-TIS-23587<br>ZFMK-DNA-0171661953	Germany, Bavaria (BY), Unterfranken, Ebern, N50°04'55" E10°45'52", 297m, 14-Jun-2014, *Cirsium vulgare*, leg. Sprick,P., det. Sprick,P.
Ceratapion onopordi onopordi<br>(Kirby, 1808)	MK892232 [new]<br>2530-PSP<br>658nt	ZFMK-TIS-23502<br>ZFMK-DNA-0171661743	Slovakia, Nitra, SW of Buc, N47°46'56" E18°25'10", 110m, 18-Mai-2014, *Carduus* sp., leg. Sprick,P., det. Sprick,P.
Ceratapion penetrans penetrans<br>(Germar, 1817)	KC784083 [new]<br>530-RST<br>658nt	ZFMK-TIS-20315<br>ZFMK-DNA-0100448440	Czech Republic, Moravia mer., Podyji NP, Havraniky - heathland , N48°48'46.24" E16°0'1.49", 320m, 12-Aug-2011, *Centaurea stoebe*, sweeping, leg. Stejskal,R., det. Stejskal,R.
Ceratapion penetrans penetrans<br>(Germar, 1817)	MK892275 [new]<br>2576-PSP<br>658nt	ZFMK-TIS-23550<br>ZFMK-DNA-0171661983	Germany, Bavaria (BY), Unterfranken, Ebern, N50°05'20.5" E10°45'59", 333m, 13-Jun-2014, *Centaurea jacea*, leg. Sprick,P., det. Sprick,P.

**Name**<br>**Authority**<br>**Additional Information**	**GenBank Acc No (Ref.)**<br>**Specimen ID**<br>**Sequence Length**	**ZFMK Tissue ID**<br>**ZFMK DNA Sample ID**<br>**(SDEI DNA Sample ID)**	**Locality, GPS, Collection Date, Plant, Collector, Identifier**
Ceratapion penetrans penetrans<br>(Germar, 1817)	MK891381 [new]<br>1434-JKR<br>658nt	ZFMK-TIS-4203<br>ZFMK-DNA-0155628499	Slovakia, Komarno, Imel, PR Liscie diery, N47°55'12" E18°09'39", 114m, 19-Jul-2013, *Centaurea stoebe*, sweeping, leg. Krátky,J., det. Krátky,J.
Ceratapion penetrans penetrans<br>(Germar, 1817)	MK892145 [new]<br>2432-JKR<br>658nt	ZFMK-TIS-26009<br>ZFMK-DNA-0171600653	Slovakia, Nitra, Koli?any, Koli?ansky vrch, N48°20'15.5" E18°10'47", 261m, 21-Sep-2014, sweeping, leg. Krátky,J., det. Stüben,P.
Ceratapion penetrans penetrans<br>(Germar, 1817)	MK892227 [new]<br>2525-PSP<br>658nt	ZFMK-TIS-23497<br>ZFMK-DNA-0171661748	Slovakia, Nitra, SW of Buc, N47°46'56" E18°25'10", 110m, 18-Mai-2014, *Centaurea stoebe*, leg. Sprick,P., det. Sprick,P.
Ceutorhynchus cf. *hirtulus*<br>Germar, 1824	MK891874 [new]<br>2138-JKR<br>658nt	ZFMK-TIS-23778<br>ZFMK-DNA-0169169600	Slovakia, Komarno, Buc, N47°46'55.5" E18°25'10", 107m, 18-Mai-2014, *Alyssum alyssoides*, sweeping, leg. Krátky,J., det. Krátky,J. Note: specimen is identified as *C. hirtulus*, but groups molecularly to *C. varius*.
Ceutorhynchus pyrrhorhynchus canariensis<br>(Har. Lindberg, 1950)<br>formerly: *C. canariensis*	KU932050 [27]<br>2724-PST<br>658nt	ZFMK-TIS-23874<br>ZFMK-DNA-0171661280	Spain, Canary Islands, Fuerteventura, Betancuria, N28°25'09" W14°03'32", 380m, 04-Jan-2015, *Sisymbrium*, beating, leg. Stüben,P., det. Stüben,P.
Ceutorhynchus pyrrhorhynchus canariensis<br>(Har. Lindberg, 1950)<br>formerly: *C. canariensis*	KU932051 [27]<br>2733-PST<br>658nt	ZFMK-TIS-23883<br>ZFMK-DNA-0171661266	Spain, Canary Islands, Fuerteventura, S of La Oliva, Vallebron, N28°35'01" W13°55'07", 252m, 06-Jan-2015, *Sisymbrium*, beating, leg. Stüben,P., det. Stüben,P.
Ceutorhynchus pyrrhorhynchus canariensis<br>(Har. Lindberg, 1950)<br>formerly: *C. canariensis*	MK347529 [40]<br>2925-PST<br>658nt	(SDEI-DNA-2925-PST)	Spain, Canary Islands, Lanzarote, Ye, N29°11'48" W13°28'42", 365m, 04-Jan-2017, *Lobularia*, beating, leg. Stüben,P., det. Stüben,P.
Ceutorhynchus pyrrhorhynchus canariensis<br>(Har. Lindberg, 1950)<br>formerly: *C. canariensis*	MK347532 [40]<br>2931-PST<br>658nt	(SDEI-DNA-2931-PST)	Spain, Canary Islands, Lanzarote, above Haria, N29°08'08" W13°30'22", 358m, 04-Jan-2017, *Lobularia*, beating, leg. Stüben,P., det. Stüben,P.
Ceutorhynchus aeneicollis<br>Germar, 1824	MK891320 [new]<br>1319-PST<br>658nt	ZFMK-TIS-4135<br>ZFMK-DNA-0100426157	Spain, Malaga, S of Mollina, N37°04'45" W04°40'08", 437m, 07-Mai-2013, *wayside vegetation in olive plantation*, beating, leg. Stüben,P. & Schütte,A., det. Stüben,P.
Ceutorhynchus alliariae<br>C. Brisout de Barneville, 1860	KC784019 [new]<br>222-PSP<br>658nt	ZFMK-TIS-2D100438525<br>ZFMK-DNA-0100439821	Germany, Lower Saxony (NI), Hannover, Linden, Park S of Leine river, N52°22'40" E09°42'30", 55m, 18-Apr-2011, *Alliaria petiolata*, beating, leg. Sprick,P., det. Sprick,P.
Ceutorhynchus alliariae<br>H. Brisout de Barneville, 1860	MK891100 [new]<br>1004-PSP<br>658nt	ZFMK-TIS-3149<br>ZFMK-DNA-0100426855	Germany, Lower Saxony (NI), Aerzen-Ahorn, Lkr. Hameln-Pyrmont, N52°03'17" E09°13'02", 171m, 05-Mai-2012, *Alliaria petiolata*, wet forest track, dipnet, leg. Sprick,P., det. Sprick,P.
Ceutorhynchus alyssi<br>Peyerimhoff, 1925	MK891500 [new]<br>1585-JKR<br>658nt	ZFMK-TIS-3633<br>ZFMK-DNA-0155635644	Morocco, Haut Atlas, Oukaimeden, N31°12'10" W07°52'13", 2691m, 20-Apr-2013, *Alyssum spinosum*, sieving, leg. Krátky,J., det. Krátky,J.
Ceutorhynchus arator<br>Gyllenhal, 1837	MK891522 [new]<br>1609-JKR<br>658nt	ZFMK-TIS-3657<br>ZFMK-DNA-0155635620	Czech Republic, Moravia, Brezi env., NPP Dunajovicke vrchy, N48°50'23" E16°33'46", 260m, 02-Mai-2013, *Crambe tataria*, sweeping, leg. Krátky,J., det. Krátky,J.

**Name**<br>**Authority**<br>**Additional Information**	**GenBank Acc No (Ref.)**<br>**Specimen ID**<br>**Sequence Length**	**ZFMK Tissue ID**<br>**ZFMK DNA Sample ID**<br>**(SDEI DNA Sample ID)**	**Locality, GPS, Collection Date, Plant, Collector, Identifier**
Ceutorhynchus arator<br>Gyllenhal, 1837	KC783939 [new]<br>340-JKR<br>658nt	ZFMK-TIS-20126<br>ZFMK-DNA-0100438754	Slovakia, Komarno, Salka env., PR Sovie vinohrady, N47°53'17.312" E18°43'2.989", 214m, 22-Mai-2011, *Crambe tataria*, hand-collecting, leg. Krátky,J., det. Krátky,J.
Ceutorhynchus assimilis<br>(Paykull, 1792)	MK891799 [new]<br>2058-JKR<br>658nt	ZFMK-TIS-23698<br>ZFMK-DNA-0169170432	Czech Republic, Bohemia, Hradec Kralove, Kocianovice, N50°13'16" E15°52'51", 237m, 06-Mai-2014, *Cardaria draba*, collecting by hand, leg. Krátky,J., det. Krátky,J.
Ceutorhynchus assimilis<br>(Paykull, 1792)	MK891893 [new]<br>2158-JKR<br>658nt	ZFMK-TIS-23798<br>ZFMK-DNA-0169169572	Slovakia, Nove Zamky, Kamenica nad Hronom, NPR Skaly, N47°49'56" E18°44'08", 171m, 20-Mai-2014, *Lepidium campestre*, collecting by hand, leg. Krátky,J., det. Krátky,J.
Ceutorhynchus assimilis<br>(Paykull, 1792)	MK892221 [40]<br>2518-PSP<br>658nt	ZFMK-TIS-23490<br>ZFMK-DNA-0171661760	Slovakia, Nitra, Imel, N47°55'10" E18°09'37", 113m, 18-Mai-2014, *Cardaria draba*, leg. Sprick,P., det. Sprick,P.
Ceutorhynchus assimilis<br>(Paykull, 1792)	MK892259 [new]<br>2559-PSP<br>658nt	ZFMK-TIS-23533<br>ZFMK-DNA-0171661995	Slovakia, Nitra, Muzla, N47°48'00" E18°31'55", 114m, 19-Mai-2014, *Cardaria draba*, leg. Sprick,P., det. Sprick,P.
Ceutorhynchus assimilis<br>(Paykull, 1792)	MK891162 [new]<br>1070-JKR<br>658nt	ZFMK-TIS-3406<br>ZFMK-DNA-0100449638	Spain, Andalucia, 6 km NW of Los Naveros, N36°22'54" W06°02'13", 25m, 16-Apr-2012, *Cardamine* sp., sweeping, leg. Krátky,J., det. Krátky,J.
Ceutorhynchus assimilis<br>(Paykull, 1792)	MK891163 [new]<br>1071-JKR<br>658nt	ZFMK-TIS-3407<br>ZFMK-DNA-0100449090	Spain, Andalucia, 6 km NW of Los Naveros, N36°22'54" W06°02'13", 25m, 16-Apr-2012, *yellow flowering Brassicaceae*, sweeping, leg. Krátky,J., det. Krátky,J.
Ceutorhynchus assimilis<br>(Paykull, 1792)	MK891167 [new]<br>1078-JKR<br>658nt	ZFMK-TIS-3414<br>ZFMK-DNA-0100449630	Spain, Andalucia, 3 km NE of El Lentiscal, N36°06'07" W05°44'37", 130m, 15-Apr-2012, *Hirschfeldia incana*, sweeping, leg. Krátky,J., det. Krátky,J.
Ceutorhynchus atomus<br>Boheman, 1845	MK891934 [new]<br>2202-JKR<br>658nt	ZFMK-TIS-23940<br>ZFMK-DNA-0169170578	Czech Republic, Moravia, Vevcice, N48°58'06" E16°01'37", 325m, 04-Apr-2014, *Arabidopsis thaliana*, sweeping, leg. Stejskal,R., det. Stejskal,R.
Ceutorhynchus atomus<br>Boheman, 1845	MK891083 [new]<br>986-PSP<br>658nt	ZFMK-TIS-3131<br>ZFMK-DNA-0100426947	Germany, Lower Saxony (NI), near Hannover, Elze, Wedemark, N52°35'11" E09°45'00", 39m, 28-Apr-2012, *Arabidopsis thaliana*, nutrient-poor sand grassland, dipnet, leg. Sprick,P., det. Sprick,P.
Ceutorhynchus barbareae<br>Suffrian, 1847	MK891971 [new]<br>2242-JKR<br>658nt	ZFMK-TIS-23980<br>ZFMK-DNA-0169170545	Slovakia, Zvolen, Hronska Dubrava, NPR Boky, N48°33'58" E19°01'10.5", 472m, 12-Okt-2014, *Barbaraea vularis*, collecting by hand, leg. Krátky,J., det. Krátky,J.
Ceutorhynchus carinatus<br>Gyllenhal, 1837	MK891831 [new]<br>2092-JKR<br>658nt	ZFMK-TIS-23732<br>ZFMK-DNA-0169169650	Czech Republic, Moravia, Bucovice, PR Sevy, N49°08'05" E16°58'19", 249m, 09-Mai-2014, *Lepidium campestre*, sweeping, leg. Krátky,J., det. Krátky,J.
Ceutorhynchus carinatus<br>Gyllenhal, 1837	KC783937 [new]<br>338-JKR<br>658nt	ZFMK-TIS-20124<br>ZFMK-DNA-0100438752	Slovakia, Komarno, Iza env., PR Bokrosske slanisko , N47°44'50.372" E18°15'34.563", 107m, 20-Mai-2011, *Lepidium campestre*, hand-collecting, leg. Krátky,J., det. Krátky,J.
Ceutorhynchus carinatus<br>Gyllenhal, 1837	MK891865 [new]<br>2129-JKR<br>658nt	ZFMK-TIS-23769<br>ZFMK-DNA-0169169604	Slovakia, Nove Zamky, Sturovo, Hegyfarok, N47°49'06" E18°38'38", 205m, 17-Mai-2014, *Erysimum* sp., sweeping, leg. Krátky,J., det. Krátky,J.
Ceutorhynchus carinatus<br>Gyllenhal, 1837	MK891867 [new]<br>2131-JKR<br>658nt	ZFMK-TIS-23771<br>ZFMK-DNA-0169169593	Slovakia, Nove Zamky, Sturovo, Hegyfarok, N47°49'06" E18°38'38", 205m, 17-Mai-2014, *Crambe tataria*, sweeping, leg. Krátky,J., det. Krátky,J.

**Name**<br>**Authority**<br>**Additional Information**	**GenBank Acc No (Ref.)**<br>**Specimen ID**<br>**Sequence Length**	**ZFMK Tissue ID**<br>**ZFMK DNA Sample ID**<br>**(SDEI DNA Sample ID)**	**Locality, GPS, Collection Date, Plant, Collector, Identifier**
Ceutorhynchus carinatus<br>Gyllenhal, 1837	MK891884 [new]<br>2149-JKR<br>658nt	ZFMK-TIS-23789<br>ZFMK-DNA-0169169584	Slovakia, Nove Zamky, Cenkov, NPR Censkovska step, N47°46'09" E18°31'09", 110m, 19-Mai-2014, *Alyssum alyssoides*, sweeping, leg. Krátky,J., det. Krátky,J.
Ceutorhynchus carinatus<br>Gyllenhal, 1837	MK891885 [new]<br>2150-JKR<br>658nt	ZFMK-TIS-23790<br>ZFMK-DNA-0169169583	Slovakia, Nove Zamky, Jursky Chlm, N47°48'04" E18°31'27", 114m, 19-Mai-2014, *Sisymbrium altissimum*, sweeping, leg. Krátky,J., det. Krátky,J.
Ceutorhynchus cf. *chalybaeus*<br>Weise, 1883<br>maybe: Ceutorhynchus chalybaeus timidus	MK891181 [new]<br>1098-JKR<br>658nt	ZFMK-TIS-3434<br>ZFMK-DNA-0100449610	Spain, Andalucia, Sierra de Grazalema, Ubrique env., N36°43'04" W05°27'03", 390m, 21-Apr-2012, *Chameaplium officinalis*, beating, leg. Krátky,J., det. Krátky,J.
Ceutorhynchus cf. *contractus*<br>formerly: *C. contractus* - Resyn!<br>Krátky in. litt	MK891497 [new]<br>1581-JKR<br>658nt	ZFMK-TIS-3629<br>ZFMK-DNA-0155635648	Morocco, Haut Atlas, Tizi N of Test, N30°52'22" W08°21'27", 2092m, 17-Apr-2013, *?Arabis*, individual collecting by hand, leg. Krátky,J., det. Krátky,J.
Ceutorhynchus cf. *erysimi*<br>formerly: *C. erysimi* - Resyn!<br>Krátky in. litt	MK891496 [new]<br>1580-JKR<br>658nt	ZFMK-TIS-3628<br>ZFMK-DNA-0155635649	Morocco, Haut Atlas, Tizi N of Test, N30°52'22" W08°21'27", 2092m, 17-Apr-2013, *Erysimum*, individual collecting by hand, leg. Krátky,J., det. Krátky,J.
Ceutorhynchus cf. *leucorhamma*<br>det. unc.	MK891173 [new]<br>1086-JKR<br>658nt	ZFMK-TIS-3422<br>ZFMK-DNA-0100449619	Spain, Andalucia, 8 km W of Espera, N36°53'17" W05°53'47", 50m, 17-Apr-2012, *yellow flowering Brassicaceae*, sweeping, leg. Krátky,J., det. Krátky,J.
Ceutorhynchus cf. *sardeanensis*	KC783872 [new]<br>136-PST<br>576nt	ZFMK-TIS-2D100440291<br>ZFMK-DNA-0100438179	Morocco, S of Berkane, N of Aïn-es-Sfa, Beni Snassen Mts., N34°49'54" W02°08'49", 985m, 13-Mai-2011, beating, leg. Stüben,P., det. Krátky,J.
Ceutorhynchus chalybaeus<br>Germar, 1824	KC784234 [new]<br>IT-0001w<br>645nt	ZFMK-TIS-2D100446111<br>ZFMK-DNA-100437874	Italy, Sardinia Isl. East, W of Siniscola, Monte Albo, N40°33'37" E09°38'01", 778m, 26-Sep-2010, *Quercus ilex*, cliff, limestone, leg. Stüben,P., det. Stüben,P.
Ceutorhynchus chalybaeus<br>Germar, 1824	MK891764 [new]<br>2020-JKR<br>658nt	ZFMK-TIS-23660<br>ZFMK-DNA-0169170490	Italy, Sicilia Isl., Catania, Nicolosi, Monti Monpilieri, N37°36'20" E15°01'19", 670m, 20-Apr-2014, *Isatis tinctoria*, sweeping, leg. Krátky,J., det. Krátky,J.
Ceutorhynchus chalybaeus<br>Germar, 1824	MK891898 [new]<br>2164-JKR<br>658nt	ZFMK-TIS-23804<br>ZFMK-DNA-0169169578	Slovakia, Komarno, Marcelova, PR Masan, N47°46'10.5" E18°19'06", 121m, 21-Mai-2014, *Sisymbrium altissimum*, sweeping, leg. Krátky,J., det. Krátky,J.
Ceutorhynchus chalybaeus<br>Germar, 1824	MK892153 [new]<br>2441-JKR<br>658nt	ZFMK-TIS-26018<br>ZFMK-DNA-0171600663	Slovakia, Nove Zamky, Imel, PR Liscie diery, N47°55'17" E18°09'37", 114m, 20-Sep-2014, *Berteroa incana*, collecting by hand, leg. Krátky,J., det. Krátky,J.
Ceutorhynchus chlorophanus<br>Rouget, 1857	KC783929 [new]<br>344-JKR<br>658nt	ZFMK-TIS-20130<br>ZFMK-DNA-0100438739	Slovakia, Komarno, Cenkov, N47°46'8.052" E18°31'18.977", 105m, 21-Mai-2011, *Erysimum* sp., sweeping, leg. Krátky,J., det. Krátky,J.
Ceutorhynchus chlorophanus<br>Rouget, 1857	MK892236 [new]<br>2534-PSP<br>658nt	ZFMK-TIS-23506<br>ZFMK-DNA-0171661728	Slovakia, Nitra, SW of Buc, N47°50'49" E18°25'10", 110m, 18-Mai-2014, *Erysimum canum*, leg. Sprick,P., det. Sprick,P.
Ceutorhynchus coarctatus<br>Gyllenhal, 1837	MK891891 [new]<br>2156-JKR<br>658nt	ZFMK-TIS-23796<br>ZFMK-DNA-0169169570	Slovakia, Nove Zamky, Jursky Chlm, N47°48'04" E18°31'27", 114m, 19-Mai-2014, *Sisymbrium altissimum*, sweeping, leg. Krátky,J., det. Krátky,J.

**Name**<br>**Authority**<br>**Additional Information**	**GenBank Acc No (Ref.)**<br>**Specimen ID**<br>**Sequence Length**	**ZFMK Tissue ID**<br>**ZFMK DNA Sample ID**<br>**(SDEI DNA Sample ID)**	**Locality, GPS, Collection Date, Plant, Collector, Identifier**
Ceutorhynchus cochleariae<br>(Gyllenhal, 1813)	MK892002 [new]<br>2274-JKR<br>658nt	ZFMK-TIS-24205<br>ZFMK-DNA-0169170988	Austria, Styria, Hochschwab, Stallmauer, N47°35'29" E15°02'20", 1260m, 27-Jun-2014, *Dentaria enneaphyllos*, collecting by hand, leg. Krátky,J., det. Krátky,J.
Ceutorhynchus cochleariae<br>(Gyllenhal, 1813)	MK891188 [new]<br>1105-JKR<br>658nt	ZFMK-TIS-3441<br>ZFMK-DNA-0100449140	Czech Republic, Bohemia, Roztoky, N50°01'05" E13°52'18", 368m, 30-Apr-2012, *Cardaminopsis / Capsella*, beating, leg. Krátky,J., det. Krátky,J.
Ceutorhynchus cochleariae<br>(Gyllenhal, 1813)	MK891231 [new]<br>1151-JKR<br>658nt	ZFMK-TIS-3487<br>ZFMK-DNA-0100449557	Czech Republic, Bohemia, Hodesovice, PR Mazurova louka, N50°09'41" E15°55'43", 270m, 20-Mai-2012, *Cardamine pratensis*, sweeping, leg. Krátky,J., det. Krátky,J.
Ceutorhynchus cochleariae<br>(Gyllenhal, 1813)	MK891093 [new]<br>997-PSP<br>658nt	ZFMK-TIS-3142<br>ZFMK-DNA-0100426964	Germany, Lower Saxony (NI), Diekholzen, Lkr. Hildesheim, N52°05'04" E09°55'17", 152m, 01-Mai-2012, *Cardamine pratensis*, forest of Hildesheim, dipnet, leg. Sprick,P., det. Sprick,P.
Ceutorhynchus cochleariae<br>(Gyllenhal, 1813)	MK892271 [new]<br>2571-PSP<br>658nt	ZFMK-TIS-23545<br>ZFMK-DNA-0171661988	Germany, Lower Saxony (NI), Harz National Park, Feuersteinwiesen, N51°45'30" E10°41'29", 614m, 11-Jun-2014, *Cardaminopsis halleri*, leg. Sprick,P., det. Sprick,P.
Ceutorhynchus cochleariae<br>(Gyllenhal, 1813)	MK891238 [new]<br>1158-JKR<br>658nt	ZFMK-TIS-3494<br>ZFMK-DNA-0100449547	Hungary, Jasz-Nagykun-Szolnok megye, Tiszaroff, N47°21'55" E20°27'32", 87m, 25-Mai-2012, sweeping, leg. Krátky,J., det. Krátky,J.
Ceutorhynchus cochleariae<br>(Gyllenhal, 1813)	MK890986 [new]<br>878-CBR<br>658nt	ZFMK-TIS-3791<br>ZFMK-DNA-0100449129	Luxembourg, 10 km E of Luxembourg, 2 km SE of Mensdorf, N49°38'18" E06°19'04", 300m, 09-Apr-2011, *Cardamine pratense*, sweeping, leg. Braunert,C., det. Braunert,C.
Ceutorhynchus cochleariae<br>(Gyllenhal, 1813)	MK890853 [new]<br>390-JKR<br>655nt	ZFMK-TIS-20176<br>ZFMK-DNA-0100449052	Slovakia, Tatry Mts., Belanske Tatry Mts., Dolina Siedmich prame?ov, N49°13'37.86" E20°16'30.6", 1500m, 10-Sep-2011, *Cardaminopsis* sp., beating, leg. Krátky,J., det. Krátky,J.
Ceutorhynchus cochleariae<br>(Gyllenhal, 1813)	MK891372 [new]<br>1423-JKR<br>658nt	ZFMK-TIS-4192<br>ZFMK-DNA-0155628509	Slovakia, Vysoke Tatry Mts., Dolina siedmich prame?ov, N49°13'38" E20°16'34", 1448m, 30-Jun-2013, *Cardamine* sp., sweeping, leg. Krátky,J., det. Krátky,J.
Ceutorhynchus cochleariae<br>(Gyllenhal, 1813)	MK891987 [new]<br>2258-JKR<br>658nt	ZFMK-TIS-23996<br>ZFMK-DNA-0169170538	Slovakia, Tatry Mts., Skalne vrata, N49°13'32" E20°16'37", 1341m, 18-Sep-2014, *Arabis alpina*, collecting by hand, leg. Krátky,J., det. Krátky,J.
Ceutorhynchus coerulescens<br>Gyllenhal, 1837	MK891548 [new]<br>1638-JKR<br>658nt	ZFMK-TIS-3686<br>ZFMK-DNA-0155635588	Czech Republic, Moravia, Oblekovice, Naceraticky kopec, N48°50'09" E16°05'45", 256m, 08-Jun-2013, *Lepidium campestre*, individual collecting by hand, leg. Krátky,J., det. Krátky,J.
Ceutorhynchus coerulescens<br>Gyllenhal, 1837	MK891919 [new]<br>2187-JKR<br>658nt	ZFMK-TIS-23925<br>ZFMK-DNA-0169170611	Czech Republic, Moravia, Cernin, N48°59'30" E16°00'57", 350m, 01-Mai-2014, *Lepidium campestre*, collecting by hand, leg. Stejskal,R., det. Stejskal,R.
Ceutorhynchus constrictus<br>(Marsham, 1802)	MK890839 [new]<br>304-JKR<br>658nt	ZFMK-TIS-20090<br>ZFMK-DNA-0100438950	Czech Republic, Bohemia or., Hradec Kralove, Fararstvi, N50°11'26.901" E15°48'57.048", 230m, 06-Mai-2011, *Alliaria petiolata*, beating, leg. Krátky,J., det. Krátky,J.
Ceutorhynchus constrictus<br>(Marsham, 1802)	MK891772 [new]<br>2029-JKR<br>658nt	ZFMK-TIS-23669<br>ZFMK-DNA-0169170472	Czech Republic, Bohemia, Kosatky, N50°19'23" E14°39'58", 218m, 02-Mai-2014, *Alliaria petiolata*, sweeping, leg. Krátky,J., det. Krátky,J.
Ceutorhynchus constrictus<br>(Marsham, 1802)	MK890829 [new]<br>274-PSP<br>658nt	ZFMK-TIS-2D100439097<br>ZFMK-DNA-0100439769	Germany, Lower Saxony (NI), Nordstemmen, Lkr. Hildesheim, N52°09'00" E09°47'00", 72m, 17-Mai-2011, *Alliaria petiolata*, garden, beating, leg. Sprick,P., det. Sprick,P.

**Name**<br>**Authority**<br>**Additional Information**	**GenBank Acc No (Ref.)**<br>**Specimen ID**<br>**Sequence Length**	**ZFMK Tissue ID**<br>**ZFMK DNA Sample ID**<br>**(SDEI DNA Sample ID)**	**Locality, GPS, Collection Date, Plant, Collector, Identifier**
Ceutorhynchus constrictus<br>(Marsham, 1802)	MK892193 [new]<br>2488-PSP<br>658nt	ZFMK-TIS-23460<br>ZFMK-DNA-0171661778	Germany, Saxony-Anhalt (ST), Harz National Park, Ilsenburg, Köhlerholz, N51°53'05" E10°40'12", 290m, 24-Apr-2014, *Alliaria petiolata*, dipnet, leg. Sprick,P., det. Sprick,P.
Ceutorhynchus contractus<br>(Marsham, 1802)	MK892014 [new]<br>2287-JKR<br>658nt	ZFMK-TIS-24218<br>ZFMK-DNA-0169170978	Austria, Styria, Hochschwab, Spitzkögel, N47°35'27" E15°01'26", 1659m, 27-Jun-2014, *Arabis alpina*, collecting by hand, leg. Krátky,J., det. Krátky,J.
Ceutorhynchus contractus<br>(Marsham, 1802)	KC783947 [new]<br>306-JKR<br>658nt	ZFMK-TIS-20092<br>ZFMK-DNA-0100438768	Czech Republic, Bohemia or., Hradec Kralove, Placky, N50°13'48.497" E15°49'25.886", 230m, 09-Mai-2011, *Berteroa incana*, beating, leg. Krátky,J., det. Krátky,J.
Ceutorhynchus contractus<br>(Marsham, 1802)	KC784111 [new]<br>500-RST<br>658nt	ZFMK-TIS-20285<br>ZFMK-DNA-0100448479	Czech Republic, Bohemia mer., Slavonice env., N48°59'32.46" E15°21'46.96", 500m, 14-Mai-2011, sweeping, leg. Stejskal,R., det. Stejskal,R.
Ceutorhynchus contractus<br>(Marsham, 1802)	MK891198 [new]<br>1115-JKR<br>658nt	ZFMK-TIS-3451<br>ZFMK-DNA-0100449600	Czech Republic, Bohemia, Hradec Kralove, Fararstvi, N50°11'33" E15°48'10", 229m, 05-Mai-2012, *Erysimum cheiranthoides*, beating, leg. Krátky,J., det. Krátky,J.
Ceutorhynchus contractus<br>(Marsham, 1802)	MK891798 [new]<br>2057-JKR<br>658nt	ZFMK-TIS-23697<br>ZFMK-DNA-0169170444	Czech Republic, Bohemia, Hradec Kralove, Kocianovice, N50°13'16" E15°52'51", 237m, 06-Mai-2014, *Cardaria draba*, collecting by hand, leg. Krátky,J., det. Krátky,J.
Ceutorhynchus contractus<br>(Marsham, 1802)	MK891802 [new]<br>2061-JKR<br>658nt	ZFMK-TIS-23701<br>ZFMK-DNA-0169170435	Czech Republic, Bohemia, Hradec Kralove, Kocianovice, N50°13'16" E15°52'51", 237m, 06-Mai-2014, *Berteroa incana*, collecting by hand, leg. Krátky,J., det. Krátky,J.
Ceutorhynchus contractus<br>(Marsham, 1802)	MK891803 [new]<br>2062-JKR<br>658nt	ZFMK-TIS-23702<br>ZFMK-DNA-0169170436	Czech Republic, Bohemia, Hradec Kralove, Kocianovice, N50°13'16" E15°52'51", 237m, 06-Mai-2014, *Arabidopsis thaliana*, collecting by hand, leg. Krátky,J., det. Krátky,J.
Ceutorhynchus contractus<br>(Marsham, 1802)	MK891804 [new]<br>2063-JKR<br>658nt	ZFMK-TIS-23703<br>ZFMK-DNA-0169170437	Czech Republic, Bohemia, Hradec Kralove, Kocianovice, N50°13'16" E15°52'51", 237m, 06-Mai-2014, *Lepidium campestre*, collecting by hand, leg. Krátky,J., det. Krátky,J.
Ceutorhynchus contractus<br>(Marsham, 1802)	MK891806 [new]<br>2065-JKR<br>658nt	ZFMK-TIS-23705<br>ZFMK-DNA-0169170439	Czech Republic, Bohemia, Hradec Kralove, Kocianovice, N50°13'16" E15°52'51", 237m, 06-Mai-2014, *Lepidium campestre*, collecting by hand, leg. Krátky,J., det. Krátky,J.
Ceutorhynchus contractus<br>(Marsham, 1802)	KC784007 [new]<br>238-PSP<br>645nt	ZFMK-TIS-2D100439132<br>ZFMK-DNA-0100439804	Germany, Bavaria (BY), Buckenhof/Uttenreuth, Lkr. Erlangen-Höchstadt, N49°35'20" E11°05'17", 474m, 29-Apr-2011, *Arabidopsis thaliana*, sand field, dipnet, leg. Sprick,P., det. Sprick,P.
Ceutorhynchus contractus<br>(Marsham, 1802)	KC784244 [new]<br>IT-0011w<br>648nt	ZFMK-TIS-2D100446561<br>ZFMK-DNA-0100437857	Italy, Sardinia Isl. East, S of Dorgali, Genna Silana, N40°09'30" E09°30'30", 1017m, 27-Sep-2010, *Quercus ilex, Ficus*, cliff, leg. Stüben,P., det. Stüben,P.
Ceutorhynchus contractus<br>(Marsham, 1802)	KC784248 [new]<br>IT-0015w<br>648nt	ZFMK-DNA-0100437861	Italy, Sardinia Isl., NE of Aritzo, Gennargentu, Mt. di Iscudu, N40°01'10" E09°16'39", 1511m, 30-Sep-2010, *alder, moss*, creek, leg. Stüben,P., det. Stüben,P.
Ceutorhynchus contractus<br>(Marsham, 1802)	MK891274 [new]<br>1218-JKR<br>658nt	ZFMK-TIS-3554<br>ZFMK-DNA-0100426217	Romania, Maramures, Rodna Mts., Gargalau Mt., N47°35'15" E24°48'27", 1549m, 07-Sep-2012, *Arabis alpina*, sieving, leg. Krátky,J., det. Krátky,J.
Ceutorhynchus contractus<br>(Marsham, 1802)	KC784171 [new]<br>391-JKR<br>658nt	ZFMK-TIS-20177<br>ZFMK-DNA-0100449051	Slovakia, Tatry Mts., Belanske Tatry Mts., Dolina Siedmich prame?ov, N49°13'37.86" E20°16'30.6", 1500m, 10-Sep-2011, *Arabis alpina*, beating, leg. Krátky,J., det. Krátky,J.

**Name**<br>**Authority**<br>**Additional Information**	**GenBank Acc No (Ref.)**<br>**Specimen ID**<br>**Sequence Length**	**ZFMK Tissue ID**<br>**ZFMK DNA Sample ID**<br>**(SDEI DNA Sample ID)**	**Locality, GPS, Collection Date, Plant, Collector, Identifier**
Ceutorhynchus contractus<br>(Marsham, 1802)	MK891374 [new]<br>1426-JKR<br>658nt	ZFMK-TIS-4195<br>ZFMK-DNA-0155628512	Slovakia, Vysoke Tatry Mts., Skalne vrata, N49°13'39" E20°16'42", 1512m, 30-Jun-2013, *Erysimum* , sieving, leg. Krátky,J., det. Krátky,J.
Ceutorhynchus contractus<br>(Marsham, 1802)	MK891861 [new]<br>2123-JKR<br>658nt	ZFMK-TIS-23763<br>ZFMK-DNA-0169169610	Slovakia, Nitra, Zobor Mt., N48°21'08.5 E18°05'41", 427m, 24-Mai-2014, *Erysimum* cf. *odoratum*, sieving, leg. Krátky,J., det. Krátky,J.
Ceutorhynchus contractus<br>(Marsham, 1802)	MK891981 [new]<br>2252-JKR<br>658nt	ZFMK-TIS-23990<br>ZFMK-DNA-0169170532	Slovakia, Kezmarok, Vysoke Tatry Mts., Bielovodska dolina valley, N49°14'59" E20°6'3.29", 994m, 22-Jun-2014, *Arabis alpina*, leg. Benedikt,S., det. Benedikt,S.
Ceutorhynchus contractus<br>(Marsham, 1802)	MK891986 [new]<br>2257-JKR<br>658nt	ZFMK-TIS-23995<br>ZFMK-DNA-0169170537	Slovakia, Zvolen, Hronska Dubrava, NPR Boky, N48°33'58" E19°01'10.5", 472m, 12-Okt-2014, *Barbaraea, Alliaria, Arabidopsis*, sieving, leg. Krátky,J., det. Krátky,J.
Ceutorhynchus contractus<br>(Marsham, 1802)	MK892248 [new]<br>2547-PSP<br>658nt	ZFMK-TIS-23521<br>ZFMK-DNA-0171662012	Slovakia, Nitra, Cenkov, N47°46'07" E18°31'11", 109m, 19-Mai-2014, *Cardaria draba*, leg. Sprick,P., det. Sprick,P.
Ceutorhynchus descurainiae<br>Stüben, 2016<br>**Paratype (DNAtype)**	KC783780 [13]<br>71-PST_2G2011<br>658nt	ZFMK-TIS-2D100446984<br>ZFMK-DNA-0100438056	Spain, Canary Islands, La Gomera, E of Hermigua, PN of Majona, El Palmar, N28°09'31" W17°09'38", 584m, 06-Feb-2011, *Descuraina millefolia*, beating, leg. Stüben,P., det. Stüben,P.
Ceutorhynchus descurainiae<br>Stüben, 2016<br>**Paratype (DNAtype)**	KU932049 [27]<br>2710-PST_23860<br>658nt	ZFMK-TIS-23860<br>ZFMK-DNA-0171661289	Spain, Canary Islands, La Gomera, E of Hermigua, Parque Natural de Majona near Casas del Palmar, N28°09'33" W17°09'36", 554m, 22-Dez-2014, beating, leg. Stüben,P., det. Stüben,P.
Ceutorhynchus descurainiae<br>Stüben, 2016<br>**Paratype (DNAtype)**	KC784315 [27]<br>764-PST_91T2012<br>658nt	ZFMK-TIS-3101<br>ZFMK-DNA-0100448123	Spain, Canary Islands, Tenerife, Anaga Mts., Afur, N28°33'10" W16°14'59", 303m, 26-Feb-2012, *Desurainia millefolia*, beating, leg. Stüben,P., det. Stüben,P.
Ceutorhynchus dubius<br>C. Brisout de Barneville, 1883	MK890854 [new]<br>392-JKR<br>658nt	ZFMK-TIS-20178<br>ZFMK-DNA-0100449039	Czech Republic, Bohemia or., cerna u Bohdance, N50°03'24" E15°40'59", 230m, 18-Sep-2011, *Berteroa incana*, sifting, leg. Krátky,J., det. Krátky,J.
Ceutorhynchus erysimi<br>(Fabricius, 1787)	MK891991 [new]<br>2262-JKR<br>658nt	ZFMK-TIS-24192<br>ZFMK-DNA-0169171004	Austria, Lower Austria, Schneeberg Mt., N47°45'25" E15°49'45", 1779m, 29-Jun-2014, *Arabis alpina*, collecting by hand, leg. Krátky,J., det. Krátky,J.
Ceutorhynchus erysimi<br>(Fabricius, 1787)	MK891235 [new]<br>1155-JKR<br>658nt	ZFMK-TIS-3491<br>ZFMK-DNA-0100449128	Czech Republic, Bohemia, Hradec Kralove, Brezhrad, N50°10'17" E15°47'32", 230m, 23-Mai-2012, *Berteroa incana*, sweeping, leg. Krátky,J., det. Krátky,J.
Ceutorhynchus erysimi<br>(Fabricius, 1787)	MK891812 [new]<br>2072-JKR<br>658nt	ZFMK-TIS-23712<br>ZFMK-DNA-0169169661	Czech Republic, Bohemia, Pohrebacka, N50°09'15" E15°46'42", 226m, 24-Jun-2014, *Erysimum cheiranthoides*, sweeping, leg. Krátky,J., det. Krátky,J.
Ceutorhynchus erysimi<br>(Fabricius, 1787)	MK891828 [new]<br>2089-JKR<br>658nt	ZFMK-TIS-23729<br>ZFMK-DNA-0169169647	Czech Republic, Moravia, Bucovice, PR Sevy, N49°08'05" E16°58'19", 249m, 09-Mai-2014, *Diplotaxus tenuifolia*, sweeping, leg. Krátky,J., det. Krátky,J.
Ceutorhynchus erysimi<br>(Fabricius, 1787)	MK890827 [new]<br>260-PSP<br>658nt	ZFMK-TIS-2D100439106<br>ZFMK-DNA-0100439778	Germany, Lower Saxony (NI), Hannover, Stöcken, Leine wetland, N52°24'24" E09°39'09", 45m, 14-Mai-2011, *Capsella bursa-pastoris*, roadside, dipnet, leg. Sprick,P., det. Sprick,P.
Ceutorhynchus erysimi<br>(Fabricius, 1787)	MK892045 [new]<br>2321-JKR<br>658nt	ZFMK-TIS-24252<br>ZFMK-DNA-0169170939	Slovakia, Tatry Mts., Dolina Siedmich prame?ov, N49°13'30" E20°16'24", 1404m, 05-Jul-2014, *Cardamine* sp., collecting by hand, leg. Krátky,J., det. Krátky,J.

**Name**<br>**Authority**<br>**Additional Information**	**GenBank Acc No (Ref.)**<br>**Specimen ID**<br>**Sequence Length**	**ZFMK Tissue ID**<br>**ZFMK DNA Sample ID**<br>**(SDEI DNA Sample ID)**	**Locality, GPS, Collection Date, Plant, Collector, Identifier**
Ceutorhynchus erysimi<br>(Fabricius, 1787)	MK892050 [new]<br>2326-JKR<br>658nt	ZFMK-TIS-24257<br>ZFMK-DNA-0169170944	Slovakia, Tatry Mts., Bujaci vrch, N49°14'10" E20°14'27", 1914m, 06-Jul-2014, *Cardaminopsis* sp., collecting by hand, leg. Krátky,J., det. Krátky,J.
Ceutorhynchus granulicollis<br>C. G. Thomson, 1865	MK891233 [new]<br>1153-JKR<br>658nt	ZFMK-TIS-3489<br>ZFMK-DNA-0100449107	Czech Republic, Bohemia, Hradec Kralove, Brezhrad, N50°10'45" E15°47'39", 229m, 21-Mai-2012, *Thlaspi arvense*, sweeping, leg. Krátky,J., det. Krátky,J.
Ceutorhynchus hampei<br>C. Brisout de Barneville, 1869	KC783926 [new]<br>349-JKR<br>658nt	ZFMK-TIS-20135<br>ZFMK-DNA-0100438734	Czech Republic, Bohemia or., Hradec Kralove, Placky, N50°13'48.497" E15°49'25.886", 230m, 29-Mai-2011, *Berteroa incana*, beating, leg. Krátky,J., det. Krátky,J.
Ceutorhynchus hampei<br>C. Brisout de Barneville, 1869	MK892075 [new]<br>2351-JKR<br>658nt	ZFMK-TIS-24282<br>ZFMK-DNA-0169170921	Czech Republic, Moravia, Lanzhot, Pohansko, N48°41'27" E16°55'58", 153m, 14-Aug-2014, *Berteroa incana*, sweeping, leg. Krátky,J., det. Krátky,J.
Ceutorhynchus hampei<br>C. Brisout de Barneville, 1869	MK891148 [new]<br>1055-PSP<br>658nt	ZFMK-TIS-3200<br>ZFMK-DNA-0100426804	Germany, Lower Saxony (NI), Braunschweig, Rautheim, Schöppenstedter Tower, N52°15'02" E10°35'33.5", 78m, 13-Jun-2012, *Berteroa incana*, south exposed slope, beating, leg. Sprick,P., det. Sprick,P.
Ceutorhynchus hirtulus<br>Germar, 1824	MK891220 [new]<br>1140-JKR<br>658nt	ZFMK-TIS-3476<br>ZFMK-DNA-0100449577	Slovakia, Komarno, Imel, N47°54'34" E18°09'16", 112m, 11-Mai-2012, sweeping, leg. Krátky,J., det. Krátky,J.
Ceutorhynchus hirtulus<br>Germar, 1824	MK891877 [new]<br>2141-JKR<br>658nt	ZFMK-TIS-23781<br>ZFMK-DNA-0169169603	Slovakia, Komarno, Imel, PR Liscie diery, N47°55'17" E18°09'37", 114m, 18-Mai-2014, *Alyssum alyssoides*, sweeping, leg. Krátky,J., det. Krátky,J.
Ceutorhynchus hutchinsiae<br>Tempere, 1975	KC783914 [new]<br>388-JKR<br>658nt	ZFMK-TIS-20174<br>ZFMK-DNA-0100438706	Slovakia, Tatry Mts., Belanske Tatry Mts., Bujaci vrch, N49°13'50.97" E20°16'15.97", 1850m, 21-Sep-2011, *Biscutella laevigata austriaca*, ex larvae, leg. Krátky,J., det. Krátky,J.
Ceutorhynchus hutchinsiae<br>Tempere, 1975	KC784172 [new]<br>389-JKR<br>658nt	ZFMK-TIS-20175<br>ZFMK-DNA-0100449053	Slovakia, Tatry Mts., Belanske Tatry Mts., Dolina Siedmich prame?ov, N49°13'37.86" E20°16'30.6", 1500m, 10-Sep-2011, *Arabis alpina*, beating, leg. Krátky,J., det. Krátky,J.
Ceutorhynchus hutchinsiae<br>Tempere, 1975	MK892033 [new]<br>2309-JKR<br>658nt	ZFMK-TIS-24240<br>ZFMK-DNA-0169170956	Slovakia, Tatry Mts., Skalne vrata, N49°13'32" E20°16'37", 1341m, 07-Jul-2014, *Arabis alpina*, collecting by hand, leg. Krátky,J., det. Krátky,J.
Ceutorhynchus ignitus<br>Germar, 1824	KC783948 [new]<br>307-JKR<br>658nt	ZFMK-TIS-20093<br>ZFMK-DNA-0100438769	Czech Republic, Bohemia or., Hradec Kralove, Placky, N50°13'48.497" E15°49'25.886", 230m, 09-Mai-2011, *Berteroa incana*, beating, leg. Krátky,J., det. Krátky,J.
Ceutorhynchus ignitus<br>Germar, 1824	MK891094 [new]<br>998-PSP<br>658nt	ZFMK-TIS-3143<br>ZFMK-DNA-0100426220	Germany, Lower Saxony (NI), Braunschweig, Lindenbergsiedlung, N52°14'43" E10°33'13", 80m, 02-Mai-2012, *Berteroa incana*, railway area, dipnet, leg. Sprick,P., det. Sprick,P.
Ceutorhynchus inaffectatus<br>Gyllenhal, 1837	MK891936 [new]<br>2204-JKR<br>658nt	ZFMK-TIS-23942<br>ZFMK-DNA-0169170580	Slovakia, Nitra District, Nitra, Zobor, N48°21'08.5 E18°05'41", 400m, 24-Mai-2014, *Hesperis tristis*, collecting by hand, leg. Stejskal,R., det. Stejskal,R.
Ceutorhynchus intersetosus<br>Weise, 1883	KC783865 [new]<br>137-PST<br>595nt	ZFMK-TIS-2D100440279<br>ZFMK-DNA-0100438167	Morocco, S of Berkane, N of Aïn-es-Sfa, Beni Snassen Mts., N34°49'54" W02°08'49", 985m, 13-Mai-2011, beating, leg. Stüben,P., det. Behne,L.
Ceutorhynchus intersetosus<br>Weise, 1883	MK891176 [new]<br>1089-JKR<br>658nt	ZFMK-TIS-3425<br>ZFMK-DNA-0100449622	Spain, Andalucia, 8 km W of Espera, N36°53'17" W05°53'47", 50m, 17-Apr-2012, *yellow flowering Brassicaceae*, sweeping, leg. Krátky,J., det. Krátky,J.

**Name**<br>**Authority**<br>**Additional Information**	**GenBank Acc No (Ref.)**<br>**Specimen ID**<br>**Sequence Length**	**ZFMK Tissue ID**<br>**ZFMK DNA Sample ID**<br>**(SDEI DNA Sample ID)**	**Locality, GPS, Collection Date, Plant, Collector, Identifier**
Ceutorhynchus jucundus<br>Colonnelli, 2005	KC783783 [13]<br>74-PST<br>657nt	ZFMK-TIS-2D100446904<br>ZFMK-DNA-0100438046	Spain, Canary Islands, La Gomera, El Cedro, Mirador de El Bailadero, N28°07'22" W17°12'29", 1045m, 08-Feb-2011, *Erysimum bicolor*, beating, leg. Stüben,P., det. Behne,L.
Ceutorhynchus leprieuri<br>C. Brisout de Barneville, 1881	KC784239 [new]<br>IT-0006w<br>648nt	ZFMK-DNA-0100437869	Italy, Sardinia Isl. East, SW of Siniscola, Monte Albo, N40°32'07" E09°36'13", 748m, 26-Sep-2010, *Quercus ilex*, limestone, leg. Stüben,P., det. Stüben,P.
Ceutorhynchus leprieuri<br>C. Brisout de Barneville, 1881	KC784260 [new]<br>IT-0030w<br>652nt	ZFMK-TIS-2D100446547<br>ZFMK-DNA-0100437846	Italy, Sardinia Isl. East, Oliena, Supramonte, N40°15'23" E09°25'16", 966m, 05-Okt-2010, *Quercus*, leg. Stüben,P., det. Stüben,P.
Ceutorhynchus leucorhamma<br>Rosenhauer, 1856	MK891175 [new]<br>1088-JKR<br>658nt	ZFMK-TIS-3424<br>ZFMK-DNA-0100449621	Spain, Andalucia, 8 km W of Espera, N36°53'17" W05°53'47", 50m, 17-Apr-2012, *yellow flowering Brassicaceae*, sweeping, leg. Krátky,J., det. Krátky,J.
Ceutorhynchus libertorum<br>Colonnelli, 2005	MK890848 [new]<br>356-JKR<br>658nt	ZFMK-TIS-20142<br>ZFMK-DNA-0100438936	Greece, Thessalia, Ossa Mts., N39°50'03.37" E22°41'04.86", 1050m, 05-Jun-2011, *Aurinia saxatilis orientalis*, beating, leg. Krátky,J., det. Krátky,J.
Ceutorhynchus liliputanus<br>Schultze, 1898	MK891236 [new]<br>1156-JKR<br>658nt	ZFMK-TIS-3492<br>ZFMK-DNA-0100449108	Slovakia, Komarno, Virt, PR Masan, N47°46'10" E18°19'10", 121m, 24-Mai-2012, *Alyssum montanum gmelini*, sweeping, leg. Krátky,J., det. Krátky,J.
Ceutorhynchus lukesi<br>Tyl, 1914	MK891187 [new]<br>1104-JKR<br>658nt	ZFMK-TIS-3440<br>ZFMK-DNA-0100449152	Czech Republic, Bohemia, Nezabudice, PR Nezabudicke skaly, N50°01'23" E13°50'45", 346m, 30-Apr-2012, *Aurinia saxatilis arduini*, beating, leg. Krátky,J., det. Krátky,J.
Ceutorhynchus lukesi<br>Tyl, 1914	KC784110 [new]<br>501-RST<br>643nt	ZFMK-TIS-20286<br>ZFMK-DNA-0100448478	Romania, Caras-Severin, Sfanta Elena env., N44°41'59.04" E21°41'55.07", 450m, 29-Apr-2011, *Alyssum murale*, sweeping, leg. Stejskal,R., det. Stejskal,R.
Ceutorhynchus merkli<br>Korotyaev, 2001	MK891205 [new]<br>1123-JKR<br>658nt	ZFMK-TIS-3459<br>ZFMK-DNA-0100449585	Slovakia, Nove Zamky, Tvrdosovce, N48°06'09" E18°02'08", 110m, 10-Mai-2012, *Cardaria draba*, sweeping, leg. Krátky,J., det. Krátky,J.
Ceutorhynchus moraviensis<br>(Dieckmann, 1966)	MK891375 [new]<br>1427-JKR<br>658nt	ZFMK-TIS-4196<br>ZFMK-DNA-0155628513	Czech Republic, Moravia, Pavlov, D?vin Mt., N48°52'28" E16°39'37", 401m, 06-Jul-2013, *Sysimbrium strictissimum*, beating, leg. Stanek,T., det. Krátky,J.
Ceutorhynchus napi<br>Gyllenhal, 1837	MK891509 [new]<br>1595-JKR<br>658nt	ZFMK-TIS-3643<br>ZFMK-DNA-0155635641	Czech Republic, Moravia, Poudrany - step, N48°56'51" E16°38'13", 260m, 01-Mai-2013, *Sisymbrium loesseli*, beating, leg. Krátky,J., det. Krátky,J.
Ceutorhynchus nevadensis<br>A. Hoffmann, 1960	MK891249 [new]<br>1180-JKR<br>658nt	ZFMK-TIS-3516<br>ZFMK-DNA-0100426097	Spain, Andalucia, Sierra Nevada, Canadillas, N37°07'27" W03°24'39", 1922m, 10-Jun-2012, *Hormatophylla spinosa*, beating, leg. Krátky,J., det. Krátky,J.
Ceutorhynchus nevadensis<br>A. Hoffmann, 1960	MK891329 [new]<br>1351-PST<br>658nt	ZFMK-TIS-4167<br>ZFMK-DNA-0100426124	Spain, Jaen, SE of Mancha Real, Sierra Almaden (El Almaden), N37°44'09" W03°31'26", 1963m, 15-Mai-2013, *Euphorbia nicaeensis, Bupleurum spinosum*, sieving, leg. Stüben,P. & Schütte,A., det. Stüben,P.
Ceutorhynchus nigritulus<br>Schultze, 1897	MK891883 [new]<br>2148-JKR<br>658nt	ZFMK-TIS-23788<br>ZFMK-DNA-0169169585	Slovakia, Nove Zamky, Cenkov, NPR Censkovska step, N47°46'09" E18°31'09", 110m, 19-Mai-2014, *Arabis* cf.*hirsuta*, sweeping, leg. Krátky,J., det. Krátky,J.
Ceutorhynchus niyazii<br>A. Hoffmann, 1957	MK891118 [new]<br>1023-PSP<br>658nt	ZFMK-TIS-3168<br>ZFMK-DNA-0100426953	Germany, Lower Saxony (NI), Braunschweig, Lindenbergsiedlung, N52°14'43" E10°33'13", 80m, 14-Mai-2012, *Sisymbrium altissimum*, railway area, beating, leg. Sprick,P., det. Sprick,P.

**Name**<br>**Authority**<br>**Additional Information**	**GenBank Acc No (Ref.)**<br>**Specimen ID**<br>**Sequence Length**	**ZFMK Tissue ID**<br>**ZFMK DNA Sample ID**<br>**(SDEI DNA Sample ID)**	**Locality, GPS, Collection Date, Plant, Collector, Identifier**
Ceutorhynchus niyazii<br>A. Hoffmann, 1957	MK891211 [new]<br>1130-JKR<br>658nt	ZFMK-TIS-3466<br>ZFMK-DNA-0100449567	Slovakia, Komarno, Imel, N47°54'34" E18°09'16", 112m, 11-Mai-2012, *Sisymbrium orientale*, beating, leg. Krátky,J., det. Krátky,J.
Ceutorhynchus niyazii<br>A. Hoffmann, 1957	MK891892 [new]<br>2157-JKR<br>658nt	ZFMK-TIS-23797<br>ZFMK-DNA-0169169571	Slovakia, Nove Zamky, Jursky Chlm, N47°48'04" E18°31'27", 114m, 19-Mai-2014, *Sisymbrium altissimum*, sweeping, leg. Krátky,J., det. Krátky,J.
Ceutorhynchus niyazii<br>A. Hoffmann, 1957	MK892257 [new]<br>2557-PSP<br>658nt	ZFMK-TIS-23531<br>ZFMK-DNA-0171661993	Slovakia, Nitra, Muzla, N47°48'00" E18°31'55", 114m, 19-Mai-2014, *Sisymbrium altissimum*, leg. Sprick,P., det. Sprick,P.
Ceutorhynchus obstrictus<br>(Marsham, 1802)	MK891989 [new]<br>2260-JKR<br>658nt	ZFMK-TIS-24190<br>ZFMK-DNA-0169171006	Austria, Lower Austria, Sieding, N47°45'47" E15°57'28", 526m, 29-Jun-2014, *Sysimbrium strictissimum*, collecting by hand, leg. Krátky,J., det. Krátky,J.
Ceutorhynchus obstrictus<br>(Marsham, 1802)	MK891990 [new]<br>2261-JKR<br>658nt	ZFMK-TIS-24191<br>ZFMK-DNA-0169171005	Austria, Lower Austria, Schneeberg Mt., N47°45'25" E15°49'45", 1779m, 29-Jun-2014, *Arabis alpina*, collecting by hand, leg. Krátky,J., det. Krátky,J.
Ceutorhynchus obstrictus<br>(Marsham, 1802)	MK891186 [new]<br>1103-JKR<br>658nt	ZFMK-TIS-3439<br>ZFMK-DNA-0100449605	Czech Republic, Bohemia, Nezabudice, PR Nezabudicke skaly, N50°01'23" E13°50'45", 346m, 30-Apr-2012, *Aurinia saxatilis arduini*, beating, leg. Krátky,J., det. Krátky,J.
Ceutorhynchus obstrictus<br>(Marsham, 1802)	MK891234 [new]<br>1154-JKR<br>658nt	ZFMK-TIS-3490<br>ZFMK-DNA-0100449082	Czech Republic, Bohemia, Hradec Kralove, Brezhrad, N50°10'45" E15°47'39", 229m, 21-Mai-2012, *Thlaspi arvense*, sweeping, leg. Krátky,J., det. Krátky,J.
Ceutorhynchus obstrictus<br>(Marsham, 1802)	MK891264 [new]<br>1201-JKR<br>614nt	ZFMK-TIS-3537<br>ZFMK-DNA-0100426083	Czech Republic, Bohemia, Hradec Kralove, Placky, N50°13'45" E15°49'23", 233m, 24-Jul-2012, *Berteroa incana*, beating, leg. Krátky,J., det. Krátky,J.
Ceutorhynchus obstrictus<br>(Marsham, 1802)	MK892071 [new]<br>2347-JKR<br>658nt	ZFMK-TIS-24278<br>ZFMK-DNA-0169170917	Czech Republic, Bohemia, Pohrebacka, N50°9'15" E15°46'42", 226m, 24-Jul-2014, *Nasturtium officinale*, collecting by hand, leg. Krátky,J., det. Krátky,J.
Ceutorhynchus obstrictus<br>(Marsham, 1802)	KC784008 [new]<br>239-PSP<br>658nt	ZFMK-TIS-2D100439133<br>ZFMK-DNA-0100439805	Germany, Lower Saxony (NI), Ockensen, Lkr. Hameln-Pyrmont, Ith Mts., N52°01'19" E09°35'07", 290m, 04-Mai-2011, *Alliaria petiolata*, beating, leg. Sprick,P., det. Sprick,P.
Ceutorhynchus obstrictus<br>(Marsham, 1802)	MK890919 [new]<br>787-PST<br>658nt	ZFMK-TIS-3700<br>ZFMK-DNA-0100414304	Germany, Rhineland-Palatinate (RLP), E of Treis-Karden near Pommern, Fellerbachtal (river valley), N50°10'10" E07°13'38", 85m, 09-Jun-2012, *Brassicaceae*, rivulet, beating, leg. Stüben,P., det. Stüben,P.
Ceutorhynchus obstrictus<br>(Marsham, 1802)	MK891412 [new]<br>1474-PSP<br>658nt	ZFMK-TIS-3234<br>ZFMK-DNA-0155633360	Germany, Lower Saxony (NI), Harz, St. Andreasberg, Jordanshöhe, N51°42'58" E10°32'06", 670m, 02-Jul-2012, *Hesperis matronalis*, perennials corridor, beating, leg. Sprick,P., det. Sprick,P.
Ceutorhynchus obstrictus<br>(Marsham, 1802)	MK891204 [new]<br>1122-JKR<br>658nt	ZFMK-TIS-3458<br>ZFMK-DNA-0100449586	Slovakia, Nove Zamky, Tvrdosovce, N48°06'09" E18°02'08", 110m, 10-Mai-2012, *Cardaria draba*, sweeping, leg. Krátky,J., det. Krátky,J.
Ceutorhynchus obstrictus<br>(Marsham, 1802)	MK891219 [new]<br>1139-JKR<br>658nt	ZFMK-TIS-3475<br>ZFMK-DNA-0100449576	Slovakia, Komarno, Imel, N47°54'34" E18°09'16", 112m, 11-Mai-2012, *Descurainia sophia*, beating, leg. Krátky,J., det. Krátky,J.
Ceutorhynchus obstrictus<br>(Marsham, 1802)	MK892261 [new]<br>2561-PSP<br>658nt	ZFMK-TIS-23535<br>ZFMK-DNA-0171661997	Slovakia, Nitra, Muzla, N47°48'00" E18°31'55", 114m, 19-Mai-2014, *Sisymbrium altissimum*, leg. Sprick,P., det. Sprick,P.

**Name**<br>**Authority**<br>**Additional Information**	**GenBank Acc No (Ref.)**<br>**Specimen ID**<br>**Sequence Length**	**ZFMK Tissue ID**<br>**ZFMK DNA Sample ID**<br>**(SDEI DNA Sample ID)**	**Locality, GPS, Collection Date, Plant, Collector, Identifier**
Ceutorhynchus pallidactylus<br>(Marsham, 1802)	MK891988 [new]<br>2259-JKR<br>658nt	ZFMK-TIS-24189<br>ZFMK-DNA-0169171007	Austria, Lower Austria, Sieding, N47°45'47" E15°57'28", 526m, 29-Jun-2014, *Sysimbrium strictissimum*, collecting by hand, leg. Krátky,J., det. Krátky,J.
Ceutorhynchus pallidactylus<br>(Marsham, 1802)	MK892339 [new]<br>2668-PSP<br>658nt	ZFMK-TIS-23818<br>ZFMK-DNA-0171661320	Cyprus, Limassol district, Akrounta, above Germasogeia dam, N34°45'38" E33°04'59", 98m, 07-Apr-2013, *Eruca sativa*, dipnet, leg. Sprick,P., det. Sprick,P.
Ceutorhynchus pallidactylus<br>(Marsham, 1802)	KC784131 [new]<br>657-PSP<br>658nt	ZFMK-TIS-20440<br>ZFMK-DNA-0100448615	Germany, Lower Saxony (NI), Lauenstein, Lkr. Hameln-Pyrmont, Ith Mts., N52°04'09" E09°32'36", 327m, 17-Nov-2011, leaf litter, leaves sifting, leg. Sprick,P., det. Sprick,P.
Ceutorhynchus pallidactylus<br>(Marsham, 1802)	KC783731 [13]<br>3-PST<br>658nt	ZFMK-TIS-2D100446934<br>ZFMK-DNA-0100438001	Spain, Canary Islands, Gran Canaria, Marzagan, Barranco de las Goteras, N28°01'28" W15°27'31", 236m, 01-Dez-2010, *Brassicaceae*, beating, leg. Stüben,P., det. Stüben,P.
Ceutorhynchus pallidactylus<br>(Marsham, 1802)	KC783767 [40]<br>57-PST<br>658nt	ZFMK-TIS-2D100446991<br>ZFMK-DNA-0100438063	Spain, Canary Islands, La Gomera, Hermigua, Las Nuevitas, Barranquillos los Alamos, N28°09'31" W17°11'09", 251m, 13-Dez-2010, *Brassicaceae*, beating, leg. Stüben,P., det. Stüben,P.
Ceutorhynchus pallidactylus<br>(Marsham, 1802)	MK891738 [40]<br>1984-JKR<br>658nt	ZFMK-TIS-23624<br>ZFMK-DNA-0169170517	Spain, Canary Islands, La Palma, San Juan de Puntallana, N28°44'53" W17°44'14", 226m, 08-Feb-2014, *Erucastrum canariense*, sweeping, leg. Krátky,J., det. Krátky,J.
Ceutorhynchus pallidactylus<br>(Marsham, 1802)	MK891653 [40]<br>1833-PST<br>658nt	ZFMK-TIS-26036<br>ZFMK-DNA-0171606133	Portugal, Madeira, Sao Jorge, Praia de S. Jorge, N32°49'59" W16°54'00", 24m, 28-Jun-2014, beating, leg. Stüben,P., det. Stüben,P.
Ceutorhynchus paroliniae<br>Krátky, 2016<br>**Paratype (DNAtype)**	KU366275 [25]<br>2764-JKR_26123<br>658nt	ZFMK-TIS-26213<br>ZFMK-DNA-0171661907	Spain, Canary Islands, Gran Canaria, Ingenio, 3 km W, Barranco de Guayadeque, N27°55'34" W15°27'45", 382m, 19-Feb-2015, *Parolinia platypetala*, beating, leg. Krátky,J., det. Krátky,J.
Ceutorhynchus parvulus<br>C. Brisout de Barneville, 1869	MK891546 [new]<br>1636-JKR<br>658nt	ZFMK-TIS-3684<br>ZFMK-DNA-0155635586	Czech Republic, Bohemia, Hradec Kralove, N50°11'36" E15°48'12", 229m, 31-Mai-2013, *Lepidium campestre*, individual collecting by hand, leg. Krátky,J., det. Krátky,J.
Ceutorhynchus pectoralis<br>Weise, 1895	MK892062 [new]<br>2338-JKR<br>658nt	ZFMK-TIS-24269<br>ZFMK-DNA-0169170927	Czech Republic, Bohemia, Horni Morava, N50°10'15" E16°49'22", 780m, 17-Jul-2014, *Barbaraea vularis*, collecting by hand, leg. Krátky,J., det. Krátky,J.
Ceutorhynchus pectoralis<br>Weise, 1895	MK891559 [new]<br>1653-JKR<br>658nt	ZFMK-TIS-4231<br>ZFMK-DNA-0155630487	Slovakia, Lucenec, Lentvora, Lysec hill, N48°21'00" E19°27'36", 690m, 12-Okt-2013, *Barbarea vulgaris*, individual collecting by hand, leg. Krátky,J., det. Krátky,J.
Ceutorhynchus pectoralis<br>Weise, 1895	MK891975 [new]<br>2246-JKR<br>658nt	ZFMK-TIS-23984<br>ZFMK-DNA-0169170541	Slovakia, Zvolen, Hronska Dubrava, NPR Boky, N48°33'58" E19°01'10.5", 472m, 12-Okt-2014, *Barbaraea vularis*, sieving, leg. Krátky,J., det. Krátky,J.
Ceutorhynchus pervicax<br>Weise, 1883	MK891523 [new]<br>1610-JKR<br>658nt	ZFMK-TIS-3658<br>ZFMK-DNA-0155635608	Czech Republic, Bohemia, Bily Potok, N50°52'18" E15°12'29", 414m, 08-Mai-2013, *Cardamine amara*, sweeping, leg. Krátky,J., det. Krátky,J.
Ceutorhynchus pervicax<br>Weise, 1883	MK891846 [new]<br>2108-JKR<br>658nt	ZFMK-TIS-23748<br>ZFMK-DNA-0169169618	Czech Republic, Moravia, Lanzhot, N48°42'37" E16°58'27", 156m, 10-Mai-2014, *Cardamine pratensis*, sweeping, leg. Krátky,J., det. Krátky,J.
Ceutorhynchus pervicax<br>Weise, 1883	MK891092 [new]<br>996-PSP<br>658nt	ZFMK-TIS-3141<br>ZFMK-DNA-0100426874	Germany, Lower Saxony (NI), Diekholzen, Lkr. Hildesheim, N52°05'04" E09°55'17", 152m, 01-Mai-2012, *Cardamine amara*, forest of Hildesheim, beating, leg. Sprick,P., det. Sprick,P.

**Name**<br>**Authority**<br>**Additional Information**	**GenBank Acc No (Ref.)**<br>**Specimen ID**<br>**Sequence Length**	**ZFMK Tissue ID**<br>**ZFMK DNA Sample ID**<br>**(SDEI DNA Sample ID)**	**Locality, GPS, Collection Date, Plant, Collector, Identifier**
Ceutorhynchus pervicax<br>Weise, 1883	MK890987 [new]<br>879-CBR<br>658nt	ZFMK-TIS-3792<br>ZFMK-DNA-0100449732	Luxembourg, 10 km E of Luxembourg, 2 km SE of Mensdorf, N49°38'18" E06°19'04", 300m, 09-Apr-2011, *Cardamine pratense*, sweeping, leg. Braunert,C., det. Braunert,C.
Ceutorhynchus picitarsis<br>Gyllenhal, 1837	MK890922 [new]<br>790-PST<br>658nt	ZFMK-TIS-3703<br>ZFMK-DNA-0100414301	Germany, Rhineland-Palatinate (RLP), E of Treis-Karden near Pommern, Fellerbachtal (river valley), N50°10'10" E07°13'38", 85m, 09-Jun-2012, rivulet, beating, leg. Stüben,P., det. Stüben,P.
Ceutorhynchus picitarsis<br>Gyllenhal, 1837<br>determination uncertain	MK891335 [new]<br>1359-PST<br>658nt	ZFMK-TIS-4655<br>ZFMK-DNA-0155628574	Italy, Abruzzo, S. Stefano di Sessanio, N42°20'24" E13°37'30", 1271m, 14-Aug-2013, *Ostrya*, sieving, leg. Stüben,P., det. Stüben,P.
Ceutorhynchus picitarsis<br>Gyllenhal, 1837	KC783866 [new]<br>138-PST<br>658nt	ZFMK-TIS-2D100440280<br>ZFMK-DNA-0100438168	Morocco, S of Berkane, N of Aïn-es-Sfa, Beni Snassen Mts., N34°49'34" W02°11'36", 1307m, 13-Mai-2011, beating, leg. Stüben,P., det. Stüben,P./vid. Behne,L.
Ceutorhynchus picitarsis<br>Gyllenhal, 1837	MK891499 [new]<br>1584-JKR<br>658nt	ZFMK-TIS-3632<br>ZFMK-DNA-0155635645	Morocco, Haut Atlas, Oukaimeden, N31°12'10" W07°52'13", 2691m, 20-Apr-2013, *Alyssum spinosum*, sieving, leg. Krátky,J., det. Krátky,J.
Ceutorhynchus picitarsis<br>Gyllenhal, 1837	KC783936 [new]<br>337-JKR<br>658nt	ZFMK-TIS-20123<br>ZFMK-DNA-0100438751	Slovakia, Komarno, Iza env., PR Bokrosske slanisko , N47°44'50.372" E18°15'34.563", 107m, 20-Mai-2011, sweeping, leg. Krátky,J., det. Krátky,J.
Ceutorhynchus picitarsis<br>Gyllenhal, 1837	MK891212 [new]<br>1131-JKR<br>658nt	ZFMK-TIS-3467<br>ZFMK-DNA-0100449568	Slovakia, Komarno, Imel, N47°54'34" E18°09'16", 112m, 11-Mai-2012, *Sisymbrium orientale*, beating, leg. Krátky,J., det. Krátky,J.
Ceutorhynchus pulvinatus<br>Gyllenhal, 1837	KC783950 [new]<br>310-JKR<br>658nt	ZFMK-TIS-20096<br>ZFMK-DNA-0100438772	Czech Republic, Bohemia or., Uhretice, N49°58'40.663" E15°51'28.144", 240m, 14-Mai-2011, *Descurainia sophia*, beating, leg. Krátky,J., det. Krátky,J.
Ceutorhynchus pulvinatus<br>Gyllenhal, 1837	MK891845 [new]<br>2107-JKR<br>658nt	ZFMK-TIS-23747<br>ZFMK-DNA-0169169617	Czech Republic, Moravia, Cejc, PR Spidlaky, N48°55'23.5" E16°57'21", 220m, 10-Mai-2014, *Descurainia sophia*, sweeping, leg. Krátky,J., det. Krátky,J.
Ceutorhynchus pulvinatus<br>Gyllenhal, 1837	MK892263 [new]<br>2563-PSP<br>658nt	ZFMK-TIS-23537<br>ZFMK-DNA-0171661999	Slovakia, Nitra, Muzla, N47°48'00" E18°31'55", 114m, 19-Mai-2014, *Descurainia sophia*, leg. Sprick,P., det. Sprick,P.
Ceutorhynchus pumilio<br>(Gyllenhal, 1827)	KC783891 [new]<br>309-JKR<br>658nt	ZFMK-TIS-20095<br>ZFMK-DNA-0100438363	Czech Republic, Bohemia or., Belec nad Orlici, N50°12'1.188" E15°56'0.889", 230m, 10-Mai-2011, *Teesdalia nudicaulis*, beating, leg. Krátky,J., det. Krátky,J.
Ceutorhynchus pumilio<br>(Gyllenhal, 1827)	MK891108 [new]<br>1013-PSP<br>658nt	ZFMK-TIS-3158<br>ZFMK-DNA-0100426843	Germany, Lower Saxony (NI), Berkhof, Hannover area, N52°36'43" E09°43'59", 36m, 10-Mai-2012, *Teesdalia nudicaulis*, nutrient-poor sand grassland, dipnet, leg. Sprick,P., det. Sprick,P.
Ceutorhynchus pumilio<br>(Gyllenhal, 1827)	MK890982 [new]<br>873-CBR<br>658nt	ZFMK-TIS-3786<br>ZFMK-DNA-0100414223	Luxembourg, 15 km NW of Luxembourg, 3 km NE of Nospelt, N49°41'25" E06°01'21", 350m, 23-Jun-2012, *Teesdalia nudicaulis*, sweeping, leg. Braunert,C., det. Braunert,C.
Ceutorhynchus puncticollis<br>Boheman, 1845	KC783949 [new]<br>308-JKR<br>658nt	ZFMK-TIS-20094<br>ZFMK-DNA-0100438770	Czech Republic, Bohemia or., Hradec Kralove, Placky, N50°13'48.497" E15°49'25.886", 230m, 09-Mai-2011, *Berteroa incana*, beating, leg. Krátky,J., det. Krátky,J.
Ceutorhynchus puncticollis<br>Boheman, 1845	MK891801 [new]<br>2060-JKR<br>658nt	ZFMK-TIS-23700<br>ZFMK-DNA-0169170434	Czech Republic, Bohemia, Hradec Kralove, Kocianovice, N50°13'16" E15°52'51", 237m, 06-Mai-2014, *Berteroa incana*, collecting by hand, leg. Krátky,J., det. Krátky,J.

**Name**<br>**Authority**<br>**Additional Information**	**GenBank Acc No (Ref.)**<br>**Specimen ID**<br>**Sequence Length**	**ZFMK Tissue ID**<br>**ZFMK DNA Sample ID**<br>**(SDEI DNA Sample ID)**	**Locality, GPS, Collection Date, Plant, Collector, Identifier**
Ceutorhynchus puncticollis<br>Boheman, 1845	MK891839 [new]<br>2100-JKR<br>658nt	ZFMK-TIS-23740<br>ZFMK-DNA-0169169633	Czech Republic, Moravia, Hodonin, Panov, N48°53'12" E17°08'10", 205m, 10-Mai-2014, *Berteroa incana*, collecting by hand, leg. Krátky,J., det. Krátky,J.
Ceutorhynchus puncticollis<br>Boheman, 1845	MK891116 [new]<br>1021-PSP<br>658nt	ZFMK-TIS-3166<br>ZFMK-DNA-0100426932	Germany, Lower Saxony (NI), Hannover, Leinhausen, N52°23'53.6" E09°41'24", 52m, 13-Mai-2012, *Berteroa incana*, train station area, beating, leg. Sprick,P., det. Sprick,P.
Ceutorhynchus puncticollis<br>Boheman, 1845<br>formerly: *C. talickyi* det. Krátky - Syn! aedeagi identical	KC783962 [new]<br>333-JKR<br>658nt	ZFMK-TIS-20119<br>ZFMK-DNA-0100438924	Slovakia, Komarno, Virt env., N47°45'35.247" E18°20'25.833", 112m, 19-Mai-2011, *Erysimum* sp., sweeping, leg. Krátky,J., det. Krátky,J.
Ceutorhynchus puncticollis<br>Boheman, 1845<br>formerly: *C. talickyi* det. Krátky - Syn! aedeagi identical	MK891526 [new]<br>1614-JKR<br>658nt	ZFMK-TIS-3662<br>ZFMK-DNA-0155635612	Slovakia, Komarno, B?c env., N47°46'55" E18°25'11", 109m, 18-Mai-2013, *Erysimum diffusum*, individual collecting by hand, leg. Krátky,J., det. Krátky,J.
Ceutorhynchus puncticollis<br>Boheman, 1845<br>formerly: *C. talickyi* det. Sprick - Syn! aedeagi identical	MK892234 [new]<br>2532-PSP<br>658nt	ZFMK-TIS-23504<br>ZFMK-DNA-0171661741	Slovakia, Nitra, SW of Buc, N47°46'56" E18°25'10", 110m, 18-Mai-2014, *Erysimum canum*, leg. Sprick,P., det. Sprick,P.
Ceutorhynchus puncticollis<br>Boheman, 1845<br>formerly: *C. talickyi* det. Sprick - Syn! aedeagi identical	MK892247 [new]<br>2546-PSP<br>658nt	ZFMK-TIS-23520<br>ZFMK-DNA-0171662013	Slovakia, Nitra, Cenkov, N47°46'07" E18°31'11", 109m, 19-Mai-2014, *Erysimum canum*, leg. Sprick,P., det. Sprick,P.
Ceutorhynchus pyrrhorhynchus pyrrhorhynchus<br>(Marsham, 1802)	KC783957 [27]<br>350-JKR<br>658nt	ZFMK-TIS-20136<br>ZFMK-DNA-0100438912	Czech Republic, Bohemia or., Hradec Kralove, Placky, N50°13'48.497" E15°49'25.886", 230m, 29-Mai-2011, *Chamaeplium officinale*, beating, leg. Krátky,J., det. Krátky,J.
Ceutorhynchus pyrrhorhynchus pyrrhorhynchus<br>(Marsham, 1802)	KU932043 [27]<br>812-PST<br>658nt	ZFMK-TIS-3725<br>ZFMK-DNA-0100414279	Germany, Rhineland-Palatinate (RLP), N of Treis-Karden, Brohl, N50°13'22" E07°16'27", 255m, 10-Jun-2012, roadside ditch, beating, leg. Stüben,P., det. Stüben,P.
Ceutorhynchus pyrrhorhynchus pyrrhorhynchus<br>(Marsham, 1802)	KU932044 [27]<br>1050-PSP<br>658nt	ZFMK-TIS-3195<br>ZFMK-DNA-0100426928	Germany, Lower Saxony (NI), Hainhaus, Hannover region, Wietzesee, N52°28'09" E09°46'30", 46m, 10-Jun-2012, *Sisymbrium officinale, Sisymbrium loeselii*, ruderal area, beating, leg. Sprick,P., det. Sprick,P.
Ceutorhynchus pyrrhorhynchus pyrrhorhynchus<br>(Marsham, 1802)	KU932048 [27]<br>2499-PSP<br>658nt	ZFMK-TIS-23471<br>ZFMK-DNA-0171661774	Germany, Saxony-Anhalt (ST), Beidersee, Halle, N51°34'12.5" E11°54'14", 154m, 17-Mai-2014, *Sisymbrium loeselii*, beating, leg. Sprick,P., det. Sprick,P.

**Name**<br>**Authority**<br>**Additional Information**	**GenBank Acc No (Ref.)**<br>**Specimen ID**<br>**Sequence Length**	**ZFMK Tissue ID**<br>**ZFMK DNA Sample ID**<br>**(SDEI DNA Sample ID)**	**Locality, GPS, Collection Date, Plant, Collector, Identifier**
Ceutorhynchus pyrrhorhynchus pyrrhorhynchus<br>(Marsham, 1802)	KU932045 [27]<br>1075-JKR<br>658nt	ZFMK-TIS-3411<br>ZFMK-DNA-0100449633	Spain, Andalucia, Pedro Valiente, N36°02'56" W05°38'21", 5m, 15-Apr-2012, *Diplotaxis erucoides*, sweeping, leg. Krátky,J., det. Krátky,J.
Ceutorhynchus rapae<br>Gyllenhal, 1837	MK891507 [new]<br>1593-JKR<br>658nt	ZFMK-TIS-3641<br>ZFMK-DNA-0155635639	Czech Republic, Moravia, Poudrany - step, N48°56'51" E16°38'13", 260m, 01-Mai-2013, *Sisymbrium loesseli*, beating, leg. Krátky,J., det. Krátky,J.
Ceutorhynchus rapae<br>Gyllenhal, 1837	MK891844 [new]<br>2106-JKR<br>658nt	ZFMK-TIS-23746<br>ZFMK-DNA-0169169616	Czech Republic, Moravia, Cejc, PR Spidlaky, N48°55'23.5" E16°57'21", 220m, 10-Mai-2014, *Descurainia sophia*, sweeping, leg. Krátky,J., det. Krátky,J.
Ceutorhynchus rapae<br>Gyllenhal, 1837	MK891221 [new]<br>1141-JKR<br>658nt	ZFMK-TIS-3477<br>ZFMK-DNA-0100449578	Slovakia, Komarno, Imel, N47°54'34" E18°09'16", 112m, 11-Mai-2012, sweeping, leg. Krátky,J., det. Krátky,J.
Ceutorhynchus resedae<br>(Marsham, 1802)	MK891117 [new]<br>1022-PSP<br>658nt	ZFMK-TIS-3167<br>ZFMK-DNA-0100426837	Germany, Lower Saxony (NI), Braunschweig, Lindenbergsiedlung, N52°14'43" E10°33'13", 80m, 14-Mai-2012, *Reseda luteola*, railway area, beating, leg. Sprick,P., det. Sprick,P.
Ceutorhynchus rhenanus<br>(Schultze, 1895)	KU932046 [27]<br>2127-JKR<br>658nt	ZFMK-TIS-23767<br>ZFMK-DNA-0169169606	Slovakia, Nitra, Zobor Mt., N48°21'08.5 E18°05'41", 427m, 24-Mai-2014, *Erysimum* cf. *odoratum*, sweeping, leg. Krátky,J., det. Krátky,J.
Ceutorhynchus roberti<br>Gyllenhal, 1837	KC783975 [new]<br>303-JKR<br>658nt	ZFMK-TIS-20089<br>ZFMK-DNA-0100438966	Czech Republic, Bohemia or., Hradec Kralove, Fararstvi, N50°11'26.901" E15°48'57.048", 230m, 06-Mai-2011, *Alliaria petiolata*, beating, leg. Krátky,J., det. Krátky,J.
Ceutorhynchus roberti<br>Gyllenhal, 1837	MK892165 [new]<br>2456-PSP<br>658nt	ZFMK-TIS-23428<br>ZFMK-DNA-0171661817	Germany, Lower Saxony (NI), Hameln-Rohrsen, Dütberg, N52°06'22" E09°25'02.5", 100m, 10-Mai-2013, *Alliaria petiolata*, beating, leg. Sprick,P., det. Sprick,P.
Ceutorhynchus roberti<br>Gyllenhal, 1837	MK890997 [new]<br>889-CBR<br>658nt	ZFMK-TIS-3802<br>ZFMK-DNA-0100449711	Luxembourg, 10 km E of Luxembourg, 1.5 km SW of Mensdorf, N49°38'47" E06°17'38", 260m, 19-Apr-2011, *Alliaria petiolata*, beating, leg. Braunert,C., det. Braunert,C.
Ceutorhynchus sardeanensis<br>Schultze, 1903	MK891177 [new]<br>1090-JKR<br>658nt	ZFMK-TIS-3426<br>ZFMK-DNA-0100449623	Spain, Andalucia, 8 km W of Espera, N36°53'17" W05°53'47", 50m, 17-Apr-2012, *yellow flowering Brassicaceae*, sweeping, leg. Krátky,J., det. Krátky,J.
Ceutorhynchus scrobicollis<br>Neresheimer & Wagner, 1924	MK891104 [new]<br>1008-PSP<br>658nt	ZFMK-TIS-3153<br>ZFMK-DNA-0100426851	Germany, Lower Saxony (NI), Hannover, Leinhausen, N52°23'53.9" E09°41'25", 52m, 07-Mai-2012, *Alliaria petiolata*, train station area, beating, leg. Sprick,P., det. Sprick,P.
Ceutorhynchus scrobicollis<br>Neresheimer & Wagner, 1924	MK891864 [new]<br>2128-JKR<br>658nt	ZFMK-TIS-23768<br>ZFMK-DNA-0169169605	Slovakia, Nitra, Zobor Mt., N48°21'08.5 E18°05'41", 427m, 24-Mai-2014, *Erysimum* cf. *odoratum*, sweeping, leg. Krátky,J., det. Krátky,J.
Ceutorhynchus scrobicollis<br>Neresheimer & Wagner, 1924	MK891539 [new]<br>1628-JKR<br>658nt	ZFMK-TIS-3676<br>ZFMK-DNA-0155635601	Slovakia, Nove Zamky, Lela, N47°51'27" E18°47'43", 153m, 19-Mai-2013, *Alliaria petiolata*, individual collecting by hand, leg. Krátky,J., det. Krátky,J.
Ceutorhynchus sisymbrii<br>(Dieckmann, 1966)	MK891549 [new]<br>1639-JKR<br>657nt	ZFMK-TIS-3687<br>ZFMK-DNA-0155635589	Czech Republic, Moravia, Oblekovice, Naceraticky kopec, N48°50'09" E16°05'45", 256m, 08-Jun-2013, *Sysimbrium loesseli*, individual collecting by hand, leg. Krátky,J., det. Krátky,J.

**Name**<br>**Authority**<br>**Additional Information**	**GenBank Acc No (Ref.)**<br>**Specimen ID**<br>**Sequence Length**	**ZFMK Tissue ID**<br>**ZFMK DNA Sample ID**<br>**(SDEI DNA Sample ID)**	**Locality, GPS, Collection Date, Plant, Collector, Identifier**
Ceutorhynchus sophiae<br>Gyllenhal, 1837	MK891843 [new]<br>2105-JKR<br>658nt	ZFMK-TIS-23745<br>ZFMK-DNA-0169169628	Czech Republic, Moravia, Cejc, PR Spidlaky, N48°55'23.5" E16°57'21", 220m, 10-Mai-2014, *Descurainia sophia*, sweeping, leg. Krátky,J., det. Krátky,J.
Ceutorhynchus sophiae<br>Gyllenhal, 1837	MK891899 [new]<br>2165-JKR<br>658nt	ZFMK-TIS-23805<br>ZFMK-DNA-0171661930	Slovakia, Komarno, Marcelova, PR Masan, N47°46'10.5" E18°19'06", 121m, 21-Mai-2014, *Descurainia sophia*, sweeping, leg. Krátky,J., det. Krátky,J.
Ceutorhynchus squamulosus<br>C. Brisout de Barneville, 1869	MK891309 [new]<br>1297-PST<br>658nt	ZFMK-TIS-4113<br>ZFMK-DNA-0100426300	Spain, Community of Madrid, S of La Serna del Monte, N41°01'28" W03°38'00", 1028m, 03-Mai-2013, *Brassicaceae*, beating, leg. Stüben,P. & Schütte,A., det. Stüben,P.
Ceutorhynchus striatellus<br>Schultze, 1900	MK891518 [new]<br>1605-JKR<br>658nt	ZFMK-TIS-3653<br>ZFMK-DNA-0155635624	Czech Republic, Moravia, Cejc - Spidlaky, N48°55'47" E16°58'03", 184m, 01-Mai-2013, *Alyssum alyssoides*, sieving, leg. Krátky,J., det. Krátky,J.
Ceutorhynchus striatellus<br>Schultze, 1900	KC783920 [new]<br>355-JKR<br>658nt	ZFMK-TIS-20141<br>ZFMK-DNA-0100438721	Greece, Thessalia, Ossa Mts., N39°50'03.37" E22°41'04.86", 1050m, 05-Jun-2011, *Alyssum murale*, beating, leg. Krátky,J., det. Krátky,J.
Ceutorhynchus striatellus<br>Schultze, 1900	MK891245 [new]<br>1172-JKR<br>658nt	ZFMK-TIS-3508<br>ZFMK-DNA-0100426272	Slovakia, Nove Zamky, Cenkov, NPR Cenkovska step, N47°46'07" E18°31'12", 110m, 28-Mai-2012, *Alyssum montanum gmelini*, beating, leg. Krátky,J., det. Krátky,J.
Ceutorhynchus striatellus<br>Schultze, 1900	MK891252 [new]<br>1183-JKR<br>658nt	ZFMK-TIS-3519<br>ZFMK-DNA-0100426273	Spain, Andalucia, Sierra Nevada, Puerto de la Ragua, N37°05'50" W03°01'20", 1938m, 11-Jun-2012, *Hormatophylla spinosa*, beating, leg. Krátky,J., det. Krátky,J.
Ceutorhynchus sulcatus<br>C. Brisout de Barneville, 1869	KU932047 [27]<br>2146-JKR<br>658nt	ZFMK-TIS-23786<br>ZFMK-DNA-0169169587	Slovakia, Nove Zamky, Cenkov, NPR Censkovska step, N47°46'09" E18°31'09", 110m, 19-Mai-2014, *Camelina microcarpa*, sweeping, leg. Krátky,J., det. Krátky,J.
Ceutorhynchus sulcicollis<br>(Paykull, 1800)	MK891248 [new]<br>1179-JKR<br>658nt	ZFMK-TIS-3515<br>ZFMK-DNA-0100426096	Czech Republic, Bohemia, Borovy, N49°31'23" E13°18'26", 452m, 25-Aug-2012, sieving, leg. Krátky,J., det. Krátky,J.
Ceutorhynchus sulcicollis<br>(Paykull, 1800)	MK891354 [new]<br>1399-PST<br>658nt	ZFMK-TIS-4695<br>ZFMK-DNA-0155628533	Italy, Lazio, NW of San Donato Val di Camino, N of Alvito/S. Onófrio, Valle di Rio, N41°43'24" E13°44'41", 671m, 27-Aug-2013, mixed forest, sieving, leg. Stüben,P., det. Stüben,P.
Ceutorhynchus syrites<br>Germar, 1824	MK891508 [new]<br>1594-JKR<br>658nt	ZFMK-TIS-3642<br>ZFMK-DNA-0155635640	Czech Republic, Moravia, Poudrany - step, N48°56'51" E16°38'13", 260m, 01-Mai-2013, sweeping, leg. Krátky,J., det. Krátky,J.
Ceutorhynchus tangerianus<br>Schultze, 1900	MK891491 [new]<br>1573-JKR<br>658nt	ZFMK-TIS-3621<br>ZFMK-DNA-0155635667	Morocco, Marrakech-Tensift-El Haouz, Essaouira, 9 km E, N31°29'35" W09°43'00", 88m, 12-Apr-2013, *Malcolmia aegyptiaca*, sweeping, leg. Krátky,J., det. Krátky,J.
Ceutorhynchus tangerianus<br>Schultze, 1900	MK891170 [new]<br>1081-JKR<br>658nt	ZFMK-TIS-3417<br>ZFMK-DNA-0100449627	Spain, Andalucia, Rociana del Condado, N37°21'01" W06°36'55", 150m, 18-Apr-2012, *Malcolmia lacera*, sweeping, leg. Krátky,J., det. Krátky,J.
Ceutorhynchus tangerianus<br>Schultze, 1900	MK891178 [new]<br>1092-JKR<br>658nt	ZFMK-TIS-3428<br>ZFMK-DNA-0100449153	Spain, Andalucia, Mazagón, N37°07'13" W06°47'16", 40m, 18-Apr-2012, *Malcolmia littorea*, sweeping, leg. Krátky,J., det. Krátky,J.
Ceutorhynchus tibialis<br>Boheman, 1845	MK891172 [new]<br>1083-JKR<br>658nt	ZFMK-TIS-3419<br>ZFMK-DNA-0100449616	Spain, Andalucia, Rociana del Condado, N37°21'01" W06°36'55", 150m, 18-Apr-2012, *Diplotaxis erucoides*, sweeping, leg. Krátky,J., det. Krátky,J.

**Name**<br>**Authority**<br>**Additional Information**	**GenBank Acc No (Ref.)**<br>**Specimen ID**<br>**Sequence Length**	**ZFMK Tissue ID**<br>**ZFMK DNA Sample ID**<br>**(SDEI DNA Sample ID)**	**Locality, GPS, Collection Date, Plant, Collector, Identifier**
Ceutorhynchus turbatus<br>Schultze, 1903	KC783994 [new]<br>261-PSP<br>658nt	ZFMK-TIS-2D100439107<br>ZFMK-DNA-0100439779	Germany, Lower Saxony (NI), Hannover, Stöcken, Leine wetland, N52°24'12" E09°39'16", 45m, 14-Mai-2011, *Cardaria draba*, roadside, dipnet, leg. Sprick,P., det. Sprick,P.
Ceutorhynchus typhae<br>(Herbst, 1795)	MK891805 [new]<br>2064-JKR<br>658nt	ZFMK-TIS-23704<br>ZFMK-DNA-0169170438	Czech Republic, Bohemia, Hradec Kralove, Kocianovice, N50°13'16" E15°52'51", 237m, 06-Mai-2014, *Cardaria draba*, collecting by hand, leg. Krátky,J., det. Krátky,J.
Ceutorhynchus typhae<br>(Herbst, 1795)	MK891857 [new]<br>2119-JKR<br>658nt	ZFMK-TIS-23759<br>ZFMK-DNA-0169169614	Czech Republic, Bohemia, Hradec Kralove, Borovinka, N50°11'24" E15°47'60", 228m, 15-Mai-2014, *Arabidopsis thaliana*, sweeping, leg. Krátky,J., det. Krátky,J.
Ceutorhynchus typhae<br>(Herbst, 1795)	MK892072 [new]<br>2348-JKR<br>658nt	ZFMK-TIS-24279<br>ZFMK-DNA-0169170918	Czech Republic, Bohemia, Pohrebacka, N50°9'15" E15°46'42", 226m, 24-Jul-2014, *Nasturtium officinale*, collecting by hand, leg. Krátky,J., det. Krátky,J.
Ceutorhynchus typhae<br>(Herbst, 1795)<br>formerly: *C. cakilis* - Syn!	KC783969 [new]<br>351-JKR<br>658nt	ZFMK-TIS-20137<br>ZFMK-DNA-0100438942	Germany, Lower Saxony (NI), Cuxhaven, Sahlenburg, N53°51'57.52" E08°35'48.51", 1m, 24-Sep-2011, *Cakile maritima*, beating, leg. Krátky,J., det. Krátky,J.
Ceutorhynchus typhae<br>(Herbst, 1795)	KC784006 [new]<br>237-PSP<br>658nt	ZFMK-TIS-2D100438543<br>ZFMK-DNA-0100439803	Germany, Bavaria (BY), Buckenhof/Uttenreuth, Lkr. Erlangen-Höchstadt, N49°35'20" E11°05'17", 474m, 29-Apr-2011, *Arabidopsis thaliana*, sand field, dipnet, leg. Sprick,P., det. Sprick,P.
Ceutorhynchus typhae<br>(Herbst, 1795)<br>formerly: *C. cakilis* - Syn!	MK891150 [new]<br>1057-PSP<br>658nt	ZFMK-TIS-3202<br>ZFMK-DNA-0100426791	Germany, Lower Saxony (NI), Langeoog, Flinthörn, N53°44'00" E07°28'02", 5m, 16-Jun-2012, *Cakile maritima*, white dune, beating, leg. Sprick,P., det. Sprick,P.
Ceutorhynchus typhae<br>(Herbst, 1795)	MK891565 [new]<br>877-CBR<br>658nt	ZFMK-TIS-3790<br>ZFMK-DNA-0155630482	Luxembourg, 10 km E of Luxembourg, 2 km SE of Mensdorf, N49°38'18" E06°19'04", 300m, 09-Apr-2011, *Cardamine pratense*, sweeping, leg. Braunert,C., det. Braunert,C.
Ceutorhynchus typhae<br>(Herbst, 1795)	MK891281 [new]<br>1232-PST<br>658nt	ZFMK-TIS-3568<br>ZFMK-DNA-0100426247	Portugal, Minho, S of Viana, Castelo do Neiva, N41°37'07 W08°48'35", 8m, 10-Mai-2012, *watercress (ger. "Brunnenkresse")*, beating, leg. Stüben,P., det. Krátky,J.
Ceutorhynchus typhae<br>(Herbst, 1795)	MK892041 [new]<br>2317-JKR<br>658nt	ZFMK-TIS-24248<br>ZFMK-DNA-0169170948	Slovakia, Tatry Mts., Dolina Siedmich prame?ov, N49°13'30" E20°16'24", 1404m, 05-Jul-2014, *Cardamine* sp., collecting by hand, leg. Krátky,J., det. Krátky,J.
Ceutorhynchus typhae<br>(Herbst, 1795)	MK892049 [new]<br>2325-JKR<br>658nt	ZFMK-TIS-24256<br>ZFMK-DNA-0169170943	Slovakia, Tatry Mts., Bujaci vrch, N49°14'10" E20°14'27", 1914m, 06-Jul-2014, *Biscutella laevigata*, collecting by hand, leg. Krátky,J., det. Krátky,J.
Ceutorhynchus varius<br>Rey, 1895	KC783932 [new]<br>332-JKR<br>658nt	ZFMK-TIS-20118<br>ZFMK-DNA-0100438746	Slovakia, Komarno, Marcelova env., PR Masan, N47°46'4.755" E18°19'13.531", 121m, 18-Mai-2011, sweeping, leg. Krátky,J., det. Krátky,J.
Ceutorhynchus wagneri<br>Smreczynski, 1937	KC783930 [new]<br>341-JKR<br>658nt	ZFMK-TIS-20127<br>ZFMK-DNA-0100438742	Slovakia, Komarno, Cenkov, N47°46'8.052" E18°31'18.977", 105m, 21-Mai-2011, *Alyssum alyssoides*, sweeping, leg. Krátky,J., det. Krátky,J.
Ceutorhynchus wagneri<br>Smreczynski, 1937	MK891868 [new]<br>2132-JKR<br>658nt	ZFMK-TIS-23772<br>ZFMK-DNA-0169169594	Slovakia, Komarno, Buc, N47°46'55.5" E18°25'10", 107m, 18-Mai-2014, *Alyssum alyssoides*, sweeping, leg. Krátky,J., det. Krátky,J.
Ceutorhynchus wagneri<br>Smreczynski, 1937	MK891882 [new]<br>2147-JKR<br>658nt	ZFMK-TIS-23787<br>ZFMK-DNA-0169169586	Slovakia, Nove Zamky, Cenkov, NPR Censkovska step, N47°46'09" E18°31'09", 110m, 19-Mai-2014, *Alyssum alyssoides*, sweeping, leg. Krátky,J., det. Krátky,J.

**Name**<br>**Authority**<br>**Additional Information**	**GenBank Acc No (Ref.)**<br>**Specimen ID**<br>**Sequence Length**	**ZFMK Tissue ID**<br>**ZFMK DNA Sample ID**<br>**(SDEI DNA Sample ID)**	**Locality, GPS, Collection Date, Plant, Collector, Identifier**
Ceutorhynchus wagneri<br>Smreczynski, 1937	MK892250 [new]<br>2549-PSP<br>658nt	ZFMK-TIS-23523<br>ZFMK-DNA-0171662010	Slovakia, Nitra, Cenkov, N47°46'07" E18°31'11", 109m, 19-Mai-2014, *Alyssum* sp., leg. Sprick,P., det. Sprick,P.
Ceutorhynchus wellschmiedi<br>Dieckmann, 1979<br>junior syn. of *C. chalybaeus*?	MK891521 [new]<br>1608-JKR<br>658nt	ZFMK-TIS-3656<br>ZFMK-DNA-0155635621	Czech Republic, Moravia, Brezi env., NPP Dunajovicke vrchy, N48°50'23" E16°33'46", 260m, 02-Mai-2013, *Crambe tataria*, sweeping, leg. Krátky,J., det. Krátky,J.
Charagmus gressorius<br>(Fabricius, 1792)	MK891478 [new]<br>1548-PSP<br>658nt	ZFMK-TIS-3308<br>ZFMK-DNA-0155633290	Germany, Lower Saxony (NI), Harz National Park, Bad Harzburg, Ottenhai, N51°53'19" E10°35'50", 307m, 20-Sep-2013, *Lotus pedunculatus*, clearing, beating, leg. Sprick,P., det. Sprick,P.
Charagmus gressorius<br>(Fabricius, 1792)	MK891587 [new]<br>582-PSP<br>658nt	ZFMK-TIS-20366<br>ZFMK-DNA-0155630425	Germany, Saxony-Anhalt (ST), Drömling, Mannhausen, Mittelland channel dam, N52°25'28" E11°13'00", 55m, 07-Jun-2011, *Lupinus polyphyllus*, beating, leg. Sprick,P., det. Sprick,P.
Charagmus gressorius<br>(Fabricius, 1792)	MK891031 [new]<br>928-CBR<br>658nt	ZFMK-TIS-3841<br>ZFMK-DNA-0100449683	Luxembourg, 10 km E of Luxembourg, 2 km S of Mensdorf, N49°38'09" E06°17'48", 330m, 05-Jun-2012, *Lupinus*, beating, leg. Braunert,C., det. Braunert,C.
Charagmus gressorius<br>(Fabricius, 1792)	MK890866 [new]<br>442-RGO<br>658nt	ZFMK-TIS-20228<br>ZFMK-DNA-0100449397	Poland, Zurawce, N50°34'3.6" E23°51'57.6", 248m, 21-Sep-2011, leg. Gosik,R., det. Gosik,R.
Charagmus gressorius<br>(Fabricius, 1792)	MK890905 [40]<br>703-PST<br>658nt	ZFMK-TIS-3040<br>ZFMK-DNA-0100448177	Spain, Canary Islands, Tenerife, Teno Mts. near Erjos, N28°19'31" W16°48'32", 1017m, 04-Jan-2012, *Bituminaria*, beating, leg. Stüben,P., det. Stüben,P.
Charagmus griseus<br>(Fabricius, 1775)	MK890895 [new]<br>584-PSP<br>658nt	ZFMK-TIS-20368<br>ZFMK-DNA-0100448398	Germany, Saxony-Anhalt (ST), Drömling, Mannhausen, N52°25'28" E11°13'00", 55m, 07-Jun-2011, *Cytisus scoparius*, wet ruderal area, beating, leg. Sprick,P., det. Sprick,P.
Charagmus griseus<br>(Fabricius, 1775)	MK890856 [new]<br>405-RGO<br>658nt	ZFMK-TIS-20191<br>ZFMK-DNA-0100449423	Poland, Rudnik, N51°16'36" E22°37'57", 191m, 05-Jun-2011, leg. Gosik,R., det. Gosik,R.
Charagmus griseus<br>(Fabricius, 1775)	MK891254 [new]<br>1186-JKR<br>658nt	ZFMK-TIS-3522<br>ZFMK-DNA-0100426237	Spain, Andalucia, San Agustin, N36°41'15" W02°42'40", 1m, 11-Jun-2012, coast, sandy dunes, sweeping, leg. Krátky,J., det. Krátky,J.
Charagmus intermedius<br>(Küster, 1847)	KC784249 [40]<br>IT-0016w<br>658nt	ZFMK-TIS-2D100446537<br>ZFMK-DNA-0100437862	Italy, Sardinia Isl., NE of Aritzo, Gennargentu, S`Arcu de Tascussi, N40°02'13" E09°14'22", 1317m, 30-Sep-2010, broom, leg. Stüben,P., det. Stüben,P.
Chlorophanus excisus<br>(Fabricius, 1801)	MK892244 [new]<br>2542-PSP<br>658nt	ZFMK-TIS-23514<br>ZFMK-DNA-0171661736	Slovakia, Nitra, Cenkov, N47°46'07" E18°31'11", 109m, 19-Mai-2014, *Rhamnus cathartica*, leg. Sprick,P., det. Sprick,P.
Chlorophanus flavescens<br>(Fabricius, 1787)	MK891237 [new]<br>1157-JKR<br>658nt	ZFMK-TIS-3493<br>ZFMK-DNA-0100449546	Hungary, Jasz-Nagykun-Szolnok megye, Tiszaroff, N47°21'55" E20°27'32", 87m, 25-Mai-2012, *Populus alba*, sweeping, leg. Krátky,J., det. Krátky,J.
Chlorophanus viridis<br>(Linnaeus, 1758)	KC784227 [new]<br>404-RGO<br>658nt	ZFMK-TIS-20190<br>ZFMK-DNA-0100449435	Poland, Rudnik, N51°16'36" E22°37'57", 191m, 05-Jun-2011, leg. Gosik,R., det. Gosik,R.
Chonostropheus tristis<br>(Fabricius, 1794)	MK891088 [new]<br>992-PSP<br>658nt	ZFMK-TIS-3137<br>ZFMK-DNA-0100426870	Germany, Lower Saxony (NI), Diekholzen, Lkr. Hildesheim, N52°05'04" E09°55'17", 152m, 01-Mai-2012, *Acer pseudoplatanus*, forest of Hildesheim, beating, leg. Sprick,P., det. Sprick,P.

**Name**<br>**Authority**<br>**Additional Information**	**GenBank Acc No (Ref.)**<br>**Specimen ID**<br>**Sequence Length**	**ZFMK Tissue ID**<br>**ZFMK DNA Sample ID**<br>**(SDEI DNA Sample ID)**	**Locality, GPS, Collection Date, Plant, Collector, Identifier**
Cimberis attelaboides<br>(Fabricius, 1787)	MK892218 [new]<br>2514-PSP<br>658nt	ZFMK-TIS-23486<br>ZFMK-DNA-0171661756	Germany, Saxony-Anhalt (ST), Harz National Park, Feuersteinwiesen, N51°45'21" E10°41'25", 604m, 13-Mai-2014, *Wiesenkrautschicht!*, leg. Sprick,P., det. Sprick,P.
Cionus alauda<br>(Herbst, 1784)	MK891018 [new]<br>915-CBR<br>658nt	ZFMK-TIS-3828<br>ZFMK-DNA-0100449689	France, Dep. Marne, 10 km NW of Chalons-en-Champagne, 1 km NW of Matougues, N49°00'13" E04°13'55", 75m, 20-Mai-2011, *Scropholaria nodosa*, beating, leg. Braunert,C., det. Braunert,C.
Cionus alauda<br>(Herbst, 1784)	MK890914 [new]<br>782-PST<br>658nt	ZFMK-TIS-3695<br>ZFMK-DNA-0100414309	Germany, Rhineland-Palatinate (RLP), E of Treis-Karden near Pommern, Fellerbachtal (river valley), N50°10'10" E07°13'38", 85m, 09-Jun-2012, *Scrophularia*, rivulet, beating, leg. Stüben,P., det. Stüben,P.
Cionus alauda<br>(Herbst, 1784)	MK891096 [new]<br>1000-PSP<br>658nt	ZFMK-TIS-3145<br>ZFMK-DNA-0100426244	Germany, Lower Saxony (NI), Aerzen-Ahorn, Lkr. Hameln-Pyrmont, N52°03'17" E09°13'02", 171m, 05-Mai-2012, *Scrophularia nodosa*, wet forest track , dipnet, leg. Sprick,P., det. Sprick,P.
Cionus alauda<br>(Herbst, 1784)	MK891758 [new]<br>2014-JKR<br>658nt	ZFMK-TIS-23654<br>ZFMK-DNA-0169170484	Italy, Sicilia Isl. (TP), Trapani, Marinella, N37°34'59" E12°51'40", 3m, 17-Apr-2014, *Scrophularia* sp., sweeping, leg. Krátky,J., det. Krátky,J.
Cionus alauda<br>(Herbst, 1784)	MK891666 [40]<br>1867-PST<br>658nt	ZFMK-TIS-26070<br>ZFMK-DNA-0171606104	Portugal, Madeira, E of Arco de S. Jorge, Caminho da Cancelinha, N32°49'22" W16°56'27", 469m, 05-Jul-2014, *Scrophularia*, beating, leg. Stüben,P., det. Stüben,P.
Cionus alauda<br>(Herbst, 1784)	MK891670 [40]<br>1875-PST<br>658nt	ZFMK-TIS-26078<br>ZFMK-DNA-0171606091	Portugal, Madeira, SW of Sao Roque de Faial, road to Faja da Nouqueira, N32°44'26" W16°54'04", 582m, 10-Jul-2014, *Scrophularia*, beating, leg. Stüben,P., det. Stüben,P.
Cionus cf. *alauda*<br>Comolli, 1837<br>formerly: *C. alauda villae*	KC783889 [new]<br>141-PST<br>658nt	ZFMK-TIS-2D100440283<br>ZFMK-DNA-0100438349	Morocco, S of Berkane, N of Aïn-es-Sfa, Beni Snassen Mts., N34°51'30" W02°08'41", 840m, 13-Mai-2011, beating, leg. Stüben,P., det. Stüben,P./vid. Behne,L.
Cionus clairvillei<br>Boheman, 1838	MK891870 [new]<br>2134-JKR<br>658nt	ZFMK-TIS-23774<br>ZFMK-DNA-0169169596	Slovakia, Komarno, Buc, N47°46'55.5" E18°25'10", 107m, 18-Mai-2014, *Verbascum* sp., sweeping, leg. Krátky,J., det. Krátky,J.
Cionus gebleri<br>Gyllenhal, 1838	KC784221 [new]<br>406-RGO<br>658nt	ZFMK-TIS-20192<br>ZFMK-DNA-0100449424	Poland, ad. Urszulin , N51°22'16" E23°15'04" , 172m, 06-Jun-2011, leg. Gosik,R., det. Gosik,R.
Cionus griseus<br>Lindberg, 1958	KC784304 [40]<br>740-PST<br>658nt	ZFMK-TIS-3077<br>ZFMK-DNA-0100448147	Spain, Canary Islands, Tenerife, Orotava valley, above Aguamansa, “Wildpret”, N28°19'11" W16°33'28", 1794m, 04-Feb-2012, *Scrophularia glabrata*, beating, leg. Stüben,P. & Schütte,A., det. Stüben,P.
Cionus griseus<br>Lindberg, 1958	KC784314 [40]<br>760-PST<br>658nt	ZFMK-TIS-3097<br>ZFMK-DNA-0100448138	Spain, Canary Islands, Tenerife, N of Vilaflor, Barranco de Eris de Carnero, N28°10'23" W16°37'12", 1656m, 23-Feb-2012, *Scrophularia glabrata*, beating, leg. Stüben,P., det. Stüben,P.
Cionus hortulanus<br>(Geoffroy, 1785)	MK890913 [new]<br>781-PST<br>658nt	ZFMK-TIS-3694<br>ZFMK-DNA-0100414310	Germany, Rhineland-Palatinate (RLP), E of Treis-Karden near Pommern, Fellerbachtal (river valley), N50°10'10" E07°13'38", 85m, 09-Jun-2012, *Scrophularia*, beating, leg. Stüben,P., det. Stüben,P.
Cionus hortulanus<br>(Geoffroy, 1785)	MK891097 [new]<br>1001-PSP<br>658nt	ZFMK-TIS-3146<br>ZFMK-DNA-0100426858	Germany, Lower Saxony (NI), Aerzen-Ahorn, Lkr. Hameln-Pyrmont, N52°03'17" E09°13'02", 171m, 05-Mai-2012, *Scrophularia nodosa*, wet forest track , dipnet, leg. Sprick,P., det. Sprick,P.
Cionus hortulanus<br>(Geoffroy, 1785)	MK891036 [new]<br>934-CBR<br>658nt	ZFMK-TIS-3847<br>ZFMK-DNA-0100449677	Luxembourg, 10 km E of Luxembourg, 1 km SW of Mensdorf, N49°38'55" E06°17'13", 260m, 17-Jun-2012, *Scropholaria nodosa*, pooter, leg. Braunert,C., det. Braunert,C.

**Name**<br>**Authority**<br>**Additional Information**	**GenBank Acc No (Ref.)**<br>**Specimen ID**<br>**Sequence Length**	**ZFMK Tissue ID**<br>**ZFMK DNA Sample ID**<br>**(SDEI DNA Sample ID)**	**Locality, GPS, Collection Date, Plant, Collector, Identifier**
Cionus hortulanus<br>(Geoffroy, 1785)	MK891685 [new]<br>1901-PST<br>658nt	ZFMK-TIS-24006<br>ZFMK-DNA-0169170422	Netherlands, Elmpt, NP "DE of Meinweg", N51°11'08" E06°08'22", 75m, 28-Aug-2014, *Scrophularia*, beating, leg. Stüben,P., det. Stüben,P.
Cionus longicollis montanus<br>Wingelmüller, 1914	GU987774 [01]<br>M-0068-CiS<br>658nt	ZFMK-TIS-cM0068	Germany, Rhineland-Palatinate (RLP), Moselle valley, Cochem, Dekernbach, mountain Wakelay, N50°09'31" E07°09'09", 301m, 15-Sep-2005, *Quercus, Carpinus*, leg. Stüben,P., det. Stüben,P.
Cionus longicollis montanus<br>Wingelmüller, 1914	MK890981 [new]<br>872-PST<br>658nt	ZFMK-TIS-3785<br>ZFMK-DNA-0100414222	Germany, Rhineland-Palatinate (RLP), Eifel, Nettersheim, Urftbach, N50°30'36" E06°37'20", 446m, 05-Aug-2012, beating, leg. Stüben,P., det. Stüben,P.
Cionus longicollis montanus<br>Wingelmüller, 1914	MK892122 [new]<br>2399-JKR<br>658nt	ZFMK-TIS-25976<br>ZFMK-DNA-0171600621	Slovakia, Zilina, Suja, N49°03'46" E18°37'19", 474m, 21-Jun-2014, *Verbascum* sp., sweeping, leg. Krátky,J., det. Krátky,J.
Cionus longicollis montanus<br>Wingelmüller, 1914	MK891060 [new]<br>961-CBR<br>658nt	ZFMK-TIS-3874<br>ZFMK-DNA-0100449639	Switzerland, 4 km SW of Zernez, Prazet, N46°40'12" E10°02'51", 1650m, 22-Jul-2011, *Verbascum*, beating, leg. Braunert,C., det. Braunert,C.
Cionus nigritarsis<br>Reitter, 1904	MK892001 [new]<br>2273-JKR<br>658nt	ZFMK-TIS-24204<br>ZFMK-DNA-0169170987	Austria, Styria, Hochschwab, Stallmauer, N47°35'29" E15°02'20", 1260m, 27-Jun-2014, *Scrophularia* sp., collecting by hand, leg. Krátky,J., det. Stüben,P.
Cionus nigritarsis<br>Reitter, 1904	MK891424 [new]<br>1487-PSP<br>658nt	ZFMK-TIS-3247<br>ZFMK-DNA-0155633342	Germany, North Rhine-Westphalia (NRW), Paderborn, Wewer, Ziegenberg, N51°41'11" E08°42'44", 152m, 15-Jul-2012, *Verbascum nigrum*, wayside, beating, leg. Sprick,P., det. Sprick,P.
Cionus olens<br>(Fabricius, 1792)	KC784181 [new]<br>477-PST<br>658nt	ZFMK-TIS-20262<br>ZFMK-DNA-0100449351	Germany, Rhineland-Palatinate (RLP), Mosellus area, Kobern-Gondorf, "Sürzer Höfe", ruderal area, N50°20'20" E07°25'25", 330m, 30-Sep-2011, hand-collecting, leg. Stüben,P., det. Stüben,P.
Cionus olivieri<br>Rosenschoeld, 1838	KC784033 [new]<br>509-RST<br>658nt	ZFMK-TIS-20294<br>ZFMK-DNA-0100446709	Czech Republic, Moravia mer., NP Podyji, Popicke kopecky hills, 3.5 km SW of Znojmo, N48°49'41.58" E16°1'10.50", 300m, 24-Jun-2011, *Verbascum thapsus*, beating, leg. Stejskal,R., det. Stejskal,R.
Cionus olivieri<br>Rosenschoeld, 1838	MK891389 [new]<br>1448-JKR<br>658nt	ZFMK-TIS-4217<br>ZFMK-DNA-0155628486	Slovakia, Nove Zamky, Buc, N47°46'55" E18°25'11", 109m, 28-Sep-2013, *Verbascum* sp., sweeping, leg. Krátky,J., det. Krátky,J.
Cionus scrophulariae<br>(Linnaeus, 1758)	MK891387 [new]<br>1442-JKR<br>658nt	ZFMK-TIS-4211<br>ZFMK-DNA-0155628480	Italy, Friuli-Venezia-Giulia, Aviano, Monte Cavalo, N46°22'19" E13°27'27", 1850m, 10-Aug-2013, *Scrophularia*, individual collecting by hand, leg. Krátky,J., det. Krátky,J.
Cionus thapsus<br>(Fabricius, 1792)	MK891534 [new]<br>1622-JKR<br>658nt	ZFMK-TIS-3670<br>ZFMK-DNA-0155635607	Slovakia, Nove Zamky, Kamenica nad Hronom, N47°49'34" E18°44'31", 109m, 19-Mai-2013, *Verbascum lichnitis*, individual collecting by hand, leg. Krátky,J., det. Krátky,J.
Cionus tuberculosus<br>(Scopoli, 1763)	MK891095 [new]<br>999-PSP<br>658nt	ZFMK-TIS-3144<br>ZFMK-DNA-0100426860	Germany, Lower Saxony (NI), Aerzen-Ahorn, Lkr. Hameln-Pyrmont, N52°03'17" E09°13'02", 171m, 05-Mai-2012, *Scrophularia nodosa*, wet forest track , dipnet, leg. Sprick,P., det. Sprick,P.
Cionus tuberculosus<br>(Scopoli, 1763)	MK891648 [new]<br>1822-PST<br>658nt	ZFMK-TIS-24127<br>ZFMK-DNA-0171624042	Germany, Rhineland-Palatinate (RLP), Ahr valley, Dernau, below Krausberg near Steinbergsmühle, N50°31'40" E07°02'43", 204m, 29-Mai-2014, beating, leg. Stüben,P., det. Stüben,P.
Cionus tuberculosus<br>(Scopoli, 1763)	MK891691 [new]<br>1908-FBA<br>658nt	ZFMK-TIS-23299<br>ZFMK-DNA-0169170375	Greece, Central Macedonia, Katerini S, Dion, N40°10'34" E22°29'38", 4m, 07-Aug-2014, leg. Bayer & Brunner, det. Bayer,F.

**Name**<br>**Authority**<br>**Additional Information**	**GenBank Acc No (Ref.)**<br>**Specimen ID**<br>**Sequence Length**	**ZFMK Tissue ID**<br>**ZFMK DNA Sample ID**<br>**(SDEI DNA Sample ID)**	**Locality, GPS, Collection Date, Plant, Collector, Identifier**
Cionus tuberculosus<br>(Scopoli, 1763)	MK891684 [new]<br>1900-PST<br>658nt	ZFMK-TIS-24005<br>ZFMK-DNA-0169170423	Netherlands, Elmpt, NP "DE of Meinweg", N51°11'08" E06°08'22", 75m, 28-Aug-2014, *Scrophularia*, beating, leg. Stüben,P., det. Stüben,P.
Cionus tuberculosus<br>(Scopoli, 1763)	MK891364 [new]<br>1415-JKR<br>658nt	ZFMK-TIS-4184<br>ZFMK-DNA-0155628518	Slovakia, Vysoke Tatry Mts., Dolina siedmich prame?ov, N49°13'38" E20°16'34", 1448m, 30-Jun-2013, *Scrophularia*, individual collecting by hand, leg. Krátky,J., det. Krátky,J.
Cionus variegatus<br>(Brullé, 1839)	KC784318 [40]<br>769-PST<br>658nt	ZFMK-TIS-3106<br>ZFMK-DNA-0100448118	Spain, Canary Islands, La Gomera, La Palmita, N28°10'23" W17°12'52", 643m, 07-Mrz-2012, *Verbascum virgatum*, beating, leg. Stüben,P., det. Stüben,P.
Cionus variegatus<br>(Brullé, 1839)	KC784303 [40]<br>736-PST<br>658nt	ZFMK-TIS-3073<br>ZFMK-DNA-0100448162	Spain, Canary Islands, Tenerife, La Guancha – Icod el Alto (surroundings of "Chafari"), N28°21'39" W16°38'32", 818m, 30-Jan-2012, *Scrophularia smithii langeana*, beating, leg. Stüben,P., det. Stüben,P.
Cistapion cyanescens<br>(Gyllenhal, 1833)	KC783850 [new]<br>173-PST<br>609nt	ZFMK-TIS-2D100440254<br>ZFMK-DNA-0100438142	Morocco, Atlas Mts., SW of Taza, Jbel Tazzeka near Bat-Bou-Idir, N34°03'09" W04°13'14", 1214m, 18-Mai-2011, beating, leg. Stüben,P., det. Behne,L.
Cleonis pigra<br>(Scopoli, 1763)	MK891937 [new]<br>2205-JKR<br>658nt	ZFMK-TIS-23943<br>ZFMK-DNA-0169170581	Czech Republic, Moravia, Znojmo, Popice, N48°49'26" E16°01'14", 280m, 03-Apr-2014, *Carduus acanthoides*, collecting by hand, leg. Stejskal,R., det. Stejskal,R.
Cleonis pigra<br>(Scopoli, 1763)	MK890901 [new]<br>643-PSP<br>658nt	ZFMK-TIS-20426<br>ZFMK-DNA-0100448624	Germany, Lower Saxony (NI), Koldingen, Hannover region, N52°16'23" E09°49'09", 56m, 10-Sep-2011, *Cirsium arvense*, Leine river wetland, beating, leg. Sprick,P., det. Sprick,P.
Cleonis pigra<br>(Scopoli, 1763)	MK890978 [new]<br>869-PST<br>658nt	ZFMK-TIS-3782<br>ZFMK-DNA-0100414219	Germany, Rhineland-Palatinate (RLP), Andernach, Nickenich, Krufter Ofen, pumice quarry, N50°23'39" E07°17'27", 277m, 25-Jul-2012, beating, leg. Stüben,P., det. Stüben,P.
Cleopomiarus graminis<br>(Gyllenhal, 1813)	KC784029 [new]<br>623-PSP<br>658nt	ZFMK-TIS-20406<br>ZFMK-DNA-0100446667	Germany, Bavaria (BY), Veitshöchheim; LWG-area, N49°50'20" E09°52'28", 185m, 03-Aug-2011, *Campanula rapunculoides*, beating, leg. Sprick,P., det. Stüben,P.
Cleopomiarus graminis<br>(Gyllenhal, 1813)	MK892039 [new]<br>2315-JKR<br>658nt	ZFMK-TIS-24246<br>ZFMK-DNA-0169170950	Slovakia, Tatry Mts., Dolina Siedmich prame?ov, N49°12'57" E20°17'46", 981m, 05-Jul-2014, *Campanula*, sweeping, leg. Krátky,J., det. Krátky,J.
Cleopomiarus micros<br>(Germar, 1821)	KC784191 [new]<br>469-PSP<br>658nt	ZFMK-TIS-20254<br>ZFMK-DNA-0100449370	Germany, Bavaria (BY), Buckenhof/Uttenreuth, Lkr. Erlangen-Höchstadt, N49°35'27" E11°05'04", 474m, 03-Jun-2011, *Jasione montana*, dipnet, leg. Sprick,P., det. Sprick,P.
Cleopus pulchellus<br>(Herbst, 1795)	MK891037 [new]<br>935-CBR<br>658nt	ZFMK-TIS-3848<br>ZFMK-DNA-0100449676	Luxembourg, 10 km E of Luxembourg, 1 km SW of Mensdorf, N49°38'55" E06°17'13", 260m, 17-Jun-2012, *Scropholaria nodosa*, pooter, leg. Braunert,C., det. Braunert,C., Note: two specimens in dataset, which show interspecific p-distance. Most likely 935-CBR is *C. pulchellus* and 1854-PST belongs to a cryptic *Cleopus* species.
Cleopus pulchellus<br>(Herbst, 1795)<br>-identification uncertain-	MK891659 [40]<br>1854-PST<br>658nt	ZFMK-TIS-26057<br>ZFMK-DNA-0171606112	Portugal, Madeira, near Boca da Encumeada, Folhadal, N32°45'10" W17°01'59", 1103m, 01-Jul-2014, *Scrophularia*, beating, leg. Stüben,P., det. Stüben,P. Note: most likely this specimen belongs to a cryptic species. Most likelly it will be described as *Cleopus maderensis* sp.n. soon.
Cleopus solani<br>(Fabricius, 1792)	MK891052 [new]<br>952-CBR<br>658nt	ZFMK-TIS-3865<br>ZFMK-DNA-0100449659	Luxembourg, Luxembourg, N49°38'00" E06°10'45", 345m, 03-Jul-2012, *Verbascum*, beating, leg. Braunert,C., det. Braunert,C.

**Name**<br>**Authority**<br>**Additional Information**	**GenBank Acc No (Ref.)**<br>**Specimen ID**<br>**Sequence Length**	**ZFMK Tissue ID**<br>**ZFMK DNA Sample ID**<br>**(SDEI DNA Sample ID)**	**Locality, GPS, Collection Date, Plant, Collector, Identifier**
Cleopus solani<br>(Fabricius, 1792)	MK892245 [new]<br>2544-PSP<br>658nt	ZFMK-TIS-23518<br>ZFMK-DNA-0171662015	Slovakia, Nitra, Cenkov, N47°46'07" E18°31'11", 109m, 19-Mai-2014, *Verbascum* sp., leg. Sprick,P., det. Sprick,P.
Cnoerhinus serranoi<br>(Alonso-Zarazaga, 1988)	MK890970 [new]<br>853-PST<br>658nt	ZFMK-TIS-3766<br>ZFMK-DNA-0100414238	Portugal, Alentejo, Serra da Arràbida, Outao, N38°30'05" W08°56'20", 70m, 20-Mai-2012, *Arbutus, Smilax*, beating, leg. Stüben,P., det. Stüben,P.
Coeliastes lamii<br>(Fabricius, 1792)	KC783958 [new]<br>301-JKR<br>658nt	ZFMK-TIS-20087<br>ZFMK-DNA-0100438919	Czech Republic, Bohemia or., Dritec, N50°7'11.648" E15°48'6.919", 230m, 06-Mai-2011, sweeping, leg. Krátky,J., det. Krátky,J.
Coeliastes lamii<br>(Fabricius, 1792)	MK891086 [new]<br>989-PSP<br>658nt	ZFMK-TIS-3134<br>ZFMK-DNA-0100426867	Germany, Lower Saxony (NI), Hannover, Herrenhausen, N52°23'02" E09°41'25", 50m, 29-Apr-2012, *Lamium album*, Leine valley, dipnet, leg. Sprick,P., det. Sprick,P.
Coeliastes lamii<br>(Fabricius, 1792)	MK891972 [new]<br>2243-JKR<br>658nt	ZFMK-TIS-23981<br>ZFMK-DNA-0169170544	Slovakia, Zilina, Suja, N49°03'46" E18°37'19", 474m, 21-Jun-2014, *Lamium purpuraeum*, sweeping, leg. Krátky,J., det. Krátky,J.
Coeliodes rana<br>(Fabricius, 1787)	MK891680 [new]<br>1896-PST<br>658nt	ZFMK-TIS-24001<br>ZFMK-DNA-0169168519	Germany, North Rhine-Westphalia (NRW), Elmpt, NSG "Elmpter Schwalmbruch", Venekotensee, N51°13'43" E06°06'59", 35m, 27-Aug-2014, *Quercus robur*, beating, leg. Stüben,P., det. Stüben,P.
Coeliodes ruber<br>(Marsham, 1802)	MK891078 [new]<br>981-PSP<br>658nt	ZFMK-TIS-3126<br>ZFMK-DNA-0100426878	Germany, Lower Saxony (NI), near Hannover, Brelingen, Klagesberg, N52°34'13" E09°42'02", 83m, 16-Apr-2012, *Quercus robur*, edge of mixed deciduous woodland, beating, leg. Sprick,P., det. Sprick,P.
Coeliodes ruber<br>(Marsham, 1802)	MK892192 [new]<br>2487-PSP<br>658nt	ZFMK-TIS-23459<br>ZFMK-DNA-0171661777	Germany, Lower Saxony (NI), Hannover region, Brelingen, N52°33'52" E09°41'09", 75m, 18-Apr-2014, *Quercus robur*, beating, leg. Sprick,P., det. Sprick,P.
Coeliodes ruber<br>(Marsham, 1802)	MK891769 [new]<br>2026-JKR<br>658nt	ZFMK-TIS-23666<br>ZFMK-DNA-0169170475	Italy, Sicilia Isl. (PA), Palermo, Bosco di Ficuzza, N37°51'50" E13°24'56", 995m, 22-Apr-2014, *Quercus*, beating, leg. Krátky,J., det. Krátky,J.
Coeliodes sp.	GU981474 [01]<br>M-0053-CoS<br>658nt	ZFMK-TIS-cM0053<br>ZFMK-DNA-0100400049	Germany, Rhineland-Palatinate (RLP), Moselle valley, 4 km S of Cochem, N of Eller-Edinger, "Calmond", N50°06'52" E07°08'39", 273m, 16-Sep-2005, *Carpinus*, leg. Stüben,P., det. Stüben,P.
Coeliodes transversealbofasciatus<br>(Goeze, 1777)	MK891917 [new]<br>2185-JKR<br>658nt	ZFMK-TIS-23923<br>ZFMK-DNA-0169170609	Czech Republic, Moravia, Vevcice, N48°58'08.5" E16°01'31", 325m, 04-Apr-2014, *Quercus* sp., beating, leg. Stejskal,R., det. Stejskal,R.
Coeliodes transversealbofasciatus<br>(Goeze, 1777)	MK891075 [new]<br>978-PSP<br>658nt	ZFMK-TIS-3123<br>ZFMK-DNA-0100426881	Germany, Lower Saxony (NI), near Hannover, Brelingen, N52°33'35" E09°41'04", 64m, 05-Apr-2012, *Quercus robur*, group of oak trees, beating, leg. Sprick,P., det. Sprick,P.
Coeliodes transversealbofasciatus<br>(Goeze, 1777)	KC784250 [new]<br>IT-0017w<br>652nt	ZFMK-DNA-0100437863	Italy, Sardinia Isl., S of Aritzo, Gadoni, R. Tistigliosi x F. Flumendosa, N39°53'57" E09°11'02", 490m, 30-Sep-2010, *Quercus ilex*, leg. Stüben,P., det. Stüben,P.
Coeliodes transversealbofasciatus<br>(Goeze, 1777)	MK891339 [new]<br>1372-PST<br>658nt	ZFMK-TIS-4668<br>ZFMK-DNA-0155628554	Italy, Abruzzo, NE of Capestrano near Ofena, N42°16'53" E13°49'20", 940m, 17-Aug-2013, sieving, leg. Stüben,P., det. Stüben,P.
Coeliodes transversealbofasciatus<br>(Goeze, 1777)	MK890990 [new]<br>882-CBR<br>658nt	ZFMK-TIS-3795<br>ZFMK-DNA-0100449729	Luxembourg, 10 km E of Luxembourg, 2 km S of Mensdorf, N49°38'09" E06°17'48", 330m, 11-Apr-2011, *Quercus*, beating, leg. Braunert,C., det. Braunert,C.

**Name**<br>**Authority**<br>**Additional Information**	**GenBank Acc No (Ref.)**<br>**Specimen ID**<br>**Sequence Length**	**ZFMK Tissue ID**<br>**ZFMK DNA Sample ID**<br>**(SDEI DNA Sample ID)**	**Locality, GPS, Collection Date, Plant, Collector, Identifier**
Coeliodinus rubicundus<br>(Herbst, 1795)	MK891465 [new]<br>1532-PSP<br>658nt	ZFMK-TIS-3292<br>ZFMK-DNA-0155633297	Germany, Lower Saxony (NI), Brelingen, Hannover region, Klagesberg, N52°34'02" E09°41'40", 84m, 16-Apr-2012, *Betula pendula*, edge of a mixed deciduous woodland, beating, leg. Sprick,P., det. Sprick,P.
Coelositona cambricus<br>(Stephens, 1831)	MK891966 [new]<br>2236-JKR<br>658nt	ZFMK-TIS-23974<br>ZFMK-DNA-0169170551	Czech Republic, Bohemia, Uborsko, N49°19'29" E13°09'42", 427m, 27-Sep-2014, *Lotus uliginosus*, leg. Benedikt,S., det. Benedikt,S.
Coelositona cambricus<br>(Stephens, 1831)	MK891477 [new]<br>1547-PSP<br>658nt	ZFMK-TIS-3307<br>ZFMK-DNA-0155633289	Germany, Lower Saxony (NI), Harz National Park, Bad Harzburg, Ottenhai, N51°53'19" E10°35'50", 307m, 20-Sep-2013, *Lotus peduculatus*, clearing, beating, leg. Sprick,P., det. Sprick,P.
Coelositona latipennis<br>(Gyllenhal, 1834)<br>formerly: C. latipennis latipennis	KC783802 [40]<br>95-PST<br>658nt	ZFMK-TIS-2D100446960<br>ZFMK-DNA-0100437952	Spain, Canary Islands, La Gomera, S of Hermigua, La Palmita, N28°10'33" W17°12'37", 632m, 07-Feb-2011, *Fabaceae*, beating, leg. Stüben,P., det. Stüben,P.
Coelositona latipennis<br>(Gyllenhal, 1834)<br>formerly: C. latipennis latipennis	MK891655 [40]<br>1835-PST<br>658nt	ZFMK-TIS-26038<br>ZFMK-DNA-0171606120	Portugal, Madeira, SW of Santana, Achada do Teixeira, N32°45'51" W16°55'12", 1589m, 28-Jun-2014, *Cytisus scoparius*, beating, leg. Stüben,P., det. Stüben,P.
Coelositona latipennis<br>(Gyllenhal, 1834)<br>formerly: C. latipennis latipennis	MK891657 [40]<br>1850-PST<br>658nt	ZFMK-TIS-26053<br>ZFMK-DNA-0171606116	Portugal, Madeira, S of Ribeira da Janela, S of Fanal near Pico Queimado, N32°47'30" W17°07'44", 1270m, 30-Jun-2014, beating, leg. Stüben,P., det. Stüben,P.
Coelositona latipennis<br>(Gyllenhal, 1834)<br>formerly: C. latipennis latipennis	MK891667 [40]<br>1869-PST<br>658nt	ZFMK-TIS-26072<br>ZFMK-DNA-0171606106	Portugal, Madeira, SW of Santana, Queimadas, Levada do Caldeirao Verde, N32°46'42" W16°54'39", 1053m, 05-Jul-2014, *Laurisilva*, beating, leg. Stüben,P., det. Stüben,P.
Coelositona latipennis<br>(Gyllenhal, 1834)<br>formerly: C. latipennis latipennis	MK891711 [40]<br>1937-PST<br>658nt	ZFMK-TIS-24016<br>ZFMK-DNA-0169170415	Portugal, Madeira, Faial, coast, N32°47'37" W16°50'57", 26m, 04-Okt-2014, sieving, leg. Stüben,P., det. Stüben,P.
Coelositona latipennis<br>(Gyllenhal, 1834)<br>formerly: C. latipennis latipennis	MK892374 [40]<br>2826-PST<br>658nt	ZFMK-TIS-25873<br>ZFMK-DNA-FC17941469	Portugal, Madeira, NE of Faja da Ovelha, N32°46'36" W17°13'14", 673m, 24-Nov-2015, *Ulex europaeus*, beating, leg. Stüben,P. & Schütte,A., det. Stüben,P.
Coelositona palmensis<br>(Har. Lindberg, 1953)<br>formerly: C. latipennis palmensis	MK891482 [40]<br>1552-JKR<br>658nt	ZFMK-TIS-3600<br>ZFMK-DNA-0155635677	Spain, Canary Islands, La Palma, Llano de las Vacas, N28°43'29" W17°47'59", 1340m, 26-Jan-2013, *Adenocarpus*, beating, leg. Krátky,J., det. Krátky,J.
Coelositona palmensis<br>(Har. Lindberg, 1953)<br>formerly: C. latipennis palmensis	MK891603 [40]<br>1687-PST<br>658nt	ZFMK-TIS-24405<br>ZFMK-DNA-0155622503	Spain, Canary Islands, La Palma, near San Isidro, N28°37'47" W17°48'02", 643m, 18-Dez-2013, *Tagasaste (Fabaceae)*, beating, leg. Stüben,P., det. Stüben,P.
Coelositona palmensis<br>(Har. Lindberg, 1953)<br>formerly: C. latipennis palmensis	MK891608 [40]<br>1692-PST<br>658nt	ZFMK-TIS-24410<br>ZFMK-DNA-0155622498	Spain, Canary Islands, La Palma, south of Jedey, N28°32'24" W17°52'00", 780m, 19-Dez-2013, *Fabaceae*, beating, leg. Stüben,P., det. Stüben,P.

**Name**<br>**Authority**<br>**Additional Information**	**GenBank Acc No (Ref.)**<br>**Specimen ID**<br>**Sequence Length**	**ZFMK Tissue ID**<br>**ZFMK DNA Sample ID**<br>**(SDEI DNA Sample ID)**	**Locality, GPS, Collection Date, Plant, Collector, Identifier**
Coelositona palmensis<br>(Har. Lindberg, 1953)<br>formerly: C. latipennis palmensis	MK891611 [40]<br>1702-PST<br>658nt	ZFMK-TIS-24420<br>ZFMK-DNA-0155622483	Spain, Canary Islands, La Palma, N of Santa Cruz, M. de Tagoja, N28°43'18" W17°47'07", 1047m, 21-Dez-2013, *Tagasaste (Fabaceae)*, beating, leg. Stüben,P., det. Stüben,P.
Coelositona palmensis<br>(Har. Lindberg, 1953)<br>formerly: C. latipennis palmensis	MK891614 [40]<br>1718-PST<br>658nt	ZFMK-TIS-24436<br>ZFMK-DNA-0155622472	Spain, Canary Islands, La Palma, Montes de Luna, N28°31'57" W17°48'37", 583m, 28-Dez-2013, *Tagasaste (Fabaceae)*, beating, leg. Stüben,P., det. Stüben,P.
Coelositona puberulus<br>(Reitter, 1903)	MK891661 [40]<br>1856-PST<br>658nt	ZFMK-TIS-26059<br>ZFMK-DNA-0171606110	Portugal, Madeira, NE of Arco da Calheta, N32°44'37" W17°07'47", 1157m, 02-Jul-2014, *Lotus*, beating, leg. Stüben,P., det. Stüben,P.
Coelositona puberulus<br>(Reitter, 1903)	MK891675 [40]<br>1884-PST<br>658nt	ZFMK-TIS-26087<br>ZFMK-DNA-0171606073	Portugal, Madeira, near Ilha, W of Sao Jorge, Achada do Marques, N32°48'18" W16°54'28", 404m, 14-Jul-2014, *Lotus*, beating, leg. Stüben,P., det. Stüben,P.
Coelositona sp.	MK891596 [40]<br>1673-PST<br>658nt	ZFMK-TIS-24391<br>ZFMK-DNA-0155622517	Spain, Canary Islands, La Gomera, Los Acevinos, N28°08'56" W17°13'38", 922m, 06-Dez-2013, beating, leg. Stüben,P., det. Stüben,P.
Coelositona verrucosus<br>(Brullé, 1839)<br>formerly: *C. latipennis* - Resyn!	KC783741 [40]<br>19-PST<br>658nt	ZFMK-TIS-2D100447029<br>ZFMK-DNA-0100438101	Spain, Canary Islands, Gran Canaria, Moya, Barranco de los Tilos, N28°04'33" W15°36'05", 643m, 07-Dez-2010, *Chamaecytiscus proliferus*, beating, leg. Stüben,P., det. Stüben,P.
Coelositona verrucosus<br>(Brullé, 1839)<br>formerly: *C. latipennis* - Resyn!	MK891563 [40]<br>1660-PST<br>658nt	ZFMK-TIS-4704<br>ZFMK-DNA-0155630475	Spain, Canary Islands, Tenerife, La Montaneta, N28°20'32" W16°45'32", 913m, 05-Jan-2012, *Chamaecytisus*, beating, leg. Stüben,P., det. Stüben,P.
Coiffaitiella cf. *remilleti*	KC784279 [new]<br>690-PST<br>658nt	ZFMK-TIS-3027<br>ZFMK-DNA-0100446736	Spain, Cadiz, Los Barrios, Parque los Alcornocales, Arroyo Valdeinfierno , N36°13'49.08" W05°35'47.34", 136m, 20-Mrz-2012, *Quercus canariensis*, leg. Torres,J. L., det. Stüben,P.
Coloracalles edoughensis<br>(Desbrochers, 1892)<br>formerly: *Acalles edoughensis*	GU987957 [01]<br>E-611-edo<br>658nt	ZFMK-TIS-cE611<br>ZFMK-DNA-0100404293	Spain, Barcelona, near Vallirana, N41°22'36" E01°55'02", 245m, 22-Jun-2008, *Quercus ilex, Ficus carica, Smilax aspera* , above dry river bed, leg. Astrin,J., det. Stüben,P.
Coloracalles humerosus<br>(Fairmaire, 1862)	EU286460 [01]<br>F-0098-hum<br>658nt	ZFMK-TIS-cF0098<br>ZFMK-DNA-0100400698	France, Gard, 15 km NE of Nimes, Pont du Gard, Collias, N43°57'03" E04°28'59", 68m, 13-Apr-2006, *Quercus ilex*, wet forest, leg. Stüben,P., det. Stüben,P.
Coloracalles humerosus<br>(Fairmaire, 1862)	MG229703 [new]<br>1407-JKR<br>658nt	ZFMK-TIS-4176<br>ZFMK-DNA-0155628526	France, Cevennes Mts., 1 km N of Pommiers, N43°57'43" E03°36'44", 456m, 13-Jun-2013, *Castanea sativa*, sieving, leg. Krátky,J., det. Krátky,J.
Coloracalles humerosus<br>(Fairmaire, 1862)	GU987952 [01]<br>E-606-hum<br>658nt	ZFMK-TIS-cE606<br>ZFMK-DNA-0100404928	Spain, Castellon, Morella, Barranco de la Bota, N40°33'12" W00°00'27", 814m, 21-Jun-2008, *Quercus ilex, Hedera helix*, leg. Astrin,J., det. Stüben,P.
Coloracalles humerosus<br>(Fairmaire, 1862)	GU987955 [01]<br>E-609-hum<br>629nt	ZFMK-TIS-cE609<br>ZFMK-DNA-0100404931	Spain, Barcelona, near Vallirana, N41°22'04" E01°55'05", 22-Jun-2008, *Silax aspera, Hedera helix, Laurus*, basin within quarry, leg. Astrin,J., det. Stüben,P.

**Name**<br>**Authority**<br>**Additional Information**	**GenBank Acc No (Ref.)**<br>**Specimen ID**<br>**Sequence Length**	**ZFMK Tissue ID**<br>**ZFMK DNA Sample ID**<br>**(SDEI DNA Sample ID)**	**Locality, GPS, Collection Date, Plant, Collector, Identifier**
Coniatus repandus<br>(Fabricius, 1792)	KC783898 [new]<br>111-PST<br>658nt	ZFMK-TIS-2D100440316<br>ZFMK-DNA-0100438389	Morocco, S of Berkane, Beni Snassen Mts., Vallee Zegzel, N34°52'31" W02°21'32", 82m, 11-Mai-2011, *Tamarix*, beating, leg. Stüben,P., det. Stüben,P./vid. Behne,L.
Coniatus repandus<br>(Fabricius, 1792)	MK891313 [new]<br>1305-PST<br>658nt	ZFMK-TIS-4121<br>ZFMK-DNA-0100426174	Spain, Cordoba, Montoro, N38°01'49" W04°22'46", 161m, 04-Mai-2013, *Tamarix*, beating, leg. Stüben,P. & Schütte,A., det. Stüben,P.
Coniatus tamarisci<br>(Fabricius, 1787)	KC783834 [new]<br>IT-0036w<br>636nt	ZFMK-TIS-2D100446521<br>ZFMK-DNA-0100437833	Italy, Sardinia Isl. East, E of Siniscola, La Caletta, coast, N40°35'30" E09°45'25", 3m, 09-Okt-2010, *Tamarix*, leg. Stüben,P., det. Stüben,P.
Coniatus tamarisci<br>(Fabricius, 1787)	KC784251 [new]<br>IT-0018w<br>655nt	ZFMK-TIS-2D100446536<br>ZFMK-DNA-0100437864	Italy, Sardinia Isl. South, SE of Ballao, F. Flumendosa, N39°31'51" E09°23'27", 75m, 01-Okt-2010, *Tamarix*, leg. Stüben,P., det. Stüben,P.
Coniatus tamarisci<br>(Fabricius, 1787)	MK891771 [new]<br>2028-JKR<br>658nt	ZFMK-TIS-23668<br>ZFMK-DNA-0169170473	Italy, Sicilia Isl. (PA), Palermo, 7 km N of Corleone, N37°52'47" E13°19'01", 522m, 22-Apr-2014, *Tamarix*, beating, leg. Krátky,J., det. Krátky,J.
Coniocleonus excoriatus<br>(Gyllenhal, 1834)	KC783864 [new]<br>149-PST<br>658nt	ZFMK-TIS-2D100440278<br>ZFMK-DNA-0100438166	Morocco, NW of Berkane, Oued Moulouya, N34°56'49" W02°33'08", 49m, 14-Mai-2011, sieving, leg. Stüben,P., det. Stüben,P./vid. Behne,L.
Coniocleonus nigrosuturatus<br>(Goeze, 1777)	KC784113 [new]<br>495-RST<br>658nt	ZFMK-TIS-20280<br>ZFMK-DNA-0100448484	Romania, Caras-Severin, Sfanta Elena env., N44°41'11.88" E21°41'46.47", 350m, 29-Apr-2011, hand-collecting, leg. Stejskal,R., det. Stejskal,R.
Coniocleonus pseudobliquus<br>(G. Müller, 1921)	KC784121 [new]<br>662-FBA<br>658nt	ZFMK-TIS-20444<br>ZFMK-DNA-0100448605	Greece, Chalcidice, Pyrgos, Sani env., (16 km Snea Moudania), N40°06'11" E23°18'43", 6m, 10-Okt-2010, leg. Bahr,F. & Winkelmann,H., det. Bahr,F.
Corimalia lunulata<br>(Wollaston, 1863)	KC783737[40]<br>13-PST<br>656nt	ZFMK-TIS-2D100447023<br>ZFMK-DNA-0100437959	Spain, Canary Islands, Gran Canaria, Telde, El Palmital, N28°01'10" W15°27'17", 476m, 05-Dez-2010, *Tamarix canariensis*, beating, leg. Stüben,P., det. Stüben,P.
Corimalia pallida<br>(Olivier, 1807)	KC784252 [new]<br>IT-0020w<br>655nt	ZFMK-TIS-2D100446557<br>ZFMK-DNA-0100433862	Italy, Sardinia Isl. South, SE of Ballao, F. Flumendosa, N39°31'51" E09°23'27", 75m, 01-Okt-2010, *Tamarix*, leg. Stüben,P., det. Stüben,P.
Corimalia pallida<br>(Olivier, 1807)	KC784266 [new]<br>IT-0038w<br>625nt	ZFMK-DNA-0100437835	Italy, Sardinia Isl. East, E of Siniscola, La Caletta, coast, N40°35'30" E09°45'25", 3m, 09-Okt-2010, *Tamarix*, leg. Stüben,P., det. Stüben,P.
Corimalia schatzmayri<br>Giordani-Soika, 1937	MK892347 [40]<br>2723-PST<br>658nt	ZFMK-TIS-23873<br>ZFMK-DNA-0171661279	Spain, Canary Islands, Fuerteventura, Betancuria, N28°25'09" W14°03'32", 380m, 04-Jan-2015, *Tamarix*, beating, leg. Stüben,P., det. Stüben,P.
Corimalia schatzmayri<br>Giordani-Soika, 1937	MK892348 [40]<br>2731-PST<br>658nt	ZFMK-TIS-23881<br>ZFMK-DNA-0171661268	Spain, Canary Islands, Fuerteventura, Barranco de los Molinos, N28°32'34" W14°03'41", 9m, 06-Jan-2015, *Tamarix*, beating, leg. Stüben,P., det. Stüben,P.
Corimalia sp.	KC783861 [new]<br>154-PST<br>658nt	ZFMK-TIS-2D100440273<br>ZFMK-DNA-0100438161	Morocco, NW of Berkane, Oued Moulouya, N35°00'03" W02°27'30" , 15m, 14-Mai-2011, *Tamarix*, beating, leg. Stüben,P., det. Behne,L.
Corimalia tamarisci<br>(Gyllenhal, 1838)	KC784170 [new]<br>491-PST<br>658nt	ZFMK-TIS-20276<br>ZFMK-DNA-0100449042	France, Vaucluse, Avignon, N43°56'20" E04°50'47", 30m, 25-Jul-2011, *Tamarix*, beating, leg. Stüben,P., det. Stüben,P.

**Name**<br>**Authority**<br>**Additional Information**	**GenBank Acc No (Ref.)**<br>**Specimen ID**<br>**Sequence Length**	**ZFMK Tissue ID**<br>**ZFMK DNA Sample ID**<br>**(SDEI DNA Sample ID)**	**Locality, GPS, Collection Date, Plant, Collector, Identifier**
Corimalia tamarisci<br>(Gyllenhal, 1838)	MK890805 [new]<br>113-PST<br>658nt	ZFMK-TIS-2D100440303<br>ZFMK-DNA-0100438191	Morocco, S of Berkane, Beni Snassen Mts., Vallee Zegzel, N34°52'31" W02°21'32", 82m, 11-Mai-2011, *Tamarix*, beating, leg. Stüben,P., det. Stüben,P./vid. Behne,L.
Coryssomerus capucinus<br>(Beck, 1817)	MK891859 [new]<br>2121-JKR<br>658nt	ZFMK-TIS-23761<br>ZFMK-DNA-0169169612	Czech Republic, Bohemia, Hradec Kralove, Borovinka, N50°11'24" E15°47'60", 228m, 15-Mai-2014, *Matricaia inodora*, sweeping, leg. Krátky,J., det. Krátky,J.
Coryssomerus capucinus<br>(Beck, 1817)	MK891113 [new]<br>1018-PSP<br>658nt	ZFMK-TIS-3163<br>ZFMK-DNA-0100426848	Germany, Lower Saxony (NI), Hannover, Nordhafen, N52°25'24.6" E09°38'53", 53m, 12-Mai-2012, *Artemisia vulgaris, Tripleurospermum perforatum*, south exposed slope with annual ruderal area, beating, leg. Sprick,P., det. Sprick,P.
Coryssomerus capucinus<br>(Beck, 1817)	MK891224 [new]<br>1144-JKR<br>658nt	ZFMK-TIS-3480<br>ZFMK-DNA-0100449564	Slovakia, Nove Zamky, Kamenica nad Hronom, N47°49'52" E18°44'20", 181m, 12-Mai-2012, sweeping, leg. Krátky,J., det. Krátky,J.
Coryssomerus capucinus<br>(Beck, 1817)	MK891528 [new]<br>1616-JKR<br>658nt	ZFMK-TIS-3664<br>ZFMK-DNA-0155635614	Slovakia, Komarno, B?c env., N47°46'55" E18°25'11", 109m, 18-Mai-2013, sweeping, leg. Krátky,J., det. Krátky,J.
Cosmobaris scolopacea<br>(Germar, 1819)	MK891556 [new]<br>1648-JKR<br>658nt	ZFMK-TIS-4226<br>ZFMK-DNA-0155630492	Romania, Buzau, Vulcanii Noroisi-Paclele Mici, N45°20'38" E26°42'37", 300m, 09-Jun-2013, sweeping, leg. Pelikan,J., det. Krátky,J.
Cryptorhynchinae sp.	MG229843 [new]<br>X-0427-Cry<br>658nt	ZFMK-TIS-24521<br>ZFMK-DNA-0155630402	Guatemala, Zacapa, 14 km NNE of Tuculutan, , 2270m, 06-Jul-2007, leg. Longino,J. with Anderson,R., det. Longino,J. with Anderson,R.
Cryptorhynchinae sp.	MG229844 [new]<br>X-0428-Cry<br>658nt	ZFMK-TIS-24524<br>ZFMK-DNA-0155630002	Mexico, Chiapas, Reserva Huitepec, , 2600m, 11-Jul-2007, leg. Longino,J. with Anderson,R., det. Longino,J. with Anderson,R.
Cryptorhynchus lapathi<br>(Linnaeus, 1758)	EU286523 [01]<br>D-0354-lap<br>658nt	ZFMK-TIS-cD0354<br>ZFMK-DNA-0100400101	Germany, North Rhine-Westphalia (NRW), Bienen near Rees, old arm of Rhine river, N51°46' E06°20', 19-Mai-2004, leg. Scharf,S., det. Scharf,S.
Cryptorhynchus lapathi<br>(Linnaeus, 1758)	MG229781 [new]<br>2400-JKR<br>658nt	ZFMK-TIS-25977<br>ZFMK-DNA-0171600622	Slovakia, Zilina, Suja, N49°03'46" E18°37'19", 474m, 21-Jun-2014, *Salix* sp., sweeping, leg. Krátky,J., det. Krátky,J.
Cucubaris villae<br>Comolli, 1837	MK891887 [new]<br>2152-JKR<br>658nt	ZFMK-TIS-23792<br>ZFMK-DNA-0169169581	Slovakia, Nove Zamky, Jursky Chlm, N47°48'04" E18°31'27", 114m, 19-Mai-2014, *Bryonia alba*, collecting by hand, leg. Krátky,J., det. Krátky,J.
Curculio betulae<br>(Stephens, 1831)	MK891437 [new]<br>1500-PSP<br>658nt	ZFMK-TIS-3260<br>ZFMK-DNA-0155633338	Germany, Lower Saxony (NI), Meitze - Hellendorf, Hannover region, N52°33'36" E09°44'30", 48m, 03-Aug-2012, *Alnus glutinosa*, beating, leg. Sprick,P., det. Sprick,P.
Curculio elephas<br>(Gyllenhal, 1835)	KC784065 [new]<br>557-RST<br>658nt	ZFMK-TIS-20342<br>ZFMK-DNA-0100448419	Czech Republic, Moravia mer., Podyji NP, Kravi hora hill, 1 km SW of Znojmo, N48°50'56.89" E16°2'26.53", 320m, 10-Sep-2011, *Quercus*, beating, leg. Stejskal,R., det. Stejskal,R.
Curculio elephas<br>(Gyllenhal, 1835)	MK892127 [new]<br>2406-JKR<br>658nt	ZFMK-TIS-25983<br>ZFMK-DNA-0171600631	Slovakia, Cerova vrchovina, Gemersky Jablonec, N48°12'59" E19°59'52", 292m, 17-Aug-2014, *Quercus cerris*, beating, leg. Pavel,F., det. Krátky,J.
Curculio elephas<br>(Gyllenhal, 1835)	MK892129 [new]<br>2408-JKR<br>658nt	ZFMK-TIS-25985<br>ZFMK-DNA-0171600629	Slovakia, Cerova vrchovina, Hajnacka, N48°13'40" E19°58'25", 377m, 17-Aug-2014, *Quercus cerris*, beating, leg. Pavel,F., det. Krátky,J.

**Name**<br>**Authority**<br>**Additional Information**	**GenBank Acc No (Ref.)**<br>**Specimen ID**<br>**Sequence Length**	**ZFMK Tissue ID**<br>**ZFMK DNA Sample ID**<br>**(SDEI DNA Sample ID)**	**Locality, GPS, Collection Date, Plant, Collector, Identifier**
Curculio glandium<br>Marsham, 1802	KC784066 [new]<br>558-RST<br>614nt	ZFMK-TIS-20343<br>ZFMK-DNA-0100448420	Czech Republic, Moravia mer., Podyji NP, Kravi hora hill, 1 km SW of Znojmo, N48°50'56.89" E16°2'26.53", 320m, 10-Sep-2011, *Quercus*, beating, leg. Stejskal,R., det. Stejskal,R.
Curculio glandium<br>Marsham, 1802	KC784023 [new]<br>215-PSP<br>658nt	ZFMK-TIS-2D100439157<br>ZFMK-DNA-0100439829	Germany, Lower Saxony (NI), Brelingen, Hannover region, N52°33'35" E09°41'04", 70m, 16-Apr-2011, *Quercus robur*, sand pit, beating, leg. Sprick,P., det. Sprick,P.
Curculio glandium<br>Marsham, 1802	MK890989 [new]<br>881-CBR<br>658nt	ZFMK-TIS-3794<br>ZFMK-DNA-0100449730	Luxembourg, 10 km E of Luxembourg, 2 km S of Mensdorf, N49°38'09" E06°17'48", 330m, 11-Apr-2011, *Quercus*, beating, leg. Braunert,C., det. Braunert,C.
Curculio glandium<br>Marsham, 1802	MK892130 [new]<br>2409-JKR<br>658nt	ZFMK-TIS-25986<br>ZFMK-DNA-0171600628	Slovakia, Cerova vrchovina, Hajnacka, N48°13'40" E19°58'25", 377m, 17-Aug-2014, *Quercus cerris*, beating, leg. Pavel,F., det. Krátky,J.
Curculio nucum<br>Linnaeus, 1758	MK891137 [new]<br>1042-PSP<br>658nt	ZFMK-TIS-3187<br>ZFMK-DNA-0100426824	Germany, Lower Saxony (NI), Königsförde, Lkr. Hameln-Pyrmont, N52°03'54.5" E09°16'22", 90m, 27-Mai-2012, *Corylus avellana*, house garden, beating, leg. Sprick,P., det. Sprick,P.
Curculio pellitus<br>(Boheman, 1843)	MK892166 [new]<br>2457-PSP<br>658nt	ZFMK-TIS-23429<br>ZFMK-DNA-0171661816	Germany, Lower Saxony (NI), Hameln-Rohrsen, Dütberg, N52°06'21" E09°24'53", 102m, 10-Mai-2013, *Quercus robur*, beating, leg. Sprick,P., det. Sprick,P.
Curculio propinquus<br>(Desbrochers des Loges, 1868)	MK892128 [new]<br>2407-JKR<br>658nt	ZFMK-TIS-25984<br>ZFMK-DNA-0171600630	Slovakia, Cerova vrchovina, Hajnacka, N48°13'40" E19°58'25", 377m, 17-Aug-2014, *Quercus cerris*, beating, leg. Pavel,F., det. Krátky,J.
Curculio propinquus<br>(Desbrochers des Loges, 1868)	MK892131 [new]<br>2410-JKR<br>658nt	ZFMK-TIS-25987<br>ZFMK-DNA-0171600627	Slovakia, Cerova vrchovina, Gemersky Jablonec, N48°12'59" E19°59'52", 292m, 17-Aug-2014, *Quercus cerris*, beating, leg. Pavel,F., det. Krátky,J.
Curculio venosus venosus<br>(Gravenhorst, 1807)	KC783903 [new]<br>224-PSP<br>658nt	ZFMK-TIS-2D100438518<br>ZFMK-DNA-0100438521	Germany, Lower Saxony (NI), Everloh, Hannover region, Benther Berg, N52°20'01" E09°36'58", 100m, 22-Apr-2011, *Quercus robur*, edge of the forest, beating, leg. Sprick,P., det. Sprick,P.
Curculio venosus venosus<br>(Gravenhorst, 1807)	MK891021 [new]<br>918-CBR<br>658nt	ZFMK-TIS-3831<br>ZFMK-DNA-0100449692	Luxembourg, 15 km SW of Wiltz, 2 km SSW of Boulaide, N49°52'16" E05°48'26", 400m, 01-Jul-2011, *Quercus*, beating, leg. Braunert,C., det. Braunert,C.
Curculio venosus venosus<br>(Gravenhorst, 1807)	MK892052 [new]<br>2328-JKR<br>658nt	ZFMK-TIS-24259<br>ZFMK-DNA-0169170946	Slovakia, Tatry Mts., Bujaci vrch, N49°14'10" E20°14'27", 1914m, 06-Jul-2014, *mountain meadow*, sweeping, leg. Krátky,J., det. Stüben,P.
Curculio villosus<br>Fabricius, 1781	MK890824 [new]<br>230-PSP<br>658nt	ZFMK-TIS-2D100438550<br>ZFMK-DNA-0100439813	Germany, Lower Saxony (NI), Lemmie, Hannover region, Gehrdener Berg, N52°17'44" E09°35'23", 100m, 22-Apr-2011, *Quercus robur*, field edge, beating, leg. Sprick,P., det. Sprick,P.
Curculio villosus<br>Fabricius, 1781	MK892191 [new]<br>2486-PSP<br>658nt	ZFMK-TIS-23458<br>ZFMK-DNA-0171661776	Germany, Lower Saxony (NI), Hannover region, Brelingen, N52°33'52" E09°41'09", 75m, 18-Apr-2014, *Quercus robur*, beating, leg. Sprick,P., det. Sprick,P.
Cyanapion columbinum<br>(Germar, 1817)	MK892094 [new]<br>2371-JKR<br>658nt	ZFMK-TIS-25948<br>ZFMK-DNA-0171600593	Czech Republic, Moravia, Znojmo, N48°51'13" E16°06'34", 250m, 23-Aug-2014, sweeping, leg. R. Stejskal, det. Stejskal,R.
Cyanapion columbinum<br>(Germar, 1817)	KC784061 [new]<br>572-PSP<br>658nt	ZFMK-TIS-20357<br>ZFMK-DNA-0100448407	Germany, Lower Saxony (NI), Neindorf, Lkr. Wolfenbüttel, Öselberg, N52°07'26" E10°35'37", 122m, 24-Jun-2011, *Lathyrus tuberosus*, dipnet, leg. Sprick,P., det. Sprick,P.

**Name**<br>**Authority**<br>**Additional Information**	**GenBank Acc No (Ref.)**<br>**Specimen ID**<br>**Sequence Length**	**ZFMK Tissue ID**<br>**ZFMK DNA Sample ID**<br>**(SDEI DNA Sample ID)**	**Locality, GPS, Collection Date, Plant, Collector, Identifier**
Cyanapion columbinum<br>(Germar, 1817)	MK892320 [new]<br>2621-PSP<br>658nt	ZFMK-TIS-23595<br>ZFMK-DNA-0171661938	Germany, Bavaria (BY), Unterfranken, Ebern, N50°05'07" E10°45'52.5", 318m, 14-Jun-2014, *Lathyrus tuberosus*, leg. Sprick,P., det. Sprick,P.
Cyanapion gyllenhalii<br>(Kirby, 1808)	MK891446 [new]<br>1511-PSP<br>658nt	ZFMK-TIS-3271<br>ZFMK-DNA-0155633318	Denmark, Syddanmark, Emmerlev Klev (Højer), N54°59'53" E08°39'08", 3m, 30-Aug-2012, *Lathyrus pratensis*, dipnet, leg. Sprick,P., det. Sprick,P.
Cyanapion gyllenhalii<br>(Kirby, 1808)	KC784139 [new]<br>639-PSP<br>658nt	ZFMK-TIS-20422<br>ZFMK-DNA-0100448628	Germany, Saxony-Anhalt (ST), Drömling, Mannhausen, N52°25'30" E11°12'51", 57m, 08-Sep-2011, *Vicia cracca*, wetlands, dipnet, leg. Sprick,P., det. Sprick,P.
Cyanapion gyllenhalii<br>(Kirby, 1808)	MK890976 [new]<br>867-PST<br>658nt	ZFMK-TIS-3780<br>ZFMK-DNA-0100414217	Germany, Rhineland-Palatinate (RLP), Adenau, N50°24'43" E06°55'48", 259m, 24-Jul-2012, *Lathyrus*, beating, leg. Stüben,P., det. Stüben,P.
Cyanapion platalea<br>(Germar, 1817)	MK892102 [new]<br>2379-JKR<br>658nt	ZFMK-TIS-25956<br>ZFMK-DNA-0171600610	Czech Republic, Moravia, Znojmo, N48°51'13" E16°06'34", 250m, 23-Aug-2014, sweeping, leg. R. Stejskal, det. Stejskal,R.
Cyanapion spencii<br>(Kirby, 1808)	MK890975 [new]<br>866-PST<br>658nt	ZFMK-TIS-3779<br>ZFMK-DNA-0100414216	Germany, Rhineland-Palatinate (RLP), Adenau, N50°24'43" E06°55'48", 259m, 24-Jul-2012, *Lathyrus*, beating, leg. Stüben,P., det. Stüben,P.
Cyanapion spencii<br>(Kirby, 1808)	MK891405 [new]<br>1467-PSP<br>658nt	ZFMK-TIS-3227<br>ZFMK-DNA-0155627304	Germany, Lower Saxony (NI), Harz, St. Andreasberg, Jordanshöhe, N51°42'58" E10°32'08", 674m, 02-Jul-2012, *Vicia cracca*, mountain meadow, dipnet, leg. Sprick,P., det. Sprick,P.
Cycloderes canescens<br>(Rossi, 1792)	KC784273 [new]<br>IT-0047w<br>658nt	ZFMK-DNA-0100417921	Italy, Sardinia Isl. East, E of Seui, Mt. Arqueri, N39°49'36" E09°22'27", 960m, 29-Sep-2010, leg. Stüben,P., det. Stüben,P.
Cycloderes pilosulus<br>(Herbst, 1795)	KC784090 [new]<br>539-RST<br>658nt	ZFMK-TIS-20324<br>ZFMK-DNA-0100448449	Czech Republic, Moravia mer., 11 km SE of Znojmo, Jecmenist? near Dyjakovicky, N48°45'10.63" E16°8'11.16", 280m, 03-Sep-2011, hand-collecting, leg. Stejskal,R., det. Stejskal,R.
Cycloderes pilosulus<br>(Herbst, 1795)	MK891571 [new]<br>1168-JKR<br>658nt	ZFMK-TIS-3504<br>ZFMK-DNA-0155630464	Hungary, Pest megye, Pecel, N47°29'50" E19°22'45", 192m, 26-Mai-2012, sweeping, leg. Krátky,J., det. Krátky,J.
Cycloderes pilosulus<br>(Herbst, 1795)	MK892224 [new]<br>2522-PSP<br>658nt	ZFMK-TIS-23494<br>ZFMK-DNA-0171661751	Slovakia, Nitra, SW of Buc, N47°46'56" E18°25'10", 110m, 18-Mai-2014, *herb layer*, leg. Sprick,P., det. Sprick,P.
Cyphocleonus achates<br>(Fahraeus, 1842)	MK892078 [40]<br>2354-JKR<br>658nt	ZFMK-TIS-25914<br>ZFMK-DNA-0171600587	Slovakia, Nove Zamky, Nesvady, N47°55'13" E18°09'39", 115m, 19-Jul-2014, *Centaurea stoebe*, collecting by hand, leg. R. Stejskal, det. Stejskal,R.
Cyphocleonus achates<br>(Fahraeus, 1842)	MK892149 [new]<br>2436-JKR<br>658nt	ZFMK-TIS-26013<br>ZFMK-DNA-0171600649	Slovakia, Nove Zamky, Imel, PR Liscie diery, N47°55'17" E18°09'37", 114m, 20-Sep-2014, *Centaurea stoebe*, sweeping, leg. Krátky,J., det. Krátky,J.
Cyphocleonus armitagei<br>(Wollaston, 1864)	KC784294 [40]<br>721-PST<br>615nt	ZFMK-TIS-3058<br>ZFMK-DNA-0100448166	Spain, Canary Islands, Tenerife, Aguamansa, N28°21'55" W16°30'38", 963m, 18-Jan-2012, *Argyranthemum broussonetii*, beating, leg. Stüben,P., det. Stüben,P.
Cyphocleonus dealbatus<br>(Gmelin, 1790)	MK891903 [new]<br>2170-JKR<br>658nt	ZFMK-TIS-23906<br>ZFMK-DNA-0169170617	Czech Republic, Moravia, Bozice, N48°49'21" E16°15'55", 220m, 02-Mai-2014, *Artemisia vulgaris*, collecting by hand, leg. Stejskal,R., det. Stejskal,R.

**Name**<br>**Authority**<br>**Additional Information**	**GenBank Acc No (Ref.)**<br>**Specimen ID**<br>**Sequence Length**	**ZFMK Tissue ID**<br>**ZFMK DNA Sample ID**<br>**(SDEI DNA Sample ID)**	**Locality, GPS, Collection Date, Plant, Collector, Identifier**
Cyphocleonus dealbatus<br>(Gmelin, 1790)	MK892073 [new]<br>2349-JKR<br>658nt	ZFMK-TIS-24280<br>ZFMK-DNA-0169170919	Czech Republic, Moravia, Lanzhot, Pohansko, N48°41'27" E16°55'58", 153m, 14-Aug-2014, *Achillea millefolium*, sweeping, leg. Krátky,J., det. Krátky,J.
Cyphocleonus dealbatus<br>(Gmelin, 1790)	MK891698 [new]<br>1916-FBA<br>658nt	ZFMK-TIS-23307<br>ZFMK-DNA-0169170364	Greece, Central Macedonia, Serres, Achladohori, N41°19'00" E23°34'20", 670m, 12-Aug-2014, leg. Bayer & Brunner, det. Bayer,F.
Cyphocleonus dealbatus<br>(Gmelin, 1790)	MK891912 [new]<br>2180-JKR<br>658nt	ZFMK-TIS-23918<br>ZFMK-DNA-0169170604	Slovakia, Nove Zamky, Imel, Liscie diery, N47°55'17" E18°09'37", 114m, 23-Mai-2014, *Asteraceae*, collecting by hand, leg. Stejskal,R., det. Stejskal,R.
Cyphocleonus dealbatus<br>(Gmelin, 1790)	MK892150 [new]<br>2437-JKR<br>658nt	ZFMK-TIS-26014<br>ZFMK-DNA-0171597934	Slovakia, Nove Zamky, Imel, PR Liscie diery, N47°55'17" E18°09'37", 114m, 20-Sep-2014, *Achillea millefolium*, sweeping, leg. Krátky,J., det. Krátky,J.
Cyphocleonus garajonay<br>Stüben, 2020<br>**Paratype (DNAtype)**	KC783777 [37]<br>68-PST_7G2010<br>605nt	ZFMK-TIS-2D100446981<br>ZFMK-DNA-0100438053	Spain, Canary Islands, La Gomera, Parque Garajonay, El Cedro (“Casa Olsen”), N28°06'24" W17°13'58", 1348m, 15-Dez-2010, *Argyranthemum broussonetii*, beating, leg. Stüben,P., det. Stüben,P.
Cyphocleonus garajonay<br>Stüben, 2020<br>**Paratype (DNAtype)**	MK892370 [37]<br>2819-PST_25865<br>658nt	ZFMK-TIS-25865<br>ZFMK-DNA-FC17941500	Spain, Canary Islands, La Gomera, Meriga, N28°09'17" W17°14'06", 829m, 16-Dez-2012, *Argyranthemum*, beating, leg. Stüben,P., det. Stüben,P.
Cyphocleonus sventeniusi<br>(Roudier, 1957)	KC783760 [40]<br>49-PST<br>658nt	ZFMK-TIS-2D100446998<br>ZFMK-DNA-0100438070	Spain, Canary Islands, Gran Canaria, 1.2 km S of San Bartolome, N27°54'43" W15°34'21", 938m, 22-Jan-2011, *Argyranthemum*, beating, leg. Stüben,P., det. Stüben,P.
Cyphocleonus trisulcatus<br>(Herbst, 1795)	MK891910 [new]<br>2177-JKR<br>658nt	ZFMK-TIS-23915<br>ZFMK-DNA-0169170601	Czech Republic, Olomouc, Mrsklesy, N49°35'47" E17°24'56", 290m, 07-Mai-2014, *Leucanthemum vulgare*, collecting by hand, leg. Trnka,F., det. Trnka,F.
Datonychidius tener<br>(Reitter, 1888)	MK891290 [new]<br>1247-PST<br>658nt	ZFMK-TIS-3583<br>ZFMK-DNA-0100426037	Greece, Zakynthos Isl., near Elies, N37°54'18" E20°41'20", 303m, 13-Okt-2012, *Marrubium* sp., by hand, leg. Stüben,P., det. Krátky,J.
Datonychus angulosus<br>(Boheman, 1845)	MK892270 [new]<br>2570-PSP<br>658nt	ZFMK-TIS-23544<br>ZFMK-DNA-0171661989	Germany, Saxony-Anhalt (ST), Harz National Park, Feuersteinwiesen, N51°45'30" E10°41'29", 614m, 11-Jun-2014, *Galeopsis speciosa*, leg. Sprick,P., det. Sprick,P.
Datonychus arquata<br>(Herbst,1795)	MK892118 [new]<br>2395-JKR<br>658nt	ZFMK-TIS-25972<br>ZFMK-DNA-0171600617	Slovakia, Zilina, Suja, N49°03'46" E18°37'19", 474m, 21-Jun-2014, *Lycopus europaeus*, sweeping, leg. Krátky,J., det. Krátky,J.
Datonychus delicatulus<br>(Hustache, 1946)	KC783839 [new]<br>189-PST<br>658nt	ZFMK-TIS-2D100440235<br>ZFMK-DNA-0100438123	Morocco, Atlas Mts., S of Ifrane, Michliffen, N33°24'48" W05°04'35", 2002m, 22-Mai-2011, *Stachys*, beating, leg. Stüben,P., det. Behne,L.
Datonychus maurus<br>(Schultze, 1899)	KC783842 [new]<br>184-PST<br>620nt	ZFMK-TIS-2D100440976<br>ZFMK-DNA-0100438131	Morocco, Atlas Mts., Imouzzer-des-Marmoucha, N33°27'35" W04°17'47", 1637m, 21-Mai-2011, beating, leg. Stüben,P., det. Stüben,P./vid. Behne,L.
Datonychus maurus<br>(Schultze, 1899)	MK891284 [new]<br>1235-PST<br>658nt	ZFMK-TIS-3571<br>ZFMK-DNA-0100426056	Portugal, Minho, near Santa Comba, Rio Lima, N41°45'31 W08°37'29", 7m, 13-Mai-2012, beating, leg. Stüben,P., det. Krátky,J.
Datonychus maurus<br>(Schultze, 1899)	KC784308 [13]<br>746-PST<br>658nt	ZFMK-TIS-3083<br>ZFMK-DNA-0100448141	Spain, Canary Islands, Tenerife, Anaga Mts. near Batan de Arriba, N28°32'23" W16°17'34", 596m, 06-Feb-2012, beating, leg. Stüben,P. & Schütte,A., det. Stüben,P.

**Name**<br>**Authority**<br>**Additional Information**	**GenBank Acc No (Ref.)**<br>**Specimen ID**<br>**Sequence Length**	**ZFMK Tissue ID**<br>**ZFMK DNA Sample ID**<br>**(SDEI DNA Sample ID)**	**Locality, GPS, Collection Date, Plant, Collector, Identifier**
Datonychus melanostictus<br>(Marsham, 1802)	MK891475 [new]<br>1543-PSP<br>658nt	ZFMK-TIS-3303<br>ZFMK-DNA-0155627325	Germany, Lower Saxony (NI), Rethen, Hannover region, N52°16'38" E09°49'31", 58m, 25-Jun-2013, *Lycopus europaeus*, dipnet, leg. Sprick,P., det. Sprick,P.
Datonychus melanostictus<br>(Marsham, 1802)	MK892185 [new]<br>2480-PSP<br>658nt	ZFMK-TIS-23452<br>ZFMK-DNA-0171661793	Germany, Lower Saxony (NI), Wendischbrome, Lkr. Salzwedel, pond ditch, N52°37'32" E10°55'00", 72m, 31-Jul-2013, *Mentha aquatica*, dipnet, leg. Sprick,P., det. Sprick,P.
Datonychus melanostictus<br>(Marsham, 1802)	KM433742 [13]<br>958-CBR<br>658nt	ZFMK-TIS-3871<br>ZFMK-DNA-0100449653	Luxembourg, 10 km E of Luxembourg, 1 km SW of Mensdorf, N49°39'00" E06°17'40", 240m, 21-Jun-2012, *Lycopus europaeus*, beating, leg. Braunert,C., det. Braunert,C.
Datonychus melanostictus<br>(Marsham, 1802)	MK892119 [new]<br>2396-JKR<br>658nt	ZFMK-TIS-25973<br>ZFMK-DNA-0171600618	Slovakia, Zilina, Suja, N49°03'46" E18°37'19", 474m, 21-Jun-2014, *Mentha* sp., sweeping, leg. Krátky,J., det. Krátky,J.
Datonychus paszlavszkyi<br>(Kuthy, 1890)	MK891208 [new]<br>1126-JKR<br>658nt	ZFMK-TIS-3462<br>ZFMK-DNA-0100449582	Slovakia, Nove Zamky, Kamenin, N47°51'51" E18°38'09", 110m, 11-Mai-2012, *Salvia nemorosa*, beating, leg. Krátky,J., det. Krátky,J.
Dendroacalles brevitarsis<br>(Wollaston, 1864)	JN701885 [16]<br>26-PST<br>658nt	ZFMK-TIS-2D100447021<br>ZFMK-DNA-0100438093	Spain, Canary Islands, Gran Canaria, S of Guia, Lomo Betancor (Barranco), N28°06'58" W15°37'37", 540m, 21-Dez-2010, *Euphorbia obtusifolia*, beating, leg. Stüben,P., det. Stüben,P.
Dendroacalles brevitarsis<br>(Wollaston, 1864)	KC783810 [40]<br>109-PST<br>607nt	ZFMK-TIS-2D100440318<br>ZFMK-DNA-0100438350	Spain, Canary Islands, Gran Canaria, SE of San Mateo, N28°01'20" W15°29'48", 732m, 31-Dez-2010, *Euphorbia obtusifolia*, beating, leg. Stüben,P., det. Stüben,P.
Dendroacalles brevitarsis<br>(Wollaston, 1864)	KJ867568 [40]<br>35-PST<br>658nt	ZFMK-TIS-2D100447012<br>ZFMK-DNA-0100437929	Spain, Canary Islands, Gran Canaria, NE of Santa Brigida, Angostura valley, N28°03'14" W15°28'49", 485m, 31-Dez-2010, *Euphorbia obtusifolia*, beating, leg. Stüben,P., det. Stüben,P.
Dendroacalles cf. *ruteri*	MG229719 [40]<br>1669-PST<br>658nt	ZFMK-TIS-24387<br>ZFMK-DNA-0155622521	Spain, Canary Islands, La Gomera, Parque Natural de Majona, El Helechal, N28°08'42" W17°09'55", 691m, 05-Dez-2013, sieving, leg. Stüben,P., det. Stüben,P.
Dendroacalles euphorbiacus<br>(Stüben, 2000)	EU286475 [01]<br>C-0138-eup<br>658nt	ZFMK-TIS-cC0138<br>ZFMK-DNA-0100400177	Spain, Canary Islands, La Palma, 4.5 km NE of Garafia, 1 km N of El Mudo, N28°50'36" W17°54'07", 254m, 09-Jul-2006, *Euphorbia regis-jubae*, leg. Stüben,P., det. Stüben,P.
Dendroacalles euphorbiacus<br>(Stüben, 2000)	GU987861 [01]<br>E-0267-eup<br>658nt	ZFMK-TIS-cE0267<br>ZFMK-DNA-0100401044	Spain, Canary Islands, La Palma, 1.5 km N of Las Caletas, N28°29'54" W17°50'00", 663m, 28-Jun-2006, *Euphorbia regis-jubae*, leg. Stüben,P., det. Stüben,P.
Dendroacalles euphorbiacus<br>(Stüben, 2000)	MG229733 [40]<br>1719-PST<br>658nt	ZFMK-TIS-24437<br>ZFMK-DNA-0155622471	Spain, Canary Islands, La Palma, Montes de Luna, N28°31'57" W17°48'37", 583m, 28-Dez-2013, *Euphorbia regis-jubae* , beating, leg. Stüben,P., det. Stüben,P.
Dendroacalles euphorbiacus<br>(Stüben, 2000)	MG229739 [40]<br>1742-PST<br>658nt	ZFMK-TIS-24460<br>ZFMK-DNA-0155622448	Spain, Canary Islands, La Palma, Las Caletas, N28°29'35" W17°49'41", 427m, 12-Jan-2014, *Euphorbia regis-jubae*, beating, leg. Stüben,P., det. Stüben,P.
Dendroacalles fortunatus garajonay<br>Stüben, 2011	GU988015 [01]<br>E-714-rut<br>658nt	ZFMK-TIS-cE714<br>ZFMK-DNA-0100404309	Spain, Canary Islands, La Gomera, S of Hermigua, El Cedro, Las Mimbreras, N28°07'27" W17°13'26", 901m, 06-Okt-2008, *Laurisilva*, leg. Astrin,J. & Stüben,P., det. Stüben,P.
Dendroacalles fortunatus garajonay<br>Stüben, 2011	GU988018 [01]<br>E-720-rut<br>658nt	ZFMK-TIS-cE720<br>ZFMK-DNA-0100404838	Spain, Canary Islands, La Gomera, S of Vallehermoso, La Meseta, "La Piedra Encantada", N28°09'15" W17°17'36", 819m, 07-Okt-2008, *Ocotea foetens*, leg. Astrin,J. & Stüben,P., det. Stüben,P.

**Name**<br>**Authority**<br>**Additional Information**	**GenBank Acc No (Ref.)**<br>**Specimen ID**<br>**Sequence Length**	**ZFMK Tissue ID**<br>**ZFMK DNA Sample ID**<br>**(SDEI DNA Sample ID)**	**Locality, GPS, Collection Date, Plant, Collector, Identifier**
Dendroacalles fortunatus<br>(Wollaston, 1864)	GU988016 [01]<br>E-715-for<br>658nt	ZFMK-TIS-cE715<br>ZFMK-DNA-0100404833	Spain, Canary Islands, La Gomera, S of Hermigua, El Cedro, Las Mimbreras, N28°07'27" W17°13'26", 901m, 06-Okt-2008, *Laurisilva*, leg. Astrin,J. & Stüben,P., det. Stüben,P.
Dendroacalles fortunatus<br>(Wollaston, 1864)	GU988020 [01]<br>E-722-for<br>658nt	ZFMK-TIS-cE722<br>ZFMK-DNA-0100404840	Spain, Canary Islands, La Gomera, S of Vallehermoso, La Meseta, "La Piedra Encantada", N28°09'15" W17°17'36", 819m, 07-Okt-2008, *Ocotea foetens*, leg. Astrin,J. & Stüben,P., det. Stüben,P.
Dendroacalles fortunatus<br>(Wollaston, 1864)	GU988081 [01]<br>E-807-for<br>658nt	ZFMK-TIS-cE807<br>ZFMK-DNA-0100405009	Spain, Canary Islands, La Gomera, SW of Hermigua, Los Acevinos, N28°08'24" W17°13'45", 992m, 11-Okt-2008, *Persea indica*, leg. Astrin,J.,Stüben,P.,Behne,L.,Floren,L., det. Stüben,P.
Dendroacalles fortunatus<br>(Wollaston, 1864)	GU988082 [01]<br>E-808-for<br>658nt	ZFMK-TIS-cE808<br>ZFMK-DNA-0100405010	Spain, Canary Islands, La Gomera, SW of Hermigua, Los Acevinos, N28°08'24" W17°13'45", 992m, 11-Okt-2008, *Persea indica*, leg. Astrin,J.,Stüben,P.,Behne,L.,Floren,L., det. Stüben,P.
Dendroacalles fortunatus<br>(Wollaston, 1864)	GU988083 [01]<br>E-809-for<br>617nt	ZFMK-TIS-cE809<br>ZFMK-DNA-0100404542	Spain, Canary Islands, La Gomera, SW of Hermigua, Los Acevinos, N28°08'24" W17°13'45", 992m, 11-Okt-2008, *Persea indica*, leg. Astrin,J.,Stüben,P.,Behne,L.,Floren,L., det. Stüben,P.
Dendroacalles fortunatus<br>(Wollaston, 1864)	KF843949 [16]<br>ES1005<br>658nt	ZFMK-TIS-cES1005<br>ZFMK-DNA-0112704664	Spain, Canary Islands, La Gomera, S of Hermigua, El Cedro, Meriga, 28°09'15"N 17°14'10"W, 824m, 04-Dez-2009, *Laurisilva*, gardens, edge of Laurisilva, leg. Stüben,P., det. Stüben,P.
Dendroacalles fortunatus<br>Stüben, 2011	MG229793 [40]<br>2707-PST<br>658nt	ZFMK-TIS-23857<br>ZFMK-DNA-0171661292	Spain, Canary Islands, La Gomera, Meriga, N28°09'14" W17°14'11", 794m, 12-Dez-2014, *Rubus*, beating, leg. Stüben,P., det. Stüben,P.
Dendroacalles ornatus<br>(Wollaston, 1854)	FJ716548 [01]<br>P-530-orn<br>658nt	ZFMK-TIS-cP530<br>ZFMK-DNA-0100400639	Portugal, Madeira, 3 km W of Seixal, "Lagoa" near "Fanal", N32°48'47" W17°08'59", 950m, 22-Mrz-2008, *Ocotea foetens*, leg. Astrin,J. & Stüben,P., det. Astrin,J. and Stüben,P.
Dendroacalles ornatus<br>(Wollaston, 1854)	MG229751 [40]<br>1846-PST<br>658nt	ZFMK-TIS-26049<br>ZFMK-DNA-0171606131	Portugal, Madeira, SE of Ribeira da Janela, Fanal, N32°48'48" W17°08'57", 1084m, 29-Jun-2014, *Ocotea foetens*, beating, leg. Stüben,P., det. Stüben,P.
Dendroacalles poneli<br>(Stüben, 2000)	EU286476 [01]<br>C-0141-pon<br>658nt	ZFMK-TIS-cC0141<br>ZFMK-DNA-0100400178	Spain, Canary Islands, El Hierro, 3 km SW of Sabinosa, La Dehesa, Piedra del Regidor, N27°43'53" W18°07'04", 725m, 26-Dez-2006, *Euphorbia regis-jubae*, leg. Stüben,P., det. Stüben,P.
Dendroacalles poneli<br>(Stüben, 2000)	GU987866 [01]<br>E-0273-pon<br>658nt	ZFMK-TIS-cE0273<br>ZFMK-DNA-0100400904	Spain, Canary Islands, El Hierro, 5 km NE of Faro de Orchilla, Roque Grande, N27°43'19" W18°06'01", 808m, 02-Jan-2007, *Euphorbia regis-jubae*, leg. Stüben,P., det. Stüben,P.
Dendroacalles poneli<br>(Stüben, 2000)	GU987805 [01]<br>C-0140-pon<br>658nt	ZFMK-TIS-cC0140<br>ZFMK-DNA-0100400011	Spain, Canary Islands, La Gomera, 3.5 km NW of Hermigua, N28°11'18" W17°12'27", 410m, 26-Dez-2004, *Euphorbia obtusifolia*, succulent bush, leg. Stüben,P., det. Stüben,P.
Dendroacalles poneli<br>(Stüben, 2000)	GU988024 [01]<br>E-730-pon<br>658nt	ZFMK-TIS-cE730<br>ZFMK-DNA-0100404825	Spain, Canary Islands, La Gomera, SE of Hermigua near Casas del Palmar, N28°09'29" W17°09'37", 627m, 08-Okt-2008, *Euphorbis regis-jubae*, thermophilic brushwood, leg. Astrin,J. & Stüben,P., det. Stüben,P.
Dendroacalles poneli<br>(Stüben, 2000)	GU988025 [01]<br>E-735-pon<br>658nt	ZFMK-TIS-cE735<br>ZFMK-DNA-0100404820	Spain, Canary Islands, La Gomera, Hermigua, Ermita de San Juan, N28°09'39" W17°12'19", 513m, 09-Okt-2008, *Euphorbia regis-jubae*, leg. Astrin,J. & Stüben,P., det. Stüben,P.
Dendroacalles poneli<br>(Stüben, 2000)	GU988027 [01]<br>E-740-pon<br>658nt	ZFMK-TIS-cE740<br>ZFMK-DNA-0100404810	Spain, Canary Islands, La Gomera, E of Hermigua, N28°09'09" W17°09'50", 708m, 10-Okt-2008, *Euphorbia regis-jubae*, leg. Astrin,J. & Stüben,P., det. Stüben,P.

**Name**<br>**Authority**<br>**Additional Information**	**GenBank Acc No (Ref.)**<br>**Specimen ID**<br>**Sequence Length**	**ZFMK Tissue ID**<br>**ZFMK DNA Sample ID**<br>**(SDEI DNA Sample ID)**	**Locality, GPS, Collection Date, Plant, Collector, Identifier**
Dendroacalles poneli<br>(Stüben, 2000)	KF843951 [16]<br>ES1017<br>658nt	ZFMK-TIS-cES1017<br>ZFMK-DNA-0112704655	Spain, Canary Islands, La Gomera, E of Hermigua, El Palmar, 28°09'26"N 17°09'47"W, 628m, 30-Dez-2009, *Ficus carica*, thermophilic brushwood, leg. Stüben,P., det. Stüben,P.
Dendroacalles poneli<br>(Stüben, 2000)	KF843953 [16]<br>ES1020<br>658nt	ZFMK-TIS-cES1020<br>ZFMK-DNA-0112704658	Spain, Canary Islands, La Gomera, W of Hermigua near Agulo, 28°11'16"N 17°12'36"W, 421m, 04-Jan-2010, *Euphorbia bravoana*, leg. Stüben,P., det. Stüben,P.
Dendroacalles poneli<br>(Stüben, 2000)	KF843954 [16]<br>ES1021<br>658nt	ZFMK-TIS-cES1021<br>ZFMK-DNA-0112704659	Spain, Canary Islands, La Gomera, W of Hermigua near Agulo, 28°11'16"N 17°12'36"W, 421m, 04-Jan-2010, *Euphorbia obtusifolia*, leg. Stüben,P., det. Stüben,P.
Dendroacalles poneli<br>(Stüben, 2000)	KF843955 [16]<br>ES1024<br>658nt	ZFMK-TIS-cES1024<br>ZFMK-DNA-0112704645	Spain, Canary Islands, La Gomera, N of Epina, Teselinde, 28°11'36"N 17°17'31"W, 705m, 06-Jan-2010, *Laurisilva (Erica): Euphorbia*, leg. Stüben,P., det. Stüben,P.
Dendroacalles poneli<br>(Stüben, 2000)	MG229720 [40]<br>1681-PST<br>658nt	ZFMK-TIS-24399<br>ZFMK-DNA-0155622510	Spain, Canary Islands, La Gomera, Arguamul, N28°11'56" W17°17'40", 445m, 15-Dez-2013, *Euphorbia regis-jubae* , beating, leg. Stüben,P., det. Stüben,P.
Dendroacalles poneli<br>(Stüben, 2000)	GU987799 [01]<br>C-0139-pon<br>658nt	ZFMK-TIS-cC0139<br>ZFMK-DNA-0100400016	Spain, Canary Islands, Tenerife, Teno Mts., 8 km NW of Santiago del Teide, Los Carrizales, N28°19'14" W16°52'03", 370m, 02-Jan-2004, *Euphorbia atropurpurea*, leg. Stüben,P., det. Stüben,P.
Dendroacalles poneli<br>(Stüben, 2000)	GU988004 [01]<br>E-694-pon<br>658nt	ZFMK-TIS-cE694<br>ZFMK-DNA-0100404860	Spain, Canary Islands, Tenerife, W of Los Silos, Teno Mts., Casa Blanca near Buenavista del Norte, N28°21'36" W16°52'10", 231m, 02-Okt-2008, *Euphorbia atropurpurea*, leg. Astrin,J. & Stüben,P., det. Stüben,P.
Dendroacalles poneli<br>(Stüben, 2000)	GU988010 [01]<br>E-705-pon<br>658nt	ZFMK-TIS-cE705<br>ZFMK-DNA-0100404850	Spain, Canary Islands, Tenerife, SW of Los Silos, Teno Mts. near Masca, Los Carizales, N28°19'11" W16°52'08", 434m, 03-Okt-2008, *Euphorbia atropurpurea*, leg. Astrin,J. & Stüben,P., det. Stüben,P.
Dendroacalles ruteri<br>(Roudier, 1954)	JN701888 [16]<br>42-PST<br>607nt	ZFMK-DNA-0100438076	Spain, Canary Islands, Gran Canaria, Moya, Barranco de los Tilos, N28°05'15" W15°35'32", 619m, 04-Jan-2011, *Laurisilva*, beating, leg. Stüben,P., det. Stüben,P.
Dendroacalles ruteri<br>(Roudier, 1954)	MG229801 [40]<br>2766-JKR<br>658nt	ZFMK-TIS-26215<br>ZFMK-DNA-0171661894	Spain, Canary Islands, Gran Canaria, San Fernando, Barranco de los Tilos, N28°05'18" W15°35'36", 529m, 22-Feb-2015, *Laurus*, beating, leg. Krátky,J., det. Krátky,J.
Dendroacalles ruteri<br>(Roudier, 1954)	EU286471 [01]<br>C-0132-rut<br>658nt	ZFMK-TIS-cC0132<br>ZFMK-TIS-cC0132	Spain, Canary Islands, La Palma, 3 km SE of Garafia, Montana de las Varas, N28°49'11" W17°54'48", 919m, 09-Jul-2006, *Laurisilva*, leg. Stüben,P., det. Stüben,P.
Dendroacalles ruteri<br>(Roudier, 1954)	MG229724 [40]<br>1698-PST<br>658nt	ZFMK-TIS-24416<br>ZFMK-DNA-0155622492	Spain, Canary Islands, La Palma, N of Santa Cruz, M. de Tagoja, N28°43'18" W17°47'07", 1047m, 21-Dez-2013, *Laurisilva*, beating, leg. Stüben,P., det. Stüben,P.
Dendroacalles ruteri<br>(Roudier, 1954)	MG229728 [40]<br>1711-PST<br>658nt	ZFMK-TIS-24429<br>ZFMK-DNA-0155622479	Spain, Canary Islands, La Palma, NW of Puntallana, Bco. de la Galga, N28°46'13" W17°46'37", 421m, 27-Dez-2013, *Laurisilva*, beating, leg. Stüben,P., det. Stüben,P.
Dendroacalles ruteri<br>(Roudier, 1954)	GU988087 [01]<br>E-815-rut<br>658nt	ZFMK-TIS-cE815<br>ZFMK-DNA-0100405570	Spain, Canary Islands, Tenerife, SW of Los Silos, Teno Mts., Monte del Agua, Chupadero, N28°19'23" W16°49'12", 940m, 02-Okt-2008, *Myrica faya* , leg. Astrin,J.,Stüben,P.,Behne,L.,Floren,L., det. Stüben,P.
Dendroacalles ruteri<br>(Roudier, 1954)	GU988089 [01]<br>E-819-rut<br>658nt	ZFMK-TIS-cE819<br>ZFMK-DNA-0100405568	Spain, Canary Islands, Tenerife, NE of La Laguna, Anaga Mts., forest Las Mercedes, N28°31'49" W16°17'16", 889m, 16-Okt-2008, *Erica arborea* , leg. Floren & Machado, det. Floren and Machado

**Name**<br>**Authority**<br>**Additional Information**	**GenBank Acc No (Ref.)**<br>**Specimen ID**<br>**Sequence Length**	**ZFMK Tissue ID**<br>**ZFMK DNA Sample ID**<br>**(SDEI DNA Sample ID)**	**Locality, GPS, Collection Date, Plant, Collector, Identifier**
Dendroacalles sigma<br>(Wollaston, 1864)	FJ716560 [01]<br>E-554-sig<br>658nt	ZFMK-TIS-cE554<br>ZFMK-DNA-0100404989	Spain, Canary Islands, La Palma, 4.5 km E of Garafia, 2 km S of Don Pedro, N28°49'38" W17°53'46", 708m, 13-Jul-2006, *Laurisilva, Juglans* , leg. Stüben,P., det. Stüben,P.
Dendroacalles sigma<br>(Wollaston, 1864)	MG229705 [40]<br>1555-JKR<br>658nt	ZFMK-TIS-3603<br>ZFMK-DNA-0155635674	Spain, Canary Islands, La Palma, Cubo de Galga, N28°45'42" W17°46'34", 467m, 28-Jan-2013, *Laurus*, beating, leg. Krátky,J., det. Krátky,J.
Dendroacalles sigma<br>(Wollaston, 1864)	MG229731 [40]<br>1714-PST<br>658nt	ZFMK-TIS-24432<br>ZFMK-DNA-0155622476	Spain, Canary Islands, La Palma, W of Barlovento (near La Tosca), N28°49'12" W17°49'16", 475m, 27-Dez-2013, beating, leg. Stüben,P., det. Stüben,P.
Dendroacalles sigma<br>(Wollaston, 1864)	MG229735 [40]<br>1730-PST<br>658nt	ZFMK-TIS-24448<br>ZFMK-DNA-0155622463	Spain, Canary Islands, La Palma, E of Barlovento, above La Fajana (Bco. de los Hombres), N28°49'36" W17°52'07", 387m, 31-Dez-2013, *Laurisilva*, beating, leg. Stüben,P., det. Stüben,P.
Dendroacalles sigma<br>(Wollaston, 1864)	MG229775 [40]<br>1989-JKR<br>658nt	ZFMK-TIS-23629<br>ZFMK-DNA-0169170507	Spain, Canary Islands, La Palma, Cubo de Galga, N28°45'42" W17°46'34", 467m, 07-Feb-2014, *Laurisilva*, beating, leg. Krátky,J., det. Krátky,J.
Deporaus betulae<br>(Linnaeus, 1758)	KC783996 [new]<br>266-PSP<br>658nt	ZFMK-TIS-2D100439112<br>ZFMK-DNA-0100439784	Germany, Lower Saxony (NI), Helstorfer Moor, Resse, Hannover region, N52°31'44" E09°36'32", 43m, 14-Mai-2011, *Betula pubescens*, bog forest, beating, leg. Sprick,P., det. Sprick,P.
Deporaus betulae<br>(Linnaeus, 1758)	MK891472 [new]<br>1540-PSP<br>658nt	ZFMK-TIS-3300<br>ZFMK-DNA-0155633282	Germany, Saxony-Anhalt (ST), Harz National Park, Ilsenburg, Meineberg, N51°51'26" E10°39'52", 413m, 28-Jun-2013, *Betula pendula*, birch trees succession, beating, leg. Sprick,P., det. Sprick,P.
Deporaus betulae<br>(Linnaeus, 1758)	MK891641 [new]<br>1794-PST<br>658nt	ZFMK-TIS-24128<br>ZFMK-DNA-0171624070	Germany, Rhineland-Palatinate (RLP), Ahr valley, Dernau, below Krausberg near Steinbergsmühle, N50°31'40" E07°02'43", 204m, 29-Mai-2014, beating, leg. Stüben,P., det. Stüben,P.
Derelomus chamaeropis<br>(Fabricius, 1798)	KC783895 [new]<br>131-PST<br>658nt	ZFMK-TIS-2D100440296<br>ZFMK-DNA-0100438368	Morocco, S of Berkane, N of Aïn-es-Sfa, Beni Snassen Mts., N34°49'54" W02°08'49", 985m, 13-Mai-2011, *Chamaerops humilis*, beating, leg. Stüben,P., det. Stüben,P./vid. Behne,L.
Derelomus chamaeropis<br>(Fabricius, 1798)	MK891322 [new]<br>1323-PST<br>658nt	ZFMK-TIS-4139<br>ZFMK-DNA-0100426144	Spain, Malaga, NW of Otivar, Sierra del Chapparal, N36°49'32" W03°42'30", 653m, 08-Mai-2013, *Quercus ilex*, sieving, leg. Stüben,P. & Schütte,A., det. Stüben,P.
Derelomus piriformis<br>(A. Hoffmann, 1938)	KC783746 [40]<br>32-PST<br>647nt	ZFMK-TIS-2D100447015<br>ZFMK-DNA-0100438087	Spain, Canary Islands, Gran Canaria, Temisas, N27°54'41" W15°30'11", 705m, 27-Dez-2010, *Phoenix canariensis*, beating, leg. Stüben,P., det. Stüben,P.
Derelomus piriformis<br>(A. Hoffmann, 1938)	KC783765 [40]<br>55-PST<br>658nt	ZFMK-TIS-2D100446993<br>ZFMK-DNA-0100438065	Spain, Canary Islands, La Gomera, Hermigua, Las Nuevitas, Barranquillos los Alamos, N28°09'31" W17°11'09", 251m, 13-Dez-2010, *Phoenix canariensis*, beating, leg. Stüben,P., det. Stüben,P.
Derelomus piriformis<br>(A. Hoffmann, 1938)	KC784323 [40]<br>688-PST<br>658nt	ZFMK-TIS-3025<br>ZFMK-DNA-0100417613	Spain, Canary Islands, Tenerife, Teno Mts., Cuevas del Palmas, N28°19'50" W16°50'33", 726m, 14-Feb-2012, *Phoenix*, leg. Stüben,P. & Schütte,A., det. Stüben,P.
Dichotrachelus sardous<br>A. & F. Solari, 1904	KC784246 [new]<br>IT-0013w<br>625nt	ZFMK-TIS-2D100446539<br>ZFMK-DNA-0100433880	Italy, Sardinia Isl., NE of Aritzo, Gennargentu, Mt. di Iscudu, N40°01'10" E09°16'39", 1511m, 30-Sep-2010, *alder, moss*, creek, leg. Stüben,P., det. Stüben,P.
Dichotrachelus variegatus<br>Daniel, 1898	MK891346 [new]<br>1383-PST<br>658nt	ZFMK-TIS-4679<br>ZFMK-DNA-0155627271	Italy, Lazio, N of San Donato Val di Camino, south side of Forca d'Acero, N41°44'36" E13°48'51", 1286m, 21-Aug-2013, *Fagus*, sieving, leg. Stüben,P., det. Stüben,P.

**Name**<br>**Authority**<br>**Additional Information**	**GenBank Acc No (Ref.)**<br>**Specimen ID**<br>**Sequence Length**	**ZFMK Tissue ID**<br>**ZFMK DNA Sample ID**<br>**(SDEI DNA Sample ID)**	**Locality, GPS, Collection Date, Plant, Collector, Identifier**
Dichromacalles albopictus<br>(Jacquet, 1888)	FJ716544 [01]<br>F-447-alb<br>658nt	ZFMK-TIS-cF447<br>ZFMK-DNA-0100400475	France, Alpes-Maritimes, 5 km S of Sospel, Mont Razet, N43°50'59" E07°28'28", 1269m, 28-Dez-2007, *Quercus ilex, Helleborus,* broom, leg. Stüben,P., det. Stüben,P.
Dichromacalles albopictus<br>(Jacquet, 1888)	GU987900 [01]<br>F-450-alb<br>619nt	ZFMK-TIS-cF450<br>ZFMK-DNA-0100400645	France, Alpes-de-Hautes-Provence, 11 km NE of Castellane near Soleilhas, Col de St. Barnabe, N43°51'55" E06°37'52", 1368m, 30-Dez-2007, *Helleborus,* broom, under stones, leg. Stüben,P., det. Stüben,P.
Dichromacalles albopictus<br>(Jacquet, 1888)	KF880840 [14]<br>815-PST<br>658nt	ZFMK-TIS-3728<br>ZFMK-DNA-0100413807	France, Vaucluse, Mt. Ventoux, N44°10'28" E05°16'44", 1700m, 06-Jul-2011, leg. Lempereur,J.-M., det. Stüben,P.
Dichromacalles algecirasensis<br>Stüben, 2013<br>**Paratype (DNAtype)**	EU286500 [01]<br>E-0204-dio<br>658nt	ZFMK-TIS-cE0204<br>ZFMK-DNA-0100400119	Spain, Cadiz, 6.5 km S of Algeciras, Punta del Carnero, N36°04'35" W05°25'46", 34m, 12-Apr-2007, *Olea, Pistacia lentiscus, thistle*, leg. Astrin,J. & Stüben,P., det. Stüben,P.
Dichromacalles algecirasensis<br>Stüben, 2013	GU987944 [01]<br>E-590-dio<br>658nt	ZFMK-TIS-cE590<br>ZFMK-DNA-0100404960	Spain, Andalucia, Costa de la Luz, Barbate, N36°11'16" W5°55'18", 9m, 04-Mrz-2008, leg. Mueller,G., det. Mueller,G.
Dichromacalles algecirasensis<br>Stüben, 2013	GU988068 [01]<br>E-782-dio<br>658nt	ZFMK-TIS-cE782<br>ZFMK-DNA-0100405021	Spain, Cadiz, La Linea, Sierra Carbonera, Puerto Higuerón, Cortijo Los Puertos, N36°12' W05°19', 18-Nov-2008, *Cynara alba*, leg. Torres,J. L., det. Torres,J. L.
Dichromacalles algecirasensis<br>Stüben, 2013	GU988074 [01]<br>E-793-dio<br>658nt	ZFMK-TIS-cE793<br>ZFMK-DNA-0100405013	Spain, Cadiz, La Linea, Sierra Carbonera, Puerto Higuerón, Cortijo Los Puertos, N36°12' W05°19', 18-Nov-2008, *Cynara*, leg. Torres,J. L., det. Torres,J. L.
Dichromacalles andalusiensis<br>Stüben, 2014	KF880842 [14]<br>1261-PST<br>658nt	ZFMK-TIS-3597<br>ZFMK-DNA-0100417865	Spain, Andalucia, Sierra Nevada Mts., Canadillas, N37°07'29" W03°25'11", 1885m, 10-Jun-2012, leg. Stüben,P., det. Stüben,P.
Dichromacalles andalusiensis<br>Stüben, 2014<br>**Paratype (DNAtype)**	KF880843 [14]<br>1306-PST_4122<br>658nt	ZFMK-TIS-4122<br>ZFMK-DNA-0100426175	Spain, Jaen, SE of Mancha Real, Sierra Almaden (El Almaden, **type locality**), N37°44'06" W03°31'51", 1867m, 15-Mai-2013, *Astragalus* sp., *Bupleurum spinosum, Euphorbia nicaeensis*, sieving, leg. Stüben,P. & Schütte,A., det. Stüben,P.
Dichromacalles andalusiensis<br>Stüben, 2014<br>**Paratype (DNAtype)**	KF880844 [14]<br>1334-PST_4150<br>658nt	ZFMK-TIS-4150<br>ZFMK-DNA-0100426142	Spain, Jaen, Sierra Magina, SE of Torres, N37°44'06" W03°30'13", 1663m, 19-Mai-2013, *Erinacea anthyllis, Euphorbia nicaensis*, sieving, leg. Stüben,P. & Schütte,A., det. Stüben,P.
Dichromacalles boroveci<br>Stüben, 1998	MG229747 [new]<br>1818-PST<br>658nt	ZFMK-TIS-24123<br>ZFMK-DNA-0171624046	Greece, Rhodes Isl., above Salakos, N36°17'07" E27°56'24", 267m, 04-Mai-2014, sieving, leg. Bahr,F., det. Bahr,F.
Dichromacalles boroveci<br>Stüben, 1998	MG322643 [new]<br>GR-953-bor<br>658nt	ZFMK-TIS-cGR953<br>ZFMK-DNA-0112704669	Greece, Samos Isl., near Karlóvassi, N37°47'15" E26°39'26", 40m, 02-Okt-2009, *Euphorbia characias*, leg. Mueller,G., det. Mueller,G., Note: specimen was sequenced two times with different primers, retrieving the same sequence, see GenBank JX228951 [09].
Dichromacalles **cf.** *algecirasensis*	GU988149 [01]<br>Mo-925-dio<br>658nt	ZFMK-TIS-cMo925<br>ZFMK-DNA-0100404806	Morocco, Sebta West, vir. Punta Leona, N35°54'22" W05°28'55", 29m, 09-Mai-2009, *Pistacia*, leg. Stüben,P., det. Stüben,P.
Dichromacalles **cf.** *algecirasensis*	JX228952 [09]<br>ES1057<br>658nt	ZFMK-TIS-cES1057<br>ZFMK-DNA-0112704612	Spain, Malaga, Algatocin near Opayar, 36°34'39"N 05°18'13"W, 576m, 17-Aug-2010, *Picris*, stream valley, leg. Stüben,P., det. Stüben,P.

**Name**<br>**Authority**<br>**Additional Information**	**GenBank Acc No (Ref.)**<br>**Specimen ID**<br>**Sequence Length**	**ZFMK Tissue ID**<br>**ZFMK DNA Sample ID**<br>**(SDEI DNA Sample ID)**	**Locality, GPS, Collection Date, Plant, Collector, Identifier**
Dichromacalles creticus<br>(Reitter, 1916)	EU286463 [01]<br>GR-0130-cre<br>658nt	ZFMK-TIS-cGR0130<br>ZFMK-DNA-0100400893	Greece, Crete Isl. West, Omalos, N35°20'44" E23°54'38", 1050m, 28-Sep-2006, *Cichorium spin.*, leg. Bahr,F. & Bayer,B., det. Bahr and Bayer
Dichromacalles diocletianus<br>(Germar, 1817)	GU988152 [01]<br>F-933-dio<br>658nt	ZFMK-TIS-cF933<br>ZFMK-DNA-0100405261	France, Dep. Vaucluse, Avignon near Montfavet, N43°56'20" E04°50'47", 31m, 19-Jul-2009, *Picris echioides*, leg. Stüben,P., det. Stüben,P.
Dichromacalles diocletianus<br>(Germar, 1817)	GU988158 [01]<br>F-934-dio<br>658nt	ZFMK-TIS-cF934<br>ZFMK-DNA-0100405239	France, Dep. Vaucluse, Avignon near Montfavet, N43°56'15" E04°51'17", 22m, 20-Jul-2009, *Carduus pycnocephalus*, leg. Stüben,P., det. Stüben,P.
Dichromacalles diocletianus<br>(Germar, 1817)	KJ867576 [new]<br>692-FBA<br>658nt	ZFMK-TIS-3029<br>ZFMK-DNA-0100448195	Greece, Peloponnese, Achaea, Diakoft, coast, N38°12'06" E22°11'44", 2m, 03-Jun-2011, *Picris echioides*, leg. Bayer,B., det. Stüben,P.
Dichromacalles diocletianus<br>(Germar, 1817)	KJ867623 [new]<br>693-FBA<br>658nt	ZFMK-TIS-3030<br>ZFMK-DNA-0100417863	Greece, Peloponnese, Messinia, SW of Mt. Taygetos, Agh.Dimitrios, N36°48'25" E22°17'58", 50m, 20-Mai-2011, hand collecting, leg. Bahr,F. et al., det. Stüben,P.
Dichromacalles diocletianus<br>(Germar, 1817)	MG229812 [new]<br>2813b-FBA<br>658nt	ZFMK-TIS-3315<br>ZFMK-DNA-FC17941547	Greece, Attica, Lake Marathonas W, N38°08'00" E23°52'00", 380m, 09-Jun-2015, meadow, sieving, leg. Bahr,F., det. Stüben,P.
Dichromacalles diocletianus<br>(Germar, 1817)	GU987986 [01]<br>I-648-dio<br>658nt	ZFMK-TIS-cI648<br>ZFMK-DNA-0100404895	Italy, Calabria, Monti di Orsomarso, 7 km SW of Orsomarso near Marcellina, N39°46'17" E15°50'17", 35m, 09-Jul-2008, leg. Stüben,P., det. Stüben,P.
Dichromacalles dromedarius<br>(Boheman, 1844)	JN701886 [16]<br>29-PST<br>641nt	ZFMK-TIS-2D100446919<br>ZFMK-DNA-0100437933	Spain, Canary Islands, Gran Canaria, Gueva Grande, Camaretas, N27°59'15" W15°33'59", 1397m, 23-Dez-2010, *Andryala pinnatifida*, beating, leg. Stüben,P., det. Stüben,P.
Dichromacalles dromedarius<br>(Boheman, 1844)	EU286469 [01]<br>C-0127-dro<br>658nt	ZFMK-TIS-cC0127<br>ZFMK-DNA-0100400193	Spain, Canary Islands, La Palma, 7.5 km E of Garafia, Bco. de los Hombres, N28°49'50" W17°52'03", 386m, 10-Jul-2006, *Sonchus hierrensis*, leg. Stüben,P., det. Stüben,P.
Dichromacalles dromedarius<br>(Boheman, 1844)	MG229777 [40]<br>2003-JKR<br>658nt	ZFMK-TIS-23643<br>ZFMK-DNA-0169170498	Spain, Canary Islands, La Palma, El Tablado, N28°49'41" W17°52'43", 496m, 13-Feb-2014, *Sonchus palmensis*, beating, leg. Krátky,J., det. Krátky,J.
Dichromacalles dromedarius<br>(Boheman, 1844)	GU987917 [01]<br>P-515-dro<br>658nt	ZFMK-TIS-cP515<br>ZFMK-DNA-0100400610	Portugal, Madeira, 1 km NE of Machico, Pico do Facho, N32°43'33" W16°45'34", 212m, 17-Mrz-2008, *Tolpis succulenta*, leg. Stüben,P., det. Stüben,P.
Dichromacalles dromedarius<br>(Boheman, 1844)	MG229753 [40]<br>1874-PST<br>658nt	ZFMK-TIS-26077<br>ZFMK-DNA-0171606092	Portugal, Madeira, Ponta de Sao Lourenco, N32°44'39" W16°42'40", 54m, 09-Jul-2014, *Cynara cardunculus*, beating, leg. Stüben,P., det. Stüben,P.
Dichromacalles dromedarius<br>(Boheman, 1844)	MG229810 [40]<br>2793-PST<br>658nt	ZFMK-TIS-26147<br>ZFMK-DNA-0169166949	Portugal, Madeira, Ponta do Pargo, N32°48'51" W17°15'35", 334m, 04-Apr-2015, ruderal vegetation, collecting by hand, leg. Stüben,P., det. Stüben,P.
Dichromacalles dromedarius<br>(Boheman, 1844)	MG229818 [40]<br>2850-PST<br>658nt	ZFMK-TIS-4245<br>ZFMK-DNA-FC17941471	Portugal, Madeira, Sao Vincente, N32°48'40" W17°02'40", 11m, 03-Dez-2015, *Sonchus*, sieving, leg. Stüben,P. & Schütte,A., det. Stüben,P.
Dichromacalles dromedarius<br>(Boheman, 1844)	GU988142 [01]<br>E-905-dro<br>658nt	ZFMK-TIS-cE905<br>ZFMK-DNA-0100405483	Morocco, Sebta West, vir. Punta Leona, N35°54'22" W05°28'55", 29m, 09-Mai-2009, *Pistacia*, leg. Stüben,P., det. Stüben,P.

**Name**<br>**Authority**<br>**Additional Information**	**GenBank Acc No (Ref.)**<br>**Specimen ID**<br>**Sequence Length**	**ZFMK Tissue ID**<br>**ZFMK DNA Sample ID**<br>**(SDEI DNA Sample ID)**	**Locality, GPS, Collection Date, Plant, Collector, Identifier**
Dichromacalles dromedarius<br>(Boheman, 1844)	EU286517 [01]<br>E-0208-dro<br>658nt	ZFMK-TIS-cE0208<br>ZFMK-DNA-0100400384	Spain, Cadiz, 10 km NW of Tarifa, Punta Palomas, N36°03'47" W05°42'04", 14m, 12-Apr-2007, *Pistacia lentiscus,* broom, at the sea, leg. Astrin,J. & Stüben,P., det. Stüben,P.
Dichromacalles dromedarius<br>(Boheman, 1844)	GU988062 [01]<br>E-771-dro<br>658nt	ZFMK-TIS-cE771<br>ZFMK-DNA-0100405026	Spain, Cadiz, N of La Linea, Torre Carbonera, N36°13'47" W05°19'03", 8m, 14-Jan-2009, *Ulex, thistle, Pistatia, Acacia*, leg. Stüben,P., det. Stüben,P.
Dichromacalles dromedarius<br>(Boheman, 1844)	GU988076 [01]<br>E-796-dro<br>658nt	ZFMK-TIS-cE796<br>ZFMK-DNA-0100405011	Spain, Cadiz, La Linea, Playa Levante, Sobrevela near Asansull (school), N36°12' W05°19', 25-Nov-2008, *Eucaliptus*, leg. Torres,J. L., det. Torres,J. L.
Dichromacalles dromedarius<br>(Boheman, 1844)	GU988012 [01]<br>E-708-dro<br>658nt	ZFMK-TIS-cE708<br>ZFMK-DNA-0100404847	Spain, Canary Islands, Tenerife, W of Los Silos, Teno Mts., Casa Blanca near Buenavista del Norte, Levada, N28°21'35" W16°52'05", 230m, 03-Okt-2008, *Sonchus, Foeniculum vulgare*, leg. Astrin,J. & Stüben,P., det. Stüben,P.
Dichromacalles lentisci<br>(Chevrolat, 1861)	GU988141 [01]<br>E-904-len<br>658nt	ZFMK-TIS-cE904<br>ZFMK-DNA-0100405482	Morocco, Sebta West, vir. Punta Leona, N35°54'22" W05°28'55", 29m, 09-Mai-2009, *Pistacia*, leg. Stüben,P., det. Stüben,P.
Dichromacalles lentisci<br>(Chevrolat, 1861)	GU988067 [01]<br>E-781-len<br>658nt	ZFMK-TIS-cE781<br>ZFMK-DNA-0100405022	Spain, Cadiz, Estación San Roque near photovoltaics factory, at Rio Guadarranque, N36°12' W05°26', 11-Jul-2008, leg. Torres,J. L., det. Torres,J. L.
Dichromacalles lentisci<br>(Chevrolat, 1861)	GU988070 [01]<br>E-786-len<br>658nt	ZFMK-TIS-cE786<br>ZFMK-DNA-0100405018	Spain, Cadiz, Estación San Roque near photovoltaics factory, at Rio Guadarranque, N36°12' W05°26', 19-Sep-2008, *Foeniculum vulgare*, leg. Torres,J. L., det. Torres,J. L.
Dichromacalles querilhaci<br>(H. Brisout de Barneville, 1864)	GU988153[01]<br>F-935-que<br>658nt	ZFMK-TIS-cF935<br>ZFMK-DNA-0100404782	France, Dep. Tarn, E of Albi, Cahuzaguet (river Tarn), N43°55'59" E02°15'17", 176m, 22-Jul-2009, *Andryala integrifolia* , leg. Stüben,P., det. Stüben,P.
Dichromacalles rolletii<br>(Germar, 1839)	GU987901 [01]<br>F-452-rol<br>658nt	ZFMK-TIS-cF452<br>ZFMK-DNA-0100400643	France, Alpes-Maritimes, 8 km E of Nizza, between Eze Village and Eze sur Mer, N43°43'46" E07°21'40", 343m, 02-Jan-2008, *Euphorbia dendroides*, leg. Stüben,P., det. Stüben,P.
Dichromacalles rolletii<br>(Germar, 1839)	KJ867575 [new]<br>670-FBA<br>639nt	ZFMK-TIS-20451<br>ZFMK-DNA-0100448597	Greece, Peloponnese, Messinia, SW of Mt. Taygetos, Agh. Dimitrios, N36°48'25" E22°17'58", 50m, 20-Mai-2011, leg. Bahr,F., det. Bahr,F.
Dichromacalles rolletii<br>(Germar, 1839)	EU286470 [01]<br>I-0129-rol<br>658nt	ZFMK-TIS-cI0129<br>ZFMK-DNA-0100400191	Italy, Sicilia Isl. (ME), 2 km N of San Fratello, P.N. dei Nebrodi, N38°01'59" E14°35'56", 700m, 21-Jul-2005, *Euphorbia dendroides*, leg. Stüben,P., det. Stüben,P.
Dichromacalles rolletii<br>(Germar, 1839)	GU987867 [01]<br>I-0276-rol<br>611nt	ZFMK-TIS-cI0276<br>ZFMK-DNA-0100401038	Italy, Sicilia Isl. (PA), Mt. Madonna di Alto 3 km N of Castellana Sicula, P.R. Madonie, N37°48'32" E14°02'24", 950m, 21-Jul-2005, *Euphorbia myrsinites*, leg. Stüben,P., det. Stüben,P.
Dichromacalles rolletii<br>(Germar, 1839)	MG229717 [new]<br>1650-JKR<br>658nt	ZFMK-TIS-4228<br>ZFMK-DNA-0155630490	Italy, Sicilia Isl. (TP), Trapani, San Vito Lo Capo, N38°10'09" E12°43'13", 54m, 23-Sep-2013, *Euphorbia*, individual collecting by hand, leg. Stejskal,R., det. Stejskal,R.
Dichromacalles tuberculatus<br>(Rosenhauer, 1856)	GU988140 [01]<br>E-903-tub<br>658nt	ZFMK-TIS-cE903<br>ZFMK-DNA-0100405481	Morocco, Sebta West, vir. Punta Leona, N35°54'22" W05°28'55", 29m, 09-Mai-2009, *Pistacia*, leg. Stüben,P., det. Stüben,P.
Dichromacalles tuberculatus<br>(Rosenhauer, 1856)	KF880841 [14]<br>829-PST<br>658nt	ZFMK-TIS-3742<br>ZFMK-DNA-0100414262	Portugal, Minho, PN of Peneda-Geres, Britelo, N41°49'29" W08°18'35", 83m, 09-Mai-2012, beating, leg. Stüben,P., det. Stüben,P.

**Name**<br>**Authority**<br>**Additional Information**	**GenBank Acc No (Ref.)**<br>**Specimen ID**<br>**Sequence Length**	**ZFMK Tissue ID**<br>**ZFMK DNA Sample ID**<br>**(SDEI DNA Sample ID)**	**Locality, GPS, Collection Date, Plant, Collector, Identifier**
Dichromacalles tuberculatus<br>(Rosenhauer, 1856)	EU286515 [01]*<br>E-0203-tub<br>658nt	ZFMK-TIS-cE0203<br>ZFMK-DNA-0100400386	Spain, Cadiz, 6.5 km S of Algeciras, Punta del Carnero, N36°04'35" W05°25'46", 34m, 12-Apr-2007, *Olea, Pistacia lentiscus, thistle*, Garrigue, leg. Astrin,J. & Stüben,P., det. Stüben,P. *NOTE: frozen specimen was collected prior to MWI and sequenced two times with different primers: GenBank KF880839 [14] and EU286515 [01].
Dichromacalles tuberculatus<br>(Rosenhauer, 1856)	GU988064 [01]<br>E-773-tub<br>658nt	ZFMK-TIS-cE773<br>ZFMK-DNA-0100405028	Spain, Cadiz, NW of Tarifa, Sierra de Fates, N36°03'53" W05°38'58", 78m, 15-Jan-2009, *Quercus suber, Ulex*, willow, leg. Stüben,P., det. Stüben,P.
Dichromacalles tuberculatus<br>(Rosenhauer, 1856)	GU988069 [01]<br>E-783-tub<br>658nt	ZFMK-TIS-cE783<br>ZFMK-DNA-0100405020	Spain, Cadiz, La Linea, Sierra Carbonera, Puerto Higuerón, Cortijo Los Puertos, N36°12' W05°19', 18-Nov-2008, *Cynara alba*, leg. Torres,J. L., det. Torres,J. L.
Dichromacalles tuberculatus<br>(Rosenhauer, 1856)	GU988072 [01]<br>E-791-tub<br>658nt	ZFMK-TIS-cE791<br>ZFMK-DNA-0100405015	Spain, Cadiz, La Linea, Sierra Carbonera, Puerto Higuerón, Cortijo Los Puertos, N36°12' W05°19', 18-Nov-2008, *Cynara*, leg. Torres,J. L., det. Torres,J. L.
Dieckmanniellus nitidulus<br>(Gyllenhal, 1838)	KC784078 [new]<br>545-RST<br>658nt	ZFMK-TIS-20330<br>ZFMK-DNA-0100448434	Czech Republic, Moravia mer., Podyji NP, 0.6 km NE of Hnanice, N48°48'6.90" E15°59'29.96", 280m, 01-Sep-2011, *Lythrum hyssopifolium*, sweeping, leg. Stejskal,R., det. Stejskal,R.
Dieckmanniellus nitidulus<br>(Gyllenhal, 1838)	KC784316 [40]<br>767-PST<br>658nt	ZFMK-TIS-3104<br>ZFMK-DNA-0100448120	Spain, Canary Islands, La Gomera, Arure, Ermita Virgen de La Salud, N28°7'59" W17°19'10", 817m, 02-Mrz-2012, beating, leg. Stüben,P., det. Stüben,P.
Diplapion confluens<br>(Kirby, 1808)	KC784267 [new]<br>IT-0040w<br>658nt	ZFMK-TIS-2D100446551<br>ZFMK-DNA-0100437837	Italy, Sardinia Isl. East, E of Siniscola, La Caletta, coast, N40°35'30" E09°45'25", 3m, 09-Okt-2010, leg. Stüben,P., det. Stüben,P.
Diplapion stolidum<br>(Germar, 1817)	MK890830 [new]<br>277-PSP<br>647nt	ZFMK-TIS-2D100438557<br>ZFMK-DNA-0100439766	Germany, Lower Saxony (NI), Pattensen, Hannover region, N52°14'35" E09°45'21", 77m, 17-Mai-2011, *Prunus spinosa*, roadside shrub, beating, leg. Sprick,P., det. Sprick,P.
Diplapion stolidum<br>(Germar, 1817)	MK891044 [new]<br>943-CBR<br>658nt	ZFMK-TIS-3856<br>ZFMK-DNA-0100449669	Luxembourg, 10 km E of Luxembourg, 1 km SW of Mensdorf, N49°39'32" E06°18'35", 300m, 09-Jun-2012, *Chrysanthemum leucanthemum*, sweeping, leg. Braunert,C., det. Braunert,C.
Diplapion westwoodi<br>(Wollaston, 1864)	KC783745 [40]<br>30-PST<br>654nt	ZFMK-TIS-2D100446918<br>ZFMK-DNA-0100438089	Spain, Canary Islands, Gran Canaria, Gueva Grande, Camaretas, N27°59'15" W15°33'59", 1397m, 23-Dez-2010, *Argyranthemum* cf. *adauctum*, beating, leg. Stüben,P., det. Stüben,P.
Diplapion westwoodi<br>(Wollaston, 1864)	MK891621 [40]<br>1729-PST<br>658nt	ZFMK-TIS-24447<br>ZFMK-DNA-0155622462	Spain, Canary Islands, La Palma, E of Barlovento near Franceses, N28°49'33" W17°51'29", 421m, 31-Dez-2013, *Argyranthemum*, beating, leg. Stüben,P., det. Stüben,P.
Diplapion westwoodi<br>(Wollaston, 1864)	MK890906 [40]<br>716-PST<br>658nt	ZFMK-TIS-3053<br>ZFMK-DNA-0100448171	Spain, Canary Islands, Tenerife, Teno Mts., Los Carrizales, Bco. del Carrizal, N28°19'14" W16°52'03", 434m, 13-Jan-2012, *Argyranthemum* cf. *frutescens*, beating, leg. Stüben,P., det. Stüben,P.
Dissoleucas niveirostris<br>(Fabricius, 1798)	MK890887 [new]<br>534-RST<br>658nt	ZFMK-TIS-20319<br>ZFMK-DNA-0100448444	Czech Republic, Bohemia occ., Belysov Nature Reserve, 2.5 km SE of Chudenice, N49°26'49.54" E13°11'53.68", 540m, 28-Aug-2011, beating, leg. Stejskal,R., det. Stejskal,R.
Dodecastichus consentaneus<br>(Boheman, 1842)	MK891334 [new]<br>1358a-PST<br>658nt	ZFMK-TIS-4654<br>ZFMK-DNA-0155628575	Italy, Abruzzo, S. Stefano di Sessanio, N42°20'24" E13°37'30", 1271m, 14-Aug-2013, *Ostrya*, sieving, leg. Stüben,P., det. Sprick,P.

**Name**<br>**Authority**<br>**Additional Information**	**GenBank Acc No (Ref.)**<br>**Specimen ID**<br>**Sequence Length**	**ZFMK Tissue ID**<br>**ZFMK DNA Sample ID**<br>**(SDEI DNA Sample ID)**	**Locality, GPS, Collection Date, Plant, Collector, Identifier**
Dodecastichus geniculatus<br>(Germar, 1817)	MK892017 [new]<br>2290-JKR<br>658nt	ZFMK-TIS-24221<br>ZFMK-DNA-0169170975	Austria, Styria, Trofaiach, Rötz, N47°26'56" E15°01'39", 758m, 28-Jun-2014, mountain forest, sweeping, leg. Krátky,J., det. Krátky,J.
Dodecastichus inflatus<br>(Gyllenhal, 1834)	MK891263 [new]<br>1200-JKR<br>658nt	ZFMK-TIS-3536<br>ZFMK-DNA-0100426084	Czech Republic, Bohemia, Hradec Kralove, N50°13'05" E15°50'16", 232m, 18-Jul-2012, nihgt beating, leg. Krátky,J., det. Krátky,J.
Dodecastichus inflatus<br>(Gyllenhal, 1834)	MK890820 [new]<br>206-PSP<br>658nt	ZFMK-TIS-2D100438561<br>ZFMK-DNA-0100439546	Germany, Bavaria (BY), Peißenberg-Wörth, N47°47'03" E11°04'31", 695m, 24-Jul-2010, *Thuja occidentalis*, garden, beating, leg. Sprick,P., det. Sprick,P.
Dodecastichus ovoideus<br>(Reitter, 1913)	MK891342 [new]<br>1379-PST<br>658nt	ZFMK-TIS-4675<br>ZFMK-DNA-0155628561	Italy, Abruzzo, SE of L'Aquila near Rocca di Cambio Parco Sirente-Yelino, N42°16'31" E13°27'47", 1012m, 18-Aug-2013, *hazelnut*, mountain mixed forest, sieving, leg. Stüben,P., det. Sprick,P.
Donus comatus<br>(Boheman, 1842)	MK891266 [new]<br>1205-JKR<br>658nt	ZFMK-TIS-3541<br>ZFMK-DNA-0100426074	Czech Republic, Moravia, Hruby jesenik, Velka Kotlina, N50°3'18" E17°14'14", 1209m, 23-Aug-2012, *Aconitum*, individeuell Pfalnzenklopf, leg. Krátky,J., det. Krátky,J.
Donus comatus<br>(Boheman, 1842)	MK892069 [new]<br>2345-JKR<br>658nt	ZFMK-TIS-24276<br>ZFMK-DNA-0169170915	Czech Republic, Bohemia, Horni Morava, N50°10'15" E16°49'22", 780m, 17-Jul-2014, sweeping, leg. Krátky,J., det. Krátky,J.
Donus comatus<br>(Boheman, 1842)	MK892048 [new]<br>2324-JKR<br>658nt	ZFMK-TIS-24255<br>ZFMK-DNA-0169170942	Slovakia, Tatry Mts., Dolina Siedmich prame?ov, N49°13'30" E20°16'24", 1404m, 05-Jul-2014, *mountain meadow*, sweeping, leg. Krátky,J., det. Krátky,J.
Donus ovalis<br>(Boheman, 1842)	MK891020 [new]<br>917-CBR<br>658nt	ZFMK-TIS-3830<br>ZFMK-DNA-0100449691	Germany, North Rhine-Westphalia (NRW), 7 km W of Wesel, N51°39'55" E06°30'19", 15m, 10-Jun-2011, beating, leg. Braunert,C., det. Braunert,C.
Donus ovalis<br>(Boheman, 1842)	MK891433 [new]<br>1496-PSP<br>658nt	ZFMK-TIS-3256<br>ZFMK-DNA-0155633334	Germany, Saxony-Anhalt (ST), Harz National Park, Schierke, Feuersteinwiesen, N51°45'23" E10°41'36", 591m, 01-Aug-2012, *Cirsium oleraceum*, beating, leg. Sprick,P., det. Sprick,P.
Donus oxalis<br>(Herbst, 1795)	MK891997 [new]<br>2268-JKR<br>658nt	ZFMK-TIS-24199<br>ZFMK-DNA-0169170997	Czech Republic, Bohemia, Lany env, Brejl, N50°06'14" E13°52'59", 356m, 23-Jun-2014, sweeping, leg. Putz,M., det. Putz,M.
Donus philanthus<br>(Olivier, 1807)	KC784236 [new]<br>IT-0003w<br>648nt	ZFMK-TIS-2D100446553<br>ZFMK-DNA-0100437872	Italy, Sardinia Isl. East, W of Siniscola, Monte Albo, N40°33'37" E09°38'01", 778m, 26-Sep-2010, *Quercus ilex*, cliff, limestone, leg. Stüben,P., det. Stüben,P.
Donus salviae<br>(Schrank, 1789)	MK891343 [new]<br>1380-PST<br>658nt	ZFMK-TIS-4676<br>ZFMK-DNA-0155628562	Italy, Abruzzo, SE of L'Aquila near Rocca di Cambio Parco Sirente-Yelino, N42°16'31" E13°27'47", 1012m, 18-Aug-2013, *hazelnut*, mountain mixed forest, sieving, leg. Stüben,P., det. Winkelmann,H.
Donus tessellatus<br>(Boheman, 1834)	KC784093 [new]<br>527-RST<br>658nt	ZFMK-TIS-20312<br>ZFMK-DNA-0100448452	Romania, Caras-Severin, Sfanta Elena env., N44°40'54.68" E21°42'39.99", 400m, 26-Jul-2011, night-sweeping, leg. Stejskal,R., det. Stejskal,R.
Donus tessellatus<br>(Boheman, 1834)	MK892141 [new]<br>2428-JKR<br>658nt	ZFMK-TIS-26005<br>ZFMK-DNA-0171600657	Slovakia, Nitra, Koli?any, Koli?ansky vrch, N48°20'15.5" E18°10'47", 261m, 21-Sep-2014, *Achillea millefolium*, sweeping, leg. Krátky,J., det. Krátky,J.
Donus viennensis<br>(Herbst, 1795)	MK890883 [new]<br>520-RST<br>658nt	ZFMK-TIS-20305<br>ZFMK-DNA-0100448459	Czech Republic, Bohemia bor., NP Podyji, Uhlirova louka meadow, 2.5 km S of cizov, N48°51'29.41" E15°52'45.26", 280m, 16-Jul-2011, *Chaerophyllum aromaticum*, beating, leg. Stejskal,R., det. Stejskal,R.

**Name**<br>**Authority**<br>**Additional Information**	**GenBank Acc No (Ref.)**<br>**Specimen ID**<br>**Sequence Length**	**ZFMK Tissue ID**<br>**ZFMK DNA Sample ID**<br>**(SDEI DNA Sample ID)**	**Locality, GPS, Collection Date, Plant, Collector, Identifier**
Donus viennensis<br>(Herbst, 1795)	MK892059 [new]<br>2335-JKR<br>658nt	ZFMK-TIS-24266<br>ZFMK-DNA-0169170930	Slovakia, Zilina, Rajec, Suja, N49°04'03" E18°36'31.5", 490m, 13-Jul-2014, sweeping, leg. Krátky,J., det. Krátky,J.
Dorytomus cf. *taeniatus*	KC784255 [new]<br>IT-0024w<br>658nt	ZFMK-TIS-2D100446534<br>ZFMK-DNA-0100433861	Italy, Sardinia Isl. South, E of Cagliari, national park M. d. Sette Fratelli near Monte Cresia, N39°15'42" E09°23'58", 684m, 03-Okt-2010, *Salix*, creek, leg. Stüben,P., det. Stüben,P.
Dorytomus dejeani<br>Faust, 1883	KC784130 [new]<br>656-PSP<br>658nt	ZFMK-TIS-20439<br>ZFMK-DNA-0100448614	Germany, Lower Saxony (NI), Hannover, Herrenhausen, N52°23'27" E09°40'59", 50m, 03-Nov-2011, *Populus tremula*, Leine valley, beating, leg. Sprick,P., det. Sprick,P.
Dorytomus longimanus<br>(Forster, 1771)	KC784128 [new]<br>654-PSP<br>658nt	ZFMK-TIS-20437<br>ZFMK-DNA-0100448612	Germany, Lower Saxony (NI), Hannover, Herrenhausen, N52°23'32" E09°40'40", 49m, 03-Nov-2011, *Populus nigra*, Leine shore, beating, leg. Sprick,P., det. Sprick,P.
Dorytomus longimanus<br>(Forster, 1771)	MK891906 [new]<br>2173-JKR<br>658nt	ZFMK-TIS-23911<br>ZFMK-DNA-0169170614	Slovakia, Nove Zamky, Cenkov, NPR Cenkovska step, N47°46'09" E18°31'09", 110m, 19-Mai-2014, *Populus*, beating, leg. Stejskal,R., det. Stejskal,R.
Dorytomus majalis<br>(Paykull, 1792)	MK891156 [new]<br>1063-PSP<br>658nt	ZFMK-TIS-3208<br>ZFMK-DNA-0100426797	Germany, Lower Saxony (NI), Langeoog, N53°45'05" E07°32'24", 3m, 16-Jun-2012, *Salix repens*, small dune valley, beating, leg. Sprick,P., det. Sprick,P.
Dorytomus melanophthalmus<br>(Paykull, 1792)	MK890897 [new]<br>603-PSP<br>658nt	ZFMK-TIS-20386<br>ZFMK-DNA-0100448657	Germany, Lower Saxony (NI), Hannover, Herrenhausen, Leine river, N52°23'29" E09°40'40", 47m, 27-Sep-2011, *Salix viminalis*, beating, leg. Sprick,P., det. Sprick,P.
Dorytomus melanophthalmus<br>(Paykull, 1792)	MK891161 [new]<br>1069-PSP<br>658nt	ZFMK-TIS-3213<br>ZFMK-DNA-0100417600	Germany, Lower Saxony (NI), Langeoog, N53°45'03" E07°34'33", 2m, 16-Jun-2012, *Salix repens*, dune/wayside, beating, leg. Sprick,P., det. Sprick,P.
Dorytomus minutus<br>(Gyllenhal, 1835)	MK891242 [new]<br>1162-JKR<br>658nt	ZFMK-TIS-3498<br>ZFMK-DNA-0100449551	Hungary, Jasz-Nagykun-Szolnok megye, Tiszaroff, N47°21'55" E20°27'32", 87m, 25-Mai-2012, *Populus alba*, sweeping, leg. Krátky,J., det. Krátky,J.
Dorytomus nebulosus<br>(Gyllenhal, 1835)	MK890825 [new]<br>233-PSP<br>648nt	ZFMK-TIS-2D100439127<br>ZFMK-DNA-0100439799	Germany, Lower Saxony (NI), Hemmingen, Hannover region, N52°19'07" E09°43'58", 54m, 23-Apr-2011, *Populus nigra*, fallow, beating, leg. Sprick,P., det. Sprick,P.
Dorytomus rufatus rufatus<br>(Bedel, 1888)	KC783952 [new]<br>314-JKR<br>658nt	ZFMK-TIS-20100<br>ZFMK-DNA-0100438776	Czech Republic, Bohemia or., Dvakacovice, N49°58'35.447" E15°54'2.243", 245m, 14-Mai-2011, *Salix* sp., beating, leg. Krátky,J., det. Krátky,J.
Dorytomus salicis<br>Walton, 1851	MK891077 [new]<br>980-PSP<br>658nt	ZFMK-TIS-3125<br>ZFMK-DNA-0100426879	Germany, Lower Saxony (NI), near Hannover, Resse, edge of Helstorfer bog, N52°31'22" E09°35'57", 48m, 14-Apr-2012, *Salix aurita*, beating, leg. Sprick,P., det. Sprick,P.
Dorytomus taeniatus<br>(Fabricius, 1781)	KC784155 [new]<br>601-PSP<br>658nt	ZFMK-TIS-20384<br>ZFMK-DNA-0100448655	Germany, Lower Saxony (NI), Koldingen, Hannover region, Leine river, N52°16'30" E09°48'31", 57m, 12-Jun-2011, *Salix viminalis*, beating, leg. Sprick,P., det. Sprick,P.
Dorytomus taeniatus<br>(Fabricius, 1781)	MK891160 [new]<br>1067-PSP<br>658nt	ZFMK-TIS-3212<br>ZFMK-DNA-0100426801	Germany, Lower Saxony (NI), Langeoog, N53°45'05" E07°32'24", 3m, 16-Jun-2012, *Salix repens*, small dune valley, beating, leg. Sprick,P., det. Sprick,P.
Dorytomus tortrix<br>(Linnaeus, 1760)	KC784129 [new]<br>655-PSP<br>656nt	ZFMK-TIS-20438<br>ZFMK-DNA-0100448613	Germany, Lower Saxony (NI), Hannover, Herrenhausen, N52°23'27" E09°40'59", 50m, 03-Nov-2011, *Populus tremula*, Leine valley, beating, leg. Sprick,P., det. Sprick,P.

**Name**<br>**Authority**<br>**Additional Information**	**GenBank Acc No (Ref.)**<br>**Specimen ID**<br>**Sequence Length**	**ZFMK Tissue ID**<br>**ZFMK DNA Sample ID**<br>**(SDEI DNA Sample ID)**	**Locality, GPS, Collection Date, Plant, Collector, Identifier**
Dorytomus tremulae<br>(Fabricius, 1787)	MK890902 [new]<br>646-PSP<br>658nt	ZFMK-TIS-20429<br>ZFMK-DNA-0100448621	Germany, Lower Saxony (NI), Hannover, Herrenhausen, N52°23'32" E09°40'40", 49m, 27-Sep-2011, *Populus nigra*, Leine shore, beating, leg. Sprick,P., det. Sprick,P.
Dorytomus tremulae<br>(Fabricius, 1787)	KC784068 [new]<br>561-RST<br>658nt	ZFMK-TIS-20346<br>ZFMK-DNA-0100448423	Slovakia, Slovakia occ., 0.7 km SE of Dolne Zelenice, N48°22'13.95" E17°45'8.62", 135m, 02-Okt-2011, *Populus alba*, beating, leg. Stejskal,R., det. Stejskal,R.
Dorytomus villosulus<br>(Gyllenhal, 1835)	KC784069 [new]<br>562-RST<br>658nt	ZFMK-TIS-20347<br>ZFMK-DNA-0100448424	Slovakia, Slovakia occ., 0.7 km SE of Dolne Zelenice, N48°22'13.95" E17°45'8.62", 135m, 02-Okt-2011, *Populus alba*, beating, leg. Stejskal,R., det. Stejskal,R.
Doydirhynchus austriacus<br>(Olivier, 1807)	KC783974 [new]<br>295-JKR<br>658nt	ZFMK-TIS-20081<br>ZFMK-DNA-0100438959	Czech Republic, Bohemia or., Hradec Kralove, Plachta, N50°11'12.892" E15°51'37.148", 230m, 19-Apr-2011, *Pinus silvestris*, beating, leg. Krátky,J., det. Krátky,J.
Drupenatus nasturtii<br>(Germar, 1824)	MK891282 [new]<br>1233-PST<br>658nt	ZFMK-TIS-3569<br>ZFMK-DNA-0100426243	Portugal, Estremadura, N of Ericeira, Ribamar, N38°59'29 W09°24'54", 28m, 18-Mai-2012, coast, beating, leg. Stüben,P., det. Krátky,J.
Echinodera adriatica<br>Stüben, 2008	GU213651 [01]<br>HR-0311-adr<br>658nt	ZFMK-TIS-cHR0311<br>ZFMK-DNA-0100400078	Croatia, Dalmatia, 21 km E of Split, Mosor Mts., N of Omis, Gata, N43°27'59" E16°41'40", 280m, 02-Jul-2007, *Olea, Quercus, Carpinus*, limestone: Olea, leg. Stüben,P., det. Stüben,P.
Echinodera adriatica<br>Stüben, 2008	GU213652 [01]<br>HR-0318-adr<br>658nt	ZFMK-TIS-cHR0318<br>ZFMK-DNA-0100400205	Croatia, Dalmatia, 7 km N of Makarska, Biokovo Mts., Bast, N43°21'26" E16°59'18", 415m, 05-Jul-2007, *Quercus*, limestone: arid hillside, leg. Stüben,P., det. Stüben,P.
Echinodera adriatica<br>Stüben, 2008	GU987869 [01]<br>HR-0328-adr<br>658nt	ZFMK-TIS-cHR0328<br>ZFMK-DNA-0100401027	Croatia, Dalmatia, 35 km E of Split, Mosor Mts., E of Omis, N of Slime, N43°25'46" E16°51'59", 69m, 11-Jul-2007, *Cetina, Robinia*, limestone, escaped garden at stream (moist), leg. Stüben,P., det. Stüben,P.
Echinodera adriatica<br>Stüben, 2008	MG229835 [new]<br>HR-0313-adr<br>658nt	ZFMK-TIS-24545<br>ZFMK-DNA-0155630405	Croatia, Dalmatia, 32 km E of Split, Mosor Mts., E of Omis, Kucice, N43°25'49" E16°49'00", 213m, 02-Jul-2007, *Cetina riv., Maccia; Olea, Quercus, Carpinus*, limestone, leg. Stüben,P., det. Stüben,P.
Echinodera adriatica<br>Stüben, 2008	MG229836 [new]<br>HR-0314-adr<br>658nt	ZFMK-TIS-24548<br>ZFMK-DNA-0155630406	Croatia, Dalmatia, 14 km E of Split, Mosor Mts., below G. Sitno, N43°30'41" E16°36'30", 498m, 03-Jul-2007, *Quercus, Carpinus*, limestone, leg. Stüben,P., det. Stüben,P.
Echinodera adriatica<br>Stüben, 2008	MG229837 [new]<br>HR-0316-adr<br>538nt	ZFMK-TIS-24552<br>ZFMK-DNA-0155630407	Croatia, Dalmatia, 20 km E of Split, Mosor Mts., W of Gata, N43°28'19" E16°40'58", 317m, 04-Jul-2007, *Quercus-Wald*, limestone, leg. Stüben,P., det. Stüben,P.
Echinodera adriatica<br>Stüben, 2008	MG229838 [new]<br>HR-0321-adr<br>658nt	ZFMK-TIS-24553<br>ZFMK-DNA-0155630408	Croatia, Dalmatia, 7 km N of Makarska, Biokovo Mts., Bast, N43°21'26" E16°59'18", 415m, 05-Jul-2007, *Kalk: Feuchthang, Quercus-Wald (13B)*, leg. Stüben,P., det. Stüben,P.
Echinodera adriatica<br>Stüben, 2008	MG229839 [new]<br>HR-0322-adr<br>658nt	ZFMK-TIS-24561<br>ZFMK-DNA-0155630409	Croatia, Dalmatia, 18 km E of Split, Mosor Mts., NW of Gata, Catici, N43°29'31" E16°39'43", 528m, 07-Jul-2007, *Quercus forest*, limestone, leg. Stüben,P., det. Stüben,P.
Echinodera adriatica<br>Stüben, 2008	MG229840 [new]<br>HR-0342-adr<br>658nt	ZFMK-TIS-24566<br>ZFMK-DNA-0155630410	Croatia, Dalmatia, 27 km E of Split, Mosor Mts., E of Omis, Podaspilje area, underneath Kula Mt., N43°25'43" E16°45'19", 248m, 19-Jul-2007, *Quercus forest*, limestone, leg. Stüben,P., det. Stüben,P.
Echinodera andalusiensis<br>Stüben, 2003	EU286516 [01]<br>E-0205-and<br>658nt	ZFMK-TIS-cE0205<br>ZFMK-DNA-0100400385	Spain, Cadiz, 10 km SW of Algeciras, El Bujeo, N36°04'10" W05°31'48", 257m, 12-Apr-2007, *Quercus suber*, leg. Astrin,J. & Stüben,P., det. Stüben,P.

**Name**<br>**Authority**<br>**Additional Information**	**GenBank Acc No (Ref.)**<br>**Specimen ID**<br>**Sequence Length**	**ZFMK Tissue ID**<br>**ZFMK DNA Sample ID**<br>**(SDEI DNA Sample ID)**	**Locality, GPS, Collection Date, Plant, Collector, Identifier**
Echinodera andalusiensis<br>Stüben, 2003	GU213667 [01]<br>E-0212-and<br>658nt	ZFMK-TIS-cE0212<br>ZFMK-DNA-0100400245	Spain, Malaga, 16 km N of Estepona, Sierra Bermeja, Jubrique, N36°33'49" W05°12'27", 557m, 13-Apr-2007, *Quercus suber*, leg. Astrin,J. & Stüben,P., det. Stüben,P.
Echinodera andalusiensis<br>Stüben, 2003	GU213740 [01]<br>E-780-and<br>658nt	ZFMK-TIS-cE780<br>ZFMK-DNA-0100404725	Spain, Cadiz, San Roque, Pinar Rey, entry to Fuente Alhaja, N36°12' W05°22', 30-Apr-2008, *Populus nigra*, leg. Torres,J. L., det. Torres,J. L.
Echinodera andalusiensis<br>Stüben, 2003	GU987833 [01]<br>E-0207-and<br>658nt	ZFMK-TIS-cE0207<br>ZFMK-DNA-0100400317	Spain, Cadiz, 10 km NW of Tarifa, Punta Palomas, N36°03'47" W05°42'04", 14m, 12-Apr-2007, *Pistacia lentiscus,* broom, at the sea, leg. Astrin,J. & Stüben,P., det. Stüben,P.
Echinodera andalusiensis<br>Stüben, 2003	GU987834 [01]<br>E-0209-and<br>658nt	ZFMK-TIS-cE0209<br>ZFMK-DNA-0100400029	Spain, Malaga, 11 km NW of Estepona, Sierra Crestellina, Casares, N36°27'06" W05°16'39", 359m, 12-Apr-2007, *Pistacia lentiscus, Olea, Smilax*, stream valley, leg. Astrin,J. & Stüben,P., det. Stüben,P.
Echinodera andalusiensis<br>Stüben, 2003	GU988055 [01]<br>E-764-and<br>658nt	ZFMK-TIS-cE764<br>ZFMK-DNA-0100405040	Spain, Malaga, NW of Estepona, Casares, Sierra Bermeja, N36°27'03" W05°15'56", 377m, 11-Jan-2009, *Quercus suber, Pistacia*, leg. Stüben,P., det. Stüben,P.
Echinodera andalusiensis<br>Stüben, 2003	GU988057 [01]<br>E-765-and<br>658nt	ZFMK-TIS-cE765<br>ZFMK-DNA-0100405036	Spain, Malaga, W of Estepona, N36°25'57" W05°14'42", 189m, 12-Jan-2009, *Quercus, Ceratonia, Pistacia*, stream, leg. Stüben,P., det. Stüben,P.
Echinodera andalusiensis<br>Stüben, 2003	GU988061 [01]<br>E-770-and<br>658nt	ZFMK-TIS-cE770<br>ZFMK-DNA-0100405025	Spain, Cadiz, N of La Linea, Torre Carbonera, N36°13'47" W05°19'03", 8m, 14-Jan-2009, *Ulex, thistle, Pistatia, Acacia*, leg. Stüben,P., det. Stüben,P.
Echinodera andalusiensis<br>Stüben, 2003	GU988063 [01]<br>E-772-and<br>658nt	ZFMK-TIS-cE772<br>ZFMK-DNA-0100405027	Spain, Cadiz, NW of La Linea, Castellar (Almoraima), N36°18'23" W05°26'30", 12m, 15-Jan-2009, *Quercus suber*, leg. Stüben,P., det. Stüben,P.
Echinodera andalusiensis<br>Stüben, 2003	GU988073 [01]<br>E-792-and<br>658nt	ZFMK-TIS-cE792<br>ZFMK-DNA-0100405014	Spain, Cadiz, La Linea, Sierra Carbonera, Puerto Higuerón, Cortijo Los Puertos, N36°12' W05°19', 18-Nov-2008, *Cynara*, leg. Torres,J. L., det. Torres,J. L.
Echinodera andalusiensis<br>Stüben, 2003	GU988075 [01]<br>E-794-and<br>658nt	ZFMK-TIS-cE794<br>ZFMK-DNA-0100405012	Spain, Cadiz, La Linea, Sierra Carbonera, Puerto Higuerón, Cortijo Los Puertos, N36°12' W05°19', 03-Dez-2008, *Chamaerops humilis*, leg. Torres,J. L., det. Torres,J. L.
Echinodera andalusiensis<br>Stüben, 2003	GU988077 [01]<br>E-795-and<br>658nt	ZFMK-TIS-cE795<br>ZFMK-DNA-0100404999	Spain, Cadiz, La Linea, Sierra Carbonera, Puerto del Higuerón, Cortijo los Puertos, N36°12' W05°19', 03-Dez-2008, *Pistacia lentiscus*, leg. Torres,J. L., det. Torres,J. L.
Echinodera andalusiensis<br>Stüben, 2003	MG322653 [new]<br>ES1056<br>658nt	ZFMK-TIS-cES1056<br>ZFMK-DNA-0112704613	Spain, Malaga, Algatocin near Opayar, 36°34'39"N 05°18'13"W, 576m, 17-Aug-2010, *Quercus*, stream valley, leg. Stüben,P., det. Stüben,P.
Echinodera angulipennis<br>Wollaston, 1864	FJ716571 [01]<br>E-703-ang<br>658nt	ZFMK-TIS-cE703<br>ZFMK-DNA-0100404241	Spain, Canary Islands, Tenerife, SW of Los Silos, Teno Mts. near Masca, N28°18'27" W16°50'29", 668m, 03-Okt-2008, *Ficus carica, Citrus*, gardens, leg. Astrin,J. & Stüben,P., det. Stüben,P.
Echinodera angulipennis<br>Wollaston, 1864	GU213715 [01]<br>E-690-ang<br>658nt	ZFMK-TIS-cE690<br>ZFMK-DNA-0100404246	Spain, Canary Islands, Tenerife, NE of La Laguna, Anaga Mts., Las Mercedes, N28°31'49" W16°17'12", 905m, 01-Okt-2008, *Laurisilva*, leg. Astrin,J. & Stüben,P., det. Stüben,P.
Echinodera angulipennis<br>Wollaston, 1864	GU987933 [01]<br>E-559-ang<br>658nt	ZFMK-TIS-cE559<br>ZFMK-DNA-0100404774	Spain, Canary Islands, Tenerife, 2 km NE of Icod, Sta. Barbara, N28°21'41" W16°41'00", 561m, 20-Dez-2003, thermophilic brushwood, leg. Stüben,P., det. Stüben,P.

**Name**<br>**Authority**<br>**Additional Information**	**GenBank Acc No (Ref.)**<br>**Specimen ID**<br>**Sequence Length**	**ZFMK Tissue ID**<br>**ZFMK DNA Sample ID**<br>**(SDEI DNA Sample ID)**	**Locality, GPS, Collection Date, Plant, Collector, Identifier**
Echinodera angulipennis<br>Wollaston, 1864	GU987934 [01]<br>E-560-ang<br>619nt	ZFMK-TIS-cE560<br>ZFMK-DNA-0100404978	Spain, Canary Islands, Tenerife, East Anaga Mts., E of La Laguna, E of Lomo de las Bodegas, N28°33'38" W16°09'20", 500m, 26-Dez-2003, cliff, leg. Stüben,P., det. Stüben,P.
Echinodera ariadnae<br>Bahr & Beyer, 2005	EU286479 [01]<br>GR-0151-ari<br>658nt	ZFMK-TIS-cGR0151<br>ZFMK-DNA-0100400173	Greece, Crete Isl. West, Levka Ori, Imbros, above the Imbros Gorge, N35°14'51" E24°10'30", 900m, 02-Okt-2006, *Quercus*, leg. Bahr,F. & Bayer,B., det. Bahr and Bayer
Echinodera aspromontensis<br>Stüben, 2008	GU213714 [01]<br>I-661-asp<br>658nt	ZFMK-TIS-cI661<br>ZFMK-DNA-0100404887	Italy, Abruzzo, P.N. Majella, Caramanico TermFiume Orfento, N42°09'48" E14°00'17", 511m, 18-Jul-2008, moist mixed forest, leg. Stüben,P., det. Stüben,P.
Echinodera aspromontensis<br>Stüben, 2008	GU213769 [01]<br>I-652-asp<br>658nt	ZFMK-TIS-cI652<br>ZFMK-DNA-0100405272	Italy, Campania, 4 km S of Cava de Tirreni (Salerno), Vietri sul Mare, N40°40'18" E14°43'14", 106m, 12-Jul-2008, *Quercus ilex*, stream valley, leg. Stüben,P., det. Stüben,P.
Echinodera aspromontensis<br>Stüben, 2008	GU987822 [01]<br>I-0415-asp2<br>630nt	ZFMK-TIS-cI0415<br>ZFMK-DNA-0100400266	Italy, Basilicata (5330), Mass. Pollino, Jazzicelli, N39°48' E16°13', 840m, 30-Mai-2002, *Quercus*, leg. Kapp,A., det. Kapp,A.
Echinodera aspromontensis<br>Stüben, 2008	GU987965 [01]<br>I-620-asp<br>658nt	ZFMK-TIS-cI620<br>ZFMK-DNA-0100404923	Italy, Campania, Cilento, 7 km SW of Vallo d. Lucania, Petrosa, N40°11'30" E15°12'14", 110m, 30-Jun-2008, *Quercus, Pistacia lentiscus* , leg. Stüben,P., det. Stüben,P.
Echinodera aspromontensis<br>Stüben, 2008	GU987973 [01]<br>I-631-asp<br>658nt	ZFMK-TIS-cI631<br>ZFMK-DNA-0100404905	Italy, Campania, Cilento, 14 km SW of Vallo d. Lucania, Marina di Ascea, N40°07'42" E15°10'53", 13m, 30-Jun-2008, *Quercus ilex*, leg. Stüben,P., det. Stüben,P.
Echinodera aspromontensis<br>Stüben, 2008	GU987974 [01]<br>I-632-asp<br>656nt	ZFMK-TIS-cI632<br>ZFMK-DNA-0100404906	Italy, Campania, Monti Picentini, 4 km E of Acerno, N40°44'17" E15°05'43", 831m, 03-Jul-2008, *Fagus, Ilex*, leg. Stüben,P., det. Stüben,P.
Echinodera aspromontensis<br>Stüben, 2008	GU987975 [01]<br>I-633-asp<br>658nt	ZFMK-TIS-cI633<br>ZFMK-DNA-0100404907	Italy, Campania, Monti Picentini, 4 km E of Acerno, N40°44'17" E15°05'43", 831m, 03-Jul-2008, *Fagus, Ilex*, leg. Stüben,P., det. Stüben,P.
Echinodera aspromontensis<br>Stüben, 2008	GU987980 [01]<br>I-640-asp<br>658nt	ZFMK-TIS-cI640<br>ZFMK-DNA-0100404914	Italy, Basilicata, Monte Pollino, 9 km E of Rotonda, Timpa d. Demonio, N39°57'31" E16°08'39", 1254m, 05-Jul-2008, *Fagus*, cliff, leg. Stüben,P., det. Stüben,P.
Echinodera aspromontensis<br>Stüben, 2008	GU987982 [01]<br>I-642-asp<br>658nt	ZFMK-TIS-cI642<br>ZFMK-DNA-0100404901	Italy, Basilicata, Monte Pollino, 4 km SE of Rotonda, Zarafa, N39°56'33" E16°04'28", 677m, 07-Jul-2008, *Quercus ilex*, leg. Stüben,P., det. Stüben,P.
Echinodera aspromontensis<br>Stüben, 2008	GU987984 [01]<br>I-645-asp<br>655nt	ZFMK-TIS-cI645<br>ZFMK-DNA-0100404898	Italy, Basilicata, Monte Pollino, 9 km NW of Rotonda, Castelluccio-Sup., N40°00'37" E15°57'59", 653m, 07-Jul-2008, *Quercus, Castanea*, city forest, leg. Stüben,P., det. Stüben,P.
Echinodera aspromontensis<br>Stüben, 2008	GU987985 [01]<br>I-647-asp<br>658nt	ZFMK-TIS-cI647<br>ZFMK-DNA-0100404242	Italy, Basilicata, Monte Pollino, 17 km N of Rotonda, N of Episcopia, N40°05'55" E16°07'13", 894m, 08-Jul-2008, *Quercus, Castanea*, river, leg. Stüben,P., det. Stüben,P.
Echinodera aspromontensis<br>Stüben, 2008	GU987987 [01]<br>I-649-asp<br>658nt	ZFMK-TIS-cI649<br>ZFMK-DNA-0100404326	Italy, Calabria, Monti di Orsomarso, 3 km N of Orsomarso near Timpone Garramillo, N39°49'36" E15°55'31", 751m, 10-Jul-2008, *Fagus, Quercus ilex*, plateau (high plain), leg. Stüben,P., det. Stüben,P.
Echinodera aspromontensis<br>Stüben, 2008	GU987992 [01]<br>I-660-asp<br>658nt	ZFMK-TIS-cI660<br>ZFMK-DNA-0100404886	Italy, Abruzzo, 28 km SE of Pescara, S. Vito, N42°17'30" E14°27'35", 79m, 17-Jul-2008, *Quercus*, moist mixed forest, leg. Stüben,P., det. Stüben,P.

**Name**<br>**Authority**<br>**Additional Information**	**GenBank Acc No (Ref.)**<br>**Specimen ID**<br>**Sequence Length**	**ZFMK Tissue ID**<br>**ZFMK DNA Sample ID**<br>**(SDEI DNA Sample ID)**	**Locality, GPS, Collection Date, Plant, Collector, Identifier**
Echinodera aspromontensis<br>Stüben, 2008	MG229690 [new]<br>1365-PST<br>658nt	ZFMK-TIS-4661<br>ZFMK-DNA-0155628568	Italy, Abruzzo, Galascio nach Ofena, „Oasi Colonia Frasca“, N42°18'32" E13°43'59", 619m, 15-Aug-2013, *Fraxinus, Quercus*, sieving, leg. Stüben,P., det. Stüben,P.
Echinodera aspromontensis<br>Stüben, 2008	MG229692 [new]<br>1370-PST<br>658nt	ZFMK-TIS-4666<br>ZFMK-DNA-0155628552	Italy, Abruzzo, P.N. Majella, E of Sulmona near Pacentro, N42°03'13" E14°01'49", 983m, 16-Aug-2013, *Acer*, sieving, leg. Stüben,P., det. Stüben,P.
Echinodera aspromontensis<br>Stüben, 2008	MG229694 [new]<br>1375-PST<br>658nt	ZFMK-TIS-4671<br>ZFMK-DNA-0155628557	Italy, Abruzzo, E of Castel del Monte, Madonnina Capo di Serra, N42°21'38" E13°45'29", 1243m, 17-Aug-2013, sieving, leg. Stüben,P., det. Stüben,P.
Echinodera aspromontensis<br>Stüben, 2008	MG229697 [new]<br>1390-PST<br>658nt	ZFMK-TIS-4686<br>ZFMK-DNA-0155628543	Italy, Abruzzo, NE of Castel di Sangro, Sangro river, N41°48'56" E14°10'00", 767m, 24-Aug-2013, *mixed forest*, humid, sieving, leg. Stüben,P., det. Stüben,P.
Echinodera atlasensis<br>Stüben, 2001	GU213762 [01]<br>E-918-atl<br>658nt	ZFMK-TIS-cE918<br>ZFMK-DNA-0100405282	Morocco, N Agadir, Tifrit, N30°35'57" W09°29'50", 641m, 13-Mai-2009, *Olea, Smilax*, leg. Stüben,P., det. Stüben,P.
Echinodera atlasensis<br>Stüben, 2001	GU213763 [01]<br>E-919-atl<br>658nt	ZFMK-TIS-cE919<br>ZFMK-DNA-0100405281	Morocco, N Agadir, Tifrit - Immouzzer, N30°39'23" W09°30'09", 1162m, 14-Mai-2009, *Ceratonia, Quercus* , leg. Stüben,P., det. Stüben,P.
Echinodera atlasensis<br>Stüben, 2001	GU213771 [01]<br>Mo-947-ant<br>658nt	ZFMK-TIS-cMo947<br>ZFMK-DNA-0100404558	Morocco, Anti-Atlas, 46 km E of Tiznit, Anezi, "El Tnine", N29°44'51" W09°15'26", 1200m, 24-Dez-2002, moist river valley, leg. Stüben,P., det. Stüben,P.
Echinodera atlasensis<br>Stüben, 2001	MG322641 [new]<br>E-920-atl<br>658nt	ZFMK-TIS-cE920<br>ZFMK-DNA-0112704671	Morocco, N Agadir, Immouzzer, N30°40'12" W09°28'54", 1122m, 14-Mai-2009, *Ceratonia, Quercus* , leg. Stüben,P., det. Stüben,P.
Echinodera bargouensis<br>Stüben & Astrin, 2011<br>**Paratype (DNAtype)**	GU213690 [01]<br>T-499-zag<br>658nt	ZFMK-TIS-cT499<br>ZFMK-DNA-0100400672	Tunisia, Dorsale, Jebel Bargou, 42 km NE of Maktar, N36°02'10" E09°37'35", 610m, 26-Okt-2003, *Rhamnus, Pistacia, Quercus ilex* , stream valley, leg. Stüben,P., det. Stüben,P.
Echinodera bargouensis<br>Stüben & Astrin, 2011	GU213770 [01]<br>TU-946-bar<br>658nt	ZFMK-TIS-cTU946<br>ZFMK-DNA-0100405259	Tunisia, Jebel Serj, 25 km E of Makta, N35°55'29" E09°28'41", 670m, 26-Okt-2003, *Smilax aspera, Salix, Ficus, Ceratonia*, leg. Stüben,P., det. Stüben,P.
Echinodera behnei<br>Stüben, 1998	MG229760 [new]<br>1926-FBA<br>658nt	ZFMK-TIS-23317<br>ZFMK-DNA-0169170357	Greece, East Macedonia and Thrace, Drama, Potami N, N41°24'52" E24°06'45", 460m, 14-Aug-2014, leg. Bayer & Brunner, det. Bayer,F.
Echinodera behnei<br>Stüben, 1998	MG229761 [new]<br>1930-FBA<br>658nt	ZFMK-TIS-23321<br>ZFMK-DNA-0169170353	Greece, Central Macedonia, Drama, Volakas, quarry, N41°18'17" E24°00'41", 960m, 15-Aug-2014, leg. Bayer & Brunner, det. Bayer,F.
Echinodera behnei<br>Stüben, 1998	MK347564 [new]<br>2987-PST<br>658nt	(SDEI-DNA-2987-PST)	Greece, Macedonia, Lailias Mts., ski center (near Serres), N41°15'33" E23°35'12", 1518m, 13-Jul-2017, *Fagus*, leg. Stüben,P., det. Stüben,P.
Echinodera behnei<br>Stüben, 1998	MK347569 [new]<br>2993-PST<br>658nt	(SDEI-DNA-2993-PST)	Greece, Macedonia, Thessaloniki, Hortiatis Mt., 1 km S Hortiatis, N40°35'58" E23°06'13", 822m, 19-Jul-2017, *Corylus*, leg. Stüben,P., det. Stüben,P.
Echinodera bellieri<br>(Reiche, 1860)	GU213653 [01]<br>KO-0367-bel<br>658nt	ZFMK-TIS-cKO0367<br>ZFMK-DNA-0100400344	Greece, Korfu Isl., 20 km NW of Kerkyra, Vistonas, N39°41'55" E19°41'46", 404m, 23-Sep-2007, *Quercus ilex, Olea*, leg. Stüben,P., det. Stüben,P.

**Name**<br>**Authority**<br>**Additional Information**	**GenBank Acc No (Ref.)**<br>**Specimen ID**<br>**Sequence Length**	**ZFMK Tissue ID**<br>**ZFMK DNA Sample ID**<br>**(SDEI DNA Sample ID)**	**Locality, GPS, Collection Date, Plant, Collector, Identifier**
Echinodera bellieri<br>(Reiche, 1860)	GU988043 [01]<br>KO-0374-bel<br>658nt	ZFMK-TIS-cKO0374<br>ZFMK-DNA-0100405067	Greece, Korfu Isl., 15 km N of Kerkyra, Mt. Pantokratoras, Paelo Chorio, N39°44'43" E19°53'44", 419m, 25-Sep-2007, *Quercus ilex, Fagus*, leg. Stüben,P., det. Stüben,P.
Echinodera bellieri<br>(Reiche, 1860)	GU213657 [01]<br>EP-0398-bel<br>658nt	ZFMK-TIS-cEP0398<br>ZFMK-DNA-0100400057	Greece, Epirus, 19 km E of Igoumenitsa, Petrovitsa, N39°33'30" E20°28'12", 355m, 01-Okt-2007, *Arbutus/Platanus*, stream, leg. Stüben,P., det. Stüben,P.
Echinodera bellieri<br>(Reiche, 1860)	GU213679 [01]<br>EP-0390-bel<br>658nt	ZFMK-TIS-cEP0390<br>ZFMK-DNA-0100400414	Greece, Epirus, 9 km SW of Metsovo, Mikro Peristeri, N39°45'03" E21°05'09", 93m, 30-Sep-2007, *Quercus ilex*, leg. Stüben,P., det. Stüben,P.
Echinodera bellieri<br>(Reiche, 1860)	MG229743 [new]<br>1786-PST<br>658nt	ZFMK-TIS-24110<br>ZFMK-DNA-0171624077	Italy, Sardinia Isl., NW of Ussassai, above Cant. Arqueri, N39°48'60" E09°21'59", 976m, 11-Mai-2014, *Quercus*, sieving, leg. Stüben,P. & Schütte,A., det. Stüben,P.
Echinodera bellieri<br>(Reiche, 1860)	MG322659 [new]<br>IT1071c<br>658nt	ZFMK-TIS-cIT1071c<br>ZFMK-DNA-0112704598	Italy, Sardinia Isl. East, S of Dorgali, Genna Silana, N40°09'30" E09°30'30", 1017m, 27-Sep-2010, *Quercus ilex, Ficus*, cliff, leg. Stüben,P., det. Stüben,P.
Echinodera bellieri<br>(Reiche, 1860)	MG322660 [new]<br>IT1073c<br>658nt	ZFMK-TIS-cIT1073c<br>ZFMK-DNA-0112704596	Italy, Sardinia Isl. East, NE of Seui, M. Tonneri, N39°53'59" E9°23'24", 926m, 28-Sep-2010, *Quercus ilex*, cliff, leg. Stüben,P., det. Stüben,P.
Echinodera bellieri<br>(Reiche, 1860)	MG322662 [new]<br>IT1077c<br>658nt	ZFMK-TIS-cIT1077c<br>ZFMK-DNA-0112704592	Italy, Sardinia Isl. East, E of Seui, M. Arqueri, N39°49'04" E09°22'02", 925m, 29-Sep-2010, *Quercus ilex*, cliff, leg. Stüben,P., det. Stüben,P.
Echinodera bellieri<br>(Reiche, 1860)	MG322663 [new]<br>IT1079c<br>658nt	ZFMK-TIS-cIT1079c<br>ZFMK-DNA-0112704590	Italy, Sardinia Isl. East, E of Seui, Ulassai, N39°48'35" E09°29'48", 745m, 29-Sep-2010, *Quercus ilex*, cliff, leg. Stüben,P., det. Stüben,P.
Echinodera bellieri<br>(Reiche, 1860)	MG322666 [new]<br>IT1084c<br>658nt	ZFMK-TIS-cIT1084c<br>ZFMK-DNA-0112704578	Italy, Sardinia Isl. South, E of Cagliari, M. d. Sette Fratelli near Pta. Moitzus, N39°16'38" E09°28'35", 167m, 02-Okt-2010, *Quercus, Pistacia*, dried-out stream, leg. Stüben,P., det. Stüben,P.
Echinodera bellieri<br>(Reiche, 1860)	MG322669 [new]<br>IT1091c<br>658nt	ZFMK-TIS-cIT1091c<br>ZFMK-DNA-0112704585	Italy, Sardinia Isl. West, E of Macomer, above Lei, N40°19'54" E08°53'49", 1020m, 04-Okt-2010, *Quercus, Acer monspessulanum*, leg. Stüben,P., det. Stüben,P.
Echinodera bellieri<br>(Reiche, 1860)	MG322672 [new]<br>IT1094c<br>658nt	ZFMK-TIS-cIT1094c	Italy, Sardinia Isl. East, N of Dorgali, M. Tuttavista (Gipfel), N40°22'46" E09°38'22", 801m, 06-Okt-2010, *Quercus ilex*, leg. Stüben,P., det. Stüben,P.
Echinodera bellieri<br>(Reiche, 1860)	EU286505 [01]<br>I-0298-bel<br>658nt	ZFMK-TIS-cI0298<br>ZFMK-DNA-0100400024	Italy, Sicilia Isl. (PA), 19 km SE of Bagheria; Monti di Calamigna, NE of Ventimiglia di Sicilia, N37°55'33" E13°35'48", 581m, 11-Okt-2006, *Quercus ilex*, leg. Stüben,P., det. Stüben,P.
Echinodera bellieri<br>(Reiche, 1860)	GU213646 [01]<br>I-0173-bel<br>618nt	ZFMK-TIS-cI0173<br>ZFMK-DNA-0100400154	Italy, Sicilia Isl. (TP), 19 km NE of Trapani, Castelluzzo, N38°06'01" E12°42'25", 47m, 13-Okt-2006, *Quercus ilex*, leg. Stüben,P., det. Stüben,P.
Echinodera bellieri<br>(Reiche, 1860)	GU213711 [01]<br>I-629-tyr<br>658nt	ZFMK-TIS-cI629<br>ZFMK-DNA-0100404311	Italy, Campania, Cilento, 14 km SW of Vallo d. Lucania, Marina di Ascea, N40°07'42" E15°10'53", 13m, 30-Jun-2008, *Quercus ilex*, leg. Stüben,P., det. Stüben,P.
Echinodera bellieri<br>(Reiche, 1860)	GU213712 [01]<br>I-646-tyr<br>658nt	ZFMK-TIS-cI646<br>ZFMK-DNA-0100404897	Italy, Basilicata, Monte Pollino, 9 km NW of Rotonda, Castelluccio-Sup., N40°00'37" E15°57'59", 653m, 07-Jul-2008, *Quercus, Castanea*, city forest, leg. Stüben,P., det. Stüben,P.

**Name**<br>**Authority**<br>**Additional Information**	**GenBank Acc No (Ref.)**<br>**Specimen ID**<br>**Sequence Length**	**ZFMK Tissue ID**<br>**ZFMK DNA Sample ID**<br>**(SDEI DNA Sample ID)**	**Locality, GPS, Collection Date, Plant, Collector, Identifier**
Echinodera bellieri<br>(Reiche, 1860)	GU213713 [01]<br>I-651-tyr<br>658nt	ZFMK-TIS-cI651<br>ZFMK-DNA-0100404255	Italy, Campania, 4 km S of Cava de Tirreni (Salerno), Vietri sul Mare, N40°40'18" E14°43'14", 106m, 12-Jul-2008, *Quercus ilex*, stream valley, leg. Stüben,P., det. Stüben,P.
Echinodera bellieri<br>(Reiche, 1860)	GU987981 [01]<br>I-641-tyr<br>658nt	ZFMK-TIS-cI641<br>ZFMK-DNA-0100404902	Italy, Basilicata, Monte Pollino, 4 km SE of Rotonda, Zarafa, N39°56'33" E16°04'28", 677m, 07-Jul-2008, *Quercus ilex*, leg. Stüben,P., det. Stüben,P.
Echinodera brachati<br>Wolf, 2002	GU213764 [01]<br>E-922-bra<br>658nt	ZFMK-TIS-cE922<br>ZFMK-DNA-0100405279	Greece, Peloponnese, Mt. Taygetos W, Saidona E, N36°52'59" E22°17'25", 800m, 25-Apr-2009, *Quercus, Onosma, Cistus, Salvia fruticosa,* broom, leg. Bahr,Bayer,Brunner & Bueche, det. Stüben,P.
Echinodera brachati<br>Wolf, 2002	KJ867595 [new]<br>1254-FBA<br>658nt	ZFMK-TIS-3590<br>ZFMK-DNA-0100426027	Greece, Peloponnese, W of Taygetos Mts., E of Saidona, N36°52'59" E22°17'25", 800m, 00-Jan-1900, leg. Bayer,B., det. Bayer,B.
Echinodera brisouti<br>(Reitter, 1885)	FJ716541 [01]<br>KO-0370-bri<br>658nt	ZFMK-TIS-cKO0370<br>ZFMK-DNA-0100400345	Greece, Korfu Isl., 18 km N of Kerkyra, Loutses, Megali Grava, N39°46'38" E19°53'21", 479m, 23-Sep-2007, *Quercus pubescens, Laurus nobilis, Asphodelus*, cave floor, under stones, leg. Stüben,P., det. Stüben,P.
Echinodera brisouti<br>(Reitter, 1885)	GU213655 [01]<br>KO-0383-bri<br>658nt	ZFMK-TIS-cKO0383<br>ZFMK-DNA-0100400347	Greece, Korfu Isl., 8 km S of Kerkyra, Mt. Pantokratoras NW of Makrata, N39°32'52" E19°52'59", 482m, 27-Sep-2007, *Quercus, Arbutus*, leg. Stüben,P., det. Stüben,P.
Echinodera brisouti<br>(Reitter, 1885)	GU213656 [01]<br>EP-0391-bri2<br>658nt	ZFMK-TIS-cEP0391<br>ZFMK-DNA-0100400348	Greece, Epirus, 9 km SW of Metsovo, Mikro Peristeri, N39°45'03" E21°05'09", 693m, 30-Sep-2007, *Quercus ilex*, leg. Stüben,P., det. Stüben,P.
Echinodera brisouti<br>(Reitter, 1885)	GU987877 [01]<br>EP-0401-bri2<br>658nt	ZFMK-TIS-cEP0401<br>ZFMK-DNA-0100400415	Greece, Epirus, 17 km E of Igoumenitsa, Avaritsa, N39°32'31" E20°27'25", 96m, 01-Okt-2007, *Quercus ilex, Platanus*, river, leg. Stüben,P., det. Stüben,P.
Echinodera bulbosa<br>Stüben & Astrin, 2008	GU213649 [01]<br>E-0188-bul<br>658nt	ZFMK-TIS-cE0188<br>ZFMK-DNA-0100400009	Spain, Almeria, 11 km NW of Laujar de Andarax, Sierra Nevada, Bayarcal, N37°02'27" W03°00'12", 1291m, 07-Apr-2007, *Quercus ilex,* broom, *Euphorbia*, leg. Astrin,J. & Stüben,P., det. Stüben,P.
Echinodera bulbosa<br>Stüben & Astrin, 2008	GU213665 [01]<br>E-0191-bul<br>658nt	ZFMK-TIS-cE0191<br>ZFMK-DNA-0100400321	Spain, Almeria, 9 km W of Laujar de Andarax, Sierra Nevada, Bayarcal, N37°00'54" W02°59'07", 1262m, 07-Apr-2007, *Quercus ilex,* broom, *Euphorbia*, leg. Astrin,J. & Stüben,P., det. Stüben,P.
Echinodera bulbosa<br>Stüben & Astrin, 2008	GU987826 [01]<br>E-0193-bul<br>658nt	ZFMK-TIS-cE0193<br>ZFMK-DNA-0100400081	Spain, Granada, 13 km W of Laujar de Andarax, Sierra Nevada, Mairena nach Jubar, N37°00'29" W03°01'55", 1162m, 08-Apr-2007, *Quercus ilex, Euphorbia*, leg. Stüben,P., det. Stüben,P.
Echinodera bulbosa<br>Stüben & Astrin, 2008	KJ867599 [new]<br>1274-PST<br>658nt	ZFMK-TIS-4090<br>ZFMK-DNA-0100426191	Spain, Malaga, NW of Otivar, Sierra del Chapparal, N36°49'32" W03°42'30", 653m, 08-Mai-2013, *Quercus ilex*, sieving, leg. Stüben,P. & Schütte,A., det. Stüben,P.
Echinodera bulbosa<br>Stüben & Astrin, 2008	KJ867601 [new]<br>1278-PST<br>658nt	ZFMK-TIS-4094<br>ZFMK-DNA-0100426471	Spain, Malaga, NW of Otivar, Sierra del Chapparal, N36°52'21" W03°44'31", 1262m, 08-Mai-2013, *Quercus ilex*, sieving, leg. Stüben,P. & Schütte,A., det. Stüben,P.
Echinodera bulbosa<br>Stüben & Astrin, 2008	KJ867602 [new]<br>1282-PST<br>658nt	ZFMK-TIS-4098<br>ZFMK-DNA-0100426199	Spain, Malaga, S of Fornes, Sierra de Almijara (N. P.), N36°53'21" W03°51'14", 1002m, 08-Mai-2013, *Quercus, Viburnum*, similar to buckthorn, sieving, leg. Stüben,P. & Schütte,A., det. Stüben,P.
Echinodera capbonensis capbonensis<br>Stüben, 2004	GU213681 [01]<br>T-455-cab<br>658nt	ZFMK-TIS-cT455<br>ZFMK-DNA-0100400945	Tunisia, 6 km NW Bizerte, J. Nador, N37°19'04" E09°49'32", 60m, 30-Okt-2003, *Quercus ilex, Pistacia*, limestone, leg. Stüben,P., det. Stüben,P.

**Name**<br>**Authority**<br>**Additional Information**	**GenBank Acc No (Ref.)**<br>**Specimen ID**<br>**Sequence Length**	**ZFMK Tissue ID**<br>**ZFMK DNA Sample ID**<br>**(SDEI DNA Sample ID)**	**Locality, GPS, Collection Date, Plant, Collector, Identifier**
Echinodera capbonensis capbonensis<br>Stüben, 2004<br>**Paratype (DNAtype)**	GU213707 [01]<br>E-588-cab<br>658nt	ZFMK-TIS-cE588<br>ZFMK-DNA-0100404958	Tunisia, Cap Bon, 13 km NNE of Soliman, Korbous, N36°47'37" E10°33'52", 30m, 29-Okt-2003, *Quercus ilex, Smilax aspera*, stream valley, leg. Stüben,P., det. Stüben,P.
Echinodera capbonensis capbonensis<br>Stüben, 2004	GU987889 [01]<br>T-502-cap<br>658nt	ZFMK-TIS-cT502<br>ZFMK-DNA-0100400669	Tunisia, Cap Bon, 16 km NNE of Soliman, Korbous, N36°49'46" E10°34'15", 40m, 29-Okt-2003, *Garrigue*, leg. Stüben,P., det. Stüben,P.
Echinodera capbonensis capbonensis<br>Stüben, 2004	GU987890 [01]<br>T-503-cap<br>658nt	ZFMK-TIS-cT503<br>ZFMK-DNA-0100400668	Tunisia, 16 km E Maktar, beneath Haute Kesra, N35°47'51" E09°21'09", 1100m, 25-Okt-2003, *Ficus, Grenadine*, gardens, leg. Stüben,P., det. Stüben,P.
Echinodera capbonensis diottii<br>(Stüben, 2010)<br>formerly: E. diottii	GU213767 [01<br>I-942-dit<br>658nt	ZFMK-TIS-cI942<br>ZFMK-DNA-0100405269	Italy, Trapani, Is. Pantelleria, Montagna Grande, N37°53'00" E14°04'00", 1280m, 01-Apr-2009, leg. Monzini,S., det. Monzini,S.
Echinodera capiomonti<br>(H. Brisout de Barneville, 1864)	GU213682 [01]<br>I-488-cap<br>658nt	ZFMK-TIS-cI488<br>ZFMK-DNA-0100400974	Italy, Emilia-Romagna, San Giovanni in Marignano, 10 km W of Pesaro (RN), N43°56'06" E12°43'00", 100m, 15-Okt-2001, *Quercus*, leg. Stüben,P., det. Stüben,P.
Echinodera casablancaensis<br>Stüben, 2001	GU213759 [01]<br>E-913-cas<br>658nt	ZFMK-TIS-cE913<br>ZFMK-DNA-0100405286	Morocco, Casablanca South-West, Dar Bouazza, N33°32'05" W07°47'17", 9m, 11-Mai-2009, *Acacia*, leg. Stüben,P., det. Stüben,P.
Echinodera casablancaensis<br>Stüben, 2001	GU213760 [01]<br>E-916-cas<br>658nt	ZFMK-TIS-cE916<br>ZFMK-DNA-0100404528	Morocco, 2 km S of Essaouira, N31°29'06" W09°45'41", 22m, 13-Mai-2009, *Acacia*, leg. Stüben,P., det. Stüben,P.
Echinodera casablancaensis<br>Stüben, 2001	GU213761 [01]<br>E-917-cas<br>658nt	ZFMK-TIS-cE917<br>ZFMK-DNA-0100405283	Morocco, S of Tamri, N30°40'39" W09°51'55", 196m, 13-Mai-2009, leg. Stüben,P., det. Stüben,P.
Echinodera casablancaensis<br>Stüben, 2001	GU988148 [01]<br>E-915-cas<br>658nt	ZFMK-TIS-cE915<br>ZFMK-DNA-0100405285	Morocco, 2 km S of Essaouira, N31°28'08" W09°45'27", 22m, 12-Mai-2009, *Pistacia*, leg. Stüben,P., det. Stüben,P.
Echinodera ceutaensis<br>Stüben, 2002	GU213756 [01]<br>E-900-ceu<br>658nt	ZFMK-TIS-cE900<br>ZFMK-DNA-0100405491	Morocco, Sebta West, vir. Biutz, N35°53'04" W05°24'08", 337m, 09-Mai-2009, *Quercus suber, Smilax, Arbutus*, leg. Stüben,P., det. Stüben,P.
Echinodera ceutaensis<br>Stüben, 2002	GU988139 [01]<br>E-902-ceu<br>658nt	ZFMK-TIS-cE902<br>ZFMK-DNA-0100405480	Morocco, Sebta West, vir. Punta Leona, N35°54'22" W05°28'55", 29m, 09-Mai-2009, *Pistacia*, leg. Stüben,P., det. Stüben,P.
Echinodera ceutaensis<br>Stüben, 2002	GU988143 [01]<br>E-906-ceu<br>658nt	ZFMK-TIS-cE906<br>ZFMK-DNA-0100405484	Morocco, 12 km NE of Tanger, N35°49'08" W05°44'05", 18m, 09-Mai-2009, *Pistacia, Quercus*, leg. Stüben,P., det. Stüben,P.
Echinodera ceutaensis<br>Stüben, 2002	GU988145 [01]<br>E-908-ceu<br>658nt	ZFMK-TIS-cE908<br>ZFMK-DNA-0100405486	Morocco, S of Ksar-es-Seghir, N35°45'16" W05°30'49", 278m, 09-Mai-2009, *Pistacia, Quercus suber*, leg. Stüben,P., det. Stüben,P.
Echinodera ceutaensis<br>Stüben, 2002	GU213749 [01]<br>E-861-lus<br>631nt	ZFMK-TIS-cE861<br>ZFMK-DNA-0100405536	Portugal, Sa. Arrabida, SW of Setubal, N38°30'40" W08°55'21", 51m, 27-Apr-2009, *Quercus ilex, Pistacia lentiscus, Phillyrea, Smilax*, leg. Astrin,J., det. Stüben,P.

**Name**<br>**Authority**<br>**Additional Information**	**GenBank Acc No (Ref.)**<br>**Specimen ID**<br>**Sequence Length**	**ZFMK Tissue ID**<br>**ZFMK DNA Sample ID**<br>**(SDEI DNA Sample ID)**	**Locality, GPS, Collection Date, Plant, Collector, Identifier**
Echinodera ceutaensis<br>Stüben, 2002	KJ867586 [new]<br>854-PST<br>658nt	ZFMK-TIS-3767<br>ZFMK-DNA-0100414237	Portugal, Alentejo, Serra da Arràbida, Outao, N38°30'05" W08°56'20", 70m, 20-Mai-2012, *Quercus*, sieving, leg. Stüben,P., det. Stüben,P.
Echinodera cf. *berkanensis*	KC783817 [new]<br>192-PST<br>609nt	ZFMK-TIS-2D100440238<br>ZFMK-DNA-0100438126	Morocco, W of Al-Hoceima, Rif Mts., Massif des Bokkoyas, Kalah-Iris, N35°08'50" W04°22'33", 13m, 24-Mai-2011, *Pistacia*, sieving, leg. Stüben,P., det. Stüben,P.
Echinodera cf. *capiomonti*	MG229702 [new]<br>1403-PST<br>658nt	ZFMK-TIS-4699<br>ZFMK-DNA-0155628537	Italy, Lazio, near Arpino, Mura Ciclopiche, N41°38'53" E13°38'15", 739m, 30-Aug-2013, mixed forest, sieving, leg. Stüben,P., det. Stüben,P.
Echinodera cf. *lusitanica*	GU213745 [01]<br>E-846-pag<br>658nt	ZFMK-TIS-cE846<br>ZFMK-DNA-0100405544	Spain, Avila, NW of Arenas de San Pedro, Gredos NP, "la plataforma", N40°16'06" W05°14'22", 1880m, 24-Apr-2009, *Cytisus*, leg. Astrin,J., det. Stüben,P.
Echinodera cf. *lusitanica*	GU213746 [01]<br>E-850-pag<br>658nt	ZFMK-TIS-cE850<br>ZFMK-DNA-0100404560	Spain, Salamanca, NW of Bejar, NW of Monforte de la Sierra, Pena de Francia near cloister, N40°31'01" W06°10'15", 1636m, 24-Apr-2009, *Cytisus*, leg. Astrin,J., det. Stüben,P.
Echinodera cf. *lusitanica*	GU988098 [01]<br>E-838-pag<br>658nt	ZFMK-TIS-cE838<br>ZFMK-DNA-0100405561	Spain, Avila, SW of Ávila near Mengamunoz, Pto. de Menga, N40°28'40" W05°00'40", 1564m, 23-Apr-2009, *Cytisus*, leg. Astrin,J., det. Stüben,P.
Echinodera cf. *lusitanica*	GU988100 [01]<br>E-840-pag<br>626nt	ZFMK-TIS-cE840<br>ZFMK-DNA-0100405550	Spain, Avila, SW of Ávila, Navacepedilla de Corneja, N40°28'59" W05°11'08", 1267m, 23-Apr-2009, *Cytisus, Crategus, Rubus, Quercus*, leg. Astrin,J., det. Stüben,P.
Echinodera cf. *lusitanica*	GU988102 [01]<br>E-842-pag<br>630nt	ZFMK-TIS-cE842<br>ZFMK-DNA-0100404520	Spain, Avila, SW of Ávila, N of San Martin de la Vega, Pto. de Chia, N40°27'16" W05°10'16", 1718m, 23-Apr-2009, *Cytisus*, leg. Astrin,J., det. Stüben,P.
Echinodera cf. *lusitanica*	GU988103 [01]<br>E-843-pag<br>658nt	ZFMK-TIS-cE843<br>ZFMK-DNA-0100405547	Spain, Avila, SW of Ávila, SE of Piedrahita, Pto. de la Pena Negra, N40°25'19" W05°18'01", 1910m, 23-Apr-2009, *Cytisus*, leg. Astrin,J., det. Stüben,P.
Echinodera cognita<br>Stüben, 2006<br>**Paratype (DNAtype)**	GU213662 [01]<br>E-0417-cog<br>658nt	ZFMK-TIS-cE0417<br>ZFMK-DNA-0100400008	Spain, Andalucia, S. Magina, 6 km S of Torres, N37°43'54" W03°29'45", 1641m, 16-Apr-2003, *Quercus ilex*, leg. Stüben,P., det. Stüben,P.
Echinodera cognita<br>Stüben, 2006<br>**Paratype (DNAtype)**	GU213684 [01]<br>E-490-cog<br>658nt	ZFMK-TIS-cE490<br>ZFMK-DNA-0100400976	Spain, Andalucia, S. Magina, 6 km S of Torres, N37°43'54" W03°29'45", 1641m, 16-Apr-2003, *Quercus ilex*, leg. Stüben,P., det. Stüben,P.
Echinodera compacta<br>Wollaston, 1864	JN701881 [40]<br>8-PST<br>651nt	ZFMK-TIS-2D100447039<br>ZFMK-DNA-0100437981	Spain, Canary Islands, Gran Canaria, Marzagan, Caldera de Bandama, N28°02'07" W15°27'36", 401m, 23-Dez-2010, sieving, leg. Stüben,P., det. Stüben,P.
Echinodera compacta<br>Wollaston, 1864	MG229684 [40]<br>33-PST<br>658nt	ZFMK-TIS-2D100447014<br>ZFMK-DNA-0155630450	Spain, Canary Islands, Gran Canaria, NW of San Bartolome, Barranco de Tirajana, N27°56'22" W15°35'29", 1162m, 27-Dez-2010, *Ficus carica* , sieving, leg. Stüben,P., det. Stüben,P.
Echinodera corcyrensis<br>Stüben, 2008	GU213650 [01]<br>HR-0307-cor<br>658nt	ZFMK-TIS-cHR0307<br>ZFMK-DNA-0100400080	Croatia, Dalmatia, 24 km W of Split, 1 km E of Marina, Poljica, N43°31'11" E16°08'31", 20m, 01-Jul-2007, *Quercus*, leg. Stüben,P., det. Stüben,P.
Echinodera corcyrensis<br>Stüben, 2008	GU213654 [01]<br>KO-0373-cor<br>658nt	ZFMK-TIS-cKO0373<br>ZFMK-DNA-0100400346	Greece, Korfu Isl., 9 km SW of Kerkyra, Kaisers'Thrones, N39°35'39" E19°49'17", 258m, 24-Sep-2007, *Quercus ilex*, leg. Stüben,P., det. Stüben,P.

**Name**<br>**Authority**<br>**Additional Information**	**GenBank Acc No (Ref.)**<br>**Specimen ID**<br>**Sequence Length**	**ZFMK Tissue ID**<br>**ZFMK DNA Sample ID**<br>**(SDEI DNA Sample ID)**	**Locality, GPS, Collection Date, Plant, Collector, Identifier**
Echinodera corcyrensis<br>Stüben, 2008	GU987821 [01]<br>KO-0366-cor<br>658nt	ZFMK-TIS-cKO0366<br>ZFMK-DNA-0100400343	Greece, Korfu Isl., 20 kmNW of Kerkyra, Makrades, N39°41'10" E19°41'31", 281m, 23-Sep-2007, *Quercus ilex, Olea*, leg. Stüben,P., det. Stüben,P.
Echinodera corcyrensis<br>Stüben, 2008	KJ867587 [new]<br>860-PST<br>658nt	ZFMK-TIS-3773<br>ZFMK-DNA-0100414231	Greece, Zakynthos Isl., near Katastari, N37°50'57" E20°44'31", 118m, 05-Okt-2012, *Quercus forest*, sieving, leg. Stüben,P., det. Stüben,P.
Echinodera corcyrensis<br>Stüben, 2008	KJ867588 [new]<br>862-PST<br>658nt	ZFMK-TIS-3775<br>ZFMK-DNA-0100414229	Greece, Zakynthos Isl., Skopos Mt., S of Argasi, Kokiareika, N37°44'45" E20°56'03", 372m, 11-Okt-2012, *Quercus forest*, sieving, leg. Stüben,P., det. Stüben,P.
Echinodera corcyrensis<br>Stüben, 2008	GU213658 [01]<br>EP-0409-cor<br>658nt	ZFMK-TIS-cEP0409<br>ZFMK-DNA-0100400350	Greece, Epirus, 19 km E of Igoumenitsa, Petrovitsa, N39°33'30" E20°28'12", 355m, 01-Okt-2007, *Quercus ilex*, stream, leg. Stüben,P., det. Stüben,P.
Echinodera crenata<br>Wollaston, 1863	FJ716573 [01]<br>E-712-cre<br>658nt	ZFMK-TIS-cE712<br>ZFMK-DNA-0100404238	Spain, Canary Islands, Tenerife, S of Orotava, Teide, Montana Mostaza, N28°17'01" W16°34'18", 2172m, 04-Okt-2008, *Spartocytisus supranubius*, leg. Astrin,J. & Stüben,P., det. Stüben,P.
Echinodera crenata<br>Wollaston, 1863	GU213702 [01]<br>E-565-cre<br>658nt	ZFMK-TIS-cE565<br>ZFMK-DNA-0100404983	Spain, Canary Islands, Tenerife, 7 km S of Teide, Zapato de la Reina, N28°12'48" W16°39'44", 2035m, 23-Dez-2003, *Spartocytisus supranubius*, leg. Stüben,P., det. Stüben,P.
Echinodera crenata<br>Wollaston, 1863	GU213719 [01]<br>E-710-cre<br>658nt	ZFMK-TIS-cE710<br>ZFMK-DNA-0100404845	Spain, Canary Islands, Tenerife, S of Orotava, Orotava valley, Mirador de la Rosa, N28°20'25" W16°31'29", 1503m, 04-Okt-2008, *Greenovia* sp.*, Aeonium spathulatum*, leg. Astrin,J. & Stüben,P., det. Stüben,P.
Echinodera cyprica<br>Stüben, 2010	MG322646 [new]<br>CY1041<br>658nt	ZFMK-TIS-cCY1041<br>ZFMK-DNA-0112704631	Cyprus, N of Pafos, Drouseia, 34°57'51"N 32°23'53.5"E, 603m, 19-Apr-2010, *sifting under Quercus infectoria veneris*, leg. Stüben,P., det. Stüben,P.
Echinodera cyprica<br>Stüben, 2010	MG322647 [new]<br>CY1042<br>658nt	ZFMK-TIS-cCY1042<br>ZFMK-DNA-0112704632	Cyprus, Troodos Mts., Cedar Valley, 34°59'28"N 32°41'18"E, 1097m, 20-Apr-2010, *Quercus alnifolia*, Quercus alnifolia-forest, leg. Stüben,P., det. Stüben,P.
Echinodera cyprica<br>Stüben, 2010	MG322655 [new]<br>CY1065<br>658nt	ZFMK-TIS-cCY1065<br>ZFMK-DNA-0112704607	Cyprus, Troodos Mts., Stavros tis Psokas, N35°01'33" E32°37'43", 546m, 20-Apr-2010, *Quercus alnifolia, Apiaceae, Acer, Cistus (forest track)*, leg. Stüben,P., det. Stüben,P.
Echinodera germanni<br>Stüben, 2003	GU213751 [01]<br>E-881-ger<br>658nt	ZFMK-TIS-cE881<br>ZFMK-DNA-0100405507	Morocco, High Atlas, N of Tizi-n-Test, Tachguette, N30°52'25" W08°21'25", 2047m, 30-Apr-2009, *Euphorbia nicaeensis, Quercus ilex,* broom, leg. Stüben,P., det. Stüben,P.
Echinodera graeca<br>Caldara, 1973	GU213677 [01<br>EP-0384-gra<br>658nt	ZFMK-TIS-cEP0384<br>ZFMK-DNA-0100400461	Greece, Epirus, N of Metsovo, Katara Pass, N39°46'59" E21°09'09", 1419m, 29-Sep-2007, *Fagus*, leg. Stüben,P., det. Stüben,P.
Echinodera guacimara<br>Stüben & Germann, 2005	FJ716566 [01<br>E-688-gua<br>658nt	ZFMK-TIS-cE688<br>ZFMK-DNA-0100404867	Spain, Canary Islands, Tenerife, NE of La Laguna, Anaga Mts., Las Bodegas, N28°33'42" W16°09'25", 502m, 30-Sep-2008, dunghill, leg. Astrin,J. & Stüben,P., det. Stüben,P.
Echinodera guacimara<br>Stüben & Germann, 2005<br>**Paratype (DNAtype)**	GU213701 [01<br>E-561-gua<br>658nt	ZFMK-TIS-cE561<br>ZFMK-DNA-0100404979	Spain, Canary Islands, Tenerife, East Anaga Mts., E of La Laguna, E of Lomo de las Bodegas, N28°33'38" W16°09'20", 500m, 26-Dez-2003, cliff, compost, leg. Stüben,P., det. Stüben,P.
Echinodera hoceimaensis<br>Stüben, 2018<br>**Paratype (DNAtype)**	KC783818 [34]<br>194-PST_54MO2011<br>658nt	ZFMK-TIS-2D100438675<br>ZFMK-DNA-0100438351	Morocco, SE of Al-Hoceima, Rif Mts., E of Imzouren, N35°07'52" W03°42'08", 598m, 25-Mai-2011, *Quercus suber, Quercus ilex*, sieving, leg. Stüben,P., det. Stüben,P.

**Name**<br>**Authority**<br>**Additional Information**	**GenBank Acc No (Ref.)**<br>**Specimen ID**<br>**Sequence Length**	**ZFMK Tissue ID**<br>**ZFMK DNA Sample ID**<br>**(SDEI DNA Sample ID)**	**Locality, GPS, Collection Date, Plant, Collector, Identifier**
Echinodera hypocrita<br>(Boheman, 1837)	EU286520 [01]<br>HR-0335-hyp<br>658nt	ZFMK-TIS-cHR0335<br>ZFMK-DNA-0100400242	Croatia, Dalmatia, 6 km E of Karlobag, Velebit Mts., Ostarijska vrata (Pass), N44°31'45" E15°08'34", 927m, 14-Jul-2007, *Fagus*, limestone, leg. Stüben,P., det. Stüben,P.
Echinodera hypocrita<br>(Boheman, 1837)	GU213676 [01]<br>HR-0337-hyp<br>658nt	ZFMK-TIS-cHR0337<br>ZFMK-DNA-0100400438	Croatia, Dalmatia, 8 km E of Karlobag, Velebit Mts., Stupacinovo, N44°32'41" E15°09'58", 1049m, 14-Jul-2007, *Fagus*, limestone, leg. Stüben,P., det. Stüben,P.
Echinodera hypocrita<br>(Boheman, 1837)	GU213672 [01]<br>CZ-0302-hyp<br>658nt	ZFMK-TIS-cCZ0302<br>ZFMK-DNA-0100400464	Czech Republic, Moravia mer., Pavlov env, Palava Protected Landscape Area, N48°52' E16°40', 28-Mai-2006, *Tilia, Carpinus, Quercus*, leg. Stejskal,R., det. Stejskal,R.
Echinodera hypocrita<br>(Boheman, 1837)	GU213741 [01]<br>Cz-800-hyp<br>658nt	ZFMK-TIS-cCz800<br>ZFMK-DNA-0100405003	Czech Republic, Bohemia W (KT), Balkovy, Doubrava Hill (6545), N49°26' E13°13', 17-Aug-2008, leg. Kresl,P., det. Kresl,P.
Echinodera hypocrita<br>(Boheman, 1837)	MG322651 [new]<br>CZ1053<br>658nt	ZFMK-TIS-cCZ1053<br>ZFMK-DNA-0112704616	Czech Republic, SE Moravia (UH), Lopenik, Bile Karpaty Mts., 48°56'00"N 17°46'00"E, 727m, 15-Mai-2009, *Fagus*, leg. Kresl,P., det. Kresl,P.
Echinodera hypocrita<br>(Boheman, 1837)	MK892135 [new]<br>2418-JKR<br>658nt	ZFMK-TIS-25995<br>ZFMK-DNA-0171600640	Czech Republic, Bohemia, Horni Morava, N50°10'15" E16°49'22", 780m, 25-Aug-2014, *Fagus sylvatica*, collecting by hand, leg. Krátky,J., det. Krátky,J.
Echinodera hypocrita<br>(Boheman, 1837)	GU213647 [01]<br>I-0014-hyp<br>658nt	ZFMK-TIS-cI0014<br>ZFMK-DNA-0100400050	France, Isere, 12 km SW of Bourgoin, NE of St. Jean de Bournay, N45°31'02" E05°10'23", 395m, 10-Aug-2005, *Quercus, Castanea*, leg. Stüben,P., det. Stüben,P.
Echinodera hypocrita<br>(Boheman, 1837)	GU213765 [01]<br>F-437-hyp<br>658nt	ZFMK-TIS-cF437<br>ZFMK-DNA-0100405265	France, Alpes-Maritimes, 9 km N of Sospel, N of Moulinet, N43°57'58" E07°24'55", 1111m, 24-Dez-2007, *Castanea sativa*, leg. Stüben,P., det. Stüben,P.
Echinodera hypocrita<br>(Boheman, 1837)	GU213768 [01]<br>F-939-hyp<br>658nt	ZFMK-TIS-cF939<br>ZFMK-DNA-0100405270	France, Dep. Indre-et-Loire, E of Tours, Vouvray (near Loire river), N47°24'35" E00°46'03", 62m, 28-Jul-2009, *Quercus*, mixed forest, leg. Stüben,P., det. Stüben,P.
Echinodera hypocrita<br>(Boheman, 1837)	GU987763 [01]<br>I-0022-hyp<br>658nt	ZFMK-TIS-cI0022<br>ZFMK-DNA-0100400950	France, Isere, 9 km N of Grenoble, Mont St. Martin, N45°16'15" E05°40'13", 719m, 12-Aug-2005, *Quercus*, leg. Stüben,P., det. Stüben,P.
Echinodera hypocrita<br>(Boheman, 1837)	GU987778 [01]<br>I-0004-hyp<br>658nt	ZFMK-TIS-cI0004<br>ZFMK-DNA-0100400894	France, Isere, 22 km S of Bourgoin, La Cote St. Andre, N45°24'04" E05°14'04", 469m, 08-Aug-2005, *Castanea*, leg. Stüben,P., det. Stüben,P.
Echinodera hypocrita<br>(Boheman, 1837)	EU286452 [01]<br>M-0047-hyp<br>658nt	ZFMK-TIS-cM0047<br>ZFMK-DNA-0100400744	Germany, Rhineland-Palatinate (RLP), Moselle valley, 5 km E of Cochem, N50°08'46" E07°12'39", 279m, 15-Sep-2005, *Quercus, Carpinus*, leg. Stüben,P., det. Stüben,P.
Echinodera hypocrita<br>(Boheman, 1837)	GU213648 [01]<br>M-0064-hyp<br>658nt	ZFMK-TIS-cM0064<br>ZFMK-DNA-0100400059	Germany, Rhineland-Palatinate (RLP), Bausenberg near Niederzissen, N50°27'52" E07°13'29", 291m, 17-Sep-2005, *Quercus, Carpinus, Fagus*, leg. Stüben,P., det. Stüben,P.
Echinodera hypocrita<br>(Boheman, 1837)	GU213673 [01]<br>D-0304-hyp<br>658nt	ZFMK-TIS-cD0304<br>ZFMK-TIS-cD0304	Germany, Baden-Wuerttemberg (BW), Kaiserstuhl, "Auf dem Eck", N48°06'32" E07°40'04", 432m, 27-Mai-2006, *Fagus, Carpinus, Quercus*, leg. Stüben,P., det. Stüben,P.
Echinodera hypocrita<br>(Boheman, 1837)	GU987766 [01]<br>M-0067-hyp<br>658nt	ZFMK-TIS-cM0067<br>ZFMK-DNA-0100400924	Germany, Rhineland-Palatinate (RLP), Ahrtal, Mayschoß, N50°31'03" E07°01'03", 200m, 17-Sep-2005, *Quercus, Carpinus*, leg. Stüben,P., det. Stüben,P.

**Name**<br>**Authority**<br>**Additional Information**	**GenBank Acc No (Ref.)**<br>**Specimen ID**<br>**Sequence Length**	**ZFMK Tissue ID**<br>**ZFMK DNA Sample ID**<br>**(SDEI DNA Sample ID)**	**Locality, GPS, Collection Date, Plant, Collector, Identifier**
Echinodera hypocrita<br>(Boheman, 1837)	GU213678 [01]<br>EP-0386-hyp<br>658nt	ZFMK-TIS-cEP0386<br>ZFMK-DNA-0100401026	Greece, Epirus, 10 km N of Metsovo, N39°50'02" E21°11'28", 1465m, 29-Sep-2007, *Fagus*, leg. Stüben,P., det. Stüben,P.
Echinodera hypocrita<br>(Boheman, 1837)	MG229759 [new]<br>1925-FBA<br>658nt	ZFMK-TIS-23316<br>ZFMK-DNA-0169170358	Greece, East Macedonia and Thrace, Drama, Potami N, N41°24'52" E24°06'45", 460m, 14-Aug-2014, leg. Bayer & Brunner, det. Bayer,F.
Echinodera hypocrita<br>(Boheman, 1837)	MK347565 [new]<br>2989-PST<br>658nt	(SDEI-DNA-2989-PST)	Greece, Macedonia, Lailias Mts., ski center (near Serres), N41°15'33" E23°35'12", 1518m, 13-Jul-2017, *Fagus*, leg. Stüben,P., det. Stüben,P.
Echinodera hypocrita<br>(Boheman, 1837)	GU213710 [01]<br>I-627-hyp<br>658nt	ZFMK-TIS-cI627<br>ZFMK-DNA-0100404916	Italy, Campania, Cilento, 6 km SE of Vallo d. Lucania, M. Sacro o Gelbison, N40°12'41" E15°19'42", 1544m, 30-Jun-2008, *Fagus*, leg. Stüben,P., det. Stüben,P.
Echinodera hypocrita<br>(Boheman, 1837)	GU987964 [01]<br>I-619-hyp<br>658nt	ZFMK-TIS-cI619<br>ZFMK-DNA-0100404924	Italy, Campania, Cilento, 7 km SW of Vallo d. Lucania, Petrosa, N40°11'30" E15°12'14", 110m, 30-Jun-2008, *Quercus, Pistacia lentiscus* , leg. Stüben,P., det. Stüben,P.
Echinodera hypocrita<br>(Boheman, 1837)	MG229693 [new]<br>1371-PST<br>658nt	ZFMK-TIS-4667<br>ZFMK-DNA-0155628553	Italy, Abruzzo, NE of Capestrano near Ofena, N42°16'53" E13°49'20", 940m, 17-Aug-2013, sieving, leg. Stüben,P., det. Stüben,P.
Echinodera hypocrita<br>(Boheman, 1837)	MK892158 [new]<br>2447-JKR<br>658nt	ZFMK-TIS-26024<br>ZFMK-DNA-0171600669	Slovakia, Bardejov, Gerlachov, N49°18'55" E21°05'57", 420m, 21-Sep-2014, *Fagetum*, sieving, leg. Pavel,F., det. Krátky,J.
Echinodera hypocrita<br>(Boheman, 1837)	GU987788 [01]<br>SLO-0172-hyp<br>658nt	ZFMK-TIS-cSLO0172<br>ZFMK-DNA-0100400680	Slovenia, Apasko Polje, Podgorje env., 30 km NE of Maribor, N46°42' E15°55', 214m, 05-Aug-2006, *Quercus, Carpinus*, leg. Stejskal,R., det. Stejskal,R.
Echinodera hystrix<br>*benahoarita*<br>Stüben, 2000<br>formerly: *E. benahoarita*	MG229725 [40]<br>1705-PST<br>528nt	ZFMK-TIS-24423<br>ZFMK-DNA-0155622486	Spain, Canary Islands, La Palma, NW of M. de Tagoja near F. de Vizcaino, N28°44'54" W17°49'48", 2069m, 24-Dez-2013, *Adenocarpus viscosus var. spartioides*, sieving, leg. Stüben,P., det. Stüben,P.
Echinodera hystrix<br>Wollaston, 1864<br>**Lectotype (DNAtype)**	EU286477 [01]<br>C-0142-hys<br>658nt	ZFMK-TIS-cC0142<br>ZFMK-DNA-0100400179	Spain, Canary Islands, El Hierro, 8 km W of La Frontera, Pista Derrabado, N27°44'10" W18°04'26", 796m, 22-Dez-2006, *Laurisilva*, leg. Stüben,P., det. Stüben,P.
Echinodera hystrix<br>Wollaston, 1864	GU213638 [01]<br>C-0143-hys<br>658nt	ZFMK-TIS-cC0143<br>ZFMK-DNA-0100400180	Spain, Canary Islands, El Hierro, 1 km SW of Guarazoca, Camino de la Pena, N27°48'06" W17°58'47", 746m, 02-Jan-2007, *Laurisilva*, leg. Stüben,P., det. Stüben,P.
Echinodera hystrix<br>Wollaston, 1864	GU213669 [01]<br>E-0277-hys<br>658nt	ZFMK-TIS-cE0277<br>ZFMK-DNA-0100401037	Spain, Canary Islands, El Hierro, 2.5 km E of Frontera, Mt. Timbarombo, N27°45'04" W17°59'02", 1311m, 28-Dez-2006, *Pericallis murrayi*, leg. Stüben,P., det. Stüben,P.
Echinodera hystrix<br>Wollaston, 1864	GU213670 [01]<br>E-0281-hys<br>658nt	ZFMK-TIS-cE0281<br>ZFMK-DNA-0100400417	Spain, Canary Islands, El Hierro, 3 km N of San Andres, Montana de la Fara, N27°47'39" W17°56'55", 936m, 25-Dez-2006, *Foeniculum vulgare*, leg. Stüben,P., det. Stüben,P.
Echinodera hystrix<br>Wollaston, 1864	MG229832 [new]<br>E-0279-hys<br>658nt	ZFMK-TIS-24590	Spain, Canary Islands, El Hierro, 4 km SW of La Frontera, El Parque, 27°44'20"N 18°01'30"W, 739m, 24-Dez-2006, *detritus with leaves*, leg. Stüben,P., det. Stüben,P.

**Name**<br>**Authority**<br>**Additional Information**	**GenBank Acc No (Ref.)**<br>**Specimen ID**<br>**Sequence Length**	**ZFMK Tissue ID**<br>**ZFMK DNA Sample ID**<br>**(SDEI DNA Sample ID)**	**Locality, GPS, Collection Date, Plant, Collector, Identifier**
Echinodera hystrix<br>Wollaston, 1864	MG229833 [new]<br>E-0282-hys<br>658nt	ZFMK-TIS-24592	Spain, Canary Islands, El Hierro, 9.5 km W of La Frontera, Pista de Mencafite, 27°44'06"N 18°05'08"W, 929m, 22-Dez-2006, *Tagasaste, Pericallis, Ficus (geklopft)*, leg. Stüben,P., det. Stüben,P.
Echinodera hystrix<br>Wollaston, 1864	MK347551 [40]<br>2964-PST<br>658nt	(SDEI-DNA-2964-PST)	Spain, Canary Islands, El Hierro, El Golfo, 1 km S. Tigaday (Los Granales), N27°44'37" W18°00'50", 601m, 13-Apr-2016, *Hypericum, Kleinia, Sonchus*, leg. Stüben & Schütte, det. Stüben,P.
Echinodera hystrix<br>Wollaston, 1864	GU213639 [01]<br>C-0144-hys<br>658nt	ZFMK-TIS-cC0144<br>ZFMK-DNA-0100400099	Spain, Canary Islands, La Palma, 11 km NE of El Paso, Mt. Tagoja, N28°43'18" W17°47'07", 1047m, 01-Jul-2006, *Laurisilva, Chamaecytisus proliferus*, leg. Stüben,P., det. Stüben,P.
Echinodera hystrix<br>Wollaston, 1864	GU213640 [01]<br>C-0145-hys<br>658nt	ZFMK-TIS-cC0145<br>ZFMK-DNA-0100400093	Spain, Canary Islands, La Palma, Cumbre Nueva, 4.5 km SE of El Paso, El Pilar, N28°37'37" W17°49'45", 1432m, 07-Jul-2006, *Laurisilva*, leg. Stüben,P., det. Stüben,P.
Echinodera hystrix<br>Wollaston, 1864	GU213643 [01]<br>C-0148-hys<br>658nt	ZFMK-TIS-cC0148<br>ZFMK-DNA-0100400066	Spain, Canary Islands, La Palma, 6.5 km SE of El Paso, San Isidro, N28°38'39" W17°48'02", 919m, 08-Jul-2006, *Ficus, Laurus*, leg. Stüben,P., det. Stüben,P.
Echinodera hystrix<br>Wollaston, 1864	MG229712 [40]<br>1569-JKR<br>658nt	ZFMK-TIS-3617<br>ZFMK-DNA-0155635663	Spain, Canary Islands, La Palma, Garafia, Barranco de Aguas, N28°48'35" W17°54'27", 931m, 31-Jan-2013, *Laurisilva*, sieving, leg. Krátky,J., det. Krátky,J.
Echinodera ibleiensis<br>Stüben, 2003<br>**Paratype (DNAtype)**	GU213687 [01]<br>I-496-ibl<br>658nt	ZFMK-TIS-cI496<br>ZFMK-DNA-0100400675	Italy, Sicilia Isl. (SR), 1.5 km S of Ferla, Valle dell'Anapo, Monti Iblei, N37°06'14" E14°56'07", 450m, 24-Okt-2002, *Castanea, Quercus, Ceratonia siliqua*, mixed forest, leg. Stüben,P., det. Stüben,P.
Echinodera ifranensis<br>Stüben, 2002	GU213752 [01]<br>E-892-ifr<br>658nt	ZFMK-TIS-cE892<br>ZFMK-DNA-0100405499	Morocco, Middle Atlas, S of Azrou, S of Äin Leuh, N33°12'50" W05°20'37", 1729m, 05-Mai-2009, *Quercus ilex*, leg. Stüben,P., det. Stüben,P.
Echinodera ifranensis<br>Stüben, 2002	KJ867570 [new]<br>191-PST<br>658nt	ZFMK-TIS-2D100440237<br>ZFMK-DNA-0100438125	Morocco, Atlas Mts., S of Azrou, Forêt de Cedres, N33°24'46" W05°12'53", 1679m, 22-Mai-2011, *Quercus*, sieving, leg. Stüben,P., det. Stüben,P.
Echinodera incognita<br>(A. Hoffmann, 1956)	GU213663 [01]<br>E-0418-inc<br>658nt	ZFMK-TIS-cE0418<br>ZFMK-DNA-0100400339	Spain, Castilla-La Mancha, S. de Alcaraz, 12 km SE of Alcaraz near Mt. Almenara, N38°34'17" W02°25'53", 1650m, 22-Apr-2003, *Quercus ilex*, leg. Stüben,P., det. Stüben,P.
Echinodera incognita<br>(A. Hoffmann, 1956)	GU213683 [01]<br>E-489-inc<br>658nt	ZFMK-TIS-cE489<br>ZFMK-DNA-0100400975	Spain, Andalucia, S. de Segura (S. del Pozo), 12 km SE of Cazorla, N37°52'53" W02°52'39", 1300m, 17-Apr-2003, *Quercus ilex* , leg. Stüben,P., det. Stüben,P.
Echinodera incognita<br>(A. Hoffmann, 1956)	GU213709 [01]<br>E-594-inc<br>658nt	ZFMK-TIS-cE594<br>ZFMK-DNA-0100404949	Spain, Albacete, Yeste, Los Prados, N38°20'47" W02°26'32", 1148m, 11-Jun-2008, *Juglans regia, Quercus ilex, Rubus*, leg. Astrin,J., det. Stüben,P.
Echinodera incognita<br>(A. Hoffmann, 1956)	GU213736 [01]<br>E-762-inc<br>658nt	ZFMK-TIS-cE762<br>ZFMK-DNA-0100405039	Spain, Malaga, NE of Malaga, Sierra de Tejeda, Comares, N36°50'38" W04°14'39", 499m, 08-Jan-2009, *Olea, Ceratonia siliqua*, deep sifting, leg. Stüben,P., det. Stüben
Echinodera incognita<br>(A. Hoffmann, 1956)	GU213737 [01]<br>E-763-inc<br>658nt	ZFMK-TIS-cE763<br>ZFMK-DNA-0100405037	Spain, Malaga, NE of Malaga, Sierra de Tejeda, Canillas de Aceituno, N36°52'25" W04°03'42", 641m, 10-Jan-2009, *Quercus ilex*, leg. Stüben,P., det. Stüben
Echinodera incognita<br>(A. Hoffmann, 1956)	KJ867598 [new]<br>1272-PST<br>658nt	ZFMK-TIS-4088<br>ZFMK-DNA-0100426204	Spain, Malaga, S of Antequera, Sierra de Chimenea, El Torcal, N36°57'18" W04°32'41", 1208m, 05-Mai-2013, *Quercus ilex*, sieving, leg. Stüben,P. & Schütte,A., det. Stüben,P.

**Name**<br>**Authority**<br>**Additional Information**	**GenBank Acc No (Ref.)**<br>**Specimen ID**<br>**Sequence Length**	**ZFMK Tissue ID**<br>**ZFMK DNA Sample ID**<br>**(SDEI DNA Sample ID)**	**Locality, GPS, Collection Date, Plant, Collector, Identifier**
Echinodera incognita<br>(A. Hoffmann, 1956)	KJ867610 [new]<br>1300-PST<br>658nt	ZFMK-TIS-4116<br>ZFMK-DNA-0100426169	Spain, Jaen, Sierra de Cazorla, N of Arroyo Frio, N38°00'13" W02°53'10", 762m, 14-Mai-2013, *Quercus ilex, Smilax*, sieving, leg. Stüben,P. & Schütte,A., det. Stüben,P.
Echinodera incognita<br>(A. Hoffmann, 1956)	KJ867622 [new]<br>1342-PST<br>658nt	ZFMK-TIS-4158<br>ZFMK-DNA-0100426134	Spain, Jaen, S of La Guardia, Sierra de la Pandera, N37°40'20" W03°41'48", 1223m, 19-Mai-2013, *Erinacea anthyllis, Cytisus* sp., sieving, leg. Stüben,P. & Schütte,A., det. Stüben,P.
Echinodera jandiaensis<br>Stüben, 2018<br>**Paratype (DNAtype)**<br>formerly: *E. montanus*	MF426964 [34]<br>2743-PST_23893<br>658nt	ZFMK-TIS-23893<br>ZFMK-DNA-0171661251	Spain, Canary Islands, Fuerteventura, Cumbre de Jandia, Morro del Jorao (via Barranco de los Canarios), N28°07'03" W14°19'59", 625m, 17-Jan-2015, beating, leg. Stüben,P., det. Stüben,P.
Echinodera jandiaensis<br>Stüben, 2018<br>formerly: *E. picta*	MG229884 [34]<br>2928-PST<br>658nt	(SDEI-DNA-2928-PST)	Spain, Canary Islands, Lanzarote, Calle La Rositas, Finca La Corona, N29°11'48" W13°29'33", 354m, 04-Jan-2017, sifting, leg. Stüben,P., det. Stüben,P.
Echinodera jandiaensis<br>Stüben, 2018<br>formerly: *E. picta*	MG229887 [34]<br>2935-PST<br>658nt	(SDEI-DNA-2935-PST)	Spain, Canary Islands, Lanzarote, above Haria, Valle de Malpaso, N29°07'57" W13°31'03", 453m, 04-Jan-2017, *Acacia*, beating, leg. Stüben,P., det. Stüben,P.
Echinodera ketamaensis<br>Stüben, 2002	GU213758 [01]<br>E-909-ket<br>658nt	ZFMK-TIS-cE909<br>ZFMK-DNA-0100405487	Morocco, Rif Mts., 10 km W of Ketama, N34°57'40" W04°40'51", 1600m, 10-Mai-2009, *Cedrus, Prunus*, leg. Stüben,P., det. Stüben,P.
Echinodera Krátkyi<br>Stüben, 2018<br>**Paratype (DNAtype)**	MF426967 [34]<br>2765-JKR_26214<br>658nt	ZFMK-TIS-26214<br>ZFMK-DNA-0171661895	Spain, Canary Islands, Gran Canaria, Angostura, Barranco de Santa Brigida, N28°03'12" W15°28'46.5", 334m, 22-Feb-2015, *Laurus*, sieving, leg. Krátky,J., det. Krátky,J.
Echinodera kroumiriensis<br>Stüben, 2004	GU213691 [01]<br>T-500-kro<br>658nt	ZFMK-TIS-cT500<br>ZFMK-DNA-0100400671	Tunisia, Kroumirie, 2 km E of Ain Draham, Jebel Bir, N36°46'20" E08°42'40", 770m, 20-Okt-2003, *Quercus suber, Quercus* sp., leg. Stüben,P., det. Stüben,P.
Echinodera lusitanica<br>Stüben, 2013	GU988115 [01]<br>E-863-pag<br>658nt	ZFMK-TIS-cE863<br>ZFMK-DNA-0100405538	Portugal, Sa. Arrabida, SW of Setubal, N38°28'13" W09°00'07", 311m, 27-Apr-2009, *Quercus ilex, Smilax*, leg. Astrin,J., det. Stüben,P.
Echinodera lusitanica<br>Stüben, 2013	GU988116 [01]<br>E-864-pag<br>658nt	ZFMK-TIS-cE864<br>ZFMK-DNA-0100405526	Portugal, NW Lisboa, Sintra, N38°47'40" W09°23'10", 269m, 28-Apr-2009, *Acer, Laurus, Hedera*, leg. Astrin,J., det. Stüben,P.
Echinodera lusitanica<br>Stüben, 2013	KF872197 [15]<br>859-PST<br>528nt	ZFMK-TIS-3772<br>ZFMK-DNA-0100414232	Portugal, Estremadura, N of Ericeira, Praia de Sao Lourenco near Ribamar, coast, N39°00'41" W09°25'14", 7m, 18-Mai-2012, *Ficus*, sieving, leg. Stüben,P., det. Stüben,P.
Echinodera lusitanica<br>Stüben, 2013	KF872199 [15]<br>857-PST<br>658nt	ZFMK-TIS-3770<br>ZFMK-DNA-0100414234	Portugal, Alentejo, Serra da Arràbida (west), N38°27'44" W09°00'09", 159m, 23-Mai-2012, *Quercus*, sieving, leg. Stüben,P., det. Stüben,P.
Echinodera lusitanica<br>Stüben, 2013	KF872200 [15]<br>855-PST<br>658nt	ZFMK-TIS-3768<br>ZFMK-DNA-0100413759	Portugal, Alentejo, Serra da Arràbida near Portinho da Arràbida, N38°39'03" W08°58'39", 51m, 20-Mai-2012, *Quercus, Ceratonia*, sieving, leg. Stüben,P., det. Stüben,P.
Echinodera lusitanica<br>Stüben, 2013<br>**Paratype (DNAtype)**	KF872201 [15]<br>844-PST_3757<br>658nt	ZFMK-TIS-3757<br>ZFMK-DNA-0100414242	Portugal, Estremadura, NW of Sintra, Serra de Sintra, Colares, Pe da Serra **(type locality)**, N38°47'13" W09°27'46", 323m, 16-Mai-2012, *Quercus*, sieving, leg. Stüben,P., det. Stüben,P.

**Name**<br>**Authority**<br>**Additional Information**	**GenBank Acc No (Ref.)**<br>**Specimen ID**<br>**Sequence Length**	**ZFMK Tissue ID**<br>**ZFMK DNA Sample ID**<br>**(SDEI DNA Sample ID)**	**Locality, GPS, Collection Date, Plant, Collector, Identifier**
Echinodera lusitanica<br>Stüben, 2014	GU213744 [01]<br>E-834-pag<br>658nt	ZFMK-TIS-cE834<br>ZFMK-DNA-0100405557	Spain, Madrid, NW of Colmenar Viejo, Pto. de Cotos – Valdesqui, N40°48'54" W03°57'33", 1784m, 20-Apr-2009, *Cytisus*, leg. Astrin,J., det. Stüben,P.
Echinodera lusitanica<br>Stüben, 2014	GU988097 [01]<br>E-837-pag<br>658nt	ZFMK-TIS-cE837<br>ZFMK-DNA-0100405560	Spain, Madrid, N of Colmenar Viejo, Pto. de Navafria, N40°59'00" W03°49'02", 1762m, 22-Apr-2009, *Cytisus* , leg. Astrin,J., det. Stüben,P.
Echinodera magnesia<br>Stüben, 2019<br>**NMB Paratype (DNAtype)**	MK347560 [36]<br>2977-PST<br>658nt	(SDEI-DNA-2977-PST)	Greece, Magnesia, Pelion Mt., 6.5 km SWAgios Dimitrios, N39°23'18" E23°04'50", 1293m, 06-Jul-2017, *Fagus*, leg. Stüben,P., det. Stüben,P.
Echinodera magnesia<br>Stüben, 2019<br>**NMB Paratype (DNAtype)**	MK347561 [36]<br>2983-PST<br>658nt	(SDEI-DNA-2983-PST)	Greece, Magnesia, Pelion Mt., N Lampinou, N39°21'19" E23°11'53", 353m, 07-Jul-2017, *Platanus*, leg. Stüben,P., det. Stüben,P.
Echinodera major<br>(A. & F. Solari, 1907)	EU286519 [01]<br>HR-0319-maj<br>618nt	ZFMK-TIS-cHR0319<br>ZFMK-DNA-0100400381	Croatia, Dalmatia, 7 km N of Makarska, Biokovo Mts., Bast, N43°21'26" E16°59'18", 415m, 05-Jul-2007, *Quercus*, limestone: moist hillside, leg. Stüben,P., det. Stüben,P.
Echinodera major<br>(A. & F. Solari, 1907)	GU213674 [01]<br>HR-0306-maj<br>658nt	ZFMK-TIS-cHR0306<br>ZFMK-DNA-0100400889	Croatia, Dalmatia, 15 km NW of Split, Kozjak Mts., Radosic, N43°36'31" E16°19'43", 240m, 01-Jul-2007, *Olea, Quercus, Carpinus*, limestone: Maccia, leg. Stüben,P., det. Stüben,P.
Echinodera major<br>(A. & F. Solari, 1907)	GU213675 [01]<br>HR-0329-maj<br>658nt	ZFMK-TIS-cHR0329<br>ZFMK-DNA-0100401015	Croatia, Dalmatia, 3 km NE of Makarska, Biokovo Mts., "Vosac", N43°18'53" E17°03'09", 1367m, 11-Jul-2007, *Fagus*, limestone, leg. Stüben,P., det. Stüben,P.
Echinodera merkli<br>(Meyer, 1896)	FJ716543 [01]<br>EP-0385-mer<br>658nt	ZFMK-TIS-cEP0385<br>ZFMK-DNA-0100400875	Greece, Epirus, N of Metsovo, Katara Pass, N39°46'59" E21°09'09", 1419m, 29-Sep-2007, *Fagus*, leg. Stüben,P., det. Stüben,P.
Echinodera merkli<br>(Meyer 1896)	MK347559 [new]<br>2976-PST<br>658nt	(SDEI-DNA-2976-PST)	Greece, Pieria, 1 km W Poroi, N39°58'08" E22°34'44", 608m, 02-Jul-2017, *Fagus*, leg. Stüben,P., det. Stüben,P.
Echinodera minosi<br>Bahr & Bayer, 2005	EU286464 [01]<br>GR-0150-min<br>658nt	ZFMK-TIS-cGR0150<br>ZFMK-DNA-0100400874	Greece, Crete Isl. West, Levka Ori, Imbros, above Imbros Gorge, N35°14'51" E24°10'30", 900m, 02-Okt-2006, *Quercus*, leg. Bahr,F. & Bayer,B., det. Bahr and Bayer
Echinodera montana<br>Stüben & Astrin, 2011	GU213735 [01]<br>E-802-mnt<br>658nt	ZFMK-TIS-cE802<br>ZFMK-DNA-0100405059	Spain, Canary Islands, Fuerteventura, Pico de la Zarza , N28°06' W14°21', 800m, 23-Dez-2008, leg. Brustel, det. Brustel
Echinodera montana<br>Stüben & Astrin, 2011	MG229796 [40]<br>2737-PST<br>658nt	ZFMK-TIS-23887<br>ZFMK-DNA-0171661262	Spain, Canary Islands, Fuerteventura, Cumbre de Jandia, Pico de Zarza , N28°06'06" W14°21'19", 802m, 12-Jan-2015, sieving, leg. Stüben,P., det. Stüben,P.
Echinodera nebrodiensis<br>Stüben, 2003<br>**Paratype (DNAtype)**	GU213661 [01]<br>I-0416-neb<br>658nt	ZFMK-TIS-cI0416<br>ZFMK-DNA-0100400341	Italy, Sicilia Isl. (ME), Mte. Soro 13 km NW of Cesarò, P.N. dei Nebrodi, N37°56'27" E14°38'08", 1400m, 19-Okt-2002, *Fagus, Ilex*, high forest, growths from the stump, leg. Stüben,P., det. Stüben,P.
Echinodera nebrodiensis<br>Stüben, 2003	GU213743 [01]<br>I-828-neb<br>658nt	ZFMK-TIS-cI828<br>ZFMK-DNA-0100405551	Italy, Sicilia Isl., Madonie Castelbuono, Rifugio Crispi Agrifol., Piano Pomo , N37°53'46" E14°04'01", 1400m, 07-Feb-2008, *Fagus*, leg. Kapp, det. Kapp
Echinodera nuraghia<br>Stüben, 2009<br>**Paratype (DNAtype)**	GU213685 [01]<br>I-493-nur<br>615nt	ZFMK-TIS-cI493<br>ZFMK-DNA-0100400211	Italy, Sardinia Isl., 13 km NE of Macomer near Lei, Massiv de Marghin, Mt. Lameddari, N40°19'43" E08°54'04", 990m, 29-Okt-2004, *Quercus ilex*, leg. Stüben,P., det. Stüben,P.

**Name**<br>**Authority**<br>**Additional Information**	**GenBank Acc No (Ref.)**<br>**Specimen ID**<br>**Sequence Length**	**ZFMK Tissue ID**<br>**ZFMK DNA Sample ID**<br>**(SDEI DNA Sample ID)**	**Locality, GPS, Collection Date, Plant, Collector, Identifier**
Echinodera nuraghia<br>Stüben, 2009	MG322670 [39]<br>IT1092c<br>658nt	ZFMK-TIS-cIT1092c<br>ZFMK-DNA-0112704586	Italy, Sardinia Isl. West, E of Macomer, above Lei, N40°19'54" E08°53'49", 1020m, 04-Okt-2010, *Quercus, Acer monspessulanum*, leg. Stüben,P., det. Stüben,P.
Echinodera ochsi<br>(F. Solari, 1952)	GU213694 [01]<br>F-444-och<br>658nt	ZFMK-TIS-cF444<br>ZFMK-DNA-0100400651	France, Alpes-Maritimes, 3 km NW of Sospel, Col de Braus, N43°52'22" E07°23'57", 1048m, 27-Dez-2007, *Quercus ilex,* broom, leg. Stüben,P., det. Stüben,P.
Echinodera ochsi<br>(F. Solari, 1952)	GU213695 [01]<br>F-446-och<br>658nt	ZFMK-TIS-cF446<br>ZFMK-DNA-0100400472	France, Alpes-Maritimes, 5 km S of Sospel, Mont Razet, N43°50'59" E07°28'28", 1269m, 28-Dez-2007, *Quercus ilex, Helleborus,* broom, leg. Stüben,P., det. Stüben,P.
Echinodera orbiculata<br>Wollaston, 1864	FJ716564 [01]<br>E-683-orb<br>658nt	ZFMK-TIS-cE683<br>ZFMK-DNA-0100404872	Spain, Canary Islands, Tenerife, NE of La Laguna, Anaga Mts. near Chinobre, N28°33'21" W16°10'46", 808m, 30-Sep-2008, *Laurus, Ixanthus viscosus*, leg. Astrin,J. & Stüben,P., det. Stüben,P.
Echinodera orbiculata<br>Wollaston, 1864	GU213716 [01]<br>E-691-orb<br>658nt	ZFMK-TIS-cE691<br>ZFMK-DNA-0100404857	Spain, Canary Islands, Tenerife, NE of La Laguna, Anaga Mts., Las Mercedes, N28°31'49" W16°17'12", 905m, 01-Okt-2008, *Laurisilva*, leg. Astrin,J. & Stüben,P., det. Stüben,P.
Echinodera orbiculata<br>Wollaston, 1864	GU213717 [01]<br>E-701-orb<br>658nt	ZFMK-TIS-cE701<br>ZFMK-DNA-0100404854	Spain, Canary Islands, Tenerife, E of Los Silos, Teno Mts., El Tanque Bajo, N28°21'39" W16°46'19", 534m, 03-Okt-2008, compost, leg. Astrin,J. & Stüben,P., det. Stüben,P.
Echinodera orbiculata<br>Wollaston, 1864	GU987935 [01]<br>E-562-orb<br>658nt	ZFMK-TIS-cE562<br>ZFMK-DNA-0100404980	Spain, Canary Islands, Tenerife, East Anaga Mts., E of La Laguna, E of Lomo de las Bodegas, N28°33'38" W16°09'20", 500m, 26-Dez-2003, cliff, compost, leg. Stüben,P., det. Stüben,P.
Echinodera orbiculata<br>Wollaston, 1864	GU987936 [01]<br>E-563-orb<br>624nt	ZFMK-TIS-cE563<br>ZFMK-DNA-0100404981	Spain, Canary Islands, Tenerife, 4 km E of Los Silos, El Tanque, N28°21'39" W16°46'18", 520m, 02-Jan-2004, *Foeniculum vulgare*, compost, leg. Stüben,P., det. Stüben,P.
Echinodera orientalis<br>(A. & F. Solari 1907)	MK347582 [new]<br>3019-KRA<br>658nt	(SDEI-DNA-3019-KRA)	Armenia, Syunik prov., Davit Bek, 2,5 km NW, N39°19'52" E46°28'8", 1312m, 07-Jun-2017, leg. Krátky,J., det. Stüben,P.
Echinodera paganettii<br>(F. Solari, 1952)	GU213747 [01]<br>E-855-pag<br>658nt	ZFMK-TIS-cE855<br>ZFMK-DNA-0100405530	Portugal, Sa. Estrela, SW of Manteigas, N40°18'45" W07°35'03", 1558m, 25-Apr-2009, *Cytisus*, leg. Astrin,J., det. Stüben,P.
Echinodera paganettii<br>(F. Solari, 1952)	GU987910 [01]<br>P-541-pag<br>658nt	ZFMK-TIS-cP541<br>ZFMK-DNA-0100400625	Portugal, Manteigas, N40°23'13" W07°32'06", 945m, 26-Jan-2008, *Quercus*, leg. Astrin,J., det. Astrin,J.
Echinodera paganettii<br>(F. Solari, 1952)	GU988113 [01]<br>E-858-pag<br>658nt	ZFMK-TIS-cE858<br>ZFMK-DNA-0100405533	Portugal, Sa. Estrela, near Manteigas, N40°23'13" W07°32'06", 946m, 25-Apr-2009, *Quercus pyrenaica, Cytisus multiflorus, Ruscus*, leg. Astrin,J., det. Stüben,P.
Echinodera paganettii<br>(F. Solari, 1952)	KF872198 [15]<br>858-PST<br>531nt	ZFMK-TIS-3771<br>ZFMK-DNA-0100414233	Portugal, Minho, PN of Peneda-Geres, N of Caldas do Geres, N41°45'47" W08°08'53", 798m, 12-Mai-2012, *Quercus, Fagus* , sieving, leg. Stüben,P., det. Stüben,P.
Echinodera paganettii<br>(F. Solari, 1952)	GU213748 [01]<br>E-860-pag<br>658nt	ZFMK-TIS-cE860<br>ZFMK-DNA-0100405535	Spain, Caceres, NE of Plasencia, S of Hervas, Pto. de Honduras, N40°13'17" W05°52'19", 1436m, 26-Apr-2009, *Cytisus* , leg. Astrin,J., det. Stüben,P.
Echinodera paganettii<br>(F. Solari, 1952)	GU987888 [01]<br>E-486-pag<br>658nt	ZFMK-TIS-cE486<br>ZFMK-DNA-0100400972	Spain, Caceres, 27 km N of Plasencia, Montes de Tras la Sierra, Puerto de Honduras, N40°13'17" W05°52'19", 1437m, 09-Apr-2004, *Cytisus* , leg. Stüben,P., det. Stüben,P.

**Name**<br>**Authority**<br>**Additional Information**	**GenBank Acc No (Ref.)**<br>**Specimen ID**<br>**Sequence Length**	**ZFMK Tissue ID**<br>**ZFMK DNA Sample ID**<br>**(SDEI DNA Sample ID)**	**Locality, GPS, Collection Date, Plant, Collector, Identifier**
Echinodera paganettii<br>(F. Solari, 1952)	GU988108 [01]<br>E-851-pag<br>658nt	ZFMK-TIS-cE851<br>ZFMK-DNA-0100405539	Spain, Salamanca, NW of Bejar near Herguijuela de la Sierra, N40°27'05" W06°04'45", 807m, 24-Apr-2009, *Fagus, Castanea, Alnus*, leg. Astrin,J., det. Stüben,P.
Echinodera pallida<br>Israelson, 1985	MG229764 [40]<br>1957-PST<br>658nt	ZFMK-TIS-24036<br>ZFMK-DNA-0169170387	Portugal, Madeira, Ilhas Selvagens, Selvagem Pequena, around Pico do Veado, N30°02'09" W16°01'39", 15m, 07-Okt-2014, *Patellifolia patellaris*, sieving, leg. Stüben,P., det. Stüben,P.
Echinodera pallida<br>Israelson, 1985	MG229766 [40]<br>1961-PST<br>658nt	ZFMK-TIS-24040<br>ZFMK-DNA-0169170391	Portugal, Madeira, Ilhas Selvagens, Selvagem Grande, in front of Cave Inferno, N30°08'19" W15°51'52", 18m, 09-Okt-2014, *Suaeda vera*, sieving, leg. Stüben,P., det. Stüben,P.
Echinodera pallida<br>Israelson, 1985	MG229767 [40]<br>1964-PST<br>658nt	ZFMK-TIS-24043<br>ZFMK-DNA-0169170394	Portugal, Madeira, Ilhas Selvagens, Selvagem Grande, slope S of (near) Cave Kidd, N30°8'30" W15°51'30", 51m, 20-Okt-2014, *Lotus glaucus*, sieving, leg. Stüben,P., det. Stüben,P.
Echinodera palmaensis<br>Stüben, 2000	GU213641 [01]<br>C-0146-pal<br>658nt	ZFMK-TIS-cC0146<br>ZFMK-DNA-0100400067	Spain, Canary Islands, La Palma, 1.5 km SW of Tijarafa, Las Caletas, N28°41'29" W17°57'29", 439m, 30-Jun-2006, *Persea*, leg. Stüben,P., det. Stüben,P.
Echinodera palmaensis<br>Stüben, 2000	GU213642 [01]<br>C-0147-pal<br>658nt	ZFMK-TIS-cC0147<br>ZFMK-DNA-0100400184	Spain, Canary Islands, La Palma, 1 km S of Las Caletas, N28°28'25" W17°50"08", 257m, 29-Jun-2006, *Ficus carica*, leg. Stüben,P., det. Stüben,P.
Echinodera palmaensis<br>Stüben, 2000	GU213671 [01]<br>E-0283-pal<br>658nt	ZFMK-TIS-cE0283<br>ZFMK-DNA-0100401035	Spain, Canary Islands, La Palma, Fuente de Candelaria, 2 km N of Tijarafe, N28°42'55" W17°56'48", 707m, 06-Jul-2006, leg. Stüben,P., det. Stüben,P.
Echinodera palmaensis<br>Stüben, 2000	MG229723 [40]<br>1695-PST<br>658nt	ZFMK-TIS-24413<br>ZFMK-DNA-0155622495	Spain, Canary Islands, La Palma, south of Tijarafe, El Jesus (Barranco), N28°42'26" W17°56'54", 610m, 20-Dez-2013, sieving, leg. Stüben,P., det. Stüben,P.
Echinodera pelionis<br>(Frieser, 1955)	MK347557 [new]<br>2974-PST<br>658nt	(SDEI-DNA-2974-PST)	Greece, Pieria, Kallipefki, N39°57'44" E22°29'17", 1489m, 29-Jun-2017, *Fagus*, leg. Stüben,P., det. Stüben,P.
Echinodera pelionis<br>(Frieser, 1955)	MK347562 [new]<br>2984-PST<br>658nt	(SDEI-DNA-2984-PST)	Greece, Magnesia, Pelion Mt., 1.5 km NE Milies, N39°20'14" E23°09'46", 674m, 08-Jul-2017, *Platanus*, leg. Stüben,P., det. Stüben,P.
Echinodera peragalloi<br>(Chevrolat, 1863)	GU213693 [01]<br>F-439-per<br>658nt	ZFMK-TIS-cF439<br>ZFMK-DNA-0100400259	France, Alpes-Maritimes, 8 km S of Sospel, Castillon, Forêt de Menton, N43°49'14" E07°28'54", 321m, 25-Dez-2007, *Quercus ilex*, leg. Stüben,P., det. Stüben,P.
Echinodera peragalloi<br>(Chevrolat, 1863)	GU213696 [01]<br>F-448-per<br>658nt	ZFMK-TIS-cF448<br>ZFMK-DNA-0100400647	France, Alpes-Maritimes, beneath Castagniers, N43°47'25" E07°13'50", 341m, 29-Dez-2007, *Quercus pubescens, Olea*, leg. Stüben,P., det. Stüben,P.
Echinodera peragalloi<br>(Chevrolat, 1863)	GU213697 [01]<br>I-492-per<br>658nt	ZFMK-TIS-cI492<br>ZFMK-DNA-0100400588	Italy, Sardinia Isl., 12 km NE of Lula, Monte Albo, N40°32'15" E09°36'11", 750m, 19-Okt-2004, *Quercus ilex*, leg. Stüben,P., det. Stüben,P.
Echinodera peragalloi<br>(Chevrolat, 1863)	MG229742 [new]<br>1784-PST<br>658nt	ZFMK-TIS-24108<br>ZFMK-DNA-0171624075	Italy, Sardinia Isl., N of Tortoli, P. Pedra Longa, N40°01'16" E09°41'17", 343m, 10-Mai-2014, *Pistacia*, sieving, leg. Stüben,P. & Schütte,A., det. Stüben,P.
Echinodera peragalloi<br>(Chevrolat, 1863)	MG322656 [new]<br>IT1067c<br>658nt	ZFMK-TIS-cIT1067c<br>ZFMK-DNA-0112704609	Italy, Sardinia Isl. East, N of Dorgali near G.ta di Ispinigoli, N40°19'12" E09°36'00", 241m, 26-Sep-2010, *Quercus ilex, Pistacia*, cliff, limestone, leg. Stüben,P., det. Stüben,P.

**Name**<br>**Authority**<br>**Additional Information**	**GenBank Acc No (Ref.)**<br>**Specimen ID**<br>**Sequence Length**	**ZFMK Tissue ID**<br>**ZFMK DNA Sample ID**<br>**(SDEI DNA Sample ID)**	**Locality, GPS, Collection Date, Plant, Collector, Identifier**
Echinodera peragalloi<br>(Chevrolat, 1863)	MG322657 [new]<br>IT1069c<br>658nt	ZFMK-TIS-cIT1069c<br>ZFMK-DNA-0112704611	Italy, Sardinia Isl. East, S of Dorgali, Genna Silana, N40°09'30" E09°30'30", 1017m, 27-Sep-2010, *Quercus ilex, Ficus*, cliff, leg. Stüben,P., det. Stüben,P.
Echinodera peragalloi<br>(Chevrolat, 1863)	MG322658 [new]<br>IT1070c<br>658nt	ZFMK-TIS-cIT1070c<br>ZFMK-DNA-0112704599	Italy, Sardinia Isl. East, S of Dorgali, Genna Silana, N40°09'30" E09°30'30", 1017m, 27-Sep-2010, *Quercus ilex, Ficus*, cliff, leg. Stüben,P., det. Stüben,P.
Echinodera peragalloi<br>(Chevrolat, 1863)	MG322661 [new]<br>IT1075c<br>658nt	ZFMK-TIS-cIT1075c<br>ZFMK-DNA-0112704594	Italy, Sardinia Isl. East, NE of Seui, M. Tonneri, N39°53'59" E9°23'24", 926m, 28-Sep-2010, *Quercus ilex*, cliff, leg. Stüben,P., det. Stüben,P.
Echinodera peragalloi<br>(Chevrolat, 1863)	MG322664 [39]<br>IT1082c<br>658nt	ZFMK-TIS-cIT1082c<br>ZFMK-DNA-0112702487	Italy, Sardinia Isl., S of Aritzo, Gadoni, R. Tistigliosi x F. Flumendosa, N39°53'57" E09°11'02", 490m, 30-Sep-2010, *Quercus ilex*, leg. Stüben,P., det. Stüben,P.
Echinodera peragalloi<br>(Chevrolat, 1863)	MG322671 [new]<br>IT1093c<br>658nt	ZFMK-TIS-cIT1093c<br>ZFMK-DNA-0112702510	Italy, Sardinia Isl. East, E of Oliena near Su Gologone, above river, N40°17'15" E09°30'29", 161m, 05-Okt-2010, *Quercus*, leg. Stüben,P., det. Stüben,P.
Echinodera peragalloi<br>(Chevrolat, 1863)	MG322673 [new]<br>IT1095c<br>658nt	ZFMK-TIS-cIT1095c	Italy, Sardinia Isl. East, N of Dorgali, M. Tuttavista (Gipfel), N40°22'46" E09°38'22", 801m, 06-Okt-2010, *Quercus ilex*, leg. Stüben,P., det. Stüben,P.
Echinodera peragalloi<br>(Chevrolat, 1863)	MG322650 [new]<br>ES1050<br>658nt	ZFMK-TIS-cES1050<br>ZFMK-DNA-0112704619	Spain, Cataluna, Girona, E of Vulpellac, 41°56'907N 03°06'580E, 109m, 24-Apr-2010, leg. Krátky, det. Krátky,J.
Echinodera personata<br>Colonnelli, 1985	FJ716568 [01]<br>E-693-per<br>658nt	ZFMK-TIS-cE693<br>ZFMK-DNA-0100404859	Spain, Canary Islands, Tenerife, W of Los Silos, Teno Mts., Casa Blanca near Buenavista del Norte, N28°21'39" W16°52'12", 130m, 02-Okt-2008, roadside, leg. Astrin,J. & Stüben,P., det. Stüben,P.
Echinodera personata<br>Colonnelli, 1985	GU213703 [01]<br>E-566-pen<br>658nt	ZFMK-TIS-cE566<br>ZFMK-DNA-0100404268	Spain, Canary Islands, Tenerife, 6 km W of Los Silos, Casa Blanca, N28°21'40" W16°52'15", 120m, 27-Dez-2003, *Foeniculum vulgare*, succulent bush, leg. Stüben,P., det. Stüben,P.
Echinodera personata<br>Colonnelli, 1985	GU213704 [01]<br>E-567-pen<br>658nt	ZFMK-TIS-cE567<br>ZFMK-DNA-0100404985	Spain, Canary Islands, Tenerife, S of Buenavista del Norte, S of Masca, N28°18'05" W16°49'36", 900m, 02-Jan-2004, sieved: leaf litter and compost, leg. Stüben,P., det. Stüben,P.
Echinodera personata<br>Colonnelli, 1985	KJ867582 [40]<br>754-PST<br>658nt	ZFMK-TIS-3091<br>ZFMK-DNA-0100448132	Spain, Canary Islands, Tenerife, Teno Mts., Teno Alto, El Bailadero, N28°20'35.39" W16°53'4.80", 740m, 17-Feb-2012, *Carlina* sp., sieving, leg. Stüben,P., det. Stüben,P.
Echinodera picta<br>Wollaston, 1864	GU213742 [01]<br>E-803-pic<br>658nt	ZFMK-TIS-cE803<br>ZFMK-DNA-0100404767	Spain, Canary Islands, Fuerteventura, Betancuria, N28°25' W14°03', 390m, 22-Dez-2008, leg. Brustel, det. Brustel
Echinodera picta<br>Wollaston, 1864	MG229795 [40]<br>2728-PST<br>658nt	ZFMK-TIS-23878<br>ZFMK-DNA-0171661271	Spain, Canary Islands, Fuerteventura, 2 km NE of Vega de Rio Palmas, N28°24'10" W14°03'39", 320m, 04-Jan-2015, *Acacia (Fabaceae)*, sieving, leg. Stüben,P., det. Stüben,P.
Echinodera pseudohystrix<br>Stüben, 2000	FJ716575 [01]<br>E-718-psh<br>658nt	ZFMK-TIS-cE718<br>ZFMK-DNA-0100404836	Spain, Canary Islands, La Gomera, S of Hermigua, El Cedro, Las Mimbreras, N28°07'27" W17°13'26", 901m, 06-Okt-2008, *Laurisilva*, leg. Astrin,J. & Stüben,P., det. Stüben,P.
Echinodera pseudohystrix<br>Stüben, 2000<br>formerly: *E. gomerensis*	GU213706 [01]<br>E-569-gom<br>658nt	ZFMK-TIS-cE569<br>ZFMK-DNA-0100404974	Spain, Canary Islands, La Gomera, 2 km W of Hermigua, N28°09'51" W17°12'20", 250m, 28-Dez-2004, *under Persea*, leg. Stüben,P., det. Stüben,P.

**Name**<br>**Authority**<br>**Additional Information**	**GenBank Acc No (Ref.)**<br>**Specimen ID**<br>**Sequence Length**	**ZFMK Tissue ID**<br>**ZFMK DNA Sample ID**<br>**(SDEI DNA Sample ID)**	**Locality, GPS, Collection Date, Plant, Collector, Identifier**
Echinodera pseudohystrix<br>Stüben, 2000	GU213720 [01]<br>E-719-psh<br>658nt	ZFMK-TIS-cE719<br>ZFMK-DNA-0100404837	Spain, Canary Islands, La Gomera, S of Vallehermoso, La Meseta, "La Piedra Encantada", N28°09'15" W17°17'36", 819m, 07-Okt-2008, *Ocotea foetens*, leg. Astrin,J. & Stüben,P., det. Stüben,P.
Echinodera pseudohystrix<br>Stüben, 2000<br>formerly: *E. gomerensis*	GU213721 [01]<br>E-731-gom<br>658nt	ZFMK-TIS-cE731<br>ZFMK-DNA-0100404824	Spain, Canary Islands, La Gomera, SE of Hermigua near Casas del Palmar, N28°09'29" W17°09'37", 627m, 08-Okt-2008, thermophilic brushwood, leg. Astrin,J. & Stüben,P., det. Stüben,P.
Echinodera pseudohystrix<br>Stüben, 2000<br>formerly: *E. praedicta*	GU213722 [01]<br>E-732-pra<br>658nt	ZFMK-TIS-cE732<br>ZFMK-DNA-0100404823	Spain, Canary Islands, La Gomera, W of Hermigua, beneath Agulo, N28°11'29" W17°11'33", 137m, 09-Okt-2008, terraces, compost, leg. Astrin,J. & Stüben,P., det. Stüben,P.
Echinodera pseudohystrix<br>Stüben, 2000<br>formerly: *E. gomerensis*	GU213723 [01]<br>E-734-gom<br>658nt	ZFMK-TIS-cE734<br>ZFMK-DNA-0100404821	Spain, Canary Islands, La Gomera, Hermigua, Ibo Alfaro, N28°09'58" W17°12'11", 255m, 09-Okt-2008, *Persea*, terraces, leg. Astrin,J. & Stüben,P., det. Stüben,P.
Echinodera pseudohystrix<br>Stüben, 2000<br>formerly: *E. gomerensis*	GU213724 [01]<br>E-739-gom<br>658nt	ZFMK-TIS-cE739<br>ZFMK-DNA-0100404809	Spain, Canary Islands, La Gomera, S of Hermigua, N28°08'45" W17°12'19", 282m, 10-Okt-2008, *Ficus, Persea*, stream valley, leg. Astrin,J. & Stüben,P., det. Stüben,P.
Echinodera pseudohystrix<br>Stüben, 2000<br>formerly: *E. praedicta*	GU213725 [01]<br>E-741-pra<br>658nt	ZFMK-TIS-cE741<br>ZFMK-DNA-0100404811	Spain, Canary Islands, La Gomera, W of Hermigua, beneath Agulo, N28°11'28" W17°11'36", 170m, 11-Okt-2008, *Persea, Mangifera*, terraces, leg. Astrin,J. & Stüben,P., det. Stüben,P.
Echinodera pseudohystrix<br>Stüben, 2000<br>formerly: *E. gomerensis*	GU213728 [01]<br>E-748-gom<br>658nt	ZFMK-TIS-cE748<br>ZFMK-DNA-0100404721	Spain, Canary Islands, La Gomera, S of Hermigua, above Hermigua, N28°08'01" W17°11'59", 569m, 14-Okt-2008, moist terraces, detritus, leg. Astrin,J., det. Stüben,P.
Echinodera pseudohystrix<br>Stüben, 2000<br>formerly: *E. gomerensis*	GU213729 [01]<br>E-749-gom<br>658nt	ZFMK-TIS-cE749<br>ZFMK-DNA-0100404722	Spain, Canary Islands, La Gomera, S of Hermigua, above Hermigua, N28°08'01" W17°11'59", 569m, 14-Okt-2008, moist terraces, detritus, leg. Astrin,J., det. Stüben,P.
Echinodera pseudohystrix<br>Stüben, 2000<br>formerly: E. gomerensis	GU213730 [01]<br>E-750-gom<br>658nt	ZFMK-TIS-cE750<br>ZFMK-DNA-0100405083	Spain, Canary Islands, La Gomera, S of Hermigua, above Hermigua, N28°08'01" W17°11'59", 569m, 14-Okt-2008, moist terraces, detritus, leg. Astrin,J., det. Stüben,P.
Echinodera pseudohystrix<br>Stüben, 2000	MG322644 [40]<br>ES1008<br>658nt	ZFMK-TIS-cES1008<br>ZFMK-DNA-0112704661	Spain, Canary Islands, La Gomera, Valle Gran Rey, La Vizcaina, 28°6'57.00"N 17°18'24.00"W, 390m, 09-Dez-2009, *Salix*, garden waste, leg. Stüben,P., det. Stüben,P.
Echinodera pseudohystrix<br>Stüben, 2000	MG322645 [40]<br>ES1019<br>658nt	ZFMK-TIS-cES1019<br>ZFMK-DNA-0112704657	Spain, Canary Islands, La Gomera, E of Hermigua, El Palmar, 28°09'26"N 17°09'47"W, 630m, 30-Dez-2009, *Pericallis*, thermophilic brushwood, leg. Stüben,P., det. Stüben,P.
Echinodera pseudohystrix<br>Stüben, 2000<br>formerly: *E. praedicta* - Syn!	MN614443 [40]<br>ES1027<br>658nt	ZFMK-TIS-cES1027<br>ZFMK-DNA-0112704642	Spain, Canary Islands, La Gomera, below Agulo/Lepe **(type locality)**, 28°11'17"N 17°11'12"W, 19m, 15-Jan-2010, gardens: trash pile near the beach, leg. Stüben,P., det. Stüben,P.
Echinodera pseudovariegata<br>Stüben, 1998	MG229890 [new]<br>2946-PST<br>658nt	(SDEI-DNA-2946-PST)	Greece, Crete Isl., Psiloritis Mts., Zoniana env., N35°14'57" E24°47'31", 1400m, 19-Apr-2015, leg. Kocian,M. via Krátky,J., det. Stüben,P.
Echinodera pseudovariegata<br>Stüben, 1998	MG229746 [new]<br>1817-PST<br>658nt	ZFMK-TIS-24122<br>ZFMK-DNA-0171624047	Greece, Rhodes Isl., above Salakos, N36°17'07" E27°56'24", 267m, 04-Mai-2014, sieving, leg. Bahr,F., det. Bahr,F.
Echinodera pseudovariegata<br>Stüben, 1998	MG229792 [new]<br>2654-PST<br>658nt	ZFMK-TIS-23516<br>ZFMK-DNA-0171600671	Greece, Rhodes Isl., above Salakos, N36°17'07" E27°56'24", 267m, 04-Mai-2014, sifting, leg. Bahr,F., det. Bahr,F.

**Name**<br>**Authority**<br>**Additional Information**	**GenBank Acc No (Ref.)**<br>**Specimen ID**<br>**Sequence Length**	**ZFMK Tissue ID**<br>**ZFMK DNA Sample ID**<br>**(SDEI DNA Sample ID)**	**Locality, GPS, Collection Date, Plant, Collector, Identifier**
Echinodera pseudovariegata<br>Stüben, 1998	MK347556 [new]<br>2972-PST<br>658nt	(SDEI-DNA-2972-PST)	Greece, Rhodos, Atavyros-Gebirge, Aghios Isidhoros, N36°9'41" E27°50'45", 500m, 08-Jun-2017, *Quercus*, evergreen Hartlaub's shrub, leg. Braunert, det. Braunert
Echinodera rifensis<br>Stüben, 2001	GU213753 [01]<br>E-893-rif<br>658nt	ZFMK-TIS-cE893<br>ZFMK-DNA-0100405498	Morocco, Rif, E Bab-Taza, E of Bab-Taza, Chefarat, N35°03'36" W05°06'57", 861m, 07-Mai-2009, *Quercus*, leg. Stüben,P., det. Stüben,P.
Echinodera rifensis<br>Stüben, 2001	GU213754 [01]<br>E-894-rif<br>658nt	ZFMK-TIS-cE894<br>ZFMK-DNA-0100405497	Morocco, Rif, N Bab-Taza, N of Bab-Taza, Talembote, N35°14'47" W05°11'31", 361m, 08-Mai-2009, *Pistacia*, leg. Stüben,P., det. Stüben,P.
Echinodera rifensis<br>Stüben, 2001	GU213755 [01]<br>E-895-rif<br>658nt	ZFMK-TIS-cE895<br>ZFMK-DNA-0100405496	Morocco, Rif, SE Oued-Laou, SE of Oued-Laou, vir. Bou Hamed, N35°21'44" W04°59'05", 121m, 08-Mai-2009, *Pistacia*, leg. Stüben,P., det. Stüben,P.
Echinodera rifensis<br>Stüben, 2001	GU988136 [01]<br>E-896-rif<br>658nt	ZFMK-TIS-cE896<br>ZFMK-DNA-0100405495	Morocco, Rif, SW Oued-Laou, SW of Oued-Laou, river, O. Laou, N35°17'47" W05°13'38", 210m, 08-Mai-2009, *Quercus suber, Smilax, Arbutus* , leg. Stüben,P., det. Stüben,P.
Echinodera roudieri<br>Stüben, 1998	EU286482 [01]<br>E-0155-rou<br>658nt	ZFMK-TIS-cE0155<br>ZFMK-DNA-0100400249	Spain, Alicante, 5km NW of Callosa d'en Sarrià, Sierra de Aixorta, Morro Blan, N38°41'38" W00°09'42", 1106m, 26-Dez-2005, *Quercus ilex*, leg. Stüben,P., det. Stüben,P.
Echinodera roudieri<br>Stüben, 1998	GU213664 [01]<br>E-0179-rou<br>658nt	ZFMK-TIS-cE0179<br>ZFMK-DNA-0100400240	Spain, Alicante, 9 km SE of Alcoi, Sierra del Rentonar, Puerto del Rentonar, N38°38'05" W00°24'25", 1011m, 05-Apr-2007, *Quercus ilex*, limestone, leg. Astrin,J. & Stüben,P., det. Stüben,P.
Echinodera roudieri<br>Stüben, 1998	GU213708 [01]<br>E-591-rou<br>658nt	ZFMK-TIS-cE591<br>ZFMK-DNA-0100404263	Spain, Alicante, near Pedreguer, N38°46'53" E00°00'52", 136m, 09-Jun-2008, *Quercus ilex* , leg. Astrin,J., det. Stüben,P.
Echinodera roudieri<br>Stüben, 1998	GU987818 [01]<br>E-0182-rou<br>658nt	ZFMK-TIS-cE0182<br>ZFMK-DNA-0100400028	Spain, Alicante, 7 km SW of Alcoi, Sierra de Menechaor, Santuario de la Font Roja, N38°39'34" W00°32'29", 296m, 05-Apr-2007, *Quercus ilex, Erinacea anthyllis*, limestone, leg. Astrin,J. & Stüben,P., det. Stüben,P.
Echinodera roudieri<br>Stüben, 1998	GU987823 [01]<br>E-0176-rou<br>658nt	ZFMK-TIS-cE0176<br>ZFMK-DNA-0100400334	Spain, Alicante, 6,5 NW of Callosa d'En Sarria Sarrià, Sierra de Aixorta, N38°41'59" W00°10'34", 1091m, 04-Apr-2007, *Erinacea anthyllis, Quercus ilex*, limestone, leg. Astrin,J. & Stüben,P., det. Stüben,P.
Echinodera roudieri<br>Stüben, 1998	GU987923 [01]<br>E-487-rou<br>658nt	ZFMK-TIS-cE487<br>ZFMK-DNA-0100400585	Spain, Alicante, 5 km NW of Callosa d'en Sarrià, Sierra de Aixorta, Morro Blan, N38°41'38" W00°09'42", 1106m, 26-Dez-2005, *Quercus ilex*, limestone, leg. Stüben,P., det. Stüben,P.
Echinodera roudieri<br>Stüben, 1998	GU987945 [01]<br>E-592-rou<br>658nt	ZFMK-TIS-cE592<br>ZFMK-DNA-0100404244	Spain, Alicante, near Pedreguer, N38°46'53" E00°00'52", 136m, 09-Jun-2008, *Quercus ilex* , leg. Astrin,J., det. Stüben,P.
Echinodera roudieri<br>Stüben, 1998	KJ867574 [new]<br>566-PST<br>651nt	ZFMK-TIS-20351<br>ZFMK-DNA-0100448413	Spain, Alicante, Port du Tudons (pass), Sierra de Aitana, N38°39'1" W00°19'29", 1050m, 03-Mai-2011, sieving, leg. Kresl,P., det. Stüben,P.
Echinodera samosa<br>Germann, 2012	KJ867584 [new]<br>818-PST<br>658nt	ZFMK-TIS-3731<br>ZFMK-DNA-0100414264	Greece, Samos Isl., Oros Ambelos, Lazarou, north side, cliff, N37°45'27" E26°50'36", 860m, 01-Apr-2010, *moss, leaf litter, cushion plants*, sieving, leg. Germann, det. Germann,C.
Echinodera setosagracilis<br>Stüben, 2004<br>**Paratype (DNAtype)**	GU213692 [01]<br>T-504-set<br>658nt	ZFMK-TIS-cT504<br>ZFMK-DNA-0100400667	Tunisia, Kef Soltane, 28 km E of Thala, N35°33'40" E08°58'39", 1200m, 24-Okt-2003, *Quercus ilex*, limestone, leg. Stüben,P., det. Stüben,P.

**Name**<br>**Authority**<br>**Additional Information**	**GenBank Acc No (Ref.)**<br>**Specimen ID**<br>**Sequence Length**	**ZFMK Tissue ID**<br>**ZFMK DNA Sample ID**<br>**(SDEI DNA Sample ID)**	**Locality, GPS, Collection Date, Plant, Collector, Identifier**
Echinodera settefratelliensis<br>Stüben, 2005<br>**Paratype (DNAtype)**	GU213686 [01]<br>I-495-set<br>658nt	ZFMK-TIS-cI495<br>ZFMK-DNA-0100400676	Italy, Sardinia Isl. South, 33 km NE of Cagliari, Sarrabus (Mts.), Rio di Cannas, N39°20'52" E09°27'18", 110m, 25-Okt-2004, *Quercus ilex, Arbutus, Salix, Pistacia lentiscus*, leg. Stüben,P., det. Stüben,P.
Echinodera settefratelliensis<br>Stüben, 2005	MG322665 [new]<br>IT1083c<br>658nt	ZFMK-TIS-cIT1083c<br>ZFMK-DNA-0112704577	Italy, Sardinia Isl. South, W of Muravera, M. Narba, N39°24'06" E09°32'30", 352m, 01-Okt-2010, *Quercus ilex*, leg. Stüben,P., det. Stüben,P.
Echinodera settefratelliensis<br>Stüben, 2005	MG322667 [new]<br>IT1085c<br>658nt	ZFMK-TIS-cIT1085c<br>ZFMK-DNA-0112704579	Italy, Sardinia Isl. South, E of Cagliari, M. d. Sette Fratelli near Pta. Moitzus, N39°16'38" E09°28'35", 167m, 02-Okt-2010, *Quercus, Pistacia*, dried-out stream, leg. Stüben,P., det. Stüben,P.
Echinodera settefratelliensis<br>Stüben, 2005	MG322668 [new]<br>IT1089c<br>658nt	ZFMK-TIS-cIT1089c<br>ZFMK-DNA-0112704583	Italy, Sardinia Isl. South, E of Cagliari, below Monte Cresia (F. Cuba), N39°15'41" E09°23'00", 507m, 03-Okt-2010, *Quercus*, leg. Stüben,P., det. Stüben,P.
Echinodera siciliensis<br>Stüben, 2003	EU286480 [01]<br>I-0153-sic<br>658nt	ZFMK-TIS-cI0153<br>ZFMK-DNA-0100400248	Italy, Sicilia Isl. (TP), 26 km NE of Trapani, SE of Capo San Vito, N38°09'00" E12°46'19", 35m, 14-Okt-2006, *Quercus, Fraxinus*, limestone cave, leg. Stüben,P., det. Stüben,P.
Echinodera siciliensis<br>Stüben, 2003	GU213645 [01]<br>I-0152-sic<br>658nt	ZFMK-TIS-cI0152<br>ZFMK-DNA-0100400096	Italy, Sicilia Isl. (PA), 19 km SE of Bagheria, Monti di Calamigna, NE of Ventimiglia di Sicilia, N37°55'33" E13°35'48", 581m, 11-Okt-2006, *Quercus ilex*, leg. Stüben,P., det. Stüben,P.
Echinodera siciliensis<br>Stüben, 2003	GU987765 [01]<br>D-0070-sic<br>658nt	ZFMK-TIS-cD0070<br>ZFMK-DNA-0100400746	Italy, Sicilia Isl., 4 km SSW of Godrano, N37°54'00" E13°25'00", 16-Okt-2002, leg. Stüben,P., det. Stüben,P.
Echinodera siciliensis<br>Stüben, 2003	GU988091 [01]<br>I-825-sic<br>658nt	ZFMK-TIS-cI825<br>ZFMK-DNA-0100405565	Italy, Sicilia Isl., Monte Soro, N37°55' E14°41', 1830m, 05-Feb-2008, *Fagus*, leg. Kapp, det. Kapp
Echinodera siciliensis<br>Stüben, 2003	MG322642 [new]<br>I-952-gar<br>658nt	ZFMK-TIS-cI952<br>ZFMK-DNA-0112704670	Italy, Puglia, Gargano, Casa D'Arguma, N41°45'35.27" E15°48'27", 584m, 26-Apr-2008, leg. Krátky,J., det. Krátky,J.
Echinodera soumasi<br>Germann, Wolf & Schütte, 2015<br>**Paratype (DNAtype)**	KT289402 [19]<br>2798-PST_26152<br>658nt	ZFMK-TIS-26152<br>ZFMK-DNA-0169166944	Greece, Peloponnese, NE of Pilos, S of Kazama, Polilimnos, N36°59'00" E21°51'16", 300m, 26-Sep-2014, creek valley, sieving, leg. Germann,Ch., det. Germann,Ch.
Echinodera spinosa<br>Stüben, 2006	EU286478 [01]<br>E-0149-spi<br>658nt	ZFMK-TIS-cE0149<br>ZFMK-DNA-0100400045	Spain, Andalucia, 11 km S of Ronda, Sierra de las Nieves **(type locality)**, N36°39'51" W05°05'01", 1047m, 03-Okt-2005, *Quercus ilex*, leg. Stüben,P., det. Stüben,P.
Echinodera spinosa<br>Stüben, 2006	GU213666 [01]<br>E-0199-spi<br>658nt	ZFMK-TIS-cE0199<br>ZFMK-DNA-0100400227	Spain, Cadiz, 2.5 km N of Ubrique, Sierra de Ubrique, N36°41'50" W05°26'20", 646m, 10-Apr-2007, *Quercus ilex, Ceratonia, Olea, Pistacia lentiscus*, leg. Astrin,J. & Stüben,P., det. Stüben,P.
Echinodera spinosa<br>Stüben, 2006	GU213668 [01]<br>E-0214-spi<br>658nt	ZFMK-TIS-cE0214<br>ZFMK-DNA-0100400312	Spain, Malaga, 7.5 km N of Estepona, Sierra Bermeja,Los Reales, N36°29'32" W05°11'45", 1071m, 13-Apr-2007, *Quercus ilex,* broom, leg. Astrin,J. & Stüben,P., det. Stüben,P.
Echinodera spinosa<br>Stüben, 2006	GU213738 [01]<br>E-776-spi<br>658nt	ZFMK-TIS-cE776<br>ZFMK-DNA-0100405029	Spain, Malaga, N of Marbella, Puerto de Ojen, N36°34'33" W04°51'25", 545m, 16-Jan-2009, *Ceratonia*, leg. Stüben,P., det. Stüben,P.

**Name**<br>**Authority**<br>**Additional Information**	**GenBank Acc No (Ref.)**<br>**Specimen ID**<br>**Sequence Length**	**ZFMK Tissue ID**<br>**ZFMK DNA Sample ID**<br>**(SDEI DNA Sample ID)**	**Locality, GPS, Collection Date, Plant, Collector, Identifier**
Echinodera spinosa<br>Stüben, 2006	GU213739 [01]<br>E-779-spi<br>658nt	ZFMK-TIS-cE779<br>ZFMK-DNA-0100405033	Spain, Malaga, NW of Marbella, Benahavis, N36°31'00" W05°02'23", 129m, 18-Jan-2009, *Ceratonia, Pistacia*, leg. Stüben,P., det. Stüben,P.
Echinodera spinosa<br>Stüben, 2006	GU987828 [01]<br>E-0196-spi<br>658nt	ZFMK-TIS-cE0196<br>ZFMK-DNA-0100400224	Spain, Malaga, 9 km SE of Ubrique, Sierra de Libar, N36°36'52" W05°23'16, 663m, 10-Apr-2007, *Quercus ilex, Ceratonia*, cliff, leg. Astrin,J. & Stüben,P., det. Stüben,P.
Echinodera spinosa<br>Stüben, 2006	GU987829 [01]<br>E-0200-spi<br>658nt	ZFMK-TIS-cE0200<br>ZFMK-DNA-0100400203	Spain, Malaga, 20 km NE of Ubrique, Sierra de Grazalema, N36°46'32" W05°15'05", 688m, 10-Apr-2007, *Quercus , Ceratonia*, stream valley, leg. Astrin,J. & Stüben,P., det. Stüben,P.
Echinodera spinosa<br>Stüben, 2006	GU987831 [01]<br>E-0202-spi<br>658nt	ZFMK-TIS-cE0202<br>ZFMK-DNA-0100400330	Spain, Malaga, 13 km SE of Ubrique, Sierra de Libar, Cortes de la Fa., N36°38'29" W05°18'26", 439m, 11-Apr-2007, *Quercus ilex,* broom, leg. Astrin,J. & Stüben,P., det. Stüben,P.
Echinodera spinosa<br>Stüben, 2006	GU987836 [01]<br>E-0217-spi<br>658nt	ZFMK-TIS-cE0217<br>ZFMK-DNA-0100400251	Spain, Cadiz, 14 km N of Ubrique, Sierra Zafalgar, Puerto de las Palomas, N36°47'16" W05°22'36", 1286m, 14-Apr-2007, broom, *Bupleurum, Euphorbia nicaensis*, leg. Astrin,J. & Stüben,P., det. Stüben,P.
Echinodera spinosa<br>Stüben, 2006	GU988059 [01]<br>E-766-spi<br>658nt	ZFMK-TIS-cE766<br>ZFMK-DNA-0100405023	Spain, Malaga, N of Estepona, Sierra Bermeja, Los Reales, N36°29'32" W05°11'47", 1071m, 12-Jan-2009, broom, leg. Stüben,P., det. Stüben,P.
Echinodera spinosa<br>Stüben, 2006	GU988065 [01]<br>E-777-spi<br>658nt	ZFMK-TIS-cE777<br>ZFMK-DNA-0100405031	Spain, Malaga, NW of Marbella, Sierra de las Nieves **(type locality)**, N36°39'52" W05°04'57", 1043m, 17-Jan-2009, *Quercus ilex*, leg. Stüben,P., det. Stüben,P.
Echinodera spinosa<br>Stüben, 2006	KJ867604 [new]<br>1286-PST<br>658nt	ZFMK-TIS-4102<br>ZFMK-DNA-0100426190	Spain, Malaga, NE of Ronda, Puerto del Viento Blanqilla, N36°47'16" W05°02'47", 1065m, 11-Mai-2013, *Quercus ilex, Crataegus*, sieving, leg. Stüben,P. & Schütte,A., det. Stüben,P.
Echinodera spinosa<br>Stüben, 2006	KJ867605 [new]<br>1288-PST<br>658nt	ZFMK-TIS-4104<br>ZFMK-DNA-0100426414	Spain, Malaga, S of Ronda, Igualeja, N36°37'29" W05°07'25", 672m, 12-Mai-2013, *Ficus carica*, sieving, leg. Stüben,P. & Schütte,A., det. Stüben,P.
Echinodera spinosa<br>Stüben, 2006	MG322654 [new]<br>ES1064<br>658nt	ZFMK-TIS-cES1064<br>ZFMK-DNA-0112704606	Spain, Malaga, S of Ronda, Serrania de Ronda, Igualeja, N36°37'33” W05°07'27”, 647m, 21-Aug-2010, *Ficus carica (Wurzelgesiebe)*, leg. Stüben,P., det. Stüben,P.
Echinodera sp.	MG229700 [new]<br>1394-PST<br>631nt	ZFMK-TIS-4690<br>ZFMK-DNA-0155628528	Italy, Abruzzo, SE of Pescara near Ortona, near the sea, N42°23'40" E14°20'10", 52m, 28-Aug-2013, *Quercus*, old Quercus forest, sieving, leg. Stüben,P., det. Stüben,P.
Echinodera suber<br>Stüben, 2001	GU213688 [01]<br>M-497-sub<br>658nt	ZFMK-TIS-cM497<br>ZFMK-DNA-0100400674	Morocco, 10 km W Sebta (Ceuta), 3 km N of Benzou, N35°53'06" W05°24'06", 330m, 25-Dez-2001, *Quercus suber, Quercus ilex, Pistacia* , leg. Stüben,P., det. Stüben,P.
Echinodera suber<br>Stüben, 2001	GU213757 [01]<br>E-901-sub<br>658nt	ZFMK-TIS-cE901<br>ZFMK-DNA-0100405479	Morocco, Sebta West, vir. Biutz, N35°53'04" W05°24'08", 337m, 09-Mai-2009, *Quercus suber, Smilax, Arbutus*, leg. Stüben,P., det. Stüben,P.
Echinodera tazzekaensis<br>Stüben, 2002	KJ867569 [new]<br>166-PST<br>658nt	ZFMK-TIS-2D100438604<br>ZFMK-DNA-0100438376	Morocco, Atlas Mts., S of Taza, Jebel Tazzeka near Bat-Bou-Idir, N34°05'02" W04°06'49", 1408m, 16-Mai-2011, *Quercus*, sieving, leg. Stüben,P., det. Stüben,P.
Echinodera tellatlasensis<br>Stüben, 2002	KC783813 [new]<br>115-PST<br>609nt	ZFMK-TIS-2D100438642<br>ZFMK-DNA-0100438392	Morocco, S of Berkane, Beni Snassen Mts., Vallee Zegzel, N34°50'27" W02°21'34", 423m, 11-Mai-2011, *Smilax/Pistacea* , sieving, leg. Stüben,P., det. Stüben,P.

**Name**<br>**Authority**<br>**Additional Information**	**GenBank Acc No (Ref.)**<br>**Specimen ID**<br>**Sequence Length**	**ZFMK Tissue ID**<br>**ZFMK DNA Sample ID**<br>**(SDEI DNA Sample ID)**	**Locality, GPS, Collection Date, Plant, Collector, Identifier**
Echinodera tellatlasensis<br>Stüben, 2002	KC783814 [new]<br>140-PST<br>658nt	ZFMK-TIS-2D100438619<br>ZFMK-DNA-0100438358	Morocco, SE of Berkane, N of Aïn-es-Sfa, Aïn Almou, Beni Snassen Mts., N34°50'59" W02°12'05", 1313m, 13-Mai-2011, *Quercus*, sieving, leg. Stüben,P., det. Stüben,P.
Echinodera tenoensis<br>Stüben, 2000	GU213705 [01]<br>E-568-ten<br>658nt	ZFMK-TIS-cE568<br>ZFMK-DNA-0100404289	Spain, Canary Islands, Tenerife, 4 km E of Los Silos, El Tanque, N28°21'39" W16°46'18", 520m, 02-Jan-2004, *Foeniculum vulgare*, leg. Stüben,P., det. Stüben,P.
Echinodera tenoensis<br>Stüben, 2000	GU213718 [01]<br>E-706-ang<br>658nt	ZFMK-TIS-cE706<br>ZFMK-DNA-0100404849	Spain, Canary Islands, Tenerife, E of Los Silos, Teno Mts., El Tanque Bajo, N28°21'39" W16°46'19", 534m, 03-Okt-2008, compost, leg. Astrin,J. & Stüben,P., det. Stüben,P.
Echinodera tenoensis<br>Stüben, 2000	GU988040 [01]<br>E-755-ten<br>658nt	ZFMK-TIS-cE755<br>ZFMK-DNA-0100404716	Spain, Canary Islands, Tenerife, 4 km E of Los Silos, El Tanque, N28°21'39" W16°46'18", 520m, 02-Jan-2004, *Foeniculum vulgare*, compost, leg. Stüben,P., det. Stüben,P.
Echinodera teplovi<br>Savitzki, 1997	MK892361 [new]<br>2755-PST<br>658nt	ZFMK-TIS-26852<br>ZFMK-DNA-0171661898	Russia, Krasnodar, Chvizhepse vill. env. , N43°38'32" E40°04'45", 300m, 11-Jul-2014, leg. Kovalev,A.V., det. Stüben,P.
Echinodera valida<br>(Hampe 1864)	MK347567 [new]<br>2991-PST<br>658nt	(SDEI-DNA-2991-PST)	Greece, Macedonia, N Neo Petritsi (near Serres), N41°19'29" E23°16'06", 861m, 15-Jul-2017, *Fagus*, leg. Stüben,P., det. Stüben,P.
Echinodera variegata<br>(Boheman, 1837)	EU286481 [01]<br>I-0154-vit<br>658nt	ZFMK-TIS-cI0154<br>ZFMK-DNA-0100400095	Italy, Sicilia Isl. (TP), 26 km NE of Trapani, SE of Capo San Vito, N38°09'00" E12°46'19", 35m, 14-Okt-2006, *Quercus, Fraxinus*, limestone cave, leg. Stüben,P., det. Stüben,P.
Echinodera variegata<br>(Boheman, 1837)	GU213659 [01]<br>I-0411-var<br>658nt	ZFMK-TIS-cI0411<br>ZFMK-DNA-0100400030	Italy, Sicilia Isl. (PA), 10 km NW of Petralia Soprana, P.R. Madonie, N37°50'29" E14°00'39", 1000m, 21-Jul-2005, *Quercus ilex*, leg. Stüben,P., det. Stüben,P.
Echinodera variegata<br>(Boheman, 1837)	GU213660 [01]<br>I-0412-var<br>658nt	ZFMK-TIS-cI0412<br>ZFMK-DNA-0100400007	Italy, Sicilia Isl. (PA), 8 km S of Carini, W of M. Gibilmesi, N38°04'03" E13°11'37", 539m, 10-Okt-2006, *Quercus*, leg. Stüben,P., det. Stüben,P.
Echinodera varroi<br>Stüben, 2018<br>**Paratype (DNAtype)**	MF464648 [34]<br>1401-PST_4697<br>658nt	ZFMK-TIS-4697<br>ZFMK-DNA-0155628535	Italy, Lazio, NW of San Donato Val di Camino, N of Alvito/S. Onófrio, Valle di Rio, N41°43'24" E13°44'41", 671m, 27-Aug-2013, mixed forest, sieving, leg. Stüben,P., det. Stüben,P.
Echinodera varroi<br>Stüben, 2018<br>**Paratype (DNAtype)**	MF464649 [34]<br>1769-PST_24093<br>658nt	ZFMK-TIS-24093<br>ZFMK-DNA-0171624095	Italy, Lazio, above S. Donato Val Di Comino, N41°42'37" E13°48'39", 756m, 05-Mai-2014, *Carpinus*, sieving, leg. Stüben,P. & Schütte,A., det. Stüben,P.
Echinodera varroi<br>Stüben, 2018<br>**Paratype (DNAtype)**	MF464650 [34]<br>1775-PST_24099<br>658nt	ZFMK-TIS-24099<br>ZFMK-DNA-0171624089	Italy, Lazio, Cassino, Terme Varroniane, N41°28'59" E13°49'52", 47m, 06-Mai-2014, Laurus forest, sieving, leg. Stüben,P. & Schütte,A., det. Stüben,P.
Echinodera zaghouanensis<br>Stüben, 2004<br>**Paratype (DNAtype)**	GU213689 [01]<br>T-498-zag<br>658nt	ZFMK-TIS-cT498<br>ZFMK-DNA-0100400673	Tunisia, Dorsale, Jebel Zaghouan, 6 km S of Zaghouan, N36°21'55" E10°06'30", 650m, 28-Okt-2003, *Ceratonia*, limestone, leg. Stüben,P., det. Stüben,P.
Echinosomidia porcellus<br>(Wollaston, 1854)	MK891721 [40]<br>1951-PST<br>658nt	ZFMK-TIS-24030<br>ZFMK-DNA-0169170398	Portugal, Madeira, SE of Ribeira da Janela, Fanal, N32°48'48" W17°08'57", 1084m, 05-Okt-2014, *Ocotea foetens*, sieving, leg. Stüben,P., det. Stüben,P.
Echiumacalles anagaensis<br>(Stüben, 2000)	GU987925 [01]<br>E-545-ana<br>658nt	ZFMK-TIS-cE545<br>ZFMK-DNA-0100404998	Spain, Canary Islands, La Gomera, 2.5 km E of Hermigua, N28°09'30" W17°09'46", 650m, 31-Dez-2004, *Echium strictum*, cliff, thermophilic brushwood, leg. Stüben,P., det. Stüben,P.

**Name**<br>**Authority**<br>**Additional Information**	**GenBank Acc No (Ref.)**<br>**Specimen ID**<br>**Sequence Length**	**ZFMK Tissue ID**<br>**ZFMK DNA Sample ID**<br>**(SDEI DNA Sample ID)**	**Locality, GPS, Collection Date, Plant, Collector, Identifier**
Echiumacalles anagaensis<br>(Stüben, 2000)	KF672594 [12]<br>ES1016<br>658nt	ZFMK-TIS-cES1016<br>ZFMK-DNA-0112704654	Spain, Canary Islands, La Gomera, E of Hermigua, El Palmar, 28°09'26"N 17°09'47"W, 627m, 30-Dez-2009, *Echium strictum*, thermophilic brushwood, leg. Stüben,P., det. Stüben,P.
Echiumacalles anagaensis<br>(Stüben, 2000)	FJ716565 [01]<br>E-686-ana<br>658nt	ZFMK-TIS-cE686<br>ZFMK-DNA-0100404869	Spain, Canary Islands, Tenerife, NE of La Laguna, Anaga Mts., Las Bodegas, N28°33'42" W16°09'25", 502m, 30-Sep-2008, *Echium strictum*, leg. Astrin,J. & Stüben,P., det. Stüben,P.
Echiumacalles anagaensis<br>(Stüben, 2000)	GU987926 [01]<br>E-546-ana<br>658nt	ZFMK-TIS-cE546<br>ZFMK-DNA-0100404997	Spain, Canary Islands, Tenerife, East Anaga Mts., 19 km NE of La Laguna, Lomo de las Bodegas, N28°33'43" W16°09'25", 500m, 22-Dez-2003, *Sonchus acaulis, Kleinia, Echium strictum*, leg. Stüben,P., det. Stüben,P.
Elescus bipunctatus<br>(Linnaeus, 1758)	KC783979 [new]<br>268-PSP<br>658nt	ZFMK-TIS-2D100438514<br>ZFMK-DNA-0100439523	Germany, Lower Saxony (NI), Helstorfer Moor, Resse, Hannover region, N52°31'44" E09°36'32", 43m, 14-Mai-2011, *Salix aurita*, bog forest, beating, leg. Sprick,P., det. Sprick,P.
Ellescus infirmus<br>(Herbst, 1795)	MK891000 [new]<br>893-CBR<br>658nt	ZFMK-TIS-3806<br>ZFMK-DNA-0100449715	Luxembourg, 10 km E of Luxembourg, 0.5 km W of Uebersyren, N49°38'19" E06°16'31", 240m, 19-Apr-2011, *Salix (fragilis)*, beating, leg. Braunert,C., det. Braunert,C.
Elliptacalles baeticus<br>(Stüben, 2008)	GU988131 [01]<br>E-885-lon<br>658nt	ZFMK-TIS-cE885<br>ZFMK-DNA-0100405511	Morocco, High Atlas, SE of Asni, Tacheddirt (near Imlil), N31°09'07" W07°50'18", 2420m, 02-Mai-2009, broom, *Daphne*, leg. Stüben,P., det. Stüben,P.
Elliptacalles baeticus<br>(Stüben, 2008)	EU286499 [01]<br>E-0198-lon<br>658nt	ZFMK-TIS-cE0198<br>ZFMK-DNA-0100400121	Spain, Cadiz, 2.5 km N of Ubrique, Sierra de Ubrique, N36°41'50" W05°26'20", 646m, 10-Apr-2007, *Quercus ilex, Ceratonia, Olea, Pistacia lentiscus*, leg. Astrin,J. & Stüben,P., det. Stüben,P.
Elliptacalles baeticus<br>(Stüben, 2008)	EU286501 [01]<br>E-0213-lon<br>658nt	ZFMK-TIS-cE0213<br>ZFMK-DNA-0100400083	Spain, Malaga, 16 km N of Estepona, Sierra Bermeja, Jubrique, N36°33'49" W05°12'27", 557m, 13-Apr-2007, *Quercus suber*, leg. Astrin,J. & Stüben,P., det. Stüben,P.
Elliptacalles baeticus<br>(Stüben, 2008)	GU988060 [01]<br>E-768-bae<br>658nt	ZFMK-TIS-cE768<br>ZFMK-DNA-0100405024	Spain, Malaga, W of Estepona, N36°25'58" W05°14'42", 189m, 13-Jan-2009, *Quercus, Ceratonia, Pistacia*, stream, leg. Stüben,P., det. Stüben,P.
Elliptacalles baeticus<br>(Stüben, 2008)	MG322648 [new]<br>ES1047<br>658nt	ZFMK-TIS-cES1047<br>ZFMK-DNA-0112704622	Spain, Cadiz, Los Barrios, Parque Natural Alcarnocales, Pista Zanana donde aparco, en la parte Alta, 36°22'52.5"N 05°59'29.5"W, 140m, 25-Nov-2009, *Quercus suber, cribando Bajo el Quercus*, leg. Torres,J. L., det. Torres,J. L.
Elliptacalles cf. *longus*<br>(Desbrochers des Loges, 1892)	EU286458 [01]<br>I-0096-lon<br>658nt	ZFMK-TIS-cI0096<br>ZFMK-DNA-0100400942	Italy, Sicilia Isl. (PA), 6 km SW of Carini, Montagna Longa, N38°07'10" E13°08'31", 647m, 09-Okt-2006, *Quercus ilex*, leg. Stüben,P., det. Stüben,P.
Elliptacalles longus<br>(Desbrochers des Loges, 1892)	MG229821 [new]<br>2860-PST<br>658nt	ZFMK-TIS-4255<br>ZFMK-DNA-FC17941551	Greece, Cyclades Islands, Andros Island, 2-3 km W of Vourkoti village, N37°51'42" E24°51'40", 750m, 16-Feb-2016, Crataegus forest, in dry ferns, collecting by hand, leg. Vandoros,G., det. Stüben,P.
Elliptacalles longus<br>(Desbrochers des Loges, 1892)	MG229686 [new]<br>780-PST<br>658nt	ZFMK-TIS-3117<br>ZFMK-DNA-0155630422	Italy, Calabria, Cosenza/Grisolia, N39°43'32" E15°53'45", 656m, 01-Jun-2011, *Castanea, Quercus*, leg. Michele,T., det. Stüben,P.
Entomoderus brevitarsis<br>(Wollaston, 1864)	MK347530 [40]<br>2927-PST<br>658nt	(SDEI-DNA-2927-PST)	Spain, Canary Islands, Lanzarote, Calle La Rositas, Finca La Corona, N29°11'48" W13°29'33", 354m, 04-Jan-2017, sifting, leg. Stüben,P., det. Stüben,P.
Eubrychius velutus<br>(Beck, 1817)	MK890849 [new]<br>368-JKR<br>658nt	ZFMK-TIS-20154<br>ZFMK-DNA-0100438715	Czech Republic, Bohemia or., Steblova, Oplatil pond, N50°6'15.584" E15°44'42.978", 230m, 29-Jun-2011, *Myriophyllum spicatum*, hand-collecting, leg. Krátky,J., det. Krátky,J.

**Name**<br>**Authority**<br>**Additional Information**	**GenBank Acc No (Ref.)**<br>**Specimen ID**<br>**Sequence Length**	**ZFMK Tissue ID**<br>**ZFMK DNA Sample ID**<br>**(SDEI DNA Sample ID)**	**Locality, GPS, Collection Date, Plant, Collector, Identifier**
Eubrychius velutus<br>(Beck, 1817)	MK890890 [new]<br>567-PSP<br>658nt	ZFMK-TIS-20352<br>ZFMK-DNA-0100448412	Germany, Lower Saxony (NI), Salzgitter-Engerode, N52°04'56" E10°22'35", 152m, 24-Jun-2011, *Myriophyllum heterophyllum*, body of standing water, water dipnet, leg. Sprick,P. & Wimmer,W., det. Sprick,P.
Eucoeliodes mirabilis<br>(A. Villa & G. B. Villa, 1835)	MK891911 [new]<br>2179-JKR<br>658nt	ZFMK-TIS-23917<br>ZFMK-DNA-0169170603	Czech Republic, Moravia, NP Podyji, Hnanice, N48°48'01" E15°58'25", 300m, 30-Mai-2014, *Euonymus europaeus*, beating, leg. Stejskal,R., det. Stejskal,R.
Eurhoptus sp.	MK892433 [new]<br>X-0425-EuS<br>658nt	ZFMK-TIS-24598	Mexico, Chiapas, 13.7 km NW of Metzabok, , 540m, 14-Jul-2007, leg. Longino,J. with Anderson,R., det. Longino,J. with Anderson,R.
Eurhoptus sp.	MK892434 [new]<br>X-0430-EuS<br>658nt	ZFMK-TIS-24615<br>ZFMK-DNA-0155630418	Mexico, Chiapas, 8.8km SE of Salto de Agua, N17°30' W92°17', 50m, 14-Jul-2007, leg. Branstetter,M. with Anderson,R., det. Branstetter,M. and Anderson,R.
Euscepes batatae<br>G. R. Waterhouse, 1849<br>formerly: *E. postfasciatus*-Syn!	MG229802 [40]<br>2771-PST<br>658nt	-TIS-26125<br>ZFMK-DNA-0169166971	Portugal, Madeira, W of Funchal, Sito das Pocas, N32°38'01" W16°56'37", 15m, 19-Mrz-2015, ruderal area, sieving, leg. Stüben,P., det. Stüben,P.
Euscepes batatae<br>G. R. Waterhouse, 1849<br>formerly: *E. postfasciatus*-Syn!	MG229809 [40]<br>2789-PST<br>658nt	ZFMK-TIS-26143<br>ZFMK-DNA-0169166962	Portugal, Madeira, E of Funchal, W of Garajau, Rua do Lazareto, N32°38'49" W16°53'17", 37m, 03-Apr-2015, *Musa*, sieving, leg. Stüben,P., det. Stüben,P.
Eusomus ovulum<br>Germar, 1824	MK890878 [new]<br>498-RST<br>648nt	ZFMK-TIS-20283<br>ZFMK-DNA-0100448481	Czech Republic, Moravia mer., Pusty kopec u Konic Nature Monument, 1.6 km E of Znojmo-Popice, N48°48'45.74" E16°2'17.33", 260m, 18-Mai-2011, sweeping, leg. Stejskal,R., det. Stejskal,R.
Eusomus ovulum<br>Germar, 1824	MK891796 [new]<br>2055-JKR<br>658nt	ZFMK-TIS-23695<br>ZFMK-DNA-0169170446	Czech Republic, Bohemia, Kosatky, N50°19'23" E14°39'58", 218m, 02-Mai-2014, sieving, leg. Krátky,J., det. Krátky,J.
Eusomus ovulum<br>Germar, 1824	MK890833 [new]<br>289-PSP<br>658nt	ZFMK-TIS-2D100438558<br>ZFMK-DNA-0100439759	Germany, Hesse (HE), Wiesbaden-Schierstein, N50°02'58" E08°12'28", 283m, 31-Mai-2011, *Silene latifolia* ssp. *alba, Cirsium arvense, Carduus acanthoides, Achillea millefolium*, dipnet, leg. Sprick,P., det. Sprick,P.
Eusomus ovulum<br>Germar, 1824	MK890932 [new]<br>801-PST<br>658nt	ZFMK-TIS-3714<br>ZFMK-DNA-0100414295	Germany, Rhineland-Palatinate (RLP), E of Treis-Karden, Pommern, Pommerner Goldberg, high road (vineyard), N50°10'14" E07°16'29", 95m, 10-Jun-2012, beating, leg. Stüben,P., det. Stüben,P.
Eusomus ovulum<br>Germar, 1824	MK892282 [new]<br>2583-PSP<br>658nt	ZFMK-TIS-23557<br>ZFMK-DNA-0171661971	Germany, Bavaria (BY), Unterfranken, Ebern, N50°05'18.5" E10°46'02", 328m, 13-Jun-2014, *Artemisia vulgaris*, leg. Sprick,P., det. Sprick,P.
Eusomus ovulum<br>Germar, 1824	MK891703 [new]<br>1921-FBA<br>658nt	ZFMK-TIS-23312<br>ZFMK-DNA-0169170369	Greece, East Macedonia and Thrace, Drama, Exochi, N41°24'25" E23°49'28", 630m, 13-Aug-2014, leg. Bayer & Brunner, det. Bayer,F.
Eusomus ovulum<br>Germar, 1824	KC784180 [new]<br>394-RGO<br>658nt	ZFMK-TIS-20180<br>ZFMK-DNA-0100449118	Poland, Katy 2, N50°40'37" E23°07'43", 270m, 30-Jun-2011, leg. Gosik,R., det. Gosik,R.
Eusomus ovulum<br>Germar, 1824	MK890860 [new]<br>415-RGO<br>658nt	ZFMK-TIS-20201<br>ZFMK-DNA-0100449433	Poland, Tarnogóra, N50°41'11.6" E23°07'13.4", 249m, 11-Jun-2011, leg. Gosik,R., det. Gosik,R.

**Name**<br>**Authority**<br>**Additional Information**	**GenBank Acc No (Ref.)**<br>**Specimen ID**<br>**Sequence Length**	**ZFMK Tissue ID**<br>**ZFMK DNA Sample ID**<br>**(SDEI DNA Sample ID)**	**Locality, GPS, Collection Date, Plant, Collector, Identifier**
Eutrichapion ervi<br>(Kirby, 1808)	MK891447 [new]<br>1512-PSP<br>658nt	ZFMK-TIS-3272<br>ZFMK-DNA-0155633317	Denmark, Syddanmark, Emmerlev Klev (Højer), N54°59'53" E08°39'08", 3m, 30-Aug-2012, *Lathyrus pratensis*, dipnet, leg. Sprick,P., det. Sprick,P.
Eutrichapion ervi<br>(Kirby, 1808)	MK891434 [new]<br>1497-PSP<br>658nt	ZFMK-TIS-3257<br>ZFMK-DNA-0155633335	Germany, Saxony-Anhalt (ST), Harz National Park, Schierke, Feuersteinwiesen, N51°45'24" E10°41'40", 591m, 01-Aug-2012, *Lathyrus pratensis*, beating, leg. Sprick,P., det. Sprick,P.
Eutrichapion ervi<br>(Kirby, 1808)	MK892292 [new]<br>2593-PSP<br>658nt	ZFMK-TIS-23567<br>ZFMK-DNA-0171661966	Germany, Bavaria (BY), Unterfranken, Ebern, N50°05'20.5" E10°45'59", 333m, 13-Jun-2014, *Lathyrus pratensis*, leg. Sprick,P., det. Sprick,P.
Eutrichapion punctiger<br>(Paykull, 1792)	MK891404 [new]<br>1466-PSP<br>658nt	ZFMK-TIS-3226<br>ZFMK-DNA-0155633352	Germany, Lower Saxony (NI), Harz, St. Andreasberg, Jordanshöhe, N51°42'58" E10°32'08", 674m, 02-Jul-2012, *Vicia cracca*, mountain meadow, dipnet, leg. Sprick,P., det. Sprick,P.
Eutrichapion punctiger<br>(Paykull, 1792)	MK892305 [new]<br>2606-PSP<br>658nt	ZFMK-TIS-23580<br>ZFMK-DNA-0171661946	Germany, Bavaria (BY), Unterfranken, Ebern, N50°05'09" E10°45'53", 319m, 14-Jun-2014, *Vicia sepium*, leg. Sprick,P., det. Sprick,P.
Eutrichapion viciae<br>(Paykull, 1800)	MK891456 [new]<br>1521-PSP<br>658nt	ZFMK-TIS-3281<br>ZFMK-DNA-0155633311	Denmark, Syddanmark, Emmerlev Klev (Højer), N54°59'16" E08°39'34", 3m, 30-Aug-2012, *Vicia cracca*, meadow near coast, leg. Sprick,P., det. Sprick,P.
Eutrichapion viciae<br>(Paykull, 1800)	KC784051 [new]<br>579-PSP<br>658nt	ZFMK-TIS-20363<br>ZFMK-DNA-0100448393	Germany, Bavaria (BY), Buckenhof-Uttenreuth, Lkr. Erlangen-Höchstadt, N49°35'23" E11°04'28", 295m, 03-Jun-2011, *Vicia villosa*, edge of field, dipnet, leg. Sprick,P., det. Sprick,P.
Eutrichapion viciae<br>(Paykull, 1800)	KC784240 [new]<br>IT-0007w<br>658nt	ZFMK-DNA-0100437868	Italy, Sardinia Isl. East, SW of Siniscola, Monte Albo, N40°32'07" E09°36'13", 748m, 26-Sep-2010, *Quercus ilex*, limestone, leg. Stüben,P., det. Stüben,P.
Eutrichapion vorax<br>(Herbst, 1797)	MK892125 [new]<br>2403-JKR<br>658nt	ZFMK-TIS-25980<br>ZFMK-DNA-0171600634	Czech Republic, Moravia, Lanzhot, Pohansko, N48°41'27" E16°55'58", 153m, 14-Aug-2014, *Galega officinalis*, sweeping, leg. Krátky,J., det. Krátky,J.
Eutrichapion vorax<br>(Herbst, 1797)	MK891351 [new]<br>1395-PST<br>658nt	ZFMK-TIS-4691<br>ZFMK-DNA-0155627277	Italy, Lazio, NW of San Donato Val di Camino, N of Alvito, N41°44'20" E13°44'29", 1051m, 26-Aug-2013, *Verbascum, Scrophularia*, meadow, beating, leg. Stüben,P., det. Stüben,P.
Eutrichapion vorax<br>(Herbst, 1797)	KC783785 [40]<br>76-PST<br>648nt	ZFMK-TIS-2D100446972<br>ZFMK-DNA-0100438044	Spain, Canary Islands, La Gomera, Arure, N28°08'23" W17°18'29", 801m, 13-Feb-2011, *Vicia*, creek, beating, leg. Stüben,P., det. Stüben,P.
Eutrichapion vorax<br>(Herbst, 1797)	MK890971 [new]<br>856-PST<br>658nt	ZFMK-TIS-3769<br>ZFMK-DNA-0100414235	Portugal, Alentejo, Serra da Arràbida (west, coast), N38°28'03" W08°59'30", 34m, 23-Mai-2012, beating, leg. Stüben,P., det. Stüben,P.
Exapion cf. *compactum deubeli*	KC784096 [new]<br>521-RST<br>658nt	ZFMK-TIS-20306<br>ZFMK-DNA-0100448458	Romania, Caras-Severin, Sfanta Elena env., N44°42'12.46" E21°42'33.45", 450m, 28-Jul-2011, *Genista sagittata*, beating, leg. Stejskal,R., det. Stüben,P.
Exapion cf. *subparallelum*	KC783873 [new]<br>135-PST<br>610nt	ZFMK-TIS-2D100440987<br>ZFMK-DNA-0100438180	Morocco, S of Berkane, N of Aïn-es-Sfa, Beni Snassen Mts., N34°49'54" W02°08'49", 985m, 13-Mai-2011, *Spartium*, beating, leg. Stüben,P., det. Behne,L.
Exapion compactum compactum<br>(Desbrochers, 1888)	MK890973 [new]<br>864-PST<br>658nt	ZFMK-TIS-3777<br>ZFMK-DNA-0100414227	Germany, Rhineland-Palatinate (RLP), Altenahr, Teufelsloch, N50°30'32" E06°59'30", 193m, 24-Jul-2012, *Geista pilosa*, sieving, leg. Stüben,P., det. Stüben,P.

**Name**<br>**Authority**<br>**Additional Information**	**GenBank Acc No (Ref.)**<br>**Specimen ID**<br>**Sequence Length**	**ZFMK Tissue ID**<br>**ZFMK DNA Sample ID**<br>**(SDEI DNA Sample ID)**	**Locality, GPS, Collection Date, Plant, Collector, Identifier**
Exapion compactum<br>(Desbrochers, 1888)	MK892079 [new]<br>2355-JKR<br>658nt	ZFMK-TIS-25915<br>ZFMK-DNA-0171600586	Czech Republic, Moravia, Znojmo-Popice, N48°49'37" E16°01'11", 300m, 16-Aug-2014, *Genista pilosa*, sweeping, leg. R. Stejskal, det. Stejskal,R.
Exapion corniculatum<br>(Germar, 1817)	KC784099 [new]<br>517-RST<br>658nt	ZFMK-TIS-20302<br>ZFMK-DNA-0100448462	Czech Republic, Bohemia bor., Bile strane National Nature Monument, 2.5 km N of Litomerice, N50°33'32.39" E14°7'58.09", 340m, 05-Jul-2011, beating, leg. Stejskal,R., det. Stejskal,R.
Exapion difficile<br>(Herbst, 1797)	MK892086 [new]<br>2362-JKR<br>658nt	ZFMK-TIS-25922<br>ZFMK-DNA-0171600579	Czech Republic, Moravia, NP Podyji, Cizov, Siroke pole, N48°51'36" E15°51'02", 300m, 17-Aug-2014, *Genista tinctoria*, sweeping, leg. R. Stejskal, det. Stejskal,R.
Exapion difficile<br>(Herbst, 1797)	MK891427 [new]<br>1490-PSP<br>658nt	ZFMK-TIS-3250<br>ZFMK-DNA-0155633328	Germany, Saxony-Anhalt (ST), Harz National Park, Ilsenburg, Ilsestein, Genisto-Quercetum, N51°50'49" E10°39'40", 405m, 01-Aug-2012, *Genista tinctoria*, beating, leg. Sprick,P., det. Sprick,P.
Exapion difficile<br>(Herbst, 1797)	MK891462 [new]<br>1529-PSP<br>658nt	ZFMK-TIS-3289<br>ZFMK-DNA-0155633300	Germany, Lower Saxony (NI), Wesseln, Lkr. Hildesheim, Steinberg National Park, N52°05'04" E10°01'00", 191m, 20-Okt-2012, *Genista tinctoria*, dipnet, leg. Sprick,P., det. Sprick,P.
Exapion difficile<br>(Herbst, 1797)	MK892273 [new]<br>2574-PSP<br>658nt	ZFMK-TIS-23548<br>ZFMK-DNA-0171661985	Germany, Bavaria (BY), Unterfranken, Ebern, N50°05'20.5" E10°45'59", 333m, 13-Jun-2014, *Genista tinctoria*, leg. Sprick,P., det. Sprick,P.
Exapion formaneki<br>(Wagner, 1929)	MK892272 [new]<br>2572-PSP<br>658nt	ZFMK-TIS-23546<br>ZFMK-DNA-0171661987	Germany, Bavaria (BY), Unterfranken, Ebern, N50°05'20.5" E10°45'59", 333m, 13-Jun-2014, *Genista tinctoria*, leg. Sprick,P., det. Sprick,P.
Exapion formaneki<br>(Wagner, 1929)	MK891043 [new]<br>942-CBR<br>658nt	ZFMK-TIS-3855<br>ZFMK-DNA-0100449668	Luxembourg, 10 km E of Luxembourg, 1 km SW of Mensdorf, N49°39'32" E06°18'35", 300m, 09-Jun-2012, *Genista tinctoria*, sweeping, leg. Braunert,C., det. Braunert,C.
Exapion fuscirostre<br>(Fabricius, 1775)	MK892104 [new]<br>2381-JKR<br>658nt	ZFMK-TIS-25958<br>ZFMK-DNA-0171600608	Czech Republic, Moravia, Znojmo-Popice, N48°49'11" E16°00'38", 310m, 28-Aug-2014, *Cytisus scoparius*, sweeping, leg. R. Stejskal, det. Stejskal,R.
Exapion fuscirostre flachi<br>(Wagner, 1906)	MK890952 [new]<br>831-PST<br>658nt	ZFMK-TIS-3744<br>ZFMK-DNA-0100414260	Portugal, Minho, PN of Peneda-Geres near Soajo, N41°52'14" W08°16'30", 200m, 09-Mai-2012, *Sarothamnus*, beating, leg. Stüben,P., det. Stüben,P.
Exapion fuscirostre fuscirostre<br>(Fabricius, 1775)	KC784089 [new]<br>538-RST<br>658nt	ZFMK-TIS-20323<br>ZFMK-DNA-0100448448	Czech Republic, Bohemia occ., Belysov Nature Reserve, 2.5 km SE of Chudenice, N49°26'49.54" E13°11'53.68", 540m, 28-Aug-2011, *Cytisus scoparius*, beating, leg. Stejskal,R., det. Stejskal,R.
Exapion fuscirostre fuscirostre<br>(Fabricius, 1775)	KC784025 [new]<br>217-PSP<br>658nt	ZFMK-TIS-2D100439159<br>ZFMK-DNA-0100439831	Germany, Lower Saxony (NI), Brelingen, Hannover region, N52°33'35" E09°41'04", 70m, 16-Apr-2011, *Cytisus scoparius*, sand pit, beating, leg. Sprick,P., det. Sprick,P.
Exapion fuscirostre fuscirostre<br>(Fabricius, 1775)	KC784182 [new]<br>478-PST<br>658nt	ZFMK-TIS-20263<br>ZFMK-DNA-0100449352	Germany, Rhineland-Palatinate (RLP), Mosellus area, Kobern-Gondorf, "Sürzer Höfe", ruderal area, N50°20'20" E07°25'25", 330m, 30-Sep-2011, *Cytisus scoparius*, hand-collecting, leg. Stüben,P., det. Stüben,P.
Exapion fuscirostre<br>(Fabricius, 1775)	MK891688 [new]<br>1905-PST<br>658nt	ZFMK-TIS-24010<br>ZFMK-DNA-0169170409	Germany, North Rhine-Westphalia (NRW), lower Rhine, 2 km S of Brüggen, Elmpter creek, N51°13'24" E06°11'31", 57m, 03-Sep-2014, *Cytisus scoparius*, beating, leg. Stüben,P., det. Stüben,P.
Exapion fuscirostre<br>(Fabricius, 1775)	MK891010 [new]<br>904-CBR<br>658nt	ZFMK-TIS-3817<br>ZFMK-DNA-0100449707	Luxembourg, 10 km NE of Mersch, 2 km NE of Nommern, N49°48'15" E06°11'16", 340m, 01-Mai-2011, *Cytisus scoparius*, beating, leg. Braunert,C., det. Braunert,C.

**Name**<br>**Authority**<br>**Additional Information**	**GenBank Acc No (Ref.)**<br>**Specimen ID**<br>**Sequence Length**	**ZFMK Tissue ID**<br>**ZFMK DNA Sample ID**<br>**(SDEI DNA Sample ID)**	**Locality, GPS, Collection Date, Plant, Collector, Identifier**
Exapion sp.	KC784169 [new]<br>490-PST<br>607nt	ZFMK-TIS-20275<br>ZFMK-DNA-0100449041	France, Vaucluse, Sault, Gorges de la Nesque, N44°03'17" E05°17'38", 522m, 01-Aug-2011, broom, beating, leg. Stüben,P., det. Stüben,P.
Exapion uliciperda<br>(Pandellé, 1867)	MK891654 [40]<br>1834-PST<br>658nt	ZFMK-TIS-26037<br>ZFMK-DNA-0171606132	Portugal, Madeira, SW of Santana, Achada do Teixeira, N32°45'51" W16°55'12", 1589m, 28-Jun-2014, *Ulex europaeus*, beating, leg. Stüben,P., det. Stüben,P.
Exapion uliciperda<br>(Pandellé, 1867)	MK891656 [40]<br>1844-PST<br>658nt	ZFMK-TIS-26047<br>ZFMK-DNA-0171606129	Portugal, Madeira, SE of Ribeira da Janela, Fanal, N32°48'48" W17°08'57", 1084m, 29-Jun-2014, *Ulex europaeus*, beating, leg. Stüben,P., det. Stüben,P.
Exapion uliciperda<br>(Pandellé, 1867)	MK891717 [40]<br>1944-PST<br>658nt	ZFMK-TIS-24023<br>ZFMK-DNA-0169170405	Portugal, Madeira, Faial, coast, N32°47'37" W16°50'57", 26m, 04-Okt-2014, sieving, leg. Stüben,P., det. Stüben,P.
Exapion uliciperda<br>Pandellé, 1867	MK890958 [new]<br>838-PST<br>658nt	ZFMK-TIS-3751<br>ZFMK-DNA-0100414253	Portugal, Minho, Serra d'Arga, Cerquido, N41°48'24" W08°40'22", 348m, 11-Mai-2012, *Ulex*, beating, leg. Stüben,P., det. Stüben,P.
Exomias araneiformis<br>(Schrank, 1781)	MK891087 [new]<br>990-PSP<br>658nt	ZFMK-TIS-3135<br>ZFMK-DNA-0100426868	Germany, Lower Saxony (NI), Stadthagen, Bruchhof, N52°18'12" E09°10'46", 90m, 30-Apr-2012, *Acer pseudoplatanus, juv.*, moderate wet broad-leaved forest, sieving, leg. Sprick,P., det. Sprick,P.
Exomias mollicomus<br>(Ahrens, 1812)	MK891793 [new]<br>2052-JKR<br>658nt	ZFMK-TIS-23692<br>ZFMK-DNA-0169170449	Czech Republic, Bohemia, Kosatky, N50°19'23" E14°39'58", 218m, 02-Mai-2014, sieving, leg. Krátky,J., det. Krátky,J.
Exomias mollicomus<br>(Ahrens, 1812)	MK891862 [new]<br>2125-JKR<br>658nt	ZFMK-TIS-23765<br>ZFMK-DNA-0169169608	Slovakia, Nitra, Zobor Mt., N48°21'08.5 E18°05'41", 427m, 24-Mai-2014, sieving, leg. Krátky,J., det. Krátky,J.
Exomias pellucidus<br>(Boheman, 1843)	KC783961 [new]<br>321-JKR<br>658nt	ZFMK-TIS-20107<br>ZFMK-DNA-0100438923	Czech Republic, Bohemia or., Dvakacovice, N49°58'35.447" E15°54'2.243", 245m, 14-Mai-2011, sifting, leg. Krátky,J., det. Krátky,J.
Exomias pellucidus<br>(Boheman, 1843)	MK891813 [new]<br>2073-JKR<br>658nt	ZFMK-TIS-23713<br>ZFMK-DNA-0169169660	Czech Republic, Bohemia, Ujezd u Sezemic, N50°07'07.5" E15°51'37", 234m, 08-Mai-2014, sweeping, leg. Krátky,J., det. Krátky,J.
Exomias pellucidus<br>(Boheman, 1843)	MK891820 [new]<br>2080-JKR<br>658nt	ZFMK-TIS-23720<br>ZFMK-DNA-0169168583	Czech Republic, Bohemia, Predhradi, N49°49'56" E16°02'33", 427m, 08-Mai-2014, sieving, leg. Krátky,J., det. Krátky,J.
Exomias pellucidus<br>(Boheman, 1843)	KC784011 [new]<br>243-PSP<br>658nt	ZFMK-TIS-2D100439137<br>ZFMK-DNA-0100439809	Germany, Lower Saxony (NI), Nordstemmen, Lkr. Hildesheim, N52°09' E09°47', 72m, 04-Mai-2011, *Filipendula ulmaria*, garden, beating, leg. Sprick,P., det. Sprick,P.
Exomias pellucidus<br>(Boheman, 1843)	MK890943 [new]<br>813-PST<br>658nt	ZFMK-TIS-3726<br>ZFMK-DNA-0100414278	Germany, Rhineland-Palatinate (RLP), N of Treis-Karden, Brohl, N50°13'22" E07°16'27", 255m, 10-Jun-2012, roadside ditch, beating, leg. Stüben,P., det. Stüben,P.
Exomias pellucidus<br>(Boheman, 1843)	MK892172 [new]<br>2464-PSP<br>658nt	ZFMK-TIS-23436<br>ZFMK-DNA-0171661802	Germany, Saxony-Anhalt (ST), Harz National Park, Ilsenburg, Wienberg, N51°52'16" E10°39'57", 292m, 29-Mai-2013, *Fagus sylvatica*, beating, leg. Sprick,P., det. Sprick,P.
Exomias pellucidus<br>(Boheman, 1843)	MK892293 [new]<br>2594-PSP<br>658nt	ZFMK-TIS-23568<br>ZFMK-DNA-0171661965	Germany, Bavaria (BY), Unterfranken, Ebern, N50°05'20.5" E10°45'59", 333m, 13-Jun-2014, *herb layer*, leg. Sprick,P., det. Sprick,P.

**Name**<br>**Authority**<br>**Additional Information**	**GenBank Acc No (Ref.)**<br>**Specimen ID**<br>**Sequence Length**	**ZFMK Tissue ID**<br>**ZFMK DNA Sample ID**<br>**(SDEI DNA Sample ID)**	**Locality, GPS, Collection Date, Plant, Collector, Identifier**
Exomias trichopterus<br>(Gautier des Cottes, 1863)	MK891130 [new]<br>1035-PSP<br>658nt	ZFMK-TIS-3180<br>ZFMK-DNA-0100426817	Germany, Lower Saxony (NI), Hannover, Herrenhausen, "Berggarten" area, N52°23'42" E09°42'00", 56m, 23-Mai-2012, sieving, leg. Sprick,P., det. Sprick,P.
Exomias trichopterus<br>(Gautier des Cottes, 1863)	MK892266 [new]<br>2566-PSP<br>658nt	ZFMK-TIS-23540<br>ZFMK-DNA-0171662002	Germany, Saxony-Anhalt (ST), Harz National Park, Ilsenburg, Wienberg, N51°52'15" E10°39'58", 290m, 10-Jun-2014, *Fagus sylvatica*, leg. Sprick,P., det. Sprick,P.
Exomias vallestris<br>Hampe, 1870	MK890880 [new]<br>508-RST<br>630nt	ZFMK-TIS-20293<br>ZFMK-DNA-0100448466	Czech Republic, Moravia mer., 1.2 km NE of Krtiny near Brno, N49°18'26.99" E16°45'7.43", 450m, 05-Mai-2011, *Larix-Picea forest*, sieving, leg. Stejskal,R., det. Stejskal,R.
Ficusacalles ficvorator<br>(Stüben, 2007)<br>formerly: *F. senilis ficvorator*	JN701889 [16]<br>53-PST<br>651nt	ZFMK-TIS-2D100446889<br>ZFMK-DNA-0100438034	Spain, Canary Islands, Gran Canaria, S of Guia, La Dehesa, N28°06'59" W15°37'03", 490m, 15-Jan-2011, *Ficus carica*, beating, leg. Stüben,P., det. Stüben,P.
Ficusacalles ficvorator<br>(Stüben, 2007)<br>formerly: *F. senilis ficvorator*	GU987781 [01]<br>C-0084-sen<br>658nt	ZFMK-TIS-cC0084<br>ZFMK-DNA-0100400707	Spain, Canary Islands, La Palma, 1 km N of Las Caletas, N28°29'36" W17°49'46", 433m, 29-Jun-2007, *Ficus carica*, leg. Stüben,P., det. Stüben,P.
Ficusacalles ficvorator<br>(Stüben, 2007)<br>formerly: *F. senilis ficvorator*	GU987782 [01]<br>C-0085-sen<br>658nt	ZFMK-TIS-cC0085<br>ZFMK-DNA-0100400708	Spain, Canary Islands, La Palma, 2 km W of Barlovento, Bco. de la Vica, N28°49'12" W17°49'17", 494m, 16-Jul-2006, *Laurisilva, Hedera canariensis*, leg. Stüben,P., det. Stüben,P.
Ficusacalles ficvorator<br>(Stüben, 2007)<br>formerly: *F. senilis ficvorator*	MG229713 [40]<br>1570-JKR<br>658nt	ZFMK-TIS-3618<br>ZFMK-DNA-0155635664	Spain, Canary Islands, La Palma, Las Caletas, N28°29'34" W17°49'46", 418m, 08-Feb-2013, *Kleinia neirofolia*, ex larvae, leg. Krátky,J., det. Stüben,P.
Ficusacalles ficvorator<br>(Stüben, 2007)<br>formerly: *F. senilis ficvorator*	MG229730 [40]<br>1713-PST<br>658nt	ZFMK-TIS-24431<br>ZFMK-DNA-0155622477	Spain, Canary Islands, La Palma, W of Barlovento (near La Tosca), N28°49'12" W17°49'16", 475m, 27-Dez-2013, *Hederae*, beating, leg. Stüben,P., det. Stüben,P.
Ficusacalles ficvorator<br>(Stüben, 2007)<br>formerly: *F. senilis ficvorator*	MG229732 [40]<br>1715-PST<br>658nt	ZFMK-TIS-24433<br>ZFMK-DNA-0155622475	Spain, Canary Islands, La Palma, Montes de Luna, N28°31'57" W17°48'37", 583m, 28-Dez-2013, *Ficus*, beating, leg. Stüben,P., det. Stüben,P.
Ficusacalles ficvorator<br>(Stüben, 2007)<br>formerly: *F. senilis ficvorator*	MG229734 [40]<br>1728-PST<br>658nt	ZFMK-TIS-24446<br>ZFMK-DNA-0155622461	Spain, Canary Islands, La Palma, S of Mazo, above Salemera, N28°34'55" W17°46'10", 210m, 30-Dez-2013, *Ficus carica*, beating, leg. Stüben,P., det. Stüben,P.
Ficusacalles ficvorator<br>(Stüben, 2007)<br>formerly: *F. senilis ficvorator*	GU987931 [01]<br>E-557-sen<br>658nt	ZFMK-TIS-cE557<br>ZFMK-DNA-0100404975	Spain, Canary Islands, Tenerife, 6 km W of Los Silos, Casa Blanca, N28°21'40" W16°52'15", 120m, 21-Dez-2003, *Foeniculum vulgare, Kleinia*, succulent bush, leg. Stüben,P., det. Stüben,P.
Ficusacalles ficvorator<br>(Stüben, 2007)<br>formerly: *F. senilis ficvorator*	GU988005 [01]<br>E-696-sen<br>658nt	ZFMK-TIS-cE696<br>ZFMK-DNA-0100404862	Spain, Canary Islands, Tenerife, W of Los Silos, Teno Mts., Casa Blanca near Buenavista del Norte, N28°21'36" W16°52'10", 231m, 02-Okt-2008, leg. Astrin,J. & Stüben,P., det. Stüben,P.
Ficusacalles ficvorator<br>(Stüben, 2007)<br>formerly: *F. senilis ficvorator*	GU988011 [01]<br>E-707-sen<br>658nt	ZFMK-TIS-cE707<br>ZFMK-DNA-0100404848	Spain, Canary Islands, Tenerife, W of Los Silos, Teno Mts., Casa Blanca near Buenavista del Norte, Levada, N28°21'35" W16°52'05", 230m, 03-Okt-2008, *Ficus carica, Sonchus, Foeniculum vulgare*, leg. Astrin,J. & Stüben,P., det. Stüben,P.
Ficusacalles ficvorator<br>(Stüben, 2007)<br>formerly: *F. senilis ficvorator*	KC783828 [16]<br>711-PST<br>658nt	ZFMK-TIS-3048<br>ZFMK-DNA-0100448185	Spain, Canary Islands, Tenerife, Teno Mts., Los Silos, Barranco de Bucarón, N28°21'36" W16°48'51", 143m, 09-Jan-2012, *Ficus carica*, beating, leg. Stüben,P., det. Stüben,P.
Ficusacalles oceanicus<br>(Stüben 2002)<br>formerly: *F. senilis oceanicus*	MG229765 [40]<br>1958-PST<br>658nt	ZFMK-TIS-24037<br>ZFMK-DNA-0169170388	Portugal, Madeira, Ilhas Selvagens, Selvagem Pequena, around Pico do Veado, N30°02'09" W16°01'39", 15m, 07-Okt-2014, *Patellifolia patellaris*, sieving, leg. Stüben,P., det. Stüben,P.

**Name**<br>**Authority**<br>**Additional Information**	**GenBank Acc No (Ref.)**<br>**Specimen ID**<br>**Sequence Length**	**ZFMK Tissue ID**<br>**ZFMK DNA Sample ID**<br>**(SDEI DNA Sample ID)**	**Locality, GPS, Collection Date, Plant, Collector, Identifier**
Ficusacalles oceanicus<br>(Stüben 2002)<br>formerly: *F. senilis oceanicus*	MG229769 [40]<br>1967-PST<br>658nt	ZFMK-TIS-24046<br>ZFMK-DNA-0169170382	Portugal, Madeira, Ilhas Selvagens, Selvagem Pequena, W of Pico do Veado, N30°02'10" W16°01'40", 30m, 20-Dez-2014, *Patellifolia patellaris, Suaeda vera*, sieving, leg. Stüben,P., det. Stüben,P.
Ficusacalles senilis<br>(Wollaston, 1864)<br>formerly: *F. senilis senilis*	FJ716542 [01]<br>E-0259-sen<br>658nt	ZFMK-TIS-cE0259<br>ZFMK-DNA-0100400439	Spain, Canary Islands, El Hierro, 2.5 km E of Frontera, Mt. Timbarombo, N27°45'04" W17°59'02", 1311m, 28-Dez-2006, *Pericallis murrayi*, leg. Stüben,P., det. Stüben,P.
Ficusacalles senilis<br>(Wollaston, 1864)<br>formerly: *F. senilis senilis*	GU987783 [01]<br>C-0086-sen<br>658nt	ZFMK-TIS-cC0086<br>ZFMK-DNA-0100400918	Spain, Canary Islands, El Hierro, 2.5 km N of San Andres, Las Montanetas, N27°48'08" W17°57'36", 847m, 23-Dez-2006, *Ficus*, leg. Stüben,P., det. Stüben,P.
Ficusacalles senilis<br>(Wollaston, 1864)<br>formerly: *F. senilis senilis*	GU987784 [01]<br>C-0087-sen<br>658nt	ZFMK-TIS-cC0087<br>ZFMK-DNA-0100400710	Spain, Canary Islands, El Hierro, 3 km N of San Andres, N of slope Montana de la Fara, N27°47'39" W17°56'55", 936m, 25-Dez-2006, *Foeniculum vulgare*, leg. Stüben,P., det. Stüben,P.
Ficusacalles senilis<br>(Wollaston, 1864)<br>formerly: *F. senilis senilis*	GU987857 [01]<br>E-0257-sen<br>658nt	ZFMK-TIS-cE0257<br>ZFMK-DNA-0100401051	Spain, Canary Islands, El Hierro, 9.5 km W of La Frontera, Pista de Mencafite, N27°44'06" W18°05'08", 929m, 22-Dez-2006, *Chamaecytisus, Ficus*, leg. Stüben,P., det. Stüben,P.
Ficusacalles senilis<br>(Wollaston, 1864)<br>formerly: *F. senilis senilis*	GU987858 [01]<br>E-0260-sen<br>658nt	ZFMK-TIS-cE0260<br>ZFMK-DNA-0100400460	Spain, Canary Islands, El Hierro, 1 km SW of Guarazoca, Camino de la Pena, N27°48'06" W17°58'47", 746m, 02-Jan-2007, *Carlina salicifolia*, leg. Stüben,P., det. Stüben,P.
Ficusacalles senilis<br>(Wollaston, 1864)<br>formerly: *F. senilis senilis*	MG229830 [new]<br>E-0258-sen<br>658nt	ZFMK-TIS-24643<br>ZFMK-DNA-0155630416	Spain, Canary Islands, El Hierro, 3 km N of San Andres, N of slope Montana de la Fara, N27°47'39" W17°56'55", 936m, 25-Dez-2006, *Sonchus hierrensis*, leg. Stüben,P., det. Stüben,P.
Foucartia liturata<br>Stierlin, 1884	MK891914 [new]<br>2182-JKR<br>658nt	ZFMK-TIS-23920<br>ZFMK-DNA-0169170606	Slovakia, Nitra District, Nitra, Zobor, N48°21'08.5 E18°05'41", 400m, 24-Mai-2014, *Astragalus*, collecting by hand, leg. Stejskal,R., det. Stejskal,R.
Foucartia liturata<br>Stierlin, 1884	MK892116 [new]<br>2393-JKR<br>658nt	ZFMK-TIS-25970<br>ZFMK-DNA-0171600615	Slovakia, Nitra, Zobor Mt., N48°21'08.5 E18°5'41", 427m, 24-Mai-2014, *Artemisia campestris*, sieving, leg. Krátky,J., det. Krátky,J.
Foucartia squamulata<br>(Herbst, 1795)	KC783924 [new]<br>363-JKR<br>658nt	ZFMK-TIS-20149<br>ZFMK-DNA-0100438729	Czech Republic, Bohemia or., Horineves, N50°18'52.155" E15°46'30.012", 230m, 25-Jun-2011, sweeping, leg. Krátky,J., det. Krátky,J.
Foucartia squamulata<br>(Herbst, 1795)	KC784219 [new]<br>419-RGO<br>623nt	ZFMK-TIS-20205<br>ZFMK-DNA-0100449420	Poland, Tarnogóra, N50°41'11.6" E23°07'13.4", 249m, 11-Jun-2011, leg. Gosik,R., det. Gosik,R.
Geonemus flabellipes<br>(Olivier, 1807)	MK891585 [new]<br>492-PST<br>658nt	ZFMK-TIS-20277<br>ZFMK-DNA-0155630438	France, Vaucluse, Malaucene, Suzette, Dentelles de Montmirail, N44°10'47" E05°03'16", 542m, 02-Aug-2011, beating, leg. Stüben,P., det. Stüben,P.
Geonemus olcesii<br>Tournier, 1873	KC783879 [new]<br>125-PST<br>627nt	ZFMK-TIS-2D100440302<br>ZFMK-DNA-0100438190	Morocco, S of Berkane, Beni Snassen Mts., Vallee Zegzel, N34°48'56" W02°24'01", 678m, 12-Mai-2011, beating, leg. Stüben,P., det. Behne,L.
Glocianus distinctus<br>(C. Brisout de Barneville, 1870)	MK891051 [new]<br>951-CBR<br>658nt	ZFMK-TIS-3864<br>ZFMK-DNA-0100449660	Luxembourg, Luxembourg, N49°38'00" E06°10'45", 345m, 03-Jul-2012, *Hieracium*, sweeping, leg. Braunert,C., det. Braunert,C.
Glocianus distinctus<br>(C. Brisout de Barneville, 1870)	KC783912 [new]<br>385-JKR<br>658nt	ZFMK-TIS-20171<br>ZFMK-DNA-0100438703	Poland, Wielkopolskie, Rogalin env., N52°14'25.971" E16°54'20.152", 58m, 09-Jul-2011, sweeping, leg. Krátky,J., det. Krátky,J.

**Name**<br>**Authority**<br>**Additional Information**	**GenBank Acc No (Ref.)**<br>**Specimen ID**<br>**Sequence Length**	**ZFMK Tissue ID**<br>**ZFMK DNA Sample ID**<br>**(SDEI DNA Sample ID)**	**Locality, GPS, Collection Date, Plant, Collector, Identifier**
Glocianus distinctus<br>(C. Brisout de Barneville, 1870)	MK891262 [new]<br>1198-JKR<br>658nt	ZFMK-TIS-3534<br>ZFMK-DNA-0100426086	Slovakia, Komarno, Komarno, N47°45'30" E18°08'50", 106m, 07-Jul-2012, *Picris hieracioides*, sweeping, leg. Krátky,J., det. Krátky,J.
Glocianus granulithorax<br>(Schultze, 1900)	MK891283 [new]<br>1234-PST<br>658nt	ZFMK-TIS-3570<br>ZFMK-DNA-0100426055	Portugal, Estremadura, NW of Sintra, Azenhas do Mar, N38°50'35 W09°27'36", 64m, 16-Mai-2012, coast, sieving, leg. Stüben,P., det. Krátky,J.
Glocianus moelleri<br>(C. G. Thomson, 1868)	MK892065 [new]<br>2341-JKR<br>658nt	ZFMK-TIS-24272<br>ZFMK-DNA-0169170924	Czech Republic, Bohemia, Horni Morava, N50°10'15" E16°49'22", 780m, 17-Jul-2014, *Crepis*, sweeping, leg. Krátky,J., det. Krátky,J.
Glocianus moelleri<br>(C. G. Thomson, 1868)	MK892055 [new]<br>2331-JKR<br>658nt	ZFMK-TIS-24262<br>ZFMK-DNA-0169170934	Slovakia, Povazska Bystrica, Precin, N49°05'55" E18°32'48", 395m, 13-Jul-2014, *Crepis*, sweeping, leg. Krátky,J., det. Krátky,J.
Glocianus moelleri<br>(C. G. Thomson, 1868)	MK892120 [new]<br>2397-JKR<br>658nt	ZFMK-TIS-25974<br>ZFMK-DNA-0171600619	Slovakia, Zilina, Suja, N49°03'46" E18°37'19", 474m, 21-Jun-2014, sweeping, leg. Krátky,J., det. Krátky,J.
Glocianus punctiger<br>(C. R. Sahlberg, 1835)	KC783965 [new]<br>302-JKR<br>658nt	ZFMK-TIS-20088<br>ZFMK-DNA-0100438932	Czech Republic, Bohemia or., Hradec Kralove, Fararstvi, N50°11'26.901" E15°48'57.048", 230m, 06-Mai-2011, *Teraxacum officinalis*, sweeping, leg. Krátky,J., det. Krátky,J.
Glocianus punctiger<br>(C. R. Sahlberg, 1835)	MK891192 [new]<br>1109-JKR<br>657nt	ZFMK-TIS-3445<br>ZFMK-DNA-0100449594	Czech Republic, Bohemia, Hradec Kralove, Fararstvi, N50°11'22" E15°48'54", 230m, 04-Mai-2012, *Taraxacum officinalis*, beating, leg. Krátky,J., det. Krátky,J.
Glocianus punctiger<br>(C. R. Sahlberg, 1835)	MK891818 [new]<br>2078-JKR<br>658nt	ZFMK-TIS-23718<br>ZFMK-DNA-0169169655	Czech Republic, Bohemia, Kutrin, N49°49'12.5" E16°03'27", 456m, 08-Mai-2014, sweeping, leg. Krátky,J., det. Krátky,J.
Glocianus punctiger<br>(C. R. Sahlberg, 1835)	MK892205 [new]<br>2501-PSP<br>658nt	ZFMK-TIS-23473<br>ZFMK-DNA-0171661772	Germany, Lower Saxony (NI), Berkhof, Hannover region, N52°36'35" E09°43'55", 38m, 29-Apr-2014, *Taraxacum officinale*, collecting by hand, leg. Sprick,P., det. Sprick,P.
Glocianus punctiger<br>(C. R. Sahlberg, 1835)	MK892046 [new]<br>2322-JKR<br>658nt	ZFMK-TIS-24253<br>ZFMK-DNA-0169170940	Slovakia, Tatry Mts., Dolina Siedmich prame?ov, N49°13'30" E20°16'24", 1404m, 05-Jul-2014, *mountain meadow*, sweeping, leg. Krátky,J., det. Krátky,J.
Gonipterus platensis<br>(Marelli, 1926)	MK891625 [40]<br>1738-PST<br>658nt	ZFMK-TIS-24456<br>ZFMK-DNA-0155622452	Spain, Canary Islands, La Palma, N of Santa Cruz near Santa Lucia, Barranco del Agua, N28°43'28" W17°45'03", 371m, 05-Jan-2014, *Eucalyptus*, beating, leg. Stüben,P., det. Stüben,P.
Gonipterus platensis<br>(Marelli, 1926)	MK891168 [new]<br>1079-JKR<br>658nt	ZFMK-TIS-3415<br>ZFMK-DNA-0100449629	Spain, Andalucia, Rociana del Condado, N37°21'01" W06°36'55", 150m, 18-Apr-2012, *Eucalyptus*, beating, leg. Krátky,J., det. Krátky,J.
Graptus alternans<br>(Wollaston, 1865)	MK890804 [40]<br>87-PST<br>658nt	ZFMK-TIS-2D100446876<br>ZFMK-DNA-0100438024	Spain, Canary Islands, La Gomera, N of Epina, Teselinde, Santa Clara, N28°11'47" W17°17'16", 748m, 19-Feb-2011, beating, leg. Stüben,P., det. Stüben,P.
Graptus magnificus<br>(Wollaston, 1864)	MK890907 [40]<br>742-PST<br>658nt	ZFMK-TIS-3079<br>ZFMK-DNA-0100448145	Spain, Canary Islands, Tenerife, Anaga Mts., El Bailadero, N28°33'01" W16°12'14", 694m, 05-Feb-2012, *Echium strictum*, beating, leg. Stüben,P. & Schütte,A., det. Stüben,P.
Graptus magnificus<br>(Wollaston, 1864)	MK891288 [40]<br>1243-PST<br>658nt	ZFMK-TIS-3579<br>ZFMK-DNA-0100426041	Spain, Canary Islands, Tenerife, Anaga Mts., Lomo de Las Bodegas, N28°33'47" W16°09'15", 529m, 05-Feb-2012, beating, leg. Stüben,P., det. Stüben,P.

**Name**<br>**Authority**<br>**Additional Information**	**GenBank Acc No (Ref.)**<br>**Specimen ID**<br>**Sequence Length**	**ZFMK Tissue ID**<br>**ZFMK DNA Sample ID**<br>**(SDEI DNA Sample ID)**	**Locality, GPS, Collection Date, Plant, Collector, Identifier**
Graptus weberi<br>(Penecke, 1901)	MK892080 [new]<br>2356-JKR<br>658nt	ZFMK-TIS-25916<br>ZFMK-DNA-0171600585	Czech Republic, Moravia, NP Podyji, Cizov, Siroke pole, N48°51'36" E15°51'02", 300m, 17-Aug-2014, sweeping, leg. R. Stejskal, det. Stejskal,R.
Gronops lunatus<br>(Fabricius, 1775)	MK892179 [new]<br>2471-PSP<br>658nt	ZFMK-TIS-23443<br>ZFMK-DNA-0171661809	Germany, Lower Saxony (NI), Hannover, Vahrenheide, Kugelfangtrift, N52°25'22" E09°45'00", 49m, 16-Jun-2013, *Spergularia rubra*, collecting by hand, leg. Sprick,P., det. Sprick,P.
Gymnetron melanarium<br>(Germar, 1821)	MK891895 [new]<br>2161-JKR<br>658nt	ZFMK-TIS-23801<br>ZFMK-DNA-0169169575	Slovakia, Nove Zamky, Sturovo, Hegyfarok, N47°49'06" E18°38'38", 205m, 21-Mai-2014, *Veronica* sp., sweeping, leg. Krátky,J., det. Krátky,J.
Gymnetron melanarium<br>(Germar, 1821)	MK892237 [new]<br>2535-PSP<br>658nt	ZFMK-TIS-23507<br>ZFMK-DNA-0171661729	Slovakia, Nitra, W of Gbelce, N47°46'56" E18°27'37", 148m, 18-Mai-2014, *Veronica teucrium*, leg. Sprick,P., det. Sprick,P.
Gymnetron melinum<br>Reitter, 1872	KC783845 [new]<br>180-PST<br>606nt	ZFMK-TIS-2D100440247<br>ZFMK-DNA-0100438135	Morocco, Atlas Mts., S of Ifrane, Timahdite, N33°14'34" W05°04'19", 1821m, 19-Mai-2011, *Veronica*, beating, leg. Stüben,P., det. Behne,L.
Gymnetron rotundicolle<br>Gyllenhal, 1838	MK891197 [new]<br>1114-JKR<br>658nt	ZFMK-TIS-3450<br>ZFMK-DNA-0100449599	Czech Republic, Bohemia, Hradec Kralove, Brezhrad, N50°10'45" E15°47'39", 229m, 05-Mai-2012, *Veronica chamaedrys*, sweeping, leg. Krátky,J., det. Krátky,J.
Gymnetron tibiellum<br>Desbrochers des Loges, 1900	MK891708 [new]<br>1932-FBA<br>658nt	ZFMK-TIS-23323<br>ZFMK-DNA-0169170351	Greece, Rhodes Isl., Kolymbia, beach, N36°15'00" E28°10'00", 0m, 26-Apr-2014, leg. Winkelmann,H., det. Winkelmann,H.
Gymnetron tibiellum<br>Desbrochers des Loges, 1900	MK891202 [new]<br>1120-JKR<br>658nt	ZFMK-TIS-3456<br>ZFMK-DNA-0100449588	Slovakia, Nove Zamky, Tvrdosovce, N48°06'09" E18°02'08", 110m, 10-Mai-2012, sweeping, leg. Krátky,J., det. Krátky,J.
Gymnetron veronicae<br>(Germar, 1821)	KC783983 [new]<br>284-PSP<br>658nt	ZFMK-TIS-2D100439082<br>ZFMK-DNA-0100439754	Germany, Saxony-Anhalt (ST), Drömling, Mannhausen, N52°25'30" E11°12'44", 74m, 25-Mai-2011, *Veronica beccabunga*, body of standing water shore, dipnet, leg. Sprick,P., det. Sprick,P.
Gymnetron veronicae<br>(Germar, 1821)	KC784168 [new]<br>591-PSP<br>658nt	ZFMK-TIS-20375<br>ZFMK-DNA-0100448676	Germany, Lower Saxony (NI), Braunschweig, Rautheim, Wabeniederung, N52°15'28" E10°35'17", 75m, 09-Jun-2011, *Veronica anagallis-aquatica s.str.*, land recultivation area, dipnet, leg. Sprick,P., det. Sprick,P.
Gymnetron villosulum<br>Gyllenhal, 1838	MK891002 [new]<br>896-CBR<br>658nt	ZFMK-TIS-3809<br>ZFMK-DNA-0100449718	France, Dep. Ardenne, 10 km SE of Vouziers, Brecy-Brieres, N49°19'25" E04°46'23", 100m, 23-Apr-2011, sweeping, leg. Braunert,C., det. Braunert,C.
Gymnetron villosulum<br>Gyllenhal, 1838	KC784040 [new]<br>589-PSP<br>658nt	ZFMK-TIS-20373<br>ZFMK-DNA-0100448245	Germany, Lower Saxony (NI), Braunschweig, Rautheim, Wabeniederung, N52°15'14" E10°35'24", 76m, 09-Jun-2011, *Veronica anagallis-aquatica s.str.*, land recultivation area, dipnet, leg. Sprick,P., det. Sprick,P.
Gymnetron villosulum<br>Gyllenhal, 1838	KC784102 [new]<br>507-RST<br>658nt	ZFMK-TIS-20292<br>ZFMK-DNA-0100448465	Slovakia, Slovakia occ., 0.7 km SE of Dolne Zelenice, N48°22'13.95" E17°45'8.62", 135m, 29-Mai-2011, *Veronica* sp., beating, leg. Stejskal,R., det. Stejskal,R.
Gymnetron villosulum<br>Gyllenhal, 1838	MK891215 [new]<br>1134-JKR<br>658nt	ZFMK-TIS-3470<br>ZFMK-DNA-0100449571	Slovakia, Komarno, Imel, N47°54'34" E18°09'16", 112m, 11-Mai-2012, sweeping, leg. Krátky,J., det. Krátky,J.
Hadroplontus litura<br>(Fabricius, 1775)	KC783918 [new]<br>366-JKR<br>658nt	ZFMK-TIS-20152<br>ZFMK-DNA-0100438717	Czech Republic, Bohemia or., Hrobice, N50°6'2.909" E15°46'50.867", 230m, 05-Jul-2011, sweeping, leg. Krátky,J., det. Krátky,J.

**Name**<br>**Authority**<br>**Additional Information**	**GenBank Acc No (Ref.)**<br>**Specimen ID**<br>**Sequence Length**	**ZFMK Tissue ID**<br>**ZFMK DNA Sample ID**<br>**(SDEI DNA Sample ID)**	**Locality, GPS, Collection Date, Plant, Collector, Identifier**
Hadroplontus litura<br>(Fabricius, 1775)	KC784106 [new]<br>513-RST<br>658nt	ZFMK-TIS-20298<br>ZFMK-DNA-0100448471	Czech Republic, Moravia mer., kaolin pit 1.5 km SE of Únanov, N48°53'17.75" E16°3'22.34", 330m, 02-Jul-2011, *Cirsium arvense*, sweeping, leg. Stejskal,R., det. Stejskal,R.
Hadroplontus litura<br>(Fabricius, 1775)	MK891146 [new]<br>1053-PSP<br>658nt	ZFMK-TIS-3198<br>ZFMK-DNA-0100426806	Germany, Lower Saxony (NI), Hannover, Nordhafen, N52°25'28" E09°39'10", 51m, 13-Jun-2012, *Cirsium arvense*, south exposed slope with annual ruderal area, beating, leg. Sprick,P., det. Sprick,P.
Hadroplontus litura<br>(Fabricius, 1775)	MK891038 [new]<br>936-CBR<br>658nt	ZFMK-TIS-3849<br>ZFMK-DNA-0100449675	Luxembourg, 10 km E of Luxembourg, 1 km SW of Mensdorf, N49°39'32" E06°18'35", 300m, 09-Jun-2012, *Cirsium arvense*, sweeping, leg. Braunert,C., det. Braunert,C.
Hadroplontus trimaculatus<br>(Fabricius, 1775)	KC784237 [new]<br>IT-0004w<br>635nt	ZFMK-TIS-2D100446127<br>ZFMK-DNA-0100437871	Italy, Sardinia Isl. East, W of Siniscola, Monte Albo, N40°33'37" E09°38'01", 778m, 26-Sep-2010, *Quercus ilex*, cliff, limestone, leg. Stüben,P., det. Stüben,P.
Hadroplontus trimaculatus<br>(Fabricius, 1775)	MK891558 [new]<br>1652-JKR<br>658nt	ZFMK-TIS-4230<br>ZFMK-DNA-0155630488	Slovakia, Krupina, Cabrad, N48°14'56" E19°06'31", 280m, 11-Okt-2013, *Carduus*, sieving, leg. Krátky,J., det. Krátky,J.
Haptomerus lepidus<br>(Brullé, 1832)	KC784030 [new]<br>679-FBA<br>658nt	ZFMK-TIS-20457<br>ZFMK-DNA-0100446683	Greece, Peloponnese, Messinia, Mt. Taygetos, E of Agh. Nikolaos, N36°49'16" E22°17'55", 30m, 22-Mai-2011, leg. Bahr,F., det. Bahr,F.
Hemitrichapion pavidum<br>(Germar, 1817)	MK892092 [new]<br>2369-JKR<br>658nt	ZFMK-TIS-25929<br>ZFMK-DNA-0171600591	Czech Republic, Moravia, NP Podyji, Cizov, Siroke pole, N48°51'36" E15°51'2", 300m, 17-Aug-2014, sweeping, leg. R. Stejskal, det. Stejskal,R.
Hemitrichapion pavidum<br>(Germar, 1817)	KC784140 [new]<br>638-PSP<br>658nt	ZFMK-TIS-20421<br>ZFMK-DNA-0100448629	Germany, Hesse (HE), Wiesbaden-Erbenheim, N50°03'02" E08°18'13", 274m, 06-Sep-2011, *Coronilla varia (Securigera varia)*, dipnet, leg. Sprick,P., det. Sprick,P.
Hemitrichapion pavidum<br>(Germar, 1817)	KC784175 [new]<br>453-RGO<br>658nt	ZFMK-TIS-20239<br>ZFMK-DNA-0100449084	Poland, Rudnik, N51°14'31.02" E22°32'22.92", 191m, 03-Okt-2011, leg. Gosik,R., det. Gosik,R.
Hemitrichapion wagneri gomerense<br>Stüben & Behne, 2010<br>formerly: H. gomerense	MK892399 [40]<br>2877-PST<br>658nt	ZFMK-TIS-25882<br>ZFMK-DNA-0169164819	Spain, Canary Islands, El Hierro, N of Sabinosa, N27°44'58" W18°05'57", 233m, 14-Apr-2016, *Lotus glaucus*, beating, leg. Stüben,P. & Schütte,A., det. Stüben,P.
Hemitrichapion wagneri gomerense<br>Stüben & Behne, 2010<br>formerly: H. gomerense	KC783794 [40]<br>85-PST<br>658nt	ZFMK-TIS-2D100446963<br>ZFMK-DNA-0100437983	Spain, Canary Islands, La Gomera, 300 m W of Epina near Alojera, N28°10'00" W17°18'07", 699m, 19-Feb-2011, *Lotus* cf. *glaucus*, beating, leg. Stüben,P., det. Stüben,P.
Hemitrichapion wagneri gomerense<br>Stüben & Behne, 2010<br>formerly: *H. gomerense*	KC784319 [40]<br>774-PST<br>658nt	ZFMK-TIS-3111<br>ZFMK-DNA-0100448104	Spain, Canary Islands, La Gomera, Hermigua, (**type locality** of H. gomerense), N28°10'4" W17°11'12", 142m, 14-Mrz-2012, *Lotus emeroides*, beating, leg. Stüben,P., det. Stüben,P.
Hemitrichapion wagneri gomerense<br>Stüben & Behne, 2010<br>formerly: *H. gomerense*	KC784283 [40]<br>698-PST<br>658nt	ZFMK-TIS-3035<br>ZFMK-DNA-0100448189	Spain, Canary Islands, Tenerife, Santa Barbara, N28°21'50" W16°41'06", 523m, 03-Jan-2012, *Lotus campylocladus*, beating, leg. Stüben,P., det. Stüben,P.

**Name**<br>**Authority**<br>**Additional Information**	**GenBank Acc No (Ref.)**<br>**Specimen ID**<br>**Sequence Length**	**ZFMK Tissue ID**<br>**ZFMK DNA Sample ID**<br>**(SDEI DNA Sample ID)**	**Locality, GPS, Collection Date, Plant, Collector, Identifier**
Hemitrichapion wagneri wagneri<br>(Flach, 1906)	MK890968 [40]<br>851-PST<br>658nt	ZFMK-TIS-3764<br>ZFMK-DNA-0100414249	Portugal, Estremadura, N of Ericeira, Ribamar, coast, N38°59'29" W09°24'54", 28m, 18-Mai-2012, *Trifolium*, beating, leg. Stüben,P., det. Stüben,P.
Hemitrichapion wagneri wagneri<br>(Flach, 1906)	MK891165 [40]<br>1076-JKR<br>658nt	ZFMK-TIS-3412<br>ZFMK-DNA-0100449632	Spain, Andalucia, Pedro Valiente, playa de Tarifa, N36°02'49" W05°38'26", 3m, 15-Apr-2012, *Lotus creticus*, individual collecting by hand, leg. Krátky,J., det. Krátky,J.
Hemitrichapion wagneri wagneri<br>(Flach, 1906)	MK891287 [40]<br>1240-PST<br>658nt	ZFMK-TIS-3576<br>ZFMK-DNA-0100426044	Spain, Canary Islands, Tenerife, Teno Mts., Teno Alto, N28°20'32" W16°51'50", 830m, 03-Jan-2012, *Lotus*, beating, leg. Stüben,P., det. Stüben,P.
Hemitrichapion wagneri wagneri<br>(Flach, 1906)	MK891588 [40]<br>705-PST<br>658nt	ZFMK-TIS-3042<br>ZFMK-DNA-0155630430	Spain, Canary Islands, Tenerife, Las Hiedras near Los Llanos, N28°19'57" W16°45'33", 1069m, 04-Jan-2012, *Lotus campylocladus under Pinus canariensis*, beating, leg. Stüben,P., det. Stüben,P.
Hemitrichapion waltoni<br>(Stephens, 1839)	MK891967 [new]<br>2237-JKR<br>658nt	ZFMK-TIS-23975<br>ZFMK-DNA-0169170550	Slovakia, Banovce nad Bebravou, Lutov env., Bradlo, N48°47'42.5" E18°17'06", 473m, 19-Sep-2014, *Hippocrepis comosa*, leg. Benedikt,S., det. Benedikt,S.
Herpisticus bobadillae<br>Machado, 2020<br>**Paratype (DNAtype)**	MH051982 [38]<br>1674-PST_24392<br>658nt	ZFMK-TIS-24392<br>ZFMK-DNA-0155622516	Spain, Canary Islands, La Gomera, Parque Natural de Majona, above Casas del Palmar, N28°09'28" W17°09'41", 641m, 07-Dez-2013, beating, leg. Stüben,P., det. Stüben,P. & Machado,A.
Herpisticus daute<br>Machado, 2020<br>**Paratype (DNAtype)**	KC784291 [38]<br>717-PST_18T2012<br>658nt	ZFMK-TIS-3054<br>ZFMK-DNA-0100448170	Spain, Canary Islands, Tenerife, Teno Mts., Los Carrizales, Bco. del Carrizal, N28°19'14" W16°52'03", 434m, 13-Jan-2012, beating, leg. Stüben,P., det. Stüben,P. & Machado,A.
Herpisticus denudatus<br>Machado, 2020<br>**Paratype (DNAtype)**	KC783762 [38]<br>51-PST_55GC2011<br>658nt	ZFMK-TIS-2D100446996<br>ZFMK-DNA-0100437949	Spain, Canary Islands, Gran Canaria, N of Maspalomas - Fataga, Degollada del Burro, N27°47'27" W15°34'46", 200m, 22-Jan-2011, *Kleinia*, beating, leg. Stüben,P., det. Stüben,P. & Machado,A.
Herpisticus famarae<br>Machado, 2020<br>**NMB Paratype (DNAtype)**	MH051985 [38]<br>2926-PST<br>658nt	(SDEI-DNA-2926-PST)	Spain, Canary Islands, Lanzarote, Calle La Rositas, Finca La Corona, N29°11'48" W13°29'33", 354m, 04-Jan-2017, sifting, leg. Stüben,P., det. Stüben,P. & Machado,A.
Herpisticus gomerensis<br>Machado, 2020<br>**Paratype (DNAtype)**	MH051981 [38]<br>1663-PST_24381<br>658nt	ZFMK-TIS-24381<br>ZFMK-DNA-0155622527	Spain, Canary Islands, La Gomera, Valle Gran Rey, Playa del Ingles, N28°05'56" W17°20'53", 10m, 03-Dez-2013, *Chenopodiaceae*, sieving, leg. Stüben,P., det. Stüben,P. & Machado,A.
Herpisticus hierrensis benahoare<br>Machado, 2020<br>**Paratype (DNAtype)**	MH051983 [38]<br>1747-PST_24465<br>658nt	ZFMK-TIS-24465<br>ZFMK-DNA-0155621472	Spain, Canary Islands, La Palma, W of Puntagorda near Puerto Puntagorda, N28°45'32" W18°00'08", 227m, 17-Jan-2014, *Kleinia*, beating, leg. Stüben,P., det. Stüben,P. & Machado,A.
Herpisticus hispidus<br>Machado, 2020	KC783792 [40]<br>83-PST<br>648nt	ZFMK-TIS-2D100446965<br>ZFMK-DNA-0100438037	Spain, Canary Islands, La Gomera, Benchijiua, N28°05'02" W17°12'26", 711m, 17-Feb-2011, *Euphorbia*, beating, leg. Stüben,P., det. Stüben,P. & Machado,A.
Herpisticus laesicollis<br>Germar, 1824	MH051980 [38]<br>763-PST<br>658nt	ZFMK-TIS-3100<br>ZFMK-DNA-0155630433	Spain, Canary Islands, Tenerife, Anaga Mts., Lomo de Las Bodegas, at night, N28°33'47" W16°09'15", 543m, 25-Feb-2012, beating, leg. Stüben,P., det. Stüben,P. & Machado,A.
Herpisticus rectipes<br>Machado, 2020<br>**Paratype (DNAtype)**	MH051984 [38]<br>2730-PST_23880<br>658nt	ZFMK-TIS-23880<br>ZFMK-DNA-0171661269	Spain, Canary Islands, Fuerteventura, Macizo de Jandia, Risco de Pasco, N28°06'54" W14°16'7", 16m, 05-Jan-2015, *Launea arborescens*, beating, leg. Stüben,P., det. Stüben,P. & Machado,A.

**Name**<br>**Authority**<br>**Additional Information**	**GenBank Acc No (Ref.)**<br>**Specimen ID**<br>**Sequence Length**	**ZFMK Tissue ID**<br>**ZFMK DNA Sample ID**<br>**(SDEI DNA Sample ID)**	**Locality, GPS, Collection Date, Plant, Collector, Identifier**
Hesperorrhynchus lineatotessellatus x glutinosus<br>horizontal gene transfer from *C. glutinosus*?	KT823492 [26]<br>1936-PST<br>658nt	ZFMK-TIS-24015<br>ZFMK-DNA-0169170414	Portugal, Madeira, Faial, coast, N32°47'37" W16°50'57", 26m, 04-Okt-2014, *Aeonium glutinosum*, sieving, leg. Stüben,P., det. Stüben,P.
Hesperorrhynchus lineatotessellatus x glutinosus<br>horizontal gene transfer from *C. glutinosus*?	KT823496 [26]<br>2773-PST<br>658nt	ZFMK-TIS-26127<br>ZFMK-DNA-0169166969	Portugal, Madeira, near Boaventura, Sao Cristovao, N32°49'32" W16°58'11", 61m, 20-Mrz-2015, *Aeonium glutinosum*, collecting by hand, leg. Stüben,P., det. Stüben,P.
Hesperorrhynchus cf. *incautus*	KT823495 [26]<br>2763-JKR<br>658nt	ZFMK-TIS-26212<br>ZFMK-DNA-0171661906	Spain, Canary Islands, Gran Canaria, Las Lagunetas, Barranco de La Mina, N27°59'55" W15°35'13", 1241m, 22-Feb-2015, *Aichryson* cf. *laxum*, sieving, leg. Krátky,J., det. Krátky,J.
Hesperorrhynchus glutinosus<br>Stüben, 2016<br>**Paratype (DNAtype)**	KT823497 [26]<br>2794-PST_26148<br>658nt	ZFMK-TIS-26148<br>ZFMK-DNA-0169166948	Portugal, Madeira, Santa Madalena, Salao, coast, N32°51'59" W17°11'57", 313m, 04-Apr-2015, *Aeonium glutinosum*, collecting by hand, leg. Stüben,P., det. Stüben,P.
Hesperorrhynchus hesperus<br>(Wollaston, 1864)	MK347552 [40]<br>2965-PST<br>658nt	(SDEI-DNA-2965-PST)	Spain, Canary Islands, El Hierro, El Golfo, 1,5 km S Los Llanillos, N27°44'22" W18°01'40", 725m, 13-Apr-2016, *Aichryson*, leg. Stüben & Schütte, det. Stüben,P.
Hesperorrhynchus hesperus<br>(Wollaston, 1864)	MK892394 [40]<br>2870-PST<br>658nt	ZFMK-TIS-4259<br>ZFMK-DNA-0169166922	Spain, Canary Islands, El Hierro, W of Frontera, Pista al Derrabado, N27°44'11" W18°04'43", 785m, 11-Apr-2016, *Aeonium holochrysum*, beating, leg. Stüben,P. & Schütte,A., det. Stüben,P.
Hesperorrhynchus hesperus<br>(Wollaston, 1864)	MK892406 [new]<br>2886-PST<br>658nt	ZFMK-TIS-25892<br>ZFMK-DNA-0169166913	Spain, Canary Islands, El Hierro, El Golfo, SW of Tigaday, Hoya del Pino, N27°44'05" W18°02'17", 1087m, 22-Apr-2016, *Aichryson laxum*, beating, leg. Stüben,P. & Schütte,A., det. Stüben,P.
Hesperorrhynchus hesperus<br>(Wollaston, 1864)	KC783797 [13]<br>89-PST<br>645nt	ZFMK-TIS-2D100446954<br>ZFMK-DNA-0100438026	Spain, Canary Islands, La Gomera, Parque Garajonay, El Cedro near “Casa Olsen”, N28°6'29" W17°13'56", 1360m, 23-Feb-2011, *Aichryson* cf. *pachycaulon*, beating, leg. Stüben,P., det. Stüben,P.
Hesperorrhynchus hesperus<br>(Wollaston, 1864)	KC783801 [26]<br>94-PST<br>646nt	ZFMK-TIS-2D100446912<br>ZFMK-DNA-0100438006	Spain, Canary Islands, La Gomera, S of Hermigua, La Palmita, N28°10'33" W17°12'37", 632m, 07-Feb-2011, *Monanthes*, beating, leg. Stüben,P., det. Stüben,P.
Hesperorrhynchus lineatotessellatus<br>(Wollaston, 1854)	KT823491 [26]<br>1847-PST<br>658nt	ZFMK-TIS-26050<br>ZFMK-DNA-0171606119	Portugal, Madeira, SE of Ribeira da Janela, Fanal, N32°48'48" W17°08'57", 1084m, 29-Jun-2014, *Aichryson divaricatum*, sieving, leg. Stüben,P., det. Stüben,P.
Hesperorrhynchus lineatotessellatus<br>(Wollaston, 1854)	KT823493 [26]<br>1950-PST<br>658nt	ZFMK-TIS-24029<br>ZFMK-DNA-0169170399	Portugal, Madeira, SE of Ribeira da Janela, Fanal, N32°48'48" W17°08'57", 1084m, 05-Okt-2014, *Aichryson divaricatum on Ocotea foetens* , sieving, leg. Stüben,P., det. Stüben,P.
Hesperorrhynchus palmensis<br>Krátky, 2016	KT823494 [26]<br>1979-JKR<br>658nt	ZFMK-TIS-23619<br>ZFMK-DNA-0169170522	Spain, Canary Islands, La Palma, Brena Alta, 3 km SW, N28°37'58" W17°48'26.5", 774m, 07-Feb-2014, *Aichryson* cf. *palmense*, sieving, leg. Krátky,J., det. Krátky,J.
Hesperorrhynchus phytobioides<br>(Wollaston, 1864)	KM433737 [13]<br>713-PST<br>658nt	ZFMK-TIS-3050<br>ZFMK-DNA-0100448174	Spain, Canary Islands, Tenerife, Anaga Mts. near Taborno, N28°32'49" W16°16'48", 562m, 11-Jan-2012, *Aichryson*, beating, leg. Stüben,P., det. Stüben,P.
Hesperorrhynchus phytobioides<br>(Wollaston, 1864)	KT823490 [26]<br>1241-PST<br>658nt	ZFMK-TIS-3577<br>ZFMK-DNA-0100426043	Spain, Canary Islands, Tenerife, Anaga Mts., Taborno, N28°33'19" W16°15'54", 662m, 11-Jan-2012, *Aichryson*, beating, leg. Stüben,P., det. Stüben,P.

**Name**<br>**Authority**<br>**Additional Information**	**GenBank Acc No (Ref.)**<br>**Specimen ID**<br>**Sequence Length**	**ZFMK Tissue ID**<br>**ZFMK DNA Sample ID**<br>**(SDEI DNA Sample ID)**	**Locality, GPS, Collection Date, Plant, Collector, Identifier**
Holotrichapion aethiops<br>(Herbst, 1797)	KC784158 [new]<br>608-PSP<br>658nt	ZFMK-TIS-20391<br>ZFMK-DNA-0100448662	Germany, Saxony-Anhalt (ST), Drömling, Mannhausen, N52°25'36" E11°12'36.5'', 54m, 06-Jul-2011, *Vicia cracca, V. hirsuta*, humid ruderal area, beating, leg. Sprick,P., det. Sprick,P.
Holotrichapion aethiops<br>(Herbst, 1797)	MK892329 [new]<br>2631-PSP<br>658nt	ZFMK-TIS-23605<br>ZFMK-DNA-0171661923	Germany, Lower Saxony (NI), Hannover, Leinhausen, N52°23'44" E09°42'53", 53m, 22-Jun-2014, *Vicia hirsuta*, dipnet, leg. Sprick,P., det. Sprick,P.
Holotrichapion ononis<br>(Kirby, 1808)	KC784107 [new]<br>515-RST<br>658nt	ZFMK-TIS-20300<br>ZFMK-DNA-0100448473	Czech Republic, Moravia mer., kaolin pit 1.5 km SE of Únanov, N48°53'17.75" E16°3'22.34", 330m, 02-Jul-2011, sweeping, leg. Stejskal,R., det. Stejskal,R.
Holotrichapion ononis<br>(Kirby, 1808)	MK891444 [new]<br>1508-PSP<br>658nt	ZFMK-TIS-3268<br>ZFMK-DNA-0155633321	Denmark, Syddanmark, Emmerlev Klev (Højer), N54°59'53" E08°39'08", 3m, 30-Aug-2012, *Ononis spinosa*, beating, leg. Sprick,P., det. Sprick,P.
Holotrichapion ononis<br>(Kirby, 1808)	MK892401 [40]<br>2879-PST<br>658nt	ZFMK-TIS-25884<br>ZFMK-DNA-0169166906	Spain, Canary Islands, El Hierro, La Dehesa, NW of Ermita Virgen de los Reyes, N27°43'55" W18°07'18", 696m, 14-Apr-2016, beating, leg. Stüben,P. & Schütte,A., det. Stüben,P.
Holotrichapion ononis<br>(Kirby, 1808)	MK890980 [new]<br>871-PST<br>658nt	ZFMK-TIS-3784<br>ZFMK-DNA-0100413784	Germany, Rhineland-Palatinate (RLP), Eifel, Nettersheim, Urftbach, N50°30'36" E06°37'20", 446m, 05-Aug-2012, beating, leg. Stüben,P., det. Stüben,P.
Holotrichapion ononis<br>(Kirby, 1808)<br>formerly: *H. umbrinum*	KC783791 [40]<br>82-PST<br>627nt	ZFMK-TIS-2D100446966<br>ZFMK-DNA-0100438038	Spain, Canary Islands, La Gomera, Hermigua near Las Nuevitas, N28°09'29" W17°11'43", 191m, 17-Feb-2011, *Ononis* sp., beating, leg. Stüben,P., det. Stüben,P.
Holotrichapion ononis<br>(Kirby, 1808)	MK891039 [new]<br>937-CBR<br>658nt	ZFMK-TIS-3850<br>ZFMK-DNA-0100449663	Luxembourg, 10 km E of Luxembourg, 1 km SW of Mensdorf, N49°39'32" E06°18'35", 300m, 09-Jun-2012, *Ononis repens*, sweeping, leg. Braunert,C., det. Braunert,C.
Holotrichapion ononis<br>(Kirby, 1808)	KC783871 [new]<br>148-PST<br>658nt	ZFMK-TIS-2D100440985<br>ZFMK-DNA-0100438178	Morocco, S of Berkane, Beni Snassen Mts., Vallee Zegzel, N34°49'26" W02°22'59", 634m, 12-Mai-2011, beating, leg. Stüben,P., det. Stüben,P.
Holotrichapion pisi<br>(Fabricius, 1801)	MK892093 [new]<br>2370-JKR<br>658nt	ZFMK-TIS-25930<br>ZFMK-DNA-0171600592	Czech Republic, Moravia, Znojmo, N48°51'05" E16°05'59", 250m, 23-Aug-2014, sweeping, leg. R. Stejskal, det. Stejskal,R.
Holotrichapion pisi<br>(Fabricius, 1801)	MK891706 [new]<br>1927-FBA<br>658nt	ZFMK-TIS-23318<br>ZFMK-DNA-0169170356	Greece, East Macedonia and Thrace, Drama, Volakas, quarry, N41°18'17" E24°00'41", 960m, 15-Aug-2014, leg. Bayer & Brunner, det. Bayer,F.
Holotrichapion pisi<br>(Fabricius, 1801)	KC784238 [new]<br>IT-0005w<br>645nt	ZFMK-TIS-2D100446564<br>ZFMK-DNA-0100437870	Italy, Sardinia Isl. East, W of Siniscola, Monte Albo, N40°33'37" E09°38'01", 778m, 26-Sep-2010, *Quercus ilex*, cliff, limestone, leg. Stüben,P., det. Stüben,P.
Holotrichapion pisi<br>(Fabricius, 1801)	KC784263 [new]<br>IT-0033w<br>658nt	ZFMK-TIS-2D100446548<br>ZFMK-DNA-0100433887	Italy, Sardinia Isl. West, SW of Macomer, M. Ferru near Badde Urbara, N40°09'49" E08°37'28", 873m, 07-Okt-2010, *Quercus*, mixed woodland , leg. Stüben,P., det. Stüben,P.
Holotrichapion pisi<br>(Fabricius, 1801)	MK891635 [new]<br>1773-PST<br>658nt	ZFMK-TIS-24097<br>ZFMK-DNA-0171624091	Italy, Lazio, Alvito, S. Onófria, Val de Rio, N41°44'19" E13°43'41", 840m, 05-Mai-2014, *Medicago (eng. lucerne)*, beating, leg. Stüben,P. & Schütte,A., det. Stüben,P.
Holotrichapion pisi<br>(Fabricius, 1801)	MK891316 [new]<br>1311-PST<br>658nt	ZFMK-TIS-4127<br>ZFMK-DNA-0100426165	Spain, Malaga, S of Antequera, Sierra de Chimenea, N36°57'38" W04°35'34", 984m, 05-Mai-2013, *roadside vegetation*, sieving, leg. Stüben,P. & Schütte,A., det. Stüben,P.

**Name**<br>**Authority**<br>**Additional Information**	**GenBank Acc No (Ref.)**<br>**Specimen ID**<br>**Sequence Length**	**ZFMK Tissue ID**<br>**ZFMK DNA Sample ID**<br>**(SDEI DNA Sample ID)**	**Locality, GPS, Collection Date, Plant, Collector, Identifier**
Holotrichapion pullum<br>(Gyllenhal, 1833)	MK892099 [new]<br>2376-JKR<br>658nt	ZFMK-TIS-25953<br>ZFMK-DNA-0171600598	Czech Republic, Moravia, Znojmo, N48°51'05" E16°05'59", 250m, 23-Aug-2014, sweeping, leg. R. Stejskal, det. Stejskal,R.
Holotrichapion rotundipenne<br>(Wollaston, 1854)	MK347547 [40]<br>2960-PST<br>658nt	(SDEI-DNA-2960-PST)	Spain, Canary Islands, El Hierro, SE Sabinosa, near El Gretime, N27°44'24" W18°04'47", 651m, 11-Apr-2016, leg. Stüben & Schütte, det. Stüben,P.
Holotrichapion rotundipenne<br>(Wollaston, 1854)	KC783739 [40]<br>17-PST<br>658nt	ZFMK-TIS-2D100447027<br>ZFMK-DNA-0100437967	Spain, Canary Islands, Gran Canaria, Moya, Barranco de los Tilos, N28°04'33" W15°36'05", 643m, 07-Dez-2010, *Vicia*, beating, leg. Stüben,P., det. Stüben,P.
Holotrichapion rotundipenne<br>(Wollaston, 1854)	KC783771 [40]<br>62-PST<br>658nt	ZFMK-TIS-2D100446975<br>ZFMK-DNA-0100438047	Spain, Canary Islands, La Gomera, Hermigua, Las Nuevitas, Barranquillos los Alamos, N28°09'31" W17°11'09", 251m, 13-Dez-2010, *Vicia*, beating, leg. Stüben,P., det. Stüben,P.
Holotrichapion rotundipenne<br>(Wollaston, 1854)	MK891592 [40]<br>1667-PST<br>658nt	ZFMK-TIS-24385<br>ZFMK-DNA-0155622523	Spain, Canary Islands, La Gomera, Parque Natural de Majona, La Guerode – Enchereda, N28°07'52" W17°09'38", 689m, 05-Dez-2013, *Vicia*, beating, leg. Stüben,P., det. Stüben,P.
Holotrichapion rotundipenne<br>(Wollaston, 1854)	MK891610 [40]<br>1697-PST<br>658nt	ZFMK-TIS-24415<br>ZFMK-DNA-0155622493	Spain, Canary Islands, La Palma, N of Santa Cruz, M. de Tagoja, N28°43'18" W17°47'07", 1047m, 21-Dez-2013, *Vicia*, beating, leg. Stüben,P., det. Stüben,P.
Holotrichapion rotundipenne<br>(Wollaston, 1854)	MK891616 [40]<br>1723-PST<br>658nt	ZFMK-TIS-24441<br>ZFMK-DNA-0155622456	Spain, Canary Islands, La Palma, nördlich Tijarafe near Aguatavar (Barranco), N28°44'20" W17°57'51", 862m, 29-Dez-2013, sieving, leg. Stüben,P., det. Stüben,P.
Holotrichapion rotundipenne<br>(Wollaston, 1854)	MK892363 [40]<br>2774-PST<br>658nt	ZFMK-TIS-26128<br>ZFMK-DNA-0169166968	Portugal, Madeira, near Boaventura, Sao Cristovao, N32°49'32" W16°58'11", 61m, 20-Mrz-2015, *Lathyrus tingitanus*, collecting by hand, leg. Stüben,P., det. Stüben,P.
Holotrichapion wollastoni<br>(Chevrolat, 1852)	MK891676 [40]<br>1885-PST<br>658nt	ZFMK-TIS-26088<br>ZFMK-DNA-0171606074	Portugal, Madeira, near Ilha, W of Sao Jorge, Achada do Marques, N32°48'18" W16°54'28", 404m, 14-Jul-2014, *Lotus*, beating, leg. Stüben,P., det. Stüben,P.
Holotrichapion wollastoni<br>(Chevrolat, 1852)	MK892376 [40]<br>2828-PST<br>658nt	ZFMK-TIS-4738<br>ZFMK-DNA-FC17941485	Portugal, Madeira, N of Santana, Quebrada, N32°48'49" W16°52'27", 124m, 25-Nov-2015, *Lotus*, beating, leg. Stüben,P. & Schütte,A., det. Stüben,P.
Homorosoma validirostre<br>(Gyllenhal, 1837)	MK891206 [new]<br>1124-JKR<br>658nt	ZFMK-TIS-3460<br>ZFMK-DNA-0100449584	Slovakia, Komarno, Landor, NPR Apali, N47°47'34" E18°07'49", 111m, 11-Mai-2012, *Persicaria* sp., sweeping, leg. Krátky,J., det. Krátky,J.
Hoplopodapion poupillieri<br>(Wencker, 1864)	KC783862 [new]<br>151-PST<br>658nt	ZFMK-TIS-2D100440276<br>ZFMK-DNA-0100438164	Morocco, NW of Berkane, Oued Moulouya, N34°56'49" W02°33'08", 49m, 14-Mai-2011, *Tamarix*, beating, leg. Stüben,P., det. Behne,L.
Hylobius abietis<br>(Linnaeus, 1758)	MK892011 [new]<br>2284-JKR<br>658nt	ZFMK-TIS-24215<br>ZFMK-DNA-0169170981	Austria, Styria, Hochschwab, Stallmauer, N47°35'29" E15°02'20", 1260m, 27-Jun-2014, mountain forest, sweeping, leg. Krátky,J., det. Krátky,J.
Hylobius abietis<br>(Linnaeus, 1758)	MK891084 [new]<br>987-PSP<br>658nt	ZFMK-TIS-3132<br>ZFMK-DNA-0100426280	Germany, Lower Saxony (NI), near Hannover, Berkhof, N52°36'48" E09°43'56", 36m, 28-Apr-2012, *Pinus sylvestris*, pine forest, beating, leg. Sprick,P., det. Sprick,P.
Hylobius abietis<br>(Linnaeus, 1758)	MK891301 [new]<br>1281-PST<br>658nt	ZFMK-TIS-4097<br>ZFMK-DNA-0100426449	Spain, Community of Madrid, NE of Montejo de la Sierra, Hayedo de Montejo, NE of Hiruela, Puerto de la Hiruela, N41°04'02" W03°28'28", 1478m, 02-Mai-2013, *Pinus*, hand collecting, leg. Stüben,P. & Schütte,A., det. Stüben,P.

**Name**<br>**Authority**<br>**Additional Information**	**GenBank Acc No (Ref.)**<br>**Specimen ID**<br>**Sequence Length**	**ZFMK Tissue ID**<br>**ZFMK DNA Sample ID**<br>**(SDEI DNA Sample ID)**	**Locality, GPS, Collection Date, Plant, Collector, Identifier**
Hylobius alpheus<br>Reiche & Saulcy, 1858	KC784034 [new]<br>680-FBA<br>658nt	ZFMK-TIS-20458<br>ZFMK-DNA-0100447291	Greece, Peloponnese, Messinia, W of Mt. Taygetos, E of Saidona, N36°53'01" E22°17'07", 680m, 23-Mai-2011, leg. Bayer,B., det. Bayer,B.
Hylobius transversovittatus<br>(Goeze, 1777)	KC783956 [new]<br>376-JKR<br>658nt	ZFMK-TIS-20162<br>ZFMK-DNA-0100438903	Czech Republic, Bohemia or., Hrobice, N50°6'2.909" E15°46'50.867", 230m, 05-Jul-2011, sweeping, leg. Krátky,J., det. Krátky,J.
Hylurgops palliatus<br>(Gyllenhal, 1813)	MK892199 [new]<br>2494-PSP<br>658nt	ZFMK-TIS-23466<br>ZFMK-DNA-0171661784	Germany, Saxony-Anhalt (ST), Harz National Park, Drei Annen Hohne, Hohneklippen, Hohnekopf, N51°46'36" E10°41'57", 835m, 24-Apr-2014, *Picea abies*, beating, leg. Sprick,P., det. Sprick,P.
Hypera cf. *postica*	KC784256 [new]<br>IT-0026w<br>658nt	ZFMK-TIS-2D100446533<br>ZFMK-DNA-0100437850	Italy, Sardinia Isl. West, E of Macomer, above Silanus, N40°19'20" E08°52'13", 1015m, 04-Okt-2010, *Acer monspessulanum, Quercus, Verbascum*, meadow, leg. Stüben,P., det. Stüben,P.
Hypera conmaculata<br>(Herbst, 1795)	KC783982 [new]<br>283-PSP<br>658nt	ZFMK-TIS-2D100438533<br>ZFMK-DNA-0100439753	Germany, Saxony-Anhalt (ST), Drömling, Mannhausen, N52°25'42" E11°12'16.5", 74m, 25-Mai-2011, *Sium latifolium*, body of standing water shore, beating, leg. Sprick,P., det. Sprick,P.
Hypera contaminata<br>(Herbst, 1795)	MK892132 [new]<br>2411-JKR<br>658nt	ZFMK-TIS-25988<br>ZFMK-DNA-0171600626	Czech Republic, Bohemia, Hradec Kralove, Kocianovice, N50°13'16" E15°52'51", 237m, 24-Aug-2014, collecting by hand, leg. Krátky,J., det. Krátky,J.
Hypera cumana<br>(Petri, 1901)	MK891925 [new]<br>2193-JKR<br>658nt	ZFMK-TIS-23931<br>ZFMK-DNA-0169170594	Slovakia, Nove Zamky, Imel, Liscie diery, N47°55'17" E18°09'37", 114m, 18-Mai-2014, sweeping, leg. Stejskal,R., det. Stejskal,R.
Hypera diversipunctata<br>(Schrank, 1798)	MK891468 [new]<br>1535-PSP<br>658nt	ZFMK-TIS-3295<br>ZFMK-DNA-0155633294	Germany, Saxony-Anhalt (ST), Wendischbrome, Lkr. Salzwedel, N52°37'32" E10°55'00", 72m, 14-Jun-2013, *Stellaria palustris*, pond ditch, Zucht, leg. Sprick,P., det. Sprick,P.
Hypera jucunda<br>(Capiomont, 1868)	MK891246 [new]<br>1175-JKR<br>658nt	ZFMK-TIS-3511<br>ZFMK-DNA-0100426227	Spain, Andalucia, 8 km S of Albunuelas, Sierra del Albunuelas, N36°53'10" W03°43'49", 1312m, 09-Jun-2012, *Anthylis*, beating, leg. Krátky,J., det. Krátky,J.
Hypera meles<br>(Fabricius, 1792)	MK891853 [new]<br>2115-JKR<br>658nt	ZFMK-TIS-23755<br>ZFMK-DNA-0169169625	Czech Republic, Moravia, Lanzhot, N48°42'37" E16°58'27", 156m, 10-Mai-2014, sweeping, leg. Krátky,J., det. Krátky,J.
Hypera meles<br>(Fabricius, 1792)	MK892114 [new]<br>2391-JKR<br>658nt	ZFMK-TIS-25968<br>ZFMK-DNA-0171600613	Czech Republic, Moravia, Znojmo, N48°51'05" E16°05'59", 250m, 16-Sep-2014, sweeping, leg. R. Stejskal, det. Stejskal,R.
Hypera meles<br>(Fabricius, 1792)	MK891140 [new]<br>1045-PSP<br>658nt	ZFMK-TIS-3190<br>ZFMK-DNA-0100426934	Germany, Lower Saxony (NI), Hannover, Ahlem, N52°23'00" E09°40'55", 50m, 01-Jun-2012, *Trifolium dubium*, Faboideae-rich ruderal area, dipnet, leg. Sprick,P., det. Sprick,P.
Hypera meles<br>(Fabricius, 1792)	MK891380 [new]<br>1433-JKR<br>658nt	ZFMK-TIS-4202<br>ZFMK-DNA-0155628500	Slovakia, Komarno, Imel, PR Liscie diery, N47°55'12" E18°09'39", 114m, 19-Jul-2013, sweeping, leg. Krátky,J., det. Krátky,J.
Hypera meles<br>(Fabricius, 1792)	MK891878 [new]<br>2142-JKR<br>658nt	ZFMK-TIS-23782<br>ZFMK-DNA-0169169591	Slovakia, Komarno, Imel, PR Liscie diery, N47°55'17" E18°09'37", 114m, 18-Mai-2014, sweeping, leg. Krátky,J., det. Krátky,J.
Hypera miles<br>(Paykull, 1792)	KC783940 [new]<br>328-JKR<br>658nt	ZFMK-TIS-20114<br>ZFMK-DNA-0100438755	Czech Republic, Bohemia or., Hradec Kralove - Fararstvi, N50°11'22.247" E15°48'53.64", 230m, 17-Mai-2011, sweeping, leg. Krátky,J., det. Krátky,J.

**Name**<br>**Authority**<br>**Additional Information**	**GenBank Acc No (Ref.)**<br>**Specimen ID**<br>**Sequence Length**	**ZFMK Tissue ID**<br>**ZFMK DNA Sample ID**<br>**(SDEI DNA Sample ID)**	**Locality, GPS, Collection Date, Plant, Collector, Identifier**
Hypera miles<br>(Paykull, 1792)	MK891121 [new]<br>1026-PSP<br>658nt	ZFMK-TIS-3171<br>ZFMK-DNA-0100426833	Germany, Lower Saxony (NI), Koldingen, Hannover region, Leine valley, N52°16'35" E09°25'05", 55m, 19-Mai-2012, *Vicia cracca*, wayside, beating, leg. Sprick,P., det. Sprick,P.
Hypera miles<br>(Paykull, 1792)	MK891377 [new]<br>1429-JKR<br>658nt	ZFMK-TIS-4198<br>ZFMK-DNA-0155628515	Slovakia, Vysoke Tatry Mts., Dolina siedmich prame?ov, N49°13'38" E20°16'34", 1448m, 16-Jul-2013, *Lathyrus pratensis*, ex larvae, leg. Krátky,J., det. Krátky,J.
Hypera nigrirostris<br>(Fabricius, 1775)	MK891194 [new]<br>1111-JKR<br>658nt	ZFMK-TIS-3447<br>ZFMK-DNA-0100449596	Czech Republic, Bohemia, Hradec Kralove, Trebes, N50°11'31" E15°49'10", 227m, 05-Mai-2012, *Trifolium*, beating, leg. Krátky,J., det. Krátky,J.
Hypera nigrirostris<br>(Fabricius, 1775)	MK891852 [new]<br>2114-JKR<br>658nt	ZFMK-TIS-23754<br>ZFMK-DNA-0169169624	Czech Republic, Moravia, Lanzhot, N48°42'37" E16°58'27", 156m, 10-Mai-2014, sweeping, leg. Krátky,J., det. Krátky,J.
Hypera nigrirostris<br>(Fabricius, 1775)	MK891454 [new]<br>1519-PSP<br>658nt	ZFMK-TIS-3279<br>ZFMK-DNA-0155633309	Denmark, Syddanmark, Emmerlev Klev (Højer), N54°59'53" E08°39'08", 3m, 30-Aug-2012, *Trifolium pratense, Trifolium repens*, leg. Sprick,P., det. Sprick,P.
Hypera nigrirostris<br>(Fabricius, 1775)	MK891139 [new]<br>1044-PSP<br>658nt	ZFMK-TIS-3189<br>ZFMK-DNA-0100426894	Germany, Lower Saxony (NI), Hannover, Ahlem, N52°23'00" E09°40'55", 50m, 01-Jun-2012, *Trifolium pratense*, Faboideae-rich ruderal area, dipnet, leg. Sprick,P., det. Sprick,P.
Hypera nigrirostris<br>(Fabricius, 1775)	MK892286 [new]<br>2587-PSP<br>658nt	ZFMK-TIS-23561<br>ZFMK-DNA-0171661975	Germany, Bavaria (BY), Unterfranken, Ebern, N50°05'17" E10°45'53", 343m, 13-Jun-2014, *Trifolium medium*, leg. Sprick,P., det. Sprick,P.
Hypera nigrirostris<br>(Fabricius, 1775)	MK891386 [new]<br>1439-JKR<br>658nt	ZFMK-TIS-4208<br>ZFMK-DNA-0155628494	Slovakia, Komarno, Martovce, Stara Nitra, N47°49'52" E18°08'13", 108m, 20-Jul-2013, sweeping, leg. Krátky,J., det. Krátky,J.
Hypera ononidis<br>(Chevrolat, 1863)<br>Syn. with *H. nigrirostris* pending	MK891247 [new]<br>1177-JKR<br>658nt	ZFMK-TIS-3513<br>ZFMK-DNA-0100426226	Spain, Andalucia, Sierra Nevada, 5 km W of Monachil, N37°08'10" W03°29'13", 1435m, 10-Jun-2012, sweeping, leg. Krátky,J., det. Krátky,J.
Hypera pandellei intermedia<br>Kippenberg, 1983	MK892136 [new]<br>2423-JKR<br>658nt	ZFMK-TIS-26000<br>ZFMK-DNA-0171600645	Slovakia, Tatry Mts., Skalne vrata, N49°13'32" E20°16'37", 1341m, 30-Jun-2013, *Lathyrus pratensis*, collecting by hand, leg. Krátky,J., det. Krátky,J.
Hypera plantaginis<br>(DeGeer, 1775)	MK891776 [new]<br>2035-JKR<br>658nt	ZFMK-TIS-23675<br>ZFMK-DNA-0169170457	Czech Republic, Bohemia, Kosatky, N50°19'23" E14°39'58", 218m, 02-Mai-2014, sieving, leg. Krátky,J., det. Krátky,J.
Hypera plantaginis<br>(DeGeer, 1775)	MK891397 [new]<br>1457-PSP<br>658nt	ZFMK-TIS-3217<br>ZFMK-DNA-0155633372	Germany, Lower Saxony (NI), Berkhof, Hannover region, N52°36'47" E09°43'49", 41m, 30-Jun-2012, *Lotus uliginosus*, humid forest track, dipnet, leg. Sprick,P., det. Sprick,P.
Hypera postica<br>(Gyllenhal, 1813)	MK891455 [new]<br>1520-PSP<br>658nt	ZFMK-TIS-3280<br>ZFMK-DNA-0155633310	Denmark, Syddanmark, Emmerlev Klev (Højer), N54°59'53" E08°39'08", 3m, 30-Aug-2012, *Medicago lupulina*, leg. Sprick,P., det. Sprick,P.
Hypera postica<br>(Gyllenhal, 1813)	KC784143 [new]<br>628-PSP<br>658nt	ZFMK-TIS-20411<br>ZFMK-DNA-0100448634	Germany, Lower Saxony (NI), Brelingen, Hannover region, N52°33'53" E09°40'55", 79m, 25-Jun-2011, *Melilotus albus*, dry-warm ruderal area, beating, leg. Sprick,P., det. Sprick,P.

**Name**<br>**Authority**<br>**Additional Information**	**GenBank Acc No (Ref.)**<br>**Specimen ID**<br>**Sequence Length**	**ZFMK Tissue ID**<br>**ZFMK DNA Sample ID**<br>**(SDEI DNA Sample ID)**	**Locality, GPS, Collection Date, Plant, Collector, Identifier**
Hypera postica<br>(Gyllenhal, 1813)	MK892291 [new]<br>2592-PSP<br>658nt	ZFMK-TIS-23566<br>ZFMK-DNA-0171661967	Germany, Bavaria (BY), Unterfranken, Ebern, N50°05'17" E10°45'53", 343m, 13-Jun-2014, *Medicago lupulina (eng. lucerne)*, leg. Sprick,P., det. Sprick,P.
Hypera postica<br>(Gyllenhal, 1813)	MK347580 [40]<br>3017-KRA<br>658nt	(SDEI-DNA-3017-KRA)<br>3017-KRA_SDEI0111	Portugal, Madeira, Caniçal, ca 1km NW, (Córrego Curralinho), N32°44'49.6" W16°45'00.48", 224m, 28-Mrz-2017, leg. Pelikan, det. Stüben,P.
Hypera rumicis<br>(Linnaeus, 1758)	MK891057 [new]<br>957-CBR<br>658nt	ZFMK-TIS-3870<br>ZFMK-DNA-0100449654	Luxembourg, 10 km E of Luxembourg, 1 km SW of Mensdorf, N49°39'00" E06°17'40", 240m, 21-Jun-2012, *Rumex acetosa*, beating, leg. Braunert,C., det. Braunert,C.
Hypera striata<br>(Boheman, 1834)	KC784100 [new]<br>505-RST<br>658nt	ZFMK-TIS-20290<br>ZFMK-DNA-0100448463	Czech Republic, Moravia mer., Podyji NP, 0.9 km N of Hnanice - Stare vinice hill, N48°48'18.81" E15°59'26.12", 300m, 23-Mai-2011, *dry heathland*, night-sweeping, leg. Stejskal,R., det. Stejskal,R.
Hypera striata<br>(Boheman, 1834)	MK891851 [new]<br>2113-JKR<br>658nt	ZFMK-TIS-23753<br>ZFMK-DNA-0169169623	Czech Republic, Moravia, Lanzhot, N48°42'37" E16°58'27", 156m, 10-Mai-2014, sweeping, leg. Krátky,J., det. Krátky,J.
Hypera subcordicollis<br>(Desbrochers, 1900)<br>Syn. with *H. nigrirostris* pending	MK891573 [new]<br>122-PST<br>658nt	ZFMK-TIS-2D100440312<br>ZFMK-DNA-0155630452	Morocco, S of Berkane, Beni Snassen Mts., Vallee Zegzel, N34°49'26" W02°22'59", 634m, 12-Mai-2011, beating, leg. Stüben,P., det. Behne,L./vid. Winkelmann,H.
Hypera suspiciosa<br>(Herbst, 1795)	MK891854 [new]<br>2116-JKR<br>658nt	ZFMK-TIS-23756<br>ZFMK-DNA-0169169626	Czech Republic, Moravia, Lanzhot, N48°42'37" E16°58'27", 156m, 10-Mai-2014, sweeping, leg. Krátky,J., det. Krátky,J.
Hypera venusta<br>(Fabricius, 1781)	MK891709 [new]<br>1933-FBA<br>658nt	ZFMK-TIS-23324<br>ZFMK-DNA-0169170350	Greece, Rhodes Isl., Laerma N, N36°09'00" E27°56'00", 260m, 26-Apr-2014, leg. Winkelmann,H., det. Winkelmann,H.
Hypera venusta<br>(Fabricius, 1781)	KC784264 [new]<br>IT-0034w<br>656nt	ZFMK-DNA-0100437831	Italy, Sardinia Isl. East, Oliena, Supramonte, N40°15'02" E09°25'17", 1022m, 08-Okt-2010, *Quercus, Verbascum*, cliff, leg. Stüben,P., det. Stüben,P.
Hypera viciae<br>(Gyllenhal, 1813)	MK891506 [new]<br>1592-JKR<br>658nt	ZFMK-TIS-3640<br>ZFMK-DNA-0155635638	Czech Republic, Moravia, Poudrany - step, N48°56'51" E16°38'13", 260m, 01-Mai-2013, *Vicia*, sweeping, leg. Krátky,J., det. Krátky,J.
Hypera viciae<br>(Gyllenhal, 1813)	MK891811 [new]<br>2071-JKR<br>658nt	ZFMK-TIS-23711<br>ZFMK-DNA-0169169662	Czech Republic, Bohemia, Ujezd u Sezemic, N50°07'7.5" E15°51'37", 234m, 08-Mai-2014, sweeping, leg. Krátky,J., det. Krátky,J.
Hypera viciae<br>(Gyllenhal, 1813)	KC784052 [new]<br>580-PSP<br>658nt	ZFMK-TIS-20364<br>ZFMK-DNA-0100448394	Germany, Bavaria (BY), Buckenhof-Uttenreuth, Lkr. Erlangen-Höchstadt, N49°35'23" E11°04'28", 295m, 03-Jun-2011, *Vicia villosa*, edge of field, dipnet, leg. Sprick,P., det. Sprick,P.
Hypophyes minutissimus<br>(Tournier, 1868)	KC783860 [new]<br>155-PST<br>658nt	ZFMK-TIS-2D100440272<br>ZFMK-DNA-0100438160	Morocco, NW of Berkane, Oued Moulouya, N35°00'03" W02°27'30" , 15m, 14-Mai-2011, *Tamarix*, beating, leg. Stüben,P., det. Behne,L.
Hypophyes pallidulus<br>(Gravenhorst, 1807)	KC784278 [new]<br>IT-0021w<br>658nt	ZFMK-TIS-2D100446556	Italy, Sardinia Isl. South, SE of Ballao, F. Flumendosa, N39°31'51" E09°23'27", 75m, 01-Okt-2010, *Tamarix*, leg. Stüben,P., det. Stüben,P.

**Name**<br>**Authority**<br>**Additional Information**	**GenBank Acc No (Ref.)**<br>**Specimen ID**<br>**Sequence Length**	**ZFMK Tissue ID**<br>**ZFMK DNA Sample ID**<br>**(SDEI DNA Sample ID)**	**Locality, GPS, Collection Date, Plant, Collector, Identifier**
Hypophyes pallidulus<br>(Gravenhorst, 1807)	MK890801 [new]<br>IT-0039w<br>658nt	ZFMK-DNA-0100437836	Italy, Sardinia Isl. East, E of Siniscola, La Caletta, coast, N40°35'30" E09°45'25", 3m, 09-Okt-2010, *Tamarix*, leg. Stüben,P., det. Stüben,P.
Hypophyes pallidulus<br>(Gravenhorst, 1807)	MK891310 [new]<br>1299-PST<br>658nt	ZFMK-TIS-4115<br>ZFMK-DNA-0100426168	Spain, Cordoba, Montoro, N38°01'49" W04°22'46", 161m, 04-Mai-2013, *Tamarix*, beating, leg. Stüben,P. & Schütte,A., det. Stüben,P.
Involvulus cupreus<br>(Linnaeus, 1758)	KR817561 [20]<br>1545-PSP<br>658nt	ZFMK-TIS-3305<br>ZFMK-DNA-0155633287	Germany, Lower Saxony (NI), Harz National Park, Torfhaus, Marienbruch, N51°49'46" E10°31'58", 616m, 28-Jun-2013, *Sorbus aucuparia*, beating, leg. Sprick,P., det. Sprick,P.
Involvulus cupreus<br>(Linnaeus, 1758)	MK892207 [new]<br>2503-PSP<br>658nt	ZFMK-TIS-23475<br>ZFMK-DNA-0171661770	Germany, Lower Saxony (NI), Helstorfer Moor, N52°33'08" E09°35'45.5", 43m, 06-Mai-2014, *Sorbus aucuparia*, peatland area, beating, leg. Sprick,P., det. Sprick,P.
Ischnopterapion cf. *plumbeomicans plumbeomicans*	KC784300 [40]<br>731-PST<br>658nt	ZFMK-TIS-3068<br>ZFMK-DNA-0100448157	Spain, Canary Islands, Tenerife, Güimar valley, Arafo near La Hidalgo, roadside, N28°20'23" W16°24'16", 339m, 28-Jan-2012, *Lotus*, beating, leg. Stüben,P., det. Stüben,P.
Ischnopterapion cf. *virens*	KC784258 [new]<br>IT-0028w<br>655nt	ZFMK-TIS-2D100446546<br>ZFMK-DNA-0100437848	Italy, Sardinia Isl. West, E of Macomer, above Silanus, N40°19'20" E08°52'13", 1015m, 04-Okt-2010, *Acer monspessulanum, Quercus, Verbascum*, meadow, leg. Stüben,P., det. Stüben,P.
Ischnopterapion fairmairei<br>(Wencker, 1864)	KC783884 [new]<br>103-PST<br>609nt	ZFMK-TIS-2D100440324<br>ZFMK-DNA-0100438212	Gibraltar, Windmill Hill Flats, Mosaic of low garique and meadow, N36°06'57" W05°20'46", 112m, 30-Mrz-2011, *Lotus longisiliquosus*, beating, leg. Perez,C. & Bensusan,K., det. Behne,L.
Ischnopterapion loti<br>(Kirby, 1808)	KC784127 [new]<br>653-PSP<br>658nt	ZFMK-TIS-20436<br>ZFMK-DNA-0100448611	Germany, Lower Saxony (NI), Hameln-Rohrsen, Dütberg, N52°06'25" E09°24'59", 117m, 03-Okt-2011, *Lotus corniculatus*, dipnet, leg. Sprick,P., det. Sprick,P.
Ischnopterapion loti<br>(Kirby, 1808)	MK891567 [new]<br>941-CBR<br>658nt	ZFMK-TIS-3854<br>ZFMK-DNA-0155630470	Luxembourg, 10 km E of Luxembourg, 1 km SW of Mensdorf, N49°39'32" E06°18'35", 300m, 09-Jun-2012, *Lotus corniculatus*, sweeping, leg. Braunert,C., det. Braunert,C.
Ischnopterapion modestum<br>(Germar, 1817)	KC784159 [new]<br>609-PSP<br>658nt	ZFMK-TIS-20392<br>ZFMK-DNA-0100448663	Germany, Saxony-Anhalt (ST), Drömling, Mannhausen, N52°25'36" E11°12'36.5", 54m, 06-Jul-2011, *Lotus pedunculatus*, body of standing water shore, beating, leg. Sprick,P., det. Sprick,P.
Ischnopterapion modestum<br>(Germar, 1817)	MK891660 [40]<br>1855-PST<br>658nt	ZFMK-TIS-26058<br>ZFMK-DNA-0171606111	Portugal, Madeira, NE of Arco da Calheta, N32°44'37" W17°07'47", 1157m, 02-Jul-2014, *Lotus*, beating, leg. Stüben,P., det. Stüben,P.
Ischnopterapion modestum<br>(Germar, 1817)	MK891672 [40]<br>1877-PST<br>658nt	ZFMK-TIS-26080<br>ZFMK-DNA-0171606089	Portugal, Madeira, SW of Sao Roque de Faial, road to Faja da Nouqueira, N32°44'26" W16°54'04", 582m, 10-Jul-2014, *Lotus*, beating, leg. Stüben,P., det. Stüben,P.
Ischnopterapion modestum<br>(Germar, 1817)	MK891674 [40]<br>1883-PST<br>658nt	ZFMK-TIS-26086<br>ZFMK-DNA-0171606072	Portugal, Madeira, near Ilha, W of Sao Jorge, Achada do Marques, N32°48'18" W16°54'28", 404m, 14-Jul-2014, *Lotus*, beating, leg. Stüben,P., det. Stüben,P.
Ischnopterapion modestum<br>(Germar, 1817)	MK891715 [40]<br>1942-PST<br>658nt	ZFMK-TIS-24021<br>ZFMK-DNA-0169170407	Portugal, Madeira, Faial, coast, N32°47'37" W16°50'57", 26m, 04-Okt-2014, sieving, leg. Stüben,P., det. Stüben,P.

**Name**<br>**Authority**<br>**Additional Information**	**GenBank Acc No (Ref.)**<br>**Specimen ID**<br>**Sequence Length**	**ZFMK Tissue ID**<br>**ZFMK DNA Sample ID**<br>**(SDEI DNA Sample ID)**	**Locality, GPS, Collection Date, Plant, Collector, Identifier**
Ischnopterapion modestum<br>(Germar, 1817)	MK891166 [new]<br>1077-JKR<br>658nt	ZFMK-TIS-3413<br>ZFMK-DNA-0100449103	Spain, Andalucia, Pedro Valiente, playa de Tarifa, N36°02'49" W05°38'26", 3m, 15-Apr-2012, *Lotus creticus*, individual collecting by hand, leg. Krátky,J., det. Krátky,J.
Ischnopterapion plumbeomicans plumbeomicans<br>(Rosenhauer, 1856)	KC783900 [new]<br>101-PST<br>658nt	ZFMK-TIS-2D100438664<br>ZFMK-DNA-0100438404	Gibraltar, Northern Talus Slope, N36°08'34" W05°20'25", 30m, 07-Jun-2011, *Lotus arenarius*, Sandy slope with dune and garique vegetation near coast, beating, leg. Perez,C. & Bensusan,K., det. Stüben,P.
Ischnopterapion plumbeomicans plumbeomicans<br>(Rosenhauer, 1856)	KC783759 [40]<br>48-PST<br>658nt	ZFMK-TIS-2D100447010<br>ZFMK-DNA-0100437948	Spain, Canary Islands, Gran Canaria, Artenaria, Pinar de Tamadaba, roadside, N28°01'49" W15°41'13", 1204m, 13-Jan-2011, *Lotus spartioides* , beating, leg. Stüben,P., det. Stüben,P.
Ischnopterapion plumbeomicans plumbeomicans<br>(Rosenhauer, 1856)	KC783793 [40]<br>84-PST<br>656nt	ZFMK-TIS-2D100446964<br>ZFMK-DNA-0100438036	Spain, Canary Islands, La Gomera, 300 m W of Epina near Alojera, N28°10'00" W17°18'07", 699m, 19-Feb-2011, *Lotus* cf. *glaucus*, beating, leg. Stüben,P., det. Stüben,P.
Ischnopterapion plumbeomicans plumbeomicans<br>(Rosenhauer, 1856)	MK890964 [new]<br>847-PST<br>658nt	ZFMK-TIS-3760<br>ZFMK-DNA-0100414245	Portugal, Estremadura, W of Sintra, Praia de Adraga, coast, N38°48'07" W09°29'04", 14m, 16-Mai-2012, *Lotus*, beating, leg. Stüben,P., det. Stüben,P.
Ischnopterapion plumbeomicans plumbeomicans<br>(Rosenhauer, 1856)	KC784299 [40]<br>728-PST<br>658nt	ZFMK-TIS-3065<br>ZFMK-DNA-0100448154	Spain, Canary Islands, Tenerife, near Los Silos, coast, Playa de Agua Dulce, Sibora, N28°22'40" W16°48'21", 19m, 24-Jan-2012, *Lotus sessilifolius*, beating, leg. Stüben,P., det. Stüben,P.
Ischnopterapion virens<br>(Herbst, 1797)	KC784075 [new]<br>548-RST<br>658nt	ZFMK-TIS-20333<br>ZFMK-DNA-0100448431	Czech Republic, Moravia mer., NP Podyji, 0.6 km NE of Hnanice, N48°48'6.90" E15°59'29.96", 280m, 30-Sep-2011, sweeping, leg. Stejskal,R., det. Stejskal,R.
Ischnopterapion virens<br>(Herbst, 1797)	KC784125 [new]<br>651-PSP<br>658nt	ZFMK-TIS-20434<br>ZFMK-DNA-0100448609	Germany, Lower Saxony (NI), Königsförde, Lkr. Hameln-Pyrmont, N52°03'58" E09°16'17", 92m, 03-Okt-2011, *Trifolium repens*, house garden, gras sifting, leg. Sprick,P., det. Sprick,P.
Ischnopterapion virens<br>(Herbst, 1797)	MK891682 [new]<br>1898-PST<br>658nt	ZFMK-TIS-24003<br>ZFMK-DNA-0169168517	Germany, North Rhine-Westphalia (NRW), Elmpt, Overhetfeld, Dilborner Mühle, N51°13'58" E06°10'22", 52m, 27-Aug-2014, beating, leg. Stüben,P., det. Stüben,P.
Ischnopterapion virens<br>(Herbst, 1797)	MK892321 [new]<br>2622-PSP<br>658nt	ZFMK-TIS-23596<br>ZFMK-DNA-0171661937	Germany, Bavaria (BY), Unterfranken, Ebern, N50°05'20.5" E10°45'59", 333m, 13-Jun-2014, *Trifolium medium*, leg. Sprick,P., det. Sprick,P.
Ischnopterapion virens<br>(Herbst, 1797)	MK890983 [new]<br>874-CBR<br>658nt	ZFMK-TIS-3787<br>ZFMK-DNA-0100413783	Luxembourg, 20 km NE of Luxembourg, 1 km NW of Altrier, N49°45'12" E06°19'10", 370m, 01-Jul-2012, sweeping, leg. Braunert,C., det. Braunert,C.
Isochnus sequensi<br>(Stierlin, 1894)	KC784138 [new]<br>640-PSP<br>658nt	ZFMK-TIS-20423<br>ZFMK-DNA-0100448627	Germany, Saxony-Anhalt (ST), Drömling, Mannhausen, N52°25'37" E11°12'09", 54m, 08-Sep-2011, *Salix fragilis*, beating, leg. Sprick,P., det. Sprick,P.
Isochnus sequensi<br>(Stierlin, 1894)	MK892053 [new]<br>2329-JKR<br>658nt	ZFMK-TIS-24260<br>ZFMK-DNA-0169170947	Slovakia, Tatry Mts., Bujaci vrch, N49°14'10" E20°14'27", 1914m, 06-Jul-2014, *Salix* cf. *alpina*, sweeping, leg. Krátky,J., det. Krátky,J.

**Name**<br>**Authority**<br>**Additional Information**	**GenBank Acc No (Ref.)**<br>**Specimen ID**<br>**Sequence Length**	**ZFMK Tissue ID**<br>**ZFMK DNA Sample ID**<br>**(SDEI DNA Sample ID)**	**Locality, GPS, Collection Date, Plant, Collector, Identifier**
Ixapion variegatum<br>(Wencker, 1864)	KC784074 [new]<br>549-RST<br>658nt	ZFMK-TIS-20334<br>ZFMK-DNA-0100448430	Czech Republic, Moravia mer., Podyji NP, 1.1 km N of Hnanice - Stare vinice hill, N48°48'25.06" E15°59'22.34", 310m, 17-Sep-2011, *Viscum album on Pinus sylvestris*, beating, leg. Stejskal,R., det. Stejskal,R.
Kalcapion fortunatum<br>(Roudier, 1963)<br>formerly: *K. semivittatum fortunatum*	MK347545 [40]<br>2958-PST<br>658nt	(SDEI-DNA-2958-PST)	Spain, Canary Islands, El Hierro, W Frontera, Pista al Derrabado, N27°44'23" W18°02'58", 929m, 11-Apr-2016, leg. Stüben & Schütte, det. Stüben,P.
Kalcapion fortunatum<br>(Roudier, 1963)<br>formerly: *K. semivittatum fortunatum*	MK347600 [40]<br>2959b-PST<br>658nt	(SDEI-DNA-2959b-PST)	Spain, Canary Islands, El Hierro, W Frontera, Pista al Derrabado, N27°44'23" W18°02'58", 929m, 11-Apr-2016, leg. Stüben & Schütte, det. Stüben,P.
Kalcapion fortunatum<br>(Roudier, 1963)<br>formerly: *K. semivittatum fortunatum*	KC783730 [new]<br>2-PST<br>658nt	ZFMK-TIS-2D100446879<br>ZFMK-DNA-0100437993	Spain, Canary Islands, Gran Canaria, Marzagan, Barranco de las Goteras, N28°01'28" W15°27'31", 236m, 01-Dez-2010, *Mercurialis annua*, beating, leg. Stüben,P., det. Stüben,P.
Kalcapion fortunatum<br>(Roudier, 1963)<br>formerly: *K. semivittatum fortunatum*	KC783768 [40]<br>58-PST<br>658nt	ZFMK-TIS-2D100446990<br>ZFMK-DNA-0100438062	Spain, Canary Islands, La Gomera, Hermigua, Las Nuevitas, Barranquillos los Alamos, N28°09'31" W17°11'09", 251m, 13-Dez-2010, *Mercurialis annua*, beating, leg. Stüben,P., det. Stüben,P.
Kalcapion fortunatum<br>(Roudier, 1963)<br>formerly: *K. semivittatum fortunatum*	MK891739 [40]<br>1991-JKR<br>658nt	ZFMK-TIS-23631<br>ZFMK-DNA-0169170509	Spain, Canary Islands, La Palma, Brena Alta, 3 km SW, N28°37'58" W17°48'26.5", 774m, 09-Feb-2014, beating, leg. Krátky,J., det. Krátky,J.
Kalcapion fortunatum<br>(Roudier, 1963)<br>formerly: *K. semivittatum fortunatum*	KC784281 [40]<br>696-PST<br>658nt	ZFMK-TIS-3033<br>ZFMK-DNA-0100448191	Spain, Canary Islands, Tenerife, Santa Barbara, N28°21'37" W16°41'07", 558m, 02-Jan-2012, *Mercurialis*, beating, leg. Stüben,P., det. Stüben,P.
Kalcapion pallipes<br>(Kirby, 1808)	KC784086 [new]<br>535-RST<br>658nt	ZFMK-TIS-20320<br>ZFMK-DNA-0100448445	Czech Republic, Bohemia occ., Belysov Nature Reserve, 2.5 km SE of Chudenice, N49°26'49.54" E13°11'53.68", 540m, 28-Aug-2011, *Mercurialis perennis*, beating, leg. Stejskal,R., det. Stejskal,R.
Kalcapion pallipes<br>(Kirby, 1808)	MK890923 [new]<br>791-PST<br>658nt	ZFMK-TIS-3704<br>ZFMK-DNA-0100414300	Germany, Rhineland-Palatinate (RLP), E of Treis-Karden, Pommern, Pommernbachtal (river valley), N50°10'52" E07°16'42", 116m, 09-Jun-2012, *Mercurialis perennis*, forest path, beating, leg. Stüben,P., det. Stüben,P.
Kalcapion pallipes<br>(Kirby, 1808)	MK891459 [new]<br>1524-PSP<br>658nt	ZFMK-TIS-3284<br>ZFMK-DNA-0155633314	Germany, Lower Saxony (NI), Springe, Hannover region, Kleiner Deister, Burgbergklippen, N52°11'13" E09°33'26", 273m, 09-Sep-2012, *Mercurialis perennis*, dipnet, leg. Sprick,P., det. Sprick,P.
Kalcapion pallipes<br>(Kirby, 1808)	MK892184 [new]<br>2478-PSP<br>658nt	ZFMK-TIS-23450<br>ZFMK-DNA-0171661795	Germany, Saxony-Anhalt (ST), Harz National Park, Ilsenburg, Wienberg, N51°52'17" E10°39'59", 293m, 19-Jul-2013, *Mercurialis perennis*, beating, leg. Sprick,P., det. Sprick,P.
Kalcapion sagittiferum<br>(Wollaston, 1854)<br>formerly: *Kalcapion semivittatum sagittiferum*	MK347537 [40]<br>2942-PST<br>658nt	(SDEI-DNA-2942-PST)	Portugal, Madeira, Ilhas Desertas, Deserta Grande, Doca (ranger station) (6), N32°30'49" W16°30'33", 23m, 22-Mrz-2015, *Mercurialis annua*, hand-collecting, leg. Stüben,P., det. Stüben,P.

**Name**<br>**Authority**<br>**Additional Information**	**GenBank Acc No (Ref.)**<br>**Specimen ID**<br>**Sequence Length**	**ZFMK Tissue ID**<br>**ZFMK DNA Sample ID**<br>**(SDEI DNA Sample ID)**	**Locality, GPS, Collection Date, Plant, Collector, Identifier**
Kalcapion semivittatum<br>(Gyllenhal, 1833)<br>formerly: *K. semivittatum semivittatum*	KC784072 [new]<br>551-RST<br>658nt	ZFMK-TIS-20336<br>ZFMK-DNA-0100448428	Czech Republic, Moravia mer., Vrbovec - Hnizdo env., 0.8 km N of the village, N48°46'46.25" E16°8'5.36", 200m, 03-Sep-2011, *Mercurialis annua*, sweeping, leg. Stejskal,R., det. Stejskal,R.
Kalcapion semivittatum<br>(Gyllenhal, 1833)<br>formerly: *Kalcapion semivittatum sagittiferum*	MK892364 [40]<br>2775-PST<br>658nt	ZFMK-TIS-26129<br>ZFMK-DNA-0169166967	Portugal, Madeira, Ilhas Desertas, Deserta Grande, Doca, N32°30'49" W16°30'33", 23m, 22-Mrz-2015, *Mercurialis annua*, collecting by hand, leg. Stüben,P., det. Stüben,P.
Kalcapion semivittatum<br>(Gyllenhal, 1833)<br>formerly: *K. semivittatum semivittatum*	MK347531 [40]<br>2930-PST<br>658nt	(SDEI-DNA-2930-PST)	Spain, Canary Islands, Lanzarote, above Haria, N29°08'08" W13°30'22", 358m, 04-Jan-2017, *Mercurialis*, beating, leg. Stüben,P., det. Stüben,P.
Kalcapion semivittatum<br>(Gyllenhal, 1833)<br>formerly: *K. semivittatum semivittatum*	MK891649 [40]<br>1825-PST<br>658nt	ZFMK-TIS-26028<br>ZFMK-DNA-0171606141	Portugal, Madeira, S of Seixal, S of Chao da Ribeira, N32°48'41" W17°06'38", 411m, 27-Jun-2014, beating, leg. Stüben,P., det. Stüben,P.
Kalcapion semivittatum<br>(Gyllenhal, 1833)<br>formerly: *K. semivittatum semivittatum*	MK891665 [40]<br>1863-PST<br>658nt	ZFMK-TIS-26066<br>ZFMK-DNA-0171606100	Portugal, Madeira, Paul do Mar, N32°45'34" W17°13'43", 62m, 02-Jul-2014, *Ficus*, sieving, leg. Stüben,P., det. Stüben,P.
Kalcapion semivittatum<br>(Gyllenhal, 1833)<br>formerly: *K. semivittatum semivittatum*	MK891673 [40]<br>1880-PST<br>658nt	ZFMK-TIS-26083<br>ZFMK-DNA-0171606086	Portugal, Madeira, Roque de Faial, Cruzinhas, N32°45'36" W16°52'30.5", 434m, 10-Jul-2014, beating, leg. Stüben,P., det. Stüben,P.
Kalcapion semivittatum<br>(Gyllenhal, 1833)<br>formerly: *K. semivittatum semivittatum*	MK891718 [40]<br>1945-PST<br>658nt	ZFMK-TIS-24024<br>ZFMK-DNA-0169168540	Portugal, Madeira, near Ilha, W of Sao Jorge, Achada do Marques, N32°47'43" W16°54'32", 395m, 04-Okt-2014, *Mercurialis*, beating, leg. Stüben,P., det. Stüben,P.
Kalcapion semivittatum<br>(Gyllenhal, 1833)<br>formerly: *K. semivittatum semivittatum*	MK891722 [40]<br>1953-PST<br>658nt	ZFMK-TIS-24032<br>ZFMK-DNA-0169170396	Portugal, Madeira, S of Seixal, S of Chao da Ribeira, N32°48'28" W17°06'55", 435m, 05-Okt-2014, *gardens*, beating, leg. Stüben,P., det. Stüben,P.
Kalcapion semivittatum<br>(Gyllenhal, 1833)<br>formerly: *K. semivittatum semivittatum*	MK892375 [40]<br>2827-PST<br>658nt	ZFMK-TIS-25874<br>ZFMK-DNA-FC17941477	Portugal, Madeira, N of Santana, Quebrada, N32°48'49" W16°52'27", 124m, 25-Nov-2015, *Mercurialis*, beating, leg. Stüben,P. & Schütte,A., det. Stüben,P.
Kalcapion semivittatum<br>(Gyllenhal, 1833)<br>formerly: *K. semivittatum semivittatum*	KC783897 [new]<br>128-PST<br>639nt	ZFMK-TIS-2D100440299<br>ZFMK-DNA-0100438380	Morocco, S of Berkane, Beni Snassen Mts., Vallee Zegzel, N34°48'56" W02°24'01", 678m, 12-Mai-2011, *Mercurialis*, beating, leg. Stüben,P., det. Stüben,P./vid. Behne,L.
Kyklioacalles abstersus<br>(Boheman, 1837)<br>formerly: *K. roboris* - Resyn!	GU981483 [01]<br>D-0291-rob<br>658nt	ZFMK-TIS-cD0291<br>ZFMK-TIS-cD0291	Germany, Baden-Wuerttemberg (BW), Kaiserstuhl, "Auf dem Eck", N48°06'32" E07°40'04", 432m, 27-Mai-2006, *Fagus, Carpinus, Quercus*, leg. Stüben,P., det. Stüben,P.

**Name**<br>**Authority**<br>**Additional Information**	**GenBank Acc No (Ref.)**<br>**Specimen ID**<br>**Sequence Length**	**ZFMK Tissue ID**<br>**ZFMK DNA Sample ID**<br>**(SDEI DNA Sample ID)**	**Locality, GPS, Collection Date, Plant, Collector, Identifier**
Kyklioacalles abstersus<br>(Boheman, 1837)<br>formerly: *K. roboris* - Resyn!	KC783825 [23]<br>106-PST<br>658nt	ZFMK-TIS-2D100440321<br>ZFMK-DNA-0100417886	Germany, Baden-Wuerttemberg (BW), Kaiserstuhl, „Auf dem Eck“, S of Kiechlinsbergen, N48°06'32“ E07°40'04“, 432m, 07-Aug-2011, *Quercus (Fagus)*, sieving, leg. Stüben,P., det. Stüben,P.
Kyklioacalles abstersus<br>(Boheman, 1837)<br>formerly: *K. roboris* - Resyn!	GU981484 [01]<br>SLO-0293-rob<br>658nt	ZFMK-TIS-cSLO0293<br>ZFMK-DNA-0100400468	Slovenia, Sveta Ana env., 20 km NE of Maribor, N46°39' E15°50', 05-Aug-2006, *Fagus, Quercus*, leg. Stejskal,R., det. Stejskal,R.
Kyklioacalles abstersus<br>(Boheman, 1837)<br>formerly: *K. roboris* - Resyn!	KJ867572 [23]<br>464-PST<br>658nt	ZFMK-TIS-c464-PST<br>ZFMK-DNA-0100449386	Slovenia, Sveta Ana env., 20 km NE of Maribor, N46°39' E15°50', 285m, 05-Aug-2006, forest with Fagus and Quercus, sieving, leg. Stejskal,R., det. Stüben,P.
Kyklioacalles abstersus<br>(Boheman, 1837)<br>formerly: *K. roboris* - Resyn!	KJ867573 [23]<br>466-PST<br>658nt	ZFMK-TIS-20251<br>ZFMK-DNA-0100449373	Slovenia, Kamnisko-Savinjske Alpe, Zgornje Jezersko, N46°23' E14°30', 888m, 15-Aug-2007, leg. Stejskal,R., det. Stüben,P.
Kyklioacalles abstersus<br>(Boheman, 1837)<br>formerly: *K. roboris* - Resyn!	MG229824 [new]<br>2895-PST<br>658nt	ZFMK-TIS-25901<br>ZFMK-DNA-0169166899	Slovenia, N of Magozd to Trnova near Soca river, N46°16'54" E13°33'43", 347m, 04-Jul-2016, sifting, leg. Stüben,P, det. Stüben,P.
Kyklioacalles alcornocalensis<br>Stüben, Torres, Astrin, 2011<br>**Paratype (DNAtype)**	JN121401 [03]<br>ES1001<br>658nt	ZFMK-TIS-cES1001<br>ZFMK-DNA-0112704668	Spain, Los Barrios (Cadiz), Parque Natural Alcarnocales, Zona Bacinete, Ventarrillo Blanco (ANTIGUO), 36°12'28"N 05°33'24'W, 01-Okt-2009, leg. Torres,J. L., det. Torres,J. L.
Kyklioacalles anthyllis<br>Stüben, 2004<br>**Paratype (DNAtype)**	EU286456 [01]<br>E-0079-ant<br>658nt	ZFMK-TIS-cE0079<br>ZFMK-DNA-0100400715	Spain, Andalucia, S. de Cazorla, 9 km S of Cazorla, N37°50'23" W02°59'26", 1577m, 15-Apr-2003, *Erinacea anthyllis*, leg. Stüben,P., det. Stüben,P.
Kyklioacalles anthyllis<br>Stüben, 2004	GU981471 [01]<br>E-0074-paa<br>658nt	ZFMK-TIS-cE0074<br>ZFMK-DNA-0100400720	Spain, Andalucia, 20 km NW of Laujar, Sierra Nevada, N of Puerto de la Ragua, N37°07'39" W03°02'14", 1921m, 12-Okt-2005, broom *under Pinus*, leg. Stüben,P., det. Stüben,P.
Kyklioacalles anthyllis<br>Stüben, 2004	GU981494 [01]<br>E-597-ant<br>658nt	ZFMK-TIS-cE597<br>ZFMK-DNA-0100404946	Spain, Jaen, Cazorla, El Chorro, N37°50'25" W02°59'24", 1559m, 12-Jun-2008, *Erinacea anthyllis*, leg. Astrin,J., det. Stüben,P.
Kyklioacalles anthyllis<br>Stüben, 2004	GU987779 [01]<br>E-0075-paa<br>658nt	ZFMK-TIS-cE0075<br>ZFMK-DNA-0100400895	Spain, Andalucia, 14 km NW of Laujar, Sierra Nevada, N37°05'14" W02°59'47", 2283m, 15-Okt-2005, *Erinacea anthyllis under Pinus*, leg. Stüben,P., det. Stüben,P.
Kyklioacalles anthyllis<br>Stüben, 2004	GU987780 [01]<br>E-0078-ant<br>658nt	ZFMK-TIS-cE0078<br>ZFMK-DNA-0100400872	Spain, Andalucia, 26 km E of Jaen, Sierra Magina, N37°44'06" W03°30'09", 1663m, 06-Okt-2005, *Erinacea anthyllis under Pinus*, leg. Stüben,P., det. Stüben,P.
Kyklioacalles anthyllis<br>Stüben, 2004	GU987947 [01]<br>E-598-ant<br>658nt	ZFMK-TIS-cE598<br>ZFMK-DNA-0100404945	Spain, Jaen, Cazorla, spring near El Chorro, N37°50'28" W02°59'11", 1449m, 12-Jun-2008, *Erinacea anthyllis*, leg. Astrin,J., det. Stüben,P.
Kyklioacalles apogeus<br>(Peyerimhoff, 1925)	GU981516 [01]<br>Mo-950-apo<br>658nt	ZFMK-TIS-cMo950<br>ZFMK-DNA-0100405246	Morocco, High Atlas, 14 km E of Asni, "Oukaimeden", N312°12'13" W07°50'29", 2300m, 27-Dez-2002, *Erinacea anthyllis*, leg. Stüben,P., det. Stüben,P.
Kyklioacalles astragali<br>Stüben, 2003<br>**Paratype (DNAtype)**	EU286455 [01]<br>E-0077-ast<br>658nt	ZFMK-TIS-cE0077<br>ZFMK-DNA-0100400717	Spain, Granada, Sierra de Baza, Santa Barbara, N37°22'09" W02°50'55", 2200m, 21-Apr-2001, *Astragalus granatensis*, leg. Stüben,P., det. Stüben,P.
Kyklioacalles astragali<br>Stüben, 2003	GU981472 [01]<br>E-0076-ast<br>658nt	ZFMK-TIS-cE0076<br>ZFMK-DNA-0100400871	Spain, Andalucia, 9 km N of Laujar, Sierra Nevada, N37°04'00" W02°55'50", 2055m, 15-Okt-2005, *Erinacea anthyllis under Pinus, Bupleurum*, leg. Stüben,P., det. Stüben,P.

**Name**<br>**Authority**<br>**Additional Information**	**GenBank Acc No (Ref.)**<br>**Specimen ID**<br>**Sequence Length**	**ZFMK Tissue ID**<br>**ZFMK DNA Sample ID**<br>**(SDEI DNA Sample ID)**	**Locality, GPS, Collection Date, Plant, Collector, Identifier**
Kyklioacalles atlasicus<br>Stüben & Astrin, 2010	GU981513 [01]<br>E-887-ats<br>658nt	ZFMK-TIS-cE887<br>ZFMK-DNA-0100405513	Morocco, High Atlas, E of Marrakech, N of Taddert, (near Tazouguerte), N31°28'18" W07°24'21", 1498m, 03-Mai-2009, *Quercus*, leg. Stüben,P., det. Stüben,P.
Kyklioacalles atlasicus<br>Stüben & Astrin, 2010<br>**Paratype (DNAtype)**	GU981514 [01]<br>E-888-ats<br>658nt	ZFMK-TIS-cE888<br>ZFMK-DNA-0100404574	Morocco, High Atlas, E of Marrakech, N of Taddert, (near Tazouguerte), N31°28'07" W07°24'59", 1727m, 03-Mai-2009, *Quercus*, leg. Stüben,P., det. Stüben,P.
Kyklioacalles aubei<br>(Boheman, 1837)	MG229780 [new]<br>2299-JKR<br>658nt	ZFMK-TIS-24230<br>ZFMK-DNA-0169170965	Austria, Kaernten, Feistritz ob Bleiburg, N46°32'17" E14°46'28", 702m, 28-Jun-2014, mountain forest, sieving, leg. Krátky,J., det. Krátky,J.
Kyklioacalles aubei<br>(Boheman, 1837)	EU286503 [01]<br>F-0226-aub<br>658nt	ZFMK-TIS-cF0226<br>ZFMK-DNA-0100400115	France, Isere, 4 km E of La Chapelle-de-Surieu, N45°24'18" E04°56'26", 325m, 09-Apr-2006, *Fagus, Alnus*, leg. Stüben,P., det. Stüben,P.
Kyklioacalles aubei<br>(Boheman, 1837)	GU981487 [01]<br>F-436-aub<br>595nt	ZFMK-TIS-cF436<br>ZFMK-DNA-0100400662	France, Alpes-Maritimes, 9 km N of Sospel, N of Moulinet, N43°57'58" E07°24'55", 1111m, 24-Dez-2007, *Castanea sativa*, leg. Stüben,P., det. Stüben,P.
Kyklioacalles aubei<br>(Boheman, 1837)	GU987757 [01]<br>I-0018-aub<br>658nt	ZFMK-TIS-cI0018<br>ZFMK-DNA-0100400933	France, Isere, 9 km N of Grenoble, Mont St. Martin, N45°16'15" E05°40'44", 813m, 12-Aug-2005, *Fagus, Fraxinus, Quercus*, limestone, leg. Stüben,P., det. Stüben,P.
Kyklioacalles aubei<br>(Boheman, 1837)	GU987775 [01]<br>I-0009-aub<br>658nt	ZFMK-TIS-cI0009<br>ZFMK-DNA-0100400881	France, Isere, 19 km S of Bourgoin, N of Semons, N45°26'01" E05°12'06", 448m, 08-Aug-2005, *Quercus, Fagus*, leg. Stüben,P., det. Stüben,P.
Kyklioacalles aubei<br>(Boheman, 1837)	GU987807 [01]<br>I-0039-aub<br>658nt	ZFMK-TIS-cI0039<br>ZFMK-DNA-0100400129	France, Isere, 14 km N of Grenoble, Massif de la Chartreus, NW of Col de Porte, N45°18'40" E05°45'17", 1649m, 13-Aug-2005, *Abies, Fagus, Fraxinus*, limestone (tree line), leg. Stüben,P., det. Stüben,P.
Kyklioacalles aubei<br>(Boheman, 1837)	GU988092 [01]<br>I-826-aub<br>658nt	ZFMK-TIS-cI826<br>ZFMK-DNA-0100404522	Italy, Sicilia Isl., Monte Soro, N37°55' E14°41', 1830m, 05-Feb-2008, *Fagus*, leg. Kapp, det. Kapp
Kyklioacalles aubei<br>(Boheman, 1837)	MG229778 [new]<br>2022-JKR<br>658nt	ZFMK-TIS-23662<br>ZFMK-DNA-0169170479	Italy, Sicilia Isl., Messina, Nebrodi NP, Monte Soro, N37°55'28" E14°39'57", 1563m, 20-Apr-2014, sieving, leg. Krátky,J., det. Krátky,J.
Kyklioacalles aubei<br>(Boheman, 1837)	GU987969 [01]<br>I-624-aub<br>658nt	ZFMK-TIS-cI624<br>ZFMK-DNA-0100404919	Italy, Campania, Cilento, 6 km SE of Vallo d. Lucania, M. Sacro o Gelbison, N40°12'41" E15°19'42", 1544m, 30-Jun-2008, *Fagus*, leg. Stüben,P., det. Stüben,P.
Kyklioacalles aubei<br>(Boheman, 1837)	MG229825 [new]<br>2896-PST<br>658nt	ZFMK-TIS-25902<br>ZFMK-DNA-0169166898	Italy, E of Stella Nevea, N46°23'55" E13°30'18", 1031m, 30-Jun-2016, *Fagus*, sifting, leg. Stüben,P, det. Stüben,P.
Kyklioacalles aubei<br>(Boheman, 1837)	EU286502 [01]<br>SLO-0224-aub<br>658nt	ZFMK-TIS-cSLO0224<br>ZFMK-DNA-0100400010	Slovenia, Sv. Ana, wnv., 20 km NE of Maribor, N46°39' E15°50', 05-Aug-2006, *Fagus, Quercus*, leg. Stejskal,R., det. Stejskal,R.
Kyklioacalles aubei<br>(Boheman, 1837)	GU987812 [01]<br>SLO-0225-aub<br>658nt	ZFMK-TIS-cSLO0225<br>ZFMK-DNA-0100400084	Slovenia, Kamnisko-Savinjske Alpe, Zg. Jezersko, Zg. Jezersko env., N46°23' E14°27', 02-Aug-2006, *Fagus*, leg. Stejskal,R., det. Stejskal,R.
Kyklioacalles bupleuri<br>Stüben, 2004	EU286486 [01]<br>E-0161-bup<br>658nt	ZFMK-TIS-cE0161<br>ZFMK-DNA-0100400042	Spain, Andalucia, 35 km E of Jaen, Sierra Magina, N37°45'53" W03°23'19", 1328m, 07-Okt-2005, *Euphorbia nicaensis*, limestone, leg. Stüben,P., det. Stüben,P.

**Name**<br>**Authority**<br>**Additional Information**	**GenBank Acc No (Ref.)**<br>**Specimen ID**<br>**Sequence Length**	**ZFMK Tissue ID**<br>**ZFMK DNA Sample ID**<br>**(SDEI DNA Sample ID)**	**Locality, GPS, Collection Date, Plant, Collector, Identifier**
Kyklioacalles bupleuri<br>Stüben, 2004	GU981482 [01]<br>E-0218-bup<br>630nt	ZFMK-TIS-cE0218<br>ZFMK-DNA-0100400264	Spain, Cadiz, 14 km N of Ubrique, Sierra Zafalgar, Puerto de las Palomas, N36°47'16" W05°22'36", 1286m, 14-Apr-2007, broom, *Bupleurum, Euphorbia nicaensis*, leg. Astrin,J. & Stüben,P., det. Stüben,P.
Kyklioacalles bupleuri<br>Stüben, 2004	KJ867611 [new]<br>1302-PST<br>658nt	ZFMK-TIS-4118<br>ZFMK-DNA-0100426171	Spain, Jaen, SE of Mancha Real, Sierra Almaden (El Almaden, **type locality**), N37°44'06" W03°31'30", 1963m, 15-Mai-2013, *Euphorbia nicaeensis*, sieving, leg. Stüben,P. & Schütte,A., det. Stüben,P.
Kyklioacalles bupleuri<br>Stüben, 2004	KJ867615 [new]<br>1322-PST<br>658nt	ZFMK-TIS-4138<br>ZFMK-DNA-0100426143	Spain, Granada, E of Baul, Sierra de Baza, Santa Barbara, N37°23'38" W02°49'47", 1787m, 17-Mai-2013, *Euphorbia nicaensis*, sieving, leg. Stüben,P. & Schütte,A., det. Stüben,P.
Kyklioacalles bupleuri<br>Stüben, 2004	KJ867617 [new]<br>1326-PST<br>658nt	ZFMK-TIS-4142<br>ZFMK-DNA-0100426147	Spain, Jaen, Sierra Magina, SE of Torres, N37°44'46" W03°29'53", 1527m, 19-Mai-2013, *Erinacea anthyllis, Quercus ilex, Astragalus* sp., *Bupleurum*, sieving, leg. Stüben,P. & Schütte,A., det. Stüben,P.
Kyklioacalles cf. *aubei*	MG229797 [new]<br>2752-PST<br>658nt	ZFMK-TIS-3980<br>ZFMK-DNA-0171661908	Russia, Krasnodar, Chvizhepse vill. env. , N43°38'32" E40°04'45", 300m, 10-Jul-2014, leg. Kovalev,A.V., det. Stüben,P.
Kyklioacalles cf. *aubei*	MG229799 [new]<br>2760-PST<br>658nt	ZFMK-TIS-26857<br>ZFMK-DNA-0171661903	Russia, Krasnodar, Chvizhepse vill. env. , N43°38'32" E40°04'45", 300m, 15-Jul-2014, leg. Kovalev,A.V., det. Stüben,P.
Kyklioacalles cf. *bupleuri*	GU987817 [01]<br>E-0178-bup<br>658nt	ZFMK-TIS-cE0178<br>ZFMK-DNA-0100400027	Spain, Alicante, 6,5 NW of Callosa d'En Sarria Sarrià, Sierra de Aixorta, N38°41'59" W00°10'34", 1091m, 04-Apr-2007, *Erinacea anthyllis, Quercus ilex*, limestone, leg. Astrin,J. & Stüben,P., det. Stüben,P.
Kyklioacalles cf. *erinaceus*	EU286496 [01]<br>E-0181-eri<br>602nt	ZFMK-TIS-cE0181<br>ZFMK-DNA-0100400124	Spain, Alicante, 7 km SW of Alcoi, Sierra de Menechaor, Santuario de la Font Roja, N38°39'34" W00°32'29", 296m, 05-Apr-2007, *Erinacea anthyllis*, limestone, leg. Astrin,J. & Stüben,P., det. Stüben,P.
Kyklioacalles cf. *olcesei*	GU981515 [01]<br>E-898-olc<br>658nt	ZFMK-TIS-cE898<br>ZFMK-DNA-0100405493	Morocco, Sebta West, vir. Biutz, N35°53'04" W05°24'08", 337m, 09-Mai-2009, *Quercus suber, Smilax, Arbutus*, leg. Stüben,P., det. Stüben,P.
Kyklioacalles cf. *olcesei*	GU981502 [01]<br>E-774-olc<br>658nt	ZFMK-TIS-cE774<br>ZFMK-DNA-0100405048	Spain, Malaga, N of Marbella, PN of de la Sierra de la Nieves, N36°35'58" W04°55'03", 394m, 16-Jan-2009, *Olea*, Tiefengesiebe, leg. Stüben,P., det. Stüben,P.
Kyklioacalles characivorus<br>Stüben, 2005	EU286485 [01]<br>I-0159-cha<br>658nt	ZFMK-TIS-cI0159<br>ZFMK-DNA-0100400250	Italy, Sardinia Isl., 15 km SW of Lanusei, Barbagia Seulo, Monte Arqueri, N39°49'36" E09°22'27", 960m, 23-Mrz-2005, *Euphorbia characias*, limestone, leg. Stüben,P., det. Stüben,P.
Kyklioacalles chaudoiri<br>(Hochhuth 1847)	MG229798 [new]<br>2758-PST<br>658nt	ZFMK-TIS-26855<br>ZFMK-DNA-0171661901	Russia, Krasnodar, Krasnaya Polyana env., N43°41'17" E40°12'20", 720m, 22-Jul-2014, leg. Kovalev,A.V., det. Stüben,P.
Kyklioacalles erinaceus<br>Stüben, 2003	GU981493 [01]<br>E-595-ks3<br>658nt	ZFMK-TIS-cE595<br>ZFMK-DNA-0100404316	Spain, Jaen, Cazorla, El Chorro, N37°50'25" W02°59'24", 1559m, 12-Jun-2008, *Erinacea anthyllis*, leg. Astrin,J., det. Stüben,P.
Kyklioacalles erinaceus<br>Stüben, 2003	KJ867609 [28]<br>1298-PST<br>658nt	ZFMK-TIS-4114<br>ZFMK-DNA-0100426167	Spain, Jaen, Sierra de Cazorla, N of Arroyo Frio, N38°00'13" W02°53'10", 762m, 14-Mai-2013, *Quercus ilex, Smilax*, sieving, leg. Stüben,P. & Schütte,A., det. Stüben,P.
Kyklioacalles erinaceus<br>Stüben, 2003	KJ867613 [new]<br>1314-PST<br>658nt	ZFMK-TIS-4130<br>ZFMK-DNA-0100426411	Spain, Jaen, Sierra de Cazorla, S of Cazorla, E of Quesada, N37°50'53" W03°00'10", 1329m, 16-Mai-2013, *Quercus ilex*, sieving, leg. Stüben,P. & Schütte,A., det. Stüben,P.

**Name**<br>**Authority**<br>**Additional Information**	**GenBank Acc No (Ref.)**<br>**Specimen ID**<br>**Sequence Length**	**ZFMK Tissue ID**<br>**ZFMK DNA Sample ID**<br>**(SDEI DNA Sample ID)**	**Locality, GPS, Collection Date, Plant, Collector, Identifier**
Kyklioacalles euphorbiophilus<br>Stüben, 2003	EU286513 [01]<br>M-0160-eup<br>658nt	ZFMK-TIS-cM0160<br>ZFMK-DNA-0100400388	Morocco, High Atlas, 56 km SW of Asni, "Tizi-n-Test", N30°52'25" W08°21'26", 2000m, 25-Dez-2002, *Euphorbia nicaeensis*, leg. Stüben,P., det. Stüben,P.
Kyklioacalles euphorbiophilus<br>Stüben, 2003	GU981511 [01]<br>E-879-eup<br>658nt	ZFMK-TIS-cE879<br>ZFMK-DNA-0100404523	Morocco, High Atlas, N of Tizi-n-Test, Tachguette, N30°52'25" W08°21'25", 2047m, 30-Apr-2009, *Euphorbia nicaeensis, Quercus ilex,* broom, leg. Stüben,P., det. Stüben,P.
Kyklioacalles euphorbiophilus<br>Stüben, 2003	MG229715 [new]<br>1582-JKR<br>658nt	ZFMK-TIS-3630<br>ZFMK-DNA-0155635647	Morocco, Haut Atlas, Tizi N of Test, N30°52'22" W08°21'27", 2092m, 17-Apr-2013, *Fabaceae*, individual collecting by hand, leg. Krátky,J., det. Krátky,J.
Kyklioacalles fausti<br>(Meyer, 1896)	MG322649 [new]<br>IT1048<br>658nt	ZFMK-TIS-cIT1048<br>ZFMK-DNA-0112704621	Italy, Sicilia Isl., Madonie Castelbuono Refugio, Crispi Agrifol. Piano Pomo, 37°53'46"N 14°04'01"E, 1400m, 07-Feb-2008, *Stechlaub*, leg. Kapp, det. Kapp
Kyklioacalles fausti<br>(Meyer, 1896)	GU213772 [04]<br>I-625-fau<br>658nt	ZFMK-TIS-cI625<br>ZFMK-DNA-0100404918	Italy, Campania, Cilento, 6 km SE of Vallo d. Lucania, M. Sacro o Gelbison, N40°12'41" E15°19'42", 1544m, 30-Jun-2008, *Fagus*, leg. Stüben,P., det. Stüben,P.
Kyklioacalles fausti<br>(Meyer, 1896)	GU981500 [01]<br>I-659-fau<br>658nt	ZFMK-TIS-cI659<br>ZFMK-DNA-0100404320	Italy, Abruzzo, P.N. Majella, 11 km N of Roccaraso, Bosco di S. Antonio, N41°56'27" E14°01'41", 1321m, 14-Jul-2008, *Pyrus/Acer*, old forest, leg. Stüben,P., det. Stüben,P.
Kyklioacalles fissicollis<br>(Penecke, 1926)	MG229811 [new]<br>2813a-FBA<br>658nt	ZFMK-TIS-3317<br>ZFMK-DNA-FC17941539	Greece, Euboia Isl., Evia (cent.), Mt. Dirfis, 5.2 km SW of Stropones, N38°36'00" E23°51'00", 648m, 05-Jun-2015, sieving, leg. Bahr,F., det. Bahr,F.
Kyklioacalles flavomaculatus<br>Stüben, 2012	JQ975013 [06]<br>195-PST<br>658nt	ZFMK-TIS-2D100440241<br>ZFMK-DNA-0100438129	Morocco, Atlas Mts., N of Imouzzer-des-Marmoucha, Jbel Bou Iblane, Tizi-Bou-Zabel, N33°38'44" W04°09'18", 2275m, 21-Mai-2011, *from Bupleurum* sp., sieving, leg. Stüben,P., det. Stüben,P.
Kyklioacalles flavomaculatus<br>Stüben, 2012	KC783824 [new]<br>185-PST<br>658nt	ZFMK-TIS-2D100440231<br>ZFMK-DNA-0100417617	Morocco, Atlas Mts., N of Imouzzer-des-Marmoucha, Jbel Bou Iblane, Tizi-Bou-Zabel, N33°38'44” W04°09'18”, 2275m, 21-Mai-2011, *Bupleurum*, sieving, leg. Stüben,P., det. Stüben,P.
Kyklioacalles granulicollis<br>(Tournier, 1875)<br>**Progenophore of Neotype***<br>**(DNAtype)**	KX950817 [24]<br>2761-PST_26210<br>658nt	ZFMK-TIS-26210<br>ZFMK-DNA-0171661904	Morocco, Rif. Mts., Talassemtane, Parc Nat. Sapiniere, N35°07'04" W05°11'50", 873m, 19-Jun-2014, leg. Brustel, det. Stüben,P., *Note: before neotype designation the specimen was identified as *Kyklioacalles bupleuri*. 2761-PST_26210 was not used for neotype designation of *Acalles granulicollis*, but derived from the same series as the neotype. The classification 'progenophore' means a voucher that is linked to the specimen sampled for molecular analysis by a parent-descendant or sibling relationship. Please refer to Stüben, P. & Bayer, C. (2015): New nomenclatural and taxonomic acts, and Comments - Snudebiller 16(246): 1-8.
Kyklioacalles igualeja<br>Stüben, 2012	GU981479 [01]<br>E-0210-ks1<br>658nt	ZFMK-TIS-cE0210<br>ZFMK-DNA-0100400315	Spain, Malaga, 11 km NW of Estepona, Sierra Crestellina, Casares, N36°27'06" W05°16'39", 359m, 12-Apr-2007, *Pistacia lentiscus, Olea, Smilax*, stream valley, leg. Astrin,J. & Stüben,P., det. Stüben,P.
Kyklioacalles igualeja<br>Stüben, 2012	JN121402 [03]<br>ES1058<br>658nt	ZFMK-TIS-cES1058<br>ZFMK-DNA-0112704600	Spain, Malaga, S of Ronda, Serrania de Ronda, Igualeja, 36°37'33”N 05°07'27”W, 647m, 21-Aug-2010, *Ficus carica (Wurzelgesiebe)*, leg. Stüben,P., det. Stüben,P.
Kyklioacalles maroccensis<br>(Stüben, 2001)	GU213773 [04]<br>E-878-mrc<br>658nt	ZFMK-TIS-cE878<br>ZFMK-DNA-0100404561	Morocco, Anti-Atlas, E of Tiznit, Anezi, "El Tnine", N29°44'27" W09°15'25", 612m, 29-Apr-2009, *Agania spinosa, Ceratonia siliqua* , leg. Stüben,P., det. Stüben,P.

**Name**<br>**Authority**<br>**Additional Information**	**GenBank Acc No (Ref.)**<br>**Specimen ID**<br>**Sequence Length**	**ZFMK Tissue ID**<br>**ZFMK DNA Sample ID**<br>**(SDEI DNA Sample ID)**	**Locality, GPS, Collection Date, Plant, Collector, Identifier**
Kyklioacalles navieresi<br>(Boheman, 1837)	EU286450 [01]<br>I-0007-nav<br>658nt	ZFMK-TIS-cI0007<br>ZFMK-DNA-0100400944	France, Isere, 19 km S of Bourgoin, N of Semons, N45°26'01" E05°12'06", 448m, 08-Aug-2005, *Quercus, Fagus*, leg. Stüben,P., det. Stüben,P.
Kyklioacalles navieresi<br>(Boheman, 1837)	GU987759 [01]<br>I-0013-nav<br>658nt	ZFMK-TIS-cI0013<br>ZFMK-DNA-0100400759	France, Isere, 12 km SW of Bourgoin, N of St. Jean de Bournay, N45°31'10" E05°10'01", 432m, 09-Aug-2005, *Quercus* , leg. Stüben,P., det. Stüben,P.
Kyklioacalles navieresi<br>(Boheman, 1837)	GU987811 [01]<br>I-0031-nav<br>658nt	ZFMK-TIS-cI0031<br>ZFMK-DNA-0100400025	France, Isere, 2 km SE of Lans en Vercors, Montagne de Lans, N45°06'45" E05°36'21", 1352m, 12-Aug-2005, *Abies, Fagus, Fraxinus*, limestone: mixed forest, leg. Stüben,P., det. Stüben,P.
Kyklioacalles navieresi<br>(Boheman, 1837)	GU981501 [01]<br>D-667-nav<br>658nt	ZFMK-TIS-cD667<br>ZFMK-DNA-0100404818	Germany, Ruegen Isl. (MV), Sassnitz near Rusewase, N54°32'29" E13°37'46", 144m, 15-Aug-2008, *Fagus*, leg. Astrin,J., det. Stüben,P.
Kyklioacalles navieresi<br>(Boheman, 1837)	GU987768 [01]<br>M-0046-nav<br>658nt	ZFMK-TIS-cM0046<br>ZFMK-DNA-0100400925	Germany, Rhineland-Palatinate (RLP), Moselle valley, Cochem, Dekernbach, mountain Wakelay, N50°09'31" E07°09'09", 301m, 15-Sep-2005, *Quercus, Carpinus*, leg. Stüben,P., det. Stüben,P.
Kyklioacalles navieresi<br>(Boheman, 1837)	GU987769 [01]<br>M-0058-nav<br>658nt	ZFMK-TIS-cM0058<br>ZFMK-DNA-0100400962	Germany, Rhineland-Palatinate (RLP), Moselle valley, 10 km S of Cochem, Bullay, N50°03'27" E07°08'51", 222m, 16-Sep-2005, *Quercus, Carpinus*, leg. Stüben,P., det. Stüben,P.
Kyklioacalles navieresi<br>(Boheman, 1837)	GU987855 [01]<br>M-0065-nav<br>658nt	ZFMK-TIS-cM0065<br>ZFMK-DNA-0100401054	Germany, Rhineland-Palatinate (RLP), Bausenberg near Niederzissen, N50°27'52" E07°13'29", 291m, 17-Sep-2005, *Quercus, Carpinus, Fagus*, leg. Stüben,P., det. Stüben,P.
Kyklioacalles navieresi<br>(Boheman, 1837)	GU988156 [01]<br>D-928-nav<br>658nt	ZFMK-TIS-cD928<br>ZFMK-DNA-0100405255	Germany, Rhineland-Palatinate (RLP), W of Karlsruhe, Bienwald, N49°00'49" E08°05'18", 130m, 12-Jun-2009, *Quercus*, leg. Stüben,P., det. Stüben,P.
Kyklioacalles navieresi<br>(Boheman, 1837)	GU981499 [01]<br>I-636-nav<br>658nt	ZFMK-TIS-cI636<br>ZFMK-DNA-0100404910	Italy, Campania, Monti Picentini, 9 km N of Acerno, Piano Laceno, N40°48'58" E15°07'35", 1210m, 03-Jul-2008, *Fagus*, leg. Stüben,P., det. Stüben,P.
Kyklioacalles navieresi<br>(Boheman, 1837)	GU987968 [01]<br>I-623-nav<br>656nt	ZFMK-TIS-cI623<br>ZFMK-DNA-0100404920	Italy, Campania, Cilento, 6 km SE of Vallo d. Lucania, M. Sacro o Gelbison, N40°12'41" E15°19'42", 1544m, 30-Jun-2008, *Fagus*, leg. Stüben,P., det. Stüben,P.
Kyklioacalles navieresi<br>(Boheman, 1837)	GU987990 [01]<br>I-657-nav<br>658nt	ZFMK-TIS-cI657<br>ZFMK-DNA-0100404883	Italy, Abruzzo, P.N. Majella, 11 km N of Roccaraso, Bosco di S. Antonio, N41°56'27" E14°01'41", 1321m, 14-Jul-2008, *Pyrus/Acer*, old forest, leg. Stüben,P., det. Stüben,P.
Kyklioacalles navieresi<br>(Boheman, 1837)	GU987991 [01]<br>I-658-nav<br>658nt	ZFMK-TIS-cI658<br>ZFMK-DNA-0100404239	Italy, Abruzzo, P.N. Majella, 11 km N of Roccaraso, Bosco di S. Antonio, N41°56'27" E14°01'41", 1321m, 14-Jul-2008, *Pyrus/Acer*, old forest, leg. Stüben,P., det. Stüben,P.
Kyklioacalles navieresi<br>(Boheman, 1837)	MG229701 [new]<br>1402-PST<br>658nt	ZFMK-TIS-4698<br>ZFMK-DNA-0155628536	Italy, Lazio, near Arpino, Mura Ciclopiche, N41°38'53" E13°38'15", 739m, 30-Aug-2013, mixed forest, sieving, leg. Stüben,P., det. Stüben,P.
Kyklioacalles navieresi<br>(Boheman, 1837)	GU988029 [01]<br>S-663-nav<br>658nt	ZFMK-TIS-cS663<br>ZFMK-DNA-0100404815	Sweden, Simrishamn, Stenshuvud N.P., N55°39'15" E14°15'50", 80m, 12-Aug-2008, leg. Astrin,J., det. Stüben,P.
Kyklioacalles navieresi<br>(Boheman, 1837)	GU988030 [01]<br>S-664-nav<br>658nt	ZFMK-TIS-cS664<br>ZFMK-DNA-0100404816	Sweden, Karlskrona, Skärva N.R., N56°12'28" E15°34'23", 20m, 23-Aug-2008, *Fagus, Quercus, Acer*, leg. Astrin,J., det. Stüben,P.

**Name**<br>**Authority**<br>**Additional Information**	**GenBank Acc No (Ref.)**<br>**Specimen ID**<br>**Sequence Length**	**ZFMK Tissue ID**<br>**ZFMK DNA Sample ID**<br>**(SDEI DNA Sample ID)**	**Locality, GPS, Collection Date, Plant, Collector, Identifier**
Kyklioacalles navieresi<br>(Boheman, 1837)	GU988031 [01]<br>S-665-nav<br>658nt	ZFMK-TIS-cS665<br>ZFMK-DNA-0100404817	Sweden, Ronneby, Almö, next to Golfclub, N56°09'18" E15°26'48", 1m, 24-Aug-2008, *Fagus, Quercus*, leg. Astrin,J., det. Stüben,P.
Kyklioacalles oblongus<br>Stüben, 2018<br>**Paratype (DNAtype)**<br>formerly: *K. erinaceus*	KJ867612 [34]<br>1308-PST_4124<br>658nt	ZFMK-TIS-4124<br>ZFMK-DNA-0100425383	Spain, Jaen, SE of Mancha Real, Sierra Almaden (El Almaden, **type locality**), N37°44'06" W03°31'51", 1867m, 15-Mai-2013, *Astragalus* sp., *Bupleurum spinosum, Euphorbia nicaeensis*, sieving, leg. Stüben,P. & Schütte,A., det. Stüben,P.
Kyklioacalles oblongus<br>Stüben, 2018<br>**Paratype (DNAtype)**<br>formerly: *K. erinaceus*	KJ867621 [34]<br>1340-PST_4156<br>658nt	ZFMK-TIS-4156<br>ZFMK-DNA-0100426136	Spain, Jaen, S of La Guardia, Sierra de la Pandera, N37°40'20" W03°41'48", 1223m, 19-Mai-2013, *Erinacea anthyllis, Cytisus* sp., sieving, leg. Stüben,P. & Schütte,A., det. Stüben,P.
Kyklioacalles olcesei<br>(Tournier, 1873)	GU981504 [01]<br>E-784-olc<br>658nt	ZFMK-TIS-cE784<br>ZFMK-DNA-0100405019	Spain, Cadiz, San Roque, Pinar del Rey, at the end of recreative zone, N36°12' W05°22', 04-Dez-2008, broom, leg. Torres,J. L., det. Torres,J. L.
Kyklioacalles olcesei<br>(Tournier, 1873)	GU988049 [01]<br>E-785-olc<br>658nt	ZFMK-TIS-cE785<br>ZFMK-DNA-0100405047	Spain, Cadiz, La Linea, Sierra Carbonera, Puerto Higuerón, Cortijo Los Portichuelos, N36°12' W05°19', 18-Nov-2008, *Chamaerops humilis*, leg. Torres,J. L., det. Torres,J. L.
Kyklioacalles olcesei<br>(Tournier, 1873)	GU988071 [01]<br>E-787-olc<br>658nt	ZFMK-TIS-cE787<br>ZFMK-DNA-0100405017	Spain, Cadiz, La Linea, Sierra Carbonera, close to hermitage, N36°12' W05°19', 03-Okt-2008, *Chamaerops humilis*, leg. Torres,J. L., det. Torres,J. L.
Kyklioacalles oukaimedensis<br>Stüben, 2010	GU213774 [04]<br>E-883-ouk<br>658nt	ZFMK-TIS-cE883<br>ZFMK-DNA-0100405509	Morocco, High Atlas, E of Asni, Oukaimeden, N31°12'14" W07°52'21", 2646m, 01-Mai-2009, *Erinacea* , under stones, leg. Stüben,P., det. Stüben,P.
Kyklioacalles oukaimedensis<br>Stüben, 2010	GU981512 [01]<br>E-886-ouk<br>658nt	ZFMK-TIS-cE886<br>ZFMK-DNA-0100405512	Morocco, High Atlas, SE of Asni, Tacheddirt (near Imlil), N31°09'07" W07°50'18", 2420m, 02-Mai-2009, broom, *Daphne*, leg. Stüben,P., det. Stüben,P.
Kyklioacalles plantapilosus<br>Stüben & Astrin, 2010<br>**Paratype (DNAtype)**	GU981475 [01]<br>E-0190-pla<br>658nt	ZFMK-TIS-cE0190<br>ZFMK-DNA-0100400360	Spain, Almeria, 11 km NW of Laujar de Andarax, Sierra Nevada, Bayarcal, N37°02'27" W03°00'12", 1291m, 07-Apr-2007, *Euphorbia*, leg. Astrin,J. & Stüben,P., det. Stüben,P.
Kyklioacalles provincialis<br>(A. Hoffmann, 1960)	GU981488 [01]<br>F-443-pro<br>658nt	ZFMK-TIS-cF443<br>ZFMK-DNA-0100400652	France, Alpes-Maritimes, 3 km NW of Sospel, Col de Braus, N43°52'22" E07°23'57", 1048m, 27-Dez-2007, *Quercus ilex,* broom, leg. Stüben,P., det. Stüben,P.
Kyklioacalles provincialis<br>(A. Hoffmann, 1960)	GU981490 [01]<br>F-451-pro<br>658nt	ZFMK-TIS-cF451<br>ZFMK-DNA-0100400277	France, Alpes-de-Hautes-Provence, 11 km NE of Castellane near Soleilhas, Col de St. Barnabe, N43°51'55" E06°37'52", 1368m, 30-Dez-2007, *Helleborus,* broom, under stones, leg. Stüben,P., det. Stüben,P.
Kyklioacalles provincialis<br>(A. Hoffmann, 1960)	GU987894 [01]<br>F-434-pro<br>658nt	ZFMK-TIS-cF434<br>ZFMK-DNA-0100400660	France, Alpes-Maritimes, 4 km W of Sospel, (near Col de Braus), N43°52'32" E07°23'33", 1055m, 24-Dez-2007, *Euphorbia spinosa,* broom, leg. Stüben,P., det. Stüben,P.
Kyklioacalles provincialis<br>(A. Hoffmann, 1960)	GU987899 [01]<br>F-445-pro<br>658nt	ZFMK-TIS-cF445<br>ZFMK-DNA-0100400650	France, Alpes-Maritimes, 6 km NW of Sospel, Col de l'Orme, N43°54'03" E07°21'46", 1008m, 27-Dez-2007, broom, leg. Stüben,P., det. Stüben,P.
Kyklioacalles punctaticollis meteoricus<br>(P. Meyer, 1909)	GU981489 [01]<br>F-449-met<br>658nt	ZFMK-TIS-cF449<br>ZFMK-DNA-0100400234	France, Alpes-de-Hautes-Provence, 11 km NE of Castellane near Soleilhas, Col de St. Barnabe, N43°51'55" E06°37'52", 1368m, 30-Dez-2007, *Helleborus,* broom, under stones, leg. Stüben,P., det. Stüben,P.

**Name**<br>**Authority**<br>**Additional Information**	**GenBank Acc No (Ref.)**<br>**Specimen ID**<br>**Sequence Length**	**ZFMK Tissue ID**<br>**ZFMK DNA Sample ID**<br>**(SDEI DNA Sample ID)**	**Locality, GPS, Collection Date, Plant, Collector, Identifier**
Kyklioacalles punctaticollis meteoricus<br>(P. Meyer, 1909)	GU987893 [01]<br>F-433-met<br>658nt	ZFMK-TIS-cF433<br>ZFMK-DNA-0100400659	France, Alpes-Maritimes, 6 km NE of Sospel, Col de Brouis, N43°55'28" E07°28'37", 882m, 23-Dez-2007, broom, *Helleborus*, leg. Stüben,P., det. Stüben,P.
Kyklioacalles punctaticollis meteoricus<br>(P. Meyer, 1909)	EU286489 [01]<br>E-0165-met<br>658nt	ZFMK-TIS-cE0165<br>ZFMK-DNA-0100400159	Spain, Alicante, 6.5 km NW of Callosa d'en Sarrià, Sierra de Aixorta, N38°41'57" W00°10'32", 1074m, 26-Dez-2005, *Erinacea anthyllis*, limestone, leg. Stüben,P., det. Stüben,P.
Kyklioacalles punctaticollis meteoricus<br>(P. Meyer, 1909)	GU981476 [01]<br>E-0177-met<br>658nt	ZFMK-TIS-cE0177<br>ZFMK-DNA-0100400218	Spain, Alicante, 6,5 NW of Callosa d'En Sarria Sarrià, Sierra de Aixorta, N38°41'59" W00°10'34", 1091m, 04-Apr-2007, *Erinacea anthyllis*, limestone, leg. Astrin,J. & Stüben,P., det. Stüben,P.
Kyklioacalles punctaticollis meteoricus<br>(P. Meyer, 1909)	GU981497 [01]<br>E-605-met<br>658nt	ZFMK-TIS-cE605<br>ZFMK-DNA-0100404927	Spain, Castellon, Morella, pass near Cinctorres, N40°33'39" W00°14'54", 1238m, 20-Jun-2008, *Erinacea anthyllis*, leg. Astrin,J., det. Stüben,P.
Kyklioacalles punctaticollis meteoricus<br>(P. Meyer, 1909)	KJ867614 [28]<br>1320-PST<br>658nt	ZFMK-TIS-4136<br>ZFMK-DNA-0100426156	Spain, Granada, E of Baul, Sierra de Baza, Santa Barbara, N37°23'10" W02°50'55", 1911m, 17-Mai-2013, *Bupleurum, (Astragalus* sp.*)*, sieving, leg. Stüben,P. & Schütte,A., det. Stüben,P.
Kyklioacalles punctaticollis meteoricus<br>(P. Meyer, 1909)	KJ867620 [new]<br>1338-PST<br>658nt	ZFMK-TIS-4154<br>ZFMK-DNA-0100426138	Spain, Jaen, S of La Guardia, Sierra de la Pandera, N37°40'20" W03°41'48", 1223m, 19-Mai-2013, *Erinacea anthyllis, Cytisus* sp., sieving, leg. Stüben,P. & Schütte,A., det. Stüben,P.
Kyklioacalles punctaticollis punctaticollis<br>(Lucas, 1846)	EU286487 [01]<br>E-0162-pun<br>658nt	ZFMK-TIS-cE0162<br>ZFMK-DNA-0100400043	Spain, Andalucia, 26 km E of Jaen, Sierra Magina, N37°44'06" W03°30'09", 1663m, 06-Okt-2005, *Erinacea anthyllis*, limestone, leg. Stüben,P., det. Stüben,P.
Kyklioacalles punctaticollis punctaticollis<br>(Lucas, 1846)	GU981473 [01]<br>E-0163-pun<br>658nt	ZFMK-TIS-cE0163<br>ZFMK-DNA-0100400151	Spain, Murcia, 13 km N of Totana, Sierra de Espuna, N37°52'07" W01°33'46", 1361m, 29-Dez-2005, *Erinacea anthyllis*, limestone, leg. Stüben,P., det. Stüben,P.
Kyklioacalles punctaticollis punctaticollis<br>(Lucas, 1846)	GU981477 [01]<br>E-0183-met<br>658nt	ZFMK-TIS-cE0183<br>ZFMK-DNA-0100400331	Spain, Alicante, 7 km SW of Alcoi, Sierra de Menechaor, Santuario de la Font Roja, N38°39'34" W00°32'29", 296m, 05-Apr-2007, *Erinacea anthyllis*, limestone, leg. Astrin,J. & Stüben,P., det. Stüben,P.
Kyklioacalles punctaticollis punctaticollis<br>(Lucas, 1846)	GU981478 [01]<br>E-0195-pun<br>658nt	ZFMK-TIS-cE0195<br>ZFMK-DNA-0100400056	Spain, Malaga, 9 km SE of Ubrique, Sierra de Libar, N36°36'52" W05°23'16, 663m, 10-Apr-2007, *Quercus ilex, Ceratonia*, cliff, leg. Astrin,J. & Stüben,P., det. Stüben,P.
Kyklioacalles punctaticollis punctaticollis<br>(Lucas, 1846)	GU981480 [01]<br>E-0211-pun<br>658nt	ZFMK-TIS-cE0211<br>ZFMK-DNA-0100400314	Spain, Malaga, 16 km N of Estepona, Sierra Bermeja, Jubrique, N36°33'49" W05°12'27", 557m, 13-Apr-2007, *Quercus suber*, leg. Astrin,J. & Stüben,P., det. Stüben,P.
Kyklioacalles punctaticollis punctaticollis<br>(Lucas, 1846)	GU981481 [01]<br>E-0216-pun<br>658nt	ZFMK-TIS-cE0216<br>ZFMK-DNA-0100400201	Spain, Cadiz, 14 km N of Ubrique, Sierra Zafalgar, Puerto de las Palomas, N36°47'16" W05°22'36", 1286m, 14-Apr-2007, broom, leg. Astrin,J. & Stüben,P., det. Stüben,P.
Kyklioacalles punctaticollis punctaticollis<br>(Lucas, 1846)	GU981503 [01]<br>E-775-pun<br>658nt	ZFMK-TIS-cE775<br>ZFMK-DNA-0100405030	Spain, Malaga, N of Marbella, Puerto de Ojen, N36°34'33" W04°51'25", 545m, 16-Jan-2009, *Ulex*, leg. Stüben,P., det. Stüben,P.
Kyklioacalles punctaticollis punctaticollis<br>(Lucas, 1846)	GU981505 [01]<br>E-790-pun<br>658nt	ZFMK-TIS-cE790<br>ZFMK-DNA-0100405016	Spain, Cadiz, La Linea, Sierra Carbonera, Puerto Higuerón, Cortijo Los Puertos, N36°12' W05°19', 18-Nov-2008, *Cynara*, leg. Torres,J. L., det. Torres,J. L.
Kyklioacalles punctaticollis punctaticollis<br>(Lucas, 1846)	GU987830 [01]<br>E-0201-pun<br>658nt	ZFMK-TIS-cE0201<br>ZFMK-DNA-0100400216	Spain, Malaga, 13 km SE of Ubrique, Sierra de Libar, Cortes de la Fa., N36°38'29" W05°18'26", 439m, 11-Apr-2007, *Quercus ilex,* broom, leg. Astrin,J. & Stüben,P., det. Stüben,P.

**Name**<br>**Authority**<br>**Additional Information**	**GenBank Acc No (Ref.)**<br>**Specimen ID**<br>**Sequence Length**	**ZFMK Tissue ID**<br>**ZFMK DNA Sample ID**<br>**(SDEI DNA Sample ID)**	**Locality, GPS, Collection Date, Plant, Collector, Identifier**
Kyklioacalles punctaticollis punctaticollis<br>(Lucas, 1846)	GU987835 [01]<br>E-0215-pun<br>658nt	ZFMK-TIS-cE0215<br>ZFMK-DNA-0100400202	Spain, Malaga, 7 km NW of Estepona, Sierra Bermeja, Los Reales, N36°28'59" W05°12'28", 1418m, 13-Apr-2007, broom, leg. Astrin,J. & Stüben,P., det. Stüben,P.
Kyklioacalles punctaticollis punctaticollis<br>(Lucas, 1846)	GU988056 [01]<br>E-767-pun<br>658nt	ZFMK-TIS-cE767<br>ZFMK-DNA-0100404718	Spain, Malaga, N of Estepona, Sierra Bermeja, Los Reales, N36°29'32" W05°11'47", 1071m, 12-Jan-2009, broom, leg. Stüben,P., det. Stüben,P.
Kyklioacalles punctaticollis punctaticollis<br>(Lucas, 1846)	KJ867618 [28]<br>1328-PST<br>658nt	ZFMK-TIS-4144<br>ZFMK-DNA-0100426149	Spain, Jaen, Sierra Magina, SE of Torres, N37°44'46" W03°29'53", 1527m, 19-Mai-2013, *Erinacea anthyllis, Quercus ilex, Astragalus* sp., *Bupleurum*, sieving, leg. Stüben,P. & Schütte,A., det. Stüben,P.
Kyklioacalles punctaticollis punctaticollis<br>(Lucas, 1846)	KU170190 [28]<br>ES1063<br>658nt	ZFMK-TIS-cES1063<br>ZFMK-DNA-0112704605	Spain, Malaga, Algatocin near Opayar, N36°34'39" W05°18'13", 576m, 17-Aug-2010, leg. Stüben,P., det. Stüben,P.
Kyklioacalles pyrenaeus<br>(Boheman, 1844)	GU981485 [01]<br>A-0296-pyr<br>658nt	ZFMK-TIS-cA0296<br>ZFMK-DNA-0100401032	Austria, Lower Austria, Merkersdorf, National Park Thayatal, N48°32' E16°17', 18-Apr-2006, *Carpinetum*, leg. Stejskal,R., det. Stejskal,R.
Kyklioacalles pyrenaeus<br>(Boheman, 1844)	GU981506 [01]<br>Cz-798-pyr<br>658nt	ZFMK-TIS-cCz798<br>ZFMK-DNA-0100405001	Czech Republic, Bohemia W (KT), Balkovy, Doubrava Hill (6545), N49°26' E13°13', 17-Aug-2008, leg. Kresl,P., det. Kresl,P.
Kyklioacalles pyrenaeus<br>(Boheman, 1844)	GU988079 [01]<br>Cz-801-pyr<br>658nt	ZFMK-TIS-cCz801<br>ZFMK-DNA-0100405004	Czech Republic, Bohemia W (DO), Kdyne, Novy Herstejn rez. (6644), N49°23' E13°02', 24-Mai-2008, leg. Kresl,P., det. Kresl,P.
Kyklioacalles pyrenaeus<br>(Boheman, 1844)	MG229687 [new]<br>1213-JKR<br>658nt	ZFMK-TIS-3549<br>ZFMK-DNA-0155630462	Czech Republic, Bohemia, sumava, Hojsova Straz, PR Lakmal, N49°13'22" E13°11'02", 712m, 26-Aug-2012, *Picea excelsa*, beating, leg. Krátky,J., det. Krátky,J.
Kyklioacalles pyrenaeus<br>(Boheman, 1844)	MG229782 [new]<br>2414-JKR<br>658nt	ZFMK-TIS-25991<br>ZFMK-DNA-0171600636	Czech Republic, Bohemia, Kralicky Sneznik, Tvarozne diry, N50°10'60" E16°50'18", 892m, 25-Aug-2014, *Picea excelsa*, collecting by hand, leg. Krátky,J., det. Krátky,J.
Kyklioacalles pyrenaeus<br>(Boheman, 1844)	MG229783 [new]<br>2415-JKR<br>658nt	ZFMK-TIS-25992<br>ZFMK-DNA-0171600637	Czech Republic, Bohemia, Kralicky Sneznik, Tvarozne diry, N50°10'60" E16°50'18", 892m, 25-Aug-2014, *Picea excelsa*, collecting by hand, leg. Krátky,J., det. Krátky,J.
Kyklioacalles pyrenaeus<br>(Boheman 1844)<br>formerly: *K. pyrenaeus germanicus* - Syn!	KJ867590 [new]<br>1211-JKR<br>658nt	ZFMK-TIS-3547<br>ZFMK-DNA-0100426080	Czech Republic, Bohemia, sumava, Hojsova Straz, PR Lakmal, N49°13'22" E13°11'02", 712m, 26-Aug-2012, *Fagus sylvatica*, beating, leg. Krátky,J., det. Krátky,J.
Kyklioacalles pyrenaeus<br>(Boheman 1844)<br>formerly: *K. pyrenaeus germanicus* - Syn!	KJ867593 [new]<br>1226-JKR<br>658nt	ZFMK-TIS-3562<br>ZFMK-DNA-0100426047	Czech Republic, Bohemia, Velke Petrovice, N50°30'32" E16°12'42", 401m, 21-Okt-2012, *Salix, Alnus*, sieving, leg. Krátky,J., det. Krátky,J.
Kyklioacalles pyrenaeus<br>(Boheman 1844)<br>formerly: *K. pyrenaeus germanicus* - Syn!	MG229787 [new]<br>2420-JKR<br>658nt	ZFMK-TIS-25997<br>ZFMK-DNA-0171600642	Czech Republic, Bohemia, Horni Morava, N50°10'15" E16°49'22", 780m, 25-Aug-2014, *Fagus sylvatica*, collecting by hand, leg. Krátky,J., det. Krátky,J.

**Name**<br>**Authority**<br>**Additional Information**	**GenBank Acc No (Ref.)**<br>**Specimen ID**<br>**Sequence Length**	**ZFMK Tissue ID**<br>**ZFMK DNA Sample ID**<br>**(SDEI DNA Sample ID)**	**Locality, GPS, Collection Date, Plant, Collector, Identifier**
Kyklioacalles pyrenaeus<br>(Boheman 1844)<br>formerly: *K. pyrenaeus germanicus* - Syn!	MG229788 [new]<br>2421-JKR<br>658nt	ZFMK-TIS-25998<br>ZFMK-DNA-0171600643	Czech Republic, Bohemia, Horni Morava, N50°10'15" E16°49'22", 780m, 25-Aug-2014, *Fagus sylvatica*, collecting by hand, leg. Krátky,J., det. Krátky,J.
Kyklioacalles pyrenaeus<br>(Boheman 1844)<br>formerly: *K. pyrenaeus germanicus* - Syn!	MG229789 [new]<br>2422-JKR<br>658nt	ZFMK-TIS-25999<br>ZFMK-DNA-0171600644	Czech Republic, Bohemia, Horni Morava, N50°10'15" E16°49'22", 780m, 25-Aug-2014, *Fagus sylvatica*, collecting by hand, leg. Krátky,J., det. Krátky,J.
Kyklioacalles pyrenaeus<br>(Boheman, 1844)	GU981470 [01]<br>I-0028-pyr<br>658nt	ZFMK-TIS-cI0028<br>ZFMK-DNA-0100400963	France, Isere, 2 km SE of Lans en Vercors, Montagne de Lans, N45°06'45" E05°36'21", 1352m, 12-Aug-2005, *Abies, Fagus, Fraxinus*, limestone: mixed forest, leg. Stüben,P., det. Stüben,P.
Kyklioacalles pyrenaeus<br>(Boheman, 1844)	GU987762 [01]<br>I-0035-pyr<br>658nt	ZFMK-TIS-cI0035<br>ZFMK-DNA-0100400959	France, Isere, 14 km N of Grenoble, Massif de la Chartreus, NW of Col de Porte, N45°18'40" E05°45'17", 1649m, 13-Aug-2005, *Abies, Fagus, Fraxinus*, limestone (tree line), leg. Stüben,P., det. Stüben,P.
Kyklioacalles reginae<br>Stüben, 2003	GU981495 [01]<br>E-600-reg<br>654nt	ZFMK-TIS-cE600<br>ZFMK-DNA-0100404943	Spain, Teruel, S. Javalambre, Fuente la Risca near Arcos de las Salinas, N39°59'56" W01°01'21", 1121m, 17-Jun-2008, *Amelanchier ovalis, Acer monspessulanum, Erinacea anthyllis, Ulex*, leg. Astrin,J., det. Stüben,P.
Kyklioacalles reginae<br>Stüben, 2003	GU981496 [01]<br>E-604-reg<br>658nt	ZFMK-TIS-cE604<br>ZFMK-DNA-0100404939	Spain, Teruel, S. Javalambre, grove on wet ground near Camarena de la Sierra, N40°08'30" W01°02'24", 1331m, 18-Jun-2008, *Juglans regia, Crataegus, Populus, Rubus*, leg. Astrin,J., det. Stüben,P.
Kyklioacalles reinosae<br>(H. Brisout de Barneville, 1867)	GU981510 [01]<br>E-854-rei<br>658nt	ZFMK-TIS-cE854<br>ZFMK-DNA-0100405529	Portugal, Sa. Estrela, SW of Manteigas, N40°18'45" W07°35'03", 1558m, 25-Apr-2009, *Cytisus*, leg. Astrin,J., det. Stüben,P.
Kyklioacalles reinosae<br>(H. Brisout de Barneville, 1867)	GU987908 [01]<br>P-539-rei<br>658nt	ZFMK-TIS-cP539<br>ZFMK-DNA-0100400627	Portugal, Manteigas, N40°23'07" W07°31'37", 1236m, 26-Jan-2008, *Fagus, Castanea, Quercus*, leg. Astrin,J., det. Astrin,J.
Kyklioacalles reinosae<br>(H. Brisout de Barneville, 1867)	GU988119 [01]<br>E-868-rei<br>658nt	ZFMK-TIS-cE868<br>ZFMK-DNA-0100405523	Portugal, Sa. Marao, W of Vila Real, Alto do Espinho, N41°16'15" W07°54'17", 1145m, 30-Apr-2009, *Amelanchier, Quercus pyrenaica, Cytisus*, leg. Astrin,J., det. Stüben,P.
Kyklioacalles reinosae<br>(H. Brisout de Barneville, 1867)	KJ867585 [new]<br>830-PST<br>658nt	ZFMK-TIS-3743<br>ZFMK-DNA-0100414261	Portugal, Minho, PN of Peneda-Geres near Soajo, N41°52'14" W08°16'30", 200m, 09-Mai-2012, *Sarothamnus*, beating, leg. Stüben,P., det. Stüben,P.
Kyklioacalles reinosae<br>(H. Brisout de Barneville, 1867)	GU981509 [01]<br>E-844-rei<br>658nt	ZFMK-TIS-cE844<br>ZFMK-DNA-0100405546	Spain, Avila, SW of Ávila, SE of Piedrahita, Pto. de la Pena Negra, N40°25'19" W05°18'01", 1910m, 23-Apr-2009, *Cytisus* , leg. Astrin,J., det. Stüben,P.
Kyklioacalles reinosae<br>(H. Brisout de Barneville, 1867)	GU987887 [01]<br>E-475-rei<br>631nt	ZFMK-TIS-cE475<br>ZFMK-DNA-0100400984	Spain, Salamanca, 7 km SE of Bejar, Sierra de Candelario, La Covatilla, N40°21'26" W05°41'06", 1951m, 07-Apr-2004, *Cytisus purgans* , leg. Stüben,P., det. Stüben,P.
Kyklioacalles reinosae<br>(H. Brisout de Barneville, 1867)	GU988099 [01]<br>E-839-rei<br>658nt	ZFMK-TIS-cE839<br>ZFMK-DNA-0100405562	Spain, Avila, SW of Ávila near Mengamunoz, Pto. de Menga, N40°28'40" W05°00'40", 1564m, 23-Apr-2009, *Cytisus*, leg. Astrin,J., det. Stüben,P.
Kyklioacalles reinosae<br>(H. Brisout de Barneville, 1867)	GU988101 [01]<br>E-841-rei<br>658nt	ZFMK-TIS-cE841<br>ZFMK-DNA-0100404521	Spain, Avila, SW of Ávila, N of San Martin de la Vega, Pto. de Chia, N40°27'16" W05°10'16", 1718m, 23-Apr-2009, *Cytisus*, leg. Astrin,J., det. Stüben,P.

**Name**<br>**Authority**<br>**Additional Information**	**GenBank Acc No (Ref.)**<br>**Specimen ID**<br>**Sequence Length**	**ZFMK Tissue ID**<br>**ZFMK DNA Sample ID**<br>**(SDEI DNA Sample ID)**	**Locality, GPS, Collection Date, Plant, Collector, Identifier**
Kyklioacalles reinosae<br>(H. Brisout de Barneville, 1867)	GU988107 [01]<br>E-849-rei<br>658nt	ZFMK-TIS-cE849<br>ZFMK-DNA-0100404559	Spain, Salamanca, NW of Bejar, NW of Monforte de la Sierra, Pena de Francia near cloister, N40°31'01" W06°10'15", 1636m, 24-Apr-2009, *Cytisus*, leg. Astrin,J., det. Stüben,P.
Kyklioacalles reinosae<br>(H. Brisout de Barneville, 1867)	GU988114 [01]<br>E-859-rei<br>625nt	ZFMK-TIS-cE859<br>ZFMK-DNA-0100405534	Spain, Caceres, NE of Plasencia, S of Hervas, Pto. de Honduras, N40°13'17" W05°52'19", 1436m, 26-Apr-2009, *Cytisus* , leg. Astrin,J., det. Stüben,P.
Kyklioacalles reinosae<br>(H. Brisout de Barneville, 1867)	GU988124 [01]<br>E-873-rei<br>658nt	ZFMK-TIS-cE873<br>ZFMK-DNA-0100404543	Spain, Leon, NE of León, NE of Riano, Pto. de Pandetrave, N43°06'24" W04°52'35", 1560m, 04-Mai-2009, *Cytisus*, leg. Astrin,J., det. Stüben,P.
Kyklioacalles roboris<br>(Curtis, 1834)	GU981498 [01]<br>AND-618-rob<br>658nt	ZFMK-TIS-cAND618<br>ZFMK-DNA-0100404925	Andorra, Arinsal, N42°34'15" E01°29'06", 1456m, 25-Jun-2008, *Corylus avellana, Ruscus, Betula*, leg. Astrin,J., det. Stüben,P.
Kyklioacalles roboris<br>(Curtis, 1834)	GU981507 [01]<br>Cz-799-rob<br>658nt	ZFMK-TIS-cCz799<br>ZFMK-DNA-0100405002	Czech Republic, Bohemia W (KT), Balkovy, Doubrava Hill (6545), N49°26' E13°13', 17-Aug-2008, leg. Kresl,P., det. Kresl,P.
Kyklioacalles roboris<br>(Curtis, 1834)	EU286494 [01]<br>I-0037-rob<br>658nt	ZFMK-TIS-cI0037<br>ZFMK-DNA-0100400012	France, Isere, 14 km N of Grenoble, Massif de la Chartreus, NW of Col de Porte, N45°18'40" E05°45'17", 1649m, 13-Aug-2005, *Abies, Fagus, Fraxinus*, limestone (tree line), leg. Stüben,P., det. Stüben,P.
Kyklioacalles roboris<br>(Curtis, 1834)	GU987760 [01]<br>I-0032-rob<br>624nt	ZFMK-TIS-cI0032<br>ZFMK-DNA-0100400936	France, Isere, 2 km SE of Lans en Vercors, Montagne de Lans, N45°06'45" E05°36'21", 1352m, 12-Aug-2005, *Abies, Fagus, Fraxinus*, limestone: mixed forest, leg. Stüben,P., det. Stüben,P.
Kyklioacalles roboris<br>(Curtis, 1834)	GU987761 [01]<br>I-0025-rob<br>658nt	ZFMK-TIS-cI0025<br>ZFMK-DNA-0100400762	France, Isere, 9 km N of Grenoble, Mont St. Martin, N45°16'15" E05°40'13", 719m, 12-Aug-2005, *Quercus*, limestone: wood pile, leg. Stüben,P., det. Stüben,P.
Kyklioacalles roboris<br>(Curtis, 1834)	GU981469 [01]<br>M-0062-rob<br>658nt	ZFMK-TIS-cM0062<br>ZFMK-DNA-0100400885	Germany, Rhineland-Palatinate (RLP), Moselle valley, 1 km N of Treis-Karden, N50°11'08" E07°18'20", 250m, 17-Sep-2005, *Quercus, Carpinus*, leg. Stüben,P., det. Stüben,P.
Kyklioacalles roboris<br>(Curtis, 1834)	GU987773 [01]<br>M-0054-rob<br>658nt	ZFMK-TIS-cM0054<br>ZFMK-DNA-0100400911	Germany, Rhineland-Palatinate (RLP), Moselle valley, 6 km SE of Cochem, above Ediger, N50°05'28" E07°11'01", 270m, 16-Sep-2005, *Quercus, Carpinus*, leg. Stüben,P., det. Stüben,P.
Kyklioacalles roboris<br>(Curtis, 1834)	GU988035 [01]<br>D-670-rob<br>658nt	ZFMK-TIS-cD670<br>ZFMK-DNA-0100405092	Germany, North Rhine-Westphalia (NRW), N of Mönchengladbach, Helenabrunn, N51°13'34" E06°24'00", 60m, 15-Nov-2007, *Fagus*, leg. Stüben,P., det. Stüben,P.
Kyklioacalles roboris<br>(Curtis, 1834)	KC783819 [new]<br>462-PST<br>626nt	ZFMK-TIS-20248<br>ZFMK-DNA-0100449384	Italy, Como, Vallassina, dintorni Barni, N45°54' E09°16', 800m, 23-Feb-2011, leg. Diotti,L., det. Stüben,P.
Kyklioacalles saccoi<br>(Colonnelli, 1973)	JN121403 [03]<br>IT1051<br>658nt	ZFMK-TIS-cIT1051<br>ZFMK-DNA-0112704618	Italy, Calabria, La Sila Mts., Spezzano de la Sila, 39°19'178N 16°23'104E, 26-Jun-2009, leg. Krátky, det. Krátky,J.
Kyklioacalles snassensis<br>Stüben, 2002	KC783812 [new]<br>114-PST<br>658nt	ZFMK-TIS-2D100440304<br>ZFMK-DNA-0100438391	Morocco, S of Berkane, Beni Snassen Mts., Vallee Zegzel, N34°50'27" W02°21'34", 423m, 11-Mai-2011, *Smilax/Pistacea* , sieving, leg. Stüben,P., det. Stüben,P.
Kyklioacalles solarii<br>(Fiori, 1903)	GU981486 [01]<br>I-473-sol<br>569nt	ZFMK-TIS-cI473<br>ZFMK-DNA-0100400986	Italy, Toscana, Passo del Spino, 3 km E of Chiusi della Verna (AR), N43°42'08" E11°57'13", 1000m, 18-Okt-2001, *Fraxinus*, leg. Stüben,P., det. Stüben,P.

**Name**<br>**Authority**<br>**Additional Information**	**GenBank Acc No (Ref.)**<br>**Specimen ID**<br>**Sequence Length**	**ZFMK Tissue ID**<br>**ZFMK DNA Sample ID**<br>**(SDEI DNA Sample ID)**	**Locality, GPS, Collection Date, Plant, Collector, Identifier**
Kyklioacalles suturatus<br>(Dieckmann, 1983)	EU286483 [01]<br>CZ-0156-sut<br>620nt	ZFMK-TIS-cCZ0156<br>ZFMK-DNA-0100400168	Czech Republic, Moravia mer., Podyji National Park, Havraniky, Podyji NP, Sealsfielduv kamen, N48°48' E16°00', 270m, 05-Jun-2006, *Quercus, Corylus*, leg. Stejskal,R., det. Stejskal,R.
Kyklioacalles teter<br>(Boheman, 1844)	EU286484 [01]<br>I-0157-tet<br>658nt	ZFMK-TIS-cI0157<br>ZFMK-DNA-0100400167	Italy, Sicilia Isl. (PA), 15 km S of Bagheria, Monti di Calamigna, Masseria Suvarita, N37°57'08" E13°32'50", 570m, 11-Okt-2006, *Quercus ilex, Fraxinus*, leg. Stüben,P., det. Stüben,P.
Kyklioacalles teter<br>(Boheman, 1844)	GU981508 [01]<br>I-827-tet<br>658nt	ZFMK-TIS-cI827<br>ZFMK-DNA-0100405563	Italy, Sicilia Isl., Monte Soro, N37°55' E14°41', 1830m, 05-Feb-2008, *Fagus*, leg. Kapp, det. Kapp
Kyklioacalles teter<br>(Boheman, 1844)	MG229718 [new]<br>1651-JKR<br>658nt	ZFMK-TIS-4229<br>ZFMK-DNA-0155630489	Italy, Sicilia Isl. (PA), Palermo, Ficuzza env., N37°51'46" E13°23'00", 986m, 23-Sep-2013, *quercetum*, sieving, leg. Stejskal,R., det. Stejskal,R.
Kyklioacalles tidiquinensis<br>Stüben, 2002	GU213775 [04]<br>E-911-tid<br>658nt	ZFMK-TIS-cE911<br>ZFMK-DNA-0100405489	Morocco, Rif Mts., 10 km W of Ketama, N34°57'40" W04°40'51", 1600m, 10-Mai-2009, *Cedrus, Prunus*, leg. Stüben,P., det. Stüben,P.
Kyklioacalles yestensis<br>Stüben, 2003	GU981492 [01]<br>E-593-yes<br>658nt	ZFMK-TIS-cE593<br>ZFMK-DNA-0100404794	Spain, Albacete, Yeste, Los Prados, N38°20'47" W02°26'32", 1148m, 11-Jun-2008, *Juglans regia, Quercus ilex, Rubus*, leg. Astrin,J., det. Stüben,P.
Lachnaeus crinitus<br>Schoenherr, 1826	MK892111 [new]<br>2388-JKR<br>658nt	ZFMK-TIS-25965<br>ZFMK-DNA-0171600601	Czech Republic, Moravia, Dyjakovicky, Jecmenist?, N48°45'11" E16°08'12", 270m, 11-Sep-2014, *Inula* sp., beating, leg. R. Stejskal, det. Stejskal,R.
Lachnaeus crinitus<br>Schoenherr, 1826	MK891693 [new]<br>1911-FBA<br>658nt	ZFMK-TIS-23302<br>ZFMK-DNA-0169170372	Greece, East Macedonia and Thrace, Drama, Potami, N41°23'55" E24°05'57", 380m, 11-Aug-2014, leg. Bayer & Brunner, det. Bayer,F.
Laparocerus aenescens<br>(Wollaston, 1854)	MK347578 [40]<br>3011-KRA<br>658nt	(SDEI-DNA-3011-KRA)	Portugal, Madeira, Ribeiro Frio, Levada de Furado, N32°44'4.6" W16°53'12.7", 897m, 20-Mrz-2017, leg. Pelikan, det. Machado,A.
Laparocerus auarita<br>Machado, 2016	MK891481 [40]<br>1551-JKR	ZFMK-TIS-3599<br>ZFMK-DNA-0155635678	Spain, Canary Islands, La Palma, Llano de las Vacas, N28°43'29" W17°47'59", 1340m, 26-Jan-2013, *Cistus*, beating, leg. Krátky,J., det. Machado,A.
Laparocerus auarita<br>Machado, 2016	MK891488 [40]<br>1565-JKR<br>658nt	ZFMK-TIS-3613<br>ZFMK-DNA-0155635659	Spain, Canary Islands, La Palma, Montana Tagoja, N28°43'16" W17°47'07", 1052m, 30-Jan-2013, *Chamaecytisus*, beating, leg. Krátky,J., det. Machado,A.
Laparocerus auarita<br>Machado, 2016	MK891749 [40]<br>2002-JKR<br>658nt	ZFMK-TIS-23642<br>ZFMK-DNA-0169170499	Spain, Canary Islands, La Palma, Mendo, 3 km S of Jedey, N28°33'38" W17°51'45", 1000m, 12-Feb-2014, *Aeonium spathulatum, Rumex lunaria*, beating, leg. Krátky,J., det. Machado,A.
Laparocerus calvus<br>Machado, 2011	MK892354 [40]<br>2744-PST<br>625nt	ZFMK-TIS-23894<br>ZFMK-DNA-0171661252	Spain, Canary Islands, Fuerteventura, Cumbre de Jandia, Morro del Jorao (via Barranco de los Canarios), N28°07'03" W14°19'59", 625m, 17-Jan-2015, *Nauplius sericeus*, beating, leg. Stüben,P., det. Stüben,P.
Laparocerus combrecitensis<br>Roudier, 1957	MK891737 [40]<br>1980-JKR<br>658nt	ZFMK-TIS-23620<br>ZFMK-DNA-0169170521	Spain, Canary Islands, La Palma, Pared Vieja, N28°37'21" W17°49'21", 1163m, 07-Feb-2014, *Laurisilva*, beating, leg. Krátky,J., det. Krátky,J.
Laparocerus cristatus<br>Machado, 2009	MK891741 [40]<br>1993-JKR<br>658nt	ZFMK-TIS-23633<br>ZFMK-DNA-0169170511	Spain, Canary Islands, La Palma, Puerto Naos, Hoyo de las Norias, N28°35'51" W17°53'53", 381m, 09-Feb-2014, *Lavandula multifida*, beating, leg. Krátky,J., det. Krátky,J.

**Name**<br>**Authority**<br>**Additional Information**	**GenBank Acc No (Ref.)**<br>**Specimen ID**<br>**Sequence Length**	**ZFMK Tissue ID**<br>**ZFMK DNA Sample ID**<br>**(SDEI DNA Sample ID)**	**Locality, GPS, Collection Date, Plant, Collector, Identifier**
Laparocerus curvipes espanoli<br>Roudier, 1954	MK892355 [40]<br>2745-PST<br>658nt	ZFMK-TIS-23895<br>ZFMK-DNA-0171661253	Spain, Canary Islands, Fuerteventura, Cumbre de Jandia, Pico de Zarza , N28°06'06" W14°21'19", 802m, 12-Jan-2015, *Nauplius sericeus*, beating, leg. Stüben,P., det. Stüben,P.
Laparocerus ellipticus<br>Wollaston, 1863	MK891744 [40]<br>1996-JKR<br>658nt	ZFMK-TIS-23636<br>ZFMK-DNA-0169170514	Spain, Canary Islands, La Palma, Los Tilos, Barranco del Agua, N28°47'06" W17°48'13", 718m, 10-Feb-2014, *Laurisilva*, beating, leg. Krátky,J., det. Krátky,J.
Laparocerus fernandezi<br>Roudier, 1957	MK891742 [40]<br>1994-JKR<br>658nt	ZFMK-TIS-23634<br>ZFMK-DNA-0169170512	Spain, Canary Islands, La Palma, Todoque, Las Norias, N28°36'29" W17°53'55", 373m, 09-Feb-2014, *Rumex lunaria*, beating, leg. Krátky,J., det. Krátky,J.
Laparocerus fritillus<br>(Wollaston, 1854)	MK892379 [40]<br>2835-PST<br>658nt	ZFMK-TIS-4745<br>ZFMK-DNA-FC17941541	Portugal, Madeira, Porto Santo Isl., Pico d Ana Ferreira, N33°02'43" W16°22'13", 206m, 26-Nov-2015, *Cynara cardunculus*, sieving, leg. Stüben,P. & Schütte,A., det. Stüben,P.
Laparocerus inaequalis globulipennis<br>Wollaston, 1864	MK891485 [40]<br>1559-JKR<br>658nt	ZFMK-TIS-3607<br>ZFMK-DNA-0155635670	Spain, Canary Islands, La Palma, Cubo de Galga, N28°45'42" W17°46'34", 467m, 28-Jan-2013, *Rubus*, beating, leg. Krátky,J., det. Krátky,J.
Laparocerus lepidopterus lepidopterus<br>Wollaston, 1864	MK891489 [40]<br>1568-JKR<br>658nt	ZFMK-TIS-3616<br>ZFMK-DNA-0155635662	Spain, Canary Islands, La Palma, Montana Tagoja, N28°43'16" W17°47'07", 1052m, 30-Jan-2013, *Cistus*, beating, leg. Krátky,J., det. Machado,A.
Laparocerus longipennis<br>Machado, 2011	MK892356 [40]<br>2746-PST<br>658nt	ZFMK-TIS-23896<br>ZFMK-DNA-0171661254	Spain, Canary Islands, Fuerteventura, Lajita–Gardon, FV56, km 5.2, N28°13'47" W14°08'16", 102m, 18-Jan-2015, *Salsola vermiculata*, beating, leg. Stüben,P., det. Stüben,P.
Laparocerus mateui<br>Roudier, 1954	MK891595 [40]<br>1672-PST<br>658nt	ZFMK-TIS-24390<br>ZFMK-DNA-0155622518	Spain, Canary Islands, La Gomera, La Palmita (La Laguneta), N28°09'29" W17°13'30", 773m, 06-Dez-2013, beating, leg. Stüben,P., det. Stüben,P.
Laparocerus maxorata<br>Machado, 2011	MK892352 [40]<br>2738-PST<br>658nt	ZFMK-TIS-23888<br>ZFMK-DNA-0171661261	Spain, Canary Islands, Fuerteventura, Cumbre de Jandia, Pico de Zarza , N28°06'06" W14°21'19", 802m, 12-Jan-2015, *Nauplius sericeus*, beating, leg. Stüben,P., det. Stüben,P.
Laparocerus mendax<br>(Wollaston, 1854)	MK892380 [40]<br>2838a-PST<br>658nt	ZFMK-TIS-4748<br>ZFMK-DNA-FC17941470	Portugal, Madeira, Porto Santo Isl., Cabeco d Ponta, N33°02'05" W16°21'49", 5m, 26-Nov-2015, *Euphorbia paralias, Crithmum maritimum*, sieving, leg. Stüben,P. & Schütte,A., det. Stüben,P.
Laparocerus merigensis<br>Machado, 2015	MK891600 [40]<br>1680-PST<br>658nt	ZFMK-TIS-24398<br>ZFMK-DNA-0155622509	Spain, Canary Islands, La Gomera, Meriga I near Laurisilva (botanical gardens), N28°09'15" W17°14'10", 824m, 08-Dez-2013, *Sonchus*, sieving, leg. Stüben,P., det. Stüben,P.
Laparocerus morio<br>Boheman, 1834	MK891716 [40]<br>1943-PST<br>658nt	ZFMK-TIS-24022<br>ZFMK-DNA-0169170406	Portugal, Madeira, Faial, coast, N32°47'37" W16°50'57", 26m, 04-Okt-2014, sieving, leg. Stüben,P., det. Stüben,P.
Laparocerus oromii<br>Machado, 2008	FJ716583 [01]<br>E-738-LAP<br>658nt	ZFMK-TIS-cE738<br>ZFMK-DNA-0100404808	Spain, Canary Islands, La Gomera, S of Hermigua, El Cedro, N28°08'18" W17°12'52", 791m, 09-Okt-2008, *Persea indica*, leg. Astrin,J. & Stüben,P., det. Stüben,P.
Laparocerus palmensis<br>Har. Lindberg, 1953	MK891743 [40]<br>1995-JKR<br>658nt	ZFMK-TIS-23635<br>ZFMK-DNA-0169170513	Spain, Canary Islands, La Palma, Los Llanos, Montana Tenisca, N28°39'22.5" W17°54'55", 377m, 09-Feb-2014, *Artemisia thuscula*, sweeping, leg. Krátky,J., det. Krátky,J.
Laparocerus rasus betancor<br>Machado, 2011	MK892360 [40]<br>2751-PST<br>658nt	ZFMK-TIS-23901<br>ZFMK-DNA-0171661909	Spain, Canary Islands, Fuerteventura, SW of Corralejo, Bayuyo-Caldera, N28°42'59" W13°53'46", 116m, 29-Jan-2015, *Salsola vermiculata*, leg. Stüben,P., det. Stüben,P.

**Name**<br>**Authority**<br>**Additional Information**	**GenBank Acc No (Ref.)**<br>**Specimen ID**<br>**Sequence Length**	**ZFMK Tissue ID**<br>**ZFMK DNA Sample ID**<br>**(SDEI DNA Sample ID)**	**Locality, GPS, Collection Date, Plant, Collector, Identifier**
Laparocerus rasus jandiensis<br>Machado, 2011	MK892353 [40]<br>2740-PST<br>658nt	ZFMK-TIS-23890<br>ZFMK-DNA-0171661248	Spain, Canary Islands, Fuerteventura, Cumbre de Jandia, Pico de Zarza , N28°06'06" W14°21'19", 802m, 12-Jan-2015, *Kleinia*, beating, leg. Stüben,P., det. Stüben,P.
Laparocerus sculptipennis montivagans<br>Machado, 2013	MK891740 [40]<br>1992-JKR<br>658nt	ZFMK-TIS-23632<br>ZFMK-DNA-0169170510	Spain, Canary Islands, La Palma, Pared Vieja, Cueva del Diablo, N28°37'11" W17°49'40.5", 1290m, 09-Feb-2014, sieving, leg. Krátky,J., det. Machado,A.
Laparocerus seriesetosus<br>(Wollaston, 1864)	MK891483 [40]<br>1553-JKR<br>644nt	ZFMK-TIS-3601<br>ZFMK-DNA-0155635676	Spain, Canary Islands, La Palma, Las Caletas, N28°29'34" W17°49'46", 418m, 28-Jan-2013, *Ficus carica*, sieving, leg. Krátky,J., det. Machado,A.
Laparocerus tanausu<br>Machado, 2009	MK891745 [40]<br>1997-JKR<br>658nt	ZFMK-TIS-23637<br>ZFMK-DNA-0169170515	Spain, Canary Islands, La Palma, Punta Santa Lucia, N28°43'20" W17°44'07", 67m, 10-Feb-2014, *Lavandula multifida*, beating, leg. Krátky,J., det. Krátky,J.
Laparocerus teselinde<br>Machado, 2015	MK892344 [40]<br>2720-PST<br>658nt	ZFMK-TIS-23870<br>ZFMK-DNA-0171661276	Spain, Canary Islands, La Gomera, N of Arguamul, Guillama, N28°12'12" W17°18'01", 159m, 30-Dez-2014, *Euphorbia balsamifera* , sieving, leg. Stüben,P., det. Machado,A.
Laparocerus vestitus<br>Wollaston, 1864	MK891486 [40]<br>1562-JKR<br>658nt	ZFMK-TIS-3610<br>ZFMK-DNA-0155635656	Spain, Canary Islands, La Palma, Juan Adalid, N28°50'28" W17°54'27", 362m, 29-Jan-2013, *Echium brevirame*, beating, leg. Krátky,J., det. Machado,A.
Laparocerus vestitus<br>Wollaston, 1864	MK891750 [40]<br>2004-JKR<br>658nt	ZFMK-TIS-23644<br>ZFMK-DNA-0169170497	Spain, Canary Islands, La Palma, near La Fajana, Barranco de los Hombres, N28°49'52" W17°52'05", 343m, 13-Feb-2014, *Argyranthemum*, beating, leg. Krátky,J., det. Machado,A.
Laparocerus xericola<br>Machado, 2011	MK892357 [40]<br>2747-PST<br>658nt	ZFMK-TIS-23897<br>ZFMK-DNA-0171661255	Spain, Canary Islands, Fuerteventura, 1 km SW of La Oliva, N28°36'32" W13°56'22", 213m, 19-Jan-2015, *Argyranthemum winteri*, beating, leg. Stüben,P., det. Stüben,P.
Larinus cf. *sturnus*	KC783874 [new]<br>134-PST<br>576nt	ZFMK-TIS-2D100440293<br>ZFMK-DNA-0100438181	Morocco, S of Berkane, N of Aïn-es-Sfa, Beni Snassen Mts., N34°49'54" W02°08'49", 985m, 13-Mai-2011, *Asteraceae*, beating, leg. Stüben,P., det. Behne,L.
Larinus cynarae<br>(Fabricius, 1787)	MK891325 [new]<br>1331-PST<br>658nt	ZFMK-TIS-4147<br>ZFMK-DNA-0100426152	Spain, Malaga, SE of Antequera, Sierra de las Cabras, N36°59'41" W04°32'04", 607m, 11-Mai-2013, *Cistus*, beating, sieving, leg. Stüben,P. & Schütte,A., det. Stüben,P.
Larinus iaceae<br>(Fabricius, 1775)	MK891995 [new]<br>2266-JKR<br>658nt	ZFMK-TIS-24196<br>ZFMK-DNA-0169171000	Austria, Styria, Hochschwab, Stallmauer, N47°35'29" E15°02'20", 1260m, 27-Jun-2014, mountain forest, sweeping, leg. Krátky,J., det. Krátky,J.
Larinus minutus<br>Gyllenhal, 1835	KC784120 [new]<br>663-FBA<br>658nt	ZFMK-TIS-20445<br>ZFMK-DNA-0100448604	Greece, Chalcidice, Polygyros env., N40°21'55" E23°24'56", 390m, 11-Okt-2010, leg. Bahr,F. & Winkelmann,H., det. Bahr,F.
Larinus nanus<br>Lucas, 1847	KC783857 [new]<br>171-PST<br>658nt	ZFMK-TIS-2D100440265<br>ZFMK-DNA-0100438153	Morocco, Atlas Mts., SW of Taza, Jbel Tazzeka, Gorges du Zireg, N34°07'37" W04°18'17", 476m, 18-Mai-2011, *Asteraceae*, beating, leg. Stüben,P., det. Behne,L.
Larinus nubeculosus<br>Gyllenhal, 1835	MK891358 [new]<br>1409-JKR<br>658nt	ZFMK-TIS-4178<br>ZFMK-DNA-0155628524	France, Cevennes Mts., 1 km N of Arphy, N44°02'23" E03°35'18", 703m, 13-Jun-2013, *Centaurea* sp., individual collecting by hand, leg. Krátky,J., det. Krátky,J.
Larinus obtusus<br>Gyllenhal, 1835	MK892020 [new]<br>2293-JKR<br>658nt	ZFMK-TIS-24224<br>ZFMK-DNA-0169170972	Austria, Kärnten, Feistritz ob Bleiburg, N46°32'17" E14°46'28", 702m, 28-Jun-2014, *Centaurea jacaea*, collecting by hand, leg. Krátky,J., det. Krátky,J.

**Name**<br>**Authority**<br>**Additional Information**	**GenBank Acc No (Ref.)**<br>**Specimen ID**<br>**Sequence Length**	**ZFMK Tissue ID**<br>**ZFMK DNA Sample ID**<br>**(SDEI DNA Sample ID)**	**Locality, GPS, Collection Date, Plant, Collector, Identifier**
Larinus planus<br>(Fabricius, 1792)	MK891581 [new]<br>367-JKR<br>658nt	ZFMK-TIS-20153<br>ZFMK-DNA-0155630445	Czech Republic, Bohemia or., Hrobice, N50°6'2.909" E15°46'50.867", 230m, 05-Jul-2011, sweeping, leg. Krátky,J., det. Krátky,J.
Larinus planus<br>(Fabricius, 1792)	MK891016 [new]<br>912-CBR<br>658nt	ZFMK-TIS-3825<br>ZFMK-DNA-0100449155	France, Dep. Ardenne, 10 km SE of Vouziers, Brecy-Brieres, N49°19'08" E04°46'30", 100m, 14-Mai-2011, *Cirsium arvense*, sweeping, leg. Braunert,C., det. Braunert,C.
Larinus planus<br>(Fabricius, 1792)	MK892180 [new]<br>2472-PSP<br>658nt	ZFMK-TIS-23444<br>ZFMK-DNA-0171661810	Germany, Lower Saxony (NI), Hannover region, Rethen, N52°16'38" E09°49'18", 59m, 08-Jul-2013, *Carduus crispus*, beating, leg. Sprick,P., det. Sprick,P.
Larinus planus<br>(Fabricius, 1792)	MK892285 [new]<br>2586-PSP<br>658nt	ZFMK-TIS-23560<br>ZFMK-DNA-0171661974	Germany, Bavaria (BY), Unterfranken, Ebern, N50°05'20.5" E10°45'59", 333m, 13-Jun-2014, *Cirsium arvense*, leg. Sprick,P., det. Sprick,P.
Larinus pollinis<br>(Laicharting, 1781)	MK891586 [new]<br>555-RST<br>658nt	ZFMK-TIS-20340<br>ZFMK-DNA-0155630424	Czech Republic, Moravia mer., Podyji NP, 1 km W of Znojmo-Popice, N48°49'18.74" E16°0'6.10", 330m, 29-Sep-2011, *Carlina vulgaris*, collecting by hand, leg. Stejskal,R., det. Stejskal,R.
Larinus pollinis<br>(Laicharting, 1781)	MK891583 [new]<br>411-RGO<br>658nt	ZFMK-TIS-20197<br>ZFMK-DNA-0155630442	Poland, Stawska Góra, N51°12'21" E23°23'42", 188m, 10-Aug-2011, leg. Gosik,R., det. Gosik,R.
Larinus sp.	KC783734 [new]<br>10-PST<br>638nt	ZFMK-TIS-2D100447037<br>ZFMK-DNA-0100437968	Spain, Canary Islands, Gran Canaria, Santa Lucia, below Cruz las Vueltas, Mirador de las Tederas, N27°54'47" W15°31'48", 818m, 03-Dez-2010, *Carlina salicifolia*, beating, leg. Stüben,P., det. Stüben,P.
Larinus sturnus<br>(Schaller, 1783)	KC784224 [new]<br>412-RGO<br>658nt	ZFMK-TIS-20198<br>ZFMK-DNA-0100449430	Poland, Katy II, N50°40'39" E23°08'25", 244m, 30-Jun-2011, leg. Gosik,R., det. Gosik,R.
Larinus sturnus<br>(Schaller, 1783)	MK891941 [new]<br>2209-JKR<br>658nt	ZFMK-TIS-23947<br>ZFMK-DNA-0169170585	Slovakia, Nove Zamky, Jursky Chlm, N47°48'04" E18°31'27", 113m, 19-Mai-2014, *Carduus* sp., beating, leg. Stejskal,R., det. Stejskal,R.
Larinus turbinatus<br>Gyllenhal, 1835	MK890835 [new]<br>291-PSP<br>658nt	ZFMK-TIS-2D100439089<br>ZFMK-DNA-0100439534	Germany, Hesse (HE), Wiesbaden-Schierstein, N50°02'58" E08°12'28", 283m, 31-Mai-2011, *Carduus acanthoides*, beating, leg. Sprick,P., det. Sprick,P.
Larinus turbinatus<br>Gyllenhal, 1835	MK892331 [40]<br>2633-PSP<br>658nt	ZFMK-TIS-23607<br>ZFMK-DNA-0171661925	Germany, Lower Saxony (NI), Hannover, Nordhafen, N52°25'26" E09°39'13", 52m, 22-Jun-2014, *Cirsium arvense*, beating, leg. Sprick,P., det. Sprick,P.
Larinus turbinatus<br>Gyllenhal, 1835	MK891345 [new]<br>1382-PST<br>658nt	ZFMK-TIS-4678<br>ZFMK-DNA-0155628551	Italy, Abruzzo, P.N. Majella, 11 km N of Roccaraso, Bosco di S. Antonio, N41°56'27" E14°01'41", 1321m, 20-Aug-2013, beating, leg. Stüben,P., det. Stüben,P.
Larinus turbinatus<br>Gyllenhal, 1835	MK891040 [new]<br>938-CBR<br>658nt	ZFMK-TIS-3851<br>ZFMK-DNA-0100449664	Luxembourg, 10 km E of Luxembourg, 1 km SW of Mensdorf, N49°39'32" E06°18'35", 300m, 09-Jun-2012, *Cirsium arvense*, sweeping, leg. Braunert,C., det. Braunert,C.
Larinus turbinatus<br>Gyllenhal, 1835	MK890857 [new]<br>408-RGO<br>658nt	ZFMK-TIS-20194<br>ZFMK-DNA-0100449426	Poland, Rudnik, N51°14'31.02" E22°32'22.92", 191m, 05-Jun-2011, leg. Gosik,R., det. Gosik,R.
Larinus turbinatus<br>Gyllenhal, 1835	MK890862 [new]<br>422-RGO<br>566nt	ZFMK-TIS-20208<br>ZFMK-DNA-0100449119	Poland, Katy II, N50°40'60" E23°06'60", 249m, 30-Jun-2011, leg. Gosik,R., det. Gosik,R.

**Name**<br>**Authority**<br>**Additional Information**	**GenBank Acc No (Ref.)**<br>**Specimen ID**<br>**Sequence Length**	**ZFMK Tissue ID**<br>**ZFMK DNA Sample ID**<br>**(SDEI DNA Sample ID)**	**Locality, GPS, Collection Date, Plant, Collector, Identifier**
Larinus turbinatus<br>Gyllenhal, 1835	MK892151 [new]<br>2438-JKR<br>658nt	ZFMK-TIS-26015<br>ZFMK-DNA-0171600660	Slovakia, Nove Zamky, Imel, PR Liscie diery, N47°55'17" E18°09'37", 114m, 20-Sep-2014, sweeping, leg. Krátky,J., det. Krátky,J.
Larinus ursus<br>(Fabricius, 1792)	KC783875 [new]<br>133-PST<br>580nt	ZFMK-TIS-2D100440294<br>ZFMK-DNA-0100438182	Morocco, S of Berkane, N of Aïn-es-Sfa, Beni Snassen Mts., N34°49'54" W02°08'49", 985m, 13-Mai-2011, *Asteraceae*, beating, leg. Stüben,P., det. Stüben,P./vid. Behne,L.
Lasiorhynchites cavifrons<br>(Gyllenhal, 1833)	KC783854 [new]<br>168-PST<br>611nt	ZFMK-TIS-2D100440262<br>ZFMK-DNA-0100438150	Morocco, Atlas Mts., S of Taza, Jbel Tazzeka near Bat-Bou-Idir, N34°03'29" W04°10'21", 1598m, 16-Mai-2011, *Quercus*, beating, leg. Stüben,P., det. Stüben,P./vid. Behne,L.
Lauriacalles acutus<br>(Wollaston, 1864)	FJ716538 [01]<br>C-0137-act<br>658nt	ZFMK-TIS-cC0137<br>ZFMK-DNA-0100400176	Spain, Canary Islands, La Palma, 2.5 km W of La Galga, above Cubo de la Galga, N28°45'18" W17°46'37", 857m, 03-Jul-2006, *Laurisilva*, leg. Stüben,P., det. Stüben,P.
Lauriacalles acutus<br>(Wollaston, 1864)	GU987837 [01]<br>E-0219-acu<br>628nt	ZFMK-TIS-cE0219<br>ZFMK-DNA-0100400241	Spain, Canary Islands, La Palma, 1.5 km W of La Galga, El Corcho, N28°45'03" W17°45'59", 598m, 05-Jul-2006, *Laurisilva, Juglans*, leg. Stüben,P., det. Stüben,P.
Lauriacalles acutus<br>(Wollaston, 1864)	MG229726 [40]<br>1709-PST<br>658nt	ZFMK-TIS-24427<br>ZFMK-DNA-0155622490	Spain, Canary Islands, La Palma, NW of Puntallana, Cubo de la Galga, N28°45'38" W17°46'42", 537m, 27-Dez-2013, *Laurisilva*, beating, leg. Stüben,P., det. Stüben,P.
Lauriacalles acutus<br>(Wollaston, 1864)	MG229727 [40]<br>1710-PST<br>658nt	ZFMK-TIS-24428<br>ZFMK-DNA-0155622491	Spain, Canary Islands, La Palma, NW of Puntallana, Cubo de la Galga, N28°45'38" W17°46'42", 537m, 27-Dez-2013, *Laurisilva*, beating, leg. Stüben,P., det. Stüben,P.
Lauriacalles acutus<br>(Wollaston, 1864)	MG229741 [40]<br>1753-PST<br>658nt	ZFMK-TIS-24471<br>ZFMK-DNA-0155622438	Spain, Canary Islands, La Palma, Pared Vieja, Ladera Espinel, N28°37'35" W17°49'16", 672m, 20-Jan-2014, *Laurisilva*, beating, leg. Stüben,P., det. Stüben,P.
Lauriacalles acutus<br>(Wollaston, 1864)	MG229770 [40]<br>1981-JKR<br>658nt	ZFMK-TIS-23621<br>ZFMK-DNA-0169170520	Spain, Canary Islands, La Palma, Pared Vieja, N28°37'21" W17°49'21", 1163m, 07-Feb-2014, *Laurisilva*, beating, leg. Krátky,J., det. Krátky,J.
Lauriacalles acutus<br>(Wollaston, 1864)	GU987924 [01]<br>E-544-acu<br>658nt	ZFMK-TIS-cE544<br>ZFMK-DNA-0100400594	Spain, Canary Islands, Tenerife, 9 km W of La Laguna, Area Recreativa de Jara, N28°27'31" W16°24'07", 870m, 04-Jan-2004, *Laurisilva*, leg. Stüben,P., det. Stüben,P.
Lauriacalles acutus<br>(Wollaston, 1864)	GU987993 [01]<br>E-674-acu<br>650nt	ZFMK-TIS-cE674<br>ZFMK-DNA-0100404888	Spain, Canary Islands, Tenerife, NE of La Laguna, Anaga Mts. near Moquinal, N28°31'55" W16°17'24", 840m, 29-Sep-2008, *Laurisilva*, leg. Astrin,J. & Stüben,P., det. Stüben,P.
Lauriacalles acutus<br>(Wollaston, 1864)	GU987998 [01]<br>E-680-acu<br>658nt	ZFMK-TIS-cE680<br>ZFMK-DNA-0100404875	Spain, Canary Islands, Tenerife, NE of La Laguna, Anaga Mts. near Chinobre, N28°33'21" W16°10'46", 808m, 30-Sep-2008, *Laurus, Ixanthus viscosus*, leg. Astrin,J. & Stüben,P., det. Stüben,P.
Lauriacalles acutus<br>(Wollaston, 1864)	GU988088 [01]<br>E-816-acu<br>658nt	ZFMK-TIS-cE816<br>ZFMK-DNA-0100404525	Spain, Canary Islands, Tenerife, SW of Los Silos, Teno Mts., Monte del Agua, Chupadero, N28°19'23" W16°49'12", 940m, 02-Okt-2008, *Laurus novocanariensis*, leg. Astrin,J.,Stüben,P.,Behne,L.,Floren,L., det. Stüben,P.
Leiosoma apionides<br>(Wollaston, 1864)	KC783781 [40]<br>72-PST<br>658nt	ZFMK-TIS-2D100446985<br>ZFMK-DNA-0100438057	Spain, Canary Islands, La Gomera, S of Hermigua, Meriga, N28°09'15" W17°14'10", 824m, 07-Feb-2011, *Urtica*, beating, leg. Stüben,P., det. Stüben,P.
Leiosoma apionides<br>(Wollaston, 1864)	KC784306 [40]<br>743-PST<br>658nt	ZFMK-TIS-3080<br>ZFMK-DNA-0100448144	Spain, Canary Islands, Tenerife, Anaga Mts., El Bailadero, N28°33'01" W16°12'14", 694m, 05-Feb-2012, *Gesnouinia arborea*, beating, leg. Stüben,P. & Schütte,A., det. Stüben,P.

**Name**<br>**Authority**<br>**Additional Information**	**GenBank Acc No (Ref.)**<br>**Specimen ID**<br>**Sequence Length**	**ZFMK Tissue ID**<br>**ZFMK DNA Sample ID**<br>**(SDEI DNA Sample ID)**	**Locality, GPS, Collection Date, Plant, Collector, Identifier**
Leiosoma concinnum<br>Boheman, 1842	MK892008 [new]<br>2281-JKR<br>658nt	ZFMK-TIS-24212<br>ZFMK-DNA-0169170995	Austria, Styria, Hochschwab, Stallmauer, N47°35'29" E15°02'20", 1260m, 27-Jun-2014, mountain forest, sweeping, leg. Krátky,J., det. Krátky,J.
Leiosoma cribrum<br>(Gyllenhal, 1834)	MK892007 [new]<br>2280-JKR<br>658nt	ZFMK-TIS-24211<br>ZFMK-DNA-0169170994	Austria, Styria, Hochschwab, Spitzkögel, N47°35'27" E15°01'26", 1659m, 27-Jun-2014, alpine grassland, sieving, leg. Krátky,J., det. Krátky,J.
Leiosoma cribrum<br>(Gyllenhal, 1834)	MK892267 [new]<br>2567-PSP<br>658nt	ZFMK-TIS-23541<br>ZFMK-DNA-0171662003	Germany, Saxony-Anhalt (ST), Harz National Park, Ilsenburg, Wienberg, N51°52'15" E10°39'58", 289m, 10-Jun-2014, *Viola hirta*, leg. Sprick,P., det. Sprick,P.
Leiosoma cribrum<br>(Gyllenhal, 1834)	MK891272 [new]<br>1216-JKR<br>658nt	ZFMK-TIS-3552<br>ZFMK-DNA-0100426068	Romania, Maramures, Rodna Mts., Gargalau Mt., N47°35'15" E24°48'27", 1549m, 07-Sep-2012, sieving, leg. Krátky,J., det. Krátky,J.
Leiosoma cyanopterum<br>L. Redtenbacher, 1847	MK892009 [new]<br>2282-JKR<br>658nt	ZFMK-TIS-24213<br>ZFMK-DNA-0169170983	Austria, Styria, Hochschwab, Spitzkögel, N47°35'27" E15°01'26", 1659m, 27-Jun-2014, *Valeriana* sp., sieving, leg. Krátky,J., det. Krátky,J.
Leiosoma deflexum<br>(Panzer, 1795)	KC783946 [new]<br>318-JKR<br>658nt	ZFMK-TIS-20104<br>ZFMK-DNA-0100438765	Czech Republic, Bohemia or., Dvakacovice, N49°58'35.447" E15°54'2.243", 245m, 14-Mai-2011, sifting, leg. Krátky,J., det. Krátky,J.
Leiosoma deflexum<br>(Panzer, 1795)	MK891277 [new]<br>1224-JKR<br>658nt	ZFMK-TIS-3560<br>ZFMK-DNA-0100426060	Czech Republic, Bohemia, Velke Petrovice, N50°30'32" E16°12'42", 401m, 21-Okt-2012, *Salix, Alnus*, sieving, leg. Krátky,J., det. Krátky,J.
Leiosoma deflexum<br>(Panzer, 1795)	MK891099 [new]<br>1003-PSP<br>658nt	ZFMK-TIS-3148<br>ZFMK-DNA-0100426856	Germany, Lower Saxony (NI), Aerzen-Ahorn, Lkr. Hameln-Pyrmont, N52°03'17" E09°13'14", 157m, 05-Mai-2012, *Anemone nemorosa*, bog forest, dipnet, leg. Sprick,P., det. Sprick,P.
Lepidapion curvipilosum<br>(Wagner, 1908)	MK892398 [40]<br>2876-PST<br>658nt	ZFMK-TIS-25881<br>ZFMK-DNA-0169166916	Spain, Canary Islands, El Hierro, El Golfo, 1.5 km S of Los Llanillos, N27°44'22" W18°01'40", 725m, 13-Apr-2016, *Chamaecytisus proliferus*, sieving, leg. Stüben,P. & Schütte,A., det. Stüben,P.
Lepidapion curvipilosum<br>(Wagner, 1908)	MK892400 [40]<br>2878-PST<br>658nt	ZFMK-TIS-25883<br>ZFMK-DNA-0169166905	Spain, Canary Islands, El Hierro, La Dehesa, NW of Ermita Virgen de los Reyes, N27°43'55" W18°07'18", 696m, 14-Apr-2016, *Chamaecytisus proliferus*, beating, leg. Stüben,P. & Schütte,A., det. Stüben,P.
Lepidapion curvipilosum<br>(Wagner, 1908)<br>formerly: *L. spartocytisi* - Syn!	KC783738 [40]<br>16-PST<br>658nt	ZFMK-TIS-2D100447026<br>ZFMK-DNA-0100438098	Spain, Canary Islands, Gran Canaria, Moya, Barranco de los Tilos, N28°04'33" W15°36'05", 643m, 07-Dez-2010, *Chamaecytiscus proliferus*, beating, leg. Stüben,P., det. Stüben,P.
Lepidapion curvipilosum<br>(Wagner, 1908)	KC783782 [40]<br>73-PST<br>633nt	ZFMK-TIS-2D100446986<br>ZFMK-DNA-0100437951	Spain, Canary Islands, La Gomera, El Cedro, Mirador de El Bailadero, N28°07'22" W17°12'29", 1045m, 08-Feb-2011, *Chamaecytisus proliferus*, beating, leg. Stüben,P., det. Stüben,P.
Lepidapion curvipilosum<br>(Wagner, 1908)	MK891594 [40]<br>1671-PST<br>658nt	ZFMK-TIS-24389<br>ZFMK-DNA-0155622519	Spain, Canary Islands, La Gomera, above Agulo, (El Chorro), N28°11'02" W17°11'58", 505m, 06-Dez-2013, *Fabaceae*, beating, leg. Stüben,P., det. Stüben,P.
Lepidapion curvipilosum<br>(Wagner, 1908)	MK891613 [40]<br>1717-PST<br>658nt	ZFMK-TIS-24435<br>ZFMK-DNA-0155622473	Spain, Canary Islands, La Palma, Montes de Luna, N28°31'57" W17°48'37", 583m, 28-Dez-2013, *Tagasaste (Fabaceae)*, beating, leg. Stüben,P., det. Stüben,P.
Lepidapion curvipilosum<br>(Wagner, 1908)	MK891619 [40]<br>1726-PST<br>658nt	ZFMK-TIS-24444<br>ZFMK-DNA-0155622459	Spain, Canary Islands, La Palma, NE of Garafia, Llano Negro, N28°48'12" W17°55'24", 602m, 29-Dez-2013, *Chamaecytisus proliferus*, beating, leg. Stüben,P., det. Stüben,P.

**Name**<br>**Authority**<br>**Additional Information**	**GenBank Acc No (Ref.)**<br>**Specimen ID**<br>**Sequence Length**	**ZFMK Tissue ID**<br>**ZFMK DNA Sample ID**<br>**(SDEI DNA Sample ID)**	**Locality, GPS, Collection Date, Plant, Collector, Identifier**
Lepidapion curvipilosum<br>(Wagner, 1908)<br>formerly: *L. spartocytisi* - Syn!	KC784289 [40]<br>710-PST<br>658nt	ZFMK-TIS-3047<br>ZFMK-DNA-0100448184	Spain, Canary Islands, Tenerife, Teno Mts., Erjos, N28°19'45" W16°48'21", 1003m, 07-Jan-2012, *Teline*, beating, leg. Stüben,P., det. Stüben,P.
Lepidapion curvipilosum<br>(Wagner, 1908)<br>formerly: *L. spartocytisi* - Syn!	KC784292 [40]<br>718-PST<br>658nt	ZFMK-TIS-3055<br>ZFMK-DNA-0100448169	Spain, Canary Islands, Tenerife, N of Vilaflor, N28°10'38" W16°38'42", 1771m, 14-Jan-2012, *Chamaecytisus*, beating, leg. Stüben,P., det. Stüben,P.
Lepidapion senex<br>(Wollaston, 1864)	KC783800 [40]<br>93-PST<br>658nt	ZFMK-TIS-2D100446958<br>ZFMK-DNA-0100438030	Spain, Canary Islands, La Gomera, W of Epina near Alojera, N28°09'18" W17°18'39", 728m, 26-Feb-2011, *Retama (Fabaceae)*, beating, leg. Stüben,P., det. Stüben,P.
Lepidapion senex<br>(Wollaston, 1864)	MK891609 [40]<br>1696-PST<br>658nt	ZFMK-TIS-24414<br>ZFMK-DNA-0155622494	Spain, Canary Islands, La Palma, near Puntagorda, coast, N28°46'14" W17°59'39", 548m, 20-Dez-2013, *Retama raetam*, beating, leg. Stüben,P., det. Stüben,P.
Lepidapion senex<br>(Wollaston, 1864)	MK891620 [40]<br>1727-PST<br>658nt	ZFMK-TIS-24445<br>ZFMK-DNA-0155622460	Spain, Canary Islands, La Palma, S of Mazo near Salemera, coast, N28°34'09" W17°45'44", 10m, 30-Dez-2013, *Retama raetam*, beating, leg. Stüben,P., det. Stüben,P.
Lepyrus capucinus<br>(Schaller, 1783)	MK891837 [new]<br>2098-JKR<br>658nt	ZFMK-TIS-23738<br>ZFMK-DNA-0169169635	Czech Republic, Moravia, Krumvir, PR Louky pod Kumstatem, N48°59'36" E16°55'20.5", 211m, 09-Mai-2014, sweeping, leg. Krátky,J., det. Krátky,J.
Leucophyes pedestris<br>(Poda, 1761)	KC784112 [new]<br>497-RST<br>658nt	ZFMK-TIS-20282<br>ZFMK-DNA-0100448482	Romania, Caras-Severin, Sfanta Elena env., N44°40'42.32" E21°43'13.01", 350m, 29-Apr-2011, hand-collecting, leg. Stejskal,R., det. Stejskal,R.
Lignyodes bischoffi<br>(Blatchley, 1916)	MK890865 [new]<br>435-RGO<br>658nt	ZFMK-TIS-20221<br>ZFMK-DNA-0100449405	Poland, Lublin, N51°13'23.7" E22°31'16.6", 192m, 05-Sep-2011, leg. Gosik,R., det. Gosik,R.
Lignyodes enucleator<br>(Panzer, 1798)	KC784185 [new]<br>484-PST<br>658nt	ZFMK-TIS-20269<br>ZFMK-DNA-0100449358	France, Isere, St.-Jeam-de-Bournay, N45°30'28" E05°09'09", 403m, 22-Jul-2011, beating, leg. Stüben,P., det. Stüben,P.
Limnobaris dolorosa<br>(Goeze, 1777)	MK891232 [new]<br>1152-JKR<br>642nt	ZFMK-TIS-3488<br>ZFMK-DNA-0100449556	Czech Republic, Bohemia, Hodesovice, PR Mazurova louka, N50°09'41" E15°55'43", 270m, 20-Mai-2012, sweeping, leg. Krátky,J., det. Krátky,J.
Limnobaris dolorosa<br>(Goeze, 1777)	MK892175 [new]<br>2467-PSP<br>658nt	ZFMK-TIS-23439<br>ZFMK-DNA-0171661805	Germany, Lower Saxony (NI), Harz National Park, Torfhaus, Marienbruch, N51°49'57" E10°31'46", 616m, 30-Mai-2013, *Carex rostrata*, beating, leg. Sprick,P., det. Sprick,P.
Limnobaris dolorosa<br>(Goeze, 1777)	MK890999 [new]<br>891-CBR<br>658nt	ZFMK-TIS-3804<br>ZFMK-DNA-0100449713	Luxembourg, 10 km E of Luxembourg, 0.5 km W of Uebersyren, N49°38'19" E06°16'31", 240m, 19-Apr-2011, *Carex*, sweeping, leg. Braunert,C., det. Braunert,C.
Limnobaris t-album<br>(Linnaeus, 1758)	MK892057 [new]<br>2333-JKR<br>658nt	ZFMK-TIS-24264<br>ZFMK-DNA-0169170932	Slovakia, Povazska Bystrica, Bodina, N49°06'46" E18°33'25", 450m, 13-Jul-2014, sweeping, leg. Krátky,J., det. Krátky,J.
Limnobaris t-album t-album<br>(Linnaeus, 1758)	MK890998 [new]<br>890-CBR<br>658nt	ZFMK-TIS-3803<br>ZFMK-DNA-0100449712	Luxembourg, 10 km E of Luxembourg, 0.5 km W of Uebersyren, N49°38'19" E06°16'31", 240m, 19-Apr-2011, *Carex*, sweeping, leg. Braunert,C., det. Braunert,C.
Limobius borealis<br>(Paykull, 1792)	KC783943 [new]<br>324-JKR<br>658nt	ZFMK-TIS-20110<br>ZFMK-DNA-0100438759	Czech Republic, Bohemia or., Hradec Kralove - Fararstvi, N50°11'22.247" E15°48'53.64", 230m, 17-Mai-2011, *Geranium* sp., sweeping, leg. Krátky,J., det. Krátky,J.

**Name**<br>**Authority**<br>**Additional Information**	**GenBank Acc No (Ref.)**<br>**Specimen ID**<br>**Sequence Length**	**ZFMK Tissue ID**<br>**ZFMK DNA Sample ID**<br>**(SDEI DNA Sample ID)**	**Locality, GPS, Collection Date, Plant, Collector, Identifier**
Limobius borealis<br>(Paykull, 1792)	MK890873 [new]<br>483-PST<br>658nt	ZFMK-TIS-20268<br>ZFMK-DNA-0100449357	France, Isere, St.-Jeam-de-Bournay, N45°30'28" E05°09'09", 403m, 22-Jul-2011, beating, leg. Stüben,P., det. Stüben,P.
Limobius borealis<br>(Paykull, 1792)	MK892204 [new]<br>2500-PSP<br>658nt	ZFMK-TIS-23472<br>ZFMK-DNA-0171661773	Germany, Lower Saxony (NI), Berkhof, Hannover region, N52°36'46" E09°43'45", 42m, 29-Apr-2014, *Geranium pusillum*, beating, leg. Sprick,P., det. Sprick,P.
Limobius borealis<br>(Paykull, 1792)	KC783835 [new]<br>118-PST<br>658nt	ZFMK-TIS-2D100440308<br>ZFMK-DNA-0100417619	Morocco, S of Berkane, Mts. des Beni Snassen, Vallee Zegzel, N34°49'33“ W02°22'37“, 590m, 11-Mai-2011, beating, leg. Stüben,P., det. Behne,L.
Limobius borealis<br>(Paykull, 1792)	MK892035 [new]<br>2311-JKR<br>658nt	ZFMK-TIS-24242<br>ZFMK-DNA-0169170954	Slovakia, Tatry Mts., Dolina Siedmich prame?ov, N49°13'05" E20°17'12", 1051m, 07-Jul-2014, mountain meadow, sweeping, leg. Krátky,J., det. Krátky,J.
Liophloeus lentus<br>Germar, 1824	MK892066 [new]<br>2342-JKR<br>658nt	ZFMK-TIS-24273<br>ZFMK-DNA-0169170912	Czech Republic, Bohemia, Horni Morava, N50°10'15" E16°49'22", 780m, 17-Jul-2014, sweeping, leg. Krátky,J., det. Krátky,J.
Liophloeus liptoviensis<br>J. Weise, 1894	KC784216 [new]<br>425-RGO<br>658nt	ZFMK-TIS-20211<br>ZFMK-DNA-0100449414	Poland, Kiry, Tatra Mountains, N49°16'26" E19°52'07", 942m, 06-Jul-2011, leg. Gosik,R., det. Gosik,R.
Liophloeus liptoviensis<br>J. Weise, 1894	MK890863 [new]<br>428-RGO<br>658nt	ZFMK-TIS-20214<br>ZFMK-DNA-0100449411	Poland, Czerwone Wierchy, Tatra Mountains, N49°16'58.14" E19°52'16.08", 1393m, 06-Jul-2011, leg. Gosik,R., det. Gosik,R.
Liophloeus liptoviensis<br>J. Weise, 1894	MK891366 [new]<br>1417-JKR<br>658nt	ZFMK-TIS-4186<br>ZFMK-DNA-0155628516	Slovakia, Vysoke Tatry Mts., Dolina siedmich prame?ov, N49°13'38" E20°16'34", 1448m, 30-Jun-2013, sweeping, leg. Krátky,J., det. Krátky,J.
Liophloeus liptoviensis<br>J. Weise, 1894	MK892038 [new]<br>2314-JKR<br>658nt	ZFMK-TIS-24245<br>ZFMK-DNA-0169170951	Slovakia, Tatry Mts., Dolina Siedmich prame?ov, N49°12'57" E20°17'46", 981m, 05-Jul-2014, mountain meadow, sweeping, leg. Krátky,J., det. Krátky,J.
Liophloeus tessulatus<br>(O. F. Müller, 1776)	MK890842 [new]<br>313-JKR<br>658nt	ZFMK-TIS-20099<br>ZFMK-DNA-0100438775	Czech Republic, Bohemia or., Dvakacovice, N49°58'35.447" E15°54'2.243", 245m, 14-Mai-2011, sweeping, leg. Krátky,J., det. Krátky,J.
Liophloeus tessulatus<br>(O. F. Müller, 1776)	MK891331 [new]<br>197-PSP<br>658nt	ZFMK-TIS-2D100438503<br>ZFMK-DNA-0100417596	Germany, Lower Saxony (NI), Hannover, Steintormasch, N52°22'57" E09°42'31", 50m, 06-Apr-2011, *Hedera helix*, hedge, beating, leg. Sprick,P., det. Sprick,P.
Liophloeus tessulatus<br>(O. F. Müller, 1776)	MK891406 [new]<br>1468-PSP<br>658nt	ZFMK-TIS-3228<br>ZFMK-DNA-0155633354	Germany, Lower Saxony (NI), Harz, St. Andreasberg, Jordanshöhe, N51°42'58" E10°32'08", 674m, 02-Jul-2012, *Meum athamanticum*, mountain meadow, dipnet, leg. Sprick,P., det. Sprick,P.
Liparus glabrirostris<br>(Küster, 1849)	MK891431 [new]<br>1494-PSP<br>658nt	ZFMK-TIS-3254<br>ZFMK-DNA-0155633332	Germany, Lower Saxony (NI), Harz National Park, Eckertal, Canyon forest (Tilio-Acerion), N51°50'50" E10°34'41", 523m, 01-Aug-2012, *Petasites albus*, Handaufsammlung, leg. Sprick,P., det. Sprick,P.
Listroderes difficilis<br>Germain, 1895	MK890912 [40]<br>772-PST<br>658nt	ZFMK-TIS-3109<br>ZFMK-DNA-0100448115	Spain, Canary Islands, La Gomera, Hermigua, N28°10'4" W17°11'36", 86m, 09-Mrz-2012, *Chenopodiaceae*, beating, leg. Stüben,P., det. Stüben,P.
Lixus anguinus<br>(Linnaeus, 1767)	MK347572 [40]<br>3004-PST<br>658nt	(SDEI-DNA-3004-PST)	Spain, Canary Islands, Lanzarote, Playa de Famara, sandy beach, dunes, N29°06'52" W13°33'22", 6m, 06-Jan-2017, *Cakile maritima*, leg. Stüben,P., det. Stüben,P.

**Name**<br>**Authority**<br>**Additional Information**	**GenBank Acc No (Ref.)**<br>**Specimen ID**<br>**Sequence Length**	**ZFMK Tissue ID**<br>**ZFMK DNA Sample ID**<br>**(SDEI DNA Sample ID)**	**Locality, GPS, Collection Date, Plant, Collector, Identifier**
Lixus anguinus<br>(Linnaeus, 1767)	KC155425 [11]<br>177-PST<br>600nt	ZFMK-TIS-2D100440250<br>ZFMK-DNA-0100438138	Morocco, Atlas Mts., SE of Ifrane, Tizi-n-Tretten, N33°27'25" W05°02'16", 1936m, 19-Mai-2011, *Sinapis*, beating, leg. Stüben,P., det. Stüben,P./vid. Behne,L.
Lixus anguinus<br>(Linnaeus, 1767)	MK891574 [new]<br>177-PST<br>658nt	ZFMK-TIS-2D100440250<br>ZFMK-DNA-0155630453	Morocco, Atlas Mts., SE of Ifrane, Tizi-n-Tretten, N33°27'25" W05°02'16", 1936m, 19-Mai-2011, *Sinapis*, beating, leg. Stüben,P., det. Stüben,P./vid. Behne,L.
Lixus angustus<br>(Herbst, 1795)	MK891388 [new]<br>1447-JKR<br>658nt	ZFMK-TIS-4216<br>ZFMK-DNA-0155628485	Slovakia, Nove Zamky, Bajtava, Ploska hora, N47°51'49" E18°45'24", 130m, 28-Sep-2013, *Picris* sp., sweeping, leg. Krátky,J., det. Krátky,J.
Lixus angustus<br>(Herbst, 1795)	MK891909 [new]<br>2176-JKR<br>658nt	ZFMK-TIS-23914<br>ZFMK-DNA-0169170600	Slovakia, Nove Zamky, Bajtava, Ploska hora, N47°51'54" E18°45'15", 126m, 22-Mai-2014, *Picris hieracioides*, collecting by hand, leg. Stejskal,R., det. Stejskal,R.
Lixus bardanae<br>(Fabricius, 1787)	KC784004 [new]<br>247-PSP<br>658nt	ZFMK-TIS-2D100439124<br>ZFMK-DNA-0100439796	Germany, Lower Saxony (NI), Essel, Lkr. Soltau-Fallingbostel, N52°41'58" E09°38'44", 25m, 10-Mai-2011, *Rumex hydrolapathum*, sand field, collecting by hand, leg. Sprick,P., det. Sprick,P.
Lixus bardanae<br>(Fabricius, 1787)	MK890938 [new]<br>807-PST<br>658nt	ZFMK-TIS-3720<br>ZFMK-DNA-0100413760	Germany, Rhineland-Palatinate (RLP), N of Treis-Karden, Brohl, N50°13'22" E07°16'27", 255m, 10-Jun-2012, *Rumex*, roadside ditch, beating, leg. Stüben,P., det. Stüben,P.
Lixus bardanae<br>(Fabricius, 1787)	MK891241 [new]<br>1161-JKR<br>658nt	ZFMK-TIS-3497<br>ZFMK-DNA-0100449550	Hungary, Jasz-Nagykun-Szolnok megye, Tiszaroff, N47°21'55" E20°27'32", 87m, 25-Mai-2012, *Rumex*, sweeping, leg. Krátky,J., det. Krátky,J.
Lixus bituberculatus<br>Smreczynski, 1968	MK892139 [new]<br>2426-JKR<br>658nt	ZFMK-TIS-26003<br>ZFMK-DNA-0171597935	Romania, Caras-Severin, Sfanta Elena, N44°40'27" E21°42'60", 324m, 06-Aug-2014, *Cichorium intybus*, ex larvae, leg. F. Trnka, det. Trnka,F.
Lixus brevipes<br>C. Brisout de Barneville, 1866	MK891214 [new]<br>1133-JKR<br>658nt	ZFMK-TIS-3469<br>ZFMK-DNA-0100449570	Slovakia, Komarno, Imel, N47°54'34" E18°09'16", 112m, 11-Mai-2012, *Silene latofolia* ssp. *Alba*, sweeping, leg. Krátky,J., det. Krátky,J.
Lixus brevirostris<br>Boheman, 1835	MK892403 [40]<br>2882-PST<br>658nt	ZFMK-TIS-25887<br>ZFMK-DNA-0169166909	Spain, Canary Islands, El Hierro, N of Los Llanillos, N27°45'18" W18°02'31", 168m, 15-Apr-2016, *Chenopodiacea*, beating, leg. Stüben,P. & Schütte,A., det. Stüben,P.
Lixus brevirostris<br>Boheman, 1835	KC783729 [40]<br>1-PST<br>629nt	ZFMK-TIS-2D100446881<br>ZFMK-DNA-0100438004	Spain, Canary Islands, Gran Canaria, Marzagan, Barranco de las Goteras, N28°01'28" W15°27'31", 236m, 01-Dez-2010, *Einadia nutans*, beating, leg. Stüben,P., det. Stüben,P.
Lixus brevirostris<br>Boheman, 1835	KC783763 [40]<br>52-PST<br>658nt	ZFMK-TIS-2D100446995<br>ZFMK-DNA-0100438067	Spain, Canary Islands, Gran Canaria, Moya, El Palmital, N28°06'41" W15°36'07", 525m, 24-Jan-2011, *Einadia nutans*, beating, leg. Stüben,P., det. Stüben,P.
Lixus brevirostris<br>Boheman, 1835	KC783790 [40]<br>81-PST<br>658nt	ZFMK-TIS-2D100446967<br>ZFMK-DNA-0100438039	Spain, Canary Islands, La Gomera, Valle Gran Rey, N28°5'55" W17°20'53", 18m, 15-Feb-2011, *Chenopodiaceae*, beating, leg. Stüben,P., det. Stüben,P.
Lixus brevirostris<br>Boheman, 1835	MK891599 [40]<br>1678-PST<br>658nt	ZFMK-TIS-24396<br>ZFMK-DNA-0155622507	Spain, Canary Islands, La Gomera, Arguamul, N28°11'56" W17°17'54", 407m, 08-Dez-2013, *Chenopodiaceae*, beating, leg. Stüben,P., det. Stüben,P.
Lixus brevirostris<br>Boheman, 1835	MK891615 [40]<br>1722-PST<br>658nt	ZFMK-TIS-24440<br>ZFMK-DNA-0155622468	Spain, Canary Islands, La Palma, NW of Los Canarios (Fuencaliente), Playa Chica, N28°30'52" W17°52'32", 33m, 28-Dez-2013, *Chenopodiaceae*, beating, leg. Stüben,P., det. Stüben,P.

**Name**<br>**Authority**<br>**Additional Information**	**GenBank Acc No (Ref.)**<br>**Specimen ID**<br>**Sequence Length**	**ZFMK Tissue ID**<br>**ZFMK DNA Sample ID**<br>**(SDEI DNA Sample ID)**	**Locality, GPS, Collection Date, Plant, Collector, Identifier**
Lixus brevirostris<br>Boheman, 1835	MK891618 [40]<br>1725-PST<br>658nt	ZFMK-TIS-24443<br>ZFMK-DNA-0155622458	Spain, Canary Islands, La Palma, near Garafia, N28°49'26" W17°57'11", 398m, 29-Dez-2013, *Chenopodiaceae*, beating, leg. Stüben,P., det. Stüben,P.
Lixus brevirostris<br>Boheman, 1835	MK891562 [40]<br>1659-PST<br>658nt	ZFMK-TIS-4703<br>ZFMK-DNA-0155630474	Spain, Canary Islands, Tenerife, Anaga Mts., Taganana, coast, N28°34'18" W16°11'40", 5m, 06-Nov-2012, *Chenopodiaceae*, beating, leg. Stüben,P., det. Stüben,P.
Lixus cardui<br>Olivier, 1807	MK891824 [new]<br>2085-JKR<br>658nt	ZFMK-TIS-23725<br>ZFMK-DNA-0169169643	Czech Republic, Moravia, Bucovice, PR Sevy, N49°08'05" E16°58'19", 249m, 09-Mai-2014, sweeping, leg. Krátky,J., det. Krátky,J.
Lixus cf. *filiformis*	KC783851 [40]<br>162-PST<br>658nt	ZFMK-TIS-2D100440256<br>ZFMK-DNA-0100438144	Morocco, S of Berkane, Mts. des Beni Snassen, Vallee Zegzel, N34°49'19" W02°23'10" , 644m, 15-Mai-2011, beating, leg. Stüben,P., det. Stüben,P./vid. Behne,L.
Lixus cf. *filiformis*	KC784290 [40]<br>715-PST<br>658nt	ZFMK-TIS-3052<br>ZFMK-DNA-0100448172	Spain, Canary Islands, Tenerife, Bosque de Esperanza, Lomo de la Jara, N28°27'27" W16°24'10", 923m, 12-Jan-2012, *thistle*, beating, leg. Stüben,P., det. Stüben,P.
Lixus cf. *pulverulentus*	KC783853 [new]<br>164-PST<br>658nt	ZFMK-TIS-2D100440258<br>ZFMK-DNA-0100438146	Morocco, S of Berkane, Mts. des Beni Snassen, Vallee Zegzel, N34°49'19" W02°23'10" , 644m, 15-Mai-2011, beating, leg. Stüben,P., det. Stüben,P./vid. Behne,L.
Lixus cheiranthi<br>Wollaston, 1854	MK891652 [40]<br>1830-PST<br>658nt	ZFMK-TIS-26033<br>ZFMK-DNA-0171606136	Portugal, Madeira, Sao Jorge, Praia de S. Jorge, N32°49'59" W16°54'00", 24m, 28-Jun-2014, *Brassicaceae*, beating, leg. Stüben,P., det. Stüben,P.
Lixus cinerascens<br>Schoenherr, 1832<br>formerly: *Lixus elegantulus*	MK891876 [new]<br>2140-JKR<br>658nt	ZFMK-TIS-23780<br>ZFMK-DNA-0169169602	Slovakia, Komarno, Imel, PR Liscie diery, N47°55'17" E18°09'37", 114m, 18-Mai-2014, *Crepis* sp., sweeping, leg. Krátky,J., det. Krátky,J.
Lixus erysimi<br>Stüben & Behne, 2013<br>**Paratype (DNAtype)**	KC155424 [11]<br>39-PST_27GC2011<br>645nt	ZFMK-DNA-0100438073	Spain, Canary Islands, Gran Canaria, Cruz de Tejeda, Mirador de Becerra, N27°59'33" W15°35'37", 1548m, 02-Jan-2011, *Erysimum bicolor*, beating, leg. Stüben,P., det. Stüben,P.
Lixus fasciculatus<br>Boheman, 1835	MK891227 [new]<br>1147-JKR<br>658nt	ZFMK-TIS-3483<br>ZFMK-DNA-0100449561	Slovakia, Nove Zamky, Salka, Sovie vinohrady, N47°53'14" E18°43'02", 190m, 12-Mai-2012, *Arthemisia vulgaris*, sweeping, leg. Krátky,J., det. Krátky,J.
Lixus filiformis<br>(Fabricius, 1781)	KC783955 [new]<br>382-JKR<br>658nt	ZFMK-TIS-20168<br>ZFMK-DNA-0100438896	Czech Republic, Bohemia or., Uhretice env., N49°58'39.655" E15°51'28.603", 240m, 05-Jul-2011, sweeping, leg. Krátky,J., det. Krátky,J.
Lixus filiformis<br>(Fabricius, 1781)	KC784062 [new]<br>570-PSP<br>621nt	ZFMK-TIS-20355<br>ZFMK-DNA-0100448409	Germany, Lower Saxony (NI), Neindorf, Lkr. Wolfenbüttel, Öselberg, N52°07'40" E10°35'32", 132m, 24-Jun-2011, *Carduus acanthoides*, beating, leg. Sprick,P., det. Sprick,P.
Lixus filiformis<br>(Fabricius, 1781)	MK891476 [new]<br>1544-PSP<br>658nt	ZFMK-TIS-3304<br>ZFMK-DNA-0155633286	Germany, Lower Saxony (NI), Rethen, Hannover region, N52°16'38" E09°49'19", 59m, 25-Jun-2013, *Carduus crispus*, beating, leg. Sprick,P., det. Sprick,P.
Lixus filiformis<br>(Fabricius, 1781)	MK892253 [new]<br>2552-PSP<br>658nt	ZFMK-TIS-23526<br>ZFMK-DNA-0171662007	Slovakia, Nitra, Muzla, N47°48'00" E18°31'55", 114m, 19-Mai-2014, *Carduus* sp., leg. Sprick,P., det. Sprick,P.
Lixus iridis<br>Olivier, 1807	KC784187 [new]<br>475-PST<br>658nt	ZFMK-TIS-20260<br>ZFMK-DNA-0100449364	Germany, Rhineland-Palatinate (RLP), Mosellus area, Kobern-Gondorf, "Sürzer Höfe", ruderal area, N50°20'20" E07°25'25", 330m, 30-Sep-2011, hand-collecting, leg. Stüben,P., det. Stüben,P.

**Name**<br>**Authority**<br>**Additional Information**	**GenBank Acc No (Ref.)**<br>**Specimen ID**<br>**Sequence Length**	**ZFMK Tissue ID**<br>**ZFMK DNA Sample ID**<br>**(SDEI DNA Sample ID)**	**Locality, GPS, Collection Date, Plant, Collector, Identifier**
Lixus iridis<br>Olivier, 1807	MK890870 [new]<br>470-PSP<br>658nt	ZFMK-TIS-20255<br>ZFMK-DNA-0100449369	Germany, Saxony-Anhalt (ST), Drömling, Mannhausen, N52°25'36" E11°12'37", 74m, 25-Mai-2011, *Angelica archangelica*, body of standing water shore, beating, leg. Sprick,P., det. Sprick,P.
Lixus iridis<br>Olivier, 1807	MK890966 [new]<br>849-PST<br>658nt	ZFMK-TIS-3762<br>ZFMK-DNA-0100414247	Portugal, Estremadura, N of Ericeira, Ribamar, coast, N38°59'29" W09°24'54", 28m, 18-Mai-2012, beating, leg. Stüben,P., det. Stüben,P.
Lixus juncii<br>Boheman, 1835	MK890962 [40]<br>845-PST<br>658nt	ZFMK-TIS-3758<br>ZFMK-DNA-0100414243	Portugal, Estremadura, W of Sintra, Serra de Sintra, S of Colares, N38°46'38" W09°28'37", 241m, 16-Mai-2012, beating, leg. Stüben,P., det. Stüben,P.
Lixus juncii<br>Boheman, 1835	MK891315 [new]<br>1309-PST<br>658nt	ZFMK-TIS-4125<br>ZFMK-DNA-0100425406	Spain, Cordoba, W of Montoro, N38°00'21" W04°24'56", 170m, 04-Mai-2013, *roadside vegetation*, beating, leg. Stüben,P. & Schütte,A., det. Stüben,P.
Lixus linearis<br>Olivier, 1807	MK892346 [40]<br>2722-PST<br>658nt	ZFMK-TIS-23872<br>ZFMK-DNA-0171661278	Spain, Canary Islands, Fuerteventura, Betancuria, N28°25'09" W14°03'32", 380m, 04-Jan-2015, beating, leg. Stüben,P., det. Stüben,P.
Lixus linearis<br>Olivier, 1807	KC783749 [40]<br>37-PST<br>658nt	ZFMK-TIS-2D100446999<br>ZFMK-DNA-0100438071	Spain, Canary Islands, Gran Canaria, E of San Mateo (Barranco), N28°01'08" W15°31'04", 668m, 31-Dez-2010, *Rumex*, beating, leg. Stüben,P., det. Stüben,P.
Lixus linearis<br>Olivier, 1807	MK891604 [40]<br>1688-PST<br>658nt	ZFMK-TIS-24406<br>ZFMK-DNA-0155622502	Spain, Canary Islands, La Palma, near San Isidro, N28°37'47" W17°48'02", 643m, 18-Dez-2013, *Rumex*, beating, leg. Stüben,P., det. Stüben,P.
Lixus linearis<br>Olivier, 1807	MK891668 [40]<br>1870-PST<br>658nt	ZFMK-TIS-26073<br>ZFMK-DNA-0171606107	Portugal, Madeira, Curral das Freiras, N32°42'37" W16°58'16", 395m, 07-Jul-2014, beating, leg. Stüben,P., det. Stüben,P.
Lixus linearis<br>Olivier, 1807	MK891671 [40]<br>1876-PST<br>658nt	ZFMK-TIS-26079<br>ZFMK-DNA-0171606090	Portugal, Madeira, SW of Sao Roque de Faial, road to Faja da Nouqueira, N32°44'26" W16°54'04", 582m, 10-Jul-2014, beating, leg. Stüben,P., det. Stüben,P.
Lixus linearis<br>Olivier, 1807	MK890969 [new]<br>852-PST<br>658nt	ZFMK-TIS-3765<br>ZFMK-DNA-0100414250	Portugal, Estremadura, Serra de Sintra, N38°46'51" W09°26'40", 19m, 19-Mai-2012, *Rumex*, beating, leg. Stüben,P., det. Stüben,P.
Lixus mucronatus<br>(Olivier, 1791)	MK890965 [new]<br>848-PST<br>658nt	ZFMK-TIS-3761<br>ZFMK-DNA-0100414246	Portugal, Estremadura, W of Sintra, Praia de Adraga, coast, N38°48'07" W09°29'04", 14m, 16-Mai-2012, beating, leg. Stüben,P., det. Stüben,P.
Lixus myagri<br>Olivier, 1807	KC784192 [new]<br>465-PST<br>630nt	ZFMK-TIS-20250<br>ZFMK-DNA-0100449374	Germany, Rhineland-Palatinate (RLP), "Korretsberg" near Kruft, Eifel, N50°22'49" E07°21'11", 238m, 30-Sep-2011, *Barbaraea vulgaris*, volcanic cone, leg. Stüben,P., det. Stüben,P.
Lixus neglectus<br>Fremuth, 1983	MK891939 [new]<br>2207-JKR<br>658nt	ZFMK-TIS-23945<br>ZFMK-DNA-0169170583	Slovakia, Senica, Kuty, N48°40'19" E17°00'18", 150m, 19-Jul-2014, *Rumex pratensis*, collecting by hand, leg. Stejskal,R., det. Stejskal,R.
Lixus ochraceus<br>Boheman, 1842	MK891932 [new]<br>2200-JKR<br>658nt	ZFMK-TIS-23938<br>ZFMK-DNA-0169170576	Slovakia, Tranava District, Brestovany, N48°22'15" E17°41'45", 120m, 20-Apr-2014, *Capsella bursa-pastoris*, collecting by hand, leg. Stejskal,R., det. Stejskal,R.
Lixus paraplecticus<br>(Linnaeus, 1758)	MK890886 [new]<br>532-RST<br>658nt	ZFMK-TIS-20317<br>ZFMK-DNA-0100448442	Czech Republic, Bohemia mer., Hrachoviste, 8.5 km S of Trebon, N48°56'4.01" E14°46'36.31", 460m, 13-Aug-2011, *Oenanthe aquatica*, hand-collecting, leg. Stejskal,R., det. Stejskal,R.

**Name**<br>**Authority**<br>**Additional Information**	**GenBank Acc No (Ref.)**<br>**Specimen ID**<br>**Sequence Length**	**ZFMK Tissue ID**<br>**ZFMK DNA Sample ID**<br>**(SDEI DNA Sample ID)**	**Locality, GPS, Collection Date, Plant, Collector, Identifier**
Lixus pinkeri<br>Voss, 1965	MK890911 [40]<br>766-PST<br>658nt	ZFMK-TIS-3103<br>ZFMK-DNA-0100448121	Spain, Canary Islands, La Gomera, Arure, Ermita Virgen de La Salud, N28°7'59" W17°19'10", 817m, 24-Dez-2011, *Artemisia canariensis (= A. thuscula)*, beating, leg. Stüben,P., det. Stüben,P.
Lixus pinkeri<br>Voss, 1965	MK891289 [40]<br>1245-PST<br>658nt	ZFMK-TIS-3581<br>ZFMK-DNA-0100426039	Spain, Canary Islands, La Gomera, Arure, Ermita Virgen de La Salud, N28°07'59" W17°19'10", 817m, 24-Dez-2011, *Artemisia canariensis (= Artemisia thuscula)*, beating, leg. Stüben,P., det. Stüben,P.
Lixus pinkeri<br>Voss, 1965	MK891293 [40]<br>1256-PST<br>658nt	ZFMK-TIS-3592<br>ZFMK-DNA-0100426279	Spain, Canary Islands, La Gomera, W of Epina, next to cementary, N28°10'01" W17°18'28", 613m, 09-Dez-2012, *Artemisia*, beating, leg. Stüben,P. & Schütte,A., det. Stüben,P.
Lixus pulverulentus<br>(Scopoli, 1763)	KC783755 [40]<br>44-PST<br>635nt	ZFMK-TIS-2D100447006<br>ZFMK-DNA-0100437927	Spain, Canary Islands, Gran Canaria, Valleseco, Balcón de Zamora, N28°03'15" W15°34'05", 925m, 08-Jan-2011, *Carduus clavulatus*, beating, leg. Stüben,P., det. Stüben,P.
Lixus pulverulentus<br>(Scopoli, 1763)	MK891916 [new]<br>2184-JKR<br>658nt	ZFMK-TIS-23922<br>ZFMK-DNA-0169170608	Slovakia, Nove Zamky, Jursky Chlm, N47°48'04" E18°31'27", 113m, 19-Mai-2014, *Carduus* sp., beating, leg. Stejskal,R., det. Stejskal,R.
Lixus pulverulentus<br>(Scopoli, 1763)	MK892254 [new]<br>2553-PSP<br>658nt	ZFMK-TIS-23527<br>ZFMK-DNA-0171662006	Slovakia, Nitra, Muzla, N47°48'00" E18°31'55", 114m, 19-Mai-2014, *Carduus* sp., leg. Sprick,P., det. Sprick,P.
Lixus punctiventris<br>Boheman, 1835	MK891847 [new]<br>2109-JKR<br>658nt	ZFMK-TIS-23749<br>ZFMK-DNA-0169169619	Czech Republic, Moravia, Lanzhot, N48°42'37" E16°58'27", 156m, 10-Mai-2014, sweeping, leg. Krátky,J., det. Krátky,J.
Lixus punctiventris<br>Boheman, 1835	MK892140 [new]<br>2427-JKR<br>658nt	ZFMK-TIS-26004<br>ZFMK-DNA-0171597915	Slovakia, Nitra, Koli?any, Koli?ansky vrch, N48°20'15.5" E18°10'47", 261m, 21-Sep-2014, *Crepis* sp., sweeping, leg. Krátky,J., det. Krátky,J.
Lixus rubicundus<br>Zoubkov, 1833	KC783909 [new]<br>381-JKR<br>658nt	ZFMK-TIS-20167<br>ZFMK-DNA-0100438699	Czech Republic, Bohemia or., Uhretice env., N49°58'39.655" E15°51'28.603", 240m, 05-Jul-2011, *Atriplex* sp., sweeping, leg. Krátky,J., det. Krátky,J.
Lixus rubicundus<br>Zoubkov, 1833	KC784080 [new]<br>543-RST<br>658nt	ZFMK-TIS-20328<br>ZFMK-DNA-0100448436	Czech Republic, Moravia mer., Vrbovec - pond, 2.1 km ESE of the village, N48°47'31.28" E16°7'43.01", 200m, 03-Sep-2011, *Atriplex sagittata*, sweeping, leg. Stejskal,R., det. Stejskal,R.
Lixus rubicundus<br>Zoubkov, 1833	KC784157 [new]<br>607-PSP<br>658nt	ZFMK-TIS-20390<br>ZFMK-DNA-0100448661	Germany, Saxony-Anhalt (ST), Drömling, Piplockenburg, N52°25'20" E11°11'33", 58m, 06-Jul-2011, *Atriplex sagittata*, nitrophile ruderal area, beating, leg. Sprick,P., det. Sprick,P.
Lixus sp.	MK892345 [40]<br>2721-PST<br>658nt	ZFMK-TIS-23871<br>ZFMK-DNA-0171661277	Spain, Canary Islands, Fuerteventura, Betancuria, N28°25'09" W14°03'32", 380m, 04-Jan-2015, *Chenopodiaceae*, beating, leg. Stüben,P., det. Stüben,P.
Lixus sp.	MK347527 [40]<br>2922-PST<br>658nt	(SDEI-DNA-2922-PST)	Spain, Canary Islands, Lanzarote, Órzola, N29°13'29" W13°27'31", 4m, 03-Jan-2017, *Emex*, sifting, leg. Stüben,P., det. Stüben,P.
Lixus subtilis<br>Boheman, 1835	MK891530 [new]<br>1618-JKR<br>658nt	ZFMK-TIS-3666<br>ZFMK-DNA-0155635616	Slovakia, Komarno, B?c env., N47°46'55" E18°25'11", 109m, 18-Mai-2013, sweeping, leg. Krátky,J., det. Krátky,J.
Lixus tibialis<br>Boheman, 1842	KC784225 [new]<br>414-RGO<br>658nt	ZFMK-TIS-20200<br>ZFMK-DNA-0100449432	Poland, Tarnogóra, N50°41'11.6" E23°07'13.4", 249m, 11-Jun-2011, leg. Gosik,R., det. Gosik,R.

**Name**<br>**Authority**<br>**Additional Information**	**GenBank Acc No (Ref.)**<br>**Specimen ID**<br>**Sequence Length**	**ZFMK Tissue ID**<br>**ZFMK DNA Sample ID**<br>**(SDEI DNA Sample ID)**	**Locality, GPS, Collection Date, Plant, Collector, Identifier**
Lixus tibiellus<br>(Desbrochers des Loges, 1904)	MK892359 [40]<br>2749-PST<br>658nt	ZFMK-TIS-23899<br>ZFMK-DNA-0171661257	Spain, Canary Islands, Fuerteventura, S of Salinas, Puerto de la Torre (Bco. de la Torre), N28°21'20" W13°52'42", 1m, 27-Jan-2015, *Salsola orotavensis*, beating, leg. Stüben,P., det. Stüben,P.
Lixus tibiellus<br>(Desbrochers des Loges, 1904)	MK892369 [40]<br>2818-PST<br>658nt	ZFMK-TIS-25864<br>ZFMK-DNA-FC17941492	Spain, Canary Islands, Fuerteventura, S of Salinas, Puerto de la Torre (Bco. de la Torre), N28°21'20" W13°52'42", 1m, 27-Jan-2015, *Salsola orotavensis*, beating, leg. Stüben,P., det. Stüben,P.
Lixus vilis<br>(Rossi, 1790)	MK892101 [new]<br>2378-JKR<br>658nt	ZFMK-TIS-25955<br>ZFMK-DNA-0171600611	Czech Republic, Moravia, Znojmo, N48°51'13" E16°06'34", 250m, 23-Aug-2014, sweeping, leg. R. Stejskal, det. Stejskal,R.
Loborhynchapion amethystinum<br>(Miller, 1857)<br>formerly: *Mesotrichapion*	KC784108 [new]<br>503-RST<br>658nt	ZFMK-TIS-20288<br>ZFMK-DNA-0100448476	Czech Republic, Moravia mer., 11 km SE of Znojmo, Jecmenist? near Dyjakovicky, N48°45'10.63" E16°8'11.16", 280m, 27-Mai-2011, *Astragalus onobrychis*, beating, leg. Stejskal,R., det. Stejskal,R.
Macrobrachonyx gounellei<br>Pic, 1902	MK891480 [40]<br>1550-JKR<br>658nt	ZFMK-TIS-3598<br>ZFMK-DNA-0155635679	Spain, Canary Islands, La Palma, Llano de las Vacas, N28°43'29" W17°47'59", 1340m, 26-Jan-2013, *Pinus canariensis*, beating, leg. Krátky,J., det. Krátky,J.
Macrobrachonyx gounellei<br>Pic, 1902	MK890910 [40]<br>758-PST<br>658nt	ZFMK-TIS-3095<br>ZFMK-DNA-0100448136	Spain, Canary Islands, Tenerife, Orotava valley, above Aguamansa, “Wildpret”, N28°19'11" W16°33'28", 1794m, 04-Feb-2012, *Pinus*, beating, leg. Stüben,P. & Mueller,G., det. Stüben,P.
Madeiracalles achadagrandensis<br>(Stüben, 2002)	FJ716550 [01]<br>P-532-ach<br>658nt	ZFMK-TIS-cP532<br>ZFMK-DNA-0100400287	Portugal, Madeira, 3 km NE of Prazeres, "Achada Grande", N32°47'05" W17°11'06", 1189m, 23-Mrz-2008, *Laurisilva*, leg. Astrin,J. & Stüben,P., det. Astrin,J. and Stüben,P.
Madeiracalles beelzebubi<br>Stüben & Krátky, 2018<br>**Holotype* (DNAtype)**	MF426968 [34]<br>2817-PST<br>658nt	ZFMK-TIS-25863<br>ZFMK-DNA-FC17941484	Portugal, Madeira, 1 km SE of Ribeira da Janela, N32°50'29.5" W17°09'03", 518m, 04-Nov-2014, *Laurus azorica*, beating, leg. Krátky,J., det. Stüben,P.<br>*Note: holotype was used for non-destructive DNA isolation. No paratypes were available for species description.
Madeiracalles cinereus<br>(Wollaston, 1860)	FJ716555 [01]<br>P-516-cin<br>658nt	ZFMK-TIS-cP516<br>ZFMK-DNA-0100400611	Portugal, Madeira, 4 km SW of Santana, Queimadas - Caldeirão Verde, N32°46'49" W16°54'54", 809m, 17-Mrz-2008, *Euphorbia mellifera*, leg. Stüben,P., det. Stüben,P.
Madeiracalles cinereus<br>(Wollaston, 1860)	GU987905 [01]<br>P-526-cin<br>658nt	ZFMK-TIS-cP526<br>ZFMK-DNA-0100400635	Portugal, Madeira, 4.5 km S of São Vicent Boca da Encumeada, "Folhadal", N32°45'08" W17°01'40", 1004m, 22-Mrz-2008, *Laurisilva, Euphorbia mellifera*, leg. Astrin,J. & Stüben,P., det. Astrin,J. and Stüben,P.
Madeiracalles coarctatus<br>(Wollaston, 1857)	MG229820 [40]<br>2856-PST<br>658nt	ZFMK-TIS-4251<br>ZFMK-DNA-FC17941519	Portugal, Madeira, S of Seixal, Chao da Rebeira, N32°48'09" W17°07'04", 493m, 02-Dez-2015, beating, leg. Stüben,P. & Schütte,A., det. Stüben,P.
Madeiracalles dispar<br>(Wollaston, 1854)	FJ716549 [01]<br>P-531-dis<br>658nt	ZFMK-TIS-cP531<br>ZFMK-DNA-0100400271	Portugal, Madeira, 3 km NE of Prazeres, "Achada Grande", N32°47'05" W17°11'06", 1189m, 23-Mrz-2008, *Laurisilva*, leg. Astrin,J. & Stüben,P., det. Astrin,J. and Stüben,P.
Madeiracalles histrionicus<br>(Wollaston, 1857)	MG229805 [40]<br>2782-PST<br>658nt	ZFMK-TIS-26136<br>ZFMK-DNA-0169166955	Portugal, Madeira, Ilhas Desertas, Ilheu Chao (above Furna), N32°35'17" W16°32'40", 38m, 26-Mrz-2015, *Crepis divaricata, Sueda vera, Silene, Sonchus ustulatus*, sieving, leg. Stüben,P., det. Stüben,P.
Madeiracalles histrionicus<br>(Wollaston, 1857)	MG229806 [40]<br>2783-PST<br>658nt	ZFMK-TIS-26137<br>ZFMK-DNA-0169166956	Portugal, Madeira, Ilhas Desertas, Ilheu Chao (above Furna), N32°35'17" W16°32'40", 38m, 26-Mrz-2015, *Crepis divaricata, Sueda vera, Silene, Sonchus ustulatus*, sieving, leg. Stüben,P., det. Stüben,P.

**Name**<br>**Authority**<br>**Additional Information**	**GenBank Acc No (Ref.)**<br>**Specimen ID**<br>**Sequence Length**	**ZFMK Tissue ID**<br>**ZFMK DNA Sample ID**<br>**(SDEI DNA Sample ID)**	**Locality, GPS, Collection Date, Plant, Collector, Identifier**
Madeiracalles machadoi<br>(Stüben, 2006)	FJ716556 [01]<br>P-517-mac<br>658nt	ZFMK-TIS-cP517<br>ZFMK-DNA-0100400612	Portugal, Madeira, 6 km N of Ponta do Sol, Paul da Serra, "Loiral", N32°44'20" W17°06'09", 1295m, 18-Mrz-2008, *Cytisus scoparius*, leg. Astrin,J. & Stüben,P., det. Astrin,J. and Stüben,P.
Madeiracalles machadoi<br>(Stüben, 2006)	MG229815 [40]<br>2821-PST<br>658nt	ZFMK-TIS-25867<br>ZFMK-DNA-FC17941516	Portugal, Madeira, below Pico Ruivo, N32°45'31" W16°56'34", 1702m, 22-Nov-2015, *Ulex europaeus, Cytisus*, sieving, leg. Stüben,P. & Schütte,A., det. Stüben,P.
Madeiracalles portosantoensis<br>Stüben, 2008	MK347543 [40]<br>2953-PST<br>658nt	(SDEI-DNA-2953-PST)	Portugal, Madeira, Canical, 1 km NW (Córrego Curralinho), N32°44'50" W16°45'00", 224m, 28-Mrz-2017, *Quercus*, leg. Kopecky, det. Stüben,P.
Madeiracalles portosantoensis<br>(Stüben, 2002)	FJ716551 [01]<br>P-534-por<br>658nt	ZFMK-TIS-cP534<br>ZFMK-DNA-0100400630	Portugal, Madeira, Porto Santo Isl., 3 km N of Vila Baleira, Pico Castelo, N33°04'51" W16°19'59", 397m, 26-Mrz-2008, *Querus ilex, Quercus suber*, leg. Astrin,J. & Stüben,P., det. Astrin,J. and Stüben,P.
Madeiracalles pulverosus<br>(Gemminger, 1871)	FJ716545 [01]<br>P-512-pul<br>658nt	ZFMK-TIS-cP512<br>ZFMK-DNA-0100400631	Portugal, Madeira, 4 km NW of Santana, São Jorge, N32°49'59" W16°54'00", 35m, 16-Mrz-2008, *Olea*, leg. Stüben,P., det. Stüben,P.
Madeiracalles pulverosus<br>(Gemminger, 1871)	GU987792 [01]<br>P-0116-pul<br>658nt	ZFMK-TIS-cP0116<br>ZFMK-DNA-0100400686	Portugal, Madeira, above Gaula-Camacha, Levada das Tornos, 600m; Tolpis succulenta, N32°40'46" W16°50'60", 600m, 31-Jul-2003, *Tolpis succulenta*, leg. Stüben,P., det. Stüben,P.
Madeiracalles pulverosus<br>(Gemminger, 1871)	GU987903 [01]<br>P-524-pul<br>583nt	ZFMK-TIS-cP524<br>ZFMK-DNA-0100400633	Portugal, Madeira, 1 km S of Curral das Freiras, Eira do Serrado, N32°42'40" W16°57'42", 1040m, 20-Mrz-2008, *Tolpis succulenta*, leg. Astrin,J. & Stüben,P., det. Astrin,J. and Stüben,P.
Madeiracalles pulverosus<br>(Gemminger, 1871)	GU987915 [01]<br>P-511-pul<br>627nt	ZFMK-TIS-cP511<br>ZFMK-DNA-0100400607	Portugal, Madeira, 4 km NW of Santana, São Jorge, N32°49'59" W16°54'00", 35m, 16-Mrz-2008, *Olea*, cliff coast, leg. Stüben,P., det. Stüben,P.
Madeiracalles pulverosus<br>(Gemminger, 1871)	MG229762 [40]<br>1952-PST<br>624nt	ZFMK-TIS-24031<br>ZFMK-DNA-0169170397	Portugal, Madeira, Ribeira da Janela near coast, N32°51'15" W17°09'14", 27m, 05-Okt-2014, *Tolpis succulenta*, beating, leg. Stüben,P., det. Stüben,P.
Madeiracalles pulverosus<br>(Gemminger, 1871)	MG229819 [40]<br>2854-PST<br>658nt	ZFMK-TIS-4249<br>ZFMK-DNA-FC17941503	Portugal, Madeira, Achadas da Cruz, N32°51'23" W17°12'41", 47m, 04-Dez-2015, *Tolpis succulenta*, beating, leg. Stüben,P. & Schütte,A., det. Stüben,P.
Madeiracalles pulverosus<br>(Gemminger, 1871)	MG229888 [40]<br>2943-PST<br>658nt	(SDEI-DNA-2943-PST)	Portugal, Madeira, 1 km S of Ribeira da Janela, N32°50'22" W17°08'59", 530m, 20-Jan-2017, *Laurus azorica*, leg. Krátky,J., det. Stüben,P.
Madeiracalles saxicola<br>(Wollaston, 1854)	MG229803 [40]<br>2777-PST<br>658nt	ZFMK-TIS-26131<br>ZFMK-DNA-0169166965	Portugal, Madeira, Ilhas Desertas, Deserta Grande (south), above Furna, N32°30'39" W16°30'09", 247m, 24-Mrz-2015, *Galactites tomentosa*, collecting by hand, leg. Stüben,P., det. Stüben,P.
Madeiracalles saxicola<br>(Wollaston, 1854)	MG229804 [40]<br>2779-PST<br>658nt	ZFMK-TIS-26133<br>ZFMK-DNA-0169166952	Portugal, Madeira, Ilhas Desertas, Deserta Grande (south), above Focinha, N32°30'18" W16°29'59", 215m, 24-Mrz-2015, *Papaver somniferum* , collecting by hand, leg. Stüben,P., det. Stüben,P.
Madeiracalles saxicola<br>(Wollaston, 1854)	MG229807 [40]<br>2784-PST<br>658nt	ZFMK-TIS-26138<br>ZFMK-DNA-0169166957	Portugal, Madeira, Ilhas Desertas, Deserta Grande (north), above Faia Grande (Baixio), N32°31'14" W16°30'26", 251m, 29-Mrz-2015, *Galactites tomentosa*, collecting by hand, leg. Stüben,P., det. Stüben,P.
Madeiracalles saxicola<br>(Wollaston, 1854)	MG229808 [40]<br>2785-PST<br>658nt	ZFMK-TIS-26139<br>ZFMK-DNA-0169166958	Portugal, Madeira, Ilhas Desertas, Deserta Grande (north), above Quebrada, N32°33'47" W16°32'07", 204m, 30-Mrz-2015, *Calendula maderensis*, collecting by hand, leg. Stüben,P., det. Stüben,P.

**Name**<br>**Authority**<br>**Additional Information**	**GenBank Acc No (Ref.)**<br>**Specimen ID**<br>**Sequence Length**	**ZFMK Tissue ID**<br>**ZFMK DNA Sample ID**<br>**(SDEI DNA Sample ID)**	**Locality, GPS, Collection Date, Plant, Collector, Identifier**
Madeiracalles saxicola<br>(Wollaston, 1854)	GU987916 [01]<br>P-514-sax<br>628nt	ZFMK-TIS-cP514<br>ZFMK-DNA-0100400609	Portugal, Madeira, Machico, Queimado, N32°42'51" W16°46'12", 239m, 17-Mrz-2008, *Euphorbia piscatoria*, leg. Stüben,P., det. Stüben,P.
Madeiracalles saxicola<br>(Wollaston, 1854)	MG229756 [40]<br>1887-PST<br>658nt	ZFMK-TIS-26090<br>ZFMK-DNA-0171606076	Portugal, Madeira, near Machico, Queimado, N32°42'45" W16°45'51", 162m, 16-Jul-2014, *Euphorbia piscatoria*, beating, leg. Stüben,P., det. Stüben,P.
Madeiracalles sp.n.	MG229882 [40]<br>2911-PST<br>658nt	(SDEI-DNA-2911-PST)	Portugal, Madeira, Boaventura, N32°49'33" W16°58'10", 90m, 03-Dez-2016, *Ficus*, sifting, leg. Stüben,P., det. Stüben,P., Note: female specimen
Madeiracalles sp.	MG229748 [40]<br>1838-PST<br>658nt	ZFMK-TIS-26041<br>ZFMK-DNA-0171606123	Portugal, Madeira, S of Sao Vicente, Lameiros, N32°47'32" W17°01'29", 288m, 28-Jun-2014, *Lauraceae*, beating, leg. Stüben,P., det. Stüben,P.
Madeiracalles succulentus<br>Stüben, 2018<br>**Paratype (DNAtype)**	MF426969 [34]<br>2851-PST_4246<br>658nt	ZFMK-TIS-4246<br>ZFMK-DNA-FC17941479	Portugal, Madeira, Achadas da Cruz, N32°51'23" W17°12'41", 47m, 04-Dez-2015, *Tolpis succulenta*, beating, leg. Stüben,P. & Schütte,A., det. Stüben,P.
Madeiracalles succulentus<br>Stüben, 2018<br>**Paratype (DNAtype)**	MF426970 [34]<br>2853-PST_4248<br>658nt	ZFMK-TIS-4248<br>ZFMK-DNA-FC17941495	Portugal, Madeira, Achadas da Cruz, N32°51'23" W17°12'41", 47m, 04-Dez-2015, *Tolpis succulenta*, beating, leg. Stüben,P. & Schütte,A., det. Stüben,P.
Madeiracalles terminalis terminalis<br>(Wollaston, 1854)<br>formerly: *M. terminalis*	MK347544 [40]<br>2954-PST<br>658nt	(SDEI-DNA-2954-PST)	Portugal, Madeira, Santa Maria Madalena, Salao, N32°51'56" W17°11'58", 322m, 29-Mrz-2017, leg. Kopecky, det. Stüben,P.
Madeiracalles terminalis terminalis<br>(Wollaston, 1854)<br>formerly: *M. terminalis*	GU987902 [01]<br>P-519-ter<br>658nt	ZFMK-TIS-cP519<br>ZFMK-DNA-0100400632	Portugal, Madeira, 3 km W of Porto Moniz, Santa Madalena, N32°51'31" W17°12'11", 391m, 19-Mrz-2008, *Ficus carica*, leg. Astrin,J. & Stüben,P., det. Astrin,J. and Stüben,P.
Madeiracalles terminalis terminalis<br>(Wollaston, 1854)<br>formerly: *M. terminalis*	MG229749 [40]<br>1840-PST<br>658nt	ZFMK-TIS-26043<br>ZFMK-DNA-0171606125	Portugal, Madeira, W of Porto Moniz, Santa Madalena, Rib. do Tristao, N32°51'20" W17°12'21", 154m, 29-Jun-2014, *Ficus*, beating, leg. Stüben,P., det. Stüben,P.
Madeiracalles terminalis terminalis<br>(Wollaston, 1854)<br>formerly: *M. terminalis*	MK347541[40]<br>2951-PST<br>658nt	(SDEI-DNA-2951-PST)	Portugal, Madeira, Santa Maria Madalena, Pombais, N32°51'32" W17°12'10", 400m, 29-Mrz-2017, leg. Kopecky, det. Stüben,P.
Madeiracalles terminalis tolpis<br>(Stüben, 2002)<br>formerly: *M. tolpis* - rank change	FJ716553 [01]<br>P-510-ter<br>658nt	ZFMK-TIS-cP510<br>ZFMK-DNA-0100400619	Portugal, Madeira, 4 km NW of Santana, São Jorge, N32°49'59" W16°54'00", 35m, 16-Mrz-2008, *Olea*, cliff coast, leg. Stüben,P., det. Stüben,P.
Madeiracalles terminalis tolpis<br>(Stüben, 2002)<br>formerly: *M. tolpis* - rank change	GU987913 [01]<br>P-508-tol<br>658nt	ZFMK-TIS-cP508<br>ZFMK-DNA-0100400289	Portugal, Madeira, 4 km NW of Santana, São Jorge, N32°49'59" W16°54'00", 35m, 16-Mrz-2008, *Tolpis succulenta*, cliff coast, leg. Stüben,P., det. Stüben,P.

**Name**<br>**Authority**<br>**Additional Information**	**GenBank Acc No (Ref.)**<br>**Specimen ID**<br>**Sequence Length**	**ZFMK Tissue ID**<br>**ZFMK DNA Sample ID**<br>**(SDEI DNA Sample ID)**	**Locality, GPS, Collection Date, Plant, Collector, Identifier**
Madeiracalles terminalis tolpis<br>(Stüben, 2002)<br>formerly: *M. tolpis* - rank change	GU987914 [01]<br>P-509-ter<br>658nt	ZFMK-TIS-cP509<br>ZFMK-DNA-0100400620	Portugal, Madeira, 4 km NW of Santana, São Jorge, N32°49'59" W16°54'00", 35m, 16-Mrz-2008, *Olea*, cliff coast, leg. Stüben,P., det. Stüben,P.
Madeiracalles terminalis tolpis<br>(Stüben, 2002)<br>formerly: *M. tolpis* - rank change	GU987918 [01]<br>P-518-ter<br>658nt	ZFMK-TIS-cP518<br>ZFMK-DNA-0100400613	Portugal, Madeira, 6 km N of Ponta do Sol, Paul da Serra, "Loiral", N32°44'20" W17°06'09", 1295m, 18-Mrz-2008, *Cytisus scoparius*, leg. Astrin,J. & Stüben,P., det. Astrin,J. and Stüben,P.
Madeiracalles terminalis tolpis<br>(Stüben, 2002)<br>formerly: *M. tolpis* - rank change	MG229883 [40]<br>2912-PST<br>658nt	(SDEI-DNA-2912-PST)	Portugal, Madeira, Boaventura, Achada da Madeira, N32°46'53" W16°58'41", 487m, 04-Dez-2016, *Euphorbia millifera*, beating, leg. Stüben,P., det. Stüben,P.
Madeiracalles tristaensis<br>(Stüben, 2002)	MK347538 [40]<br>2948-PST<br>658nt	(SDEI-DNA-2948-PST)	Portugal, Madeira, Santa Maria Madalena, Pombais, N32°51'32" W17°12'10", 400m, 29-Mrz-2017, leg. Kopecky, det. Stüben,P.
Madeiracalles tristaensis<br>(Stüben, 2002)	MK347539 [40]<br>2949-PST<br>658nt	(SDEI-DNA-2949-PST)	Portugal, Madeira, Santa Maria Madalena, Pombais, N32°51'32" W17°12'10", 400m, 29-Mrz-2017, leg. Kopecky, det. Stüben,P.
Madeiracalles tristaensis<br>(Stüben, 2002)	MK347540 [40]<br>2950-PST<br>658nt	(SDEI-DNA-2950-PST)	Portugal, Madeira, Santa Maria Madalena, Pombais, N32°51'32" W17°12'10", 400m, 29-Mrz-2017, leg. Kopecky, det. Stüben,P.
Madeiracalles vau<br>(Wollaston, 1854)	MG229755 [40]<br>1879-PST<br>658nt	ZFMK-TIS-26082<br>ZFMK-DNA-0171606087	Portugal, Madeira, Roque de Faial, Cruzinhas, N32°45'36" W16°52'30.5", 434m, 10-Jul-2014, beating, leg. Stüben,P., det. Stüben,P.
Magdalis armigera<br>(Geoffroy, 1785)	KC783953 [new]<br>315-JKR<br>658nt	ZFMK-TIS-20101<br>ZFMK-DNA-0100438777	Czech Republic, Bohemia or., Dvakacovice, N49°58'35.447" E15°54'2.243", 245m, 14-Mai-2011, *Ulmus campestris*, beating, leg. Krátky,J., det. Krátky,J.
Magdalis armigera<br>(Geoffroy, 1785)	MK891122 [new]<br>1027-PSP<br>658nt	ZFMK-TIS-3172<br>ZFMK-DNA-0100426832	Germany, Lower Saxony (NI), Koldingen, Hannover region, Leine valley, N52°16'25" E09°48'19", 58m, 19-Mai-2012, *Ulmus glabra*, wayside, beating, leg. Sprick,P., det. Sprick,P.
Magdalis cerasi<br>(Linnaeus, 1758)	MK891111 [new]<br>1016-PSP<br>658nt	ZFMK-TIS-3161<br>ZFMK-DNA-0100426846	Germany, Lower Saxony (NI), Berkhof, Hannover area, N52°33'44" E09°40'57", 75m, 10-Mai-2012, *Quercus robur*, mixed broad-leaved shrubs, beating, leg. Sprick,P., det. Sprick,P.
Magdalis flavicornis<br>(Gyllenhal, 1836)	MK892206 [new]<br>2502-PSP<br>658nt	ZFMK-TIS-23474<br>ZFMK-DNA-0171661771	Germany, Lower Saxony (NI), Helstorfer Moor, N52°33'08" E09°35'45.5", 43m, 06-Mai-2014, *Quercus robur*, peatland area, beating, leg. Sprick,P., det. Sprick,P.
Magdalis linearis<br>(Gyllenhal, 1827)	MK891398 [new]<br>1458-PSP<br>658nt	ZFMK-TIS-3218<br>ZFMK-DNA-0155633371	Germany, Lower Saxony (NI), Berkhof, Hannover region, N52°36'48" E09°43'56", 36m, 30-Jun-2012, *Pinus sylvestris*, pine woodland slope, beating, leg. Sprick,P., det. Sprick,P.
Magdalis linearis<br>(Gyllenhal, 1827)	MK891902 [new]<br>2169-JKR<br>658nt	ZFMK-TIS-23905<br>ZFMK-DNA-0169170618	Slovakia, Nove Zamky, Cenkov, NPR Cenkovska step, N47°46'09" E18°31'09", 110m, 19-Mai-2014, *Pinus sylvestris*, beating, leg. Stejskal,R., det. Stejskal,R.
Magdalis memnonia<br>(Gyllenhal, 1837)	MK891157 [new]<br>1064-PSP<br>658nt	ZFMK-TIS-3209<br>ZFMK-DNA-0100426798	Germany, Lower Saxony (NI), near Hannover, Berkhof, N52°36'48" E09°43'56", 36m, 17-Jun-2012, *Pinus sylvestris*, pine forest, beating, leg. Sprick,P., det. Sprick,P.

**Name**<br>**Authority**<br>**Additional Information**	**GenBank Acc No (Ref.)**<br>**Specimen ID**<br>**Sequence Length**	**ZFMK Tissue ID**<br>**ZFMK DNA Sample ID**<br>**(SDEI DNA Sample ID)**	**Locality, GPS, Collection Date, Plant, Collector, Identifier**
Magdalis memnonia<br>(Gyllenhal, 1837)	MK890955 [new]<br>835-PST<br>658nt	ZFMK-TIS-3748<br>ZFMK-DNA-0100414256	Portugal, Minho, S of Viana, Castelo do Neiva, N41°37'07" W08°48'35", 8m, 10-Mai-2012, *Pinus*, beating, leg. Stüben,P., det. Stüben,P.
Magdalis memnonia<br>(Gyllenhal, 1837)	MK892243 [new]<br>2541-PSP<br>658nt	ZFMK-TIS-23513<br>ZFMK-DNA-0171661735	Slovakia, Nitra, Cenkov, N47°46'03" E18°31'09", 109m, 19-Mai-2014, *Pinus sylvestris*, leg. Sprick,P., det. Sprick,P.
Magdalis phlegmatica<br>(Herbst, 1797)	MK891074 [new]<br>977-PSP<br>658nt	ZFMK-TIS-3122<br>ZFMK-DNA-0100426218	Germany, Lower Saxony (NI), near Hannover, Berkhof, N52°36'48" E09°43'56", 36m, 05-Apr-2012, *Pinus sylvestris*, pine forest, beating, leg. Sprick,P., det. Sprick,P.
Magdalis rufa<br>Germar, 1824	MK890875 [new]<br>489-PST<br>658nt	ZFMK-TIS-20274<br>ZFMK-DNA-0100449040	France, Vaucluse, Malaucene, Suzette, Dentelles de Montmirail, N44°10'47" E05°03'16", 542m, 02-Aug-2011, *Pinus*, beating, leg. Stüben,P., det. Stüben,P.
Magdalis rufa<br>Germar, 1824	MK891928 [new]<br>2196-JKR<br>658nt	ZFMK-TIS-23934<br>ZFMK-DNA-0169170591	Slovakia, Nove Zamky, Cenkov, NPR Cenkovska step, N47°46'09" E18°31'09", 110m, 19-Mai-2014, *Pinus sylvestris*, beating, leg. Stejskal,R., det. Stejskal,R.
Magdalis ruficornis<br>(Linnaeus, 1758)	KC783993 [new]<br>259-PSP<br>658nt	ZFMK-TIS-2D100439105<br>ZFMK-DNA-0100439777	Germany, Lower Saxony (NI), Hannover, Stöcken, Leine wetland, N52°24'14" E09°39'15", 45m, 14-Mai-2011, *Crataegus x media*, whithethorn shrub, beating, leg. Sprick,P., det. Sprick,P.
Magdalis ruficornis<br>(Linnaeus, 1758)	MK891645 [new]<br>1802-PST<br>582nt	ZFMK-TIS-24136<br>ZFMK-DNA-0171624062	Germany, Rhineland-Palatinate (RLP), Ahr valley, Dernau, below Krausberg near Steinbergsmühle, N50°31'40" E07°02'43", 204m, 29-Mai-2014, beating, leg. Stüben,P., det. Stüben,P.
Magdalis ruficornis<br>(Linnaeus, 1758)	MK892319 [new]<br>2620-PSP<br>658nt	ZFMK-TIS-23594<br>ZFMK-DNA-0171661939	Germany, Bavaria (BY), Unterfranken, Ebern, N50°05'20.5" E10°45'59", 333m, 13-Jun-2014, *Prunus spinosa*, leg. Sprick,P., det. Sprick,P.
Magdalis violacea<br>(Linnaeus, 1758)	MK892182 [new]<br>2476-PSP<br>658nt	ZFMK-TIS-23448<br>ZFMK-DNA-0171661797	Germany, Lower Saxony (NI), Harz National Park, Torfhaus, Radauer Born, N51°48'03" E10°32'51", 798m, 18-Jul-2013, *Picea abies*, beating, leg. Sprick,P., det. Sprick,P.
Malvaevora timida<br>(Rossi, 1792)	MK892340 [new]<br>2676-PSP<br>658nt	ZFMK-TIS-23826<br>ZFMK-DNA-0171661328	Cyprus, Limassol district, Akrotiri, Episkopi, Asomatos, N34°37'48" E32°56'18", 2m, 09-Apr-2013, *Lavatera cretica*, dipnet, leg. Sprick,P., det. Sprick,P.
Malvaevora timida<br>(Rossi, 1792)	MK891762 [new]<br>2018-JKR<br>658nt	ZFMK-TIS-23658<br>ZFMK-DNA-0169170488	Italy, Sicilia Isl., Ragusa, Modica env., N36°51'03" E14°45'03", 319m, 18-Apr-2014, *Malvaceae*, sweeping, leg. Krátky,J., det. Krátky,J.
Malvaevora timida<br>(Rossi, 1792)	KC783844 [new]<br>181-PST<br>655nt	ZFMK-TIS-2D100440246<br>ZFMK-DNA-0100438134	Morocco, Atlas Mts., S of Ifrane, S of Timahdite, N33°09'35" W05°03'59", 1918m, 19-Mai-2011, beating, leg. Stüben,P., det. Behne,L.
Malvaevora timida<br>(Rossi, 1792)	MK890963 [new]<br>846-PST<br>658nt	ZFMK-TIS-3759<br>ZFMK-DNA-0100414244	Portugal, Estremadura, W of Sintra, Serra de Sintra, S of Colares, N38°46'38" W09°28'37", 241m, 16-Mai-2012, *Malvae*, beating, leg. Stüben,P., det. Stüben,P.
Malvaevora timida<br>(Rossi, 1792)	MK891319 [new]<br>1317-PST<br>658nt	ZFMK-TIS-4133<br>ZFMK-DNA-0100426159	Spain, Malaga, S of Mollina, N37°04'45" W04°40'08", 437m, 07-Mai-2013, *Malveae*, beating, leg. Stüben,P. & Schütte,A., det. Stüben,P.
Malvapion malvae<br>(Fabricius, 1775)	KC784173 [new]<br>479-PST<br>658nt	ZFMK-TIS-20264<br>ZFMK-DNA-0100449066	Germany, Rhineland-Palatinate (RLP), Mosellus area, Kobern-Gondorf, "Sürzer Höfe", ruderal area, N50°20'20" E07°25'25", 330m, 30-Sep-2011, *Malva*, hand-collecting, leg. Stüben,P., det. Stüben,P.

**Name**<br>**Authority**<br>**Additional Information**	**GenBank Acc No (Ref.)**<br>**Specimen ID**<br>**Sequence Length**	**ZFMK Tissue ID**<br>**ZFMK DNA Sample ID**<br>**(SDEI DNA Sample ID)**	**Locality, GPS, Collection Date, Plant, Collector, Identifier**
Malvapion malvae<br>(Fabricius, 1775)	MK890916 [new]<br>784-PST<br>658nt	ZFMK-TIS-3697<br>ZFMK-DNA-0100414307	Germany, Rhineland-Palatinate (RLP), E of Treis-Karden near Pommern, Fellerbachtal (river valley), N50°10'10" E07°13'38", 85m, 09-Jun-2012, *Malva*, rivulet, beating, leg. Stüben,P., det. Stüben,P.
Malvapion malvae<br>(Fabricius, 1775)	MK891136 [new]<br>1041-PSP<br>658nt	ZFMK-TIS-3186<br>ZFMK-DNA-0100426823	Germany, Lower Saxony (NI), Königsförde, Lkr. Hameln-Pyrmont, N52°03'58" E09°16'17", 94m, 27-Mai-2012, *Malva sylvestris*, house garden, beating, leg. Sprick,P., det. Sprick,P.
Malvapion malvae<br>(Fabricius, 1775)	MK891757 [new]<br>2012-JKR<br>658nt	ZFMK-TIS-23652<br>ZFMK-DNA-0169170482	Italy, Sicilia Isl. (TP), Trapani, Marinella, N37°34'59" E12°51'40", 3m, 17-Apr-2014, *Malvaceae*, sweeping, leg. Krátky,J., det. Krátky,J.
Malvapion malvae<br>(Fabricius, 1775)	MK891053 [new]<br>953-CBR<br>658nt	ZFMK-TIS-3866<br>ZFMK-DNA-0100449658	Luxembourg, Luxembourg, N49°38'00" E06°10'45", 345m, 03-Jul-2012, *Malva moschata*, beating, leg. Braunert,C., det. Braunert,C.
Malvapion malvae<br>(Fabricius, 1775)	MK891662 [40]<br>1857-PST<br>658nt	ZFMK-TIS-26060<br>ZFMK-DNA-0171606109	Portugal, Madeira, Faja da Ovelha, N32°46'24" W17°13'48", 550m, 02-Jul-2014, *Malva*, beating, leg. Stüben,P., det. Stüben,P.
Malvapion malvae<br>(Fabricius, 1775)	KC783893 [new]<br>160-PST<br>658nt	ZFMK-TIS-2D100440977<br>ZFMK-DNA-0100438365	Morocco, S of Berkane, Mts. des Beni Snassen, Vallee Zegzel, N34°49'19" W02°23'10" , 644m, 15-Mai-2011, *Malvaceae*, beating, leg. Stüben,P., det. Stüben,P./vid. Behne,L.
Marmaropus besseri<br>Gyllenhal, 1837	KC784055 [new]<br>586-PSP<br>658nt	ZFMK-TIS-20370<br>ZFMK-DNA-0100448400	Germany, Lower Saxony (NI), Braunschweig, Hafen, N52°18'53" E10°29'36", 66m, 07-Jun-2011, *Rumex thyrsiflorus*, nutrient-poor grassland, dipnet, leg. Sprick,P., det. Sprick,P.
Mecaspis alternans<br>(Herbst, 1795)	MK891907 [new]<br>2174-JKR<br>658nt	ZFMK-TIS-23912<br>ZFMK-DNA-0169170613	Slovakia, Nove Zamky, Bajtava, Ploska hora, N47°51'54" E18°45'15", 126m, 22-Mai-2014, *Daucus carota*, collecting by hand, leg. Stejskal,R., det. Stejskal,R.
Mecinus barbarus<br>Gyllenhal, 1838	MK891179 [new]<br>1093-JKR<br>658nt	ZFMK-TIS-3429<br>ZFMK-DNA-0100449139	Spain, Andalucia, Mazagón, N37°07'13" W06°47'16", 40m, 18-Apr-2012, *Linaria oblongifolia sub*sp. *haenseleri Valdes*, sweeping, leg. Krátky,J., det. Krátky,J.
Mecinus circulatus<br>(Marsham, 1802)	KC783778 [40]<br>69-PST<br>658nt	ZFMK-TIS-2D100446982<br>ZFMK-DNA-0100438054	Spain, Canary Islands, La Gomera, E of Hermigua, PN of Majona, El Palmar, N28°9'44" W17°10'00", 321m, 06-Feb-2011, *Plantago*, beating, leg. Stüben,P., det. Stüben,P.
Mecinus collaris<br>Germar, 1821	MK891151 [new]<br>1058-PSP<br>658nt	ZFMK-TIS-3203<br>ZFMK-DNA-0100426792	Germany, Lower Saxony (NI), Langeoog, N53°45'00" E07°33'04", 1m, 16-Jun-2012, *Plantago maritima*, salt marsh, soil-search, leg. Sprick,P., det. Sprick,P.
Mecinus heydeni<br>Wencker, 1866	MK891542 [new]<br>1631-JKR<br>658nt	ZFMK-TIS-3679<br>ZFMK-DNA-0155635598	Slovakia, Komarno, Palarikovo, Neded, Jahodnianske jazierko NR, N48°01'33" E17°59'49", 110m, 20-Mai-2013, *Linaria vulgaris*, sweeping, leg. Krátky,J., det. Krátky,J.
Mecinus ictericus<br>(Gyllenhal, 1838)	MK891697 [new]<br>1915-FBA<br>658nt	ZFMK-TIS-23306<br>ZFMK-DNA-0169170363	Greece, Central Macedonia, Serres, Achladohori, N41°19'00" E23°34'20", 670m, 12-Aug-2014, leg. Bayer & Brunner, det. Bayer,F.
Mecinus ictericus<br>(Gyllenhal, 1838)	MK891958 [new]<br>2227-JKR<br>658nt	ZFMK-TIS-23965<br>ZFMK-DNA-0169170555	Slovakia, Komarno, Virt, N47°45'39" E18°20'13.5", 123m, 16-Jun-2014, *Plantago arenaria*, leg. Benedikt,S., det. Benedikt,S.
Mecinus ictericus<br>(Gyllenhal, 1838)	MK892154 [new]<br>2442-JKR<br>658nt	ZFMK-TIS-26019<br>ZFMK-DNA-0171600664	Slovakia, Nove Zamky, Imel, PR Liscie diery, N47°55'17" E18°09'37", 114m, 20-Sep-2014, *Plantago arenaria*, sweeping, leg. Krátky,J., det. Krátky,J.

**Name**<br>**Authority**<br>**Additional Information**	**GenBank Acc No (Ref.)**<br>**Specimen ID**<br>**Sequence Length**	**ZFMK Tissue ID**<br>**ZFMK DNA Sample ID**<br>**(SDEI DNA Sample ID)**	**Locality, GPS, Collection Date, Plant, Collector, Identifier**
Mecinus janthinus<br>Germar, 1821	KC783938 [new]<br>339-JKR<br>658nt	ZFMK-TIS-20125<br>ZFMK-DNA-0100438753	Slovakia, Komarno, Iza env., PR Bokrosske slanisko , N47°44'50.372" E18°15'34.563", 107m, 20-Mai-2011, *Linaria vulgaris*, sweeping, leg. Krátky,J., det. Krátky,J.
Mecinus janthinus<br>Germar, 1821	MK891536 [new]<br>1625-JKR<br>658nt	ZFMK-TIS-3673<br>ZFMK-DNA-0155635604	Slovakia, Nove Zamky, Kamenica nad Hronom, N47°49'34" E18°44'31", 109m, 19-Mai-2013, *Linaria genistifolia*, individual collecting by hand, leg. Krátky,J., det. Krátky,J.
Mecinus labilis<br>(Herbst, 1795)	MK892077 [new]<br>2353-JKR<br>658nt	ZFMK-TIS-24284<br>ZFMK-DNA-0169170623	Czech Republic, Moravia, Lanzhot, Pohansko, N48°41'27" E16°55'58", 153m, 14-Aug-2014, sweeping, leg. Krátky,J., det. Krátky,J.
Mecinus laeviceps<br>Tournier, 1873	MK891547 [new]<br>1637-JKR<br>658nt	ZFMK-TIS-3685<br>ZFMK-DNA-0155635587	Czech Republic, Moravia, Oblekovice, Naceraticky kopec, N48°50'09" E16°05'45", 256m, 08-Jun-2013, *Linaria genistifolia*, individual collecting by hand, leg. Krátky,J., det. Krátky,J.
Mecinus pascuorum<br>(Gyllenhal, 1813)	KC784104 [new]<br>511-RST<br>658nt	ZFMK-TIS-20296<br>ZFMK-DNA-0100448469	Czech Republic, Moravia mer., NP Podyji, 2 km NW of Hnanice, N48°48'38.76" E15°58'11.42", 350m, 29-Jun-2011, sweeping, leg. Stejskal,R., det. Stejskal,R.
Mecinus pascuorum<br>(Gyllenhal, 1813)	MK890930 [new]<br>799-PST<br>658nt	ZFMK-TIS-3712<br>ZFMK-DNA-0100414293	Germany, Rhineland-Palatinate (RLP), E of Treis-Karden, Pommern, Pommerner Goldberg, high road (vineyard), N50°10'14" E07°16'29", 95m, 10-Jun-2012, *Plantago lanceolata*, beating, leg. Stüben,P., det. Stüben,P.
Mecinus pascuorum<br>(Gyllenhal, 1813)	MK891420 [new]<br>1483-PSP<br>658nt	ZFMK-TIS-3243<br>ZFMK-DNA-0155633346	Germany, Lower Saxony (NI), Hameln-Rohrsen, Dütberg, N52°06'27" E09°25'02", 116m, 15-Jul-2012, *Plantago lanceolata*, dipnet, leg. Sprick,P., det. Sprick,P.
Mecinus pascuorum<br>(Gyllenhal, 1813)	MK892178 [new]<br>2470-PSP<br>658nt	ZFMK-TIS-23442<br>ZFMK-DNA-0171661808	Germany, Lower Saxony (NI), Hameln-Rohrsen, Dütberg, N52°06'26" E09°24'52", 116m, 07-Jun-2013, *Plantago lanceolata*, dipnet, leg. Sprick,P., det. Sprick,P.
Mecinus pascuorum<br>(Gyllenhal, 1813)	MK892315 [new]<br>2616-PSP<br>658nt	ZFMK-TIS-23590<br>ZFMK-DNA-0171661943	Germany, Bavaria (BY), Unterfranken, Ebern, N50°05'07" E10°45'52.5", 318m, 14-Jun-2014, *Plantago lanceolata*, leg. Sprick,P., det. Sprick,P.
Mecinus pascuorum<br>(Gyllenhal, 1813)	MK891067 [new]<br>968-CBR<br>658nt	ZFMK-TIS-3881<br>ZFMK-DNA-0100449646	Luxembourg, 10 km NW of Esch/Alzette, 1 km NW of Niedercorn, N49°32'31" E05°52'39", 370m, 28-Jun-2012, *Plantago lanceolata*, sweeping, leg. Braunert,C., det. Braunert,C.
Mecinus pirazzolii<br>(Stierlin, 1867)	MK891704 [new]<br>1922-FBA<br>634nt	ZFMK-TIS-23313<br>ZFMK-DNA-0169170370	Greece, East Macedonia and Thrace, Drama, Lefkoghia, N41°23'59" E23°54'53", 620m, 13-Aug-2014, leg. Bayer & Brunner, det. Bayer,F.
Mecinus pirazzolii<br>(Stierlin, 1867)	MK891954 [new]<br>2223-JKR<br>658nt	ZFMK-TIS-23961<br>ZFMK-DNA-0169170564	Slovakia, Komarno, Balvany, N47°50'28" E18°00'19", 110m, 15-Jun-2015, *Plantago arenaria*, leg. Benedikt,S., det. Benedikt,S.
Mecinus plantaginis<br>(Eppelsheim, 1875)	MK892318 [new]<br>2619-PSP<br>658nt	ZFMK-TIS-23593<br>ZFMK-DNA-0171661940	Germany, Bavaria (BY), Unterfranken, Ebern, N50°05'02.5" E10°45'52", 313m, 14-Jun-2014, *Plantago media*, leg. Sprick,P., det. Sprick,P.
Mecinus pyraster<br>(Herbst, 1795)	MK892090 [new]<br>2366-JKR<br>658nt	ZFMK-TIS-25926<br>ZFMK-DNA-0171600588	Czech Republic, Moravia, NP Podyji, Podmyce, Jejkal Pond, N48°51'24" E15°54'15.5", 420m, 17-Aug-2014, *Plantago*, sweeping, leg. R. Stejskal, det. Stejskal,R.
Mecinus pyraster<br>(Herbst, 1795)	MK891125 [new]<br>1030-PSP<br>658nt	ZFMK-TIS-3175<br>ZFMK-DNA-0100426829	Germany, Lower Saxony (NI), Hannover, Stöcken, N52°24'24" E09°39'13", 42m, 22-Mai-2012, *Plantago lanceolata*, species-rich hay meadow, dipnet, leg. Sprick,P., det. Sprick,P.

**Name**<br>**Authority**<br>**Additional Information**	**GenBank Acc No (Ref.)**<br>**Specimen ID**<br>**Sequence Length**	**ZFMK Tissue ID**<br>**ZFMK DNA Sample ID**<br>**(SDEI DNA Sample ID)**	**Locality, GPS, Collection Date, Plant, Collector, Identifier**
Mecinus pyraster<br>(Herbst, 1795)	MK891013 [new]<br>909-CBR<br>658nt	ZFMK-TIS-3822<br>ZFMK-DNA-0100449702	Luxembourg, 10 km E of Luxembourg, 2 km S of Mensdorf, N49°38'09" E06°17'48", 330m, 08-Mai-2011, *Plantago lanceolata*, sweeping, leg. Braunert,C., det. Braunert,C.
Mecinus tychioides<br>(H. Brisout de Barneville, 1862)	MK892350 [40]<br>2734-PST<br>658nt	ZFMK-TIS-23884<br>ZFMK-DNA-0171661265	Spain, Canary Islands, Fuerteventura, NE of Betancuria, Mirador de Morro Velosa, N28°25'50" W14°03'27", 446m, 07-Jan-2015, sieving, leg. Stüben,P., det. Stüben,P.
Mecinus variabilis<br>(Rosenhauer, 1856)	MK891164 [new]<br>1073-JKR<br>658nt	ZFMK-TIS-3409<br>ZFMK-DNA-0100449635	Spain, Andalucia, Malaga, Guadalmar, N36°39'39" W04°27'47", 2m, 14-Apr-2012, *Plantago* sp., individual collecting by hand, leg. Krátky,J., det. Krátky,J.
Melanapion minimum<br>(Herbst, 1797)	MK891155 [new]<br>1062-PSP<br>658nt	ZFMK-TIS-3207<br>ZFMK-DNA-0100426910	Germany, Lower Saxony (Nl), Langeoog, N53°45'05" E07°32'24", 3m, 16-Jun-2012, *Salix repens*, small dune valley, beating, leg. Sprick,P., det. Sprick,P.
Melanobaris atramentaria<br>(Boheman, 1836)	MK892159 [new]<br>2449-JKR<br>658nt	ZFMK-TIS-24068<br>ZFMK-DNA-0169170622	Czech Republic, Moravia, Znojmo-centre, N48°50'59" E16°03'17", 255m, 11-Apr-2014, *Sisymbrium* sp., collecting by hand, leg. R. Stejskal, det. Stejskal,R.
Melanobaris atramentaria<br>(Boheman, 1836)	KC783933 [new]<br>334-JKR<br>658nt	ZFMK-TIS-20120<br>ZFMK-DNA-0100438748	Slovakia, Komarno, Virt env., N47°45'35.247" E18°20'25.833", 112m, 19-Mai-2011, *Erysimum* sp., sweeping, leg. Krátky,J., det. Krátky,J.
Melanobaris atramentaria<br>(Boheman, 1836)	MK892225 [new]<br>2523-PSP<br>658nt	ZFMK-TIS-23495<br>ZFMK-DNA-0171661750	Slovakia, Nitra, SW of Buc, N47°46'56" E18°25'10", 110m, 18-Mai-2014, *Syrenia cana*, leg. Sprick,P., det. Sprick,P.
Melanobaris laticollis<br>(Marsham, 1802)	MK892385 [40]<br>2846-PST<br>658nt	ZFMK-TIS-4241<br>ZFMK-DNA-FC17941534	Portugal, Madeira, Sao Vincente, Rota da Cal, N32°47'52" W17°01'46", 322m, 30-Nov-2015, *ruderal vegetation*, ruderal vegetation, beating, leg. Stüben,P. & Schütte,A., det. Stüben,P.
Melanobaris laticollis<br>(Marsham, 1802)	KC784228 [new]<br>403-RGO<br>658nt	ZFMK-TIS-20189<br>ZFMK-DNA-0100449436	Poland, ad. Janowiec, N51°19'46" E21°51'53", 161m, 07-Mai-2011, leg. Gosik,R., det. Gosik,R.
Mesagroicus obscurus<br>Boheman, 1840	MK891537 [new]<br>1626-JKR<br>658nt	ZFMK-TIS-3674<br>ZFMK-DNA-0155635603	Slovakia, Nove Zamky, Bajtava, Ploska hora, N47°49'34" E18°45'06", 148m, 19-Mai-2013, sieving, leg. Krátky,J., det. Krátky,J.
Mesites ater<br>Lindberg, 1950	KC783747 [40]<br>34-PST<br>623nt	ZFMK-TIS-2D100447013<br>ZFMK-DNA-0100438085	Spain, Canary Islands, Gran Canaria, NE of Santa Brigida, Angostura valley, N28°03'14" W15°28'49", 485m, 31-Dez-2010, *Euphorbia obtusifolia*, beating, leg. Stüben,P., det. Stüben,P.
Mesites cf. *fusiformis*	MH644790 [40]<br>2921-PST<br>658nt	(SDEI-DNA-2921-PST)	Spain, Canary Islands, Lanzarote, Órzola, N29°13'29" W13°27'31", 4m, 03-Jan-2017, *Euphorbia regis-jubae*, sifting, leg. Stüben,P., det. Stüben,P.
Mesites fusiformis fusiformis<br>Wollaston, 1861	KC783773 [40]<br>64-PST<br>658nt	ZFMK-TIS-2D100446977<br>ZFMK-DNA-0100438049	Spain, Canary Islands, La Gomera, E of Hermigua, El Palmar, NP Mojana, N28°09'26“ W17°09'47“, 627m, 14-Dez-2010, *Euphorbia*, collecting by hand, leg. Stüben,P., det. Stüben,P.
Mesites fusiformis fusiformis<br>Wollaston, 1861	MH051934 [33]<br>770-PST<br>658nt	ZFMK-TIS-3107<br>ZFMK-DNA-0100417605	Spain, Canary Islands, La Gomera, Meriga, N28°9'16" W17°14'12", 825m, 07-Mrz-2012, *Euphorbia mellifera*, garden, beating, leg. Stüben,P., det. Stüben,P.
Mesites fusiformis fusiformis<br>Wollaston, 1861	MH618675 [40]<br>771-PST<br>658nt	ZFMK-TIS-3108<br>ZFMK-DNA-0100417606	Spain, Canary Islands, La Gomera, Meriga, N28°9'16" W17°14'12", 825m, 07-Mrz-2012, *Euphorbia mellifera*, garden, beating, leg. Stüben,P., det. Stüben,P.

**Name**<br>**Authority**<br>**Additional Information**	**GenBank Acc No (Ref.)**<br>**Specimen ID**<br>**Sequence Length**	**ZFMK Tissue ID**<br>**ZFMK DNA Sample ID**<br>**(SDEI DNA Sample ID)**	**Locality, GPS, Collection Date, Plant, Collector, Identifier**
Mesites fusiformis fusiformis<br>Wollaston, 1861	MH618676 [40]<br>1670-PST<br>658nt	ZFMK-TIS-24388<br>ZFMK-DNA-0155622520	Spain, Canary Islands, La Gomera, above Agulo, (El Chorro), N28°11'02" W17°11'58", 505m, 06-Dez-2013, beating, leg. Stüben,P., det. Stüben,P.
Mesites pallidipennis<br>Boheman, 1838	MH618684 [new]<br>3099-PST<br>658nt	ZFMK-DNA-FD02298683	Ukraine, Kyliia Distr., Vylkove env., Kubanu Isl. SE coast, N45°17'02.5" E29°44'43.5", 1m, 30-Aug-2010, *Populus* sp. *(det.unc.)*, beating, leg. Nazarenko, det. Stüben,P.
Mesites pubipennis<br>Wollaston, 1861	MH051953 [33]<br>2768-PST<br>658nt	ZFMK-TIS-26122<br>ZFMK-DNA-0169166974	Spain, Canary Islands, La Palma, S of Los Sauces, San Juan, N28°47'15" W17°45'58", 218m, 24-Jan-2014, *Euphorbia*, sieving, leg. Stüben,P., det. Stüben,P.
Mesites pubipennis<br>Wollaston, 1861	MH618680 [40]<br>2767-PST<br>658nt	ZFMK-TIS-26121<br>ZFMK-DNA-0169164818	Spain, Canary Islands, La Palma, N of Juan Adalid, N28°50'37" W17°54'07", 296m, 14-Jan-2014, *Euphorbia canariensis*, beating, leg. Stüben,P., det. Stüben,P.
Mesites pubipennis<br>Wollaston, 1861	MH618681 [40]<br>2770-PST<br>658nt	ZFMK-TIS-26124<br>ZFMK-DNA-0169166972	Spain, Canary Islands, La Palma, W of Garafia near Puerto Garafia, N28°49'27" W17°57'40", 204m, 17-Jan-2014, *Euphorbia balsamifera, Euphorbia canariensis*, sieving, leg. Stüben,P., det. Stüben,P.
Mesites pubipennis<br>Wollaston, 1861	MH644789 [40]<br>2769-PST<br>658nt	ZFMK-TIS-26123<br>ZFMK-DNA-0169166973	Spain, Canary Islands, La Palma, N of Los Llanos, Barranco de las Angustias (Caldera de Taburiente), N28°40'54" W17°54'46", 272m, 23-Jan-2014, *Euphorbia regis-jubae*, beating, leg. Stüben,P., det. Stüben,P.
Mesites sp.	MH618685 [40]<br>2971-PST<br>658nt	(SDEI-DNA-2971-PST)	Spain, Canary Islands, El Hierro, Tancojote Mt., N27°49'28" W17°57'50", 333m, 21-Apr-2016, *Euphoribia regis-jubae*, leg. Stüben & Schütte, det. Stüben,P.
Mesoxyonyx sicardi<br>(Hustache, 1931)	MK891355 [new]<br>1404-JKR<br>658nt	ZFMK-TIS-3693<br>ZFMK-DNA-0155628538	France, Herault, Carnon, Le Petit Travers, N43°33'12" E04°00'33", 2m, 11-Jun-2013, *Ephedra dystachia*, sweeping, leg. Krátky,J., det. Krátky,J.
Metacinops rhinomacer<br>Kraatz, 1862	MK890903 [new]<br>667-FBA<br>658nt	ZFMK-TIS-20448<br>ZFMK-DNA-0100448600	Greece, Peloponnese, Messinia, Mt. Taygetos, S of Marathos, N36°53'46" E22°19'09", 1300m, 21-Mai-2011, leg. Bahr,F., det. Bahr,F.
Miarus ajugae<br>(Herbst, 1795)	MK892004 [new]<br>2276-JKR<br>658nt	ZFMK-TIS-24207<br>ZFMK-DNA-0169170990	Austria, Styria, Hochschwab, Spitzkögel, N47°35'27" E15°01'26", 1659m, 27-Jun-2014, *Phyteuma* cf. *orbiculare*, sweeping, leg. Krátky,J., det. Krátky,J.
Miarus ajugae<br>(Herbst, 1795)	MK892297 [new]<br>2598-PSP<br>658nt	ZFMK-TIS-23572<br>ZFMK-DNA-0171661961	Germany, Bavaria (BY), Unterfranken, Ebern, N50°05'03" E10°45'15", 331m, 14-Jun-2014, *Campanula rapunculus*, leg. Sprick,P., det. Sprick,P.
Miarus ajugae<br>(Herbst, 1795)	MK892333 [new]<br>2635-PSP<br>658nt	ZFMK-TIS-23609<br>ZFMK-DNA-0171661927	Germany, Saxony-Anhalt (ST), Schierke, Drei Annen Hohne, Hohnekopf, N51°46'34.5" E10°41'58", 829m, 10-Jul-2014, *Campanula rotundifolia?*, dipnet, leg. Sprick,P., det. Sprick,P.
Miarus ajugae<br>(Herbst, 1795)	KC784232 [new]<br>396-RGO<br>658nt	ZFMK-TIS-20182<br>ZFMK-DNA-0100449443	Poland, Katy 2, N50°40'37" E23°07'43", 270m, 30-Jun-2011, leg. Gosik,R., det. Gosik,R.
Miarus ajugae<br>(Herbst, 1795)	MK892040 [new]<br>2316-JKR<br>658nt	ZFMK-TIS-24247<br>ZFMK-DNA-0169170949	Slovakia, Tatry Mts., Dolina Siedmich prame?ov, N49°12'57" E20°17'46", 981m, 05-Jul-2014, *Campanula*, sweeping, leg. Krátky,J., det. Krátky,J.
Micrelus ericae<br>(Gyllenhal, 1813)	MK891399 [new]<br>1459-PSP<br>658nt	ZFMK-TIS-3219<br>ZFMK-DNA-0155633370	Germany, Lower Saxony (NI), Berkhof, Hannover region, N52°36'58" E09°43'49", 39m, 30-Jun-2012, *Calluna vulgaris*, pine forest track, dipnet, leg. Sprick,P., det. Sprick,P.

**Name**<br>**Authority**<br>**Additional Information**	**GenBank Acc No (Ref.)**<br>**Specimen ID**<br>**Sequence Length**	**ZFMK Tissue ID**<br>**ZFMK DNA Sample ID**<br>**(SDEI DNA Sample ID)**	**Locality, GPS, Collection Date, Plant, Collector, Identifier**
Micrelus ericae<br>(Gyllenhal, 1813)	MK891070 [new]<br>971-CBR<br>658nt	ZFMK-TIS-3884<br>ZFMK-DNA-0100449649	Luxembourg, 15 km NW of Luxembourg, 3 km NE of Nospelt, N49°41'25" E06°01'21", 350m, 23-Jun-2012, *Calluna vulgaris*, sweeping, leg. Braunert,C., det. Braunert,C.
Micrelus ericae<br>(Gyllenhal, 1813)	MK890954 [new]<br>834-PST<br>658nt	ZFMK-TIS-3747<br>ZFMK-DNA-0100414257	Portugal, Minho, PN of Peneda-Geres near Soajo, N41°52'14" W08°16'30", 200m, 09-Mai-2012, beating, leg. Stüben,P., det. Stüben,P.
Micrelus ferrugatus<br>(Perris, 1847)	KM433741 [13]<br>752-PST<br>658nt	ZFMK-TIS-3089<br>ZFMK-DNA-0100448130	Spain, Canary Islands, Tenerife, Anaga Mts., Chinobre, N28°33'23" W16°10'48", 798m, 13-Feb-2012, *Erica arborea (eng. "tree heath", ger. "Baumheide")*, beating, leg. Stüben,P. & Schütte,A., det. Stüben,P.
Microplontus campestris<br>(Gyllenhal, 1837)	KC783970 [new]<br>322-JKR<br>658nt	ZFMK-TIS-20108<br>ZFMK-DNA-0100438947	Czech Republic, Bohemia or., Hradec Kralove - Fararstvi, N50°11'22.247" E15°48'53.64", 230m, 17-Mai-2011, *Leucanthemum vulgare*, sweeping, leg. Krátky,J., det. Krátky,J.
Microplontus campestris<br>(Gyllenhal, 1837)	KC783985 [new]<br>276-PSP<br>645nt	ZFMK-TIS-2D100439095<br>ZFMK-DNA-0100439767	Germany, Lower Saxony (NI), Pattensen, Hannover region, N52°14'35" E09°45'21", 77m, 17-Mai-2011, *Leucanthemum vulgare*, roadside, beating, leg. Sprick,P., det. Sprick,P.
Microplontus melanostigma<br>(Marsham, 1802)	KC783916 [new]<br>371-JKR<br>658nt	ZFMK-TIS-20157<br>ZFMK-DNA-0100438712	Czech Republic, Bohemia or., Hrobice, N50°6'2.909" E15°46'50.867", 230m, 29-Jun-2011, *Matricaria recutita*, sweeping, leg. Krátky,J., det. Krátky,J.
Microplontus melanostigma<br>(Marsham, 1802)	MK891551 [new]<br>1641-JKR<br>658nt	ZFMK-TIS-3689<br>ZFMK-DNA-0155635591	Czech Republic, Moravia, Rudlice, N48°58'20" E16°06'45", 256m, 08-Jun-2013, sweeping, leg. Krátky,J., det. Krátky,J.
Microplontus melanostigma<br>(Marsham, 1802)	MK891858 [new]<br>2120-JKR<br>658nt	ZFMK-TIS-23760<br>ZFMK-DNA-0169169613	Czech Republic, Bohemia, Hradec Kralove, Borovinka, N50°11'24" E15°47'60", 228m, 15-Mai-2014, *Matricaia inodora*, sweeping, leg. Krátky,J., det. Krátky,J.
Microplontus melanostigma<br>(Marsham, 1802)	MK891114 [new]<br>1019-PSP<br>658nt	ZFMK-TIS-3164<br>ZFMK-DNA-0100426246	Germany, Lower Saxony (NI), Hannover, Nordhafen, N52°25'27" E09°39'05", 53m, 12-Mai-2012, *Tripleurospermum perforatum*, south exposed slope with annual ruderal area, dipnet, leg. Sprick,P., det. Sprick,P.
Microplontus melanostigma<br>(Marsham, 1802)	MK892186 [new]<br>2481-PSP<br>658nt	ZFMK-TIS-23453<br>ZFMK-DNA-0171661792	Germany, Thuringia (TH), Ranis, Ludwigshof, N50°40'03" E11°35'04", 388m, 24-Jun-2013, *Matricaria recutita*, rearing of larvae, leg. M. Hommes, det. Sprick,P.
Microplontus melanostigma<br>(Marsham, 1802)	MK891258 [new]<br>1192-JKR<br>658nt	ZFMK-TIS-3528<br>ZFMK-DNA-0100426235	Spain, Andalucia, Sierra de la Sagra, Cortijos Nuevos de la Sierra, N37°58'27" W02°32'37", 1341m, 15-Jun-2012, *Matricaria*, beating, leg. Krátky,J., det. Krátky,J.
Microplontus millefolii<br>(Schultze, 1897)	KC783922 [new]<br>361-JKR<br>658nt	ZFMK-TIS-20147<br>ZFMK-DNA-0100438727	Czech Republic, Bohemia or., Hradec Kralove, PP Na Plachte, N50°11'35.975" E15°51'34.234", 230m, 25-Jun-2011, *Tanacetum vulgare*, beating, leg. Krátky,J., det. Krátky,J.
Microplontus millefolii<br>(Schultze, 1897)	MK890929 [new]<br>798-PST<br>658nt	ZFMK-TIS-3711<br>ZFMK-DNA-0100414292	Germany, Rhineland-Palatinate (RLP), E of Treis-Karden, Pommern, Pommerner Goldberg, high road (vineyard), N50°10'14" E07°16'29", 95m, 10-Jun-2012, *Tanacetum vulgare*, beating, leg. Stüben,P., det. Stüben,P.
Microplontus rugulosus<br>(Herbst, 1795)	KC783921 [new]<br>360-JKR<br>658nt	ZFMK-TIS-20146<br>ZFMK-DNA-0100438726	Czech Republic, Bohemia or., Hradec Kralove, Svinary, N50°12'28.312" E15°53'55.794", 230m, 25-Jun-2011, *Artemisia vulgaris*, beating, leg. Krátky,J., det. Krátky,J.
Microplontus rugulosus<br>(Herbst, 1795)	MK891359 [new]<br>1410-JKR<br>658nt	ZFMK-TIS-4179<br>ZFMK-DNA-0155628523	France, Cantal, Sainte Eugenie de Villeneuve, N45°08'11" E03°37'40", 973m, 15-Jun-2013, *Artemisia vulgaris*, sweeping, leg. Krátky,J., det. Krátky,J.

**Name**<br>**Authority**<br>**Additional Information**	**GenBank Acc No (Ref.)**<br>**Specimen ID**<br>**Sequence Length**	**ZFMK Tissue ID**<br>**ZFMK DNA Sample ID**<br>**(SDEI DNA Sample ID)**	**Locality, GPS, Collection Date, Plant, Collector, Identifier**
Microplontus rugulosus<br>(Herbst, 1795)	MK891103 [new]<br>1007-PSP<br>658nt	ZFMK-TIS-3152<br>ZFMK-DNA-0100426852	Germany, Lower Saxony (NI), Hannover, Leinhausen, N52°23'53.6" E09°41'24", 52m, 07-Mai-2012, *Artemisia vulgaris*, train station area, beating, leg. Sprick,P., det. Sprick,P.
Minyops variolosus<br>(Fabricius, 1775)	KC783972 [new]<br>293-JKR<br>626nt	ZFMK-TIS-20079<br>ZFMK-DNA-0100438957	Czech Republic, Bohemia or., Stary Svojanov, N49°37'30.948" E16°25'24.879", 525m, 17-Apr-2011, hand-collecting, leg. Krátky,J., det. Krátky,J.
Mitoplinthus caliginosus<br>(Fabricius, 1775)	MK891460 [new]<br>1525-PSP<br>658nt	ZFMK-TIS-3285<br>ZFMK-DNA-0155633315	Germany, Lower Saxony (NI), Hannover, Herrenhausen, Berggarten area, N52°23'39" E09°41'55", 55m, 17-Sep-2012, *Bergenia cordifolia*, Larvensuche, leg. Sprick,P., det. Sprick,P.
Mitoplinthus caliginosus<br>(Fabricius, 1775)	MK892325 [new]<br>2626-PSP<br>658nt	ZFMK-TIS-23600<br>ZFMK-DNA-0171661933	Germany, Bavaria (BY), Unterfranken, Ebern, N50°05'06" E10°45'47", 320m, 14-Jun-2014, collecting by hand, leg. Sprick,P., det. Sprick,P.
Mogulones abbreviatulus<br>(Fabricius, 1792)	MK891545 [new]<br>1635-JKR<br>658nt	ZFMK-TIS-3683<br>ZFMK-DNA-0155635585	Czech Republic, Bohemia, Krasna Hora, N49°35'53" E15°28'22", 443m, 29-Mai-2013, *Symphytum officinale*, individual collecting by hand, leg. Krátky,J., det. Krátky,J.
Mogulones abbreviatulus<br>(Fabricius, 1792)	MK891848 [new]<br>2110-JKR<br>658nt	ZFMK-TIS-23750<br>ZFMK-DNA-0169169620	Czech Republic, Moravia, Lanzhot, N48°42'37" E16°58'27", 156m, 10-Mai-2014, *Symphytum officinale*, sweeping, leg. Krátky,J., det. Krátky,J.
Mogulones andreae<br>Desbrochers des Loges, 1902	KC783935 [new]<br>336-JKR<br>658nt	ZFMK-TIS-20122<br>ZFMK-DNA-0100438750	Slovakia, Komarno, Iza env., PR Bokrosske slanisko , N47°44'50.372" E18°15'34.563", 107m, 20-Mai-2011, *Cerinthe minor*, beating, leg. Krátky,J., det. Krátky,J.
Mogulones andreae<br>(Germar, 1824)	MK891759 [new]<br>2015-JKR<br>658nt	ZFMK-TIS-23655<br>ZFMK-DNA-0169170485	Slovakia, Nove Zamky, Bajtava, Ploska hora, N47°51'54" E18°45'15", 126m, 22-Mai-2014, *Cerinthe minor*, sweeping, leg. Krátky,J., det. Krátky,J.
Mogulones angulicollis<br>(Schultze, 1897)	MK892044 [new]<br>2320-JKR<br>658nt	ZFMK-TIS-24251<br>ZFMK-DNA-0169170938	Slovakia, Tatry Mts., Dolina Siedmich prame?ov, N49°13'30" E20°16'24", 1404m, 05-Jul-2014, *Myosotis*, collecting by hand, leg. Krátky,J., det. Krátky,J.
Mogulones asperifoliarum<br>(Gyllenhal, 1813)	MK891190 [new]<br>1107-JKR<br>658nt	ZFMK-TIS-3443<br>ZFMK-DNA-0100449592	Czech Republic, Moravia, Dukovany, PR Dukovansky mlyn, N49°05'45" E16°11'53", 304m, 01-Mai-2012, *Myosotis* sp., beating, leg. Krátky,J., det. Krátky,J.
Mogulones asperifoliarum<br>(Gyllenhal, 1813)	KC783986 [new]<br>275-PSP<br>658nt	ZFMK-TIS-2D100439096<br>ZFMK-DNA-0100439768	Germany, Lower Saxony (NI), Nordstemmen, Lkr. Hildesheim, N52°09'00" E09°47'00", 72m, 17-Mai-2011, *Myosotis arvensis, Symphytum* sp., garden, beating, leg. Sprick,P., det. Sprick,P.
Mogulones asperifoliarum<br>(Gyllenhal, 1813)	MK890920 [new]<br>788-PST<br>658nt	ZFMK-TIS-3701<br>ZFMK-DNA-0100414303	Germany, Rhineland-Palatinate (RLP), E of Treis-Karden near Pommern, Fellerbachtal (river valley), N50°10'10" E07°13'38", 85m, 09-Jun-2012, *Boraginaceae*, rivulet, beating, leg. Stüben,P., det. Stüben,P.
Mogulones asperifoliarum<br>(Gyllenhal, 1813)	MK891223 [new]<br>1143-JKR<br>658nt	ZFMK-TIS-3479<br>ZFMK-DNA-0100449565	Slovakia, Komarno, Imel, N47°54'34" E18°09'16", 112m, 11-Mai-2012, *Lythospermum arvense*, sweeping, leg. Krátky,J., det. Krátky,J.
Mogulones austriacus<br>(C. Brisout de Barneville, 1869)	MK891775 [new]<br>2034-JKR<br>658nt	ZFMK-TIS-23674<br>ZFMK-DNA-0169170456	Czech Republic, Bohemia, Kosatky, N50°19'23" E14°39'58", 218m, 02-Mai-2014, *Nonea pulla*, collecting by hand, leg. Krátky,J., det. Krátky,J.
Mogulones austriacus<br>(C. Brisout de Barneville, 1869)	MK891826 [new]<br>2087-JKR<br>658nt	ZFMK-TIS-23727<br>ZFMK-DNA-0169169645	Czech Republic, Moravia, Bucovice, PR Sevy, N49°08'05" E16°58'19", 249m, 09-Mai-2014, *Nonea pulla*, sweeping, leg. Krátky,J., det. Krátky,J.

**Name**<br>**Authority**<br>**Additional Information**	**GenBank Acc No (Ref.)**<br>**Specimen ID**<br>**Sequence Length**	**ZFMK Tissue ID**<br>**ZFMK DNA Sample ID**<br>**(SDEI DNA Sample ID)**	**Locality, GPS, Collection Date, Plant, Collector, Identifier**
Mogulones austriacus<br>(C. Brisout de Barneville, 1869)	MK891225 [new]<br>1145-JKR<br>658nt	ZFMK-TIS-3481<br>ZFMK-DNA-0100449563	Slovakia, Nove Zamky, Kamenica nad Hronom, N47°49'52" E18°44'20", 181m, 12-Mai-2012, *Nonea pulla*, beating, leg. Krátky,J., det. Krátky,J.
Mogulones biondii<br>Colonnelli, 1990	KC783733 [13]<br>5-PST<br>629nt	ZFMK-TIS-2D100447042<br>ZFMK-DNA-0100437932	Spain, Canary Islands, Gran Canaria, Marzagan, Barranco de las Goteras, N28°01'28" W15°27'31", 236m, 01-Dez-2010, *Echium strictum*, beating, leg. Stüben,P., det. Stüben,P.
Mogulones biondii<br>Colonnelli, 1990	MK347571 [40]<br>3003-PST<br>658nt	(SDEI-DNA-3003-PST)	Spain, Canary Islands, Lanzarote, above Haria, N29°08'08" W13°30'22", 358m, 04-Jan-2017, *Echium lancerottense*, leg. Stüben,P., det. Stüben,P.
Mogulones cingulatus<br>(Schultze, 1897)	KC783899 [new]<br>112-PST<br>658nt	ZFMK-TIS-2D100440315<br>ZFMK-DNA-0100438390	Morocco, S of Berkane, Beni Snassen Mts., Vallee Zegzel, N34°52'31" W02°21'32", 82m, 11-Mai-2011, sieving, leg. Stüben,P., det. Stüben,P./vid. Behne,L.
Mogulones crucifer<br>(Pallas, 1771)	MK891519 [new]<br>1606-JKR<br>658nt	ZFMK-TIS-3654<br>ZFMK-DNA-0155635623	Czech Republic, Moravia, Cejc - Spidlaky, N48°55'47" E16°58'03", 184m, 01-Mai-2013, *Cynoglossum ofiicinale*, sieving, leg. Krátky,J., det. Krátky,J.
Mogulones crucifer<br>(Pallas, 1771)	MK891908 [new]<br>2175-JKR<br>658nt	ZFMK-TIS-23913<br>ZFMK-DNA-0169170612	Czech Republic, Moravia, Masovice, N48°50'43" E15°58'26", 400m, 01-Apr-2014, *Cynoglossum officinalis*, collecting by hand, leg. Stejskal,R., det. Stejskal,R.
Mogulones crucifer<br>(Pallas, 1771)	MK890871 [new]<br>472-PST<br>658nt	ZFMK-TIS-20257<br>ZFMK-DNA-0100449367	Germany, Rhineland-Palatinate (RLP), "Korretsberg" near Kruft, Eifel, N50°22'49" E07°21'11", 238m, 30-Sep-2011, *Cynoglossum officinale*, volcanic cone, hand-collecting, leg. Stüben,P., det. Stüben,P.
Mogulones crucifer<br>(Pallas, 1771)	MK890893 [new]<br>571-PSP<br>658nt	ZFMK-TIS-20356<br>ZFMK-DNA-0100448408	Germany, Lower Saxony (NI), Neindorf, Lkr. Wolfenbüttel, Öselberg, N52°07'40" E10°35'32", 132m, 24-Jun-2011, *Cynoglossum officinale*, beating, leg. Sprick,P., det. Sprick,P.
Mogulones crucifer<br>(Pallas, 1771)	MK890846 [new]<br>342-JKR<br>658nt	ZFMK-TIS-20128<br>ZFMK-DNA-0100438741	Slovakia, Komarno, Cenkov, N47°46'8.052" E18°31'18.977", 105m, 21-Mai-2011, *Cynoglossum officinale*, beating, leg. Krátky,J., det. Krátky,J.
Mogulones crucifer<br>(Pallas, 1771)	MK891871 [new]<br>2135-JKR<br>658nt	ZFMK-TIS-23775<br>ZFMK-DNA-0169169597	Slovakia, Komarno, Buc, N47°46'55.5" E18°25'10", 107m, 18-Mai-2014, *Cynoglossum* cf. *hungaricum*, sweeping, leg. Krátky,J., det. Krátky,J.
Mogulones cynoglossi<br>(Frauenfeld, 1866)	MK891216 [new]<br>1135-JKR<br>658nt	ZFMK-TIS-3471<br>ZFMK-DNA-0100449572	Slovakia, Komarno, Imel, N47°54'34" E18°09'16", 112m, 11-Mai-2012, *Cynoglossum officinale*, beating, leg. Krátky,J., det. Krátky,J.
Mogulones cynoglossi<br>(Frauenfeld, 1866)	MK891527 [new]<br>1615-JKR<br>658nt	ZFMK-TIS-3663<br>ZFMK-DNA-0155635613	Slovakia, Komarno, B?c env., N47°46'55" E18°25'11", 109m, 18-Mai-2013, *Cynglossum officinale*, individual collecting by hand, leg. Krátky,J., det. Krátky,J.
Mogulones deiectus<br>Colonnelli, 1992	MK347533 [40]<br>2938-PST<br>607nt	(SDEI-DNA-2938-PST)	Spain, Canary Islands, Lanzarote, 1 km W of Ermita de las Nieves, N29°06'27" W13°31'12", 537m, 08-Jan-2017, *Echium lancerottense*, beating, leg. Stüben,P., det. Stüben,P.
Mogulones dimidiatus<br>(J. Frivaldszky, 1865)	MK891510 [new]<br>1596-JKR<br>658nt	ZFMK-TIS-3644<br>ZFMK-DNA-0155635642	Czech Republic, Moravia, Poudrany - step, N48°56'51" E16°38'13", 260m, 01-Mai-2013, *Nonea pulla*, individual collecting by hand, leg. Krátky,J., det. Krátky,J.
Mogulones dimidiatus<br>(J. Frivaldszky, 1865)	MK891783 [new]<br>2042-JKR<br>658nt	ZFMK-TIS-23682<br>ZFMK-DNA-0169170464	Czech Republic, Bohemia, Kosatky, N50°19'23" E14°39'58", 218m, 02-Mai-2014, *Nonea pulla*, sweeping, leg. Krátky,J., det. Krátky,J.

**Name**<br>**Authority**<br>**Additional Information**	**GenBank Acc No (Ref.)**<br>**Specimen ID**<br>**Sequence Length**	**ZFMK Tissue ID**<br>**ZFMK DNA Sample ID**<br>**(SDEI DNA Sample ID)**	**Locality, GPS, Collection Date, Plant, Collector, Identifier**
Mogulones euphorbiae<br>(C. Brisout de Barneville, 1866)	MK891943 [new]<br>2211-JKR<br>658nt	ZFMK-TIS-23949<br>ZFMK-DNA-0169170587	Czech Republic, Bohemia, Hradec pr. Stod, N49°38'10" E13°08'10", 355m, 01-Mai-2014, *Myosotis stricta*, leg. Benedikt,S., det. Benedikt,S.
Mogulones euphorbiae<br>(C. Brisout de Barneville, 1866)	MK891872 [new]<br>2136-JKR<br>658nt	ZFMK-TIS-23776<br>ZFMK-DNA-0169169598	Slovakia, Komarno, Buc, N47°46'55.5" E18°25'10", 107m, 18-Mai-2014, *Myosotis* sp., sweeping, leg. Krátky,J., det. Krátky,J.
Mogulones euphorbiae<br>(C. Brisout de Barneville, 1866)	MK891918 [new]<br>2186-JKR<br>658nt	ZFMK-TIS-23924<br>ZFMK-DNA-0169170610	Slovakia, Nove Zamky, Kamenin, PP Cistiny, N47°51'55" E18°38'05", 111m, 22-Mai-2014, *Myosotis* sp., sweeping, leg. Krátky,J., det. Krátky,J.
Mogulones geographicus<br>(Goeze, 1777)	KC784063 [new]<br>554-RST<br>658nt	ZFMK-TIS-20339<br>ZFMK-DNA-0100448416	Czech Republic, Moravia mer., Podyji NP, 0.9 km N of Hnanice - Stare vinice hill, N48°48'18.81" E15°59'26.12", 300m, 30-Sep-2011, dry heathland, hand-collecting, leg. Stejskal,R., det. Stejskal,R.
Mogulones geographicus<br>(Goeze, 1777)	KC784189 [new]<br>473-PST<br>658nt	ZFMK-TIS-20258<br>ZFMK-DNA-0100449366	Germany, Rhineland-Palatinate (RLP), "Korretsberg" near Kruft, Eifel, N50°22'49" E07°21'11", 238m, 30-Sep-2011, volcanic cone, hand-collecting, leg. Stüben,P., det. Stüben,P.
Mogulones geographicus<br>(Goeze, 1777)	MK890917 [new]<br>785-PST<br>658nt	ZFMK-TIS-3698<br>ZFMK-DNA-0100414306	Germany, Rhineland-Palatinate (RLP), E of Treis-Karden near Pommern, Fellerbachtal (river valley), N50°10'10" E07°13'38", 85m, 09-Jun-2012, *Echium vulgare*, rivulet, beating, leg. Stüben,P., det. Stüben,P.
Mogulones geographicus<br>(Goeze, 1777)	MK891131 [new]<br>1036-PSP<br>658nt	ZFMK-TIS-3181<br>ZFMK-DNA-0100426818	Germany, Lower Saxony (NI), Hannover, Vahrenheide, Kugelfangtrift, N52°25'34" E09°45'31", 49m, 26-Mai-2012, *Echium vulgare*, soil-search under rosette, leg. Sprick,P., det. Sprick,P.
Mogulones geographicus<br>(Goeze, 1777)	MK891023 [new]<br>920-CBR<br>658nt	ZFMK-TIS-3833<br>ZFMK-DNA-0100449694	Luxembourg, 6 km E of Luxembourg, Oetrange, N49°36'20" E06°15'32", 250m, 14-Jun-2011, *Echium vulgare*, beating, leg. Braunert,C., det. Braunert,C.
Mogulones geographicus<br>(Goeze, 1777)	MK891279 [new]<br>1228-PST<br>657nt	ZFMK-TIS-3564<br>ZFMK-DNA-0100426049	Portugal, Minho, Ponte de Lima, Rio Lima, N41°45'48 W08°35'40", 13m, 06-Mai-2012, *Echium*, beating, leg. Stüben,P., det. Krátky,J.
Mogulones geographicus<br>(Goeze, 1777)	MK892152 [new]<br>2440-JKR<br>658nt	ZFMK-TIS-26017<br>ZFMK-DNA-0171600662	Slovakia, Nove Zamky, Imel, PR Liscie diery, N47°55'17" E18°09'37", 114m, 20-Sep-2014, *Echium vulgare*, sweeping, leg. Krátky,J., det. Krátky,J.
Mogulones gratiosus<br>(C. Brisout de Barneville, 1869)	KC783894 [new]<br>179-PST<br>658nt	ZFMK-TIS-2D100438691<br>ZFMK-DNA-0100438366	Morocco, Atlas Mts., SE of Ifrane, Tizi-n-Tretten, N33°27'25" W05°02'16", 1936m, 19-Mai-2011, *Echium*, beating, leg. Stüben,P., det. Behne,L.
Mogulones grisescens<br>(Pic, 1940)	KC784259 [new]<br>IT-0029w<br>656nt	ZFMK-TIS-2D100446532<br>ZFMK-DNA-0100437847	Italy, Sardinia Isl. West, E of Macomer, above Lei, N40°19'54" E08°53'49", 1020m, 04-Okt-2010, *Acer monspessulanum, Quercus*, leg. Stüben,P., det. Stüben,P.
Mogulones grisescens<br>(Pic, 1940)	MK891755 [new]<br>2010-JKR<br>658nt	ZFMK-TIS-23650<br>ZFMK-DNA-0169170480	Italy, Sicilia Isl. (TP), Trapani, Salinagrande, Isolotto, N37°57'14" E12°29'45", 3m, 17-Apr-2014, *Echium plantagineum*, sweeping, leg. Krátky,J., det. Krátky,J.
Mogulones grisescens<br>(Pic, 1940)	KC783882 [new]<br>119-PST<br>658nt	ZFMK-TIS-2D100438640<br>ZFMK-DNA-0100438197	Morocco, S of Berkane, Beni Snassen Mts., Vallee Zegzel, N34°49'33" W02°22'37", 590m, 11-Mai-2011, *Echium*, beating, leg. Stüben,P., det. Stüben,P./vid. Behne,L.
Mogulones grisescens<br>(Pic, 1940)	MK892381 [40]<br>2839-PST<br>658nt	ZFMK-TIS-4235<br>ZFMK-DNA-FC17941486	Portugal, Madeira, Porto Santo Isl., Pico do Castelo, N33°04'44" W16°19'57", 273m, 27-Nov-2015, *Quercus ilex*, sieving, leg. Stüben,P., det. Stüben,P.

**Name**<br>**Authority**<br>**Additional Information**	**GenBank Acc No (Ref.)**<br>**Specimen ID**<br>**Sequence Length**	**ZFMK Tissue ID**<br>**ZFMK DNA Sample ID**<br>**(SDEI DNA Sample ID)**	**Locality, GPS, Collection Date, Plant, Collector, Identifier**
Mogulones javetii<br>(Gerhardt, 1867)	MK891869 [new]<br>2133-JKR<br>658nt	ZFMK-TIS-23773<br>ZFMK-DNA-0169169595	Slovakia, Komarno, Buc, N47°46'55.5" E18°25'10", 107m, 18-Mai-2014, *Anchusa officinalis*, sweeping, leg. Krátky,J., det. Krátky,J.
Mogulones javetii<br>(Gerhardt, 1867)	MK891977 [new]<br>2248-JKR<br>658nt	ZFMK-TIS-23986<br>ZFMK-DNA-0169170528	Slovakia, Komarno, Moca, N47°46'55" E18°25'11", 109m, 20-Sep-2014, *Anchusa officinalis*, leg. Benedikt,S., det. Benedikt,S.
Mogulones larvatus<br>(Schultze, 1897)	MK891228 [new]<br>1148-JKR<br>658nt	ZFMK-TIS-3484<br>ZFMK-DNA-0100449560	Slovakia, Nove Zamky, Salka, Sovie vinohrady, N47°53'14" E18°43'02", 190m, 12-Mai-2012, sweeping, leg. Krátky,J., det. Krátky,J.
Mogulones pallidicornis<br>(Gougelet & H. Brisout de Barneville, 1860)	MK891089 [new]<br>993-PSP<br>658nt	ZFMK-TIS-3138<br>ZFMK-DNA-0100426871	Germany, Lower Saxony (NI), Diekholzen, Lkr. Hildesheim, N52°05'04" E09°55'17", 152m, 01-Mai-2012, *Pulmonaria obscura*, forest of Hildesheim, beating, leg. Sprick,P., det. Sprick,P.
Mogulones peregrinus<br>(Gyllenhal, 1837)	MK891761 [new]<br>2017-JKR<br>658nt	ZFMK-TIS-23657<br>ZFMK-DNA-0169170487	Italy, Sicilia Isl., Agrigento, 7 km S of Ribera, N37°25'48" E13°16'36", 22m, 17-Apr-2014, *Borrago officinalis*, sweeping, leg. Krátky,J., det. Krátky,J.
Mogulones peregrinus<br>(Gyllenhal, 1837)	KC783868 [new]<br>143-PST<br>658nt	ZFMK-TIS-2D100440285<br>ZFMK-DNA-0100438173	Morocco, S of Berkane, N of Aïn-es-Sfa, Beni Snassen Mts., N34°51'30" W02°08'41", 840m, 13-Mai-2011, beating, leg. Stüben,P., det. Behne,L.
Mogulones peregrinus<br>(Gyllenhal, 1837)	MK891180 [new]<br>1097-JKR<br>658nt	ZFMK-TIS-3433<br>ZFMK-DNA-0100449611	Spain, Andalucia, 3 km NE of El Lentiscal, N36°06'07" W05°44'37", 130m, 15-Apr-2012, *Borrago officinalis*, sweeping, leg. Krátky,J., det. Krátky,J.
Mogulones pseudopollinarius<br>(Har. Lindberg, 1950)	MK891605 [40]<br>1689-PST<br>658nt	ZFMK-TIS-24407<br>ZFMK-DNA-0155622501	Spain, Canary Islands, La Palma, near San Isidro, N28°37'47" W17°48'02", 643m, 18-Dez-2013, *Echium plantagineum* , beating, leg. Stüben,P., det. Stüben,P.
Mogulones pseudopollinarius<br>(Har. Lindberg, 1950)	KM433740 [13]<br>723-PST<br>658nt	ZFMK-TIS-3060<br>ZFMK-DNA-0100448164	Spain, Canary Islands, Tenerife, Teno Mts., Teno Alto, N28°20'31" W16°52'12", 752m, 20-Jan-2012, *Echium*, beating, leg. Stüben,P., det. Stüben,P.
Mogulones pseudopollinarius<br>(Har. Lindberg, 1950)	MK890908 [40]<br>744-PST<br>658nt	ZFMK-TIS-3081<br>ZFMK-DNA-0100448143	Spain, Canary Islands, Tenerife, Anaga Mts., El Bailadero, N28°33'01" W16°12'14", 694m, 05-Feb-2012, *Echium strictum*, beating, leg. Stüben,P. & Schütte,A., det. Stüben,P.
Mogulones raphani<br>(Fabricius, 1792)	MK891849 [new]<br>2111-JKR<br>658nt	ZFMK-TIS-23751<br>ZFMK-DNA-0169169621	Czech Republic, Moravia, Lanzhot, N48°42'37" E16°58'27", 156m, 10-Mai-2014, *Symphytum officinale*, sweeping, leg. Krátky,J., det. Krátky,J.
Mogulones raphani<br>(Fabricius, 1792)	KC784046 [new]<br>590-PSP<br>658nt	ZFMK-TIS-20374<br>ZFMK-DNA-0100448284	Germany, Lower Saxony (NI), Braunschweig, Rautheim, Wabeniederung, N52°15'15" E10°35'24", 75m, 09-Jun-2011, *Symphytum officinale*, land recultivation area, dipnet, leg. Sprick,P., det. Sprick,P.
Mogulones raphani<br>(Fabricius, 1792)	KC783934 [new]<br>335-JKR<br>658nt	ZFMK-TIS-20121<br>ZFMK-DNA-0100438749	Slovakia, Komarno, Lohot, N47°51'20.062" E18°0'42.342", 105m, 20-Mai-2011, *Symphytum officinale*, beating, leg. Krátky,J., det. Krátky,J.
Mogulones soricinus<br>(C. Brisout de Barneville, 1869)	MK891174 [new]<br>1087-JKR<br>658nt	ZFMK-TIS-3423<br>ZFMK-DNA-0100449620	Spain, Andalucia, 8 km W of Espera, N36°53'17" W05°53'47", 50m, 17-Apr-2012, *Nonea vesicaria*, beating, leg. Krátky,J., det. Krátky,J.
Mogulones uncipes<br>(Korotyaev, 1980)<br>subspecies of *M. larvatus* ?	MK891280 [new]<br>1230-PST<br>658nt	ZFMK-TIS-3566<br>ZFMK-DNA-0100426051	Portugal, Minho, Barrio, N41°50'42 W08°33'16", 355m, 14-Mai-2012, *Pentaglottis sempervirens*, beating, leg. Stüben,P., det. Krátky,J.

**Name**<br>**Authority**<br>**Additional Information**	**GenBank Acc No (Ref.)**<br>**Specimen ID**<br>**Sequence Length**	**ZFMK Tissue ID**<br>**ZFMK DNA Sample ID**<br>**(SDEI DNA Sample ID)**	**Locality, GPS, Collection Date, Plant, Collector, Identifier**
Mogulones uncipes<br>(Korotyaev, 1980)<br>subspecies of *M. larvatus* ?	MK891286 [new]<br>1239-PST<br>658nt	ZFMK-TIS-3575<br>ZFMK-DNA-0100426045	Portugal, Minho, NW of Ponte de Lima, Vila Nova de Cerveira, Rio Coura, N41°52'27 W08°41'57", 123m, 07-Mai-2012, *Pentaglottis sempervirens*, beating, leg. Stüben,P., det. Krátky,J.
Mogulonoides radula<br>(Germar, 1824)	KC784071 [new]<br>552-RST<br>658nt	ZFMK-TIS-20337<br>ZFMK-DNA-0100448427	Czech Republic, Moravia mer., Znojmo, intravilan, N48°51'31.06" E16°2'40.56", 300m, 23-Sep-2011, *Anchusa officinalis*, hand-collecting, leg. Stejskal,R., det. Stejskal,R.
Mononychus punctumalbum<br>(Herbst, 1784)	MK891017 [new]<br>914-CBR<br>658nt	ZFMK-TIS-3827<br>ZFMK-DNA-0100449688	France, Dep. Marne, 15 km SW of Vertus, 2 km SE of Coizard-Joches, N48°49'03" E03°53'11", 140m, 19-Mai-2011, *Iris pseudacorus*, beating, leg. Braunert,C., det. Braunert,C.
Mononychus punctumalbum<br>(Herbst, 1784)	MK891129 [new]<br>1034-PSP<br>658nt	ZFMK-TIS-3179<br>ZFMK-DNA-0100426952	Germany, Lower Saxony (NI), Braunschweig, Rautheim, N52°14'23.5" E10°35'45", 78m, 24-Mai-2012, *Iris pseudacorus*, Wabe river shore, beating, leg. Sprick,P., det. Sprick,P.
Mononychus punctumalbum<br>(Herbst, 1784)	MK891642 [new]<br>1795-PST<br>658nt	ZFMK-TIS-24129<br>ZFMK-DNA-0171624069	Germany, Rhineland-Palatinate (RLP), Ahr valley, Dernau, below Krausberg near Steinbergsmühle, N50°31'40" E07°02'43", 204m, 29-Mai-2014, *Iris*, beating, leg. Stüben,P., det. Stüben,P.
Mononychus punctumalbum<br>(Herbst, 1784)	KC784231 [new]<br>397-RGO<br>658nt	ZFMK-TIS-20183<br>ZFMK-DNA-0100449442	Poland, ad. Urszulin , N51°22'16" E23°15'04" , 172m, 06-Jun-2011, leg. Gosik,R., det. Gosik,R.
Mononychus punctumalbum<br>var. *salviae*<br>(Herbst, 1784)	MK891538 [new]<br>1627-JKR<br>658nt	ZFMK-TIS-3675<br>ZFMK-DNA-0155635602	Slovakia, Nove Zamky, Male Kosihy, N47°49'34" E18°45'18", 107m, 19-Mai-2013, *Iris lutea*, individual collecting by hand, leg. Krátky,J., det. Krátky,J.
Montanacalles nevadaensis<br>(Stüben, 2001)	EU286498 [01]<br>E-0189-nev<br>658nt	ZFMK-TIS-cE0189<br>ZFMK-DNA-0100400226	Spain, Almeria, 11 km NW of Laujar de Andarax, Sierra Nevada, Bayarcal, N37°02'27" W03°00'12", 1291m, 07-Apr-2007, *Quercus ilex,* broom, *Euphorbia*, leg. Astrin,J. & Stüben,P., det. Stüben,P.
Moreiba canariensis<br>(Franz, 1995)	MK892397 [40]<br>2874-PST<br>658nt	ZFMK-TIS-25879<br>ZFMK-DNA-0169166918	Spain, Canary Islands, El Hierro, El Golfo, Las Pundas, Los Gusmines, N27°47'40" W17°59'32", 29m, 12-Apr-2016, sieving, leg. Stüben,P. & Schütte,A., det. Stüben,P.
Moreiba canariensis<br>(Franz, 1995)	MK892405 [40]<br>2884-PST<br>658nt	ZFMK-TIS-25889<br>ZFMK-DNA-0169166911	Spain, Canary Islands, El Hierro, S of Tamaduste, N27°49'09" W17°53'35", 59m, 18-Apr-2016, *Euphorbia balsamifera, Periploca*, sieving, leg. Stüben,P. & Schütte,A., det. Stüben,P.
Nanodiscus transversus<br>(Aubé, 1850)	KC783764 [40]<br>54-PST<br>630nt	ZFMK-TIS-2D100446994<br>ZFMK-DNA-0100438066	Spain, Canary Islands, La Gomera, Hermigua, Las Nuevitas, Barranquillos los Alamos, N28°09'31" W17°11'09", 251m, 13-Dez-2010, *Juniperus*, beating, leg. Stüben,P., det. Stüben,P.
Nanodiscus transversus<br>(Aubé, 1850)	MK890961 [new]<br>842-PST<br>658nt	ZFMK-TIS-3755<br>ZFMK-DNA-0100414240	Portugal, Estremadura, NW of Sintra, Magoito, coast , N38°52'03" W09°26'31", 29m, 16-Mai-2012, *Juniperus*, beating, leg. Stüben,P., det. Stüben,P.
Nanophyes brevis<br>Boheman, 1845	MK892311 [new]<br>2612-PSP<br>658nt	ZFMK-TIS-23586<br>ZFMK-DNA-0171661952	Germany, Bavaria (BY), Unterfranken, Ebern, N50°04'57" E10°45'49", 300m, 14-Jun-2014, *Lythrum salicaria*, leg. Sprick,P., det. Sprick,P.
Nanophyes globiformis<br>Kiesenwetter, 1864	KC784079 [new]<br>544-RST<br>658nt	ZFMK-TIS-20329<br>ZFMK-DNA-0100448435	Czech Republic, Moravia mer., Podyji NP, 0.6 km NE of Hnanice, N48°48'6.90" E15°59'29.96", 280m, 01-Sep-2011, *Lythrum hyssopifolium*, sweeping, leg. Stejskal,R., det. Stejskal,R.
Nanophyes marmoratus<br>(Goeze, 1777)	KC784085 [new]<br>533-RST<br>658nt	ZFMK-TIS-20318<br>ZFMK-DNA-0100448443	Czech Republic, Bohemia mer., Lasenice, 2.5 km SSW of the village, N49°3'8.20" E14°57'40.32", 450m, 19-Aug-2011, *Lythrum salicaria*, beating, leg. Stejskal,R., det. Stejskal,R.

**Name**<br>**Authority**<br>**Additional Information**	**GenBank Acc No (Ref.)**<br>**Specimen ID**<br>**Sequence Length**	**ZFMK Tissue ID**<br>**ZFMK DNA Sample ID**<br>**(SDEI DNA Sample ID)**	**Locality, GPS, Collection Date, Plant, Collector, Identifier**
Nanophyes marmoratus<br>(Goeze, 1777)	MK891423 [new]<br>1486-PSP<br>658nt	ZFMK-TIS-3246<br>ZFMK-DNA-0155633343	Germany, Lower Saxony (NI), Königsförde, Lkr. Hameln-Pyrmont, N52°03'59" E09°16'17", 94m, 15-Jul-2012, *Lythrum salicaria*, housegarden, pond, dipnet, leg. Sprick,P., det. Sprick,P.
Nanophyes marmoratus<br>(Goeze, 1777)	MK891683 [new]<br>1899-PST<br>658nt	ZFMK-TIS-24004<br>ZFMK-DNA-0169170424	Germany, North Rhine-Westphalia (NRW), Elmpt, Overhetfeld, Dilborner Mühle, N51°13'58" E06°10'22", 52m, 27-Aug-2014, *Lythrum salicaria*, beating, leg. Stüben,P., det. Stüben,P.
Nanophyes marmoratus<br>(Goeze, 1777)	MK891352 [new]<br>1397-PST<br>658nt	ZFMK-TIS-4693<br>ZFMK-DNA-0155628531	Italy, Lazio, SE of Sora, Lago di Fibrano, N41°41'39" E13°41'38", 444m, 30-Aug-2013, *Lythrum*, beating, leg. Stüben,P., det. Stüben,P.
Nanophyes marmoratus<br>(Goeze, 1777)	MK891058 [new]<br>959-CBR<br>658nt	ZFMK-TIS-3872<br>ZFMK-DNA-0100449652	Luxembourg, 10 km E of Luxembourg, 1 km SW of Mensdorf, N49°39'00" E06°17'40", 240m, 21-Jun-2012, *Lythrum salicaria*, beating, leg. Braunert,C., det. Braunert,C.
Nanophyes marmoratus<br>(Goeze, 1777)	KC784202 [new]<br>447-RGO<br>658nt	ZFMK-TIS-20233<br>ZFMK-DNA-0100449392	Poland, Rudnik, N51°14'31.02" E22°32'22.92", 191m, 03-Okt-2011, leg. Gosik,R., det. Gosik,R.
Naupactus cervinus<br>(Boheman, 1840)	KC783754 [40]<br>43-PST<br>658nt	ZFMK-TIS-2D100447005<br>ZFMK-DNA-0100438077	Spain, Canary Islands, Gran Canaria, Teror, Barranco de Teror, N28°03'41" W15°32'04", 482m, 08-Jan-2011, herbaceous vegetation next to river, beating, leg. Stüben,P., det. Stüben,P.
Naupactus cervinus<br>(Boheman, 1840)	KC783796 [40]<br>88-PST<br>658nt	ZFMK-TIS-2D100446953<br>ZFMK-DNA-0100438025	Spain, Canary Islands, La Gomera, S of Hermigua, El Estanquilo, N28°8'47" W17°12'20", 365m, 21-Feb-2011, garden, beating, leg. Stüben,P., det. Stüben,P.
Naupactus cervinus<br>(Boheman, 1840)	MK891601 [40]<br>1683-PST<br>658nt	ZFMK-TIS-24401<br>ZFMK-DNA-0155622512	Spain, Canary Islands, La Palma, near San Isidro, N28°38'48" W17°47'51", 526m, 18-Dez-2013, beating, leg. Stüben,P., det. Stüben,P.
Naupactus cervinus<br>(Boheman, 1840)	MK891650 [40]<br>1828-PST<br>658nt	ZFMK-TIS-26031<br>ZFMK-DNA-0171606138	Portugal, Madeira, S of Seixal, S of Chao da Ribeira, N32°48'41" W17°06'38", 411m, 27-Jun-2014, beating, leg. Stüben,P., det. Stüben,P.
Nedyus quadrimaculatus<br>(Linnaeus, 1758)	KC783967 [new]<br>299-JKR<br>658nt	ZFMK-TIS-20085<br>ZFMK-DNA-0100438935	Czech Republic, Bohemia or., Dritec, N50°7'11.648" E15°48'6.919", 230m, 06-Mai-2011, *Urtica dioica*, sweeping, leg. Krátky,J., det. Krátky,J.
Nedyus quadrimaculatus<br>(Linnaeus, 1758)	MK891278 [new]<br>1225-JKR<br>645nt	ZFMK-TIS-3561<br>ZFMK-DNA-0100426059	Czech Republic, Bohemia, Velke Petrovice, N50°30'32" E16°12'42", 401m, 21-Okt-2012, *Salix, Alnus*, sieving, leg. Krátky,J., det. Krátky,J.
Nedyus quadrimaculatus<br>(Linnaeus, 1758)	KC784015 [new]<br>227-PSP<br>658nt	ZFMK-TIS-2D100439144<br>ZFMK-DNA-0100439816	Germany, Lower Saxony (NI), Lemmie, Hannover region, Gehrdener Berg, N52°17'44" E09°35'23", 100m, 22-Apr-2011, *Urtica dioica*, field edge, beating, leg. Sprick,P., det. Sprick,P.
Nedyus quadrimaculatus<br>(Linnaeus, 1758)	MK890921 [new]<br>789-PST<br>658nt	ZFMK-TIS-3702<br>ZFMK-DNA-0100414302	Germany, Rhineland-Palatinate (RLP), E of Treis-Karden near Pommern, Fellerbachtal (river valley), N50°10'10" E07°13'38", 85m, 09-Jun-2012, *Urtica*, rivulet, beating, leg. Stüben,P., det. Stüben,P.
Nedyus quadrimaculatus<br>(Linnaeus, 1758)	MK890994 [new]<br>886-CBR<br>658nt	ZFMK-TIS-3799<br>ZFMK-DNA-0100449725	Luxembourg, 10 km E of Luxembourg, 2 km S of Mensdorf, N49°38'09" E06°17'48", 330m, 11-Apr-2011, *Urtica dioica*, sweeping, leg. Braunert,C., det. Braunert,C.
Nedyus quadrimaculatus<br>(Linnaeus, 1758)	KC784176 [new]<br>454-RGO<br>658nt	ZFMK-TIS-20240<br>ZFMK-DNA-0100449096	Poland, Rudnik, N51°14'31.02" E22°32'22.92", 191m, 03-Okt-2011, leg. Gosik,R., det. Gosik,R.

**Name**<br>**Authority**<br>**Additional Information**	**GenBank Acc No (Ref.)**<br>**Specimen ID**<br>**Sequence Length**	**ZFMK Tissue ID**<br>**ZFMK DNA Sample ID**<br>**(SDEI DNA Sample ID)**	**Locality, GPS, Collection Date, Plant, Collector, Identifier**
Nedyus quadrimaculatus<br>(Linnaeus, 1758)	KC784178 [new]<br>401-RGO<br>658nt	ZFMK-TIS-20187<br>ZFMK-DNA-0100449102	Poland, ad. Urszulin , N51°22'16" E23°15'04" , 172m, 06-Jun-2011, leg. Gosik,R., det. Gosik,R.
Nedyus quadrimaculatus<br>(Linnaeus, 1758)	MK891873 [new]<br>2137-JKR<br>658nt	ZFMK-TIS-23777<br>ZFMK-DNA-0169169599	Slovakia, Komarno, Buc, N47°46'55.5" E18°25'10", 107m, 18-Mai-2014, *Urtica dioica*, sweeping, leg. Krátky,J., det. Krátky,J.
Nemonyx lepturoides<br>(Fabricius, 1801)	KC784222 [new]<br>407-RGO<br>658nt	ZFMK-TIS-20193<br>ZFMK-DNA-0100449425	Poland, Rudnik, N51°14'31.02" E22°32'22.92", 191m, 05-Jun-2011, leg. Gosik,R., det. Gosik,R.
Neocoenorrhinus aeneovirens<br>(Marsham, 1802)	MK892188 [new]<br>2483-PSP<br>658nt	ZFMK-TIS-23455<br>ZFMK-DNA-0171661790	Germany, Lower Saxony (NI), Bredenbeck-Steinkrug, Hannover region, Deister, N52°14'04" E09°37'41", 179m, 27-Mrz-2014, *Quercus robur*, beating, leg. Sprick,P., det. Sprick,P.
Neocoenorrhinus germanicus<br>(Herbst, 1797)	MK891832 [new]<br>2093-JKR<br>658nt	ZFMK-TIS-23733<br>ZFMK-DNA-0169169651	Czech Republic, Moravia, Bucovice, PR Sevy, N49°08'05" E16°58'19", 249m, 09-Mai-2014, sweeping, leg. Krátky,J., det. Krátky,J.
Neocoenorrhinus germanicus<br>(Herbst, 1797)	MK892168 [new]<br>2459-PSP<br>658nt	ZFMK-TIS-23431<br>ZFMK-DNA-0171661814	Germany, Lower Saxony (NI), Hameln-Rohrsen, Dütberg, N52°06'21" E09°24'49", 104m, 10-Mai-2013, *Prunus spinosa*, beating, leg. Sprick,P., det. Sprick,P.
Neocoenorrhinus germanicus<br>(Herbst, 1797)	MK891897 [new]<br>2163-JKR<br>658nt	ZFMK-TIS-23803<br>ZFMK-DNA-0169169577	Slovakia, Komarno, Marcelova, PR Masan, N47°46'10.5" E18°19'06", 121m, 21-Mai-2014, sweeping, leg. Krátky,J., det. Krátky,J.
Neocoenorrhinus pauxillus<br>(Germar, 1824)	KC784013 [new]<br>231-PSP<br>658nt	ZFMK-TIS-2D100438515<br>ZFMK-DNA-0100439812	Germany, Lower Saxony (NI), Hemmingen, Hannover region, N52°19'08" E09°44'00", 54m, 23-Apr-2011, *Cotoneaster dammeri*, tennis court, edge cultivation, beating, leg. Sprick,P., det. Sprick,P.
Neoglocianus albovittatus<br>(Germar, 1824)	KC784118 [new]<br>668-FBA<br>658nt	ZFMK-TIS-20449<br>ZFMK-DNA-0100448599	Greece, Peloponnese, Messinia, W of Mt. Taygetos, Kastania, N36°51'46" E22°18'38", 550m, 18-Mai-2011, leg. Bahr,F., det. Bahr,F.
Neoglocianus maculaalba<br>(Herbst, 1795)	MK891550 [new]<br>1640-JKR<br>658nt	ZFMK-TIS-3688<br>ZFMK-DNA-0155635590	Czech Republic, Moravia, Rudlice, N48°58'20" E16°06'45", 256m, 08-Jun-2013, *Papaver rhoeas*, individual collecting by hand, leg. Krátky,J., det. Krátky,J.
Neoglocianus maculaalba<br>(Herbst, 1795)	MK891296 [new]<br>1267-PST<br>658nt	ZFMK-TIS-4083<br>ZFMK-DNA-0100426435	Spain, Zaragoza, Aragon, NO of Calatayud, N41°22'20" W01°35'01", 703m, 30-Apr-2013, *Papaver*, beating, leg. Stüben,P. & Schütte,A., det. Stüben,P.
Neoglocianus maculaalba<br>(Herbst, 1795)	MK891318 [new]<br>1315-PST<br>658nt	ZFMK-TIS-4131<br>ZFMK-DNA-0100426412	Spain, Malaga, S of Mollina, N37°04'45" W04°40'08", 437m, 07-Mai-2013, *Papaveraceae*, beating, leg. Stüben,P. & Schütte,A., det. Stüben,P.
Neophytobius granatus<br>(Gyllenhal, 1835)	KC783959 [new]<br>358-JKR<br>658nt	ZFMK-TIS-20144<br>ZFMK-DNA-0100438921	Bulgaria, Blagoevgrad, Pirin Mts., Kresna env., Struma riv., N41°45'51.67" E23°09'20.16", 200m, 10-Jun-2011, hand-collecting, leg. Krátky,J., det. Krátky,J.
Neophytobius granatus<br>(Gyllenhal, 1835)	MK891382 [new]<br>1435-JKR<br>658nt	ZFMK-TIS-4204<br>ZFMK-DNA-0155628498	Slovakia, Levice, Cata, Hron. Riv., N47°57'22" E18°39'39", 121m, 21-Jul-2013, *Polygonum mite*, individual collecting by hand, leg. Krátky,J., det. Krátky,J.
Notaris acridulus<br>(Linnaeus, 1758)	MK891003 [new]<br>897-CBR<br>658nt	ZFMK-TIS-3810<br>ZFMK-DNA-0100449719	France, Dep. Ardenne, 10 km SE of Vouziers, Brecy-Brieres, N49°19'25" E04°46'23", 100m, 23-Apr-2011, sweeping, leg. Braunert,C., det. Braunert,C.

**Name**<br>**Authority**<br>**Additional Information**	**GenBank Acc No (Ref.)**<br>**Specimen ID**<br>**Sequence Length**	**ZFMK Tissue ID**<br>**ZFMK DNA Sample ID**<br>**(SDEI DNA Sample ID)**	**Locality, GPS, Collection Date, Plant, Collector, Identifier**
Notaris acridulus<br>(Linnaeus, 1758)	KC784002 [new]<br>249-PSP<br>658nt	ZFMK-TIS-2D100438535<br>ZFMK-DNA-0100439794	Germany, Lower Saxony (NI), Essel, Lkr. Soltau-Fallingbostel, N52°41'57" E09°38'36", 25m, 10-Mai-2011, *Phalaris arundinacea*, sand field, dipnet, leg. Sprick,P., det. Sprick,P.
Notaris acridulus<br>(Linnaeus, 1758)	KC784149 [new]<br>622-PSP<br>658nt	ZFMK-TIS-20405<br>ZFMK-DNA-0100448645	Germany, Lower Saxony (NI), Hemmingen, Hannover region, N52°18'56" E09°45'03", 58m, 29-Jul-2011, *Glyceria fluitans*, wet grassland, beating, leg. Sprick,P., det. Sprick,P.
Notaris aterrimus<br>(Hampe, 1850)	MK891268 [new]<br>1208-JKR<br>658nt	ZFMK-TIS-3544<br>ZFMK-DNA-0100426077	Czech Republic, Moravia, Hruby jesenik, Velka Kotlina, N50°3'18" E17°14'14", 1209m, 23-Aug-2012, sieving, leg. Krátky,J., det. Krátky,J.
Notaris scirpi<br>(Fabricius, 1792)	KC784137 [new]<br>641-PSP<br>658nt	ZFMK-TIS-20424<br>ZFMK-DNA-0100448626	Germany, Saxony-Anhalt (ST), Drömling, Mannhausen, N52°25'36" E11°12'36.5'', 54m, 08-Sep-2011, *Glyceria maxima*, beating, leg. Sprick,P., det. Sprick,P.
Oedecnemidius varius<br>(Brullé, 1832)	JX448424 [new]<br>659-PSP<br>658nt	ZFMK-TIS-c659-PSP<br>ZFMK-DNA-0100448617	Cyprus, Troodos, Cedar Valley, N34°59'30" E32°41'19", 1146m, 20-Apr-2010, *Quercus alnifolia*, submontaneous mixed deciduous woodland, beating, leg. Sprick,P., det. Sprick,P.
Omiamima mollina<br>(Boheman, 1834)	MK890844 [new]<br>323-JKR<br>658nt	ZFMK-TIS-20109<br>ZFMK-DNA-0100438760	Czech Republic, Bohemia or., Hradec Kralove - Fararstvi, N50°11'22.247" E15°48'53.64", 230m, 17-Mai-2011, sweeping, leg. Krátky,J., det. Krátky,J.
Omiamima mollina<br>(Boheman, 1834)	MK891520 [new]<br>1607-JKR<br>658nt	ZFMK-TIS-3655<br>ZFMK-DNA-0155635622	Czech Republic, Bohemia, Detrichov pr. Svitavy, N49°48'12" E16°33'43", 570m, 06-Mai-2013, sweeping, leg. Krátky,J., det. Krátky,J.
Omiamima mollina<br>(Boheman, 1834)	MK892195 [new]<br>2490-PSP<br>658nt	ZFMK-TIS-23462<br>ZFMK-DNA-0171661780	Germany, Saxony-Anhalt (ST), Harz National Park, Drei Annen Hohne, Hohneklippen, Hohnekopf, N51°46'37" E10°41'55", 840m, 24-Apr-2014, *Picea abies, Calamagrostis villosa*, dipnet, leg. Sprick,P., det. Sprick,P.
Omias puberulus<br>Boheman, 1834	MK891512 [new]<br>1598-JKR<br>658nt	ZFMK-TIS-3646<br>ZFMK-DNA-0155635631	Czech Republic, Moravia, Poudrany - step, N48°56'51" E16°38'13", 260m, 01-Mai-2013, sweeping, leg. Krátky,J., det. Krátky,J.
Omias winkelmanni<br>Borovec & Bahr, 2012	JX448428 [08]<br>665-FBA<br>658nt	ZFMK-TIS-c665-FBA<br>ZFMK-DNA-0100448602	Greece, Chalcidice, Taxiarchis env., NE of Polygyros, N40°23'21" E23°27'53", 925m, 19-Okt-2010, leg. Bahr,F. & Winkelmann,H., det. Bahr,F.
Omphalapion hookerorum<br>(Kirby, 1808)	MK892091 [new]<br>2367-JKR<br>658nt	ZFMK-TIS-25927<br>ZFMK-DNA-0171600589	Czech Republic, Moravia, NP Podyji, Podmyce, Jejkal Pond, N48°51'24" E15°54'15.5", 420m, 17-Aug-2014, *Tripleurospermum*, sweeping, leg. R. Stejskal, det. Stejskal,R.
Omphalapion hookerorum<br>(Kirby, 1808)	MK891133 [new]<br>1038-PSP<br>658nt	ZFMK-TIS-3183<br>ZFMK-DNA-0100426912	Germany, Lower Saxony (NI), Isernhagen-Süd, Hannover region, N52°26'41" E09°47'07", 49m, 26-Mai-2012, *Tripleurospermum perforatum*, weeds area at hill, dipnet, leg. Sprick,P., det. Sprick,P.
Omphalapion hookerorum<br>(Kirby, 1808)	MK892317 [new]<br>2618-PSP<br>658nt	ZFMK-TIS-23592<br>ZFMK-DNA-0171661941	Germany, Bavaria (BY), Unterfranken, Ebern, N50°05'00" E10°45'55", 308m, 14-Jun-2014, *Tripleurospermum perforatum*, leg. Sprick,P., det. Sprick,P.
Omphalapion rhodopense<br>(Angelov, 1962)	MK891692 [new]<br>1909-FBA<br>630nt	ZFMK-TIS-23300<br>ZFMK-DNA-0169170374	Greece, East Macedonia and Thrace, Drama, Limni Katafyto, N41°21'24" E23°38'19", 1270m, 10-Aug-2014, leg. Bayer & Brunner, det. Bayer,F.
Onychapion tamarisci<br>(Gyllenhal, 1839)	KC783863 [new]<br>150-PST<br>658nt	ZFMK-TIS-2D100440277<br>ZFMK-DNA-0100438165	Morocco, NW of Berkane, Oued Moulouya, N34°56'49" W02°33'08", 49m, 14-Mai-2011, *Tamarix*, beating, leg. Stüben,P., det. Stüben,P./vid. Behne,L.

**Name**<br>**Authority**<br>**Additional Information**	**GenBank Acc No (Ref.)**<br>**Specimen ID**<br>**Sequence Length**	**ZFMK Tissue ID**<br>**ZFMK DNA Sample ID**<br>**(SDEI DNA Sample ID)**	**Locality, GPS, Collection Date, Plant, Collector, Identifier**
Onycholips bifurcatus<br>Wollaston, 1861	MH051973 [33]<br>2936-PST<br>658nt	(SDEI-DNA-2936-PST)	Spain, Canary Islands, Lanzarote, SE of Órzola, sandy beach, N29°12'18" W13°25'29", 6m, 06-Jan-2017, *on roots*, sifting, leg. Stüben,P., det. Stüben,P.
Onyxacalles balearicus<br>Stüben, 2005<br>**Paratype (DNAtype)**	EU286521 [01]<br>E-0168-bal<br>658nt	ZFMK-TIS-cE0168<br>ZFMK-DNA-0100400367	Spain, Mallorca, 3 km SE of Lluc, Sra. de Tramuntana, Sa Maleta, N39°48'47" E02°53'23", 571m, 22-Aug-2004, *Quercus ilex*, riverside, leg. Stüben,P., det. Stüben,P.
Onyxacalles bermejaensis<br>Stüben, 2001	EU286514 [01]<br>E-0167-ber<br>658nt	ZFMK-TIS-cE0167<br>ZFMK-DNA-0100400387	Spain, Andalucia, 11 km S of Ronda, Sierra de las Nieves, N36°39'51" W05°05'01", 1047m, 03-Okt-2005, *Quercus ilex*, limestone, leg. Stüben,P., det. Stüben,P.
Onyxacalles bermejaensis<br>Stüben, 2001	GU987827 [01]<br>E-0194-ber<br>658nt	ZFMK-TIS-cE0194<br>ZFMK-DNA-0100400076	Spain, Malaga, 9 km SE of Ubrique, Sierra de Libar, N36°36'52" W05°23'16, 663m, 10-Apr-2007, *Quercus ilex, Ceratonia*, cliff, leg. Astrin,J. & Stüben,P., det. Stüben,P.
Onyxacalles bermejaensis<br>Stüben, 2001	GU988066 [01]<br>E-778-ber<br>658nt	ZFMK-TIS-cE778<br>ZFMK-DNA-0100405032	Spain, Malaga, NW of Marbella, Sierra de las Nieves, N36°39'52" W05°04'57", 1043m, 17-Jan-2009, *Quercus ilex with Echinodera spinosa*, leg. Stüben,P., det. Stüben,P.
Onyxacalles bermejaensis<br>Stüben, 2001	JN121398 [05]<br>ES1062<br>658nt	ZFMK-TIS-cES1062<br>ZFMK-DNA-0112704604	Spain, Malaga, Algatocin near Opayar, N36°34'39" W05°18'13", 576m, 17-Aug-2010, *Quercus* sp., leg. Stüben,P., det. Stüben,P.
Onyxacalles bermejaensis<br>Stüben, 2001	KJ867603 [new]<br>1284-PST<br>658nt	ZFMK-TIS-4100<br>ZFMK-DNA-0100426201	Spain, Malaga, NE of Ronda, Puerto del Viento Blanqilla, N36°47'16" W05°02'47", 1065m, 11-Mai-2013, *Quercus ilex, Crataegus*, sieving, leg. Stüben,P. & Schütte,A., det. Stüben,P.
Onyxacalles bermejaensis<br>Stüben, 2001	KJ867606 [new]<br>1290-PST<br>658nt	ZFMK-TIS-4106<br>ZFMK-DNA-0100426413	Spain, Malaga, S of Ronda, Igualeja, N36°37'29" W05°07'25", 672m, 12-Mai-2013, *Ficus carica*, sieving, leg. Stüben,P. & Schütte,A., det. Stüben,P.
Onyxacalles caucasicus<br>(Reitter, 1891)<br>**Paratype (DNAtype)**<br>formerly: *Acalles caucasicus*	MF464646 [34]<br>2753-PST_3981<br>658nt	ZFMK-TIS-3981<br>ZFMK-DNA-0171661896	Russia, Krasnodar, Chvizhepse vill. env. , N43°38'32" E40°04'45", 300m, 10-Jul-2014, leg. Kovalev,A.V., det. Stüben,P.
Onyxacalles caucasicus<br>(Reitter, 1891)<br>**Paratype (DNAtype)**<br>formerly: *Acalles caucasicus*	MF464647 [34]<br>2757-PST_26854<br>658nt	ZFMK-TIS-26854<br>ZFMK-DNA-0171661900	Russia, Krasnodar, Chvizhepse vill. env. , N43°38'32" E40°04'45", 300m, 11-Jul-2014, leg. Kovalev,A.V., det. Stüben,P.
Onyxacalles cf. *luigionii*	GU987897 [01]<br>F-440-lui<br>658nt	ZFMK-TIS-cF440<br>ZFMK-DNA-0100400666	France, Alpes-Maritimes, 3 km W of Sospel, Col de Braus, N43°52'34" E07°24'17", 1051m, 26-Dez-2007, *Quercus pubescens, Ostrya carpinifolia,* broom, leg. Stüben,P., det. Stüben,P.
Onyxacalles cf. *luigionii*	KC783821 [new]<br>564-PST<br>658nt	ZFMK-TIS-20349<br>ZFMK-DNA-0100448426	Italy, Venetto, Bocca di Navene, Monte Balso Mts., N45°47'16" E10°52'21", 1415m, 12-Aug-2011, *Fagus* sp., sieving, leg. Kresl,P., det. Stüben,P.
Onyxacalles ganglbaueri<br>(A. & F. Solari 1907)	MG229822 [new]<br>2887-PST<br>658nt	ZFMK-TIS-25893<br>ZFMK-DNA-0169166914	Greece, Drama, E-Macedonia, Falakro Mts., N41°17'47" E24°00'12", 1080m, 25-Mai-2016, Fagus forest with Carpinus, sieving, leg. Behne,L., det. Stüben,P.
Onyxacalles ganglbaueri<br>(A. & F. Solari 1907)	MG229823 [new]<br>2889-PST<br>658nt	ZFMK-TIS-25895<br>ZFMK-DNA-0169166903	Greece, Drama, E-Macedonia, Falakro Mts., N41°17'47" E24°00'12", 1080m, 25-Mai-2016, Fagus forest with Carpinus, sieving, leg. Behne,L., det. Stüben,P.

**Name**<br>**Authority**<br>**Additional Information**	**GenBank Acc No (Ref.)**<br>**Specimen ID**<br>**Sequence Length**	**ZFMK Tissue ID**<br>**ZFMK DNA Sample ID**<br>**(SDEI DNA Sample ID)**	**Locality, GPS, Collection Date, Plant, Collector, Identifier**
Onyxacalles georgius<br>Stüben & Krátky 2018	MF946564 [34]<br>2945-PST<br>658nt	(SDEI-DNA-2945-PST)	Georgia, Caucasus, Lagodechi nat. res., Shromiskhevi riv., N41°51'51" E46°17'34", 778m, 21-Apr-2016, sifting, leg. Krátky,J., det. Stüben,P. & Krátky,J.
Onyxacalles gibraltarensis<br>Stüben, 2002	KY211664 [new]<br>2893-PST<br>658nt	ZFMK-TIS-25899<br>ZFMK-DNA-FC19438832	England, Kent, Richborough, N51°18'48" E01°20'18", 3m, 21-Apr-2016, leg. Telfer, det. Stüben,P.
Onyxacalles gibraltarensis<br>Stüben, 2002	KY211665 [new]<br>2894-PST<br>658nt	ZFMK-TIS-25900<br>ZFMK-DNA-FC19438824	England, Kent, Richborough, N51°18'28" E01°20'46", 5m, 30-Mai-2016, beating, leg. Denton, det. Stüben,P.
Onyxacalles gibraltarensis<br>Stüben, 2002	GU988137 [01]<br>E-897-gib<br>606nt	ZFMK-TIS-cE897<br>ZFMK-DNA-0100405494	Morocco, Rif, SW Oued-Laou, SW of Oued-Laou, river, O. Laou, N35°17'47" W05°13'38", 210m, 08-Mai-2009, *Quercus suber, Smilax, Arbutus* , leg. Stüben,P., det. Stüben,P.
Onyxacalles gibraltarensis<br>Stüben, 2002	GU988138 [01]<br>E-899-gib<br>658nt	ZFMK-TIS-cE899<br>ZFMK-DNA-0100405492	Morocco, Sebta West, vir. Biutz, N35°53'04" W05°24'08", 337m, 09-Mai-2009, *Quercus suber, Smilax, Arbutus*, leg. Stüben,P., det. Stüben,P.
Onyxacalles gibraltarensis<br>Stüben, 2002	GU988144 [01]<br>E-907-gib<br>658nt	ZFMK-TIS-cE907<br>ZFMK-DNA-0100405485	Morocco, S of Ksar-es-Seghir, N35°45'16" W05°30'49", 278m, 09-Mai-2009, *Pistacia, Quercus suber*, leg. Stüben,P., det. Stüben,P.
Onyxacalles gibraltarensis<br>Stüben, 2002	GU987832 [01]<br>E-0206-gib<br>658nt	ZFMK-TIS-cE0206<br>ZFMK-DNA-0100400318	Spain, Cadiz, 10 km SW of Algeciras, El Bujeo, N36°04'10" W05°31'48", 257m, 12-Apr-2007, *Quercus suber*, leg. Astrin,J. & Stüben,P., det. Stüben,P.
Onyxacalles luigionii<br>(A. & F. Solari, 1907)	GU987967 [01]<br>I-622-lui<br>618nt	ZFMK-TIS-cI622<br>ZFMK-DNA-0100404292	Italy, Campania, Cilento, 6 km SE of Vallo d. Lucania, M. Sacro o Gelbison, N40°12'41" E15°19'42", 1544m, 30-Jun-2008, *Fagus*, leg. Stüben,P., det. Stüben,P.
Onyxacalles luigionii<br>(A. & F. Solari, 1907)	GU987977 [01]<br>I-635-lui<br>620nt	ZFMK-TIS-cI635<br>ZFMK-DNA-0100404909	Italy, Campania, Monti Picentini, 9 km N of Acerno, Piano Laceno, N40°48'58" E15°07'35", 1210m, 03-Jul-2008, *Fagus*, leg. Stüben,P., det. Stüben,P.
Onyxacalles luigionii<br>(A. & F. Solari, 1907)	GU987979 [01]<br>I-638-lui<br>658nt	ZFMK-TIS-cI638<br>ZFMK-DNA-0100404912	Italy, Basilicata, Monte Pollino, 9 km SE of Rotonda, Rif. de Gasperi, N39°54'37" E16°07'15", 1486m, 05-Jul-2008, *Fagus*, leg. Stüben,P., det. Stüben,P.
Onyxacalles maginaensis<br>Stüben, 2004	EU286491 [01]<br>E-0169-mag<br>632nt	ZFMK-TIS-cE0169<br>ZFMK-DNA-0100400150	Spain, Andalucia, 28 km E of Jaen, Sierra Magina, N37°43'21" W03°29'11", 1600m, 06-Okt-2005, *Quercus ilex*, leg. Stüben,P., det. Stüben,P.
Onyxacalles neglectus<br>Kulbe, 1999	GU988014 [01]<br>E-713-neg<br>658nt	ZFMK-TIS-cE713<br>ZFMK-DNA-0100404831	Spain, Canary Islands, La Gomera, S of Hermigua, El Cedro, Las Mimbreras, N28°07'27" W17°13'26", 901m, 06-Okt-2008, *Laurisilva*, leg. Astrin,J. & Stüben,P., det. Stüben,P.
Onyxacalles nuraghi<br>Stüben, 2012<br>**Paratype (DNAtype)**	JN121399 [05]<br>IT1090c<br>658nt	ZFMK-TIS-cIT1090c<br>ZFMK-DNA-0112704584	Italy, Sardinia Isl. West, E of Macomer, above Lei, N40°19'54" E08°53'49", 1020m, 04-Okt-2010, *Quercus, Acer monspessulanum*, leg. Stüben,P., det. Stüben,P.
Onyxacalles nuraghi<br>Stüben, 2012<br>**Paratype (DNAtype)**	JN121400 [05]<br>IT1097c<br>658nt	ZFMK-TIS-cIT1097c	Italy, Sardinia Isl. West, E of Macomer, above Lei, N40°19'17" E08°53'52", 586m, 07-Okt-2010, *Quercus ilex*, leg. Stüben,P., det. Stüben,P.
Onyxacalles nuraghi<br>Stüben, 2012	MG229744 [new]<br>1789-PST<br>658nt	ZFMK-TIS-24113<br>ZFMK-DNA-0171624080	Italy, Sardinia Isl., near Seulo, N39°52'30" E09°14'01", 714m, 13-Mai-2014, mixed forest, sieving, leg. Stüben,P. & Schütte,A., det. Stüben,P.

**Name**<br>**Authority**<br>**Additional Information**	**GenBank Acc No (Ref.)**<br>**Specimen ID**<br>**Sequence Length**	**ZFMK Tissue ID**<br>**ZFMK DNA Sample ID**<br>**(SDEI DNA Sample ID)**	**Locality, GPS, Collection Date, Plant, Collector, Identifier**
Onyxacalles portusveneris<br>(Mayet, 1903)	EU286490 [01]<br>F-0166-por<br>658nt	ZFMK-TIS-cF0166<br>ZFMK-DNA-0100400160	France, Gard, 15 km NE of Nimes, Pont du Gard, Collias, N43°57'03" E04°28'59", 68m, 13-Apr-2006, *Quercus ilex*, moist forest, leg. Stüben,P., det. Stüben,P.
Onyxacalles portusveneris<br>(Mayet, 1903)	GU987922 [01]<br>M-482-mag<br>658nt	ZFMK-TIS-cM482<br>ZFMK-DNA-0100400583	Morocco, High Atlas, 59 km SE of Marrakech, N31°28'19" W07°24'22", 1500m, 28-Dez-2002, *Quercus ilex, Quercus suber*, leg. Stüben,P., det. Stüben,P.
Onyxacalles portusveneris<br>(Mayet, 1903)	GU988133 [01]<br>E-889-port<br>658nt	ZFMK-TIS-cE889<br>ZFMK-DNA-0100405502	Morocco, High Atlas, E of Marrakech, N of Taddert, (near Tazouguerte), N31°28'07" W07°24'59", 1727m, 03-Mai-2009, *Quercus*, leg. Stüben,P., det. Stüben,P.
Onyxacalles portusveneris<br>(Mayet, 1903)	GU988134 [01]<br>E-890-port<br>658nt	ZFMK-TIS-cE890<br>ZFMK-DNA-0100405501	Morocco, Middle Atlas, S of Azrou, Äin Leuh, N33°16'50" W05°20'18", 1582m, 05-Mai-2009, *Quercus ilex, Euphorbia*, leg. Stüben,P., det. Stüben,P.
Onyxacalles portusveneris<br>(Mayet, 1903)	GU988135 [01]<br>E-891-port<br>614nt	ZFMK-TIS-cE891<br>ZFMK-DNA-0100405500	Morocco, Middle Atlas, S of Azrou, S of Äin Leuh, N33°14'57" W05°21'04", 1715m, 05-Mai-2009, *Quercus ilex*, leg. Stüben,P., det. Stüben,P.
Onyxacalles portusveneris<br>(Mayet, 1903)	GU988147 [01]<br>E-912-port<br>658nt	ZFMK-TIS-cE912<br>ZFMK-DNA-0100405490	Morocco, Rif Mts., 10 km W of Ketama, N34°57'40" W04°40'51", 1600m, 10-Mai-2009, *Cedrus, Prunus*, leg. Stüben,P., det. Stüben,P.
Onyxacalles portusveneris<br>(Mayet, 1903)	KC783815 [new]<br>167-PST<br>658nt	ZFMK-TIS-2D100440261<br>ZFMK-DNA-0100438149	Morocco, Atlas Mts., S of Taza, Jebel Tazzeka near Bat-Bou-Idir, N34°05'02" W04°06'49", 1408m, 16-Mai-2011, *Quercus*, sieving, leg. Stüben,P., det. Stüben,P.
Onyxacalles portusveneris<br>(Mayet, 1903)	GU987950 [01]<br>E-602-mag<br>658nt	ZFMK-TIS-cE602<br>ZFMK-DNA-0100404941	Spain, Teruel, S. Javalambre, Fuente la Risca near Arcos de las Salinas, N39°59'56" W01°01'21", 1121m, 17-Jun-2008, *Amelanchier ovalis, Acer monspessulanum, Erinacea anthyllis, Ulex*, leg. Astrin,J., det. Stüben,P.
Onyxacalles portusveneris<br>(Mayet, 1903)	KJ867600 [new]<br>1276-PST<br>658nt	ZFMK-TIS-4092<br>ZFMK-DNA-0100426447	Spain, Malaga, NW of Otivar, Sierra del Chapparal, N36°49'32" W03°42'30", 653m, 08-Mai-2013, *Quercus ilex*, sieving, leg. Stüben,P. & Schütte,A., det. Stüben,P.
Onyxacalles portusveneris<br>(Mayet, 1903)	KJ867607 [new]<br>1294-PST<br>658nt	ZFMK-TIS-4110<br>ZFMK-DNA-0100426182	Spain, Granada, NE of Granada, Sierra de La Yedra, N37°15'50" W03°32'13", 1414m, 13-Mai-2013, *Quercus ilex*, sieving, leg. Stüben,P. & Schütte,A., det. Stüben,P.
Onyxacalles portusveneris<br>(Mayet, 1903)	MG322640 [new]<br>E-0187-mag<br>631nt	ZFMK-TIS-cE0187<br>ZFMK-DNA-0100400320	Spain, Almeria, 11 km NW of Laujar de Andarax, Sierra Nevada, Bayarcal, N37°02'27" W03°00'12", 1291m, 07-Apr-2007, *Quercus ilex*, leg. Astrin,J. & Stüben,P., det. Stüben,P.
Onyxacalles ringeli<br>Kulbe, 1999	EU286465 [01]<br>C-0171-rin<br>658nt	ZFMK-TIS-cC0171<br>ZFMK-DNA-0100400679	Spain, Canary Islands, La Palma, Cumbre Nueva, 4.5 km SE of El Paso, El Pilar, N28°37'37" W17°49'45", 1432m, 07-Jul-2006, *Laurisilva*, leg. Stüben,P., det. Stüben,P.
Onyxacalles ringeli<br>Kulbe, 1999	KY211662 [40]<br>1755-PST<br>658nt	ZFMK-TIS-24473<br>ZFMK-DNA-0155622440	Spain, Canary Islands, La Palma, SW of Mazo, N28°35'53" W17°48'34", 1242m, 21-Jan-2014, *Laurisilva*, beating, leg. Stüben,P., det. Stüben,P.
Onyxacalles ringeli<br>Kulbe, 1999	KY211663 [40]<br>1982-JKR<br>658nt	ZFMK-TIS-23622<br>ZFMK-DNA-0169170519	Spain, Canary Islands, La Palma, Pared Vieja, N28°37'21" W17°49'21", 1163m, 07-Feb-2014, *Laurisilva*, beating, leg. Krátky,J., det. Krátky,J.
Onyxacalles sp.	MK347583 [new]<br>3022-PST<br>658nt	(SDEI-DNA-3022-PST)	Georgia, Bakuriani, N41°43'09" E43°31'41", 1912m, 23-Jul-2014, leg. Konvicka, det. Stüben,P.

**Name**<br>**Authority**<br>**Additional Information**	**GenBank Acc No (Ref.)**<br>**Specimen ID**<br>**Sequence Length**	**ZFMK Tissue ID**<br>**ZFMK DNA Sample ID**<br>**(SDEI DNA Sample ID)**	**Locality, GPS, Collection Date, Plant, Collector, Identifier**
Onyxacalles valencianus<br>Germann, 2005	EU286495 [01]<br>E-0180-val<br>658nt	ZFMK-TIS-cE0180<br>ZFMK-DNA-0100400125	Spain, Alicante, 7 km SW of Alcoi, Sierra de Menechaor, Santuario de la Font Roja, N38°39'34" W00°32'29", 296m, 05-Apr-2007, *Quercus ilex*, limestone, leg. Astrin,J. & Stüben,P., det. Stüben,P.
Onyxacalles valencianus<br>Germann, 2005	GU987953 [01]<br>E-607-val<br>608nt	ZFMK-TIS-cE607<br>ZFMK-DNA-0100404312	Spain, Castellon, Morella, Barranco de la Bota, N40°33'12" W00°00'27", 814m, 21-Jun-2008, *Quercus ilex, Hedera helix*, leg. Astrin,J., det. Stüben,P.
Onyxacalles valencianus<br>Germann, 2005	GU987958 [01]<br>E-612-val<br>658nt	ZFMK-TIS-cE612<br>ZFMK-DNA-0100404934	Spain, Barcelona, near Vallirana, N41°22'36" E01°55'02", 245m, 22-Jun-2008, *Quercus ilex, Ficus carica, Smilax aspera* , above dry river bed, leg. Astrin,J., det. Stüben,P.
Onyxacalles valencianus<br>Germann, 2005	GU987960 [01]<br>E-614-val<br>627nt	ZFMK-TIS-cE614<br>ZFMK-DNA-0100404936	Spain, Barcelona, S. Montseny, Tordera valley near St. Marçal, N41°48'01" E02°25'15", 1060m, 23-Jun-2008, very diverse mixed forest, leg. Astrin,J., det. Stüben,P.
Onyxacalles verrucosus<br>(Wollaston, 1863)	EU286488 [01]<br>C-0170-ver<br>658nt	ZFMK-TIS-cC0170<br>ZFMK-DNA-0100400153	Spain, Canary Islands, El Hierro, 7 km W of La Frontera, Pista Derrabado, N27°44'29" W18°03'24", 895m, 30-Dez-2006, *Laurus azorica*, leg. Stüben,P., det. Stüben,P.
Onyxacalles verrucosus<br>(Wollaston, 1863)	GU987937 [01]<br>E-570-ver<br>658nt	ZFMK-TIS-cE570<br>ZFMK-DNA-0100404775	Spain, Canary Islands, Tenerife, 6 km N of La Laguna, Monte de las Mercedes, N28°31'50" W16°17'09", 950m, 29-Dez-2003, *Laurisilva*, deadwood, leg. Stüben,P., det. Stüben,P.
Onyxacalles verrucosus<br>(Wollaston, 1863)	GU987938 [01]<br>E-571-ver<br>658nt	ZFMK-TIS-cE571<br>ZFMK-DNA-0100404972	Spain, Canary Islands, Tenerife, 4 km S of Los Silos, Teno Mts., Monte del Aqua, N28°19'20" W16°49'14", 700m, 30-Dez-2003, *Laurisilva*, deadwood, leg. Stüben,P., det. Stüben,P.
Onyxacalles verrucosus<br>(Wollaston, 1863)	GU987995 [01]<br>E-677-ver<br>658nt	ZFMK-TIS-cE677<br>ZFMK-DNA-0100404878	Spain, Canary Islands, Tenerife, NE of La Laguna, Anaga Mts. near Moquinal, N28°31'55" W16°17'24", 840m, 29-Sep-2008, *Laurisilva*, leg. Astrin,J. & Stüben,P., det. Stüben,P.
Onyxacalles verrucosus<br>(Wollaston, 1863)	GU987996 [01]<br>E-678-ver<br>658nt	ZFMK-TIS-cE678<br>ZFMK-DNA-0100404877	Spain, Canary Islands, Tenerife, NE of La Laguna, Anaga Mts. near Chinobre, N28°33'21" W16°10'46", 808m, 30-Sep-2008, *Laurus, Ixanthus viscosus*, leg. Astrin,J. & Stüben,P., det. Stüben,P.
Onyxacalles verrucosus<br>(Wollaston, 1863)	GU988085 [01]<br>E-813-ver<br>632nt	ZFMK-TIS-cE813<br>ZFMK-DNA-0100404537	Spain, Canary Islands, Tenerife, SW of Los Silos, Teno Mts., Monte del Agua, Chupadero, N28°19'23" W16°49'12", 940m, 02-Okt-2008, *Laurus novocanariensis*, leg. Astrin,J.,Stüben,P.,Behne,L.,Floren,L., det. Stüben,P.
Onyxacalles vilae<br>Stüben, 2012<br>**Paratype (DNAtype)**	EU286512 [01]<br>HR-0339-lui<br>658nt	ZFMK-TIS-cHR0339<br>ZFMK-DNA-0100400390	Croatia, Dalmatia, 8 km E of Karlobag, Velebit Mts., Stupacinovo, N44°32'41" E15°09'58", 1049m, 14-Jul-2007, *Fagus*, limestone, leg. Stüben,P., det. Stüben,P.
Onyxacalles vilae<br>Stüben, 2012	EU286451 [01]<br>I-0027-lui<br>658nt	ZFMK-TIS-cI0027<br>ZFMK-DNA-0100400750	France, Isere, 2 km SE of Lans en Vercors, Montagne de Lans, N45°06'45" E05°36'21", 1352m, 12-Aug-2005, *Abies, Fagus, Fraxinus*, limestone: mixed forest, leg. Stüben,P., det. Stüben,P.
Onyxacalles vilae<br>Stüben, 2012	KC783807 [new]<br>105-PST<br>604nt	ZFMK-TIS-2D100440322<br>ZFMK-DNA-0100438210	France, Isere, NW of Lans en Vercors near Autrars, Parc Regional du Vercors, N45°14'12" E5°34'58", 1370m, 23-Jul-2011, *Fagus*, beating, leg. Stüben,P., det. Stüben,P.
Onyxacalles vilae<br>Stüben, 2012	KC783820 [new]<br>463-PST<br>658nt	ZFMK-TIS-20249<br>ZFMK-DNA-0100449385	Italy, Como, Vallassina, dintorni Barni, N45°54' E09°16', 800m, 23-Feb-2011, leg. Diotti,L., det. Stüben,P.
Oprohinus consputus<br>(Germar, 1824)	MK891210 [new]<br>1128-JKR<br>658nt	ZFMK-TIS-3464<br>ZFMK-DNA-0100449580	Slovakia, Nove Zamky, Kamenin, N47°51'51" E18°38'09", 110m, 11-Mai-2012, *Alium* sp., sweeping, leg. Krátky,J., det. Krátky,J.

**Name**<br>**Authority**<br>**Additional Information**	**GenBank Acc No (Ref.)**<br>**Specimen ID**<br>**Sequence Length**	**ZFMK Tissue ID**<br>**ZFMK DNA Sample ID**<br>**(SDEI DNA Sample ID)**	**Locality, GPS, Collection Date, Plant, Collector, Identifier**
Oprohinus suturalis<br>(Fabricius, 1775)	MK891513 [new]<br>1599-JKR<br>658nt	ZFMK-TIS-3647<br>ZFMK-DNA-0155635630	Czech Republic, Moravia, Cejkovice - Spidlaky, N48°54'57" E16°57'44", 210m, 01-Mai-2013, sweeping, leg. Krátky,J., det. Krátky,J.
Orchestes alni<br>(Linnaeus, 1758)	KC784164 [new]<br>597-PSP<br>658nt	ZFMK-TIS-20380<br>ZFMK-DNA-0100448670	Germany, Lower Saxony (NI), Bad Zwischenahn, Aschhausen, N53°12'00" E08°04'00", 11m, 10-Jun-2011, *Ulmus minor 'Wredei'*, beating, leg. Sprick,P., det. Sprick,P.
Orchestes avellanae<br>(Donovan, 1797)	MK892189 [new]<br>2484-PSP<br>658nt	ZFMK-TIS-23456<br>ZFMK-DNA-0171661789	Germany, Lower Saxony (NI), Hannover region, Brelingen, N52°33'52" E09°41'09", 75m, 18-Apr-2014, *Quercus robur*, beating, leg. Sprick,P., det. Sprick,P.
Orchestes avellanae<br>(Donovan, 1797)	MK891770 [new]<br>2027-JKR<br>658nt	ZFMK-TIS-23667<br>ZFMK-DNA-0169170474	Italy, Sicilia Isl. (PA), Palermo, Bosco di Ficuzza, N37°51'50" E13°24'56", 995m, 22-Apr-2014, *Quercus*, beating, leg. Krátky,J., det. Krátky,J.
Orchestes calceatus calceatus<br>(O. F. Müller, 1776)	MK891579 [new]<br>353-JKR<br>658nt	ZFMK-TIS-20139<br>ZFMK-DNA-0155630459	Czech Republic, Bohemia or., Borohradek env., Nova Ves , N50°3'24.032" E16°9'14.372", 240m, 30-Mai-2011, *Betula pubescens*, beating, leg. Krátky,J., det. Krátky,J.
Orchestes fagi<br>(Linnaeus, 1758)	MK891091 [40]<br>995-PSP<br>658nt	ZFMK-TIS-3140<br>ZFMK-DNA-0100426873	Germany, Lower Saxony (NI), Diekholzen, Lkr. Hildesheim, N52°04'42" E09°56'32", 204m, 01-Mai-2012, *Fagus sylvatica*, forest of Hildesheim, beating, leg. Sprick,P., det. Sprick,P.
Orchestes hirtellus<br>(Miller, 1862)	MK891292 [new]<br>1251-FBA<br>658nt	ZFMK-TIS-3587<br>ZFMK-DNA-0100426024	Greece, Peloponnese, Messinia, W of Taygetos Mts., E of Saidona, N36°53'01" E22°17'07", 680m, 23-Mai-2011, leg. Bahr,F., det. Bayer,B.
Orchestes irroratus irroratus<br>Kiesenwetter, 1852	KC784276 [new]<br>IT-0050w<br>652nt	ZFMK-DNA-0100417915	Italy, Sardinia Isl. South, E of Cagliari, national park M. d. Sette Fratelli, Mt. Funtana-Ona, N39°17'30" E09°23'17", 596m, 03-Okt-2010, *Quercus, Arbutus, Pistacia*, leg. Stüben,P., det. Stüben,P.
Orchestes irroratus irroratus<br>Kiesenwetter, 1852	KC783855 [new]<br>169-PST<br>658nt	ZFMK-TIS-2D100440263<br>ZFMK-DNA-0100438151	Morocco, Atlas Mts., S of Taza, Jbel Tazzeka near Bat-Bou-Idir, N34°03'29" W04°10'21", 1598m, 16-Mai-2011, *Quercus*, beating, leg. Stüben,P., det. Stüben,P./vid. Behne,L.
Orchestes jota<br>(Fabricius, 1787)	KC783997 [new]<br>267-PSP<br>658nt	ZFMK-TIS-2D100439113<br>ZFMK-DNA-0100439785	Germany, Lower Saxony (NI), Helstorfer Moor, Resse, Hannover region, N52°31'44" E09°36'32", 43m, 14-Mai-2011, *Betula pubescens*, bog forest, beating, leg. Sprick,P., det. Sprick,P.
Orchestes pilosus<br>(Fabricius, 1781)	MK892081 [new]<br>2357-JKR<br>658nt	ZFMK-TIS-25917<br>ZFMK-DNA-0171600584	Czech Republic, Moravia, NP Podyji, Cizov, Siroke pole, N48°51'36" E15°51'02", 300m, 17-Aug-2014, *Quercus robur*, beating, leg. R. Stejskal, det. Stejskal,R.
Orchestes pilosus<br>(Fabricius, 1781)	MK891080 [new]<br>983-PSP<br>658nt	ZFMK-TIS-3128<br>ZFMK-DNA-0100426876	Germany, Lower Saxony (NI), near Hannover, Brelingen, N52°33'35" E09°41'04", 64m, 16-Apr-2012, *Quercus robur*, group of oak trees, beating, leg. Sprick,P., det. Sprick,P.
Orchestes pilosus<br>(Fabricius, 1781)	MK891306 [new]<br>1291-PST<br>658nt	ZFMK-TIS-4107<br>ZFMK-DNA-0100426185	Spain, Community of Madrid, SW of Lozoya, Pinilla del Valle, N40°55'44" W03°49'29", 1108m, 03-Mai-2013, *Quercus*, beating, leg. Stüben,P. & Schütte,A., det. Stüben,P.
Orchestes quedenfeldtii<br>Gerhardt, 1865	MK891974 [new]<br>2245-JKR<br>658nt	ZFMK-TIS-23983<br>ZFMK-DNA-0169170542	Slovakia, Galanta, Vinohrady nad Vahom, N48°21'15" E17°45'24.5", 140m, 14-Jun-2014, *Ulmus minor*, beating, leg. Benedikt,S., det. Benedikt,S.
Orchestes quercus<br>(Linnaeus, 1758)	KC784024 [new]<br>216-PSP<br>658nt	ZFMK-TIS-2D100439158<br>ZFMK-DNA-0100439830	Germany, Lower Saxony (NI), Brelingen, Hannover region, N52°33'35" E09°41'04", 70m, 16-Apr-2011, *Quercus robur*, sand pit, beating, leg. Sprick,P., det. Sprick,P.

**Name**<br>**Authority**<br>**Additional Information**	**GenBank Acc No (Ref.)**<br>**Specimen ID**<br>**Sequence Length**	**ZFMK Tissue ID**<br>**ZFMK DNA Sample ID**<br>**(SDEI DNA Sample ID)**	**Locality, GPS, Collection Date, Plant, Collector, Identifier**
Orchestes rufus<br>Olivier, 1807	KC784154 [new]<br>613-PSP<br>658nt	ZFMK-TIS-20396<br>ZFMK-DNA-0100448654	Germany, Lower Saxony (NI), Bad Zwischenahn-Aschhausen, N53°12'17" E08°04'11", 14m, 10-Jun-2011, *Ulmus minor 'Wredei'*, beating, leg. Sprick,P., det. Sprick,P.
Orchestes rusci<br>(Herbst, 1795)	MK891442 [new]<br>1505-PSP<br>658nt	ZFMK-TIS-3265<br>ZFMK-DNA-0155633324	Germany, Schleswig-Holstein (SH), Ellhöft, Lkr. Nordfriesland, "Schwarzberger Moor", N54°52'45" E08°59'10", 9m, 28-Aug-2012, *Betula pendula*, peatland, beating, leg. Sprick,P., det. Sprick,P.
Orchestes testaceus<br>(O. F. Müller, 1776)	KC783925 [new]<br>352-JKR<br>624nt	ZFMK-TIS-20138<br>ZFMK-DNA-0100438731	Czech Republic, Bohemia or., Borohradek env., Nova Ves , N50°3'24.032" E16°9'14.372", 240m, 30-Mai-2011, *Alnus glutinosa*, beating, leg. Krátky,J., det. Krátky,J.
Orchestes testaceus<br>(O. F. Müller, 1776)	MK891428 [new]<br>1491-PSP<br>658nt	ZFMK-TIS-3251<br>ZFMK-DNA-0155633329	Germany, Lower Saxony (NI), Harz National Park, Eckertal valley, Carici remotae-Fraxinetum, N51°53'13" E10°37'33", 380m, 01-Aug-2012, *Alnus glutinosa*, beating, leg. Sprick,P., det. Sprick,P.
Orchestes testaceus<br>(O. F. Müller, 1776)	MK891438 [new]<br>1501-PSP<br>658nt	ZFMK-TIS-3261<br>ZFMK-DNA-0155629928	Germany, Lower Saxony (NI), Meitze - Hellendorf, Hannover region, N52°33'39" E09°44'36", 50m, 03-Aug-2012, *Alnus glutinosa*, beating, leg. Sprick,P., det. Sprick,P.
Orchestes testaceus<br>(O. F. Müller, 1776)	MK891973 [new]<br>2244-JKR<br>658nt	ZFMK-TIS-23982<br>ZFMK-DNA-0169170543	Slovakia, Zilina, Rajecke Teplice, N49°07'14" E18°41'19", 434m, 21-Jun-2014, *Alnus glutinosa*, sweeping, leg. Krátky,J., det. Krátky,J.
Oreorrhynchaeus alpicola<br>Otto, 1894	MK892021 [new]<br>2294-JKR<br>658nt	ZFMK-TIS-24225<br>ZFMK-DNA-0169170960	Austria, Lower Austria, Schneeberg Mt., N47°45'25" E15°49'45", 1779m, 29-Jun-2014, *Heliosperma tetraphyllum*, sieving, leg. Krátky,J., det. Krátky,J.
Orthochaetes hirticulus<br>A. Hoffmann, 1963	MK891328 [new]<br>1349-PST<br>658nt	ZFMK-TIS-4165<br>ZFMK-DNA-0100425391	Spain, Granada, E of Baul, Sierra de Baza, Santa Barbara, N37°22'25" W02°51'03", 2023m, 17-Mai-2013, *Bupleurum, (Astragalus* sp.*)*, sieving, leg. Stüben,P. & Schütte,A., det. Sprick,P.
Orthochaetes insignis<br>(Aubé, 1863)	MK890960 [new]<br>840-PST<br>658nt	ZFMK-TIS-3753<br>ZFMK-DNA-0100414251	Portugal, Estremadura, Sao Martinho do Porto, N39°30'06" W09°08'30", 11m, 15-Mai-2012, sieving, leg. Stüben,P., det. Stüben,P.
Oryxolaemus flavifemoratus<br>(Herbst, 1797)	MK891463 [new]<br>1530-PSP<br>628nt	ZFMK-TIS-3290<br>ZFMK-DNA-0155633299	Germany, Lower Saxony (NI), Wesseln, Lkr. Hildesheim, Steinberg National Park, N52°05'04" E10°01'00", 191m, 20-Okt-2012, *Genista tinctoria*, dipnet, leg. Sprick,P., det. Sprick,P.
Oryxolaemus scabiosus<br>(Weise, 1889)	KC784268 [new]<br>IT-0041w<br>655nt	ZFMK-DNA-0100437838	Italy, Sardinia Isl. East, Oliena, N40°16'23" E09°24'20", 966m, 05-Okt-2010, garden, leg. Stüben,P., det. Stüben,P.
Otiorhynchus allardi<br>Stierlin, 1872	KC783852 [new]<br>163-PST<br>658nt	ZFMK-TIS-2D100440257<br>ZFMK-DNA-0100438145	Morocco, S of Berkane, Mts. des Beni Snassen, Vallee Zegzel, N34°49'19" W02°23'10" , 644m, 15-Mai-2011, beating, leg. Stüben,P., det. Stüben,P./vid. Behne,L.
Otiorhynchus alpestris<br>(Comolli, 1837)	MK891996 [new]<br>2267-JKR<br>658nt	ZFMK-TIS-24197<br>ZFMK-DNA-0169170999	Austria, Styria, Hochschwab, Spitzkögel, N47°35'27" E15°01'26", 1659m, 27-Jun-2014, alpine grassland, collecting by hand, leg. Krátky,J., det. Krátky,J.
Otiorhynchus arcticus arcticus<br>(O. Fabricius, 1780)	KC784214 [new]<br>427-RGO<br>658nt	ZFMK-TIS-20213<br>ZFMK-DNA-0100449412	Poland, Czerwone Wierchy, Tatra Mountains, N49°16'58.14" E19°52'16.08", 1393m, 06-Jul-2011, leg. Gosik,R., det. Gosik,R.
Otiorhynchus arcticus<br>(O. Fabricius, 1780)	MK891978 [new]<br>2249-JKR<br>658nt	ZFMK-TIS-23987<br>ZFMK-DNA-0169170529	Slovakia, Poprad, Vysoke Tatry Mts., Lomnicky stit mt., N49°11'33.5" E20°13'29", 1903m, 23-Jun-2014, leg. Benedikt,S., det. Benedikt,S.

**Name**<br>**Authority**<br>**Additional Information**	**GenBank Acc No (Ref.)**<br>**Specimen ID**<br>**Sequence Length**	**ZFMK Tissue ID**<br>**ZFMK DNA Sample ID**<br>**(SDEI DNA Sample ID)**	**Locality, GPS, Collection Date, Plant, Collector, Identifier**
Otiorhynchus armadillo<br>(Rossi, 1792)	MK891948 [new]<br>2216-JKR<br>658nt	ZFMK-TIS-23954<br>ZFMK-DNA-0169170571	Czech Republic, Moravia, Olomouc, Tabulovy vrch, N49°35'12" E17°13'16", 257m, 04-Sep-2014, beating, leg. Bednarik,M., det. Bednarik,M.
Otiorhynchus armadillo<br>(Rossi, 1792)	KC783906 [new]<br>234-PSP<br>645nt	ZFMK-TIS-2D100439128<br>ZFMK-DNA-0100438554	Germany, Hesse (HE), Wiesbaden-Erbenheim, N50°03'00" E08°18'00, 274m, 27-Apr-2011, *Thuja occidentalis*, garden, beating, leg. Sprick,P., det. Sprick,P.
Otiorhynchus armadillo<br>(Rossi, 1792)	KC783980 [new]<br>207-PSP<br>658nt	ZFMK-TIS-2D100438563<br>ZFMK-DNA-0100439547	Germany, Baden-Wuerttemberg (BW), Mühlacker, N48°56'26" E08°50'33", 333m, 16-Jun-2010, *Euonymus fortunei*, beating, leg. Sprick,P., det. Sprick,P.
Otiorhynchus armatus<br>Boheman, 1843	MK892169 [new]<br>2460-PSP<br>658nt	ZFMK-TIS-23432<br>ZFMK-DNA-0171661813	Italy, Tyrol South, Meran, N46°39'59" E11°10'14", 347m, 15-Mai-2013, *Hedera helix*, beating, leg. K. Schrameyer, det. Sprick,P.
Otiorhynchus aurifer<br>Boheman, 1842	KC784162 [new]<br>612-PSP<br>658nt	ZFMK-TIS-20395<br>ZFMK-DNA-0100448666	Germany, Baden-Wuerttemberg (BW), Heilbronn, Alter Kaiser, N49°07'57" E09°13'29", 178m, 21-Jun-2011, *Viburnum tinus*, beating, leg. Schrameyer,K./Sprick,P., det. Sprick,P.
Otiorhynchus aurifer<br>Boheman, 1842	KC784269 [new]<br>IT-0042w<br>658nt	ZFMK-DNA-0100437839	Italy, Sardinia Isl. East, N of Dorgali near G.ta di Ispinigoli, N40°19'12" E09°36'00", 241m, 26-Sep-2010, *Quercus ilex, Pistacia*, cliff, limestone, leg. Stüben,P., det. Stüben,P.
Otiorhynchus balcanicus<br>Stierlin, 1861	MK891951 [new]<br>2219-JKR<br>658nt	ZFMK-TIS-23957<br>ZFMK-DNA-0169170568	Slovakia, Nove Zamky, Kovacov, NPR Skaly, N47°49'22" E18°45'41", 111m, 10-Sep-2014, beating, leg. Bednarik,M., det. Bednarik,M.
Otiorhynchus bisulcatus<br>(Fabricius, 1781)	MK892015 [new]<br>2288-JKR<br>658nt	ZFMK-TIS-24219<br>ZFMK-DNA-0169170977	Austria, Styria, Trofaiach, Rötz, N47°26'56" E15°01'39", 758m, 28-Jun-2014, mountain forest, sweeping, leg. Krátky,J., det. Krátky,J.
Otiorhynchus cf. *spinipennis*	KC783888 [new]<br>188-PST<br>658nt	ZFMK-TIS-2D100440234<br>ZFMK-DNA-0100438342	Morocco, Atlas Mts., N of Imouzzer-des-Marmoucha, Jbel Bou Iblane, Tizi-Bou-Zabel, N33°38'44" W04°09'18", 2275m, 21-Mai-2011, broom*, Bupleurum, Astragalus*, sieving, leg. Stüben,P., det. Behne,L.
Otiorhynchus chrysostictus<br>Gyllenhal, 1834	KC784207 [new]<br>431-RGO<br>633nt	ZFMK-TIS-20217<br>ZFMK-DNA-0100449401	Poland, Tarnogóra, N50°41'11.6" E23°07'13.4", 249m, 11-Jun-2011, leg. Gosik,R., det. Gosik,R.
Otiorhynchus coecus<br>Germar, 1824	KC784042 [new]<br>621-PSP<br>658nt	ZFMK-TIS-20404<br>ZFMK-DNA-0100448257	Germany, Lower Saxony (NI), Harz Mts., 2.5 km N of Hohegeiß, N51°41'02'' E10°39'16'', 581m, 23-Jul-2011, *Picea abies, Vaccinium myrtillus*, coniferous forest, beating, leg. Sprick,P., det. Sprick,P.
Otiorhynchus coecus<br>Germar, 1824	KC784217 [new]<br>424-RGO<br>658nt	ZFMK-TIS-20210<br>ZFMK-DNA-0100449415	Poland, Kiry, Tatra Mountains, N49°16'26" E19°52'07", 942m, 06-Jul-2011, leg. Gosik,R., det. Gosik,R.
Otiorhynchus coecus<br>Germar, 1824	MK892031 [new]<br>2307-JKR<br>658nt	ZFMK-TIS-24238<br>ZFMK-DNA-0169170958	Slovakia, Tatry Mts., Bujaci vrch, N49°14'10" E20°14'27", 1914m, 06-Jul-2014, alpine grassland, sieving, leg. Krátky,J., det. Krátky,J.
Otiorhynchus coecus<br>Germar, 1824	MK892037 [new]<br>2313-JKR<br>658nt	ZFMK-TIS-24244<br>ZFMK-DNA-0169170952	Slovakia, Tatry Mts., Dolina Siedmich prame?ov, N49°12'57" E20°17'46", 981m, 05-Jul-2014, mountain meadow, sweeping, leg. Krátky,J., det. Krátky,J.
Otiorhynchus corvus<br>Boheman, 1842	MK891371 [new]<br>1422-JKR<br>658nt	ZFMK-TIS-4191<br>ZFMK-DNA-0155628508	Slovakia, Vysoke Tatry Mts., Dolina siedmich prame?ov, N49°13'38" E20°16'34", 1448m, 30-Jun-2013, sweeping, leg. Krátky,J., det. Krátky,J.

**Name**<br>**Authority**<br>**Additional Information**	**GenBank Acc No (Ref.)**<br>**Specimen ID**<br>**Sequence Length**	**ZFMK Tissue ID**<br>**ZFMK DNA Sample ID**<br>**(SDEI DNA Sample ID)**	**Locality, GPS, Collection Date, Plant, Collector, Identifier**
Otiorhynchus crataegi<br>Germar, 1824	MK890888 [new]<br>553-RST<br>658nt	ZFMK-TIS-20338<br>ZFMK-DNA-0100446685	Czech Republic, Moravia mer., Znojmo, 0.8 km NE of Konice, N48°50'6.17" E16°2'3.34", 270m, 29-Sep-2011, *Ligustrum*, sieving, leg. Stejskal,R., det. Stejskal,R.
Otiorhynchus crataegi<br>Germar, 1824	MK891333 [new]<br>593-PSP<br>656nt	ZFMK-TIS-20377<br>ZFMK-DNA-0100417594	Germany, Lower Saxony (NI), Bad Zwischenahn, Aschhausen, N53°12'00" E08°04'00", 11m, 10-Jun-2011, *Taxus baccata*, beating, leg. Sprick,P., det. Sprick,P.
Otiorhynchus crataegi<br>Germar, 1824	MK891461 [new]<br>1528-PSP<br>658nt	ZFMK-TIS-3288<br>ZFMK-DNA-0155633301	Germany, Lower Saxony (NI), Hildesheim-Rottsberg, N52°08'25" E09°54'36", 200m, 26-Sep-2012, *Symphoricarpos albus*, hedge, beating, leg. Sprick,P., det. Sprick,P.
Otiorhynchus crataegi<br>Germar, 1824	MK891348 [new]<br>1387-PST<br>658nt	ZFMK-TIS-4683<br>ZFMK-DNA-0155628546	Italy, Abruzzo, P.N. Majella, Roccaraso near Valico d. Forchetta, Mad. dell Altare, N41°56'19" E14°07'09", 1240m, 22-Aug-2013, *wet mixed forest*, sieving, leg. Stüben,P., det. Stüben,P.
Otiorhynchus cribricollis<br>Gyllenhal, 1834	KC784161 [new]<br>611-PSP<br>658nt	ZFMK-TIS-20394<br>ZFMK-DNA-0100448665	Germany, Baden-Wuerttemberg (BW), Heilbronn, N49°07'24" E09°13'12", 191m, 14-Jun-2011, *Olea europaea*, beating, leg. Schrameyer,K., det. Sprick,P.
Otiorhynchus cribricollis<br>Gyllenhal, 1834	MK891257 [new]<br>1191-JKR<br>658nt	ZFMK-TIS-3527<br>ZFMK-DNA-0100426093	Spain, Andalucia, Huescar, El Salado env., N37°53'42" W02°37'33", 1149m, 14-Jun-2012, night sweeping, leg. Krátky,J., det. Krátky,J.
Otiorhynchus dieckmanni<br>Magnano, 1979	KC784134 [new]<br>645-PSP<br>645nt	ZFMK-TIS-20428<br>ZFMK-DNA-0100448622	Germany, Lower Saxony (NI), Braunschweig, Riddagshausen, N52°16'38" E10°33'58", 81m, 21-Sep-2011, *Lonicera pileata*, beating, leg. Sprick,P., det. Sprick,P.
Otiorhynchus dieckmanni<br>Magnano, 1979	MK890815 [new]<br>199-PSP<br>658nt	ZFMK-TIS-2D100439172<br>ZFMK-DNA-0100439844	Germany, Lower Saxony (NI), Hannover, Steintormasch, N52°22'57" E09°42'31", 50m, 06-Apr-2011, *Thuja occidentalis*, hedge, beating, leg. Sprick,P., det. Sprick,P.
Otiorhynchus equestris<br>(Richter, 1820)	MK892067 [new]<br>2343-JKR<br>658nt	ZFMK-TIS-24274<br>ZFMK-DNA-0169170913	Czech Republic, Bohemia, Horni Morava, N50°10'15" E16°49'22", 780m, 17-Jul-2014, sweeping, leg. Krátky,J., det. Krátky,J.
Otiorhynchus fullo<br>(Schrank, 1781)	KC783945 [new]<br>319-JKR<br>658nt	ZFMK-TIS-20105<br>ZFMK-DNA-0100438764	Czech Republic, Bohemia or., Dvakacovice, N49°58'35.447" E15°54'2.243", 245m, 14-Mai-2011, sweeping, leg. Krátky,J., det. Krátky,J.
Otiorhynchus fullo<br>(Schrank, 1781)	MK891920 [new]<br>2188-JKR<br>658nt	ZFMK-TIS-23926<br>ZFMK-DNA-0169170599	Czech Republic, Moravia, Znojmo, N48°51'32" E16°04'28.5", 255m, 25-Apr-2014, *shrubs*, beating, leg. Stejskal,R., det. Stejskal,R.
Otiorhynchus fullo<br>(Schrank, 1781)	MK891467 [new]<br>1534-PSP<br>658nt	ZFMK-TIS-3294<br>ZFMK-DNA-0155633295	Germany, Thuringia (TH), Erfurt, Großfahner, "Fahnersche Höhe", N51°03'50" E10°47'05", 247m, 04-Jun-2013, *Malus domestica*, beating, leg. Maring,E., det. Sprick,P.
Otiorhynchus fullo<br>(Schrank, 1781)	KC784218 [new]<br>421-RGO<br>658nt	ZFMK-TIS-20207<br>ZFMK-DNA-0100449418	Poland, Tarnogóra, N50°41'11.6" E23°07'13.4", 249m, 11-Jun-2011, leg. Gosik,R., det. Gosik,R.
Otiorhynchus gemmatus<br>(Scopoli, 1763)	MK892010 [new]<br>2283-JKR<br>658nt	ZFMK-TIS-24214<br>ZFMK-DNA-0169170982	Austria, Styria, Hochschwab, Stallmauer, N47°35'29" E15°02'20", 1260m, 27-Jun-2014, mountain forest, sweeping, leg. Krátky,J., det. Krátky,J.
Otiorhynchus krattereri<br>Boheman, 1842	MK891960 [new]<br>2229-JKR<br>658nt	ZFMK-TIS-23967<br>ZFMK-DNA-0169170557	Slovakia, Kezmarok, Vysoke Tatry Mts., Zadne Medodoly valley, N49°14'09" E20°12'15", 1509m, 24-Jun-2014, leg. Benedikt,S., det. Benedikt,S.

**Name**<br>**Authority**<br>**Additional Information**	**GenBank Acc No (Ref.)**<br>**Specimen ID**<br>**Sequence Length**	**ZFMK Tissue ID**<br>**ZFMK DNA Sample ID**<br>**(SDEI DNA Sample ID)**	**Locality, GPS, Collection Date, Plant, Collector, Identifier**
Otiorhynchus laevigatus<br>(Fabricius, 1792)	JX448422 [new]<br>278-PSP<br>658nt	ZFMK-TIS-2D100439093<br>ZFMK-DNA-0100439765	Germany, Lower Saxony (NI), Pattensen, Hannover region, N52°14'35" E09°45'21", 77m, 17-Mai-2011, *Prunus spinosa*, species-rich roadside, beating, leg. Sprick,P., det. Sprick,P.
Otiorhynchus lepidopterus<br>(Fabricius, 1794)	MK891994 [new]<br>2265-JKR<br>658nt	ZFMK-TIS-24195<br>ZFMK-DNA-0169171001	Austria, Styria, Hochschwab, Stallmauer, N47°35'29" E15°02'20", 1260m, 27-Jun-2014, mountain forest, sweeping, leg. Krátky,J., det. Krátky,J.
Otiorhynchus lepidopterus<br>(Fabricius, 1794)	MK892194 [new]<br>2489-PSP<br>658nt	ZFMK-TIS-23461<br>ZFMK-DNA-0171661779	Germany, Saxony-Anhalt (ST), Harz National Park, Drei Annen Hohne, Hohneklippen, Hohnekopf, N51°46'37" E10°41'55", 840m, 24-Apr-2014, *Picea abies*, beating, leg. Sprick,P., det. Sprick,P.
Otiorhynchus lepidopterus<br>(Fabricius, 1794)	MK892042 [new]<br>2318-JKR<br>658nt	ZFMK-TIS-24249<br>ZFMK-DNA-0169170936	Slovakia, Tatry Mts., Dolina Siedmich prame?ov, N49°13'30" E20°16'24", 1404m, 05-Jul-2014, *Salix*, collecting by hand, leg. Krátky,J., det. Krátky,J.
Otiorhynchus ligneus<br>(Olivier, 1807)	MK891457 [new]<br>1522-PSP<br>658nt	ZFMK-TIS-3282<br>ZFMK-DNA-0155633312	Denmark, Syddanmark, Emmerlev Klev (Højer), N54°59'53" E08°39'08", 3m, 30-Aug-2012, *Plantago maritima, Trifolium pratense*, leg. Sprick,P., det. Sprick,P.
Otiorhynchus ligneus<br>(Olivier, 1807)<br>formerly: *Otiorhynchus frisius*	MK891152 [new]<br>1059-PSP<br>658nt	ZFMK-TIS-3204<br>ZFMK-DNA-0100426793	Germany, Lower Saxony (NI), Langeoog, N53°45'00" E07°33'04", 1m, 16-Jun-2012, *Plantago maritima*, salt marsh, dipnet at night, leg. Sprick,P., det. Sprick,P.
Otiorhynchus ligustici<br>(Linnaeus, 1758)	MK891795 [new]<br>2054-JKR<br>658nt	ZFMK-TIS-23694<br>ZFMK-DNA-0169170447	Czech Republic, Bohemia, Kosatky, N50°19'23" E14°39'58", 218m, 02-Mai-2014, sieving, leg. Krátky,J., det. Krátky,J.
Otiorhynchus ligustici<br>(Linnaeus, 1758)	MK891904 [new]<br>2171-JKR<br>658nt	ZFMK-TIS-23909<br>ZFMK-DNA-0169170616	Czech Republic, Moravia, Cernin, N48°59'30.5" E16°00'55", 350m, 01-Mai-2014, collecting by hand, leg. Stejskal,R., det. Stejskal,R.
Otiorhynchus ligustici<br>(Linnaeus, 1758)	MK890818 [40]<br>204-PSP<br>658nt	ZFMK-TIS-2D100438890<br>ZFMK-DNA-0100439839	Germany, Brandenburg (BB), Kittlitz, Lkr. Cottbus, lucern field, N51°49'25" E13°55'32", 91m, 07-Apr-2011, *Medicago sativa*, collecting by hand, leg. Mathan,G., det. Sprick,P.
Otiorhynchus lugdunensis<br>Boheman 1842<br>formerly: *O. tenebricosus lugdunensis* - Resyn!	KC783905 [new]<br>201-PSP<br>658nt	ZFMK-TIS-2D100438906<br>ZFMK-DNA-0100438545	Germany, Lower Saxony (NI), Hannover, Linden, parque south of Leine river, N52°22'40" E09°42'30", 55m, 06-Apr-2011, *Euonymus fortunei*, beating, leg. Sprick,P., det. Sprick,P., Note: *Otiorhynchus lugdunensis* Boheman, 1843 was resynonymized in: Germann, C. (2011): Supplement zur Checkliste der Rüsselkäfer der Schweiz (Coleoptera, Curculionoidea) - *Mitteilungen der Schweizerischen Entomologischen Gesellschaft* 84: 155-169.
Otiorhynchus lugdunensis<br>Boheman, 1842	MK891466 [new]<br>1533-PSP<br>658nt	ZFMK-TIS-3293<br>ZFMK-DNA-0155633296	Germany, Schleswig-Holstein (SH), Bullenkuhlen, Lkr. Pinneberg, N53°46'23" E09°45'04", 7m, 04-Jun-2013, *Thuja plicata 'Aurescens'*, hedge, beating, leg. Sprick,P., det. Sprick,P.
Otiorhynchus lutosus<br>Stierlin, 1858	MK891338 [new]<br>1368-PST<br>658nt	ZFMK-TIS-4664<br>ZFMK-DNA-0155628565	Italy, Abruzzo, P.N. Majella, E of Sulmona near Pacentro, N42°03'13" E14°01'49", 983m, 16-Aug-2013, *Acer*, sieving, leg. Stüben,P., det. Sprick,P.
Otiorhynchus mandibularis<br>Redtenbacher, 1842	KC784094 [new]<br>525-RST<br>658nt	ZFMK-TIS-20310<br>ZFMK-DNA-0100448454	Romania, Caras-Severin, Sfanta Elena env., N44°40'54.68" E21°42'39.99", 400m, 26-Jul-2011, night-sweeping, leg. Stejskal,R., det. Stejskal,R.
Otiorhynchus meridionalis<br>Gyllenhal, 1834	KC783837 [new]<br>487-PST<br>658nt	ZFMK-TIS-20272<br>ZFMK-DNA-0100417919	France, Vaucluse, Bedoin, N44°07'42" E05°11'43", 345m, 30-Jul-2011, beating, leg. Stüben,P., det. Stüben,P.

**Name**<br>**Authority**<br>**Additional Information**	**GenBank Acc No (Ref.)**<br>**Specimen ID**<br>**Sequence Length**	**ZFMK Tissue ID**<br>**ZFMK DNA Sample ID**<br>**(SDEI DNA Sample ID)**	**Locality, GPS, Collection Date, Plant, Collector, Identifier**
Otiorhynchus meridionalis<br>Gyllenhal, 1834	MK890894 [new]<br>574-PSP<br>658nt	ZFMK-TIS-20359<br>ZFMK-DNA-0100448405	Germany, Baden-Wuerttemberg (BW), Heilbronn, Zentrum, N49°08'51" E09°13'26", 168m, 21-Jun-2011, *Forsythia x intermedia*, front garden, beating, leg. Schrameyer,K., det. Sprick,P.
Otiorhynchus morio<br>(Fabricius, 1781)	MK891576 [new]<br>241-PSP<br>658nt	ZFMK-TIS-2D100439135<br>ZFMK-DNA-0155630455	Germany, Lower Saxony (NI), Nordstemmen, Lkr. Hildesheim, N52°09' E09°47', 72m, 04-Mai-2011, *Centaurea dealbata*, beating, leg. Sprick,P., det. Sprick,P.
Otiorhynchus nodosus<br>(O. F. Müller, 1764)	MK892335 [new]<br>2637-PSP<br>658nt	ZFMK-TIS-23611<br>ZFMK-DNA-0171661929	Germany, Saxony-Anhalt (ST), Schierke, Drei Annen Hohne, Hohneklippen, N51°46'46" E10°41'34", 861m, 10-Jul-2014, *Vaccinium myrtillus*, dipnet, leg. Sprick,P., det. Sprick,P.
Otiorhynchus obtusus<br>Boheman, 1842	MK892034 [new]<br>2310-JKR<br>658nt	ZFMK-TIS-24241<br>ZFMK-DNA-0169170955	Slovakia, Tatry Mts., Bujaci vrch, N49°14'10" E20°14'27", 1914m, 06-Jul-2014, alpine grassland, sieving, leg. Krátky,J., det. Krátky,J.
Otiorhynchus ovatus<br>(Linne, 1758)	MK892113 [new]<br>2390-JKR<br>658nt	ZFMK-TIS-25967<br>ZFMK-DNA-0171600612	Czech Republic, Moravia, Znojmo, N48°51'05" E16°05'59", 250m, 16-Sep-2014, sweeping, leg. R. Stejskal, det. Stejskal,R.
Otiorhynchus ovatus<br>(Linnaeus, 1758)	KC784163 [new]<br>598-PSP<br>658nt	ZFMK-TIS-20381<br>ZFMK-DNA-0100448669	Germany, Lower Saxony (NI), Bad Zwischenahn, Aschhausen, N53°12'00" E08°04'00", 11m, 10-Jun-2011, *Thuja occidentalis, Prunus lusitanica*, beating, leg. Sprick,P., det. Sprick,P.
Otiorhynchus ovatus<br>(Linne, 1758)	MK891432 [new]<br>1495-PSP<br>652nt	ZFMK-TIS-3255<br>ZFMK-DNA-0155633333	Germany, Saxony-Anhalt (ST), Harz National Park, Eckertal, N51°52'41" E10°38'15", 286m, 01-Aug-2012, *Krautschicht*, heavy metal lawn ("Schwermetallrasen"), beating, leg. Sprick,P., det. Sprick,P.
Otiorhynchus ovatus<br>(Linnaeus, 1758)	MK891063 [new]<br>964-CBR<br>657nt	ZFMK-TIS-3877<br>ZFMK-DNA-0100449116	Luxembourg, 10 km NW of Esch/Alzette, 1 km NW of Niedercorn, N49°32'31" E05°52'39", 370m, 28-Jun-2012, sweeping, leg. Braunert,C., det. Braunert,C.
Otiorhynchus ovatus<br>(Linne, 1758)	MK891863 [new]<br>2126-JKR<br>658nt	ZFMK-TIS-23766<br>ZFMK-DNA-0169169607	Slovakia, Nitra, Zobor Mt., N48°21'08.5 E18°05'41", 427m, 24-Mai-2014, sieving, leg. Krátky,J., det. Krátky,J.
Otiorhynchus ovatus<br>(Linne, 1758)	MK892220 [40]<br>2516-PSP<br>658nt	ZFMK-TIS-23488<br>ZFMK-DNA-0171661758	Slovakia, Nitra, Zlatna na Ostrove, N47°45'34" E17°58'40", 110m, 17-Mai-2014, *herb layer*, leg. Sprick,P., det. Sprick,P.
Otiorhynchus parvicollis<br>Gyllenhal, 1834	KC783838 [new]<br>187-PST<br>658nt	ZFMK-TIS-2D100440233<br>ZFMK-DNA-0100438121	Morocco, Atlas Mts., N of Imouzzer-des-Marmoucha, Jbel Bou Iblane, Tizi-Bou-Zabel, N33°38'44" W04°09'18", 2275m, 21-Mai-2011, broom*, Bupleurum, Astragalus*, sieving, leg. Stüben,P., det. Behne,L.
Otiorhynchus pauxillus<br>Rosenhauer, 1847	MK892061 [new]<br>2337-JKR<br>658nt	ZFMK-TIS-24268<br>ZFMK-DNA-0169170928	Czech Republic, Bohemia, Horni Morava, N50°10'15" E16°49'22", 780m, 17-Jul-2014, sieving, leg. Krátky,J., det. Krátky,J.
Otiorhynchus pauxillus<br>Rosenhauer, 1847	MK891271 [new]<br>1215-JKR<br>658nt	ZFMK-TIS-3551<br>ZFMK-DNA-0100426069	Romania, Maramures, Rodna Mts., Gargalau Mt., N47°35'15" E24°48'27", 1549m, 07-Sep-2012, *Rhododendron myrtifolium*, sieving, leg. Krátky,J., det. Krátky,J.
Otiorhynchus pinastri<br>(Herbst, 1795)	KC784141 [new]<br>626-PSP<br>658nt	ZFMK-TIS-20409<br>ZFMK-DNA-0100448632	Germany, Bavaria (BY), Thüngersheim (Lkr. Würzburg), N49°53'38" E09°50'13", 215m, 03-Aug-2011, *Rubus fruticosus agg.*, vineyard fallow, beating, leg. Sprick,P., det. Sprick,P.
Otiorhynchus porcatus<br>(Herbst, 1795)	MK891860 [new]<br>2122-JKR<br>658nt	ZFMK-TIS-23762<br>ZFMK-DNA-0169169611	Czech Republic, Bohemia, Predhradi, N49°49'56" E16°02'33", 427m, 08-Mai-2014, sieving, leg. Krátky,J., det. Krátky,J.

**Name**<br>**Authority**<br>**Additional Information**	**GenBank Acc No (Ref.)**<br>**Specimen ID**<br>**Sequence Length**	**ZFMK Tissue ID**<br>**ZFMK DNA Sample ID**<br>**(SDEI DNA Sample ID)**	**Locality, GPS, Collection Date, Plant, Collector, Identifier**
Otiorhynchus porcatus<br>(Herbst, 1795)	KC783991 [new]<br>269-PSP<br>658nt	ZFMK-TIS-2D100439102<br>ZFMK-DNA-0100439774	Germany, Lower Saxony (NI), Nordstemmen, Lkr. Hildesheim, N52°09'00" E09°47'00", 72m, 17-Mai-2011, *Primula veris*, garden, leg. Sprick,P., det. Sprick,P.
Otiorhynchus procerus cazorlae<br>Roudier, 1958<br>Löbl states synonym for this taxa	MK891326 [new]<br>1337-PST<br>658nt	ZFMK-TIS-4153<br>ZFMK-DNA-0100426139	Spain, Jaen, Sierra de Cazorla, Sierra del Pozo, N37°54'31" W02°53'57", 1452m, 14-Mai-2013, *Quercus ilex, Erinacea anthyllis*, sieving, leg. Stüben,P. & Schütte,A., det. Stüben,P.
Otiorhynchus pyrenaeus<br>Gyllenhal, 1834	MK891327 [new]<br>1339-PST<br>658nt	ZFMK-TIS-4155<br>ZFMK-DNA-0100426137	Spain, Jaen, SE of Mancha Real, Sierra Almaden (El Almaden), N37°44'06" W03°31'30", 1963m, 15-Mai-2013, *Euphorbia nicaeensis*, sieving, leg. Stüben,P. & Schütte,A., det. Stüben,P.
Otiorhynchus raucus<br>(Fabricius, 1777)	MK891789 [new]<br>2048-JKR<br>658nt	ZFMK-TIS-23688<br>ZFMK-DNA-0169170453	Czech Republic, Bohemia, Kosatky, N50°19'23" E14°39'58", 218m, 02-Mai-2014, sieving, leg. Krátky,J., det. Krátky,J.
Otiorhynchus raucus<br>(Fabricius, 1777)	MK890823 [40]<br>212-PSP<br>658nt	ZFMK-TIS-2D100439154<br>ZFMK-DNA-0100439826	Germany, Hesse (HE), Wiesbaden-Schierstein, N50°02'59" E08°12'27", 283m, 06-Apr-2011, *Paeonia officinalis*, garden, soil-trap, leg. Kubach,G., det. Sprick,P.
Otiorhynchus raucus<br>(Fabricius, 1777)	MK891417 [new]<br>1479-PSP<br>658nt	ZFMK-TIS-3239<br>ZFMK-DNA-0155633350	Germany, Lower Saxony (NI), Bad Zwischenahn, Aschhausen, N53°12'19" E08°04'22", 11m, 09-Jul-2012, *Thuja occidentalis*, beating, leg. Sprick,P., det. Sprick,P.
Otiorhynchus raucus<br>(Fabricius, 1777)	MK890858 [new]<br>410-RGO<br>557nt	ZFMK-TIS-20196<br>ZFMK-DNA-0100449428	Poland, Rudnik, N51°14'31.02" E22°32'22.92", 191m, 05-Jun-2011, leg. Gosik,R., det. Gosik,R.
Otiorhynchus raucus<br>(Fabricius, 1777)	MK892265 [new]<br>2565-PSP<br>658nt	ZFMK-TIS-23539<br>ZFMK-DNA-0171662001	Slovakia, Nitra, Sturovo, N47°47'53" E18°43'30", 107m, 19-Mai-2014, *Symphoricarpos chenaultii, Juniperus* sp., leg. Sprick,P., det. Sprick,P.
Otiorhynchus rotundus<br>Marseul, 1872<br>formerly: *O. smreczynskii* - Syn!	MK891332 [new]<br>198-PSP<br>658nt	ZFMK-TIS-2D100439173<br>ZFMK-DNA-0100417595	Germany, Lower Saxony (NI), Hannover, Steintormasch, N52°22'57" E09°42'31", 50m, 06-Apr-2011, *Ligustrum vulgare*, hedge, beating, leg. Sprick,P., det. Sprick,P.
Otiorhynchus rotundus<br>Marseul, 1872<br>formerly: *O. smreczynskii* - Syn!	MK892161 [new]<br>2452-PSP<br>658nt	ZFMK-TIS-23424<br>ZFMK-DNA-0171661821	Germany, Saxony-Anhalt (ST), Harz, Ilsenburg, N51°51'20" E10°40'20", 282m, 06-Mai-2013, *Syringa* sp., garden national car park, beating, leg. Sprick,P., det. Sprick,P.
Otiorhynchus rotundus<br>Marseul, 1872	MK891134 [new]<br>1039-PSP<br>658nt	ZFMK-TIS-3184<br>ZFMK-DNA-0100426941	Poland, Lesser Poland, Krakow, Pradnik Czerwony , N50°05'31.3" E19°58'33.04", 220m, 27-Aug-2012, *Ligustrum vulgare*, beating, leg. Knutelski,S., det. Knutelski,S.
Otiorhynchus rugosostriatus<br>(Goeze, 1777)	MK891949 [new]<br>2217-JKR<br>658nt	ZFMK-TIS-23955<br>ZFMK-DNA-0169170570	Czech Republic, Moravia, Olomouc, Tabulovy vrch, N49°35'12" E17°13'16", 257m, 04-Sep-2014, beating, leg. Bednarik,M., det. Bednarik,M.
Otiorhynchus rugosostriatus<br>(Goeze, 1777)	MK890822 [new]<br>211-PSP<br>658nt	ZFMK-TIS-2D100439153<br>ZFMK-DNA-0100439825	Germany, Hesse (HE), Wiesbaden-Schierstein, N50°02'59" E08°12'27", 283m, 06-Apr-2011, *Panicum virgatum*, garden, soil-trap, leg. Kubach,G., det. Sprick,P.

**Name**<br>**Authority**<br>**Additional Information**	**GenBank Acc No (Ref.)**<br>**Specimen ID**<br>**Sequence Length**	**ZFMK Tissue ID**<br>**ZFMK DNA Sample ID**<br>**(SDEI DNA Sample ID)**	**Locality, GPS, Collection Date, Plant, Collector, Identifier**
Otiorhynchus rugosostriatus<br>(Goeze, 1777)	MK891115 [new]<br>1020-PSP<br>658nt	ZFMK-TIS-3165<br>ZFMK-DNA-0100426850	Germany, Lower Saxony (NI), Hannover, Linden, S of Leine river, N52°22'40" E09°42'30", 56m, 11-Mai-2012, *Euonymus japonica*, park, beating, leg. Sprick,P., det. Sprick,P.
Otiorhynchus rugosostriatus<br>(Goeze, 1777)	KC784262 [40]<br>IT-0032w<br>655nt	ZFMK-TIS-2D100446519<br>ZFMK-DNA-0100437844	Italy, Sardinia Isl. West, SW of Macomer, M. Ferru near S. Leonardo, N40°11'03" E08°40'09", 697m, 07-Okt-2010, *Quercus*, ditch, leg. Stüben,P., det. Stüben,P.
Otiorhynchus salicicola<br>Heyden, 1908	KC784142 [new]<br>627-PSP<br>658nt	ZFMK-TIS-20410<br>ZFMK-DNA-0100448633	Germany, Brandenburg (BB), Frankfurt an der Oder, N52°20'00'' E14°32'00'', 75m, 10-Aug-2011, *Forsythia x intermedia*, beating, leg. Sprick,P., det. Sprick,P.
Otiorhynchus salicicola<br>Heyden, 1908	KC784151 [new]<br>618-PSP<br>658nt	ZFMK-TIS-20401<br>ZFMK-DNA-0100448649	Germany, Hamburg (HH), Hamburg-Lohbrügge, N53°30'00'' E10°12'00'', 29m, 21-Jul-2011, *Thuja occidentalis*, garden, beating, leg. Sprick,P., det. Sprick,P.
Otiorhynchus scaber<br>(Linne, 1758)	MK892016 [new]<br>2289-JKR<br>658nt	ZFMK-TIS-24220<br>ZFMK-DNA-0169170976	Austria, Styria, Trofaiach, Rötz, N47°26'56" E15°01'39", 758m, 28-Jun-2014, mountain forest, sweeping, leg. Krátky,J., det. Krátky,J.
Otiorhynchus scaber<br>(Linne, 1758)	MK892023 [new]<br>2296-JKR<br>658nt	ZFMK-TIS-24227<br>ZFMK-DNA-0169170962	Austria, Kaernten, Feistritz ob Bleiburg, N46°32'17" E14°46'28", 702m, 28-Jun-2014, mountain forest, sieving, leg. Krátky,J., det. Krátky,J.
Otiorhynchus scaber<br>(Linne, 1758)	MK892070 [new]<br>2346-JKR<br>658nt	ZFMK-TIS-24277<br>ZFMK-DNA-0169170916	Czech Republic, Bohemia, Horni Morava, N50°10'15" E16°49'22", 780m, 17-Jul-2014, sieving, leg. Krátky,J., det. Krátky,J.
Otiorhynchus scaber<br>(Linnaeus, 1758)	KC783901 [new]<br>286-PSP<br>658nt	ZFMK-TIS-2D100439084<br>ZFMK-DNA-0100438506	Germany, Lower Saxony (NI), Hannover, Herrenhausen, "Berggarten" area, N52°23'43" E09°41'59", 50m, 30-Mai-2011, *Picea abies, Taxus baccata*, beating, leg. Sprick,P., det. Sprick,P.
Otiorhynchus scaber<br>(Linne, 1758)	MK891410 [new]<br>1472-PSP<br>658nt	ZFMK-TIS-3232<br>ZFMK-DNA-0155633358	Germany, Lower Saxony (NI), Harz, St. Andreasberg, Jordanshöhe, N51°42'58" E10°32'06", 670m, 02-Jul-2012, *Sorbus aucuparia*, tree group, beating, leg. Sprick,P., det. Sprick,P.
Otiorhynchus scaber<br>(Linne, 1758)	MK891367 [new]<br>1418-JKR<br>658nt	ZFMK-TIS-4187<br>ZFMK-DNA-0155628504	Slovakia, Vysoke Tatry Mts., Dolina siedmich prame?ov, N49°13'38" E20°16'34", 1448m, 30-Jun-2013, sweeping, leg. Krátky,J., det. Krátky,J.
Otiorhynchus singularis<br>(Linnaeus, 1767)	MK891800 [new]<br>2059-JKR<br>658nt	ZFMK-TIS-23699<br>ZFMK-DNA-0169170433	Czech Republic, Bohemia, Hradec Kralove, Kocianovice, N50°13'16" E15°52'51", 237m, 06-Mai-2014, sweeping, leg. Krátky,J., det. Krátky,J.
Otiorhynchus singularis<br>(Linnaeus, 1767)	KC784020 [new]<br>221-PSP<br>658nt	ZFMK-TIS-2D100439150<br>ZFMK-DNA-0100439822	Germany, Lower Saxony (NI), Hannover, Herrenhausen, "Berggarten" area, N52°23'44" E09°41'59", 50m, 18-Apr-2011, *Ilex aquifolium*, beating, leg. Sprick,P., det. Sprick,P.
Otiorhynchus singularis<br>(Linnaeus, 1767)	MK891407 [new]<br>1469-PSP<br>658nt	ZFMK-TIS-3229<br>ZFMK-DNA-0155633355	Germany, Lower Saxony (NI), Harz, St. Andreasberg, Jordanshöhe, N51°42'58" E10°32'03", 667m, 02-Jul-2012, *Rubus idaeus*, tree group, beating, leg. Sprick,P., det. Sprick,P.
Otiorhynchus singularis<br>(Linnaeus, 1767)	MK891644 [40]<br>1797-PST<br>658nt	ZFMK-TIS-24131<br>ZFMK-DNA-0171624067	Germany, Rhineland-Palatinate (RLP), Ahr valley, Dernau, below Krausberg near Steinbergsmühle, N50°31'40" E07°02'43", 204m, 29-Mai-2014, beating, leg. Stüben,P., det. Stüben,P.
Otiorhynchus subdentatus<br>Stierlin, 1858	MK892068 [new]<br>2344-JKR<br>658nt	ZFMK-TIS-24275<br>ZFMK-DNA-0169170914	Czech Republic, Bohemia, Horni Morava, N50°10'15" E16°49'22", 780m, 17-Jul-2014, sweeping, leg. Krátky,J., det. Krátky,J.

**Name**<br>**Authority**<br>**Additional Information**	**GenBank Acc No (Ref.)**<br>**Specimen ID**<br>**Sequence Length**	**ZFMK Tissue ID**<br>**ZFMK DNA Sample ID**<br>**(SDEI DNA Sample ID)**	**Locality, GPS, Collection Date, Plant, Collector, Identifier**
Otiorhynchus sulcatus<br>(Fabricius, 1775)	MK890816 [40]<br>200-PSP<br>658nt	ZFMK-TIS-2D100438914<br>ZFMK-DNA-0100438546	Germany, Lower Saxony (NI), Hannover, Linden, parque south of Leine river, N52°22'40" E09°42'30", 55m, 06-Apr-2011, *Parthenocissus inserta*, beating, leg. Sprick,P., det. Sprick,P.
Otiorhynchus sulcatus<br>(Fabricius, 1775)	MK891418 [new]<br>1480-PSP<br>658nt	ZFMK-TIS-3240<br>ZFMK-DNA-0155633349	Germany, Lower Saxony (NI), Bad Zwischenahn, Aschhausen, N53°12'19" E08°04'22", 11m, 09-Jul-2012, *Thuja occidentalis*, beating, leg. Sprick,P., det. Sprick,P.
Otiorhynchus tenebricosus<br>(Herbst, 1784)<br>formerly: *O. tenebricosus fuscipes* - Syn!	KC784215 [new]<br>426-RGO<br>658nt	ZFMK-TIS-3235<br>ZFMK-DNA-0155633361	Germany, Lower Saxony (NI), Harz National Park, Oderteich, Fichtenwald, N51°46'16" E10°32'03", 734m, 02-Jul-2012, *Picea abies, Vaccinium myrtillus*, beating, leg. Sprick,P., det. Sprick,P.
Otiorhynchus tenebricosus<br>(Herbst, 1784)	MK891413 [new]<br>1475-PSP<br>658nt	ZFMK-TIS-3120<br>ZFMK-DNA-0100426292	Germany, Lower Saxony (NI), Lauenstein, Lkr.Hameln-Pyrmont, Ith Mts., N52°04'13' E09°32'30", 370m, 26-Mrz-2012, *Fraxinus excelsior*, *Fagus* forest ("Kalkbuchen"), limestone, soil-search, leg. Sprick,P., det. Sprick,P.
Otiorhynchus tenebricosus<br>(Herbst, 1784)	MK891425 [new]<br>1488-PSP<br>658nt	ZFMK-TIS-3248<br>ZFMK-DNA-0155633341	Germany, North Rhine-Westphalia (NRW), Paderborn, Wewer, Ziegenberg, N51°41'13" E08°42'43", 153m, 15-Jul-2012, *Fraxinus excelsior*, wayside, beating, leg. Sprick,P., det. Sprick,P.
Otiorhynchus tenebricosus<br>(Herbst, 1784)	MK891073 [new]<br>975-PSP<br>658nt	ZFMK-TIS-20212<br>ZFMK-DNA-0100449413	Poland, Kiry, Tatra Mountains, N49°16'26" E19°52'07", 942m, 06-Jul-2011, leg. Gosik,R., det. Gosik,R.
Otiorhynchus velutinus<br>Germar, 1824	MK891838 [new]<br>2099-JKR<br>658nt	ZFMK-TIS-23739<br>ZFMK-DNA-0169169634	Czech Republic, Moravia, Krumvir, PR Louky pod Kumstatem, N48°59'36" E16°55'20.5", 211m, 09-Mai-2014, sweeping, leg. Krátky,J., det. Krátky,J.
Otiorhynchus velutinus<br>Germar, 1824	MK891856 [new]<br>2118-JKR<br>658nt	ZFMK-TIS-23758<br>ZFMK-DNA-0169169615	Czech Republic, Bohemia, Kosatky, N50°19'23" E14°39'58", 218m, 02-Mai-2014, sieving, leg. Krátky,J., det. Krátky,J.
Otiorhynchus veterator<br>Uyttenboogaart, 1932	MK891030 [new]<br>927-CBR<br>658nt	ZFMK-TIS-3840<br>ZFMK-DNA-0100449684	Luxembourg, 10 km E of Luxembourg, 2 km S of Mensdorf, N49°38'09" E06°17'48", 330m, 05-Jun-2012, beating, leg. Braunert,C., det. Braunert,C.
Oxystoma craccae<br>(Linnaeus, 1767)	MK890974 [new]<br>865-PST<br>658nt	ZFMK-TIS-3778<br>ZFMK-DNA-0100414215	Germany, Rhineland-Palatinate (RLP), Adenau, N50°24'43" E06°55'48", 259m, 24-Jul-2012, beating, leg. Stüben,P., det. Stüben,P.
Oxystoma craccae<br>(Linnaeus, 1767)	MK892313 [new]<br>2614-PSP<br>658nt	ZFMK-TIS-23588<br>ZFMK-DNA-0171661954	Germany, Bavaria (BY), Unterfranken, Ebern, N50°05'07" E10°45'52.5", 318m, 14-Jun-2014, *Vicia cracca*, leg. Sprick,P., det. Sprick,P.
Oxystoma craccae<br>(Linnaeus, 1767)	MK891686 [new]<br>1902-PST<br>658nt	ZFMK-TIS-24007<br>ZFMK-DNA-0169170421	Netherlands, Elmpt, NP "DE of Meinweg", N51°11'08" E06°08'22", 75m, 28-Aug-2014, *Vicia*, beating, leg. Stüben,P., det. Stüben,P.
Oxystoma opeticum<br>(Bach, 1854)	MK892201 [new]<br>2496-PSP<br>658nt	ZFMK-TIS-23468<br>ZFMK-DNA-0171661786	Germany, Saxony-Anhalt (ST), Harz National Park, Schierke, Feuersteinwiesen, N51°45'21" E10°41'25", 604m, 24-Apr-2014, *Lathyrus linifolius*, dipnet, leg. Sprick,P., det. Sprick,P.
Oxystoma pomonae<br>(Fabricius, 1798)	MK892212 [new]<br>2508-PSP<br>658nt	ZFMK-TIS-23480<br>ZFMK-DNA-0171661765	Germany, Lower Saxony (NI), Hannover region, Brelingen, N52°33'52" E09°41'09", 75m, 08-Mai-2014, *Waldkrautschicht*, dipnet, leg. Sprick,P., det. Sprick,P.

**Name**<br>**Authority**<br>**Additional Information**	**GenBank Acc No (Ref.)**<br>**Specimen ID**<br>**Sequence Length**	**ZFMK Tissue ID**<br>**ZFMK DNA Sample ID**<br>**(SDEI DNA Sample ID)**	**Locality, GPS, Collection Date, Plant, Collector, Identifier**
Oxystoma subulatum<br>(Kirby, 1808)	MK892296 [new]<br>2597-PSP<br>658nt	ZFMK-TIS-23571<br>ZFMK-DNA-0171661962	Germany, Bavaria (BY), Unterfranken, Ebern, N50°05'20.5" E10°45'59", 333m, 13-Jun-2014, *Lathyrus pratensis*, leg. Sprick,P., det. Sprick,P.
Oxystoma subulatum<br>(Kirby, 1808)	MK891373 [new]<br>1424-JKR<br>658nt	ZFMK-TIS-4193<br>ZFMK-DNA-0155628510	Slovakia, Vysoke Tatry Mts., Dolina siedmich prame?ov, N49°13'38" E20°16'34", 1448m, 30-Jun-2013, sweeping, leg. Krátky,J., det. Krátky,J.
Pachycerus segnis<br>(Germar, 1824)	MK891933 [new]<br>2201-JKR<br>658nt	ZFMK-TIS-23939<br>ZFMK-DNA-0169170577	Czech Republic, Moravia, Znojmo, Oblekovice, N48°50'15" E16°04'43", 220m, 08-Mai-2014, *Echium vulgare*, collecting by hand, leg. Stejskal,R., det. Stejskal,R.
Pachyrhinus glabratus<br>(Chevrolat, 1866)	MK890956 [new]<br>836-PST<br>658nt	ZFMK-TIS-3749<br>ZFMK-DNA-0100414255	Portugal, Minho, S of Viana, Castelo do Neiva, N41°37'07" W08°48'35", 8m, 10-Mai-2012, *Pinus*, beating, leg. Stüben,P., det. Stüben,P.
Pachyrhinus glabratus<br>(Chevrolat, 1866)	MK891295 [new]<br>1265-PST<br>658nt	ZFMK-TIS-4081<br>ZFMK-DNA-0100426211	Spain, Zaragoza, Aragon, NO of Calatayud, N41°22'20" W01°35'01", 703m, 30-Apr-2013, *Pinus*, beating, leg. Stüben,P. & Schütte,A., det. Stüben,P.
Pachyrhinus lethierryi<br>Desbrochers, 1875	KC783999 [new]<br>253-PSP<br>658nt	ZFMK-TIS-2D100438512<br>ZFMK-DNA-0100439790	Germany, Lower Saxony (NI), Hannover, Steintormasch, N52°22'57" E09°42'31", 50m, 11-Mai-2011, *Thuja occidentalis*, hedge, leg. Sprick,P., det. Sprick,P.
Pachyrhinus squamulosus<br>Herbst, 1795	MK891929 [new]<br>2197-JKR<br>658nt	ZFMK-TIS-23935<br>ZFMK-DNA-0169170590	Czech Republic, Moravia, Cernin, N48°59'30" E16°00'57", 350m, 01-Mai-2014, *Pinus sylvestris*, beating, leg. Stejskal,R., det. Stejskal,R.
Pachyrhinus warioni<br>(Marseul, 1876)	KC783886 [new]<br>183-PST<br>658nt	ZFMK-TIS-2D100438665<br>ZFMK-DNA-0100438333	Morocco, Atlas Mts., NE of Ifrane, N33°36'08" W04°55'30", 1659m, 20-Mai-2011, *Pinus*, beating, leg. Stüben,P., det. Stüben,P./vid. Behne,L.
Pachytychius haematocephalus<br>(Gyllenhal, 1835)	MK891291 [new]<br>1250-FBA<br>658nt	ZFMK-TIS-3586<br>ZFMK-DNA-0100426023	Greece, Peloponnese, SW of Taygetos Mts., S. Agios Nikolao, N36°49'33" E22°16'45", 2m, 28-Apr-2009, coast, leg. Bahr,F., det. Bayer,B.
Pachytychius haematocephalus<br>(Gyllenhal, 1835)	MK891695 [new]<br>1913-FBA<br>658nt	ZFMK-TIS-23304<br>ZFMK-DNA-0169170361	Greece, East Macedonia and Thrace, Drama, Potami, N41°23'55" E24°05'57", 380m, 11-Aug-2014, leg. Bayer & Brunner, det. Bayer,F.
Pachytychius haematocephalus<br>(Gyllenhal, 1835)	MK891065 [new]<br>966-CBR<br>658nt	ZFMK-TIS-3879<br>ZFMK-DNA-0100449644	Luxembourg, 10 km NW of Esch/Alzette, 1 km NW of Niedercorn, N49°32'31" E05°52'39", 370m, 28-Jun-2012, *Lotus corniculatus*, sweeping, leg. Braunert,C., det. Braunert,C.
Pachytychius hordei grandicollis<br>(Waltl, 1835)	MK891760 [new]<br>2016-JKR<br>658nt	ZFMK-TIS-23656<br>ZFMK-DNA-0169170486	Italy, Sicilia Isl. (TP), Trapani, Marinella, N37°34'59" E12°51'40", 3m, 17-Apr-2014, sweeping, leg. Krátky,J., det. Krátky,J.
Pachytychius hordei grandicollis<br>(Waltl, 1835)	MK891321 [new]<br>1321-PST<br>658nt	ZFMK-TIS-4137<br>ZFMK-DNA-0100426299	Spain, Malaga, S of Mollina, N37°04'45" W04°40'08", 437m, 07-Mai-2013, *wayside vegetation in olive plantation*, beating, leg. Stüben,P. & Schütte,A., det. Stüben,P.
Pachytychius hordei hordei<br>(Brullé, 1832)	MK892342 [new]<br>2684-PSP<br>658nt	ZFMK-TIS-23834<br>ZFMK-DNA-0171661315	Cyprus, Limassol district, Akrotiri-salt lake, Ostseite, Zakaki, N34°38'38" E33°00'22", 2m, 05-Apr-2013, *herb layer*, dipnet, leg. Sprick,P., det. Sprick,P.
Pachytychius robustus<br>(Wollaston, 1854)	MK891710 [40]<br>1934-PST<br>658nt	ZFMK-TIS-24013<br>ZFMK-DNA-0169170412	Portugal, Madeira, Ilhas Desertas, south of Bugio, Planalto, N32°24'41" W16°28'24", 350m, 03-Jul-2014, beating, leg. Silva,H., det. Stüben,P.

**Name**<br>**Authority**<br>**Additional Information**	**GenBank Acc No (Ref.)**<br>**Specimen ID**<br>**Sequence Length**	**ZFMK Tissue ID**<br>**ZFMK DNA Sample ID**<br>**(SDEI DNA Sample ID)**	**Locality, GPS, Collection Date, Plant, Collector, Identifier**
Pachytychius robustus<br>(Wollaston, 1854)	MK892365 [40]<br>2776-PST<br>658nt	ZFMK-TIS-26130<br>ZFMK-DNA-0169166966	Portugal, Madeira, Ilhas Desertas, Deserta Grande, Doca, N32°30'49" W16°30'33", 23m, 22-Mrz-2015, *Lotus*, collecting by hand, leg. Stüben,P., det. Stüben,P.
Pachytychius robustus<br>(Wollaston, 1854)	MK347525 [40]<br>2914-PST<br>658nt	(SDEI-DNA-2914-PST)	Portugal, Madeira, Achada da Cruz, Quebrada Nova, N32°51'21" W17°12'50", 21m, 05-Dez-2016, sifting, leg. Stüben,P., det. Stüben,P.
Pachytychius robustus<br>(Wollaston, 1854)	MK892372 [40]<br>2822-PST<br>658nt	ZFMK-TIS-25868<br>ZFMK-DNA-FC17941524	Portugal, Madeira, Ponta de Sao Lourenco, Pedras Brancas, N32°44'49" W16°42'12", 100m, 23-Nov-2015, *Cynara cardunculus*, under stones, sieving, leg. Stüben,P. & Schütte,A., det. Stüben,P.
Pachytychius sparsutus<br>(Olivier, 1807)	MK891825 [new]<br>2086-JKR<br>658nt	ZFMK-TIS-23726<br>ZFMK-DNA-0169169644	Czech Republic, Moravia, Bucovice, PR Sevy, N49°08'05" E16°58'19", 249m, 09-Mai-2014, *Genista* sp., sweeping, leg. Krátky,J., det. Krátky,J.
Pachytychius sparsutus<br>(Olivier, 1807)	MK891011 [new]<br>906-CBR<br>658nt	ZFMK-TIS-3819<br>ZFMK-DNA-0100449705	Luxembourg, 10 km NW of Ettelbruck, 1 km E of Goebelsmühle, N49°55'15" E06°03'34", 320m, 07-Mai-2011, *Cytisus scoparius*, beating, leg. Braunert,C., det. Braunert,C.
Pachytychius sparsutus<br>(Olivier, 1807)	MK891555 [new]<br>1646-JKR<br>658nt	ZFMK-TIS-4224<br>ZFMK-DNA-0155630494	Romania, Buzau, Vulcanii Noroisi-Paclele Mici, N45°20'38" E26°42'37", 300m, 09-Jun-2013, sweeping, leg. Pelikan,J., det. Krátky,J.
Palophagoides vargasorum<br>Kuschel, 1996	FJ867810<br>658 nt	not applicable	Note: used as outgroup in this study
Paophilus afflatus<br>(Boheman, 1833)	MK891201 [new]<br>1119-JKR<br>658nt	ZFMK-TIS-3455<br>ZFMK-DNA-0100449589	Slovakia, Nove Zamky, Tvrdosovce, N48°06'09" E18°02'08", 110m, 10-Mai-2012, sweeping, leg. Krátky,J., det. Krátky,J.
Paophilus afflatus<br>(Boheman, 1833)	MK891922 [new]<br>2190-JKR<br>658nt	ZFMK-TIS-23928<br>ZFMK-DNA-0169170597	Slovakia, Nove Zamky, Kamenin, Cistiny, N47°51'55" E18°38'05", 111m, 22-Mai-2014, *Artemisia*, collecting by hand, leg. Stejskal,R., det. Stejskal,R.
Parameira krueperi<br>Faust, 1894	KC784038 [new]<br>674-FBA<br>658nt	ZFMK-TIS-20453<br>ZFMK-DNA-0100448234	Greece, Peloponnese, Messinia, Mt. Taygetos, E of Agh. Nikolaos, N36°49'16" E22°17'55", 30m, 22-Mai-2011, leg. Bahr,F., det. Bahr,F.
Parascythopus apollinis<br>(Miller, 1862)	JX448427 [new]<br>676-FBA<br>658nt	ZFMK-TIS-c676-FBA<br>ZFMK-DNA-0100448586	Greece, Peloponnese, Messinia, Mt. Taygetos, S of Marathos, N36°53'46" E22°19'09", 1300m, 21-Mai-2011, leg. Bahr,F., det. Bahr,F.
Parethelcus nesicola<br>Colonnelli, 1990	MK892392 [40]<br>2868-PST<br>658nt	ZFMK-TIS-4257<br>ZFMK-DNA-0169166924	Spain, Canary Islands, El Hierro, W of Frontera, Pista al Derrabado, N27°44'29" W18°03'20", 914m, 11-Apr-2016, *Urtica*, beating, leg. Stüben,P. & Schütte,A., det. Stüben,P.
Parethelcus nesicola<br>Colonnelli, 1990	KC783732 [13]<br>4-PST<br>627nt	ZFMK-TIS-2D100447043<br>ZFMK-DNA-0100437945	Spain, Canary Islands, Gran Canaria, Marzagan, Barranco de las Goteras, N28°01'28" W15°27'31", 236m, 01-Dez-2010, *Urtica*, beating, leg. Stüben,P., det. Stüben,P.
Parethelcus nesicola<br>Colonnelli, 1990	MK891589 [40]<br>706-PST<br>658nt	ZFMK-TIS-3043<br>ZFMK-DNA-0155630431	Spain, Canary Islands, Tenerife, Icod, La Vega, N28°21'25" W16°43'44", 647m, 04-Jan-2012, beating, leg. Stüben,P., det. Stüben,P.
Parethelcus pollinarius<br>(Forster, 1771)	KC783977 [new]<br>298-JKR<br>658nt	ZFMK-TIS-20084<br>ZFMK-DNA-0100438969	Czech Republic, Bohemia or., Dritec, N50°7'11.648" E15°48'6.919", 230m, 06-Mai-2011, *Urtica dioica*, sweeping, leg. Krátky,J., det. Krátky,J.

**Name**<br>**Authority**<br>**Additional Information**	**GenBank Acc No (Ref.)**<br>**Specimen ID**<br>**Sequence Length**	**ZFMK Tissue ID**<br>**ZFMK DNA Sample ID**<br>**(SDEI DNA Sample ID)**	**Locality, GPS, Collection Date, Plant, Collector, Identifier**
Parethelcus pollinarius<br>(Forster, 1771)	KC784016 [new]<br>226-PSP<br>658nt	ZFMK-TIS-2D100439145<br>ZFMK-DNA-0100439817	Germany, Lower Saxony (NI), Lemmie, Hannover region, Gehrdener Berg, N52°17'44" E09°35'23", 100m, 22-Apr-2011, *Urtica dioica*, field edge, beating, leg. Sprick,P., det. Sprick,P.
Parethelcus pollinarius<br>(Forster, 1771)	KC784321 [new]<br>776-PST<br>658nt	ZFMK-TIS-3113<br>ZFMK-DNA-0100448106	Germany, North Rhine-Westphalia (NRW), Mönchengladbach-Neuwerk, N51°13'16" E06°26'25", 48m, 29-Apr-2012, *Urtica dioica*, beating, leg. Stüben,P., det. Stüben,P.
Parethelcus pollinarius<br>(Forster, 1771)	MK891056 [new]<br>956-CBR<br>658nt	ZFMK-TIS-3869<br>ZFMK-DNA-0100449655	Luxembourg, 10 km E of Luxembourg, 1 km SW of Mensdorf, N49°39'00" E06°17'40", 240m, 21-Jun-2012, *Urtica dioica*, beating, leg. Braunert,C., det. Braunert,C.
Parethelcus pollinarius<br>(Forster, 1771)	KM433743 [13]<br>1231-PST<br>658nt	ZFMK-TIS-3567<br>ZFMK-DNA-0100426224	Portugal, Minho, S of Viana, Castelo do Neiva, N41°37'07 W08°48'35", 8m, 10-Mai-2012, *Urticaceae*, beating, leg. Stüben,P., det. Krátky,J.
Paroxyonyx audisioi<br>Colonelli, 2005	MK891253 [new]<br>1185-JKR<br>658nt	ZFMK-TIS-3521<br>ZFMK-DNA-0100426102	Spain, Andalucia, San Agustin, N36°41'15" W02°42'40", 1m, 12-Jun-2012, *Ephedra major*, coast, sandy dunes, beating, leg. Krátky,J., det. Krátky,J.
Paroxyonyx cinctus<br>(Chevrolat, 1861)	MK891490 [new]<br>1572-JKR<br>658nt	ZFMK-TIS-3620<br>ZFMK-DNA-0155635666	Morocco, Marrakech-Tensift-El Haouz, Essaouira, 9 km E, N31°29'35" W09°43'00", 88m, 12-Apr-2013, *Ephedra altissima*, beating, leg. Krátky,J., det. Krátky,J.
Paroxyonyx cinctus<br>(Chevrolat, 1861)	MK891184 [new]<br>1101-JKR<br>658nt	ZFMK-TIS-3437<br>ZFMK-DNA-0100449607	Spain, Andalucia, 3 km SW of Monda, N36°36'37" W04°51'04", 460m, 22-Apr-2012, *Ephedra alata*, beating, leg. Krátky,J., det. Krátky,J.
Paroxyonyx fallaciosus<br>(Desbrochers des Loges, 1896)	MK891495 [new]<br>1578-JKR<br>658nt	ZFMK-TIS-3626<br>ZFMK-DNA-0155635651	Morocco, Marrakech-Tensift-El Haouz, Essaouira, 9 km E, N31°29'35" W09°43'00", 88m, 12-Apr-2013, *Ephedra ?major*, beating, leg. Krátky,J., det. Krátky,J.
Paroxyonyx imitator<br>(Wagner, 1928)	MK891185 [new]<br>1102-JKR<br>658nt	ZFMK-TIS-3438<br>ZFMK-DNA-0100449606	Spain, Andalucia, 3 km SW of Monda, N36°36'37" W04°51'04", 460m, 22-Apr-2012, *Ephedra alata*, beating, leg. Krátky,J., det. Krátky,J.
Pelenomus canaliculatus<br>(Fahraeus, 1843)	MK891945 [new]<br>2213-JKR<br>658nt	ZFMK-TIS-23951<br>ZFMK-DNA-0169170574	Czech Republic, Bohemia, Liboc, N50°06'42" E12°30'48", 417m, 26-Jul-2014, leg. Benedikt,S., det. Benedikt,S.
Pelenomus canaliculatus<br>(Fahraeus, 1843)	MK890889 [new]<br>559-RST<br>658nt	ZFMK-TIS-20344<br>ZFMK-DNA-0100448421	Slovakia, Slovakia occ., 0.7 km SE of Dolne Zelenice, N48°22'13.95" E17°45'8.62", 135m, 02-Okt-2011, hand-collecting, leg. Stejskal,R., det. Stejskal,R.
Pelenomus commari<br>(Panzer, 1795)	MK891959 [new]<br>2228-JKR<br>658nt	ZFMK-TIS-23966<br>ZFMK-DNA-0169170556	Czech Republic, Bohemia, Brazec, N50°10'20" E13°02'13", 697m, 08-Jun-2014, *Comarum palustre*, leg. Benedikt,S., det. Benedikt,S.
Pelenomus commari<br>(Panzer, 1795)	MK890826 [new]<br>245-PSP<br>658nt	ZFMK-TIS-2D100438519<br>ZFMK-DNA-0100439798	Germany, Lower Saxony (NI), Essel, Lkr. Soltau-Fallingbostel, N52°41'57" E09°38'47", 25m, 10-Mai-2011, *Comarum palustre*, sand field, dipnet, leg. Sprick,P., det. Sprick,P.
Pelenomus commari<br>(Panzer, 1795)	MK891470 [new]<br>1537-PSP<br>658nt	ZFMK-TIS-3297<br>ZFMK-DNA-0155633292	Germany, Saxony-Anhalt (ST), Gladdenstedt, Lkr. Salzwedel, Ohreaue, N52°39'46" E10°52'53", 74m, 14-Jun-2013, *Comarum palustre*, wetlands, dipnet, leg. Sprick,P., det. Sprick,P.
Pelenomus commari<br>(Panzer, 1795)	MK891529 [new]<br>1617-JKR<br>658nt	ZFMK-TIS-3665<br>ZFMK-DNA-0155635615	Slovakia, Komarno, B?c env., N47°46'55" E18°25'11", 109m, 18-Mai-2013, sweeping, leg. Krátky,J., det. Krátky,J.

**Name**<br>**Authority**<br>**Additional Information**	**GenBank Acc No (Ref.)**<br>**Specimen ID**<br>**Sequence Length**	**ZFMK Tissue ID**<br>**ZFMK DNA Sample ID**<br>**(SDEI DNA Sample ID)**	**Locality, GPS, Collection Date, Plant, Collector, Identifier**
Pelenomus olssoni<br>(Israelson, 1972)	MK892123 [new]<br>2401-JKR<br>658nt	ZFMK-TIS-25978<br>ZFMK-DNA-0171600623	Czech Republic, Bohemia, Hradec Kralove, PP Na Plachte, N50°11'17" E15°51'41", 244m, 28-Sep-2014, *Peplis portula*, collecting by hand, leg. Krátky,J., det. Krátky,J.
Pelenomus velaris<br>(Gyllenhal, 1827)	MK890838 [new]<br>300-JKR<br>658nt	ZFMK-TIS-20086<br>ZFMK-DNA-0100438967	Czech Republic, Bohemia or., Dritec, N50°7'11.648" E15°48'6.919", 230m, 06-Mai-2011, hand-collecting, leg. Krátky,J., det. Krátky,J.
Pelenomus velaris<br>(Gyllenhal, 1827)	MK891004 [new]<br>898-CBR<br>658nt	ZFMK-TIS-3811<br>ZFMK-DNA-0100449720	France, Dep. Ardenne, 10 km SE of Vouziers, Brecy-Brieres, N49°19'20" E04°46'15", 100m, 23-Apr-2011, leg. Braunert,C., det. Braunert,C.
Pelenomus waltoni<br>(Boheman, 1843)	MK891677 [new]<br>1892-PST<br>658nt	ZFMK-TIS-23997<br>ZFMK-DNA-0169170431	Germany, North Rhine-Westphalia (NRW), Elmpt, NSG "Elmpter Schwalmbruch", Venekotensee, N51°13'43" E06°06'59", 35m, 27-Aug-2014, *Polygonum*, beating, leg. Stüben,P., det. Stüben,P.
Pelenomus waltoni<br>(Boheman, 1843)	MK892252 [new]<br>2551-PSP<br>658nt	ZFMK-TIS-23525<br>ZFMK-DNA-0171662008	Germany, Lower Saxony (NI), Harz National Park, Herzberg, N51°41'42" E10°19'47", 364m, 11-Jul-2014, *Persicaria hydropiper*, leg. Sprick,P., det. Sprick,P.
Pelenomus waltoni<br>(Boheman, 1843)	MK891694 [new]<br>1912-FBA<br>658nt	ZFMK-TIS-23303<br>ZFMK-DNA-0169170360	Greece, East Macedonia and Thrace, Drama, Potami, N41°23'55" E24°05'57", 380m, 11-Aug-2014, leg. Bayer & Brunner, det. Bayer,F.
Pentatemnus arenarius arenarius<br>Wollaston, 1861	MK892351 [40]<br>2735-PST<br>658nt	ZFMK-TIS-23885<br>ZFMK-DNA-0171661264	Spain, Canary Islands, Fuerteventura, Barranco de los Molinos, N28°32'34" W14°02'59", 54m, 08-Jan-2015, *Chenopodiaceae*, sieving, leg. Stüben,P., det. Stüben,P.
Pentatemnus arenarius incognitus Wollaston, 1861<br>formerly: *P. arenarius incognitus* - Syn!	MK891724 [40]<br>1956-PST<br>658nt	ZFMK-TIS-24035<br>ZFMK-DNA-0169170386	Portugal, Madeira, Ilhas Selvagens, Selvagem Pequena, around Pico do Veado, N30°02'09" W16°01'39", 15m, 07-Okt-2014, sieving, leg. Stüben,P., det. Stüben,P.
Pentatemnus arenarius incognitus Wollaston, 1861<br>formerly: *P. arenarius incognitus*- Syn!	MK891731 [40]<br>1971-PST<br>658nt	ZFMK-TIS-24050<br>ZFMK-DNA-0169170378	Portugal, Madeira, Ilhas Selvagens, Selvagem Pequena, E of Pico do Veado, N30°02'03" W16°01'28", 15m, 20-Dez-2014, *Zygophyllum fontanesii*, sieving, leg. Stüben,P., det. Stüben,P.
Pentatemnus arenarius<br>Wollaston, 1861	MH051972 [33]<br>2934-PST<br>658nt	(SDEI-DNA-2934-PST)	Spain, Canary Islands, Lanzarote, W of Órzola, sandy beach, N29°13'28" W13°27'44", 3m, 05-Jan-2017, *Zygophyllum fontanesli*, sifting, leg. Stüben,P., det. Stüben,P.
Perapion curtirostre<br>(Germar, 1817)	MK891132 [new]<br>1037-PSP<br>658nt	ZFMK-TIS-3182<br>ZFMK-DNA-0100426939	Germany, Lower Saxony (NI), Hannover, Vahrenheide, Kugelfangtrift, N52°25'17" E09°45'10", 50m, 26-Mai-2012, *Rumex acetosa*, dipnet, leg. Sprick,P., det. Sprick,P.
Perapion curtirostre<br>(Germar, 1817)	MK892200 [new]<br>2495-PSP<br>658nt	ZFMK-TIS-23467<br>ZFMK-DNA-0171661785	Germany, Saxony-Anhalt (ST), Harz National Park, Schierke, Feuersteinwiesen, N51°45'21" E10°41'25", 604m, 24-Apr-2014, *Rumex acetosa*, dipnet, leg. Sprick,P., det. Sprick,P.
Perapion fallax<br>(Wollaston, 1864)	KC783750 [40]<br>38-PST<br>637nt	ZFMK-TIS-2D100447000<br>ZFMK-DNA-0100438072	Spain, Canary Islands, Gran Canaria, E of San Mateo (Barranco), N28°01'08" W15°31'04", 668m, 31-Dez-2010, *Rumex*, beating, leg. Stüben,P., det. Stüben,P.
Perapion fallax<br>(Wollaston, 1864)	KC783779 [40]<br>70-PST<br>620nt	ZFMK-TIS-2D100446983<br>ZFMK-DNA-0100438055	Spain, Canary Islands, La Gomera, E of Hermigua, PN of Majona, El Palmar, N28°9'44" W17°10'00", 321m, 06-Feb-2011, *Rumex*, beating, leg. Stüben,P., det. Stüben,P.

**Name**<br>**Authority**<br>**Additional Information**	**GenBank Acc No (Ref.)**<br>**Specimen ID**<br>**Sequence Length**	**ZFMK Tissue ID**<br>**ZFMK DNA Sample ID**<br>**(SDEI DNA Sample ID)**	**Locality, GPS, Collection Date, Plant, Collector, Identifier**
Perapion fallax<br>(Wollaston, 1864)	MK891606 [40]<br>1690-PST<br>658nt	ZFMK-TIS-24408<br>ZFMK-DNA-0155622500	Spain, Canary Islands, La Palma, near San Isidro, N28°37'47" W17°48'02", 643m, 18-Dez-2013, *Rumex*, beating, leg. Stüben,P., det. Stüben,P.
Perapion fallax<br>(Wollaston, 1864)	KC783892 [new]<br>159-PST<br>658nt	ZFMK-TIS-2D100440268<br>ZFMK-DNA-0100438364	Morocco, S of Berkane, Mts. des Beni Snassen, Vallee Zegzel, N34°49'19" W02°23'10" , 644m, 15-Mai-2011, *Rumex*, beating, leg. Stüben,P., det. Stüben,P./vid. Behne,L.
Perapion fallax<br>(Wollaston, 1864)	MK892383 [40]<br>2842-PST<br>658nt	ZFMK-TIS-4238<br>ZFMK-DNA-FC17941510	Portugal, Madeira, Porto Santo Isl., Pico do Branco (Urze), N33°05'28" W16°18'17", 288m, 28-Nov-2015, beating, leg. Stüben,P. & Schütte,A., det. Stüben,P.
Perapion fallax<br>(Wollaston, 1864)	KC784305 [40]<br>741-PST<br>658nt	ZFMK-TIS-3078<br>ZFMK-DNA-0100448146	Spain, Canary Islands, Tenerife, Anaga Mts., El Bailadero, N28°32'51" W16°12'11", 607m, 05-Feb-2012, *Rumex*, beating, leg. Stüben,P. & Schütte,A., det. Stüben,P.
Perapion marchicum<br>(Herbst, 1797)	MK892108 [new]<br>2385-JKR<br>658nt	ZFMK-TIS-25962<br>ZFMK-DNA-0171600604	Czech Republic, Moravia, NP Podyji, Znojmo-Kravi hora hill, N48°50'46" E16°2'23.5", 300m, 10-Sep-2014, *Rumex acetosella*, sweeping, leg. R. Stejskal, det. Stejskal,R.
Perapion marchicum<br>(Herbst, 1797)	KC783836 [new]<br>648-PSP<br>658nt	ZFMK-TIS-20431<br>ZFMK-DNA-0100417869	Germany, Lower Saxony (NI), Hannover, Vahrenheide, "Kugelfangtrift", N52°25'22" E09°45'25", 51m, 02-Okt-2011, *Rumex acetosella*, nutrient-poor sand grassland, dipnet, leg. Sprick,P., det. Sprick,P.
Perapion marchicum<br>(Herbst, 1797)	MK891469 [new]<br>1536-PSP<br>658nt	ZFMK-TIS-3296<br>ZFMK-DNA-0155633293	Germany, Saxony-Anhalt (ST), Steimke, Lkr. Salzwedel, N52°36'17" E10°58'27", 70m, 14-Jun-2013, *Rumex acetosella*, sand lean lawn ("Sandmagerrasen"), dipnet, leg. Sprick,P., det. Sprick,P.
Perapion marchicum<br>(Herbst, 1797)	MK891689 [new]<br>1906-PST<br>658nt	ZFMK-TIS-24011<br>ZFMK-DNA-0169170410	Germany, North Rhine-Westphalia (NRW), lower Rhine, 2 km S of Brüggen, Elmpter creek, N51°13'24" E06°11'31", 57m, 03-Sep-2014, beating, leg. Stüben,P., det. Stüben,P.
Perapion marchicum<br>(Herbst, 1797)	MK891071 [new]<br>972-CBR<br>658nt	ZFMK-TIS-3885<br>ZFMK-DNA-0100449104	Luxembourg, 15 km NW of Luxembourg, 3 km NE of Nospelt, N49°41'25" E06°01'21", 350m, 23-Jun-2012, *Rumex acetosella*, sweeping, leg. Braunert,C., det. Braunert,C.
Perapion oblongum<br>(Gyllenhal, 1839)<br>Synonym of *P. curtirostre*!	KC784056 [new]<br>587-PSP<br>658nt	ZFMK-TIS-20371<br>ZFMK-DNA-0100448401	Germany, Lower Saxony (NI), Braunschweig, Hafen, N52°18'53" E10°29'36", 66m, 07-Jun-2011, *Rumex thyrsiflorus*, nutrient-poor grassland, dipnet, leg. Sprick,P., det. Sprick,P.
Perapion violaceum violaceum<br>(Kirby, 1808)	MK891270 [new]<br>1210-JKR<br>658nt	ZFMK-TIS-3546<br>ZFMK-DNA-0100426079	Czech Republic, Moravia, Hruby jesenik, Velka Kotlina, N50°3'18" E17°14'14", 1209m, 23-Aug-2012, *Rumex* sp., sieving, leg. Krátky,J., det. Krátky,J.
Perapion violaceum violaceum<br>(Kirby, 1808)	MK891819 [new]<br>2079-JKR<br>658nt	ZFMK-TIS-23719<br>ZFMK-DNA-0169169654	Czech Republic, Bohemia, Kutrin, N49°49'12.5" E16°03'27", 456m, 08-Mai-2014, *Rumex* sp., sweeping, leg. Krátky,J., det. Krátky,J.
Perapion violaceum violaceum<br>(Kirby, 1808)	KC784031 [new]<br>585-PSP<br>658nt	ZFMK-TIS-20369<br>ZFMK-DNA-0100446695	Germany, Lower Saxony (NI), Braunschweig, Hafen, N52°18'53" E10°29'36", 66m, 07-Jun-2011, *Rumex thyrsiflorus*, nutrient-poor grassland, dipnet, leg. Sprick,P., det. Sprick,P.
Perapion violaceum violaceum<br>(Kirby, 1808)	MK890942 [new]<br>811-PST<br>658nt	ZFMK-TIS-3724<br>ZFMK-DNA-0100414280	Germany, Rhineland-Palatinate (RLP), N of Treis-Karden, Brohl, N50°13'22" E07°16'27", 255m, 10-Jun-2012, *Rumex*, roadside ditch, beating, leg. Stüben,P., det. Stüben,P.
Perapion violaceum violaceum<br>(Kirby, 1808)	MK891055 [new]<br>955-CBR<br>658nt	ZFMK-TIS-3868<br>ZFMK-DNA-0100449656	Luxembourg, 10 km E of Luxembourg, 1 km SW of Mensdorf, N49°39'00" E06°17'40", 240m, 21-Jun-2012, *Rumex acetosa*, beating, leg. Braunert,C., det. Braunert,C.

**Name**<br>**Authority**<br>**Additional Information**	**GenBank Acc No (Ref.)**<br>**Specimen ID**<br>**Sequence Length**	**ZFMK Tissue ID**<br>**ZFMK DNA Sample ID**<br>**(SDEI DNA Sample ID)**	**Locality, GPS, Collection Date, Plant, Collector, Identifier**
Perioxyonyx splendidus<br>(C. Brisout de Barneville, 1890)	MK890814 [new]<br>193-PST<br>658nt	ZFMK-TIS-2D100438689<br>ZFMK-DNA-0100438334	Morocco, SW of Al-Hoceima, Rif Mts., Massif des Bokkoyas, Torres, N35°09'21" W04°19'30", 15m, 24-Mai-2011, *Ephedra*, beating, leg. Stüben,P., det. Behne,L.
Perioxyonyx splendidus<br>(C. Brisout de Barneville, 1890)	MK891494 [new]<br>1577-JKR<br>658nt	ZFMK-TIS-3625<br>ZFMK-DNA-0155635652	Morocco, Taroudant, N of Aoulouz, N30°41'47" W08°09'10", 708m, 16-Apr-2013, *Ephedra*, beating, leg. Krátky,J., det. Krátky,J.
Peritelus familiaris<br>Boheman, 1834	KC783931 [new]<br>330-JKR<br>658nt	ZFMK-TIS-20116<br>ZFMK-DNA-0100438744	Slovakia, Komarno, Marcelova env., PR Masan, N47°46'4.755" E18°19'13.531", 121m, 18-Mai-2011, sweeping, leg. Krátky,J., det. Krátky,J.
Peritelus familiaris<br>Boheman, 1834	MK892226 [new]<br>2524-PSP<br>658nt	ZFMK-TIS-23496<br>ZFMK-DNA-0171661749	Slovakia, Nitra, SW of Buc, N47°46'56" E18°25'10", 110m, 18-Mai-2014, *herb layer*, leg. Sprick,P., det. Sprick,P.
Peritelus sphaeroides<br>Germar, 1824	KC783902 [new]<br>202-PSP<br>658nt	ZFMK-TIS-2D100439169<br>ZFMK-DNA-0100438508	Germany, Lower Saxony (NI), Braunschweig, Riddagshausen, JKI area, N52°16'38" E10°33'51", 76m, 08-Apr-2011, *Juniperus virginiana*, beating, leg. Sprick,P., det. Sprick,P.
Peritelus sphaeroides<br>Germar, 1824	MK891014 [new]<br>910-CBR<br>658nt	ZFMK-TIS-3823<br>ZFMK-DNA-0100449701	Luxembourg, 10 km E of Luxembourg, 2 km S of Mensdorf, N49°38'09" E06°17'48", 330m, 08-Mai-2011, *Quercus*, beating, leg. Braunert,C., det. Braunert,C.
Philopedon plagiatum<br>(Schaller, 1783)	MK890821 [40]<br>210-PSP<br>658nt	ZFMK-TIS-2D100438511<br>ZFMK-DNA-0100439824	Germany, Lower Saxony (NI), Hannover, Vahrenheide, Kugelfangtrift, N52°25'18" E09°45'07", 50m, 10-Apr-2011, *Plantago lanceolata*, collecting by hand, leg. Sprick,P., det. Sprick,P.
Phloeophagus lignarius<br>(Marsham, 1802)	MK891391 [new]<br>1450-JKR<br>616nt	ZFMK-TIS-4219<br>ZFMK-DNA-0155628488	Slovakia, Zlate Moravce, Obyce - Jadova, N48°26'25" E18°30'02", 450m, 13-Okt-2013, *Fagus*, individual collecting by hand, leg. Krátky,J., det. Krátky,J.
Phrissotrichum tubiferum<br>(Gyllenhal, 1833)	MK891035 [new]<br>932-CBR<br>658nt	ZFMK-TIS-3845<br>ZFMK-DNA-0100449679	France, Dep. Var, Gonfaron, Massif des Maures, N48°17'38" E06°19'00", 400m, 08-Apr-2012, *Cystus*, beating, leg. Braunert,C., det. Braunert,C.
Phrissotrichum tubiferum<br>(Gyllenhal, 1833)	MK890813 [new]<br>172-PST<br>658nt	ZFMK-TIS-2D100440914<br>ZFMK-DNA-0100438352	Morocco, Atlas Mts., SW of Taza, Jbel Tazzeka near Bat-Bou-Idir, N34°03'09" W04°13'14", 1214m, 18-Mai-2011, *Cistus*, beating, leg. Stüben,P., det. Stüben,P./vid. Behne,L.
Phrissotrichum tubuliferum<br>(Wollaston, 1864)	MK347548 [40]<br>2961-PST<br>658nt	(SDEI-DNA-2961-PST)	Spain, Canary Islands, El Hierro, W Sabinosa, near Montana Escoba, N27°44'54" W18°08'32", 303m, 11-Apr-2016, leg. Stüben & Schütte, det. Stüben,P.
Phrissotrichum tubuliferum<br>(Wollaston, 1864)	KC783757 [40]<br>46-PST<br>655nt	ZFMK-TIS-2D100447008<br>ZFMK-DNA-0100438080	Spain, Canary Islands, Gran Canaria, San Bartolome, N27°54'51" W15°34'26", 946m, 09-Jan-2011, *Cistus monspeliensis*, beating, leg. Stüben,P., det. Stüben,P.
Phrissotrichum tubuliferum<br>(Wollaston, 1864)	KC783788 [40]<br>79-PST<br>658nt	ZFMK-TIS-2D100446969<br>ZFMK-DNA-0100438014	Spain, Canary Islands, La Gomera, E of Chipude, Fayal-Brezal, N28°6'26" W17°15'33", 1247m, 14-Feb-2011, *Cistus monspeliensis*, beating, leg. Stüben,P., det. Stüben,P.
Phrissotrichum tubuliferum<br>(Wollaston, 1864)	MK891487 [40]<br>1564-JKR<br>658nt	ZFMK-TIS-3612<br>ZFMK-DNA-0155635658	Spain, Canary Islands, La Palma, Montana Tagoja, N28°43'16" W17°47'07", 1052m, 30-Jan-2013, *Cistus*, beating, leg. Krátky,J., det. Krátky,J.
Phrissotrichum tubuliferum<br>(Wollaston, 1864)	MK891624 [40]<br>1736-PST<br>658nt	ZFMK-TIS-24454<br>ZFMK-DNA-0155622454	Spain, Canary Islands, La Palma, E of Barlovento, above El Tablado, N28°49'33" W17°52'48", 615m, 02-Jan-2014, *Cistus*, beating, leg. Stüben,P., det. Stüben,P.

**Name**<br>**Authority**<br>**Additional Information**	**GenBank Acc No (Ref.)**<br>**Specimen ID**<br>**Sequence Length**	**ZFMK Tissue ID**<br>**ZFMK DNA Sample ID**<br>**(SDEI DNA Sample ID)**	**Locality, GPS, Collection Date, Plant, Collector, Identifier**
Phrissotrichum tubuliferum<br>(Wollaston, 1864)	KC784302 [40]<br>733-PST<br>658nt	ZFMK-TIS-3070<br>ZFMK-DNA-0100448159	Spain, Canary Islands, Tenerife, 2 km S of Los Silos, Barranco de Blas, N28°21'31" W16°49'36", 211m, 29-Jan-2012, *Cistus*, beating, leg. Stüben,P., det. Stüben,P.
Phrydiuchus augusti<br>Colonnelli, 2003	KC784109 [new]<br>502-RST<br>634nt	ZFMK-TIS-20287<br>ZFMK-DNA-0100448477	Czech Republic, Moravia mer., 11 km SE of Znojmo, Jecmenist? near Dyjakovicky, N48°45'10.63" E16°8'11.16", 280m, 27-Mai-2011, *Salvia nemorosa*, sweeping, leg. Stejskal,R., det. Stejskal,R.
Phrydiuchus augusti<br>Colonnelli, 2003	MK891209 [new]<br>1127-JKR<br>658nt	ZFMK-TIS-3463<br>ZFMK-DNA-0100449581	Slovakia, Nove Zamky, Kamenin, N47°51'51" E18°38'09", 110m, 11-Mai-2012, *Salvia nemorosa*, beating, leg. Krátky,J., det. Krátky,J.
Phrydiuchus cf. *topiarius*	KC784081 [new]<br>542-RST<br>658nt	ZFMK-TIS-20327<br>ZFMK-DNA-0100448437	Czech Republic, Moravia mer., 11 km SE of Znojmo, Jecmenist? near Dyjakovicky, N48°45'10.63" E16°8'11.16", 280m, 03-Sep-2011, *Salvia nemorosa*, sieving, leg. Stejskal,R., det. Stejskal,R.
Phrydiuchus cf. *topiarius*	MK891888 [new]<br>2153-JKR<br>658nt	ZFMK-TIS-23793<br>ZFMK-DNA-0169169580	Slovakia, Nove Zamky, Jursky Chlm, N47°48'04" E18°31'27", 114m, 19-Mai-2014, *Salvia nemorosa*, collecting by hand, leg. Krátky,J., det. Krátky,J.
Phrydiuchus cf. *topiarius*	MK892258 [new]<br>2558-PSP<br>658nt	ZFMK-TIS-23532<br>ZFMK-DNA-0171661994	Slovakia, Nitra, Muzla, N47°48'00" E18°31'55", 114m, 19-Mai-2014, *Salvia nemorosa*, leg. Sprick,P., det. Sprick,P.
Phrydiuchus quijote<br>Sánchez-Ruiz & Alonso-Zarazaga, 1995	MK891251 [new]<br>1182-JKR<br>658nt	ZFMK-TIS-3518<br>ZFMK-DNA-0100426236	Spain, Andalucia, Sierra del Albunuelas, 8 km S of Albunuelas, N36°53'10" W03°43'49", 1312m, 11-Jun-2012, *Salvia aethiopis*, individual collecting by hand, leg. Krátky,J., det. Krátky,J.
Phrydiuchus tau<br>Warner, 1969	MK891889 [new]<br>2154-JKR<br>658nt	ZFMK-TIS-23794<br>ZFMK-DNA-0169169568	Slovakia, Nove Zamky, Jursky Chlm, N47°48'04" E18°31'27", 114m, 19-Mai-2014, *Salvia aethiopis*, collecting by hand, leg. Krátky,J., det. Krátky,J.
Phrydiuchus topiarius<br>(Germar, 1824)	KC783923 [new]<br>362-JKR<br>658nt	ZFMK-TIS-20148<br>ZFMK-DNA-0100438728	Czech Republic, Bohemia or., Horineves, N50°18'52.155" E15°46'30.012", 230m, 25-Jun-2011, *Salvia pratensis*, beating, leg. Krátky,J., det. Krátky,J.
Phrydiuchus topiarius<br>(Germar, 1824)	MK892143 [new]<br>2430-JKR<br>658nt	ZFMK-TIS-26007<br>ZFMK-DNA-0171600655	Slovakia, Nitra, Koli?any, Koli?ansky vrch, N48°20'15.5" E18°10'47", 261m, 21-Sep-2014, *Salvia pratensis*, sweeping, leg. Krátky,J., det. Krátky,J.
Phyllobius alpinus<br>Stierlin, 1859	MK892051 [new]<br>2327-JKR<br>658nt	ZFMK-TIS-24258<br>ZFMK-DNA-0169170945	Slovakia, Tatry Mts., Bujaci vrch, N49°14'10" E20°14'27", 1914m, 06-Jul-2014, *Alchemilla* sp., collecting by hand, leg. Krátky,J., det. Krátky,J.
Phyllobius arborator<br>(Herbst, 1797)	MK891999 [new]<br>2270-JKR<br>658nt	ZFMK-TIS-24201<br>ZFMK-DNA-0169170984	Austria, Styria, Hochschwab, Stallmauer, N47°35'29" E15°02'20", 1260m, 27-Jun-2014, *Acer pseudoplatanus*, collecting by hand, leg. Krátky,J., det. Krátky,J.
Phyllobius arborator<br>(Herbst, 1797)	MK892269 [new]<br>2569-PSP<br>658nt	ZFMK-TIS-23543<br>ZFMK-DNA-0171661990	Germany, Saxony-Anhalt (ST), Harz National Park, Feuersteinwiesen, N51°45'23" E10°41'14", 622m, 11-Jun-2014, *Crataegus monogyna*, leg. Sprick,P., det. Sprick,P.
Phyllobius arborator<br>(Herbst, 1797)	KC784206 [new]<br>430-RGO<br>658nt	ZFMK-TIS-20216<br>ZFMK-DNA-0100449400	Poland, Tarnogóra, N50°41'11.6" E23°7'13.4", 249m, 11-Jun-2011, leg. Gosik,R., det. Gosik,R.
Phyllobius arborator<br>(Herbst, 1797)	MK891368 [new]<br>1419-JKR<br>658nt	ZFMK-TIS-4188<br>ZFMK-DNA-0155627298	Slovakia, Vysoke Tatry Mts., Dolina siedmich prame?ov, N49°13'38" E20°16'34", 1448m, 30-Jun-2013, sweeping, leg. Krátky,J., det. Krátky,J.

**Name**<br>**Authority**<br>**Additional Information**	**GenBank Acc No (Ref.)**<br>**Specimen ID**<br>**Sequence Length**	**ZFMK Tissue ID**<br>**ZFMK DNA Sample ID**<br>**(SDEI DNA Sample ID)**	**Locality, GPS, Collection Date, Plant, Collector, Identifier**
Phyllobius argentatus<br>(Linne, 1758)	MK891544 [new]<br>1634-JKR<br>658nt	ZFMK-TIS-3682<br>ZFMK-DNA-0155635584	Czech Republic, Bohemia, Zalibene, N49°43'48" E15°54'35", 639m, 29-Mai-2013, sweeping, leg. Krátky,J., det. Krátky,J.
Phyllobius argentatus<br>(Linnaeus, 1758)	MK891577 [new]<br>262-PSP<br>658nt	ZFMK-TIS-2D100439108<br>ZFMK-DNA-0155630456	Germany, Lower Saxony (NI), Helstorfer Moor, Resse, Hannover region, N52°31'50" E09°36'35", 43m, 14-Mai-2011, *Corylus avellana*, edge of broad-leaved forest (east side), beating, leg. Sprick,P., det. Sprick,P.
Phyllobius argentatus<br>(Linne, 1758)	MK892171 [new]<br>2462-PSP<br>658nt	ZFMK-TIS-23434<br>ZFMK-DNA-0171661800	Germany, Saxony-Anhalt (ST), Harz National Park, Ilsenburg, Köhlerholz, N51°53'04" E10°40'06", 296m, 29-Mai-2013, *Quercus petraea*, beating, leg. Sprick,P., det. Sprick,P.
Phyllobius argentatus<br>(Linnaeus, 1758)	MK891007 [new]<br>901-CBR<br>658nt	ZFMK-TIS-3814<br>ZFMK-DNA-0100449710	Luxembourg, 10 km NE of Mersch, 2 km NE of Nommern, N49°48'15" E06°11'16", 340m, 01-Mai-2011, *Quercus*, beating, leg. Braunert,C., det. Braunert,C.
Phyllobius betulinus<br>(Bechstein & Scharffenberg, 1805)	MK890869 [new]<br>467-PSP<br>658nt	ZFMK-TIS-20252<br>ZFMK-DNA-0100449372	Germany, Hesse (HE), Wiesbaden-Erbenheim, N50°03'02" E08°18'13", 274m, 31-Mai-2011, *Rosa canina*, shrubbery an railway embankment, beating, leg. Sprick,P., det. Sprick,P.
Phyllobius betulinus<br>(Bechstein & Scharffenberg, 1805)	MK890941 [new]<br>810-PST<br>658nt	ZFMK-TIS-3723<br>ZFMK-DNA-0100414281	Germany, Rhineland-Palatinate (RLP), N of Treis-Karden, Brohl, N50°13'22" E07°16'27", 255m, 10-Jun-2012, *Rosaceae*, roadside ditch, beating, leg. Stüben,P., det. Stüben,P.
Phyllobius betulinus<br>(Bechstein & Scharffenberg, 1805)	MK892322 [new]<br>2623-PSP<br>658nt	ZFMK-TIS-23597<br>ZFMK-DNA-0171661936	Germany, Bavaria (BY), Unterfranken, Ebern, N50°05'07" E10°45'52.5", 318m, 14-Jun-2014, *Crataegus monogyna*, leg. Sprick,P., det. Sprick,P.
Phyllobius betulinus<br>(Bechstein & Scharffenberg, 1805)	MK892229 [new]<br>2527-PSP<br>658nt	ZFMK-TIS-23499<br>ZFMK-DNA-0171661746	Slovakia, Nitra, SW of Buc, N47°46'56" E18°25'10", 110m, 18-Mai-2014, *Crataegus* sp., leg. Sprick,P., det. Sprick,P.
Phyllobius brenskei<br>Schilsky, 1911	JX448426 [new]<br>672-FBA<br>658nt	ZFMK-TIS-c672-FBA<br>ZFMK-DNA-0100448279	Greece, Peloponnese, Messinia, W of Mt. Taygetos, E of Saidona, N36°53'01" E22°17'07", 680m, 23-Mai-2011, leg. Bahr,F., det. Bahr,F.
Phyllobius brevis<br>Gyllenhal, 1834	MK892230 [new]<br>2528-PSP<br>658nt	ZFMK-TIS-23500<br>ZFMK-DNA-0171661745	Slovakia, Nitra, SW of Buc, N47°46'56" E18°25'10", 110m, 18-Mai-2014, *herb layer*, leg. Sprick,P., det. Sprick,P.
Phyllobius emgei<br>Stierlin, 1887	JX448425 [new]<br>671-FBA<br>658nt	ZFMK-TIS-c671-FBA<br>ZFMK-DNA-0100448596	Greece, Peloponnese, Messinia, W of Mt. Taygetos, E of Daidona, N36°52'59" E22°17'25", 800m, 19-Mai-2011, leg. Bahr,F., det. Bahr,F.
Phyllobius glaucus<br>(Scopoli, 1763)	MK892012 [new]<br>2285-JKR<br>658nt	ZFMK-TIS-24216<br>ZFMK-DNA-0169170980	Austria, Styria, Hochschwab, Stallmauer, N47°35'29" E15°02'20", 1260m, 27-Jun-2014, mountain forest, sweeping, leg. Krátky,J., det. Krátky,J.
Phyllobius glaucus<br>(Scopoli, 1763)	MK891808 [new]<br>2067-JKR<br>658nt	ZFMK-TIS-23707<br>ZFMK-DNA-0169170441	Czech Republic, Bohemia, Ujezd u Sezemic, N50°07'7.5" E15°51'37", 234m, 08-Mai-2014, sweeping, leg. Krátky,J., det. Krátky,J.
Phyllobius glaucus<br>(Scopoli, 1763)	JX448417 [new]<br>255-PSP<br>658nt	ZFMK-TIS-2D100439116<br>ZFMK-DNA-0100439788	Germany, Schleswig-Holstein (SH), Bullenkuhlen, Lkr. Pinneberg, N53°46'27" E09°45'07", 50m, 11-Mai-2011, *Corylus avellana*, woody plants on roadside, beating, leg. Sprick,P., det. Sprick,P.
Phyllobius glaucus<br>(Scopoli, 1763)	MK891098 [new]<br>1002-PSP<br>658nt	ZFMK-TIS-3147<br>ZFMK-DNA-0100426257	Germany, Lower Saxony (NI), Aerzen-Ahorn, Lkr. Hameln-Pyrmont, N52°03'17" E09°13'14", 157m, 05-Mai-2012, *Alnus glutinosa*, bog forest, dipnet, leg. Sprick,P., det. Sprick,P.

**Name**<br>**Authority**<br>**Additional Information**	**GenBank Acc No (Ref.)**<br>**Specimen ID**<br>**Sequence Length**	**ZFMK Tissue ID**<br>**ZFMK DNA Sample ID**<br>**(SDEI DNA Sample ID)**	**Locality, GPS, Collection Date, Plant, Collector, Identifier**
Phyllobius glaucus<br>(Scopoli, 1763)	KC784205 [new]<br>429-RGO<br>658nt	ZFMK-TIS-20215<br>ZFMK-DNA-0100449399	Poland, Kiry, Tatra Mountains, N49°16'26" E19°52'07", 942m, 06-Jul-2011, leg. Gosik,R., det. Gosik,R.
Phyllobius glaucus<br>(Scopoli, 1763)	MK890985 [new]<br>876-CBR<br>658nt	ZFMK-TIS-3789<br>ZFMK-DNA-0100414226	Switzerland, 4 km SW of Zernez, Prazet, N46°40'12" E10°02'51", 1650m, 22-Jul-2011, beating, leg. Braunert,C., det. Braunert,C.
Phyllobius intrusus<br>Kôno, 1948	JX448423 [new]<br>595-PSP<br>658nt	ZFMK-TIS-c595-PSP<br>ZFMK-DNA-0100448672	Germany, Lower Saxony (NI), Bad Zwischenahn, Aschhausen, N53°12'00" E08°04'00", 11m, 10-Jun-2011, *Thuja occidentalis*, beating, leg. Sprick,P., det. Sprick,P.
Phyllobius maculicornis<br>Germar, 1824	MK891842 [new]<br>2104-JKR<br>658nt	ZFMK-TIS-23744<br>ZFMK-DNA-0169169629	Czech Republic, Moravia, Cejc, PR Spidlaky, N48°55'23.5" E16°57'21", 220m, 10-Mai-2014, sweeping, leg. Krátky,J., det. Krátky,J.
Phyllobius maculicornis<br>Germar, 1824	MK890831 [new]<br>281-PSP<br>658nt	ZFMK-TIS-2D100439079<br>ZFMK-DNA-0100439751	Germany, Lower Saxony (NI), Pattensen, Hannover region, N52°14'35" E09°45'21", 77m, 17-Mai-2011, *Prunus spinosa*, roadside shrub, beating, leg. Sprick,P., det. Sprick,P.
Phyllobius maculicornis<br>Germar, 1824	MK891403 [new]<br>1465-PSP<br>658nt	ZFMK-TIS-3225<br>ZFMK-DNA-0155633364	Germany, Lower Saxony (NI), Harz, St. Andreasberg, Jordanshöhe, N51°42'58" E10°32'06", 670m, 02-Jul-2012, *Sorbus aucuparia*, tree group, beating, leg. Sprick,P., det. Sprick,P.
Phyllobius oblongus<br>(Linnaeus, 1758)	JX448421 [new]<br>279-PSP<br>658nt	ZFMK-TIS-2D100438559<br>ZFMK-DNA-0100439764	Germany, Lower Saxony (NI), Pattensen, Hannover region, N52°14'37" E09°45'26", 77m, 17-Mai-2011, *Corylus avellana, Prunus spinosa*, roadside shrub, beating, leg. Sprick,P., det. Sprick,P.
Phyllobius oblongus<br>(Linnaeus, 1758)	MK891646 [new]<br>1819-PST<br>658nt	ZFMK-TIS-24124<br>ZFMK-DNA-0171624045	Germany, Rhineland-Palatinate (RLP), Ahr valley, Dernau, below Krausberg near Steinbergsmühle, N50°31'40" E07°02'43", 204m, 29-Mai-2014, beating, leg. Stüben,P., det. Stüben,P.
Phyllobius oblongus<br>(Linnaeus, 1758)	KC784036 [new]<br>673-FBA<br>658nt	ZFMK-TIS-20452<br>ZFMK-DNA-0100448225	Greece, Peloponnese, Messinia, Mt. Taygetos, S of Marathos, N36°53'19" E22°17'45", 1000m, 21-Mai-2011, leg. Bahr,F., det. Bahr,F.
Phyllobius oblongus<br>(Linnaeus, 1758)	MK891032 [new]<br>929-CBR<br>658nt	ZFMK-TIS-3842<br>ZFMK-DNA-0100449682	Luxembourg, 10 km E of Luxembourg, 2 km S of Mensdorf, N49°38'09" E06°17'48", 330m, 05-Jun-2012, beating, leg. Braunert,C., det. Braunert,C.
Phyllobius oblongus<br>(Linnaeus, 1758)	MK892240 [new]<br>2538-PSP<br>658nt	ZFMK-TIS-23510<br>ZFMK-DNA-0171661732	Slovakia, Nitra, W of Gbelce, N47°46'56" E18°27'37", 148m, 18-Mai-2014, *Acer campestre*, leg. Sprick,P., det. Sprick,P.
Phyllobius oblongus<br>(Linnaeus, 1758)	MK891324 [new]<br>1327-PST<br>658nt	ZFMK-TIS-4143<br>ZFMK-DNA-0100426148	Spain, Malaga, S of Antequera, Sierra de Chimenea, El Torcal, N36°57'01" W04°33'13", 1198m, 10-Mai-2013, *Quercus ilex*, sieving, leg. Stüben,P. & Schütte,A., det. Stüben,P.
Phyllobius pomaceus<br>Gyllenhal, 1834	KC783976 [new]<br>297-JKR<br>658nt	ZFMK-TIS-20083<br>ZFMK-DNA-0100438968	Czech Republic, Bohemia or., Dritec, N50°7'11.648" E15°48'6.919", 230m, 06-Mai-2011, *Urtica dioica*, sweeping, leg. Krátky,J., det. Krátky,J.
Phyllobius pomaceus<br>Gyllenhal, 1834	MK891780 [new]<br>2039-JKR<br>658nt	ZFMK-TIS-23679<br>ZFMK-DNA-0169170461	Czech Republic, Bohemia, Kosatky, N50°19'23" E14°39'58", 218m, 02-Mai-2014, *Urtica dioica*, sweeping, leg. Krátky,J., det. Krátky,J.
Phyllobius pomaceus<br>Gyllenhal, 1834	JX448416 [new]<br>250-PSP<br>658nt	ZFMK-TIS-2D100439121<br>ZFMK-DNA-0100439793	Germany, Lower Saxony (NI), Essel, Lkr. Soltau-Fallingbostel, N52°41'57" E09°38'37", 25m, 10-Mai-2011, *Urtica dioica*, sand field, dipnet, leg. Sprick,P., det. Sprick,P.

**Name**<br>**Authority**<br>**Additional Information**	**GenBank Acc No (Ref.)**<br>**Specimen ID**<br>**Sequence Length**	**ZFMK Tissue ID**<br>**ZFMK DNA Sample ID**<br>**(SDEI DNA Sample ID)**	**Locality, GPS, Collection Date, Plant, Collector, Identifier**
Phyllobius pomaceus<br>Gyllenhal, 1834	MK891647 [new]<br>1820-PST<br>658nt	ZFMK-TIS-24125<br>ZFMK-DNA-0171624044	Germany, Rhineland-Palatinate (RLP), Ahr valley, Dernau, below Krausberg near Steinbergsmühle, N50°31'40" E07°02'43", 204m, 29-Mai-2014, beating, leg. Stüben,P., det. Stüben,P.
Phyllobius pomaceus<br>Gyllenhal, 1834	MK890855 [new]<br>399-RGO<br>631nt	ZFMK-TIS-20185<br>ZFMK-DNA-0100449440	Poland, ad. Urszulin , N51°22'16" E23°15'04" , 172m, 06-Jun-2011, leg. Gosik,R., det. Gosik,R.
Phyllobius pyri<br>(Linnaeus, 1758)<br>formerly*: P. vespertinus* det. Krátky- Syn!	MK891196 [new]<br>1113-JKR<br>658nt	ZFMK-TIS-3449<br>ZFMK-DNA-0100449598	Czech Republic, Bohemia, Hradec Kralove, Brezhrad, N50°10'45" E15°47'39", 229m, 05-Mai-2012, sweeping, leg. Krátky,J., det. Krátky,J.
Phyllobius pyri<br>(Linnaeus, 1758)<br>formerly: *P. pyri vespertinus* det. Sprick- Syn!	JX448414 [new]<br>208-PSP<br>658nt	ZFMK-TIS-2D100438539<br>ZFMK-DNA-0100439545	Germany, Lower Saxony (NI), Hannover, Vahrenheide, Kugelfangtrift, N52°25'29" E09°45'21", 50m, 10-Apr-2011, *Festuca rubra*, collecting by hand, leg. Sprick,P., det. Sprick,P.
Phyllobius pyri<br>(Linnaeus, 1758)	JX448420 [new]<br>264-PSP<br>658nt	ZFMK-TIS-2D100439110<br>ZFMK-DNA-0100439782	Germany, Lower Saxony (NI), Helstorfer Moor, Resse, Hannover region, N52°31'44" E09°36'32", 43m, 14-Mai-2011, *Betula pubescens*, bog forest, beating, leg. Sprick,P., det. Sprick,P.
Phyllobius pyri<br>(Linnaeus, 1758)	JX448422 [new]<br>578-PSP<br>658nt	ZFMK-TIS-c578-PSP<br>ZFMK-DNA-0100446678	Germany, Saxony-Anhalt (ST), Drömling, Mannhausen, N52°25'31" E11°12'45", 56m, 23-Mai-2011, *Populus tremula*, wayside shrub, beating, leg. Sprick,P., det. Sprick,P.
Phyllobius pyri<br>(Linnaeus, 1758)<br>formerly: *P. vespertinus* det. Sprick- Syn!	MK891107 [new]<br>1012-PSP<br>658nt	ZFMK-TIS-3157<br>ZFMK-DNA-0100426842	Germany, Lower Saxony (NI), Hannover, Stöcken, N52°24'23" E09°39'07", 42m, 07-Mai-2012, *Alopecurus pratensis, Phalaris arundinacea, Arrhenatherum elatius*, beating/dipnet, leg. Sprick,P., det. Behne,L.
Phyllobius pyri<br>(Linnaeus, 1758)	MK892197 [new]<br>2492-PSP<br>658nt	ZFMK-TIS-23464<br>ZFMK-DNA-0171661782	Germany, Saxony-Anhalt (ST), Harz National Park, Drei Annen Hohne, Hohneklippen, Hohnekopf, N51°46'37" E10°41'55", 840m, 24-Apr-2014, *Betula pendula*, beating, leg. Sprick,P., det. Sprick,P.
Phyllobius pyri<br>(Linnaeus, 1758)	MK890992 [new]<br>884-CBR<br>658nt	ZFMK-TIS-3797<br>ZFMK-DNA-0100449727	Luxembourg, 10 km E of Luxembourg, 2 km S of Mensdorf, N49°38'09" E06°17'48", 330m, 11-Apr-2011, beating, leg. Braunert,C., det. Braunert,C.
Phyllobius roboretanus<br>Gredler, 1882	JX448415 [new]<br>236-PSP<br>658nt	ZFMK-TIS-2D100438544<br>ZFMK-DNA-0100439802	Germany, Hesse (HE), Wiesbaden-Erbenheim, N50°03'02" E08°18'16", 274m, 28-Apr-2011, *Crataegus monogyna*, shrubbery, beating, leg. Sprick,P., det. Sprick,P.
Phyllobius roboretanus<br>Gredler, 1882	MK890926 [new]<br>795-PST<br>658nt	ZFMK-TIS-3708<br>ZFMK-DNA-0100414289	Germany, Rhineland-Palatinate (RLP), E of Treis-Karden, Pommern, Pommernbachtal (river valley), N50°10'52" E07°16'42", 116m, 09-Jun-2012, forest path, beating, leg. Stüben,P., det. Stüben,P.
Phyllobius roboretanus<br>Gredler, 1882	MK892326 [new]<br>2627-PSP<br>658nt	ZFMK-TIS-23601<br>ZFMK-DNA-0171661932	Germany, Bavaria (BY), Unterfranken, Ebern, N50°05'07" E10°45'52.5", 318m, 14-Jun-2014, *Prunus spinosa*, leg. Sprick,P., det. Sprick,P.
Phyllobius virideaeris<br>(Laicharting, 1781)	KC783928 [new]<br>345-JKR<br>658nt	ZFMK-TIS-20131<br>ZFMK-DNA-0100438738	Czech Republic, Bohemia or., Hradec Kralove, Placky, N50°13'48.497" E15°49'25.886", 230m, 25-Mai-2011, sweeping, leg. Krátky,J., det. Krátky,J.
Phyllobius virideaeris<br>(Laicharting, 1781)	JX448419 [new]<br>258-PSP<br>658nt	ZFMK-TIS-2D100438504<br>ZFMK-DNA-0100438523	Germany, Lower Saxony (NI), Hannover, Stöcken, Leine wetland, N52°24'12" E09°39'16", 45m, 14-Mai-2011, *Artemisia vulgaris*, roadside, beating, leg. Sprick,P., det. Sprick,P.

**Name**<br>**Authority**<br>**Additional Information**	**GenBank Acc No (Ref.)**<br>**Specimen ID**<br>**Sequence Length**	**ZFMK Tissue ID**<br>**ZFMK DNA Sample ID**<br>**(SDEI DNA Sample ID)**	**Locality, GPS, Collection Date, Plant, Collector, Identifier**
Phyllobius virideaeris<br>(Laicharting, 1781)	MK891019 [new]<br>916-CBR<br>658nt	ZFMK-TIS-3829<br>ZFMK-DNA-0100449690	Germany, North Rhine-Westphalia (NRW), 10 km NW of Wesel, N51°42'52" E06°33'51", 25m, 09-Jun-2011, *Artemisia vulgaris*, beating, leg. Braunert,C., det. Braunert,C.
Phyllobius virideaeris<br>(Laicharting, 1781)	KC784229 [new]<br>400-RGO<br>658nt	ZFMK-TIS-20186<br>ZFMK-DNA-0100449439	Poland, ad. Urszulin , N51°22'16" E23°15'04" , 172m, 06-Jun-2011, leg. Gosik,R., det. Gosik,R.
Phyllobius virideaeris<br>(Laicharting, 1781)	MK891213 [new]<br>1132-JKR<br>658nt	ZFMK-TIS-3468<br>ZFMK-DNA-0100449569	Slovakia, Komarno, Imel, N47°54'34" E18°09'16", 112m, 11-Mai-2012, *Populus alba*, beating, leg. Krátky,J., det. Krátky,J.
Phyllobius virideaeris<br>(Laicharting, 1781)	MK892228 [new]<br>2526-PSP<br>658nt	ZFMK-TIS-23498<br>ZFMK-DNA-0171661747	Slovakia, Nitra, SW of Buc, N47°46'56" E18°25'10", 110m, 18-Mai-2014, *Strauchschicht*, leg. Sprick,P., det. Sprick,P.
Phyllobius viridicollis<br>(Fabricius, 1792)	MK892018 [new]<br>2291-JKR<br>658nt	ZFMK-TIS-24222<br>ZFMK-DNA-0169170974	Austria, Kaernten, Petzen, N46°31'01" E14°46'15", 1698m, 28-Jun-2014, mountain meadow, sweeping, leg. Krátky,J., det. Krátky,J.
Phyllobius viridicollis<br>(Fabricius, 1792)	MK891817 [new]<br>2077-JKR<br>658nt	ZFMK-TIS-23717<br>ZFMK-DNA-0169169656	Czech Republic, Bohemia, Kutrin, N49°49'12.5" E16°03'27", 456m, 08-Mai-2014, sweeping, leg. Krátky,J., det. Krátky,J.
Phyllobius viridicollis<br>(Fabricius, 1792)	JX448418 [new]<br>256-PSP<br>658nt	ZFMK-TIS-2D100439115<br>ZFMK-DNA-0100439787	Germany, Lower Saxony (NI), Berkhof, Hannover region, N52°36'41" E09°43'59", 36m, 11-Mai-2011, *Cytisus scoparius*, dry-warm edge of the forest, beating, leg. Sprick,P., det. Sprick,P.
Phytobius leucogaster<br>(Marsham, 1802)	KC783968 [new]<br>369-JKR<br>629nt	ZFMK-TIS-20155<br>ZFMK-DNA-0100438937	Czech Republic, Bohemia or., Steblova, Oplatil pond, N50°6'15.584" E15°44'42.978", 230m, 29-Jun-2011, *Myriophyllum spicatum*, hand-collecting, leg. Krátky,J., det. Krátky,J.
Pissodes castaneus<br>(DeGeer, 1775)	MK891464 [new]<br>1531-PSP<br>658nt	ZFMK-TIS-3291<br>ZFMK-DNA-0155633298	Germany, Lower Saxony (NI), Berkhof, Hannover region, N52°36'48" E09°43'56", 36m, 16-Aug-2011, *Pinus sylvestris*, pine forest, beating, leg. Sprick,P., det. Sprick,P.
Pissodes castaneus<br>(DeGeer, 1775)	MK892386 [40]<br>2847-PST<br>658nt	ZFMK-TIS-4242<br>ZFMK-DNA-FC17941542	Portugal, Madeira, Sao Vincente, Rota da Cal, N32°47'51" W17°01'22", 415m, 30-Nov-2015, *Pinus*, beating, leg. Stüben,P. & Schütte,A., det. Stüben,P.
Pissodes castaneus<br>(DeGeer, 1775)	MK892251 [new]<br>2550-PSP<br>658nt	ZFMK-TIS-23524<br>ZFMK-DNA-0171662009	Slovakia, Nitra, Cenkov, N47°46'03" E18°31'09", 109m, 19-Mai-2014, *Pinus sylvestris*, leg. Sprick,P., det. Sprick,P.
Pissodes castaneus<br>(DeGeer, 1775)	MK891302 [new]<br>1283-PST<br>658nt	ZFMK-TIS-4099<br>ZFMK-DNA-0100426200	Spain, Community of Madrid, NE of Montejo de la Sierra, Hayedo de Montejo, NE of Hiruela, Puerto de la Hiruela, N41°04'02" W03°28'28", 1478m, 02-Mai-2013, *Pinus*, hand collecting, leg. Stüben,P. & Schütte,A., det. Stüben,P.
Pissodes pini<br>(Linnaeus, 1758)	MK891085 [new]<br>988-PSP<br>632nt	ZFMK-TIS-3133<br>ZFMK-DNA-0100426866	Germany, Lower Saxony (NI), near Hannover, Berkhof, N52°36'48" E09°43'56", 36m, 28-Apr-2012, *Pinus sylvestris*, pine forest, beating, leg. Sprick,P., det. Sprick,P.
Pissodes piniphilus<br>(Herbst, 1797)	MK891143 [new]<br>1048-PSP<br>658nt	ZFMK-TIS-3193<br>ZFMK-DNA-0100426811	Germany, Lower Saxony (NI), near Hannover, Brelingen, Klagesberg, N52°34'02" E09°41'14", 92m, 02-Jun-2012, *Pinus sylvestris*, mixed deciduous woodland, beating, leg. Sprick,P., det. Sprick,P.
Platystomos albinus<br>(Linnaeus, 1758)	MK892217 [new]<br>2513-PSP<br>658nt	ZFMK-TIS-23485<br>ZFMK-DNA-0171661755	Germany, Lower Saxony (NI), Harz National Park, NW of Herzberg, Mühlenberg, N51°40'45" E10°19'10", 262m, 12-Mai-2014, *Quercus robur*, leg. Sprick,P., det. Sprick,P.

**Name**<br>**Authority**<br>**Additional Information**	**GenBank Acc No (Ref.)**<br>**Specimen ID**<br>**Sequence Length**	**ZFMK Tissue ID**<br>**ZFMK DNA Sample ID**<br>**(SDEI DNA Sample ID)**	**Locality, GPS, Collection Date, Plant, Collector, Identifier**
Pleurodirus carinula<br>(Olivier, 1807)	MK890951 [new]<br>826-PST<br>658nt	ZFMK-TIS-3739<br>ZFMK-DNA-0100414272	Portugal, Minho, NW of Ponte de Lima, Vila Nova de Cerveira, Rio Coura, N41°52'27" W08°41'57", 123m, 07-Mai-2012, beating, leg. Stüben,P., det. Stüben,P.
Polydius hispanicus<br>(Herbst, 1797)	MK890947 [new]<br>822-PST<br>658nt	ZFMK-TIS-3735<br>ZFMK-DNA-0100414268	Portugal, Minho, Ponte de Lima, Rio Lima, N41°45'48" W08°35'40", 13m, 06-Mai-2012, beating, leg. Stüben,P., det. Stüben,P.
Polydrusus abeillei<br>(Desbrochers, 1869)	MK891553 [new]<br>1644-JKR<br>658nt	ZFMK-TIS-3692<br>ZFMK-DNA-0155635594	France, Alpes-Marritimes, N of Coursegoules, N43°47'49" E07°03'09", 1366m, 11-Jun-2013, sweeping, leg. Krátky,J., det. Krátky,J.
Polydrusus aeratus<br>(Gravenhorst, 1807)	MK892000 [new]<br>2271-JKR<br>658nt	ZFMK-TIS-24202<br>ZFMK-DNA-0169170985	Austria, Styria, Hochschwab, Stallmauer, N47°35'29" E15°02'20", 1260m, 27-Jun-2014, mountain forest, sweeping, leg. Krátky,J., det. Krátky,J.
Polydrusus aeratus<br>(Gravenhorst, 1807)	MK891414 [new]<br>1476-PSP<br>658nt	ZFMK-TIS-3236<br>ZFMK-DNA-0155633362	Germany, Lower Saxony (NI), Harz National Park, Oder-pond, N51°46'16" E10°32'03", 734m, 02-Jul-2012, *Picea abies*, spruce forest, beating, leg. Sprick,P., det. Sprick,P.
Polydrusus amoenus<br>(Germar, 1824)	MK891402 [new]<br>1464-PSP<br>658nt	ZFMK-TIS-3928<br>ZFMK-DNA-0155633365	Germany, Lower Saxony (NI), Harz, St. Andreasberg, Jordanshöhe, N51°42'56" E10°32'19", 679m, 02-Jul-2012, *Rubus idaeus*, perennials corridor, beating, leg. Sprick,P., det. Sprick,P.
Polydrusus amoenus<br>(Germar, 1824)	MK891369 [new]<br>1420-JKR<br>658nt	ZFMK-TIS-4189<br>ZFMK-DNA-0155628506	Slovakia, Vysoke Tatry Mts., Dolina siedmich prame?ov, N49°13'38" E20°16'34", 1448m, 30-Jun-2013, sweeping, leg. Krátky,J., det. Krátky,J.
Polydrusus amoenus<br>(Germar, 1824)	MK891061 [new]<br>962-CBR<br>658nt	ZFMK-TIS-3875<br>ZFMK-DNA-0100449640	Switzerland, 4 km SW of Zernez, Prazet, N46°40'12" E10°02'51", 1650m, 22-Jul-2011, beating, leg. Braunert,C., det. Braunert,C.
Polydrusus angustus<br>(Lucas, 1854)	MK891707 [new]<br>1931-FBA<br>658nt	ZFMK-TIS-23322<br>ZFMK-DNA-0169170352	Greece, Rhodes Isl., Kalithea env. Beach, N36°22'00" E28°14'00", 0m, 24-Apr-2014, leg. Winkelmann,H., det. Winkelmann,H.
Polydrusus cervinus<br>(Linnaeus, 1758)	KC783998 [new]<br>254-PSP<br>627nt	ZFMK-TIS-2D100439117<br>ZFMK-DNA-0100439789	Germany, Schleswig-Holstein (SH), Bullenkuhlen, Lkr. Pinneberg, N53°46'27" E09°45'01", 50m, 11-Mai-2011, *Quercus robur*, woody plants on roadside, beating, leg. Sprick,P., det. Sprick,P.
Polydrusus cervinus<br>(Linnaeus, 1758)	MK891158 [new]<br>1065-PSP<br>658nt	ZFMK-TIS-3210<br>ZFMK-DNA-0100426799	Germany, Lower Saxony (NI), Langeoog, N53°45'05" E07°32'24", 3m, 16-Jun-2012, *Betula pendula*, small dune valley, beating, leg. Sprick,P., det. Sprick,P.
Polydrusus cervinus<br>(Linnaeus, 1758)	MK891049 [new]<br>948-CBR<br>658nt	ZFMK-TIS-3861<br>ZFMK-DNA-0100449081	Luxembourg, 8 km N of Vianden, 3 km E of Wahlhausen, N49°58'46" E06°9'32", 330m, 28-Mai-2012, leg. Braunert,C., det. Braunert,C.
Polydrusus cf. *impressifrons*	KC783858 [new]<br>158-PST<br>658nt	ZFMK-TIS-2D100440269<br>ZFMK-DNA-0100438157	Morocco, SW of Berkane near Taforalt, Beni Snassen Mts., N34°47'52" W02°25'31" , 839m, 15-Mai-2011, *Quercus ilex*, beating, leg. Stüben,P., det. Stüben,P./vid. Behne,L.
Polydrusus confluens<br>Stephens, 1831	MK891830 [new]<br>2091-JKR<br>658nt	ZFMK-TIS-23731<br>ZFMK-DNA-0169169649	Czech Republic, Moravia, Bucovice, PR Sevy, N49°08'05" E16°58'19", 249m, 09-Mai-2014, *Genista* sp., sweeping, leg. Krátky,J., det. Krátky,J.
Polydrusus confluens<br>Stephens, 1831	MK892362 [new]<br>905-CBR<br>658nt	ZFMK-TIS-3818<br>ZFMK-DNA-0171661893	Luxembourg, 10 km NW of Ettelbruck, 1 km E of "Goebelsmuehle" mill, N49°55'15" E06°03'34", 320m, 07-Mai-2011, *Cytisus scoparius*, beating, leg. Braunert,C., det. Braunert,C.

**Name**<br>**Authority**<br>**Additional Information**	**GenBank Acc No (Ref.)**<br>**Specimen ID**<br>**Sequence Length**	**ZFMK Tissue ID**<br>**ZFMK DNA Sample ID**<br>**(SDEI DNA Sample ID)**	**Locality, GPS, Collection Date, Plant, Collector, Identifier**
Polydrusus corruscus<br>Germar, 1824	MK891240 [new]<br>1160-JKR<br>658nt	ZFMK-TIS-3496<br>ZFMK-DNA-0100449154	Hungary, Jasz-Nagykun-Szolnok megye, Tiszaroff, N47°21'55" E20°27'32", 87m, 25-Mai-2012, sweeping, leg. Krátky,J., det. Krátky,J.
Polydrusus flavipes<br>(DeGeer, 1775)	MK891970 [new]<br>2241-JKR<br>658nt	ZFMK-TIS-23979<br>ZFMK-DNA-0169170546	Slovakia, Kezmarok, Busovce, N49°13'23.5" E20°28'32", 600m, 21-Jun-2014, leg. Benedikt,S., det. Benedikt,S.
Polydrusus formosus<br>(Mayer, 1779)	MK891829 [new]<br>2090-JKR<br>658nt	ZFMK-TIS-23730<br>ZFMK-DNA-0169169648	Czech Republic, Moravia, Bucovice, PR Sevy, N49°08'05" E16°58'19", 249m, 09-Mai-2014, sweeping, leg. Krátky,J., det. Krátky,J.
Polydrusus formosus<br>(Mayer, 1779)	KC784165 [new]<br>596-PSP<br>658nt	ZFMK-TIS-20379<br>ZFMK-DNA-0100448671	Germany, Lower Saxony (NI), Bad Zwischenahn, Aschhausen, N53°12'00" E08°04'00", 11m, 10-Jun-2011, *Carpinus betulus*, beating, leg. Sprick,P., det. Sprick,P.
Polydrusus formosus<br>(Mayer, 1779)	MK890927 [new]<br>796-PST<br>639nt	ZFMK-TIS-3709<br>ZFMK-DNA-0100414290	Germany, Rhineland-Palatinate (RLP), E of Treis-Karden, Pommern, Pommernbachtal (river valley), N50°10'52" E07°16'42", 116m, 09-Jun-2012, forest path, beating, leg. Stüben,P., det. Stüben,P.
Polydrusus formosus<br>(Mayer, 1779)	MK891426 [new]<br>1489-PSP<br>658nt	ZFMK-TIS-3249<br>ZFMK-DNA-0155633340	Germany, North Rhine-Westphalia (NRW), Paderborn, Wewer, Ziegenberg, N51°41'21" E08°42'40", 143m, 15-Jul-2012, *Corylus avellana*, wayside, beating, leg. Sprick,P., det. Sprick,P.
Polydrusus formosus<br>(Mayer, 1779)	MK892289 [new]<br>2590-PSP<br>658nt	ZFMK-TIS-23564<br>ZFMK-DNA-0171661978	Germany, Bavaria (BY), Unterfranken, Ebern, N50°05'22" E10°46'02", 327m, 13-Jun-2014, *Salix caprea*, leg. Sprick,P., det. Sprick,P.
Polydrusus impar<br>Des Gozis, 1882	KC784043 [new]<br>599-PSP<br>658nt	ZFMK-TIS-20382<br>ZFMK-DNA-0100448268	Germany, Lower Saxony (NI), Königsförde, Lkr. Hameln-Pyrmont, N52°03'58" E09°16'17", 92m, 12-Jun-2011, *Picea abies*, house garden, beating, leg. Sprick,P., det. Sprick,P.
Polydrusus impressifrons<br>Gyllenhal, 1834	KC784044 [new]<br>600-PSP<br>658nt	ZFMK-TIS-20383<br>ZFMK-DNA-0100448280	Germany, Lower Saxony (NI), Koldingen, Hannover region, Leine river, N52°16'30" E09°48'31", 57m, 12-Jun-2011, *Salix viminalis*, beating, leg. Sprick,P., det. Sprick,P.
Polydrusus impressifrons<br>Gyllenhal, 1834	MK892160 [new]<br>2450-PSP<br>658nt	ZFMK-TIS-23422<br>ZFMK-DNA-0171661823	Germany, Lower Saxony (NI), Hameln-Rohrsen, Dütberg, SW-Rand, N52°06'21" E09°24'49", 104m, 10-Mai-2013, *Salix alba*, beating, leg. Sprick,P., det. Sprick,P.
Polydrusus impressifrons<br>Gyllenhal, 1834	MK891979 [new]<br>2250-JKR<br>658nt	ZFMK-TIS-23988<br>ZFMK-DNA-0169170530	Slovakia, Kezmarok, Busovce, N49°13'23.5" E20°28'32", 600m, 21-Jun-2014, leg. Benedikt,S., det. Benedikt,S.
Polydrusus impressifrons<br>Gyllenhal, 1834	MK891062 [new]<br>963-CBR<br>658nt	ZFMK-TIS-3876<br>ZFMK-DNA-0100449117	Switzerland, 4 km SW of Zernez, Prazet, N46°40'12" E10°02'51", 1650m, 22-Jul-2011, beating, leg. Braunert,C., det. Braunert,C.
Polydrusus inustus<br>Germar, 1824	KC784179 [new]<br>417-RGO<br>658nt	ZFMK-TIS-20203<br>ZFMK-DNA-0100449109	Poland, Tarnogóra, N50°41'11.6" E23°07'13.4", 249m, 11-Jun-2011, leg. Gosik,R., det. Gosik,R.
Polydrusus inustus<br>Germar, 1824	MK891582 [new]<br>393-RGO<br>658nt	ZFMK-TIS-20179<br>ZFMK-DNA-0155630443	Poland, K?tyll, N50°40'37" E23°07'43", 270m, 30-Jun-2011, leg. Gosik,R., det. Gosik,R.
Polydrusus inustus<br>Germar, 1824	MK891532 [new]<br>1620-JKR<br>658nt	ZFMK-TIS-3668<br>ZFMK-DNA-0155635618	Slovakia, Nove Zamky, Chlaba env., N47°49'28" E18°50'34", 108m, 18-Mai-2013, sweeping, leg. Krátky,J., det. Krátky,J.

**Name**<br>**Authority**<br>**Additional Information**	**GenBank Acc No (Ref.)**<br>**Specimen ID**<br>**Sequence Length**	**ZFMK Tissue ID**<br>**ZFMK DNA Sample ID**<br>**(SDEI DNA Sample ID)**	**Locality, GPS, Collection Date, Plant, Collector, Identifier**
Polydrusus inustus<br>Germar, 1824	MK891962 [new]<br>2231-JKR<br>658nt	ZFMK-TIS-23969<br>ZFMK-DNA-0169170559	Slovakia, Komarno, Komarno, N47°46'28" E18°06'40", 107m, 15-Jun-2015, *Tetragonolobus maritimus*, leg. Benedikt,S., det. Benedikt,S.
Polydrusus inustus<br>Germar, 1824	MK892219 [new]<br>2515-PSP<br>658nt	ZFMK-TIS-23487<br>ZFMK-DNA-0171661757	Slovakia, Nitra, Zlatna na Ostrove, N47°45'34" E17°58'40", 110m, 17-Mai-2014, *herb layer*, leg. Sprick,P., det. Sprick,P.
Polydrusus marginatus<br>Stephens, 1831	MK890996 [new]<br>888-CBR<br>658nt	ZFMK-TIS-3801<br>ZFMK-DNA-0100449723	Luxembourg, 10 km E of Luxembourg, 2 km SE of Mensdorf, N49°38'24" E06°18'34", 305m, 16-Apr-2011, *Quercus*, beating, leg. Braunert,C., det. Braunert,C.
Polydrusus mollis<br>(Ström, 1768)	MK891081 [new]<br>984-PSP<br>658nt	ZFMK-TIS-3129<br>ZFMK-DNA-0100426291	Germany, Lower Saxony (NI), Braunschweig, Mascherode, Oberdahlumer Holz, N52°12'44" E10°34'31", 106m, 27-Apr-2012, *Carpinus betulus*, edge of the forest, beating, leg. Sprick,P., det. Sprick,P.
Polydrusus mollis<br>(Ström, 1768)	MK892268 [new]<br>2568-PSP<br>658nt	ZFMK-TIS-23542<br>ZFMK-DNA-0171661991	Germany, Lower Saxony (NI), Harz National Park, Herzberg, N51°41'42" E10°19'47", 364m, 10-Jun-2014, *Alnus glutinosa*, leg. Sprick,P., det. Sprick,P.
Polydrusus mollis<br>(Ström, 1768)	MK892309 [new]<br>2610-PSP<br>658nt	ZFMK-TIS-23584<br>ZFMK-DNA-0171661950	Germany, Bavaria (BY), Unterfranken, Ebern, N50°05'00" E10°45'40", 320m, 14-Jun-2014, *Carpinus betulus*, leg. Sprick,P., det. Sprick,P.
Polydrusus pilosus<br>Gredler, 1866	MK892196 [new]<br>2491-PSP<br>658nt	ZFMK-TIS-23463<br>ZFMK-DNA-0171661781	Germany, Saxony-Anhalt (ST), Harz National Park, Drei Annen Hohne, Hohneklippen, Hohnekopf, N51°46'37" E10°41'55", 840m, 24-Apr-2014, *Betula pendula*, beating, leg. Sprick,P., det. Sprick,P.
Polydrusus pterygomalis<br>Boheman, 1840	KC783951 [new]<br>312-JKR<br>658nt	ZFMK-TIS-20098<br>ZFMK-DNA-0100438774	Czech Republic, Bohemia or., Dvakacovice, N49°58'35.447" E15°54'2.243", 245m, 14-Mai-2011, beating, leg. Krátky,J., det. Krátky,J.
Polydrusus pterygomalis<br>Boheman, 1840	KC784167 [new]<br>592-PSP<br>658nt	ZFMK-TIS-20376<br>ZFMK-DNA-0100448675	Germany, Lower Saxony (NI), Bad Zwischenahn, Aschhausen, N53°12'00" E08°04'00", 11m, 10-Jun-2011, *Carpinus betulus*, beating, leg. Sprick,P., det. Sprick,P.
Polydrusus pterygomalis<br>Boheman, 1840	MK892242 [new]<br>2540-PSP<br>658nt	ZFMK-TIS-23512<br>ZFMK-DNA-0171661734	Slovakia, Nitra, W of Gbelce, N47°46'56" E18°27'37", 148m, 18-Mai-2014, *Ulmus minor*, leg. Sprick,P., det. Sprick,P.
Polydrusus pulchellus<br>Stephens, 1831	MK891149 [new]<br>1056-PSP<br>658nt	ZFMK-TIS-3201<br>ZFMK-DNA-0100426918	Germany, Lower Saxony (NI), Langeoog, N53°44'20" E07°30'15", -1m, 16-Jun-2012, *Artemisia maritima*, salt marsh, dipnet, leg. Sprick,P., det. Sprick,P.
Polydrusus pulchellus<br>Stephens, 1831	MK890967 [new]<br>850-PST<br>658nt	ZFMK-TIS-3763<br>ZFMK-DNA-0100414248	Portugal, Estremadura, N of Ericeira, Ribamar, coast, N38°59'29" W09°24'54", 28m, 18-Mai-2012, beating, leg. Stüben,P., det. Stüben,P.
Polydrusus setifrons<br>Jaquelin du Val, 1852	KC783856 [new]<br>170-PST<br>654nt	ZFMK-TIS-2D100440264<br>ZFMK-DNA-0100438152	Morocco, Atlas Mts., S of Taza, Jbel Tazzeka near Bat-Bou-Idir, N34°03'29" W04°10'21", 1598m, 16-Mai-2011, *Quercus*, beating, leg. Stüben,P., det. Stüben,P./vid. Behne,L.
Polydrusus subglaber<br>Desbrochers des Loges, 1870	KC783843 [new]<br>182-PST<br>658nt	ZFMK-TIS-2D100440245<br>ZFMK-DNA-0100438133	Morocco, Atlas Mts., S of Ifrane, S of Timahdite near Aguelmame de Sidi-Ali, N33°05'09" W05°01'05", 2077m, 19-Mai-2011, *Crataegus*, beating, leg. Stüben,P., det. Behne,L.
Polydrusus subglaber<br>Desbrochers des Loges, 1870	KC783870 [new]<br>147-PST<br>658nt	ZFMK-TIS-2D100440289<br>ZFMK-DNA-0100438177	Morocco, Atlas Mts., S of Ifrane, S of Timahdite near Aguelmame de Sidi-Ali, N33°05'09" W05°01'05", 2077m, 19-Mai-2011, *Crataegus*, beating, leg. Stüben,P., det. Behne,L.

**Name**<br>**Authority**<br>**Additional Information**	**GenBank Acc No (Ref.)**<br>**Specimen ID**<br>**Sequence Length**	**ZFMK Tissue ID**<br>**ZFMK DNA Sample ID**<br>**(SDEI DNA Sample ID)**	**Locality, GPS, Collection Date, Plant, Collector, Identifier**
Polydrusus tereticollis<br>(DeGeer, 1775)	MK891815 [new]<br>2075-JKR<br>658nt	ZFMK-TIS-23715<br>ZFMK-DNA-0169168582	Czech Republic, Bohemia, Predhradi, N49°49'56" E16°02'33", 427m, 08-Mai-2014, sweeping, leg. Krátky,J., det. Krátky,J.
Polydrusus tereticollis<br>(Degeer, 1775)	KC784018 [new]<br>223-PSP<br>658nt	ZFMK-TIS-2D100439148<br>ZFMK-DNA-0100439820	Germany, Lower Saxony (NI), Everloh, Hannover region, Benther Berg, N52°20'01" E09°36'58", 100m, 22-Apr-2011, *Quercus robur*, edge of the forest, beating, leg. Sprick,P., det. Sprick,P.
Polydrusus tibialis<br>(Gyllenhal, 1834)	KC784117 [new]<br>669-FBA<br>658nt	ZFMK-TIS-20450<br>ZFMK-DNA-0100448598	Greece, Peloponnese, Lakonia, Gythio W, Sminos riv., N36°44'57'' E22°31'01'', 16m, 25-Mai-2011, leg. Bahr,F., det. Bahr,F.
Polydrusus viridicinctus<br>Gyllenhal, 1834	MK891969 [new]<br>2239-JKR<br>658nt	ZFMK-TIS-23977<br>ZFMK-DNA-0169170548	Slovakia, Velky Krtis, Potocik, N48°10'27" E19°25'45", 254m, 17-Jun-2014, *Quercus cerris*, leg. Benedikt,S., det. Benedikt,S.
Polydrusus xanthopus<br>Gyllenhal, 1834	KC783876 [new]<br>129-PST<br>657nt	ZFMK-TIS-2D100438626<br>ZFMK-DNA-0100438186	Morocco, S of Berkane, Beni Snassen Mts., Vallee Zegzel, N34°48'56" W02°24'01", 678m, 12-Mai-2011, *Olea*, beating, leg. Stüben,P., det. Behne,L.
Poophagus sisymbrii<br>(Fabricius, 1777)	MK891001 [new]<br>894-CBR<br>658nt	ZFMK-TIS-3807<br>ZFMK-DNA-0100449716	France, Dep. Ardenne, 10 km SE of Vouziers, Brecy-Brieres, N49°19'25" E04°46'23", 100m, 23-Apr-2011, *Rorippa amphibia*, beating, leg. Braunert,C., det. Braunert,C.
Poophagus sisymbrii<br>(Fabricius, 1777)	MK891128 [new]<br>1033-PSP<br>658nt	ZFMK-TIS-3178<br>ZFMK-DNA-0100426815	Germany, Lower Saxony (NI), Hannover, Stöcken, N52°24'21" E09°39'04", 44m, 22-Mai-2012, *Rorippa amphibia*, Leine river steep bank, beating, leg. Sprick,P., det. Sprick,P.
Poophagus sisymbrii<br>(Fabricius, 1777)	MK891392 [new]<br>1451-JKR<br>658nt	ZFMK-TIS-4220<br>ZFMK-DNA-0155628489	Slovakia, Nove Zamky, Male Kosihy, N47°55'58" E18°45'14", 108m, 28-Sep-2013, *Rorippa amphibia*, sieving, leg. Krátky,J., det. Krátky,J.
Prisistus obsoletus<br>(Germar, 1824)	MK891881 [new]<br>2145-JKR<br>658nt	ZFMK-TIS-23785<br>ZFMK-DNA-0169169588	Slovakia, Nove Zamky, Cenkov, NPR Censkovska step, N47°46'09" E18°31'09", 110m, 19-Mai-2014, sweeping, leg. Krátky,J., det. Krátky,J.
Prisistus suturalba<br>(Schultze, 1903)	MK891880 [new]<br>2144-JKR<br>658nt	ZFMK-TIS-23784<br>ZFMK-DNA-0169169589	Slovakia, Nove Zamky, Cenkov, NPR Censkovska step, N47°46'09" E18°31'09", 110m, 19-Mai-2014, sweeping, leg. Krátky,J., det. Krátky,J.
Procas armillatus<br>(Fabricius, 1801)	KC783795 [40]<br>86-PST<br>658nt	ZFMK-TIS-2D100446951<br>ZFMK-DNA-0100437982	Spain, Canary Islands, La Gomera, N of Epina, Teselinde, Santa Clara, N28°11'47" W17°17'16", 748m, 19-Feb-2011, beating, leg. Stüben,P., det. Stüben,P.
Procas armillatus<br>(Fabricius, 1801)	MK891597 [40]<br>1676-PST<br>658nt	ZFMK-TIS-24394<br>ZFMK-DNA-0155622505	Spain, Canary Islands, La Gomera, Parque Natural de Majona, above Casas del Palmar, N28°09'28" W17°09'41", 641m, 07-Dez-2013, beating, leg. Stüben,P., det. Stüben,P.
Procas armillatus<br>(Fabricius, 1801)	KC784307 [40]<br>745-PST<br>658nt	ZFMK-TIS-3082<br>ZFMK-DNA-0100448142	Spain, Canary Islands, Tenerife, Anaga Mts. near Batan de Arriba, N28°32'23" W16°17'34", 596m, 06-Feb-2012, beating, leg. Stüben,P. & Schütte,A., det. Stüben,P.
Proeces acicula<br>(Wollaston, 1854)	MH051956 [33]<br>2781-PST<br>658nt	ZFMK-TIS-26135<br>ZFMK-DNA-0169166954	Portugal, Madeira, Ilhas Desertas, Deserta Grande (south), above Focinha, N32°30'18" W16°29'59", 215m, 24-Mrz-2015, *Crithmum maritimum (ger. "Meeresfenchel")*, collecting by hand, leg. Stüben,P., det. Stüben,P.
Proeces reticulatus<br>(Roudier, 1957)	KC783761 [40]<br>50-PST<br>640nt	ZFMK-TIS-2D100446997<br>ZFMK-DNA-0100438069	Spain, Canary Islands, Gran Canaria, N of Maspalomas, Arteara, N27°50'53" W15°34'00", 336m, 22-Jan-2011, *Phoenix canariensis*, beating, leg. Stüben,P., det. Stüben,P.

**Name**<br>**Authority**<br>**Additional Information**	**GenBank Acc No (Ref.)**<br>**Specimen ID**<br>**Sequence Length**	**ZFMK Tissue ID**<br>**ZFMK DNA Sample ID**<br>**(SDEI DNA Sample ID)**	**Locality, GPS, Collection Date, Plant, Collector, Identifier**
Proeces reticulatus<br>(Roudier, 1957)	KC783770 [40]<br>61-PST<br>630nt	ZFMK-TIS-2D100446987<br>ZFMK-DNA-0100437992	Spain, Canary Islands, La Gomera, Hermigua, Las Nuevitas, Barranquillos los Alamos, N28°09'31" W17°11'09", 251m, 13-Dez-2010, *Phoenix canariensis*, beating, leg. Stüben,P., det. Stüben,P.
Protapion apricans<br>(Herbst, 1797)	KC784047 [new]<br>636-PSP<br>658nt	ZFMK-TIS-20419<br>ZFMK-DNA-0100448287	Germany, Lower Saxony (NI), Groß-Berkel, Lkr. Hameln-Pyrmont, N52°03'54" E09°17'39", 81m, 03-Sep-2011, *Trifolium pratense*, mesophilic grassland, dipnet, leg. Sprick,P., det. Sprick,P.
Protapion apricans<br>(Herbst, 1797)	MK892288 [new]<br>2589-PSP<br>658nt	ZFMK-TIS-23563<br>ZFMK-DNA-0171661977	Germany, Bavaria (BY), Unterfranken, Ebern, N50°05'20.5" E10°45'59", 333m, 13-Jun-2014, *Trifolium pratense*, leg. Sprick,P., det. Sprick,P.
Protapion apricans<br>(Herbst, 1797)	MK891344 [new]<br>1381-PST<br>658nt	ZFMK-TIS-4677<br>ZFMK-DNA-0155628563	Italy, Abruzzo, P.N. Majella, 11 km N of Roccaraso, Bosco di S. Antonio, N41°56'27" E14°01'41", 1321m, 20-Aug-2013, *Pyrus/Acer, Fagus*, beating, leg. Stüben,P., det. Stüben,P.
Protapion apricans<br>(Herbst, 1797)	MK890984 [new]<br>875-CBR<br>658nt	ZFMK-TIS-3788<br>ZFMK-DNA-0100413805	Luxembourg, 20 km NE of Luxembourg, 1 km NW of Altrier, N49°45'12" E06°19'10", 370m, 07-Jul-2012, sweeping, leg. Braunert,C., det. Braunert,C.
Protapion assimile<br>(Kirby, 1808)	MK891452 [new]<br>1517-PSP<br>658nt	ZFMK-TIS-3277<br>ZFMK-DNA-0155633307	Denmark, Syddanmark, Emmerlev Klev (Højer), N54°59'53" E08°39'08", 3m, 30-Aug-2012, *Trifolium pratense*, leg. Sprick,P., det. Sprick,P.
Protapion assimile<br>(Kirby, 1808)	MK891126 [new]<br>1031-PSP<br>658nt	ZFMK-TIS-3176<br>ZFMK-DNA-0100426828	Germany, Lower Saxony (NI), Hannover, Stöcken, N52°24'24" E09°39'13", 42m, 22-Mai-2012, *Trifolium pratense*, species-rich hay meadow, dipnet, leg. Sprick,P., det. Sprick,P.
Protapion assimile<br>(Kirby, 1808)	MK892287 [new]<br>2588-PSP<br>658nt	ZFMK-TIS-23562<br>ZFMK-DNA-0171661976	Germany, Bavaria (BY), Unterfranken, Ebern, N50°05'20.5" E10°45'59", 333m, 13-Jun-2014, *Trifolium medium, pratense*, leg. Sprick,P., det. Sprick,P.
Protapion dissimile<br>(Germar, 1817)	MK892084 [new]<br>2360-JKR<br>658nt	ZFMK-TIS-25920<br>ZFMK-DNA-0171600581	Czech Republic, Moravia, NP Podyji, Cizov, Siroke pole, N48°51'36" E15°51'02", 300m, 17-Aug-2014, *Trifolium arvense*, sweeping, leg. R. Stejskal, det. Stejskal,R.
Protapion dissimile<br>(Germar, 1817)	MK891416 [new]<br>1478-PSP<br>658nt	ZFMK-TIS-3238<br>ZFMK-DNA-0155633351	Germany, Lower Saxony (NI), Berkhof, Hannover region, N52°36'40" E09°43'59", 38m, 09-Jul-2012, *Trifolium arvense*, sand lean lawn, dipnet, leg. Sprick,P., det. Sprick,P.
Protapion filirostre<br>(Kirby, 1808)	MK892103 [new]<br>2380-JKR<br>658nt	ZFMK-TIS-25957<br>ZFMK-DNA-0171600609	Czech Republic, Moravia, Vevcice, N48°57'45" E16°03'07", 275m, 24-Aug-2014, sweeping, leg. R. Stejskal, det. Stejskal,R.
Protapion filirostre<br>(Kirby, 1808)	KC784198 [new]<br>451-RGO<br>658nt	ZFMK-TIS-20237<br>ZFMK-DNA-0100449388	Poland, Rudnik, N51°14'31.02" E22°32'22.92", 191m, 03-Okt-2011, leg. Gosik,R., det. Gosik,R.
Protapion fulvipes fulvipes<br>(Geoffroy, 1785)	MK892082 [new]<br>2358-JKR<br>658nt	ZFMK-TIS-25918<br>ZFMK-DNA-0171600583	Czech Republic, Moravia, NP Podyji, Cizov, Siroke pole, N48°51'36" E15°51'02", 300m, 17-Aug-2014, sweeping, leg. R. Stejskal, det. Stejskal,R.
Protapion fulvipes fulvipes<br>(Geoffroy, 1785)	MK890900 [new]<br>630-PSP<br>658nt	ZFMK-TIS-20413<br>ZFMK-DNA-0100448636	Germany, Schleswig-Holstein (SH), Rosenkranz, Lkr. Nordfriesland, N54°53'12" E08°45'57", -1m, 24-Aug-2011, *Salix cinerea*, wayside shrub, beating, leg. Sprick,P., det. Sprick,P.
Protapion fulvipes fulvipes<br>(Geoffroy, 1785)	KC784196 [new]<br>459-RGO<br>658nt	ZFMK-TIS-20245<br>ZFMK-DNA-0100449381	Poland, Rudnik, N51°14'31.02" E22°32'22.92", 191m, 03-Okt-2011, leg. Gosik,R., det. Gosik,R.

**Name**<br>**Authority**<br>**Additional Information**	**GenBank Acc No (Ref.)**<br>**Specimen ID**<br>**Sequence Length**	**ZFMK Tissue ID**<br>**ZFMK DNA Sample ID**<br>**(SDEI DNA Sample ID)**	**Locality, GPS, Collection Date, Plant, Collector, Identifier**
Protapion fulvipes fulvipes<br>(Geoffroy, 1785)	KC784212 [new]<br>439-RGO<br>658nt	ZFMK-TIS-20225<br>ZFMK-DNA-0100449409	Poland, Zemborzyce, N51°09'56.7" E22°30'10.5", 178m, 18-Sep-2011, leg. Gosik,R., det. Gosik,R.
Protapion gracilipes<br>(Dietrich, 1857)	MK892284 [new]<br>2585-PSP<br>658nt	ZFMK-TIS-23559<br>ZFMK-DNA-0171661973	Germany, Bavaria (BY), Unterfranken, Ebern, N50°05'20.5" E10°45'59", 333m, 13-Jun-2014, *Trifolium medium*, leg. Sprick,P., det. Sprick,P.
Protapion laevicolle<br>(Kirby, 1811)	KC784254 [new]<br>IT-0023w<br>656nt	ZFMK-TIS-2D100446543<br>ZFMK-DNA-0100433831	Italy, Sardinia Isl. South, E of Cagliari, M. d. Sette Fratelli near Pta. Moitzus, N39°16'38" E09°28'35", 167m, 02-Okt-2010, *Quercus, Pistacia*, drained creek, leg. Stüben,P., det. Stüben,P.
Protapion laevicolle<br>(Kirby, 1811)	KC784257 [new]<br>IT-0027w<br>621nt	ZFMK-TIS-2D100446545<br>ZFMK-DNA-0100437849	Italy, Sardinia Isl. West, E of Macomer, above Silanus, N40°19'20" E08°52'13", 1015m, 04-Okt-2010, *Acer monspessulanum, Quercus, Verbascum*, meadow, leg. Stüben,P., det. Stüben,P.
Protapion nigritarse<br>(Kirby, 1808)	MK892083 [new]<br>2359-JKR<br>658nt	ZFMK-TIS-25919<br>ZFMK-DNA-0171600582	Czech Republic, Moravia, NP Podyji, Cizov, Siroke pole, N48°51'36" E15°51'02", 300m, 17-Aug-2014, sweeping, leg. R. Stejskal, det. Stejskal,R.
Protapion nigritarse<br>(Kirby, 1808)	MK891141 [new]<br>1046-PSP<br>658nt	ZFMK-TIS-3191<br>ZFMK-DNA-0100426813	Germany, Lower Saxony (NI), Hannover, Ahlem, N52°23'00" E09°40'55", 50m, 01-Jun-2012, *Trifolium dubium*, Faboideae-rich ruderal area, dipnet, leg. Sprick,P., det. Sprick,P.
Protapion nigritarse<br>(Kirby, 1808)	MK892299 [new]<br>2600-PSP<br>658nt	ZFMK-TIS-23574<br>ZFMK-DNA-0171661959	Germany, Bavaria (BY), Unterfranken, Ebern, N50°05'17" E10°45'53", 343m, 13-Jun-2014, *Trifolium campestre/dubium*, leg. Sprick,P., det. Sprick,P.
Protapion nigritarse<br>(Kirby, 1808)	MK892255 [new]<br>2554-PSP<br>658nt	ZFMK-TIS-23528<br>ZFMK-DNA-0171662005	Slovakia, Nitra, Muzla, N47°48'00" E18°31'55", 114m, 19-Mai-2014, *Trifolium campestre*, leg. Sprick,P., det. Sprick,P.
Protapion ononidis<br>(Gyllenhal, 1827)	KC784010 [new]<br>242-PSP<br>658nt	ZFMK-TIS-2D100438527<br>ZFMK-DNA-0100439808	Germany, Lower Saxony (NI), Nordstemmen, Lkr. Hildesheim, N52°09' E09°47', 72m, 04-Mai-2011, *Ononis repens*, garden, beating, leg. Sprick,P., det. Sprick,P.
Protapion ononidis<br>(Gyllenhal, 1827)	MK891699 [new]<br>1917-FBA<br>658nt	ZFMK-TIS-23308<br>ZFMK-DNA-0169170365	Greece, East Macedonia and Thrace, Drama, Lefkoghia, N41°23'59" E23°54'53", 620m, 13-Aug-2014, leg. Bayer & Brunner, det. Bayer,F.
Protapion ononidis<br>(Gyllenhal, 1827)	MK892256 [new]<br>2555-PSP<br>658nt	ZFMK-TIS-23529<br>ZFMK-DNA-0171662004	Slovakia, Nitra, Muzla, N47°48'00" E18°31'55", 114m, 19-Mai-2014, *Ononis spinosa*, leg. Sprick,P., det. Sprick,P.
Protapion ruficroides<br>(Dieckmann, 1973)	MK891350 [new]<br>1391-PST<br>658nt	ZFMK-TIS-4687<br>ZFMK-DNA-0155628542	Italy, Abruzzo, near Roccaraso, N41°50'24" E14°05'03", 1258m, 24-Aug-2013, *Fagus*, sieving, leg. Stüben,P., det. Stüben,P.
Protapion trifolii<br>(Linnaeus, 1768)	KC783833 [new]<br>635-PSP<br>653nt	ZFMK-TIS-20418<br>ZFMK-DNA-0100448641	Germany, Lower Saxony (NI), Groß-Berkel, Lkr. Hameln-Pyrmont, N52°03'54" E09°17'39", 81m, 03-Sep-2011, *Trifolium pratense*, mesophilic grassland, dipnet, leg. Sprick,P., det. Sprick,P.
Protopirapion atratulum<br>(Germar, 1817)	MK890879 [new]<br>499-RST<br>658nt	ZFMK-TIS-20284<br>ZFMK-DNA-0100448480	Czech Republic, Moravia mer., Pusty kopec u Konic Nature Monument, 1.6 km E of Znojmo-Popice, N48°48'45.74" E16°2'17.33", 260m, 18-Mai-2011, *Cytisus procumbens*, sweeping, leg. Stejskal,R., det. Stejskal,R.
Psallidium kraatzi<br>(Kiesenwetter, 1864)	MK891564 [new]<br>1662-FBA<br>658nt	ZFMK-TIS-4706<br>ZFMK-DNA-0155630477	Greece, Crete Isl., Rethimno, Bali, N35°24'24" E24°46'53", 32m, 01-Apr-2013, leg. Winkelmann,H., det. Winkelmann,H.

**Name**<br>**Authority**<br>**Additional Information**	**GenBank Acc No (Ref.)**<br>**Specimen ID**<br>**Sequence Length**	**ZFMK Tissue ID**<br>**ZFMK DNA Sample ID**<br>**(SDEI DNA Sample ID)**	**Locality, GPS, Collection Date, Plant, Collector, Identifier**
Psallidium maxillosum<br>(Fabricius, 1792)	MK891890 [new]<br>2155-JKR<br>658nt	ZFMK-TIS-23795<br>ZFMK-DNA-0169169569	Slovakia, Nove Zamky, Jursky Chlm, N47°48'04" E18°31'27", 114m, 19-Mai-2014, sweeping, leg. Krátky,J., det. Krátky,J.
Psallidium spinimanum<br>Reiche, 1861	KC784116 [new]<br>678-FBA<br>658nt	ZFMK-TIS-20456<br>ZFMK-DNA-0100448588	Greece, Peloponnese, Messinia, Mt. Taygetos, E of Agh. Nikolaos, N36°49'16" E22°17'55", 30m, 22-Mai-2011, leg. Bahr,F., det. Bahr,F.
Psallidium talparum<br>Bahr & Winkelmann, 2015	KC784049 [18]<br>683-FBA<br>658nt	ZFMK-TIS-20461<br>ZFMK-DNA-0100448291	Greece, Peloponnese, W of Mt. Taygetos, E of Saidona, N36°52'59" E22°17'25", 800m, 21-Apr-2009, leg. Bayer,B., det. Bayer,B.
Pselactus affinis<br>(Wollaston, 1861)<br>formerly: *P. proximus* - Syn!	KP776631 [40]<br>C-0090-COS<br>658nt	ZFMK-TIS-cC0090<br>ZFMK-DNA-0100400873	Spain, Canary Islands, El Hierro, 2 km SE of Sabinosa, El Gretime, N27°44'22" W18°04'52", 677m, 26-Dez-2006, *Laurus, Ficus, Sonchus*, leg. Stüben,P., det. Stüben,P.
Pselactus affinis<br>(Wollaston, 1861)<br>formerly: *P. proximus* - Syn!	MK891330 [new]<br>C-0091-COS<br>658nt	ZFMK-TIS-24520<br>ZFMK-DNA-0155630401	Spain, Canary Islands, El Hierro, 2.5 km N of San Andres, Las Montanetas, 27°48'08"N 17°57'36"W, 847m, 23-Dez-2006, *Ficus carica*, leg. Stüben,P., det. Stüben,P.
Pselactus affinis<br>(Wollaston, 1861)<br>formerly: *P. proximus* - Syn!	MK892393 [40]<br>2869-PST<br>658nt	ZFMK-TIS-4258<br>ZFMK-DNA-0169166923	Spain, Canary Islands, El Hierro, W of Frontera, Pista al Derrabado, N27°44'11" W18°04'43", 785m, 11-Apr-2016, beating, leg. Stüben,P. & Schütte,A., det. Stüben,P.
Pselactus affinis<br>Colonnelli, 1990<br>formerly: *P. proximus* - Syn!	MK892396 [40]<br>2873-PST<br>658nt	ZFMK-TIS-25878<br>ZFMK-DNA-0169166919	Spain, Canary Islands, El Hierro, S of Guarazoca, N27°48'16" W17°58'12", 721m, 12-Apr-2016, *Foeniculum vulgare*, sieving, leg. Stüben,P. & Schütte,A., det. Stüben,P.
Pselactus affinis<br>Wollaston, 1861<br>formerly: *P. folwacznyi* - Syn!	KC783805 [40]<br>90-PST<br>628nt	ZFMK-TIS-2D100446955<br>ZFMK-DNA-0100438027	Spain, Canary Islands, La Gomera, Agulo, N28°11'31" W17°11'33", 61m, 25-Feb-2011, *detritus*, coast, garden, beating, leg. Stüben,P., det. Stüben,P.
Pselactus affinis<br>Wollaston, 1861<br>formerly: *P. folwacznyi* - Syn!	MK891598 [40]<br>1677-PST<br>658nt	ZFMK-TIS-24395<br>ZFMK-DNA-0155622506	Spain, Canary Islands, La Gomera, Parque Natural de Majona, above Casas del Palmar, N28°09'28" W17°09'41", 641m, 07-Dez-2013, beating, leg. Stüben,P., det. Stüben,P.
Pselactus affinis<br>Wollaston, 1861<br>formerly: *P. capitulatus* - Syn!	MK891484 [40]<br>1554-JKR<br>658nt	ZFMK-TIS-3602<br>ZFMK-DNA-0155635675	Spain, Canary Islands, La Palma, Las Caletas, N28°29'34" W17°49'46", 418m, 28-Jan-2013, *Ficus carica*, sieving, leg. Krátky,J., det. Krátky,J.
Pselactus affinis<br>Wollaston, 1861<br>formerly: *P. capitulatus* - Syn!	MK891626 [40]<br>1740-PST<br>658nt	ZFMK-TIS-24458<br>ZFMK-DNA-0155622450	Spain, Canary Islands, La Palma, W of Mazo near M. las Toscas, N28°36'01" W17°47'31", 739m, 09-Jan-2014, *Brassicaceae*, beating, leg. Stüben,P., det. Stüben,P.
Pselactus affinis<br>Wollaston, 1861<br>formerly: *P. capitulatus* - Syn!	MK891628 [40]<br>1748-PST<br>658nt	ZFMK-TIS-24466<br>ZFMK-DNA-0155622433	Spain, Canary Islands, La Palma, W of Garafia near Puerto Garafia, N28°49'27" W17°57'40", 204m, 17-Jan-2014, *Euphorbia balsamifera*, sieving, leg. Stüben,P., det. Stüben,P.
Pselactus affinis<br>Wollaston, 1861<br>formerly: *P. capitulatus* - Syn!	MK891630 [40]<br>1751-PST<br>658nt	ZFMK-TIS-24469<br>ZFMK-DNA-0155622436	Spain, Canary Islands, La Palma, Montes de Luna, N28°31'50" W17°48'22", 453m, 18-Jan-2014, *Ficus*, sieving, leg. Stüben,P., det. Stüben,P.
Pselactus affinis<br>(Wollaston, 1861)	KC783830 [40]<br>734-PST<br>658nt	ZFMK-TIS-3071<br>ZFMK-DNA-0100448160	Spain, Canary Islands, Tenerife, 2 km S of Los Silos, Barranco de Blas, N28°21'31" W16°49'36", 211m, 29-Jan-2012, *Phoenix*, dry leaves, beating, leg. Stüben,P., det. Stüben,P.
Pselactus affinis<br>(Wollaston, 1861)	MK890909 [40]<br>748-PST<br>658nt	ZFMK-TIS-3085<br>ZFMK-DNA-0100448139	Spain, Canary Islands, Tenerife, 2 km S of Los Silos, Barranco del Agua, N28°21'44" W16°49'02", 209m, 09-Feb-2012, *Euphorbia*, beating, leg. Stüben,P. & Schütte,A., det. Stüben,P.

**Name**<br>**Authority**<br>**Additional Information**	**GenBank Acc No (Ref.)**<br>**Specimen ID**<br>**Sequence Length**	**ZFMK Tissue ID**<br>**ZFMK DNA Sample ID**<br>**(SDEI DNA Sample ID)**	**Locality, GPS, Collection Date, Plant, Collector, Identifier**
Pselactus piceus subparallelus<br>(Wollaston, 1861)	MK347535 [40]<br>2940-PST<br>658nt	(SDEI-DNA-2940-PST)	Spain, Canary Islands, Lanzarote, 1 km S of Ermita de las Nieves, N29°05'45" W13°31'49", 496m, 16-Jan-2017, *Cynara cardunculus*, sifting, leg. Stüben,P., det. Stüben,P.
Pselactus sp.	MH051951 [33]<br>2729-PST<br>658nt	ZFMK-TIS-23879<br>ZFMK-DNA-0171661270	Spain, Canary Islands, Fuerteventura, 2 km NE of Vega de Rio Palmas, N28°24'10" W14°03'39", 320m, 04-Jan-2015, *Acacia (Fabaceae)*, sieving, leg. Stüben,P., det. Stüben,P.
Pselactus spadix spadix<br>(Herbst, 1795)<br>formerly: *Pselactus spadix sulcipennis* - Syn!	MH051954 [33]<br>2772-PST<br>658nt	ZFMK-TIS-26126<br>ZFMK-DNA-0169166970	Portugal, Madeira, W of Funchal, Cavaca, N32°38'13" W16°56'48", 53m, 19-Mrz-2015, babana plantation, collecting by hand, leg. Stüben,P., det. Stüben,P.
Pselactus varipennis caulium<br>Folwaczny, 1971<br>formerly: *P. caulium varipennis*	MH051971 [33]<br>2924-PST<br>658nt	(SDEI-DNA-2924-PST)	Spain, Canary Islands, Lanzarote, Ye, N29°11'48" W13°28'42", 365m, 04-Jan-2017, *under wood*, sifting, leg. Stüben,P., det. Stüben,P.
Pseudapion fulvirostre<br>(Gyllenhal, 1833)	MK891261 [new]<br>1197-JKR<br>658nt	ZFMK-TIS-3533<br>ZFMK-DNA-0100426087	Slovakia, Komarno, Komarno, N47°45'30" E18°08'50", 106m, 07-Jul-2012, *Althea* sp., sweeping, leg. Krátky,J., det. Krátky,J.
Pseudapion moschatae<br>(A. Hoffmann, 1938)	MK890915 [new]<br>783-PST<br>658nt	ZFMK-TIS-3696<br>ZFMK-DNA-0100414308	Germany, Rhineland-Palatinate (RLP), E of Treis-Karden near Pommern, Fellerbachtal (river valley), N50°10'10" E07°13'38", 85m, 09-Jun-2012, *Malva moschata L.*, rivulet, beating, leg. Stüben,P., det. Stüben,P.
Pseudapion moschatae<br>(A. Hoffmann, 1938)	MK890936 [new]<br>805-PST<br>658nt	ZFMK-TIS-3718<br>ZFMK-DNA-0100414286	Germany, Rhineland-Palatinate (RLP), E of Cochem, Valwig, Apolloweg, N50°08'45" E07°12'35", 234m, 10-Jun-2012, high road (vineyard), beating, leg. Stüben,P., det. Stüben,P.
Pseudapion moschatae<br>(A. Hoffmann, 1938)	MK891069 [new]<br>970-CBR<br>658nt	ZFMK-TIS-3883<br>ZFMK-DNA-0100449648	Luxembourg, 1 km E of Canach, N49°36'35" E06°20'08", 220m, 10-Jul-2012, *Malva moschata*, sweeping, leg. Braunert,C., det. Braunert,C.
Pseudapion rufirostre<br>(Fabricius, 1775)	MK890937 [new]<br>806-PST<br>658nt	ZFMK-TIS-3719<br>ZFMK-DNA-0100414285	Germany, Rhineland-Palatinate (RLP), E of Cochem, Valwig, Apolloweg, N50°08'45" E07°12'35", 234m, 10-Jun-2012, high road (vineyard), beating, leg. Stüben,P., det. Stüben,P.
Pseudapion rufirostre<br>(Fabricius, 1775)	MK891144 [new]<br>1049-PSP<br>658nt	ZFMK-TIS-3194<br>ZFMK-DNA-0100426911	Germany, Lower Saxony (NI), Königsförde, Lkr. Hameln-Pyrmont, N52°03'58" E09°16'17", 94m, 07-Jun-2012, *Malva sylvestris*, house garden, beating, leg. Sprick,P., det. Sprick,P.
Pseudaplemonus artemisiae<br>(Morawitz, 1861)	MK891554 [new]<br>1645-JKR<br>658nt	ZFMK-TIS-4223<br>ZFMK-DNA-0155630495	Romania, Constanta, Histria, Grindul Saiele Nat.Res., N44°32'40" E28°46'22", 5m, 06-Jun-2013, *Limonium* cf.*bellidifolium*, sweeping, leg. Pelikan,J., det. Krátky,J.
Pseudocaulotrupis parvus<br>(Israelson, 1985)<br>**Paratype (DNAtype)**<br>formerly: *Caulotrupis parvus*	MH915691 [32]<br>3106-PST<br>658nt	ZFMK-DNA-FD02298667	Portugal, Azores, Santa Maria, Pico Alto (3), N36°58'59" W25°05'28", 559m, 03-Jun-2018, *Laurisilva remains*, small artificial clearing inside conservation area, sieving, leg. Stüben & Schütte, det. Stüben,P.
Pseudocaulotrupis schuettei<br>Stüben, 2018<br>**Paratype (DNAtype)**	MH915692 [32]<br>3107-PST<br>658nt	ZFMK-DNA-FD02298659	Portugal, Azores, Sao Jorge, NW Rosais: E Vigla da Baleia (near light house) (26), N38°44'52" W28°17'39", 333m, 11-Jun-2018, Laurisilva remains, only native Laurisilva trees left on opposite of bunker, beating, leg. Stüben & Schütte, det. Stüben,P.
Pseudocleonus cinereus<br>(Schrank, 1781)	MK891921 [new]<br>2189-JKR<br>658nt	ZFMK-TIS-23927<br>ZFMK-DNA-0169170598	Czech Republic, Moravia, Vranov nad Dyji, N48°54'46" E15°49'06", 355m, 26-Apr-2014, collecting by hand, leg. Stejskal,R., det. Stejskal,R.

**Name**<br>**Authority**<br>**Additional Information**	**GenBank Acc No (Ref.)**<br>**Specimen ID**<br>**Sequence Length**	**ZFMK Tissue ID**<br>**ZFMK DNA Sample ID**<br>**(SDEI DNA Sample ID)**	**Locality, GPS, Collection Date, Plant, Collector, Identifier**
Pseudocleonus cinereus<br>(Schrank, 1781)	MK891915 [new]<br>2183-JKR<br>658nt	ZFMK-TIS-23921<br>ZFMK-DNA-0169170607	Slovakia, Nove Zamky, Kamenica nad Hronom, Kovacovske kopce, NPR Skaly, N47°49'56" E18°44'08", 171m, 20-Mai-2014, *Taraxacum*, collecting by hand, leg. Stejskal,R., det. Stejskal,R.
Pseudodichromacalles fernandezi<br>(Roudier, 1954)	FJ716581 [01]<br>E-737-fer<br>658nt	ZFMK-TIS-cE737<br>ZFMK-DNA-0100404807	Spain, Canary Islands, La Gomera, S of Hermigua, El Cedro, N28°08'18" W17°12'52", 791m, 09-Okt-2008, *Sonchus*, leg. Astrin,J. & Stüben,P., det. Stüben,P.
Pseudodichromacalles fernandezi<br>(Roudier, 1954)	GU988023 [01]<br>E-728-fer<br>658nt	ZFMK-TIS-cE728<br>ZFMK-DNA-0100404827	Spain, Canary Islands, La Gomera, SE of Hermigua near Casas del Palmar, N28°09'29" W17°09'37", 627m, 08-Okt-2008, *Sonchus gomerensis*, thermophilic brushwood, leg. Astrin,J. & Stüben,P., det. Stüben,P.
Pseudodichromacalles fernandezi<br>(Roudier, 1954)	KF843950 [16]<br>ES1015<br>658nt	ZFMK-TIS-cES1015<br>ZFMK-DNA-0112704653	Spain, Canary Islands, La Gomera, E of Hermigua, El Palmar, 28°09'26"N 17°09'47"W, 627m, 30-Dez-2009, *Aeonium subplanum*, thermophilic brushwood, leg. Stüben,P., det. Stüben,P.
Pseudodichromacalles fernandezi<br>(Roudier, 1954)	KF843952 [16]<br>ES1018<br>658nt	ZFMK-TIS-cES1018<br>ZFMK-DNA-0112704656	Spain, Canary Islands, La Gomera, E of Hermigua, El Palmar, 28°09'26"N 17°09'47"W, 629m, 30-Dez-2009, *Pericallis*, thermophilic brushwood, leg. Stüben,P., det. Stüben,P.
Pseudodichromacalles fernandezi<br>(Roudier, 1954)	MG229721 [40]<br>1682-PST<br>658nt	ZFMK-TIS-24400<br>ZFMK-DNA-0155622511	Spain, Canary Islands, La Gomera, Arguamul, Teselinde Mt., Erimita Santa Clara, N28°11'46" W17°17'17", 737m, 15-Dez-2013, *Aeonium*, sieving, leg. Stüben,P., det. Stüben,P.
Pseudodichromacalles fernandezi<br>(Roudier, 1954)	GU987939 [01]<br>E-573-fer<br>658nt	ZFMK-TIS-cE573<br>ZFMK-DNA-0100404771	Spain, Canary Islands, Tenerife, 1 km S of Los Silos, N28°21'16" W16°48'49", 300m, 23-Dez-2003, *Sonchus spp., Aeonium tabulaeforme*, thermophilic brushwood, leg. Stüben,P., det. Stüben,P.
Pseudodichromacalles fernandezi<br>(Roudier, 1954)	GU988002 [01]<br>E-687-fer<br>658nt	ZFMK-TIS-cE687<br>ZFMK-DNA-0100404868	Spain, Canary Islands, Tenerife, NE of La Laguna, Anaga Mts., Las Bodegas, N28°33'42" W16°09'25", 502m, 30-Sep-2008, *Sonchus acaulis*, leg. Astrin,J. & Stüben,P., det. Stüben,P.
Pseudodichromacalles pericallis<br>Stüben, 2018<br>**Paratype (DNAtype)**	GU987796 [01]<br>C-0128-fer<br>658nt	ZFMK-TIS-cC0128<br>ZFMK-DNA-0100400192	Spain, Canary Islands, El Hierro, 9.5 km W of La Frontera, Pista de Mencafite, N27°44'06" W18°05'08", 929m, 22-Dez-2006, *Pericallis murrayi*, leg. Stüben,P., det. Stüben,P.
Pseudodichromacalles pericallis<br>Stüben, 2018<br>**Paratype (DNAtype)**	MF426962 [34]<br>E-0275-fer<br>658nt	ZFMK-TIS-24648<br>ZFMK-DNA-0155630415	Spain, Canary Islands, El Hierro, 7 km W of La Frontera, Pista Derrabado, N27°44'26" W18°03'07", 931m, 22-Dez-2006, *Pericallis murrayi*, leg. Stüben,P., det. Stüben,P.
Pseudodichromacalles pericallis<br>Stüben, 2018<br>**Paratype (DNAtype)**	MF426963 [34]<br>1704-PST_24422<br>658nt	ZFMK-TIS-24422<br>ZFMK-DNA-0155622485	Spain, Canary Islands, La Palma, NW of Puntallana, El Corcho, N28°45'22" W17°45'34", 405m, 23-Dez-2013, *Pericallis*, beating, leg. Stüben,P., det. Stüben,P.
Pseudodichromacalles xerampelinus<br>(Wollaston, 1864)	KF672592 [12]<br>1244-PST<br>658nt	ZFMK-TIS-3580<br>ZFMK-DNA-0100426270	Spain, Canary Islands, Tenerife, Anaga Mts., Chinobre, N28°33'31" W16°10'14", 830m, 13-Feb-2012, *Woodwardia radicans*, Laurisilva, beating, leg. Stüben,P. & Schütte,A., det. Stüben,P.
Pseudodichromacalles xerampelinus<br>(Wollaston, 1864)	KF672593 [12]<br>687-PST<br>658nt	ZFMK-TIS-3024<br>ZFMK-DNA-0100417603	Spain, Canary Islands, Tenerife, Anaga Mts. near Chinobre, N28°33'31" W16°10'13", 830m, 13-Feb-2012, *Woodwardia radicans*, Laurisilva (at night), beating, leg. Stüben,P. & Schütte,A., det. Stüben,P.
Pseudomeira eleonorae<br>Pierotti & Bellò, 1996	KC784275 [new]<br>IT-0049w<br>658nt	ZFMK-DNA-0100417934	Italy, Sardinia Isl., NE of Aritzo, Gennargentu, Mt. di Iscudu, N40°01'10" E09°16'39", 1511m, 30-Sep-2010, *alder, moss*, creek, leg. Stüben,P., det. Stüben,P.

**Name**<br>**Authority**<br>**Additional Information**	**GenBank Acc No (Ref.)**<br>**Specimen ID**<br>**Sequence Length**	**ZFMK Tissue ID**<br>**ZFMK DNA Sample ID**<br>**(SDEI DNA Sample ID)**	**Locality, GPS, Collection Date, Plant, Collector, Identifier**
Pseudomeira gougeletii<br>Seidlitz, 1865	MK891311 [new]<br>1301-PST<br>658nt	ZFMK-TIS-4117<br>ZFMK-DNA-0100426170	Spain, Cordoba, Montoro, N38°01'49" W04°22'46", 161m, 04-Mai-2013, *Brassicaceae*, beating, leg. Stüben,P. & Schütte,A., det. Stüben,P.
Pseudomeira sardoa<br>(A. Costa, 1884)	KC784243 [new]<br>IT-0010w<br>656nt	ZFMK-DNA-0100437856	Italy, Sardinia Isl. East, S of Dorgali, Genna Silana, N40°09'30" E09°30'30", 1017m, 27-Sep-2010, *Quercus ilex, Ficus*, cliff, leg. Stüben,P., det. Stüben,P.
Pseudomeira sp.	KC784271 [new]<br>IT-0045w<br>655nt	ZFMK-DNA-0100437842	Italy, Sardinia Isl. East, NE of Seui, Mt. Tonneri, N39°53'59" E09°23'24", 926m, 28-Sep-2010, *Quercus ilex*, cliff, leg. Stüben,P., det. Stüben,P.
Pseudomeira transversicollis<br>F. Solari, 1955	KC784253 [new]<br>IT-0022w<br>650nt	ZFMK-TIS-2D100446535<br>ZFMK-DNA-0100437853	Italy, Sardinia Isl. South, E of Cagliari, Flumini, Capitana, N39°11'57" E09°19'30", 13m, 02-Okt-2010, *Olea, Quercus, Pistacia*, coast, leg. Stüben,P., det. Stüben,P.
Pseudomyllocerus sinuatus<br>(Fabricius, 1801)	MK891384 [new]<br>1437-JKR<br>658nt	ZFMK-TIS-4206<br>ZFMK-DNA-0155628496	Slovakia, Nove Zamky, Bi?a, Hron. Riv., N47°56'22" E18°39'08", 125m, 21-Jul-2013, *Salix*, sweeping, leg. Krátky,J., det. Krátky,J.
Pseudomyllocerus sinuatus<br>(Fabricius, 1801)	MK891955 [new]<br>2224-JKR<br>658nt	ZFMK-TIS-23962<br>ZFMK-DNA-0169170552	Slovakia, Zilina, Suja, N49°03'46" E18°37'19", 474m, 21-Jun-2014, *Salix*, sweeping, leg. Krátky,J., det. Krátky,J.
Pseudoperapion brevirostre<br>(Herbst, 1797)	KC784133 [new]<br>647-PSP<br>658nt	ZFMK-TIS-20430<br>ZFMK-DNA-0100448620	Germany, Lower Saxony (NI), Hannover, Vahrenheide, Kugelfangtrift, N52°25'22" E09°45'25", 51m, 02-Okt-2011, *Hypericum perforatum*, nutrient-poor sand grassland, dipnet, leg. Sprick,P., det. Sprick,P.
Pseudoperapion brevirostre<br>(Herbst, 1797)	MK890934 [new]<br>803-PST<br>658nt	ZFMK-TIS-3716<br>ZFMK-DNA-0100413779	Germany, Rhineland-Palatinate (RLP), E of Treis-Karden, Pommern, Pommerner Goldberg, high road (vineyard), N50°10'14" E07°16'29", 95m, 10-Jun-2012, *Hypericum*, beating, leg. Stüben,P., det. Stüben,P.
Pseudoperapion brevirostre<br>(Herbst, 1797)	MK892314 [new]<br>2615-PSP<br>658nt	ZFMK-TIS-23589<br>ZFMK-DNA-0171661955	Germany, Bavaria (BY), Unterfranken, Ebern, N50°05'07" E10°45'52.5", 318m, 14-Jun-2014, *Hypericum perforatum*, leg. Sprick,P., det. Sprick,P.
Pseudoperapion brevirostre<br>(Herbst, 1797)	MK891041 [new]<br>939-CBR<br>658nt	ZFMK-TIS-3852<br>ZFMK-DNA-0100449665	Luxembourg, 10 km E of Luxembourg, 1 km SW of Mensdorf, N49°39'32" E06°18'35", 300m, 09-Jun-2012, sweeping, leg. Braunert,C., det. Braunert,C.
Pseudophloeophagus tenax<br>(Wollaston, 1854)	MH051937 [33]<br>1824-PST<br>658nt	ZFMK-TIS-26027<br>ZFMK-DNA-0171606142	Portugal, Madeira, S of Seixal, S of Chao da Ribeira, N32°47'28" W17°06'50", 580m, 27-Jun-2014, *Laurisilva*, beating, leg. Stüben,P., det. Stüben,P.
Pseudophloeophagus tenax<br>(Wollaston, 1854)	MK891658 [40]<br>1851-PST<br>658nt	ZFMK-TIS-26054<br>ZFMK-DNA-0171606115	Portugal, Madeira, near Boca da Encumeada, Folhadal, N32°45'10" W17°01'59", 1103m, 01-Jul-2014, beating, leg. Stüben,P., det. Stüben,P.
Pseudophloeophagus tenax<br>(Wollaston, 1854)	MK892388 [40]<br>2849-PST<br>658nt	ZFMK-TIS-4244<br>ZFMK-DNA-FC17941558	Portugal, Madeira, Sao Vincente, Lameiros, Rota da Cal, N32°47'34" W17°01'29", 288m, 02-Dez-2015, ruderal vegetation, secondary forest, beating, leg. Stüben,P. & Schütte,A., det. Stüben,P.
Pseudophytobius acalloides acalloides<br>(Fairmaire, 1857)	MK891753 [new]<br>2008-JKR<br>658nt	ZFMK-TIS-23648<br>ZFMK-DNA-0169170493	Italy, Sicilia Isl. (TP), Trapani, Salinagrande, Isolotto, N37°57'14" E12°29'45", 3m, 17-Apr-2014, *Suaeda* cf. *fruticosa*, sweeping, leg. Krátky,J., det. Krátky,J.
Pseudoprotapion astragali<br>(Paykull, 1800)	MK892029 [new]<br>2304-JKR<br>658nt	ZFMK-TIS-24235<br>ZFMK-DNA-0169170970	Czech Republic, Bohemia, Krinec, PP Chotuc, N50°15'59" E15°06'59", 236m, 02-Jul-2014, *Astragalus glycyphyllos*, sweeping, leg. Krátky,J., det. Krátky,J.

**Name**<br>**Authority**<br>**Additional Information**	**GenBank Acc No (Ref.)**<br>**Specimen ID**<br>**Sequence Length**	**ZFMK Tissue ID**<br>**ZFMK DNA Sample ID**<br>**(SDEI DNA Sample ID)**	**Locality, GPS, Collection Date, Plant, Collector, Identifier**
Pseudoprotapion astragali<br>(Paykull, 1800)	MK892106 [new]<br>2383-JKR<br>658nt	ZFMK-TIS-25960<br>ZFMK-DNA-0171600606	Czech Republic, Moravia, Hnanice, N48°47'59" E15°58'15", 310m, 10-Sep-2014, sweeping, leg. R. Stejskal, det. Stejskal,R.
Pseudoprotapion astragali<br>(Paykull, 1800)	KC784058 [new]<br>576-PSP<br>658nt	ZFMK-TIS-20361<br>ZFMK-DNA-0100448403	Germany, Lower Saxony (NI), Wesseln, Lkr. Hildesheim, Steinberg National Park, N52°04'59" E10°01'22", 157m, 26-Jun-2011, *Astragalus glycyphyllos*, dipnet, leg. Sprick,P., det. Sprick,P.
Pseudorchestes ermischi<br>(Dieckmann, 1958)	MK892239 [new]<br>2537-PSP<br>658nt	ZFMK-TIS-23509<br>ZFMK-DNA-0171661731	Slovakia, Nitra, W of Gbelce, N47°46'56" E18°27'37", 148m, 18-Mai-2014, *Centaurea scabiosa*, leg. Sprick,P., det. Sprick,P.
Pseudorchestes flavidus<br>(H. Brisout de Barneville, 1865)	MK890808 [new]<br>132-PST<br>658nt	ZFMK-TIS-2D100440295<br>ZFMK-DNA-0100438183	Morocco, S of Berkane, N of Aïn-es-Sfa, Beni Snassen Mts., N34°49'54" W02°08'49", 985m, 13-Mai-2011, *Asteraceae*, beating, leg. Stüben,P., det. Stüben,P./vid. Behne,L.
Pseudorchestes horioni<br>(Dieckmann, 1958)	MK891222 [new]<br>1142-JKR<br>658nt	ZFMK-TIS-3478<br>ZFMK-DNA-0100449566	Slovakia, Komarno, Imel, N47°54'34" E18°09'16", 112m, 11-Mai-2012, *Centaurea stoebe*, sweeping, leg. Krátky,J., det. Krátky,J.
Pseudorchestes smreczynskii<br>(Dieckmann, 1958)	MK891511 [new]<br>1597-JKR<br>658nt	ZFMK-TIS-3645<br>ZFMK-DNA-0155635643	Czech Republic, Moravia, Poudrany - step, N48°56'51" E16°38'13", 260m, 01-Mai-2013, *Artemisia absinthium*, beating, leg. Krátky,J., det. Krátky,J.
Pseudostenapion simum<br>(Germar, 1817)	MK891834 [new]<br>2095-JKR<br>658nt	ZFMK-TIS-23735<br>ZFMK-DNA-0169169638	Czech Republic, Moravia, Bucovice, PR Sevy, N49°08'05" E16°58'19", 249m, 09-Mai-2014, sweeping, leg. Krátky,J., det. Krátky,J.
Pseudostenapion simum<br>(Germar, 1817)	MK892089 [new]<br>2365-JKR<br>658nt	ZFMK-TIS-25925<br>ZFMK-DNA-0171600576	Czech Republic, Moravia, NP Podyji, Podmyce, Jejkal Pond, N48°51'24" E15°54'15.5", 420m, 17-Aug-2014, *Hypericum*, sweeping, leg. R. Stejskal, det. Stejskal,R.
Pseudostenapion simum<br>(Germar, 1817)	MK891421 [new]<br>1484-PSP<br>658nt	ZFMK-TIS-3244<br>ZFMK-DNA-0155633345	Germany, Lower Saxony (NI), Hameln-Rohrsen, Dütberg, N52°06'27" E09°25'02", 116m, 15-Jul-2012, *Hypericum perforatum*, dipnet, leg. Sprick,P., det. Sprick,P.
Pseudostenapion simum<br>(Germar, 1817)	MK892316 [new]<br>2617-PSP<br>658nt	ZFMK-TIS-23591<br>ZFMK-DNA-0171661942	Germany, Bavaria (BY), Unterfranken, Ebern, N50°05'07" E10°45'52.5", 318m, 14-Jun-2014, *Hypericum perforatum*, leg. Sprick,P., det. Sprick,P.
Pseudostenapion simum<br>(Germar, 1817)	KC784201 [new]<br>448-RGO<br>658nt	ZFMK-TIS-20234<br>ZFMK-DNA-0100449391	Poland, Rudnik, N51°14'31.02" E22°32'22.92", 191m, 03-Okt-2011, leg. Gosik,R., det. Gosik,R.
Pseudostenapion simum<br>(Germar, 1817)	KC784211 [new]<br>438-RGO<br>658nt	ZFMK-TIS-20224<br>ZFMK-DNA-0100449408	Poland, Zemborzyce, N51°09'56.7" E22°30'10.5", 178m, 18-Sep-2011, leg. Gosik,R., det. Gosik,R.
Pseudostyphlus pillumus<br>(Gyllenhal, 1835)<br>formerly: *P. pilumnus* - Syn!	MK891790 [new]<br>2049-JKR<br>658nt	ZFMK-TIS-23689<br>ZFMK-DNA-0169170452	Czech Republic, Bohemia, Pohrebacka, N50°09'15" E15°46'42", 226m, 24-Jun-2014, *Matricaia chamomylla*, sweeping, leg. Krátky,J., det. Krátky,J.
Pseudostyphlus pillumus<br>(Gyllenhal, 1835)	MK891145 [new]<br>1052-PSP<br>658nt	ZFMK-TIS-3197<br>ZFMK-DNA-0100426807	Germany, Lower Saxony (NI), Hannover, Nordhafen, N52°25'28" E09°39'10", 51m, 13-Jun-2012, *Matricaria recutita*, south exposed slope with annual ruderal area, beating, leg. Sprick,P., det. Sprick,P.
Ranunculiphilus faeculentus<br>(Gyllenhal, 1837)	MK891217 [new]<br>1136-JKR<br>658nt	ZFMK-TIS-3472<br>ZFMK-DNA-0100449573	Slovakia, Komarno, Imel, N47°54'34" E18°09'16", 112m, 11-Mai-2012, *Consolida regalis*, sweeping, leg. Krátky,J., det. Krátky,J.

**Name**<br>**Authority**<br>**Additional Information**	**GenBank Acc No (Ref.)**<br>**Specimen ID**<br>**Sequence Length**	**ZFMK Tissue ID**<br>**ZFMK DNA Sample ID**<br>**(SDEI DNA Sample ID)**	**Locality, GPS, Collection Date, Plant, Collector, Identifier**
Ranunculiphilus pseudinclemens<br>(Dieckmann, 1970)<br>Synonym of *R. faeculentus*?	MK892134 [new]<br>2413-JKR<br>658nt	ZFMK-TIS-25990<br>ZFMK-DNA-0171600624	Czech Republic, Moravia, Hruby jesenik, Velka kotlina, N50°03'17" E17°14'18", 1177m, 10-Aug-2014, *Delphinium elatum*, sweeping, leg. Bednarik,M., det. Bednarik,M.
Raymondiellus sardous sardous<br>(Perris, 1869)	MK891639 [new]<br>1790-PST<br>658nt	ZFMK-TIS-24114<br>ZFMK-DNA-0171624081	Italy, Sardinia Isl., NW of Bosa, Tre Argentina, N40°19'10" E08°27'41", 41m, 15-Mai-2014, *Ficus carica*, sieving, leg. Stüben,P. & Schütte,A., det. Stüben,P.
Raymondiellus sardous sardous<br>(Perris, 1869)	MK891640 [new]<br>1792-PST<br>658nt	ZFMK-TIS-24116<br>ZFMK-DNA-0171624083	Italy, Sardinia Isl., S of Cuglieri, R.S. Caterina , N40°08'51" E08°32'19", 254m, 16-Mai-2014, *Quercus, Pistacia*, sieving, leg. Stüben,P. & Schütte,A., det. Stüben,P.
Rhabdorrhynchus echii<br>(Brahm, 1790)	MK891379 [new]<br>1431-JKR<br>658nt	ZFMK-TIS-4200<br>ZFMK-DNA-0155628502	Slovakia, Komarno, Imel, PR Liscie diery, N47°55'12" E18°09'39", 114m, 19-Jul-2013, *Echium vulgare*, sweeping, leg. Krátky,J., det. Krátky,J.
Rhamphus oxyacanthae<br>(Marsham, 1802)	KC784050 [new]<br>577-PSP<br>658nt	ZFMK-TIS-20362<br>ZFMK-DNA-0100448391	Germany, Lower Saxony (NI), Wesseln, Lkr. Hildesheim, Steinberg National Park, N52°04'58" E10°01'22", 154m, 26-Jun-2011, *Crataegus monogyna*, beating, leg. Sprick,P., det. Sprick,P.
Rhamphus pulicarius<br>(Herbst, 1795)	MK891401 [new]<br>1461-PSP<br>658nt	ZFMK-TIS-3221<br>ZFMK-DNA-0155633368	Germany, Lower Saxony (NI), Berkhof, Hannover region, N52°36'47" E09°43'57", 36m, 30-Jun-2012, *Salix cinerea, Salix multinervis*, former gravel-pit, beating, leg. Sprick,P., det. Sprick,P.
Rhamphus pulicarius<br>(Herbst, 1795)	MK892281 [new]<br>2582-PSP<br>658nt	ZFMK-TIS-23556<br>ZFMK-DNA-0171661970	Germany, Bavaria (BY), Unterfranken, Ebern, N50°05'22" E10°46'02", 327m, 13-Jun-2014, *Salix caprea*, leg. Sprick,P., det. Sprick,P.
Rhamphus subaeneus<br>Illiger, 1807	KC783877 [40]<br>127-PST<br>587nt	ZFMK-TIS-2D100440300<br>ZFMK-DNA-0100438188	Morocco, S of Berkane, Beni Snassen Mts., Vallee Zegzel, N34°48'56" W02°24'01", 678m, 12-Mai-2011, beating, leg. Stüben,P., det. Stüben,P./vid. Behne,L.
Rhinocyllus conicus<br>(Frölich, 1792)	MK892112 [new]<br>2389-JKR<br>658nt	ZFMK-TIS-25966<br>ZFMK-DNA-0171600600	Czech Republic, Moravia, Znojmo, N48°51'05" E16°05'59", 250m, 16-Sep-2014, sweeping, leg. R. Stejskal, det. Stejskal,R.
Rhinocyllus conicus<br>(Frölich, 1792)	MK890834 [40]<br>290-PSP<br>654nt	ZFMK-TIS-2D100439088<br>ZFMK-DNA-0100439760	Germany, Hesse (HE), Wiesbaden-Schierstein, N50°02'58" E08°12'28", 283m, 31-Mai-2011, *Carduus acanthoides*, beating, leg. Sprick,P., det. Sprick,P.
Rhinocyllus conicus<br>(Frölich, 1792)	MK891474 [new]<br>1542-PSP<br>658nt	ZFMK-TIS-3302<br>ZFMK-DNA-0155633284	Germany, Lower Saxony (NI), Rethen, Hannover region, N52°16'35" E09°49'36", 60m, 25-Jun-2013, *Cirsium vulgare*, beating, leg. Sprick,P., det. Sprick,P.
Rhinocyllus conicus<br>(Frölich, 1792)	MK890859 [new]<br>413-RGO<br>658nt	ZFMK-TIS-20199<br>ZFMK-DNA-0100449431	Poland, Katy II, N50°40'39" E23°08'25", 244m, 30-Jun-2011, leg. Gosik,R., det. Gosik,R.
Rhinocyllus oblongus<br>Capiomont, 1873	MK892343 [new]<br>2703-PSP<br>658nt	ZFMK-TIS-23853<br>ZFMK-DNA-0171661307	Cyprus, Limassol district, Korfi alt, N34°48'18" E32°58'12", 505m, 09-Apr-2013, *herb layer*, dipnet, leg. Makris,C., det. Sprick,P.
Rhinocyllus oblongus<br>Capiomont, 1873	MK890809 [new]<br>142-PST<br>658nt	ZFMK-TIS-2D100440284<br>ZFMK-DNA-0100438172	Morocco, S of Berkane, N of Aïn-es-Sfa, Beni Snassen Mts., N34°51'30" W02°08'41", 840m, 13-Mai-2011, *thistle*, beating, leg. Stüben,P., det. Behne,L.

**Name**<br>**Authority**<br>**Additional Information**	**GenBank Acc No (Ref.)**<br>**Specimen ID**<br>**Sequence Length**	**ZFMK Tissue ID**<br>**ZFMK DNA Sample ID**<br>**(SDEI DNA Sample ID)**	**Locality, GPS, Collection Date, Plant, Collector, Identifier**
Rhinomias austriacus<br>(Reitter, 1894)	MK892022 [new]<br>2295-JKR<br>658nt	ZFMK-TIS-24226<br>ZFMK-DNA-0169170961	Austria, Kaernten, Feistritz ob Bleiburg, N46°32'17" E14°46'28", 702m, 28-Jun-2014, mountain forest, sieving, leg. Krátky,J., det. Krátky,J.
Rhinomias forticornis<br>(Boheman, 1842)	MK891191 [new]<br>1108-JKR<br>658nt	ZFMK-TIS-3444<br>ZFMK-DNA-0100449593	Czech Republic, Moravia, Kladeruby nad Oslavou, N49°09'31" E16°09'45", 341m, 01-Mai-2012, *quercetum*, sieving, leg. Krátky,J., det. Krátky,J.
Rhinomias forticornis<br>(Boheman, 1842)	MK891821 [new]<br>2081-JKR<br>658nt	ZFMK-TIS-23721<br>ZFMK-DNA-0169169652	Czech Republic, Bohemia, Predhradi, N49°49'56" E16°02'33", 427m, 08-Mai-2014, sieving, leg. Krátky,J., det. Krátky,J.
Rhinomias forticornis<br>(Boheman, 1842)	MK892060 [new]<br>2336-JKR<br>657nt	ZFMK-TIS-24267<br>ZFMK-DNA-0169170929	Czech Republic, Bohemia, Horni Morava, N50°10'15" E16°49'22", 780m, 17-Jul-2014, sieving, leg. Krátky,J., det. Krátky,J.
Rhinoncus albicinctus<br>Gyllenhal, 1837	MK890852 [new]<br>379-JKR<br>658nt	ZFMK-TIS-20165<br>ZFMK-DNA-0100438697	Czech Republic, Bohemia or., Hradec Kralove, Stribrny rybnik, N50°12'20.549" E15°53'32.694", 230m, 11-Jul-2011, *Polygonum aphibium*, sweeping, leg. Krátky,J., det. Krátky,J.
Rhinoncus albicinctus<br>Gyllenhal, 1837	MK891569 [new]<br>1165-JKR<br>658nt	ZFMK-TIS-3501<br>ZFMK-DNA-0155630467	Hungary, Jasz-Nagykun-Szolnok megye, Tiszaroff, N47°21'55" E20°27'32", 87m, 25-Mai-2012, *Persicaria* sp., leg. Krátky,J., det. Krátky,J.
Rhinoncus bosnicus<br>Schultze, 1900[new	MK892337 [new]<br>2658-PSP<br>658nt	ZFMK-TIS-23808<br>ZFMK-DNA-0171661341	Germany, Lower Saxony (NI), Hannover, Herrenhausen, N52°23'22.5" E09°40'36", 47m, 02-Aug-2014, *Rumex maritimus*, dipnet, leg. Sprick,P., det. Sprick,P.
Rhinoncus bruchoides<br>(Herbst, 1784)	MK892209 [new]<br>2505-PSP<br>658nt	ZFMK-TIS-23477<br>ZFMK-DNA-0171661768	Germany, Lower Saxony (NI), Helstorfer Moor, N52°32'50" E09°35'54", 46m, 06-Mai-2014, *herb layer*, peatland area, dipnet, leg. Sprick,P., det. Sprick,P.
Rhinoncus bruchoides<br>(Herbst, 1784)	KC783913 [new]<br>387-JKR<br>658nt	ZFMK-TIS-20173<br>ZFMK-DNA-0100438705	Poland, Wielkopolskie, Rogalin env., N52°14'25.971" E16°54'20.152", 58m, 09-Jul-2011, sweeping, leg. Krátky,J., det. Krátky,J.
Rhinoncus bruchoides<br>(Herbst, 1784)	MK891383 [new]<br>1436-JKR<br>658nt	ZFMK-TIS-4205<br>ZFMK-DNA-0155628497	Slovakia, Levice, Cata, Hron. Riv., N47°57'22" E18°39'39", 121m, 21-Jul-2013, *Polygonum mite*, individual collecting by hand, leg. Krátky,J., det. Krátky,J.
Rhinoncus castor<br>(Fabricius, 1792)	KC783885 [new]<br>294-JKR<br>658nt	ZFMK-TIS-20080<br>ZFMK-DNA-0100438314	Czech Republic, Bohemia or., Hradec Kralove, Plachta, N50°11'12.892" E15°51'37.148", 230m, 19-Apr-2011, *Rumex acetosella*, hand-collecting, leg. Krátky,J., det. Krátky,J.
Rhinoncus castor<br>(Fabricius, 1792)	KC784076 [new]<br>547-RST<br>658nt	ZFMK-TIS-20332<br>ZFMK-DNA-0100448432	Czech Republic, Moravia mer., Podyji NP, 0.9 km N of Hnanice - Stare vinice hill, N48°48'18.81" E15°59'26.12", 300m, 30-Sep-2011, dry heathland, hand-collecting, leg. Stejskal,R., det. Stejskal,R.
Rhinoncus castor<br>(Fabricius, 1792)	MK891733 [new]<br>1975-JKR<br>658nt	ZFMK-TIS-23615<br>ZFMK-DNA-0169170526	Czech Republic, Bohemia, Zastava, N50°06'42" E15°50'10", 234m, 11-Mrz-2014, sieving, leg. Krátky,J., det. Krátky,J.
Rhinoncus castor<br>(Fabricius, 1792)	MK891119 [new]<br>1024-PSP<br>658nt	ZFMK-TIS-3169<br>ZFMK-DNA-0100426948	Germany, Lower Saxony (NI), Braunschweig, Lindenbergsiedlung, N52°14'42" E10°33'15", 80m, 14-Mai-2012, *Rumex acetosella*, railway area, nutrient-poor sand grassland, dipnet, leg. Sprick,P., det. Sprick,P.
Rhinoncus castor<br>(Fabricius, 1792)	MK891048 [new]<br>947-CBR<br>658nt	ZFMK-TIS-3860<br>ZFMK-DNA-0100449673	Luxembourg, 8 km N of Vianden, 3 km E of Wahlhausen, N49°58'46" E06°9'32", 330m, 28-Mai-2012, *Rumex acetosella*, beating, leg. Braunert,C., det. Braunert,C.

**Name**<br>**Authority**<br>**Additional Information**	**GenBank Acc No (Ref.)**<br>**Specimen ID**<br>**Sequence Length**	**ZFMK Tissue ID**<br>**ZFMK DNA Sample ID**<br>**(SDEI DNA Sample ID)**	**Locality, GPS, Collection Date, Plant, Collector, Identifier**
Rhinoncus henningsi<br>Wagner, 1936	KC783988 [new]<br>272-PSP<br>658nt	ZFMK-TIS-2D100439099<br>ZFMK-DNA-0100439771	Germany, Lower Saxony (NI), Nordstemmen, Lkr. Hildesheim, N52°09'00" E09°47'00", 72m, 17-Mai-2011, *Bistorta affinis, Bistorta officinalis*, garden, collecting by hand, leg. Sprick,P., det. Sprick,P.
Rhinoncus inconspectus<br>(Herbst, 1795)	KC783915 [new]<br>373-JKR<br>658nt	ZFMK-TIS-20159<br>ZFMK-DNA-0100438710	Czech Republic, Bohemia or., Hrobice, N50°6'2.909" E15°46'50.867", 230m, 05-Jul-2011, *Persicaria* sp., sweeping, leg. Krátky,J., det. Krátky,J.
Rhinoncus inconspectus<br>(Herbst, 1795)	KC784091 [new]<br>540-RST<br>658nt	ZFMK-TIS-20325<br>ZFMK-DNA-0100448450	Czech Republic, Moravia mer., Vrbovec - pond, 2.1 km ESE of the village, N48°47'31.28" E16°7'43.01", 200m, 03-Sep-2011, *Polygonum*, sweeping, leg. Stejskal,R., det. Stejskal,R.
Rhinoncus inconspectus<br>(Herbst, 1795)	KC784054 [new]<br>583-PSP<br>658nt	ZFMK-TIS-20367<br>ZFMK-DNA-0100448397	Germany, Saxony-Anhalt (ST), Drömling, Mannhausen, N52°25'36" E11°12'36.5'', 54m, 07-Jun-2011, *Persicaria amphibia*, wet ruderal area, dipnet, leg. Sprick,P., det. Sprick,P.
Rhinoncus pericarpius<br>(Linnaeus, 1758)	KC783941 [new]<br>326-JKR<br>658nt	ZFMK-TIS-20112<br>ZFMK-DNA-0100438757	Czech Republic, Bohemia or., Hradec Kralove - Fararstvi, N50°11'22.247" E15°48'53.64", 230m, 17-Mai-2011, *Bistorta officinalis*, beating, leg. Krátky,J., det. Krátky,J.
Rhinoncus pericarpius<br>(Linnaeus, 1758)	MK891193 [new]<br>1110-JKR<br>658nt	ZFMK-TIS-3446<br>ZFMK-DNA-0100449595	Czech Republic, Bohemia, Hradec Kralove, Fararstvi, N50°11'22" E15°48'54", 230m, 04-Mai-2012, *Bistorta major*, beating, leg. Krátky,J., det. Krátky,J.
Rhinoncus pericarpius<br>(Linnaeus, 1758)	MK891195 [new]<br>1112-JKR<br>658nt	ZFMK-TIS-3448<br>ZFMK-DNA-0100449597	Czech Republic, Bohemia, Hradec Kralove, Trebes, N50°11'31" E15°49'10", 227m, 05-Mai-2012, *Persicaria hydropiper*, beating, leg. Krátky,J., det. Krátky,J.
Rhinoncus pericarpius<br>(Linnaeus, 1758)	MK891269 [new]<br>1209-JKR<br>658nt	ZFMK-TIS-3545<br>ZFMK-DNA-0100426078	Czech Republic, Moravia, Hruby jesenik, Velka Kotlina, N50°3'18" E17°14'14", 1209m, 23-Aug-2012, *Rumex* sp., sieving, leg. Krátky,J., det. Krátky,J.
Rhinoncus pericarpius<br>(Linnaeus, 1758)	MK892064 [new]<br>2340-JKR<br>658nt	ZFMK-TIS-24271<br>ZFMK-DNA-0169170925	Czech Republic, Bohemia, Horni Morava, N50°10'15" E16°49'22", 780m, 17-Jul-2014, *Rumex*, sweeping, leg. Krátky,J., det. Krátky,J.
Rhinoncus pericarpius<br>(Linnaeus, 1758)	KC784003 [new]<br>248-PSP<br>658nt	ZFMK-TIS-2D100438526<br>ZFMK-DNA-0100439795	Germany, Lower Saxony (NI), Essel, Lkr. Soltau-Fallingbostel, N52°41'57" E09°38'36", 25m, 10-Mai-2011, *Rumex acetosa*, Aller grassland, dipnet, leg. Sprick,P., det. Sprick,P.
Rhinoncus pericarpius<br>(Linnaeus, 1758)	MK890925 [new]<br>793-PST<br>658nt	ZFMK-TIS-3706<br>ZFMK-DNA-0100413780	Germany, Rhineland-Palatinate (RLP), E of Treis-Karden, Pommern, Pommernbachtal (river valley), N50°10'52" E07°16'42", 116m, 09-Jun-2012, *Rumex*, forest path, beating, leg. Stüben,P., det. Stüben,P.
Rhinoncus pericarpius<br>(Linnaeus, 1758)	MK891239 [new]<br>1159-JKR<br>658nt	ZFMK-TIS-3495<br>ZFMK-DNA-0100449548	Hungary, Jasz-Nagykun-Szolnok megye, Tiszaroff, N47°21'55" E20°27'32", 87m, 25-Mai-2012, *Persicaria* sp., sweeping, leg. Krátky,J., det. Krátky,J.
Rhinoncus pericarpius<br>(Linnaeus, 1758)	MK891012 [new]<br>908-CBR<br>658nt	ZFMK-TIS-3821<br>ZFMK-DNA-0100449703	Luxembourg, 10 km E of Luxembourg, 2 km S of Mensdorf, N49°38'09" E06°17'48", 330m, 08-Mai-2011, *Rumex acetosa*, sweeping, leg. Braunert,C., det. Braunert,C.
Rhinoncus pericarpius<br>(Linnaeus, 1758)	MK891285 [new]<br>1236-PST<br>658nt	ZFMK-TIS-3572<br>ZFMK-DNA-0100426057	Portugal, Minho, near Santa Comba, Rio Lima, N41°45'31 W08°37'29", 7m, 13-Mai-2012, beating, leg. Stüben,P., det. Krátky,J.
Rhinoncus pericarpius<br>(Linnaeus, 1758)	MK891218 [new]<br>1138-JKR<br>658nt	ZFMK-TIS-3474<br>ZFMK-DNA-0100449575	Slovakia, Komarno, Imel, N47°54'34" E18°09'16", 112m, 11-Mai-2012, *Rumex*, sweeping, leg. Krátky,J., det. Krátky,J.

**Name**<br>**Authority**<br>**Additional Information**	**GenBank Acc No (Ref.)**<br>**Specimen ID**<br>**Sequence Length**	**ZFMK Tissue ID**<br>**ZFMK DNA Sample ID**<br>**(SDEI DNA Sample ID)**	**Locality, GPS, Collection Date, Plant, Collector, Identifier**
Rhinoncus perpendicularis<br>(Reich, 1797)	KC784077 [new]<br>546-RST<br>629nt	ZFMK-TIS-20331<br>ZFMK-DNA-0100448433	Czech Republic, Moravia mer., Vrbovec - pond, 2.1 km ESE of the village, N48°47'31.28" E16°7'43.01", 200m, 03-Sep-2011, *Polygonum*, sweeping, leg. Stejskal,R., det. Stejskal,R.
Rhinoncus perpendicularis<br>(Reich, 1797)	KC783987 [new]<br>273-PSP<br>658nt	ZFMK-TIS-2D100438509<br>ZFMK-DNA-0100439770	Germany, Lower Saxony (NI), Nordstemmen, Lkr. Hildesheim, N52°09'00" E09°47'00", 72m, 17-Mai-2011, *Bistorta affinis, Bistorta officinalis, Bistorta amplexicaulis*, garden, collecting by hand, leg. Sprick,P., det. Sprick,P.
Rhinoncus perpendicularis<br>(Reich, 1797)	MK891244 [new]<br>1164-JKR<br>658nt	ZFMK-TIS-3500<br>ZFMK-DNA-0100449553	Hungary, Jasz-Nagykun-Szolnok megye, Tiszaroff, N47°21'55" E20°27'32", 87m, 25-Mai-2012, *Persicaria* sp., sweeping, leg. Krátky,J., det. Krátky,J.
Rhinoncus perpendicularis<br>(Reich, 1797)	KC783971 [new]<br>386-JKR<br>658nt	ZFMK-TIS-20172<br>ZFMK-DNA-0100438952	Poland, Wielkopolskie, Rogalin env., N52°14'25.971" E16°54'20.152", 58m, 09-Jul-2011, *Polygonum aviculare*, sweeping, leg. Krátky,J., det. Krátky,J.
Rhinoncus perpendicularis<br>(Reich, 1797)	MK891207 [new]<br>1125-JKR<br>658nt	ZFMK-TIS-3461<br>ZFMK-DNA-0100449583	Slovakia, Komarno, Landor, NPR Apali, N47°47'34" E18°07'49", 111m, 11-Mai-2012, *Persicaria* sp., sweeping, leg. Krátky,J., det. Krátky,J.
Rhinoncus smreczynskii<br>Wagner, 1937<br>junior synonym of Rhinoncus perpendicularis!	KC783966 [new]<br>327-JKR<br>658nt	ZFMK-TIS-20113<br>ZFMK-DNA-0100438933	Czech Republic, Bohemia or., Hradec Kralove - Fararstvi, N50°11'22.247" E15°48'53.64", 230m, 17-Mai-2011, *Bistorta officinalis*, beating, leg. Krátky,J., det. Krátky,J.
Rhinoncus smreczynskii<br>Wagner, 1937<br>junior synonym of *Rhinoncus perpendicularis*!	MK891807 [new]<br>2066-JKR<br>658nt	ZFMK-TIS-23706<br>ZFMK-DNA-0169170440	Czech Republic, Bohemia, Ujezd u Sezemic, N50°07'7.5" E15°51'37", 234m, 08-Mai-2014, *Bistorta officinalis*, sweeping, leg. Krátky,J., det. Krátky,J.
Rhinoncus smreczynskii<br>Wagner, 1937<br>junior synonym of *Rhinoncus perpendicularis*!	MK892137 [new]<br>2424-JKR<br>658nt	ZFMK-TIS-26001<br>ZFMK-DNA-0171600646	Czech Republic, Bohemia, Hradec Kralove, Slezske Predmesti, N50°12'29" E15°51'45", 231m, 13-Sep-2014, *Bistorta officinalis*, beating, leg. Krátky,J., det. Krátky,J.
Rhinoncus smreczynskii<br>Wagner, 1937<br>junior synonym of *Rhinoncus perpendicularis*!	MK892138 [new]<br>2425-JKR<br>658nt	ZFMK-TIS-26002<br>ZFMK-DNA-0171600647	Czech Republic, Bohemia, Hradec Kralove, Slezske Predmesti, N50°12'29" E15°51'45", 231m, 13-Sep-2014, *Bistorta officinalis*, beating, leg. Krátky,J., det. Krátky,J.
Rhinusa algirica<br>(H. Brisout de Barneville, 1862)	MK890807 [new]<br>130-PST<br>658nt	ZFMK-TIS-2D100438627<br>ZFMK-DNA-0100438185	Morocco, S of Berkane, N of Aïn-es-Sfa, Beni Snassen Mts., N34°46'04" W02°08'34", 688m, 12-Mai-2011, *Scrophularia*, beating, leg. Stüben,P., det. Stüben,P./vid. Behne,L.
Rhinusa antirrhini<br>(Paykull, 1800)	MK890851 [new]<br>378-JKR<br>658nt	ZFMK-TIS-20164<br>ZFMK-DNA-0100438696	Czech Republic, Bohemia or., Hrobice, N50°6'2.909" E15°46'50.867", 230m, 05-Jul-2011, *Linaria vulgaris*, sweeping, leg. Krátky,J., det. Krátky,J.
Rhinusa antirrhini<br>(Paykull, 1800)	MK891430 [new]<br>1493-PSP<br>658nt	ZFMK-TIS-3253<br>ZFMK-DNA-0155633331	Germany, Saxony-Anhalt (ST), Harzrand, Stapelburg, N51°54'01" E10°39'04", 226m, 01-Aug-2012, *Linaria vulgaris*, wyaside, dipnet, leg. Sprick,P., det. Sprick,P.
Rhinusa asellus<br>(Gravenhorst, 1807)	MK890841 [new]<br>311-JKR<br>658nt	ZFMK-TIS-20097<br>ZFMK-DNA-0100438773	Czech Republic, Bohemia or., Uhretice, N49°58'40.663" E15°51'28.144", 240m, 14-Mai-2011, *Verbascum* sp., beating, leg. Krátky,J., det. Krátky,J.

**Name**<br>**Authority**<br>**Additional Information**	**GenBank Acc No (Ref.)**<br>**Specimen ID**<br>**Sequence Length**	**ZFMK Tissue ID**<br>**ZFMK DNA Sample ID**<br>**(SDEI DNA Sample ID)**	**Locality, GPS, Collection Date, Plant, Collector, Identifier**
Rhinusa asellus<br>(Gravenhorst, 1807)	MK891105 [new]<br>1010-PSP<br>658nt	ZFMK-TIS-3155<br>ZFMK-DNA-0100426840	Germany, Lower Saxony (NI), Hannover, Leinhausen, N52°23'58" E09°41'12", 49m, 07-Mai-2012, *Verbascum densiflorum*, train station area, by hand, leg. Sprick,P., det. Sprick,P.
Rhinusa asellus<br>(Gravenhorst, 1807)	MK892246 [new]<br>2545-PSP<br>658nt	ZFMK-TIS-23519<br>ZFMK-DNA-0171662014	Slovakia, Nitra, Cenkov, N47°46'07" E18°31'11", 109m, 19-Mai-2014, *Verbascum* sp., leg. Sprick,P., det. Sprick,P.
Rhinusa florum<br>(Rübsaamen, 1895)	MK891963 [new]<br>2232-JKR<br>658nt	ZFMK-TIS-23970<br>ZFMK-DNA-0169170560	Slovakia, Komarno, Virt, N47°45'39" E18°20'13.5", 123m, 16-Jun-2014, *Linaria genistifolia*, leg. Benedikt,S., det. Benedikt,S.
Rhinusa linariae<br>(Panzer, 1795)	MK891940 [new]<br>2208-JKR<br>658nt	ZFMK-TIS-23946<br>ZFMK-DNA-0169170584	Czech Republic, Moravia, Znojmo, N48°51'12.5" E16°06'36", 245m, 08-Mai-2014, *Linaria vulgaris*, beating, leg. Stejskal,R., det. Stejskal,R.
Rhinusa linariae<br>(Panzer, 1795)	MK891154 [new]<br>1061-PSP<br>658nt	ZFMK-TIS-3206<br>ZFMK-DNA-0100426795	Germany, Lower Saxony (NI), Langeoog, N53°45'07" E07°32'25", 3m, 16-Jun-2012, *Linaria vulgaris*, gray/brown dues, beating, leg. Sprick,P., det. Sprick,P.
Rhinusa linariae<br>(Panzer, 1795)	MK892210 [new]<br>2506-PSP<br>658nt	ZFMK-TIS-23478<br>ZFMK-DNA-0171661767	Germany, Lower Saxony (NI), Helstorfer Moor, N52°33'08" E09°35'45.5", 43m, 06-Mai-2014, *Linaria vulgaris*, peatland area, beating, leg. Sprick,P., det. Sprick,P.
Rhinusa linariae<br>(Panzer, 1795)	MK891543 [new]<br>1632-JKR<br>658nt	ZFMK-TIS-3680<br>ZFMK-DNA-0155635597	Slovakia, Komarno, Palarikovo, Neded, Jahodnianske jazierko NR, N48°01'33" E17°59'49", 110m, 20-Mai-2013, *Linaria vulgaris*, sweeping, leg. Krátky,J., det. Krátky,J.
Rhinusa neta<br>(Germar, 1821)	KC783907 [new]<br>377-JKR<br>658nt	ZFMK-TIS-20163<br>ZFMK-DNA-0100438695	Czech Republic, Bohemia or., Hrobice, N50°6'2.909" E15°46'50.867", 230m, 05-Jul-2011, *Linaria vulgaris*, sweeping, leg. Krátky,J., det. Krátky,J.
Rhinusa neta<br>(Germar, 1821)	KC784035 [new]<br>605-PSP<br>658nt	ZFMK-TIS-20388<br>ZFMK-DNA-0100448224	Germany, Hesse (HE), Wiesbaden-Schierstein, N50°02'58" E08°12'28", 97m, 29-Jun-2011, *Linaria vulgaris*, beating, leg. Sprick,P., det. Sprick,P.
Rhinusa neta<br>(Germar, 1821)	MK891439 [new]<br>1502-PSP<br>658nt	ZFMK-TIS-3262<br>ZFMK-DNA-0155633327	Germany, Lower Saxony (NI), Braunschweig, Lindenbergsiedlung, N52°14'51" E10°33'17", 83m, 24-Aug-2012, *Linaria vulgaris*, beating, leg. Sprick,P., det. Sprick,P.
Rhinusa neta<br>(Germar, 1821)	MK891535 [new]<br>1624-JKR<br>658nt	ZFMK-TIS-3672<br>ZFMK-DNA-0155635605	Slovakia, Nove Zamky, Kamenica nad Hronom, N47°49'34" E18°44'31", 109m, 19-Mai-2013, *Linaria genistifolia*, individual collecting by hand, leg. Krátky,J., det. Krátky,J.
Rhinusa tetra<br>(Fabricius, 1792)	MK890881 [new]<br>514-RST<br>658nt	ZFMK-TIS-20299<br>ZFMK-DNA-0100448472	Czech Republic, Moravia mer., kaolin pit 1.5 km SE of Únanov, N48°53'17.75" E16°3'22.34", 330m, 02-Jul-2011, sweeping, leg. Stejskal,R., det. Stejskal,R.
Rhinusa tetra<br>(Fabricius, 1792)	MK891260 [new]<br>1196-JKR<br>658nt	ZFMK-TIS-3532<br>ZFMK-DNA-0100426088	Czech Republic, Bohemia, Hradec Kralove - Brezhrad, N50°10'45" E15°47'39", 229m, 29-Jun-2012, *Verbascum*, beating, leg. Krátky,J., det. Krátky,J.
Rhinusa tetra<br>(Fabricius, 1792)	MK892327 [new]<br>2628-PSP<br>658nt	ZFMK-TIS-23602<br>ZFMK-DNA-0171661920	Germany, Bavaria (BY), Unterfranken, Ebern, N50°04'55" E10°45'52", 297m, 14-Jun-2014, *Verbascum densiflorum*, leg. Sprick,P., det. Sprick,P.
Rhinusa tetra<br>(Fabricius, 1792)	MK892330 [new]<br>2632-PSP<br>658nt	ZFMK-TIS-23606<br>ZFMK-DNA-0171661924	Germany, Lower Saxony (NI), Hannover, Nordhafen, N52°25'28" E09°39'12", 52m, 22-Jun-2014, *Verbascum phlomoides*, beating, leg. Sprick,P., det. Sprick,P.

**Name**<br>**Authority**<br>**Additional Information**	**GenBank Acc No (Ref.)**<br>**Specimen ID**<br>**Sequence Length**	**ZFMK Tissue ID**<br>**ZFMK DNA Sample ID**<br>**(SDEI DNA Sample ID)**	**Locality, GPS, Collection Date, Plant, Collector, Identifier**
Rhinusa tetra<br>(Fabricius, 1792)	KC784245 [new]<br>IT-0012w<br>651nt	ZFMK-TIS-2D100446540<br>ZFMK-DNA-0100437858	Italy, Sardinia Isl. East, E of Seui, Mt. Arqueri, N39°49'36" E09°22'27", 960m, 29-Sep-2010, *Verbascum*, leg. Stüben,P., det. Stüben,P.
Rhinusa tetra<br>(Fabricius, 1792)	MK891024 [new]<br>921-CBR<br>658nt	ZFMK-TIS-3834<br>ZFMK-DNA-0100449695	Luxembourg, 6 km E of Luxembourg, Oetrange, N49°36'20" E06°15'32", 250m, 14-Jun-2011, *Verbascum densiflorum*, beating, leg. Braunert,C., det. Braunert,C.
Rhinusa tetra<br>(Fabricius, 1792)	MK892235 [new]<br>2533-PSP<br>658nt	ZFMK-TIS-23505<br>ZFMK-DNA-0171661740	Slovakia, Nitra, SW of Buc, N47°46'56" E18°25'10", 110m, 18-Mai-2014, *Verbascum* sp., leg. Sprick,P., det. Sprick,P.
Rhopalapion longirostre<br>(Olivier, 1807)	MK890874 [new]<br>486-PST<br>658nt	ZFMK-TIS-20271<br>ZFMK-DNA-0100449360	France, Vaucluse, Bedoin, N44°07'42" E05°11'43", 345m, 30-Jul-2011, *Alcea rosea*, beating, leg. Stüben,P., det. Stüben,P.
Rhopalapion longirostre<br>(Olivier, 1807)	MK891138 [new]<br>1043-PSP<br>658nt	ZFMK-TIS-3188<br>ZFMK-DNA-0100426825	Germany, Lower Saxony (NI), Hannover, Linden, Stammestraße, N52°21'01" E09°43'44", 55m, 30-Mai-2012, *Alcea rosea*, beating, leg. Sprick,P., det. Sprick,P.
Rhopalapion longirostre<br>(Olivier, 1807)	MK891045 [new]<br>944-CBR<br>658nt	ZFMK-TIS-3857<br>ZFMK-DNA-0100449670	Luxembourg, 10 km E of Luxembourg, Mensdorf, N49°39'33" E06°18'13", 240m, 10-Jun-2012, *Alcea rosea*, pooter, leg. Braunert,C., det. Braunert,C.
Rhopalomesites complanatus<br>(Wollaston, 1861)	MH051935 [33]<br>1707-PST<br>658nt	ZFMK-TIS-24425<br>ZFMK-DNA-0155622488	Spain, Canary Islands, La Palma, NW of Puntallana, Cubo de la Galga, N28°45'38" W17°46'42", 537m, 27-Dez-2013, *Laurus*, beating, leg. Stüben,P., det. Stüben,P.
Rhopalomesites complanatus<br>(Wollaston, 1861)	MH618679 [40]<br>1985-JKR<br>658nt	ZFMK-TIS-23625<br>ZFMK-DNA-0169170516	Spain, Canary Islands, La Palma, Cubo de Galga, N28°45'42" W17°46'34", 467m, 07-Feb-2014, *Laurisilva*, beating, leg. Krátky,J., det. Krátky,J.
Rhopalomesites euphorbiae<br>(Wollaston, 1854)	MH051946 [33]<br>1865-PST<br>658nt	ZFMK-TIS-26068<br>ZFMK-DNA-0171606102	Portugal, Madeira, S of Boaventura, Achada da Madeira, N32°47'03" W16°58'41", 488m, 03-Jul-2014, *Euphorbia mellifera*, beating, leg. Stüben,P., det. Stüben,P.
Rhopalomesites euphorbiae<br>(Wollaston, 1854)	MH618677 [40]<br>1826-PST<br>658nt	ZFMK-TIS-26029<br>ZFMK-DNA-0171606140	Portugal, Madeira, S of Seixal, S of Chao da Ribeira, N32°48'41" W17°06'38", 411m, 27-Jun-2014, *Euphorbia mellifera*, beating, leg. Stüben,P., det. Stüben,P.
Rhopalomesites euphorbiae<br>(Wollaston, 1854)	MH618678 [40]<br>1849-PST<br>658nt	ZFMK-TIS-26052<br>ZFMK-DNA-0171606117	Portugal, Madeira, Ribeira da Janela near coast, N32°51'15" W17°09'14", 27m, 29-Jun-2014, *Euphorbia piscatoria*, beating, leg. Stüben,P., det. Stüben,P.
Rhopalomesites euphorbiae<br>(Wollaston, 1854)	MH618682 [40]<br>2825-PST<br>658nt	ZFMK-TIS-25872<br>ZFMK-DNA-FC17941556	Portugal, Madeira, near Machico, Queimada, N32°42'47" W16°45'56", 114m, 23-Nov-2015, *Euphorbia piscatoria*, beating, leg. Stüben,P. & Schütte,A., det. Stüben,P.
Rhopalomesites maderensis<br>(Wollaston, 1854)	MH051942 [33]<br>1843-PST<br>658nt	ZFMK-TIS-26046<br>ZFMK-DNA-0171606128	Portugal, Madeira, SE of Ribeira da Janela, Fanal, N32°48'48" W17°08'57", 1084m, 29-Jun-2014, *Ocotea foetens*, beating, leg. Stüben,P., det. Stüben,P.
Rhopalomesites proximus<br>(Wollaston, 1861)	MH051936 [33]<br>1752-PST<br>658nt	ZFMK-TIS-24470<br>ZFMK-DNA-0155622437	Spain, Canary Islands, La Palma, Pared Vieja, Ladera Espinel, N28°37'35" W17°49'16", 672m, 20-Jan-2014, *Euphorbia mellifera* , beating, leg. Stüben,P., det. Stüben,P.
Rhynchaenus xylostei<br>Clairville, 1798	MK891090 [new]<br>994-PSP<br>658nt	ZFMK-TIS-3139<br>ZFMK-DNA-0100426293	Germany, Lower Saxony (NI), Diekholzen, Lkr. Hildesheim, N52°05'04" E09°55'17", 152m, 01-Mai-2012, *Lonicera xylosteum*, forest of Hildesheim, beating, leg. Sprick,P., det. Sprick,P.

**Name**<br>**Authority**<br>**Additional Information**	**GenBank Acc No (Ref.)**<br>**Specimen ID**<br>**Sequence Length**	**ZFMK Tissue ID**<br>**ZFMK DNA Sample ID**<br>**(SDEI DNA Sample ID)**	**Locality, GPS, Collection Date, Plant, Collector, Identifier**
Rhynchites auratus<br>(Scopoli, 1763)	MK890892 [new]<br>569-PSP<br>658nt	ZFMK-TIS-20354<br>ZFMK-DNA-0100448410	Germany, Lower Saxony (NI), Neindorf, Lkr. Wolfenbüttel, Öselberg, N52°07'33'' E10°35'40'', 136m, 24-Jun-2011, *Prunus spinosa*, beating, leg. Sprick,P., det. Sprick,P.
Rhynchites auratus<br>(Scopoli, 1763)	MK890904 [new]<br>681-FBA<br>658nt	ZFMK-TIS-20459<br>ZFMK-DNA-0100448591	Greece, Peloponnese, Messinia, W of Mt. Taygetos, E of Saidona, N36°53'01'' E22°17'07'', 680m, 23-Mai-2011, leg. Bayer,B., det. Stüben,P.
Rhynchites bacchus<br>(Linnaeus, 1758)	KR817567 [20]<br>2167-JKR<br>658nt	ZFMK-TIS-23903<br>ZFMK-DNA-0169170620	Czech Republic, Moravia, Znojmo, N48°51'32" E16°4'28.5", 255m, 12-Apr-2014, *Prunus avium*, beating, leg. Stejskal,R., det. Stejskal,R.
Rhyncolus ater<br>(Linne, 1758)	MK892334 [new]<br>2636-PSP<br>658nt	ZFMK-TIS-23610<br>ZFMK-DNA-0171661928	Germany, Saxony-Anhalt (ST), Schierke, Drei Annen Hohne, Hohnehof, N51°46'35" E10°43'10", 608m, 10-Jul-2014, *Picea abies*, collecting by hand, leg. Sprick,P., det. Sprick,P.
Rhyncolus punctatulus<br>Boheman, 1838	MK892332 [new]<br>2634-PSP<br>658nt	ZFMK-TIS-23608<br>ZFMK-DNA-0171661926	Germany, Lower Saxony (NI), Hannover, Herrenhausen, "Berggarten" area, N52°23'41" E09°41'56", 55m, 04-Jul-2014, *Tilia europaea*, sieving, leg. Sprick,P., det. Sprick,P.
Rhyncolus sculpturatus<br>Waltl, 1839	MK891560 [new]<br>1654-JKR<br>658nt	ZFMK-TIS-4232<br>ZFMK-DNA-0155630486	Slovakia, Lucenec, Lentvora, Lysec hill, N48°21'00" E19°27'36", 690m, 12-Okt-2013, *Pinus*, individual collecting by hand, leg. Krátky,J., det. Krátky,J.
Rhytideres plicatus<br>(Olivier, 1790)	MK890802 [40]<br>27-PST<br>658nt	ZFMK-TIS-2D100447020<br>ZFMK-DNA-0100438092	Spain, Canary Islands, Gran Canaria, S of Guia, Barranco del Pinar, field edge, N28°04'21" W15°37'38", 969m, 21-Dez-2010, *Brassicaceae*, beating, leg. Stüben,P., det. Stüben,P.
Romualdius angustisetulus<br>(V. Hansen, 1915)	KC784070 [new]<br>563-RST<br>658nt	ZFMK-TIS-20348<br>ZFMK-DNA-0100448425	Czech Republic, Moravia mer., 1 km NE of Derflice, N48°48'58.36" E16°9'13.58", 300m, 16-Okt-2011, sieving, leg. Stejskal,R., det. Stejskal,R.
Romualdius angustisetulus<br>(V. Hansen, 1915)	MK891028 [new]<br>925-CBR<br>658nt	ZFMK-TIS-3838<br>ZFMK-DNA-0100449131	Luxembourg, 10 km NW of Ettelbruck, 1 km E of Goebelsmühle, N49°55'15" E06°03'34", 320m, 15-Okt-2011, sieving, leg. Braunert,C., det. Braunert,C.
Romualdius angustisetulus<br>(V. Hansen, 1915)	MK891300 [new]<br>1277-PST<br>658nt	ZFMK-TIS-4093<br>ZFMK-DNA-0100426194	Spain, Community of Madrid, NE of Montejo de la Sierra, Hayedo de Montejo, Puerto de El Cardoso, N41°05'14" W03°29'21", 1386m, 02-Mai-2013, *Quercus*, sieving, leg. Stüben,P. & Schütte,A., det. Borovec,R.
Romualdius scaber<br>(Beck, 1817)	MK891732 [new]<br>1974-JKR<br>658nt	ZFMK-TIS-23614<br>ZFMK-DNA-0169170527	Czech Republic, Bohemia, Zastava, N50°06'42" E15°50'10", 234m, 11-Mrz-2014, sieving, leg. Krátky,J., det. Krátky,J.
Romualdius scaber<br>(Beck, 1817)	MK891794 [40]<br>2053-JKR<br>658nt	ZFMK-TIS-23693<br>ZFMK-DNA-0169170448	Czech Republic, Bohemia, Kosatky, N50°19'23" E14°39'58", 218m, 02-Mai-2014, sieving, leg. Krátky,J., det. Krátky,J.
Romualdius scaber<br>(Beck, 1817)	KC784021 [new]<br>209-PSP<br>658nt	ZFMK-TIS-2D100439151<br>ZFMK-DNA-0100439823	Germany, Lower Saxony (NI), Hannover, Vahrenheide, Kugelfangtrift, N52°25'18" E09°45'07", 50m, 10-Apr-2011, *Plantago lanceolata*, collecting by hand, leg. Sprick,P., det. Sprick,P.
Rutidosoma graminosus<br>Gistel, 1857	MK892211 [new]<br>2507-PSP<br>658nt	ZFMK-TIS-23479<br>ZFMK-DNA-0171661766	Germany, Lower Saxony (NI), Otternhagener peatland, NO edge, N52°31'09" E09°36'03", 48m, 06-Mai-2014, *Populus tremula*, beating, leg. Sprick,P., det. Sprick,P.
Sciaphilus asperatus<br>(Bonsdorff, 1785)	MK890845 [new]<br>329-JKR<br>658nt	ZFMK-TIS-20115<br>ZFMK-DNA-0100438743	Czech Republic, Bohemia or., Hradec Kralove - Fararstvi, N50°11'22.247" E15°48'53.64", 230m, 17-Mai-2011, sweeping, leg. Krátky,J., det. Krátky,J.

**Name**<br>**Authority**<br>**Additional Information**	**GenBank Acc No (Ref.)**<br>**Specimen ID**<br>**Sequence Length**	**ZFMK Tissue ID**<br>**ZFMK DNA Sample ID**<br>**(SDEI DNA Sample ID)**	**Locality, GPS, Collection Date, Plant, Collector, Identifier**
Sciaphilus asperatus<br>(Bonsdorff, 1785)	MK890817 [new]<br>203-PSP<br>658nt	ZFMK-TIS-2D100438891<br>ZFMK-DNA-0100438522	Germany, Lower Saxony (NI), Nordstemmen, Lkr. Hildesheim, N52°09' E09°47', 72m, 06-Apr-2011, *Paeonia officinalis*, garden, beating, leg. Sprick,P., det. Sprick,P.
Sciaphilus asperatus<br>(Bonsdorff, 1785)	MK891409 [new]<br>1471-PSP<br>658nt	ZFMK-TIS-3231<br>ZFMK-DNA-0155633357	Germany, Lower Saxony (NI), Harz, St. Andreasberg, Jordanshöhe, N51°42'58" E10°32'03", 667m, 02-Jul-2012, *Geranium sylvaticum*, perennials corridor, beating, leg. Sprick,P., det. Sprick,P.
Sciaphilus asperatus<br>(Bonsdorff, 1785)	MK892202 [new]<br>2497-PSP<br>658nt	ZFMK-TIS-23469<br>ZFMK-DNA-0171661787	Germany, Saxony-Anhalt (ST), Harz National Park, Schierke, Feuersteinwiesen, N51°45'21" E10°41'25", 604m, 24-Apr-2014, *herb layer*, dipnet, leg. Sprick,P., det. Sprick,P.
Sciaphilus asperatus<br>(Bonsdorff, 1785)	MK890867 [new]<br>443-RGO<br>658nt	ZFMK-TIS-20229<br>ZFMK-DNA-0100449396	Poland, Zurawce, N50°34'3.6" E23°51'57.6", 248m, 21-Sep-2011, leg. Gosik,R., det. Gosik,R.
Sciaphilus asperatus<br>(Bonsdorff, 1785)	MK890868 [new]<br>458-RGO<br>627nt	ZFMK-TIS-20244<br>ZFMK-DNA-0100449380	Poland, Rudnik, N51°14'31.02" E22°32'22.92", 191m, 03-Okt-2011, leg. Gosik,R., det. Gosik,R.
Sciaphobus caesius<br>(Hampe, 1870)	KC784092 [new]<br>528-RST<br>658nt	ZFMK-TIS-20313<br>ZFMK-DNA-0100448451	Romania, Caras-Severin, Sfanta Elena env., N44°40'54.68" E21°42'39.99", 400m, 26-Jul-2011, night-sweeping, leg. Stejskal,R., det. Stejskal,R.
Sciaphobus rubi<br>(Gyllenhal, 1813)	MK890840 [new]<br>305-JKR<br>658nt	ZFMK-TIS-20091<br>ZFMK-DNA-0100438767	Czech Republic, Bohemia or., Hradec Kralove, Fararstvi, N50°11'26.901" E15°48'57.048", 230m, 06-Mai-2011, sweeping, leg. Krátky,J., det. Krátky,J.
Sciaphobus scitulus<br>(Germar, 1824)	MK890882 [new]<br>516-RST<br>658nt	ZFMK-TIS-20301<br>ZFMK-DNA-0100448474	Czech Republic, Bohemia bor., Bile strane National Nature Monument, 2.5 km N of Litomerice, N50°33'32.39" E14°7'58.09", 340m, 05-Jul-2011, *Cytisus*, beating, leg. Stejskal,R., det. Stejskal,R.
Sciaphobus scitulus<br>(Germar, 1824)	MK892028 [new]<br>2303-JKR<br>658nt	ZFMK-TIS-24234<br>ZFMK-DNA-0169170969	Czech Republic, Bohemia, Krinec, PP Chotuc, N50°15'59" E15°06'59", 236m, 02-Jul-2014, steppic meadow, sweeping, leg. Krátky,J., det. Krátky,J.
Scleropteridius fallax<br>Otto, 1897	MK891998 [new]<br>2269-JKR<br>658nt	ZFMK-TIS-24200<br>ZFMK-DNA-0169170996	Austria, Styria, Hochschwab, Stallmauer, N47°35'29" E15°02'20", 1260m, 27-Jun-2014, mountain forest, sweeping, leg. Krátky,J., det. Krátky,J.
Scleropteridius fallax<br>Otto, 1897	MK892024 [new]<br>2297-JKR<br>658nt	ZFMK-TIS-24228<br>ZFMK-DNA-0169170963	Austria, Kaernten, Feistritz ob Bleiburg, N46°32'17" E14°46'28", 702m, 28-Jun-2014, mountain forest, sieving, leg. Krátky,J., det. Krátky,J.
Scleropteridius fallax<br>Otto, 1897	MK891102 [new]<br>1006-PSP<br>658nt	ZFMK-TIS-3151<br>ZFMK-DNA-0100426245	Germany, Lower Saxony (NI), near Hannover, Bredenbeck-Steinkrug, Deister, N52°14'01" E09°37'41", 182m, 05-Mai-2012, *Oxalis acetosella*, broad-leaved forest, dipnet, leg. Sprick,P., det. Sprick,P.
Scleropteridius fallax<br>Otto, 1897	MK892174 [new]<br>2466-PSP<br>658nt	ZFMK-TIS-23438<br>ZFMK-DNA-0171661804	Germany, Saxony-Anhalt (ST), Harz National Park, Eckertal, N51°50'50" E10°34'41", 524m, 30-Mai-2013, *Oxalis acetosella*, collecting by hand, leg. Sprick,P., det. Sprick,P.
Scleropterus offensus<br>Boheman, 1837	MK892019 [new]<br>2292-JKR<br>658nt	ZFMK-TIS-24223<br>ZFMK-DNA-0169170973	Austria, Kaernten, Petzen, N46°31'01" E14°46'15", 1698m, 28-Jun-2014, *Geum rivale*, collecting by hand, leg. Krátky,J., det. Krátky,J.
Scleropterus serratus noesskei<br>Apfelbeck, 1928	MK891580 [new]<br>359-JKR<br>658nt	ZFMK-TIS-20145<br>ZFMK-DNA-0155630446	Bulgaria, Stara Planina, Berkovska Planina Mts., Petrochan pass, N43°07'24.37" E23°07'40.50", 1390m, 11-Jun-2011, *Geum rivale*, beating, leg. Krátky,J., det. Krátky,J.

**Name**<br>**Authority**<br>**Additional Information**	**GenBank Acc No (Ref.)**<br>**Specimen ID**<br>**Sequence Length**	**ZFMK Tissue ID**<br>**ZFMK DNA Sample ID**<br>**(SDEI DNA Sample ID)**	**Locality, GPS, Collection Date, Plant, Collector, Identifier**
Scleropterus serratus serratus<br>(Germar, 1824)	MK892063 [new]<br>2339-JKR<br>658nt	ZFMK-TIS-24270<br>ZFMK-DNA-0169170926	Czech Republic, Bohemia, Horni Morava, N50°10'15" E16°49'22", 780m, 17-Jul-2014, sweeping, leg. Krátky,J., det. Krátky,J.
Scleropterus serratus serratus<br>(Germar, 1824)	MK891362 [new]<br>1413-JKR<br>658nt	ZFMK-TIS-4182<br>ZFMK-DNA-0155628520	Slovakia, Vysoke Tatry Mts., Skalne vrata, N49°13'39" E20°16'42", 1512m, 30-Jun-2013, sweeping, leg. Krátky,J., det. Krátky,J.
Scleropterus serratus serratus<br>(Germar, 1824)	MK891944 [new]<br>2212-JKR<br>658nt	ZFMK-TIS-23950<br>ZFMK-DNA-0169170575	Slovakia, Kezmarok, Vysoke Tatry Mts., Zadne Medodoly valley, N49°14'09" E20°12'15", 1509m, 24-Jun-2014, leg. Benedikt,S., det. Benedikt,S.
Scleropterus serratus serratus<br>(Germar, 1824)	MK892121 [new]<br>2398-JKR<br>658nt	ZFMK-TIS-25975<br>ZFMK-DNA-0171600620	Slovakia, Zilina, Suja, N49°03'46" E18°37'19", 474m, 21-Jun-2014, sweeping, leg. Krátky,J., det. Krátky,J.
Sibinia albosquamosa<br>Pic, 1904	MK891725 [40]<br>1959-PST<br>658nt	ZFMK-TIS-24038<br>ZFMK-DNA-0169170389	Portugal, Madeira, Ilhas Selvagens, Selvagem Pequena, around Pico do Veado, N30°02'09" W16°01'39", 15m, 07-Okt-2014, sieving, leg. Stüben,P., det. Stüben,P.
Sibinia albosquamosa<br>Pic, 1904	MK891629 [40]<br>1749-PST<br>658nt	ZFMK-TIS-24467<br>ZFMK-DNA-0155622434	Spain, Canary Islands, La Palma, W of Garafia near Puerto Garafia, N28°49'33" W17°57'53", 126m, 17-Jan-2014, *Limonium pectinatum*, beating, leg. Stüben,P., det. Stüben,P.
Sibinia albosquamosa<br>Pic, 1904	KC784295 [40]<br>724-PST<br>610nt	ZFMK-TIS-3061<br>ZFMK-DNA-0100446728	Spain, Canary Islands, Tenerife, Reserva Natural Especial Mailpais de la Rasco, N28°00'57" W16°41'19", 56m, 23-Jan-2012, *Limonium pectinatum*, beating, leg. Stüben,P., det. Stüben,P.
Sibinia albosquamosa<br>Pic, 1904	KC784311 [40]<br>756-PST<br>658nt	ZFMK-TIS-3093<br>ZFMK-DNA-0100448134	Spain, Canary Islands, Tenerife, near San Juan de Rambla, Playa del Socorro, coast, N28°23'38" W16°36'11", 5m, 20-Feb-2012, *Limonium*, beating/sifting, leg. Stüben,P., det. Stüben,P.
Sibinia albosquamosa<br>Pic, 1904	MK891590 [40]<br>729-PST<br>563nt	ZFMK-TIS-3066<br>ZFMK-DNA-0155630432	Spain, Canary Islands, Tenerife, near Los Silos, coast, Playa de Agua Dulce, Sibora, N28°22'40" W16°48'21", 19m, 24-Jan-2012, *Limonium pectinatum*, beating, leg. Stüben,P., det. Stüben,P.
Sibinia beckeri<br>Desbrochers des Loges, 1873	MK892146 [new]<br>2433-JKR<br>658nt	ZFMK-TIS-26010<br>ZFMK-DNA-0171600652	Slovakia, Nove Zamky, Kamenin, PP Cistiny, N47°51'55" E18°38'05", 111m, 21-Sep-2014, *Limonium gmelinii* ssp. *hungaricum*, beating, leg. Krátky,J., det. Krátky,J.
Sibinia femoralis<br>Germar, 1824	MK891756 [new]<br>2011-JKR<br>658nt	ZFMK-TIS-23651<br>ZFMK-DNA-0169170481	Italy, Sicilia Isl. (TP), Trapani, Salinagrande, Isolotto, N37°57'14" E12°29'45", 3m, 17-Apr-2014, sweeping, leg. Krátky,J., det. Krátky,J.
Sibinia femoralis<br>Germar, 1824	MK892181 [new]<br>2474-PSP<br>658nt	ZFMK-TIS-23446<br>ZFMK-DNA-0171661799	Slovakia, Nitra, Cenkov, N47°46'07" E18°31'11", 109m, 19-Mai-2014, *Silene otites*, collecting by hand, leg. Sprick,P., det. Sprick,P.
Sibinia pellucens<br>(Scopoli, 1772)	KC783984 [new]<br>288-PSP<br>658nt	ZFMK-TIS-2D100438553<br>ZFMK-DNA-0100439758	Germany, Hesse (HE), Wiesbaden-Schierstein, N50°02'58" E08°12'28", 283m, 31-Mai-2011, *Silene latifolia* ssp. *alba*, dipnet, leg. Sprick,P., det. Sprick,P.
Sibinia pellucens<br>(Scopoli, 1772)	MK890935 [new]<br>804-PST<br>645nt	ZFMK-TIS-3717<br>ZFMK-DNA-0100414298	Germany, Rhineland-Palatinate (RLP), E of Cochem, Valwig, Apolloweg, N50°08'45" E07°12'35", 234m, 10-Jun-2012, high road (vineyard), beating, leg. Stüben,P., det. Stüben,P.
Sibinia pellucens<br>(Scopoli, 1772)	MK892298 [new]<br>2599-PSP<br>658nt	ZFMK-TIS-23573<br>ZFMK-DNA-0171661960	Germany, Bavaria (BY), Unterfranken, Ebern, N50°05'03" E10°45'15", 331m, 14-Jun-2014, *Sibinia latifolia* ssp.*alba*, leg. Sprick,P., det. Sprick,P.

**Name**<br>**Authority**<br>**Additional Information**	**GenBank Acc No (Ref.)**<br>**Specimen ID**<br>**Sequence Length**	**ZFMK Tissue ID**<br>**ZFMK DNA Sample ID**<br>**(SDEI DNA Sample ID)**	**Locality, GPS, Collection Date, Plant, Collector, Identifier**
Sibinia pellucens<br>(Scopoli, 1772)	MK891015 [new]<br>911-CBR<br>658nt	ZFMK-TIS-3824<br>ZFMK-DNA-0100449700	Luxembourg, 10 km E of Luxembourg, 2 km S of Mensdorf, N49°38'09" E06°17'48", 330m, 08-Mai-2011, *Melandrium album*, sweeping, leg. Braunert,C., det. Braunert,C.
Sibinia pellucens<br>(Scopoli, 1772)	MK892231 [new]<br>2529-PSP<br>658nt	ZFMK-TIS-23501<br>ZFMK-DNA-0171661744	Slovakia, Nitra, SW of Buc, N47°46'56" E18°25'10", 110m, 18-Mai-2014, *Silene latifolia*, leg. Sprick,P., det. Sprick,P.
Sibinia phalerata<br>Gyllenhal, 1835	MK892110 [new]<br>2387-JKR<br>658nt	ZFMK-TIS-25964<br>ZFMK-DNA-0171600602	Czech Republic, Moravia, Dyjakovicky, Jecmenist?, N48°44'48" E16°08'47", 250m, 11-Sep-2014, sweeping, leg. R. Stejskal, det. Stejskal,R.
Sibinia phalerata<br>Gyllenhal, 1835	MK892155 [new]<br>2443-JKR<br>658nt	ZFMK-TIS-26020<br>ZFMK-DNA-0171600665	Slovakia, Nove Zamky, Imel, PR Liscie diery, N47°55'17" E18°09'37", 114m, 20-Sep-2014, *G.* sp. *prope Minuartia*, sweeping, leg. Krátky,J., det. Krátky,J./vid. Stüben,P.
Sibinia pyrrhodactyla<br>(Marsham, 1802)	MK892170 [new]<br>2461-PSP<br>658nt	ZFMK-TIS-23433<br>ZFMK-DNA-0171661812	Germany, Saxony-Anhalt (ST), Jübar, Lkr. Salzwedel, Ohreaue, N52°40'13" E10°52'06", 71m, 25-Mai-2013, *Spergula arvensis*, dipnet, leg. Sprick,P., det. Sprick,P.
Sibinia sodalis<br>Germar, 1824	MK890885 [new]<br>529-RST<br>658nt	ZFMK-TIS-20314<br>ZFMK-DNA-0100448439	Czech Republic, Moravia mer., Podyji NP, Havraniky - heathland , N48°48'46.24" E16°0'1.49", 320m, 12-Aug-2011, *Armeria vulgaris*, sweeping, leg. Stejskal,R., det. Stejskal,R.
Sibinia sodalis<br>Germar, 1824	MK892124 [new]<br>2402-JKR<br>658nt	ZFMK-TIS-25979<br>ZFMK-DNA-0171600635	Czech Republic, Moravia, Lanzhot, Pohansko, N48°41'27" E16°55'58", 153m, 14-Aug-2014, *Armeria vulgaris*, sweeping, leg. Krátky,J., det. Krátky,J.
Sibinia sodalis<br>Germar, 1824	KC783910 [new]<br>383-JKR<br>658nt	ZFMK-TIS-20169<br>ZFMK-DNA-0100438701	Poland, Wielkopolskie, Rogalin env., N52°14'25.971" E16°54'20.152", 58m, 09-Jul-2011, *Armeria vulgaris*, sweeping, leg. Krátky,J., det. Krátky,J.
Sibinia sodalis<br>Germar, 1824	MK891250 [new]<br>1181-JKR<br>658nt	ZFMK-TIS-3517<br>ZFMK-DNA-0100426098	Spain, Andalucia, Sierra Nevada, Canadillas, N37°07'27" W03°24'39", 1922m, 10-Jun-2012, *Plumbaginaceae G.*sp., sweeping, leg. Krátky,J., det. Krátky,J.
Sibinia unicolor<br>(Fahraeus, 1843)	KC783896 [new]<br>331-JKR<br>627nt	ZFMK-TIS-20117<br>ZFMK-DNA-0100438379	Slovakia, Komarno, Marcelova env., PR Masan, N47°46'4.755" E18°19'13.531", 121m, 18-Mai-2011, *Gypsophila paniculata*, sweeping, leg. Krátky,J., det. Krátky,J.
Sibinia unicolor<br>(Fahraeus, 1843)	MK891983 [new]<br>2254-JKR<br>658nt	ZFMK-TIS-23992<br>ZFMK-DNA-0169170534	Slovakia, Komarno, Moca, N47°46'55" E18°25'11", 109m, 16-Jun-2014, *Gypsophila paniculata*, leg. Benedikt,S., det. Benedikt,S.
Sibinia unicolor<br>(Fahraeus, 1843)	MK892223 [new]<br>2520-PSP<br>658nt	ZFMK-TIS-23492<br>ZFMK-DNA-0171661762	Slovakia, Nitra, Imel, N47°55'10" E18°09'37", 113m, 18-Mai-2014, *Gypsophila paniculata*, leg. Sprick,P., det. Sprick,P.
Sibinia variata<br>Gyllenhal, 1835	MK892133 [new]<br>2412-JKR<br>658nt	ZFMK-TIS-25989<br>ZFMK-DNA-0171600625	Czech Republic, Bohemia, Belec nad Orlici, N50°11'05" E15°57'47", 273m, 24-Aug-2014, *Spergularia rubra*, collecting by hand, leg. Krátky,J., det. Krátky,J.
Sibinia viscariae<br>(Linnaeus, 1760)	MK891064 [new]<br>965-CBR<br>658nt	ZFMK-TIS-3878<br>ZFMK-DNA-0100449643	Luxembourg, 10 km NW of Esch/Alzette, 1 km NW of Niedercorn, N49°32'31" E05°52'39", 370m, 28-Jun-2012, sweeping, leg. Braunert,C., det. Braunert,C.
Sibinia viscariae<br>(Linnaeus, 1760)	MK891968 [new]<br>2238-JKR<br>658nt	ZFMK-TIS-23976<br>ZFMK-DNA-0169170549	Slovakia, Galanta, Vinohrady nad Vahom, N48°21'15" E17°45'24.5", 139m, 14-Jun-2014, *Melandryum album*, leg. Benedikt,S., det. Benedikt,S.

**Name**<br>**Authority**<br>**Additional Information**	**GenBank Acc No (Ref.)**<br>**Specimen ID**<br>**Sequence Length**	**ZFMK Tissue ID**<br>**ZFMK DNA Sample ID**<br>**(SDEI DNA Sample ID)**	**Locality, GPS, Collection Date, Plant, Collector, Identifier**
Sibinia viscariae<br>(Linnaeus, 1760)	MK891259 [new]<br>1194-JKR<br>658nt	ZFMK-TIS-3530<br>ZFMK-DNA-0100426294	Spain, Andalucia, Sierra de la Sagra, Cortijos Nuevos de la Sierra, N37°58'27" W02°32'37", 1341m, 15-Jun-2012, *Silene*, beating, leg. Krátky,J., det. Krátky,J.
Sibinia vittata<br>Germar, 1824	MK891894 [new]<br>2159-JKR<br>658nt	ZFMK-TIS-23799<br>ZFMK-DNA-0169169573	Slovakia, Nove Zamky, Salka, PR Sovie vinohrady, N47°53'15" E18°43'04", 185m, 20-Mai-2014, *Dianthus carthusianorum*, sweeping, leg. Krátky,J., det. Krátky,J.
Silvacalles carlinavorus<br>Stüben & Schütte, 2014<br>**Paratype (DNAtype)**	KF724493 [16]<br>1353-PST_4169<br>658nt	ZFMK-TIS-4169<br>ZFMK-DNA-0100426126	Spain, Canary Islands, La Gomera, N of Epina, Teselinde, Santa Clara, N28°11'46" W17°17'17", 748m, 09-Dez-2012, *Carlina salicifolia*, beating, leg. Stüben,P. & Schütte,A., det. Stüben,P.
Silvacalles carlinavorus<br>Stüben & Schütte, 2014<br>**Paratype (DNAtype)**	KF724496 [16]<br>1257-PST_3593<br>658nt	ZFMK-TIS-3593<br>ZFMK-DNA-0100426030	Spain, Canary Islands, La Gomera, N of Epina, Teselinde, Santa Clara, N28°11'46" W17°17'17", 748m, 09-Dez-2012, *Carlina salicifolia*, beating, leg. Stüben,P. & Schütte,A., det. Stüben,P.
Silvacalles carlinavorus<br>Stüben & Schütte, 2014	MG229685 [40]<br>773-PST<br>658nt	ZFMK-TIS-3110<br>ZFMK-DNA-0155630435	Spain, Canary Islands, La Gomera, N of Epina, Teselinde, Santa Clara, N28°11'47" W17°17'16", 748m, 10-Mrz-2012, *Laurisilva*, sieving, leg. Stüben,P., det. Stüben,P.
Silvacalles carlinavorus<br>Stüben & Schütte, 2014	MG229736 [40]<br>1735-PST<br>658nt	ZFMK-TIS-24453<br>ZFMK-DNA-0155622455	Spain, Canary Islands, La Palma, E of Barlovento, above El Tablado, N28°49'33" W17°52'48", 615m, 02-Jan-2014, *Tolpis*, beating, leg. Stüben,P., det. Stüben,P.
Silvacalles carlinavorus<br>Stüben & Schütte, 2014	MG229737 [40]<br>1737-PST<br>658nt	ZFMK-TIS-24455<br>ZFMK-DNA-0155622453	Spain, Canary Islands, La Palma, E of Barlovento, above El Tablado, N28°49'33" W17°52'48", 615m, 02-Jan-2014, beating, leg. Stüben,P., det. Stüben,P.
Silvacalles carlinavorus<br>Stüben & Schütte, 2014	MG229738 [40]<br>1741-PST<br>658nt	ZFMK-TIS-24459<br>ZFMK-DNA-0155622449	Spain, Canary Islands, La Palma, E of Barlovento, above El Tablado, N28°49'36" W17°52'50", 590m, 11-Jan-2014, *Tolpis*, beating, leg. Stüben,P., det. Stüben,P.
Silvacalles carlinavorus<br>Stüben & Schütte, 2014	KJ867581 [40]<br>753-PST<br>658nt	ZFMK-TIS-3090<br>ZFMK-DNA-0100448131	Spain, Canary Islands, Tenerife, Anaga Mts., Chinobre, N28°33'23" W16°10'48", 798m, 13-Feb-2012, *Geranium canariense*, beating, leg. Stüben,P. & Schütte,A., det. Stüben,P.
Silvacalles cedroensis<br>(Kulbe, 2000)	FJ716574 [01]<br>E-716-ced<br>658nt	ZFMK-TIS-cE716<br>ZFMK-DNA-0100404243	Spain, Canary Islands, La Gomera, S of Hermigua, El Cedro, Las Mimbreras, N28°07'27" W17°13'26", 901m, 06-Okt-2008, *Laurisilva*, leg. Astrin,J. & Stüben,P., det. Stüben,P.
Silvacalles cedroensis<br>(Kulbe, 2000)	GU988019 [01]<br>E-721-ced<br>658nt	ZFMK-TIS-cE721<br>ZFMK-DNA-0100404839	Spain, Canary Islands, La Gomera, S of Vallehermoso, La Meseta, "La Piedra Encantada", N28°09'15" W17°17'36", 819m, 07-Okt-2008, *Ocotea foetens*, leg. Astrin,J. & Stüben,P., det. Stüben,P.
Silvacalles cedroensis<br>(Kulbe, 2000)	KF724489 [16]<br>ES1009<br>658nt	ZFMK-TIS-cES1009<br>ZFMK-DNA-0112704660	Spain, Canary Islands, La Gomera, El Cedro, Mirador de El Bailadero, 28°7'27"N 17°12'29"W, 1015m, 09-Dez-2009, *Laurisilva*, leg. Stüben,P., det. Stüben,P.
Silvacalles cedroensis<br>(Kulbe, 2000)	KF724490 [16]<br>ES1032<br>658nt	ZFMK-TIS-cES1032<br>ZFMK-DNA-0112704637	Spain, Canary Islands, La Gomera, S of Hermigua, El Cedro, Meriga, 28°9'17"N 17°14'6"W, 829m, 29-Jan-2010, *Ficus carica*, gardens connected to Laurisilva, leg. Stüben,P., det. Stüben,P.
Silvacalles cedroensis<br>(Kulbe, 2000)	KF724495 [16]<br>ES1004<br>658nt	ZFMK-TIS-cES1004<br>ZFMK-DNA-0112704665	Spain, Canary Islands, La Gomera, S of Hermigua, El Cedro, Meriga, 28°09'15"N 17°14'10"W, 824m, 04-Dez-2009, *Castanea sativa*, gardens, leg. Stüben,P., det. Stüben,P.
Silvacalles hakani tagasaste<br>(Stüben, 2000)	FJ716540 [01]<br>C-0106-tag<br>658nt	ZFMK-TIS-cC0106<br>ZFMK-DNA-0100400200	Spain, Canary Islands, La Palma, 11 km NE of El Paso, Mt. Tagoja, N28°43'18" W17°47'07", 1047m, 01-Jul-2006, *Laurisilva, Chamaecytisus proliferus*, leg. Stüben,P., det. Stüben,P.

**Name**<br>**Authority**<br>**Additional Information**	**GenBank Acc No (Ref.)**<br>**Specimen ID**<br>**Sequence Length**	**ZFMK Tissue ID**<br>**ZFMK DNA Sample ID**<br>**(SDEI DNA Sample ID)**	**Locality, GPS, Collection Date, Plant, Collector, Identifier**
Silvacalles hakani tagasaste<br>(Stüben, 2000)	MG229710 [40]<br>1566-JKR<br>658nt	ZFMK-TIS-3614<br>ZFMK-DNA-0155635660	Spain, Canary Islands, La Palma, Montana Tagoja, N28°43'16" W17°47'07", 1052m, 30-Jan-2013, *Chamaecytisus*, beating, leg. Krátky,J., det. Krátky,J.
Silvacalles instabilis<br>(Wollaston, 1864)	JN701883 [16]<br>15-PST<br>628nt	ZFMK-TIS-2D100447025<br>ZFMK-DNA-0100438097	Spain, Canary Islands, Gran Canaria, Moya, Barranco de los Tilos, N28°05'18" W15°35'36", 446m, 07-Dez-2010, *Laurisilva*, beating, leg. Stüben,P., det. Stüben,P.
Silvacalles instabilis<br>(Wollaston, 1864)	EU286474 [01]<br>C-0136-ins<br>658nt	ZFMK-TIS-cC0136<br>ZFMK-DNA-0100400085	Spain, Canary Islands, Tenerife, Teno Mt., 3.5 km SE of Erjos near Ruigomez, N28°20'00" W16°47'10", 500m, 07-Jan-2005, *Laurus*, leg. Stüben,P., det. Stüben,P.
Silvacalles instabilis<br>(Wollaston, 1864)	GU987994 [01]<br>E-675-ins<br>658nt	ZFMK-TIS-cE675<br>ZFMK-DNA-0100404889	Spain, Canary Islands, Tenerife, NE of La Laguna, Anaga Mts. near Moquinal, N28°31'55" W16°17'24", 840m, 29-Sep-2008, *Laurisilva*, leg. Astrin,J. & Stüben,P., det. Stüben,P.
Silvacalles instabilis<br>(Wollaston, 1864)	GU987999 [01]<br>E-681-ins<br>658nt	ZFMK-TIS-cE681<br>ZFMK-DNA-0100404272	Spain, Canary Islands, Tenerife, NE of La Laguna, Anaga Mts. near Chinobre, N28°33'21" W16°10'46", 808m, 30-Sep-2008, *Laurus, Ixanthus viscosus*, leg. Astrin,J. & Stüben,P., det. Stüben,P.
Silvacalles instabilis<br>(Wollaston, 1864)	GU988000 [01]<br>E-684-ins<br>652nt	ZFMK-TIS-cE684<br>ZFMK-DNA-0100404871	Spain, Canary Islands, Tenerife, NE of La Laguna, Anaga Mts. near Chinobre, N28°33'21" W16°10'46", 808m, 30-Sep-2008, *Laurus, Ixanthus viscosus*, leg. Astrin,J. & Stüben,P., det. Stüben,P.
Silvacalles instabilis<br>(Wollaston, 1864)	GU988003 [01]<br>E-692-ins<br>657nt	ZFMK-TIS-cE692<br>ZFMK-DNA-0100404858	Spain, Canary Islands, Tenerife, NE of La Laguna, Anaga Mts., Las Mercedes, N28°31'49" W16°17'12", 905m, 01-Okt-2008, *Laurisilva*, leg. Floren,A., det. Floren,A.
Silvacalles instabilis<br>(Wollaston, 1864)	GU988086 [01]<br>E-814-ins<br>658nt	ZFMK-TIS-cE814<br>ZFMK-DNA-0100405571	Spain, Canary Islands, Tenerife, SW of Los Silos, Teno Mts., Monte del Agua, Chupadero, N28°19'23" W16°49'12", 940m, 02-Okt-2008, *Persea indica*, leg. Astrin,J.,Stüben,P.,Behne,L.,Floren,L., det. Stüben,P.
Silvacalles instabilis<br>(Wollaston, 1864)	KC783823 [40]<br>686-PST<br>658nt	ZFMK-TIS-3023<br>ZFMK-DNA-0100417615	Spain, Canary Islands, Tenerife, Anaga Mts., Chinobre, N28°38'23" W16°10'48", 798m, 13-Feb-2012, *Woodwardia radicans, Ixanthus, Geranium canariense, Rubus*, Laurisilva (at night), leg. Stüben,P. & Schütte,A., det. Stüben,P.
Silvacalles instabilis<br>(Wollaston, 1864)	KF724492 [16]<br>1242-PST<br>658nt	ZFMK-TIS-3578<br>ZFMK-DNA-0100426042	Spain, Canary Islands, Tenerife, La Tierra del Trigo, N28°21'42" W16°47'30", 630m, 25-Jan-2012, *Pinus*, beating, leg. Stüben,P., det. Stüben,P.
Silvacalles instabilis<br>(Wollaston, 1864)	KJ867578 [40]<br>702-PST<br>658nt	ZFMK-TIS-3039<br>ZFMK-DNA-0100448176	Spain, Canary Islands, Tenerife, Teno Mts. near Erjos, N28°19'31" W16°48'32", 1017m, 04-Jan-2012, *Laurus*, beating, leg. Stüben,P., det. Stüben,P.
Silvacalles instabilis<br>(Wollaston, 1864)	KJ867580 [40]<br>730-PST<br>658nt	ZFMK-TIS-3067<br>ZFMK-DNA-0100448156	Spain, Canary Islands, Tenerife, La Tierra del Trigo, N28°21'42" W16°47'30", 630m, 25-Jan-2012, *Pinus*, beating, leg. Stüben,P., det. Stüben,P.
Silvacalles lepidus<br>(Kulbe, 2000)	EU286473 [01]<br>C-0135-lep<br>658nt	ZFMK-TIS-cC0135<br>ZFMK-DNA-0100400187	Spain, Canary Islands, La Palma, 7 km SE of El Paso, San Isidro, N28°38'22" W17°47'54", 624m, 02-Jul-2006, *Laurus azorica*, leg. Stüben,P., det. Stüben,P.
Silvacalles lepidus<br>(Kulbe, 2000)	GU987863 [01]<br>E-0269-lep<br>658nt	ZFMK-TIS-cE0269<br>ZFMK-DNA-0100401046	Spain, Canary Islands, La Palma, 11 km NE of El Paso, Mt. Tagoja, N28°43'18" W17°47'07", 1047m, 01-Jul-2006, *Laurisilva*, leg. Stüben,P., det. Stüben,P.
Silvacalles lepidus<br>(Kulbe, 2000)	GU987864 [01]<br>E-0271-lep<br>653nt	ZFMK-TIS-cE0271<br>ZFMK-DNA-0100400915	Spain, Canary Islands, La Palma, 1.5 km W of La Galga, El Corcho, N28°45'03" W17°45'59", 598m, 05-Jul-2006, *Laurisilva, Juglans*, leg. Stüben,P., det. Stüben,P.

**Name**<br>**Authority**<br>**Additional Information**	**GenBank Acc No (Ref.)**<br>**Specimen ID**<br>**Sequence Length**	**ZFMK Tissue ID**<br>**ZFMK DNA Sample ID**<br>**(SDEI DNA Sample ID)**	**Locality, GPS, Collection Date, Plant, Collector, Identifier**
Silvacalles lepidus<br>(Kulbe, 2000)	MG229771 [40]<br>1983-JKR<br>658nt	ZFMK-TIS-23623<br>ZFMK-DNA-0169170518	Spain, Canary Islands, La Palma, Pared Vieja, N28°37'21" W17°49'21", 1163m, 07-Feb-2014, *Laurisilva*, beating, leg. Krátky,J., det. Krátky,J.
Silvacalles lepidus<br>(Kulbe, 2000)	MG229831 [new]<br>E-0270-lep<br>658nt	ZFMK-TIS-24650<br>ZFMK-DNA-0155630414	Spain, Canary Islands, La Palma, 2.5 km W of La Galga, above Cubo de la Galga, N28°45'18" W17°46'37", 857m, 03-Jul-2006, *Laurisilva, walnut*, leg. Stüben,P., det. Stüben,P.
Silvacalles lunulatus<br>(Wollaston, 1854)	FJ716554 [01]<br>P-513-lun<br>658nt	ZFMK-TIS-cP513<br>ZFMK-DNA-0100400608	Portugal, Madeira, 7 km S of Santana, Fajã da Nogueira, N32°44'34" W16°53'40", 514m, 16-Mrz-2008, *Laurus azorica*, leg. Stüben,P., det. Stüben,P.
Silvacalles lunulatus<br>(Wollaston, 1854)	GU987904 [01]<br>P-525-lun<br>658nt	ZFMK-TIS-cP525<br>ZFMK-DNA-0100400634	Portugal, Madeira, 3 km S of Seixal, Ribeira do Seixal, N32°47'42" W17°06'50", 516m, 21-Mrz-2008, *Laurus azorica*, leg. Astrin,J. & Stüben,P., det. Astrin,J. and Stüben,P.
Silvacalles lunulatus<br>(Wollaston, 1854)	GU987906 [01]<br>P-527-lun<br>658nt	ZFMK-TIS-cP527<br>ZFMK-DNA-0100400636	Portugal, Madeira, 4.5 km S of São Vicent Boca da Encumeada, "Folhadal", N32°45'08" W17°01'40", 1004m, 22-Mrz-2008, *Laurisilva, Euphorbia mellifera*, leg. Astrin,J. & Stüben,P., det. Astrin,J. and Stüben,P.
Silvacalles lunulatus<br>(Wollaston, 1854)	GU987907 [01]<br>P-533-lun<br>658nt	ZFMK-TIS-cP533<br>ZFMK-DNA-0100400288	Portugal, Madeira, 3 km NE of Prazeres, "Achada Grande", N32°47'05" W17°11'06", 1189m, 23-Mrz-2008, *Laurisilva*, leg. Astrin,J. & Stüben,P., det. Astrin,J. and Stüben,P.
Silvacalles lunulatus<br>(Wollaston, 1854)	MG229752 [40]<br>1853-PST<br>658nt	ZFMK-TIS-26056<br>ZFMK-DNA-0171606113	Portugal, Madeira, near Boca da Encumeada, Folhadal, N32°45'10" W17°01'59", 1103m, 01-Jul-2014, *Laurisilva*, beating, leg. Stüben,P., det. Stüben,P.
Silvacalles lunulatus<br>(Wollaston, 1854)	MG229754 [40]<br>1878-PST<br>658nt	ZFMK-TIS-26081<br>ZFMK-DNA-0171606088	Portugal, Madeira, SW of Sao Roque de Faial, road to Faja da Nouqueira, N32°44'26" W16°54'04", 582m, 10-Jul-2014, *Laurisilva*, beating, leg. Stüben,P., det. Stüben,P.
Silvacalles mundus<br>(Wollaston, 1864)	EU286472 [01]<br>C-0133-mun<br>658nt	ZFMK-TIS-cC0133<br>ZFMK-TIS-cC0133	Spain, Canary Islands, La Palma, 2.5 km W of La Galga, above Cubo de la Galga, N28°45'18" W17°46'37", 857m, 03-Jul-2006, *Laurisilva, Juglans regia*, leg. Stüben,P., det. Stüben,P.
Silvacalles mundus<br>(Wollaston, 1864)	GU987797 [01]<br>C-0134-mun<br>658nt	ZFMK-TIS-cC0134<br>ZFMK-DNA-0100400188	Spain, Canary Islands, La Palma, 7 km SE of El Paso, San Isidro, N28°38'22" W17°47'54", 624m, 02-Jul-2006, *Laurus azorica*, leg. Stüben,P., det. Stüben,P.
Silvacalles mundus<br>(Wollaston, 1864)	GU987862 [01]<br>E-0268-mun<br>658nt	ZFMK-TIS-cE0268<br>ZFMK-DNA-0100401045	Spain, Canary Islands, La Palma, 3 km SE of Garafia, Montana de las Varas, N28°49'11" W17°54'48", 919m, 09-Jul-2006, *Laurisilva*, leg. Stüben,P., det. Stüben,P.
Silvacalles mundus<br>(Wollaston, 1864)	GU987865 [01]<br>E-0272-mun<br>658nt	ZFMK-TIS-cE0272<br>ZFMK-DNA-0100400914	Spain, Canary Islands, La Palma, 1.5 km W of La Galga, El Corcho, N28°45'03" W17°45'59", 598m, 05-Jul-2006, *Laurisilva, Juglans*, leg. Stüben,P., det. Stüben,P.
Silvacalles mundus<br>(Wollaston, 1864)	MG229706 [40]<br>1556-JKR<br>658nt	ZFMK-TIS-3604<br>ZFMK-DNA-0155635673	Spain, Canary Islands, La Palma, Cubo de Galga, N28°45'42" W17°46'34", 467m, 28-Jan-2013, *Laurus*, beating, leg. Krátky,J., det. Stüben,P.
Silvacalles mundus<br>(Wollaston, 1864)	MG229707 [40]<br>1557-JKR<br>658nt	ZFMK-TIS-3605<br>ZFMK-DNA-0155635672	Spain, Canary Islands, La Palma, Cubo de Galga, N28°45'42" W17°46'34", 467m, 28-Jan-2013, *Rubus*, beating, leg. Krátky,J., det. Krátky,J.
Silvacalles mundus<br>(Wollaston, 1864)	MG229708 [40]<br>1558-JKR<br>658nt	ZFMK-TIS-3606<br>ZFMK-DNA-0155635671	Spain, Canary Islands, La Palma, Cubo de Galga, N28°45'42" W17°46'34", 467m, 28-Jan-2013, *Rubus*, beating, leg. Krátky,J., det. Krátky,J.

**Name**<br>**Authority**<br>**Additional Information**	**GenBank Acc No (Ref.)**<br>**Specimen ID**<br>**Sequence Length**	**ZFMK Tissue ID**<br>**ZFMK DNA Sample ID**<br>**(SDEI DNA Sample ID)**	**Locality, GPS, Collection Date, Plant, Collector, Identifier**
Silvacalles mundus<br>(Wollaston, 1864)	MG229722 [40]<br>1685-PST<br>658nt	ZFMK-TIS-24403<br>ZFMK-DNA-0155622514	Spain, Canary Islands, La Palma, near San Isidro, N28°38'02" W17°47'52", 648m, 18-Dez-2013, *Juglans regia*, beating, leg. Stüben,P., det. Stüben,P.
Silvacalles mundus<br>(Wollaston, 1864)	MG229729 [40]<br>1712-PST<br>658nt	ZFMK-TIS-24430<br>ZFMK-DNA-0155622478	Spain, Canary Islands, La Palma, NW of Puntallana, Bco. de la Galga, N28°46'13" W17°46'37", 421m, 27-Dez-2013, *Laurisilva*, beating, leg. Stüben,P., det. Stüben,P.
Silvacalles mundus<br>(Wollaston, 1864)	MG229772 [40]<br>1986-JKR<br>658nt	ZFMK-TIS-23626<br>ZFMK-DNA-0169170504	Spain, Canary Islands, La Palma, Cubo de Galga, N28°45'42" W17°46'34", 467m, 07-Feb-2014, *Rubus*, beating, leg. Krátky,J., det. Krátky,J.
Silvacalles mundus<br>(Wollaston, 1864)	MG229773 [40]<br>1987-JKR<br>658nt	ZFMK-TIS-23627<br>ZFMK-DNA-0169170505	Spain, Canary Islands, La Palma, Cubo de Galga, N28°45'42" W17°46'34", 467m, 07-Feb-2014, *Rubus*, beating, leg. Krátky,J., det. Krátky,J.
Silvacalles mundus<br>(Wollaston, 1864)	MG229774 [40]<br>1988-JKR<br>658nt	ZFMK-TIS-23628<br>ZFMK-DNA-0169170506	Spain, Canary Islands, La Palma, Cubo de Galga, N28°45'42" W17°46'34", 467m, 07-Feb-2014, *Rubus*, beating, leg. Krátky,J., det. Krátky,J.
Silvacalles mundus<br>(Wollaston, 1864)	MG229834 [new]<br>E-0943-lep<br>658nt	ZFMK-TIS-24616<br>ZFMK-DNA-0155630417	Spain, Canary Islands, La Palma, 3 km SE of Garafia, Montana de las Varas, N28°49'11" W17°54'48", 919m, 09-Jul-2006, *Laurisilva*, leg. Stüben,P., det. Stüben,P.
Silvacalles nubilosus<br>(Wollaston, 1864)	FJ716563 [01]<br>E-682-nub<br>658nt	ZFMK-TIS-cE682<br>ZFMK-DNA-0100404873	Spain, Canary Islands, Tenerife, NE of La Laguna, Anaga Mts. near Chinobre, N28°33'21" W16°10'46", 808m, 30-Sep-2008, *Laurus, Ixanthus viscosus*, leg. Astrin,J. & Stüben,P., det. Stüben,P.
Silvacalles nubilosus<br>(Wollaston, 1864)	GU987785 [01]<br>C-0088-nub<br>658nt	ZFMK-TIS-cC0088<br>ZFMK-DNA-0100400711	Spain, Canary Islands, Tenerife, 11 km NO Laguna, Anaga Mts. near "Roque de los Pasos", N28°32'25" W16°13'30", 870m, 25-Dez-2004, *Ixanthus viscosus*, leg. Stüben,P., det. Stüben,P.
Silvacalles nubilosus<br>(Wollaston, 1864)	GU987932 [01]<br>E-558-nub<br>654nt	ZFMK-TIS-cE558<br>ZFMK-DNA-0100404976	Spain, Canary Islands, Tenerife, Teno Mt., Monte del Agua, 4 km S of Erjos, N28°19'20" W16°49'14", 700m, 07-Jan-2005, *Ixanthus viscosus*, leg. Stüben,P., det. Stüben,P.
Silvacalles pedestris<br>(Stüben, 2000)	GU987804 [01]<br>C-0108-ped<br>658nt	ZFMK-TIS-cC0108<br>ZFMK-DNA-0100400247	Spain, Canary Islands, El Hierro, 9.5 km W of La Frontera, Pista de Mencafite, N27°44'06" W18°05'08", 929m, 22-Dez-2006, *Chamaecytisus, Ficus*, leg. Stüben,P., det. Stüben,P.
Silvacalles pedestris<br>(Stüben, 2000)	FJ716576 [01]<br>E-723-ped<br>658nt	ZFMK-TIS-cE723<br>ZFMK-DNA-0100404841	Spain, Canary Islands, La Gomera, S of Vallehermoso, La Meseta, "La Piedra Encantada", N28°09'15" W17°17'36", 819m, 07-Okt-2008, *Ocotea foetens*, leg. Astrin,J. & Stüben,P., det. Stüben,P.
Silvacalles pedestris<br>(Stüben, 2000)	GU988084 [01]<br>E-810-ped<br>658nt	ZFMK-TIS-cE810<br>ZFMK-DNA-0100405573	Spain, Canary Islands, La Gomera, S of Vallehermoso, La Meseta, "La Piedra Encantada", N28°09'15" W17°17'36", 819m, 06-Okt-2008, *Ocotea foetens*, leg. Astrin,J.,Stüben,P.,Behne,L.,Floren,L., det. Stüben,P.
Silvacalles pedestris<br>(Stüben, 2000)	KF724491 [16]<br>ES1010<br>658nt	ZFMK-TIS-cES1010<br>ZFMK-DNA-0112704648	Spain, Canary Islands, La Gomera, El Cedro, Mirador de El Bailadero, 28°7'27"N 17°12'29"W, 1015m, 09-Dez-2009, *Chamaecytisus proliferus*, leg. Stüben,P., det. Stüben,P.
Silvacalles pedestris<br>(Stüben, 2000)	KF724494 [16]<br>ES1033<br>658nt	ZFMK-TIS-cES1033<br>ZFMK-DNA-0112704636	Spain, Canary Islands, La Gomera, S of Vallehermoso, Los Loros, 28°8'57"N 17°16'36"W, 609m, 11-Feb-2010, *Castanea sativa*, gardens at river, connected to Laurisilva, leg. Stüben,P., det. Stüben,P.
Silvacalles pedestris<br>(Stüben, 2000)	GU987803 [01]<br>C-0107-ped<br>658nt	ZFMK-TIS-cC0107<br>ZFMK-DNA-0100400142	Spain, Canary Islands, La Palma, 11 km NE of El Paso, Mt. Tagoja, N28°43'18" W17°47'07", 1047m, 01-Jul-2006, *Laurisilva, Chamaecytisus proliferus*, leg. Stüben,P., det. Stüben,P.

**Name**<br>**Authority**<br>**Additional Information**	**GenBank Acc No (Ref.)**<br>**Specimen ID**<br>**Sequence Length**	**ZFMK Tissue ID**<br>**ZFMK DNA Sample ID**<br>**(SDEI DNA Sample ID)**	**Locality, GPS, Collection Date, Plant, Collector, Identifier**
Silvacalles pedestris<br>(Stüben, 2000)	MG229711 [40]<br>1567-JKR<br>658nt	ZFMK-TIS-3615<br>ZFMK-DNA-0155635661	Spain, Canary Islands, La Palma, Montana Tagoja, N28°43'16" W17°47'07", 1052m, 30-Jan-2013, *Chamaecytisus*, beating, leg. Krátky,J., det. Krátky,J.
Silvacalles pedestris<br>(Stüben, 2000)	GU988013 [01]<br>E-711-ped<br>658nt	ZFMK-TIS-cE711<br>ZFMK-DNA-0100404844	Spain, Canary Islands, Tenerife, S of Orotava, Orotava valley, Mirador de la Rosa, N28°20'27" W16°31'31", 1503m, 04-Okt-2008, *Chamaecytisus proliferus*, leg. Astrin,J. & Stüben,P., det. Stüben,P.
Silvacalles pedestris<br>(Stüben, 2000)	KC783826 [16]<br>708-PST<br>658nt	ZFMK-TIS-3045<br>ZFMK-DNA-0100448182	Spain, Canary Islands, Tenerife, La Montaneta, N28°20'32" W16°45'32", 913m, 05-Jan-2012, *Brassicaceae (ger. "Kohl")*, beating, leg. Stüben,P., det. Stüben,P.
Silvacalles tolpivorus<br>(Germann & Stüben, 2006)	FJ716578 [01]<br>E-727-tol<br>658nt	ZFMK-TIS-cE727<br>ZFMK-DNA-0100404828	Spain, Canary Islands, La Gomera, SE of Hermigua near Casas del Palmar, N28°09'29" W17°09'37", 627m, 08-Okt-2008, *Tolpis proustii*, thermophilic brushwood, leg. Astrin,J. & Stüben,P., det. Stüben,P.
Silvacalles tolpivorus<br>(Germann & Stüben, 2006)	GU987793 [01]<br>C-0117-tol<br>658nt	ZFMK-TIS-cC0117<br>ZFMK-DNA-0100400687	Spain, Canary Islands, La Gomera, 2.5 km E of Hermigua, N28°09'30" W17°09'46", 650m, 31-Dez-2004, *Tolpis* cf. *proustii*, thermophilic brushwood, leg. Stüben,P., det. Stüben,P.
Silvacalles tolpivorus<br>(Germann & Stüben, 2006)	KF724497 [16]<br>ES1022<br>658nt	ZFMK-TIS-cES1022<br>ZFMK-DNA-0112704647	Spain, Canary Islands, La Gomera, Roque de Agando, 28°6'22"N 17°12'42"W, 1061m, 02-Jan-2010, *Tolpis prousti*, leg. Stüben,P., det. Stüben,P.
Silvacalles tolpivorus<br>(Germann & Stüben, 2006)	KF724498 [16]<br>ES1023<br>658nt	ZFMK-TIS-cES1023<br>ZFMK-DNA-0112704646	Spain, Canary Islands, La Gomera, Roque de Agando, 28°6'22"N 17°12'42"W, 1061m, 02-Jan-2010, *Pimpinella junoniae*, leg. Stüben,P., det. Stüben,P.
Silvacalles tolpivorus<br>(Germann & Stüben, 2006)	KF724499 [16]<br>ES1013<br>658nt	ZFMK-TIS-cES1013<br>ZFMK-DNA-0112704651	Spain, Canary Islands, La Gomera, Arure, N28°07'57" W17°19'11", 806m, 13-Dez-2009, *Tolpis proustii*, meadow, leg. Stüben,P., det. Stüben,P.
Simmeiropsis virginum<br>(Pierotti & Bellò, 2006)	KC784242 [new]<br>IT-0009w<br>655nt	ZFMK-TIS-2D100446541<br>ZFMK-DNA-0100437855	Italy, Sardinia Isl. East, N of Dorgali near G.ta di Ispinigoli, N40°19'12" E09°36'00", 241m, 26-Sep-2010, *Quercus ilex, Pistacia*, cliff, limestone, leg. Stüben,P., det. Stüben,P.
Simo hirticornis<br>(Herbst, 1795)	KC784150 [new]<br>620-PSP<br>658nt	ZFMK-TIS-20403<br>ZFMK-DNA-0100448647	Germany, Lower Saxony (NI), Bad Sachsa, Sachsenstein, N51°35'14" E10°34'56", 291m, 23-Jul-2011, *Vaccinium myrtillus*, beating, leg. Sprick,P., det. Sprick,P.
Simo hirticornis<br>(Herbst, 1795)	MK891059 [new]<br>960-CBR<br>658nt	ZFMK-TIS-3873<br>ZFMK-DNA-0100449651	Switzerland, 4 km SW of Zernez, Prazet, N46°40'12" E10°02'51", 1650m, 22-Jul-2011, beating, leg. Braunert,C., det. Braunert,C.
Simopsis astragali<br>(Stierlin, 1861)	KC784272 [new]<br>IT-0046w<br>652nt	ZFMK-DNA-0100417643	Italy, Sardinia Isl. East, E of Seui, Mt. Arqueri, N39°49'04" E09°22'02", 925m, 29-Sep-2010, *Quercus ilex*, cliff, leg. Stüben,P., det. Stüben,P.
Simopsis cf. *anachoreta*	KC784261 [new]<br>IT-0031w<br>654nt	ZFMK-TIS-2D100446128<br>ZFMK-DNA-0100437845	Italy, Sardinia Isl. East, E of Oliena near Su Gologone, N40°17'15" E09°30'29", 161m, 05-Okt-2010, *Quercus*, above creek, leg. Stüben,P., det. Stüben,P.
Simo variegatus<br>(Boheman, 1842)	MK892167 [new]<br>2458-PSP<br>658nt	ZFMK-TIS-23430<br>ZFMK-DNA-0171661815	Germany, Lower Saxony (NI), Hameln-Rohrsen, Dütberg, N52°06'21" E09°24'49", 104m, 10-Mai-2013, *Prunus spinosa*, beating, leg. Sprick,P., det. Sprick,P.
Simo variegatus<br>(Boheman, 1842)	MK891340 [new]<br>1374-PST<br>658nt	ZFMK-TIS-4670<br>ZFMK-DNA-0155628556	Italy, Abruzzo, E of Castel del Monte, Madonnina Capo di Serra, N42°21'38" E13°45'29", 1243m, 17-Aug-2013, sieving, leg. Stüben,P., det. Stüben,P.

**Name**<br>**Authority**<br>**Additional Information**	**GenBank Acc No (Ref.)**<br>**Specimen ID**<br>**Sequence Length**	**ZFMK Tissue ID**<br>**ZFMK DNA Sample ID**<br>**(SDEI DNA Sample ID)**	**Locality, GPS, Collection Date, Plant, Collector, Identifier**
Sirocalodes cf. *nigroterminatus*<br>(Wollaston, 1854)	KC783740 [13]<br>18-PST<br>656nt	ZFMK-TIS-2D100447028<br>ZFMK-DNA-0100438100	Spain, Canary Islands, Gran Canaria, Moya, Barranco de los Tilos, N28°04'33" W15°36'05", 643m, 07-Dez-2010, *Fumaria*, beating, leg. Stüben,P., det. Stüben,P.
Sirocalodes depressicollis<br>(Gyllenhal, 1813)	MK891923 [new]<br>2191-JKR<br>658nt	ZFMK-TIS-23929<br>ZFMK-DNA-0169170596	Czech Republic, Moravia, Znojmo, N48°51'32" E16°04'28.5", 255m, 12-Apr-2014, *Fumaria*, beating, leg. Stejskal,R., det. Stejskal,R.
Sirocalodes depressicollis<br>(Gyllenhal, 1813)	MK892215 [new]<br>2511-PSP<br>658nt	ZFMK-TIS-23483<br>ZFMK-DNA-0171661753	Germany, Lower Saxony (NI), Braunschweig, N52°16'36" E10°33'51", 81m, 09-Mai-2014, *Fumaria officinalis*, leg. Sprick,P., det. Sprick,P.
Sirocalodes depressicollis<br>(Gyllenhal, 1813)	KC783973 [new]<br>343-JKR<br>658nt	ZFMK-TIS-20129<br>ZFMK-DNA-0100438958	Slovakia, Komarno, Cenkov, N47°46'8.052" E18°31'18.977", 105m, 21-Mai-2011, *Fumaria* sp., sweeping, leg. Krátky,J., det. Krátky,J.
Sirocalodes mixtus<br>(Mulsant & Rey, 1859)	MK892208 [new]<br>2504-PSP<br>658nt	ZFMK-TIS-23476<br>ZFMK-DNA-0171661769	Germany, Lower Saxony (NI), Helstorfer Moor, N52°32'50" E09°35'54", 46m, 06-Mai-2014, *Ceratocapnos claviculata*, peatland area, dipnet, leg. Sprick,P., det. Sprick,P.
Sirocalodes mixtus<br>(Mulsant & Rey, 1859)	MK891341 [new]<br>1376-PST<br>658nt	ZFMK-TIS-4672<br>ZFMK-DNA-0155628558	Italy, Abruzzo, E of Castel del Monte, Madonnina Capo di Serra, N42°21'38" E13°45'29", 1243m, 17-Aug-2013, sieving, leg. Stüben,P., det. Stüben,P.
Sirocalodes nigroterminatus<br>(Wollaston, 1854)	MK892367 [40]<br>2797-PST<br>658nt	ZFMK-TIS-26151<br>ZFMK-DNA-0169166945	Portugal, Madeira, Santa Madalena, Salao, coast, N32°51'59" W17°11'57", 313m, 04-Apr-2015, *Fumaria*, collecting by hand, leg. Stüben,P., det. Stüben,P.
Sirocalodes nigroterminatus<br>(Wollaston, 1854)	MK892378 [40]<br>2831-PST<br>658nt	ZFMK-TIS-4741<br>ZFMK-DNA-FC17941509	Portugal, Madeira, N of Santana, Quebrada, N32°48'49" W16°52'27", 124m, 25-Nov-2015, *Fumaria*, beating, leg. Stüben,P. & Schütte,A., det. Stüben,P.
Sitona ambiguus<br>Gyllenhal, 1834	KC783942 [new]<br>325-JKR<br>658nt	ZFMK-TIS-20111<br>ZFMK-DNA-0100438758	Czech Republic, Bohemia or., Hradec Kralove - Fararstvi, N50°11'22.247" E15°48'53.64", 230m, 17-Mai-2011, sweeping, leg. Krátky,J., det. Krátky,J.
Sitona brachypterus<br>Israelson, 1980	MK347536 [40]<br>2941-PST<br>658nt	(SDEI-DNA-2941-PST)	Spain, Canary Islands, Lanzarote, Haria - Magues, N29°09'07" W13°29'31", 339m, 13-Jan-2017, *Carlina salicifolia*, beating, leg. Stüben,P., det. Stüben,P.
Sitona brucki<br>Allard, 1870	KC783883 [new]<br>120-PST<br>602nt	ZFMK-TIS-2D100440310<br>ZFMK-DNA-0100438198	Morocco, S of Berkane, Beni Snassen Mts., Vallee Zegzel, N34°49'33" W02°22'37", 590m, 11-Mai-2011, beating, leg. Stüben,P., det. Stüben,P./vid. Behne,L.
Sitona callosus<br>Gyllenhal, 1834	MK891946 [new]<br>2214-JKR<br>658nt	ZFMK-TIS-23952<br>ZFMK-DNA-0169170573	Slovakia, Komarno, Virt, N47°45'39" E18°20'13.5", 123m, 20-Sep-2014, leg. Benedikt,S., det. Benedikt,S.
Sitona callosus<br>Gyllenhal, 1834	MK892157 [new]<br>2445-JKR<br>658nt	ZFMK-TIS-26022<br>ZFMK-DNA-0171600667	Slovakia, Nove Zamky, Imel, PR Liscie diery, N47°55'17" E18°09'37", 114m, 20-Sep-2014, sweeping, leg. Krátky,J., det. Krátky,J.
Sitona callosus<br>Gyllenhal, 1834	MK891256 [new]<br>1190-JKR<br>658nt	ZFMK-TIS-3526<br>ZFMK-DNA-0100426094	Spain, Andalucia, Sierra da Baza, Santa Barbara, N37°21'53" W02°51'00", 2019m, 11-Jun-2012, sieving, leg. Krátky,J., det. Krátky,J.
Sitona cylindricollis<br>Fahraeus, 1840	MK892076 [new]<br>2352-JKR<br>658nt	ZFMK-TIS-24283<br>ZFMK-DNA-0169170922	Czech Republic, Moravia, Lanzhot, Pohansko, N48°41'27" E16°55'58", 153m, 14-Aug-2014, *Galega officinalis*, sweeping, leg. Krátky,J., det. Krátky,J.

**Name**<br>**Authority**<br>**Additional Information**	**GenBank Acc No (Ref.)**<br>**Specimen ID**<br>**Sequence Length**	**ZFMK Tissue ID**<br>**ZFMK DNA Sample ID**<br>**(SDEI DNA Sample ID)**	**Locality, GPS, Collection Date, Plant, Collector, Identifier**
Sitona cylindricollis<br>Fahraeus, 1840	MK890898 [new]<br>615-PSP<br>658nt	ZFMK-TIS-20398<br>ZFMK-DNA-0100448652	Germany, Lower Saxony (NI), Brelingen, Hannover region, N52°33'53" E09°40'55", 79m, 16-Jul-2011, *Melilotus albus*, dry-warm ruderal area, beating, leg. Sprick,P., det. Sprick,P.
Sitona cylindricollis<br>Fahraeus, 1840	MK892276 [new]<br>2577-PSP<br>658nt	ZFMK-TIS-23551<br>ZFMK-DNA-0171661982	Germany, Bavaria (BY), Unterfranken, Ebern, N50°05'22" E10°46'02", 327m, 13-Jun-2014, *Melilotus officinalis*, leg. Sprick,P., det. Sprick,P.
Sitona cylindricollis<br>Fahraeus, 1840	MK891034 [new]<br>931-CBR<br>658nt	ZFMK-TIS-3844<br>ZFMK-DNA-0100449680	Luxembourg, 12 km NE of Luxembourg, 1 km NW of Wecker, N49°41'53" E06°22'53", 215m, 07-Jun-2012, *Melilotus*, beating, leg. Braunert,C., det. Braunert,C.
Sitona discoideus<br>Gyllenhal, 1834	KC784235 [new]<br>IT-0002w<br>658nt	ZFMK-TIS-2D100446552<br>ZFMK-DNA-0100433813	Italy, Sardinia Isl. East, W of Siniscola, Monte Albo, N40°33'37" E09°38'01", 778m, 26-Sep-2010, *Quercus ilex*, cliff, limestone, leg. Stüben,P., det. Stüben,P.
Sitona discoideus<br>Gyllenhal, 1834	KC784241 [40]<br>IT-0008w<br>653nt	ZFMK-TIS-2D100446542<br>ZFMK-DNA-0100433854	Italy, Sardinia Isl. East, N of Dorgali near G.ta di Ispinigoli, N40°19'12" E09°36'00", 241m, 26-Sep-2010, *Quercus ilex, Pistacia*, cliff, limestone, leg. Stüben,P., det. Stüben,P.
Sitona hispidulus<br>(Fabricius, 1777)	MK891797 [new]<br>2056-JKR<br>658nt	ZFMK-TIS-23696<br>ZFMK-DNA-0169170445	Czech Republic, Bohemia, Kosatky, N50°19'23" E14°39'58", 218m, 02-Mai-2014, sieving, leg. Krátky,J., det. Krátky,J.
Sitona hispidulus<br>(Fabricius, 1777)	MK891850 [new]<br>2112-JKR<br>658nt	ZFMK-TIS-23752<br>ZFMK-DNA-0169169622	Czech Republic, Moravia, Lanzhot, N48°42'37" E16°58'27", 156m, 10-Mai-2014, sweeping, leg. Krátky,J., det. Krátky,J.
Sitona hispidulus<br>(Fabricius, 1777)	MK891961 [new]<br>2230-JKR<br>658nt	ZFMK-TIS-23968<br>ZFMK-DNA-0169170558	Czech Republic, Bohemia, Chudenin, N49°17'54" E13°06'00", 475m, 27-Sep-2014, leg. Benedikt,S., det. Benedikt,S.
Sitona hispidulus fulvipes<br>(Fabricius, 1777)	KC784213 [new]<br>440-RGO<br>658nt	ZFMK-TIS-20226<br>ZFMK-DNA-0100449410	Poland, Dobu?ek, N50°23'48.3" E23°32'54.8", 260m, 21-Sep-2011, leg. Gosik,R., det. Gosik,R.
Sitona hispidulus<br>(Fabricius, 1777)	KC784147 [new]<br>633-PSP<br>658nt	ZFMK-TIS-20416<br>ZFMK-DNA-0100448639	Germany, Lower Saxony (NI), Königsförde, Lkr. Hameln-Pyrmont, N52°03'58" E09°16'17", 92m, 03-Okt-2011, *Trifolium repens*, house garden, gras sifting, leg. Sprick,P., det. Sprick,P.
Sitona hispidulus<br>(Fabricius, 1777)	MK891701 [new]<br>1919-FBA<br>658nt	ZFMK-TIS-23310<br>ZFMK-DNA-0169170367	Greece, East Macedonia and Thrace, Drama, Lefkoghia, N41°23'59" E23°54'53", 620m, 13-Aug-2014, leg. Bayer & Brunner, det. Bayer,F.
Sitona hispidulus<br>(Fabricius, 1777)	KC784194 [new]<br>456-RGO<br>658nt	ZFMK-TIS-20242<br>ZFMK-DNA-0100449378	Poland, Rudnik, N51°14'31.02" E22°32'22.92", 191m, 03-Okt-2011, leg. Gosik,R., det. Gosik,R.
Sitona humeralis<br>Stephens, 1831	MK891450 [new]<br>1515-PSP<br>658nt	ZFMK-TIS-3275<br>ZFMK-DNA-0155627319	Denmark, Syddanmark, Emmerlev Klev (Højer), N54°59'53" E08°39'08", 3m, 30-Aug-2012, *Medicago lupulina*, dipnet, leg. Sprick,P., det. Sprick,P.
Sitona humeralis<br>Stephens, 1831	KC784135 [new]<br>644-PSP<br>658nt	ZFMK-TIS-20427<br>ZFMK-DNA-0100448623	Germany, Lower Saxony (NI), Braunschweig, Riddagshausen, N52°16'34" E10°33'50", 80m, 21-Sep-2011, *Rhododendron catawbiense-Hybride*, beating, leg. Sprick,P., det. Sprick,P.
Sitona humeralis<br>Stephens, 1831	MK892147 [new]<br>2434-JKR<br>658nt	ZFMK-TIS-26011<br>ZFMK-DNA-0171600651	Slovakia, Nove Zamky, Kamenin, PP Cistiny, N47°51'55" E18°38'05", 111m, 21-Sep-2014, sweeping, leg. Krátky,J., det. Krátky,J.

**Name**<br>**Authority**<br>**Additional Information**	**GenBank Acc No (Ref.)**<br>**Specimen ID**<br>**Sequence Length**	**ZFMK Tissue ID**<br>**ZFMK DNA Sample ID**<br>**(SDEI DNA Sample ID)**	**Locality, GPS, Collection Date, Plant, Collector, Identifier**
Sitona humeralis<br>Stephens, 1831	MK892241 [new]<br>2539-PSP<br>658nt	ZFMK-TIS-23511<br>ZFMK-DNA-0171661733	Slovakia, Nitra, W of Gbelce, N47°46'56" E18°27'37", 148m, 18-Mai-2014, *Medicago* sp. *(eng. lucerne)*, leg. Sprick,P., det. Sprick,P.
Sitona languidus<br>Gyllenhal, 1834	MK891791 [new]<br>2050-JKR<br>658nt	ZFMK-TIS-23690<br>ZFMK-DNA-0169170451	Czech Republic, Bohemia, Kosatky, N50°19'23" E14°39'58", 218m, 02-Mai-2014, sieving, leg. Krátky,J., det. Krátky,J.
Sitona languidus<br>Gyllenhal, 1834	MK890836 [new]<br>292-PSP<br>648nt	ZFMK-TIS-2D100439090<br>ZFMK-DNA-0100439499	Germany, Hesse (HE), Wiesbaden-Schierstein, N50°03'02" E08°18'13", 274m, 31-Mai-2011, *Coronilla varia (Securigera varia)*, dipnet, leg. Sprick,P., det. Sprick,P.
Sitona lateralis<br>Gyllenhal, 1834<br>Synonym of *S. suturalis*?	KC783992 [new]<br>257-PSP<br>658nt	ZFMK-TIS-2D100439103<br>ZFMK-DNA-0100439775	Germany, Lower Saxony (NI), Hannover, Stöcken, Leine wetland, N52°24'24" E09°39'09", 45m, 14-Mai-2011, *Vicia cracca*, grassland, dipnet, leg. Sprick,P., det. Sprick,P.
Sitona lateralis<br>Gyllenhal, 1834<br>Synonym of *S. suturalis*?	MK891203 [new]<br>1121-JKR<br>658nt	ZFMK-TIS-3457<br>ZFMK-DNA-0100449587	Slovakia, Nove Zamky, Tvrdosovce, N48°06'09" E18°02'08", 110m, 10-Mai-2012, *Lotus tenuis*, sweeping, leg. Krátky,J., det. Krátky,J.
Sitona lineatus<br>(Linnaeus, 1758)	KC784053 [new]<br>581-PSP<br>658nt	ZFMK-TIS-20365<br>ZFMK-DNA-0100448395	Germany, Bavaria (BY), Buckenhof-Uttenreuth, Lkr. Erlangen-Höchstadt, N49°35'18" E11°05'11", 300m, 03-Jun-2011, *Vicia villosa*, edge of field, dipnet, leg. Sprick,P., det. Sprick,P.
Sitona lineatus<br>(Linnaeus, 1758)	KC784148 [new]<br>634-PSP<br>658nt	ZFMK-TIS-20417<br>ZFMK-DNA-0100448640	Germany, Lower Saxony (NI), Groß-Berkel, Lkr. Hameln-Pyrmont, N52°03'54" E09°17'39", 81m, 03-Sep-2011, *Trifolium pratense*, mesophilic grassland, dipnet, leg. Sprick,P., det. Sprick,P.
Sitona lineatus<br>(Linnaeus, 1758)	MK891700 [new]<br>1918-FBA<br>658nt	ZFMK-TIS-23309<br>ZFMK-DNA-0169170366	Greece, East Macedonia and Thrace, Drama, Lefkoghia, N41°23'59" E23°54'53", 620m, 13-Aug-2014, leg. Bayer & Brunner, det. Bayer,F.
Sitona lineatus<br>(Linnaeus, 1758)	MK891651 [40]<br>1829-PST<br>658nt	ZFMK-TIS-26032<br>ZFMK-DNA-0171606137	Portugal, Madeira, S of Seixal, S of Chao da Ribeira, N32°48'41" W17°06'38", 411m, 27-Jun-2014, beating, leg. Stüben,P., det. Stüben,P.
Sitona lineatus<br>(Linnaeus, 1758)	MK891712 [40]<br>1938-PST<br>658nt	ZFMK-TIS-24017<br>ZFMK-DNA-0169168515	Portugal, Madeira, Faial, coast, N32°47'37" W16°50'57", 26m, 04-Okt-2014, sieving, leg. Stüben,P., det. Stüben,P.
Sitona lineatus<br>(Linnaeus, 1758)	KC784200 [new]<br>449-RGO<br>658nt	ZFMK-TIS-20235<br>ZFMK-DNA-0100449390	Poland, Rudnik, N51°14'31.02" E22°32'22.92", 191m, 03-Okt-2011, leg. Gosik,R., det. Gosik,R.
Sitona lineatus<br>(Linnaeus, 1758)	KC784208 [new]<br>434-RGO<br>658nt	ZFMK-TIS-20220<br>ZFMK-DNA-0100449404	Poland, Zurawce, N50°34'3.6" E23°51'57.6", 248m, 17-Aug-2011, leg. Gosik,R., det. Gosik,R.
Sitona lineatus<br>(Linnaeus, 1758)	MK892047 [new]<br>2323-JKR<br>658nt	ZFMK-TIS-24254<br>ZFMK-DNA-0169170941	Slovakia, Tatry Mts., Dolina Siedmich prame?ov, N49°13'30" E20°16'24", 1404m, 05-Jul-2014, *Lathyrus pratensis*, collecting by hand, leg. Krátky,J., det. Krátky,J.
Sitona macularius<br>(Marsham, 1802)	KC783978 [new]<br>280-PSP<br>658nt	ZFMK-TIS-2D100439091<br>ZFMK-DNA-0100439512	Germany, Lower Saxony (NI), Pattensen, Hannover region, N52°14'35" E09°45'21", 77m, 17-Mai-2011, *Vicia angustifolia*, species-rich roadside, beating, leg. Sprick,P., det. Sprick,P.
Sitona macularius<br>(Marsham, 1802)	KC783735 [40]<br>11-PST<br>658nt	ZFMK-TIS-2D100447036<br>ZFMK-DNA-0100438108	Spain, Canary Islands, Gran Canaria, Santa Lucia, below Cruz las Vueltas, Mirador de las Tederas, N27°54'47" W15°31'48", 818m, 03-Dez-2010, beating, leg. Stüben,P., det. Stüben,P.

**Name**<br>**Authority**<br>**Additional Information**	**GenBank Acc No (Ref.)**<br>**Specimen ID**<br>**Sequence Length**	**ZFMK Tissue ID**<br>**ZFMK DNA Sample ID**<br>**(SDEI DNA Sample ID)**	**Locality, GPS, Collection Date, Plant, Collector, Identifier**
Sitona macularius<br>(Marsham, 1802)	KC783804 [40]<br>60-PST<br>658nt	ZFMK-TIS-2D100446988<br>ZFMK-DNA-0100417880	Spain, Canary Islands, La Gomera, Hermigua, Las Nuevitas, Barranquillos los Alamos, N28°09'31" W17°11'09", 251m, 13-Dez-2010, beating, leg. Stüben,P., det. Stüben,P.
Sitona macularius<br>Fahraeus, 1840	MK891748 [40]<br>2001-JKR<br>658nt	ZFMK-TIS-23641<br>ZFMK-DNA-0169170500	Spain, Canary Islands, La Palma, Mendo, 3 km S of Jedey, N28°33'38" W17°51'45", 1000m, 12-Feb-2014, *Aeonium spathulatum, Rumex lunaria*, beating, leg. Krátky,J., det. Krátky,J.
Sitona macularius<br>(Marsham, 1802)	MK892382 [40]<br>2840-PST<br>658nt	ZFMK-TIS-4236<br>ZFMK-DNA-FC17941494	Portugal, Madeira, Porto Santo Isl., Pico do Castelo, N33°04'44" W16°19'57", 273m, 27-Nov-2015, *Quercus ilex*, sieving, leg. Stüben,P., det. Stüben,P.
Sitona macularius<br>(Marsham, 1802)	MK891370 [new]<br>1421-JKR<br>658nt	ZFMK-TIS-4190<br>ZFMK-DNA-0155628507	Slovakia, Vysoke Tatry Mts., Dolina siedmich prame?ov, N49°13'38" E20°16'34", 1448m, 30-Jun-2013, sweeping, leg. Krátky,J., det. Krátky,J.
Sitona macularius<br>(Marsham, 1802)	MK891879 [new]<br>2143-JKR<br>658nt	ZFMK-TIS-23783<br>ZFMK-DNA-0169169590	Slovakia, Komarno, Imel, PR Liscie diery, N47°55'17" E18°09'37", 114m, 18-Mai-2014, sweeping, leg. Krátky,J., det. Krátky,J.
Sitona obsoletus obsoletus<br>Gmelin, 1790	MK891982 [new]<br>2253-JKR<br>658nt	ZFMK-TIS-23991<br>ZFMK-DNA-0169170533	Czech Republic, Bohemia, Chudenin, N49°17'54" E13°6'0.04", 475m, 27-Sep-2014, leg. Benedikt,S., det. Benedikt,S.
Sitona obsoletus obsoletus<br>Gmelin, 1790	MK890899 [new]<br>625-PSP<br>658nt	ZFMK-TIS-20408<br>ZFMK-DNA-0100448631	Germany, Lower Saxony (NI), Königsförde, Lkr.Hameln-Pyrmont, N52°03'58" E09°16'17", 92m, 07-Aug-2011, *Trifolium repens*, house garden, gras sifting, leg. Sprick,P., det. Sprick,P.
Sitona striatellus<br>Gyllenhal, 1834	MK891778 [new]<br>2037-JKR<br>658nt	ZFMK-TIS-23677<br>ZFMK-DNA-0169170459	Czech Republic, Bohemia, Kosatky, N50°19'23" E14°39'58", 218m, 02-Mai-2014, *Genista* sp., sweeping, leg. Krátky,J., det. Krátky,J.
Sitona striatellus<br>Gyllenhal, 1834	MK891833 [new]<br>2094-JKR<br>658nt	ZFMK-TIS-23734<br>ZFMK-DNA-0169169639	Czech Republic, Moravia, Bucovice, PR Sevy, N49°08'05" E16°58'19", 249m, 09-Mai-2014, *Genista* sp., sweeping, leg. Krátky,J., det. Krátky,J.
Sitona sulcifrons argutulus<br>Gyllenhal, 1834	MK891505 [new]<br>1591-JKR<br>658nt	ZFMK-TIS-3639<br>ZFMK-DNA-0155635637	Czech Republic, Moravia, Cejc - Spidlaky, N48°55'47" E16°58'03", 184m, 01-Mai-2013, sieving, leg. Krátky,J., det. Krátky,J.
Sitona sulcifrons argutulus<br>Gyllenhal, 1834	MK891525 [new]<br>1613-JKR<br>658nt	ZFMK-TIS-3661<br>ZFMK-DNA-0155635611	Czech Republic, Bohemia, Bily Kun, N49°53'04" E16°04'15", 348m, 15-Mai-2013, sweeping, leg. Krátky,J., det. Krátky,J.
Sitona sulcifrons argutulus<br>Gyllenhal, 1834	MK891816 [new]<br>2076-JKR<br>658nt	ZFMK-TIS-23716<br>ZFMK-DNA-0169169657	Czech Republic, Bohemia, Kutrin, N49°49'12.5" E16°03'27", 456m, 08-Mai-2014, sweeping, leg. Krátky,J., det. Krátky,J.
Sitona sulcifrons argutulus<br>Gyllenhal, 1834	MK891385 [new]<br>1438-JKR<br>658nt	ZFMK-TIS-4207<br>ZFMK-DNA-0155628495	Slovakia, Komarno, Martovce, Stara Nitra, N47°49'52" E18°08'13", 108m, 20-Jul-2013, sweeping, leg. Krátky,J., det. Krátky,J.
Sitona sulcifrons sulcifrons<br>(Thunberg, 1798)	MK891267 [new]<br>1206-JKR<br>658nt	ZFMK-TIS-3542<br>ZFMK-DNA-0100426075	Czech Republic, Moravia, Hruby jesenik, Velka Kotlina, N50°3'18" E17°14'14", 1209m, 23-Aug-2012, sweeping, leg. Krátky,J., det. Krátky,J.
Sitona sulcifrons sulcifrons<br>(Thunberg, 1798)	MK891453 [new]<br>1518-PSP<br>658nt	ZFMK-TIS-3278<br>ZFMK-DNA-0155633308	Denmark, Syddanmark, Emmerlev Klev (Højer), N54°59'53" E08°39'08", 3m, 30-Aug-2012, *Trifolium pratense, Trifolium repens*, leg. Sprick,P., det. Sprick,P.

**Name**<br>**Authority**<br>**Additional Information**	**GenBank Acc No (Ref.)**<br>**Specimen ID**<br>**Sequence Length**	**ZFMK Tissue ID**<br>**ZFMK DNA Sample ID**<br>**(SDEI DNA Sample ID)**	**Locality, GPS, Collection Date, Plant, Collector, Identifier**
Sitona sulcifrons sulcifrons<br>(Thunberg, 1798)	KC784126 [new]<br>652-PSP<br>658nt	ZFMK-TIS-20435<br>ZFMK-DNA-0100448610	Germany, Lower Saxony (NI), Hameln-Rohrsen, Dütberg, N52°06'31" E09°25'19", 86m, 03-Okt-2011, *Trifolium pratense*, dipnet, leg. Sprick,P., det. Sprick,P.
Sitona sulcifrons sulcifrons<br>(Thunberg, 1798)	MK892283 [new]<br>2584-PSP<br>658nt	ZFMK-TIS-23558<br>ZFMK-DNA-0171661972	Germany, Bavaria (BY), Unterfranken, Ebern, N50°05'20.5" E10°45'59", 333m, 13-Jun-2014, *Trifolium medium*, leg. Sprick,P., det. Sprick,P.
Sitona sulcifrons sulcifrons<br>(Thunberg, 1798)	KC784195 [new]<br>457-RGO<br>658nt	ZFMK-TIS-20243<br>ZFMK-DNA-0100449379	Poland, Rudnik, N51°14'31.02" E22°32'22.92", 191m, 03-Okt-2011, leg. Gosik,R., det. Gosik,R.
Sitona suturalis<br>Stephens, 1831	KC783944 [new]<br>320-JKR<br>658nt	ZFMK-TIS-20106<br>ZFMK-DNA-0100438763	Czech Republic, Bohemia or., Dvakacovice, N49°58'35.447" E15°54'2.243", 245m, 14-Mai-2011, sweeping, leg. Krátky,J., det. Krátky,J.
Sitona suturalis<br>Stephens, 1831	MK891809 [new]<br>2069-JKR<br>658nt	ZFMK-TIS-23709<br>ZFMK-DNA-0171661738	Czech Republic, Bohemia, Ujezd u Sezemic, N50°07'7.5" E15°51'37", 234m, 08-Mai-2014, sweeping, leg. Krátky,J., det. Krátky,J.
Sitona suturalis<br>Stephens, 1831	MK891956 [new]<br>2225-JKR<br>658nt	ZFMK-TIS-23963<br>ZFMK-DNA-0169170553	Czech Republic, Bohemia, Bezdekov, N49°22'26" E13°14'49", 397m, 07-Sep-2014, *Lathyrus pratensis*, leg. Benedikt,S., det. Benedikt,S.
Sitona suturalis<br>Stephens, 1831	MK892088 [new]<br>2364-JKR<br>658nt	ZFMK-TIS-25924<br>ZFMK-DNA-0171600577	Czech Republic, Moravia, NP Podyji, Podmyce, Jejkal Pond, N48°51'24" E15°54'15.5", 420m, 17-Aug-2014, sweeping, leg. R. Stejskal, det. Stejskal,R.
Sitona suturalis<br>Stephens, 1831	MK891445 [new]<br>1510-PSP<br>658nt	ZFMK-TIS-3270<br>ZFMK-DNA-0155633319	Denmark, Syddanmark, Emmerlev Klev (Højer), N54°59'53" E08°39'08", 3m, 30-Aug-2012, *Lathyrus pratensis*, dipnet, leg. Sprick,P., det. Sprick,P.
Sitona suturalis<br>Stephens, 1831	MK891124 [new]<br>1029-PSP<br>658nt	ZFMK-TIS-3174<br>ZFMK-DNA-0100426830	Germany, Lower Saxony (NI), Hannover, Ricklingen, Leine bottomland W of Maschsee, N52°21'02" E09°44'22", 53m, 22-Mai-2012, *Vicia sepium*, dipnet, leg. Sprick,P., det. Sprick,P.
Sitona suturalis<br>Stephens, 1831	MK891394 [new]<br>1454-PSP<br>658nt	ZFMK-TIS-3214<br>ZFMK-DNA-0155633375	Germany, Lower Saxony (NI), Langeoog, dunes-/wayside, N53°45'03" E07°34'33", 2m, 16-Jun-2012, *Lathyrus pratensis*, beating, leg. Sprick,P., det. Sprick,P.
Sitona suturalis<br>Stephens, 1831	MK892280 [new]<br>2581-PSP<br>658nt	ZFMK-TIS-23555<br>ZFMK-DNA-0171661969	Germany, Bavaria (BY), Unterfranken, Ebern, N50°05'20.5" E10°45'59", 333m, 13-Jun-2014, *Vicia cracca*, leg. Sprick,P., det. Sprick,P.
Sitona suturalis<br>Stephens, 1831	KC784204 [new]<br>445-RGO<br>658nt	ZFMK-TIS-20231<br>ZFMK-DNA-0100449394	Poland, Rudnik, N51°14'31.02" E22°32'22.92", 191m, 03-Okt-2011, leg. Gosik,R., det. Gosik,R.
Sitophilus kakourisi<br>Alziar & Colonnelli, 2009	MK890872 [new]<br>482-PST<br>658nt	ZFMK-TIS-20267<br>ZFMK-DNA-0100449095	Cyprus, Trodos Mts., N of Stavros, N35°02'59" E32°37'35", 986m, 24-Apr-2010, *Quercus alnifolia (acorn)*, sieving / hand-collecting, leg. Stüben,P., det. Stüben,P.
Sitophilus oryzae<br>(Linnaeus, 1763)	MK892142 [40]<br>2429-JKR<br>658nt	ZFMK-TIS-26006<br>ZFMK-DNA-0171600656	Slovakia, Nitra, Koli?any, Koli?ansky vrch, N48°20'15.5" E18°10'47", 261m, 21-Sep-2014, sweeping, leg. Krátky,J., det. Krátky,J.
Sitophilus zeamais<br>Motschulsky, 1855	MK891378 [new]<br>1430-JKR<br>658nt	ZFMK-TIS-4199<br>ZFMK-DNA-0155628503	France, Camargue, Saintes Maries de la Mer, N43°27'12" E04°25'54", 4m, 25-Jul-2013, *Oryza sativa*, ex larvae, leg. Krátky,J., det. Krátky,J.

**Name**<br>**Authority**<br>**Additional Information**	**GenBank Acc No (Ref.)**<br>**Specimen ID**<br>**Sequence Length**	**ZFMK Tissue ID**<br>**ZFMK DNA Sample ID**<br>**(SDEI DNA Sample ID)**	**Locality, GPS, Collection Date, Plant, Collector, Identifier**
Sitophilus zeamais<br>Motschulsky, 1855	MK891353 [new]<br>1398-PST<br>658nt	ZFMK-TIS-4694<br>ZFMK-DNA-0155628532	Italy, Lazio, NW of San Donato Val di Camino, N of Alvito/S. Onófrio, Valle di Rio, N41°43'24" E13°44'41", 671m, 27-Aug-2013, mixed forest, sieving, leg. Stüben,P., det. Stüben,P.
Sitophilus zeamais<br>Motschulsky, 1855	MK891664 [40]<br>1862-PST<br>658nt	ZFMK-TIS-26065<br>ZFMK-DNA-0171606099	Portugal, Madeira, Paul do Mar, N32°45'34" W17°13'43", 62m, 02-Jul-2014, *Ficus*, sieving, leg. Stüben,P., det. Stüben,P.
Smicronyx albosquamosus<br>Wollaston, 1854	MK347549 [40]<br>2962-PST<br>658nt	(SDEI-DNA-2962-PST)	Spain, Canary Islands, El Hierro, W Pozo de La Salud, N27°45'29" W18°06'41", 30m, 11-Apr-2016, *Cuscuta*, leg. Stüben & Schütte, det. Stüben,P.
Smicronyx albosquamosus<br>Wollaston, 1854	MK892404 [40]<br>2883-PST<br>658nt	ZFMK-TIS-25888<br>ZFMK-DNA-0169166910	Spain, Canary Islands, El Hierro, Punta de La Dehesa, Arenas Blancas, N27°46'00" W18°07'19", 9m, 16-Apr-2016, *Cuscuta*, beating, leg. Stüben,P. & Schütte,A., det. Stüben,P.
Smicronyx albosquamosus<br>Wollaston, 1854	KU942332 [30]<br>2726-PST<br>658nt	ZFMK-TIS-23876<br>ZFMK-DNA-0171661282	Spain, Canary Islands, Fuerteventura, Betancuria, N28°25'09" W14°03'32", 380m, 04-Jan-2015, *Cuscuta*, beating, leg. Stüben,P., det. Stüben,P.
Smicronyx albosquamosus<br>Wollaston, 1854	KC783753 [30]<br>41-PST<br>655nt	ZFMK-TIS-2D100447003<br>ZFMK-DNA-0100438075	Spain, Canary Islands, Gran Canaria, Agüimes, Barranco de Guayadeque, N27°56'09" W15°29'25", 671m, 03-Jan-2011, *Phagnalon*, beating, leg. Stüben,P., det. Stüben,P.
Smicronyx albosquamosus<br>Wollaston, 1854	KU942331 [30]<br>2652-HAR (JHA-15)<br>658nt	ZFMK-TIS-24067<br>ZFMK-DNA-0169170993	Israel, Yizhar, N32°10'04" E35°14'13", 788m, 11-Jun-2013, *Quercus calliprinos*, leg. Friedmann,L., det. Haran,J.
Smicronyx albosquamosus<br>Wollaston, 1854	KC783772 [30]<br>63-PST<br>658nt	ZFMK-TIS-2D100446976<br>ZFMK-DNA-0100438048	Spain, Canary Islands, La Gomera, Hermigua, Las Nuevitas, Barranquillos los Alamos, N28°09'31" W17°11'09", 251m, 13-Dez-2010, *Cuscuta*, beating, leg. Stüben,P., det. Stüben,P.
Smicronyx coecus<br>(Reich, 1797)	KU942325 [30]<br>2646-HAR (JHA-9)<br>658nt	ZFMK-TIS-24061<br>ZFMK-DNA-0169170342	Armenia, Gosh, N40°43'46" E44°59'52", 1220m, 08-Mai-2013, sweeping, leg. Haran,J., det. Haran,J.
Smicronyx coecus<br>(Reich, 1797)	KU942323 [30]<br>2644-HAR (JHA-7)<br>658nt	ZFMK-TIS-24059<br>ZFMK-DNA-0169170340	France, Ardon, N47°49'48" E01°54'45", 108m, 20-Jul-2011, *Cuscuta epithymum*, beating, leg. Haran,J., det. Haran,J.
Smicronyx coecus<br>(Reich, 1797)	KU942324 [30]<br>2645-HAR (JHA-8)<br>658nt	ZFMK-TIS-24060<br>ZFMK-DNA-0169170341	France, Col de la Colombiere, N45°59'31" E06°28'32", 1611m, 08-Jun-2014, under stones, leg. Haran,J., det. Haran,J.
Smicronyx coecus<br>(Reich, 1797)	KC784156 [30]<br>604-PSP<br>658nt	ZFMK-TIS-20387<br>ZFMK-DNA-0100448658	Germany, Lower Saxony (NI), Hannover, Herrenhausen, Leine river, N52°23'29" E09°40'40", 47m, 15-Jun-2011, *Cuscuta europaea*, beating, leg. Sprick,P., det. Sprick,P.
Smicronyx coecus<br>(Reich, 1797)	KU942314 [30]<br>1923-FBA<br>658nt	ZFMK-TIS-23314<br>ZFMK-DNA-0169170371	Greece, East Macedonia and Thrace, Drama, Potami N, N41°24'52" E24°06'45", 460m, 14-Aug-2014, leg. Bayer & Brunner, det. Bayer,F.
Smicronyx jordanicus<br>Haran, 2017<br>**Paratype (DNAtype)**	KU942329 [30]<br>2650-HAR_24065 (JHA-13)<br>658nt	ZFMK-TIS-24065<br>ZFMK-DNA-0169170346	Israel, Jericho, N31°51'44" E35°29'21", -293m, 07-Mai-2014, *Cuscuta* sp. *(monogyna ?)*, beating, leg. Haran,J., det. Haran,J.

**Name**<br>**Authority**<br>**Additional Information**	**GenBank Acc No (Ref.)**<br>**Specimen ID**<br>**Sequence Length**	**ZFMK Tissue ID**<br>**ZFMK DNA Sample ID**<br>**(SDEI DNA Sample ID)**	**Locality, GPS, Collection Date, Plant, Collector, Identifier**
Smicronyx jungermanniae<br>(Reich, 1797)	KU942330 [30]<br>2651-HAR (JHA-14)<br>658nt	ZFMK-TIS-24066<br>ZFMK-DNA-0169170998	Armenia, Sardarapat, N40°08'01" E44°06'36", 863m, 30-Apr-2013, sweeping, leg. Haran,J., det. Haran,J.
Smicronyx jungermanniae<br>(Reich, 1797)	KU942316 [30]<br>2302-JKR<br>658nt	ZFMK-TIS-24233<br>ZFMK-DNA-0169170968	Czech Republic, Bohemia, Krinec, PP Chotuc, N50°15'59" E15°06'59", 236m, 02-Jul-2014, *Cuscuta epithymum*, sweeping, leg. Krátky,J., det. Krátky,J.
Smicronyx jungermanniae<br>(Reich, 1797)	MK347570 [new]<br>2996-PST<br>658nt	(SDEI-DNA-2996-PST)	Greece, Macedonia, Thessaloniki, Agios Vasileios, near Koronia Lake, N40°40'05" E23°07'06", 75m, 20-Jul-2017, *Cuscuta*, leg. Stüben,P., det. Stüben,P.
Smicronyx jungermanniae<br>(Reich, 1797)	MK891705 [new]<br>1924-FBA<br>658nt	ZFMK-TIS-23315<br>ZFMK-DNA-0169170359	Greece, East Macedonia and Thrace, Drama, Potami N, N41°24'52" E24°06'45", 460m, 14-Aug-2014, leg. Bayer & Brunner, det. Bayer,F.
Smicronyx jungermanniae<br>(Reich, 1797)	KU942320 [30]<br>2640-HAR (JHA-3)<br>658nt	ZFMK-TIS-24055<br>ZFMK-DNA-0169168531	Israel, Ga' ash, N32°13'42" E34°40'27", 29m, 01-Mai-2014, *Cuscuta campestris*, beating, leg. Haran,J., det. Haran,J.
Smicronyx jungermanniae<br>(Reich, 1797)	KU942315 [30]<br>2033-JKR<br>658nt	ZFMK-TIS-23673<br>ZFMK-DNA-0169170468	Slovakia, Nove Zamky, Bajtava, Ploska hora, N47°51'54" E18°45'15", 126m, 22-Mai-2014, sweeping, leg. Krátky,J., det. Krátky,J.
Smicronyx jungermanniae<br>(Reich, 1797)	KU942321 [30]<br>2641-HAR (JHA-4)<br>658nt	ZFMK-TIS-24056<br>ZFMK-DNA-0169170337	Spain, Lugo, N43°00'35" E07°33'24", 466m, 14-Jul-2014, *Cuscuta* sp., beating, leg. Haran,J., det. Haran,J.
Smicronyx longitarsis<br>Haran, 2017<br>**Paratype (DNAtype)**	KU942328 [30]<br>2649-HAR_24064 (JHA-12)<br>658nt	ZFMK-TIS-24064<br>ZFMK-DNA-0169170345	Israel, Hebron, N31°29'41" E35°08'23", 877m, 11-Mai-2014, *Cuscuta palaestiana*, sweeping, leg. Haran,J., det. Haran,J.
Smicronyx nebulosus<br>Tournier, 1874	KU942319 [30]<br>2639-HAR (JHA-2)<br>658nt	ZFMK-TIS-24054<br>ZFMK-DNA-0169170348	Portugal, lamego, N41°05'04" E07°49'46", 730m, 14-Jul-2014, *Cuscuta* sp., beating, leg. Haran,J., det. Haran,J.
Smicronyx pauperculus<br>Wollaston, 1864	KU942318 [30]<br>2638-HAR (JHA-1)<br>658nt	ZFMK-TIS-24053<br>ZFMK-DNA-0169170349	Israel, Ga' ash, N32°13'42" E34°40'27", 29m, 01-Mai-2014, *Cuscuta campestris*, beating, leg. Haran,J., det. Haran,J.
Smicronyx pauperculus<br>Wollaston, 1864	KU942313 [30]<br>1732-PST<br>658nt	ZFMK-TIS-24450<br>ZFMK-DNA-0155622465	Spain, Canary Islands, La Palma, E of Barlovento, above La Fajana (Bco. de los Hombres), N28°49'49" W17°52'11", 185m, 31-Dez-2013, *Cuscuta*, beating, leg. Stüben,P., det. Stüben,P.
Smicronyx pauperculus<br>Wollaston, 1864	KC784301 [40]<br>732-PST<br>658nt	ZFMK-TIS-3069<br>ZFMK-DNA-0100448158	Spain, Canary Islands, Tenerife, Güimar valley, Arafo near La Hidalgo, roadside, N28°20'23" W16°24'16", 339m, 28-Jan-2012, *Cuscuta*, beating, leg. Stüben,P., det. Stüben,P.
Smicronyx smreczynskii<br>F. Solari, 1952	KU942317 [30]<br>2305-JKR<br>658nt	ZFMK-TIS-24236<br>ZFMK-DNA-0169170971	Czech Republic, Bohemia, Krinec, PP Chotuc, N50°15'59" E15°06'59", 236m, 02-Jul-2014, *Cuscuta epithymum*, sweeping, leg. Krátky,J., det. Krátky,J.
Smicronyx smreczynskii<br>F. Solari, 1952	KU942322 [30]<br>2643-HAR (JHA-6)<br>658nt	ZFMK-TIS-24058<br>ZFMK-DNA-0169170339	France, Floirac, N44°55'38" E01°40'10", 115m, 10-Jul-2013, *Cuscuta scandens*, rearing galls, leg. Haran,J., det. Haran,J.

**Name**<br>**Authority**<br>**Additional Information**	**GenBank Acc No (Ref.)**<br>**Specimen ID**<br>**Sequence Length**	**ZFMK Tissue ID**<br>**ZFMK DNA Sample ID**<br>**(SDEI DNA Sample ID)**	**Locality, GPS, Collection Date, Plant, Collector, Identifier**
Smicronyx syriacus<br>Faust, 1887	KU942327 [30]<br>2648-HAR (JHA-11)<br>658nt	ZFMK-TIS-24063<br>ZFMK-DNA-0169170344	Israel, Yizhar, N32°10'04" E35°14'13", 788m, 14-Mai-2014, sweeping, leg. Haran,J., det. Haran,J.
Smicronyx syriacus<br>Faust, 1887	KU942326 [30]<br>2647-HAR (JHA-10)<br>658nt	ZFMK-TIS-24062<br>ZFMK-DNA-0169170343	Spain, Palencia, N41°59'27" E04°27'05", 756m, 28-Mai-2013, beating, leg. Haran,J., det. Haran,J.
Sonchiacalles muelleri<br>(Stüben, 2000)	GU987791 [01]<br>C-0113-mue<br>658nt	ZFMK-TIS-cC0113<br>ZFMK-DNA-0100400685	Spain, Canary Islands, El Hierro, 3 km N of San Andres, N-Hang Montana de la Fara, N27°47'39" W17°56'55", 936m, 25-Dez-2006, *Tolpis lacinata*, leg. Stüben,P., det. Stüben,P.
Sonchiacalles muelleri<br>(Stüben, 2000)	GU987798 [01]<br>C-0114-mue<br>658nt	ZFMK-TIS-cC0114<br>ZFMK-DNA-0100400246	Spain, Canary Islands, El Hierro, 3 km SW of La Frontera, road to Frontera, N27°44'06" W18°00'13", 1302m, 22-Dez-2006, *Tolpis proustii*, leg. Stüben,P., det. Stüben,P.
Sonchiacalles muelleri<br>(Stüben, 2000)	FJ716537 [01]<br>C-0112-mue<br>658nt	ZFMK-TIS-cC0112<br>ZFMK-DNA-0100400684	Spain, Canary Islands, La Palma, 6 km N of Las Caletas, Cumbre Vieja, N28°32'15" W17°49'42", 1212m, 28-Jun-2006, *Tolpis calderae*, leg. Stüben,P., det. Stüben,P.
Sonchiacalles muelleri<br>(Stüben, 2000)	GU987847 [01]<br>E-0245-mue<br>658nt	ZFMK-TIS-cE0245<br>ZFMK-DNA-0100401062	Spain, Canary Islands, La Palma, 7.5 km E of Garafia, Bco. de los Hombres, N28°49'50" W17°52'03", 386m, 10-Jul-2006, *Tolpis lagoboda/laciniata*, leg. Stüben,P., det. Stüben,P.
Sonchiacalles muelleri<br>(Stüben, 2000)	GU987848 [01]<br>E-0246-mue<br>629nt	ZFMK-TIS-cE0246<br>ZFMK-DNA-0100401061	Spain, Canary Islands, La Palma, 4.5 km E of Puntagorda, Lomo de la Ciudad, N28°46'24" W17°56'16", 1241m, 15-Jul-2006, *Tolpis lagopoda*, leg. Stüben,P., det. Stüben,P.
Sonchiacalles muelleri<br>(Stüben, 2000)	MG229776 [40]<br>1999-JKR<br>658nt	ZFMK-TIS-23639<br>ZFMK-DNA-0169170502	Spain, Canary Islands, La Palma, Caldera de Taburiente, Lomo de Pablo, N28°44'39" W17°49'53.5", 2051m, 11-Feb-2014, *Tolpis calderae*, sieving, leg. Krátky,J., det. Krátky,J.
Sonchiacalles muelleri<br>(Stüben, 2000)	MK891623 [40]<br>1733-PST<br>658nt	ZFMK-TIS-24451<br>ZFMK-DNA-0155622466	Spain, Canary Islands, La Palma, E of Barlovento, above La Fajana (Bco. de los Hombres), N28°49'49" W17°52'11", 185m, 31-Dez-2013, *Tolpis*, beating, leg. Stüben,P., det. Stüben,P.
Sonchiacalles muelleri<br>(Stüben, 2000)	MK891627 [40]<br>1745-PST<br>658nt	ZFMK-TIS-24463<br>ZFMK-DNA-0155622445	Spain, Canary Islands, La Palma, E of Roque de los Muchachos near F. de Vizcaino, N28°44'39" W17°49'53", 2057m, 16-Jan-2014, *Tolpis*, sieving, leg. Stüben,P., det. Stüben,P.
Sonchiacalles silosensis<br>(Stüben, 2000)	FJ716579 [01]<br>E-729-sil<br>658nt	ZFMK-TIS-cE729<br>ZFMK-DNA-0100404826	Spain, Canary Islands, La Gomera, SE of Hermigua near Casas del Palmar, N28°09'29" W17°09'37", 627m, 08-Okt-2008, *Sonchus gomerensis*, thermophilic brushwood, leg. Astrin,J. & Stüben,P., det. Stüben,P.
Sonchiacalles silosensis<br>(Stüben, 2000)	GU987929 [01]<br>E-551-sil<br>628nt	ZFMK-TIS-cE551<br>ZFMK-DNA-0100404992	Spain, Canary Islands, Tenerife, 1 km S of Los Silos, N28°21'16" W16°48'49", 300m, 23-Dez-2003, *Sonchus* sp., thermophilic brushwood, leg. Stüben,P., det. Stüben,P.
Sonchiacalles silosensis<br>(Stüben, 2000)	KJ867579 [40]<br>712-PST<br>658nt	ZFMK-TIS-3049<br>ZFMK-DNA-0100448186	Spain, Canary Islands, Tenerife, Teno Mts., Los Silos, Barranco de Bucarón, N28°21'36" W16°48'51", 143m, 09-Jan-2012, *Sonchus* sp., beating, leg. Stüben,P., det. Stüben,P.
Sonchiacalles sonchi<br>(Stüben, 2000)	GU988022 [01]<br>E-726-son<br>658nt	ZFMK-TIS-cE726<br>ZFMK-DNA-0100404829	Spain, Canary Islands, La Gomera, SE of Hermigua near Casas del Palmar, N28°09'29" W17°09'37", 627m, 08-Okt-2008, *Sonchus gomerensis*, thermophilic brushwood, leg. Astrin,J. & Stüben,P., det. Stüben,P.
Sonchiacalles sonchi<br>(Stüben, 2000)	KF672595 [12]<br>ES1040<br>658nt	ZFMK-TIS-cES1040<br>ZFMK-DNA-0112704630	Spain, Canary Islands, La Gomera, Agulo – LasRosas, 28°11'26"N 17°12'6"W, 388m, 28-Feb-2010, *Sonchus regis jubae*, leg. Stüben,P., det. Stüben,P.

**Name**<br>**Authority**<br>**Additional Information**	**GenBank Acc No (Ref.)**<br>**Specimen ID**<br>**Sequence Length**	**ZFMK Tissue ID**<br>**ZFMK DNA Sample ID**<br>**(SDEI DNA Sample ID)**	**Locality, GPS, Collection Date, Plant, Collector, Identifier**
Sonchiacalles sonchi<br>(Stüben, 2000)	GU987787 [01]<br>C-0110-son<br>658nt	ZFMK-TIS-cC0110<br>ZFMK-DNA-0100400695	Spain, Canary Islands, La Palma, 6 km E of Garafia, 3 km S of El Tablado, N28°49'42" W17°52'43", 404m, 12-Jul-2006, *Sonchus palmensis*, leg. Stüben,P., det. Stüben,P.
Sonchiacalles sonchi<br>(Stüben, 2000)	FJ716569 [01]<br>E-699-son<br>658nt	ZFMK-TIS-cE699<br>ZFMK-DNA-0100404865	Spain, Canary Islands, Tenerife, E of Los Silos, Teno Mts., El Tanque Bajo, N28°21'39" W16°46'19", 534m, 03-Okt-2008, compost, leg. Astrin,J. & Stüben,P., det. Stüben,P.
Sonchiacalles sonchi<br>(Stüben, 2000)	GU987930 [01]<br>E-552-son<br>658nt	ZFMK-TIS-cE552<br>ZFMK-DNA-0100404295	Spain, Canary Islands, Tenerife, Anaga Mts., 10 km NE of La Laguna, Afur, N28°33'10" W16°14'59", 300m, 29-Dez-2003, *Sonchus acaulis*, succulent bush, leg. Stüben,P., det. Stüben,P.
Sonchiacalles sonchi<br>(Stüben, 2000)	GU988001 [01]<br>E-685-son<br>658nt	ZFMK-TIS-cE685<br>ZFMK-DNA-0100404870	Spain, Canary Islands, Tenerife, NE of La Laguna, Anaga Mts., Las Bodegas, N28°33'42" W16°09'25", 502m, 30-Sep-2008, *Sonchus acaulis*, leg. Astrin,J. & Stüben,P., det. Stüben,P.
Sonchiacalles sonchi<br>(Stüben, 2000)	GU988006 [01]<br>E-697-son<br>658nt	ZFMK-TIS-cE697<br>ZFMK-DNA-0100404863	Spain, Canary Islands, Tenerife, W of Los Silos, Teno Mts., Casa Blanca near Buenavista del Norte, N28°21'36" W16°52'10", 231m, 02-Okt-2008, leg. Astrin,J. & Stüben,P., det. Stüben,P.
Sphenophorus parumpunctatus<br>(Gyllenhal, 1838)	KC783887 [new]<br>165-PST<br>658nt	ZFMK-TIS-2D100440259<br>ZFMK-DNA-0100438335	Morocco, Atlas Mts., S of Taza, Jebel Tazzeka near Bat-Bou-Idir, N34°05'02" W04°06'49", 1408m, 16-Mai-2011, *meadow/gras*, beating, leg. Stüben,P., det. Behne,L.
Sphenophorus piceus<br>(Pallas, 1771)	KC784320 [new]<br>775-ASC<br>658nt	ZFMK-TIS-3112<br>ZFMK-DNA-0100448105	Croatia, Split, Solin, parking lot near river, N43°32.464" E16°30.187", 15m, 22-Apr-2012, *Arctium* sp., sieving, leg. Schütte,A., det. Stüben,P.
Sphenophorus striatopunctatus<br>(Goeze, 1777)	MK891900 [new]<br>2166-JKR<br>658nt	ZFMK-TIS-23902<br>ZFMK-DNA-0169168530	Czech Republic, Moravia, Znojmo-Popice, N48°49'26" E16°01'14", 280m, 03-Apr-2014, collecting by hand, leg. Stejskal,R., det. Stejskal,R.
Sphincticraerus lethierryi<br>(Desbrochers des Loges, 1869)	MK891498 [new]<br>1583-JKR<br>658nt	ZFMK-TIS-3631<br>ZFMK-DNA-0155635646	Morocco, Haut Atlas, Tinmel env., N30°59'00" W08°13'06", 1226m, 19-Apr-2013, beating, leg. Krátky,J., det. Krátky,J.
Squamapion elongatum<br>(Germar, 1817)	MK891835 [new]<br>2096-JKR<br>658nt	ZFMK-TIS-23736<br>ZFMK-DNA-0169169637	Czech Republic, Moravia, Bucovice, PR Sevy, N49°08'05" E16°58'19", 249m, 09-Mai-2014, *Salvia pratensis*, sweeping, leg. Krátky,J., det. Krátky,J.
Squamapion elongatum<br>(Germar, 1817)	MK892095 [new]<br>2372-JKR<br>658nt	ZFMK-TIS-25949<br>ZFMK-DNA-0171600594	Czech Republic, Moravia, Znojmo, N48°51'05" E16°05'59", 250m, 23-Aug-2014, *Salvia nemorosa*, sweeping, leg. R. Stejskal, det. Stejskal,R.
Squamapion elongatum<br>(Germar, 1817)	KC784199 [new]<br>450-RGO<br>658nt	ZFMK-TIS-20236<br>ZFMK-DNA-0100449389	Poland, Rudnik, N51°14'31.02" E22°32'22.92", 191m, 03-Okt-2011, leg. Gosik,R., det. Gosik,R.
Squamapion flavimanum<br>(Gyllenhal, 1833)	MK892085 [new]<br>2361-JKR<br>658nt	ZFMK-TIS-25921<br>ZFMK-DNA-0171600580	Czech Republic, Moravia, NP Podyji, Cizov, Siroke pole, N48°51'36" E15°51'02", 300m, 17-Aug-2014, *Origanum vulgare*, sweeping, leg. R. Stejskal, det. Stejskal,R.
Squamapion flavimanum<br>(Gyllenhal, 1833)	MK347573 [new]<br>3005-PST<br>658nt	(SDEI-DNA-3005-PST)	Germany, Rhineland-Palatinate (RLP), Mosel: Zummelhöhe (cave)near Leiwen, N49°48'53” E 6°54'41“, 265m, 06-Sep-2017, *Origanum vulgare*, leg. Stüben,P., det. Stüben,P.
Stenocarus cardui<br>(Herbst, 1784)	MK891531 [new]<br>1619-JKR<br>658nt	ZFMK-TIS-3667<br>ZFMK-DNA-0155635617	Slovakia, Komarno, B?c env., N47°46'55" E18°25'11", 109m, 18-Mai-2013, *Papaver rhoeas*, individual collecting by hand, leg. Krátky,J., det. Krátky,J.

**Name**<br>**Authority**<br>**Additional Information**	**GenBank Acc No (Ref.)**<br>**Specimen ID**<br>**Sequence Length**	**ZFMK Tissue ID**<br>**ZFMK DNA Sample ID**<br>**(SDEI DNA Sample ID)**	**Locality, GPS, Collection Date, Plant, Collector, Identifier**
Stenocarus cf. *cardui*	KC783867 [new]<br>139-PST<br>658nt	ZFMK-TIS-2D100440281<br>ZFMK-DNA-0100438169	Morocco, SE of Berkane, N of Aïn-es-Sfa, Aïn Almou, Beni Snassen Mts., N34°50'59" W02°12'05", 1313m, 13-Mai-2011, *Quercus*, sieving, leg. Stüben,P., det. Stüben,P./vid. Behne,L.
Stenocarus ruficornis<br>(Stephens, 1831)	KC783954 [new]<br>364-JKR<br>658nt	ZFMK-TIS-20150<br>ZFMK-DNA-0100438894	Czech Republic, Bohemia or., Horineves, N50°18'52.155" E15°46'30.012", 230m, 25-Jun-2011, sweeping, leg. Krátky,J., det. Krátky,J.
Stenocarus ruficornis<br>(Stephens, 1831)	MK890940 [new]<br>809-PST<br>658nt	ZFMK-TIS-3722<br>ZFMK-DNA-0100413827	Germany, Rhineland-Palatinate (RLP), N of Treis-Karden, Brohl, N50°13'22" E07°16'27", 255m, 10-Jun-2012, *Papaver*, roadside ditch, beating, leg. Stüben,P., det. Stüben,P.
Stenocarus ruficornis<br>(Stephens, 1831)	MK891419 [new]<br>1481-PSP<br>658nt	ZFMK-TIS-3241<br>ZFMK-DNA-0155633348	Germany, Lower Saxony (NI), Hameln-Rohrsen, Dütberg, N52°06'29" E09°25'12", 97m, 15-Jul-2012, *Trifoilum medium, Trifolium pratense*, dipnet, leg. Sprick,P., det. Sprick,P.
Stenopelmus rufinasus<br>Gyllenhal, 1835	KC784067 [new]<br>560-RST<br>658nt	ZFMK-TIS-20345<br>ZFMK-DNA-0100448422	Slovakia, Slovakia occ., 0.7 km SE of Dolne Zelenice, N48°22'13.95" E17°45'8.62", 135m, 02-Okt-2011, *Utricularia* sp., hand-collecting, leg. Stejskal,R., det. Stejskal,R.
Stenopterapion argamani<br>Friedman & Freidberg, 2007	MK892338 [new]<br>2665-PSP<br>658nt	ZFMK-TIS-23815<br>ZFMK-DNA-0171661334	Cyprus, Troodos, Pano Platres, N34°53'30" E32°51'58", 1149m, 10-Apr-2013, *Acer obtusifolium*, beating, leg. Sprick,P., det. Sprick,P.
Stenopterapion cf. *meliloti*	KC784105 [new]<br>512-RST<br>658nt	ZFMK-TIS-20297<br>ZFMK-DNA-0100448470	Czech Republic, Moravia mer., kaolin pit 1.5 km SE of Únanov, N48°53'17.75" E16°3'22.34", 330m, 02-Jul-2011, *Melilotus*, beating, leg. Stejskal,R., det. Stejskal,R.
Stenopterapion meliloti<br>(Kirby, 1808)	KC784160 [new]<br>610-PSP<br>658nt	ZFMK-TIS-20393<br>ZFMK-DNA-0100448664	Germany, Lower Saxony (NI), Brelingen, Hannover region, N52°33'53" E09°40'55", 79m, 25-Jun-2011, *Melilotus albus*, dry-warm ruderal area, beating, leg. Sprick,P., det. Sprick,P.
Stenopterapion meliloti<br>(Kirby, 1808)	MK892277 [new]<br>2578-PSP<br>658nt	ZFMK-TIS-23552<br>ZFMK-DNA-0171661981	Germany, Bavaria (BY), Unterfranken, Ebern, N50°05'22" E10°46'02", 327m, 13-Jun-2014, *Melilotus officinalis*, leg. Sprick,P., det. Sprick,P.
Stenopterapion meliloti<br>(Kirby, 1808)	MK891025 [new]<br>922-CBR<br>658nt	ZFMK-TIS-3835<br>ZFMK-DNA-0100449696	Luxembourg, 6 km E of Luxembourg, Oetrange, N49°36'20" E06°15'32", 250m, 14-Jun-2011, *Melilotus albus*, beating, leg. Braunert,C., det. Braunert,C.
Stenopterapion tenue<br>(Kirby, 1808)	MK892098 [new]<br>2375-JKR<br>658nt	ZFMK-TIS-25952<br>ZFMK-DNA-0171600597	Czech Republic, Moravia, Znojmo, N48°51'05" E16°05'59", 250m, 23-Aug-2014, sweeping, leg. R. Stejskal, det. Stejskal,R.
Stenopterapion tenue<br>(Kirby, 1808)	MK891449 [new]<br>1514-PSP<br>658nt	ZFMK-TIS-3274<br>ZFMK-DNA-0155633304	Denmark, Syddanmark, Emmerlev Klev (Højer), N54°59'53" E08°39'08", 3m, 30-Aug-2012, *Medicago lupulina*, dipnet, leg. Sprick,P., det. Sprick,P.
Stenopterapion tenue<br>(Kirby, 1808)	MK892294 [new]<br>2595-PSP<br>658nt	ZFMK-TIS-23569<br>ZFMK-DNA-0171661964	Germany, Bavaria (BY), Unterfranken, Ebern, N50°05'17" E10°45'53", 343m, 13-Jun-2014, *Medicago lupulina (eng. lucerne)*, leg. Sprick,P., det. Sprick,P.
Stenopterapion tenue<br>(Kirby, 1808)	KC784197 [new]<br>452-RGO<br>658nt	ZFMK-TIS-20238<br>ZFMK-DNA-0100449387	Poland, Rudnik, N51°14'31.02" E22°32'22.92", 191m, 03-Okt-2011, leg. Gosik,R., det. Gosik,R.
Stereocorynes truncorum<br>(Germar, 1824)	MK891561 [new]<br>1655-JKR<br>658nt	ZFMK-TIS-4233<br>ZFMK-DNA-0155630485	Slovakia, Zlate Moravce, Obyce - Jadova, N48°26'25" E18°30'02", 450m, 13-Okt-2013, *Fagus*, individual collecting by hand, leg. Krátky,J., det. Krátky,J.

**Name**<br>**Authority**<br>**Additional Information**	**GenBank Acc No (Ref.)**<br>**Specimen ID**<br>**Sequence Length**	**ZFMK Tissue ID**<br>**ZFMK DNA Sample ID**<br>**(SDEI DNA Sample ID)**	**Locality, GPS, Collection Date, Plant, Collector, Identifier**
Stereonychus fraxini<br>(DeGeer, 1775)	KC784097 [new]<br>519-RST<br>658nt	ZFMK-TIS-20304<br>ZFMK-DNA-0100448460	Czech Republic, Bohemia bor., Milesovka, 13.5 km W of Litomerice, N50°33'32.98" E13°55'52.71", 600m, 06-Jul-2011, *Fraxinus*, beating, leg. Stejskal,R., det. Stejskal,R.
Stereonychus fraxini<br>(DeGeer, 1775)	KC784009 [new]<br>240-PSP<br>658nt	ZFMK-TIS-2D100439134<br>ZFMK-DNA-0100439806	Germany, Lower Saxony (NI), Ockensen, Lkr. Hameln-Pyrmont, Ith Mts., N52°01'19" E09°35'07", 290m, 04-Mai-2011, *Fraxinus excelsior*, beating, leg. Sprick,P., det. Sprick,P.
Stereonychus fraxini<br>(DeGeer, 1775)	MK890924 [new]<br>792-PST<br>658nt	ZFMK-TIS-3705<br>ZFMK-DNA-0100414299	Germany, Rhineland-Palatinate (RLP), E of Treis-Karden, Pommern, Pommernbachtal (river valley), N50°10'52" E07°16'42", 116m, 09-Jun-2012, *Fraxinus excelsior*, forest path, beating, leg. Stüben,P., det. Stüben,P.
Stereonychus phyllireae<br>(Chevrolat, 1859)	MK891305 [new]<br>1289-PST<br>658nt	ZFMK-TIS-4105<br>ZFMK-DNA-0100426424	Spain, Community of Madrid, SW of Lozoya, Pinilla del Valle, N40°55'44" W03°49'29", 1108m, 03-Mai-2013, *Fraxinus*, beating, leg. Stüben,P. & Schütte,A., det. Stüben,P.
Stereonychus rufobrunneus<br>(H. Lindberg, 1953)	KC783766 [40]<br>56-PST<br>618nt	ZFMK-TIS-2D100446992<br>ZFMK-DNA-0100438064	Spain, Canary Islands, La Gomera, Hermigua, Las Nuevitas, Barranquillos los Alamos, N28°09'31" W17°11'09", 251m, 13-Dez-2010, *Globularia*, beating, leg. Stüben,P., det. Stüben,P.
Stereonychus rufobrunneus<br>(H. Lindberg, 1953)	MK891622 [40]<br>1731-PST<br>658nt	ZFMK-TIS-24449<br>ZFMK-DNA-0155622464	Spain, Canary Islands, La Palma, E of Barlovento, above La Fajana (Bco. de los Hombres), N28°49'49" W17°52'11", 185m, 31-Dez-2013, *Globularia salicina*, beating, leg. Stüben,P., det. Stüben,P.
Stereonychus rufobrunneus<br>(H. Lindberg, 1953)	MK891746 [40]<br>1998-JKR<br>658nt	ZFMK-TIS-23638<br>ZFMK-DNA-0169170503	Spain, Canary Islands, La Palma, Punta Santa Lucia, N28°43'20" W17°44'07", 67m, 10-Feb-2014, *Globularia salicina*, beating, leg. Krátky,J., det. Krátky,J.
Stereonychus rufobrunneus<br>(H. Lindberg, 1953)	KC784285 [13]<br>700-PST<br>658nt	ZFMK-TIS-3037<br>ZFMK-DNA-0100448187	Spain, Canary Islands, Tenerife, Teno Mts. near Erjos, N28°19'31" W16°48'32", 1017m, 04-Jan-2012, *Globularia*, beating, leg. Stüben,P., det. Stüben,P.
Stomodes gyrosicollis<br>(Boheman, 1842)	MK891792 [new]<br>2051-JKR<br>658nt	ZFMK-TIS-23691<br>ZFMK-DNA-0169170450	Czech Republic, Bohemia, Kosatky, N50°19'23" E14°39'58", 218m, 02-Mai-2014, sieving, leg. Krátky,J., det. Krátky,J.
Strophomorphus albarius<br>(Reiche & Saulcy, 1858)	KC784048 [new]<br>660-PSP<br>658nt	ZFMK-TIS-20442<br>ZFMK-DNA-0100448289	Cyprus, Paphos, Agios Georgios, N34°54'10" E32°19'01", 4m, 21-Apr-2010, *Pistacia lentiscus*, shrub near cost, beating, leg. Sprick,P., det. Sprick,P.
Strophomorphus albarius<br>(Reiche & Saulcy, 1858)	KC784122 [new]<br>661-PSP<br>658nt	ZFMK-TIS-20443<br>ZFMK-DNA-0100448606	Cyprus, Limassol, Souni-Zanakia, Phrygana, N34°41'00" E32°53'00", 240m, 23-Apr-2010, *Pistacia* sp., beating, leg. Sprick,P., det. Sprick,P.
Strophomorphus albarius<br>(Reiche & Saulcy, 1858)	KC784115 [new]<br>677-FBA<br>658nt	ZFMK-TIS-20455<br>ZFMK-DNA-0100448587	Greece, Attica, SE of Athens, Sounio, N37°39'00" E24°01'40", 50m, 17-Mai-2011, leg. Bahr,F., det. Bahr,F.
Strophomorphus porcellus<br>(Schoenherr, 1832)	MK891751 [new]<br>2005-JKR<br>658nt	ZFMK-TIS-23645<br>ZFMK-DNA-0169170496	Italy, Sicilia Isl. (TP), Trapani, Salinagrande, Isolotto, N37°57'14" E12°29'45", 3m, 17-Apr-2014, sweeping, leg. Krátky,J., det. Krátky,J.
Strophosoma capitatum<br>(DeGeer, 1775)	KC783904 [new]<br>213-PSP<br>645nt	ZFMK-TIS-2D100439155<br>ZFMK-DNA-0100438532	Germany, Lower Saxony (NI), Brelingen, Hannover region, N52°33'35" E09°41'04", 70m, 16-Apr-2011, *Quercus robur*, huge sand pit, beating, leg. Sprick,P., det. Sprick,P.
Strophosoma capitatum<br>(DeGeer, 1775)	MK891159 [new]<br>1066-PSP<br>658nt	ZFMK-TIS-3211<br>ZFMK-DNA-0100426800	Germany, Lower Saxony (NI), Langeoog, N53°44'58" E07°30'40", 1m, 16-Jun-2012, *Salix repens*, dune/wayside, beating, leg. Sprick,P., det. Sprick,P.

**Name**<br>**Authority**<br>**Additional Information**	**GenBank Acc No (Ref.)**<br>**Specimen ID**<br>**Sequence Length**	**ZFMK Tissue ID**<br>**ZFMK DNA Sample ID**<br>**(SDEI DNA Sample ID)**	**Locality, GPS, Collection Date, Plant, Collector, Identifier**
Strophosoma capitatum<br>(DeGeer, 1775)	MK891415 [new]<br>1477-PSP<br>658nt	ZFMK-TIS-3237<br>ZFMK-DNA-0155633363	Germany, Lower Saxony (NI), Harz National Park, Oder-pond, N51°46'16" E10°32'03", 734m, 02-Jul-2012, *Picea abies, Calluna vulgaris*, spruce forest, beating, leg. Sprick,P., det. Sprick,P.
Strophosoma capitatum<br>(DeGeer, 1775)	MK891681 [new]<br>1897-PST<br>658nt	ZFMK-TIS-24002<br>ZFMK-DNA-0169168516	Germany, North Rhine-Westphalia (NRW), Elmpt, Overhetfeld, Dilborner Mühle, N51°13'58" E06°10'22", 52m, 27-Aug-2014, beating, leg. Stüben,P., det. Stüben,P.
Strophosoma capitatum<br>(DeGeer, 1775)	MK891009 [new]<br>903-CBR<br>658nt	ZFMK-TIS-3816<br>ZFMK-DNA-0100449708	Luxembourg, 10 km NE of Mersch, 2 km NE of Nommern, N49°48'15" E06°11'16", 340m, 01-Mai-2011, *Quercus*, beating, leg. Braunert,C., det. Braunert,C.
Strophosoma capitatum<br>(DeGeer, 1775)	KC784210 [new]<br>437-RGO<br>658nt	ZFMK-TIS-20223<br>ZFMK-DNA-0100449407	Poland, Zemborzyce, N51°09'56.7" E22°30'10.5", 178m, 18-Sep-2011, leg. Gosik,R., det. Gosik,R.
Strophosoma constrictum<br>(Seidlitz, 1867)	MK891323 [new]<br>1325-PST<br>658nt	ZFMK-TIS-4141<br>ZFMK-DNA-0100426146	Spain, Malaga, NW of Otivar, Sierra del Chapparal, N36°52'21" W03°44'31", 1262m, 08-Mai-2013, *Quercus ilex*, sieving, leg. Stüben,P. & Schütte,A., det. Stüben,P.
Strophosoma faber<br>(Herbst, 1784)<br>formerly: *Neliocarus faber*	MK892074 [new]<br>2350-JKR<br>658nt	ZFMK-TIS-24281<br>ZFMK-DNA-0169170920	Czech Republic, Moravia, Lanzhot, Pohansko, N48°41'27" E16°55'58", 153m, 14-Aug-2014, sweeping, leg. Krátky,J., det. Krátky,J.
Strophosoma faber<br>(Herbst, 1784)<br>formerly: *Neliocarus faber*	MK891304 [new]<br>1287-PST<br>658nt	ZFMK-TIS-4103<br>ZFMK-DNA-0100426189	Spain, Community of Madrid, NE of Montejo de la Sierra, Hayedo de Montejo, NE of Hiruela, Puerto de la Hiruela, N41°04'02" W03°28'28", 1478m, 02-Mai-2013, hand collecting, leg. Stüben,P. & Schütte,A., det. Stüben,P.
Strophosoma fulvicorne<br>(Walton, 1846)	MK891440 [new]<br>1503-PSP<br>658nt	ZFMK-TIS-3263<br>ZFMK-DNA-0155633326	Germany, Schleswig-Holstein (SH), Süderlügum, Lkr. Nordfriesland, N54°52'23" E08°55'48", 6m, 28-Aug-2012, *Calluna vulgaris*, inland dunes area, beating, leg. Sprick,P., det. Sprick,P.
Strophosoma melanogrammum<br>(Forster, 1771)	KC783995 [new]<br>263-PSP<br>658nt	ZFMK-TIS-2D100438528<br>ZFMK-DNA-0100439781	Germany, Lower Saxony (NI), Helstorfer Moor, Resse, Hannover region, N52°31'50" E09°36'35", 43m, 14-Mai-2011, *Salix aurita, Corylus avellana, Quercus robur*, edge of broad-leaved forest (east side), beating, leg. Sprick,P., det. Sprick,P.
Strophosoma melanogrammum<br>(Forster, 1771)	MK891408 [new]<br>1470-PSP<br>658nt	ZFMK-TIS-3230<br>ZFMK-DNA-0155633356	Germany, Lower Saxony (NI), Harz, St. Andreasberg, Jordanshöhe, N51°42'58" E10°32'03", 667m, 02-Jul-2012, *Sorbus aucuparia, Rubus idaeus*, tree group, beating, leg. Sprick,P., det. Sprick,P.
Strophosoma melanogrammum<br>(Forster, 1771)	MN614438 [new]<br>M-0051-StM<br>658nt	ZFMK-TIS-cM0051<br>ZFMK-DNA-0100400126	Germany, Rhineland-Palatinate (RLP), Moselle valley, 5 km E of Cochem, N50°08'46" E07°12'39", 279m, 15-Sep-2005, *Quercus, Carpinus*, leg. Stüben,P., det. Stüben,P.
Strophosoma melanogrammum<br>(Forster, 1771)	KC784247 [new]<br>IT-0014w<br>658nt	ZFMK-TIS-2D100446538<br>ZFMK-DNA-0100433812	Italy, Sardinia Isl., NE of Aritzo, Gennargentu, Mt. di Iscudu, N40°01'10" E09°16'39", 1511m, 30-Sep-2010, *alder, moss*, creek, leg. Stüben,P., det. Stüben,P.
Strophosoma melanogrammum<br>(Forster, 1771)	MK891005 [new]<br>899-CBR<br>658nt	ZFMK-TIS-3812<br>ZFMK-DNA-0100449721	Luxembourg, 10 km NE of Mersch, 2 km NE of Nommern, N49°48'15" E06°11'16", 340m, 01-Mai-2011, *Quercus*, beating, leg. Braunert,C., det. Braunert,C.
Strophosoma melanogrammum<br>(Forster, 1771)	MK891299 [new]<br>1275-PST<br>658nt	ZFMK-TIS-4091<br>ZFMK-DNA-0100426192	Spain, Community of Madrid, NE of Montejo de la Sierra, Hayedo de Montejo, Puerto de El Cardoso, N41°05'14" W03°29'21", 1386m, 02-Mai-2013, *Quercus*, sieving, leg. Stüben,P. & Schütte,A., det. Stüben,P.
Strophosoma sagitta<br>(Seidlitz, 1870)	MK891171 [new]<br>1082-JKR<br>658nt	ZFMK-TIS-3418<br>ZFMK-DNA-0100449615	Spain, Andalucia, Rociana del Condado, N37°21'01" W06°36'55", 150m, 18-Apr-2012, sweeping, leg. Krátky,J., det. Krátky,J.

**Name**<br>**Authority**<br>**Additional Information**	**GenBank Acc No (Ref.)**<br>**Specimen ID**<br>**Sequence Length**	**ZFMK Tissue ID**<br>**ZFMK DNA Sample ID**<br>**(SDEI DNA Sample ID)**	**Locality, GPS, Collection Date, Plant, Collector, Identifier**
Strophosoma sus<br>(Stephens, 1831)	KC784144 [new]<br>629-PSP<br>658nt	ZFMK-TIS-20412<br>ZFMK-DNA-0100448635	Germany, Lower Saxony (NI), Berkhof, Hannover region, N52°36'57" E09°43'51", 41m, 16-Aug-2011, *Calluna vulgaris*, pine forest, sandy wayside, beating, leg. Sprick,P., det. Sprick,P.
Strophosoma sus<br>(Stephens, 1831)	MK891441 [new]<br>1504-PSP<br>658nt	ZFMK-TIS-3264<br>ZFMK-DNA-0155633325	Germany, Schleswig-Holstein (SH), Süderlügum, Lkr. Nordfriesland, N54°52'23" E08°55'48", 6m, 28-Aug-2012, *Calluna vulgaris*, inland dunes area, beating, leg. Sprick,P., det. Sprick,P.
Stuebenius frivaldszkyi<br>(Kuthy, 1887)	MK891229 [new]<br>1149-JKR<br>658nt	ZFMK-TIS-3485<br>ZFMK-DNA-0100449559	Hungary, Pest megye, Gödöllö, Mariabesnyo, N47°34'48" E19°24'11", 190m, 13-Mai-2012, *Silene latofolia* ssp. *Alba*, beating, leg. Krátky,J., det. Krátky,J.
Styphloderes lindbergi<br>(Roudier, 1963)<br>formerly: *Parastyphloderes lindbergi*	MK892368 [40]<br>2811-PST<br>658nt	ZFMK-TIS-24092<br>ZFMK-DNA-FC17941523	Portugal, Madeira, Ilhas Desertas, Deserta Grande, Doca, ranger station, N32°30'49" W16°30'33", 23m, 22-Mrz-2015, under stone, collecting by hand, leg. Stüben,P., det. Stüben,P.
Styphloderes lindbergi<br>(Roudier, 1963)<br>formerly: *Parastyphloderes lindbergi*	MK892390 [40]<br>2855-PST<br>658nt	ZFMK-TIS-4250<br>ZFMK-DNA-FC17941511	Portugal, Madeira, Achadas da Cruz, N32°51'23" W17°12'41", 47m, 04-Dez-2015, beating, leg. Stüben,P. & Schütte,A., det. Stüben,P.
Synapion ebeninum<br>(Kirby, 1808)	KC784059 [new]<br>575-PSP<br>658nt	ZFMK-TIS-20360<br>ZFMK-DNA-0100448404	Germany, Lower Saxony (NI), Wesseln, Lkr. Hildesheim, Steinberg National Park, N52°04'59" E10°01'22", 157m, 26-Jun-2011, *Lotus corniculatus, Trifolium medium, Astragalus glycyphyllos*, beating, leg. Sprick,P., det. Sprick,P.
Synapion ebeninum<br>(Kirby, 1808)	MK891363 [new]<br>1414-JKR<br>658nt	ZFMK-TIS-4183<br>ZFMK-DNA-0155628519	Slovakia, Vysoke Tatry Mts., Dolina siedmich prame?ov, N49°13'38" E20°16'34", 1448m, 30-Jun-2013, sweeping, leg. Krátky,J., det. Krátky,J.
Tachyerges decoratus<br>(Germar, 1821)	MK891276 [new]<br>1223-JKR<br>658nt	ZFMK-TIS-3559<br>ZFMK-DNA-0100426061	Czech Republic, Bohemia, Velke Petrovice, N50°30'32" E16°12'42", 401m, 21-Okt-2012, *Salix, Alnus*, sieving, leg. Krátky,J., det. Krátky,J.
Tachyerges salicis<br>(Linnaeus, 1758)	MK891153 [new]<br>1060-PSP<br>658nt	ZFMK-TIS-3205<br>ZFMK-DNA-0100426794	Germany, Lower Saxony (NI), Langeoog, N53°45'05" E07°32'24", 3m, 16-Jun-2012, *Salix repens*, small dune valley, beating, leg. Sprick,P., det. Sprick,P.
Tachyerges salicis<br>(Linnaeus, 1758)	MK891400 [new]<br>1460-PSP<br>658nt	ZFMK-TIS-3220<br>ZFMK-DNA-0155633369	Germany, Lower Saxony (NI), Berkhof, Hannover region, N52°36'47" E09°43'57", 36m, 30-Jun-2012, *Salix aurita*, former gravel-pit, dipnet, leg. Sprick,P., det. Sprick,P.
Tachyerges stigma<br>(Germar, 1821)	MK891678 [new]<br>1894-PST<br>658nt	ZFMK-TIS-23999<br>ZFMK-DNA-0169168521	Germany, North Rhine-Westphalia (NRW), Elmpt, NSG "Elmpter Schwalmbruch", Venekotensee, N51°13'43" E06°06'59", 35m, 27-Aug-2014, *Salix*, beating, leg. Stüben,P., det. Stüben,P.
Tachyerges stigma<br>(Germar, 1821)	MK892214 [new]<br>2510-PSP<br>658nt	ZFMK-TIS-23482<br>ZFMK-DNA-0171661752	Germany, Lower Saxony (NI), Hannover region, Brelingen, N52°33'52" E09°41'09", 75m, 08-Mai-2014, *Salix viminalis*, beating, leg. Sprick,P., det. Sprick,P.
Taeniapion atlanticum<br>(Uyttenboogaart, 1935)<br>formerly: *Taeniapion urticarium atlanticum*	KC783748 [40]<br>36-PST<br>624nt	ZFMK-TIS-2D100447011<br>ZFMK-DNA-0100438016	Spain, Canary Islands, Gran Canaria, NE of Santa Brigida, Angostura valley, N28°03'14" W15°28'49", 485m, 31-Dez-2010, *Urtica*, beating, leg. Stüben,P., det. Stüben,P.
Taeniapion delicatulum<br>(Wollaston, 1857)	MK347550 [40]<br>2963-PST<br>658nt	(SDEI-DNA-2963-PST)	Spain, Canary Islands, El Hierro, El Golfo, Montana Colorada: Vivere Forestal, N27°44'04" W18°01'50", 979m, 12-Apr-2016, beating, leg. Stüben & Schütte, det. Stüben,P.

**Name**<br>**Authority**<br>**Additional Information**	**GenBank Acc No (Ref.)**<br>**Specimen ID**<br>**Sequence Length**	**ZFMK Tissue ID**<br>**ZFMK DNA Sample ID**<br>**(SDEI DNA Sample ID)**	**Locality, GPS, Collection Date, Plant, Collector, Identifier**
Taeniapion delicatulum<br>(Wollaston, 1857)	MK347553 [40]<br>2967-PST<br>658nt	(SDEI-DNA-2967-PST)	Spain, Canary Islands, El Hierro, S Sabinosa: Las Tabladas to Fuente Mecafete, N27°44'14" W18°05'05", 841m, 16-Apr-2016, beating, leg. Stüben & Schütte, det. Stüben,P.
Taeniapion delicatulum<br>(Wollaston, 1857)	MK892391 [40]<br>2867-PST<br>658nt	ZFMK-TIS-4256<br>ZFMK-DNA-0169166925	Spain, Canary Islands, El Hierro, W of Frontera, Pista al Derrabado, N27°44'23" W18°02'58", 929m, 11-Apr-2016, *Urtica*, beating, leg. Stüben,P. & Schütte,A., det. Stüben,P.
Taeniapion delicatulum<br>(Wollaston, 1857)	KC783742 [40]<br>21-PST<br>650nt	ZFMK-TIS-2D100447031<br>ZFMK-DNA-0100438103	Spain, Canary Islands, Gran Canaria, Moya, Barranco de los Tilos (upper section), N28°04'15" W15°36'27", 798m, 07-Dez-2010, *Urtica*, beating, leg. Stüben,P., det. Stüben,P.
Taeniapion delicatulum<br>(Wollaston, 1857)	KC783776 [40]<br>67-PST<br>645nt	ZFMK-TIS-2D100446980<br>ZFMK-DNA-0100437990	Spain, Canary Islands, La Gomera, Agulo – Las Rosas, N28°11'26" W17°12'6", 388m, 16-Dez-2010, *Urtica*, beating, leg. Stüben,P., det. Stüben,P.
Taeniapion delicatulum<br>(Wollaston, 1857)	MK891607 [40]<br>1691-PST<br>658nt	ZFMK-TIS-24409<br>ZFMK-DNA-0155622499	Spain, Canary Islands, La Palma, near San Isidro, N28°37'47" W17°48'02", 643m, 18-Dez-2013, *Forsskaolea*, beating, leg. Stüben,P., det. Stüben,P.
Taeniapion rufulum<br>(Wencker, 1864)	MK892409 [new]<br>161-PST<br>657nt	ZFMK-TIS-2D100440255<br>ZFMK-DNA-0100438143	Morocco, S of Berkane, Mts. des Beni Snassen, Vallee Zegzel, N34°49'19" W02°23'10" , 644m, 15-Mai-2011, *Urtica*, beating, leg. Stüben,P., det. Stüben,P./vid. Behne,L.
Taeniapion rufulum<br>(Wencker, 1864)	MK891308 [new]<br>1295-PST<br>658nt	ZFMK-TIS-4111<br>ZFMK-DNA-0100426181	Spain, Community of Madrid, S of La Serna del Monte, N41°01'28" W03°38'00", 1028m, 03-Mai-2013, *roadside vegetation*, beating, leg. Stüben,P. & Schütte,A., det. Stüben,P.
Taeniapion urticarium<br>(Herbst, 1784)	MK892025 [new]<br>2298-JKR<br>658nt	ZFMK-TIS-24229<br>ZFMK-DNA-0169170964	Austria, Kaernten, Feistritz ob Bleiburg, N46°32'17" E14°46'28", 702m, 28-Jun-2014, mountain forest, sieving, leg. Krátky,J., det. Krátky,J.
Taeniapion urticarium<br>(Herbst, 1784)	MK891779 [new]<br>2038-JKR<br>658nt	ZFMK-TIS-23678<br>ZFMK-DNA-0169170460	Czech Republic, Bohemia, Kosatky, N50°19'23" E14°39'58", 218m, 02-Mai-2014, *Urtica dioica*, sweeping, leg. Krátky,J., det. Krátky,J.
Taeniapion urticarium<br>(Herbst, 1784)	MK892100 [new]<br>2377-JKR<br>658nt	ZFMK-TIS-25954<br>ZFMK-DNA-0171597932	Czech Republic, Moravia, Znojmo, N48°51'05" E16°05'59", 250m, 23-Aug-2014, sweeping, leg. R. Stejskal, det. Stejskal,R.
Taeniapion urticarium<br>(Herbst, 1784)	KC784183 [new]<br>480-PST<br>658nt	ZFMK-TIS-20265<br>ZFMK-DNA-0100449354	Germany, Rhineland-Palatinate (RLP), Mosellus area, Kobern-Gondorf, "Sürzer Höfe", ruderal area, N50°20'20" E07°25'25", 330m, 30-Sep-2011, *Urtica dioica*, hand-collecting, leg. Stüben,P., det. Stüben,P.
Taeniapion urticarium<br>(Herbst, 1784)	KC784322 [new]<br>777-PST<br>658nt	ZFMK-TIS-3114<br>ZFMK-DNA-0100448107	Germany, North Rhine-Westphalia (NRW), Mönchengladbach-Neuwerk, N51°13'16" E06°26'25", 48m, 29-Apr-2012, *Urtica dioica*, beating, leg. Stüben,P., det. Stüben,P.
Taeniapion urticarium<br>(Herbst, 1784)	MK347574 [new]<br>3007-PST<br>658nt	(SDEI-DNA-3007-PST)	Germany, Rhineland-Palatinate (RLP), Mosel: Zummelhöhe (cave)near Leiwen, N49°48'53” E 6°54'41“, 265m, 06-Sep-2017, *Urtica*, leg. Stüben,P., det. Stüben,P.
Tanymecus palliatus<br>(Fabricius, 1787)	MK891773 [new]<br>2031-JKR<br>658nt	ZFMK-TIS-23671<br>ZFMK-DNA-0169170470	Czech Republic, Bohemia, Kosatky, N50°19'23" E14°39'58", 218m, 02-Mai-2014, *Silene latifolia ssp.alba*, collecting by hand, leg. Krátky,J., det. Krátky,J.
Tanymecus palliatus<br>(Fabricius, 1787)	KC784017 [new]<br>225-PSP<br>658nt	ZFMK-TIS-2D100439146<br>ZFMK-DNA-0100439818	Germany, Lower Saxony (NI), Lemmie, Hannover region, Gehrdener Berg, N52°17'44" E09°35'23", 100m, 22-Apr-2011, *Urtica dioica*, field edge, beating, leg. Sprick,P., det. Sprick,P.

**Name**<br>**Authority**<br>**Additional Information**	**GenBank Acc No (Ref.)**<br>**Specimen ID**<br>**Sequence Length**	**ZFMK Tissue ID**<br>**ZFMK DNA Sample ID**<br>**(SDEI DNA Sample ID)**	**Locality, GPS, Collection Date, Plant, Collector, Identifier**
Tanymecus palliatus<br>(Fabricius, 1787)	KC784223 [new]<br>409-RGO<br>658nt	ZFMK-TIS-20195<br>ZFMK-DNA-0100449427	Poland, Rudnik, N51°14'31.02" E22°32'22.92", 191m, 05-Jun-2011, leg. Gosik,R., det. Gosik,R.
Tanymecus palliatus<br>(Fabricius, 1787)	KC784226 [new]<br>416-RGO<br>631nt	ZFMK-TIS-20202<br>ZFMK-DNA-0100449434	Poland, Tarnogóra, N50°41'11.6" E23°07'13.4", 249m, 11-Jun-2011, leg. Gosik,R., det. Gosik,R.
Tanymecus palliatus<br>(Fabricius, 1787)	MK892238 [new]<br>2536-PSP<br>658nt	ZFMK-TIS-23508<br>ZFMK-DNA-0171661730	Slovakia, Nitra, W of Gbelce, N47°46'56" E18°27'37", 148m, 18-Mai-2014, *Cirsium arvense*, leg. Sprick,P., det. Sprick,P.
Tanysphyrus ater<br>Blatchley, 1928	MK891964 [new]<br>2233-JKR<br>658nt	ZFMK-TIS-23971<br>ZFMK-DNA-0169170561	Czech Republic, Bohemia, Brazec, N50°10'25" E13°02'16", 697m, 08-Jun-2014, leg. Benedikt,S., det. Benedikt,S.
Tanysphyrus lemnae<br>(Paykull, 1792)	MK892126 [new]<br>2404-JKR<br>658nt	ZFMK-TIS-25981<br>ZFMK-DNA-0171600633	Czech Republic, Moravia, Lanzhot, Pohansko, N48°41'27" E16°55'58", 153m, 14-Aug-2014, *Lemna* sp., sweeping, leg. Krátky,J., det. Krátky,J.
Tanysphyrus lemnae<br>(Paykull, 1792)	MK891436 [new]<br>1499-PSP<br>658nt	ZFMK-TIS-3259<br>ZFMK-DNA-0155633337	Germany, Lower Saxony (NI), Meitze - Hellendorf, Hannover region, N52°33'39" E09°44'36", 50m, 03-Aug-2012, *Lemna minor, Spirodela polyrhiza*, Handaufsammlung, leg. Sprick,P., det. Sprick,P.
Tanysphyrus lemnae<br>(Paykull, 1792)	KC784101 [new]<br>506-RST<br>658nt	ZFMK-TIS-20291<br>ZFMK-DNA-0100448464	Slovakia, Slovakia occ., 0.7 km SE of Dolne Zelenice, N48°22'13.95" E17°45'8.62", 135m, 29-Mai-2011, *Veronica* sp., beating, leg. Stejskal,R., det. Stejskal,R.
Tapeinotus sellatus<br>(Fabricius, 1794)	MK890850 [new]<br>374-JKR<br>658nt	ZFMK-TIS-20160<br>ZFMK-DNA-0100438909	Czech Republic, Bohemia or., Hrobice, N50°6'2.909" E15°46'50.867", 230m, 05-Jul-2011, *Lysimachia nummularia*, sweeping, leg. Krátky,J., det. Krátky,J.
Tapeinotus sellatus<br>(Fabricius, 1794)	MK891123 [new]<br>1028-PSP<br>658nt	ZFMK-TIS-3173<br>ZFMK-DNA-0100426831	Germany, Lower Saxony (NI), Hannover, Ricklingen, Leine bottomland W of Maschsee, N52°21'06" E09°44'10", 50m, 22-Mai-2012, *Lysimachia vulgaris*, dipnet, leg. Sprick,P., det. Sprick,P.
Taphrotopium sulcifrons<br>(Herbst, 1797)	MK892162 [new]<br>2453-PSP<br>658nt	ZFMK-TIS-23425<br>ZFMK-DNA-0171661820	Germany, Berlin (BE), Berlin-Tegel, N52°34'16.5" E13°16'58", 35m, 20-Okt-2013, *Artemisia campestris*, collecting by hand, leg. Sprick,P., det. Sprick,P.
Tatianaerhynchites aequatus<br>(Linnaeus, 1767)	KC784012 [new]<br>232-PSP<br>631nt	ZFMK-TIS-2D100438538<br>ZFMK-DNA-0100439811	Germany, Lower Saxony (NI), Hemmingen, Hannover region, N52°19'07" E09°44'02", 54m, 23-Apr-2011, *Crataegus monogyna*, fallow, beating, leg. Sprick,P., det. Sprick,P.
Tatianaerhynchites aequatus<br>(Linnaeus, 1767)	MK890939 [new]<br>808-PST<br>658nt	ZFMK-TIS-3721<br>ZFMK-DNA-0100413761	Germany, Rhineland-Palatinate (RLP), N of Treis-Karden, Brohl, N50°13'22" E07°16'27", 255m, 10-Jun-2012, roadside ditch, beating, leg. Stüben,P., det. Stüben,P.
Tatianaerhynchites aequatus<br>(Linnaeus, 1767)	MK892203 [new]<br>2498-PSP<br>658nt	ZFMK-TIS-23470<br>ZFMK-DNA-0171661775	Germany, Saxony-Anhalt (ST), Harz National Park, Ilsenburg, Ilsestein, N51°50'49" E10°39'40", 405m, 24-Apr-2014, *Sorbus aucuparia*, beating, leg. Sprick,P., det. Sprick,P.
Tatianaerhynchites aequatus<br>(Linnaeus, 1767)	KC784037 [new]<br>675-FBA<br>658nt	ZFMK-TIS-20454<br>ZFMK-DNA-0100448233	Greece, Peloponnese, Messinia, Mt. Taygetos, S of Marathos, N36°53'19" E22°17'45", 1000m, 21-Mai-2011, leg. Bahr,F., det. Bahr,F.
Tatianaerhynchites aequatus<br>(Linnaeus, 1767)	MK891767 [new]<br>2024-JKR<br>658nt	ZFMK-TIS-23664<br>ZFMK-DNA-0169170477	Italy, Sicilia Isl. (PA), Palermo, Bosco di Ficuzza, N37°51'50" E13°24'56", 995m, 22-Apr-2014, *Crataegus*, beating, leg. Krátky,J., det. Krátky,J.

**Name**<br>**Authority**<br>**Additional Information**	**GenBank Acc No (Ref.)**<br>**Specimen ID**<br>**Sequence Length**	**ZFMK Tissue ID**<br>**ZFMK DNA Sample ID**<br>**(SDEI DNA Sample ID)**	**Locality, GPS, Collection Date, Plant, Collector, Identifier**
Tatianaerhynchites aequatus<br>(Linnaeus, 1767)	MK890993 [new]<br>885-CBR<br>658nt	ZFMK-TIS-3798<br>ZFMK-DNA-0100449726	Luxembourg, 10 km E of Luxembourg, 2 km S of Mensdorf, N49°38'09" E06°17'48", 330m, 11-Apr-2011, *Prunus spinosa*, beating, leg. Braunert,C., det. Braunert,C.
Tatianaerhynchites aequatus<br>(Linnaeus, 1767)	MK891317 [new]<br>1313-PST<br>658nt	ZFMK-TIS-4129<br>ZFMK-DNA-0100426163	Spain, Malaga, SE of Antequera, Sierra de Camorolos, N36°57'02" W04°20'48", 907m, 05-Mai-2013, *Crataegus*, beating, leg. Stüben,P. & Schütte,A., det. Stüben,P.
Temnocerus longiceps<br>(C. G. Thomson, 1888)	MK891109 [new]<br>1014-PSP<br>658nt	ZFMK-TIS-3159<br>ZFMK-DNA-0100426222	Germany, Lower Saxony (NI), Berkhof, Hannover area, N52°36'47" E09°43'57", 36m, 10-Mai-2012, *Salix aurita*, Salix shrubs, beating, leg. Sprick,P., det. Sprick,P.
Temnocerus longiceps<br>(C. G. Thomson, 1888)	MK891435 [new]<br>1498-PSP<br>658nt	ZFMK-TIS-3258<br>ZFMK-DNA-0155633336	Germany, Saxony-Anhalt (ST), Harz National Park, Schierke, Feuersteinwiesen, N51°45'23" E10°41'33", 595m, 01-Aug-2012, *Salix aurita*, beating, leg. Sprick,P., det. Sprick,P.
Temnocerus nanus<br>(Paykull, 1792)	MK891135 [new]<br>1040-PSP<br>658nt	ZFMK-TIS-3185<br>ZFMK-DNA-0100426917	Germany, Lower Saxony (NI), near Hannover, Berkhof, N52°36'45" E09°43'56", 35m, 26-Mai-2012, *Betula pendula*, former gravel pit, beating, leg. Sprick,P., det. Sprick,P.
Temnocerus nanus<br>(Paykull, 1792)	MK891473 [new]<br>1541-PSP<br>658nt	ZFMK-TIS-3301<br>ZFMK-DNA-0155633283	Germany, Saxony-Anhalt (ST), Harz National Park, Ilsenburg, Meineberg, N51°51'26" E10°39'52", 413m, 28-Jun-2013, *Betula pendula*, birch trees succession, beating, leg. Sprick,P., det. Sprick,P.
Temnocerus tomentosus<br>(Gyllenhal, 1839)	MK892301 [new]<br>2602-PSP<br>658nt	ZFMK-TIS-23576<br>ZFMK-DNA-0171661957	Germany, Bavaria (BY), Unterfranken, Ebern, N50°05'00" E10°45'40", 320m, 14-Jun-2014, *Salix caprea*, leg. Sprick,P., det. Sprick,P.
Temnorhinus conicirostris<br>(Olivier, 1807)	MK890811 [new]<br>152-PST<br>658nt	ZFMK-TIS-2D100438602<br>ZFMK-DNA-0100438360	Morocco, NW of Berkane, Oued Moulouya, N35°00'03" W02°27'30" , 15m, 14-Mai-2011, *thistle*, beating, leg. Stüben,P., det. Stüben,P./vid. Behne,L.
Thamiocolus garajonay<br>Stüben, 2014<br>**Paratype (DNAtype)**	KC783774 [13]<br>65-PST_2G2010<br>658nt	ZFMK-TIS-2D100446978<br>ZFMK-DNA-0100437966	Spain, Canary Islands, La Gomera, E of Hermigua, El Palmar, NP Mojana, N28°09'26“ W17°09'47“, 627m, 14-Dez-2010, *Sideritis*, beating, leg. Stüben,P., det. Stüben,P.
Thamiocolus garajonay<br>Stüben, 2014<br>**Paratype (DNAtype)**	KM433746 [13]<br>1657-PST_4701<br>658nt	ZFMK-TIS-4701<br>ZFMK-DNA-0155630472	Spain, Canary Islands, La Gomera, S of Hermigua, Tunel de la Cumbre, Agua Jilva, N28°07'53" W17°10'53", 539m, 03-Dez-2012, *Sideritis*, beating, leg. Stüben,P., det. Stüben,P.
Thamiocolus garajonay<br>Stüben, 2014<br>**Paratype (DNAtype)**	KM433749 [13]<br>1675-PST_24393<br>658nt	ZFMK-TIS-24393<br>ZFMK-DNA-0155622504	Spain, Canary Islands, La Gomera, Parque Natural de Majona, above Casas del Palmar, N28°09'28" W17°09'41", 641m, 07-Dez-2013, *Sideritis*, beating, leg. Stüben,P., det. Stüben,P.
Thamiocolus garajonay<br>Stüben, 2014<br>**Paratype (DNAtype)**	KM433750 [13]<br>1810-PST_4077<br>658nt	ZFMK-TIS-4077<br>ZFMK-DNA-0171624053	Spain, Canary Islands, La Gomera, above Agulo, barrier lake, N28°11'04" W17°11'58", 496m, 15-Dez-2012, *Sideritis*, beating, leg. Stüben,P., det. Stüben,P.
Thamiocolus grancanariensis<br>Stüben & Schütte, 2014<br>**Paratype (DNAtype)**	KC783752 [13]<br>40-PST_20GC2011<br>658nt	ZFMK-TIS-2D100447002<br>ZFMK-DNA-0100437934	Spain, Canary Islands, Gran Canaria, S of La Culata, Llanos de la Pez, N27°57'54" W15°35'15", 1653m, 27-Dez-2010, *Sideritis*, beating, leg. Stüben,P., det. Stüben,P.
Thamiocolus niveus<br>(Chevrolat, 1859)	KM433744 [13]<br>1329-PST<br>658nt	ZFMK-TIS-4145<br>ZFMK-DNA-0100426150	Spain, Malaga, S of Antequera, Sierra de Chimenea, El Torcal, N36°57'01" W04°33'13", 1198m, 10-Mai-2013, *rocks*, sieving, leg. Stüben,P. & Schütte,A., det. Stüben,P.
Thamiocolus nubeculosus<br>(Gyllenhal, 1837)	MK891840 [new]<br>2101-JKR<br>658nt	ZFMK-TIS-23741<br>ZFMK-DNA-0169169632	Czech Republic, Moravia, Cejc, PR Spidlaky, N48°55'23.5" E16°57'21", 220m, 10-Mai-2014, *Phlomis tuberosa*, sweeping, leg. Krátky,J., det. Krátky,J.

**Name**<br>**Authority**<br>**Additional Information**	**GenBank Acc No (Ref.)**<br>**Specimen ID**<br>**Sequence Length**	**ZFMK Tissue ID**<br>**ZFMK DNA Sample ID**<br>**(SDEI DNA Sample ID)**	**Locality, GPS, Collection Date, Plant, Collector, Identifier**
Thamiocolus signatus<br>(Gyllenhal, 1837)	KC784098 [13]<br>518-RST<br>658nt	ZFMK-TIS-20303<br>ZFMK-DNA-0100448461	Czech Republic, Bohemia bor., Radobyl Nature Monument, 2.5 km W of Litomerice, N50°31'47.88" E14°5'29.15", 350m, 06-Jul-2011, *Stachys recta*, beating, leg. Stejskal,R., det. Stejskal,R.
Thamiocolus signatus<br>(Gyllenhal, 1837)	MK891841 [new]<br>2103-JKR<br>658nt	ZFMK-TIS-23743<br>ZFMK-DNA-0169169630	Czech Republic, Moravia, Cejc, PR Spidlaky, N48°55'23.5" E16°57'21", 220m, 10-Mai-2014, *Stachys recta*, sweeping, leg. Krátky,J., det. Krátky,J.
Thamiocolus sinapis<br>(Desbrochers des Loges, 1893)	KC783848 [13]<br>175-PST<br>658nt	ZFMK-TIS-2D100440252<br>ZFMK-DNA-0100438140	Morocco, Atlas Mts., E of Ifrane, N33°27'26" W04°51'24", 1566m, 18-Mai-2011, *Micromeria*, beating, leg. Stüben,P., det. Stüben,P./vid. Behne,L.
Thamiocolus viduatus<br>(Gyllenhal, 1813)	KC783911 [13]<br>384-JKR<br>621nt	ZFMK-TIS-20170<br>ZFMK-DNA-0100438702	Poland, Wielkopolskie, Rogalin env., N52°14'25.971" E16°54'20.152", 58m, 09-Jul-2011, *Stachys palustris*, beating, leg. Krátky,J., det. Krátky,J.
Thamiocolus viduatus<br>(Gyllenhal, 1813)	MK892058 [new]<br>2334-JKR<br>658nt	ZFMK-TIS-24265<br>ZFMK-DNA-0169170931	Slovakia, Povazska Bystrica, Bodina, N49°06'46" E18°33'25", 450m, 13-Jul-2014, *Stachys palustris*, sweeping, leg. Krátky,J., det. Krátky,J.
Thamiocolus virgatus<br>(Gyllenhal, 1837)	KM433745 [13]<br>1600-JKR<br>658nt	ZFMK-TIS-3648<br>ZFMK-DNA-0155635629	Czech Republic, Moravia, Cejkovice - Spidlaky, N48°54'57" E16°57'44", 210m, 01-Mai-2013, *Phlomis tuberosa*, sweeping, leg. Krátky,J., det. Krátky,J.
Thamiocolus virgatus<br>(Gyllenhal, 1837)	MK891836 [new]<br>2097-JKR<br>658nt	ZFMK-TIS-23737<br>ZFMK-DNA-0169169636	Czech Republic, Moravia, Krumvir, PR Louky pod Kumstatem, N48°59'36" E16°55'20.5", 211m, 09-Mai-2014, *Phlomis tuberosa*, sweeping, leg. Krátky,J., det. Krátky,J.
Thamiocolus wollastoni<br>(Uyttenboogaart, 1930)	KM433738 [13]<br>714-PST<br>658nt	ZFMK-TIS-3051<br>ZFMK-DNA-0100448173	Spain, Canary Islands, Tenerife, Anaga Mts., Taborno, N28°33'19" W16°15'54", 626m, 11-Jan-2012, *Sideritis macrostachys*, beating, leg. Stüben,P., det. Stüben,P.
Thamiocolus wollastoni<br>(Uyttenboogaart, 1930)	KM433739 [13]<br>720-PST<br>658nt	ZFMK-TIS-3057<br>ZFMK-DNA-0100448167	Spain, Canary Islands, Tenerife, 4 km W of Buenavista del Norte, N28°21'55" W16°52'59", 155m, 15-Jan-2012, *Sideritis* cf. *cretica*, beating, leg. Stüben,P., det. Stüben,P.
Thamiocolus wollastoni<br>(Uyttenboogaart, 1930)	KM433747 [13]<br>1661-PST<br>658nt	ZFMK-TIS-4705<br>ZFMK-DNA-0155630476	Spain, Canary Islands, Tenerife, N of Vilaflor, Barranco de Eris de Carnero, N28°10'23" W16°37'12", 1656m, 23-Feb-2012, beating, leg. Stüben,P., det. Stüben,P.
Thamiocolus wollastoni<br>(Uyttenboogaart, 1930)	KM433748 [13]<br>685-PST<br>658nt	ZFMK-TIS-3022<br>ZFMK-DNA-0155630428	Spain, Canary Islands, Tenerife, Anaga Mts., Roque Negro, N28°32'08" W16°15'10", 644m, 12-Feb-2012, *Sideritis* cf. *macrostachys, Echium plantagineum, Aeonium, Sonchus*, next to road (at night), leg. Stüben,P. & Schütte,A., det. Stüben,P.
Thryogenes nereis<br>(Paykull, 1800)	MK890832 [new]<br>282-PSP<br>658nt	ZFMK-TIS-2D100438505<br>ZFMK-DNA-0100439752	Germany, Saxony-Anhalt (ST), Drömling, Mannhausen, N52°25'30" E11°12'44", 74m, 25-Mai-2011, *Eleocharis palustris*, body of standing water shore, dipnet, leg. Sprick,P., det. Sprick,P.
Thryogenes scirrhosus<br>(Gyllenhal, 1835)	MK891957 [new]<br>2226-JKR<br>658nt	ZFMK-TIS-23964<br>ZFMK-DNA-0169170554	Czech Republic, Bohemia, Hradec Kralove, Trebes, N50°11'33" E15°49'15", 227m, 29-Sep-2014, sieving, leg. Krátky,J., det. Krátky,J.
Torneuma alexi<br>Stüben, 2018<br>**Holotype* (DNAtype)**	KX246401 [28]<br>2861-PST_ADN567<br>658nt	ZFMK-TIS-ADN567	Spain, Canary Islands, Gran Canaria, Barranco de Los Rios, Los Berrazales, N28°04'08" W15°39'22.5", 503m, 30-Dez-2013, MSS trap, leg. López,H., det. Stüben,P.<br>Note: holotype was used for non-destructive DNA isolation.
Torneuma alexi<br>Stüben, 2018<br>**Paratype (DNAtype)**	KX246405 [28]<br>2865-PST_ADN573<br>658nt	ZFMK-TIS-ADN573	Spain, Canary Islands, Gran Canaria, Barranco de Los Rios, Los Berrazales, N28°04'08" W15°39'23", 499m, 30-Dez-2013, MSS trap, leg. López,H., det. Stüben,P.

**Name**<br>**Authority**<br>**Additional Information**	**GenBank Acc No (Ref.)**<br>**Specimen ID**<br>**Sequence Length**	**ZFMK Tissue ID**<br>**ZFMK DNA Sample ID**<br>**(SDEI DNA Sample ID)**	**Locality, GPS, Collection Date, Plant, Collector, Identifier**
Torneuma alexi<br>Stüben, 2018<br>**Paratype (DNAtype)**	KX246406 [28]<br>2866-PST_ADN574<br>658nt	ZFMK-TIS-ADN574	Spain, Canary Islands, Gran Canaria, Barranco de Los Rios, Los Berrazales, N28°04'08" W15°39'23", 499m, 30-Dez-2013, MSS trap, leg. López,H., det. Stüben,P.
Torneuma aphroditae<br>(Germann & Stüben, 2006)<br>formerly: *Paratorneuma aphroditae*	JX181783 [09]<br>ES1014<br>658nt	ZFMK-TIS-cES1014<br>ZFMK-DNA-0112704652	Spain, Canary Islands, La Gomera, S of Hermigua, El Cedro, Meriga, 28°09'12"N 17°14'13"W, 829m, 28-Dez-2009, *Laurisilva, Persea indica*, stream valley, leg. Stüben,P., det. Stüben,P.
Torneuma aphroditae<br>(Germann & Stüben, 2006)<br>formerly: *Paratorneuma aphroditae*	KX246402 [28]<br>2862-PST<br>658nt	ZFMK-TIS-ADN569	Spain, Canary Islands, La Gomera, Parque Nacional de Garajonay, 500m SW of Eremita de Nuestra Senora de Lourdes, Campamento viejo, N28°07'11.4" W17°13'33.2", 991m, 17-Nov-2013, MSS trap, leg. Oromí,P., det. Stüben,P.
Torneuma aphroditae<br>(Germann & Stüben, 2006)<br>formerly: *Paratorneuma aphroditae*	KX246403 [28]<br>2863-PST<br>658nt	ZFMK-TIS-ADN570	Spain, Canary Islands, La Gomera, Parque Nacional de Garajonay, 500m SW of Eremita de Nuestra Senora de Lourdes, Campamento viejo, N28°07'11.4" W17°13'33.2", 991m, 17-Nov-2013, MSS trap, leg. Oromí,P., det. Stüben,P.
Torneuma baeticum<br>Stüben, 2007	KP776630 [28]<br>GI1044<br>658nt	ZFMK-TIS-cGI1044<br>ZFMK-DNA-0112704634	Uk, Gibraltar, Engineer Road, Upper Rock, 36°07'26N 5°20'50"W, 160m, 03-Feb-2010, *Ficus carica*, soil under Ficus carica, leg. Perez & Bensusan, det. Perez and Bensusan
Torneuma cadizense<br>Stüben, 2016<br>**Paratype (DNAtype)**	KC783806 [28]<br>100-PST_div<br>657nt	ZFMK-DNA-0100417927	Spain, Cadiz, La Linea, Sierra Carbonera, Puerto Higueron, Cordel Ruta Verde cribando Bajo Coscoja, N36°13'17" W5°20'60" , 155m, 28-Sep-2010, *Quercus coceifera*, sieving, leg. Torres,J. L., det. Stüben,P.
Torneuma caecum<br>Wollaston, 1860	FJ716559 [01]<br>P-523-cae<br>658nt	ZFMK-TIS-cP523<br>ZFMK-DNA-0100400286	Portugal, Madeira, 1 km S of Curral das Freiras, Seara Velha, N32°42'35" W16°58'17", 384m, 20-Mrz-2008, *Ficus carica*, leg. Astrin,J. & Stüben,P., det. Astrin,J. and Stüben,P.
Torneuma cf. *bensusani*	KP776632 [28]<br>ES1043<br>658nt	ZFMK-TIS-cES1043<br>ZFMK-DNA-0112704633	Spain, Ceuta, Mirador Isabel II, 35°53'33"N 5°21'47"W, 207m, 08-Mai-2010, *Asphodelus* sp., leg. Bensusan & Guillem, det. Bensusan and Guillem
Torneuma cf. *desilvai*	MK347542 [40]<br>2952-PST<br>658nt	(SDEI-DNA-2952-PST)	Portugal, Madeira, Ribeira da Janela, "Fanal", 4 km S, N32°48'45" W17°08'58", 1075m, 31-Mrz-2017, *Ocotea foetens (small roots)*, leg. Pelikán, det. Stüben,P.
Torneuma deplanatum deplanatum<br>(Hampe, 1864)<br>formerly: *Typhloporus*	GU987874 [01]<br>KO-0371-dep<br>658nt	ZFMK-TIS-cKO0371<br>ZFMK-DNA-0100400462	Greece, Korfu Isl., 18 km N of Kerkyra, Loutses, Megali Grava, N39°46'38" E19°53'21", 479m, 23-Sep-2007, *Quercus pubescens, Laurus nobilis, Asphodelus*, cave floor, under stones, leg. Stüben,P., det. Stüben,P.
Torneuma deplanatum deplanatum<br>(Hampe, 1864)<br>formerly: *Typhloporus*	EU286518 [01]<br>I-0305-dep<br>658nt	ZFMK-TIS-cI0305<br>ZFMK-DNA-0100400382	Italy, Sicilia Isl. (PA), 8 km S of Carini, W of M. Gibilmesi, N38°04'03" E13°11'37", 539m, 10-Okt-2006, *Asphodelus albus*, limestone, leg. Stüben,P., det. Stüben,P.
Torneuma deplanatum deplanatum<br>(Hampe, 1864)<br>formerly: *Typhloporus*	GU987789 [01]<br>I-0174-dep<br>658nt	ZFMK-TIS-cI0174<br>ZFMK-DNA-0100400681	Italy, Sicilia Isl. (PA), 6 km SW of Carini, Montagna Longa, N38°07'10" E13°08'31", 647m, 09-Okt-2006, *Asphodelus albus*, leg. Stüben,P., det. Stüben,P.

**Name**<br>**Authority**<br>**Additional Information**	**GenBank Acc No (Ref.)**<br>**Specimen ID**<br>**Sequence Length**	**ZFMK Tissue ID**<br>**ZFMK DNA Sample ID**<br>**(SDEI DNA Sample ID)**	**Locality, GPS, Collection Date, Plant, Collector, Identifier**
Torneuma deplanatum deplanatum<br>(Hampe, 1864)<br>formerly: *Typhloporus*	GU987790 [01]<br>I-0175-dep<br>658nt	ZFMK-TIS-cI0175<br>ZFMK-DNA-0100400682	Italy, Sicilia Isl. (PA), 10 km W of Palermo, Monte Cuccio, Pass Torretta, N38°07'47" E13°14'55", 577m, 05-Okt-2006, *Asphodelus albus*, limestone: below stones, leg. Stüben,P., det. Stüben,P.
Torneuma desilvai<br>G. Osella & Zuppa, 1998	FJ716547 [01]<br>P-529-des<br>658nt	ZFMK-TIS-cP529<br>ZFMK-DNA-0100400638	Portugal, Madeira, São Vicent, N32°47'51" W17°02'33", 85m, 22-Mrz-2008, *Laurus azorica*, leg. Astrin,J. & Stüben,P., det. Astrin,J. and Stüben,P.
Torneuma feloi<br>(Stüben, 2007)<br>formerly: *Paratyphloporus feloi*	KX246399 [28]<br>2857-PST<br>658nt	ZFMK-TIS-4252<br>ZFMK-DNA-FC17941527	Spain, Canary Islands, Tenerife, Balo Blanco (Lavada de Tierra), N28°21'58" W16°35'14", 660m, 15-Okt-2009, washing soil , leg. Oromí,P. & López,H, det. Stüben,P.
Torneuma feloi<br>(Stüben, 2007)<br>formerly: *Paratyphloporus feloi*	KX246400 [28]<br>2859-PST<br>658nt	ZFMK-TIS-4254<br>ZFMK-DNA-FC17941543	Spain, Canary Islands, Tenerife, Balo Blanco (Lavada de Tierra), N28°21'58" W16°35'14", 660m, 10-Feb-2014, MSS trap, leg. Oromí,P. & López,H, det. Stüben,P.
Torneuma isambertoi<br>Stüben, 2016<br>**Paratype (DNAtype)**	KU170192 [28]<br>2790-PST_26144<br>658nt	ZFMK-TIS-26144<br>ZFMK-DNA-0169166963	Portugal, Madeira, Paul do Mar, N32°45'34" W17°13'43", 62m, 04-Apr-2015, *Ficus carica, E. piscatorial*, banana terraces, sieving, leg. Stüben,P., det. Stüben,P.
Torneuma isambertoi<br>Stüben, 2016<br>**Paratype (DNAtype)**	KU170193 [28]<br>2791-PST_26145<br>658nt	ZFMK-TIS-26145<br>ZFMK-DNA-0169166951	Portugal, Madeira, Paul do Mar, N32°45'34" W17°13'43", 62m, 04-Apr-2015, *Ficus carica, E. piscatorial*, banana terraces, sieving, leg. Stüben,P., det. Stüben,P.
Torneuma karamani<br>(Formánek, 1912)<br>formerly: *Paratyphloporus karamani*	EU286506 [01]<br>HR-0312-kar<br>658nt	ZFMK-TIS-cHR0312<br>ZFMK-DNA-0100400108	Croatia, Dalmatia, 21 km E of Split, Mosor Mts., N of Omis, Gata, N43°27'59" E16°41'40", 280m, 02-Jul-2007, *Olea, Quercus, Carpinus*, limestone, leg. Stüben,P., det. Stüben,P.
Torneuma korwitzi<br>Stüben, 2015<br>**Paratype (DNAtype)**	KP776628 [21]<br>1860-PST_26063<br>658nt	ZFMK-TIS-26063<br>ZFMK-DNA-0171606097	Portugal, Madeira, Paul do Mar, N32°45'34" W17°13'43", 62m, 02-Jul-2014, *Ficus*, sieving, leg. Stüben,P., det. Stüben,P.
Torneuma korwitzi<br>Stüben, 2015	KP776629 [21]<br>1864-PST<br>658nt	ZFMK-TIS-26067<br>ZFMK-DNA-0171606101	Portugal, Madeira, Paul do Mar, N32°45'34" W17°13'43", 62m, 02-Jul-2014, *Ficus*, sieving, leg. Stüben,P., det. Stüben,P.
Torneuma maderense<br>Stüben, 2002	FJ716558 [01]<br>P-522-mad<br>658nt	ZFMK-TIS-cP522<br>ZFMK-DNA-0100400291	Portugal, Madeira, 3.5 km W of Porto Moniz, Santa Madalena, N32°51'25" W17°12'18", 296m, 19-Mrz-2008, *Ficus carica*, leg. Astrin,J. & Stüben,P., det. Astrin,J. and Stüben,P.
Torneuma maderense<br>Stüben, 2002	KP869115 [21]<br>1842-PST<br>658nt	ZFMK-TIS-26045<br>ZFMK-DNA-0171606127	Portugal, Madeira, W of Porto Moniz, Santa Madalena, Rib. do Tristao, N32°51'20" W17°12'21", 154m, 29-Jun-2014, *Moraceae*, sieving, leg. Stüben,P., det. Stüben,P.
Torneuma mesegueri lineaensis<br>Stüben, 2009	GU988047 [01]<br>E-788-lin<br>658nt	ZFMK-TIS-cE788<br>ZFMK-DNA-0100405063	Spain, Cadiz, La Linea, Sierra Carbonera, between Puerto Higuerón and Zabal Alto, N36°12' W05°19', 09-Okt-2008, *Pistacia lentiscus*, leg. Torres,J. L., det. Torres,J. L.
Torneuma orbatum<br>(Wollaston, 1865)<br>formerly: *Paratorneuma orbatum*	FJ716580 [01]<br>E-733-orb<br>658nt	ZFMK-TIS-cE733<br>ZFMK-DNA-0100404822	Spain, Canary Islands, La Gomera, Hermigua, Ibo Alfaro, N28°09'58" W17°12'11", 255m, 09-Okt-2008, *Persea*, terraces, leg. Astrin,J. & Stüben,P., det. Stüben,P.

**Name**<br>**Authority**<br>**Additional Information**	**GenBank Acc No (Ref.)**<br>**Specimen ID**<br>**Sequence Length**	**ZFMK Tissue ID**<br>**ZFMK DNA Sample ID**<br>**(SDEI DNA Sample ID)**	**Locality, GPS, Collection Date, Plant, Collector, Identifier**
Torneuma orbatum<br>(Wollaston, 1865)<br>formerly: *Paratorneuma orbatum*	KP776634 [28]<br>ES1003<br>658nt	ZFMK-TIS-cES1003<br>ZFMK-DNA-0112704666	Spain, Canary Islands, La Gomera, S of Hermigua, El Cedro, behind Acevinos, 28°08'02"N 17°13'44"W, 890m, 04-Dez-2009, *Persea indica*, Laurisilva, leg. Stüben,P., det. Stüben,P.
Torneuma orbatum<br>(Wollaston, 1865)<br>formerly: *Paratorneuma orbatum*	KX246404 [28]<br>2864-PST<br>606nt	ZFMK-TIS-ADN571	Spain, Canary Islands, La Gomera, Parque Nacional de Garajonay, 500m E of Eremita de Nuestra Senora de Lourdes, Reventón Oscuro **(type locality)**, N28°07'27" W17°12'58.5", 1071m, 16-Nov-2013, MSS trap, leg. Oromí,P., det. Stüben,P.
Torneuma picocasteloense<br>Stüben, 2002	FJ716552 [01]<br>P-535-pic<br>658nt	ZFMK-TIS-cP535<br>ZFMK-DNA-0100400629	Portugal, Madeira, Porto Santo Isl., 2.5 km N of Vila Baleira, Pico Castelo - Pico do Facho, N33°04'27" W16°19'25", 146m, 26-Mrz-2008, *Cynara cardunculus*, willow, leg. Astrin,J. & Stüben,P., det. Astrin,J. and Stüben,P.
Torneuma sp.n.	GU988048 [01]<br>E-758-TOR<br>658nt	ZFMK-TIS-cE758<br>ZFMK-DNA-0100405061	Spain, Malaga, E of Malaga, Macharaviaya, N36°45'54" W04°12'47", 218m, 06-Jan-2009, *Olea europaea, Ulex*, depth sieving (german: Tiefengesiebe), leg. Stüben,P., det. Stüben,P., Note: only female specimen available.
Tournotaris bimaculata<br>(Fabricius, 1787)	MK891395 [new]<br>1455-PSP<br>658nt	ZFMK-TIS-3215<br>ZFMK-DNA-0155633374	Germany, Lower Saxony (NI), Hannover, Stöcken, N52°24'16" E09°39'06", 44m, 21-Jun-2012, *Phalaris arundinacea, Elymus repens*, beating, leg. Sprick,P., det. Sprick,P.
Trachodes hispidus<br>(Linnaeus, 1758)	MK890918 [new]<br>786-PST<br>658nt	ZFMK-TIS-3699<br>ZFMK-DNA-0100414305	Germany, Rhineland-Palatinate (RLP), E of Treis-Karden near Pommern, Fellerbachtal (Feller river valley), N50°10'10" E07°13'38", 85m, 09-Jun-2012, rivulet, beating, leg. Stüben,P., det. Stüben,P.
Trachodes hispidus<br>(Linnaeus, 1758)	MK892216 [new]<br>2512-PSP<br>658nt	ZFMK-TIS-23484<br>ZFMK-DNA-0171661754	Germany, Lower Saxony (NI), Harz National Park, NW of Herzberg, Mühlenberg, N51°40'45" E10°19'10", 262m, 12-Mai-2014, *Quercus robur*, leg. Sprick,P., det. Sprick,P.
Trachodes hispidus<br>(Linnaeus, 1758)	MK890884 [new]<br>526-RST<br>658nt	ZFMK-TIS-20311<br>ZFMK-DNA-0100448453	Romania, Caras-Severin, 4.8 km NE of Moldova Nou, N44°44'59.78" E21°43'18.12", 300m, 28-Jul-2011, beating, leg. Stejskal,R., det. Stejskal,R.
Trachyphloeus alternans<br>Gyllenhal, 1834	MK891516 [new]<br>1603-JKR<br>658nt	ZFMK-TIS-3651<br>ZFMK-DNA-0155635626	Czech Republic, Moravia, Cejc - Spidlaky, N48°55'47" E16°58'03", 184m, 01-Mai-2013, sieving, leg. Krátky,J., det. Krátky,J.
Trachyphloeus alternans<br>Gyllenhal, 1834	MK891784 [new]<br>2043-JKR<br>658nt	ZFMK-TIS-23683<br>ZFMK-DNA-0169170465	Czech Republic, Bohemia, Kosatky, N50°19'23" E14°39'58", 218m, 02-Mai-2014, sieving, leg. Krátky,J., det. Krátky,J.
Trachyphloeus alternans<br>Gyllenhal, 1834	MK892096 [new]<br>2373-JKR<br>658nt	ZFMK-TIS-25950<br>ZFMK-DNA-0171600595	Czech Republic, Moravia, Znojmo, N48°51'05" E16°05'59", 250m, 23-Aug-2014, sweeping, leg. R. Stejskal, det. Stejskal,R.
Trachyphloeus laticollis<br>Boheman, 1842	MK891714 [40]<br>1941-PST<br>658nt	ZFMK-TIS-24020<br>ZFMK-DNA-0169170419	Portugal, Madeira, Faial, coast, N32°47'37" W16°50'57", 26m, 04-Okt-2014, sieving, leg. Stüben,P., det. Stüben,P.
Trachyphloeus laticollis<br>Boheman, 1842	KC784280 [40]<br>695-PST<br>658nt	ZFMK-TIS-3032<br>ZFMK-DNA-0100448192	Spain, Canary Islands, Tenerife, Santa Barbara, N28°21'37" W16°41'07", 558m, 02-Jan-2012, hand-collection on house wall, leg. Stüben,P., det. Stüben,P.
Trachyphloeus parallelus<br>Seidlitz, 1868	MK891515 [new]<br>1602-JKR<br>658nt	ZFMK-TIS-3650<br>ZFMK-DNA-0155635627	Czech Republic, Moravia, Cejc - Spidlaky, N48°55'47" E16°58'03", 184m, 01-Mai-2013, sieving, leg. Krátky,J., det. Krátky,J.

**Name**<br>**Authority**<br>**Additional Information**	**GenBank Acc No (Ref.)**<br>**Specimen ID**<br>**Sequence Length**	**ZFMK Tissue ID**<br>**ZFMK DNA Sample ID**<br>**(SDEI DNA Sample ID)**	**Locality, GPS, Collection Date, Plant, Collector, Identifier**
Trachyphloeus rectus<br>C. G. Thomson, 1865	MK891822 [new]<br>2082-JKR<br>658nt	ZFMK-TIS-23722<br>ZFMK-DNA-0169169640	Czech Republic, Bohemia, Predhradi, N49°49'56" E16°02'33", 427m, 08-Mai-2014, sieving, leg. Krátky,J., det. Krátky,J.
Trachyphloeus rectus<br>C. G. Thomson, 1865	KC784190 [new]<br>471-PST<br>658nt	ZFMK-TIS-20256<br>ZFMK-DNA-0100449368	Germany, Rhineland-Palatinate (RLP), Mosellus valley "Backesberg" near Kobern-Gondorf, rock slope at railway embankment, N50°17'24" E07°27'27", 103m, 30-Sep-2011, *herbaceous vegetation*, sieving, leg. Stüben,P., det. Borovec,R.
Trachyphloeus rectus<br>C. G. Thomson, 1865	MN614440 [new]<br>E-756-Trec<br>658nt	ZFMK-TIS-cE756<br>ZFMK-DNA-0100405076	Germany, Rhineland-Palatinate (RLP), Altenahr,Teufelsloch, N50°30'37" E06°59'31", 267m, 26-Okt-2008, *Genista pilosa, Fagus*, leg. Stüben,P., det. Stüben,P.
Trachyphloeus rectus<br>C. G. Thomson, 1865	MN614441 [new]<br>E-757-Trec<br>658nt	ZFMK-TIS-cE757<br>ZFMK-DNA-0100405077	Germany, Rhineland-Palatinate (RLP), Bad Neuenahr-Ahrweiler, Marienthal, Altenahr, Teufelsloch, N50°32'16" E07°03'24", 175m, 26-Okt-2008, *Genista pilosa, Cytisus scoparius*, leg. Stüben,P., det. Stüben,P.
Trachyphloeus spinimanus<br>*incertae sedis*<br>Germar, 1824	MK891517 [new]<br>1604-JKR<br>658nt	ZFMK-TIS-3652<br>ZFMK-DNA-0155635625	Czech Republic, Moravia, Cejc - Spidlaky, N48°55'47" E16°58'03", 184m, 01-Mai-2013, sieving, leg. Krátky,J., det. Krátky,J.
Trachyphloeus spinimanus<br>Germar, 1824	MK891788 [new]<br>2047-JKR<br>658nt	ZFMK-TIS-23687<br>ZFMK-DNA-0169170454	Czech Republic, Bohemia, Kosatky, N50°19'23" E14°39'58", 218m, 02-Mai-2014, sieving, leg. Krátky,J., det. Krátky,J.
Trachyphloeus spinimanus<br>Germar, 1824	MK892115 [40]<br>2392-JKR<br>658nt	ZFMK-TIS-25969<br>ZFMK-DNA-0171600614	Czech Republic, Moravia, Znojmo, N48°51'05" E16°05'59", 250m, 16-Sep-2014, sweeping, leg. R. Stejskal, det. Stejskal,R.
Trachyphloeus spinimanus<br>Germar, 1824	KC784039 [new]<br>619-PSP<br>658nt	ZFMK-TIS-20402<br>ZFMK-DNA-0100448236	Germany, Lower Saxony (NI), Bad Sachsa, Sachsenstein, N51°35'14" E10°34'56", 291m, 23-Jul-2011, *Helianthemum nummularium u.a.*, beating, leg. Sprick,P., det. Sprick,P.
Trachyphloeus spinimanus<br>Germar, 1824	MK891393 [new]<br>1453-JKR<br>658nt	ZFMK-TIS-4222<br>ZFMK-DNA-0155630479	Hungary, Nograd, Pilisszanto, Pilis Mt., N47°40'47" E18°52'43", 466m, 27-Sep-2013, sieving, leg. Krátky,J., det. Krátky,J.
Trachyphloeus spinimanus<br>Germar, 1824	KC784203 [new]<br>446-RGO<br>658nt	ZFMK-TIS-20232<br>ZFMK-DNA-0100449393	Poland, Rudnik, N51°14'31.02" E22°32'22.92", 191m, 03-Okt-2011, leg. Gosik,R., det. Gosik,R.
Trachystyphlus alpinus<br>(Penecke, 1894)	MK892026 [new]<br>2300-JKR<br>658nt	ZFMK-TIS-24231<br>ZFMK-DNA-0169170966	Austria, Kaernten, Petzen, N46°31'01" E14°46'15", 1698m, 28-Jun-2014, alpine grassland, sieving, leg. Krátky,J., det. Krátky,J.
Trachystyphlus alpinus<br>*italocentralis*<br>(G. Osella & Zuppa, 1994)	MK891633 [new]<br>1770-PST<br>658nt	ZFMK-TIS-24094<br>ZFMK-DNA-0171622157	Italy, Lazio, Alvito, S. Onófria, Val de Rio, N41°43'47" E13°44'06", 741m, 05-Mai-2014, *Quercus*, sieving, leg. Stüben,P. & Schütte,A., det. Stüben,P.
Trachystyphlus beigerae<br>(Smreczynski, 1975)	MK892032 [new]<br>2308-JKR<br>658nt	ZFMK-TIS-24239<br>ZFMK-DNA-0169170957	Slovakia, Tatry Mts., Bujaci vrch, N49°14'10" E20°14'27", 1914m, 06-Jul-2014, *Cerastium latifolium*, sieving, leg. Krátky,J., det. Krátky,J.
Trichopterapion holosericeum<br>(Gyllenhal, 1833)	MK890876 [new]<br>493-RST<br>658nt	ZFMK-TIS-20278<br>ZFMK-DNA-0100447282	Czech Republic, Moravia mer., Podyji NP, 2.4 km NW of Hnanice, N48°49'1.00" E15°58'7.51", 300m, 10-Apr-2011, *Quercus, Carpinus*, Quercus-Carpinus forest, sieving, leg. Stejskal,R., det. Stejskal,R.
Trichopterapion holosericeum<br>(Gyllenhal, 1833)	MK891347 [new]<br>1385-PST<br>658nt	ZFMK-TIS-4681<br>ZFMK-DNA-0155628548	Italy, Lazio, forest above San Donato Val di Camino, N41°42'34" E13°48'49", 679m, 21-Aug-2013, *Carpinus*, sieving, leg. Stüben,P., det. Stüben,P.

**Name**<br>**Authority**<br>**Additional Information**	**GenBank Acc No (Ref.)**<br>**Specimen ID**<br>**Sequence Length**	**ZFMK Tissue ID**<br>**ZFMK DNA Sample ID**<br>**(SDEI DNA Sample ID)**	**Locality, GPS, Collection Date, Plant, Collector, Identifier**
Trichopterapion holosericeum<br>(Gyllenhal, 1833)	MK891275 [new]<br>1220-JKR<br>658nt	ZFMK-TIS-3556<br>ZFMK-DNA-0100426064	Slovakia, Krupinska planina, Dolne Strhare, Koprovnica, N48°16'27" E19°22'42", 251m, 28-Sep-2012, *Carpinus betulus*, sieving, leg. Krátky,J., det. Krátky,J.
Trichosirocalus barnevillei<br>(Grenier, 1866)	MK892105 [new]<br>2382-JKR<br>658nt	ZFMK-TIS-25959<br>ZFMK-DNA-0171600607	Czech Republic, Moravia, NP Podyji, Cizov, Siroke pole, N48°51'36" E15°51'02", 300m, 17-Aug-2014, *Achillea*, sweeping, leg. R. Stejskal, det. Stejskal,R.
Trichosirocalus barnevillei<br>(Grenier, 1866)	MK891361 [new]<br>1412-JKR<br>658nt	ZFMK-TIS-4181<br>ZFMK-DNA-0155628521	France, Cantal, Montagnac, N45°08'11" E03°43'19", 932m, 15-Jun-2013, *Achillea millefolium*, sweeping, leg. Krátky,J., det. Krátky,J.
Trichosirocalus histrix<br>(Perris, 1852)	MK891169 [new]<br>1080-JKR<br>658nt	ZFMK-TIS-3416<br>ZFMK-DNA-0100449113	Spain, Andalucia, Rociana del Condado, N37°21'01" W06°36'55", 150m, 18-Apr-2012, *Anthemis mixta*, sweeping, leg. Krátky,J., det. Krátky,J.
Trichosirocalus horridus<br>(Panzer, 1801)	KC783919 [new]<br>365-JKR<br>658nt	ZFMK-TIS-20151<br>ZFMK-DNA-0100438718	Czech Republic, Bohemia or., Horineves, N50°18'52.155" E15°46'30.012", 230m, 25-Jun-2011, *Carduus acanthoides*, sweeping, leg. Krátky,J., det. Krátky,J.
Trichosirocalus horridus<br>(Panzer, 1801)	MK891886 [new]<br>2151-JKR<br>658nt	ZFMK-TIS-23791<br>ZFMK-DNA-0169169582	Slovakia, Nove Zamky, Jursky Chlm, N47°48'04" E18°31'27", 114m, 19-Mai-2014, sweeping, leg. Krátky,J., det. Krátky,J.
Trichosirocalus spurnyi<br>(Schultze, 1901)	KC783908 [new]<br>380-JKR<br>658nt	ZFMK-TIS-20166<br>ZFMK-DNA-0100438698	Czech Republic, Bohemia or., Uhretice env., N49°58'39.655" E15°51'28.603", 240m, 05-Jul-2011, *Achilea millefolium*, sweeping, leg. Krátky,J., det. Krátky,J.
Trichosirocalus thalhammeri<br>(Schultze, 1906)	MK891451 [new]<br>1516-PSP<br>658nt	ZFMK-TIS-3276<br>ZFMK-DNA-0155624844	Denmark, Syddanmark, Emmerlev Klev (Højer), N54°59'53" E08°39'08", 3m, 30-Aug-2012, *Plantago maritima*, search on ground, leg. Sprick,P., det. Sprick,P.
Trichosirocalus thalhammeri<br>(Schultze, 1906)	MK891200 [new]<br>1118-JKR<br>658nt	ZFMK-TIS-3454<br>ZFMK-DNA-0100449590	Slovakia, Nove Zamky, Tvrdosovce, N48°06'09" E18°02'08", 110m, 10-Mai-2012, *Plantago maritima*, sweeping, leg. Krátky,J., det. Krátky,J.
Trichosirocalus troglodytes<br>(Fabricius, 1787)	MK890847 [new]<br>347-JKR<br>658nt	ZFMK-TIS-20133<br>ZFMK-DNA-0100438736	Czech Republic, Bohemia or., Uhretice env., N49°58'39.655" E15°51'28.603", 240m, 28-Mai-2011, sweeping, leg. Krátky,J., det. Krátky,J.
Trichosirocalus troglodytes<br>(Fabricius, 1787)	MK891735 [new]<br>1977-JKR<br>658nt	ZFMK-TIS-23617<br>ZFMK-DNA-0169170524	Czech Republic, Bohemia, Zastava, N50°06'42" E15°50'10", 234m, 11-Mrz-2014, sieving, leg. Krátky,J., det. Krátky,J.
Trichosirocalus troglodytes<br>(Fabricius, 1787)	MK890928 [new]<br>797-PST<br>658nt	ZFMK-TIS-3710<br>ZFMK-DNA-0100413785	Germany, Rhineland-Palatinate (RLP), E of Treis-Karden, Pommern, Pommerner Goldberg, high road (vineyard), N50°10'14" E07°16'29", 95m, 10-Jun-2012, *Plantago lanceolata*, beating, leg. Stüben,P., det. Stüben,P.
Trichosirocalus troglodytes<br>(Fabricius, 1787)	MK892324 [new]<br>2625-PSP<br>658nt	ZFMK-TIS-23599<br>ZFMK-DNA-0171661934	Germany, Bavaria (BY), Unterfranken, Ebern, N50°05'07" E10°45'52.5", 318m, 14-Jun-2014, *Plantago lanceolata*, leg. Sprick,P., det. Sprick,P.
Trichosirocalus troglodytes<br>(Fabricius, 1787)	MK891027 [new]<br>924-CBR<br>658nt	ZFMK-TIS-3837<br>ZFMK-DNA-0100449698	Luxembourg, 10 km E of Luxembourg, Mensdorf, N49°39'33" E06°18'13", 240m, 03-Jul-2011, *Plantago lanceolata*, sweeping, leg. Braunert,C., det. Braunert,C.
Tropiphorus elevatus<br>(Herbst, 1795)	MK892013 [new]<br>2286-JKR<br>658nt	ZFMK-TIS-24217<br>ZFMK-DNA-0169170979	Austria, Styria, Hochschwab, Stallmauer, N47°35'29" E15°02'20", 1260m, 27-Jun-2014, mountain forest, sweeping, leg. Krátky,J., det. Krátky,J.

**Name**<br>**Authority**<br>**Additional Information**	**GenBank Acc No (Ref.)**<br>**Specimen ID**<br>**Sequence Length**	**ZFMK Tissue ID**<br>**ZFMK DNA Sample ID**<br>**(SDEI DNA Sample ID)**	**Locality, GPS, Collection Date, Plant, Collector, Identifier**
Tropiphorus elevatus<br>(Herbst, 1795)	KC784132 [new]<br>658-PSP<br>658nt	ZFMK-TIS-20441<br>ZFMK-DNA-0100448616	Germany, Lower Saxony (NI), Lauenstein, Lkr. Hameln-Pyrmont, Ith Mts., N52°04'09" E09°32'36", 327m, 17-Nov-2011, *Allium ursinum, Fraxinus excelsior*, digging, leg. Sprick,P., det. Sprick,P.
Tropiphorus elevatus<br>(Herbst, 1795)	MK891072 [new]<br>974-PSP<br>658nt	ZFMK-TIS-3119<br>ZFMK-DNA-0100426219	Germany, Lower Saxony (NI), Lauenstein, Lkr.Hameln-Pyrmont, Ith Mts., N52°04'13' E09°32'30", 370m, 26-Mrz-2012, *Allium ursinum*, Fagus forest ("Kalkbuchen"), limestone, soil-search, leg. Sprick,P., det. Sprick,P.
Tropiphorus styriacus<br>(Grimmer, 1841)	MK891993 [new]<br>2264-JKR<br>658nt	ZFMK-TIS-24194<br>ZFMK-DNA-0169171002	Austria, Styria, Hochschwab, Spitzkögel, N47°35'27" E15°01'26", 1659m, 27-Jun-2014, alpine grassland, collecting by hand, leg. Krátky,J., det. Krátky,J.
Tychius aureolus<br>Kiesenwetter, 1852	MK892030 [new]<br>2306-JKR<br>658nt	ZFMK-TIS-24237<br>ZFMK-DNA-0169170959	Czech Republic, Bohemia, Krinec, PP Chotuc, N50°15'59" E15°06'59", 236m, 02-Jul-2014, steppic meadow, sweeping, leg. Krátky,J., det. Krátky,J.
Tychius bicolor<br>C. Brisout de Barneville, 1862	KC784317 [40]<br>768-PST<br>658nt	ZFMK-TIS-3105<br>ZFMK-DNA-0100448119	Spain, Canary Islands, La Gomera, Arure, Ermita Virgen de La Salud, N28°7'59" W17°19'10", 817m, 02-Mrz-2012, *Melilotus officinalis*, beating, leg. Stüben,P., det. Stüben,P.
Tychius breviusculus<br>Desbrochers des Loges, 1873	KC784153 [new]<br>614-PSP<br>658nt	ZFMK-TIS-20397<br>ZFMK-DNA-0100448653	Germany, Lower Saxony (NI), Brelingen, Hannover region, N52°33'53" E09°40'55", 14m, 16-Jul-2011, *Melilotus albus*, dry-warm ruderal area, beating, leg. Sprick,P., det. Sprick,P.
Tychius breviusculus<br>Desbrochers des Loges, 1873	MK892278 [new]<br>2579-PSP<br>658nt	ZFMK-TIS-23553<br>ZFMK-DNA-0171661980	Germany, Bavaria (BY), Unterfranken, Ebern, N50°05'22" E10°46'02", 327m, 13-Jun-2014, *Melilotus officinalis*, leg. Sprick,P., det. Sprick,P.
Tychius breviusculus<br>Desbrochers des Loges, 1873	MK891026 [new]<br>923-CBR<br>658nt	ZFMK-TIS-3836<br>ZFMK-DNA-0100449697	Luxembourg, 6 km E of Luxembourg, Oetrange, N49°36'20" E06°15'32", 250m, 14-Jun-2011, *Melilotus albus*, beating, leg. Braunert,C., det. Braunert,C.
Tychius capucinus<br>Boheman, 1843	KC783869 [30]<br>146-PST<br>658nt	ZFMK-TIS-2D100440288<br>ZFMK-DNA-0100438176	Morocco, NW of Berkane, Arkmane, N35°06'56" W02°43'43" , 13m, 14-Mai-2011, *Lotus*, beating, leg. Stüben,P., det. Stüben,P./vid. Behne,L.
Tychius colonnellii<br>Caldara, 1991	KC783799 [40]<br>92-PST<br>658nt	ZFMK-TIS-2D100446892<br>ZFMK-DNA-0100438029	Spain, Canary Islands, La Gomera, La Caleta – Punta San Lorenzo, N28°10'15" W17°9'38", 41m, 25-Feb-2011, *Lotus*, beating, leg. Stüben,P., det. Stüben,P.
Tychius crassior<br>Desbrochers des Loges, 1908	MK890806 [new]<br>123-PST<br>658nt	ZFMK-TIS-2D100440978<br>ZFMK-DNA-0100438201	Morocco, S of Berkane, Beni Snassen Mts., Vallee Zegzel, N34°49'26" W02°22'59", 634m, 12-Mai-2011, beating, leg. Stüben,P., det. Behne,L.
Tychius filirostris<br>Wollaston, 1854	MK892373 [40]<br>2824b-PST<br>658nt	ZFMK-TIS-25871<br>ZFMK-DNA-FC17941548	Portugal, Madeira, Ponta de Sao Lourenco, Pedras Brancas, N32°44'49" W16°42'12", 100m, 23-Nov-2015, *Cynara cardunculus*, sieving, leg. Stüben,P. & Schütte,A., det. Stüben,P.
Tychius filirostris<br>Wollaston, 1854	MK892387 [40]<br>2848-PST<br>658nt	ZFMK-TIS-4243<br>ZFMK-DNA-FC17941550	Portugal, Madeira, Janela, N32°51'19" W17°09'10", 32m, 02-Dez-2015, coast, sieving, leg. Stüben,P. & Schütte,A., det. Stüben,P.
Tychius filirostris<br>Wollaston, 1854	MK892389 [40]<br>2852-PST<br>658nt	ZFMK-TIS-4247<br>ZFMK-DNA-FC17941487	Portugal, Madeira, Achadas da Cruz, N32°51'23" W17°12'41", 47m, 04-Dez-2015, *Lotus*, beating, leg. Stüben,P. & Schütte,A., det. Stüben,P.
Tychius filirostris<br>Wollaston, 1854	MK892407 [40]<br>2903-PST<br>658nt	ZFMK-TIS-26209<br>ZFMK-DNA-0169166880	Portugal, Madeira, Porto Santo Isl., Cabeco d'Ponta, N33°02'05" W16°21'49", 5m, 26-Nov-2015, *Lotus glaucus*, leg. Stüben,P. & Schütte,A., det. Stüben,P.

**Name**<br>**Authority**<br>**Additional Information**	**GenBank Acc No (Ref.)**<br>**Specimen ID**<br>**Sequence Length**	**ZFMK Tissue ID**<br>**ZFMK DNA Sample ID**<br>**(SDEI DNA Sample ID)**	**Locality, GPS, Collection Date, Plant, Collector, Identifier**
Tychius junceus<br>(Reich, 1797)	MK891147 [new]<br>1054-PSP<br>658nt	ZFMK-TIS-3199<br>ZFMK-DNA-0100426805	Germany, Lower Saxony (NI), Braunschweig, Riddagshausen, N52°16'33" E10°33'51", 79m, 13-Jun-2012, *Lotus corniculatus*, grassland seeding, dipnet, leg. Sprick,P., det. Sprick,P.
Tychius junceus<br>(Reich, 1797)	MK891422 [new]<br>1485-PSP<br>658nt	ZFMK-TIS-3245<br>ZFMK-DNA-0155633344	Germany, Lower Saxony (NI), Hameln-Rohrsen, Dütberg, N52°06'27" E09°25'02", 116m, 15-Jul-2012, *Lotus corniculatus*, dipnet, leg. Sprick,P., det. Sprick,P.
Tychius junceus<br>(Reich, 1797)	MK892307 [new]<br>2608-PSP<br>658nt	ZFMK-TIS-23582<br>ZFMK-DNA-0171661948	Germany, Bavaria (BY), Unterfranken, Ebern, N50°05'07" E10°45'52.5", 318m, 14-Jun-2014, *Lotus corniculatus*, leg. Sprick,P., det. Sprick,P.
Tychius junceus<br>(Reich, 1797)	MK891029 [new]<br>926-CBR<br>658nt	ZFMK-TIS-3839<br>ZFMK-DNA-0100449685	Luxembourg, 20 km NE of Luxembourg, 1 km NW of Altrier, N49°45'12" E06°19'10", 370m, 07-Jul-2012, sweeping, leg. Braunert,C., det. Braunert,C.
Tychius kulzeri<br>Penecke, 1934	MK891557 [new]<br>1649-JKR<br>658nt	ZFMK-TIS-4227<br>ZFMK-DNA-0155630491	Romania, Buzau, Vulcanii Noroisi-Paclele Mici, N45°20'38" E26°42'37", 300m, 09-Jun-2013, sweeping, leg. Pelikan,J., det. Krátky,J.
Tychius kulzeri<br>Penecke, 1934	MK891896 [new]<br>2162-JKR<br>658nt	ZFMK-TIS-23802<br>ZFMK-DNA-0169169576	Slovakia, Nove Zamky, Sturovo, Hegyfarok, N47°49'06" E18°38'38", 205m, 21-Mai-2014, sweeping, leg. Krátky,J., det. Krátky,J.
Tychius meliloti<br>Stephens, 1831	KC784152 [new]<br>616-PSP<br>658nt	ZFMK-TIS-20399<br>ZFMK-DNA-0100448651	Germany, Lower Saxony (NI), Brelingen, Hannover region, N52°33'53" E09°40'55", 79m, 16-Jul-2011, *Melilotus albus*, dry-warm ruderal area, beating, leg. Sprick,P., det. Sprick,P.
Tychius meliloti<br>Stephens, 1831	MK892279 [new]<br>2580-PSP<br>658nt	ZFMK-TIS-23554<br>ZFMK-DNA-0171661968	Germany, Bavaria (BY), Unterfranken, Ebern, N50°05'22" E10°46'02", 327m, 13-Jun-2014, *Melilotus officinalis*, leg. Sprick,P., det. Sprick,P.
Tychius meliloti<br>Stephens, 1831	MK891930 [new]<br>2198-JKR<br>658nt	ZFMK-TIS-23936<br>ZFMK-DNA-0169170589	Slovakia, Nove Zamky, Kamenin, PP Cistiny, N47°51'55" E18°38'05", 111m, 22-Mai-2014, sweeping, leg. Krátky,J., det. Krátky,J.
Tychius parallelus<br>(Panzer, 1794)	MK891827 [new]<br>2088-JKR<br>658nt	ZFMK-TIS-23728<br>ZFMK-DNA-0169169646	Czech Republic, Moravia, Bucovice, PR Sevy, N49°08'05" E16°58'19", 249m, 09-Mai-2014, *Genista* sp., sweeping, leg. Krátky,J., det. Krátky,J.
Tychius parallelus<br>(Panzer, 1794)	MK891110 [new]<br>1015-PSP<br>658nt	ZFMK-TIS-3160<br>ZFMK-DNA-0100426845	Germany, Lower Saxony (NI), Berkhof, Hannover area, N52°36'48" E09°43'58", 36m, 10-Mai-2012, *Cytisus scoparius*, shrubs on edge of the forest, beating, leg. Sprick,P., det. Sprick,P.
Tychius parallelus<br>(Panzer, 1794)	MK891006 [new]<br>900-CBR<br>658nt	ZFMK-TIS-3813<br>ZFMK-DNA-0100449138	Luxembourg, 10 km NE of Mersch, 2 km NE of Nommern, N49°48'15" E06°11'16", 340m, 01-Mai-2011, *Cytisus scoparius*, beating, leg. Braunert,C., det. Braunert,C.
Tychius parallelus<br>(Panzer, 1794)	MK890953 [new]<br>832-PST<br>658nt	ZFMK-TIS-3745<br>ZFMK-DNA-0100414259	Portugal, Minho, PN of Peneda-Geres near Soajo, N41°52'14" W08°16'30", 200m, 09-Mai-2012, *Sarothamnus*, beating, leg. Stüben,P., det. Stüben,P.
Tychius picirostris<br>(Fabricius, 1787)	MK891199 [new]<br>1116-JKR<br>658nt	ZFMK-TIS-3452<br>ZFMK-DNA-0100449601	Czech Republic, Bohemia, Hradec Kralove, Fararstvi, N50°11'33" E15°48'10", 229m, 05-Mai-2012, sweeping, leg. Krátky,J., det. Krátky,J.
Tychius picirostris<br>(Fabricius, 1787)	MK891781 [new]<br>2040-JKR<br>658nt	ZFMK-TIS-23680<br>ZFMK-DNA-0169170462	Czech Republic, Bohemia, Kosatky, N50°19'23" E14°39'58", 218m, 02-Mai-2014, sweeping, leg. Krátky,J., det. Krátky,J.

**Name**<br>**Authority**<br>**Additional Information**	**GenBank Acc No (Ref.)**<br>**Specimen ID**<br>**Sequence Length**	**ZFMK Tissue ID**<br>**ZFMK DNA Sample ID**<br>**(SDEI DNA Sample ID)**	**Locality, GPS, Collection Date, Plant, Collector, Identifier**
Tychius picirostris<br>(Fabricius, 1787)	MK891127 [new]<br>1032-PSP<br>658nt	ZFMK-TIS-3177<br>ZFMK-DNA-0100426827	Germany, Lower Saxony (NI), Hannover, Stöcken, N52°24'24" E09°39'13", 42m, 22-Mai-2012, *Trifolium pratense*, species-rich hay meadow, dipnet, leg. Sprick,P., det. Sprick,P.
Tychius picirostris<br>(Fabricius, 1787)	MK892300 [new]<br>2601-PSP<br>658nt	ZFMK-TIS-23575<br>ZFMK-DNA-0171661958	Germany, Bavaria (BY), Unterfranken, Ebern, N50°05'17" E10°45'53", 343m, 13-Jun-2014, *Trifolium repens*, leg. Sprick,P., det. Sprick,P.
Tychius picirostris<br>(Fabricius, 1787)	MK892054 [new]<br>2330-JKR<br>658nt	ZFMK-TIS-24261<br>ZFMK-DNA-0169170935	Slovakia, Tatry Mts., Skalne vrata, N49°13'32" E20°16'37", 1341m, 07-Jul-2014, *Trifolium* cf.*spadiceum*, collecting by hand, leg. Krátky,J., det. Krátky,J.
Tychius pumilus<br>C. Brisout de Barneville, 1863	MK891734 [new]<br>1976-JKR<br>658nt	ZFMK-TIS-23616<br>ZFMK-DNA-0169170525	Czech Republic, Bohemia, Zastava, N50°06'42" E15°50'10", 234m, 11-Mrz-2014, sieving, leg. Krátky,J., det. Krátky,J.
Tychius pusillus<br>Germar, 1842	MK891142 [new]<br>1047-PSP<br>658nt	ZFMK-TIS-3192<br>ZFMK-DNA-0100426812	Germany, Lower Saxony (NI), Hannover, Ahlem, N52°23'00" E09°40'55", 50m, 01-Jun-2012, *Trifolium dubium*, Faboideae-rich ruderal area, dipnet, leg. Sprick,P., det. Sprick,P.
Tychius pusillus<br>Germar, 1842	MK892303 [new]<br>2604-PSP<br>658nt	ZFMK-TIS-23578<br>ZFMK-DNA-0171661944	Germany, Bavaria (BY), Unterfranken, Ebern, N50°05'02.5" E10°45'52", 313m, 14-Jun-2014, *Trifolium campestre*, leg. Sprick,P., det. Sprick,P.
Tychius quinquepunctatus<br>(Linnaeus, 1758)	MK890979 [new]<br>870-PST<br>658nt	ZFMK-TIS-3783<br>ZFMK-DNA-0100413781	Germany, Rhineland-Palatinate (RLP), Andernach, Kruft, Korretsberg, N50°23'00" E07°20'57", 211m, 25-Jul-2012, beating, leg. Stüben,P., det. Stüben,P.
Tychius quinquepunctatus<br>(Linnaeus, 1758)	MK891120 [new]<br>1025-PSP<br>658nt	ZFMK-TIS-3170<br>ZFMK-DNA-0100426834	Germany, Lower Saxony (NI), Hameln-Rohrsen, Dütberg, N52°06'27" E09°24'59", 117m, 18-Mai-2012, *Vicia angustifolia*, dipnet, leg. Sprick,P., det. Sprick,P.
Tychius quinquepunctatus<br>(Linne, 1758)	MK892302 [new]<br>2603-PSP<br>658nt	ZFMK-TIS-23577<br>ZFMK-DNA-0171661956	Germany, Bavaria (BY), Unterfranken, Ebern, N50°05'17" E10°45'53", 343m, 14-Jun-2014, *Vicia sativa agg.*, leg. Sprick,P., det. Sprick,P.
Tychius quinquepunctatus<br>(Linne, 1758)	MK891702 [new]<br>1920-FBA<br>658nt	ZFMK-TIS-23311<br>ZFMK-DNA-0169170368	Greece, East Macedonia and Thrace, Drama, Exochi, N41°24'25" E23°49'28", 630m, 13-Aug-2014, leg. Bayer & Brunner, det. Bayer,F.
Tychius quinquepunctatus<br>(Linnaeus, 1758)	MK891050 [new]<br>950-CBR<br>658nt	ZFMK-TIS-3863<br>ZFMK-DNA-0100449661	Luxembourg, Luxembourg, N49°38'00" E06°10'45", 345m, 03-Jul-2012, sweeping, leg. Braunert,C., det. Braunert,C.
Tychius quinquepunctatus<br>(Linnaeus, 1758)	MK890861 [new]<br>420-RGO<br>658nt	ZFMK-TIS-20206<br>ZFMK-DNA-0100449419	Poland, Tarnogóra, N50°41'11.6" E23°07'13.4", 249m, 11-Jun-2011, leg. Gosik,R., det. Gosik,R.
Tychius rufipennis<br>C. Brisout de Barneville, 1863	KC784084 [new]<br>531-RST<br>658nt	ZFMK-TIS-20316<br>ZFMK-DNA-0100448441	Czech Republic, Moravia mer., Podyji NP, Havraniky - heathland , N48°48'46.24" E16°0'1.49", 320m, 12-Aug-2011, *Trifolium*, sweeping, leg. Stejskal,R., det. Stejskal,R.
Tychius schneideri<br>(Herbst, 1795)	MK891066 [new]<br>967-CBR<br>658nt	ZFMK-TIS-3880<br>ZFMK-DNA-0100449645	Luxembourg, 10 km NW of Esch/Alzette, 1 km NW of Niedercorn, N49°32'31" E05°52'39", 370m, 28-Jun-2012, *Anthyllis vulneraria*, sweeping, leg. Braunert,C., det. Braunert,C.
Tychius sharpi<br>Tournier, 1874	MK891931 [new]<br>2199-JKR<br>658nt	ZFMK-TIS-23937<br>ZFMK-DNA-0169170588	Czech Republic, Moravia, NP Podyji, Hnanice, N48°48'26" E15°58'23", 330m, 30-Mai-2014, *Trifolium montanum*, beating, leg. Stejskal,R., det. Stejskal,R.

**Name**<br>**Authority**<br>**Additional Information**	**GenBank Acc No (Ref.)**<br>**Specimen ID**<br>**Sequence Length**	**ZFMK Tissue ID**<br>**ZFMK DNA Sample ID**<br>**(SDEI DNA Sample ID)**	**Locality, GPS, Collection Date, Plant, Collector, Identifier**
Tychius squamulatus<br>Gyllenhal, 1835	MK892323 [new]<br>2624-PSP<br>658nt	ZFMK-TIS-23598<br>ZFMK-DNA-0171661935	Germany, Lower Saxony (NI), Hameln-Rohrsen, Dütberg, N52°06'24" E09°24'57", 116m, 21-Jul-2014, *Lotus corniculatus*, leg. Sprick,P., det. Sprick,P.
Tychius stephensi<br>Schoenherr, 1835	MK891855 [new]<br>2117-JKR<br>658nt	ZFMK-TIS-23757<br>ZFMK-DNA-0169169627	Czech Republic, Moravia, Lanzhot, N48°42'37" E16°58'27", 156m, 10-Mai-2014, sweeping, leg. Krátky,J., det. Krátky,J.
Tychius stephensi<br>Schoenherr, 1835	MK892310 [40]<br>2611-PSP<br>658nt	ZFMK-TIS-23585<br>ZFMK-DNA-0171661951	Germany, Bavaria (BY), Unterfranken, Ebern, N50°05'03" E10°45'15", 331m, 14-Jun-2014, *Trifolium pratense*, leg. Sprick,P., det. Sprick,P.
Tychius striatulus<br>Gyllenhal, 1835	KC783881 [new]<br>117-PST<br>603nt	ZFMK-TIS-2D100440307<br>ZFMK-DNA-0100438195	Morocco, S of Berkane, Beni Snassen Mts., Vallee Zegzel, N34°49'33" W02°22'37", 590m, 11-Mai-2011, *Ononis natrix*, beating, leg. Stüben,P., det. Stüben,P./vid. Behne,L.
Zacladus exiguus<br>(Olivier, 1807)	MK891360 [new]<br>1411-JKR<br>658nt	ZFMK-TIS-4180<br>ZFMK-DNA-0155628522	France, Cantal, Sainte Eugenie de Villeneuve, N45°08'11" E03°37'40", 973m, 15-Jun-2013, *Geranium* sp., sweeping, leg. Krátky,J., det. Krátky,J.
Zacladus exiguus<br>(Olivier, 1807)	MK891042 [new]<br>940-CBR<br>658nt	ZFMK-TIS-3853<br>ZFMK-DNA-0100449666	Luxembourg, 10 km E of Luxembourg, 1 km SW of Mensdorf, N49°39'32" E06°18'35", 300m, 09-Jun-2012, *Geranium robertianum*, sweeping, leg. Braunert,C., det. Braunert,C.
Zacladus exiguus<br>(Olivier, 1807)	MK891952 [new]<br>2220-JKR<br>658nt	ZFMK-TIS-23958<br>ZFMK-DNA-0169170567	Slovakia, Galanta, Vinohrady nad Vahom, N48°21'15" E17°45'24.5", 140m, 14-Jun-2014, *Geranium pusillum*, leg. Benedikt,S., det. Benedikt,S.
Zacladus geranii<br>(Paykull, 1800)	MK892005 [new]<br>2277-JKR<br>658nt	ZFMK-TIS-24208<br>ZFMK-DNA-0169170991	Austria, Styria, Hochschwab, Stallmauer, N47°35'29" E15°02'20", 1260m, 27-Jun-2014, mountain forest, sweeping, leg. Krátky,J., det. Krátky,J.
Zacladus geranii<br>(Paykull, 1800)	KC783963 [new]<br>372-JKR<br>658nt	ZFMK-TIS-20158<br>ZFMK-DNA-0100438926	Czech Republic, Bohemia or., Hrobice, N50°6'2.909" E15°46'50.867", 230m, 05-Jul-2011, *Geranium* sp., sweeping, leg. Krátky,J., det. Krátky,J.
Zacladus geranii<br>(Paykull, 1800)	KC784082 [new]<br>541-RST<br>658nt	ZFMK-TIS-20326<br>ZFMK-DNA-0100448438	Czech Republic, Moravia mer., NP Podyji, Lipinska louka meadow, 4 km SE of Podmoli, N48°49'3.85" E15°57'45.33", 300m, 01-Sep-2011, *Geranium pratense*, sweeping, leg. Stejskal,R., det. Stejskal,R.
Zacladus geranii<br>(Paykull, 1800)	MK891814 [new]<br>2074-JKR<br>658nt	ZFMK-TIS-23714<br>ZFMK-DNA-0169169659	Czech Republic, Bohemia, Predhradi, N49°49'56" E16°02'33", 427m, 08-Mai-2014, sweeping, leg. Krátky,J., det. Krátky,J.
Zacladus geranii<br>(Paykull, 1800)	MK891411 [new]<br>1473-PSP<br>658nt	ZFMK-TIS-3233<br>ZFMK-DNA-0155633359	Germany, Lower Saxony (NI), Harz, St. Andreasberg, Jordanshöhe, N51°42'58" E10°32'06", 670m, 02-Jul-2012, *Geranium sylvaticum*, perennials corridor, beating, leg. Sprick,P., det. Sprick,P.
Zacladus geranii<br>(Paykull, 1800)	MK891984 [new]<br>2255-JKR<br>658nt	ZFMK-TIS-23993<br>ZFMK-DNA-0169170535	Slovakia, Kezmarok, Vysoke Tatry Mts., Zadne Medodoly valley, N49°14'09" E20°12'15", 1510m, 24-Jun-2014, *Geranium sylvatica*, leg. Benedikt,S., det. Benedikt,S.
Zacladus geranii<br>(Paykull, 1800)	MK892117 [new]<br>2394-JKR<br>658nt	ZFMK-TIS-25971<br>ZFMK-DNA-0171600616	Slovakia, Zilina, Rajecke Teplice, N49°07'14" E18°41'19", 434m, 21-Jun-2014, *Geranium* sp., sweeping, leg. Krátky,J., det. Krátky,J.
