# Supplementary material for: Molecular Weevil Identification Project: A thoroughly curated barcode release of 1300 Western Palearctic weevil species (Coleoptera, Curculionoidea)
Source: Biodivers Data J. 2023 Jan 24;11:e96438. doi: 10.3897/BDJ.11.e96438 (PMC10865102; doi:10.3897/BDJ.11.e96438)
Supplement: Supplementary material 4 — Results of Taxonomy [file bdj-11-e96438-s004.pdf]

Results Taxonomy

Eighteen cases of contradictions between morphological identification and molecular results are discussed here (for NJ tree, see Suppl. material 3). Most of these cases await synonymization or constitute very young species or species complexes challenging to disentangle with the CO1 gene. References are listed at the end of this chapter (page 6) and in the main article.

Ceutorhynchus wellschmiedi Dieckmann, 1979 seems to be a **junior synonym of *Ceutorhynchus chalybaeus*** Germar, 1824. As discussed in the Ceutorhynchinae key part 2 of the Curculio Institute (Stüben et al. 2013b), the two species were separated using the tip of the slim elytral bristles. They either reach the base of the following bristle or end significantly before it. However, during key creation, it was discovered that the aedeagi (and internal sack structures) do not feature noticeable differences. The mentioned morphological differential characters challenge the limits of reproducibility, as with molecular results. CO1 distance between both species is <1.2%). We suggest synonymizing the species *Ceutorhynchus wellschmiedi*, described by Dieckmann (1979). The latter is claimed to occur in Central Europe monophagous on *Crambe tataria*, while *Ceutorhynchus chalybaeus* lives on a wide range of Brassicaceae (e.g., *Sysimbrium*, *Capsella*, *Sinapis*, *Alliaria*, *Erysimum*). One could also consider a different perspective: In Central Europe, *Ceutorhynchus chalybaeus* uses one additional species of Brassicaceae as a host plant, on which the species has not been previously found (including by L. Dieckmann). It seems to be a tautology: the monophagous host plant relationship with *Crambe tataria* is used as a species delineation criterion. Dieckmann wrote, "Thus, through the use of the host plant, the new species can be told apart from the other six species of the *chalybaeus* group, as these live on other genera of Cruciferae (= Brassicaceae)." For images and further information see Stüben et al. 2015.

Ceutorhynchus erysimi (Fabricius, 1787) and ***Ceutorhynchus contractus*** (Marsham, 1802) might be a **species complex**. *Ceutorhynchus erysimi*: metallic shining, greenish or bluish elytra; *Ceutorhynchus contractus*: metallic dark blue or bluish-black elytra. The aedeagi offer little help for a species delineation, see the Ceutorhynchinae key part 2 of the Curculio Institute (Stüben et al. 2013b: 8). In the FHL 1983 catalogue (Freude et al. 1983: 218) the situation is presented differently: "here, different aedeagi and elytral contours are depicted," but just two are offered for the species *C. contractus*. In our CO1-based reconstruction, *C. contractus* is paraphyletic concerning *C. erysimi*, the latter appearing nested within the first multiple times. However, the genetic distances are too large to lump into a single species. The species pair *Ceutorhynchus erysimi/contractus* thus seems to comprise of a species complex composed of several species. Perhaps the hint given in FHL 1983 catalogue might serve as a starting point for a new morphological approach, targeting the distinct contours of the aedeagi. For example, the apical protrusion of the median lobe could be used as a differential character. A revision of this species complex may be required. See Stüben et al. 2015 for images and further information.

Hesperorrhynchus linaeotesselatus x glutinosus might be a rare case of **horizontal gene transfer**. The specimens 1936-PST and 2773-PST are identified as *Hesperorrhynchus glutinosus* Stüben, 2016, but contain a CO1 barcode of *Hesperorrhynchus linaeotesselatus* (Wollaston, 1854). This contradictory observation is a possible rare case of horizontal gene transfer in weevils, likely caused by a recent hybridization event. Both species currently

occur allopatric on Madeira island. The introduction to Madeira and their subsequent separation led to the evolution of two young species. The volcanic island Madeira is just 5 million years old. It is likely that we have found an example of introgression of mitochondrial genes as a result of hybridization. While evolutionary young splits can be found in Cryptorhynchinae genera of *Silvacalles* and *Dendroacalles* from the Canary Islands as well (Stüben and Astrin 2010), for *H. linaeotesselatus and H. glutinosus* we also observe significant morphological differences in a combination of molecular similarity. This is rare amongst the Curculionidae but has been observed before (Funk and Omland 2003). Additional sequences from nuclear genes are needed for deeper insights. With a high possibility, nuclear genetic data does not show the same inconsistency since hybridized chromosomes might be outcrossed while the maternally inherited mitochondrial genes are persistant. For more information about these species, especially their biology and distribution on Madeira island, see Stüben and Krátký 2016. A more comprehensive ***Hesperorrhynchus*** dataset is being presented in Stüben et al. 2021.

Cionus griseus Lindberg, 1958 might be **a synonym of *Cionus variegatus*** (Brullé, 1838) or have been hybridized recently: Two colors, but molecularly just one species. Both *Cionus* species occur on Tenerife. They are completely differently coloured and feed on different endemic Scrophulariaceae (*Scrophularia glabrata* Aiton, 1789 and *Scrophularia smithii langeana* (Bolle) Dalgaard, 1979. The molecular analysis of CO1 shows an interspecific p-distance of < 0.9%. Molecularly we are dealing with one species, but both species can be distinguished morphologically from their markings. Therefore, the initial suspicion is that *Cionus griseus* Har. & Hal. Lindberg, 1958 on Tenerife could be a younger synonym of *Cionus variegatus* (Brullé, 1839). Peter Stüben found additional specimens of *C. variegatus* on La Gomera, feeding on the introduced Scrophulariaceae *Verbascum virgatum* Stokes, 1787. This was an initial indication a strict host-plant binding might not exist on every island. Though it cannot be conclusively clarified at the mitochondrial level, if we have found another case of mitochondrial introgression after hybridization on La Palma and Tenerife. A very young species separation could also be possible. However, nuclear gene sequences are required to solve this contradiction. It is likely, that on La Palma we are dealing with yellow-black intermediate forms of two morphs. On Tenerife, we observe host plant specific behavior without intermediate forms and without featuring distinct molecular differences, see images in Stüben and Behne 2015 and Stüben et al. 2021 for a more detailed discussion.

Cleopus pulchellus (Herbst, 1795): Most likely a **cryptic species** has been uncovered. Two *Cleopus pulchellus* specimens were collected and sequenced: specimen ID 935-CBR (GenBank acc. MK891037) from Luxembourg (leg./det. Carlo Braunert) and 1854-PST (GenBank acc. MK891659) from Madeira island (leg./det. Peter Stüben). The distance of 16.4% between those two specimens leaves room for two possible conclusions. Either one is a misidentification, or this taxon contains a new cryptic species. The latter is most likely to apply for the specimen from Madeira (1854-PST). At the current stage, the specimens are still being processed. If it turns out 1854-PST is an undescribed species, it will be newly described, likely as ***Cleopus maderensis* sp.n.**

Rhinoncus smreczynskii Wagner, 1937 is likely a **junior synonym of *Rhinoncus perpendicularis*** (Reich, 1797). Specimen IDs of *Rhinoncus smreczynskii* in the NJ tree (Appendix 03): 2424-JKR, 327-JKR, 2425-JKR, 2066-JKR. In the first digital determination key on Ceutorhynchinae by the Curculio Institute (Stüben et al. 2012), *Rhinoncus perpen-*

dicularis and *Rhinoncus smreczynskii* are differentiated by the scale spots on the elytra. Their aedeagi are identical. There are no further differences known so far. The few isolated collection sites of *Rhinoncus smreczynskii* are allopatric occurrences within the distribution area of *Rhinoncus perpendicularis*. A refuge area during glaciation is unknown. *Rhinoncus smreczynskii* was released from synonymy with *Rhinoncus perpendicularis* about 20 years ago (Wanat, 2000). This molecular analysis does not support the reinstatement of *Rhinoncus smreczynskii*. The interspecific distance between both taxa is between 0% and 1.5% only (0% pairs: 2425-JKR vs. 273-PSP/386-JKR; 1.5% pair: 2424-JKR vs. 1125-JKR). Additional information and image stackings are provided in Schütte et al. 2013.

Anthonomus conspersus Desbrochers des Loges, 1868 is most likely a **junior synonym** of ***Anthonomus pedicularius*** (Reich, 1797). Specimen IDs of *A. conspersus*: 2477-PSP, 919-CBR. Specimen IDs of *A. pedicularius*: 2025-JKR, 2206-JKR, 251-PSP. In the Digital-Weevil-Determination key of *Anthonomus* (Stüben et al. 2016b), the aedeagus of *A. pedicularius* is clearly larger compared to *A. conspersus*, but *A. pedicularius* is larger (2.9 - 3.8mm) than *A. conspersus* (2.5 - 3.0mm) in general. The second differential character is the width of the elytrae: for *A. pedicularius*, the elytra are widest in front or the center. For *A. conspersus*, the elytra are widest behind the center. Based on the genetic distances, the current species status can not be confirmed. The lowest interspecific distance between both taxa 2206-JKR (*A. pedicularius*) vs. 2499-PSP (*A. conspersus*) is 1.2%. Their ecology delivers a strong argument to maintain the current state of two species: *A. conspersus* lives on *Sorbus aucuparia* (Rosaceae family), often in a very cold and shady habitat, while *A. pedicularius* lives on *Crataegus* (Rosaceae family). Of course, neither a recent split nor a hybridization event cannot be ruled out, leading to opposite conclusions.

Amaurorhinus punctipennis Osella & Giusto, 1985 is a junior synonym of ***Amaurorhinus monizianus*** (Wollaston, 1860). Peter Stüben suggested the synonymization of *Amaurorhinus punctipennis* in Stüben 2014a and Stüben 2014b. Although the taxonomic change has not carried out formally yet, *A. punctipennis* is already listed as a known synonym in the "Cooperative Catalogue of Palearctic Coleoptera Curculionoidea" (Alonso-Zarazaga et al. 2017). To facilitate a formal synonymization, we used the currently valid name *Amaurorhinus punctipennis* (ES1028, 80-PST, 757-PST, 1664-PST, 1668-PST) in this barcode release. However, there is no doubt that *A. punctipennis* is a junior synonym. See Stüben 2014a for an in depth discussion including genetic distances and focus stacked images. Previously, *A. folwacznyi* G. Osella & Giusto, 1985 was synonymized with *A. punctipennis* in Stüben 2014b.

The ***maraoensis*** **clade** of the ***Acalles sierrae*** **complex** shows small distances and high morphological distinctness: ***A. sarothamni*** (E-466-sar, E-467-sar), ***A. monasterialis*** (E-505-mon, E-848-mon), ***A. cytisi*** (E-462-cyt, E-845-cyt, E-847-cyt) and ***A. maraoensis*** (E-581-mar, E-835-mar, E-875-mar, E-867-mar, E-869-mar). Those previous mentioned taxa can not be separated based on the present sequence data, which is uncommon for Cryptorhynchinae weevils. These characteristics are adressed in detail in Schütte and Stüben 2015.

Hypera ononidis (Chevrolat, 1863) and ***Hypera subcordicollis*** (Desbrochers, 1900) are most likely **junior synonyms** of ***Hypera nigrirostris*** (Fabricius, 1775). In our opinion the morphological subdivision according to the length of the elytral bristles and a different host plant association cannot be supported anymore (Skuhrovec 2009). Both species are likely

younger synonyms of *Hypera nigrirostris* (Fabricius, 1775). The samples available to us differ by at most 2.7% CO1 distance for these three species. More certainty exists about *Hypera ononidis* and the Moroccan form *Hypera subcordicollis*, where the studied specimens cannot be separated from each other molecularly (p-distance of 0.15%). The closest delineable species from the so-called *nigrirostris*-group is *Hypera venusta* (Fabricius, 1781) with a distance of >4.9%. These results have already been addressed in Stüben et al. 2015a, where additional information and pictures are provided. A formal synonymization is still pending.

Sitona lateralis Gyllenhal, 1834 is most likely a **junior synonym of *Sitona suturalis*** Stephens, 1831. Specimen IDs of *S. lateralis* are: 257-PSP and 1121-JKR. A distinct cluster formation cannot be observed. Neither the scattered pattern in the tree nor the few marginal morphological differential characters support the two species. This issue was already discussed in Schütte et al. 2013 and Stüben et al. 2015, but a formal synonymization is pending.

The subspecies ***Sitona sulcifrons argutulus*** Gyllenhal, 1834 cannot be distinguished from its nominotypical taxon ***Sitona sulcifrons sulcifrons*** (Thunberg, 1798) with the molecular data. In the current determination key of the Curculio Institute, the eyes of *Sitona sulcifrons* are relatively flat compared to *S. sulcifrons argutulus* which has much stronger curvature (Bahr et al. 2008). Thus, we think the current subspecies' status is reasonable.

Romualdius angustisetulus (Hansen, 1915) is likely a synonym of ***Romualdius scaber*** (Linnaeus, 1758) = *Romualdius bifoveolatus* (Beck, 1817). Specimen IDs of *R. angustisetulus*: 563-RST, 925-CBR, 1277-PST). There are no morphologic characters known to distinguish these three species. The three differential characters used so far do not occur on every specimen. This issue has been addressed previously: Stüben 1994, based on morphology; Schütte et al. 2013 and Stüben 2014a, based on morphology and molecular data. A formal synonymization is pending.

The ***Brachyderes pubescens*** (Boheman, 1833) specimen from France (488-PST) shows an intraspecific distance of 12.3% to the specimen from Morocco (157-PST). The specimen from Morocco (157-PST) shows 4.8% to the specimen from Italy (IT-0048w), which seems reasonable for intraspecific distances. Surprisingly, the geographically more distant specimen from Morocco is genetically closer to the Italian specimen than the one from France. The reason for this observation is unknown.

Catapion meieri Desbrochers, 1901 is likely a synonym of ***Catapion seniculus*** Kirby, 1808. Specimen IDs of C. meieri: 617-PSP, 2596-PSP. The morphological differences for *C. meieri* described in FHL 11/1983 catalogue (Freude et al. 1983) are: eyes are smaller and more rounded in females, proboscis is thinner, *C. meieri* living monophagous on *Trifolium hybridum*. Those differences do not suggest a distinct species. The average inter-specific distance for the genus Catapion is 11.2%. In comparison, the lowest interspecific distance based on the reference dataset of this barcode release is 8.8% (*Catapion pubescens* vs. *Catapion meieri*). A relatively low genetic interspecific distance of 3.5% between *C. meieri* and *C. seniculus* points to an intraspecific relationship as opposed to an interspecific one. See Stüben et al. 2015 for additional images and in-depth discussion.

Ischnopterapion plumbeomicans plumbeomicans (Rosenhauer, 1856) and ***Ischnopterapion fairmairei*** (Wencker, 1864) are likely synonyms of ***Ischnopterapion modestum*** (Germar, 1817). Specimen IDs of *Ischnopterapion plumbeomicans plumbeomicans*: 101-PST, 728-PST, 48-PST, 84-PST, 847-PST. Specimen ID of *Ischnopterapion fairmairei*: 103-PST. The distances between the previous three mentioned species are mostly below 1%, with a maximum distance of 1.8% between *Ischnopterapion plumbeomicans* from Gran Canaria (48-PST) and *Ischnopterapion fairmairei* from Gibraltar (103-PST). These findings have been discussed in detail in Stüben et al. 2015.

Perapion oblongum (Gyllenhal, 1839) is likely a synonym of ***Perapion curtirostre*** (Germar, 1817). The specimen ID of *Perapion oblongum* det. Sprick from *Rumex thyrsiflorus* is 587-PSP. Specimen IDs of *Perapion curtirostre* det. Sprick from *Rumex acetosa* are 1037-PSP and 2495-PSP. These "two species" are exclusively separated by body size. However, they show significant overlapping size ranges based on the FHL 10/1981 catalogue (Freude et al. 1981: 138). We think the host plant association with different *Rumex* species does not support the species delineation either.
Additionally, the species of the genus *Perapion* Wagner, 1907 currently belong to the tribe Aplemonini Kissinger, 1968. The same applies to *Aizobius*, *Phrissotrichum*, *Pseudoperapion*, *Pseudaplemonus* and *Cistapion*. The molecular data do not confirm those representing a monophylum but belong to several somewhat different clades or genera of this tribe. These findings have been previously addressed in Stüben et al. 2015.

Hypophyes minutissimus (Tournier, 1868) might be a **junior synonym of *Hypophyes pallidulus*** (Gravenhorst, 1807). The distances are between 0.9 and 2% between *Hypophyes minutissimus* (155-PST) and *Hypophyes pallidulus* (IT-0021w, IT-0039w, 1299-PST). These very low distances point toward an intra-specific variation of one species as opposed to interspecific distances between two. Unfortunately, specimen 155-PST was destroyed during the DNA extraction process. Other specimens of *Hypophyes* are not available in the MWI dataset. Without additional specimens it is difficult to provide a statement about the taxonmic relationship at this time. A geographical cline could become an observation.

References

- Alonso-Zarazaga MA, Barrios H, Borovec R, Bouchard P, Caldara R, Colonnelli E, Gültekin L, Hlaváč P, Korotyaev BA, Lyal C, Machado A, Meregalli M, Pierotti H, Ren L, Sánchez-Ruiz M, Sforzi A, Silfverberg H, Skuhrovec J, Trýzna M, Yunakov NN (2017): 'Cooperative Catalogue of Palaearctic Coleoptera Curculionoidea.' (Monografías electrónicas S.E.A.: Zaragoza, Spain): 729 pp.
- Bahr F, Bayer C, Behne L, Sprick P, Stüben PE (2008) Digital-Weevil-Determination for Curculionoidea of West Palaearctic. Transalpina: Sitona (Entiminae: Sitonini). *SNUDEBILLER: Studies on taxonomy, biology and ecology of Curculionoidea* **7**(86): 21-27. URL: https://www.curci.de/?beitrag=86
- Dieckmann L (1979) Neue paläarktische Arten aus der Unterfamilie Ceutorhynchinae (Coleoptera, Curculionidae). *Reichenbachia, Staatliches Museum für Naturkunde in Dresden* **17**(8): 49-56.
- Freude H, Harde KW, Lohse GA (1981) Die Käfer Mitteleuropas. Band 10. Curculionidae I. Vol. 10,Goecke & Evers, Krefeld, Germany, 310 pp. [In German] [ISBN 9783827406842]
- Freude H, Harde KW, Lohse GA (1983) Die Käfer Mitteleuropas. Band 11. Curculionidae II. Vol. 11,Goecke & Evers, Krefeld, Germany, 344 pp. [In German] [ISBN 9783872630315]
- Funk DJ, Omland KE (2003) Species-Level Paraphyly and Polyphyly: Frequency, Causes, and Consequences, with Insights from Animal Mitochondrial DNA. *Annual Review of Ecology, Evolution, and Systematics* **34**(1): 397-423. DOI: 10.1146/annurev.ecolsys.34.011802.132421.
- Schütte A, Stüben PE, Sprick P (2013): The Molecular Weevil Identification Project (Coleoptera: Curculionoidea), Part I - A contribution to Integrative Taxonomy and Phylogenetic Systematics - *SNUDEBILLER: Studies on taxonomy, biology and ecology of Curculionoidea* **14**(211): 1-77. URL: https://www.curci.de/?beitrag=211
- Schütte A and Stüben PE (2015): Molecular systematics and morphological identification of the cryptic species of the genus *Acalles* Schoenherr, 1825, with descriptions of new species (Coleoptera: Curculionidae: Cryptorhynchinae) - *Zootaxa* **3915**(1): 1-51. DOI: 10.11646/zootaxa.3915.1.1.
- Skuhrovec J (2009): Digital-Weevil-Determination for Curculionoidea of West Palaearctic. Transalpina: Hypera / Limobius / Metadonus (Hyperinae: Hyperini) - *SNUDEBILLER: Studies on taxonomy, biology and ecology of Curculionoidea* **10**(121): 39-47. URL: http://www.curci.de/?article=247
- Stüben PE (1994): Das *Trachyphloeus bifoveolatus-angustisetulus*-Problem und die intermediären Exemplare des Rheinlandes: Gibt es eine morphologische Lösung? (Coleoptera, Curculionidae) - *Mitteilungen der Arbeitsgemeinschaft Rheinischer Koleopterologen (Bonn)* **4** (3): 111-129. [In German]
- Stüben PE and Astrin JJ (2010) Molecular phylogeny in endemic weevils: revision of the genera of Macaronesian Cryptorhynchinae (Coleoptera: Curculionidae). *Zoological Journal of the Linnean Society* **160**(1): 40-87. DOI: 10.1111/j.1096-3642.2009.00609.x.

- Stüben PE, Sprick P, Müller G, Bayer Ch , Behne L, Krátký J (2012): Digital-Weevil-Determination for Curculionoidea of West Palaearctic: Transalpina: Ceutorhynchinae (1. Part). (Mononychini, Phytobiini, Hypurini, Cnemogonini, Scleropterini & Amalini) - *SNUDEBILLER: Studies on taxonomy, biology and ecology of Curculionoidea* **13**(191): 18-33. URL: https://www.curci.de/?beitrag=191
- Stüben PE, Müller G, Krátký J, Bayer C, Behne L, Sprick P (2013b) Digital-Weevil-Determination for Curculionoidea of West Palaearctic: Transalpina: Ceutorhynchinae (2. Part). (Ceuthorhynchini: Amalorrhynchus, Drupenatus, Poophagus, Coeliodes, Pseudocoeliodes, Coeliodinus, Eucoeliodes, Neoxyonyx, Thamiocolus, Micrelus, Zacladus, Phrydiuchus, Stenocarus, Nedyus, Ceutorhynchus: Marklissus). *SNUDEBILLER: Studies on taxonomy, biology and ecology of Curculionoidea* **14**(210): 1-23. URL: https://www.curci.de/?article=210
- Stüben PE (2014a): Die Curculionoidea (Coleoptera) Tenerifes. - SNUDEBILLER: Studies on taxonomy, biology and ecology of Curculionoidea 15(226): 1-118. URL: https://www.curci.de/?beitrag=226
- Stüben PE (2014b): New nomenclatural and taxonomic acts, and Comments (2014) - *SNUDEBILLER: Studies on taxonomy, biology and ecology of Curculionoidea* **15**(231): 1-5. URL: https://www.curci.de/?beitrag=231
- Stüben PE and Behne L (2015) Die Curculionoidea (Coleoptera) La Palmas. *SNUDEBILLER: Studies on taxonomy, biology and ecology of Curculionoidea* **16**(242): 1-86. URL: https://www.curci.de/?beitrag=242
- Stüben PE, Schütte A, Bayer C, Astrin JJ (2015) The Molecular Weevil Identification Project (Coleoptera: Curculionoidea), Part II - Towards an Integrative Taxonomy. *SNUDEBILLER: Studies on taxonomy, biology and ecology of Curculionoidea* **16**(237): 1-294. URL: https://www.curci.de/?beitrag=237
- Stüben PE and Krátký J (2016) *Hesperorrhynchus glutinosus* sp.n. and *H. palmensis* sp.n. - with a review of all *Hesperorrhynchus* species from the Macaronesian Islands (Coleoptera: Curculionidae: Ceutorhynchinae) - An contribution to Integrative Taxonomy. *SNUDEBILLER: Studies on taxonomy, biology and ecology of Curculionoidea* **17**(247): 1-8. URL: https://www.curci.de/?beitrag=247
- Stüben PE, Bayer Ch, Bahr F, Sprick P, Behne L (2016b): Digital-Weevil-Determination for Curculionoidea of the West Palearctic: Transalpina: Anthonomini. - *SNUDEBILLER: Studies on taxonomy, biology and ecology of Curculionoidea* **17**(249): 1-11. URL: https://www.curci.de/?beitrag=249
- Stüben PE, Schütte A, Astrin JJ (2021) Barcoding and interspecific relationships of Macaronesian Weevils (Coleoptera: Curculionoidea). *Contributions to Entomology* **71**(1): 127–135. DOI: 10.21248/contrib.entomol.71.1.127-135.
