# Supplementary figures and images for: Molecular Weevil Identification Project: A thoroughly curated barcode release of 1300 Western Palearctic weevil species (Coleoptera, Curculionoidea)
Source: Biodivers Data J. 2023 Jan 24;11:e96438. doi: 10.3897/BDJ.11.e96438 (PMC10865102; doi:10.3897/BDJ.11.e96438)

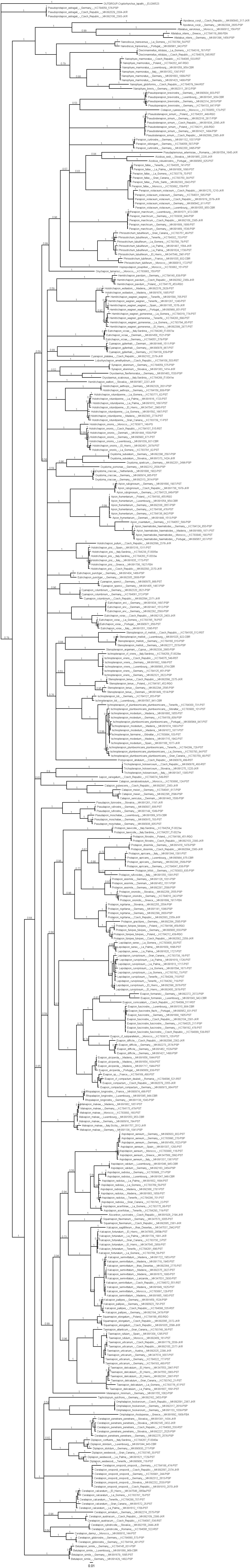

Supplement: Supplementary material 5 — Bayesian Trees [file bdj-11-e96438-s005.zip › MWI Appendix 5 - Bayesian Trees/Apioninae Nanophyinae Attelabidae bayesian tree.png]

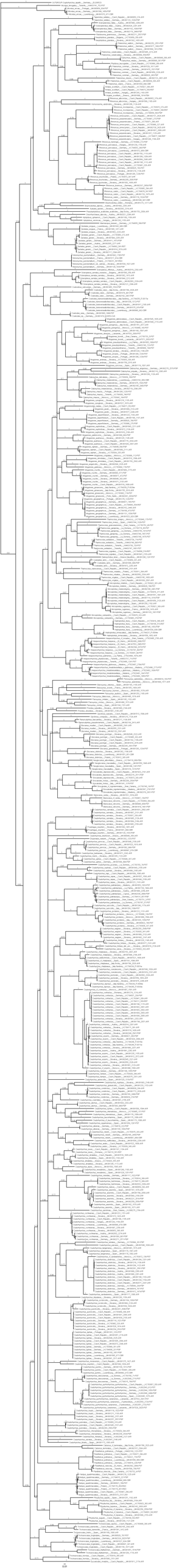

Supplement: Supplementary material 5 — Bayesian Trees [file bdj-11-e96438-s005.zip › MWI Appendix 5 - Bayesian Trees/Ceutorhynchinae bayesian tree.png]

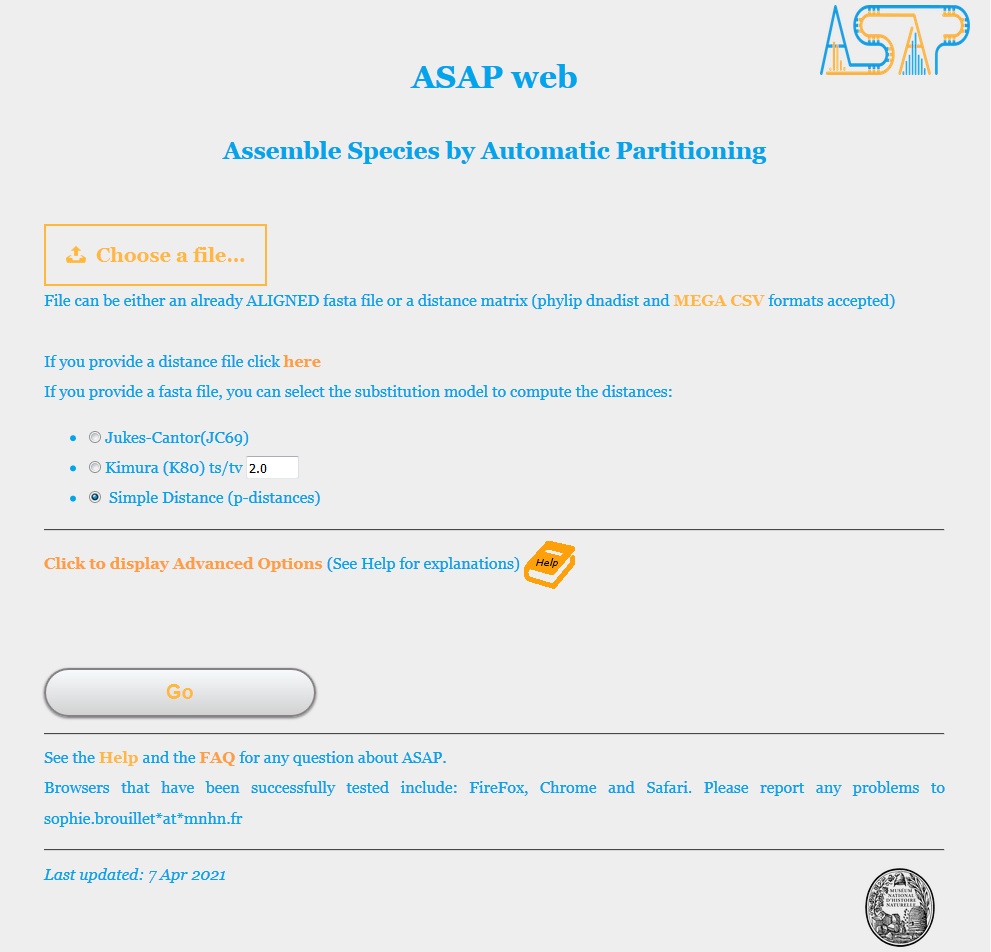

Supplement: Supplementary material 7 — ASAP analyses [file bdj-11-e96438-s007.zip › Suppl. material 7 - ASAP analyses/Apioninae - raw data and concordance evaluation/01 - ASAP input data/Settings.jpg]

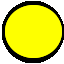

Supplement: Supplementary material 7 — ASAP analyses [file bdj-11-e96438-s007.zip › Suppl. material 7 - ASAP analyses/Apioninae - raw data and concordance evaluation/02 - ASAP html output/ASAP Results-Dateien/jaune.png]

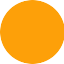

Supplement: Supplementary material 7 — ASAP analyses [file bdj-11-e96438-s007.zip › Suppl. material 7 - ASAP analyses/Apioninae - raw data and concordance evaluation/02 - ASAP html output/ASAP Results-Dateien/orange.png]

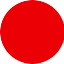

Supplement: Supplementary material 7 — ASAP analyses [file bdj-11-e96438-s007.zip › Suppl. material 7 - ASAP analyses/Apioninae - raw data and concordance evaluation/02 - ASAP html output/ASAP Results-Dateien/rouge.png]

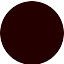

Supplement: Supplementary material 7 — ASAP analyses [file bdj-11-e96438-s007.zip › Suppl. material 7 - ASAP analyses/Ceutorhynchinae - raw data and concordance evaluation/02 - ASAP html output/ASAP Results-Dateien/noir.png]

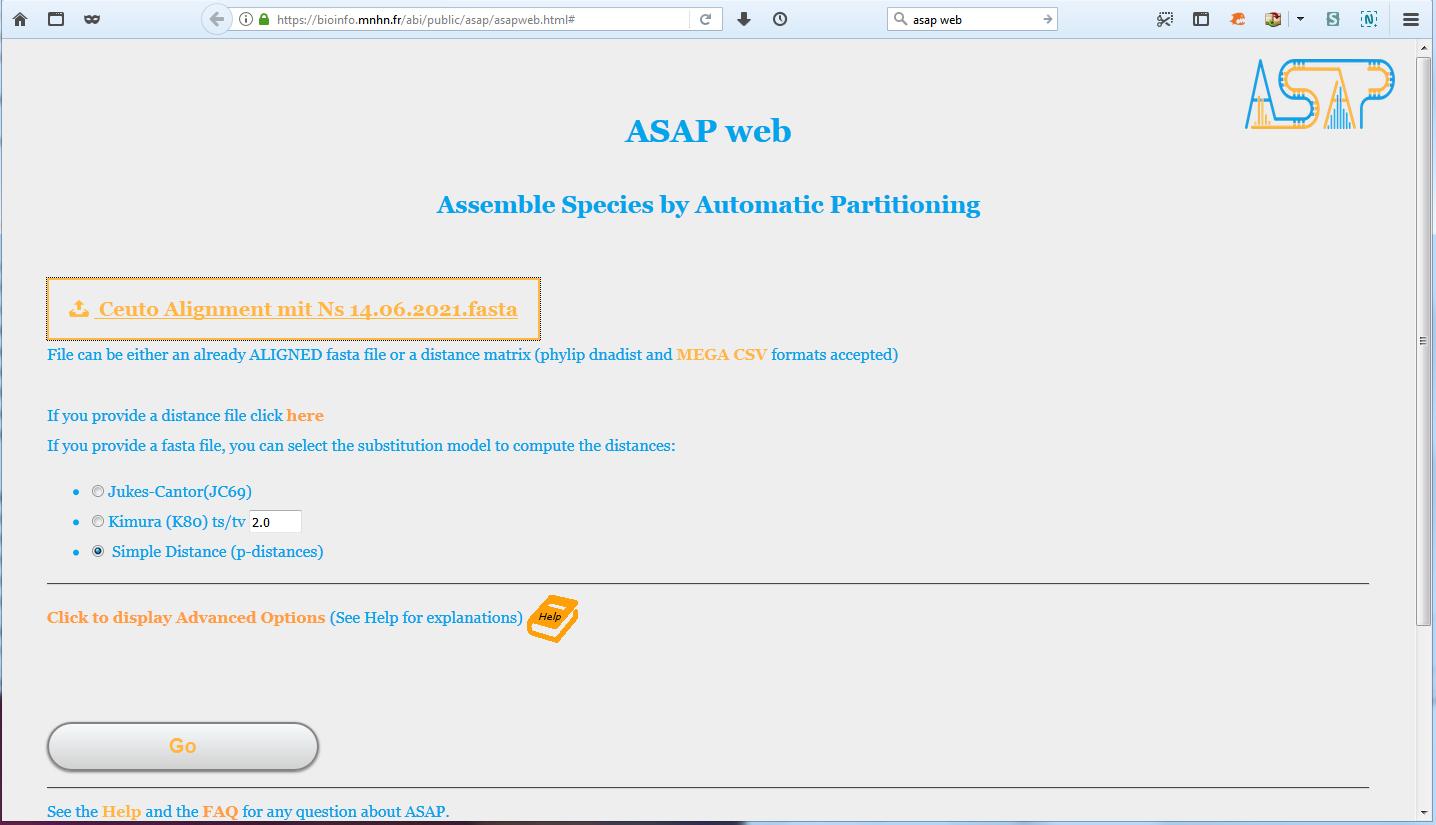

Supplement: Supplementary material 7 — ASAP analyses [file bdj-11-e96438-s007.zip › Suppl. material 7 - ASAP analyses/Ceutorhynchinae - raw data and concordance evaluation/xxx_müll/screenshot 01 - input settings.jpg]

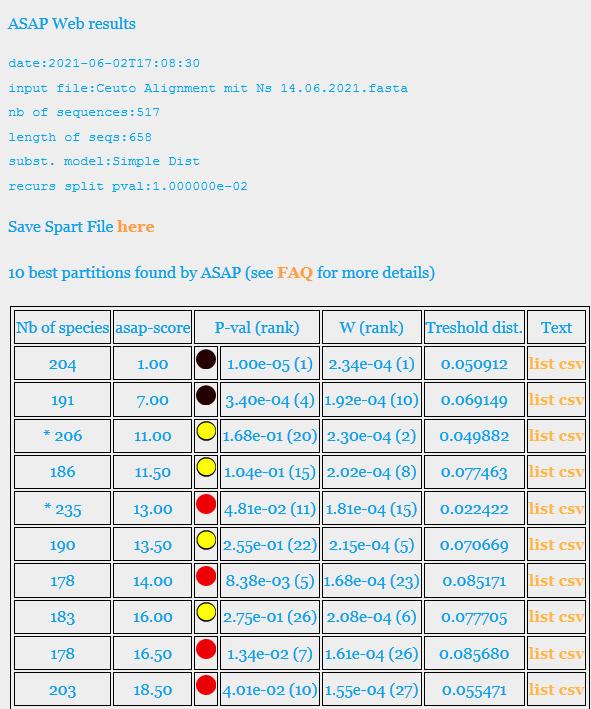

Supplement: Supplementary material 7 — ASAP analyses [file bdj-11-e96438-s007.zip › Suppl. material 7 - ASAP analyses/Ceutorhynchinae - raw data and concordance evaluation/xxx_müll/screenshot 02 - best partitions.jpg]

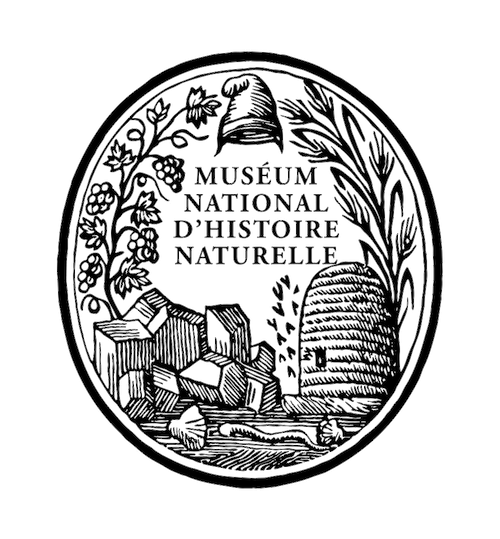

Supplement: Supplementary material 7 — ASAP analyses [file bdj-11-e96438-s007.zip › Suppl. material 7 - ASAP analyses/Cryptorhynchinae - raw data and concordance evaluation/02 - ASAP html output/ASAP FAQ-Dateien/museumb.png]

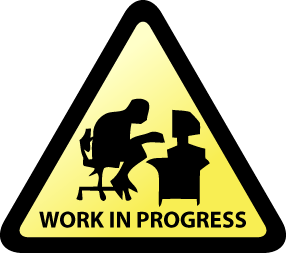

Supplement: Supplementary material 7 — ASAP analyses [file bdj-11-e96438-s007.zip › Suppl. material 7 - ASAP analyses/Cryptorhynchinae - raw data and concordance evaluation/02 - ASAP html output/ASAP FAQ-Dateien/wip-sign.png]
