# Supplementary material for: Molecular Weevil Identification Project: A thoroughly curated barcode release of 1300 Western Palearctic weevil species (Coleoptera, Curculionoidea)
Source: Biodivers Data J. 2023 Jan 24;11:e96438. doi: 10.3897/BDJ.11.e96438 (PMC10865102; doi:10.3897/BDJ.11.e96438)
Supplement: Supplementary material 6 — DiStats statistics [file bdj-11-e96438-s006.zip › Data Preparation.pdf]

DiStats data preparation step 1 of 2: assigning taxa to confidence groups

Confidence group 1: reference species dataset ("good species")

The confidence group 1 dataset contains valid taxa with clear morphological characters for reliable identification; no subspecies are known (currently or in the past); since synonyms indicate difficult species delineation, we allowed for 1 synomyn for Cryptorhynchinae and up to 3 synonyms for Ceutorhynchinae and Apioninae. Reason: Apioninae and Ceutorhynchinae weevils generally cover a much larger distribution area than Cryptorhynchinae species. Thus, for Apioninae and Ceutorhynchinae, two researchers might have discovered the same species independently. Thus a higher number of synonyms seem to be reasonable to assume and allow.

Confidence group 2: congener dataset (taxa with subspecies, many synonyms)

The confidence group 2 dataset contains valid taxa with known subspecies, regardless of the current taxonomic status; the species' identification is difficult or the group has been subject to several revisions; taxa with certain ambiguous morphological characters, which led to some or many synonyms: Cryptorhynchinae: more than 1 synonym, Ceutorhynchinae: more than 3 synonyms, Apioninae: more than 3 synonyms.

Confidence group 3: omitted species or specimen (taxonomic unresolved issues)

The confidence group 3 dataset contains taxa with an unclear species status and taxa with unclear or ambiguous identification ("cf."). Those were omitted in DiStats statistics. However, those are included in the tree and barcode release.

Only the sequences/specimens from confidence group 1 (reference species/good species) and confidence group 2 (congener dataset) were used as input data in DiStats statistics.

DiStats data preparation step 2 of 2: assigning geographical distribution groups

The geographical distribution of each species has been evaluated by literature research from two sources:
1) the Curculio Institute distribution maps (www.curci.de), which are updated with each new finding; the maps are provided as external supplement via Zenodo DOI 10.5281/zenodo.7150576
2) the textual information about each species distribution in Löbl and Smetana (2011, 2013)

With both information sources pooled, the maximum distribution range from the most distant geographical positions were measured with Google Earth's ruler function. In case of conflicting information that the CURCI specialists could not efficiently resolve, the larger distribution is assumed. Each species was to one out of **four defined distribution groups** (see Table below):

- island(s) distribution area (ISL)
- endemic continental distribution area (C1)
- medium continental distribution area (C2)
- large continental distribution area (C3)

distribution group	ISL (island)	C1 (endemic)	C2 (medium)	C3 (large)
Cryptorhynchinae	island(s)	up to 50 km	50 to **500 km**	500 km and above
Apioninae	island(s)	up to 50 km	50 to **2,000 km**	2,000 km and above
Ceutorhynchinae	island(s)	up to 50 km	50 to **2,000 km**	2,000 km and above

Table: defined groups for island(s) and continental distribution

Suppose the occurrence of a species is the result of a recent trade event or an invasive species status, the species is placed in its original geographical position regardless of its current locale.

Species from islands are assigned to distribution group ISL, irrespective of the island's size. Most Cryptorhynchinae weevils are wingless and show a smaller distribution range than Ceutorhynchinae and Apioninae weevils. Thus, the distribution ranges of the continental distribution groups C2 and C3 are adopted for the wingless Cryptorhynchinae. C2 to C3 transition value is decreased from 2,000 km to 500 km to reflect a more practical distribution area for Cryptorhynchinae. The combined information from Löbl catalogs and Google Earth maps with the measured maximum distribution for all taxa involved are provided as external supplement via Zenodo DOI 10.5281/zenodo.7150576. The values are transferred to the Excel spreadsheets in root of ZIP folder, first tab "reference species distribution".

<u>References</u>

- Löbl L, Smetana A (2011) 'Catalogue of the Coleoptera. Curculionoiodea I.' 1st edn. (Apollo Books: Stenstrup) 373.
- Löbl L, Smetana A (2013) 'Catalogue of the Coleoptera. Curculionoiodea II.' 1st edn. (Brill: Leiden & Boston) 700.

DiStats analysis

DiStats statistics per subfamily. Three sub-datasets (CO1 nucleotide alignments) were created for Cryptorhynchinae, Apioninae and Ceutorhynchinae (one for each). Only sequences from confidence group 1 (reference species/"good species") and group 2 are included. Sequences from confidence group 3 (questionable species status) were omitted. Infraspecific epithets were removed. The sub-datasets contain the following number of sequences: 1146 for Cryptorhynchinae, 342 for Apioninae and 516 for Ceutorhynchinae, representing species count as follows: 276 for Cryptorhynchinae, 114 for Apioninae and 200 for Ceutorhynchinae. The sub-datasets are calculated with the DiStats Perl script (Astrin et al. 2016). DiStats is started under MS Windows via the following CMD command:

perl distats.pl --num_threads=2 *xxx*.fasta yyy.out --print_dist_matrix
(xxx must be replaced by name of the input fasta file, yyy can be chosen).

The DiStats input fasta files are provided in Suppl. material 6 (subfolder "DiStats input"). The DiStats result files (tab delimited *.OUT files as txt spreadsheets) are provided in Suppl. material 6 (subfolder "DiStats output"). The p-distance value to the closest sister species for each taxon is listed in the row "Closest_Sp_Min_Dist." Only this minimum inter-specific value is used for subsequent data compilation (Excel spreadsheets, tab "DiStats_results").

DiStats data compilation after analysis

See data compilation in Excel spreadsheets (Suppl. material 6):

- DiStats statistics Apioninae.xlsx
- DiStats statistics Ceutorhynchinae.xlsx
- DiStats statistics Cryptorhynchinae.xls

Interspecific distances per genus and distribution. DiStats results were assembled: Only for the reference species, the p-distance values to each congener were taken to create genus lists with minimum interspecific distance values per geographic distribution group (the closest congener can be another reference species or a taxon from confidence group 2). Leaving out the data of confidence level group 2 would lead to an artificial rise of all p-distance values due to the smaller amount of available congeners in the dataset, comparable to creating artificial deep splits in trees when removing congeners.

The **average interspecific distance per genus** and the **minimum interspecific distances per genus** are presented in summarized tables in the Excel spreadsheets itself (tab "results"), but also in the main text as Figs. 5, 6, 7.

Reference

- Astrin JJ, Hofer H, Spelda J, Holstein J, Bayer S, Hendrich L, Huber BA, Kielhorn KH, Krammer HJ, Lemke M, Monje JC, Moriniere J, Rulik B, Petersen M, Janssen H, Muster C (2016) Towards a DNA Barcode Reference Database for Spiders and Harvestmen of Germany. *PLoS One* **11**(9): e0162624.
DOI: 10.1371/journal.pone.0162624.

Comments to the Excel spreadsheets in Suppl. material 6

Three Excel spreadsheets are provided in Suppl. material 6:

- DiStats statistics Cryptorhynchinae.xlsx
- DiStats statistics Apioninae.xlsx
- DiStats statistics Ceutorhynchinae.xlsx

The **first tab** ("reference species distribution") contains the taxon list with synonym counts and distribution range. The non-reference species are excluded via Excel filter in row "F"'. The distribution ranges were taken from maps of the Curculio Institute website and both Löbl catalogs (Löbl & Smetana 2011, 2013). The maps with distribution information are also provided as external supplement via Zenodo DOI 10.5281/zenodo.7150576.
The **second tab** ("DiStats_results") contains the entire DiStats output data. Rows that are not needed for the final result compilation are hidden. To unhide: mark all row captions, right-click on any row caption, and choose "unhide row"; only the values in row "F" are used: "Closest_Sp_Min_Dist." These values are the smallest available p-distances to the closest congener (often sister species). Those smallest available interspecific p-distance values primarily result from a taxon within the same genus. If there are no congeners from the same genus in the dataset, it will be a taxon from another genus.
The **tabs** island**,** endemic**,** medium*, and* large *contain the re*ference species for each distribution group and their closest p-distance value from the "DiStats_results" tab. The minimum, average and median p-distance values were calculated in Excel within those four distribution groups.
The **last tab** ("results") contains the summarized p-distance values per genus and distribution group.

Note: Most Excel functions, for example, "=average()", are language-dependant and provide error messages when opened on a different language version of MS Excel, all functions were replaced with their result values. In the last tab, "results," the previously calculated minimum and average p-distance values are copied from the tabs "*island*," "*endemic*," "*medium*," and "*large*."

If more details are of interest, for example, "which taxa is evaluated for distribution '*large*' of *Calacalles* with 14.3% average distance?" the taxa names are listed in the "*large*" tab (*Calacalles kabylianus* and *Calacalles theryi*). If the information about the closest congeners for those two taxa is of interest, please refer to tab "DiStats_results" row "A," and the closest congener is listed in row "B."

Additional information to Cryptorhynchinae taxa

Sicily island in Italy was defined as mainland for any weevil found exclusively in this area.

The below-listed Cryptorhynchinae taxa were excluded from the reference species dataset (confidence group 1), though they formally match the reference species' criteria.

Most of the below mentioned species are already known for exceptional small interspecific distances although showing sufficient morphological, ecological or even ethological characters for identification. One could state excluding formally good species with low interspecific p-distance values will insert a bias into the dataset, which is correct. We prefer conservative and safe values to misleading ones creating synonyms in the future.

Two species that could not be assigned to island distribution (ISL) or continental distribution (C1, C2 or C2) were excluded from the reference species dataset and placed into the congener dataset (confidence group 2): ***Acalles globulipennis*** (Portugal mainland, Canary Islands and Madeira, the origin of dispersal is uncertain). ***Dichromacalles boroveci*** can be found on Rhodos island und Samos island. Those islands are close to the mainland and just 200km distant from each other. The dispersion in the past is uncertain. At this stage, there is no possibility of grouping them into distribution groups ISL (island) or C1 (continental). Still, they are included in the DiStats statistics and thus can match as the closest congener for other reference species.

Seven Cryptorhynchinae species were excluded from the reference species dataset and placed into the congener dataset (confidence group 2), even they though formally match the requirements: ***Dendroacalles ornatus*** and ***Dendroacalles sigma*** (p-distance between both: 1.2%), ***Torneuma maderense*** and ***Torneuma korwitzi*** (p-distance between both: 2.3%), ***Torneuma feloi*** and ***Torneuma orbatum*** (p-distance between both: 3.5%), and ***Madeiracalles tristaensis***.

The ***Acalles sierrae* species complex** contains twelve, mostly highly endemic, species from Spanish and Moroccan mountain ranges (Schütte & Stüben 2015: 24, Fig. 7). Species delineation is difficult either morphology-wise or molecular-wise. Most species of the *Acalles sierrae* species complex show large p-distances but little morphological distinctness: *Acalles sierrae*, *A. parasierrae*, *A. guadarramaensis*, *A. alcarazensis*, *A. cazorlaensis*, *A. iblanensis*, *A. asniensis*, and *A. testensis.* The "***maraoensis* clade**" also belongs to *A. sierrae* complex, but - vice versa, shows very small p-distances but high morphological distinctness: *A. sarothamni*, *A. monasterialis*, *A. cytisi*, and *A. maraoensis*. Five species have been excluded from the reference species dataset and assigned to the congener dataset (confidence group 2): all species of the *maraoensis clade:* ***A. maraonsis, A. cytisi, A. monasterialis***, ***A. sarothamni,*** and also ***A. sierrae*** from the *sierrae* clade***.*** Remaining in reference species dataset (confidence group 1): *A. alcarazensis* Stüben, 2009 (endemic), *A. asniensis* Stüben, 2003 (endemic), *A. testensis* Stüben, 2003 (endemic), *A. cazorlaensis* Stüben, 2004 (endemic), *A. guadarramaensis* Stüben, 2004 (medium), *A. iblanensis* Stüben, 2015 (endemic), *A. parasierrae* Stüben, 2002 (medium).

Echinodera atlasensis Stüben, 2001 from Morocco was excluded from the reference species dataset and assigned to the congener dataset (confidence group 2). This taxon likely contains a cryptic species, which would bias the DiStats p-distance statistics. All four available sequences were obtained from MWI´s predecessor project. The specimens were destroyed during the DNA extraction process (*Echinodera atlasensis* specimen IDs and GenBank accessions: E-918-atl / GU213762, E-919-atl / GU213763, Mo-947-ant / GU213771, E-920-atl / MG322641).

Echinodera casablancaensis Stüben, 2001 was excluded from the reference species dataset (confidence group 1) and assigned to the congener dataset (confidence group 2) since it likely includes a cryptic species, which would bias the DiStats p-distance statistics.

Reference for remarks above

- Löbl L and Smetana A (2013) Catalogue of the Coleoptera. Vol. 8, Curculionoiodea II. 1st, 8. Brill, Leiden & Boston, 700 pp. [In English]. [ISBN 9789004252066].

The below-listed Cryptorhynchinae taxa were added to the reference species dataset (confidence group 1), though they do not match the criteria *formally*.

Ficusacalles ficvorator (Stüben, 2007) and ***Ficusacalles oceanicus*** (Stüben, 2002) were elevated from subspecies to species level in Stüben & Bayer 2015. Thus, these taxa formally do not match the "good species" criteria of no available (or former available) subspecies. Assigning both *Ficusacalles* species into the reference species dataset (confidence group 1) is helpful in this context; their species status was justified by both clear morphological characters and genetic distance.

Ficusacalles senilis (Wollaston, 1864) was the nominotypical of *F. senilis ficvorator* and *F. senilis oceanicus*. Both subspecies were elevated to species level in Stüben & Bayer 2015. Formally *F. senilis* does not match the reference species criteria because subspecies are known. Due to distinctive morphological characters, we have decided to place *F. senilis* into the reference species dataset (confidence group 1). For further information about geographical dispersal and host plant associations, please refer to Stüben & Behne 2015.

References for remarks above

- Stüben, PE and Bayer, C. (2015) New nomenclatural and taxonomic acts, and Comments (2015) - *SNUDEBILLER: Studies on taxonomy, biology and ecology of Curculionoidea* **16**(246): 1-8. URL: https://curci.de/institute/index.php?beitrag=246
- Stüben, P. and Behne, L. (2015) Die Curculionoidea (Coleoptera) La Palmas - *SNUDEBILLER: Studies on taxonomy, biology and ecology of Curculionoidea* **16**(242): 1-86. URL: https://curci.de/institute/index.php?beitrag=242

Cryptorhynchinae miscellaneous

The distribution area of ***Echinodera incognita*** is only 250 km when connecting the farthest points. The distribution area is not 700 km. Most likely Hoffmann (the collector) mixed up the collecting tubes.

Echinodera pseudovariegata has been classified as continental distributed species. Island distribution is likely based on drift events to the closest islands [personal communication between the first and second author].

Cryptorhynchus lapathi (Linnaeus, 1758) is one of the rare Cryptorhynchinae species able to fly. Its geographical distribution is widespread. Eight synonyms are known (Löbl & Smetana 2013: 231). Thus this taxa was assigned to the congener dataset (confidence group 2). Species with a geographically wide distribution range have a higher chance of being described several times than endemic ones.

225 Cryptorhynchinae taxa were counted as reference species (confidence group 1):

Acalles alcarazensis Stüben, 2009, *Acalles almeriaensis* Stüben, 2001, *Acalles asniensis* Stüben, 2003, *Acalles biokovoensis* Stüben, 2008, *Acalles breiti* A. & F. Solari, 1909, Acalles *cazorlaensis* Stüben, 2004, *Acalles dieckmanni* Péricart, 1989, *Acalles dubius* A. & F. Solari, 1907, *Acalles echinatus* (Germar, 1824), *Acalles fallax* Boheman, 1844, *Acalles gadorensis* Stüben, 2001, *Acalles guadarramaensis* Stüben, 2004, *Acalles granulimaculosus* Stüben, 2014, *Acalles iblanensis* Stüben, 2015, *Acalles kippenbergi* Dieckmann, 1982, *Acalles micros* Dieckmann, 1982, *Acalles misellus* Boheman, 1844, *Acalles ossetiensis* Stüben, 2018, *Acalles parasierrae* Stüben, 2002, *Acalles pilula* Wollaston, 1864, *Acalles ptinoides* (Marsham, 1802), *Acalles reitteri* Meyer, 1896, Acalles *sardiniaensis* Stüben, 2001, *Acalles sintraniensis* Stüben, 1999, *Acalles testensis* Stüben 2003, *Acalles tibialis* (Weise, 1891), *Acalles vorsti* Stüben, 2014, *Acallocrates colonnellii* Bahr, 2003, *Acallocrates denticollis* (Germar, 1824), *Acallocrates minutesquamosus* (Reiche, 1860), *Acallorneuma doderoi* A. & F. Solari, 1909, *Acallorneuma ibericum* Stüben, 2005, *Acallorneuma ingoi* G. Osella & Zuppa, 2002, *Acallorneuma mainardii* A. & F. Solari, 1909, *Acallorneuma montisalbi* G. Osella & Zuppa, 2002, *Acallorneuma reitteri* Mainardi, 1906, *Aeoniacalles aeonisimilis* (Stüben, 2000), *Aeoniacalles argillosus* (Boheman, 1837), *Aeoniacalles grancanariensis* (Stüben, 2000), *Aeoniacalles neptunus* (Wollaston, 1854), *Aeoniacalles tabladoensis* Stüben & Astrin, 2011, *Calacalles affinis* Bahr, 2000, *Calacalles atomarius* Bahr, 2000, *Calacalles bandamaensis* Stüben, 2012, Calacalles *citvorator* Stüben, 2015, *Calacalles exiguus* Bahr, 2000, *Calacalles fuerteventurensis* Bahr, 2000, *Calacalles hermigua* Stüben & Astrin, 2009, *Calacalles kabylianus* (Desbrochers des Loges, 1897), *Calacalles lepensis* Stüben, 2013, *Calacalles manriquei* Stüben, 2018, *Calacalles minutus* Bahr, 2000, *Calacalles montelunatus* Stüben, 2015, *Calacalles mulagua* Stüben, 2010, *Calacalles nataliae* Astrin & Stüben, 2009, *Calacalles palmensis* (Roudier, 1954), *Calacalles pumilio* Bahr, 2000, *Calacalles pusillus* Bahr, 2000, *Calacalles seticollis* (Wollaston, 1864), *Calacalles theryi* (Peyerimhoff, 1925), *Calacalles wollastoni* (Chevrolat, 1852), *Canariacalles alluaudi* (Uyttenboogaart, 1940), *Canariacalles lanzarotensis* (Stüben, 2000), *Caucasusacalles lederi* Stüben, 2018, *Coloracalles edoughensis* (Desbrochers, 1892), *Coloracalles humerosus* (Fairmaire, 1862), *Dendroacalles brevitarsis* (Wollaston, 1864), *Dendroacalles euphorbiacus* (Stüben, 2000), *Dendroacalles poneli* (Stüben, 2000), *Dendroacalles ruteri* (Roudier, 1954), *Dichromacalles*

albopictus (Jacquet, 1888), *Dichromacalles algecirasensis* Stüben, 2013, *Dichromacalles andalusiensis* Stüben, 2014, *Dichromacalles creticus* (Reitter, 1916), *Dichromacalles lentisci* (Chevrolat, 1861), *Dichromacalles querilhaci* (H. Brisout de Barneville, 1864), *Dichromacalles rolletii* (Germar, 1839), *Echinodera adriatica* Stüben, 2008, *Echinodera andalusiensis* Stüben, 2003, *Echinodera angulipennis* Wollaston, 1864, *Echinodera ariadnae* Bahr & Beyer, 2005, *Echinodera aspromontensis* Stüben, 2008, *Echinodera bargouensis* Stüben & Astrin, 2011, *Echinodera behnei* Stüben, 1998, *Echinodera brachati* Wolf, 2002, *Echinodera bulbosa* Stüben & Astrin, 2008, *Echinodera capiomonti* (H. Brisout de Barneville, 1864), *Echinodera ceutaensis* Stüben, 2002, *Echinodera cognita* Stüben, 2006, *Echinodera compacta* Wollaston, 1864, *Echinodera corcyrensis* Stüben, 2008, *Echinodera crenata* Wollaston, 1863, *Echinodera cyprica* Stüben, 2010, *Echinodera germanni* Stüben, 2003, *Echinodera graeca* Caldara, 1973, *Echinodera guacimara* Stüben & Germann, 2005, *Echinodera hoceimaensis* Stüben, 2018, *Echinodera hypocrita* (Boheman, 1837), *Echinodera ibleiensis* Stüben, 2003, *Echinodera ifranensis* Stüben, 2002, *Echinodera incognita* (A. Hoffmann, 1956), *Echinodera jandiaensis* Stüben, 2018, *Echinodera ketamaensis* Stüben, 2002, *Echinodera kratkyi* Stüben, 2018, *Echinodera kroumiriensis* Stüben, 2004, *Echinodera major* (A. & F. Solari, 1907), *Echinodera merkli* (Meyer, 1896), *Echinodera minosi* Bahr & Bayer, 2005, *Echinodera montana* Stüben & Astrin, 2011, *Echinodera nebrodiensis* Stüben, 2003, *Echinodera nuraghia* Stüben, 2009, *Echinodera ochsi* (F. Solari, 1952), *Echinodera orbiculata* Wollaston, 1864, *Echinodera orientalis* (A. & F. Solari 1907), *Echinodera pallida* Israelson, 1985, *Echinodera palmaensis* Stüben, 2000, *Echinodera pelionis* (Frieser, 1955), *Echinodera personata* Colonnelli, 1985, *Echinodera picta* Wollaston, 1864, *Echinodera pseudovariegata* Stüben, 1998, *Echinodera rifensis* Stüben, 2001, *Echinodera roudieri* Stüben, 1998, *Echinodera samosa* Germann, 2012, *Echinodera setosagracilis* Stüben, 2004, *Echinodera settefratelliensis* Stüben, 2005, *Echinodera siciliensis* Stüben, 2003, *Echinodera soumasi* Germann, Wolf & Schütte, 2015, *Echinodera spinosa* Stüben, 2006, *Echinodera suber* Stüben, 2001, *Echinodera tazzekaensis* Stüben, 2002, *Echinodera tellatlasensis* Stüben, 2002, *Echinodera tenoensis* Stüben, 2000, *Echinodera valida* (Hampe, 1864), *Echinodera variegata* (Boheman, 1837), *Echinodera varroi* Stüben, 2018, *Echinodera zaghouanensis* Stüben, 2004, *Echiumacalles anagaensis* (Stüben, 2000), *Elliptacalles baeticus* (Stüben, 2008), *Elliptacalles longus* (Desbrochers des Loges, 1892), *Ficusacalles ficvorator* (Stüben, 2007), *Ficusacalles oceanicus* (Stüben 2002), *Ficusacalles senilis* (Wollaston, 1864), *Kyklioacalles alcornocalensis* Stüben, Torres, Astrin, 2011, *Kyklioacalles anthyllis* Stüben, 2004, *Kyklioacalles apogeus* (Peyerimhoff, 1925), *Kyklioacalles astragali* Stüben, 2003, *Kyklioacalles atlasicus* Stüben & Astrin, 2010, *Kyklioacalles aubei* (Boheman, 1837), *Kyklioacalles bupleuri* Stüben, 2004, *Kyklioacalles characivorus* Stüben, 2005, *Kyklioacalles chaudoiri* (Hochhuth 1847), *Kyklioacalles erinaceus* Stüben, 2003, *Kyklioacalles euphorbiophilus* Stüben, 2003, *Kyklioacalles fissicollis* (Penecke, 1926), *Kyklioacalles flavomaculatus* Stüben, 2012, *Kyklioacalles granulicollis* (Tournier, 1875), *Kyklioacalles igualeja* Stüben, 2012, *Kyklioacalles maroccensis* (Stüben, 2001), *Kyklioacalles navieresi* (Boheman, 1837), *Kyklioacalles oblongus* Stüben, 2018, *Kyklioacalles olcesei* (Tournier, 1873), *Kyklioacalles oukaimedensis* Stüben, 2010, *Kyklioacalles plantapilosus* Stüben & Astrin, 2010, *Kyklioacalles provincialis* (A. Hoffmann, 1960), *Kyklioacalles reginae* Stüben, 2003, *Kyklioacalles reinosae* (H. Brisout de Barneville, 1867), *Kyklioacalles saccoi* (Colonnelli, 1973), *Kyklioacalles snassensis* Stüben, 2002, *Kyklioacalles solarii* (Fiori, 1903), *Kyklioacalles suturatus* (Dieckmann, 1983), *Kyklioacalles teter* (Boheman, 1844), *Kyklioacalles tidiquinensis* Stüben, 2002, *Kyklioacalles yestensis* Stüben, 2003, *Lauriacalles*

acutus (Wollaston, 1864), *Madeiracalles achadagrandensis* (Stüben, 2002), *Madeiracalles beelzebubi* Stüben & Kratky, 2018, *Madeiracalles cinereus* (Wollaston, 1860), *Madeiracalles coarctatus* (Wollaston, 1857), *Madeiracalles dispar* (Wollaston, 1854), *Madeiracalles histrionicus* (Wollaston, 1857), *Madeiracalles machadoi* (Stüben, 2006), *Madeiracalles portosantoensis* (Stüben, 2002), *Madeiracalles saxicola* (Wollaston, 1854), *Madeiracalles succulentus* Stüben, 2018, *Madeiracalles vau* (Wollaston, 1854), *Montanacalles nevadaensis* (Stüben, 2001), *Onyxacalles balearicus* Stüben, 2005, *Onyxacalles bermejaensis* Stüben, 2001, *Onyxacalles caucasicus* (Reitter, 1891), *Onyxacalles ganglbaueri* (A. & F. Solari 1907), *Onyxacalles georgius* Stüben & Krátky 2018, *Onyxacalles gibraltarensis* Stüben, 2002, *Onyxacalles luigionii* (A. & F. Solari, 1907), *Onyxacalles maginaensis* Stüben, 2004, *Onyxacalles neglectus* Kulbe, 1999, *Onyxacalles nuraghi* Stüben, 2012, *Onyxacalles portusveneris* (Mayet, 1903), *Onyxacalles ringeli* Kulbe, 1999, *Onyxacalles valencianus* Germann, 2005, *Onyxacalles verrucosus* (Wollaston, 1863), *Onyxacalles vilae* Stüben, 2012, *Pseudodichromacalles fernandezi* (Roudier, 1954), *Pseudodichromacalles pericallis* Stüben, 2018, *Pseudodichromacalles xerampelinus* (Wollaston, 1864), *Silvacalles carlinavorus* Stüben & Schütte, 2014, *Silvacalles cedroensis* (Kulbe, 2000), *Silvacalles instabilis* (Wollaston, 1864), *Silvacalles lepidus* (Kulbe, 2000), *Silvacalles lunulatus* (Wollaston, 1854), *Silvacalles mundus* (Wollaston, 1864), *Silvacalles nubilosus* (Wollaston, 1864), *Silvacalles pedestris* (Stüben, 2000), *Silvacalles tolpivorus* (Germann & Stüben, 2006), *Sonchiacalles muelleri* (Stüben, 2000), *Sonchiacalles silosensis* (Stüben, 2000), *Sonchiacalles sonchi* (Stüben, 2000), *Torneuma alexi* Stüben, 2018, *Torneuma aphroditae* (Germann & Stüben, 2006), *Torneuma* baeticum Stüben, 2007, *Torneuma cadizense* Stüben, 2016, *Torneuma caecum* Wollaston, 1860, *Torneuma desilvai* G. Osella & Zuppa, 1998, *Torneuma isambertoi* Stüben, 2016, *Torneuma karamani* (Formánek, 1912), *Torneuma picocasteloense* Stüben, 2002.

45 Cryptorhynchinae taxa were <u>not</u> counted as reference species (confidence group 2):

Acalles camelus (Fabricius, 1792), *Acalles cytisi* Stüben, 2004, *Acalles globulipennis* Wollaston, 1854, *Acalles lemur* (Germar, 1824), *Acalles minutissimus* (LeConte, 1876), *Acalles maraoensis* Stüben, 2001, *Acalles monasterialis* Stüben, 2004, *Acalles papei* A. & F. Solari, 1905, *Acalles parvulus* Boheman, 1837, *Acalles sablensis* Blatchley, 1920, *Acalles sarothamni* Stüben 2003, *Acalles sierrae* H. Brisout de Barneville, 1865, *Acalles sylvosus* Blatchley, 1916, *Acallorneuma sardiniense* G. Osella & Zuppa, 2002, *Aeoniacalles aeonii* (Wollaston, 1864), *Calacalles droueti* (Crotch, 1867), *Cryptorhynchus lapathi* (Linnaeus, 1758), *Dendroacalles fortunatus* (Wollaston, 1864), *Dichromacalles boroveci* Stüben, 1998, *Dichromacalles diocletianus* (Germar, 1817), *Dichromacalles dromedarius* (Boheman, 1844), *Dichromacalles tuberculatus* (Rosenhauer, 1856), *Echinodera atlasensis* Stüben, 2001, *Echinodera bellieri* (Reiche, 1860), *Echinodera brisouti* (Reitter, 1885), *Echinodera capbonensis* Stüben, 2004, *Echinodera casablancaensis* Stüben, 2001, *Echinodera hystrix* Wollaston, 1864, *Echinodera lusitanica* Stüben, 2014, *Echinodera paganettii* (F. Solari, 1952), *Echinodera peragalloi* (Chevrolat, 1863), *Echinodera pseudohystrix* Stüben, 2000, *Euscepes batatae* G. R. Waterhouse, 1849, *Kyklioacalles abstersus* (Boheman, 1837), *Kyklioacalles fausti* (Meyer, 1896), *Kyklioacalles punctaticollis* (Lucas, 1846), *Kyklioacalles pyrenaeus* (Boheman, 1844), *Kyklioacalles roboris* (Curtis, 1834), *Madeiracalles pulverosus* (Gemminger, 1871), *Madeiracalles terminalis* (Wollaston, 1854), *Madeiracalles tristaensis* (Stüben, 2002), *Silvacalles hakani* (Roudier, 1954), *Torneuma deplanatum* (Hampe, 1864), *Torneuma korwitzi* Stüben, 2015, *Torneuma mesegueri* Gonzalez, 1971

Additional information to Apioninae taxa

The below-listed Apioninae taxa were excluded from the reference species dataset (confidence group 1), though they formally match the reference species' criteria.

Ischnopterapion modestum (Germar, 1817), ***Ischnopterapion fairmairei*** (Wencker, 1864) and ***Ischnopterapion plumbeomicans plumbeomicans*** (Rosenhauer, 1856) have been excluded from the reference species dataset and placed into the congener dataset (confidence group 2). *Ischnopterapion plumbeomicans* does not match the formal criteria "no subspecies" but also might be a synonym of *Ischnopterapion modestum. Ischnopterapion fairmairei* might be a synonym of *Ischnopterapion modestum* as well. Unfortunately, there is no CO1 sequence available for *Ischnopterapion plumbeomicans pericarti* (Ehret, 1991). Please refer to Stüben et al. 2015 for a discussion of delineating the previously mentioned subspecies.

Catapion meieri (Desbrochers des Loges, 1901) is likely a junior synonym of *Catapion seniculus* Kirby, 1808. Thus, it was not assigned to the reference species and placed into the congener dataset. *Catapion seniculus* was also set to the congener dataset due to 7 known synonyms. Please refer to Stüben et al. 2015 for a discussion of delineating these two species.

Perapion oblongum (Gyllenhal, 1839) is likely a junior synonym of *Perapion curtirostre* (Germar, 1817). Thus, it was omitted from DiStats statistics (confidence group 3). *Perapion curtirostre* was also not assigned to the reference species dataset because of 4 known synonyms. Please refer to Stüben et al. 2015 for a discussion of delineating these two species. *Perapion curtirostre* was assigned to the congener dataset (confidence group 2).

Reference for remark above

- Stüben PE, Schütte A, Bayer Ch, Astrin JJ (2015) The Molecular Weevil Identification Project (Coleoptera: Curculionoidea), Part II - Towards an Integrative Taxonomy - *SNUDEBILLER: Studies on taxonomy, biology and ecology of Curculionoidea* **16**(237): 1-294. URL: https://www.curci.de/?beitrag=237

The below-listed Apioninae taxa were added to the reference species dataset (confidence group 1), though they do not match the criteria *formally*.

Kalcapion fortunatum (Roudier, 1963) and ***Kalcapion sagittiferum*** (Wollaston, 1854) were added to the list of reference species dataset (confidence group 1), while *Kalcapion semivittatum* (Gyllenhal, 1833) as the previous nominal taxon was not. *Kalcapion semivittatum* does not match the synonym rule for being a Ceutorhynchinae reference species (max. 3 synonyms allowed). The second author planned the changes of *Kalcapion fortunatum* and *Kalcapion sagittiferum* to species rank for many years based on morphological characters before molecular results became available and were finally published in 2017 (Stüben 2017**a**). Also, Karel Schön (Czech Republic) has undoubtedly suggested upgrading both taxa to species rank (Email communication between the second author and Karel Schön in 2017).

Pseudoprotapion astragali (Paykull, 1800) was added to the reference species dataset (confidence group 1), although it was previously the nominotypical taxon of *Pseudoprotapion astragali dumeei* (Hoffmann, 1957). *Pseudoprotapion dumeei* was elevated to species status in 2015 based on morphological characters only (Russel & Velazquez de Castro 2015).

Taeniapion atlanticum (Uyttenboogaart, 1935) was added to the reference species dataset. However, it was previously stated as subspecies of *Taeniapion urticarium* (Herbst, 1784) while *T. urticarium* has not been included in the reference species dataset due to 7 known synonyms. *Taeniapion urticarium atlanticum* has already been suggested to be upgraded to species status in Stüben 2017b. Formal rank change is in preparation by Stüben & Schön.

Protapion assimile assimile (Kirby, 1808) was added to the reference species dataset as ***Protapion assimile***, although a subspecies is known (*Protapion assimile ryei*). The ssp. *ryei* is only distributed in three islands of Great Britain: the Outer Hebrides, Orkney and Zetland. The *P. assimile* sequences in the MWI dataset were derived from specimens collected in Germany and Denmark (specimen IDs: 1031-PSP, 1517-PSP, 2588-PSP) and not Great Britain. It can be considered safe using the nominotypical taxon *Protapion assimile* as a reference species since the ssp. *reyi* is not included in the dataset. For an ecological assessment of both subspecies, see Sprick 2019 (Suppl.: 23).

References for remarks above

- Russell MI and Velazquez de Castro AJ (2015) A revision of the genus *Pseudoprotapion* Ehret, 1990 in the Iberian Peninsula, with description of a new species - *Boletín de la Sociedad Entomológica Aragonesa* **57**: 1-18.
- Sprick P (2019) Supplement to the Apionidae Keys I-V (DWD: Digital-Weevil-Determination for Curculionoidea of the West Palaearctic: Transalpina): Distribution, Host Plants, Biology. *SNUDEBILLER: Studies on taxonomy, biology and ecology of Curculionoidea* **20**(280): 1-29. URL: https://www.curci.de/?beitrag=280
- Stüben PE (2017a): Die Curculionoidea (Coleoptera) von den Inseln Madeiras und den Selvagens - *SNUDEBILLER: Studies on taxonomy, biology and ecology of Curculionoidea* **18**(261): 1-92. URL: https://www.curci.de/?beitrag=261
- Stüben PE (2017b): New nomenclatural and taxonomic acts, and Comments (2016/2017) - *SNUDEBILLER: Studies on taxonomy, biology and ecology of Curculionoidea* **18**(265): 1-16. URL: https://www.curci.de/?beitrag=265

Apioninae miscellaneous

Phrissotrichum tubiferum (Gyllenhal 1833) was only categorized as **continental** distributed species. In Löbl and Smetana (2011: 152), Madeira Archipelago (MR) is also listed as a finding spot, but not correct to our knowledge.

Malvapion malvae (Fabricius, 1775) was categorized as **continental** distributed species. In Löbl and Smetana (2011: 163), Madeira Archipelago (MR) is also listed, but most likely not the origin.

Perapion fallax (Wollaston, 1864) was categorized as **continental** species. Although 7 of 8 sequences in MWI have been collected at the Canaries or Madeira Archipelago and just one sequence derived from a Moroccan specimen (159-PST), we are sure the original distribution is north Africa but was likely introduced with *Rumex* or *Emex spinosa* plants. The island finding spots were mostly close to agricultural land.

Hemitrichapion rotundipenne: In contrast to Löbl and Smetana (2011: 168) and Alonso-Zarazaga (Alonso-Zarazaga et al. 2017), we refer to *H. rotundipenne* (Wollaston, 1854) as **valid species** after published re-synonymization in 2010 (Stüben & Behne 2010) and confirmed by morphological (Morris 2011) and molecular data (Stüben et al. 2015).

Catapion pubescens (Kirby, 1811) was categorized as **continental** distributed species but has been introduced to Tenerife island recently. There is only one sequence from the Czech Republic in the MWI dataset (specimen ID 2363-JKR, GenBank MK892087).

Ischnopterapion virens (Herbst, 1797) was categorized as **mainland** distributed species, although it can be found in the Canary Islands as well.

<u>References for remarks above</u>

- Alonso-Zarazaga MA, Barrios H, Borovec R, Bouchard P, Caldara R, Colonnelli E, Gültekin L, Hlaváč P, Korotyaev B, Lyal C, Machado A, Meregalli M, Pierotti H, Ren L, Sánchez-Ruiz M, Sforzi A, Silfverberg H, Skuhrovec J, Trýzna M, Yunakov N (2017) Cooperative Catalogue of Palaearctic Coleoptera Curculionoidea. 1st, 8. Monografías electrónicas S.E.A., Zaragoza, Spain, 729 pp. [In English]. URL: https://zoobank.org/References/911EF526-33F0-4970-8EC2-A7F5AC1E1D3D
- Morris MG (2011) The Apionidae (Coleoptera) of the Canary Islands, with particular reference to the contribution of T. Vernon Wollaston. *Acta Entomologica Musei Nationalis Pragae* 51(1): 157–182. [In English]. URL: http://www.aemnp.eu/PDF/51_1/51_1_157.pdf
- Löbl L, Smetana A (2011) Catalogue of the Coleoptera. Curculionoiodea I. Vol. 7,Apollo Books, Stenstrup, 373 pp. [ISBN: 9788788757934]
- Stüben PE and Behne L (2010) Hemitrichapion gomerense sp.n. (Coleoptera: Curculionoidea: Apionidae). *Weevil News* **53**: 1-3. URL: https://www.curci.de/?beitrag=162
- Stüben PE, Schütte A, Bayer Ch, Astrin JJ (2015): The Molecular Weevil Identification Project (Coleoptera: Curculionoidea), Part II - Towards an Integrative Taxonomy - *SNUDEBILLER: Studies on taxonomy, biology and ecology of Curculionoidea* **16**(237): 1-294. URL: https://www.curci.de/?beitrag=237

79 Apioninae taxa were categorized as reference species (confidence group 1):

Aizobius robustirostris (Desbrochers des Loges, 1870), *Aizobius sedi* (Germar, 1818), *Alocentron curvirostre* (Gyllenhal, 1833), *Apion cruentatum* Walton, 1844, *Apion frumentarium* (Linnaeus, 1758), Apion *rubiginosum* Grill, 1893, *Aspidapion acerifoliae* Suppantschitsch, 1996, *Aspidapion validum* (Germar, 1817), *Catapion pubescens* (Kirby, 1811), *Catapion seriatosetosulum* (Wencker, 1864), *Ceratapion armatum* (Gerstaecker, 1854), *Ceratapion austriacum* (Wagner, 1904), *Ceratapion calcaratum* (Wollaston, 1864), *Ceratapion cylindricolle* (Gyllenhal, 1839), *Ceratapion damryi* (Desbrochers des Loges, 1894), *Cistapion cyanescens* (Gyllenhal, 1833), *Cyanapion columbinum* (Germar, 1817), *Cyanapion gyllenhalii* (Kirby, 1808), *Cyanapion platalea* (Germar, 1817), *Cyanapion spencii* (Kirby, 1808), *Diplapion confluens* (Kirby, 1808), *Diplapion stolidum* (Germar, 1817), *Diplapion westwoodi* (Wollaston, 1864), *Eutrichapion punctiger* (Paykull, 1792), *Exapion corniculatum* (Germar, 1817), *Exapion* difficile (Herbst, 1797), *Exapion formaneki* (Wagner, 1929), *Exapion uliciperda* Pandellé, 1867, *Hemitrichapion pavidum* (Germar, 1817), *Hemitrichapion waltoni* (Stephens, 1839), *Holotrichapion pullum* (Gyllenhal, 1833), *Holotrichapion rotundipenne* (Wollaston, 1854), *Holotrichapion wollastoni* (Chevrolat, 1852), *Hoplopodapion poupillieri* (Wencker, 1864), *Ischnopterapion virens* (Herbst, 1797), *Ixapion variegatum* (Wencker, 1864), Kalcapion *fortunatum* (Roudier, 1963), *Kalcapion pallipes* (Kirby, 1808), *Kalcapion sagittiferum* (Wollaston, 1854), *Lepidapion curvipilosum* (Wagner, 1908), *Lepidapion senex* (Wollaston, 1864), *Malvapion malvae* (Fabricius, 1775), *Loborhynchapion amethystinum* (Miller, 1857), *Omphalapion hookerorum* (Kirby, 1808), *Omphalapion rhodopense* (Angelov, 1962), *Onychapion tamarisci* (Gyllenhal, 1839), *Oryxolaemus flavifemoratus* (Herbst, 1797), *Oryxolaemus scabiosus* (Weise, 1889), *Oxystoma craccae* (Linnaeus, 1767), *Oxystoma opeticum* (Bach, 1854), *Oxystoma subulatum* (Kirby, 1808), *Perapion fallax* (Wollaston, 1864), *Phrissotrichum tubiferum* (Gyllenhal, 1833), *Phrissotrichum tubuliferum* (Wollaston, 1864), *Protapion assimile* (Kirby, 1808), *Protapion dissimile* (Germar, 1817), *Protapion filirostre* (Kirby, 1808), *Protapion gracilipes* (Dietrich, 1857), *Protapion laevicolle* (Kirby, 1811), *Protapion nigritarse* (Kirby, 1808), *Protapion ononidis* (Gyllenhal, 1827), *Protapion ruficroides* (Dieckmann, 1973), *Protopirapion atratulum* (Germar, 1817), *Pseudapion fulvirostre* (Gyllenhal, 1833), *Pseudapion moschatae* (A. Hoffmann, 1938), *Pseudapion rufirostre* (Fabricius, 1775), *Pseudaplemonus artemisiae* (Morawitz, 1861), *Pseudoperapion brevirostre* (Herbst, 1797), *Pseudoprotapion astragali* (Paykull, 1800), *Pseudostenapion simum* (Germar, 1817), *Rhopalapion longirostre* (Olivier, 1807), *Stenopterapion argamani* Friedman & Freidberg, 2007, *Stenopterapion meliloti* (Kirby, 1808), *Stenopterapion tenue* (Kirby, 1808), *Synapion ebeninum* (Kirby, 1808), *Taeniapion atlanticum* (Uyttenboogaart, 1935), *Taeniapion delicatulum* (Wollaston, 1857), *Taeniapion rufulum* (Wencker, 1864), *Taphrotopium sulcifrons* (Herbst, 1797), *Trichopterapion holosericeum* (Gyllenhal, 1833)

32 Apioninae taxa were not categorized as reference species (confidence group 2):

Aspidapion aeneum (Fabricius, 1775), *Aspidapion radiolus* (Marsham, 1802), *Betulapion simile* (Kirby, 1811), *Catapion meieri* (Desbrochers des Loges, 1901), *Catapion seniculus* (Kirby, 1808), *Ceratapion gibbirostre* (Gyllenhal, 1813), *Eutrichapion ervi* (Kirby, 1808), *Exapion fuscirostre fuscirostre* (Fabricius, 1775), *Eutrichapion viciae* (Paykull, 1800), *Eutrichapion vorax* (Herbst, 1797), *Hemitrichapion wagneri gomerense* Stüben & Behne, 2010, *Hemitrichapion wagneri wagneri* (Flach, 1906), *Holotrichapion aethiops* (Herbst, 1797), *Holotrichapion ononis* (Kirby, 1808), *Holotrichapion pisi* (Fabricius, 1801), *Ischnopterapion fairmairei* (Wencker, 1864), *Ischnopterapion loti* (Kirby, 1808), *Ischnopterapion modestum* (Germar, 1817), *Ischnopterapion plumbeomicans plumbeomicans* (Rosenhauer, 1856), *Kalcapion semivittatum* (Gyllenhal, 1833), *Melanapion minimum* (Herbst, 1797), *Oxystoma pomonae* (Fabricius, 1798), *Perapion curtirostre* (Germar, 1817), *Perapion marchicum* (Herbst, 1797), *Perapion violaceum* (Kirby, 1808), *Protapion apricans* (Herbst, 1797), *Protapion fulvipes* (Geoffroy, 1785), *Protapion trifolii* (Linnaeus, 1768), *Squamapion elongatum* (Germar, 1817), *Squamapion flavimanum* (Gyllenhal, 1833), *Taeniapion urticarium* (Herbst, 1784)

1 Apioninae taxa was omitted from DiStats statistics (confidende group 3):

Perapion oblongum (Gyllenhal, 1839)

Additional information to Ceutorhynchinae taxa

The below-listed Ceutorhynchinae taxa were excluded from the reference species dataset (confidence group 1), though they formally match the reference species' criteria.

In contrast to Löbl and Smetana (2013), we have listed ***Microplontus melanostigma*** (Marsham, 1802) as valid species and not as a synonym of ***Microplontus rugulosus*** (Herbst, 1795). Further information can be found in Morris & Barclay 2015. Due to its problematic species status, we have omitted *Microplontus melanostigma* from the DiStats statistics (assigned to confidence group 3).

We think ***Mogulones uncipes*** (Korotayev, 1980) is a synonym or subspecies of ***Mogulonus larvatus*** (Schultze, 1897). We have omitted *M. uncipes* from the DiStats statistics (both sequences of *M. uncipes* were set to confidence group 3: MK891286/1239-PST, MK891280/1230-PST) but are included in the NJ tree and Bayesian tree. Since they were excluded from the DiStats statistics, they were not considered congeners of *M. larvatus* in the DiStats statistics. The p-distance between *M. larvatus* and *M. uncipes* is just 5.17%. The next closest congener is *Mogulones andreae*. Genetic distances to *M. andreae* are as follows: *M. uncipes* vs. *M. andreae*: 12.2% and *M. larvatus* vs. *M. andreae*: 12.5%.
Korotayev used a **single specimen** from France for the description of *Mogulones uncipes*. The second author, Peter Stüben, could not make a clear morphological delineation based on the aedeagi drawings from the first description.
Jiri Kratky could compare a photo of the *M. uncipes* holotype with two specimens he collected. He states in email correspondence that *M. uncipes* is clear to distinguish and also mentioned the two specimens of *M. uncipes* from Western Europe are likely *Mogulones* cf. *uncipes.*, but not a synonym of *M. larvatus.* He also stated, "I don't think *Mogulones uncipes* is a synonym to *Mogulones larvatus*. We have only a picture of the holotype of *M. uncipes* to compare with the collected specimens for MWI, but those from Portugal (MK891286/1239-PST, MK891280/1230-PST) are exactly the same. Mainly the large male mucro on the middle tibiae is quite distinctive and is never present in the typical *M. larvatus* (I have a big lot of specimens). Also, the host plant is different. The difference of about 5% [between *M. uncipes* and *M. larvatus*] is not large but also not so small. The shape of the aedeagus is not so essential. It is quite variable in *Mogulones*. We need more specimens to separate the "*larvatus*" from Ibero-Moroccan territory, living on *Echium* and being also genetically about 10% from the typical *M. larvatus*. However, I can not find stable morphological differences."

We are sure the endemic distributed ***Rhinoncus smreczynskii*** H. Wagner, 1937 is a junior synonym of *Rhinoncus perpendicularis* (Reich, 1797). The molecular data confirms this assumption. There are even two *R. smreczynskii* (2425-JKR, 327-JKR) and two *R. perpendicularis* (386-JKR, 273-PSP) having the exact same CO1 sequence with 0% p-distance. Besides this, there are already 9 known synonyms of *Rhinoncus perpendicularis* listed in Löbl and Smetana (2013: 212). Although ***Rhinoncus smreczynskii*** formally fits the good species criteria, we excluded *R. smreczynskii* from the reference species dataset (confidence group 1) and placed it into the congener dataset (confidence group 2), the same group as *Rhinoncus perpendicularis*.

Ceutorhynchus wellschmiedi Dieckmann, 1979 is likely a junior synonym of ***Ceutorhynchus chalybaeus*** Germar, 1824. It matches the criteria for being assigned to the reference species list (confidence group 1). Still, it was omitted from the DiStats statistics and

placed in confidence group 3 (dismissed in DiStats statistics) for not distorting the distance calculation of *C. chalybaeus.* Further details have been discussed already in Stüben et al. 2015.

Ranunculiphilus pseudinclemens (Dieckmann, 1969) might be a junior synonym of *Ranunculiphilus faeculentus* (Gyllenhal, 1837). Thus the only sequence (2413-JKR/MK892134) has been omitted from the DiStats statistics and placed into confidence group 3 for not distorting the distance calculation of *R. faeculentus*. From our perspective, there are no morphologic differences and hardly any differences in their aedeagi to support the species' status. The low p-distance value of 2.6% to its sister species also points in this direction. Of course, a recent split (very young species) could also be the reason.

References for remarks above

- Löbl L and Smetana A (2013) Catalogue of the Coleoptera. Vol. 8, Curculionoiodea II. 1st, 8. Brill, Leiden & Boston, 700 pp. [In English]. [ISBN 9789004252066].
- Morris M and Barclay M (2015) On the nomenclature of *Microplontus melanostigma* (Marsham, 1802), the weevil formerly referred to as *Microplontus rugulosus* (Herbst, 1795) (Curculionidae) and its synonyms, with lectotype and neotype designations. *The Coleopterist* **24:** 133-139.
- Stüben PE, Schütte A, Bayer Ch, Astrin JJ (2015) The Molecular Weevil Identification Project (Coleoptera: Curculionoidea), Part II - Towards an Integrative Taxonomy. *SNUDEBILLER: Studies on taxonomy, biology and ecology of Curculionoidea* **16**(237): 1-294. URL: https://www.curci.de/?beitrag=237

One below-listed Ceutorhynchinae taxon was added to the reference species dataset (confidence group 1), though it does not match the criteria *formally*.

In contrast to the catalog of Löbl and Smetana (2013) we have assigned ***Aphytobius veronicae*** (Frivaldszky, 1884) to the reference species dataset. It was resynonymized recently by Kratky (2015). The resynonymisation (stat. rev.) we have counted as "1 synonym" in the DiStats statistics data compilation spreadsheet of Ceutorhynchinae.

References for remarks above

- Kratky J (2015) *Aphytobius veronicae* (Frivaldszky, 1884) (Ceutorhynchinae, Hypurini) – species status revised. *SNUDEBILLER: Studies on taxonomy, biology and ecology of Curculionoidea* **16**(245): 1-9. URL: https://www.curci.de/?beitrag=245

- Löbl L and Smetana A (2013) Catalogue of the Coleoptera. Vol. 8, Curculionoiodea II. 1st, 8. Brill, Leiden & Boston, 700 pp. [In English]. [ISBN 9789004252066].

Ceutorhynchinae miscellaneous

Two sequences from the *Ceutorhynchus erysimi* species complex were renamed to cf. by the first author: ***Ceutorhynchus* cf. *erysimi*** (1580-JKR) and ***Ceutorhynchus* cf. *contractus*** (1581-JKR), both from Morocco. They were omitted from DiStats statistics (placed into confidence group 3). Most likely, both species were previously wrongly synonymized, and re-synonymizations (stat. rev.) for both species need to be carried out (in litt. Jiri Kratky, CZ).

One sequence of *Ceutorhynchus leucorhamma* was renamed to cf. by the first author and omitted in DiStats statistics (placed into confidence group 3): ***Ceutorhynchus* cf. *leucorhamma*** (1086-JKR). The second author and Jiri Kratky were uncertain regarding its identification.

One sequence of ***Ceutorhynchus hirtulus*** (2138-JKR) has been renamed by the first author to *Ceutorhynchus hirtulus* **det. unc.** and omitted from DiStats statistics (placed into confidence group 3). From a molecular point of view, 1086-JKR is most likely a *Ceutorhynchus varius*. Reliable identification can only be made with the male aedeagus, which is impossible on this female specimen.

As previously published, the genus **Datonychus** Wagner, 1944 is grouped [in NJ tree] within species of the genus ***Mogulones*** Reitter, 1916 (Stüben et al. 2015). Since we believe the genus *Datonychus* needs to be synonymized with *Mogulones*, we have combined the genera *Datonychus* and *Mogulones* in the statistics to a "combined genus."

We have defined the distribution range for ***Micrelus ferrugatus*** and ***Mogulones grisescens*** as continental distribution although they can be collected in the Canaries nowadays. We are sure they have been introduced to the Canaries in the past century.

Mogulones beckeri (Schultze, 1900) was counted as mainland species in the DiStats statistics, but it is also distributed in Cyprus island.

Mogulones grisescens (Pic, 1940) was defined as mainland species in the DiStats statistics. It was described from Egypt and is mainly distributed in the Western Mediterranean. It was introduced to Spain and the Canaries, and later to Madeira and the Azores.

Mogulones peregrinus (Gyllenhal, 1837) was counted as mainland species, but it can also be collected on Malta island within the Mediterranean Sea.

Two specimens were named ***Hesperorrhynchus lineatotessellatus x glutinosus*** because those were determined and verified as *H. glutinosus* but feature a COI barcode sequence of *H. lineatotessellatus:* 1936-PST / KT823492 and 2773-PST / KT823496. With a high probability, *H. glutinosus* is prone to mitochondrial introgression from *H. lineatotessellatus* because the finding spots are close. Both contradictory sequences have been omitted from the DiStats statistics (placed into confidence group 3). For more details, please refer to Stüben & Kratky 2016. *Hesperorrhynchus lineatotessellatus* (Wollaston, 1854) and its new sister species *H. glutinosus* Stüben, 2016 were evaluated as reference species.

Reference for the previous remarks

- Stüben PE, Schütte A, Bayer, Ch, Astrin JJ (2015) The Molecular Weevil Identification Project (Coleoptera: Curculionoidea), Part II - Towards an Integrative Taxonomy. *SNUDEBILLER: Studies on taxonomy, biology and ecology of Curculionoidea* **16**(237): 1-294. URL: https://www.curci.de/?beitrag=237

166 Ceutorhynchinae taxa were categorized as reference species (confidence group 1):

Aphytobius sphaerion (Boheman, 1845), *Aphytobius veronicae* (Frivaldszky, 1884), *Auleutes* epil*obii* (Paykull, 1800), *Barioxyonyx retectus* (Peyerimhoff, 1925), *Barioxyonyx tournieri* (Fourcroi, 1891), *Brachiodontus alpinus* (Hampe, 1867), *Brachiodontus reitteri* (Weise, 1878), *Ceutorhynchus aeneicollis* Germar, 1824, *Ceutorhynchus alliariae* C. Brisout de Barneville, 1860, *Ceutorhynchus alyssi* Peyerimhoff, 1925, *Ceutorhynchus arator* Gyllenhal, 1837, *Ceutorhynchus assimilis* (Paykull, 1792), *Ceutorhynchus atomus* Boheman, 1845, *Ceutorhynchus barbareae* Suffrian, 1847, *Ceutorhynchus carinatus* Gyllenhal, 1837, *Ceutorhynchus chalybaeus* Germar, 1824, *Ceutorhynchus chlorophanus* Rouget, 1857, *Ceutorhynchus coarctatus* Gyllenhal, 1837, *Ceutorhynchus coerulescens* Gyllenhal, 1837, *Ceutorhynchus constrictus* (Marsham, 1802), *Ceutorhynchus davidyani* Korotyaev, 1989, *Ceutorhynchus descurainiae* Stüben, 2016, *Ceutorhynchus dieckmannianus* Colonnelli, 1987, *Ceutorhynchus dubius* C. Brisout de Barneville, 1883, *Ceutorhynchus granulicollis* C. G. Thomson, 1865, *Ceutorhynchus hampei* C. Brisout de Barneville, 1869, *Ceutorhynchus hirtulus* Germar, 1824, *Ceutorhynchus hutchinsiae* Tempère, 1975, *Ceutorhynchus ignitus* Germar, 1824, *Ceutorhynchus inaffectatus* Gyllenhal, 1837, *Ceutorhynchus intersetosus* Weise, 1883, *Ceutorhynchus jucundus* Colonnelli, 2005, *Ceutorhynchus leprieuri* C. Brisout de Barneville, 1881, *Ceutorhynchus leucorhamma* Rosenhauer, 1856, *Ceutorhynchus libertorum* Colonnelli, 2005, *Ceutorhynchus liliputanus* Schultze, 1898, *Ceutorhynchus lukesi* Tyl, 1914, *Ceutorhynchus merkli* Korotyaev, 2001, *Ceutorhynchus moraviensis* (Dieckmann, 1966), *Ceutorhynchus napi* Gyllenhal, 1837, *Ceutorhynchus nevadensis* A. Hoffmann, 1960, *Ceutorhynchus nigritulus* Schultze, 1897, *Ceutorhynchus niyazii* A. Hoffmann, 1957, *Ceutorhynchus obstrictus* (Marsham, 1802), *Ceutorhynchus pallidactylus* (Marsham, 1802), *Ceutorhynchus paroliniae* Kratky, 2016, *Ceutorhynchus parvulus* C. Brisout de Barneville, 1869, *Ceutorhynchus pectoralis* Weise, 1895, *Ceutorhynchus pervicax* Weise, 1883, *Ceutorhynchus picitarsis* Gyllenhal, 1837, *Ceutorhynchus plumbeus* C. Brisout de Barneville, 1869, *Ceutorhynchus pulvinatus* Gyllenhal, 1837, *Ceutorhynchus pumilio* (Gyllenhal, 1827), *Ceutorhynchus puncticollis* Boheman, 1845, *Ceutorhynchus rapae* Gyllenhal, 1837, *Ceutorhynchus resedae* (Marsham, 1802), *Ceutorhynchus rhenanus* (Schultze, 1895), *Ceutorhynchus roberti* Gyllenhal, 1837, *Ceutorhynchus sardeanensis* Schultze, 1903, *Ceutorhynchus scrobicollis* Neresheimer & Wagner, 1924, *Ceutorhynchus sisymbrii* (Dieckmann, 1966), *Ceutorhynchus sophiae* Gyllenhal, 1837, *Ceutorhynchus squamulosus* C. Brisout de Barneville, 1869, *Ceutorhynchus striatellus* Schultze, 1900, *Ceutorhynchus sulcatus* C. Brisout de Barneville, 1869, *Ceutorhynchus syrites* Germar, 1824, *Ceutorhynchus tangerianus* Schultze, 1900, *Ceutorhynchus turbatus* Schultze, 1903, *Ceutorhynchus varius* Rey, 1895, *Ceutorhynchus wagneri* Smreczynski, 1937, *Coeliodinus rubicundus* (Herbst, 1795), *Datonychidius tener* (Reitter, 1888), *Datonychus delicatulus* (Hustache, 1946), *Datonychus maurus* (Schultze, 1899), *Datonychus paszlavszkyi* (Kuthy, 1890), *Drupenatus nasturtii* (Germar, 1824), *Eubrychius velutus* (Beck, 1817), *Eucoeliodes*

mirabilis (A. Villa & G. B. Villa, 1835), *Glocianus distinctus* (C. Brisout de Barneville, 1870), *Glocianus granulithorax* (Schultze, 1900), *Glocianus moelleri* (C. G. Thomson, 1868), *Glocianus punctiger* (C. R. Sahlberg, 1835), *Hadroplontus litura* (Fabricius, 1775), *Hadroplontus trimaculatus* (Fabricius, 1775), *Hesperorrhynchus glutinosus* Stüben, 2016, *Hesperorrhynchus hesperus* (Wollaston, 1864), *Hesperorrhynchus lineatotessellatus* (Wollaston, 1854), *Hesperorrhynchus palmensis* Kratky, 2016, *Hesperorrhynchus phytobioides* (Wollaston, 1864), *Homorosoma validirostre* (Gyllenhal, 1837), *Marmaropus besseri* Gyllenhal, 1837, *Mesoxyonyx sicardi* (Hustache, 1931), *Micrelus ericae* (Gyllenhal, 1813), *Micrelus ferrugatus* (Perris, 1847), *Microplontus campestris* (Gyllenhal, 1837), *Microplontus millefolii* (Schultze, 1897), *Mogulones abbreviatulus* (Fabricius, 1792), *Mogulones andreae* Desbrochers des Loges, 1902, *Mogulones angulicollis* (Schultze, 1897), *Datonychus angulosus* (Boheman, 1845), *Datonychus arquata* (Herbst,1795), *Mogulones asperifoliarum* (Gyllenhal, 1813), *Mogulones austriacus* (C. Brisout de Barneville, 1869), *Mogulones beckeri* (Schultze, 1900), *Mogulones biondii* Colonnelli, 1990, *Mogulones cingulatus* (Schultze, 1897), *Mogulones crucifer* (Pallas, 1771), *Mogulones cynoglossi* (Frauenfeld, 1866), *Mogulones deiectus* Colonnelli, 1992, *Mogulones dimidiatus* (J. Frivaldszky, 1865), *Mogulones euphorbiae* (C. Brisout de Barneville, 1866), *Mogulones gratiosus* (C. Brisout de Barneville, 1869), *Mogulones grisescens* (Pic, 1940), *Mogulones javetii* (Gerhardt, 1867), *Mogulones larvatus* (Schultze, 1897), *Mogulones pallidicornis* (Gougelet & H. Brisout de Barneville, 1860), *Mogulones peregrinus* (Gyllenhal, 1837), *Mogulones pseudopollinarius* (Har. Lindberg, 1950), *Mogulones raphani* (Fabricius, 1792), *Mogulones soricinus* (C. Brisout de Barneville, 1869), *Mogulonoides radula* (Germar, 1824), *Neoglocianus albovittatus* (Germar, 1824), *Neophytobius granatus* (Gyllenhal, 1835), *Oprohinus suturalis* (Fabricius, 1775), *Oreorrhynchaeus alpicola* Otto, 1894, *Parethelcus nesicola* Colonnelli, 1990, *Paroxyonyx audisioi* Colonelli, 2005, *Paroxyonyx cinctus* (Chevrolat, 1861), *Paroxyonyx fallaciosus* (Desbrochers des Loges, 1896), *Paroxyonyx imitator* (Wagner, 1928), *Pelenomus canaliculatus* (Fahraeus, 1843), *Pelenomus commari* (Panzer, 1795), *Pelenomus olssoni* (Israelson, 1972), *Pelenomus velaris* (Gyllenhal, 1827), *Pelenomus waltoni* (Boheman, 1843), *Perioxyonyx splendidus* (C. Brisout de Barneville, 1890), *Phrydiuchus augusti* Colonnelli, 2003, *Phrydiuchus quijote* Sánchez-Ruiz & Alonso-Zarazaga, 1995, *Phrydiuchus tau* Warner, 1969, *Phrydiuchus topiarius* (Germar, 1824), *Poophagus sisymbrii* (Fabricius, 1777), *Prisistus obsoletus* (Germar, 1824), *Prisistus suturalba* (Schultze, 1903), *Ranunculiphilus faeculentus* (Gyllenhal, 1837), *Rhinoncus albicinctus* Gyllenhal, 1837, *Rhinoncus bosnicus* Schultze, 1900, *Rhinoncus henningsi* Wagner, 1936, *Rhinoncus pericarpius* (Linnaeus, 1758), *Scleropteridius fallax* Otto, 1897, *Scleropterus offensus* Boheman, 1837, *Sirocalodes mixtus* (Mulsant & Rey, 1859), *Sirocalodes nigroterminatus* (Wollaston, 1854), *Thamiocolus garajonay* Stüben, 2014, *Thamiocolus grancanariensis* Stüben & Schütte, 2014, *Thamiocolus niveus* (Chevrolat, 1859), *Thamiocolus nubeculosus* (Gyllenhal, 1837), *Thamiocolus signatus* (Gyllenhal, 1837), *Thamiocolus sinapis* (Desbrochers des Loges, 1893), *Thamiocolus viduatus* (Gyllenhal, 1813), *Thamiocolus virgatus* (Gyllenhal, 1837), *Thamiocolus wollastoni* (Uyttenboogaart, 1930), *Trichosirocalus barnevillei* (Grenier, 1866), *Trichosirocalus histrix* (Perris, 1852), *Trichosirocalus horridus* (Panzer, 1801), *Trichosirocalus spurnyi* (Schultze, 1901), *Trichosirocalus thalhammeri* (Schultze, 1906), *Zacladus exiguus* (Olivier, 1807)

33 Ceutorhynchinae taxa were <u>not</u> categorized as reference species (confidence group 2):

Amalus scortillum (Herbst, 1795), *Calosirus terminatus* (Herbst, 1795), Ceutorhynchus *cochleariae* (Gyllenhal, 1813), *Ceutorhynchus contractus* (Marsham, 1802), *Ceutorhynchus erysimi* (Fabricius, 1787), *Ceutorhynchus pandellei* Brisout de Barneville, 1869, *Ceutorhynchus sulcicollis* (Paykull, 1800), *Ceutorhynchus tibialis* Boheman, 1845, *Ceutorhynchus typhae* (Herbst, 1795), *Coeliastes lamii* (Fabricius, 1792), *Coeliodes transversealbofasciatus* (Goeze, 1777), *Datonychus melanostictus* (Marsham, 1802), *Microplontus melanostigma* (Marsham, 1802), *Microplontus rugulosus* (Herbst, 1795), *Mogulones geographicus* (Goeze, 1777), *Mononychus punctumalbum* (Herbst, 1784), *Nedyus quadrimaculatus* (Linnaeus, 1758), *Neoglocianus maculaalba* (Herbst, 1795), *Oprohinus consputus* (Germar, 1824), *Parethelcus pollinarius* (Forster, 1771), *Phytobius leucogaster* (Marsham, 1802), Rhinoncus *bruchoides* (Herbst, 1784), *Rhinoncus castor* (Fabricius, 1792), *Rhinoncus inconspectus* (Herbst, 1795), *Rhinoncus perpendicularis* (Reich, 1797), *Rhinoncus smreczynskii* H. Wagner, 1937, *Rutidosoma graminosus* Gistel, 1857, *Sirocalodes depressicollis* (Gyllenhal, 1813), *Stenocarus cardui* (Herbst, 1784), *Stenocarus ruficornis* (Stephens, 1831), *Tapeinotus sellatus* (Fabricius, 1794), *Trichosirocalus troglodytes* (Fabricius, 1787), *Zacladus geranii* (Paykull, 1800)

3 Ceutorhynchinae taxa were <u>omitted</u> from DiStats statistics (confidende group 3):

Ceutorhynchus wellschmiedi Dieckmann, 1979,
Mogulones uncipes (Korotyaev, 1980),
Ranunculiphilus pseudinclemens (Dieckmann, 1970)
