# Supplementary material for: Molecular Weevil Identification Project: A thoroughly curated barcode release of 1300 Western Palearctic weevil species (Coleoptera, Curculionoidea)
Source: Biodivers Data J. 2023 Jan 24;11:e96438. doi: 10.3897/BDJ.11.e96438 (PMC10865102; doi:10.3897/BDJ.11.e96438)
Supplement: Supplementary material 7 — ASAP analyses [file bdj-11-e96438-s007.zip › Suppl. material 7 - ASAP analyses/Apioninae - raw data and concordance evaluation/02 - ASAP html output/ASAP Results.html]

ASAP Results 

ASAP Web results  

```
input file:Apio alignment mit Ns_Geneious_exported.fasta
nb of sequences:342
length of seqs:658
subst. model:Simple Dist
recurs split pval:1.000000e-02
```

10 best partitions found by ASAP (see FAQ for more details)

|  |  |  |  |  |  |  |  |  |  |  |  |  |  |  |  |  |  |  |  |  |  |  |  |  |  |  |  |  |  |  |  |  |  |  |  |  |  |  |  |  |  |  |  |  |  |  |  |  |  |  |  |  |  |  |  |  |  |  |  |  |  |  |  |  |  |  |  |  |  |  |  |  |  |  |  |  |  |  |  |  |  |  |
| --- | --- | --- | --- | --- | --- | --- | --- | --- | --- | --- | --- | --- | --- | --- | --- | --- | --- | --- | --- | --- | --- | --- | --- | --- | --- | --- | --- | --- | --- | --- | --- | --- | --- | --- | --- | --- | --- | --- | --- | --- | --- | --- | --- | --- | --- | --- | --- | --- | --- | --- | --- | --- | --- | --- | --- | --- | --- | --- | --- | --- | --- | --- | --- | --- | --- | --- | --- | --- | --- | --- | --- | --- | --- | --- | --- | --- | --- | --- | --- | --- | --- | --- |
| | Nb of species | asap-score | P-val (rank) | | W (rank) | Treshold dist. | Text | | --- | --- | --- | --- | --- | --- | --- | | 95 | 3.00 |  | 9.21e-02 (5) | 4.04e-04 (1) | 0.060521 | list csv | | 93 | 4.50 |  | 3.91e-02 (3) | 2.59e-04 (6) | 0.073708 | list csv | | 92 | 5.00 |  | 1.14e-01 (7) | 2.72e-04 (3) | 0.075988 | list csv | | 87 | 5.50 |  | 3.21e-02 (2) | 1.96e-04 (9) | 0.082084 | list csv | | 94 | 7.50 |  | 3.47e-01 (13) | 2.91e-04 (2) | 0.068120 | list csv | | \* 111 | 9.50 |  | 9.11e-02 (4) | 1.66e-04 (15) | 0.037994 | list csv | | 88 | 11.00 |  | 4.05e-01 (14) | 1.96e-04 (8) | 0.081615 | list csv | | \* 129 | 14.50 |  | 2.32e-01 (8) | 1.46e-04 (21) | 0.018997 | list csv | | \* 116 | 14.50 |  | 2.75e-01 (10) | 1.48e-04 (19) | 0.029635 | list csv | | \* 112 | 15.00 |  | 6.21e-01 (23) | 2.27e-04 (7) | 0.033435 | list csv | | |  |  | | --- | --- | | Histogram of distances [save] | Ranked distances [save] | |  |  | |

  
 

## **View/Save Boxed species graph here**

  


Asap Score
1.0

12.9

24.8

36.7

48.6

60.5

72.4

84.3

96.2

108.1

0.024

0.047

0.071

0.095

0.119

0.142

0.166


dist


Legend:

<0.001

<0.05

<0.1

>0.1

N/A


Aizobius\_rob

Aizobius\_sed

Alocentron\_c

Apion\_cruent

Apion\_frumen

Apion\_frumen

Apion\_frumen

Apion\_frumen

Apion\_frumen

Apion\_frumen

Apion\_haemat

Apion\_haemat

Apion\_haemat

Apion\_haemat

Apion\_rubigi

Apion\_rubigi

Apion\_rubigi

Aspidapion\_a

Aspidapion\_a

Aspidapion\_a

Aspidapion\_a

Aspidapion\_a

Aspidapion\_a

Aspidapion\_a

Aspidapion\_a

Aspidapion\_a

Aspidapion\_r

Aspidapion\_r

Aspidapion\_r

Aspidapion\_r

Aspidapion\_r

Aspidapion\_r

Aspidapion\_r

Aspidapion\_r

Aspidapion\_v

Aspidapion\_v

Betulapion\_s

Betulapion\_s

Betulapion\_s

Betulapion\_s

Catapion\_mei

Catapion\_mei

Catapion\_pub

Catapion\_sen

Catapion\_ser

Ceratapion\_a

Ceratapion\_a

Ceratapion\_a

Ceratapion\_c

Ceratapion\_c

Ceratapion\_c

Ceratapion\_c

Ceratapion\_c

Ceratapion\_c

Ceratapion\_c

Ceratapion\_d

Ceratapion\_g

Ceratapion\_g

Ceratapion\_o

Ceratapion\_o

Ceratapion\_o

Ceratapion\_o

Ceratapion\_o

Ceratapion\_o

Ceratapion\_p

Ceratapion\_p

Ceratapion\_p

Ceratapion\_p

Ceratapion\_p

Cistapion\_cy

Cyanapion\_co

Cyanapion\_co

Cyanapion\_co

Cyanapion\_gy

Cyanapion\_gy

Cyanapion\_gy

Cyanapion\_pl

Cyanapion\_sp

Cyanapion\_sp

Diplapion\_co

Diplapion\_st

Diplapion\_st

Diplapion\_we

Diplapion\_we

Diplapion\_we

Eutrichapion

Eutrichapion

Eutrichapion

Eutrichapion

Eutrichapion

Eutrichapion

Eutrichapion

Eutrichapion

Eutrichapion

Eutrichapion

Eutrichapion

Eutrichapion

Exapion\_comp

Exapion\_comp

Exapion\_corn

Exapion\_diff

Exapion\_diff

Exapion\_diff

Exapion\_diff

Exapion\_form

Exapion\_form

Exapion\_fusc

Exapion\_fusc

Exapion\_fusc

Exapion\_fusc

Exapion\_fusc

Exapion\_fusc

Exapion\_fusc

Exapion\_ulic

Exapion\_ulic

Exapion\_ulic

Exapion\_ulic

Hemitrichapi

Hemitrichapi

Hemitrichapi

Hemitrichapi

Hemitrichapi

Hemitrichapi

Hemitrichapi

Hemitrichapi

Hemitrichapi

Hemitrichapi

Hemitrichapi

Hemitrichapi

Holotrichapi

Holotrichapi

Holotrichapi

Holotrichapi

Holotrichapi

Holotrichapi

Holotrichapi

Holotrichapi

Holotrichapi

Holotrichapi

Holotrichapi

Holotrichapi

Holotrichapi

Holotrichapi

Holotrichapi

Holotrichapi

Holotrichapi

Holotrichapi

Holotrichapi

Holotrichapi

Holotrichapi

Holotrichapi

Holotrichapi

Holotrichapi

Holotrichapi

Hoplopodapio

Ischnopterap

Ischnopterap

Ischnopterap

Ischnopterap

Ischnopterap

Ischnopterap

Ischnopterap

Ischnopterap

Ischnopterap

Ischnopterap

Ischnopterap

Ischnopterap

Ischnopterap

Ischnopterap

Ischnopterap

Ischnopterap

Ischnopterap

Ischnopterap

Ischnopterap

Ixapion\_vari

Kalcapion\_fo

Kalcapion\_fo

Kalcapion\_fo

Kalcapion\_fo

Kalcapion\_fo

Kalcapion\_fo

Kalcapion\_pa

Kalcapion\_pa

Kalcapion\_pa

Kalcapion\_pa

Kalcapion\_sa

Kalcapion\_se

Kalcapion\_se

Kalcapion\_se

Kalcapion\_se

Kalcapion\_se

Kalcapion\_se

Kalcapion\_se

Kalcapion\_se

Kalcapion\_se

Kalcapion\_se

Lepidapion\_c

Lepidapion\_c

Lepidapion\_c

Lepidapion\_c

Lepidapion\_c

Lepidapion\_c

Lepidapion\_c

Lepidapion\_c

Lepidapion\_c

Lepidapion\_s

Lepidapion\_s

Lepidapion\_s

Loborhynchap

Malvapion\_ma

Malvapion\_ma

Malvapion\_ma

Malvapion\_ma

Malvapion\_ma

Malvapion\_ma

Malvapion\_ma

Melanapion\_m

Omphalapion\_

Omphalapion\_

Omphalapion\_

Omphalapion\_

Onychapion\_t

Oryxolaemus\_

Oryxolaemus\_

Oxystoma\_cra

Oxystoma\_cra

Oxystoma\_cra

Oxystoma\_ope

Oxystoma\_pom

Oxystoma\_sub

Oxystoma\_sub

Perapion\_cur

Perapion\_cur

Perapion\_fal

Perapion\_fal

Perapion\_fal

Perapion\_fal

Perapion\_fal

Perapion\_fal

Perapion\_mar

Perapion\_mar

Perapion\_mar

Perapion\_mar

Perapion\_mar

Perapion\_vio

Perapion\_vio

Perapion\_vio

Perapion\_vio

Perapion\_vio

Phrissotrich

Phrissotrich

Phrissotrich

Phrissotrich

Phrissotrich

Phrissotrich

Phrissotrich

Phrissotrich

Protapion\_ap

Protapion\_ap

Protapion\_ap

Protapion\_ap

Protapion\_as

Protapion\_as

Protapion\_as

Protapion\_di

Protapion\_di

Protapion\_fi

Protapion\_fi

Protapion\_fu

Protapion\_fu

Protapion\_fu

Protapion\_fu

Protapion\_gr

Protapion\_la

Protapion\_la

Protapion\_ni

Protapion\_ni

Protapion\_ni

Protapion\_ni

Protapion\_on

Protapion\_on

Protapion\_on

Protapion\_ru

Protapion\_tr

Protopirapio

Pseudapion\_f

Pseudapion\_m

Pseudapion\_m

Pseudapion\_m

Pseudapion\_r

Pseudapion\_r

Pseudaplemon

Pseudoperapi

Pseudoperapi

Pseudoperapi

Pseudoperapi

Pseudoprotap

Pseudoprotap

Pseudoprotap

Pseudostenap

Pseudostenap

Pseudostenap

Pseudostenap

Pseudostenap

Pseudostenap

Rhopalapion\_

Rhopalapion\_

Rhopalapion\_

Squamapion\_e

Squamapion\_e

Squamapion\_e

Squamapion\_f

Squamapion\_f

Stenopterapi

Stenopterapi

Stenopterapi

Stenopterapi

Stenopterapi

Stenopterapi

Stenopterapi

Stenopterapi

Synapion\_ebe

Synapion\_ebe

Taeniapion\_a

Taeniapion\_d

Taeniapion\_d

Taeniapion\_d

Taeniapion\_d

Taeniapion\_d

Taeniapion\_d

Taeniapion\_r

Taeniapion\_r

Taeniapion\_u

Taeniapion\_u

Taeniapion\_u

Taeniapion\_u

Taeniapion\_u

Taeniapion\_u

Taphrotopium

Trichopterap

Trichopterap

Trichopterap


Tooltip
  
 View/save curves and dendrogram here  
  
*Responsive Crossing lines Legend: Green Line= grouping distance (Dc)- Red line = treshold distance(Dt)*  
Running time:
0 min 38 seconds
  
