# Supplementary material for: Molecular Weevil Identification Project: A thoroughly curated barcode release of 1300 Western Palearctic weevil species (Coleoptera, Curculionoidea)
Source: Biodivers Data J. 2023 Jan 24;11:e96438. doi: 10.3897/BDJ.11.e96438 (PMC10865102; doi:10.3897/BDJ.11.e96438)
Supplement: Supplementary material 7 — ASAP analyses [file bdj-11-e96438-s007.zip › Suppl. material 7 - ASAP analyses/ASAP analyses.pdf]

ASAP analyses - material and method

The program "assemble species by automatic partitioning" has been released recently (ASAP, Puillandre et al. 2021). ASAP is the successor of the program "automated barcode gap discovery" (ABGD, Puillandre et al. 2012). ASAP is meant as a tool for taxonomists to examine DNA barcode datasets concerning the putative number of species. ASAP uses a new clustering algorithm that only uses pairwise genetic distances, avoiding the computational burden of phylogenetic reconstruction to delineate species. As ABGD does, ASAP provides not just one but several partitions, each based on one distance threshold, more or less fine-grained. Those partitions are ranked using a new scoring system, i.e., a confidence value named "ASAP-score". The ASAP-score is based on the p-value and each partition's relative barcode gap width. This score should help to identify the "best" partition/threshold in respect to the input dataset (nucleotide alignment). The lower the ASAP-score, the better the calculated partition and its threshold is rated, and therefore the higher the confidence level.

We test the program's limits with three sub-datasets, Cryptorhynchinae, Apioninae, and Ceutorhynchinae. Those contain reliably identified species previously created for DiStats statistics (Suppl. material 6). We refer to the calculated species by ASAP as molecular operational taxonomic units (MOTUs, Floyd et al. 2002, Blaxter 2004) to separate morphologically identified species and calculated ones. Our main question is: How reliable can ASAP assign the sequences to MOTUs matching the morphological identification based on one (p-distance) threshold value per dataset computed by the program? We will manually compare the program's calculated MOTUs with the identified species and flag deviations. This evaluation of concordance between MOTUs and morphologically identified species shows how reliable ASAP can categorize sequences into natural taxa.

We will evaluate the concordance from all ten partitions/thresholds per sub-dataset calculated by ASAP. Especially the "best partition/best threshold" based on the ASAP-score is of interest. By doing so, the general idea of "one threshold fits all" for the three datasets is also being tested using 10 different computed thresholds for MOTU delineation.

There are some cases with known limitations of the CO1 gene in respect to species delineation, mostly pending synonyms. Those taxa were already removed from the DiStats sub-datasets as keeping those would unnecessary distort the results. If there are no significant molecular differences between species, no algorithm will be able to make the delineation. The DiStats sub-datasets (Cryptorhynchinae, Apioninae, Ceutorhynchinae) only contain sequences of confidence groups 1 (reference species) and 2 (congeners); ambiguous identifications like "cf." and "sp." from confidence group 3 were omitted; infraspecific epithets were omitted. See in-depth description of DiStats method and data preparation in Suppl. material 6. Thus no questionable taxa are included in the DiStats sub-datasets used for ASAP as well.

Furthermore we discarded 40 additional sequences from the Cryptorhynchinae sub-dataset, namely, those belonging to the *Acalles sierrae* complex: *Acalles alcarazensis*, *A. asniensis*, *A. cazorlaensis*, *A. cytisi, A. guadarramaensis*, *A. iblanensis*, *A. maraoensis*, *A. monasterialis*, *A. parasierrae*, *A. sarothamni*, *A. sierrae*, *A. testensis*. Instead of 1146 sequences, the Cryptorhynchinae sub-dataset for ASAP contains 1106 sequences.

See Suppl. material 8 for nucleotide alignments as input data, ASAP output files, and spreadsheets used to evaluate the concordance of MOTUs with morpho species.

ASAP creates a result table with 10 proposed partitions; each partition is based on one threshold value used for species delineation. ASAP also provides the calculated number of MOTUs (putative species) separated by that threshold. To put it simply, the threshold is roughly the center of the ASAP-internal calculated barcode gap. ASAP also provides a confidence value called "ASAP-score" for each partition/threshold; the lower this value is, the higher the confidence level in that threshold. Partition 1 and its threshold value is the preferred one by ASAP. The ASAP-score in partition 1 is the lowest in all 10 partitions calculated by ASAP.

See Table 6 "summarized ASAP results" in the main article and the end of this chapter for the three sub-datasets and concordance evaluation between MOTUs and morpho species. The left side of the table represents the ASAP results (rows 1 to 4). The right side of the table represents the concordance evaluation (rows 5 to 7), based on sequence counts of wrongly assigned morpho species to non-matching MOTUs. For a more straightforward assessment, the grey highlighted thresholds show the lowest amount of wrongly assigned taxa possible or the lowest available error rate out of the 10 proposed thresholds. Please note that the best matching thresholds are not those suggested by ASAP (partition 1 with the lowest ASAP-score). Thus, the "manually selected" partitions (with grey highlighted thresholds) result in a higher matching ratio between MOTUs and morpho species. Of course, the selection is based on the morpho species evaluation and could not be carried out automatically by ASAP.

The concordance evaluation focuses only on the amount of wrongly assigned sequences (MOTU vs. morpho species), not on the estimated sheer amount of taxa (row 2 in Table 6) in the dataset. Even if the number of taxa was spot on, for example, 265 MOTUs in 265 morpho species, there would not be any insight into how many sequences were assigned correctly.

ASAP results for Cryptorhynchinae sub-dataset

ASAP proposed thresholds ranging from **0.4%** to **7.3%.**
Partition 1 (ASAP-score: 8.5) uses a threshold value of **7.3%**. While the ASAP result estimates 236 species in the dataset, the actual species count is 265.
214 of 1106 sequences have been assigned to a wrong MOTU (oversplit or lumped to a sister species), an **error rate of 19%** based on the sequence count.
Partition 6, with a threshold of **6.8%,** revealed a slightly better concordance with a 16% error rate based on the sequence count.
Partition 5, on the other hand, with a threshold of just 0.4%, shows the worst concordance with a 43% error rate based on the sequence count.

ASAP results for Apioninae sub-dataset

ASAP proposed thresholds ranging from **1.9%** to **8.1%.**
Partition 1 (ASAP-score: 3.0) uses a threshold value of **6.0%**. While the ASAP result estimates 95 species in the dataset, the actual species count is 114.
47 of 342 sequences have been assigned to a wrong MOTU (oversplit or lumped to a sister species),
an **error rate of 14%** based on the sequence count.
Partition 6, with a threshold of 3.8**%,** revealed a much better concordance with a 7% error rate based on the sequence count.
Partition 4, on the other hand, with a threshold of 8.2%, shows the worst concordance with an 18% error rate based on the sequence count.

ASAP results for Ceutorhynchinae sub-dataset

ASAP proposed thresholds ranging from **2.2%** to **8.6%**.
Partition 1 (ASAP-score: 1.0) uses a threshold value of **5.1%**. While the ASAP result estimates 204 species in the dataset, the actual species count is 199.
28 of 491 sequences have been assigned to a wrong MOTU (oversplit or lumped to a sister species),
an **error rate of 6%** based on the sequence count. Depending on where to emphasis, Partition 1 and Partition 3 performed almost identically.
Partition 1 shows the best prediction in the sheer amount of taxa in the dataset (204 MOTUs vs. 199 morphos species). Still, partition 3 performs slightly better correctly assigning MOTUS to morpho species: 24 wrongly assigned sequences instead of 28 wrongly assigned in Partition 1.
Partition 3 shows an error rate of 5%, while Partition 1 shows an error rate of 6%.
Partition 5, with a threshold value of 2.2%, shows the worst concordance with a 12% error rate based on the sequence count.

Concerning the 3 sub-datasets, the ASAP-score is not a reliable identifier for the confidence level of the suggested threshold (ASAP-score: the lower, the better), at least not for the three sub-datasets:

Cryptorhynchinae partition 1 with the lowest ASAP-score of "**8.5**" shows 19% wrongly assigned taxa, while partition 6 with an ASAP-score of "17" shows 16% wrongly assigned taxa (3% less). ASAP-score does not deliver helpful information here.

Apioninae partition 1 with the lowest ASAP-score of "**3**" shows 14% of wrongly assigned taxa, while partition 6 with an ASAP-score of "9.5" shows just 7% of wrongly assigned taxa. Thus, the partition/threshold with the higher ASAP-score provides a much better threshold for that subfamily.

Ceutorhynchinae partition 1, with the lowest ASAP-score of "**1**", shows 6% wrongly assigned taxa, while Partition 10, with an ASAP-score of "18.5", also shows 6% wrongly assigned taxa. The suggested p-distance threshold value for partition 1 is 5.1%, while it is 5.5% for partition 10. The threshold values do not show not a significant difference, and a similar amount of wrongly assigned taxa are to expect. Still, one time the ASAP-score is the lowest (highest confidence) and the other time the highest (worst confidence). On the contrary, partition 3, with a relatively high ASAP-score of "11" and a threshold value of 5.0% (nearly the same as partition 1), shows the lowest percentage of wrongly assigned taxa (5% error rate). Thus, the partition/threshold with the higher ASAP-score can provide a better one.

References

- Puillandre N, Lambert A, Broillet S, Achaz G (2012) ABGD, Automatic Barcode Gap Discovery for primary species delimitation. *Molecular ecology* **21**(8): 1864-1877. DOI: 10.1111/j.1365-294X.2011.05239.x
- Puillandre N, Brouillet S, Achaz G (2021) ASAP: assemble species by automatic partitioning. *Molecular ecology resources* **21**(2): 609-620. DOI: 10.1111/1755-0998.13281
- Blaxter ML (2004) The promise of a DNA taxonomy. *Philosophical Transactions of the Royal Society B-Biological Sciences* **359**(1444): 669-679. DOI: 10.1098/rstb.2003.1447
- Floyd R, Abebe E, Papert A, Blaxter M (2002) Molecular barcodes for soil nematode identification. *Molecular ecology* **11**(4): 839-850. DOI: 10.1046/j.1365-294x.2002.01485.x

Note: The table below is also available in the main article and as spreadsheet in Suppl. material 8.

Table 6: Left side of table: summarized ASAP results, right side of table: evaluation of concordance between MOTUs and morphospecies. For each subfamily dataset, 10 different thresholds ("ASAP partitions") and derived MOTUs are calculated by ASAP. The evaluation of concordance provides the deviations between MOTUs and morphospecies for each given threshold, wrongly assigned MOTUs are given in absolute numbers and in percent. Marked tables point to the threshold which fits best to each subfamily dataset (lowest number of deviation between MOTUs and morphospecies).

	ASAP results			evaluation of concordance		
ASAP Partition	MOTUs	Threshold [%]	ASAP-score	no of wrongly assigned taxa	no of wrongly assigned seqs	% of wrongly assigned seqs
Cryptorhynchinae sub-dataset (contains 265 morphospecies, 1106 sequences)						
1	236	7.3	8.5	74	214	19%
2	241	7.2	9.0	73	214	19%
3	315	3.8	9.5	84	190	17%
4	348	2.4	15.0	104	206	19%
5	639	0.4	16.5	373	480	43%
6	251	6.8	17.0	68	181	16%
7	325	3.4	24.5	86	193	17%
8	316	3.7	25.5	83	186	17%
9	302	4.3	29.0	84	205	19%
10	329	3.1	29.5	89	189	17%
Apioninae sub-dataset (contains 114 morphospecies, 342 sequences)						
1	95	6.0	3.0	19	47	14%
2	93	7.3	4.5	21	52	15%
3	92	7.5	5.0	22	52	15%
4	87	8.2	5.5	28	61	18%
5	94	6.8	7.5	17	41	12%
6	111	3.8	9.5	13	25	7%
7	88	8.1	11.0	24	57	17%
8	129	1.9	14.5	24	29	8%
9	116	3.0	14.5	16	28	8%
10	112	3.3	15.0	13	27	8%
Ceutorhynchinae sub-dataset (contains 199 morphospecies, 491 sequences)						
1	204	5.1	1.0	17	28	6%
2	191	6.9	7.0	19	38	8%
3	206	5.0	11.0	15	24	5%
4	186	7.7	11.5	19	40	8%
5	235	2.2	13.0	37	59	12%
6	190	7.1	13.5	18	37	8%
7	178	8.5	14.0	23	64	13%
8	183	7.8	16.0	20	42	9%
9	178	8.6	16.5	23	46	9%
10	203	5.5	18.5	18	31	6%
