# Supplementary material for: Molecular Weevil Identification Project: A thoroughly curated barcode release of 1300 Western Palearctic weevil species (Coleoptera, Curculionoidea)
Source: Biodivers Data J. 2023 Jan 24;11:e96438. doi: 10.3897/BDJ.11.e96438 (PMC10865102; doi:10.3897/BDJ.11.e96438)
Supplement: Supplementary material 7 — ASAP analyses [file bdj-11-e96438-s007.zip › Suppl. material 7 - ASAP analyses/Ceutorhynchinae - raw data and concordance evaluation/02 - ASAP html output/ASAP Results.html]

ASAP Results 

ASAP Web results  

```
date:2021-06-02T17:08:30
input file:Ceuto Alignment mit Ns 14.06.2021.fasta
nb of sequences:517
length of seqs:658
subst. model:Simple Dist
recurs split pval:1.000000e-02
```

Save Spart File  here

10 best partitions found by ASAP (see FAQ for more details)

|  |  |  |  |  |  |  |  |  |  |  |  |  |  |  |  |  |  |  |  |  |  |  |  |  |  |  |  |  |  |  |  |  |  |  |  |  |  |  |  |  |  |  |  |  |  |  |  |  |  |  |  |  |  |  |  |  |  |  |  |  |  |  |  |  |  |  |  |  |  |  |  |  |  |  |  |  |  |  |  |  |  |  |
| --- | --- | --- | --- | --- | --- | --- | --- | --- | --- | --- | --- | --- | --- | --- | --- | --- | --- | --- | --- | --- | --- | --- | --- | --- | --- | --- | --- | --- | --- | --- | --- | --- | --- | --- | --- | --- | --- | --- | --- | --- | --- | --- | --- | --- | --- | --- | --- | --- | --- | --- | --- | --- | --- | --- | --- | --- | --- | --- | --- | --- | --- | --- | --- | --- | --- | --- | --- | --- | --- | --- | --- | --- | --- | --- | --- | --- | --- | --- | --- | --- | --- | --- |
| | Nb of species | asap-score | P-val (rank) | | W (rank) | Treshold dist. | Text | | --- | --- | --- | --- | --- | --- | --- | | 204 | 1.00 |  | 1.00e-05 (1) | 2.34e-04 (1) | 0.050912 | list csv | | 191 | 7.00 |  | 3.40e-04 (4) | 1.92e-04 (10) | 0.069149 | list csv | | \* 206 | 11.00 |  | 1.68e-01 (20) | 2.30e-04 (2) | 0.049882 | list csv | | 186 | 11.50 |  | 1.04e-01 (15) | 2.02e-04 (8) | 0.077463 | list csv | | \* 235 | 13.00 |  | 4.81e-02 (11) | 1.81e-04 (15) | 0.022422 | list csv | | 190 | 13.50 |  | 2.55e-01 (22) | 2.15e-04 (5) | 0.070669 | list csv | | 178 | 14.00 |  | 8.38e-03 (5) | 1.68e-04 (23) | 0.085171 | list csv | | 183 | 16.00 |  | 2.75e-01 (26) | 2.08e-04 (6) | 0.077705 | list csv | | 178 | 16.50 |  | 1.34e-02 (7) | 1.61e-04 (26) | 0.085680 | list csv | | 203 | 18.50 |  | 4.01e-02 (10) | 1.55e-04 (27) | 0.055471 | list csv | | |  |  | | --- | --- | | Histogram of distances [save] | Ranked distances [save] | |  |  | |

  
 

## **View/Save Boxed species graph here**

  


Asap Score
1.0

15.8

30.6

45.4

60.2

75.0

89.8

104.6

119.4

134.2

0.026

0.052

0.078

0.103

0.129

0.155

0.181


dist


Legend:

<0.001

<0.05

<0.1

>0.1

N/A


Amalus\_scort

Amalus\_scort

Amalus\_scort

Amalus\_scort

Aphytobius\_s

Aphytobius\_v

Auleutes\_epi

Barioxyonyx\_

Barioxyonyx\_

Brachiodontu

Brachiodontu

Calosirus\_te

Ceutorhynchu

Ceutorhynchu

Ceutorhynchu

Ceutorhynchu

Ceutorhynchu

Ceutorhynchu

Ceutorhynchu

Ceutorhynchu

Ceutorhynchu

Ceutorhynchu

Ceutorhynchu

Ceutorhynchu

Ceutorhynchu

Ceutorhynchu

Ceutorhynchu

Ceutorhynchu

Ceutorhynchu

Ceutorhynchu

Ceutorhynchu

Ceutorhynchu

Ceutorhynchu

Ceutorhynchu

Ceutorhynchu

Ceutorhynchu

Ceutorhynchu

Ceutorhynchu

Ceutorhynchu

Ceutorhynchu

Ceutorhynchu

Ceutorhynchu

Ceutorhynchu

Ceutorhynchu

Ceutorhynchu

Ceutorhynchu

Ceutorhynchu

Ceutorhynchu

Ceutorhynchu

Ceutorhynchu

Ceutorhynchu

Ceutorhynchu

Ceutorhynchu

Ceutorhynchu

Ceutorhynchu

Ceutorhynchu

Ceutorhynchu

Ceutorhynchu

Ceutorhynchu

Ceutorhynchu

Ceutorhynchu

Ceutorhynchu

Ceutorhynchu

Ceutorhynchu

Ceutorhynchu

Ceutorhynchu

Ceutorhynchu

Ceutorhynchu

Ceutorhynchu

Ceutorhynchu

Ceutorhynchu

Ceutorhynchu

Ceutorhynchu

Ceutorhynchu

Ceutorhynchu

Ceutorhynchu

Ceutorhynchu

Ceutorhynchu

Ceutorhynchu

Ceutorhynchu

Ceutorhynchu

Ceutorhynchu

Ceutorhynchu

Ceutorhynchu

Ceutorhynchu

Ceutorhynchu

Ceutorhynchu

Ceutorhynchu

Ceutorhynchu

Ceutorhynchu

Ceutorhynchu

Ceutorhynchu

Ceutorhynchu

Ceutorhynchu

Ceutorhynchu

Ceutorhynchu

Ceutorhynchu

Ceutorhynchu

Ceutorhynchu

Ceutorhynchu

Ceutorhynchu

Ceutorhynchu

Ceutorhynchu

Ceutorhynchu

Ceutorhynchu

Ceutorhynchu

Ceutorhynchu

Ceutorhynchu

Ceutorhynchu

Ceutorhynchu

Ceutorhynchu

Ceutorhynchu

Ceutorhynchu

Ceutorhynchu

Ceutorhynchu

Ceutorhynchu

Ceutorhynchu

Ceutorhynchu

Ceutorhynchu

Ceutorhynchu

Ceutorhynchu

Ceutorhynchu

Ceutorhynchu

Ceutorhynchu

Ceutorhynchu

Ceutorhynchu

Ceutorhynchu

Ceutorhynchu

Ceutorhynchu

Ceutorhynchu

Ceutorhynchu

Ceutorhynchu

Ceutorhynchu

Ceutorhynchu

Ceutorhynchu

Ceutorhynchu

Ceutorhynchu

Ceutorhynchu

Ceutorhynchu

Ceutorhynchu

Ceutorhynchu

Ceutorhynchu

Ceutorhynchu

Ceutorhynchu

Ceutorhynchu

Ceutorhynchu

Ceutorhynchu

Ceutorhynchu

Ceutorhynchu

Ceutorhynchu

Ceutorhynchu

Ceutorhynchu

Ceutorhynchu

Ceutorhynchu

Ceutorhynchu

Ceutorhynchu

Ceutorhynchu

Ceutorhynchu

Ceutorhynchu

Ceutorhynchu

Ceutorhynchu

Ceutorhynchu

Ceutorhynchu

Ceutorhynchu

Ceutorhynchu

Ceutorhynchu

Ceutorhynchu

Ceutorhynchu

Ceutorhynchu

Ceutorhynchu

Ceutorhynchu

Ceutorhynchu

Ceutorhynchu

Ceutorhynchu

Ceutorhynchu

Ceutorhynchu

Ceutorhynchu

Ceutorhynchu

Ceutorhynchu

Ceutorhynchu

Ceutorhynchu

Ceutorhynchu

Ceutorhynchu

Ceutorhynchu

Ceutorhynchu

Ceutorhynchu

Ceutorhynchu

Ceutorhynchu

Ceutorhynchu

Ceutorhynchu

Ceutorhynchu

Ceutorhynchu

Ceutorhynchu

Ceutorhynchu

Ceutorhynchu

Ceutorhynchu

Ceutorhynchu

Ceutorhynchu

Ceutorhynchu

Ceutorhynchu

Ceutorhynchu

Ceutorhynchu

Ceutorhynchu

Ceutorhynchu

Ceutorhynchu

Ceutorhynchu

Ceutorhynchu

Ceutorhynchu

Ceutorhynchu

Ceutorhynchu

Ceutorhynchu

Ceutorhynchu

Ceutorhynchu

Ceutorhynchu

Ceutorhynchu

Ceutorhynchu

Ceutorhynchu

Ceutorhynchu

Ceutorhynchu

Ceutorhynchu

Coeliastes\_l

Coeliastes\_l

Coeliastes\_l

Coeliodes\_ra

Coeliodes\_ru

Coeliodes\_ru

Coeliodes\_ru

Coeliodes\_tr

Coeliodes\_tr

Coeliodes\_tr

Coeliodes\_tr

Coeliodes\_tr

Coeliodinus\_

Datonychidiu

Datonychus\_a

Datonychus\_a

Datonychus\_d

Datonychus\_m

Datonychus\_m

Datonychus\_m

Datonychus\_m

Datonychus\_m

Datonychus\_m

Datonychus\_m

Datonychus\_p

Drupenatus\_n

Eubrychius\_v

Eubrychius\_v

Eucoeliodes\_

Glocianus\_di

Glocianus\_di

Glocianus\_di

Glocianus\_gr

Glocianus\_mo

Glocianus\_mo

Glocianus\_mo

Glocianus\_pu

Glocianus\_pu

Glocianus\_pu

Glocianus\_pu

Glocianus\_pu

Hadroplontus

Hadroplontus

Hadroplontus

Hadroplontus

Hadroplontus

Hadroplontus

Hesperorrhyn

Hesperorrhyn

Hesperorrhyn

Hesperorrhyn

Hesperorrhyn

Hesperorrhyn

Hesperorrhyn

Hesperorrhyn

Hesperorrhyn

Hesperorrhyn

Hesperorrhyn

Homorosoma\_v

Marmaropus\_b

Mesoxyonyx\_s

Micrelus\_eri

Micrelus\_eri

Micrelus\_eri

Micrelus\_fer

Microplontus

Microplontus

Microplontus

Microplontus

Microplontus

Microplontus

Microplontus

Microplontus

Microplontus

Microplontus

Microplontus

Microplontus

Microplontus

Mogulones\_ab

Mogulones\_ab

Mogulones\_an

Mogulones\_an

Mogulones\_an

Mogulones\_as

Mogulones\_as

Mogulones\_as

Mogulones\_as

Mogulones\_au

Mogulones\_au

Mogulones\_au

Mogulones\_bi

Mogulones\_ci

Mogulones\_cr

Mogulones\_cr

Mogulones\_cr

Mogulones\_cr

Mogulones\_cr

Mogulones\_cr

Mogulones\_cy

Mogulones\_cy

Mogulones\_de

Mogulones\_di

Mogulones\_di

Mogulones\_eu

Mogulones\_eu

Mogulones\_eu

Mogulones\_ge

Mogulones\_ge

Mogulones\_ge

Mogulones\_ge

Mogulones\_ge

Mogulones\_ge

Mogulones\_ge

Mogulones\_gr

Mogulones\_gr

Mogulones\_gr

Mogulones\_gr

Mogulones\_gr

Mogulones\_ja

Mogulones\_ja

Mogulones\_la

Mogulones\_pa

Mogulones\_pe

Mogulones\_pe

Mogulones\_pe

Mogulones\_ps

Mogulones\_ps

Mogulones\_ps

Mogulones\_ra

Mogulones\_ra

Mogulones\_ra

Mogulones\_so

Mogulonoides

Mononychus\_p

Mononychus\_p

Mononychus\_p

Mononychus\_p

Mononychus\_p

Nedyus\_quadr

Nedyus\_quadr

Nedyus\_quadr

Nedyus\_quadr

Nedyus\_quadr

Nedyus\_quadr

Nedyus\_quadr

Nedyus\_quadr

Neoglocianus

Neoglocianus

Neoglocianus

Neoglocianus

Neophytobius

Neophytobius

Oprohinus\_co

Oprohinus\_su

Oreorrhyncha

Parethelcus\_

Parethelcus\_

Parethelcus\_

Parethelcus\_

Parethelcus\_

Parethelcus\_

Parethelcus\_

Parethelcus\_

Paroxyonyx\_a

Paroxyonyx\_c

Paroxyonyx\_c

Paroxyonyx\_f

Paroxyonyx\_i

Pelenomus\_ca

Pelenomus\_ca

Pelenomus\_co

Pelenomus\_co

Pelenomus\_co

Pelenomus\_co

Pelenomus\_ol

Pelenomus\_ve

Pelenomus\_ve

Pelenomus\_wa

Pelenomus\_wa

Pelenomus\_wa

Perioxyonyx\_

Perioxyonyx\_

Phrydiuchus\_

Phrydiuchus\_

Phrydiuchus\_

Phrydiuchus\_

Phrydiuchus\_

Phrydiuchus\_

Phytobius\_le

Poophagus\_si

Poophagus\_si

Poophagus\_si

Prisistus\_ob

Prisistus\_su

Pseudophytob

Ranunculiphi

Ranunculiphi

Rhinoncus\_al

Rhinoncus\_al

Rhinoncus\_bo

Rhinoncus\_br

Rhinoncus\_br

Rhinoncus\_br

Rhinoncus\_ca

Rhinoncus\_ca

Rhinoncus\_ca

Rhinoncus\_ca

Rhinoncus\_ca

Rhinoncus\_he

Rhinoncus\_in

Rhinoncus\_in

Rhinoncus\_in

Rhinoncus\_pe

Rhinoncus\_pe

Rhinoncus\_pe

Rhinoncus\_pe

Rhinoncus\_pe

Rhinoncus\_pe

Rhinoncus\_pe

Rhinoncus\_pe

Rhinoncus\_pe

Rhinoncus\_pe

Rhinoncus\_pe

Rhinoncus\_pe

Rhinoncus\_pe

Rhinoncus\_pe

Rhinoncus\_pe

Rhinoncus\_pe

Rhinoncus\_sm

Rhinoncus\_sm

Rhinoncus\_sm

Rhinoncus\_sm

Rutidosoma\_g

Scleropterid

Scleropterid

Scleropterid

Scleropterid

Scleropterus

Scleropterus

Scleropterus

Scleropterus

Scleropterus

Scleropterus

Sirocalodes\_

Sirocalodes\_

Sirocalodes\_

Sirocalodes\_

Sirocalodes\_

Sirocalodes\_

Sirocalodes\_

Stenocarus\_c

Stenocarus\_r

Stenocarus\_r

Stenocarus\_r

Tapeinotus\_s

Tapeinotus\_s

Thamiocolus\_

Thamiocolus\_

Thamiocolus\_

Thamiocolus\_

Thamiocolus\_

Thamiocolus\_

Thamiocolus\_

Thamiocolus\_

Thamiocolus\_

Thamiocolus\_

Thamiocolus\_

Thamiocolus\_

Thamiocolus\_

Thamiocolus\_

Thamiocolus\_

Thamiocolus\_

Thamiocolus\_

Thamiocolus\_

Trichosiroca

Trichosiroca

Trichosiroca

Trichosiroca

Trichosiroca

Trichosiroca

Trichosiroca

Trichosiroca

Trichosiroca

Trichosiroca

Trichosiroca

Trichosiroca

Trichosiroca

Zacladus\_exi

Zacladus\_exi

Zacladus\_exi

Zacladus\_ger

Zacladus\_ger

Zacladus\_ger

Zacladus\_ger

Zacladus\_ger

Zacladus\_ger

Zacladus\_ger


Tooltip
  
 View/save curves and dendrogram here  
  
*Responsive Crossing lines Legend: Green Line= grouping distance (Dc)- Red line = treshold distance(Dt)*  
Running time:
0 min 16 seconds
  
