# Supplementary material for: Molecular Weevil Identification Project: A thoroughly curated barcode release of 1300 Western Palearctic weevil species (Coleoptera, Curculionoidea)
Source: Biodivers Data J. 2023 Jan 24;11:e96438. doi: 10.3897/BDJ.11.e96438 (PMC10865102; doi:10.3897/BDJ.11.e96438)
Supplement: Supplementary material 7 — ASAP analyses [file bdj-11-e96438-s007.zip › Suppl. material 7 - ASAP analyses/Cryptorhynchinae - raw data and concordance evaluation/02 - ASAP html output/ASAP FAQ.html]

ASAP FAQ


# FAQ ASAP web: Assemble Species by Automatic Partitioning

---

## Concerning the method:

What is ASAP ? 

ASAP is a tool designed to propose partitions of species hypotheses using genetic distances calculated between DNA sequences.

How many sequences can I load?

ASAP can handle more than 10 000 sequences, but the computation time
can be quite important in this case (several hours). If you need to
work with a very large dataset, we recommend you to download the command
line version of ASAP to run it on your computer instead of overloading
the server.

What are the data formats accepted by ASAP?

The Fasta format is the most convenient format. If you provide a
distance matrix (phylip dnadist or MEGA CSV) you must provide the length
of the alignment used to compute the matrix.

When I try to run a MEGA CSV file, it produces a blank page/ Why can't I use a file name with ".csv" as extension?

Please rename the ".csv" extension into ".txt" as CSV can be interpreted
as special objects by some browsers and will produce unexpected results

Can I run ASAP as a command line? /Where can I download the command line version of ASAP?

Yes, you can download the source code here

## Concerning results:

Please, explain the different column titles in results table (groups,score,proba,W and dc)

- **Nbgroups** is the number of species as identified by ASAP in the corresponding partition
- ASAP identifies different partitions, and the **score** is an
  indicator of which partition you have to look at. It is a combination
  beetween the two following parameters (probability and slope)
- **Proba** is the probability that the partition at the step n is
  different from the partition at the step n-1. Please, refer to the
  publication for more details.
- **W** is the slope of the curve shown on the right ("Ranked
  distances") at a given genetic distance value (see below). A high value
  means that the next distances (bigger and smaller) values are far.
- **Dc** is the value of the "jump" distance used to calculate the slope.
- **Text** is a text file containing the different partitions. Two formats are available:
  - a **csv** file: each line is a sequence label followed by the group number, both separated with a semicolon.
  - a **list** file: each line is a group and all the sequences belonging to that group are listed

Why is there a star in front of some ASAP results? / Why is there a blue area on the curve of the ASAP scores? 

On the main ASAP page you can tune some parameters (see the help
page).
All the partitions within the range of genetic distances you
provided will be preceded by a star and a blue area corresponding to
this range will be drawn on the curve. Default values are 0.005 and
0.05.

What is the meaning of the different colors of the dots/squares in the curve and in the dendrogram?

For each partition and for each node for which a probability
has been calculated, the darker the color of the dots and squares, the
higher the probability. When the probability was not computed, the
square is grey. We choose to use different symbols (dots and squares)
for the table and the curve in one hand and for the dendrogram in the
other hand, because the probability is not calculated the same way. For
the table and the curve, it corresponds to the probability of the
partition. For the dendrogram, it corresponds to the probability that
merging the groups within the node is compatible with the known
distances inside each of these groups. A very low probability (dark
color) indicates that this group is unlikely, i.e. that the groups
within this node probably correspond to different species. . Please
refer to the publication for more details.

Why results can be slightly different if I re-run ASAP with the same data?

ASAP uses a seed to generate random partitions in order to estimate
the probability of a partition. A new seed can slightly change the
probabilities.

Something happens if I click/roll over on some parts of the graphic

Some regions are responsive:

- A click on a line of the results table will draw two lines. A
  green one coresponding to the grouping distance and a red one
  corresponding to the jumping distance (see paper for more details)
  The lines will be drawn on every graphics showing where it cuts the
  histogram, where it cuts the cumulative ranked distance curve,
to which value on the ASAP score curve it corresponds and where it cuts the dendrogram- A click on a node of the dendrogram will color in red all the sequences grouped in the clicked node
- A roll over a sequence name on the dendrogram will show the full sequence label.
- A roll over a node will provide some information as: **proba** (the probability associated to the considered node), **dist** (the genetic distance to which this node corresponds), **nbgroup** (the number of groups in a partition that would correspond to the genetic distance of the node),
  **node number**  (for internal use only) and **nbunder** (how many sequences are grouped by this node)

The sequence label is truncated after 20 characters, how can I see it in full?

Roll over the name and the full label will be displayed

I have my own hypothesis concerning the number of species included in my dataset, but ASAP doesn't class it at first rank

Remember that ASAP is an exploratory tool designed to
identify the best partitions of species, given the criteria used by ASAP
(in particular the genetic distances). Your own species hypotheses
might be based on other data, methods or criteria of species
delimitation, and might thus be different from the best ASAP partitions.
Combining all these results in an integrative taxonomy approach is
generally a good idea.

---

*Last updated: 7 Apr 2021*
