# Supplementary material for: Molecular Weevil Identification Project: A thoroughly curated barcode release of 1300 Western Palearctic weevil species (Coleoptera, Curculionoidea)
Source: Biodivers Data J. 2023 Jan 24;11:e96438. doi: 10.3897/BDJ.11.e96438 (PMC10865102; doi:10.3897/BDJ.11.e96438)
Supplement: Supplementary material 7 — ASAP analyses [file bdj-11-e96438-s007.zip › Suppl. material 7 - ASAP analyses/Cryptorhynchinae - raw data and concordance evaluation/02 - ASAP html output/ASAP Results.html]

ASAP Results 

ASAP Web results  

```
date:2021-04-07T15:36:43
input file:Crypto_2021.05.24.fasta
nb of sequences:1106
length of seqs:658
subst. model:Simple Dist
recurs split pval:1.000000e-02
```

Save Spart File  here

10 best partitions found by ASAP (see FAQ for more details)

|  |  |  |  |  |  |  |  |  |  |  |  |  |  |  |  |  |  |  |  |  |  |  |  |  |  |  |  |  |  |  |  |  |  |  |  |  |  |  |  |  |  |  |  |  |  |  |  |  |  |  |  |  |  |  |  |  |  |  |  |  |  |  |  |  |  |  |  |  |  |  |  |  |  |  |  |  |  |  |  |  |  |  |
| --- | --- | --- | --- | --- | --- | --- | --- | --- | --- | --- | --- | --- | --- | --- | --- | --- | --- | --- | --- | --- | --- | --- | --- | --- | --- | --- | --- | --- | --- | --- | --- | --- | --- | --- | --- | --- | --- | --- | --- | --- | --- | --- | --- | --- | --- | --- | --- | --- | --- | --- | --- | --- | --- | --- | --- | --- | --- | --- | --- | --- | --- | --- | --- | --- | --- | --- | --- | --- | --- | --- | --- | --- | --- | --- | --- | --- | --- | --- | --- | --- | --- | --- |
| | Nb of species | asap-score | P-val (rank) | | W (rank) | Treshold dist. | Text | | --- | --- | --- | --- | --- | --- | --- | | 236 | 8.50 |  | 9.90e-03 (5) | 2.25e-05 (12) | 0.073708 | list csv | | 241 | 9.00 |  | 1.06e-03 (2) | 2.21e-05 (16) | 0.072107 | list csv | | \* 315 | 9.50 |  | 1.56e-01 (13) | 2.45e-05 (6) | 0.038365 | list csv | | \* 348 | 15.00 |  | 2.17e-02 (6) | 2.05e-05 (24) | 0.024353 | list csv | | 639 | 16.50 |  | 5.01e-01 (32) | 4.89e-05 (1) | 0.003571 | list csv | | 251 | 17.00 |  | 6.65e-02 (9) | 2.05e-05 (25) | 0.067917 | list csv | | \* 325 | 24.50 |  | 6.71e-01 (45) | 2.47e-05 (4) | 0.034195 | list csv | | \* 316 | 25.50 |  | 6.81e-01 (48) | 2.58e-05 (3) | 0.036845 | list csv | | \* 302 | 29.00 |  | 2.22e-01 (17) | 1.81e-05 (41) | 0.042842 | list csv | | \* 329 | 29.50 |  | 1.42e-01 (11) | 1.72e-05 (48) | 0.031155 | list csv | | |  |  | | --- | --- | | Histogram of distances [save] | Ranked distances [save] | |  |  | |

  
 

## **View/Save Boxed species graph here**

  


Asap Score
1.0

25.7

50.4

75.1

99.8

124.5

149.2

173.9

198.6

223.3

0.028

0.057

0.085

0.114

0.142

0.171

0.199


dist


Legend:

<0.001

<0.05

<0.1

>0.1

N/A


Acalles\_alme

Acalles\_alme

Acalles\_alme

Acalles\_alme

Acalles\_alme

Acalles\_alme

Acalles\_alme

Acalles\_alme

Acalles\_biok

Acalles\_biok

Acalles\_brei

Acalles\_brei

Acalles\_came

Acalles\_came

Acalles\_came

Acalles\_came

Acalles\_came

Acalles\_came

Acalles\_came

Acalles\_came

Acalles\_came

Acalles\_came

Acalles\_came

Acalles\_came

Acalles\_came

Acalles\_came

Acalles\_came

Acalles\_came

Acalles\_came

Acalles\_came

Acalles\_diec

Acalles\_dubi

Acalles\_dubi

Acalles\_dubi

Acalles\_dubi

Acalles\_echi

Acalles\_echi

Acalles\_echi

Acalles\_echi

Acalles\_echi

Acalles\_echi

Acalles\_echi

Acalles\_echi

Acalles\_echi

Acalles\_echi

Acalles\_echi

Acalles\_echi

Acalles\_echi

Acalles\_fall

Acalles\_fall

Acalles\_fall

Acalles\_fall

Acalles\_fall

Acalles\_fall

Acalles\_fall

Acalles\_fall

Acalles\_fall

Acalles\_fall

Acalles\_fall

Acalles\_fall

Acalles\_fall

Acalles\_fall

Acalles\_gado

Acalles\_glob

Acalles\_glob

Acalles\_glob

Acalles\_glob

Acalles\_glob

Acalles\_glob

Acalles\_glob

Acalles\_glob

Acalles\_gran

Acalles\_gran

Acalles\_kipp

Acalles\_lemu

Acalles\_lemu

Acalles\_lemu

Acalles\_lemu

Acalles\_lemu

Acalles\_lemu

Acalles\_lemu

Acalles\_lemu

Acalles\_lemu

Acalles\_lemu

Acalles\_lemu

Acalles\_lemu

Acalles\_lemu

Acalles\_lemu

Acalles\_lemu

Acalles\_micr

Acalles\_micr

Acalles\_micr

Acalles\_micr

Acalles\_micr

Acalles\_micr

Acalles\_micr

Acalles\_minu

Acalles\_mise

Acalles\_mise

Acalles\_mise

Acalles\_mise

Acalles\_mise

Acalles\_mise

Acalles\_mise

Acalles\_mise

Acalles\_mise

Acalles\_mise

Acalles\_mise

Acalles\_mise

Acalles\_osse

Acalles\_osse

Acalles\_pape

Acalles\_pape

Acalles\_pape

Acalles\_pape

Acalles\_pape

Acalles\_parv

Acalles\_parv

Acalles\_parv

Acalles\_parv

Acalles\_parv

Acalles\_parv

Acalles\_parv

Acalles\_parv

Acalles\_parv

Acalles\_parv

Acalles\_parv

Acalles\_parv

Acalles\_parv

Acalles\_parv

Acalles\_parv

Acalles\_pilu

Acalles\_pilu

Acalles\_pilu

Acalles\_pilu

Acalles\_pilu

Acalles\_pilu

Acalles\_pilu

Acalles\_ptin

Acalles\_ptin

Acalles\_ptin

Acalles\_ptin

Acalles\_ptin

Acalles\_ptin

Acalles\_ptin

Acalles\_ptin

Acalles\_ptin

Acalles\_reit

Acalles\_sabl

Acalles\_sard

Acalles\_sard

Acalles\_sard

Acalles\_sard

Acalles\_sard

Acalles\_sard

Acalles\_sard

Acalles\_sint

Acalles\_sint

Acalles\_sint

Acalles\_sint

Acalles\_sylv

Acalles\_tibi

Acalles\_tibi

Acalles\_vors

Acallocrates

Acallocrates

Acallocrates

Acallocrates

Acallocrates

Acallocrates

Acallocrates

Acallocrates

Acallocrates

Acallocrates

Acallocrates

Acallocrates

Acallocrates

Acallocrates

Acallocrates

Acallocrates

Acallocrates

Acallorneuma

Acallorneuma

Acallorneuma

Acallorneuma

Acallorneuma

Acallorneuma

Acallorneuma

Acallorneuma

Acallorneuma

Acallorneuma

Acallorneuma

Acallorneuma

Acallorneuma

Acallorneuma

Acallorneuma

Acallorneuma

Acallorneuma

Acallorneuma

Aeoniacalles

Aeoniacalles

Aeoniacalles

Aeoniacalles

Aeoniacalles

Aeoniacalles

Aeoniacalles

Aeoniacalles

Aeoniacalles

Aeoniacalles

Aeoniacalles

Aeoniacalles

Aeoniacalles

Aeoniacalles

Aeoniacalles

Aeoniacalles

Aeoniacalles

Aeoniacalles

Aeoniacalles

Aeoniacalles

Aeoniacalles

Aeoniacalles

Aeoniacalles

Aeoniacalles

Aeoniacalles

Calacalles\_a

Calacalles\_a

Calacalles\_a

Calacalles\_b

Calacalles\_b

Calacalles\_b

Calacalles\_c

Calacalles\_c

Calacalles\_d

Calacalles\_d

Calacalles\_d

Calacalles\_d

Calacalles\_e

Calacalles\_e

Calacalles\_e

Calacalles\_f

Calacalles\_f

Calacalles\_h

Calacalles\_h

Calacalles\_h

Calacalles\_h

Calacalles\_k

Calacalles\_l

Calacalles\_l

Calacalles\_l

Calacalles\_m

Calacalles\_m

Calacalles\_m

Calacalles\_m

Calacalles\_m

Calacalles\_m

Calacalles\_m

Calacalles\_m

Calacalles\_m

Calacalles\_m

Calacalles\_m

Calacalles\_m

Calacalles\_m

Calacalles\_n

Calacalles\_n

Calacalles\_n

Calacalles\_n

Calacalles\_n

Calacalles\_p

Calacalles\_p

Calacalles\_p

Calacalles\_p

Calacalles\_p

Calacalles\_p

Calacalles\_p

Calacalles\_p

Calacalles\_p

Calacalles\_p

Calacalles\_p

Calacalles\_s

Calacalles\_s

Calacalles\_t

Calacalles\_t

Calacalles\_t

Calacalles\_w

Calacalles\_w

Calacalles\_w

Calacalles\_w

Calacalles\_w

Canariacalle

Canariacalle

Canariacalle

Canariacalle

Canariacalle

Canariacalle

Canariacalle

Canariacalle

Caucasusacal

Cionus\_longi

Coloracalles

Coloracalles

Coloracalles

Coloracalles

Coloracalles

Cryptorhynch

Cryptorhynch

Dendroacalle

Dendroacalle

Dendroacalle

Dendroacalle

Dendroacalle

Dendroacalle

Dendroacalle

Dendroacalle

Dendroacalle

Dendroacalle

Dendroacalle

Dendroacalle

Dendroacalle

Dendroacalle

Dendroacalle

Dendroacalle

Dendroacalle

Dendroacalle

Dendroacalle

Dendroacalle

Dendroacalle

Dendroacalle

Dendroacalle

Dendroacalle

Dendroacalle

Dendroacalle

Dendroacalle

Dendroacalle

Dendroacalle

Dendroacalle

Dendroacalle

Dendroacalle

Dendroacalle

Dendroacalle

Dendroacalle

Dendroacalle

Dendroacalle

Dendroacalle

Dendroacalle

Dendroacalle

Dendroacalle

Dendroacalle

Dendroacalle

Dendroacalle

Dichromacall

Dichromacall

Dichromacall

Dichromacall

Dichromacall

Dichromacall

Dichromacall

Dichromacall

Dichromacall

Dichromacall

Dichromacall

Dichromacall

Dichromacall

Dichromacall

Dichromacall

Dichromacall

Dichromacall

Dichromacall

Dichromacall

Dichromacall

Dichromacall

Dichromacall

Dichromacall

Dichromacall

Dichromacall

Dichromacall

Dichromacall

Dichromacall

Dichromacall

Dichromacall

Dichromacall

Dichromacall

Dichromacall

Dichromacall

Dichromacall

Dichromacall

Dichromacall

Dichromacall

Dichromacall

Dichromacall

Dichromacall

Dichromacall

Dichromacall

Dichromacall

Dichromacall

Dichromacall

Echinodera\_a

Echinodera\_a

Echinodera\_a

Echinodera\_a

Echinodera\_a

Echinodera\_a

Echinodera\_a

Echinodera\_a

Echinodera\_a

Echinodera\_a

Echinodera\_a

Echinodera\_a

Echinodera\_a

Echinodera\_a

Echinodera\_a

Echinodera\_a

Echinodera\_a

Echinodera\_a

Echinodera\_a

Echinodera\_a

Echinodera\_a

Echinodera\_a

Echinodera\_a

Echinodera\_a

Echinodera\_a

Echinodera\_a

Echinodera\_a

Echinodera\_a

Echinodera\_a

Echinodera\_a

Echinodera\_a

Echinodera\_a

Echinodera\_a

Echinodera\_a

Echinodera\_a

Echinodera\_a

Echinodera\_a

Echinodera\_a

Echinodera\_a

Echinodera\_a

Echinodera\_a

Echinodera\_a

Echinodera\_a

Echinodera\_a

Echinodera\_a

Echinodera\_a

Echinodera\_a

Echinodera\_a

Echinodera\_b

Echinodera\_b

Echinodera\_b

Echinodera\_b

Echinodera\_b

Echinodera\_b

Echinodera\_b

Echinodera\_b

Echinodera\_b

Echinodera\_b

Echinodera\_b

Echinodera\_b

Echinodera\_b

Echinodera\_b

Echinodera\_b

Echinodera\_b

Echinodera\_b

Echinodera\_b

Echinodera\_b

Echinodera\_b

Echinodera\_b

Echinodera\_b

Echinodera\_b

Echinodera\_b

Echinodera\_b

Echinodera\_b

Echinodera\_b

Echinodera\_b

Echinodera\_b

Echinodera\_b

Echinodera\_b

Echinodera\_b

Echinodera\_b

Echinodera\_b

Echinodera\_b

Echinodera\_b

Echinodera\_c

Echinodera\_c

Echinodera\_c

Echinodera\_c

Echinodera\_c

Echinodera\_c

Echinodera\_c

Echinodera\_c

Echinodera\_c

Echinodera\_c

Echinodera\_c

Echinodera\_c

Echinodera\_c

Echinodera\_c

Echinodera\_c

Echinodera\_c

Echinodera\_c

Echinodera\_c

Echinodera\_c

Echinodera\_c

Echinodera\_c

Echinodera\_c

Echinodera\_c

Echinodera\_c

Echinodera\_c

Echinodera\_c

Echinodera\_c

Echinodera\_c

Echinodera\_c

Echinodera\_c

Echinodera\_c

Echinodera\_c

Echinodera\_g

Echinodera\_g

Echinodera\_g

Echinodera\_g

Echinodera\_h

Echinodera\_h

Echinodera\_h

Echinodera\_h

Echinodera\_h

Echinodera\_h

Echinodera\_h

Echinodera\_h

Echinodera\_h

Echinodera\_h

Echinodera\_h

Echinodera\_h

Echinodera\_h

Echinodera\_h

Echinodera\_h

Echinodera\_h

Echinodera\_h

Echinodera\_h

Echinodera\_h

Echinodera\_h

Echinodera\_h

Echinodera\_h

Echinodera\_h

Echinodera\_h

Echinodera\_h

Echinodera\_h

Echinodera\_h

Echinodera\_h

Echinodera\_h

Echinodera\_h

Echinodera\_h

Echinodera\_h

Echinodera\_h

Echinodera\_h

Echinodera\_i

Echinodera\_i

Echinodera\_i

Echinodera\_i

Echinodera\_i

Echinodera\_i

Echinodera\_i

Echinodera\_i

Echinodera\_i

Echinodera\_i

Echinodera\_i

Echinodera\_j

Echinodera\_j

Echinodera\_j

Echinodera\_k

Echinodera\_k

Echinodera\_k

Echinodera\_l

Echinodera\_l

Echinodera\_l

Echinodera\_l

Echinodera\_l

Echinodera\_l

Echinodera\_l

Echinodera\_l

Echinodera\_m

Echinodera\_m

Echinodera\_m

Echinodera\_m

Echinodera\_m

Echinodera\_m

Echinodera\_m

Echinodera\_m

Echinodera\_m

Echinodera\_m

Echinodera\_n

Echinodera\_n

Echinodera\_n

Echinodera\_n

Echinodera\_o

Echinodera\_o

Echinodera\_o

Echinodera\_o

Echinodera\_o

Echinodera\_o

Echinodera\_o

Echinodera\_o

Echinodera\_p

Echinodera\_p

Echinodera\_p

Echinodera\_p

Echinodera\_p

Echinodera\_p

Echinodera\_p

Echinodera\_p

Echinodera\_p

Echinodera\_p

Echinodera\_p

Echinodera\_p

Echinodera\_p

Echinodera\_p

Echinodera\_p

Echinodera\_p

Echinodera\_p

Echinodera\_p

Echinodera\_p

Echinodera\_p

Echinodera\_p

Echinodera\_p

Echinodera\_p

Echinodera\_p

Echinodera\_p

Echinodera\_p

Echinodera\_p

Echinodera\_p

Echinodera\_p

Echinodera\_p

Echinodera\_p

Echinodera\_p

Echinodera\_p

Echinodera\_p

Echinodera\_p

Echinodera\_p

Echinodera\_p

Echinodera\_p

Echinodera\_p

Echinodera\_p

Echinodera\_p

Echinodera\_p

Echinodera\_p

Echinodera\_p

Echinodera\_p

Echinodera\_p

Echinodera\_p

Echinodera\_p

Echinodera\_p

Echinodera\_p

Echinodera\_p

Echinodera\_p

Echinodera\_r

Echinodera\_r

Echinodera\_r

Echinodera\_r

Echinodera\_r

Echinodera\_r

Echinodera\_r

Echinodera\_r

Echinodera\_r

Echinodera\_r

Echinodera\_r

Echinodera\_r

Echinodera\_s

Echinodera\_s

Echinodera\_s

Echinodera\_s

Echinodera\_s

Echinodera\_s

Echinodera\_s

Echinodera\_s

Echinodera\_s

Echinodera\_s

Echinodera\_s

Echinodera\_s

Echinodera\_s

Echinodera\_s

Echinodera\_s

Echinodera\_s

Echinodera\_s

Echinodera\_s

Echinodera\_s

Echinodera\_s

Echinodera\_s

Echinodera\_s

Echinodera\_s

Echinodera\_s

Echinodera\_s

Echinodera\_s

Echinodera\_s

Echinodera\_s

Echinodera\_t

Echinodera\_t

Echinodera\_t

Echinodera\_t

Echinodera\_t

Echinodera\_t

Echinodera\_v

Echinodera\_v

Echinodera\_v

Echinodera\_v

Echinodera\_v

Echinodera\_v

Echinodera\_v

Echinodera\_z

Echiumacalle

Echiumacalle

Echiumacalle

Echiumacalle

Elliptacalle

Elliptacalle

Elliptacalle

Elliptacalle

Elliptacalle

Elliptacalle

Elliptacalle

Euscepes\_bat

Euscepes\_bat

Ficusacalles

Ficusacalles

Ficusacalles

Ficusacalles

Ficusacalles

Ficusacalles

Ficusacalles

Ficusacalles

Ficusacalles

Ficusacalles

Ficusacalles

Ficusacalles

Ficusacalles

Ficusacalles

Ficusacalles

Ficusacalles

Ficusacalles

Ficusacalles

Ficusacalles

Kyklioacalle

Kyklioacalle

Kyklioacalle

Kyklioacalle

Kyklioacalle

Kyklioacalle

Kyklioacalle

Kyklioacalle

Kyklioacalle

Kyklioacalle

Kyklioacalle

Kyklioacalle

Kyklioacalle

Kyklioacalle

Kyklioacalle

Kyklioacalle

Kyklioacalle

Kyklioacalle

Kyklioacalle

Kyklioacalle

Kyklioacalle

Kyklioacalle

Kyklioacalle

Kyklioacalle

Kyklioacalle

Kyklioacalle

Kyklioacalle

Kyklioacalle

Kyklioacalle

Kyklioacalle

Kyklioacalle

Kyklioacalle

Kyklioacalle

Kyklioacalle

Kyklioacalle

Kyklioacalle

Kyklioacalle

Kyklioacalle

Kyklioacalle

Kyklioacalle

Kyklioacalle

Kyklioacalle

Kyklioacalle

Kyklioacalle

Kyklioacalle

Kyklioacalle

Kyklioacalle

Kyklioacalle

Kyklioacalle

Kyklioacalle

Kyklioacalle

Kyklioacalle

Kyklioacalle

Kyklioacalle

Kyklioacalle

Kyklioacalle

Kyklioacalle

Kyklioacalle

Kyklioacalle

Kyklioacalle

Kyklioacalle

Kyklioacalle

Kyklioacalle

Kyklioacalle

Kyklioacalle

Kyklioacalle

Kyklioacalle

Kyklioacalle

Kyklioacalle

Kyklioacalle

Kyklioacalle

Kyklioacalle

Kyklioacalle

Kyklioacalle

Kyklioacalle

Kyklioacalle

Kyklioacalle

Kyklioacalle

Kyklioacalle

Kyklioacalle

Kyklioacalle

Kyklioacalle

Kyklioacalle

Kyklioacalle

Kyklioacalle

Kyklioacalle

Kyklioacalle

Kyklioacalle

Kyklioacalle

Kyklioacalle

Kyklioacalle

Kyklioacalle

Kyklioacalle

Kyklioacalle

Kyklioacalle

Kyklioacalle

Kyklioacalle

Kyklioacalle

Kyklioacalle

Kyklioacalle

Kyklioacalle

Kyklioacalle

Kyklioacalle

Kyklioacalle

Kyklioacalle

Kyklioacalle

Kyklioacalle

Kyklioacalle

Kyklioacalle

Kyklioacalle

Kyklioacalle

Kyklioacalle

Kyklioacalle

Kyklioacalle

Kyklioacalle

Kyklioacalle

Kyklioacalle

Kyklioacalle

Kyklioacalle

Kyklioacalle

Kyklioacalle

Kyklioacalle

Kyklioacalle

Kyklioacalle

Kyklioacalle

Kyklioacalle

Kyklioacalle

Kyklioacalle

Kyklioacalle

Kyklioacalle

Kyklioacalle

Kyklioacalle

Kyklioacalle

Kyklioacalle

Kyklioacalle

Kyklioacalle

Kyklioacalle

Kyklioacalle

Kyklioacalle

Kyklioacalle

Kyklioacalle

Kyklioacalle

Kyklioacalle

Kyklioacalle

Kyklioacalle

Lauriacalles

Lauriacalles

Lauriacalles

Lauriacalles

Lauriacalles

Lauriacalles

Lauriacalles

Lauriacalles

Lauriacalles

Lauriacalles

Madeiracalle

Madeiracalle

Madeiracalle

Madeiracalle

Madeiracalle

Madeiracalle

Madeiracalle

Madeiracalle

Madeiracalle

Madeiracalle

Madeiracalle

Madeiracalle

Madeiracalle

Madeiracalle

Madeiracalle

Madeiracalle

Madeiracalle

Madeiracalle

Madeiracalle

Madeiracalle

Madeiracalle

Madeiracalle

Madeiracalle

Madeiracalle

Madeiracalle

Madeiracalle

Madeiracalle

Madeiracalle

Madeiracalle

Madeiracalle

Madeiracalle

Madeiracalle

Madeiracalle

Madeiracalle

Madeiracalle

Madeiracalle

Madeiracalle

Madeiracalle

Madeiracalle

Madeiracalle

Montanacalle

Onyxacalles\_

Onyxacalles\_

Onyxacalles\_

Onyxacalles\_

Onyxacalles\_

Onyxacalles\_

Onyxacalles\_

Onyxacalles\_

Onyxacalles\_

Onyxacalles\_

Onyxacalles\_

Onyxacalles\_

Onyxacalles\_

Onyxacalles\_

Onyxacalles\_

Onyxacalles\_

Onyxacalles\_

Onyxacalles\_

Onyxacalles\_

Onyxacalles\_

Onyxacalles\_

Onyxacalles\_

Onyxacalles\_

Onyxacalles\_

Onyxacalles\_

Onyxacalles\_

Onyxacalles\_

Onyxacalles\_

Onyxacalles\_

Onyxacalles\_

Onyxacalles\_

Onyxacalles\_

Onyxacalles\_

Onyxacalles\_

Onyxacalles\_

Onyxacalles\_

Onyxacalles\_

Onyxacalles\_

Onyxacalles\_

Onyxacalles\_

Onyxacalles\_

Onyxacalles\_

Onyxacalles\_

Onyxacalles\_

Onyxacalles\_

Onyxacalles\_

Onyxacalles\_

Onyxacalles\_

Onyxacalles\_

Onyxacalles\_

Onyxacalles\_

Onyxacalles\_

Onyxacalles\_

Onyxacalles\_

Pseudodichro

Pseudodichro

Pseudodichro

Pseudodichro

Pseudodichro

Pseudodichro

Pseudodichro

Pseudodichro

Pseudodichro

Pseudodichro

Pseudodichro

Pseudodichro

Silvacalles\_

Silvacalles\_

Silvacalles\_

Silvacalles\_

Silvacalles\_

Silvacalles\_

Silvacalles\_

Silvacalles\_

Silvacalles\_

Silvacalles\_

Silvacalles\_

Silvacalles\_

Silvacalles\_

Silvacalles\_

Silvacalles\_

Silvacalles\_

Silvacalles\_

Silvacalles\_

Silvacalles\_

Silvacalles\_

Silvacalles\_

Silvacalles\_

Silvacalles\_

Silvacalles\_

Silvacalles\_

Silvacalles\_

Silvacalles\_

Silvacalles\_

Silvacalles\_

Silvacalles\_

Silvacalles\_

Silvacalles\_

Silvacalles\_

Silvacalles\_

Silvacalles\_

Silvacalles\_

Silvacalles\_

Silvacalles\_

Silvacalles\_

Silvacalles\_

Silvacalles\_

Silvacalles\_

Silvacalles\_

Silvacalles\_

Silvacalles\_

Silvacalles\_

Silvacalles\_

Silvacalles\_

Silvacalles\_

Silvacalles\_

Silvacalles\_

Silvacalles\_

Silvacalles\_

Silvacalles\_

Silvacalles\_

Silvacalles\_

Silvacalles\_

Silvacalles\_

Silvacalles\_

Silvacalles\_

Silvacalles\_

Silvacalles\_

Silvacalles\_

Silvacalles\_

Silvacalles\_

Silvacalles\_

Sonchiacalle

Sonchiacalle

Sonchiacalle

Sonchiacalle

Sonchiacalle

Sonchiacalle

Sonchiacalle

Sonchiacalle

Sonchiacalle

Sonchiacalle

Sonchiacalle

Sonchiacalle

Sonchiacalle

Sonchiacalle

Sonchiacalle

Sonchiacalle

Torneuma\_ale

Torneuma\_ale

Torneuma\_ale

Torneuma\_aph

Torneuma\_aph

Torneuma\_aph

Torneuma\_bae

Torneuma\_cad

Torneuma\_cae

Torneuma\_dep

Torneuma\_dep

Torneuma\_dep

Torneuma\_dep

Torneuma\_des

Torneuma\_fel

Torneuma\_fel

Torneuma\_isa

Torneuma\_isa

Torneuma\_kar

Torneuma\_kor

Torneuma\_kor

Torneuma\_mad

Torneuma\_mad

Torneuma\_mes

Torneuma\_orb

Torneuma\_orb

Torneuma\_orb

Torneuma\_pic


Tooltip
  
 View/save curves and dendrogram here  
  
*Responsive Crossing lines Legend: Green Line= grouping distance (Dc)- Red line = treshold distance(Dt)*  
Running time:
0 min 18 seconds
  
