# Supplementary material for: Molecular Weevil Identification Project: A thoroughly curated barcode release of 1300 Western Palearctic weevil species (Coleoptera, Curculionoidea)
Source: Biodivers Data J. 2023 Jan 24;11:e96438. doi: 10.3897/BDJ.11.e96438 (PMC10865102; doi:10.3897/BDJ.11.e96438)
Supplement: Supplementary material 8 — Thresholds in various taxa [file bdj-11-e96438-s008.pdf]

Thresholds for species delineation

Sharp thresholds and barcode gap ranges are often discussed to define species boundaries since DNA barcoding has been introduced (Hebert et al. 2003a, 2003b) but were never consistent between different animal groups. Thus the search for "the optimal threshold" is ongoing.

The ***10x rule*** was introduced as an indicator of cryptic speciation. It was used as a starting point to define minimum interspecific distances where single sequences were flagged as possible cryptic species if they diverge by ten times higher than the average of typical intraspecific variability of the group (Hebert 2004a). To the bird sequences examined, this seems reliable and safe to apply in other animal groups if those had sufficient time for divergence. The amount of "sufficient time" has been evaluated on sequence data from fruit flies (Coyne and Orr 1997), butterflies (Presgraves 2002), urchins (Zigler et al. 2005), frogs (Sasa et al. 1998), and darters (birds, Mendelson 2003). The 10x rule seems safe if the species in focus were separated for around *4 million generations*. That can be 4 million years for one group or just 1 million years for another group, depending on the number of generations within a typical year (Hickerson et al. 2006).

Examples of thresholds in various groups

Tropical water beetles from Madagascar (Coleoptera: Dytiscidae, Hydrophilidae) showed interspecific distances of at least 14% by low intraspecific distances of max. 2%. A threshold has not been provided in the study, but any value between **2%** and **14%** would work for a successful identification (Monaghan et al. 2005). **Neotropical dung beetles** (Coleoptera: Scarabaeidae, gen. *Canthon*) showed interspecific distances of at least 9.9% by low intraspecific distances of a maximum of 1%. Any threshold between **1%** and **9.9%** would be able to delineate the *Canthon* beetles (Monaghan et al. 2005). A threshold of **3%** (K2P) for skipper **butterflies** of Ontario, Canada (singletons, 200 sequences of 200 species) was able to assign 98% to the correct morphological identified species (Hebert et al. 2004b). For Pholcidae **spiders** of the genus *Modisimus* in Haiti, a **5%** (K2P) threshold would be very well suited, except for two populations. Thus, those have been omitted in the threshold suggestion; the gap shows 4.6-9.6% without the two previously mentioned populations (Hubert et al. 2010). For neotropical **butterflies** (Lepidoptera: Lycaenidae, genus *Agrodiaetus*) the best available threshold of **2.8%** (K2P) was be able to deliminate 88% of the species studies correctly. 350 newly generated barcodes and 300 GenBank barcodes have been used. Some species showed no interspecific distance (no barcode gap between species but no overlap), some even 23%. The overlap led to the conclusion a barcode gap does not exist for butterflies (Wiemers & Fiedler 2007). However, some species with low divergences include species with debatable species status, which could also lead to the previous assumption. The authors state that in the practical application, the problem of misidentified specimens and sequences in GenBank remains a real threat to the accuracy of barcode-based identifications. During their study, they could also prove wrongly assigned taxa names in GenBank (Wiemers & Fiedler 2007). In globally distributed **moths** (Lepidoptera: Sphingidae, gen. *Hyles*), the studied genus comprises 29 species, where 17 species have been collected by the authors and 5 added from GenBank. No threshold corresponds perfectly to current species delimitations in *Hyles*. A 1% threshold value would lead to lumps and oversplits at the same time (Hundsdoerfer et al. 2009). Another study in

Western Palearctic inquiline oak **gall wasps** (Hymenoptera: Cynipidae, gen. *Synergus*) processed 184 specimens comprising 33 of the 45 described species. Three thresholds have been evaluated (6.4%, 3.8% and 1.1%). By applying **6.4%** as a threshold, the results were in concordance with morphology-based species identification in general. Still, there were also a dozen sequences clustering in the wrong morpho species cluster. However, those species-level taxonomies were stated as problematic by the authors (Acs et al. 2010). For **crustaceans** (Cladocera, Branchiopoda, Copepoda, Decapoda, Ostracoda), a threshold of **16%** (K2P) delivers a 98% success rate in species delineation (Lefébure et al. 2006). In a dataset with 327 sequences of 203 different **arachnid** species of North America (including 35 species of Acari and Scorpiones), a threshold of **4%** (K2P) successfully delineated 96% of the 327 sequences in concordance with previous morphological identifications (Barrett & Hebert 2005). The aforementioned results were later confirmed based on a dataset of broader geographic scale and denser taxon sampling. The average maximum intraspecific K2P distance was 3.16%, while the mean distance between nearest congeners was 6.77%, demonstrating the typical presence of a barcode gap (Robinson et al. 2009) thus, the **4%** (K2P) threshold would be able to delineate the species in this dataset as well. Within 216 sequences (8 species) of **orb-weaving spiders** from Asia, Amerika and Europe (Araneae, Araneidae, genus *Araniella*), any threshold value between **1.3%** and **3.5%** would result in a minimal cumulative error with no false positives and 2 true negatives (Spasojevic et al. 2016). For mantellid **frogs** from Madagascar (Mantellidae) and climbing **salamanders** (genus *Aneides*) from North America, a K2P threshold of **10%** is applicable in general. Still, for some frogs, an intraspecific divergence of up to 18% was observed, and the mean interspecific divergence was actually 20.7% for the frogs and 13.5% for the salamanders (Vences et al. 2005). In **Palearctic marine mussels** (Mollusca: Conchifera, Bivalves, genera: *Thyasira*, *Ennucula*, *Nucula*, *Yoldiella*), any threshold value between **1.9%** and **14%** would distinguish between the species investigated (Mikkelsen et al. 2007). A study with Canadian **freshwater fish** (1360 specimens, reflecting 190 out of 203 taxa) showed a variation in their genetic distances. Although a barcode gap existed for 93% of the species, the genetic distances between congeners ranged between 0% and 19.3% (K2P). Any applied threshold would automatically lead to wrongly assigned taxa (Hubert et al. 2008).

References

- Acs Z, Challis RJ, Bihari P, Blaxter M, Hayward A, Melika G, Csoka G, Penzes Z, Pujade-Villar J, Nieves-Aldrey JL, Schonrogge K, Stone GN (2010) Phylogeny and DNA barcoding of inquiline oak gallwasps (Hymenoptera: Cynipidae) of the Western Palaearctic. *Molecular Phylogenetics and Evolution* **55**(1): 210-225. DOI: 10.1016/j.ympev.2009.12.004
- Barrett RDH, Hebert PDN (2005) Identifying spiders through DNA barcodes. *Canadian Journal of Zoology* **83**(3): 481-491. DOI:10.1139/z05-024
- Coyne JA, Orr HA (1997) "Patterns of Speciation in Drosophila" Revisited. *Evolution* **51**(1): 295-303. DOI: 10.2307/2410984
- Hickerson MJ, Meyer CP, Moritz C (2006) DNA barcoding will often fail to discover new animal species over broad parameter space. *Systematic Biology* **55**(5): 729-739. DOI: 10.1080/10635150600969898
- Hebert PD, Ratnasingham S, deWaard JR (2003a) Barcoding animal life: cytochrome c oxidase subunit 1 divergences among closely related species. *Proceedings of the Royal Society of London, Series B*: *Biological Sciences* **270**: 96-99. DOI:10.1098/rsbl.2003.0025
- Hebert PD, Cywinska A, Ball SL, deWaard JR (2003b) Biological identifications through DNA barcodes. *Proceedings of the Royal Society of London, Series B: Biological Sciences* **270**(1512): 313-321. DOI: 10.1098/rspb.2002.2218
- Hebert PD, Stoeckle MY, Zemlak TS, Francis CM (2004a) Identification of birds through DNA barcodes. *PLoS biology* **2**(10): e312 (1657-1633). DOI: 10.1371/journal.pbio.0020312
- Hebert PD, Penton EH, Burns JM, Janzen DH, Hallwachs W (2004b) Ten species in one: DNA barcoding reveals cryptic species in the neotropical skipper butterfly *Astraptes fulgerator*. *Proceedings of the National Academy of Sciences of the USA* **101**(41): 14812-14817. DOI: 10.1073/pnas.0406166101
- Hubert N, Hanner R, Holm E, Mandrak N, Taylor E, Burridge M, Watkinson D, Dumont P, Curry R, Bentzen P, Zhang J, April J, Bernatchez L (2008) Identifying Canadian Freshwater Fishes through DNA Barcodes. *PLoS One* **3**(6): 1-8 (e2490). DOI: 10.1371/journal.pone.0002490
- Hubert N, Delrieu-Trottin E, Irisson JO, Meyer C, Planes S (2010) Identifying coral reef fish larvae through DNA barcoding: a test case with the families Acanthuridae and Holocentridae. *Molecular phylogenetics and evolution* **55**(3): 1195-1203. DOI: 10.1016/j.ympev.2010.02.023
- Hundsdoerfer AK, Rubinoff D, Attie M, Wink M, Kitching IJ (2009) A revised molecular phylogeny of the globally distributed hawkmoth genus Hyles (Lepidoptera: Sphingidae), based on mitochondrial and nuclear DNA sequences. *Molecular Phylogenetics and Evolution* **52**(3): 852-865. DOI: 10.1016/j.ympev.2009.05.023
- Lefebure T, Douady CJ, Gouy M, Gibert J (2006) Relationship between morphological taxonomy and molecular divergence within Crustacea: proposal of a molecular threshold to help species delimitation. *Molecular phylogenetics and evolution* **40**(2): 435-447. DOI: 10.1016/j.ympev.2006.03.014
- Mendelson TC (2003) Evidence of intermediate and asymmetrical behavioral isolation between orangethroat and orangebelly darters (Teleostei : Percidae). *American Midland Naturalist* **150**(2): 343-347. DOI: 10.1674/0003-0031(2003)150[0343:EOIAAB]2.0.CO;2
- Mikkelsen N, Schander C, Willassen E (2007) Local scale DNA barcoding of bivalves (Mollusca): A case study. *Zoologica Scripta* **36**: 455-463. DOI: 10.1111/j.1463-6409.2006.00289.x

- Monaghan M, Balke M, Gregory TR, Vogler A (2005) DNA-based species delineation in tropical beetles using mitochondrial and nuclear markers. *Philosophical transactions of the Royal Society of London Series B, Biological sciences* **360**: 1925-1933. DOI: 10.1098/rstb.2005.1724
- Presgraves DC (2002) Patterns of postzygotic isolation in Lepidoptera. *Evolution* **56**(6): 1168-1183. DOI: 10.1111/j.0014-3820.2002.tb01430.x
- Robinson EA, Blagoev GA, Hebert PDN, Adamowicz SJ (2009) Prospects for using DNA barcoding to identify spiders in species-rich genera. *Zookeys* **16**: 27-46. DOI: 10.3897/zookeys.16.239
- Sasa MM, Chippindale PT, Johnson NA (1998) Patterns of Postzygotic Isolation in Frogs. *Evolution* **52**(6): 1811-1820. DOI: 10.1111/j.1558-5646.1998.tb02258.x
- Spasojevic T, Kropf C, Nentwig W, Lasut L (2016) Combining morphology, DNA sequences, and morphometrics: revising closely related species in the orb-weaving spider genus Araniella (Araneae, Araneidae). *Zootaxa* **4111**(4): 448-470. DOI: 10.11646/zootaxa.4111.4.6
- Wiemers M, Fiedler K (2007) Does the DNA barcoding gap exist? - a case study in blue butterflies (Lepidoptera: Lycaenidae). *Frontiers in Zoology* **4**(8): 1-16. DOI: 10.1186/1742-9994-4-8
- Vences M, Thomas M, Bonett RM, Vieites DR (2005) Deciphering amphibian diversity through DNA barcoding: chances and challenges. *Philosophical transactions of the Royal Society of London. Series B, Biological sciences* **360**(1462): 1859-1868. DOI: 10.1098/rstb.2005.1717
- Zigler KS, McCartney MA, Levitan DR, Lessios HA (2005) Sea urchin bindin divergence predicts gamete compatibility. *Evolution* **59**(11): 2399-2404. DOI: 10.1554/05-098.1
