# Supplementary material for: Molecular Weevil Identification Project: A thoroughly curated barcode release of 1300 Western Palearctic weevil species (Coleoptera, Curculionoidea)
Source: Biodivers Data J. 2023 Jan 24;11:e96438. doi: 10.3897/BDJ.11.e96438 (PMC10865102; doi:10.3897/BDJ.11.e96438)
Supplement: Supplementary material 9 — Aeoniacallesaeoniibodegensis [file bdj-11-e96438-s009.pdf]

Aeoniacalles aeonii bodegensis (Stüben, 2000)

A somewhat unsolved taxonomic case is *Aeoniacalles aeonii bodegensis* (Stüben, 2000). It was described as *Acalles bodegensis* and can be distinguished by clear morphological characters. Cross-breeding experiments between *Acalles aeonii* and the newly described *Acalles bodegensis* (taxonomic status in the year 2005) over three generations for two years have produced healthy offspring without any indication of degenerations or anomalies. Based on those results, *Acalles bodegensis* has been synonymized (Stüben 2005). The morphological characters were accepted as an unusually large variability within the species *Acalles aeonii* (Stüben 2005, Stüben & Germann 2005). In 2010 the genus *Aeoniacalles* was described (Stüben & Astrin 2010). Molecular data has become available around the same time, revealing a **10% p-distance** between both taxa. The previously synonymized species was reinstated, but this time as a subspecies (Stüben & Astrin 2011): *Aeoniacalles aeonii bodegensis* (Stüben, 2000), which is still valid up to date.

This is a rare case where CO1 barcode data and cross-breeding experiments led to opposite conclusions. While the cross-breeding experiments refuted reproductive isolation following the *biological species concept* (Mayr 1942), the genetic distance between *A. aeonii* and the subspecies *bodegensis* is even further above the average value for genetic distances between species of the genus *Aeoniacalles.*The average interspecific genetic distance for species of the genus *Aeoniacalles* on islands is **9.1%** (see Table 3 in the main text). If molecular data were the only reference point for species delineation, *A. aeonii bodegensis* would have been raised to species level instead of being kept as subspecies.

References

- Mayr E (1942) Systematics and the Origin of Species from the Viewpoint of a Zoologist. 1st edition. Columbia University Press, New York, 334 pp. [In English].
- Stüben PE (2005) "Basar Taxonomie"? - Ein erfolgreiches Kreuzungsexperiment zu *Acalles aeonii* Wollaston, 1864 (Coleoptera: Curculionidae). *Weevil News* **31**: 1-13. URL: https://www.curci.de/?beitrag=144
- Stüben PE, Germann C (2005) Neue Erkenntnisse zur Taxonomie, Biologie und Ökologie der Cryptorhynchinae von den Makaronesischen Inseln. 1. Beitrag: Kanaren / Tenerife (Coleoptera: Curculionidae: Cryptorhynchinae). *SNUDEBILLER: Studies on taxonomy, biology and ecology of Curculionoidea* **6** (61): 37-83. [In German] URL: https://www.curci.de/?beitrag=61
- Stüben PE, Astrin JJ (2010) Molecular phylogeny in endemic weevils: Revision of the genera of Macaronesian Cryptorhynchinae (Coleoptera: Curculionidae). *Zoological Journal of the Linnean Society* **160** (1): 40-87. URL: https://doi.org/10.1111/j.1096-3642.2009.00609.x
- Stüben PE, Astrin JJ (2011) *Aeoniacalles tabladoensis* sp.n. and *Aeoniacalles aeonii bodegensis* (Stüben 2000) resyn. from the Canary Islands (Coleoptera: Curculionidae: Cryptorhynchinae). *Weevil News* **68**: 1-5. URL: https://www.curci.de/?beitrag=186
